# Extensive spontaneous genome reduction in *Paraburkholderia sabiae*

**DOI:** 10.1038/s41598-026-49026-9

**Published:** 2026-04-29

**Authors:** Kim Bolli, Colin Waichler, Yilei Liu, Daphné Golaz, Adam M. Deutschbauer, Leo Eberl, Sebastian J. Hug, Marta Torres, Gabriella Pessi

**Affiliations:** 1 Department of Plant and Microbial Biology, University of Zurich, Zurich, CH-8057 Switzerland; 2 Environmental Genomics and Systems Biology Division, Lawrence Berkeley National Laboratory, Berkeley, CA 94720 USA; 3 Department of Plant and Microbial Biology, University of California, Berkeley, CA 94720 USA

**Keywords:** Rhizobium, Secretion systems, Large megaplasmid, Chromosome, Killing, Biotechnology, Genetics, Microbiology, Molecular biology

## Abstract

*Paraburkholderia sabiae* LMG24235^T^ is a beta-proteobacterium originally isolated from *Mimosa caesalpiniifolia* nodules that efficiently outcompetes phytopathogens both in vitro and *in planta*. Here we show that *P. sabiae* LMG24235^T^ can spontaneously lose a 2.3-Mbp replicon, resulting in a reduction of genome size by approximately one quarter and representing the first reported case of a bacterium losing 23% of its genome in a single event. Phylogenetic analysis of the replication initiation protein A (RepA) revealed that this replicon is closely related to megaplasmids found in other *Paraburkholderia* strains and a randomly barcoded transposon mutagenesis (RB-TnSeq) analysis showed that it contains very few predicted likely-essential genes, all of which are dispensable for growth. In contrast to the wild-type strain, *P. sabiae* lacking the second replicon (Δ2) exhibited a growth defect on D-trehalose and L-leucine, reduced motility and decreased survival under low-oxygen conditions. While growth in rich medium was otherwise unaffected, *P. sabiae* Δ2 was outcompeted by the wild type in vitro, and *M. caesalpiniifolia* nodules occupied by Δ2 fixed 40% less nitrogen compared to wild-type nodules. These results suggest that the second replicon is not an essential chromosomal element but rather a dispensable large megaplasmid that confers a competitive advantage to *P. sabiae* LMG24235^T^ in its natural environment.

**Supplementary Information:**

The online version contains supplementary material available at 10.1038/s41598-026-49026-9.

## Introduction

*Paraburkholderia* is one of several genera in the family *Burkholderiaceae*, which is well recognized for its high metabolic versatility and capability to thrive in different environmental niches. Established in 2014, this genus within the Pseudomonadota phylum comprises more than 95 species isolated from soil, water, fungi, and plants^1^. *Paraburkholderia* species have considerable agricultural and biotechnological potential, since they display notable features including the ability to promote plant growth, degrade complex organic compounds, and inhibit the growth of phytopathogens by producing antimicrobial agents^2^. They can colonize the endophytic tissues of crops such as tomato, maize, potato, and rice, particularly the root tissues. They are known for their potential to fix nitrogen^3–16^, improve nutrient uptake^2^ and produce phytohormones^17^. Most *Paraburkholderia* species possess large genomes compared to other bacteria, generally exceeding 7 megabase pairs (Mbp), with many strains ranging from approximately 8 to more than 11 Mbp. Examples include *Paraburkholderia phymatum* STM815^T^ (8.7 Mbp), *Paraburkholderia xenovorans* LB400 (9.7 Mbp), and *Paraburkholderia caribensis* MWAP64 (9.03 Mbp). *Paraburkholderia* genomes are often organized into multiple replicons, including several chromosomes and plasmids^18–20^.

Some *Paraburkholderia* members can fix atmospheric nitrogen through symbiotic association with legumes and hence are classified as beta-rhizobia. The genes required to establish symbiosis with plants are usually localized on a dedicated plasmid. In rhizobia belonging to the Alphaproteobacteria (i.e. alpha-rhizobia), it was shown that these symbiotic plasmids can be lost when the strains are grown in non-selective environments, such as an in vitro culture^21,22^. Most rhizobial *Paraburkholderia* species (e.g. *Paraburkholderia mimosarum*,* Paraburkholderia tuberum*, and *P. phymatum*) were isolated from nodules of *Mimosa* plants in South America and Southeast Asia^23–26^, but members of this group have also been isolated from legumes native to the South African Fynbos^12,27–29^. Importantly, beta-rhizobia consistently outcompete alpha-rhizobia in nodulating *Mimosa* species^11,13,30^, a dominance shaped by genetic and environmental factors. *Paraburkholderia sabiae* LMG24235^T^ was isolated from nodules of *Mimosa caesalpiniifolia* in Brazil^8^ and has since been shown to inhibit the growth of important phytopathogens, such as *Pseudomonas syringae*,* Burkholderia gladioli*,* Ralstonia solanacearum* and *Pectobacterium carotovorum*^31^. Additionally, *P. sabiae* was observed to protect potato tubers from bacterial soft rot disease caused by *P. carotovorum*^31^. Sequencing of *P. sabiae* genome revealed the presence of four replicons, provisionally classified as chromosomes 1 (6.58 Mbp), chromosome 2 (2.3 Mbp), plasmid pSymb (0.615 Mbp) containing the symbiotic genes, and plasmid 2 (0.399 Mbp). A type VI secretion system (T6SS) on chromosome 2, T6SS-1, was previously demonstrated to be essential for outcompeting bacterial phytophathogens^31^. In the present work, we characterized a *P. sabiae* LMG24235^T^ derivative that spontaneously lost its ability to kill pathogens in vitro.

Genome sequencing revealed the complete loss of *P. sabiae*’s 2.3-Mbp replicon, suggesting that this replicon is a megaplasmid rather than a true essential chromosome. This assessment is additionally supported by analyses examining the replicon’s replication and partitioning features and essential gene content. We further investigated the functional consequences of this large-scale genome reduction, showing that the megaplasmid contributes to metabolic versatility, motility, competitiveness, and symbiotic nitrogen fixation. Overall, this study reports a striking large-scale spontaneous genome reduction event and examines the impact of this loss on bacterial physiology and symbiosis, suggesting that the deleted genes are either redundant or conditionally beneficial, with loss resulting in a more streamlined but less versatile genome.

## Materials and methods

### Bacterial strains, media, and culture conditions

All bacteria strains, plasmids, oligonucleotides and primers are listed in Supplementary Table 1. If not stated otherwise, *Paraburkholderia* strains were grown aerobically at 28 °C either stationary or shaking at 180 rpm in LB without salt (LBNS) or AB minimal medium^32^ supplemented with 15 mM potassium succinate as a carbon source (ABS). For antibiotic-resistant *Paraburkholderia* strains, chloramphenicol (Cm) was used at 80 µg/mL, gentamicin (Gm) at 20 µg/mL, kanamycin (Km) at 10–50 µg/mL, polymyxin B (pB) at 50 ug/mL, and carbenicillin (Cb) at 50 µg/mL. *Pseudomonas syringae* DC3000 and *Pectobacterium carotovorum* were grown in LBNS with Gm 20 µg/mL. *Escherichia coli* was grown on LB with Cm 20 µg/mL, Gm 10 µg/mL, Cb at 50 µg/mL or Km 25 µg/mL. When needed, diaminopimelic acid (DAP) was added at a final concentration of 300 µM. The spontaneous Δ2 mutant was isolated by picking a single colony from a freshly streaked bacterial plate prepared from the original glycerol stock of *P. sabiae* (see first Results section entitled “Isolation of a *Paraburkholderia sabiae* LMG24235 strain unable to kill phytopathogens that has lost the 2.3 Mbp megaplasmid”).

### Construction of a GFP-tagged *P. sabiae* strain

A *kanR-gfp* construct was inserted between loci QEN71_RS00040 and QEN71_RS00045 (Supplementary Table 1) to generate WT-GFP. Gibson assembly was used to clone *kanR* (p34E-KmR) and *gfp* (pSHAFT-GFP) into pSHAFT2 linearized with XhoI^33^. Assembly reactions were performed with a molar ratio of 1 backbone to 7 of each insert fragment, and the reaction product was transformed into *E. coli* c118 λ-pir^34^. The resulting plasmid pSHAFT2_GFP_sabiae was sequenced using primers pSHAFT_F and pSHAFT_R1 (Supplementary Table 1). After triparental mating using *E. coli* c118 λ-pir carrying pSHAFT2_GFP_sabiae as donor strain, *E. coli* DH5a carrying a pRK2013 (Invitrogen) as helper strain and *P. sabiae* wild-type as recipient, *P. sabiae* fluorescent colonies were selected on ABS Cm80 Km50 plates to identify deletion mutants with a *kanR-gfp* insert (WT-GFP). A clone of this strain that spontaneously lost the second replicon was identified (*P. sabiae* Δ2-GFP). The genome of WT-GFP was sequenced using a MiSeq i100 system (Illumina, Inc, CA. USA).

### Construction of a *P. sabiae* randomly barcoded transposon mutant library (RB-TnSeq)

The *P. sabiae* RB-TnSeq library was constructed via conjugation with *E. coli* WM3064 harboring the pHLL250 *mariner* transposon vector library (strain AMD290)^35^. Briefly, *P. sabiae* was grown overnight in 50 mL of LBNS at 28 °C. The next morning, a 2-mL freezer stock of strain AMD290 (DAP auxotroph) was recovered in 50 mL LB with Cb50 and DAP at 37 °C. When the OD_600_ of the *E. coli* donor strain reached 1, 20 mL of the culture were harvested and washed three times with LB DAP medium. Then, 20 mL of *P. sabiae* cells (OD_600_=1) were harvested, mixed with the washed donor cells, centrifuged, and resuspended in 0.5 mL LBNS DAP. The resuspension was spotted onto 0.45-µm membrane filters (Millipore Sigma, MA. USA) and incubated at 28 °C on LBNS DAP plates supplemented with 1.5% agar. The next day, the conjugation was scraped from the membrane, resuspended in 10 mL LBNS Km10 and plated at different dilutions on LBNS Km10 plates, which were incubated at 28 °C for 48 h to let visible colonies develop. Thousands of colonies were pooled and grown in LBNS Km10 for two population doublings. Glycerol was then added to a final volume of 15% v/v, multiple 1-mL of library stocks were prepared and frozen at -80 °C, and cell pellets were collected to extract genomic DNA for TnSeq mapping. Genomic locations of transposon insertions were mapped and linked to DNA barcodes using a variation of a previously described TnSeq protocol^36,37^. To map transposon insertions and identify genes that are likely to be essential for viability (or nearly so) in LBNS, a previously established pipeline was used that applies previously published heuristics^38,39^ to distinguish likely-essential genes from genes that are too short or that are too repetitive to map insertions in. Briefly, for each protein-coding gene, the total read density in TnSeq (reads/nucleotides across the entire gene) and the density of insertion sites within the central 10–90% of each gene (sites/nucleotides) was computed. Genes that were very similar to other regions of the genome, or < 275 bp, were excluded. For the remaining genes, read density was normalized by GC content by dividing each gene’s read density by the running median of read density over a window of 201 genes (sorted by GC content). The insertion density was normalized so that the median gene’s value was 1. Protein-coding genes were considered likely-essential if both the normalized insertion density (dens) and the normalized read density (normreads) were under 0.2^39^. Analysis scripts are available at https://bitbucket.org/berkeleylab/feba/.

### Genome sequencing and comparative analyses

Genomic DNA of the *P. sabiae* ∆2 strain was extracted using the GenElute Bacterial Genomic DNA kit (Sigma-Aldrich, MO, USA) and sequenced with MiSeq i100 (Illumina, Inc, CA. USA). Reads were mapped against the *P. sabiae* LMG23245^T^ (NCBI, Genbank accessions CP125295 to CP125298) using the CLC Genomic Workbench v.11.0 (QIAGEN, Aarhus, DEN). Functional classifications was done using eggNOG-Mapper v2 5.0^40^. The *repA* sequence (QEN71_RS30105) was searched (BLASTN) on the NCBI server to the “nr” (non-redundant) database (June 2025). Replicons carrying a homolog with 100% coverage and more than 80% identity were aligned using Proksee^41^. For building a phylogenetic tree, *P. sabiae* RepA homologs with a coverage of 100% and an identity > 92% (BLASTP) were downloaded from NCBI and compared with the Molecular Evolutionary Genetics Analysis software 11 (MEGA11)^42^. Sequence alignment was performed using ClustalW^43^ and phylogenetic inference was conducted using the maximum-likelihood method.

### Phenotypic characterisation of *P. sabiae* Δ2

Aerobic growth of *P. sabiae* wild-type and ∆2 was assessed in 250 mL Erlenmeyer flasks containing 50 mL medium at 28 °C with constant shaking at 180 rpm for 26 h. Colony forming units (CFU) were determined on LBNS and 0.5X LBNS plates. Microoxic growth was tested on modified ABS plates (A component diluted 1:9 with water) using GasPak EZ containers (BD; Becton, Dickinson and Company, Sparks, NV, USA) in which the oxygen level was maintained (6–16% O_2_) with GasPak microaerophilic sachets that were replaced every 24 h. Anaerobic survival was tested as described previously^44^ in ABS media. In vitro swimming motility assays were performed as previously described^45^ and the swimming zone diameter was measured using ImageJ^46^. Competition assays in vitro were performed as previously described^31^. For intraspecies competition assay a ratio of 1:1 was used. The outcome of competition assays was measured both by imaging fluorescence with an Odyssey Fc Imager (LI-COR, USA) and by CFU determination.

### Phenotypic microarrays

BIOLOG phenotype microarrays (PM1 and PM2; BIOLOG Inc, Hayward, CA, USA) were incubated at 28 °C for 5 days. OD_600_ of each well was measured every 12 h using an infinite M200Pro plate reader (Tecan, Männerdorf, CH). Data were normalized to the OD_600_ values measured at the 0 h timepoint for each well. A phenotype was considered different when a minimum of a 50% difference in OD_600_ was observed after 120 h, provided that the lowest value in the wild-type strain was at least 0.25. Validation was done in 96-well microtiter plates with AB liquid media supplemented with the different carbon sources (15 mM potassium succinate, 10 mM citric acid, 10 mM D-glucose, 10 mM L-leucine, 5mM of D-trehalose; Sigma-Aldrich, MO, USA).

## Transmission electron microscopy

Transmission electron microscopy (TEM) was performed using a FEI Tecnai G2 Spirit microscope (Field Electron and Ion Company, NE, USA) at the Center for Microscopy and Image Analysis in the University of Zurich. Acceleration voltage was aligned to 120 kV and a side-mounted digital camera Gatan Orius 1000 (Ametek, PA, USA) was used. *P. sabiae* strains were grown in 3 mL ABS (OD_600_ 0.5) and spotted on a formvar/carbon 300-mesh copper grid (Electron Microscopy Sciences, PE, USA) and stained using 1% w/v uranyl acetate (Sigma-Aldrich, MA, USA). Cell size was measured manually in Fiji^47^.

### Characterization of symbiotic properties

*Mimosa caesalpiniifolia* seeds (Sunshine-Seeds, Ahlen, Germany) were surface sterilized for 15 min in 96% sulfuric acid (H_2_SO_4_, Merk, Darmstadt, Germany). Germinated seedlings were transferred to autoclaved 120 mL amber yogurt jars filled with vermiculite and one tablespoon of sand supplemented with Jensen medium^48^, and were inoculated with the bacterial strain (10^4^ CFU)^13^. Plants were maintained in the greenhouse (25 °C/22°C during day/night, 60% humidity, and 16/8 h light cycle). Nitrogenase activity was measured after 28 days by acetylene reduction assay (ARA)^49,50^. For bacteroid reisolation, two nodules were selected per biological replicate and crushed in 100 µL of 0.5X LBNS containing 25% v/v glycerol and plated on LBNS plates^51^.

### Statistical analysis

Data visualization and analysis was performed in RStudio v2024.04.1 + 748 with R v4.2.0^52^ using *ggplot2*^53,54^, *ggpubr*^55^ and *tidyverse*^56^ packages. For the statistical analyses of competition assay, growth, CFU, and symbiotic properties, a one-way ANOVA with Tukey’s multiple comparison was performed using *stat_compare_means* from the *ggpubr* package. For swimming, anaerobic survival, cell length and nodules occupancy, a Student’s *t-test* was performed.

## Results

### Isolation of a *Paraburkholderia sabiae* LMG24235 strain unable to kill phytopathogens that has lost the 2.3 Mbp megaplasmid

**Fig. 1:**
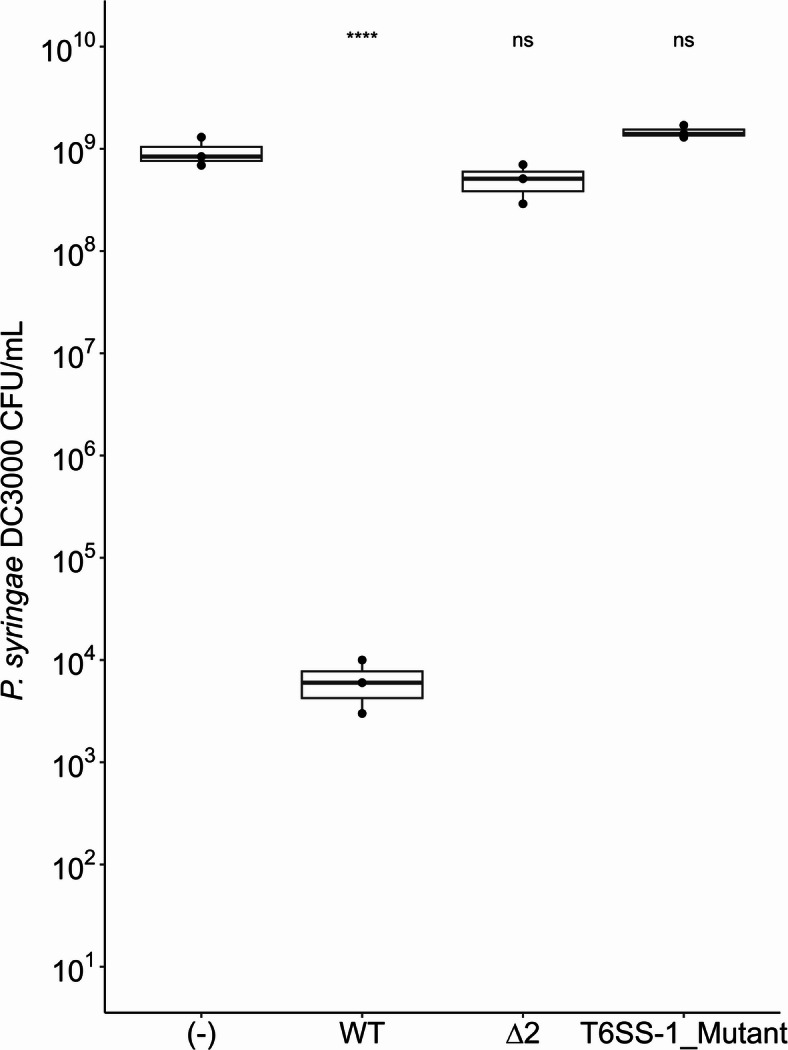
Discovery of a non-killing *P. sabiae* strain. Competition assay performed with *P. sabiae* wild-type (WT), the non-killing strain (Δ2), and a T6SS-1 mutant (T6SS-1) as attackers against *P. syringae* DC3000 as target. CFU/mL of the target strain *P. syringae* DC3000 is shown. Three biological replicates were performed. An ANOVA with Tukey’s multiple-comparison test was performed to assess the statistical significance of differences compared to *P. syringae* incubated without attacker (-) (ns: not significative, *p* ≥ 0.05; ****, *p* ≤ 0.0001).

**Fig. 2:**
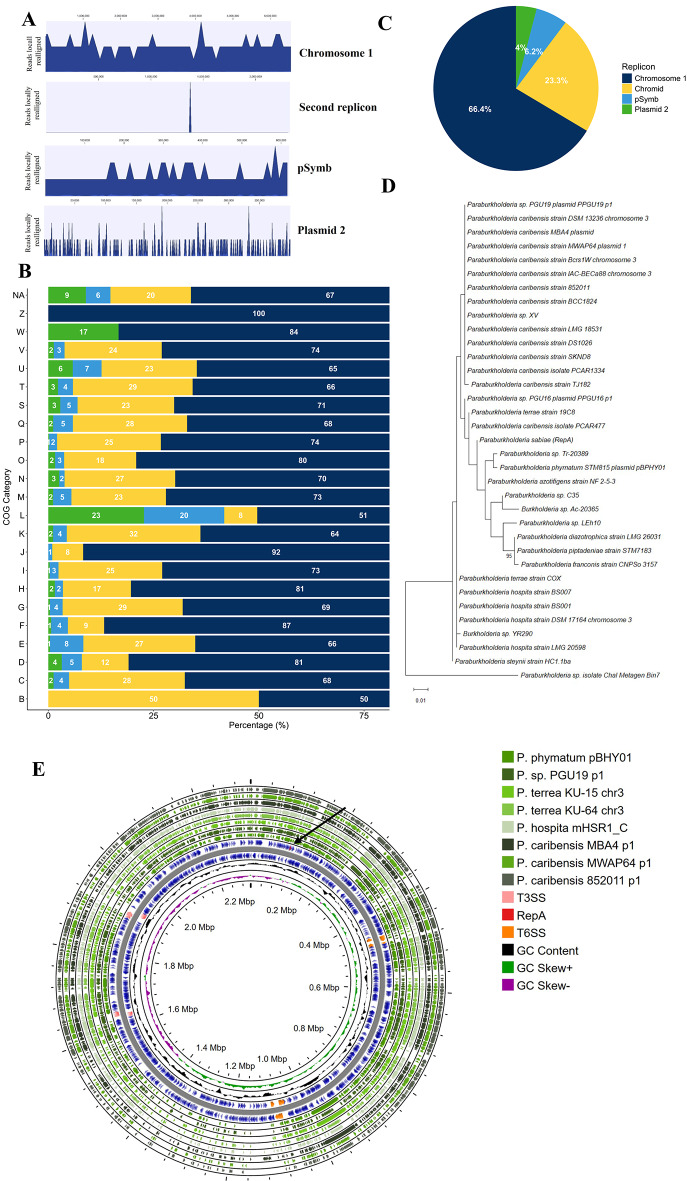
The second replicon is absent in the non-killing *P. sabiae* strain (Δ2) **A**: Mapping of DNA sequencing from non-killing *P. sabiae* (Δ2). **B**: Distribution of COG categories across the *P. sabiae* genome using eggNOG-Mapper v2 5.0. Different colors represent the different replicons. Green is the plasmid 2, turquoise is pSymb, yellow is second replicon (megaplasmid), and dark blue is chromosome 1. The different categories are indicated by the following letters: B, chromatin structure and dynamics; C, energy production and conversion; D, cell cycle control, cell division, chromosome partitioning; E, amino acid transport and metabolism; F, nucleotide transport and metabolism; G, carbohydrate transport and metabolism; H, coenzyme transport and metabolism; I, lipid transport and metabolism; J, translation, ribosomal structure and biogenesis; K, transcription; L, replication, recombination and repair; M, cell wall/membrane/envelope biogenesis; N, cell motility; O, posttranslational modification, protein turnover, chaperone; P, inorganic ion transport and metabolism; Q, secondary metabolites biosynthesis, transport and catabolism; R, general function prediction only; S, function unknown; T, signal transduction mechanisms; U, intracellular trafficking, secretion, and vesicular transport; V, defence mechanisms; W, extracellular structures; Z, cytoskeleton; NA, not categorized. **C**: Size of the four replicons displayed in % compared to the whole genome size (9.9 Mb total genome, chromosome 1: 6.5 Mb, second replicon (megaplasmid): 2.3 Mbp, pSym: 6.15 Kbp, plasmid 2: 3.99 Kbp). **D**: Maximum likelihood phylogenetic tree based on RepA amino acid sequence. **E**: The closest replicons containing homologs of *repA* on *P. sabiae* LMG24235^T^ second replicon were aligned using Proksee. *P. sabiae* wild-type second replicon is used as a backbone (dark blue). Different green shades indicate the different replicon of each species. The GC content is shown in black. *P. sabiae* LMG23245^T^ *repA* (QEN71_RS30105) is shown in red, indicated by the black arrow.

We previously showed that *P. sabiae* LMG24235^T^ efficiently inhibits *P. syringae* pv. tomato DC3000 growth in a T6SS-1-dependent manner^31^. During a standard competition experiment, we observed that *P. sabiae* had lost its ability to kill DC3000 (Pto) (Fig. 1, Supplementary Fig. 1). PCR with primers targeting the T6SS-1 gene cluster on chromosome 2 (Supplementary Table 1) confirmed the absence of the T6SS-1 cluster in this spontaneously obtained strain. However, genes present on chromosome 1, plasmid 1 and plasmid 2 (QEN71_RS43065) could be amplified (Supplementary Fig. 2), suggesting that the non-killing *P.sabiae* strain had lost its second replicon. Genome sequencing confirmed that the non-killing strain had lost chromosome 2 (Fig. 2A), which contains 23% of the genes in the *P. sabiae* genome and was therefore named *P. sabiae* Δ2.

Given its dispensability, we propose to refer to the second replicon as a large megaplasmid rather than a chromosome. Moreover, as previously reported by our group, this replicon lacks rRNA genes^31^.

To assess the general function encoded in the second replicon, the representation of each Cluster of Orthologous Group (COG) category on every *P. sabiae* replicon was analysed using the eggNOG database^40,57^. Category K was over-represented on the second replicon compared to chromosome 1 (Fig. 2B, Supplementary Fig. 3). Moreover, genes related to carbohydrate transport and metabolism (category G), signal transduction mechanisms (T), amino acid transport and metabolism (E), energy production and conversion (C), lipid transport and metabolism (I), inorganic ion transport and metabolism (P), motility (N) and secondary metabolites biosynthesis, transport and catabolism (Q) were over-represented on the second replicon (Supplementary Fig. 3). In contrast, genes involved in translation, ribosomal structure and biogenesis (J) and nucleotide transport and metabolism (F) were under-represented on the second replicon (Fig. 2B, Supplementary Fig. 3). The number of genes belonging to the replication, recombination and repair category (L) was also low on the second replicon (8%) compared to the rest of the genome (Fig. 2C).

Bioinformatic analysis of the replication and partitioning system of the second replicon revealed the presence of *repA* (QEN71_RS30105), *parA* (QEN71_RS30110) and *parB* (QEN71_RS30115). Together, these genes enable the regulated partitioning of multipartite genomes such as those found in *Burkholderia* species^58,59^. RepA (QEN71_RS30105) of the *P. sabiae* second replicon showed high similarity to RepB of plasmid pBPHY01 from *P. phymatum* STM815^T^ (PHY_RS35745, 100% coverage and 87.13% identity, BLASTN) and the phylogenetic analysis suggests a shared evolutionary origin (Fig. 2D). Alignment of replicons of closely related *Paraburkholderia* strains carrying highly similar *repA* genes showed strong conservation of genes adjacent to the replication origin, whereas genes located on the opposite side of the replication origin showed greater variability (Fig. 2E). Consistently, the second replicon of *P. sabiae* displayed high similarity to megaplasmids identified in other *Paraburkholderia* strains (Fig. 2E, Supplementary Fig. 4).

### Randomly barcoded transposon mutagenesis sequencing (RB-TnSeq) supports the non-essentiality of the large megaplasmid

**Fig. 3:**
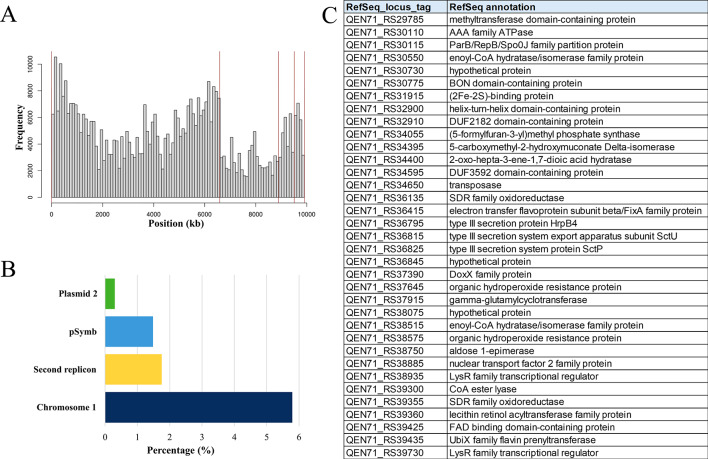
Genome-wide mutant profiling by random barcoded transposon mutagenesis (RB Tn-Seq) in *P. sabiae* LMG24235^T^ reveals a lower percentage of essential genes on dispensable replicons. **A**: Histogram of transposon insertions in the *P. sabiae* _ML3 mutant library. Red lines indicate scaffold boundaries (replicons). **B**: Percentage of genes classified as likely essential on each replicon based on Tn-Seq. **C**: List of the 35 genes classified as likely essential on the second replicon. A complete list of genes is provided in Supplementary Table 2.

To identify essential genes in *P. sabiae* LMG24235^T^, we constructed a RB-TnSeq mutant library (*Psabiae* _ML3) with 470,292 uniquely barcoded strains at 159,151 locations distributed across the genome (Fig. 3A). Of the 8,591 predicted protein-coding genes in *P. sabiae* LMG24235^T^, we generated at least one centrally-located mutation in 7,764 genes (90.3% of the genome). For the remaining 827 genes we do not have gene data, which can be due to reasons such as gene size, gene duplicates, GC content, low sequencing coverage, or chance^39^. To identify which of those 827 genes are likely essential for viability (or nearly so) in the conditions in which we constructed the library (i.e. LBNS), we used a previously established pipeline^39^. Genes with redundancies or duplicates (dupScore > 0) were ignored. Genes that are protein-coding, with dupScore = 0 and ≥ 275 bp were classified as likely-essential or non-essential according to insertion density (Supplementary Table 2). In total, 336, 35, 7 and 1 genes were classified as likely essential (ess=TRUE, Supplementary Table 2) in chromosome 1, second replicon, pSymb and plasmid 2. This accounts for 5.79, 1.75, 1.48 and 0.3% of genes encoded on each replicon, respectively (Fig. 3B and C).

The 35 genes identified as likely essential on the second replicon (Fig. 3C, Supplementary Table 2) showed normalized insertion density (dens) and normalized read density (normreads) values below 0.2. This indicates that these genes are relatively small and/or have a low density of transposon insertion sites, potentially due to high GC content, as the *mariner* transposon inserts into TA sites. Examples among these 35 genes include the *parA* and *parB* (QEN71_RS30110-QEN71_RS30115), Type III secretion system (T3SS) related genes (QEN71_RS36795, QEN71_RS36815, QEN71_RS36825) and LysR-type regulators (QEN71_RS38935, QEN71_RS39730). Given that the second replicon is dispensable, these genes are not truly essential (see Discussion).

### Absence of the second replicon leads to changes in carbon source utilization and to decreased survival in microoxic growth conditions

**Fig. 4:**
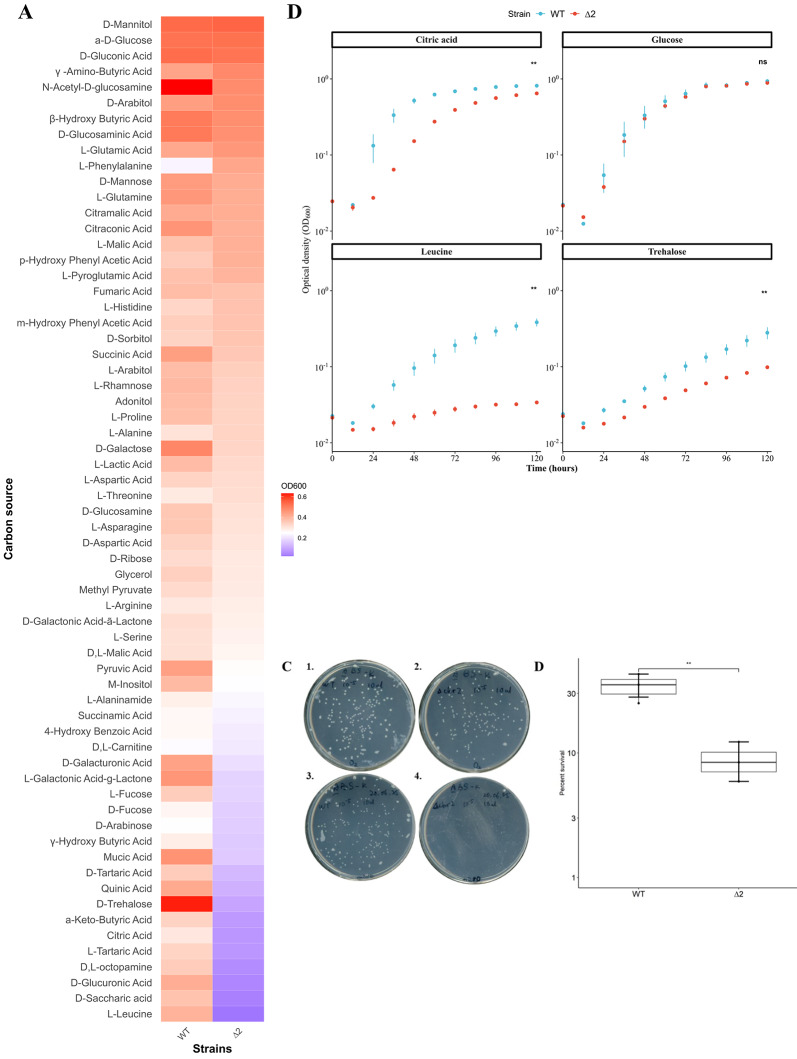
Carbon source utilization of *P. sabiae* wild-type (WT) and Δ2 **A**: Heatmap from the growth of *P. sabiae* WT and Δ2 in BIOLOG Phenotype Microarrays with different carbon sources (PM1-2). **B**: Growth of *P. sabiae* WT (blue) and Δ2 (red) in AB minimal media with different carbon sources (citric acid, glucose, leucine, trehalose) (*N*=3, *n*=2, Wilcox test, ns: not significative, *p-*value > 0.05, **p-value ≤ 0.01). **C**: Growth of *P. sabiae* wild-type (1 and 3), and Δ2 (2 and 4) on ABS plates in aerobic (1 and 2) or microoxic conditions (2 and 4) for 4 days. **D**: Percentage survival of *P. sabiae* wild-type (WT) and Δ2 under anoxic growth conditions in ABS media for 5 days (*N*= 3, *n*=2, *t-test*, **p-value ≤ 0.01).

In complex and minimal media (ABS), *P. sabiae* Δ2 is not affected in growth (Supplementary Fig. 5). However, genes important for carbohydrate and amino acid metabolism and transport are over-represented on the second replicon (Fig. 2B). To assess the metabolic functions encoded by the second replicon, *P. sabiae* Δ2 and wild-type strain were grown on 190 different carbon sources (PM1 and PM2 plates) (Fig. 4A). *P. sabiae* Δ2 was impaired in growth in the presence of D-trehalose, citric acid and L-leucine as a sole carbon sources. To confirm these results, *P. sabiae* Δ2 was grown with AB minimal medium supplemented with 10 mM citric acid, 10 mM L-leucine, or 5 mM of D-trehalose. Glucose (10 mM) was used as negative control (Fig. 4B). While *P. sabiae* Δ2 could not use L-leucine as a carbon source and was impaired in growth on D-trehalose as compared to *P. sabiae* wild-type (Fig. 4B), the growth defect of the Δ2 strain in citric acid was just delayed. As previously mentioned, several genes important for energy production and conservation (C) are over-represented on the second replicon. A search for terminal cytochrome oxidases in the *P. sabiae* genome revealed two gene clusters on the second replicon potentially encoding terminal oxidases (QEN71_RS37515-QEN71_RS37520 and QEN71_RS36885-QEN71_RS36900). These clusters resemble gene clusters in *Burkholderia cenocepacia* H111: QEN71_RS37515-QEN71_RS37520 is similar to the cyanide-insensitive *bd*-type terminal oxidase Cio-2 (plasmid 3) and QEN71_RS36885-QEN71_RS36900 to the heme-copper cytochrome *bo*_3_ ubiquinol oxidase^60^. Accordingly, microoxic growth of *P. sabiae* Δ2 on minimal medium plates containing succinate as carbon source was much slower compared to the wild type (Fig. 4C: 3–4). After 4 days of incubation in a microoxic jar, only *P. sabiae* wild-type colonies were visible. *P. sabiae* Δ2 colonies appeared only after 6 days of incubation. This growth delay was not observed on minimal medium plates under aerobic conditions (Fig. 4C: 1–2). We also looked at the ability of the obligate aerobe *P. sabiae* wild-type and Δ2 to survive under anaerobic conditions in minimal medium and observed that wild-type cells had a survival rate of 30%, while the survival rate of *P. sabiae* Δ2 dropped to 10% (Fig. 4D). These results demonstrate that the second replicon provides *P. sabiae* with a growth advantage in minimal media and in environments with low or no oxygen availability.

### *P. sabiae* Δ2 is impaired in swimming

**Fig. 5:**
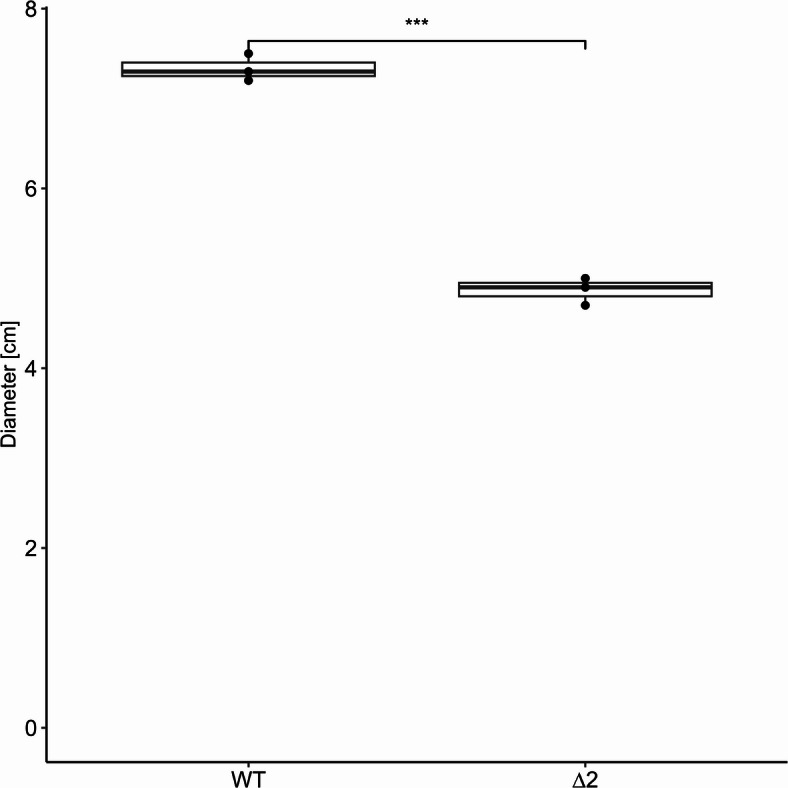
Swimming motility of *P. sabiae* wild-type (WT) and Δ2 in 0.1X LBNS. The diameter of each halo was measured after 48 h incubation at 28 °C (*N* = 3, *n* = 2, *t-test* ***p-value ≤ 0.0001).

Since 27% of the *P. sabiae* genes involved in motility (Category N) are present on the second replicon, the ability of *P. sabiae* Δ2 to move was assessed and compared to the wild-type strain. In complex medium, swimming ability of *P. sabiae* Δ2 was reduced by 40% compared to the wild type (Fig. 5). To determine if this effect was due to impacts on cell morphology, the wild type and the *P. sabiae* Δ2 mutant were examined by TEM. Both strains possess one or more flagella (Supplementary Fig. 6A-B). However, the cell length of *P. sabiae* Δ2 was significantly shorter than that of the wild type (Supplementary Fig. 6C). Bioinformatic analysis revealed that 16 genes related to flagellar synthesis and regulation are present on the second replicon. In comparison, chromosome 1 contains three times as many flagellar genes, including the main components (*fliE*,* fliF*,* fliGHIJKLMNOPQR*), and *fliA*, a flagellum-specific sigma factor. All flagellar genes present on the second replicon are also found on chromosome 1.

### *P. sabiae* wild-type outcompetes Δ2 in vitro

**Fig. 6:**
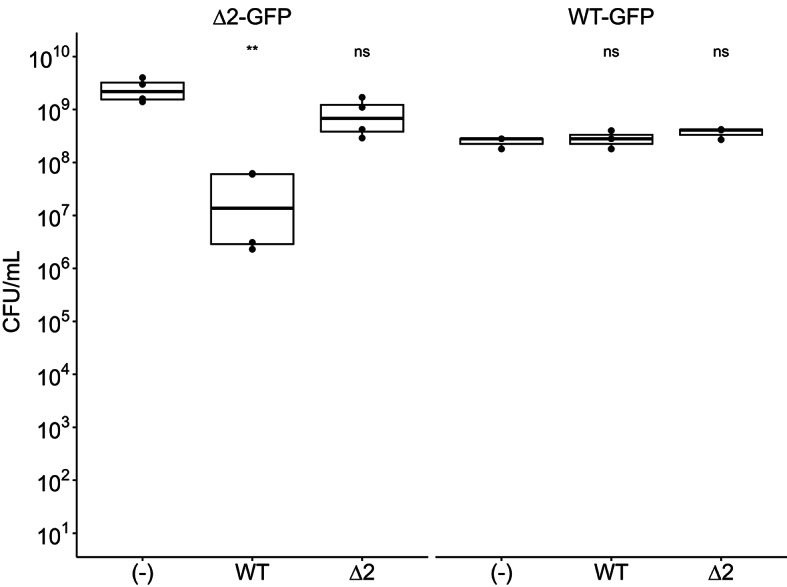
Competition assay between *P. sabiae* wild-type and the Δ2 mutant. CFU of the GFP-tagged *P. sabiae* wild-type (WT-GFP) and Δ2 mutant (Δ2-GFP) in competition against *P. sabiae* wild-type (WT) and *P. sabiae* Δ2 mutant (Δ2) is indicated. The starting ratio is 1:1. An ANOVA with Tukey’s multiple-comparison test was performed and the differences compared to *P. sabiae* wild-type (WT-GFP) and Δ2 mutant (Δ2-GFP) incubated without attacker (-) (ns, *P* ≥ 0.05; **, *P* ≤ 0.01).

The second replicon is enriched in secretion systems, including two T6SS and two T3SS. These secretion systems are large multiprotein molecular complexes, and producing and maintaining them carries a high energy burden for the cell. To evaluate if the Δ2 strain was affected in intraspecific competition, we tested a GFP-tagged *P. sabiae* wild-type (WT-GFP) against a *P. sabiae* Δ2 mutant, and *P. sabiae* wild-type against a GFP-tagged Δ2 (Δ2-GFP). *P. sabiae* wild-type outcompeted the Δ2 strain by 2 orders of magnitude (Fig. 6). The presence of GFP did not affect competitive ability. These results show that the absence of the second replicon reduces the competitiveness of the *P. sabiae* Δ2 strain.

### *P. sabiae* Δ2 enters symbiosis with *Mimosa caesalpiniifolia* but shows reduced nitrogenase activity

**Fig. 7:**
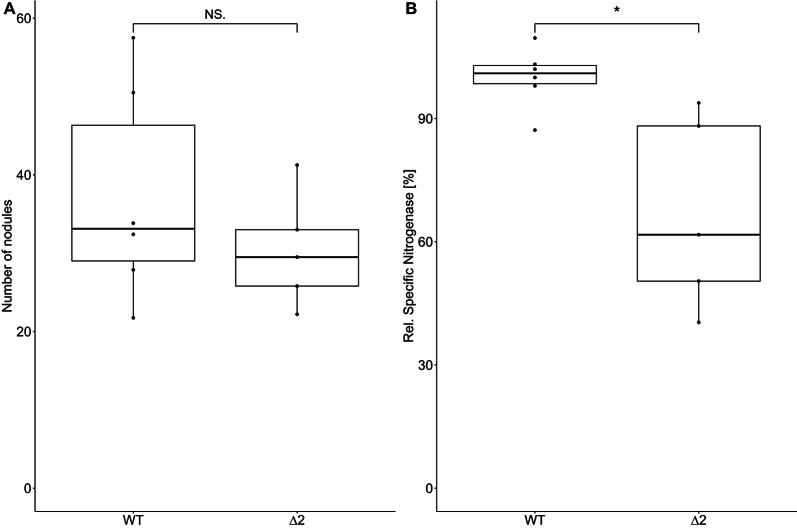
Symbiotic properties of *M. caesalpiniifolia* plants inoculated with *P. sabiae* wild-type (WT) and Δ2. **A**: Number of nodules per plant. **B**: Relative specific nitrogenase activity of *P. sabiae* WT (normalized as 100%) and Δ2. The statistical significance was calculated with one-way ANOVA with Tukey’s test (NS: p-value > 0.05 *: p-value < 0.05).

To evaluate if the absence of the 2.3 Mbp large megaplasmid affects symbiosis, the wild-type and the *P. sabiae* Δ2 mutant strains were inoculated onto *M. caesalpiniifolia*. Twenty-eight days post-inoculation, the number of nodules (Fig. 7A) and their dry weight (not shown) were similar between plants inoculated with wild-type and *P. sabiae* Δ2 strains. However, nitrogenase activity in nodules induced by *P. sabiae* Δ2 was reduced by 35% compared to nodules inoculated with wild-type strain (Fig. 7B). Nodule occupancy analyses further indicated that both strains colonized *M. caesalpiniifolia* nodules at roughly equal rates (Supplementary Fig. 7).

## Discussion

*P. sabiae* LMG24235^T^ possesses a large, multipartite genome, a feature often associated with highly versatile bacteria capable of adapting to diverse ecological niches. Such genome organization is thought to confer advantages for rapid adaptation to changing environments, particularly in bacteria that establish symbiotic relationships with eukaryotic hosts. *P. sabiae* was thought to possess two chromosomes (6.58 Mbp and 2.3 Mbp, respectively) and two plasmids, one of which is a symbiotic plasmid carrying genes required for plant symbiosis and nitrogen fixation. Several secretion systems were identified on the previously annotated second replicon (i.e. chromosome 2), among them the T6SS-1 shown by our group to mediate *P. sabiae’s* killing of phytopathogens^31^. During a competition assay using *P. sabiae* as an attacker, we isolated a mutant that had lost its killing ability. PCR and whole-genome sequencing revealed that this strain had lost its large second replicon of 2.3 Mbp, representing nearly one fourth of its genome. To our knowledge, this represents the largest spontaneous genome reduction reported in a free-living prokaryote. In rhizobia, spontaneous loss of the symbiotic plasmid (ranging from approximately 300 kb to 1.35 Mb) or substantial portions thereof, has been observed in the absence of selective pressure^61,62^. Similarly, within the closely related *Burkholderia cepacia* complex (Bcc), strains lacking the third replicon (typically 0.5–1.1 Mb in size) have been reported^63^. In contrast to these spontaneous genome reduction events, engineered strains with defined large genomic deletions have been generated^62,64–66^.

Closer inspection of the genes encoded on the second replicon revealed the presence of *repA*, *parA* and *parB*, which are important for plasmid replication and partitioning. We show here that *P. sabiae repA* from the second replicon is highly similar to *repA/B* genes present on megaplasmids in other *Paraburkholderia* strains, such as *P. phymatum* STM815^T^ (pBPHY01)^67^, *P. caribensis* MBA4 and MWA61 (plasmid 1)^68^ and *P. caribensis* DSM13236 (third replicon)^1,69^. Moreover, similarly to the observation by Agnoli and colleagues^63^, the replicons labelled as chromosome 3 in the tree presented in Fig. 2D are most likely plasmids. In contrast to the consistent GC-skew observed on chromosome 1, the GC skew on the second replicon is weak^70,71^, and ribosomal RNA (rRNA) and aminoacyl-tRNA synthesis (tRNA) encoding genes, typically found on essential replicons, are absent. Construction and analysis of an RB-TnSeq library confirmed that the second replicon contains the fewest predicted essential genes (35 in total). However, because the second replicon is dispensable, these genes are not truly essential for viability. For instance, at least 17 genes had non-essential homologues (> 33% homology) in other regions of the *P. sabiae* genome, and *parAB* genes were expected to lack transposon insertions, as disruption of these genes leads to loss of the replicon. Similarly, gene QEN71_RS34595, encoding a homolog of a T6SS immunity protein, would be expected to appear essential, as its deletion would result in self-intoxication. For some of the remaining genes, the reason for their apparent essentiality is unclear. However, it is important to note that the threshold of 0.2 used to classify genes as likely essential is arbitrary and lacks a direct biological basis. Although this criteria has been applied in more than 125 bacterial species to estimate genes that are likely to be essential (or nearly essential) for viability, it is a conservative threshold, meaning that there is a risk that genes are misidentified as likely-essential if (1) they are too short, (2) they have few TA sites (where the transposon lands in), or (3) they have low number of reads (due to sequencing) in the central part of the gene (10–90% of sequence). In conclusion, the TnSeq analysis strongly supports the hypothesis that the vast majority, if not all, of the genes required for growth of *P. sabiae* LMG24235^T^ are encoded on chromosome 1. To further confirm the non-essentiality of the second replicon, we examined whether homologues of the 35 genes predicted as essential were required for viability in other bacteria using the publicly available Fitness Browser website (https://fit.genomics.lbl.gov)^39^, which compiles 9,262 genome-wide RB-TnSeq experiments across 57 bacteria and 2 archaea. For 33 of the 35 genes (Fig. 3C), we identified homologues that were not essential in closely related species, including *Paraburkholderia graminis* OAS925, *Paraburkholderia phytofirmans* PsJN, *Paraburkholderia bryophila* 376MFSha3.1 and *Pseudomonas* species^37,39,72^. We also compared the essential genes detected on each replicon of *P. sabiae* LMG23245^T^ with essential gene sets of *Burkholderia cenocepacia* J2315 and *Burkholderia thailandensis* E264 available in the Database of Essential Genes (DEG, https://tubic.org/deg/public/index.php)^73^, which is based on TnSeq studies. None of the 35 genes predicted as essential on the second replicon overlapped with conserved essential functions, such as replication, DNA repair, transcription, RNA translation and protein maturation^74^. Moreover, whereas *Burkholderia* genomes typically encode for two RpoD sigma factors, with the essential copy usually located on the second replicon^18,75,76^, both *rpoD* genes in *P. sabiae* are localized on chromosome 1. Collectively, our findings are fully consistent with previous observations that chromosome 1 in *Burkholderia sensu lato* strains harbours house-keeping genes constituting the core genome, whereas additional replicons are often dispensable and primarily carry accessory functions^18,63^.

To evaluate the impact of the loss of the second replicon, we phenotypically characterized wild type and the Δ2 strain. We observed that the absence of the second replicon affected the utilization of D-trehalose and L-leucine as carbon sources. Genome inspection revealed the presence of *treF* (QEN71_RS30375) encoding a putative periplasmic alpha-alpha trehalase on the second replicon. Whether this gene itself is critical for trehalose utilization awaits investigation via generation of individual mutant strains. In contrast, genes coding for enzymes involved in L-leucine metabolic pathways were not found on the second replicon. These results could be exploited in the design of a growth medium containing L-leucine and/or D-trehalose as carbon source, selective for the presence of the second replicon.

COG analysis enumerated disparities in gene category representation between replicons that correlate to expected phenotypes in the Δ2 mutant. An enrichment of motility genes (category N) on the second replicon explains why Δ2 swims slower than WT (Fig. 5), while the loss of energy production and conversion genes (category C), also overrepresented on the second replicon, is demonstrated by *P. sabiae* Δ2’s impaired fitness under oxygen limitation (Fig. 4). More specifically, the latter deficit may be due to the loss of cytochromes encoded on second replicon: QEN71_RS37515-RS37520 encodes a potential cyanide-insensitive *bd*-type terminal oxidase (CioAB), while QEN71_RS36885-QEN71_RS36900 encodes a potential heme-copper cytochrome *bo*_3_ ubiquinol oxidase (CyoABCD). Cytochrome bd-type oxidases generally have very high affinity for oxygen, enabling bacteria to maintain aerobic respiration under low-oxygen conditions. CioAB in *B. cenocepacia* H111 is also important for survival in the presence of cyanide-producing bacteria^44^. The presence of this oxidase in *P. sabiae* could give the strain an advantage in niches occupied by cyanide-producing soil bacteria, such as members of the *Pseudomonas* genus. Consistent with this, *P. sabiae* Δ2 was impaired under microoxic conditions, and survival assays under anaerobic conditions showed that *P. sabiae* Δ2 survived less well than the wild type (Fig. 4). Other *bd*-oxidases encoded on chromosome 1 (*cioABX*, QEN71_RS07815-QEN71_RS078309 and *cydABX*, QEN71_RS01605-QEN71_RS016215) likely contribute to residual growth under microoxic conditions, allowing survival within plant nodules.

All bacteria we re-isolated from nodules occupied by *P. sabiae* wild-type strain contained the second replicon, suggesting that the nodule is a selective environment and promotes the maintenance of the second replicon. Future experiments, including dual RNA-sequencing of *M. caesalpiniifolia* nodules colonized by *P. sabiae* may help identify: (i) other genes on the second replicon required for efficient nitrogenase activity and (ii) plant genes responding to the absence of the second replicon. The GFP tagged *P. sabiae* wild-type and Δ2 strains could be used to visualize nodule occupancy of both strains and to test if the second replicon provides a competitive advantage during nodulation. The fact that *in vitro P. sabiae* ∆2 is outcompeted by the wild-type strain suggests that the second replicon enhances *P. sabiae’*s competitiveness. Although this megaplasmid is non-essential, its persistence in the natural environment is likely maintained by strong selective pressure. Therefore, we predict that *P. sabiae* Δ2 would be less competitive in the soil against other bacteria due to the absence of several secretion systems (two T6SS and two uncharacterized T3SS), its reduced swimming motility and its reduced fitness in low oxygen environments. At the same time, the possibility of deleting the second replicon opens new perspectives on the potential advantages conferred by the multipartite genomes of *Paraburkholderia* species, particularly in this genus’ adaptation to diverse environments, competition, colonization of new niches, and host interactions, which can ultimately explain their evolutionary success and ecological adaptability.

## Electronic Supplementary Material

Below is the link to the electronic supplementary material.

Supplementary Material 1

Supplementary Material 2

## Data Availability

The datasets generated and analysed during this study are available in the NCBI short reads archive (SRA) repository, https://www.ncbi.nlm.nih.gov/sra/PRJNA1423734.
