# Supplementary material for: Extensive spontaneous genome reduction in Paraburkholderia sabiae
Source: Sci Rep. 2026 Apr 29;16:19843. doi: 10.1038/s41598-026-49026-9 (PMC13316101; doi:10.1038/s41598-026-49026-9)
Supplement: Supplementary file 1 — Supplementary Material 1 [file 41598_2026_49026_MOESM1_ESM.pdf]

Supplementary Table 2: *Psabiae* _ML3 gene information, including scaffold, begin, end, strand, number of insertions, number of reads, duplicates (dupScore), number of TA sites, GC content

RefSeq_locus_tag	RefSeq annotation	GeneBank_old_l	PGAP_old_locus	type	scaffoldId	replicon	begin	end	strand	ntLenNoOv	dupScore	nPos	nReads	nPosCentra	nReadsCen	essential
QEN71_RS29685	indolepyruvate ferredoxin oxidoreductase family pro	QEN71_29680	paras_006859	protein-codi	NZ_CP1252	chromosom	1	3594	+	3594	0	89	20329	75	17665	
QEN71_RS29690	hypothetical protein	QEN71_29685	paras_006860	protein-codi	NZ_CP1252	chromosom	3699	3842	+	144	0	4	394	2	84	
QEN71_RS29695	TonB-dependent siderophore receptor	QEN71_29690	paras_006861	protein-codi	NZ_CP1252	chromosom	4258	6471	+	2165	0	51	10625	40	8662	
QEN71_RS29700	PepSY-associated TM helix domain-containing prot	QEN71_29695	paras_006862	protein-codi	NZ_CP1252	chromosom	6423	8057	-	1586	0	39	8307	27	5406	
QEN71_RS29705	hypothetical protein	QEN71_29700	paras_006863	protein-codi	NZ_CP1252	chromosom	8062	8376	-	311	0	7	1005	5	533	
QEN71_RS29710	hypothetical protein	QEN71_29705	paras_006864	protein-codi	NZ_CP1252	chromosom	8373	8645	-	269	0	9	1474	8	1343	
QEN71_RS29715	helix-turn-helix transcriptional regulator	QEN71_29710	paras_006865	protein-codi	NZ_CP1252	chromosom	8736	8963	-	228	0	9	2577	9	2577	
QEN71_RS29720	hypothetical protein	QEN71_29715		protein-codi	NZ_CP1252	chromosom	9047	9583	-	537	0	10	1096	8	865	
QEN71_RS29725	aldolase	QEN71_29720	paras_006866	protein-codi	NZ_CP1252	chromosom	9670	10440	-	771	0	5	1060	5	1060	
QEN71_RS29730	MFS transporter	QEN71_29725	paras_006867	protein-codi	NZ_CP1252	chromosom	10471	11799	-	1329	0	27	6002	17	3527	
QEN71_RS29735	FAD-dependent monooxygenase	QEN71_29730	paras_006868	protein-codi	NZ_CP1252	chromosom	11933	13522	-	1590	0	32	5573	29	5254	
QEN71_RS29740	catalase/peroxidase HPI	QEN71_29735	paras_006869	protein-codi	NZ_CP1252	chromosom	13856	16120	+	2265	0	33	6191	27	5460	
QEN71_RS29745	enolase C-terminal domain-like protein	QEN71_29740	paras_006870	protein-codi	NZ_CP1252	chromosom	16371	17516	+	1146	0	29	3194	22	2835	
QEN71_RS29750	hypothetical protein	QEN71_29745	paras_006871	protein-codi	NZ_CP1252	chromosom	17593	18534	+	934	0	12	2506	10	1911	
QEN71_RS29755	gluconate 2-dehydrogenase subunit 3 family protein	QEN71_29750	paras_006872	protein-codi	NZ_CP1252	chromosom	18527	19183	+	638	0	14	3336	10	2525	
QEN71_RS29760	GMC family oxidoreductase	QEN71_29755	paras_006873	protein-codi	NZ_CP1252	chromosom	19173	20816	+	1633	0	34	8063	29	6995	
QEN71_RS29765	SDR family oxidoreductase	QEN71_29760	paras_006874	protein-codi	NZ_CP1252	chromosom	20862	21878	+	1017	0	19	2802	14	2432	
QEN71_RS29770	hypothetical protein	QEN71_29765	paras_006875	protein-codi	NZ_CP1252	chromosom	21899	22174	-	276	0	11	797	10	754	
QEN71_RS29775	phosphate signaling complex protein PhoU	QEN71_29770	paras_006876	protein-codi	NZ_CP1252	chromosom	22504	23211	+	708	0	13	2211	9	1224	
QEN71_RS29780	response regulator	QEN71_29775	paras_006877	protein-codi	NZ_CP1252	chromosom	23414	24040	+	627	0	8	1843	3	623	
QEN71_RS29785	methyltransferase domain-containing protein	QEN71_29780	paras_006878	protein-codi	NZ_CP1252	chromosom	24229	25038	+	810	0	4	74	2	10	TRUE
QEN71_RS29790	ABC transporter substrate-binding protein	QEN71_29785	paras_006879	protein-codi	NZ_CP1252	chromosom	25090	26172	+	1083	0	9	541	6	366	
QEN71_RS29795	ABC transporter permease	QEN71_29790	paras_006880	protein-codi	NZ_CP1252	chromosom	26184	27041	+	858	0	7	1076	7	1076	
QEN71_RS29800	ABC transporter permease	QEN71_29795	paras_006881	protein-codi	NZ_CP1252	chromosom	27065	27964	+	900	0	12	507	12	507	
QEN71_RS29805	ABC transporter ATP-binding protein	QEN71_29800	paras_006882	protein-codi	NZ_CP1252	chromosom	27991	28848	+	858	0	8	679	6	514	
QEN71_RS29810	LLM class flavin-dependent oxidoreductase	QEN71_29805	paras_006883	protein-codi	NZ_CP1252	chromosom	28886	30256	+	1371	0	16	1360	9	1021	
QEN71_RS29815	cytochrome c	QEN71_29810	paras_006884	protein-codi	NZ_CP1252	chromosom	30318	30920	-	595	0	12	1567	8	1155	
QEN71_RS29820	sulfite dehydrogenase	QEN71_29815	paras_006885	protein-codi	NZ_CP1252	chromosom	30913	32190	-	1247	0	17	1449	12	1019	
QEN71_RS29825	amino acid ABC transporter permease	QEN71_29820	paras_006886	protein-codi	NZ_CP1252	chromosom	32168	32908	-	714	0	14	1120	12	1070	
QEN71_RS29830	amino acid ABC transporter permease	QEN71_29825	paras_006887	protein-codi	NZ_CP1252	chromosom	32905	33636	-	728	0	13	1829	11	1396	
QEN71_RS29835	ABC transporter substrate-binding protein	QEN71_29830	paras_006888	protein-codi	NZ_CP1252	chromosom	33644	34462	-	819	0	6	274	5	267	
QEN71_RS29840	efflux transporter outer membrane subunit	QEN71_29835	paras_006889	protein-codi	NZ_CP1252	chromosom	34591	36084	-	1490	0	24	2033	19	1615	
QEN71_RS29845	efflux RND transporter permease subunit	QEN71_29840	paras_006890	protein-codi	NZ_CP1252	chromosom	36081	39173	-	3085	0	48	3216	39	2494	
QEN71_RS29850	efflux RND transporter permease subunit	QEN71_29845	paras_006891	protein-codi	NZ_CP1252	chromosom	39170	42283	-	3110	0	26	2771	24	2737	
QEN71_RS29855	efflux RND transporter periplasmic adaptor subunit	QEN71_29850	paras_006892	protein-codi	NZ_CP1252	chromosom	42308	43453	-	1146	0	15	1043	11	889	
QEN71_RS29860	efflux transporter outer membrane subunit	QEN71_29855	paras_006893	protein-codi	NZ_CP1252	chromosom	43643	45154	-	1512	0	32	2927	23	1960	
QEN71_RS29865	efflux RND transporter permease subunit	QEN71_29860	paras_006894	protein-codi	NZ_CP1252	chromosom	45169	48327	-	3159	0	40	3192	34	2588	
QEN71_RS29870	efflux RND transporter periplasmic adaptor subunit	QEN71_29865	paras_006895	protein-codi	NZ_CP1252	chromosom	48342	49541	-	1200	0	17	3463	17	3463	
QEN71_RS29875	TetR family transcriptional regulator	QEN71_29870	paras_006896	protein-codi	NZ_CP1252	chromosom	49772	50407	+	636	0	5	1040	5	1040	
QEN71_RS29880	response regulator	QEN71_29875	paras_006897	protein-codi	NZ_CP1252	chromosom	50592	51359	+	764	0	8	696	5	256	
QEN71_RS29885	ATP-binding protein	QEN71_29880	paras_006898	protein-codi	NZ_CP1252	chromosom	51356	52609	+	1250	0	23	2537	18	2328	
QEN71_RS29890	RcnB family protein	QEN71_29885	paras_006899	protein-codi	NZ_CP1252	chromosom	52670	53074	-	405	0	9	402	4	127	
QEN71_RS29895	hypothetical protein	QEN71_29890	paras_006900	protein-codi	NZ_CP1252	chromosom	53176	53838	-	663	0	13	454	13	454	
QEN71_RS29900	glycine zipper family protein	QEN71_29895	paras_006901	protein-codi	NZ_CP1252	chromosom	53847	54776	-	926	0	35	3481	27	2957	
QEN71_RS29905	PAAR domain-containing protein	QEN71_29900	paras_006902	protein-codi	NZ_CP1252	chromosom	54773	55324	-	548	0	11	456	9	425	
QEN71_RS29910	hypothetical protein	QEN71_29905	paras_006903	protein-codi	NZ_CP1252	chromosom	55671	56195	+	525	0	5	319	4	298	
QEN71_RS29915	DUF899 domain-containing protein	QEN71_29910	paras_006904	protein-codi	NZ_CP1252	chromosom	56399	57184	+	786	0	17	1664	16	1644	
QEN71_RS29920	DUF4136 domain-containing protein	QEN71_29915	paras_006905	protein-codi	NZ_CP1252	chromosom	57212	57805	-	594	0	17	1913	17	1913	
QEN71_RS29925	CHASE2 domain-containing protein	QEN71_29920	paras_006906	protein-codi	NZ_CP1252	chromosom	57846	60287	-	2441	0	44	9609	35	7868	
QEN71_RS29930	FecR domain-containing protein	QEN71_29925	paras_006907	protein-codi	NZ_CP1252	chromosom	60287	61675	-	1384	0	25	6165	13	3589	
QEN71_RS29935	response regulator transcription factor	QEN71_29930	paras_006908	protein-codi	NZ_CP1252	chromosom	61672	62370	-	695	0	11	3206	5	1399	
QEN71_RS29940	hypothetical protein	QEN71_29935	paras_006909	protein-codi	NZ_CP1252	chromosom	62991	63263	+	273	0	2	96	0	0	
QEN71_RS29945	3-hydroxyacyl-CoA dehydrogenase NAD-binding do	QEN71_29940	paras_006910	protein-codi	NZ_CP1252	chromosom	63447	65567	-	2121	0	29	5024	23	4452	
QEN71_RS29950	LysR family transcriptional regulator	QEN71_29945	paras_006911	protein-codi	NZ_CP1252	chromosom	65691	66608	+	918	0	25	4804	24	4796	
QEN71_RS29955	IclR family transcriptional regulator	QEN71_29950	paras_006912	protein-codi	NZ_CP1252	chromosom	66621	67487	-	867	0	14	1702	10	1205	
QEN71_RS29960	sugar phosphate isomerase/epimerase	QEN71_29955	paras_006913	protein-codi	NZ_CP1252	chromosom	67617	68450	-	834	0	8	534	6	302	
QEN71_RS29965	NIPSNAP family protein	QEN71_29960	paras_006914	protein-codi	NZ_CP1252	chromosom	68500	69111	-	608	0	15	2683	11	1249	
QEN71_RS29970	FAD-dependent oxidoreductase	QEN71_29965	paras_006915	protein-codi	NZ_CP1252	chromosom	69108	70859	-	1748	0	21	1661	17	1380	
QEN71_RS29975	NAD(P)-dependent oxidoreductase	QEN71_29970	paras_006916	protein-codi	NZ_CP1252	chromosom	70974	71879	+	906	0	18	1153	14	951	
QEN71_RS29980	Gfo/Idh/MocA family oxidoreductase	QEN71_29975	paras_006917	protein-codi	NZ_CP1252	chromosom	71887	72924	-	1038	0	7	573	5	430	

QEN71_RS29985	ABC transporter substrate-binding protein	QEN71_29980	paras_006918	protein-codi	NZ_CP1252	chromosom	73111	74430	+	1320	0	14	1114	13	1075	
QEN71_RS29990	branched-chain amino acid ABC transporter permea	QEN71_29985	paras_006919	protein-codi	NZ_CP1252	chromosom	74522	75391	+	866	0	12	1765	7	225	
QEN71_RS29995	branched-chain amino acid ABC transporter ATP-bi	QEN71_29990	paras_006920	protein-codi	NZ_CP1252	chromosom	75388	77940	+	2545	0	37	5234	27	3982	
QEN71_RS30000	shikimate dehydrogenase	QEN71_29995	paras_006921	protein-codi	NZ_CP1252	chromosom	77937	78779	+	835	0	7	708	5	630	
QEN71_RS30005	SDR family NAD(P)-dependent oxidoreductase	QEN71_30000	paras_006922	protein-codi	NZ_CP1252	chromosom	78776	79516	+	737	0	3	251	1	73	
QEN71_RS30010	FAD-binding protein	QEN71_30005	paras_006923	protein-codi	NZ_CP1252	chromosom	79565	81334	+	1770	0	22	2920	18	1972	
QEN71_RS30015	organic hydroperoxide resistance protein	QEN71_30010	paras_006924	protein-codi	NZ_CP1252	chromosom	81523	81939	+	417	0	9	911	2	166	
QEN71_RS30020	TetR/AcrR family transcriptional regulator	QEN71_30015	paras_006925	protein-codi	NZ_CP1252	chromosom	81963	82583	-	621	0	7	626	4	283	
QEN71_RS30025	ester cyclase	QEN71_30020	paras_006926	protein-codi	NZ_CP1252	chromosom	82585	83007	-	423	0	8	535	7	492	
QEN71_RS30030	Na+/H+ antiporter	QEN71_30025	paras_006927	protein-codi	NZ_CP1252	chromosom	83204	84787	+	1584	0	24	4845	22	4615	
QEN71_RS30035	extracellular solute-binding protein	QEN71_30030	paras_006928	protein-codi	NZ_CP1252	chromosom	85165	86424	+	1256	0	28	7125	22	5520	
QEN71_RS30040	EAL domain-containing protein	QEN71_30035	paras_006929	protein-codi	NZ_CP1252	chromosom	86421	89357	+	2933	0	46	6675	31	3979	
QEN71_RS30045	DUF4410 domain-containing protein	QEN71_30040	paras_006930	protein-codi	NZ_CP1252	chromosom	89382	90038	-	657	0	13	1899	11	1781	
QEN71_RS30050	(2Fe-2S)-binding protein	QEN71_30045	paras_006931	protein-codi	NZ_CP1252	chromosom	90268	90729	+	462	0	7	1056	7	1056	
QEN71_RS30055	xanthine dehydrogenase family protein molybdopter	QEN71_30050	paras_006932	protein-codi	NZ_CP1252	chromosom	90733	92925	+	2193	0	35	4394	30	3996	
QEN71_RS30060	DUF4148 domain-containing protein	QEN71_30055	paras_006933	protein-codi	NZ_CP1252	chromosom	92993	93316	-	324	0	8	859	2	48	
QEN71_RS30065	efflux RND transporter permease subunit	QEN71_30060	paras_006934	protein-codi	NZ_CP1252	chromosom	93678	96875	+	3198	0	56	7649	46	6070	
QEN71_RS30070	efflux RND transporter periplasmic adaptor subunit	QEN71_30065	paras_006935	protein-codi	NZ_CP1252	chromosom	96885	98123	+	1239	0	19	2364	15	1004	
QEN71_RS30075	efflux transporter outer membrane subunit	QEN71_30070	paras_006936	protein-codi	NZ_CP1252	chromosom	98136	99596	+	1461	0	23	2829	19	2661	
QEN71_RS30080	extracellular solute-binding protein	QEN71_30075	paras_006937	protein-codi	NZ_CP1252	chromosom	99683	100558	+	872	0	19	1757	19	1757	
QEN71_RS30085	ATP-binding cassette domain-containing protein	QEN71_30080	paras_006938	protein-codi	NZ_CP1252	chromosom	100555	102438	+	1880	0	34	6340	30	5968	
QEN71_RS30090	LysR family transcriptional regulator	QEN71_30085	paras_006939	protein-codi	NZ_CP1252	chromosom	102449	103390	-	942	0	32	4723	21	3485	
QEN71_RS30095	MarR family winged helix-turn-helix transcriptional r	QEN71_30090	paras_006940	protein-codi	NZ_CP1252	chromosom	103571	103987	-	417	0	8	1014	5	610	
QEN71_RS30100	MipA/OmpV family protein	QEN71_30095	paras_006941	protein-codi	NZ_CP1252	chromosom	104193	105032	+	840	0	46	14312	36	13715	
QEN71_RS30105	replication initiation protein	QEN71_30100	paras_006942	protein-codi	NZ_CP1252	chromosom	105866	107398	+	1533	0	6	539	4	434	
QEN71_RS30110	AAA family ATPase	QEN71_30105	paras_006943	protein-codi	NZ_CP1252	chromosom	107486	108637	+	1152	0	2	10	1	4	TRUE
QEN71_RS30115	ParB/RepB/Spo0J family partition protein	QEN71_30110	paras_006944	protein-codi	NZ_CP1252	chromosom	108651	109616	+	966	0	1	4	1	4	TRUE
QEN71_RS30120	hypothetical protein	QEN71_30115	paras_006945	protein-codi	NZ_CP1252	chromosom	110255	111391	-	1137	0	15	4504	10	1983	
QEN71_RS30125	DUF445 family protein	QEN71_30120	paras_006946	protein-codi	NZ_CP1252	chromosom	112422	113702	+	1281	0	23	5543	16	4438	
QEN71_RS30130	hypothetical protein	QEN71_30125	paras_006947	protein-codi	NZ_CP1252	chromosom	113710	114033	-	324	0	10	2348	6	1825	
QEN71_RS30135	MOSC domain-containing protein	QEN71_30130	paras_006948	protein-codi	NZ_CP1252	chromosom	114146	114649	+	504	0	8	324	6	295	
QEN71_RS30140	peptide-methionine (R)-S-oxide reductase MsrB	QEN71_30135	paras_006949	protein-codi	NZ_CP1252	chromosom	114673	115125	-	453	0	17	1983	12	1427	
QEN71_RS30145	DNA-3-methyladenine glycosylase I	QEN71_30140	paras_006950	protein-codi	NZ_CP1252	chromosom	115446	116141	+	696	0	5	1077	4	935	
QEN71_RS30150	zinc-finger-containing protein	QEN71_30145	paras_006951	protein-codi	NZ_CP1252	chromosom	116145	116585	-	441	0	7	618	3	69	
QEN71_RS30155	hypothetical protein	QEN71_30150	paras_006952	protein-codi	NZ_CP1252	chromosom	116701	117462	-	762	0	16	3274	12	1858	
QEN71_RS30160	DUF2950 domain-containing protein	QEN71_30155	paras_006953	protein-codi	NZ_CP1252	chromosom	117630	118550	-	921	0	21	2878	19	2784	
QEN71_RS30165	DUF3300 domain-containing protein	QEN71_30160	paras_006954	protein-codi	NZ_CP1252	chromosom	118562	119932	-	1371	0	31	2873	24	2211	
QEN71_RS30170	c-type cytochrome	QEN71_30165	paras_006955	protein-codi	NZ_CP1252	chromosom	120260	121486	-	1227	0	22	2390	14	1575	
QEN71_RS30175	methanol/ethanol family PQQ-dependent dehydroge	QEN71_30170	paras_006956	protein-codi	NZ_CP1252	chromosom	121545	123272	-	1728	0	59	7348	45	5587	
QEN71_RS30180	hypothetical protein	QEN71_30175	paras_006957	protein-codi	NZ_CP1252	chromosom	123653	123895	+	243	0	2	55	2	55	
QEN71_RS30185	pyrroloquinoline quinone biosynthesis peptide chap	QEN71_30180	paras_006958	protein-codi	NZ_CP1252	chromosom	123924	124205	-	282	0	9	970	9	970	
QEN71_RS30190	pyrroloquinoline quinone precursor peptide PqqA	QEN71_30185	paras_006959	protein-codi	NZ_CP1252	chromosom	124282	124356	-	75	0	5	905	5	905	
QEN71_RS30195	SDR family oxidoreductase	QEN71_30190	paras_006960	protein-codi	NZ_CP1252	chromosom	124581	125327	-	747	0	8	537	8	537	
QEN71_RS30200	SDR family oxidoreductase	QEN71_30195	paras_006961	protein-codi	NZ_CP1252	chromosom	125368	126111	-	744	0	8	576	4	336	
QEN71_RS30205	DUF427 domain-containing protein	QEN71_30200	paras_006962	protein-codi	NZ_CP1252	chromosom	126121	126519	-	399	0	14	1908	10	1230	
QEN71_RS30210	SDR family oxidoreductase	QEN71_30205	paras_006963	protein-codi	NZ_CP1252	chromosom	126595	127341	-	747	0	5	969	5	969	
QEN71_RS30215	LysR family transcriptional regulator	QEN71_30210	paras_006964	protein-codi	NZ_CP1252	chromosom	127558	128442	+	885	0	10	1428	5	139	
QEN71_RS30220	hypothetical protein	QEN71_30215	paras_006965	protein-codi	NZ_CP1252	chromosom	128526	129053	-	528	0	3	268	3	268	
QEN71_RS30225	hypothetical protein	QEN71_30220	paras_006966	protein-codi	NZ_CP1252	chromosom	130378	130518	+	141	0	4	433	4	433	
QEN71_RS30230	sigma-54-dependent Fis family transcriptional regul	QEN71_30225	paras_006967	protein-codi	NZ_CP1252	chromosom	130814	132805	-	1992	0	24	2916	16	2239	
QEN71_RS30235	alpha/beta fold hydrolase	QEN71_30230	paras_006968	protein-codi	NZ_CP1252	chromosom	132957	133925	+	937	0	14	1226	10	642	
QEN71_RS30240	hypothetical protein	QEN71_30235	paras_006969	protein-codi	NZ_CP1252	chromosom	133894	134169	-	244	0	4	312	4	312	
QEN71_RS30245	hypothetical protein	QEN71_30240	paras_006970	protein-codi	NZ_CP1252	chromosom	134324	134605	-	282	0	10	1077	10	1077	
QEN71_RS30250	glycine zipper 2TM domain-containing protein	QEN71_30245	paras_006971	protein-codi	NZ_CP1252	chromosom	134759	135532	-	774	0	14	1510	6	534	
QEN71_RS30255	cupin domain-containing protein	QEN71_30250	paras_006972	protein-codi	NZ_CP1252	chromosom	135658	136002	-	345	0	5	1618	2	1189	
QEN71_RS30260	transposase	QEN71_30255	paras_006973	protein-codi	NZ_CP1252	chromosom	136174	136962	+	789	0	12	2181	12	2181	
QEN71_RS30265	DUF4148 domain-containing protein	QEN71_30260	paras_006974	protein-codi	NZ_CP1252	chromosom	137159	137470	+	312	0	11	1432	8	972	
QEN71_RS30270	phosphatase PAP2 family protein	QEN71_30265	paras_006975	protein-codi	NZ_CP1252	chromosom	137573	138175	-	603	0	15	3040	11	2687	
QEN71_RS30275	Rieske 2Fe-2S domain-containing protein	QEN71_30270	paras_006976	protein-codi	NZ_CP1252	chromosom	138249	138593	-	338	0	5	196	4	180	
QEN71_RS30280	alpha/beta hydrolase	QEN71_30275	paras_006977	protein-codi	NZ_CP1252	chromosom	138587	139171	-	578	0	4	430	2	86	
QEN71_RS30285	MFS transporter	QEN71_30280	paras_006978	protein-codi	NZ_CP1252	chromosom	139184	140512	-	1329	0	30	2726	18	1661	
QEN71_RS30290	3-keto-5-aminohexanoate cleavage protein	QEN71_30285	paras_006979	protein-codi	NZ_CP1252	chromosom	140682	141734	-	1053	0	13	1382	12	1362	

QEN71_RS30295	TauD/TfdA family dioxygenase	QEN71_30290	paras_006980	protein-codi	NZ_CP1252	chromosom	141823	142671	-	849	0	15	1371	11	981	
QEN71_RS30300	quinone oxidoreductase	QEN71_30295	paras_006981	protein-codi	NZ_CP1252	chromosom	142673	143656	-	984	0	21	2315	19	2167	
QEN71_RS30305	hemerythrin domain-containing protein	QEN71_30300	paras_006982	protein-codi	NZ_CP1252	chromosom	143669	144595	-	927	0	8	1130	3	260	
QEN71_RS30310	LuxR C-terminal-related transcriptional regulator	QEN71_30305	paras_006983	protein-codi	NZ_CP1252	chromosom	144837	147542	+	2706	0	29	3102	25	2372	
QEN71_RS30315	fatty acid--CoA ligase	QEN71_30310	paras_006984	protein-codi	NZ_CP1252	chromosom	147698	149359	+	1662	0	30	2697	24	2225	
QEN71_RS30320	porin	QEN71_30315	paras_006985	protein-codi	NZ_CP1252	chromosom	149412	150539	+	1128	0	28	2298	22	2092	
QEN71_RS30325	AraC family transcriptional regulator	QEN71_30320	paras_006986	protein-codi	NZ_CP1252	chromosom	150676	151683	+	962	0	16	1107	15	1104	
QEN71_RS30330	hypothetical protein	QEN71_30325	paras_006987	protein-codi	NZ_CP1252	chromosom	151638	152144	-	461	0	4	1189	4	1189	
QEN71_RS30335	SDR family NAD(P)-dependent oxidoreductase	QEN71_30330	paras_006988	protein-codi	NZ_CP1252	chromosom	152603	153304	+	702	0	3	272	2	133	
QEN71_RS30340	DUF839 domain-containing protein	QEN71_30335	paras_006989	protein-codi	NZ_CP1252	chromosom	153467	155446	+	1980	0	39	7625	32	5856	
QEN71_RS30345	hypothetical protein	QEN71_30340	paras_006990	protein-codi	NZ_CP1252	chromosom	155536	155679	-	144	0	5	577	5	577	
QEN71_RS30350	hypothetical protein	QEN71_30345	paras_006991	protein-codi	NZ_CP1252	chromosom	156018	156251	+	234	0	8	1469	5	850	
QEN71_RS30355	bifunctional diguanylate cyclase/phosphodiesterase	QEN71_30350	paras_006992	protein-codi	NZ_CP1252	chromosom	156526	158613	+	2088	0	30	7137	23	4839	
QEN71_RS30360	lysophospholipid acyltransferase family protein	QEN71_30355	paras_006993	protein-codi	NZ_CP1252	chromosom	159035	159745	+	711	0	17	3694	9	2033	
QEN71_RS30365	hypothetical protein	QEN71_30360	paras_006994	protein-codi	NZ_CP1252	chromosom	159788	160264	-	477	0	7	2304	7	2304	
QEN71_RS30370	hypothetical protein	QEN71_30365	paras_006995	protein-codi	NZ_CP1252	chromosom	160459	160698	-	240	0	7	1827	3	1347	
QEN71_RS30375	alpha,alpha-trehalase TreF	QEN71_30370	paras_006996	protein-codi	NZ_CP1252	chromosom	160887	162716	-	1830	0	53	8080	44	7269	
QEN71_RS30380	FUSC family protein	QEN71_30375	paras_006997	protein-codi	NZ_CP1252	chromosom	162826	163878	+	1053	0	9	3110	5	1445	
QEN71_RS30385	hypothetical protein	QEN71_30380	paras_006998	protein-codi	NZ_CP1252	chromosom	164003	164203	+	201	0	8	1586	6	1478	
QEN71_RS30390	rubredoxin	QEN71_30385	paras_006999	protein-codi	NZ_CP1252	chromosom	164764	165186	+	423	0	8	2469	4	982	
QEN71_RS30395	hypothetical protein	QEN71_30390	paras_007000	protein-codi	NZ_CP1252	chromosom	165201	165548	-	348	0	9	571	4	310	
QEN71_RS30400	hypothetical protein	QEN71_30395	paras_007001	protein-codi	NZ_CP1252	chromosom	165699	166121	+	423	0	11	1876	9	1772	
QEN71_RS30405	hypothetical protein	QEN71_30400	paras_007002	protein-codi	NZ_CP1252	chromosom	166134	166445	-	312	0	12	3027	10	1719	
QEN71_RS30410	LysR family transcriptional regulator	QEN71_30405	paras_007003	protein-codi	NZ_CP1252	chromosom	166566	167483	-	918	0	13	1786	11	1725	
QEN71_RS30415	LysR family transcriptional regulator	QEN71_30410	paras_007004	protein-codi	NZ_CP1252	chromosom	167537	168448	-	912	0	21	3204	15	1469	
QEN71_RS30420	acetamidase/formamidase family protein	QEN71_30415	paras_007005	protein-codi	NZ_CP1252	chromosom	168540	169892	-	1353	0	20	2097	9	916	
QEN71_RS30425	creatininase family protein	QEN71_30420	paras_007006	protein-codi	NZ_CP1252	chromosom	170261	171010	+	750	0	16	3664	8	1600	
QEN71_RS30430	nitronate monooxygenase	QEN71_30425	paras_007007	protein-codi	NZ_CP1252	chromosom	171171	172271	+	1101	0	9	717	6	400	
QEN71_RS30435	MOSC domain-containing protein	QEN71_30430	paras_007008	protein-codi	NZ_CP1252	chromosom	172294	172959	+	666	0	27	5182	25	4885	
QEN71_RS30440	4-oxalocrotonate tautomerase family protein	QEN71_30435	paras_007009	protein-codi	NZ_CP1252	chromosom	173026	173262	-	237	0	5	271	2	23	
QEN71_RS30445	SDR family NAD(P)-dependent oxidoreductase	QEN71_30440	paras_007010	protein-codi	NZ_CP1252	chromosom	173333	174043	-	711	0	9	976	8	662	
QEN71_RS30450	TIGR03571 family LLM class oxidoreductase	QEN71_30445	paras_007011	protein-codi	NZ_CP1252	chromosom	174289	175311	-	1023	0	16	1205	13	1138	
QEN71_RS30455	DUF485 domain-containing protein	QEN71_30450	paras_007012	protein-codi	NZ_CP1252	chromosom	175584	175898	+	311	0	10	2189	8	1880	
QEN71_RS30460	cation/acetate symporter ActP	QEN71_30455	paras_007013	protein-codi	NZ_CP1252	chromosom	175895	177565	+	1667	0	27	2622	18	1883	
QEN71_RS30465	cytochrome b/b6 domain-containing protein	QEN71_30460	paras_007014	protein-codi	NZ_CP1252	chromosom	177610	178203	+	594	0	6	462	4	365	
QEN71_RS30470	nitronate monooxygenase	QEN71_30465	paras_007015	protein-codi	NZ_CP1252	chromosom	178207	179196	+	990	0	14	1395	10	1172	
QEN71_RS30475	LysR family transcriptional regulator	QEN71_30470	paras_007016	protein-codi	NZ_CP1252	chromosom	179267	180214	-	948	0	9	1364	9	1364	
QEN71_RS30480	DUF2784 domain-containing protein	QEN71_30475	paras_007017	protein-codi	NZ_CP1252	chromosom	180387	180755	+	369	0	8	1006	6	810	
QEN71_RS30485	MFS transporter	QEN71_30480	paras_007018	protein-codi	NZ_CP1252	chromosom	180774	181940	-	1167	0	23	6665	18	5252	
QEN71_RS30490	LysR family transcriptional regulator	QEN71_30485	paras_007019	protein-codi	NZ_CP1252	chromosom	182058	182945	+	888	0	12	2951	6	788	
QEN71_RS30495	4-hydroxy-tetrahydrodipicolinate synthase	QEN71_30490	paras_007020	protein-codi	NZ_CP1252	chromosom	182971	183852	-	882	0	10	2914	6	1352	
QEN71_RS30500	hypothetical protein	QEN71_30495	paras_007021	protein-codi	NZ_CP1252	chromosom	183865	184191	-	327	0	2	942	2	942	
QEN71_RS30505	flagellar transcriptional regulator FlhD	QEN71_30500	paras_007022	protein-codi	NZ_CP1252	chromosom	184571	184891	+	321	0	5	1514	2	758	
QEN71_RS30510	flagellar transcriptional regulator FlhC	QEN71_30505	paras_007023	protein-codi	NZ_CP1252	chromosom	184905	185561	+	657	0	12	2706	12	2706	
QEN71_RS30515	flagellar biosynthesis anti-sigma factor FlgM	QEN71_30510	paras_007024	protein-codi	NZ_CP1252	chromosom	185770	186072	+	303	0	7	2239	4	1052	
QEN71_RS30520	porin	QEN71_30515	paras_007025	protein-codi	NZ_CP1252	chromosom	186117	187241	-	1125	0	48	7834	40	6637	
QEN71_RS30525	nitronate monooxygenase	QEN71_30520	paras_007026	protein-codi	NZ_CP1252	chromosom	187338	188288	-	951	0	11	1389	9	1248	
QEN71_RS30530	MFS transporter	QEN71_30525	paras_007027	protein-codi	NZ_CP1252	chromosom	188360	189697	-	1338	0	19	1488	15	926	
QEN71_RS30535	AMP-binding protein	QEN71_30530	paras_007028	protein-codi	NZ_CP1252	chromosom	189758	191428	-	1663	0	19	2308	19	2308	
QEN71_RS30540	enoyl-CoA hydratase/isomerase family protein	QEN71_30535	paras_007029	protein-codi	NZ_CP1252	chromosom	191421	192194	-	766	0	5	189	5	189	
QEN71_RS30545	CaiB/BaiF CoA-transferase family protein	QEN71_30540	paras_007030	protein-codi	NZ_CP1252	chromosom	192231	193430	-	1196	0	9	627	6	513	
QEN71_RS30550	enoyl-CoA hydratase/isomerase family protein	QEN71_30545	paras_007031	protein-codi	NZ_CP1252	chromosom	193427	194191	-	761	0	2	176	2	176	TRUE
QEN71_RS30555	hydroxymethylglutaryl-CoA lyase	QEN71_30550	paras_007032	protein-codi	NZ_CP1252	chromosom	194536	195486	+	947	0	12	1689	10	1374	
QEN71_RS30560	PaaI family thioesterase	QEN71_30555	paras_007033	protein-codi	NZ_CP1252	chromosom	195483	195869	+	383	0	12	1330	9	757	
QEN71_RS30565	MarR family transcriptional regulator	QEN71_30560	paras_007034	protein-codi	NZ_CP1252	chromosom	195974	196525	+	552	0	5	333	4	281	
QEN71_RS30570	DUF2945 domain-containing protein	QEN71_30565	paras_007035	protein-codi	NZ_CP1252	chromosom	196594	196812	+	219	0	2	498	2	498	
QEN71_RS30575	esterase	QEN71_30570	paras_007036	protein-codi	NZ_CP1252	chromosom	197112	197789	+	678	0	18	3795	18	3795	
QEN71_RS30580	ATP-binding protein	QEN71_30575	paras_007037	protein-codi	NZ_CP1252	chromosom	197844	199466	+	1623	0	32	4962	26	4665	
QEN71_RS30585	class I poly(R)-hydroxyalkanoic acid synthase	QEN71_30580	paras_007038	protein-codi	NZ_CP1252	chromosom	199479	201185	-	1707	0	32	3999	28	3355	
QEN71_RS30590	MaoC family dehydratase	QEN71_30585	paras_007039	protein-codi	NZ_CP1252	chromosom	201200	201670	-	471	0	7	701	6	698	
QEN71_RS30595	ligase-associated DNA damage response endonucl	QEN71_30590	paras_007040	protein-codi	NZ_CP1252	chromosom	201806	202474	-	668	0	7	1508	5	1257	
QEN71_RS30600	ligase-associated DNA damage response DEXH bo	QEN71_30595	paras_007041	protein-codi	NZ_CP1252	chromosom	202474	205140	-	2666	0	19	2344	17	2266	

QEN71_RS30605	GGDEF domain-containing protein	QEN71_30600	paras_007042	protein-codi	NZ_CP1252	chromosom	205346	206521	+	1176	0	28	6613	18	4809	
QEN71_RS30610	TIGR00366 family protein	QEN71_30605	paras_007043	protein-codi	NZ_CP1252	chromosom	206614	208008	-	1395	0	29	5685	19	3135	
QEN71_RS30615	methyl-accepting chemotaxis protein	QEN71_30610	paras_007044	protein-codi	NZ_CP1252	chromosom	208399	209967	-	1569	0	19	4027	16	3906	
QEN71_RS30620	GNAT family N-acetyltransferase	QEN71_30615	paras_007045	protein-codi	NZ_CP1252	chromosom	210186	210893	-	708	0	12	1684	12	1684	
QEN71_RS30625	TetR/AcrR family transcriptional regulator	QEN71_30620	paras_007046	protein-codi	NZ_CP1252	chromosom	211006	211584	-	579	0	10	1822	10	1822	
QEN71_RS30630	SDR family NAD(P)-dependent oxidoreductase	QEN71_30625	paras_007047	protein-codi	NZ_CP1252	chromosom	211702	212679	+	978	0	6	780	6	780	
QEN71_RS30635	winged helix-turn-helix domain-containing protein	QEN71_30630	paras_007048	protein-codi	NZ_CP1252	chromosom	212742	213071	-	330	0	7	762	5	738	
QEN71_RS30640	type 1 glutamine amidotransferase domain-containi	QEN71_30635	paras_007049	protein-codi	NZ_CP1252	chromosom	213288	213980	+	693	0	2	106	2	106	
QEN71_RS30645	enoyl-CoA hydratase/isomerase family protein	QEN71_30640	paras_007050	protein-codi	NZ_CP1252	chromosom	214012	214851	+	840	0	11	580	7	386	
QEN71_RS30650	trifunctional serine/threonine-protein kinase/ATP-bi	QEN71_30645	paras_007051	protein-codi	NZ_CP1252	chromosom	215030	220984	+	5955	0	65	4864	52	3975	
QEN71_RS30655	LysR family transcriptional regulator	QEN71_30650	paras_007052	protein-codi	NZ_CP1252	chromosom	221121	222062	+	942	0	8	539	7	516	
QEN71_RS30660	response regulator transcription factor	QEN71_30655	paras_007053	protein-codi	NZ_CP1252	chromosom	222390	223088	+	699	0	7	395	5	134	
QEN71_RS30665	hypothetical protein	QEN71_30660	paras_007054	protein-codi	NZ_CP1252	chromosom	223251	223505	+	255	0	2	74	0	0	
QEN71_RS30670	heme-binding protein	QEN71_30665	paras_007055	protein-codi	NZ_CP1252	chromosom	223590	224120	+	531	0	8	320	6	253	
QEN71_RS30675	AEC family transporter	QEN71_30670	paras_007056	protein-codi	NZ_CP1252	chromosom	224129	225070	-	942	0	11	870	5	520	
QEN71_RS30680	mandelate racemase/muconate lactonizing enzyme	QEN71_30675	paras_007057	protein-codi	NZ_CP1252	chromosom	225125	226231	-	1107	0	12	1138	9	913	
QEN71_RS30685	FadR/GntR family transcriptional regulator	QEN71_30680	paras_007058	protein-codi	NZ_CP1252	chromosom	226500	227231	+	732	0	7	832	3	510	
QEN71_RS30690	DsbA family protein	QEN71_30685	paras_007059	protein-codi	NZ_CP1252	chromosom	227391	228023	+	633	0	15	1640	5	372	
QEN71_RS30695	AEC family transporter	QEN71_30690	paras_007060	protein-codi	NZ_CP1252	chromosom	228030	228950	-	921	0	18	2698	13	2011	
QEN71_RS30700	aspartate aminotransferase family protein	QEN71_30695	paras_007061	protein-codi	NZ_CP1252	chromosom	229163	230464	-	1302	0	24	4201	23	4174	
QEN71_RS30705	LysR family transcriptional regulator	QEN71_30700	paras_007062	protein-codi	NZ_CP1252	chromosom	230569	231426	+	858	0	6	225	4	177	
QEN71_RS30710	AraC family transcriptional regulator	QEN71_30705	paras_007063	protein-codi	NZ_CP1252	chromosom	231471	232007	+	536	0	6	1420	2	723	
QEN71_RS30715	VOC family protein	QEN71_30710	paras_007064	protein-codi	NZ_CP1252	chromosom	232007	232414	+	407	0	12	2879	6	1599	
QEN71_RS30720	hybrid sensor histidine kinase/response regulator	QEN71_30715	paras_007065	protein-codi	NZ_CP1252	chromosom	232430	234604	-	2175	0	40	8784	23	4586	
QEN71_RS30725	MFS transporter	QEN71_30720	paras_007066	protein-codi	NZ_CP1252	chromosom	234827	236083	+	1257	0	11	2138	6	1634	
QEN71_RS30730	hypothetical protein	QEN71_30725	paras_007067	protein-codi	NZ_CP1252	chromosom	236152	236439	-	288	0	2	128	0	0	TRUE
QEN71_RS30735	TonB-dependent siderophore receptor	QEN71_30730	paras_007068	protein-codi	NZ_CP1252	chromosom	236756	238900	+	2145	0	71	11523	54	8162	
QEN71_RS30740	aldo/keto reductase	QEN71_30735	paras_007069	protein-codi	NZ_CP1252	chromosom	238965	239960	-	996	0	26	5924	17	3958	
QEN71_RS30745	hypothetical protein	QEN71_30740	paras_007070	protein-codi	NZ_CP1252	chromosom	240089	240334	+	246	0	3	304	3	304	
QEN71_RS30750	mechanosensitive ion channel	QEN71_30745	paras_007071	protein-codi	NZ_CP1252	chromosom	240347	241636	-	1290	0	23	5360	20	4056	
QEN71_RS30755	hypothetical protein	QEN71_30750	paras_007072	protein-codi	NZ_CP1252	chromosom	241832	242065	+	234	0	3	641	3	641	
QEN71_RS30760	sulfate permease	QEN71_30755	paras_007073	protein-codi	NZ_CP1252	chromosom	242096	243823	-	1728	0	32	4827	23	3808	
QEN71_RS30765	monovalent cation:proton antiporter-2 (CPA2) family	QEN71_30760	paras_007074	protein-codi	NZ_CP1252	chromosom	244154	245326	+	1173	0	9	909	8	760	
QEN71_RS30770	DsbA family oxidoreductase	QEN71_30765	paras_007075	protein-codi	NZ_CP1252	chromosom	245644	246345	+	702	0	18	1453	13	1202	
QEN71_RS30775	BON domain-containing protein	QEN71_30770	paras_007076	protein-codi	NZ_CP1252	chromosom	246453	246830	+	378	0	3	161	1	16	TRUE
QEN71_RS30780	porin	QEN71_30775	paras_007077	protein-codi	NZ_CP1252	chromosom	247069	248262	-	1194	0	36	3209	32	2655	
QEN71_RS30785	DUF4148 domain-containing protein	QEN71_30780	paras_007078	protein-codi	NZ_CP1252	chromosom	248686	248949	-	264	0	5	264	3	203	
QEN71_RS30790	potassium channel family protein	QEN71_30785	paras_007079	protein-codi	NZ_CP1252	chromosom	249256	249945	+	690	0	22	2512	17	1958	
QEN71_RS30795	porin	QEN71_30790	paras_007080	protein-codi	NZ_CP1252	chromosom	249977	251155	-	1179	0	25	2316	20	1824	
QEN71_RS30800	hypothetical protein	QEN71_30795	paras_007081	protein-codi	NZ_CP1252	chromosom	251389	251706	-	318	0	11	713	8	507	
QEN71_RS30805	hypothetical protein	QEN71_30800	paras_007082	protein-codi	NZ_CP1252	chromosom	252057	252863	+	807	0	21	1789	17	1446	
QEN71_RS30810	DUF2334 domain-containing protein	QEN71_30805	paras_007083	protein-codi	NZ_CP1252	chromosom	252933	254558	-	1626	0	35	2287	34	2283	
QEN71_RS30815	hypothetical protein	QEN71_30810	paras_007084	protein-codi	NZ_CP1252	chromosom	254601	254948	-	348	0	1	18	1	18	
QEN71_RS30820	UDP-N-acetylglucosamine 2-epimerase (non-hydrol	QEN71_30815	paras_007085	protein-codi	NZ_CP1252	chromosom	255012	256337	-	1326	0	19	1513	17	1313	
QEN71_RS30825	bacteriophage N4 adsorption protein A	QEN71_30820	paras_007086	protein-codi	NZ_CP1252	chromosom	256362	258047	-	1682	0	30	1920	24	1485	
QEN71_RS30830	glycosyl transferase family protein	QEN71_30825	paras_007087	protein-codi	NZ_CP1252	chromosom	258044	260002	-	1955	0	28	1476	28	1476	
QEN71_RS30835	glutamine--fructose-6-phosphate transaminase (iso	QEN71_30830	paras_007088	protein-codi	NZ_CP1252	chromosom	260158	261975	+	1818	0	18	912	13	704	
QEN71_RS30840	helix-turn-helix transcriptional regulator	QEN71_30835	paras_007089	protein-codi	NZ_CP1252	chromosom	262029	263096	-	1068	0	16	792	12	574	
QEN71_RS30845	hypothetical protein	QEN71_30840	paras_007090	protein-codi	NZ_CP1252	chromosom	263787	264761	-	975	0	26	2356	22	2224	
QEN71_RS30850	methyltransferase	QEN71_30845	paras_007091	protein-codi	NZ_CP1252	chromosom	264863	265924	-	1062	0	20	1778	17	1692	
QEN71_RS30855	multicopper oxidase family protein	QEN71_30850	paras_007092	protein-codi	NZ_CP1252	chromosom	266307	267866	+	1560	0	41	4987	29	4251	
QEN71_RS30860	multicopper oxidase family protein	QEN71_30855	paras_007093	protein-codi	NZ_CP1252	chromosom	267952	269529	+	1578	0	39	5810	32	4314	
QEN71_RS30865	Ig-like domain-containing protein	QEN71_30860	paras_007094	protein-codi	NZ_CP1252	chromosom	269549	277720	+	8168	0	154	23556	130	21165	
QEN71_RS30870	hypothetical protein	QEN71_30865	paras_007095	protein-codi	NZ_CP1252	chromosom	277717	278214	+	494	0	7	283	7	283	
QEN71_RS30875	outer membrane lipoprotein-sorting protein	QEN71_30870	paras_007096	protein-codi	NZ_CP1252	chromosom	278250	279065	+	816	0	15	1488	13	1248	
QEN71_RS30880	hypothetical protein	QEN71_30875	paras_007097	protein-codi	NZ_CP1252	chromosom	279092	279913	+	822	0	9	433	7	278	
QEN71_RS30885	c-type cytochrome	QEN71_30880	paras_007098	protein-codi	NZ_CP1252	chromosom	279916	281544	+	1629	0	37	3538	30	3120	
QEN71_RS30890	lytic transglycosylase domain-containing protein	QEN71_30885	paras_007099	protein-codi	NZ_CP1252	chromosom	281572	282213	-	642	0	7	555	6	543	
QEN71_RS30895	response regulator transcription factor	QEN71_30890	paras_007100	protein-codi	NZ_CP1252	chromosom	282772	283548	+	777	0	10	1052	10	1052	
QEN71_RS30900	histidine kinase	QEN71_30895	paras_007101	protein-codi	NZ_CP1252	chromosom	283559	284686	-	1128	0	8	430	5	94	
QEN71_RS30905	SCO family protein	partial;pseudo;QEN71_30900		pseudogene	NZ_CP1252	chromosom	285349	285882	+	534	0	9	236	8	194	
QEN71_RS30910	response regulator transcription factor	QEN71_30905	paras_007103	protein-codi	NZ_CP1252	chromosom	285905	286597	+	693	0	12	864	9	440	

QEN71_RS30915	type II secretion system major pseudopilin GspG	QEN71_30910	paras_007104	protein-codi	NZ_CP1252	chromosom	286743	287192	+	450	0	13	1284	13	1284	
QEN71_RS30920	type II secretion system F family protein	QEN71_30915	paras_007105	protein-codi	NZ_CP1252	chromosom	287247	288449	+	1203	0	20	1150	11	553	
QEN71_RS30925	GspE/PulE family protein	QEN71_30920	paras_007106	protein-codi	NZ_CP1252	chromosom	288480	290201	+	1702	0	11	433	11	433	
QEN71_RS30930	hypothetical protein	QEN71_30925	paras_007107	protein-codi	NZ_CP1252	chromosom	290182	291072	+	867	0	8	1210	7	1207	
QEN71_RS30935	hypothetical protein	QEN71_30930	paras_007108	protein-codi	NZ_CP1252	chromosom	291069	291599	+	523	0	4	535	2	163	
QEN71_RS30940	hypothetical protein	QEN71_30935	paras_007109	protein-codi	NZ_CP1252	chromosom	291596	292165	+	562	0	6	667	5	648	
QEN71_RS30945	hypothetical protein	QEN71_30940	paras_007110	protein-codi	NZ_CP1252	chromosom	292162	292758	+	593	0	7	454	5	317	
QEN71_RS30950	secretin N-terminal domain-containing protein	QEN71_30945	paras_007111	protein-codi	NZ_CP1252	chromosom	292759	295050	+	2292	0	23	1413	19	1350	
QEN71_RS30955	type II secretion system protein	QEN71_30950	paras_007112	protein-codi	NZ_CP1252	chromosom	295054	295623	+	570	0	8	948	8	948	
QEN71_RS30960	type II secretion system protein	QEN71_30955	paras_007113	protein-codi	NZ_CP1252	chromosom	295632	296024	+	389	0	6	438	6	438	
QEN71_RS30965	type II secretion system protein	QEN71_30960	paras_007114	protein-codi	NZ_CP1252	chromosom	296021	296641	+	617	0	11	616	10	505	
QEN71_RS30970	sigma-54 dependent transcriptional regulator	QEN71_30965	paras_007115	protein-codi	NZ_CP1252	chromosom	296931	298316	+	1386	0	25	2565	19	2409	
QEN71_RS30975	divalent metal cation transporter	QEN71_30970	paras_007116	protein-codi	NZ_CP1252	chromosom	298336	299643	-	1308	0	16	1539	14	1389	
QEN71_RS30980	hypothetical protein	QEN71_30975	paras_007117	protein-codi	NZ_CP1252	chromosom	299724	299912	-	189	0	4	823	4	823	
QEN71_RS30985	hypothetical protein	QEN71_30980	paras_007118	protein-codi	NZ_CP1252	chromosom	300124	300417	-	294	0	10	604	5	308	
QEN71_RS30990	hypothetical protein	QEN71_30985	paras_007119	protein-codi	NZ_CP1252	chromosom	300753	301388	-	636	0	5	222	3	54	
QEN71_RS30995	hypothetical protein	QEN71_30990	paras_007120	protein-codi	NZ_CP1252	chromosom	301610	303166	-	1557	0	50	7840	40	5681	
QEN71_RS31000	hypothetical protein	QEN71_30995	paras_007121	protein-codi	NZ_CP1252	chromosom	303940	304464	+	525	0	7	596	7	596	
QEN71_RS31005	hypothetical protein	QEN71_31000	paras_007122	protein-codi	NZ_CP1252	chromosom	304884	305573	+	668	0	25	2352	22	1961	
QEN71_RS31010	DUF3293 domain-containing protein	QEN71_31005	paras_007123	protein-codi	NZ_CP1252	chromosom	305552	305980	-	407	0	4	525	3	507	
QEN71_RS31015	ureidoglycolate lyase	QEN71_31010	paras_007124	protein-codi	NZ_CP1252	chromosom	306128	306973	+	846	0	13	596	7	346	
QEN71_RS31020	SDR family NAD(P)-dependent oxidoreductase	QEN71_31015	paras_007125	protein-codi	NZ_CP1252	chromosom	306988	307740	+	753	0	8	1001	6	754	
QEN71_RS31025	LysR family transcriptional regulator	QEN71_31020	paras_007126	protein-codi	NZ_CP1252	chromosom	307760	308710	-	951	0	9	1810	5	1533	
QEN71_RS31030	IlvD/Edd family dehydratase	QEN71_31025	paras_007127	protein-codi	NZ_CP1252	chromosom	308899	310632	+	1734	99.0	21	3379	13	1829	
QEN71_RS31035	aldehyde dehydrogenase (NADP(+))	QEN71_31030	paras_007128	protein-codi	NZ_CP1252	chromosom	310664	312244	+	1581	0	16	1160	11	904	
QEN71_RS31040	MFS transporter	QEN71_31035	paras_007129	protein-codi	NZ_CP1252	chromosom	312314	313663	+	1350	0	25	2140	20	1665	
QEN71_RS31045	dihydrodipicolinate synthase family protein	QEN71_31040	paras_007130	protein-codi	NZ_CP1252	chromosom	313706	314635	+	930	0	7	546	7	546	
QEN71_RS31050	MFS transporter	QEN71_31045	paras_007131	protein-codi	NZ_CP1252	chromosom	314747	316045	+	1299	0	25	2384	18	1329	
QEN71_RS31055	2-methylaconitate cis-trans isomerase PrpF	QEN71_31050	paras_007132	protein-codi	NZ_CP1252	chromosom	316657	317841	-	1185	0	23	1971	15	1061	
QEN71_RS31060	Fe/S-dependent 2-methylisocitrate dehydratase Acr	QEN71_31055	paras_007133	protein-codi	NZ_CP1252	chromosom	317916	320513	-	2598	0	26	2114	18	1494	
QEN71_RS31065	2-methylcitrate synthase	QEN71_31060	paras_007134	protein-codi	NZ_CP1252	chromosom	320746	321915	-	1170	352.0	15	4400	14	4391	
QEN71_RS31070	methylisocitrate lyase	QEN71_31065	paras_007135	protein-codi	NZ_CP1252	chromosom	321943	322836	-	894	0	19	3005	10	2047	
QEN71_RS31075	propionate catabolism operon regulatory protein Prp	QEN71_31070	paras_007136	protein-codi	NZ_CP1252	chromosom	323034	324983	+	1950	0	20	2463	18	2445	
QEN71_RS31080	DUF4337 domain-containing protein	QEN71_31075	paras_007137	protein-codi	NZ_CP1252	chromosom	324989	325567	-	579	0	10	720	8	596	
QEN71_RS31085	helix-turn-helix domain-containing protein	QEN71_31080	paras_007138	protein-codi	NZ_CP1252	chromosom	325696	326685	-	990	0	9	577	8	361	
QEN71_RS31090	hypothetical protein	QEN71_31085	paras_007139	protein-codi	NZ_CP1252	chromosom	326957	327184	-	228	0	2	250	2	250	
QEN71_RS31095	glycosyltransferase family 4 protein	QEN71_31090	paras_007140	protein-codi	NZ_CP1252	chromosom	327218	328345	-	1128	0	20	1177	18	1039	
QEN71_RS31100	RICIN domain-containing protein	QEN71_31095	paras_007141	protein-codi	NZ_CP1252	chromosom	328609	330873	-	2166	0	27	1215	21	814	
QEN71_RS31105	hypothetical protein			protein-codi	NZ_CP1252	chromosom	330775	331113	+	240	0	3	138	2	113	
QEN71_RS31110	glycosyltransferase family 4 protein	QEN71_31105	paras_007142	protein-codi	NZ_CP1252	chromosom	331452	332636	+	1185	0	11	980	10	971	
QEN71_RS31115	hypothetical protein	QEN71_31110	paras_007143	protein-codi	NZ_CP1252	chromosom	332685	332870	+	170	0	0	0	0	0	
QEN71_RS31120	LysR family transcriptional regulator	QEN71_31115	paras_007144	protein-codi	NZ_CP1252	chromosom	332855	333778	-	908	0	5	920	5	920	
QEN71_RS31125	EthD family reductase	QEN71_31120	paras_007145	protein-codi	NZ_CP1252	chromosom	333810	334490	-	681	0	7	683	7	683	
QEN71_RS31130	4-hydroxyphenylacetate 3-monooxygenase, reducta	QEN71_31125	paras_007146	protein-codi	NZ_CP1252	chromosom	334493	335017	-	525	0	3	167	3	167	
QEN71_RS31135	acyl-CoA dehydrogenase family protein	QEN71_31130	paras_007147	protein-codi	NZ_CP1252	chromosom	335050	336261	-	1212	0	13	581	13	581	
QEN71_RS31140	MFS transporter	QEN71_31135	paras_007148	protein-codi	NZ_CP1252	chromosom	336292	337608	-	1317	0	13	884	8	285	
QEN71_RS31145	porin	QEN71_31140	paras_007149	protein-codi	NZ_CP1252	chromosom	338000	339217	+	1218	0	25	1435	23	1423	
QEN71_RS31150	porin	QEN71_31145	paras_007150	protein-codi	NZ_CP1252	chromosom	339279	340418	-	1140	0	27	1872	18	1244	
QEN71_RS31155	ABC transporter ATP-binding protein	QEN71_31150	paras_007151	protein-codi	NZ_CP1252	chromosom	340552	341367	-	812	0	4	68	3	50	
QEN71_RS31160	iron ABC transporter permease	QEN71_31155	paras_007152	protein-codi	NZ_CP1252	chromosom	341364	342446	-	1079	0	10	971	8	459	
QEN71_RS31165	ABC transporter substrate-binding protein	QEN71_31160	paras_007153	protein-codi	NZ_CP1252	chromosom	342462	343700	-	1239	0	31	2994	25	2582	
QEN71_RS31170	response regulator transcription factor	QEN71_31165	paras_007154	protein-codi	NZ_CP1252	chromosom	343841	344554	+	713	0	13	4377	8	1973	
QEN71_RS31175	ATP-binding protein	QEN71_31170	paras_007155	protein-codi	NZ_CP1252	chromosom	344554	345969	+	1415	0	14	2522	12	2371	
QEN71_RS31180	chloride channel protein	QEN71_31175	paras_007156	protein-codi	NZ_CP1252	chromosom	346115	347464	+	1350	0	14	4775	7	1832	
QEN71_RS31185	methyl-accepting chemotaxis protein	QEN71_31180	paras_007157	protein-codi	NZ_CP1252	chromosom	347468	349192	-	1725	0	19	6383	10	2886	
QEN71_RS31190	LysR family transcriptional regulator	QEN71_31185	paras_007158	protein-codi	NZ_CP1252	chromosom	349349	350281	-	933	0	18	3210	16	2794	
QEN71_RS31195	MFS transporter	QEN71_31190	paras_007159	protein-codi	NZ_CP1252	chromosom	350426	351754	+	1329	0	36	11002	27	8937	
QEN71_RS31200	M81 family metallopeptidase	QEN71_31195	paras_007160	protein-codi	NZ_CP1252	chromosom	351775	353307	+	1533	0	25	4675	23	4274	
QEN71_RS31205	NUDIX hydrolase	QEN71_31200	paras_007161	protein-codi	NZ_CP1252	chromosom	353358	353873	+	516	0	6	720	6	720	
QEN71_RS31210	hypothetical protein	QEN71_31205	paras_007162	protein-codi	NZ_CP1252	chromosom	353944	354780	-	837	0	15	1795	12	1445	
QEN71_RS31215	flagellar transcriptional regulator FlhD	QEN71_31210	paras_007163	protein-codi	NZ_CP1252	chromosom	355215	355535	+	317	0	6	671	5	350	
QEN71_RS31220	flagellar transcriptional regulator FlhC	QEN71_31215	paras_007164	protein-codi	NZ_CP1252	chromosom	355532	356128	+	593	0	14	1863	13	1854	

QEN71_RS31225	NUDIX domain-containing protein	QEN71_31220	paras_007165	protein-codi	NZ_CP1252	chromosom	356135	356503	-	369	0	9	845	4	424	
QEN71_RS31230	CHAD domain-containing protein	QEN71_31225	paras_007166	protein-codi	NZ_CP1252	chromosom	356741	357652	+	912	0	13	1316	13	1316	
QEN71_RS31235	hypothetical protein	QEN71_31230	paras_007167	protein-codi	NZ_CP1252	chromosom	357663	357980	-	304	0	2	50	2	50	
QEN71_RS31240	acyclic terpene utilization AtuA family protein	QEN71_31235	paras_007168	protein-codi	NZ_CP1252	chromosom	357967	359325	-	1345	0	30	2631	25	2362	
QEN71_RS31245	citrate:proton symporter	QEN71_31240	paras_007169	protein-codi	NZ_CP1252	chromosom	359339	360643	-	1305	0	10	651	6	343	
QEN71_RS31250	LysR family transcriptional regulator	QEN71_31245	paras_007170	protein-codi	NZ_CP1252	chromosom	360744	361637	+	894	0	10	2033	8	1801	
QEN71_RS31255	carbonic anhydrase	QEN71_31250	paras_007171	protein-codi	NZ_CP1252	chromosom	361695	362147	-	453	0	6	932	3	659	
QEN71_RS31260	SDR family NAD(P)-dependent oxidoreductase	QEN71_31255	paras_007172	protein-codi	NZ_CP1252	chromosom	362267	363013	-	747	0	7	472	6	297	
QEN71_RS31265	NmrA family NAD(P)-binding protein	QEN71_31260	paras_007173	protein-codi	NZ_CP1252	chromosom	363089	363967	-	879	0	6	242	5	168	
QEN71_RS31270	NmrA family NAD(P)-binding protein	QEN71_31265	paras_007174	protein-codi	NZ_CP1252	chromosom	364023	364916	-	894	0	12	1320	11	1177	
QEN71_RS31275	LysR family transcriptional regulator	QEN71_31270	paras_007175	protein-codi	NZ_CP1252	chromosom	365172	366080	+	909	0	10	1244	9	1228	
QEN71_RS31280	DUF4148 domain-containing protein	QEN71_31275	paras_007176	protein-codi	NZ_CP1252	chromosom	366182	366604	-	423	0	6	421	6	421	
QEN71_RS31285	porin	QEN71_31280	paras_007177	protein-codi	NZ_CP1252	chromosom	366817	367962	+	1146	0	31	3484	26	2970	
QEN71_RS31290	ABC transporter substrate-binding protein	QEN71_31285	paras_007178	protein-codi	NZ_CP1252	chromosom	368086	369102	-	1017	0	15	1095	14	1051	
QEN71_RS31295	LysR substrate-binding domain-containing protein	QEN71_31290	paras_007179	protein-codi	NZ_CP1252	chromosom	369273	370223	+	951	0	15	1823	13	1712	
QEN71_RS31300	tripartite tricarboxylate transporter substrate-binding	QEN71_31295	paras_007180	protein-codi	NZ_CP1252	chromosom	370576	371562	+	987	0	17	2091	16	2059	
QEN71_RS31305	amidohydrolase family protein	QEN71_31300	paras_007181	protein-codi	NZ_CP1252	chromosom	371639	372493	+	855	0	12	792	11	785	
QEN71_RS31310	tripartite tricarboxylate transporter permease	QEN71_31305	paras_007182	protein-codi	NZ_CP1252	chromosom	372542	374548	+	2007	0	26	3112	23	2865	
QEN71_RS31315	carboxymuconolactone decarboxylase	QEN71_31310	paras_007183	protein-codi	NZ_CP1252	chromosom	374594	375124	+	531	0	4	298	4	298	
QEN71_RS31320	LysR substrate-binding domain-containing protein	QEN71_31315	paras_007184	protein-codi	NZ_CP1252	chromosom	375165	376172	+	1008	0	12	1684	10	982	
QEN71_RS31325	hypothetical protein	QEN71_31320	paras_007185	protein-codi	NZ_CP1252	chromosom	376322	376621	+	300	0	5	371	5	371	
QEN71_RS31330	ABC transporter substrate-binding protein	QEN71_31325	paras_007186	protein-codi	NZ_CP1252	chromosom	377103	378074	+	972	0	15	1480	14	1467	
QEN71_RS31335	amidohydrolase family protein	QEN71_31330	paras_007187	protein-codi	NZ_CP1252	chromosom	378118	379143	+	1026	0	11	393	9	333	
QEN71_RS31340	amidohydrolase family protein	QEN71_31335	paras_007188	protein-codi	NZ_CP1252	chromosom	379167	380015	+	849	0	10	1486	8	1086	
QEN71_RS31345	aldolase/citrate lyase family protein	QEN71_31340	paras_007189	protein-codi	NZ_CP1252	chromosom	380087	380944	+	857	0	18	3575	14	2534	
QEN71_RS31350	acetolactate synthase large subunit	QEN71_31345	paras_007190	protein-codi	NZ_CP1252	chromosom	380944	382494	+	1550	0	14	2261	13	2167	
QEN71_RS31355	phosphate ABC transporter substrate-binding protei	QEN71_31350	paras_007191	protein-codi	NZ_CP1252	chromosom	382533	383426	+	894	135.0	12	490	8	192	
QEN71_RS31360	MFS transporter	QEN71_31355	paras_007192	protein-codi	NZ_CP1252	chromosom	383475	384776	+	1302	0	23	1073	23	1073	
QEN71_RS31365	MFS transporter	QEN71_31360	paras_007193	protein-codi	NZ_CP1252	chromosom	384927	386192	+	1266	0	13	739	10	550	
QEN71_RS31370	aldehyde dehydrogenase (NADP(+))	QEN71_31365	paras_007194	protein-codi	NZ_CP1252	chromosom	386574	388175	+	1602	0	12	1369	8	976	
QEN71_RS31375	DUF4148 domain-containing protein	QEN71_31370	paras_007195	protein-codi	NZ_CP1252	chromosom	388311	388595	+	285	0	1	17	1	17	
QEN71_RS31380	branched-chain amino acid ABC transporter substra	QEN71_31375	paras_007196	protein-codi	NZ_CP1252	chromosom	389029	390201	+	1173	0	16	1809	14	1665	
QEN71_RS31385	MFS transporter	QEN71_31380	paras_007197	protein-codi	NZ_CP1252	chromosom	390318	391682	+	1365	0	24	3120	16	2069	
QEN71_RS31390	FadR/GntR family transcriptional regulator	QEN71_31385	paras_007198	protein-codi	NZ_CP1252	chromosom	391737	392486	-	750	0	8	976	6	858	
QEN71_RS31395	SDR family oxidoreductase	QEN71_31390	paras_007199	protein-codi	NZ_CP1252	chromosom	392680	393432	-	753	0	15	2203	5	774	
QEN71_RS31400	SDR family oxidoreductase	QEN71_31395	paras_007200	protein-codi	NZ_CP1252	chromosom	393466	394206	-	741	0	3	227	2	155	
QEN71_RS31405	FAD-dependent oxidoreductase	QEN71_31400	paras_007201	protein-codi	NZ_CP1252	chromosom	394239	395435	-	1197	0	22	1761	18	1677	
QEN71_RS31410	NAD(P)-dependent oxidoreductase	QEN71_31405	paras_007202	protein-codi	NZ_CP1252	chromosom	395473	396306	-	834	0	16	1242	16	1242	
QEN71_RS31415	hydantoinase B/oxoprolinase family protein	QEN71_31410	paras_007203	protein-codi	NZ_CP1252	chromosom	396316	397998	-	1683	0	23	1715	15	961	
QEN71_RS31420	hydantoinase/oxoprolinase family protein	QEN71_31415	paras_007204	protein-codi	NZ_CP1252	chromosom	398012	400111	-	2100	0	26	2163	23	2010	
QEN71_RS31425	electron transfer flavoprotein subunit beta/FixA fami	QEN71_31420	paras_007205	protein-codi	NZ_CP1252	chromosom	400461	401210	+	750	147.0	4	86	2	52	
QEN71_RS31430	electron transfer flavoprotein subunit alpha/FixB fan	QEN71_31425	paras_007206	protein-codi	NZ_CP1252	chromosom	401225	402157	+	933	99.0	13	865	11	650	
QEN71_RS31435	class II aldolase/adducin family protein	QEN71_31430	paras_007207	protein-codi	NZ_CP1252	chromosom	402237	403019	+	783	0	17	936	16	769	
QEN71_RS31440	hypothetical protein	QEN71_31435	paras_007208	protein-codi	NZ_CP1252	chromosom	403047	403604	+	558	0	6	523	4	508	
QEN71_RS31445	hypothetical protein	QEN71_31440		protein-codi	NZ_CP1252	chromosom	403683	403835	-	153	0	4	421	3	356	
QEN71_RS31450	amino acid permease	QEN71_31445	paras_007209	protein-codi	NZ_CP1252	chromosom	403839	405170	+	1332	0	38	7222	29	5187	
QEN71_RS31455	hypothetical protein	QEN71_31450	paras_007210	protein-codi	NZ_CP1252	chromosom	405281	405583	+	303	0	1	11	1	11	
QEN71_RS31460	response regulator	QEN71_31455	paras_007211	protein-codi	NZ_CP1252	chromosom	405833	409834	+	3995	0	46	8303	38	6938	
QEN71_RS31465	diguanylate cyclase	QEN71_31460	paras_007212	protein-codi	NZ_CP1252	chromosom	409828	410781	+	947	0	11	1741	9	1430	
QEN71_RS31470	LysR family transcriptional regulator	QEN71_31465	paras_007213	protein-codi	NZ_CP1252	chromosom	410796	411677	-	882	0	13	1880	8	1471	
QEN71_RS31475	ABC transporter ATP-binding protein	QEN71_31470	paras_007214	protein-codi	NZ_CP1252	chromosom	411826	412848	+	1019	0	11	2445	9	1479	
QEN71_RS31480	ATP-binding cassette domain-containing protein	QEN71_31475	paras_007215	protein-codi	NZ_CP1252	chromosom	412845	413924	+	1076	0	10	1448	8	1291	
QEN71_RS31485	ABC transporter substrate-binding protein	QEN71_31480	paras_007216	protein-codi	NZ_CP1252	chromosom	413968	415467	+	1500	0	27	3461	23	3104	
QEN71_RS31490	ABC transporter permease	QEN71_31485	paras_007217	protein-codi	NZ_CP1252	chromosom	415473	416417	+	944	0	12	2650	7	646	
QEN71_RS31495	ABC transporter permease	QEN71_31490	paras_007218	protein-codi	NZ_CP1252	chromosom	416417	417301	+	884	0	12	880	8	574	
QEN71_RS31500	gamma-glutamyltransferase family protein	QEN71_31495	paras_007219	protein-codi	NZ_CP1252	chromosom	417353	418990	+	1638	0	31	5267	24	4096	
QEN71_RS31505	response regulator	QEN71_31500	paras_007220	protein-codi	NZ_CP1252	chromosom	419013	419933	-	921	0	11	921	7	722	
QEN71_RS31510	VTT domain-containing protein	QEN71_31505	paras_007221	protein-codi	NZ_CP1252	chromosom	420789	421805	+	1017	0	19	2527	13	1573	
QEN71_RS31515	sterol desaturase family protein	QEN71_31510	paras_007222	protein-codi	NZ_CP1252	chromosom	422065	422982	+	918	0	23	3971	22	3954	
QEN71_RS31520	aldo/keto reductase	QEN71_31515	paras_007223	protein-codi	NZ_CP1252	chromosom	423248	424276	+	1029	0	32	5488	26	5115	
QEN71_RS31525	response regulator	QEN71_31520	paras_007224	protein-codi	NZ_CP1252	chromosom	424455	424856	+	402	0	9	854	6	449	
QEN71_RS31530	hypothetical protein	QEN71_31525	paras_007225	protein-codi	NZ_CP1252	chromosom	424886	425065	-	178	0	6	938	4	620	

QEN71_RS31535	porin	QEN71_31530	paras_007226	protein-codi	NZ_CP1252	chromosom	425064	426224	+	1159	0	34	2868	27	2400	
QEN71_RS31540	RNA polymerase sigma-70 factor	QEN71_31535	paras_007227	protein-codi	NZ_CP1252	chromosom	426592	427473	+	882	0	5	532	4	373	
QEN71_RS31545	carboxymuconolactone decarboxylase family protei	QEN71_31540	paras_007228	protein-codi	NZ_CP1252	chromosom	427642	428121	+	480	0	7	1039	3	124	
QEN71_RS31550	cupin domain-containing protein	QEN71_31545	paras_007229	protein-codi	NZ_CP1252	chromosom	428195	428602	+	408	0	14	1575	11	1507	
QEN71_RS31555	LysR family transcriptional regulator	QEN71_31550	paras_007230	protein-codi	NZ_CP1252	chromosom	428710	429711	+	1002	0	17	2798	12	2160	
QEN71_RS31560	alpha/beta fold hydrolase	QEN71_31555	paras_007231	protein-codi	NZ_CP1252	chromosom	429719	430816	-	1098	0	24	3996	22	3602	
QEN71_RS31565	pyrrolo-quinoline quinone	QEN71_31560	paras_007232	protein-codi	NZ_CP1252	chromosom	431000	432694	+	1695	0	46	6714	35	4549	
QEN71_RS31570	FAD-dependent oxidoreductase	QEN71_31565	paras_007233	protein-codi	NZ_CP1252	chromosom	432705	434447	-	1743	0	28	2904	22	2444	
QEN71_RS31575	hypothetical protein	QEN71_31570	paras_007234	protein-codi	NZ_CP1252	chromosom	434850	435260	+	411	0	8	850	5	690	
QEN71_RS31580	Ohr family peroxiredoxin	QEN71_31575	paras_007235	protein-codi	NZ_CP1252	chromosom	435448	435897	+	450	0	6	280	2	52	
QEN71_RS31585	hypothetical protein	QEN71_31580	paras_007236	protein-codi	NZ_CP1252	chromosom	435978	436706	+	729	0	7	771	5	221	
QEN71_RS31590	IclR family transcriptional regulator	QEN71_31585	paras_007237	protein-codi	NZ_CP1252	chromosom	436716	437561	-	846	0	5	567	5	567	
QEN71_RS31595	ABC transporter ATP-binding protein	QEN71_31590	paras_007238	protein-codi	NZ_CP1252	chromosom	437863	438954	+	1084	0	11	1903	9	1431	
QEN71_RS31600	ABC transporter permease	QEN71_31595	paras_007239	protein-codi	NZ_CP1252	chromosom	438947	439852	+	898	0	17	3019	13	1746	
QEN71_RS31605	ABC transporter permease	QEN71_31600	paras_007240	protein-codi	NZ_CP1252	chromosom	439877	440671	+	795	0	5	1874	4	1648	
QEN71_RS31610	(2Fe-2S)-binding protein	QEN71_31605	paras_007241	protein-codi	NZ_CP1252	chromosom	440695	440997	+	295	0	1	110	1	110	
QEN71_RS31615	NAD(P)/FAD-dependent oxidoreductase	QEN71_31610	paras_007242	protein-codi	NZ_CP1252	chromosom	440990	442408	+	1403	0	26	5139	24	5039	
QEN71_RS31620	FAD-binding oxidoreductase	QEN71_31615	paras_007243	protein-codi	NZ_CP1252	chromosom	442401	443528	+	1120	0	15	1803	12	1591	
QEN71_RS31625	extracellular solute-binding protein	QEN71_31620	paras_007244	protein-codi	NZ_CP1252	chromosom	443549	444595	+	1047	0	19	2881	15	1510	
QEN71_RS31630	peroxiredoxin	QEN71_31625	paras_007245	protein-codi	NZ_CP1252	chromosom	444879	445532	+	654	0	27	4867	15	2484	
QEN71_RS31635	MBL fold metallo-hydrolase	QEN71_31630	paras_007246	protein-codi	NZ_CP1252	chromosom	445642	446712	+	1071	0	39	11614	25	7110	
QEN71_RS31640	PLP-dependent aminotransferase family protein	QEN71_31635	paras_007247	protein-codi	NZ_CP1252	chromosom	446731	448173	-	1443	0	32	6326	22	4313	
QEN71_RS31645	amino acid permease	QEN71_31640	paras_007248	protein-codi	NZ_CP1252	chromosom	448421	449905	+	1484	0	22	3843	20	3801	
QEN71_RS31650	FAD-dependent oxidoreductase	QEN71_31645	paras_007249	protein-codi	NZ_CP1252	chromosom	449905	451188	+	1283	0	21	3499	18	2977	
QEN71_RS31655	branched-chain amino acid ABC transporter substra	QEN71_31650	paras_007250	protein-codi	NZ_CP1252	chromosom	451280	452428	+	1149	0	25	5266	18	3623	
QEN71_RS31660	type VI secretion system tube protein Hcp	QEN71_31655	paras_007251	protein-codi	NZ_CP1252	chromosom	452525	452992	-	468	0	18	1058	10	682	
QEN71_RS31665	DUF2778 domain-containing protein	QEN71_31660	paras_007252	protein-codi	NZ_CP1252	chromosom	453251	453730	-	480	0	19	2965	15	2561	
QEN71_RS31670	GNAT family N-acetyltransferase	QEN71_31665	paras_007253	protein-codi	NZ_CP1252	chromosom	453874	454338	-	465	0	13	1847	12	1807	
QEN71_RS31675	type IVB secretion system protein IcmH/DotU	QEN71_31670	paras_007254	protein-codi	NZ_CP1252	chromosom	454479	455261	-	779	0	21	5698	15	4425	
QEN71_RS31680	type VI secretion system baseplate subunit TssK	QEN71_31675	paras_007255	protein-codi	NZ_CP1252	chromosom	455258	456601	-	1340	0	38	12067	25	7798	
QEN71_RS31685	type VI secretion system lipoprotein TssJ	QEN71_31680	paras_007256	protein-codi	NZ_CP1252	chromosom	456725	457351	-	627	0	36	14520	29	13249	
QEN71_RS31690	tetratricopeptide repeat protein	QEN71_31685	paras_007257	protein-codi	NZ_CP1252	chromosom	457701	458381	+	681	0	17	5511	12	4599	
QEN71_RS31695	type VI secretion system contractile sheath small su	QEN71_31690	paras_007258	protein-codi	NZ_CP1252	chromosom	458422	458955	+	534	0	12	8301	6	3918	
QEN71_RS31700	type VI secretion system contractile sheath large su	QEN71_31695	paras_007259	protein-codi	NZ_CP1252	chromosom	458980	460467	+	1488	0	46	14521	39	12946	
QEN71_RS31705	type VI secretion system tube protein Hcp	QEN71_31700	paras_007260	protein-codi	NZ_CP1252	chromosom	460542	461045	+	504	0	19	3771	12	2479	
QEN71_RS31710	type VI secretion system baseplate subunit TssE	QEN71_31705	paras_007261	protein-codi	NZ_CP1252	chromosom	461121	461600	+	480	0	9	1368	7	1294	
QEN71_RS31715	type VI secretion system baseplate subunit TssF	QEN71_31710	paras_007262	protein-codi	NZ_CP1252	chromosom	461656	463494	+	1802	0	49	11783	40	8544	
QEN71_RS31720	type VI secretion system baseplate subunit TssG	QEN71_31715	paras_007263	protein-codi	NZ_CP1252	chromosom	463458	464543	+	1049	0	24	3926	23	3758	
QEN71_RS31725	type VI secretion system ATPase TssH	QEN71_31720	paras_007264	protein-codi	NZ_CP1252	chromosom	464586	467240	+	2655	0	46	8355	36	6346	
QEN71_RS31730	type VI secretion system protein TssA	QEN71_31725	paras_007265	protein-codi	NZ_CP1252	chromosom	467254	468384	+	1131	0	33	4932	26	3731	
QEN71_RS31735	OmpA family protein	QEN71_31730	paras_007266	protein-codi	NZ_CP1252	chromosom	468403	469317	-	915	0	24	4832	22	4492	
QEN71_RS31740	type VI secretion system-associated protein TagF	QEN71_31735	paras_007267	protein-codi	NZ_CP1252	chromosom	469328	470317	-	986	0	14	3522	7	1629	
QEN71_RS31745	type VI secretion system membrane subunit TssM	QEN71_31740	paras_007268	protein-codi	NZ_CP1252	chromosom	470314	474171	-	3854	0	86	28274	72	25063	
QEN71_RS31750	M15 family metallopeptidase	QEN71_31745	paras_007269	protein-codi	NZ_CP1252	chromosom	474337	475182	-	846	0	42	11747	32	9217	
QEN71_RS31755	hypothetical protein	QEN71_31750	paras_007270	protein-codi	NZ_CP1252	chromosom	475310	476239	+	930	0	7	1375	6	1343	
QEN71_RS31760	hypothetical protein	QEN71_31755	paras_007271	protein-codi	NZ_CP1252	chromosom	476293	476559	-	267	0	18	12150	14	11113	
QEN71_RS31765	phospholipase C, phosphocholine-specific	QEN71_31760	paras_007272	protein-codi	NZ_CP1252	chromosom	476697	478763	-	2067	0	64	19130	54	17818	
QEN71_RS31770	hypothetical protein	QEN71_31765	paras_007273	protein-codi	NZ_CP1252	chromosom	479207	479446	+	240	0	5	788	5	788	
QEN71_RS31775	bacteriohopanetetrol glucosamine biosynthesis glyc	QEN71_31770	paras_007274	protein-codi	NZ_CP1252	chromosom	479500	480678	+	1179	0	25	3250	21	2824	
QEN71_RS31780	lysylphosphatidylglycerol synthase domain-containi	QEN71_31775	paras_007275	protein-codi	NZ_CP1252	chromosom	480697	481665	+	969	0	21	3687	15	1444	
QEN71_RS31785	HAD family hydrolase	QEN71_31780	paras_007276	protein-codi	NZ_CP1252	chromosom	482006	482695	+	690	0	20	5268	19	5176	
QEN71_RS31790	LysR family transcriptional regulator	QEN71_31785	paras_007277	protein-codi	NZ_CP1252	chromosom	482726	483586	-	861	0	10	2591	8	2356	
QEN71_RS31795	multidrug transporter subunit MdtD	QEN71_31790	paras_007278	protein-codi	NZ_CP1252	chromosom	483621	485126	+	1506	0	24	4177	21	3734	
QEN71_RS31800	LysR substrate-binding domain-containing protein	QEN71_31795	paras_007279	protein-codi	NZ_CP1252	chromosom	485135	486052	-	918	0	14	2381	12	2097	
QEN71_RS31805	hydroxymethylglutaryl-CoA lyase	QEN71_31800	paras_007280	protein-codi	NZ_CP1252	chromosom	486190	487137	+	948	0	11	1356	10	1335	
QEN71_RS31810	CaiB/BaiF CoA-transferase family protein	QEN71_31805	paras_007281	protein-codi	NZ_CP1252	chromosom	487146	488297	+	1152	0	16	1806	13	1051	
QEN71_RS31815	MFS transporter	QEN71_31810	paras_007282	protein-codi	NZ_CP1252	chromosom	488394	489833	+	1440	0	25	3472	19	2551	
QEN71_RS31820	malate synthase G	QEN71_31815	paras_007283	protein-codi	NZ_CP1252	chromosom	489913	492093	-	2181	0	24	3586	18	2898	
QEN71_RS31825	heme-binding protein	QEN71_31820	paras_007284	protein-codi	NZ_CP1252	chromosom	492162	492578	-	417	0	8	1125	8	1125	
QEN71_RS31830	helix-turn-helix domain-containing GNAT family N-a	QEN71_31825	paras_007285	protein-codi	NZ_CP1252	chromosom	492959	493963	-	1005	0	20	2801	18	2750	
QEN71_RS31835	thioredoxin family protein	QEN71_31830	paras_007286	protein-codi	NZ_CP1252	chromosom	494044	494595	-	552	0	10	1096	8	938	
QEN71_RS31840	glucosidase	QEN71_31835	paras_007287	protein-codi	NZ_CP1252	chromosom	494673	497417	-	2745	0	65	8213	57	7298	

QEN71_RS31845	pseudouridine-5'-phosphate glycosidase	QEN71_31840	paras_007288	protein-codi	NZ_CP1252	chromosom	497630	498556	+	927	0	4	794	3	743	
QEN71_RS31850	molybdopterin-dependent oxidoreductase	QEN71_31845	paras_007289	protein-codi	NZ_CP1252	chromosom	498578	499174	-	597	0	17	2692	14	2096	
QEN71_RS31855	cupin domain-containing protein	QEN71_31850	paras_007290	protein-codi	NZ_CP1252	chromosom	499359	499772	+	414	0	11	3528	7	2690	
QEN71_RS31860	alpha/beta hydrolase	QEN71_31855	paras_007291	protein-codi	NZ_CP1252	chromosom	499898	500749	+	852	0	8	1161	5	509	
QEN71_RS31865	GNAT family N-acetyltransferase	QEN71_31860	paras_007292	protein-codi	NZ_CP1252	chromosom	500766	501281	-	516	0	13	1716	12	1649	
QEN71_RS31870	MFS transporter	QEN71_31865	paras_007293	protein-codi	NZ_CP1252	chromosom	501591	502976	+	1386	0	41	8171	28	5197	
QEN71_RS31875	GNAT family N-acetyltransferase	QEN71_31870	paras_007294	protein-codi	NZ_CP1252	chromosom	503023	503919	-	897	0	3	1091	2	53	
QEN71_RS31880	aldehyde dehydrogenase family protein	QEN71_31875	paras_007295	protein-codi	NZ_CP1252	chromosom	504132	505622	+	1491	0	18	3639	12	2312	
QEN71_RS31885	haloacid dehalogenase type II	QEN71_31880	paras_007296	protein-codi	NZ_CP1252	chromosom	505839	506507	+	669	0	15	1921	8	940	
QEN71_RS31890	FAD-binding oxidoreductase	QEN71_31885	paras_007297	protein-codi	NZ_CP1252	chromosom	506548	507822	+	1275	0	25	1949	18	1316	
QEN71_RS31895	ABC transporter substrate-binding protein	QEN71_31890	paras_007298	protein-codi	NZ_CP1252	chromosom	507901	509523	+	1623	0	19	1433	17	1410	
QEN71_RS31900	ABC transporter permease	QEN71_31895	paras_007299	protein-codi	NZ_CP1252	chromosom	509591	510547	+	953	0	19	1366	18	1257	
QEN71_RS31905	ABC transporter permease	QEN71_31900	paras_007300	protein-codi	NZ_CP1252	chromosom	510544	511503	+	952	0	16	1208	15	1193	
QEN71_RS31910	ABC transporter ATP-binding protein	QEN71_31905	paras_007301	protein-codi	NZ_CP1252	chromosom	511500	513329	+	1822	0	21	1456	18	1403	
QEN71_RS31915	(2Fe-2S)-binding protein	QEN71_31910	paras_007302	protein-codi	NZ_CP1252	chromosom	513326	513646	+	295	0	1	62	0	0	TRUE
QEN71_RS31920	NAD(P)/FAD-dependent oxidoreductase	QEN71_31915	paras_007303	protein-codi	NZ_CP1252	chromosom	513625	515064	+	1414	0	16	1927	16	1927	
QEN71_RS31925	FAD-binding oxidoreductase	QEN71_31920	paras_007304	protein-codi	NZ_CP1252	chromosom	515061	516224	+	1156	0	18	1231	14	952	
QEN71_RS31930	AraC family transcriptional regulator	QEN71_31925	paras_007305	protein-codi	NZ_CP1252	chromosom	516221	516559	+	335	0	12	1112	12	1112	
QEN71_RS31935	LysR family transcriptional regulator	QEN71_31930	paras_007306	protein-codi	NZ_CP1252	chromosom	517189	518160	-	972	0	18	1789	15	1571	
QEN71_RS31940	ABC transporter ATP-binding protein	QEN71_31935	paras_007307	protein-codi	NZ_CP1252	chromosom	518261	519337	+	1077	0	14	2307	9	1433	
QEN71_RS31945	HAD-IA family hydrolase	QEN71_31940	paras_007308	protein-codi	NZ_CP1252	chromosom	519429	520139	+	711	0	12	1708	12	1708	
QEN71_RS31950	FAD-binding oxidoreductase	QEN71_31945	paras_007309	protein-codi	NZ_CP1252	chromosom	520142	521470	+	1325	0	22	2374	22	2374	
QEN71_RS31955	ABC transporter permease	QEN71_31950	paras_007310	protein-codi	NZ_CP1252	chromosom	521467	522378	+	904	0	16	1663	15	1576	
QEN71_RS31960	ABC transporter permease	QEN71_31955	paras_007311	protein-codi	NZ_CP1252	chromosom	522375	523172	+	794	0	9	970	9	970	
QEN71_RS31965	aldehyde dehydrogenase family protein	QEN71_31960	paras_007312	protein-codi	NZ_CP1252	chromosom	523193	524617	+	1425	0	17	2259	11	1183	
QEN71_RS31970	helix-turn-helix domain-containing protein	QEN71_31965	paras_007313	protein-codi	NZ_CP1252	chromosom	524717	525184	+	464	0	11	382	6	164	
QEN71_RS31975	PIN domain-containing protein	QEN71_31970	paras_007314	protein-codi	NZ_CP1252	chromosom	525181	525756	+	468	0	11	735	10	436	
QEN71_RS31980	GMC family oxidoreductase N-terminal domain-cont	QEN71_31975	paras_007315	protein-codi	NZ_CP1252	chromosom	525653	527419	-	1663	0	34	3143	27	2628	
QEN71_RS31985	ABC transporter substrate-binding protein	QEN71_31980	paras_007316	protein-codi	NZ_CP1252	chromosom	527518	528528	-	1011	0	13	1441	10	1098	
QEN71_RS31990	cupin domain-containing protein	QEN71_31985	paras_007317	protein-codi	NZ_CP1252	chromosom	528791	529135	-	345	0	5	229	4	117	
QEN71_RS31995	M14 family metallopeptidase	QEN71_31990	paras_007318	protein-codi	NZ_CP1252	chromosom	529173	530150	-	974	0	15	2874	6	655	
QEN71_RS32000	NAD-dependent succinate-semialdehyde dehydrog	QEN71_31995	paras_007319	protein-codi	NZ_CP1252	chromosom	530147	531619	-	1469	0	27	3438	20	2646	
QEN71_RS32005	tartrate dehydrogenase	QEN71_32000	paras_007320	protein-codi	NZ_CP1252	chromosom	531664	532743	-	1080	0	17	1152	12	882	
QEN71_RS32010	LysR substrate-binding domain-containing protein	QEN71_32005	paras_007321	protein-codi	NZ_CP1252	chromosom	532873	533790	+	911	0	9	1226	9	1226	
QEN71_RS32015	winged helix-turn-helix transcriptional regulator	QEN71_32010	paras_007322	protein-codi	NZ_CP1252	chromosom	533784	534281	-	491	0	13	1318	13	1318	
QEN71_RS32020	aspartate aminotransferase family protein	QEN71_32015	paras_007323	protein-codi	NZ_CP1252	chromosom	534326	535735	-	1410	0	22	2692	17	2579	
QEN71_RS32025	glyoxylate/hydroxypyruvate reductase A	QEN71_32020	paras_007324	protein-codi	NZ_CP1252	chromosom	535880	536806	-	927	0	8	1361	6	841	
QEN71_RS32030	GNAT family N-acetyltransferase	QEN71_32025	paras_007325	protein-codi	NZ_CP1252	chromosom	536810	537694	-	885	0	9	545	5	251	
QEN71_RS32035	hypothetical protein	QEN71_32030	paras_007326	protein-codi	NZ_CP1252	chromosom	537905	538069	+	165	0	2	35	0	0	
QEN71_RS32040	universal stress protein	QEN71_32035	paras_007327	protein-codi	NZ_CP1252	chromosom	538125	538610	+	486	0	10	921	8	845	
QEN71_RS32045	hypothetical protein	QEN71_32040	paras_007328	protein-codi	NZ_CP1252	chromosom	538624	538908	-	285	0	6	1185	4	1099	
QEN71_RS32050	hypothetical protein	QEN71_32045	paras_007329	protein-codi	NZ_CP1252	chromosom	538919	539128	-	210	0	3	53	3	53	
QEN71_RS32055	hypothetical protein	QEN71_32050	paras_007330	protein-codi	NZ_CP1252	chromosom	539193	539441	-	249	0	0	0	0	0	
QEN71_RS32060	hypothetical protein	QEN71_32055	paras_007331	protein-codi	NZ_CP1252	chromosom	539489	539758	+	266	0	6	1395	6	1395	
QEN71_RS32065	hypothetical protein	QEN71_32060	paras_007332	protein-codi	NZ_CP1252	chromosom	539755	540108	+	350	0	4	572	3	318	
QEN71_RS32070	ATP-binding protein	QEN71_32065	paras_007333	protein-codi	NZ_CP1252	chromosom	540206	542368	+	2159	111.0	25	2892	17	1996	
QEN71_RS32075	response regulator	QEN71_32070	paras_007334	protein-codi	NZ_CP1252	chromosom	542365	543990	+	1618	0	13	1101	7	677	
QEN71_RS32080	response regulator transcription factor	QEN71_32075	paras_007335	protein-codi	NZ_CP1252	chromosom	543987	544619	+	629	0	5	593	3	338	
QEN71_RS32085	hypothetical protein	QEN71_32080	paras_007336	protein-codi	NZ_CP1252	chromosom	544794	545669	+	876	0	13	1726	7	992	
QEN71_RS32090	hypothetical protein	QEN71_32085	paras_007337	protein-codi	NZ_CP1252	chromosom	545782	545961	+	180	0	3	221	2	82	
QEN71_RS32095	DUF1840 domain-containing protein	QEN71_32090	paras_007338	protein-codi	NZ_CP1252	chromosom	546144	546458	+	315	0	8	765	6	688	
QEN71_RS32100	universal stress protein	QEN71_32095	paras_007339	protein-codi	NZ_CP1252	chromosom	546494	546940	-	447	0	9	1298	8	1075	
QEN71_RS32105	hypothetical protein	QEN71_32100	paras_007340	protein-codi	NZ_CP1252	chromosom	546973	547158	-	186	0	4	449	0	0	
QEN71_RS32110	phasin family protein	QEN71_32105	paras_007341	protein-codi	NZ_CP1252	chromosom	547545	548069	+	525	0	4	319	3	226	
QEN71_RS32115	acetoacetyl-CoA reductase	QEN71_32110	paras_007342	protein-codi	NZ_CP1252	chromosom	548260	549003	+	744	0	3	276	3	276	
QEN71_RS32120	hypothetical protein	QEN71_32115	paras_007343	protein-codi	NZ_CP1252	chromosom	549090	549266	+	177	0	1	18	1	18	
QEN71_RS32125	hypothetical protein	QEN71_32120	paras_007344	protein-codi	NZ_CP1252	chromosom	549312	549470	+	159	0	3	193	3	193	
QEN71_RS32130	LysR substrate-binding domain-containing protein	QEN71_32125	paras_007345	protein-codi	NZ_CP1252	chromosom	549492	550739	-	1248	0	13	1632	10	1178	
QEN71_RS32135	GntR family transcriptional regulator	QEN71_32130	paras_007346	protein-codi	NZ_CP1252	chromosom	550789	551490	-	702	0	14	1848	11	1098	
QEN71_RS32140	porin	QEN71_32135	paras_007347	protein-codi	NZ_CP1252	chromosom	552017	553237	+	1221	0	29	2822	29	2822	
QEN71_RS32145	5'/3'-nucleotidase SurE	QEN71_32140	paras_007348	protein-codi	NZ_CP1252	chromosom	553328	554113	+	786	0	5	386	2	147	
QEN71_RS32150	DEAD/DEAH box helicase	QEN71_32145	paras_007349	protein-codi	NZ_CP1252	chromosom	554203	555666	+	1438	0	23	2568	16	1472	

QEN71_RS32155	hypothetical protein	QEN71_32150	paras_007350	protein-codi	NZ_CP1252	chromosom	555641	556042	+	376	0	9	831	4	451	
QEN71_RS32160	helix-turn-helix domain-containing protein	QEN71_32155	paras_007351	protein-codi	NZ_CP1252	chromosom	556081	557118	-	1038	0	19	2299	14	1083	
QEN71_RS32165	homocysteine S-methyltransferase family protein	QEN71_32160	paras_007352	protein-codi	NZ_CP1252	chromosom	557276	558250	+	975	0	18	4179	18	4179	
QEN71_RS32170	glycoside hydrolase family 28 protein	QEN71_32165	paras_007353	protein-codi	NZ_CP1252	chromosom	558322	560514	-	2193	0	41	5245	33	4589	
QEN71_RS32175	glycosyltransferase			pseudogene	NZ_CP1252	chromosom	561626	562102	+	477	0	6	477	6	477	
QEN71_RS32180	ATP-grasp fold amidoligase family protein	QEN71_32175	paras_007354	protein-codi	NZ_CP1252	chromosom	562256	563152	+	897	0	21	2000	19	1983	
QEN71_RS32185	GNAT family N-acetyltransferase	QEN71_32180	paras_007355	protein-codi	NZ_CP1252	chromosom	563158	564252	-	1095	0	14	977	10	886	
QEN71_RS32190	glycosyltransferase family A protein	QEN71_32185	paras_007356	protein-codi	NZ_CP1252	chromosom	564363	565361	-	999	0	42	6217	26	3352	
QEN71_RS32195	hypothetical protein	QEN71_32190	paras_007357	protein-codi	NZ_CP1252	chromosom	565956	566240	+	285	0	6	572	6	572	
QEN71_RS32200	MarR family transcriptional regulator	QEN71_32195	paras_007358	protein-codi	NZ_CP1252	chromosom	566273	566719	-	447	0	7	903	6	835	
QEN71_RS32205	molybdopterin-dependent oxidoreductase	QEN71_32200	paras_007359	protein-codi	NZ_CP1252	chromosom	566803	569322	-	2520	0	52	8299	36	5313	
QEN71_RS32210	FAD binding domain-containing protein	QEN71_32205	paras_007360	protein-codi	NZ_CP1252	chromosom	569549	570394	-	846	0	21	2268	15	1363	
QEN71_RS32215	sodium:solute symporter	QEN71_32210	paras_007361	protein-codi	NZ_CP1252	chromosom	570445	571902	-	1454	0	57	6604	52	5977	
QEN71_RS32220	DUF3311 domain-containing protein	QEN71_32215	paras_007362	protein-codi	NZ_CP1252	chromosom	571899	572252	-	350	0	12	893	8	581	
QEN71_RS32225	ATP-binding protein	QEN71_32220	paras_007363	protein-codi	NZ_CP1252	chromosom	572665	573657	+	993	0	23	4062	20	3905	
QEN71_RS32230	alpha/beta hydrolase	QEN71_32225	paras_007364	protein-codi	NZ_CP1252	chromosom	574015	575049	+	1035	0	28	3017	21	2202	
QEN71_RS32235	cytochrome P460 family protein	QEN71_32230	paras_007365	protein-codi	NZ_CP1252	chromosom	575065	575577	+	513	0	7	1181	4	481	
QEN71_RS32240	anti-sigma factor	QEN71_32235	paras_007366	protein-codi	NZ_CP1252	chromosom	575670	576335	-	666	0	16	586	12	457	
QEN71_RS32245	RNA polymerase sigma factor	QEN71_32240	paras_007367	protein-codi	NZ_CP1252	chromosom	576506	577048	-	543	0	5	226	5	226	
QEN71_RS32250	GNAT family N-acetyltransferase	QEN71_32245	paras_007368	protein-codi	NZ_CP1252	chromosom	577225	578004	+	780	0	24	1762	21	1614	
QEN71_RS32255	alpha/beta hydrolase	QEN71_32250	paras_007369	protein-codi	NZ_CP1252	chromosom	578023	578907	-	885	0	25	1968	17	1081	
QEN71_RS32260	LysR substrate-binding domain-containing protein	QEN71_32255	paras_007370	protein-codi	NZ_CP1252	chromosom	579009	579911	+	903	0	9	1346	9	1346	
QEN71_RS32265	diguanylate cyclase	QEN71_32260	paras_007371	protein-codi	NZ_CP1252	chromosom	579996	581531	+	1536	0	34	5377	30	4833	
QEN71_RS32270	ubiquinol-cytochrome c reductase iron-sulfur subun	QEN71_32265	paras_007372	protein-codi	NZ_CP1252	chromosom	581729	582355	+	627	0	8	892	6	599	
QEN71_RS32275	hypothetical protein	QEN71_32270	paras_007373	protein-codi	NZ_CP1252	chromosom	582400	582711	+	312	0	8	916	5	592	
QEN71_RS32280	lyase	QEN71_32275	paras_007374	protein-codi	NZ_CP1252	chromosom	582916	583863	-	948	0	21	3058	19	2900	
QEN71_RS32285	branched-chain amino acid ABC transporter substra	QEN71_32280	paras_007375	protein-codi	NZ_CP1252	chromosom	584442	585578	+	1137	0	30	3411	24	3078	
QEN71_RS32290	metallophosphoesterase family protein	QEN71_32285	paras_007376	protein-codi	NZ_CP1252	chromosom	585687	587405	-	1719	0	37	4692	29	3886	
QEN71_RS32295	hypothetical protein	QEN71_32290	paras_007377	protein-codi	NZ_CP1252	chromosom	587910	588215	+	306	0	5	750	3	524	
QEN71_RS32300	pyridoxal phosphate-dependent aminotransferase	QEN71_32295	paras_007378	protein-codi	NZ_CP1252	chromosom	588288	589445	+	1158	0	14	2783	10	2240	
QEN71_RS32305	alpha/beta hydrolase	QEN71_32300	paras_007379	protein-codi	NZ_CP1252	chromosom	589465	590268	-	804	0	18	1954	10	863	
QEN71_RS32310	hypothetical protein	QEN71_32305	paras_007380	protein-codi	NZ_CP1252	chromosom	590397	590612	-	216	0	5	792	4	736	
QEN71_RS32315	ParA family protein	QEN71_32310	paras_007381	protein-codi	NZ_CP1252	chromosom	590840	591604	+	765	0	25	5049	18	3959	
QEN71_RS32320	OmpW family outer membrane protein	QEN71_32315	paras_007382	protein-codi	NZ_CP1252	chromosom	591729	592460	+	732	0	22	3807	18	3377	
QEN71_RS32325	hypothetical protein	QEN71_32320	paras_007383	protein-codi	NZ_CP1252	chromosom	592566	592985	-	420	0	16	3755	9	871	
QEN71_RS32330	beta-galactosidase	QEN71_32325	paras_007384	protein-codi	NZ_CP1252	chromosom	593170	595071	+	1874	0	56	10058	49	9582	
QEN71_RS32335	enterotoxin	QEN71_32330	paras_007385	protein-codi	NZ_CP1252	chromosom	595044	596990	-	1919	0	45	7006	36	5542	
QEN71_RS32340	antibiotic biosynthesis monooxygenase	QEN71_32335	paras_007386	protein-codi	NZ_CP1252	chromosom	597006	597320	-	315	0	5	381	4	370	
QEN71_RS32345	MFS transporter	QEN71_32340	paras_007387	protein-codi	NZ_CP1252	chromosom	597422	598684	-	1263	0	19	2262	14	1698	
QEN71_RS32350	YbfB/YjiJ family MFS transporter	QEN71_32345	paras_007388	protein-codi	NZ_CP1252	chromosom	598736	599950	-	1215	0	15	794	8	331	
QEN71_RS32355	SDR family oxidoreductase	QEN71_32350	paras_007389	protein-codi	NZ_CP1252	chromosom	599975	600685	-	711	0	12	1494	11	1412	
QEN71_RS32360	LysR family transcriptional regulator	QEN71_32355	paras_007390	protein-codi	NZ_CP1252	chromosom	600801	601727	-	927	0	12	1470	10	1029	
QEN71_RS32365	serine hydrolase	QEN71_32360	paras_007391	protein-codi	NZ_CP1252	chromosom	601816	602637	-	822	0	12	1239	12	1239	
QEN71_RS32370	M23 family metallopeptidase	QEN71_32365	paras_007392	protein-codi	NZ_CP1252	chromosom	602707	603717	-	1011	0	10	1003	10	1003	
QEN71_RS32375	carboxypeptidase-like regulatory domain-containing	QEN71_32370	paras_007393	protein-codi	NZ_CP1252	chromosom	604023	604475	-	453	0	6	1158	6	1158	
QEN71_RS32380	VOC family protein	QEN71_32375	paras_007394	protein-codi	NZ_CP1252	chromosom	604612	604992	-	377	0	8	722	7	712	
QEN71_RS32385	MFS transporter	QEN71_32380	paras_007395	protein-codi	NZ_CP1252	chromosom	604989	606296	-	1304	0	25	5040	23	4758	
QEN71_RS32390	LysR family transcriptional regulator	QEN71_32385	paras_007396	protein-codi	NZ_CP1252	chromosom	606399	607271	+	873	0	7	573	6	535	
QEN71_RS32395	DUF3563 family protein	QEN71_32390	paras_007397	protein-codi	NZ_CP1252	chromosom	607276	607413	-	138	0	3	381	2	328	
QEN71_RS32400	CPBP family glutamic-type intramembrane protease	QEN71_32395	paras_007398	protein-codi	NZ_CP1252	chromosom	607629	608117	+	489	0	10	1075	10	1075	
QEN71_RS32405	MFS transporter	QEN71_32400	paras_007399	protein-codi	NZ_CP1252	chromosom	608145	609353	+	1209	0	16	1557	10	1077	
QEN71_RS32410	flagellin domain-containing protein	QEN71_32405	paras_007400	protein-codi	NZ_CP1252	chromosom	609752	610570	+	819	0	6	532	3	73	
QEN71_RS32415	aldo/keto reductase family oxidoreductase	QEN71_32410	paras_007401	protein-codi	NZ_CP1252	chromosom	610627	611502	-	876	0	18	3461	14	3130	
QEN71_RS32420	LysR family transcriptional regulator	QEN71_32415	paras_007402	protein-codi	NZ_CP1252	chromosom	611607	612518	+	912	0	10	1118	8	1011	
QEN71_RS32425	dihydroxyacetone kinase family protein	QEN71_32420	paras_007403	protein-codi	NZ_CP1252	chromosom	612623	614329	+	1707	0	14	2084	10	1374	
QEN71_RS32430	ATP-binding protein	QEN71_32425	paras_007404	protein-codi	NZ_CP1252	chromosom	614647	615759	+	1113	0	6	690	6	690	
QEN71_RS32435	DUF1003 domain-containing protein	QEN71_32430	paras_007405	protein-codi	NZ_CP1252	chromosom	615777	616352	-	576	0	3	307	2	170	
QEN71_RS32440	alpha/beta hydrolase	QEN71_32435	paras_007406	protein-codi	NZ_CP1252	chromosom	616707	617528	+	822	0	14	536	6	346	
QEN71_RS32445	DUF971 domain-containing protein	QEN71_32440	paras_007407	protein-codi	NZ_CP1252	chromosom	617588	617875	-	284	0	3	56	3	56	
QEN71_RS32450	HEAT repeat domain-containing protein	QEN71_32445	paras_007408	protein-codi	NZ_CP1252	chromosom	617872	618867	-	992	0	5	104	4	100	
QEN71_RS32455	ABC transporter ATP-binding protein	QEN71_32450	paras_007409	protein-codi	NZ_CP1252	chromosom	618887	619678	-	788	0	2	121	2	121	
QEN71_RS32460	ABC transporter permease	QEN71_32455	paras_007410	protein-codi	NZ_CP1252	chromosom	619675	620520	-	842	0	6	362	6	362	

QEN71_RS32465	ABC transporter substrate-binding protein	QEN71_32460	paras_007411	protein-codi	NZ_CP1252	chromosom	620558	621991	-	1434	0	18	1736	15	1524	
QEN71_RS32470	ferredoxin family protein	QEN71_32465	paras_007412	protein-codi	NZ_CP1252	chromosom	622041	622286	-	246	0	6	1105	4	928	
QEN71_RS32475	fumarate reductase/succinate dehydrogenase flavo	QEN71_32470	paras_007413	protein-codi	NZ_CP1252	chromosom	622301	624067	-	1763	0	30	3361	19	2100	
QEN71_RS32480	GntR family transcriptional regulator	QEN71_32475	paras_007414	protein-codi	NZ_CP1252	chromosom	624064	624855	-	788	0	9	797	7	675	
QEN71_RS32485	aminotransferase class V-fold PLP-dependent enzy	QEN71_32480	paras_007415	protein-codi	NZ_CP1252	chromosom	625047	626390	-	1344	0	10	788	3	270	
QEN71_RS32490	5-dehydro-4-deoxyglucarate dehydratase	QEN71_32485	paras_007416	protein-codi	NZ_CP1252	chromosom	626757	627674	-	918	0	17	2338	16	2326	
QEN71_RS32495	glucarate dehydratase	QEN71_32490	paras_007417	protein-codi	NZ_CP1252	chromosom	627758	629107	-	1350	0	15	887	8	507	
QEN71_RS32500	beta-propeller fold lactonase family protein	QEN71_32495	paras_007418	protein-codi	NZ_CP1252	chromosom	629199	630278	-	1080	0	24	3622	20	3334	
QEN71_RS32505	2-hydroxy-3-oxopropionate reductase	QEN71_32500	paras_007419	protein-codi	NZ_CP1252	chromosom	630310	631200	-	891	0	3	231	2	149	
QEN71_RS32510	2-dehydro-3-deoxyglucarate aldolase	QEN71_32505	paras_007420	protein-codi	NZ_CP1252	chromosom	631243	632028	-	786	0	10	2043	8	1866	
QEN71_RS32515	aldehyde dehydrogenase (NADP(+))	QEN71_32510	paras_007421	protein-codi	NZ_CP1252	chromosom	632257	633840	+	1584	0	9	497	6	262	
QEN71_RS32520	FadR/GntR family transcriptional regulator	QEN71_32515	paras_007422	protein-codi	NZ_CP1252	chromosom	633894	634655	-	762	0	9	1849	8	1752	
QEN71_RS32525	porin	QEN71_32520	paras_007423	protein-codi	NZ_CP1252	chromosom	635210	636361	+	1152	0	26	3536	24	3318	
QEN71_RS32530	flagellin domain-containing protein	QEN71_32525	paras_007424	protein-codi	NZ_CP1252	chromosom	636601	637419	+	819	0	5	472	3	369	
QEN71_RS32535	GGDEF domain-containing protein	QEN71_32530	paras_007425	protein-codi	NZ_CP1252	chromosom	637637	638767	+	1131	0	18	4575	14	3990	
QEN71_RS32540	GntR family transcriptional regulator	QEN71_32535	paras_007426	protein-codi	NZ_CP1252	chromosom	638909	639595	+	687	0	16	2567	10	1432	
QEN71_RS32545	amino acid synthesis family protein	QEN71_32540	paras_007427	protein-codi	NZ_CP1252	chromosom	639832	640419	+	577	0	14	2710	6	1301	
QEN71_RS32550	alpha/beta fold hydrolase	QEN71_32545	paras_007428	protein-codi	NZ_CP1252	chromosom	640409	641305	+	878	0	13	1333	10	754	
QEN71_RS32555	flavin reductase family protein	QEN71_32550	paras_007429	protein-codi	NZ_CP1252	chromosom	641298	642257	+	952	0	14	1534	10	1092	
QEN71_RS32560	LLM class flavin-dependent oxidoreductase	QEN71_32555	paras_007430	protein-codi	NZ_CP1252	chromosom	642274	643317	+	1044	0	17	1700	8	773	
QEN71_RS32565	aldehyde dehydrogenase	QEN71_32560	paras_007431	protein-codi	NZ_CP1252	chromosom	643354	644826	+	1473	0	16	902	12	717	
QEN71_RS32570	NIPSNAP family protein	QEN71_32565	paras_007432	protein-codi	NZ_CP1252	chromosom	644854	645168	+	315	0	7	720	6	173	
QEN71_RS32575	ABC transporter substrate-binding protein	QEN71_32570	paras_007433	protein-codi	NZ_CP1252	chromosom	645252	646271	+	1020	152.0	13	1097	6	446	
QEN71_RS32580	ABC transporter permease	QEN71_32575	paras_007434	protein-codi	NZ_CP1252	chromosom	646330	647214	+	885	0	13	1177	10	681	
QEN71_RS32585	ABC transporter permease	QEN71_32580	paras_007435	protein-codi	NZ_CP1252	chromosom	647216	648010	+	795	117.0	5	525	2	251	
QEN71_RS32590	ABC transporter ATP-binding protein	QEN71_32585	paras_007436	protein-codi	NZ_CP1252	chromosom	648037	649134	+	1098	0	14	922	11	898	
QEN71_RS32595	GntR family transcriptional regulator	QEN71_32590	paras_007437	protein-codi	NZ_CP1252	chromosom	649143	649838	+	696	0	4	191	3	123	
QEN71_RS32600	nuclear transport factor 2 family protein	QEN71_32595	paras_007438	protein-codi	NZ_CP1252	chromosom	649964	650443	+	480	0	5	220	3	28	
QEN71_RS32605	TIGR00366 family protein	QEN71_32600	paras_007439	protein-codi	NZ_CP1252	chromosom	650589	651911	+	1323	0	16	1097	10	682	
QEN71_RS32610	histone deacetylase family protein	QEN71_32605	paras_007440	protein-codi	NZ_CP1252	chromosom	651974	653008	+	1035	0	30	3482	18	1506	
QEN71_RS32615	hypothetical protein	QEN71_32610	paras_007441	protein-codi	NZ_CP1252	chromosom	653146	653430	+	285	0	7	1929	4	602	
QEN71_RS32620	CDP-alcohol phosphatidyltransferase family protein	QEN71_32615	paras_007442	protein-codi	NZ_CP1252	chromosom	653443	654051	-	609	0	18	2145	17	1926	
QEN71_RS32625	efflux RND transporter permease subunit	QEN71_32620	paras_007443	protein-codi	NZ_CP1252	chromosom	654289	657531	+	3235	0	32	2725	30	2606	
QEN71_RS32630	efflux RND transporter periplasmic adaptor subunit	QEN71_32625	paras_007444	protein-codi	NZ_CP1252	chromosom	657524	658762	+	1231	0	20	1578	19	1566	
QEN71_RS32635	efflux transporter outer membrane subunit	QEN71_32630	paras_007445	protein-codi	NZ_CP1252	chromosom	658771	660270	+	1500	0	11	700	9	522	
QEN71_RS32640	heavy metal response regulator transcription factor	QEN71_32635	paras_007446	protein-codi	NZ_CP1252	chromosom	660348	661034	+	683	0	11	1660	7	1291	
QEN71_RS32645	heavy metal sensor histidine kinase	QEN71_32640	paras_007447	protein-codi	NZ_CP1252	chromosom	661031	662491	+	1457	0	14	1504	13	1501	
QEN71_RS32650	hypothetical protein	QEN71_32645	paras_007448	protein-codi	NZ_CP1252	chromosom	662641	662826	+	186	0	2	1081	2	1081	
QEN71_RS32655	DUF3280 domain-containing protein	QEN71_32650	paras_007449	protein-codi	NZ_CP1252	chromosom	662951	663520	+	570	0	4	501	4	501	
QEN71_RS32660	cytochrome D1 domain-containing protein	QEN71_32655	paras_007450	protein-codi	NZ_CP1252	chromosom	663688	664869	+	1182	0	25	3408	14	2566	
QEN71_RS32665	histidine kinase	QEN71_32660	paras_007451	protein-codi	NZ_CP1252	chromosom	665065	666069	+	944	0	10	1712	10	1712	
QEN71_RS32670	response regulator transcription factor	QEN71_32665	paras_007452	protein-codi	NZ_CP1252	chromosom	666009	666725	-	652	0	13	1682	13	1682	
QEN71_RS32675	pyrroloquinoline quinone biosynthesis protein PqqE	QEN71_32670	paras_007453	protein-codi	NZ_CP1252	chromosom	666722	667939	-	1214	0	13	1366	12	1328	
QEN71_RS32680	pyrroloquinoline quinone biosynthesis peptide chap	QEN71_32675	paras_007454	protein-codi	NZ_CP1252	chromosom	667944	668234	-	287	0	2	4	1	2	
QEN71_RS32685	pyrroloquinoline-quinone synthase PqqC	QEN71_32680	paras_007455	protein-codi	NZ_CP1252	chromosom	668231	668938	-	704	0	12	836	11	826	
QEN71_RS32690	pyrroloquinoline quinone biosynthesis protein PqqB	QEN71_32685	paras_007456	protein-codi	NZ_CP1252	chromosom	668969	669889	-	921	0	11	1633	11	1633	
QEN71_RS32695	cytochrome b	QEN71_32690	paras_007457	protein-codi	NZ_CP1252	chromosom	669960	670526	-	567	0	9	1366	9	1366	
QEN71_RS32700	formylmethanofuran dehydrogenase subunit C	QEN71_32695	paras_007458	protein-codi	NZ_CP1252	chromosom	670558	671421	-	860	0	7	1222	7	1222	
QEN71_RS32705	formylmethanofuran--tetrahydromethanopterin N-for	QEN71_32700	paras_007459	protein-codi	NZ_CP1252	chromosom	671418	672362	-	941	0	9	788	6	499	
QEN71_RS32710	formylmethanofuran dehydrogenase subunit A	QEN71_32705	paras_007460	protein-codi	NZ_CP1252	chromosom	672376	674076	-	1697	0	20	3024	13	2206	
QEN71_RS32715	formylmethanofuran dehydrogenase	QEN71_32710	paras_007461	protein-codi	NZ_CP1252	chromosom	674073	675482	-	1406	0	8	688	5	245	
QEN71_RS32720	HisA/HisF-related TIM barrel protein	QEN71_32715	paras_007462	protein-codi	NZ_CP1252	chromosom	675775	676554	-	780	0	6	368	5	247	
QEN71_RS32725	ATP-grasp domain-containing protein	QEN71_32720	paras_007463	protein-codi	NZ_CP1252	chromosom	676571	677596	+	1022	0	11	1676	7	1165	
QEN71_RS32730	hydantoinase/oxoprolinase family protein	QEN71_32725	paras_007464	protein-codi	NZ_CP1252	chromosom	677593	678672	+	1076	0	9	1228	9	1228	
QEN71_RS32735	aspartate kinase	QEN71_32730	paras_007465	protein-codi	NZ_CP1252	chromosom	678770	679360	+	591	0	7	630	6	514	
QEN71_RS32740	(5-formylfuran-3-yl)methyl phosphate synthase	QEN71_32735	paras_007466	protein-codi	NZ_CP1252	chromosom	679400	680098	-	699	0	3	780	3	780	
QEN71_RS32745	DUF447 family protein	QEN71_32740	paras_007467	protein-codi	NZ_CP1252	chromosom	680123	680734	-	612	0	9	1318	7	645	
QEN71_RS32750	hypothetical protein	QEN71_32745	paras_007468	protein-codi	NZ_CP1252	chromosom	681161	681463	+	277	252.0	13	3425	10	2790	
QEN71_RS32755	pyrroloquinoline quinone precursor peptide PqqA	QEN71_32750	paras_007469	protein-codi	NZ_CP1252	chromosom	681438	681512	-	49	0	5	742	4	579	
QEN71_RS32760	form I ribulose bisphosphate carboxylase large subu	QEN71_32755	paras_007470	protein-codi	NZ_CP1252	chromosom	681908	683398	+	1491	0	21	2168	15	1601	
QEN71_RS32765	ribulose bisphosphate carboxylase small subunit	QEN71_32760	paras_007471	protein-codi	NZ_CP1252	chromosom	683437	683838	+	402	0	4	627	3	415	
QEN71_RS32770	CbbX protein	QEN71_32765	paras_007472	protein-codi	NZ_CP1252	chromosom	683872	684840	+	969	0	13	2477	12	2374	

QEN71_RS32775	class 1 fructose-bisphosphatase	QEN71_32770	paras_007473	protein-codi	NZ_CP1252	chromosom	684850	685950	+	1101	0	12	677	11	636	
QEN71_RS32780	phosphoribulokinase	QEN71_32775	paras_007474	protein-codi	NZ_CP1252	chromosom	685984	686856	+	873	0	18	1460	14	1266	
QEN71_RS32785	DUF6513 domain-containing protein	QEN71_32780	paras_007475	protein-codi	NZ_CP1252	chromosom	687483	688862	-	1358	0	13	1570	11	1230	
QEN71_RS32790	flavoprotein	QEN71_32785	paras_007476	protein-codi	NZ_CP1252	chromosom	688841	689440	-	578	0	9	1429	9	1429	
QEN71_RS32795	sigma-54-dependent Fis family transcriptional regul	QEN71_32790	paras_007477	protein-codi	NZ_CP1252	chromosom	689459	691564	-	2106	0	19	4715	15	3863	
QEN71_RS32800	dihydroneopterin aldolase	QEN71_32795	paras_007478	protein-codi	NZ_CP1252	chromosom	691888	692367	+	480	0	7	479	3	133	
QEN71_RS32805	hypothetical protein	QEN71_32800	paras_007479	protein-codi	NZ_CP1252	chromosom	692403	692819	-	417	0	9	1416	9	1416	
QEN71_RS32810	formaldehyde-activating enzyme	QEN71_32805	paras_007480	protein-codi	NZ_CP1252	chromosom	692959	693468	-	510	0	8	825	5	699	
QEN71_RS32815	triphosphoribosyl-dephospho-CoA synthase	QEN71_32810	paras_007481	protein-codi	NZ_CP1252	chromosom	693603	694478	-	862	0	5	970	4	775	
QEN71_RS32820	RimK family alpha-L-glutamate ligase	QEN71_32815	paras_007482	protein-codi	NZ_CP1252	chromosom	694465	695448	-	966	0	18	2087	18	2087	
QEN71_RS32825	methenyltetrahydromethanopterin cyclohydrolase	QEN71_32820	paras_007483	protein-codi	NZ_CP1252	chromosom	695445	696548	-	1074	0	10	894	10	894	
QEN71_RS32830	ATP-grasp domain-containing protein	QEN71_32825	paras_007484	protein-codi	NZ_CP1252	chromosom	696523	697737	-	1189	0	16	1144	14	1072	
QEN71_RS32835	methylenetetrahydromethanopterin dehydrogenase	QEN71_32830	paras_007485	protein-codi	NZ_CP1252	chromosom	697765	698691	-	927	0	19	2406	11	1496	
QEN71_RS32840	'beta-ribofuranosylaminobenzene 5'-phosphate synt	pseudo;QEN71_32835		pseudogene	NZ_CP1252	chromosom	698779	699817	-	1039	0	14	1876	11	1050	
QEN71_RS32845	4a-hydroxytetrahydrobiopterin dehydratase	QEN71_32840	paras_007487	protein-codi	NZ_CP1252	chromosom	700066	700452	-	387	0	17	4044	8	1549	
QEN71_RS32850	methanol/ethanol family PQQ-dependent dehydroge	QEN71_32845	paras_007488	protein-codi	NZ_CP1252	chromosom	700822	702642	+	1821	0	51	9343	42	7663	
QEN71_RS32855	c-type cytochrome	QEN71_32850	paras_007489	protein-codi	NZ_CP1252	chromosom	702770	703207	+	438	0	14	1410	13	1408	
QEN71_RS32860	substrate-binding domain-containing protein	QEN71_32855	paras_007490	protein-codi	NZ_CP1252	chromosom	703310	704227	+	914	0	14	1390	12	1143	
QEN71_RS32865	hypothetical protein	QEN71_32860	paras_007491	protein-codi	NZ_CP1252	chromosom	704224	705048	+	821	0	11	522	11	522	
QEN71_RS32870	LysR family transcriptional regulator	QEN71_32865	paras_007492	protein-codi	NZ_CP1252	chromosom	705106	706074	+	969	0	14	1214	14	1214	
QEN71_RS32875	YbdD/YjiX family protein	QEN71_32870	paras_007493	protein-codi	NZ_CP1252	chromosom	706763	706969	-	207	0	10	1019	10	1019	
QEN71_RS32880	carbon starvation CstA family protein	QEN71_32875	paras_007494	protein-codi	NZ_CP1252	chromosom	706984	709062	-	2079	0	37	3207	24	2760	
QEN71_RS32885	cache domain-containing protein	QEN71_32880	paras_007495	protein-codi	NZ_CP1252	chromosom	709268	710683	+	1412	0	20	2475	15	2057	
QEN71_RS32890	response regulator transcription factor	QEN71_32885	paras_007496	protein-codi	NZ_CP1252	chromosom	710680	711321	+	634	0	8	699	8	699	
QEN71_RS32895	PQQ-binding-like beta-propeller repeat protein	QEN71_32890	paras_007497	protein-codi	NZ_CP1252	chromosom	711318	711986	-	665	0	6	1831	5	1826	
QEN71_RS32900	helix-turn-helix domain-containing protein	QEN71_32895	paras_007498	protein-codi	NZ_CP1252	chromosom	712025	713245	-	1221	0	6	125	1	2	TRUE
QEN71_RS32905	DUF899 domain-containing protein	QEN71_32900	paras_007499	protein-codi	NZ_CP1252	chromosom	713332	714102	+	771	0	19	1942	14	1237	
QEN71_RS32910	DUF2182 domain-containing protein	QEN71_32905	paras_007500	protein-codi	NZ_CP1252	chromosom	714111	714920	+	810	0	2	13	2	13	TRUE
QEN71_RS32915	SDR family oxidoreductase	QEN71_32910	paras_007501	protein-codi	NZ_CP1252	chromosom	714962	715732	-	771	0	4	911	3	705	
QEN71_RS32920	zinc-binding alcohol dehydrogenase family protein	QEN71_32915	paras_007502	protein-codi	NZ_CP1252	chromosom	715765	716736	-	972	0	11	461	7	340	
QEN71_RS32925	alpha/beta hydrolase	QEN71_32920	paras_007503	protein-codi	NZ_CP1252	chromosom	716811	717665	-	855	0	25	4724	23	4593	
QEN71_RS32930	CGNR zinc finger domain-containing protein	QEN71_32925	paras_007504	protein-codi	NZ_CP1252	chromosom	717815	718447	+	633	0	3	224	2	222	
QEN71_RS32935	type 1 glutamine amidotransferase domain-containi	QEN71_32930	paras_007505	protein-codi	NZ_CP1252	chromosom	718859	719536	+	678	0	9	348	7	239	
QEN71_RS32940	enoyl-CoA hydratase/isomerase family protein	QEN71_32935	paras_007506	protein-codi	NZ_CP1252	chromosom	719579	720415	+	837	0	11	661	7	439	
QEN71_RS32945	MmgE/PrpD family protein	QEN71_32940	paras_007507	protein-codi	NZ_CP1252	chromosom	720682	722097	+	1416	0	16	1111	11	954	
QEN71_RS32950	tautomerase family protein	QEN71_32945	paras_007508	protein-codi	NZ_CP1252	chromosom	722116	722346	+	231	0	3	198	1	33	
QEN71_RS32955	tautomerase family protein	QEN71_32950	paras_007509	protein-codi	NZ_CP1252	chromosom	722364	722570	+	207	0	1	14	1	14	
QEN71_RS32960	hypothetical protein	QEN71_32955	paras_007510	protein-codi	NZ_CP1252	chromosom	722722	722979	+	258	0	3	305	1	42	
QEN71_RS32965	AI-2E family transporter	QEN71_32960	paras_007511	protein-codi	NZ_CP1252	chromosom	723400	724503	+	1104	0	8	347	6	303	
QEN71_RS32970	efflux transporter outer membrane subunit	QEN71_32965	paras_007512	protein-codi	NZ_CP1252	chromosom	724531	726072	+	1538	0	17	1704	16	1475	
QEN71_RS32975	HlyD family secretion protein	QEN71_32970	paras_007513	protein-codi	NZ_CP1252	chromosom	726069	727223	+	1151	0	22	1583	16	1047	
QEN71_RS32980	DUF2955 domain-containing protein	QEN71_32975	paras_007514	protein-codi	NZ_CP1252	chromosom	727231	728274	+	1044	0	11	601	11	601	
QEN71_RS32985	potassium channel family protein	QEN71_32980	paras_007515	protein-codi	NZ_CP1252	chromosom	728287	728673	+	387	0	6	295	5	267	
QEN71_RS32990	hypothetical protein	QEN71_32985	paras_007516	protein-codi	NZ_CP1252	chromosom	728712	729731	+	1020	0	21	1921	15	1368	
QEN71_RS32995	hypothetical protein	QEN71_32990	paras_007517	protein-codi	NZ_CP1252	chromosom	729873	730502	+	630	0	9	349	7	320	
QEN71_RS33000	DUF2252 domain-containing protein	pseudo;QEN71_32995		pseudogene	NZ_CP1252	chromosom	730592	732020	+	1429	0	21	1860	12	1205	
QEN71_RS33005	YSC84-related protein	QEN71_33000	paras_007519	protein-codi	NZ_CP1252	chromosom	732049	732621	+	573	0	4	128	4	128	
QEN71_RS33010	hypothetical protein	QEN71_33005	paras_007520	protein-codi	NZ_CP1252	chromosom	732637	733140	+	504	0	11	813	9	368	
QEN71_RS33015	Orn/Lys/Arg decarboxylase N-terminal domain-conta	pseudo;QEN71_33010		pseudogene	NZ_CP1252	chromosom	733154	735471	-	2318	0	27	1742	25	1680	
QEN71_RS33020	putrescine-ornithine antiporter	QEN71_33015	paras_007522	protein-codi	NZ_CP1252	chromosom	735491	737740	-	2250	0	41	5291	33	4131	
QEN71_RS33025	ornithine decarboxylase	QEN71_33020	paras_007523	protein-codi	NZ_CP1252	chromosom	737773	740136	-	2364	0	57	4506	39	3058	
QEN71_RS33030	hypothetical protein	QEN71_33025	paras_007524	protein-codi	NZ_CP1252	chromosom	740886	741152	+	267	0	2	241	1	173	
QEN71_RS33035	arginine deiminase	QEN71_33030	paras_007525	protein-codi	NZ_CP1252	chromosom	741449	742702	+	1254	0	12	727	11	631	
QEN71_RS33040	ornithine carbamoyltransferase	QEN71_33035	paras_007526	protein-codi	NZ_CP1252	chromosom	742752	743762	+	1011	0	15	1918	10	1325	
QEN71_RS33045	carbamate kinase	QEN71_33040	paras_007527	protein-codi	NZ_CP1252	chromosom	743799	744737	+	939	0	9	599	6	180	
QEN71_RS33050	BamA/TamA family outer membrane protein	QEN71_33045	paras_007528	protein-codi	NZ_CP1252	chromosom	744757	745908	+	1152	0	21	2003	16	1549	
QEN71_RS33055	MipA/OmpV family protein	QEN71_33050	paras_007529	protein-codi	NZ_CP1252	chromosom	745975	746781	+	775	0	31	2758	28	2431	
QEN71_RS33060	RT0821/Lpp0805 family surface protein	QEN71_33055	paras_007530	protein-codi	NZ_CP1252	chromosom	746750	747217	+	436	0	8	515	8	515	
QEN71_RS33065	hypothetical protein	QEN71_33060	paras_007531	protein-codi	NZ_CP1252	chromosom	747322	747912	+	591	0	11	841	11	841	
QEN71_RS33070	YMGG-like glycine zipper-containing protein	QEN71_33065	paras_007532	protein-codi	NZ_CP1252	chromosom	747996	748472	+	477	0	11	1501	3	235	
QEN71_RS33075	amidohydrolase family protein	QEN71_33070	paras_007533	protein-codi	NZ_CP1252	chromosom	748536	749783	-	1248	0	19	2625	18	2614	
QEN71_RS33080	GAF domain-containing protein	QEN71_33075	paras_007534	protein-codi	NZ_CP1252	chromosom	749845	750342	-	494	0	12	1877	12	1877	

QEN71_RS33085	MFS transporter	QEN71_33080	paras_007535	protein-codi	NZ_CP1252	chromosom	750339	751703	-	1361	0	34	7642	29	7088	
QEN71_RS33090	LysR family transcriptional regulator	QEN71_33085	paras_007536	protein-codi	NZ_CP1252	chromosom	751875	752795	+	921	0	12	2541	10	2201	
QEN71_RS33095	ABC transporter substrate-binding protein	QEN71_33090	paras_007537	protein-codi	NZ_CP1252	chromosom	752826	753455	-	630	0	13	2082	11	1682	
QEN71_RS33100	DUF4922 domain-containing protein	QEN71_33095	paras_007538	protein-codi	NZ_CP1252	chromosom	753587	754495	-	909	0	11	1090	8	852	
QEN71_RS33105	adenosine-specific kinase	QEN71_33100	paras_007539	protein-codi	NZ_CP1252	chromosom	754513	754998	-	486	0	5	1697	5	1697	
QEN71_RS33110	zinc-dependent alcohol dehydrogenase family prote	QEN71_33105	paras_007540	protein-codi	NZ_CP1252	chromosom	755237	756226	+	990	0	19	4379	16	3311	
QEN71_RS33115	hypothetical protein	QEN71_33110	paras_007541	protein-codi	NZ_CP1252	chromosom	756305	756556	+	252	0	3	273	3	273	
QEN71_RS33120	DUF1269 domain-containing protein	QEN71_33115	paras_007542	protein-codi	NZ_CP1252	chromosom	756693	757202	+	510	0	4	546	2	110	
QEN71_RS33125	DMT family transporter	QEN71_33120	paras_007543	protein-codi	NZ_CP1252	chromosom	757436	758383	+	944	0	24	4808	20	4214	
QEN71_RS33130	metallophosphoesterase family protein	QEN71_33125	paras_007544	protein-codi	NZ_CP1252	chromosom	758380	759195	-	812	0	15	4006	12	3625	
QEN71_RS33135	penicillin acylase family protein	QEN71_33130	paras_007545	protein-codi	NZ_CP1252	chromosom	759221	761641	-	2421	0	58	14430	49	12727	
QEN71_RS33140	nucleoside hydrolase	QEN71_33135	paras_007546	protein-codi	NZ_CP1252	chromosom	761856	762953	+	1098	0	22	5216	17	4599	
QEN71_RS33145	alcohol dehydrogenase	QEN71_33140	paras_007547	protein-codi	NZ_CP1252	chromosom	763112	764125	+	1014	0	14	4818	6	2986	
QEN71_RS33150	DUF3348 domain-containing protein	QEN71_33145	paras_007548	protein-codi	NZ_CP1252	chromosom	764306	765055	+	750	0	8	1738	4	1210	
QEN71_RS33155	DUF802 domain-containing protein	QEN71_33150	paras_007549	protein-codi	NZ_CP1252	chromosom	765066	767501	+	2432	0	26	4044	8	1320	
QEN71_RS33160	OmpA family protein	QEN71_33155	paras_007550	protein-codi	NZ_CP1252	chromosom	767498	768145	+	636	0	5	425	5	425	
QEN71_RS33165	DUF2894 domain-containing protein	QEN71_33160	paras_007551	protein-codi	NZ_CP1252	chromosom	768138	768764	+	619	0	10	899	9	870	
QEN71_RS33170	elongation factor G	QEN71_33165	paras_007552	protein-codi	NZ_CP1252	chromosom	768768	770813	-	2046	0	38	6293	26	4356	
QEN71_RS33175	DsbA family oxidoreductase	QEN71_33170	paras_007553	protein-codi	NZ_CP1252	chromosom	771000	771653	+	654	0	25	4172	21	3880	
QEN71_RS33180	cytochrome b/b6 domain-containing protein	QEN71_33175	paras_007554	protein-codi	NZ_CP1252	chromosom	771806	772399	+	594	0	9	1080	9	1080	
QEN71_RS33185	molybdopterin-dependent oxidoreductase	QEN71_33180	paras_007555	protein-codi	NZ_CP1252	chromosom	772403	773194	+	792	0	26	5227	24	4799	
QEN71_RS33190	hypothetical protein	QEN71_33185	paras_007556	protein-codi	NZ_CP1252	chromosom	773249	773866	+	618	0	11	1907	7	715	
QEN71_RS33195	acyltransferase	QEN71_33190	paras_007557	protein-codi	NZ_CP1252	chromosom	773982	775130	+	1149	0	54	11847	43	9304	
QEN71_RS33200	SMP-30/gluconolactonase/LRE family protein	QEN71_33195	paras_007558	protein-codi	NZ_CP1252	chromosom	775131	777053	-	1923	0	74	15295	51	10068	
QEN71_RS33205	zinc ribbon domain-containing protein	QEN71_33200	paras_007559	protein-codi	NZ_CP1252	chromosom	777344	777508	+	165	0	3	763	0	0	
QEN71_RS33210	hypothetical protein	QEN71_33205	paras_007560	protein-codi	NZ_CP1252	chromosom	777598	777852	+	251	0	15	1602	14	1525	
QEN71_RS33215	MFS transporter	QEN71_33210	paras_007561	protein-codi	NZ_CP1252	chromosom	777849	779192	-	1340	0	21	3170	17	2993	
QEN71_RS33220	enoyl-CoA hydratase	QEN71_33215	paras_007562	protein-codi	NZ_CP1252	chromosom	779226	780002	-	773	0	11	1304	9	1001	
QEN71_RS33225	CoA transferase	QEN71_33220	paras_007563	protein-codi	NZ_CP1252	chromosom	779999	781201	-	1199	0	10	1025	9	708	
QEN71_RS33230	LysR family transcriptional regulator	QEN71_33225	paras_007564	protein-codi	NZ_CP1252	chromosom	781464	782354	-	891	0	16	2675	11	1759	
QEN71_RS33235	sensor domain-containing diguanylate cyclase	QEN71_33230	paras_007565	protein-codi	NZ_CP1252	chromosom	782614	784131	+	1518	0	24	4266	17	3812	
QEN71_RS33240	RidA family protein	QEN71_33235	paras_007566	protein-codi	NZ_CP1252	chromosom	784170	784523	-	354	0	3	694	1	141	
QEN71_RS33245	FAD-binding oxidoreductase	QEN71_33240	paras_007567	protein-codi	NZ_CP1252	chromosom	784557	785741	-	1177	0	10	740	10	740	
QEN71_RS33250	FAD-dependent oxidoreductase	QEN71_33245	paras_007568	protein-codi	NZ_CP1252	chromosom	785734	787173	-	1421	0	20	3994	15	3203	
QEN71_RS33255	(2Fe-2S)-binding protein	QEN71_33250	paras_007569	protein-codi	NZ_CP1252	chromosom	787163	787504	-	331	0	4	260	2	37	
QEN71_RS33260	FAD-dependent oxidoreductase	QEN71_33255	paras_007570	protein-codi	NZ_CP1252	chromosom	787512	788648	-	1133	0	15	1627	15	1627	
QEN71_RS33265	ABC transporter permease	QEN71_33260	paras_007571	protein-codi	NZ_CP1252	chromosom	788645	789442	-	794	0	4	633	2	566	
QEN71_RS33270	ABC transporter permease	QEN71_33265	paras_007572	protein-codi	NZ_CP1252	chromosom	789450	790322	-	859	0	14	1984	14	1984	
QEN71_RS33275	ABC transporter ATP-binding protein	QEN71_33270	paras_007573	protein-codi	NZ_CP1252	chromosom	790309	791379	-	1057	0	8	939	7	547	
QEN71_RS33280	ABC transporter substrate-binding protein	QEN71_33275	paras_007574	protein-codi	NZ_CP1252	chromosom	791433	792497	-	1065	0	24	2579	16	1918	
QEN71_RS33285	LysR substrate-binding domain-containing protein	QEN71_33280	paras_007575	protein-codi	NZ_CP1252	chromosom	792632	793594	+	963	0	14	2302	12	2181	
QEN71_RS33290	pyrimidine/purine nucleoside phosphorylase	QEN71_33285	paras_007576	protein-codi	NZ_CP1252	chromosom	793657	793977	-	321	0	10	2022	4	619	
QEN71_RS33295	MFS transporter	QEN71_33290	paras_007577	protein-codi	NZ_CP1252	chromosom	794331	795716	+	1386	0	40	6670	27	4862	
QEN71_RS33300	shikimate dehydrogenase	QEN71_33295	paras_007578	protein-codi	NZ_CP1252	chromosom	795752	796585	+	834	0	9	449	5	277	
QEN71_RS33305	Ohr family peroxiredoxin	QEN71_33300	paras_007579	protein-codi	NZ_CP1252	chromosom	796635	797135	-	501	0	4	55	3	50	
QEN71_RS33310	LysR family transcriptional regulator	QEN71_33305	paras_007580	protein-codi	NZ_CP1252	chromosom	797413	798441	+	1029	0	11	916	8	787	
QEN71_RS33315	TetR/AcrR family transcriptional regulator	QEN71_33310	paras_007581	protein-codi	NZ_CP1252	chromosom	798749	799360	+	612	0	7	485	5	350	
QEN71_RS33320	VOC family protein	QEN71_33315	paras_007582	protein-codi	NZ_CP1252	chromosom	799541	799948	+	408	0	10	584	7	377	
QEN71_RS33325	O-methyltransferase	partial;pseudo;QEN71_33320		pseudogene	NZ_CP1252	chromosom	800128	800304	+	177	0	1	95	1	95	
QEN71_RS33330	hypothetical protein	QEN71_33325	paras_007584	protein-codi	NZ_CP1252	chromosom	800519	800695	+	177	0	3	140	0	0	
QEN71_RS33335	hypothetical protein	QEN71_33330	paras_007585	protein-codi	NZ_CP1252	chromosom	800830	801882	+	1053	0	18	2490	15	2204	
QEN71_RS33340	ester cyclase	QEN71_33335	paras_007586	protein-codi	NZ_CP1252	chromosom	802070	802507	-	438	0	10	1400	5	940	
QEN71_RS33345	hypothetical protein	QEN71_33340	paras_007587	protein-codi	NZ_CP1252	chromosom	802546	802737	-	192	0	3	570	3	570	
QEN71_RS33350	RidA family protein	QEN71_33345	paras_007588	protein-codi	NZ_CP1252	chromosom	802814	803224	-	411	0	3	592	3	592	
QEN71_RS33355	PhzF family phenazine biosynthesis protein	QEN71_33350	paras_007589	protein-codi	NZ_CP1252	chromosom	803336	804136	+	797	0	18	3204	13	2260	
QEN71_RS33360	sigma factor	QEN71_33355	paras_007590	protein-codi	NZ_CP1252	chromosom	804133	805404	-	1264	0	22	4225	17	3811	
QEN71_RS33365	YciI family protein	QEN71_33360	paras_007591	protein-codi	NZ_CP1252	chromosom	805401	805772	-	368	0	18	3993	9	1639	
QEN71_RS33370	XRE family transcriptional regulator	QEN71_33365	paras_007592	protein-codi	NZ_CP1252	chromosom	805895	806464	-	570	0	14	3180	8	1957	
QEN71_RS33375	GNAT family N-acetyltransferase	QEN71_33370	paras_007593	protein-codi	NZ_CP1252	chromosom	806512	807036	+	525	0	13	4787	7	1807	
QEN71_RS33380	hypothetical protein	QEN71_33375	paras_007594	protein-codi	NZ_CP1252	chromosom	807088	807384	-	297	0	7	706	4	337	
QEN71_RS33385	hypothetical protein	QEN71_33380	paras_007595	protein-codi	NZ_CP1252	chromosom	807509	808561	-	1053	0	9	552	8	485	
QEN71_RS33390	3-oxoacyl-ACP reductase family protein	QEN71_33385	paras_007596	protein-codi	NZ_CP1252	chromosom	808809	809555	-	747	0	9	2248	5	2070	

QEN71_RS33395	FdhF/YdeP family oxidoreductase	QEN71_33390	paras_007597	protein-codi	NZ_CP1252	chromosom	809788	812091	+	2304	0	45	9628	36	7796	
QEN71_RS33400	helix-turn-helix domain-containing protein	QEN71_33395	paras_007598	protein-codi	NZ_CP1252	chromosom	812126	812767	-	628	0	6	1782	1	23	
QEN71_RS33405	hypothetical protein	QEN71_33400	paras_007599	protein-codi	NZ_CP1252	chromosom	812754	813338	-	571	0	10	6911	10	6911	
QEN71_RS33410	CheR family methyltransferase	QEN71_33405	paras_007600	protein-codi	NZ_CP1252	chromosom	813452	817528	-	4077	0	53	8199	44	6601	
QEN71_RS33415	chemotaxis protein CheB	QEN71_33410	paras_007601	protein-codi	NZ_CP1252	chromosom	817616	818608	-	993	0	19	3588	11	2409	
QEN71_RS33420	SDR family NAD(P)-dependent oxidoreductase	QEN71_33415	paras_007602	protein-codi	NZ_CP1252	chromosom	818705	819490	-	786	0	17	5156	12	3417	
QEN71_RS33425	hypothetical protein	QEN71_33420	paras_007603	protein-codi	NZ_CP1252	chromosom	819532	819702	-	171	0	4	1652	4	1652	
QEN71_RS33430	hypothetical protein	QEN71_33425	paras_007604	protein-codi	NZ_CP1252	chromosom	819790	820032	-	243	0	5	750	1	87	
QEN71_RS33435	hypothetical protein	QEN71_33430	paras_007605	protein-codi	NZ_CP1252	chromosom	820333	820635	+	303	0	2	37	1	22	
QEN71_RS33440	glycosyltransferase	QEN71_33435	paras_007606	protein-codi	NZ_CP1252	chromosom	820767	821063	-	297	0	12	1952	10	1698	
QEN71_RS33445	DUF2795 domain-containing protein	QEN71_33440	paras_007607	protein-codi	NZ_CP1252	chromosom	821081	821314	-	234	0	7	1864	7	1864	
QEN71_RS33450	DUF4142 domain-containing protein	QEN71_33445	paras_007608	protein-codi	NZ_CP1252	chromosom	821425	821970	-	546	0	16	7924	6	5903	
QEN71_RS33455	SDR family NAD(P)-dependent oxidoreductase	QEN71_33450	paras_007609	protein-codi	NZ_CP1252	chromosom	822042	822809	-	768	0	6	3349	6	3349	
QEN71_RS33460	hypothetical protein	QEN71_33455	paras_007610	protein-codi	NZ_CP1252	chromosom	822962	823123	-	162	0	5	1733	5	1733	
QEN71_RS33465	sensor domain-containing diguanylate cyclase	QEN71_33460	paras_007611	protein-codi	NZ_CP1252	chromosom	823520	824656	+	1137	0	24	8933	14	6160	
QEN71_RS33470	MFS transporter	QEN71_33465	paras_007612	protein-codi	NZ_CP1252	chromosom	824738	826012	+	1275	0	32	4337	19	2551	
QEN71_RS33475	NO-inducible flavohemoprotein	QEN71_33470	paras_007613	protein-codi	NZ_CP1252	chromosom	826051	827265	-	1215	0	50	8765	32	5666	
QEN71_RS33480	high-potential iron-sulfur protein	QEN71_33475	paras_007614	protein-codi	NZ_CP1252	chromosom	827460	827771	-	312	0	7	1072	4	392	
QEN71_RS33485	GNAT family N-acetyltransferase	QEN71_33480	paras_007615	protein-codi	NZ_CP1252	chromosom	828076	828531	+	456	0	3	289	3	289	
QEN71_RS33490	LysR substrate-binding domain-containing protein	QEN71_33485	paras_007616	protein-codi	NZ_CP1252	chromosom	828543	829499	-	957	0	16	954	13	904	
QEN71_RS33495	mandelate racemase/muconate lactonizing enzyme	QEN71_33490	paras_007617	protein-codi	NZ_CP1252	chromosom	829632	830798	+	1167	0	26	1791	20	1556	
QEN71_RS33500	C4-dicarboxylate transporter DctA	QEN71_33495	paras_007618	protein-codi	NZ_CP1252	chromosom	830955	832298	+	1344	0	22	2659	18	1820	
QEN71_RS33505	methyl-accepting chemotaxis protein	QEN71_33500	paras_007619	protein-codi	NZ_CP1252	chromosom	832394	833941	+	1548	0	12	985	6	275	
QEN71_RS33510	hypothetical protein	QEN71_33505	paras_007620	protein-codi	NZ_CP1252	chromosom	833954	834217	-	264	0	4	266	2	139	
QEN71_RS33515	MFS transporter	QEN71_33510	paras_007621	protein-codi	NZ_CP1252	chromosom	834299	835528	-	1230	0	10	1031	8	764	
QEN71_RS33520	alkene reductase	QEN71_33515	paras_007622	protein-codi	NZ_CP1252	chromosom	835644	836753	-	1110	0	25	2298	20	2153	
QEN71_RS33525	zinc-dependent alcohol dehydrogenase family prote	QEN71_33520	paras_007623	protein-codi	NZ_CP1252	chromosom	837067	838056	+	990	0	17	1481	13	1020	
QEN71_RS33530	flavin reductase family protein	QEN71_33525	paras_007624	protein-codi	NZ_CP1252	chromosom	838088	838720	+	633	0	13	1851	10	1491	
QEN71_RS33535	Gfo/Idh/MocA family oxidoreductase	QEN71_33530	paras_007625	protein-codi	NZ_CP1252	chromosom	838757	839875	+	1119	0	18	926	16	891	
QEN71_RS33540	carboxymuconolactone decarboxylase family protei	QEN71_33535	paras_007626	protein-codi	NZ_CP1252	chromosom	839972	840529	+	558	0	6	323	4	172	
QEN71_RS33545	helix-turn-helix transcriptional regulator	QEN71_33540	paras_007627	protein-codi	NZ_CP1252	chromosom	840575	841402	+	828	0	11	1032	7	783	
QEN71_RS33550	MarR family transcriptional regulator	QEN71_33545	paras_007628	protein-codi	NZ_CP1252	chromosom	841434	841865	-	432	0	7	703	7	703	
QEN71_RS33555	FAD-binding monooxygenase	QEN71_33550	paras_007629	protein-codi	NZ_CP1252	chromosom	841885	843777	-	1893	0	31	2603	24	1503	
QEN71_RS33560	PA14 domain-containing protein	QEN71_33555	paras_007630	protein-codi	NZ_CP1252	chromosom	843990	846275	-	2286	0	56	8475	50	7454	
QEN71_RS33565	MFS transporter	QEN71_33560	paras_007631	protein-codi	NZ_CP1252	chromosom	846572	847930	-	1359	0	38	7599	29	6821	
QEN71_RS33570	5-dehydro-4-deoxyglucarate dehydratase	QEN71_33565	paras_007632	protein-codi	NZ_CP1252	chromosom	848015	848932	-	918	0	27	4197	25	4089	
QEN71_RS33575	galactarate dehydratase	QEN71_33570	paras_007633	protein-codi	NZ_CP1252	chromosom	849132	850721	+	1590	0	21	5409	18	4857	
QEN71_RS33580	beta-propeller fold lactonase family protein	QEN71_33575	paras_007634	protein-codi	NZ_CP1252	chromosom	850789	851901	+	1113	0	29	5083	21	2550	
QEN71_RS33585	hypothetical protein	QEN71_33580	paras_007635	protein-codi	NZ_CP1252	chromosom	852040	852240	+	201	0	9	1487	5	788	
QEN71_RS33590	GlxA family transcriptional regulator	QEN71_33585	paras_007636	protein-codi	NZ_CP1252	chromosom	852308	853321	+	1014	0	24	4847	18	3801	
QEN71_RS33595	M24 family metallopeptidase	QEN71_33590	paras_007637	protein-codi	NZ_CP1252	chromosom	853484	854695	+	1212	0	26	1960	23	1851	
QEN71_RS33600	serine hydroxymethyltransferase	QEN71_33595	paras_007638	protein-codi	NZ_CP1252	chromosom	854762	856021	+	1260	0	19	2188	17	1933	
QEN71_RS33605	creatininase	QEN71_33600	paras_007639	protein-codi	NZ_CP1252	chromosom	856085	856879	+	795	0	13	620	10	556	
QEN71_RS33610	cytosine permease	QEN71_33605	paras_007640	protein-codi	NZ_CP1252	chromosom	856896	858371	+	1476	0	19	1290	18	1285	
QEN71_RS33615	MFS transporter	QEN71_33610	paras_007641	protein-codi	NZ_CP1252	chromosom	858604	859971	+	1368	0	20	1257	16	1200	
QEN71_RS33620	GlxA family transcriptional regulator	QEN71_33615	paras_007642	protein-codi	NZ_CP1252	chromosom	860068	861120	+	1053	0	3	149	2	137	
QEN71_RS33625	hypothetical protein	QEN71_33620	paras_007643	protein-codi	NZ_CP1252	chromosom	861429	861971	+	543	0	7	340	7	340	
QEN71_RS33630	gallate dioxygenase	QEN71_33625	paras_007644	protein-codi	NZ_CP1252	chromosom	862030	863343	+	1314	0	20	2124	20	2124	
QEN71_RS33635	aromatic acid/H+ symport family MFS transporter	QEN71_33630	paras_007645	protein-codi	NZ_CP1252	chromosom	863438	864787	+	1350	0	12	1698	9	1323	
QEN71_RS33640	amidohydrolase family protein	QEN71_33635	paras_007646	protein-codi	NZ_CP1252	chromosom	864898	865923	+	1026	390.0	18	1354	11	564	
QEN71_RS33645	4-carboxy-4-hydroxy-2-oxoadipate aldolase/oxaloac	QEN71_33640	paras_007647	protein-codi	NZ_CP1252	chromosom	865926	866609	+	684	402.0	10	1565	5	274	
QEN71_RS33650	substrate-binding domain-containing protein	QEN71_33645	paras_007648	protein-codi	NZ_CP1252	chromosom	866620	867327	+	708	0	6	416	6	416	
QEN71_RS33655	hypothetical protein	QEN71_33650	paras_007649	protein-codi	NZ_CP1252	chromosom	867513	868595	+	1083	0	21	1539	17	1285	
QEN71_RS33660	KUP/HAK/KT family potassium transporter	QEN71_33655	paras_007650	protein-codi	NZ_CP1252	chromosom	868618	870534	+	1862	0	26	2541	19	1815	
QEN71_RS33665	EAL domain-containing protein	QEN71_33660	paras_007651	protein-codi	NZ_CP1252	chromosom	870480	873278	-	2744	0	36	5747	32	5481	
QEN71_RS33670	excinuclease ABC subunit UvrA	QEN71_33665	paras_007652	protein-codi	NZ_CP1252	chromosom	873612	876275	+	2664	0	49	7388	43	6853	
QEN71_RS33675	amino acid ABC transporter permease	QEN71_33670	paras_007653	protein-codi	NZ_CP1252	chromosom	876307	877089	-	783	0	15	2029	13	1943	
QEN71_RS33680	basic amino acid ABC transporter substrate-binding	QEN71_33675	paras_007654	protein-codi	NZ_CP1252	chromosom	877179	877940	-	762	0	15	962	10	533	
QEN71_RS33685	UbiD family decarboxylase	QEN71_33680	paras_007655	protein-codi	NZ_CP1252	chromosom	878190	879683	+	1444	0	20	1430	16	1324	
QEN71_RS33690	acyl-CoA dehydrogenase family protein	QEN71_33685	paras_007656	protein-codi	NZ_CP1252	chromosom	879634	881028	-	1345	0	16	1147	14	1130	
QEN71_RS33695	acyl-CoA dehydrogenase family protein	QEN71_33690	paras_007657	protein-codi	NZ_CP1252	chromosom	881101	882396	-	1296	0	27	1473	22	1170	
QEN71_RS33700	ArsR family transcriptional regulator	QEN71_33695	paras_007658	protein-codi	NZ_CP1252	chromosom	882584	883225	-	642	0	11	884	8	655	

QEN71_RS33705	cystathionine gamma-synthase family protein	QEN71_33700	paras_007659	protein-codi	NZ_CP1252	chromosom	883424	884665	+	1242	0	22	2709	13	1385	
QEN71_RS33710	FAD-dependent monooxygenase	QEN71_33705	paras_007660	protein-codi	NZ_CP1252	chromosom	884724	885947	-	1224	0	13	597	12	468	
QEN71_RS33715	LysR family transcriptional regulator	QEN71_33710	paras_007661	protein-codi	NZ_CP1252	chromosom	886112	887053	+	942	0	7	291	7	291	
QEN71_RS33720	hypothetical protein	QEN71_33715	paras_007662	protein-codi	NZ_CP1252	chromosom	887507	887770	+	264	0	1	22	0	0	
QEN71_RS33725	N-acetyl-gamma-glutamyl-phosphate reductase	QEN71_33720	paras_007663	protein-codi	NZ_CP1252	chromosom	887863	888786	-	924	0	5	460	5	460	
QEN71_RS33730	LysR family transcriptional regulator	QEN71_33725	paras_007664	protein-codi	NZ_CP1252	chromosom	888916	889800	+	885	0	11	439	7	130	
QEN71_RS33735	SDR family oxidoreductase	QEN71_33730	paras_007665	protein-codi	NZ_CP1252	chromosom	889835	890560	-	726	0	2	318	2	318	
QEN71_RS33740	LysR family transcriptional regulator	QEN71_33735	paras_007666	protein-codi	NZ_CP1252	chromosom	890681	891613	+	933	0	6	508	4	279	
QEN71_RS33745	isochorismatase family protein	QEN71_33740	paras_007667	protein-codi	NZ_CP1252	chromosom	891862	892515	+	654	0	8	465	5	277	
QEN71_RS33750	FAD-dependent oxidoreductase	QEN71_33745	paras_007668	protein-codi	NZ_CP1252	chromosom	892558	893928	+	1371	0	6	388	5	386	
QEN71_RS33755	glutamine ABC transporter substrate-binding protei	QEN71_33750	paras_007669	protein-codi	NZ_CP1252	chromosom	894178	894924	+	747	0	18	2165	14	1200	
QEN71_RS33760	glutamine ABC transporter permease GlnP	QEN71_33755	paras_007670	protein-codi	NZ_CP1252	chromosom	894951	895607	+	653	0	10	1305	9	1090	
QEN71_RS33765	glutamine ABC transporter ATP-binding protein Gln	QEN71_33760	paras_007671	protein-codi	NZ_CP1252	chromosom	895604	896341	+	734	0	14	1216	11	834	
QEN71_RS33770	C45 family peptidase	QEN71_33765	paras_007672	protein-codi	NZ_CP1252	chromosom	896352	897437	+	1021	0	20	1797	11	942	
QEN71_RS33775	MurR/RpiR family transcriptional regulator	QEN71_33770	paras_007673	protein-codi	NZ_CP1252	chromosom	897373	898371	+	934	0	18	1674	18	1674	
QEN71_RS33780	hypothetical protein	QEN71_33775	paras_007674	protein-codi	NZ_CP1252	chromosom	898552	898953	+	402	0	13	562	11	504	
QEN71_RS33785	ATP-binding cassette domain-containing protein	QEN71_33780	paras_007675	protein-codi	NZ_CP1252	chromosom	898966	899739	-	773	0	12	1239	8	862	
QEN71_RS33790	ABC transporter permease	QEN71_33785	paras_007676	protein-codi	NZ_CP1252	chromosom	899739	900737	-	994	0	8	855	8	855	
QEN71_RS33795	sugar ABC transporter substrate-binding protein	QEN71_33790	paras_007677	protein-codi	NZ_CP1252	chromosom	900734	901813	-	1076	0	26	2684	20	2177	
QEN71_RS33800	asparaginase	QEN71_33795	paras_007678	protein-codi	NZ_CP1252	chromosom	902304	903422	+	1119	0	17	1064	14	873	
QEN71_RS33805	LysR substrate-binding domain-containing protein	QEN71_33800	paras_007679	protein-codi	NZ_CP1252	chromosom	903588	904502	+	915	0	19	2500	17	2320	
QEN71_RS33810	dicarboxylate/amino acid:cation symporter	QEN71_33805	paras_007680	protein-codi	NZ_CP1252	chromosom	904684	905964	+	1277	0	16	1622	12	1328	
QEN71_RS33815	amino acid racemase	QEN71_33810	paras_007681	protein-codi	NZ_CP1252	chromosom	905961	907460	+	1496	0	23	1359	21	1101	
QEN71_RS33820	aspartate ammonia-lyase	QEN71_33815	paras_007682	protein-codi	NZ_CP1252	chromosom	907533	908981	+	1449	0	15	1054	11	723	
QEN71_RS33825	IclR family transcriptional regulator	QEN71_33820	paras_007683	protein-codi	NZ_CP1252	chromosom	908991	909752	-	762	0	13	717	11	519	
QEN71_RS33830	amino acid ABC transporter ATP-binding protein	QEN71_33825	paras_007684	protein-codi	NZ_CP1252	chromosom	909841	910593	-	749	0	3	32	3	32	
QEN71_RS33835	amino acid ABC transporter permease	QEN71_33830	paras_007685	protein-codi	NZ_CP1252	chromosom	910590	911237	-	644	0	9	618	8	551	
QEN71_RS33840	amino acid ABC transporter permease	QEN71_33835	paras_007686	protein-codi	NZ_CP1252	chromosom	911260	911979	-	720	0	17	1332	13	1304	
QEN71_RS33845	transporter substrate-binding domain-containing pro	QEN71_33840	paras_007687	protein-codi	NZ_CP1252	chromosom	912029	912862	-	834	0	17	1042	13	675	
QEN71_RS33850	alcohol dehydrogenase catalytic domain-containing	QEN71_33845	paras_007688	protein-codi	NZ_CP1252	chromosom	913135	914223	-	1089	0	13	1207	13	1207	
QEN71_RS33855	NAD(P)-dependent alcohol dehydrogenase	QEN71_33850	paras_007689	protein-codi	NZ_CP1252	chromosom	914309	915349	-	1041	0	20	1896	15	1489	
QEN71_RS33860	SDR family oxidoreductase	QEN71_33855	paras_007690	protein-codi	NZ_CP1252	chromosom	915432	916178	-	747	0	6	363	5	166	
QEN71_RS33865	sugar-binding transcriptional regulator	QEN71_33860	paras_007691	protein-codi	NZ_CP1252	chromosom	916187	917167	-	981	0	14	661	8	366	
QEN71_RS33870	carbohydrate ABC transporter permease	QEN71_33865	paras_007692	protein-codi	NZ_CP1252	chromosom	917217	918113	-	893	0	12	681	10	303	
QEN71_RS33875	sugar ABC transporter permease	QEN71_33870	paras_007693	protein-codi	NZ_CP1252	chromosom	918110	919045	-	932	0	13	1246	11	871	
QEN71_RS33880	sugar ABC transporter substrate-binding protein	QEN71_33875	paras_007694	protein-codi	NZ_CP1252	chromosom	919071	920423	-	1353	0	22	1125	14	680	
QEN71_RS33885	sn-glycerol-3-phosphate ABC transporter ATP-bindi	QEN71_33880	paras_007695	protein-codi	NZ_CP1252	chromosom	920696	921820	+	1125	0	15	1259	13	1174	
QEN71_RS33890	hypothetical protein	QEN71_33885	paras_007696	protein-codi	NZ_CP1252	chromosom	921835	922584	-	750	0	14	885	11	705	
QEN71_RS33895	hypothetical protein	QEN71_33890	paras_007697	protein-codi	NZ_CP1252	chromosom	922647	922856	-	210	0	2	35	2	35	
QEN71_RS33900	hypothetical protein	QEN71_33895	paras_007698	protein-codi	NZ_CP1252	chromosom	923106	923363	-	258	0	2	213	2	213	
QEN71_RS33905	hypothetical protein	QEN71_33900	paras_007699	protein-codi	NZ_CP1252	chromosom	923690	924118	+	429	0	10	1267	10	1267	
QEN71_RS33910	hypothetical protein	QEN71_33905	paras_007700	protein-codi	NZ_CP1252	chromosom	924176	924421	+	246	0	0	0	0	0	
QEN71_RS33915	two-component system response regulator NarL	QEN71_33910	paras_007701	protein-codi	NZ_CP1252	chromosom	924505	925164	+	660	0	7	602	6	576	
QEN71_RS33920	XRE family transcriptional regulator	QEN71_33915	paras_007702	protein-codi	NZ_CP1252	chromosom	925287	925928	+	642	0	14	2215	10	1941	
QEN71_RS33925	formyltetrahydrofolate deformylase	QEN71_33920	paras_007703	protein-codi	NZ_CP1252	chromosom	926255	927118	+	864	0	16	1164	10	889	
QEN71_RS33930	FAD-dependent oxidoreductase	QEN71_33925	paras_007704	protein-codi	NZ_CP1252	chromosom	927245	928432	+	1188	0	24	2147	19	1772	
QEN71_RS33935	sarcosine oxidase subunit delta	QEN71_33930	paras_007705	protein-codi	NZ_CP1252	chromosom	928447	928704	+	258	0	3	369	3	369	
QEN71_RS33940	glycine cleavage T C-terminal barrel domain-contai	QEN71_33935	paras_007706	protein-codi	NZ_CP1252	chromosom	928705	931671	+	2963	0	44	3133	31	1632	
QEN71_RS33945	hypothetical protein	QEN71_33940	paras_007707	protein-codi	NZ_CP1252	chromosom	931668	932327	+	656	0	9	1144	9	1144	
QEN71_RS33950	type III glutamate--ammonia ligase	QEN71_33945	paras_007708	protein-codi	NZ_CP1252	chromosom	932423	933751	+	1329	0	29	2183	23	1526	
QEN71_RS33955	amidophosphoribosyltransferase	QEN71_33950	paras_007709	protein-codi	NZ_CP1252	chromosom	933801	934709	+	896	0	9	2157	6	1004	
QEN71_RS33960	protein glxC	QEN71_33955	paras_007710	protein-codi	NZ_CP1252	chromosom	934697	935395	+	686	0	14	1665	10	1240	
QEN71_RS33965	FMN-binding glutamate synthase family protein	QEN71_33960	paras_007711	protein-codi	NZ_CP1252	chromosom	935410	936777	+	1364	0	17	2239	11	1644	
QEN71_RS33970	bifunctional methylenetetrahydrofolate dehydrogena	QEN71_33965	paras_007712	protein-codi	NZ_CP1252	chromosom	936774	937673	+	896	0	9	1063	7	850	
QEN71_RS33975	aspartate aminotransferase family protein	QEN71_33970	paras_007713	protein-codi	NZ_CP1252	chromosom	937701	939152	+	1452	0	13	1846	8	1069	
QEN71_RS33980	CoA ester lyase	QEN71_33975	paras_007714	protein-codi	NZ_CP1252	chromosom	939257	940234	-	978	0	19	1717	14	1410	
QEN71_RS33985	phosphoenolpyruvate carboxylase	QEN71_33980	paras_007715	protein-codi	NZ_CP1252	chromosom	940280	943192	-	2890	0	27	3294	22	2910	
QEN71_RS33990	succinate--CoA ligase subunit alpha	QEN71_33985	paras_007716	protein-codi	NZ_CP1252	chromosom	943170	944075	-	883	0	10	1036	6	456	
QEN71_RS33995	malate--CoA ligase subunit beta	QEN71_33990	paras_007717	protein-codi	NZ_CP1252	chromosom	944087	945274	-	1188	0	16	1832	12	1660	
QEN71_RS34000	D-2-hydroxyacid dehydrogenase	QEN71_33995	paras_007718	protein-codi	NZ_CP1252	chromosom	945322	946278	-	957	0	4	468	3	448	
QEN71_RS34005	aminotransferase class V-fold PLP-dependent enzy	QEN71_34000	paras_007719	protein-codi	NZ_CP1252	chromosom	946345	947610	-	1266	0	8	696	7	694	
QEN71_RS34010	serine hydroxymethyltransferase	QEN71_34005	paras_007720	protein-codi	NZ_CP1252	chromosom	947670	948992	-	1323	0	15	627	13	613	

QEN71_RS34015	LysR family transcriptional regulator	QEN71_34010	paras_007721	protein-codi	NZ_CP1252	chromosom	949260	950183	+	920	0	4	478	4	478	
QEN71_RS34020	formylmethanofuran dehydrogenase subunit C	QEN71_34015	paras_007722	protein-codi	NZ_CP1252	chromosom	950180	951004	-	817	0	9	571	6	339	
QEN71_RS34025	formylmethanofuran--tetrahydromethanopterin N-for	QEN71_34020	paras_007723	protein-codi	NZ_CP1252	chromosom	951001	951966	-	958	0	6	544	3	296	
QEN71_RS34030	formylmethanofuran dehydrogenase subunit A	QEN71_34025	paras_007724	protein-codi	NZ_CP1252	chromosom	951963	953654	-	1684	0	23	1856	18	1329	
QEN71_RS34035	formylmethanofuran dehydrogenase	QEN71_34030	paras_007725	protein-codi	NZ_CP1252	chromosom	953651	954979	-	1325	0	11	657	7	464	
QEN71_RS34040	ATP-grasp domain-containing protein	QEN71_34035	paras_007726	protein-codi	NZ_CP1252	chromosom	955178	956224	+	1036	0	15	623	10	539	
QEN71_RS34045	hydantoinase/oxoprolinase family protein	QEN71_34040	paras_007727	protein-codi	NZ_CP1252	chromosom	956214	957290	+	1066	0	6	450	6	450	
QEN71_RS34050	aspartate kinase	QEN71_34045	paras_007728	protein-codi	NZ_CP1252	chromosom	957321	957911	+	572	0	6	649	5	568	
QEN71_RS34055	(5-formylfuran-3-yl)methyl phosphate synthase	QEN71_34050	paras_007729	protein-codi	NZ_CP1252	chromosom	957893	958660	-	745	0	3	256	2	244	TRUE
QEN71_RS34060	DUF447 family protein	QEN71_34055	paras_007730	protein-codi	NZ_CP1252	chromosom	958657	959250	-	590	0	6	838	4	753	
QEN71_RS34065	DUF6513 domain-containing protein	QEN71_34060	paras_007731	protein-codi	NZ_CP1252	chromosom	959253	960638	-	1385	0	11	1151	10	1109	
QEN71_RS34070	flavoprotein	QEN71_34065	paras_007732	protein-codi	NZ_CP1252	chromosom	960638	961243	-	605	0	8	535	5	244	
QEN71_RS34075	dihydroneopterin aldolase	QEN71_34070	paras_007733	protein-codi	NZ_CP1252	chromosom	961457	961870	+	414	0	4	232	4	232	
QEN71_RS34080	triphosphoribosyl-dephospho-CoA synthase	QEN71_34075	paras_007734	protein-codi	NZ_CP1252	chromosom	961890	962819	-	929	0	11	1006	11	1006	
QEN71_RS34085	RimK family alpha-L-glutamate ligase	QEN71_34080	paras_007735	protein-codi	NZ_CP1252	chromosom	962819	963772	-	953	0	8	381	6	289	
QEN71_RS34090	methenyltetrahydromethanopterin cyclohydrolase	QEN71_34085	paras_007736	protein-codi	NZ_CP1252	chromosom	963774	964802	-	1003	0	4	478	4	478	
QEN71_RS34095	ATP-grasp domain-containing protein	QEN71_34090	paras_007737	protein-codi	NZ_CP1252	chromosom	964777	965961	-	1159	0	8	326	8	326	
QEN71_RS34100	methylenetetrahydromethanopterin dehydrogenase	QEN71_34095	paras_007738	protein-codi	NZ_CP1252	chromosom	965969	966889	-	921	0	8	664	5	371	
QEN71_RS34105	beta-ribofuranosylaminobenzene 5'-phosphate synt	QEN71_34100	paras_007739	protein-codi	NZ_CP1252	chromosom	966913	967932	-	1020	0	12	1915	11	1793	
QEN71_RS34110	LysR substrate-binding domain-containing protein	QEN71_34105	paras_007740	protein-codi	NZ_CP1252	chromosom	968126	969097	-	972	0	5	357	5	357	
QEN71_RS34115	class 1 fructose-bisphosphatase	QEN71_34110	paras_007741	protein-codi	NZ_CP1252	chromosom	969231	970373	+	1143	0	17	1295	16	1289	
QEN71_RS34120	phosphoribulokinase	QEN71_34115	paras_007742	protein-codi	NZ_CP1252	chromosom	970388	971260	+	873	0	5	350	4	339	
QEN71_RS34125	phosphoglycolate phosphatase	QEN71_34120	paras_007743	protein-codi	NZ_CP1252	chromosom	971308	972075	+	768	0	5	153	5	153	
QEN71_RS34130	sigma-54-dependent Fis family transcriptional regul	QEN71_34125	paras_007744	protein-codi	NZ_CP1252	chromosom	972093	974093	-	2001	103.0	14	1183	12	876	
QEN71_RS34135	two-component system response regulator NarL	QEN71_34130	paras_007745	protein-codi	NZ_CP1252	chromosom	974330	974977	-	648	0	5	660	3	343	
QEN71_RS34140	electron transfer flavoprotein subunit alpha/FixB fan	QEN71_34135	paras_007746	protein-codi	NZ_CP1252	chromosom	975196	976146	-	950	0	11	786	9	718	
QEN71_RS34145	electron transfer flavoprotein subunit beta/FixA fami	QEN71_34140	paras_007747	protein-codi	NZ_CP1252	chromosom	976146	976946	-	796	0	9	1537	5	1003	
QEN71_RS34150	hypothetical protein	QEN71_34145	paras_007748	protein-codi	NZ_CP1252	chromosom	976943	978499	-	1553	0	7	543	6	520	
QEN71_RS34155	heterodisulfide reductase-related iron-sulfur binding	QEN71_34150	paras_007749	protein-codi	NZ_CP1252	chromosom	978521	980683	-	2163	0	22	1651	16	1395	
QEN71_RS34160	FAD-dependent oxidoreductase	QEN71_34155	paras_007750	protein-codi	NZ_CP1252	chromosom	980822	983014	-	2193	0	42	3810	35	3130	
QEN71_RS34165	aminomethyltransferase family protein	QEN71_34160	paras_007751	protein-codi	NZ_CP1252	chromosom	983203	984315	-	1113	0	15	1312	11	911	
QEN71_RS34170	PDR/VanB family oxidoreductase	QEN71_34165	paras_007752	protein-codi	NZ_CP1252	chromosom	984817	985773	+	957	0	16	2214	12	1370	
QEN71_RS34175	DUF3445 domain-containing protein	QEN71_34170	paras_007753	protein-codi	NZ_CP1252	chromosom	985789	986859	+	1071	0	18	2339	13	1377	
QEN71_RS34180	APC family permease	QEN71_34175	paras_007754	protein-codi	NZ_CP1252	chromosom	987048	988727	+	1680	0	35	2990	23	2317	
QEN71_RS34185	dimethylamine monooxygenase subunit DmmA fami	QEN71_34180	paras_007755	protein-codi	NZ_CP1252	chromosom	988763	989356	+	594	0	14	1279	5	496	
QEN71_RS34190	porin	QEN71_34185	paras_007756	protein-codi	NZ_CP1252	chromosom	989492	990637	+	1142	0	33	3636	24	2511	
QEN71_RS34195	histidine kinase	QEN71_34190	paras_007757	protein-codi	NZ_CP1252	chromosom	990634	992475	-	1838	0	23	3562	19	3507	
QEN71_RS34200	PAS domain-containing protein	QEN71_34195	paras_007758	protein-codi	NZ_CP1252	chromosom	992542	994413	+	1872	0	23	2541	15	1501	
QEN71_RS34205	hypothetical protein	QEN71_34200	paras_007759	protein-codi	NZ_CP1252	chromosom	994422	994910	-	489	0	9	1119	6	637	
QEN71_RS34210	APC family permease	QEN71_34205	paras_007760	protein-codi	NZ_CP1252	chromosom	994952	996595	-	1644	0	28	1711	26	1492	
QEN71_RS34215	aminomethyltransferase family protein	QEN71_34210	paras_007761	protein-codi	NZ_CP1252	chromosom	996751	997881	-	1131	0	25	2661	20	2403	
QEN71_RS34220	hypothetical protein	QEN71_34215	paras_007762	protein-codi	NZ_CP1252	chromosom	998112	998399	-	274	0	6	555	6	555	
QEN71_RS34225	hypothetical protein	QEN71_34220	paras_007763	protein-codi	NZ_CP1252	chromosom	998386	999063	-	664	0	6	515	5	460	
QEN71_RS34230	amino acid permease	QEN71_34225	paras_007764	protein-codi	NZ_CP1252	chromosom	999081	1000475	-	1395	0	49	4520	38	3435	
QEN71_RS34235	hypothetical protein	QEN71_34230	paras_007765	protein-codi	NZ_CP1252	chromosom	1000509	1000649	-	137	0	3	240	1	104	
QEN71_RS34240	hypothetical protein	QEN71_34235	paras_007766	protein-codi	NZ_CP1252	chromosom	1000646	1001032	-	383	0	9	916	8	666	
QEN71_RS34245	hypothetical protein	QEN71_34240	paras_007767	protein-codi	NZ_CP1252	chromosom	1001155	1001490	-	336	0	6	954	6	954	
QEN71_RS34250	DUF4148 domain-containing protein	QEN71_34245	paras_007768	protein-codi	NZ_CP1252	chromosom	1001724	1002047	+	324	0	9	1864	8	1430	
QEN71_RS34255	transporter substrate-binding domain-containing pro	QEN71_34250	paras_007769	protein-codi	NZ_CP1252	chromosom	1002588	1003310	+	723	0	11	1659	9	1536	
QEN71_RS34260	amino acid ABC transporter ATP-binding protein	QEN71_34255	paras_007770	protein-codi	NZ_CP1252	chromosom	1003471	1004304	+	834	0	12	2357	12	2357	
QEN71_RS34265	amino acid ABC transporter permease	QEN71_34260	paras_007771	protein-codi	NZ_CP1252	chromosom	1004313	1004984	+	672	0	9	1922	7	1656	
QEN71_RS34270	amino acid ABC transporter permease	QEN71_34265	paras_007772	protein-codi	NZ_CP1252	chromosom	1004995	1005657	+	663	0	16	1639	14	1510	
QEN71_RS34275	dihydrodipicolinate synthase family protein	QEN71_34270	paras_007773	protein-codi	NZ_CP1252	chromosom	1005695	1006573	+	879	0	24	3160	22	2941	
QEN71_RS34280	4-hydroxyproline epimerase	QEN71_34275	paras_007774	protein-codi	NZ_CP1252	chromosom	1006625	1007641	+	1013	0	18	3985	9	1865	
QEN71_RS34285	FAD-dependent oxidoreductase	QEN71_34280	paras_007775	protein-codi	NZ_CP1252	chromosom	1007638	1008927	+	1286	0	10	563	6	273	
QEN71_RS34290	proline racemase family protein	QEN71_34285	paras_007776	protein-codi	NZ_CP1252	chromosom	1008950	1010023	+	1074	0	17	1858	17	1858	
QEN71_RS34295	aldehyde dehydrogenase family protein	QEN71_34290	paras_007777	protein-codi	NZ_CP1252	chromosom	1010026	1011507	+	1482	0	17	1249	10	610	
QEN71_RS34300	proline racemase family protein	QEN71_34295	paras_007778	protein-codi	NZ_CP1252	chromosom	1011520	1012551	+	1032	0	13	2537	9	1603	
QEN71_RS34305	GntR family transcriptional regulator	QEN71_34300	paras_007779	protein-codi	NZ_CP1252	chromosom	1012624	1013331	+	708	0	17	1480	8	722	
QEN71_RS34310	hypothetical protein	QEN71_34305	paras_007780	protein-codi	NZ_CP1252	chromosom	1013544	1013858	+	315	0	5	815	1	205	
QEN71_RS34315	serine hydroxymethyltransferase	QEN71_34310	paras_007781	protein-codi	NZ_CP1252	chromosom	1014148	1015398	+	1251	141.0	19	1096	12	746	
QEN71_RS34320	helix-turn-helix domain-containing protein	QEN71_34315	paras_007782	protein-codi	NZ_CP1252	chromosom	1015594	1016589	-	996	0	10	949	10	949	

QEN71_RS34325	glucose 1-dehydrogenase	QEN71_34320	paras_007783	protein-codi	NZ_CP1252	chromosom	1016777	1017556	+	780	0	6	673	4	457	
QEN71_RS34330	amino acid permease	QEN71_34325	paras_007784	protein-codi	NZ_CP1252	chromosom	1017629	1019161	-	1533	0	25	1579	21	1230	
QEN71_RS34335	cytosine permease	QEN71_34330	paras_007785	protein-codi	NZ_CP1252	chromosom	1019557	1020921	+	1365	0	21	811	18	742	
QEN71_RS34340	haloacid dehalogenase type II	QEN71_34335	paras_007786	protein-codi	NZ_CP1252	chromosom	1021080	1021805	+	726	0	18	1558	17	1554	
QEN71_RS34345	PAS and helix-turn-helix domain-containing protein	QEN71_34340	paras_007787	protein-codi	NZ_CP1252	chromosom	1021818	1022297	-	480	0	7	287	4	55	
QEN71_RS34350	dipeptidase	QEN71_34345	paras_007788	protein-codi	NZ_CP1252	chromosom	1022542	1023510	+	969	0	6	161	6	161	
QEN71_RS34355	MFS transporter	QEN71_34350	paras_007789	protein-codi	NZ_CP1252	chromosom	1023556	1024896	+	1341	0	27	2363	17	1632	
QEN71_RS34360	MarR family transcriptional regulator	QEN71_34355	paras_007790	protein-codi	NZ_CP1252	chromosom	1024941	1025489	-	549	0	7	570	7	570	
QEN71_RS34365	sulfatase-like hydrolase/transferase	QEN71_34360	paras_007791	protein-codi	NZ_CP1252	chromosom	1025636	1027207	+	1572	0	24	1677	22	1661	
QEN71_RS34370	MFS transporter	QEN71_34365	paras_007792	protein-codi	NZ_CP1252	chromosom	1027270	1028604	+	1335	0	24	2713	19	2467	
QEN71_RS34375	fumarylacetoacetate hydrolase family protein	QEN71_34370	paras_007793	protein-codi	NZ_CP1252	chromosom	1028792	1029556	+	761	0	10	1343	9	1233	
QEN71_RS34380	fumarylacetoacetate hydrolase family protein	QEN71_34375	paras_007794	protein-codi	NZ_CP1252	chromosom	1029553	1030311	+	755	0	7	189	6	172	
QEN71_RS34385	5-carboxymethyl-2-hydroxymuconate semialdehyde	QEN71_34380	paras_007795	protein-codi	NZ_CP1252	chromosom	1030329	1031786	+	1458	0	8	669	7	637	
QEN71_RS34390	3,4-dihydroxyphenylacetate 2,3-dioxygenase	QEN71_34385	paras_007796	protein-codi	NZ_CP1252	chromosom	1031800	1032663	+	864	0	13	761	12	756	
QEN71_RS34395	5-carboxymethyl-2-hydroxymuconate Delta-isomera	QEN71_34390	paras_007797	protein-codi	NZ_CP1252	chromosom	1032684	1033082	+	399	0	3	66	1	3	TRUE
QEN71_RS34400	2-oxo-hepta-3-ene-1,7-dioic acid hydratase	QEN71_34395	paras_007798	protein-codi	NZ_CP1252	chromosom	1033101	1033904	+	804	0	1	7	1	7	TRUE
QEN71_RS34405	4-hydroxy-2-oxoheptanedioate aldolase	QEN71_34400	paras_007799	protein-codi	NZ_CP1252	chromosom	1033914	1034717	+	804	0	6	231	5	225	
QEN71_RS34410	helix-turn-helix domain-containing protein	QEN71_34405	paras_007800	protein-codi	NZ_CP1252	chromosom	1034739	1035722	-	984	0	9	1105	6	725	
QEN71_RS34415	hypothetical protein	QEN71_34410	paras_007801	protein-codi	NZ_CP1252	chromosom	1035733	1036077	-	345	0	7	445	6	441	
QEN71_RS34420	porin	QEN71_34415	paras_007802	protein-codi	NZ_CP1252	chromosom	1036183	1037385	-	1203	0	20	1200	17	944	
QEN71_RS34425	amidase	QEN71_34420	paras_007803	protein-codi	NZ_CP1252	chromosom	1037534	1038997	-	1464	0	12	931	9	769	
QEN71_RS34430	MFS transporter	QEN71_34425	paras_007804	protein-codi	NZ_CP1252	chromosom	1039055	1040368	-	1314	0	12	1299	9	1114	
QEN71_RS34435	LysR substrate-binding domain-containing protein	QEN71_34430	paras_007805	protein-codi	NZ_CP1252	chromosom	1040633	1041559	+	927	0	7	335	5	250	
QEN71_RS34440	MoaF C-terminal domain-containing protein	QEN71_34435	paras_007806	protein-codi	NZ_CP1252	chromosom	1041695	1042561	+	863	0	22	4356	20	4147	
QEN71_RS34445	SDR family oxidoreductase	QEN71_34440	paras_007807	protein-codi	NZ_CP1252	chromosom	1042558	1043361	+	800	0	8	614	7	189	
QEN71_RS34450	SDR family oxidoreductase	QEN71_34445	paras_007808	protein-codi	NZ_CP1252	chromosom	1043376	1044176	-	801	0	13	2937	12	2922	
QEN71_RS34455	LuxR C-terminal-related transcriptional regulator	QEN71_34450	paras_007809	protein-codi	NZ_CP1252	chromosom	1044404	1045105	+	702	0	9	639	9	639	
QEN71_RS34460	hypothetical protein	QEN71_34455	paras_007810	protein-codi	NZ_CP1252	chromosom	1045121	1045570	+	450	0	9	952	9	952	
QEN71_RS34465	aldehyde dehydrogenase family protein	QEN71_34460	paras_007811	protein-codi	NZ_CP1252	chromosom	1045735	1047234	+	1500	0	20	3268	18	3223	
QEN71_RS34470	helix-turn-helix domain-containing protein	QEN71_34465	paras_007812	protein-codi	NZ_CP1252	chromosom	1047382	1048320	+	939	0	19	1964	15	1523	
QEN71_RS34475	APC family permease	QEN71_34470	paras_007813	protein-codi	NZ_CP1252	chromosom	1048570	1050018	+	1449	0	20	2525	18	2389	
QEN71_RS34480	porin	QEN71_34475	paras_007814	protein-codi	NZ_CP1252	chromosom	1050181	1051395	+	1215	0	28	2454	22	1882	
QEN71_RS34485	FAD-binding oxidoreductase	QEN71_34480	paras_007815	protein-codi	NZ_CP1252	chromosom	1051580	1052881	+	1302	0	27	3223	22	2513	
QEN71_RS34490	cupin domain-containing protein	QEN71_34485	paras_007816	protein-codi	NZ_CP1252	chromosom	1052912	1053280	+	369	0	7	780	4	562	
QEN71_RS34495	cyclopropane-fatty-acyl-phospholipid synthase fami	QEN71_34490	paras_007817	protein-codi	NZ_CP1252	chromosom	1053310	1054251	-	942	0	13	1105	10	933	
QEN71_RS34500	chloride channel protein	QEN71_34495	paras_007818	protein-codi	NZ_CP1252	chromosom	1054279	1056099	-	1821	0	25	2539	24	2536	
QEN71_RS34505	GNAT family N-acetyltransferase	partial;pseudo;QEN71_34500		pseudogene	NZ_CP1252	chromosom	1056220	1056378	-	159	0	5	305	4	303	
QEN71_RS34510	YdcF family protein	QEN71_34505	paras_007819	protein-codi	NZ_CP1252	chromosom	1056453	1057127	-	674	0	21	1562	16	1149	
QEN71_RS34515	type VI secretion system membrane subunit TssM	QEN71_34510	paras_007820	protein-codi	NZ_CP1252	chromosom	1057127	1061137	-	4010	0	75	6951	60	6201	
QEN71_RS34520	type VI secretion system protein TssL, long form	QEN71_34515	paras_007821	protein-codi	NZ_CP1252	chromosom	1061162	1062412	-	1251	0	18	1822	15	1213	
QEN71_RS34525	type VI secretion system baseplate subunit TssK	QEN71_34520	paras_007822	protein-codi	NZ_CP1252	chromosom	1062560	1063906	-	1347	0	19	2320	9	1744	
QEN71_RS34530	type VI secretion system lipoprotein TssJ	QEN71_34525	paras_007823	protein-codi	NZ_CP1252	chromosom	1063919	1064428	-	510	0	6	847	4	396	
QEN71_RS34535	Hcp family type VI secretion system effector	QEN71_34530	paras_007824	protein-codi	NZ_CP1252	chromosom	1064506	1064988	-	483	0	7	1237	5	1186	
QEN71_RS34540	hypothetical protein	QEN71_34535	paras_007825	protein-codi	NZ_CP1252	chromosom	1065082	1065588	-	503	0	8	641	6	632	
QEN71_RS34545	DUF2778 domain-containing protein	QEN71_34540	paras_007826	protein-codi	NZ_CP1252	chromosom	1065585	1065947	-	359	0	4	297	2	30	
QEN71_RS34550	type VI secretion system contractile sheath large su	QEN71_34545	paras_007827	protein-codi	NZ_CP1252	chromosom	1066023	1067516	-	1494	0	29	2346	28	2285	
QEN71_RS34555	type VI secretion system contractile sheath small su	QEN71_34550	paras_007828	protein-codi	NZ_CP1252	chromosom	1067578	1068105	-	528	0	7	917	5	716	
QEN71_RS34560	type VI secretion system ATPase TssH	QEN71_34555	paras_007829	protein-codi	NZ_CP1252	chromosom	1068143	1070869	-	2727	0	27	2633	23	2284	
QEN71_RS34565	TagK domain-containing protein	QEN71_34560	paras_007830	protein-codi	NZ_CP1252	chromosom	1071396	1072043	+	644	0	3	26	2	20	
QEN71_RS34570	type VI secretion system accessory protein TagJ	QEN71_34565	paras_007831	protein-codi	NZ_CP1252	chromosom	1072040	1072909	+	862	0	15	1869	15	1869	
QEN71_RS34575	type VI secretion system baseplate subunit TssE	QEN71_34570	paras_007832	protein-codi	NZ_CP1252	chromosom	1072906	1073442	+	533	0	4	599	4	599	
QEN71_RS34580	type VI secretion system baseplate subunit TssF	QEN71_34575	paras_007833	protein-codi	NZ_CP1252	chromosom	1073460	1075349	+	1889	0	43	4041	31	3208	
QEN71_RS34585	type VI secretion system baseplate subunit TssG	QEN71_34580	paras_007834	protein-codi	NZ_CP1252	chromosom	1075349	1076413	+	1056	0	12	797	10	558	
QEN71_RS34590	type VI secretion system ImpA family N-terminal dor	QEN71_34585	paras_007835	protein-codi	NZ_CP1252	chromosom	1076406	1077515	+	1102	0	14	1499	10	1287	
QEN71_RS34595	DUF3592 domain-containing protein	QEN71_34590	paras_007836	protein-codi	NZ_CP1252	chromosom	1077550	1077963	+	414	0	0	0	0	0	TRUE
QEN71_RS34600	glycoside hydrolase family protein	QEN71_34595	paras_007837	protein-codi	NZ_CP1252	chromosom	1078017	1078517	+	497	0	5	472	5	472	
QEN71_RS34605	lysozyme inhibitor LprI family protein	QEN71_34600	paras_007838	protein-codi	NZ_CP1252	chromosom	1078514	1078933	+	416	0	2	206	2	206	
QEN71_RS34610	type VI secretion system tip protein TssI/VgrG	QEN71_34605	paras_007839	protein-codi	NZ_CP1252	chromosom	1079040	1081280	+	2241	0	33	1315	28	973	
QEN71_RS34615	hypothetical protein	QEN71_34610	paras_007840	protein-codi	NZ_CP1252	chromosom	1081295	1082668	+	1373	0	17	1057	11	639	
QEN71_RS34620	RHS repeat-associated core domain-containing pro	QEN71_34615	paras_007841	protein-codi	NZ_CP1252	chromosom	1082668	1087056	+	4388	0	89	5724	71	4856	
QEN71_RS34625	hypothetical protein	QEN71_34620	paras_007842	protein-codi	NZ_CP1252	chromosom	1087072	1087347	+	276	0	6	147	5	142	
QEN71_RS34630	SymE family type I addiction module toxin	QEN71_34625	paras_007843	protein-codi	NZ_CP1252	chromosom	1087424	1087657	-	234	0	1	64	1	64	

QEN71_RS34635	helix-turn-helix transcriptional regulator	QEN71_34630	paras_007844	protein-codi	NZ_CP1252	chromosome	1087735	1088151	-		417	0	4	470	1	101	
QEN71_RS34640	IS66 family transposase	QEN71_34635	paras_007845	protein-codi	NZ_CP1252	chromosome	1088230	1089822	-		1593	0	20	560	17	535	
QEN71_RS34645	IS66 family insertion sequence element accessory p	QEN71_34640	paras_007846	protein-codi	NZ_CP1252	chromosome	1089875	1090168	-		294	0	7	329	5	221	
QEN71_RS34650	transposase	QEN71_34645	paras_007847	protein-codi	NZ_CP1252	chromosome	1090213	1090623	-		411	0	1	6	1	6	TRUE
QEN71_RS34655	cytochrome P450	pseudo;QEN71_34650		pseudogene	NZ_CP1252	chromosome	1090932	1092316	-		1385	0	16	1263	13	1153	
QEN71_RS34660	sterol desaturase family protein	QEN71_34655	paras_007850	protein-codi	NZ_CP1252	chromosome	1092336	1093526	-		1191	0	13	762	7	310	
QEN71_RS34665	hypothetical protein	QEN71_34660		protein-codi	NZ_CP1252	chromosome	1094057	1094440	+		384	0	8	447	6	167	
QEN71_RS34670	hypothetical protein	QEN71_34665	paras_007852	protein-codi	NZ_CP1252	chromosome	1094626	1095330	+		705	0	11	1147	10	1142	
QEN71_RS34675	hypothetical protein	partial;pseudo		protein-codi	NZ_CP1252	chromosome	1095503	1095550	+		48	0	0	0	0	0	
QEN71_RS34680	proline dehydrogenase family protein	QEN71_34670	paras_007854	protein-codi	NZ_CP1252	chromosome	1095940	1096851	+		912	0	10	348	7	221	
QEN71_RS34685	type VI secretion system tip protein TssI/VgrG	QEN71_34675	paras_007855	protein-codi	NZ_CP1252	chromosome	1097302	1099479	+		2178	0	24	1681	22	1636	
QEN71_RS34690	hypothetical protein	QEN71_34680	paras_007856	protein-codi	NZ_CP1252	chromosome	1099522	1102035	+		2514	0	42	2497	39	2398	
QEN71_RS34695	hypothetical protein	QEN71_34685	paras_007857	protein-codi	NZ_CP1252	chromosome	1102145	1102996	+		852	0	13	1160	11	854	
QEN71_RS34700	PAAR domain-containing protein	QEN71_34690	paras_007858	protein-codi	NZ_CP1252	chromosome	1103012	1103266	+		255	0	5	443	4	428	
QEN71_RS34705	hypothetical protein	QEN71_34695	paras_007859	protein-codi	NZ_CP1252	chromosome	1103462	1103719	+		258	0	2	48	2	48	
QEN71_RS34710	hypothetical protein	QEN71_34700	paras_007860	protein-codi	NZ_CP1252	chromosome	1104203	1104475	+		273	0	3	141	2	94	
QEN71_RS34715	zinc ribbon domain-containing protein	QEN71_34705	paras_007861	protein-codi	NZ_CP1252	chromosome	1104519	1105634	+		1116	0	20	695	18	625	
QEN71_RS34720	DUF1254 domain-containing protein	QEN71_34710	paras_007862	protein-codi	NZ_CP1252	chromosome	1106282	1107685	+		1404	0	24	1269	23	1254	
QEN71_RS34725	DUF262 domain-containing protein	QEN71_34715	paras_007863	protein-codi	NZ_CP1252	chromosome	1107872	1109584	-		1713	0	33	3371	26	2707	
QEN71_RS34730	hypothetical protein	QEN71_34720	paras_007864	protein-codi	NZ_CP1252	chromosome	1109809	1110945	-		1137	0	37	2303	31	2030	
QEN71_RS34735	hypothetical protein	QEN71_34725	paras_007865	protein-codi	NZ_CP1252	chromosome	1111760	1112830	-		1071	0	10	589	6	444	
QEN71_RS34740	hypothetical protein	QEN71_34730	paras_007866	protein-codi	NZ_CP1252	chromosome	1113383	1114852	+		1470	0	23	1368	20	1223	
QEN71_RS34745	hypothetical protein	QEN71_34735		protein-codi	NZ_CP1252	chromosome	1115607	1115819	-		211	0	1	23	0	0	
QEN71_RS34750	hypothetical protein	QEN71_34740	paras_007867	protein-codi	NZ_CP1252	chromosome	1115818	1118643	+		2824	0	44	4334	34	3364	
QEN71_RS34755	ATP-binding protein	QEN71_34745	paras_007868	protein-codi	NZ_CP1252	chromosome	1118745	1119722	+		978	0	7	845	5	659	
QEN71_RS34760	phage integrase family protein	QEN71_34750	paras_007869	protein-codi	NZ_CP1252	chromosome	1119767	1121797	+		2031	0	33	5856	28	5591	
QEN71_RS34765	helix-turn-helix transcriptional regulator	QEN71_34755	paras_007870	protein-codi	NZ_CP1252	chromosome	1122631	1122876	-		246	0	16	2464	16	2464	
QEN71_RS34770	hypothetical protein	QEN71_34760	paras_007871	protein-codi	NZ_CP1252	chromosome	1123346	1123843	+		498	0	9	1651	8	1574	
QEN71_RS34775	hypothetical protein	QEN71_34765	paras_007872	protein-codi	NZ_CP1252	chromosome	1124866	1125225	+		360	0	15	2138	9	1267	
QEN71_RS34780	HAD domain-containing protein	QEN71_34770	paras_007873	protein-codi	NZ_CP1252	chromosome	1125431	1125907	+		477	0	18	1610	13	950	
QEN71_RS34785	HAD domain-containing protein	QEN71_34775	paras_007874	protein-codi	NZ_CP1252	chromosome	1126103	1126657	+		554	0	11	475	9	424	
QEN71_RS34790	hypothetical protein	QEN71_34780	paras_007875	protein-codi	NZ_CP1252	chromosome	1126657	1127049	+		392	0	15	1969	11	1643	
QEN71_RS34795	TniQ family protein	QEN71_34785	paras_007876	protein-codi	NZ_CP1252	chromosome	1127642	1129090	+		1449	0	33	3095	27	2874	
QEN71_RS34800	threonine--tRNA ligase	QEN71_34790	paras_007877	protein-codi	NZ_CP1252	chromosome	1129262	1130401	-		1027	0	14	1976	12	1842	
QEN71_RS34805	hypothetical protein	QEN71_34795		protein-codi	NZ_CP1252	chromosome	1130289	1130537	-		136	0	5	468	5	468	
QEN71_RS34810	PHB depolymerase family esterase	QEN71_34800	paras_007878	protein-codi	NZ_CP1252	chromosome	1131070	1132188	+		1119	0	42	4255	32	3064	
QEN71_RS34815	hypothetical protein	QEN71_34805	paras_007879	protein-codi	NZ_CP1252	chromosome	1132315	1135053	-		2735	0	53	4967	44	4341	
QEN71_RS34820	alpha-amylase family glycosyl hydrolase	QEN71_34810	paras_007880	protein-codi	NZ_CP1252	chromosome	1135050	1136888	-		1834	0	28	1804	26	1726	
QEN71_RS34825	hypothetical protein	QEN71_34815	paras_007881	protein-codi	NZ_CP1252	chromosome	1136888	1137352	-		464	0	9	176	5	99	
QEN71_RS34830	heme peroxidase family protein	QEN71_34820	paras_007882	protein-codi	NZ_CP1252	chromosome	1138009	1139736	+		1728	0	22	1098	15	602	
QEN71_RS34835	hypothetical protein	QEN71_34825	paras_007883	protein-codi	NZ_CP1252	chromosome	1139916	1142348	+		2433	0	32	2681	26	2379	
QEN71_RS34840	hypothetical protein	QEN71_34830	paras_007884	protein-codi	NZ_CP1252	chromosome	1142529	1143050	+		522	0	12	1334	6	883	
QEN71_RS34845	HNH endonuclease	QEN71_34835	paras_007885	protein-codi	NZ_CP1252	chromosome	1143340	1144380	-		1041	0	26	3094	20	1981	
QEN71_RS34850	hypothetical protein	QEN71_34840	paras_007886	protein-codi	NZ_CP1252	chromosome	1144441	1145034	-		594	0	24	2548	19	1766	
QEN71_RS34855	hypothetical protein	QEN71_34845	paras_007887	protein-codi	NZ_CP1252	chromosome	1145834	1146385	+		552	0	23	4032	19	2768	
QEN71_RS34860	hypothetical protein	QEN71_34850	paras_007888	protein-codi	NZ_CP1252	chromosome	1146569	1147765	+		1193	0	26	2098	23	1813	
QEN71_RS34865	DUF6521 family protein	QEN71_34855	paras_007889	protein-codi	NZ_CP1252	chromosome	1147762	1148217	+		448	0	9	452	8	445	
QEN71_RS34870	DUF3732 domain-containing protein	QEN71_34860	paras_007890	protein-codi	NZ_CP1252	chromosome	1148214	1150064	+		1847	0	42	3478	36	3126	
QEN71_RS34875	hypothetical protein	QEN71_34865	paras_007891	protein-codi	NZ_CP1252	chromosome	1150229	1153012	+		2784	0	60	8438	49	6989	
QEN71_RS34880	M15 family metallopeptidase	QEN71_34870	paras_007892	protein-codi	NZ_CP1252	chromosome	1153219	1153902	+		680	0	21	1951	18	1584	
QEN71_RS34885	TIGR02594 family protein	pseudo;QEN71_34875		pseudogene	NZ_CP1252	chromosome	1153899	1154342	+		440	0	10	745	8	448	
QEN71_RS34890	ester cyclase			protein-codi	NZ_CP1252	chromosome	1154722	1155519	-		798	0	14	1383	8	681	
QEN71_RS34895	filamentous hemagglutinin	QEN71_34885	paras_007895	protein-codi	NZ_CP1252	chromosome	1155816	1163855	+		8040	0	198	25798	158	21489	
QEN71_RS34900	filamentous hemagglutinin N-terminal domain-conta	QEN71_34890	paras_007896	protein-codi	NZ_CP1252	chromosome	1164400	1166436	+		2036	0	28	1637	20	1356	
QEN71_RS34905	ShlB/FhaC/HecB family hemolysin secretion/activat	QEN71_34895	paras_007897	protein-codi	NZ_CP1252	chromosome	1166436	1168265	+		1829	0	51	7009	44	6353	
QEN71_RS34910	peptidyl-prolyl cis-trans isomerase	QEN71_34900	paras_007898	protein-codi	NZ_CP1252	chromosome	1168273	1169142	+		870	0	19	2410	17	2228	
QEN71_RS34915	IS1182 family transposase	QEN71_34905	paras_007899	protein-codi	NZ_CP1252	chromosome	1169296	1170741	+		1446	1288.0	48	6083	34	3994	
QEN71_RS34920	hypothetical protein	QEN71_34910	paras_007900	protein-codi	NZ_CP1252	chromosome	1170944	1171222	+		279	104.0	21	4666	14	1854	
QEN71_RS34925	DMT family transporter	QEN71_34915	paras_007901	protein-codi	NZ_CP1252	chromosome	1171337	1172278	-		942	0	24	2440	22	2390	
QEN71_RS34930	LysE family transporter	QEN71_34920	paras_007902	protein-codi	NZ_CP1252	chromosome	1172303	1172914	-		612	0	21	3043	16	2528	
QEN71_RS34935	transcriptional regulator GcvA	QEN71_34925	paras_007903	protein-codi	NZ_CP1252	chromosome	1173046	1173954	+		909	0	18	1572	16	1343	
QEN71_RS34940	lecithin retinol acyltransferase family protein	QEN71_34930	paras_007904	protein-codi	NZ_CP1252	chromosome	1174694	1175230	+		537	0	17	1667	11	978	

QEN71_RS34945	hypothetical protein	QEN71_34935	paras_007905	protein-codi	NZ_CP1252	chromosom	1175384	1175848	+	465	0	8	593	6	316	
QEN71_RS34950	collagen-like protein	QEN71_34940	paras_007906	protein-codi	NZ_CP1252	chromosom	1175992	1176984	-	993	0	37	3184	28	2237	
QEN71_RS34955	hypothetical protein	QEN71_34945	paras_007907	protein-codi	NZ_CP1252	chromosom	1177185	1178093	-	909	0	30	3472	25	3393	
QEN71_RS34960	hypothetical protein	QEN71_34950	paras_007908	protein-codi	NZ_CP1252	chromosom	1178264	1178917	+	654	0	13	1348	13	1348	
QEN71_RS34965	DUF2384 domain-containing protein	QEN71_34955	paras_007909	protein-codi	NZ_CP1252	chromosom	1178938	1179282	+	345	0	2	243	0	0	
QEN71_RS34970	class I tRNA ligase family protein	QEN71_34960	paras_007910	protein-codi	NZ_CP1252	chromosom	1179505	1181055	+	1547	0	44	9345	31	6492	
QEN71_RS34975	hypothetical protein	QEN71_34965	paras_007911	protein-codi	NZ_CP1252	chromosom	1181052	1181417	-	358	0	10	3700	8	2534	
QEN71_RS34980	glycoside hydrolase family 73 protein	QEN71_34970	paras_007912	protein-codi	NZ_CP1252	chromosom	1181414	1181950	-	533	0	23	3482	21	3143	
QEN71_RS34985	hypothetical protein	QEN71_34975	paras_007913	protein-codi	NZ_CP1252	chromosom	1181960	1182184	-	225	0	6	2615	3	1524	
QEN71_RS34990	hypothetical protein	QEN71_34980		protein-codi	NZ_CP1252	chromosom	1182662	1182901	+	240	0	3	342	1	156	
QEN71_RS34995	patatin-like phospholipase family protein	QEN71_34985	paras_007914	protein-codi	NZ_CP1252	chromosom	1183306	1185522	+	2217	0	59	13460	48	12108	
QEN71_RS35000	hypothetical protein	QEN71_34990	paras_007915	protein-codi	NZ_CP1252	chromosom	1185563	1186513	+	951	0	17	3953	14	3327	
QEN71_RS35005	ATP-binding protein	QEN71_34995	paras_007916	protein-codi	NZ_CP1252	chromosom	1186698	1187840	-	1143	0	20	2185	17	1869	
QEN71_RS35010	response regulator	QEN71_35000	paras_007917	protein-codi	NZ_CP1252	chromosom	1187920	1188627	-	708	0	14	2716	8	1501	
QEN71_RS35015	hypothetical protein	QEN71_35005	paras_007918	protein-codi	NZ_CP1252	chromosom	1189042	1189512	-	471	0	2	36	2	36	
QEN71_RS35020	efflux RND transporter periplasmic adaptor subunit	QEN71_35010	paras_007919	protein-codi	NZ_CP1252	chromosom	1190551	1191843	+	1293	0	18	5177	15	4857	
QEN71_RS35025	efflux RND transporter permease subunit	QEN71_35015	paras_007920	protein-codi	NZ_CP1252	chromosom	1191852	1195049	+	3198	0	41	7947	34	6820	
QEN71_RS35030	efflux transporter outer membrane subunit	QEN71_35020	paras_007921	protein-codi	NZ_CP1252	chromosom	1195058	1196713	+	1656	0	23	3610	18	3379	
QEN71_RS35035	outer membrane protein assembly factor BamA	QEN71_35025	paras_007922	protein-codi	NZ_CP1252	chromosom	1197429	1199732	+	2304	371.0	65	5938	55	5451	
QEN71_RS35040	bacteriohopanetetrol glucosamine biosynthesis glyc	QEN71_35030	paras_007923	protein-codi	NZ_CP1252	chromosom	1200182	1201420	+	1239	0	24	2129	16	745	
QEN71_RS35045	hopanoid biosynthesis associated radical SAM prot	QEN71_35035	paras_007924	protein-codi	NZ_CP1252	chromosom	1201466	1202890	+	1421	507.0	31	8311	24	7858	
QEN71_RS35050	hopanoid biosynthesis-associated protein HpnK	QEN71_35040	paras_007925	protein-codi	NZ_CP1252	chromosom	1202887	1203741	+	851	0	26	4860	22	2927	
QEN71_RS35055	lysylphosphatidylglycerol synthase domain-containi	QEN71_35045	paras_007926	protein-codi	NZ_CP1252	chromosom	1203762	1204739	+	978	0	23	4683	21	4455	
QEN71_RS35060	ATP-binding protein	QEN71_35050	paras_007927	protein-codi	NZ_CP1252	chromosom	1204804	1205895	-	1092	0	24	4760	15	2566	
QEN71_RS35065	response regulator	QEN71_35055	paras_007928	protein-codi	NZ_CP1252	chromosom	1205898	1206584	-	687	0	25	6061	21	4938	
QEN71_RS35070	ABC transporter ATP-binding protein	QEN71_35060	paras_007929	protein-codi	NZ_CP1252	chromosom	1207010	1208818	+	1809	0	36	6927	26	5082	
QEN71_RS35075	hypothetical protein	QEN71_35065	paras_007930	protein-codi	NZ_CP1252	chromosom	1208954	1209964	+	1011	0	25	2555	20	2148	
QEN71_RS35080	hypothetical protein	QEN71_35070	paras_007931	protein-codi	NZ_CP1252	chromosom	1210108	1213383	+	3276	0	83	10543	61	8705	
QEN71_RS35085	PsiF family protein	QEN71_35075	paras_007932	protein-codi	NZ_CP1252	chromosom	1213687	1213980	-	294	0	2	325	0	0	
QEN71_RS35090	flagellar filament capping protein FliD	QEN71_35080	paras_007933	protein-codi	NZ_CP1252	chromosom	1214486	1215913	-	1428	0	24	2987	15	1952	
QEN71_RS35095	HU family DNA-binding protein	QEN71_35085	paras_007934	protein-codi	NZ_CP1252	chromosom	1216477	1216755	-	279	0	2	131	2	131	
QEN71_RS35100	response regulator transcription factor	QEN71_35090	paras_007935	protein-codi	NZ_CP1252	chromosom	1216947	1217324	-	378	0	3	45	3	45	
QEN71_RS35105	methyl-accepting chemotaxis protein	partial;pseudo;QEN71_35095		pseudogene	NZ_CP1252	chromosom	1217674	1219230	+	1557	0	19	1988	11	1070	
QEN71_RS35110	alpha/beta fold hydrolase	QEN71_35100	paras_007937	protein-codi	NZ_CP1252	chromosom	1219600	1220430	+	831	0	19	3450	18	3426	
QEN71_RS35115	DUF2971 domain-containing protein	QEN71_35105	paras_007938	protein-codi	NZ_CP1252	chromosom	1220694	1221647	-	954	0	36	3935	30	2803	
QEN71_RS35120	hypothetical protein	QEN71_35110	paras_007939	protein-codi	NZ_CP1252	chromosom	1221989	1224046	+	2058	0	42	7032	33	5728	
QEN71_RS35125	hypothetical protein	QEN71_35115	paras_007940	protein-codi	NZ_CP1252	chromosom	1224099	1226345	-	2247	0	63	8693	54	7795	
QEN71_RS35130	RiPP maturation radical SAM C-methyltransferase	QEN71_35120	paras_007941	protein-codi	NZ_CP1252	chromosom	1226581	1228584	-	2004	0	49	6220	43	5734	
QEN71_RS35135	hypothetical protein	QEN71_35125	paras_007942	protein-codi	NZ_CP1252	chromosom	1228589	1229431	-	843	0	4	1044	3	770	
QEN71_RS35140	hypothetical protein	QEN71_35130	paras_007943	protein-codi	NZ_CP1252	chromosom	1229646	1230152	-	507	0	16	2371	10	1652	
QEN71_RS35145	hypothetical protein	QEN71_35135	paras_007944	protein-codi	NZ_CP1252	chromosom	1230185	1230574	-	390	0	7	219	2	66	
QEN71_RS35150	EAL domain-containing protein	QEN71_35140	paras_007945	protein-codi	NZ_CP1252	chromosom	1231075	1232355	-	1281	0	23	3718	21	3368	
QEN71_RS35155	porin	QEN71_35145	paras_007946	protein-codi	NZ_CP1252	chromosom	1232824	1233954	+	1131	0	58	11773	54	11649	
QEN71_RS35160	efflux transporter outer membrane subunit	QEN71_35150	paras_007947	protein-codi	NZ_CP1252	chromosom	1234143	1235585	-	1443	0	28	8901	20	6400	
QEN71_RS35165	MacB family efflux pump subunit	QEN71_35155	paras_007948	protein-codi	NZ_CP1252	chromosom	1235599	1237680	-	2078	0	32	5745	27	5268	
QEN71_RS35170	macrolide transporter subunit MacA	QEN71_35160	paras_007949	protein-codi	NZ_CP1252	chromosom	1237677	1238891	-	1211	0	17	3244	13	2745	
QEN71_RS35175	polysaccharide deacetylase family protein	QEN71_35165	paras_007950	protein-codi	NZ_CP1252	chromosom	1239110	1239850	-	741	0	24	3861	15	2124	
QEN71_RS35180	glycosyltransferase	QEN71_35170	paras_007951	protein-codi	NZ_CP1252	chromosom	1239995	1241140	-	1146	0	23	7604	16	3425	
QEN71_RS35185	hypothetical protein	QEN71_35175	paras_007952	protein-codi	NZ_CP1252	chromosom	1241537	1241761	+	225	0	8	2962	6	2388	
QEN71_RS35190	hypothetical protein	QEN71_35180	paras_007953	protein-codi	NZ_CP1252	chromosom	1241995	1242273	-	279	0	23	3941	15	2888	
QEN71_RS35195	phosphodiesterase	QEN71_35185	paras_007954	protein-codi	NZ_CP1252	chromosom	1242829	1244703	-	1875	0	40	5595	37	5220	
QEN71_RS35200	flagellin domain-containing protein	QEN71_35190	paras_007955	protein-codi	NZ_CP1252	chromosom	1245189	1246007	-	819	0	3	445	0	0	
QEN71_RS35205	flagellar hook protein FlgE	QEN71_35195	paras_007956	protein-codi	NZ_CP1252	chromosom	1246714	1248213	+	1500	0	32	3927	26	3344	
QEN71_RS35210	flagellar basal-body rod protein FlgG	QEN71_35200	paras_007957	protein-codi	NZ_CP1252	chromosom	1248371	1249153	+	783	0	13	1030	10	915	
QEN71_RS35215	flagellar hook-associated protein FlgK	QEN71_35205	paras_007958	protein-codi	NZ_CP1252	chromosom	1249263	1251248	+	1986	0	30	4192	27	4058	
QEN71_RS35220	hypothetical protein	QEN71_35210	paras_007959	protein-codi	NZ_CP1252	chromosom	1251263	1251589	+	327	0	4	247	2	232	
QEN71_RS35225	hypothetical protein	QEN71_35215	paras_007960	protein-codi	NZ_CP1252	chromosom	1252940	1253935	-	996	0	20	1219	14	794	
QEN71_RS35230	hypothetical protein	QEN71_35220	paras_007961	protein-codi	NZ_CP1252	chromosom	1254317	1255138	-	822	0	15	1200	12	1105	
QEN71_RS35235	hypothetical protein	QEN71_35225	paras_007962	protein-codi	NZ_CP1252	chromosom	1255150	1256814	-	1665	0	30	1951	23	1720	
QEN71_RS35240	IS5/IS1182 family transposase	partial;pseudo;QEN71_35230		pseudogene	NZ_CP1252	chromosom	1256895	1256993	-	99	0	1	243	1	243	
QEN71_RS35245	hypothetical protein			protein-codi	NZ_CP1252	chromosom	1257529	1258047	-	519	0	5	531	3	269	
QEN71_RS35250	hypothetical protein	QEN71_35240		protein-codi	NZ_CP1252	chromosom	1258057	1258248	-	190	0	4	167	1	8	

QEN71_RS35255	tetratricopeptide repeat protein	QEN71_35245	paras_007965	protein-codi	NZ_CP1252	chromosom	1258247	1259734	+	1486	0	14	917	8	483	
QEN71_RS35260	M15 family metallopeptidase	QEN71_35250	paras_007966	protein-codi	NZ_CP1252	chromosom	1260817	1261320	+	504	0	7	340	3	197	
QEN71_RS35265	hypothetical protein	QEN71_35255	paras_007967	protein-codi	NZ_CP1252	chromosom	1261575	1262330	+	756	0	6	360	6	360	
QEN71_RS35270	hypothetical protein	QEN71_35260	paras_007968	protein-codi	NZ_CP1252	chromosom	1262402	1264216	+	1815	0	43	3503	36	2513	
QEN71_RS35275	FAD-dependent oxidoreductase	QEN71_35265	paras_007970	protein-codi	NZ_CP1252	chromosom	1264707	1265282	+	544	0	8	639	7	637	
QEN71_RS35280	FAD-binding oxidoreductase	QEN71_35270	paras_007971	protein-codi	NZ_CP1252	chromosom	1265251	1265775	+	493	0	4	405	3	379	
QEN71_RS35285	porin	QEN71_35275	paras_007972	protein-codi	NZ_CP1252	chromosom	1265964	1267118	-	1155	0	28	1913	23	1688	
QEN71_RS35290	NAD(P)/FAD-dependent oxidoreductase	QEN71_35280	paras_007973	protein-codi	NZ_CP1252	chromosom	1267605	1268978	+	1374	0	15	1602	12	1226	
QEN71_RS35295	BPL-N domain-containing protein	QEN71_35285	paras_007974	protein-codi	NZ_CP1252	chromosom	1269077	1269814	+	738	0	24	2580	22	2539	
QEN71_RS35300	winged helix-turn-helix domain-containing protein	QEN71_35290		protein-codi	NZ_CP1252	chromosom	1270584	1271024	+	441	0	11	2297	11	2297	
QEN71_RS35305	DUF535 family protein	QEN71_35295	paras_007976	protein-codi	NZ_CP1252	chromosom	1271176	1272225	-	1050	0	20	3428	16	3255	
QEN71_RS35310	TonB-dependent receptor	QEN71_35300	paras_007977	protein-codi	NZ_CP1252	chromosom	1272985	1275198	+	2214	0	73	11040	60	9763	
QEN71_RS35315	SET domain-containing protein-lysine N-methyltrans	QEN71_35305	paras_007978	protein-codi	NZ_CP1252	chromosom	1275346	1275783	-	438	0	9	2049	9	2049	
QEN71_RS35320	HU family DNA-binding protein	QEN71_35310	paras_007979	protein-codi	NZ_CP1252	chromosom	1276071	1276349	+	279	0	3	157	3	157	
QEN71_RS35325	hypothetical protein	QEN71_35315	paras_007980	protein-codi	NZ_CP1252	chromosom	1276719	1279082	-	2360	0	50	8283	40	6860	
QEN71_RS35330	alpha/beta fold hydrolase	QEN71_35320	paras_007981	protein-codi	NZ_CP1252	chromosom	1279079	1280410	-	1328	0	32	2225	27	2024	
QEN71_RS35335	hypothetical protein	QEN71_35325	paras_007982	protein-codi	NZ_CP1252	chromosom	1280934	1281692	-	759	0	13	640	12	636	
QEN71_RS35340	aminotransferase class III-fold pyridoxal phosphate-	QEN71_35330	paras_007983	protein-codi	NZ_CP1252	chromosom	1282238	1283602	-	1365	0	29	2348	23	2125	
QEN71_RS35345	MFS transporter	QEN71_35335	paras_007984	protein-codi	NZ_CP1252	chromosom	1283726	1285027	-	1302	0	24	2655	19	2263	
QEN71_RS35350	porin	QEN71_35340	paras_007985	protein-codi	NZ_CP1252	chromosom	1285157	1286242	-	1086	0	31	2602	26	2023	
QEN71_RS35355	TetR/AcrR family transcriptional regulator	QEN71_35345	paras_007986	protein-codi	NZ_CP1252	chromosom	1286589	1287413	+	825	0	10	1737	9	1735	
QEN71_RS35360	aryldialkylphosphatase	QEN71_35350	paras_007987	protein-codi	NZ_CP1252	chromosom	1287423	1288466	-	1044	0	14	1042	7	442	
QEN71_RS35365	3-keto-5-aminohexanoate cleavage protein	QEN71_35355	paras_007988	protein-codi	NZ_CP1252	chromosom	1288739	1289656	+	918	0	17	1504	15	1213	
QEN71_RS35370	DUF4268 domain-containing protein	QEN71_35360	paras_007989	protein-codi	NZ_CP1252	chromosom	1289719	1290942	+	1224	0	20	3260	13	1129	
QEN71_RS35375	hypothetical protein	QEN71_35365	paras_007990	protein-codi	NZ_CP1252	chromosom	1290959	1291828	-	847	0	18	2635	14	2184	
QEN71_RS35380	chromosome partitioning protein ParA	QEN71_35370	paras_007991	protein-codi	NZ_CP1252	chromosom	1291806	1294535	-	2703	0	46	6938	30	3151	
QEN71_RS35385	hypothetical protein	QEN71_35375	paras_007992	protein-codi	NZ_CP1252	chromosom	1294532	1295083	-	534	0	15	2525	14	2522	
QEN71_RS35390	hypothetical protein	QEN71_35380	paras_007993	protein-codi	NZ_CP1252	chromosom	1295070	1296302	-	1219	0	39	5544	32	4480	
QEN71_RS35395	alkaline phosphatase family protein	QEN71_35385	paras_007994	protein-codi	NZ_CP1252	chromosom	1296710	1298110	-	1401	0	32	5455	28	5211	
QEN71_RS35400	SulP family inorganic anion transporter	QEN71_35390	paras_007995	protein-codi	NZ_CP1252	chromosom	1298535	1300094	-	1560	226.0	14	2027	13	2022	
QEN71_RS35405	carbonic anhydrase	QEN71_35395	paras_007996	protein-codi	NZ_CP1252	chromosom	1300198	1300863	-	666	141.0	11	804	11	804	
QEN71_RS35410	DUF2059 domain-containing protein	partial;pseudo;QEN71_35400		pseudogene	NZ_CP1252	chromosom	1301962	1302567	-	606	0	14	2461	12	2432	
QEN71_RS35415	hypothetical protein	QEN71_35405	paras_007998	protein-codi	NZ_CP1252	chromosom	1302608	1303129	-	522	0	17	2723	15	2437	
QEN71_RS35420	hypothetical protein	QEN71_35410	paras_007999	protein-codi	NZ_CP1252	chromosom	1303420	1303602	+	183	0	3	870	0	0	
QEN71_RS35425	cytosine permease	QEN71_35415	paras_008000	protein-codi	NZ_CP1252	chromosom	1303932	1305467	-	1536	0	39	3867	29	2985	
QEN71_RS35430	nuclear transport factor 2 family protein	QEN71_35420	paras_008001	protein-codi	NZ_CP1252	chromosom	1305705	1306109	+	405	0	7	972	5	413	
QEN71_RS35435	ShlB/FhaC/HecB family hemolysin secretion/activat	QEN71_35425	paras_008002	protein-codi	NZ_CP1252	chromosom	1307003	1308763	+	1757	0	56	10476	46	9256	
QEN71_RS35440	hemagglutinin repeat-containing protein	QEN71_35430	paras_008003	protein-codi	NZ_CP1252	chromosom	1308760	1318779	+	10012	0	261	48897	199	36678	
QEN71_RS35445	PoNi-like cognate immunity protein	QEN71_35435	paras_008004	protein-codi	NZ_CP1252	chromosom	1318776	1319666	+	887	0	30	1410	26	1264	
QEN71_RS35450	hypothetical protein	QEN71_35440	paras_008005	protein-codi	NZ_CP1252	chromosom	1320156	1320707	-	552	0	8	1556	8	1556	
QEN71_RS35455	hypothetical protein	QEN71_35445	paras_008006	protein-codi	NZ_CP1252	chromosom	1320874	1321353	+	480	0	13	2053	13	2053	
QEN71_RS35460	nicotinate phosphoribosyltransferase	QEN71_35450	paras_008007	protein-codi	NZ_CP1252	chromosom	1321941	1323662	-	1722	0	54	11285	42	9379	
QEN71_RS35465	ribose-phosphate diphosphokinase	QEN71_35455	paras_008008	protein-codi	NZ_CP1252	chromosom	1323689	1324504	-	816	0	18	4083	16	3970	
QEN71_RS35470	NUDIX hydrolase	QEN71_35460	paras_008009	protein-codi	NZ_CP1252	chromosom	1324631	1325338	+	708	0	18	2767	13	2273	
QEN71_RS35475	SMI1/KNR4 family protein	QEN71_35465	paras_008011	protein-codi	NZ_CP1252	chromosom	1325908	1326297	+	390	0	19	1862	14	1547	
QEN71_RS35480	DUF1910 domain-containing protein	partial;pseudo;QEN71_35470		pseudogene	NZ_CP1252	chromosom	1326624	1326806	+	183	0	8	1450	8	1450	
QEN71_RS35485	DUF1911 domain-containing protein	QEN71_35475	paras_008012	protein-codi	NZ_CP1252	chromosom	1326857	1327510	+	654	0	15	1578	13	1546	
QEN71_RS35490	tyrosine-type recombinase/integrase	QEN71_35480	paras_008013	protein-codi	NZ_CP1252	chromosom	1327660	1328928	-	1269	0	37	5804	23	2271	
QEN71_RS35495	DNA-binding protein	QEN71_35485	paras_008014	protein-codi	NZ_CP1252	chromosom	1329273	1330301	+	1029	0	11	2165	4	631	
QEN71_RS35500	P-loop NTPase fold protein	QEN71_35490	paras_008015	protein-codi	NZ_CP1252	chromosom	1330853	1334929	+	4077	0	152	37228	117	26191	
QEN71_RS35505	peptide ligase PGM1-related protein	QEN71_35495	paras_008016	protein-codi	NZ_CP1252	chromosom	1335426	1336745	+	1320	0	54	9173	51	8629	
QEN71_RS35510	DMT family transporter	QEN71_35500	paras_008017	protein-codi	NZ_CP1252	chromosom	1336773	1337687	+	915	0	28	3792	26	3778	
QEN71_RS35515	amino acid racemase	QEN71_35505	paras_008018	protein-codi	NZ_CP1252	chromosom	1337740	1338504	+	754	0	11	1046	10	1027	
QEN71_RS35520	serine hydroxymethyltransferase	QEN71_35510	paras_008019	protein-codi	NZ_CP1252	chromosom	1338494	1339843	+	1339	0	27	3831	23	3329	
QEN71_RS35525	very short patch repair endonuclease	QEN71_35515	paras_008020	protein-codi	NZ_CP1252	chromosom	1340307	1340708	-	400	0	7	1537	7	1537	
QEN71_RS35530	DNA cytosine methyltransferase	QEN71_35520	paras_008021	protein-codi	NZ_CP1252	chromosom	1340707	1341984	+	1276	0	41	9078	25	5860	
QEN71_RS35535	hypothetical protein	QEN71_35525	paras_008022	protein-codi	NZ_CP1252	chromosom	1342006	1344549	+	2544	0	72	15750	48	12202	
QEN71_RS35540	hypothetical protein	QEN71_35530	paras_008023	protein-codi	NZ_CP1252	chromosom	1344560	1346632	+	2073	0	40	7575	23	4857	
QEN71_RS35545	DEAD/DEAH box helicase	QEN71_35535	paras_008024	protein-codi	NZ_CP1252	chromosom	1346659	1352550	+	5892	0	159	28275	118	22042	
QEN71_RS35550	VOC family protein	QEN71_35540	paras_008025	protein-codi	NZ_CP1252	chromosom	1353124	1353600	+	477	0	21	3187	13	1249	
QEN71_RS35555	winged helix-turn-helix domain-containing protein	QEN71_35545	paras_008026	protein-codi	NZ_CP1252	chromosom	1354097	1354816	+	720	0	19	5351	11	2573	
QEN71_RS35560	IclR family transcriptional regulator	QEN71_35550	paras_008027	protein-codi	NZ_CP1252	chromosom	1354967	1355773	-	807	0	28	6134	21	5324	

QEN71_RS35565	ABC transporter substrate-binding protein	QEN71_35555	paras_008028	protein-codi	NZ_CP1252	chromosom	1355938	1356708	+	771	0	19	3615	17	3560	
QEN71_RS35570	amino acid ABC transporter permease	QEN71_35560	paras_008029	protein-codi	NZ_CP1252	chromosom	1356791	1357447	+	653	0	17	3958	17	3958	
QEN71_RS35575	amino acid ABC transporter ATP-binding protein	QEN71_35565	paras_008030	protein-codi	NZ_CP1252	chromosom	1357444	1358202	+	755	0	8	975	8	975	
QEN71_RS35580	FAD-binding oxidoreductase	QEN71_35570	paras_008031	protein-codi	NZ_CP1252	chromosom	1358205	1359500	+	1296	0	38	9000	35	8512	
QEN71_RS35585	hypothetical protein	QEN71_35575	paras_008032	protein-codi	NZ_CP1252	chromosom	1360154	1360735	+	582	0	16	3187	13	2648	
QEN71_RS35590	hypothetical protein	QEN71_35580	paras_008033	protein-codi	NZ_CP1252	chromosom	1360921	1361694	-	763	0	11	2374	9	2072	
QEN71_RS35595	hypothetical protein	QEN71_35585	paras_008034	protein-codi	NZ_CP1252	chromosom	1361684	1362019	-	325	0	5	868	4	784	
QEN71_RS35600	hypothetical protein	QEN71_35590	paras_008035	protein-codi	NZ_CP1252	chromosom	1362716	1363828	+	1113	0	26	5480	24	5224	
QEN71_RS35605	DUF6434 domain-containing protein	QEN71_35595	paras_008036	protein-codi	NZ_CP1252	chromosom	1363994	1364218	+	225	0	7	894	5	611	
QEN71_RS35610	DUF2262 domain-containing protein	QEN71_35600	paras_008037	protein-codi	NZ_CP1252	chromosom	1364349	1365179	+	831	0	36	7435	27	4353	
QEN71_RS35615	DUF4209 domain-containing protein	QEN71_35605	paras_008038	protein-codi	NZ_CP1252	chromosom	1365401	1367299	+	1899	0	57	10587	43	8398	
QEN71_RS35620	HNH endonuclease	QEN71_35610	paras_008039	protein-codi	NZ_CP1252	chromosom	1367461	1368342	+	882	0	42	7220	29	5055	
QEN71_RS35625	amino acid permease	QEN71_35615	paras_008040	protein-codi	NZ_CP1252	chromosom	1368509	1370038	-	1530	0	35	9129	28	7522	
QEN71_RS35630	SDR family oxidoreductase	QEN71_35620	paras_008041	protein-codi	NZ_CP1252	chromosom	1370106	1370882	-	777	0	16	2824	13	2680	
QEN71_RS35635	helix-turn-helix domain-containing protein	QEN71_35625	paras_008042	protein-codi	NZ_CP1252	chromosom	1371113	1372111	+	999	0	16	3536	7	1640	
QEN71_RS35640	HNH endonuclease	QEN71_35630	paras_008043	protein-codi	NZ_CP1252	chromosom	1372486	1373568	-	1083	0	47	14480	37	11775	
QEN71_RS35645	TetR/AcrR family transcriptional regulator	QEN71_35635	paras_008044	protein-codi	NZ_CP1252	chromosom	1373571	1374251	-	681	0	32	6009	26	4171	
QEN71_RS35650	cyclase family protein	QEN71_35640	paras_008045	protein-codi	NZ_CP1252	chromosom	1374505	1375311	+	807	0	27	5383	26	5344	
QEN71_RS35655	fumarylacetoacetase	QEN71_35645	paras_008046	protein-codi	NZ_CP1252	chromosom	1375337	1376650	+	1314	0	49	14144	40	9408	
QEN71_RS35660	IS21 family transposase	QEN71_35650	paras_008047	protein-codi	NZ_CP1252	chromosom	1377002	1378525	+	1524	3027.0	8	147	7	145	
QEN71_RS35665	IS21-like element helper ATPase IstB	QEN71_35655	paras_008048	protein-codi	NZ_CP1252	chromosom	1378532	1379320	+	789	1561.0	4	345	4	345	
QEN71_RS35670	alpha/beta hydrolase	QEN71_35660	paras_008049	protein-codi	NZ_CP1252	chromosom	1380089	1380793	-	705	0	17	942	9	698	
QEN71_RS35675	MFS transporter	QEN71_35665	paras_008050	protein-codi	NZ_CP1252	chromosom	1380941	1382185	-	1245	0	20	699	19	692	
QEN71_RS35680	porin	QEN71_35670	paras_008051	protein-codi	NZ_CP1252	chromosom	1382338	1383435	-	1098	0	20	876	17	740	
QEN71_RS35685	CocE/NonD family hydrolase	QEN71_35675	paras_008052	protein-codi	NZ_CP1252	chromosom	1384437	1386170	+	1734	0	44	3163	30	1939	
QEN71_RS35690	acyl-CoA synthetase	QEN71_35680	paras_008053	protein-codi	NZ_CP1252	chromosom	1386631	1388271	+	1641	0	47	3544	34	1881	
QEN71_RS35695	TauD/TfdA family dioxygenase	QEN71_35685	paras_008054	protein-codi	NZ_CP1252	chromosom	1388363	1389466	-	1104	0	27	1890	25	1876	
QEN71_RS35700	MFS transporter	QEN71_35690	paras_008055	protein-codi	NZ_CP1252	chromosom	1389727	1391127	-	1401	0	27	1689	23	1353	
QEN71_RS35705	altronate dehydratase family protein	QEN71_35695	paras_008056	protein-codi	NZ_CP1252	chromosom	1391327	1392856	-	1530	0	14	1553	11	988	
QEN71_RS35710	FAD-dependent oxidoreductase	QEN71_35700	paras_008057	protein-codi	NZ_CP1252	chromosom	1393287	1394654	+	1368	0	30	2913	24	2324	
QEN71_RS35715	dihydroxy-acid dehydratase	QEN71_35705	paras_008058	protein-codi	NZ_CP1252	chromosom	1394760	1396439	+	1680	0	41	7801	24	3383	
QEN71_RS35720	LacI family DNA-binding transcriptional regulator	QEN71_35710	paras_008059	protein-codi	NZ_CP1252	chromosom	1396600	1397682	+	1083	0	21	3266	17	2249	
QEN71_RS35725	hypothetical protein	QEN71_35715	paras_008060	protein-codi	NZ_CP1252	chromosom	1397722	1398099	-	378	0	10	1136	8	1045	
QEN71_RS35730	substrate-binding domain-containing protein	QEN71_35720	paras_008061	protein-codi	NZ_CP1252	chromosom	1398219	1399163	-	945	0	13	1321	10	1223	
QEN71_RS35735	NADP-dependent glyceraldehyde-3-phosphate deh	QEN71_35725	paras_008062	protein-codi	NZ_CP1252	chromosom	1399315	1400904	-	1590	0	25	1530	22	1457	
QEN71_RS35740	MFS transporter	QEN71_35730	paras_008063	protein-codi	NZ_CP1252	chromosom	1401014	1402291	-	1278	0	24	1739	17	1173	
QEN71_RS35745	SDR family NAD(P)-dependent oxidoreductase	QEN71_35735	paras_008064	protein-codi	NZ_CP1252	chromosom	1402487	1403173	-	683	0	12	1498	8	1314	
QEN71_RS35750	Gfo/Idh/MocA family oxidoreductase	QEN71_35740	paras_008065	protein-codi	NZ_CP1252	chromosom	1403170	1404156	-	983	0	11	1001	6	463	
QEN71_RS35755	sugar kinase	QEN71_35745	paras_008066	protein-codi	NZ_CP1252	chromosom	1404202	1405158	-	957	0	19	1072	16	847	
QEN71_RS35760	glycoside hydrolase family 88 protein	QEN71_35750	paras_008067	protein-codi	NZ_CP1252	chromosom	1405255	1406367	-	1113	0	18	1427	14	919	
QEN71_RS35765	CaiB/BaiF CoA-transferase family protein	QEN71_35755	paras_008068	protein-codi	NZ_CP1252	chromosom	1406385	1407659	-	1271	0	28	1644	23	1457	
QEN71_RS35770	ABC transporter permease	QEN71_35760	paras_008069	protein-codi	NZ_CP1252	chromosom	1407656	1408642	-	983	0	20	1635	12	931	
QEN71_RS35775	HAD family hydrolase	QEN71_35765	paras_008070	protein-codi	NZ_CP1252	chromosom	1408673	1409491	-	815	0	21	2411	13	1005	
QEN71_RS35780	aldehyde dehydrogenase family protein	QEN71_35770	paras_008071	protein-codi	NZ_CP1252	chromosom	1409488	1410972	-	1481	0	15	1078	13	950	
QEN71_RS35785	sugar ABC transporter ATP-binding protein	QEN71_35775	paras_008072	protein-codi	NZ_CP1252	chromosom	1410983	1412476	-	1494	0	36	5133	28	4473	
QEN71_RS35790	LacI family DNA-binding transcriptional regulator	QEN71_35780	paras_008073	protein-codi	NZ_CP1252	chromosom	1412728	1413822	+	1095	0	34	5198	30	4911	
QEN71_RS35795	GMC family oxidoreductase N-terminal domain-cont	QEN71_35785	paras_008074	protein-codi	NZ_CP1252	chromosom	1414006	1415649	-	1644	0	75	20396	58	13411	
QEN71_RS35800	MaoC family dehydratase	QEN71_35790	paras_008075	protein-codi	NZ_CP1252	chromosom	1415657	1416112	-	456	0	22	5132	16	3524	
QEN71_RS35805	substrate-binding domain-containing protein	QEN71_35795	paras_008076	protein-codi	NZ_CP1252	chromosom	1416172	1417116	-	945	0	20	5346	12	3006	
QEN71_RS35810	CaiB/BaiF CoA-transferase family protein	QEN71_35800	paras_008077	protein-codi	NZ_CP1252	chromosom	1417173	1418411	-	1239	0	31	6612	24	5479	
QEN71_RS35815	hypothetical protein	QEN71_35805	paras_008078	protein-codi	NZ_CP1252	chromosom	1418577	1418837	+	261	0	3	471	0	0	
QEN71_RS35820	MFS transporter	QEN71_35810	paras_008079	protein-codi	NZ_CP1252	chromosom	1419023	1420300	-	1278	0	34	2486	27	2220	
QEN71_RS35825	shikimate dehydrogenase	QEN71_35815	paras_008080	protein-codi	NZ_CP1252	chromosom	1420563	1421378	-	816	0	16	1110	14	1019	
QEN71_RS35830	YciI family protein	QEN71_35820	paras_008081	protein-codi	NZ_CP1252	chromosom	1421391	1421714	-	324	0	8	424	6	310	
QEN71_RS35835	CoA transferase	QEN71_35825	paras_008082	protein-codi	NZ_CP1252	chromosom	1421716	1422921	-	1206	0	19	1696	18	1538	
QEN71_RS35840	enoyl-CoA hydratase-related protein	QEN71_35830	paras_008083	protein-codi	NZ_CP1252	chromosom	1422959	1423750	-	792	0	15	948	12	672	
QEN71_RS35845	dihydrodipicolinate synthase family protein	QEN71_35835	paras_008084	protein-codi	NZ_CP1252	chromosom	1423758	1424732	-	975	0	16	1600	15	1474	
QEN71_RS35850	LacI family DNA-binding transcriptional regulator	QEN71_35840	paras_008085	protein-codi	NZ_CP1252	chromosom	1425023	1426078	-	1056	0	55	10819	48	10215	
QEN71_RS35855	TetR/AcrR family transcriptional regulator	QEN71_35845	paras_008086	protein-codi	NZ_CP1252	chromosom	1426241	1426807	+	567	0	13	1658	10	1035	
QEN71_RS35860	hypothetical protein	QEN71_35850	paras_008087	protein-codi	NZ_CP1252	chromosom	1426855	1427541	+	687	0	28	3257	22	2901	
QEN71_RS35865	SDR family oxidoreductase	QEN71_35855	paras_008088	protein-codi	NZ_CP1252	chromosom	1427603	1428475	+	873	0	24	4420	22	4348	
QEN71_RS35870	ISNCY family transposase	partial;pseudo;QEN71_35860		pseudogene	NZ_CP1252	chromosom	1429582	1429767	+	186	0	4	1337	4	1337	

QEN71_RS35875	cold-shock protein	QEN71_35865	paras_008090	protein-codi	NZ_CP1252	chromosome	1430037	1430240	-	204	0	2	139	1	122	
QEN71_RS35880	ABC transporter substrate-binding protein	QEN71_35870	paras_008091	protein-codi	NZ_CP1252	chromosome	1430801	1431832	+	1031	0	31	8805	30	8595	
QEN71_RS35885	hypothetical protein	QEN71_35875	paras_008092	protein-codi	NZ_CP1252	chromosome	1431832	1432260	+	428	0	10	1621	5	544	
QEN71_RS35890	PAS domain S-box protein	QEN71_35880	paras_008093	protein-codi	NZ_CP1252	chromosome	1432416	1433690	+	1275	0	20	1793	18	1625	
QEN71_RS35895	dihydrodipicolinate synthase family protein	QEN71_35885	paras_008094	protein-codi	NZ_CP1252	chromosome	1433823	1434770	-	948	0	18	3998	16	3816	
QEN71_RS35900	MFS transporter	QEN71_35890	paras_008095	protein-codi	NZ_CP1252	chromosome	1434832	1436154	-	1323	0	24	2727	20	2457	
QEN71_RS35905	ribokinase	QEN71_35895	paras_008096	protein-codi	NZ_CP1252	chromosome	1436244	1437182	-	939	0	9	2062	7	1685	
QEN71_RS35910	LacI family DNA-binding transcriptional regulator	QEN71_35900	paras_008097	protein-codi	NZ_CP1252	chromosome	1437572	1438651	+	1080	0	32	5308	30	4978	
QEN71_RS35915	branched-chain amino acid ABC transporter substra	QEN71_35905	paras_008098	protein-codi	NZ_CP1252	chromosome	1438673	1439770	-	1098	0	29	4982	25	4408	
QEN71_RS35920	amidase	QEN71_35910	paras_008099	protein-codi	NZ_CP1252	chromosome	1439948	1441303	-	1356	0	22	2561	17	1610	
QEN71_RS35925	DUF2848 domain-containing protein	QEN71_35915	paras_008100	protein-codi	NZ_CP1252	chromosome	1441309	1441992	-	684	0	13	1143	6	521	
QEN71_RS35930	LysR family transcriptional regulator	QEN71_35920	paras_008101	protein-codi	NZ_CP1252	chromosome	1442179	1443123	+	945	0	9	819	9	819	
QEN71_RS35935	ureidoglycolate lyase	QEN71_35925	paras_008102	protein-codi	NZ_CP1252	chromosome	1443155	1443715	+	561	0	8	791	7	786	
QEN71_RS35940	DUF3455 domain-containing protein	QEN71_35930	paras_008103	protein-codi	NZ_CP1252	chromosome	1444019	1444477	+	459	0	14	2108	10	1782	
QEN71_RS35945	hypothetical protein	QEN71_35935	paras_008104	protein-codi	NZ_CP1252	chromosome	1445808	1446170	+	363	0	3	218	3	218	
QEN71_RS35950	GGDEF domain-containing protein	QEN71_35940	paras_008105	protein-codi	NZ_CP1252	chromosome	1446197	1447360	-	1164	0	31	2447	27	2164	
QEN71_RS35955	porin	QEN71_35945	paras_008106	protein-codi	NZ_CP1252	chromosome	1447419	1448717	-	1299	0	45	6841	32	4891	
QEN71_RS35960	hypothetical protein	QEN71_35950	paras_008107	protein-codi	NZ_CP1252	chromosome	1448924	1449718	+	795	0	26	7771	19	5899	
QEN71_RS35965	EAL domain-containing protein	QEN71_35955	paras_008108	protein-codi	NZ_CP1252	chromosome	1449844	1452789	+	2946	0	64	16766	56	15519	
QEN71_RS35970	sensor domain-containing diguanylate cyclase	QEN71_35960	paras_008109	protein-codi	NZ_CP1252	chromosome	1452797	1454764	-	1968	0	58	10187	52	6960	
QEN71_RS35975	GntR family transcriptional regulator	QEN71_35965	paras_008110	protein-codi	NZ_CP1252	chromosome	1455522	1456214	-	693	0	17	2430	11	914	
QEN71_RS35980	hypothetical protein	QEN71_35970	paras_008111	protein-codi	NZ_CP1252	chromosome	1456323	1456874	-	552	0	10	1797	8	1418	
QEN71_RS35985	Rieske 2Fe-2S domain-containing protein	QEN71_35975	paras_008112	protein-codi	NZ_CP1252	chromosome	1457408	1458955	+	1548	0	34	4276	32	4231	
QEN71_RS35990	PDR/VanB family oxidoreductase	QEN71_35980	paras_008113	protein-codi	NZ_CP1252	chromosome	1459046	1460056	+	1011	0	14	1330	12	1185	
QEN71_RS35995	ketol-acid reductoisomerase	QEN71_35985	paras_008114	protein-codi	NZ_CP1252	chromosome	1460080	1461093	+	1014	0	16	957	13	864	
QEN71_RS36000	GntR family transcriptional regulator	QEN71_35990	paras_008115	protein-codi	NZ_CP1252	chromosome	1461173	1461886	+	714	0	9	435	7	232	
QEN71_RS36005	class II aldolase/adducin family protein	QEN71_35995	paras_008116	protein-codi	NZ_CP1252	chromosome	1462035	1462889	+	855	0	12	462	7	154	
QEN71_RS36010	MFS transporter	QEN71_36000	paras_008117	protein-codi	NZ_CP1252	chromosome	1463017	1464324	+	1308	0	18	495	12	336	
QEN71_RS36015	LysR family transcriptional regulator	QEN71_36005	paras_008118	protein-codi	NZ_CP1252	chromosome	1464462	1465397	+	936	0	15	706	10	533	
QEN71_RS36020	choline dehydrogenase	QEN71_36010	paras_008119	protein-codi	NZ_CP1252	chromosome	1465492	1467126	-	1635	0	14	1075	12	743	
QEN71_RS36025	cupin domain-containing protein	QEN71_36015	paras_008120	protein-codi	NZ_CP1252	chromosome	1467129	1467428	-	300	0	4	250	4	250	
QEN71_RS36030	glycine betaine ABC transporter substrate-binding p	QEN71_36020	paras_008121	protein-codi	NZ_CP1252	chromosome	1467480	1468343	-	864	0	5	588	5	588	
QEN71_RS36035	Xaa-Pro peptidase family protein	QEN71_36025	paras_008122	protein-codi	NZ_CP1252	chromosome	1468399	1469655	-	1257	0	16	985	10	414	
QEN71_RS36040	LysR substrate-binding domain-containing protein	QEN71_36030	paras_008123	protein-codi	NZ_CP1252	chromosome	1469813	1470748	+	936	0	15	985	14	934	
QEN71_RS36045	DAK2 domain-containing protein	QEN71_36035	paras_008124	protein-codi	NZ_CP1252	chromosome	1471388	1472011	-	624	0	4	270	3	247	
QEN71_RS36050	dihydroxyacetone kinase subunit DhaK	QEN71_36040	paras_008125	protein-codi	NZ_CP1252	chromosome	1472037	1473035	-	999	0	14	1805	12	1695	
QEN71_RS36055	ABC transporter permease	QEN71_36045	paras_008126	protein-codi	NZ_CP1252	chromosome	1473055	1474056	-	998	0	8	993	8	993	
QEN71_RS36060	sugar ABC transporter ATP-binding protein	QEN71_36050	paras_008127	protein-codi	NZ_CP1252	chromosome	1474053	1475615	-	1559	0	15	1521	13	1300	
QEN71_RS36065	sugar ABC transporter substrate-binding protein	QEN71_36055	paras_008128	protein-codi	NZ_CP1252	chromosome	1475674	1476783	-	1110	0	10	413	7	221	
QEN71_RS36070	class II aldolase/adducin family protein	QEN71_36060	paras_008129	protein-codi	NZ_CP1252	chromosome	1476878	1477552	-	675	0	6	575	6	575	
QEN71_RS36075	S-methyl-5-thioribose-1-phosphate isomerase	QEN71_36065	paras_008130	protein-codi	NZ_CP1252	chromosome	1477555	1478643	-	1089	0	9	668	8	627	
QEN71_RS36080	S-methyl-5-thioribose kinase	QEN71_36070	paras_008131	protein-codi	NZ_CP1252	chromosome	1478682	1479950	-	1269	0	15	1329	11	1315	
QEN71_RS36085	GntR family transcriptional regulator	QEN71_36075	paras_008132	protein-codi	NZ_CP1252	chromosome	1479979	1480677	-	699	0	4	62	2	15	
QEN71_RS36090	hypothetical protein	QEN71_36080	paras_008133	protein-codi	NZ_CP1252	chromosome	1480766	1480960	-	195	0	1	84	1	84	
QEN71_RS36095	filamentous hemagglutinin N-terminal domain-conta	QEN71_36085	paras_008134	protein-codi	NZ_CP1252	chromosome	1481232	1483382	-	2151	0	16	1464	13	1226	
QEN71_RS36100	response regulator	QEN71_36090	paras_008135	protein-codi	NZ_CP1252	chromosome	1483707	1485851	-	2145	0	25	2116	20	1610	
QEN71_RS36105	HAMP domain-containing protein	QEN71_36095	paras_008136	protein-codi	NZ_CP1252	chromosome	1485861	1491908	-	6048	0	37	2356	29	1404	
QEN71_RS36110	aldo/keto reductase	QEN71_36100	paras_008137	protein-codi	NZ_CP1252	chromosome	1492310	1493326	-	1017	0	15	1170	12	736	
QEN71_RS36115	ABC transporter substrate-binding protein	QEN71_36105	paras_008138	protein-codi	NZ_CP1252	chromosome	1493381	1494628	-	1248	0	21	1233	18	1089	
QEN71_RS36120	DUF1479 domain-containing protein	QEN71_36110	paras_008139	protein-codi	NZ_CP1252	chromosome	1494706	1495950	-	1245	0	21	2188	19	1834	
QEN71_RS36125	helix-turn-helix domain-containing protein	QEN71_36115	paras_008140	protein-codi	NZ_CP1252	chromosome	1496044	1497036	+	986	0	19	2209	17	2111	
QEN71_RS36130	MBL fold metallo-hydrolase	QEN71_36120	paras_008141	protein-codi	NZ_CP1252	chromosome	1497030	1497962	-	926	0	5	262	5	262	
QEN71_RS36135	SDR family oxidoreductase	QEN71_36125	paras_008142	protein-codi	NZ_CP1252	chromosome	1498211	1499002	-	792	0	6	364	2	18	TRUE
QEN71_RS36140	TRAP transporter large permease	QEN71_36130	paras_008143	protein-codi	NZ_CP1252	chromosome	1499088	1500395	-	1304	0	17	1253	15	1183	
QEN71_RS36145	TRAP transporter small permease	QEN71_36135	paras_008144	protein-codi	NZ_CP1252	chromosome	1500392	1500940	-	545	0	14	871	14	871	
QEN71_RS36150	TRAP transporter substrate-binding protein DctP	QEN71_36140	paras_008145	protein-codi	NZ_CP1252	chromosome	1500950	1501969	-	1020	0	29	2679	26	2292	
QEN71_RS36155	GTP-binding protein	QEN71_36145	paras_008146	protein-codi	NZ_CP1252	chromosome	1502028	1502942	-	911	0	10	469	5	138	
QEN71_RS36160	CoA transferase	QEN71_36150	paras_008147	protein-codi	NZ_CP1252	chromosome	1502939	1504114	-	1168	0	16	1540	12	1142	
QEN71_RS36165	cobalamin B12-binding domain-containing protein	QEN71_36155	paras_008148	protein-codi	NZ_CP1252	chromosome	1504111	1504554	-	436	0	3	401	3	401	
QEN71_RS36170	methylmalonyl-CoA mutase family protein	QEN71_36160	paras_008149	protein-codi	NZ_CP1252	chromosome	1504551	1506170	-	1616	0	20	3120	17	2935	
QEN71_RS36175	enoyl-CoA hydratase/isomerase family protein	QEN71_36165	paras_008150	protein-codi	NZ_CP1252	chromosome	1506189	1507010	-	821	0	12	605	12	605	
QEN71_RS36180	IclR family transcriptional regulator	QEN71_36170	paras_008151	protein-codi	NZ_CP1252	chromosome	1507010	1507741	-	731	0	13	1529	13	1529	

QEN71_RS36185	MFS transporter	QEN71_36175	paras_008152	protein-codi	NZ_CP1252	chromosom	1508105	1509391	+	1287	0	37	8369	25	3925	
QEN71_RS36190	amidohydrolase family protein	QEN71_36180	paras_008153	protein-codi	NZ_CP1252	chromosom	1509417	1510286	+	870	0	32	5074	26	4469	
QEN71_RS36195	class II aldolase/adducin family protein	QEN71_36185	paras_008154	protein-codi	NZ_CP1252	chromosom	1510318	1511097	+	780	0	37	7430	31	7137	
QEN71_RS36200	hypothetical protein	QEN71_36190	paras_008155	protein-codi	NZ_CP1252	chromosom	1511110	1511247	-	138	0	3	1080	1	25	
QEN71_RS36205	flagellin domain-containing protein	QEN71_36195	paras_008156	protein-codi	NZ_CP1252	chromosom	1511569	1512387	-	819	463.0	5	608	2	531	
QEN71_RS36210	hypothetical protein	QEN71_36200	paras_008157	protein-codi	NZ_CP1252	chromosom	1512618	1512872	-	255	0	5	314	5	314	
QEN71_RS36215	TetR/AcrR family transcriptional regulator	QEN71_36205	paras_008158	protein-codi	NZ_CP1252	chromosom	1513133	1513675	+	543	0	7	504	7	504	
QEN71_RS36220	glycoside hydrolase family 19 protein	QEN71_36210	paras_008159	protein-codi	NZ_CP1252	chromosom	1513808	1514497	+	690	0	17	2478	11	1229	
QEN71_RS36225	hypothetical protein	QEN71_36215	paras_008160	protein-codi	NZ_CP1252	chromosom	1514519	1514950	-	432	0	8	1231	6	669	
QEN71_RS36230	hypothetical protein	QEN71_36220	paras_008161	protein-codi	NZ_CP1252	chromosom	1515045	1515620	-	576	0	14	1097	11	669	
QEN71_RS36235	diaminopropionate ammonia-lyase	QEN71_36225	paras_008162	protein-codi	NZ_CP1252	chromosom	1515738	1516955	-	1218	0	20	1492	16	1268	
QEN71_RS36240	M20 aminoacylase family protein	QEN71_36230	paras_008163	protein-codi	NZ_CP1252	chromosom	1517007	1518182	-	1176	0	14	2977	10	2840	
QEN71_RS36245	hypothetical protein	QEN71_36235	paras_008164	protein-codi	NZ_CP1252	chromosom	1518319	1518456	+	138	0	5	415	5	415	
QEN71_RS36250	Lrp/AsnC family transcriptional regulator	QEN71_36240	paras_008165	protein-codi	NZ_CP1252	chromosom	1518790	1519269	-	480	0	26	3810	20	3079	
QEN71_RS36255	hypothetical protein	QEN71_36245	paras_008166	protein-codi	NZ_CP1252	chromosom	1519469	1519963	+	495	0	13	1915	9	1293	
QEN71_RS36260	single-stranded DNA-binding protein	QEN71_36250	paras_008167	protein-codi	NZ_CP1252	chromosom	1520174	1520491	-	318	0	7	1528	4	1263	
QEN71_RS36265	LysR family transcriptional regulator	partial;pseudo;QEN71_36255		pseudogene	NZ_CP1252	chromosom	1520603	1520988	-	386	0	17	2864	12	2539	
QEN71_RS36270	cytochrome c	QEN71_36260	paras_008169	protein-codi	NZ_CP1252	chromosom	1521137	1521535	-	399	0	14	890	12	839	
QEN71_RS36275	cold-shock protein	QEN71_36265	paras_008170	protein-codi	NZ_CP1252	chromosom	1521553	1521756	-	204	0	7	1088	2	163	
QEN71_RS36280	lytic transglycosylase domain-containing protein	QEN71_36270	paras_008171	protein-codi	NZ_CP1252	chromosom	1522354	1524324	+	1971	0	65	11976	45	7638	
QEN71_RS36285	translation initiation factor Sui1	QEN71_36275	paras_008172	protein-codi	NZ_CP1252	chromosom	1524763	1525125	-	363	0	12	969	7	543	
QEN71_RS36290	site-specific integrase	QEN71_36280	paras_008173	protein-codi	NZ_CP1252	chromosom	1525352	1527058	+	1707	0	21	4313	20	4197	
QEN71_RS36295	DUF1488 family protein	pseudo;QEN71_36285		pseudogene	NZ_CP1252	chromosom	1527173	1527441	+	269	0	3	1170	1	49	
QEN71_RS36300	HU family DNA-binding protein	QEN71_36290	paras_008175	protein-codi	NZ_CP1252	chromosom	1527558	1527836	-	279	0	6	757	4	94	
QEN71_RS36305	IS21-like element helper ATPase IstB	QEN71_36295	paras_008176	protein-codi	NZ_CP1252	chromosom	1528229	1529017	-	789	1561.0	7	37	6	35	
QEN71_RS36310	IS21 family transposase	QEN71_36300	paras_008177	protein-codi	NZ_CP1252	chromosom	1529024	1530547	-	1524	3027.0	9	351	6	198	
QEN71_RS36315	SET domain-containing protein-lysine N-methyltrans	partial;pseudo;QEN71_36305		pseudogene	NZ_CP1252	chromosom	1530832	1531017	+	186	0	2	245	2	245	
QEN71_RS36320	GlxA family transcriptional regulator	QEN71_36310	paras_008178	protein-codi	NZ_CP1252	chromosom	1531188	1532090	-	903	0	32	3940	28	3538	
QEN71_RS36325	hybrid-cluster NAD(P)-dependent oxidoreductase	QEN71_36315	paras_008179	protein-codi	NZ_CP1252	chromosom	1532186	1533328	-	1143	0	24	1890	19	1342	
QEN71_RS36330	aromatic ring-hydroxylating dioxygenase subunit alp	QEN71_36320	paras_008180	protein-codi	NZ_CP1252	chromosom	1533334	1534596	-	1263	0	16	1156	14	1033	
QEN71_RS36335	LysR substrate-binding domain-containing protein	QEN71_36325	paras_008181	protein-codi	NZ_CP1252	chromosom	1534847	1535776	+	930	0	14	1575	12	1384	
QEN71_RS36340	Xaa-Pro peptidase family protein	QEN71_36330	paras_008182	protein-codi	NZ_CP1252	chromosom	1535924	1537156	+	1233	0	26	2430	22	2217	
QEN71_RS36345	RidA family protein	QEN71_36335	paras_008183	protein-codi	NZ_CP1252	chromosom	1537216	1537605	-	390	0	6	418	5	359	
QEN71_RS36350	FAD-dependent oxidoreductase	QEN71_36340	paras_008184	protein-codi	NZ_CP1252	chromosom	1537640	1538971	-	1332	0	36	2925	29	2404	
QEN71_RS36355	LysR family transcriptional regulator	QEN71_36345	paras_008185	protein-codi	NZ_CP1252	chromosom	1539230	1540135	-	906	0	17	1705	14	1237	
QEN71_RS36360	lysine 2,3-aminomutase	QEN71_36350	paras_008186	protein-codi	NZ_CP1252	chromosom	1540328	1541734	+	1407	0	12	553	12	553	
QEN71_RS36365	ferredoxin family protein	QEN71_36355	paras_008187	protein-codi	NZ_CP1252	chromosom	1541797	1542138	+	342	0	8	441	2	176	
QEN71_RS36370	DUF1330 domain-containing protein	QEN71_36360	paras_008188	protein-codi	NZ_CP1252	chromosom	1542178	1542471	-	294	0	3	394	3	394	
QEN71_RS36375	Ldh family oxidoreductase	QEN71_36365	paras_008189	protein-codi	NZ_CP1252	chromosom	1542491	1543471	-	981	0	13	1308	8	943	
QEN71_RS36380	proline racemase family protein	QEN71_36370	paras_008190	protein-codi	NZ_CP1252	chromosom	1543554	1544606	-	1053	0	16	1644	9	1294	
QEN71_RS36385	dimethylsulfonioproprionate lyase family protein	QEN71_36375	paras_008191	protein-codi	NZ_CP1252	chromosom	1545212	1545799	+	588	0	8	1293	7	1097	
QEN71_RS36390	ABC transporter substrate-binding protein	QEN71_36380	paras_008192	protein-codi	NZ_CP1252	chromosom	1545899	1546975	-	1077	0	16	1971	12	1862	
QEN71_RS36395	TetR family transcriptional regulator C-terminal dom	QEN71_36385	paras_008193	protein-codi	NZ_CP1252	chromosom	1547558	1548322	+	765	0	13	1169	10	1107	
QEN71_RS36400	electron transfer flavoprotein-ubiquinone oxidoredu	QEN71_36390	paras_008194	protein-codi	NZ_CP1252	chromosom	1548330	1550003	-	1674	1680.0	28	2487	22	2038	
QEN71_RS36405	FAD-binding oxidoreductase	QEN71_36395	paras_008195	protein-codi	NZ_CP1252	chromosom	1550285	1551472	+	1188	0	35	3224	30	2517	
QEN71_RS36410	HutD family protein	QEN71_36400	paras_008196	protein-codi	NZ_CP1252	chromosom	1551483	1552070	+	588	0	4	153	4	153	
QEN71_RS36415	electron transfer flavoprotein subunit beta/FixA fami	QEN71_36405	paras_008197	protein-codi	NZ_CP1252	chromosom	1552096	1552845	+	750	0	6	332	2	83	TRUE
QEN71_RS36420	electron transfer flavoprotein subunit alpha/FixB fan	QEN71_36410	paras_008198	protein-codi	NZ_CP1252	chromosom	1552846	1553772	+	927	0	14	830	10	740	
QEN71_RS36425	N-formylglutamate deformylase	QEN71_36415	paras_008199	protein-codi	NZ_CP1252	chromosom	1553853	1554668	-	816	0	19	1668	19	1668	
QEN71_RS36430	bifunctional proline dehydrogenase/L-glutamate gar	QEN71_36420	paras_008200	protein-codi	NZ_CP1252	chromosom	1554777	1558544	-	3768	0	62	9523	53	8204	
QEN71_RS36435	ABC transporter permease	QEN71_36425	paras_008201	protein-codi	NZ_CP1252	chromosom	1558591	1559427	-	837	0	7	437	3	118	
QEN71_RS36440	ABC transporter permease	QEN71_36430	paras_008202	protein-codi	NZ_CP1252	chromosom	1559432	1560664	-	1233	0	21	1598	19	1443	
QEN71_RS36445	ABC transporter ATP-binding protein	QEN71_36435	paras_008203	protein-codi	NZ_CP1252	chromosom	1560962	1562038	-	1077	0	15	878	14	837	
QEN71_RS36450	L-glutamate gamma-semialdehyde dehydrogenase	QEN71_36440	paras_008204	protein-codi	NZ_CP1252	chromosom	1562070	1563680	-	1611	0	29	3408	22	2165	
QEN71_RS36455	TetR family transcriptional regulator C-terminal dom	QEN71_36445	paras_008205	protein-codi	NZ_CP1252	chromosom	1564235	1564897	+	663	0	14	1927	11	1293	
QEN71_RS36460	LysR family transcriptional regulator	QEN71_36450	paras_008206	protein-codi	NZ_CP1252	chromosom	1564943	1565953	+	1011	0	16	1720	10	976	
QEN71_RS36465	N-formylglutamate amidohydrolase	QEN71_36455	paras_008207	protein-codi	NZ_CP1252	chromosom	1566003	1566869	-	867	0	18	1280	15	962	
QEN71_RS36470	pyrroline-5-carboxylate reductase	QEN71_36460	paras_008208	protein-codi	NZ_CP1252	chromosom	1567135	1567905	+	771	0	17	1417	14	1340	
QEN71_RS36475	ABC transporter substrate-binding protein	QEN71_36465	paras_008209	protein-codi	NZ_CP1252	chromosom	1567987	1569048	-	1062	0	28	1902	19	1320	
QEN71_RS36480	FAD-dependent oxidoreductase	QEN71_36470	paras_008210	protein-codi	NZ_CP1252	chromosom	1569242	1570813	-	1536	0	26	1028	22	976	
QEN71_RS36485	FAD-dependent oxidoreductase	QEN71_36475	paras_008211	protein-codi	NZ_CP1252	chromosom	1570778	1571851	+	1038	0	17	1092	14	520	
QEN71_RS36490	hypothetical protein	partial;pseudo;QEN71_36480		pseudogene	NZ_CP1252	chromosom	1572080	1572482	+	403	0	8	312	6	283	

QEN71_RS36495	ornithine cyclodeaminase	QEN71_36485	paras_008213	protein-codi	NZ_CP1252	chromosom	1572561	1573493	-	933	0	13	1719	3	210	
QEN71_RS36500	proline racemase family protein	QEN71_36490	paras_008214	protein-codi	NZ_CP1252	chromosom	1573505	1574563	-	1059	0	16	1118	14	970	
QEN71_RS36505	LysR substrate-binding domain-containing protein	QEN71_36495	paras_008215	protein-codi	NZ_CP1252	chromosom	1574664	1575638	-	975	0	16	843	15	764	
QEN71_RS36510	helix-turn-helix domain-containing protein	QEN71_36500	paras_008216	protein-codi	NZ_CP1252	chromosom	1576181	1577209	-	1029	0	27	2689	22	1647	
QEN71_RS36515	mandelate racemase/muconate lactonizing enzyme	QEN71_36505	paras_008217	protein-codi	NZ_CP1252	chromosom	1577319	1578428	+	1110	0	29	3451	19	2022	
QEN71_RS36520	aminotransferase class I/II-fold pyridoxal phosphate	QEN71_36510	paras_008218	protein-codi	NZ_CP1252	chromosom	1578529	1579083	-	555	0	13	555	6	140	
QEN71_RS36525	helix-turn-helix domain-containing protein	QEN71_36515	paras_008219	protein-codi	NZ_CP1252	chromosom	1579178	1579576	+	399	0	19	2169	14	2026	
QEN71_RS36530	Lrp/AsnC ligand binding domain-containing protein	QEN71_36520	paras_008220	pseudogene	NZ_CP1252	chromosom	1579685	1579918	+	234	0	9	657	9	657	
QEN71_RS36535	MFS transporter	QEN71_36525	paras_008221	protein-codi	NZ_CP1252	chromosom	1579939	1581168	-	1230	0	20	2726	15	2234	
QEN71_RS36540	SDR family NAD(P)-dependent oxidoreductase	QEN71_36530	paras_008222	protein-codi	NZ_CP1252	chromosom	1581280	1582059	-	780	0	12	1038	9	902	
QEN71_RS36545	transcriptional regulator GcvA	QEN71_36535	paras_008223	protein-codi	NZ_CP1252	chromosom	1582208	1583122	+	915	0	16	1948	11	1235	
QEN71_RS36550	MFS transporter	QEN71_36540	paras_008224	protein-codi	NZ_CP1252	chromosom	1583135	1584448	-	1314	0	18	1780	12	1319	
QEN71_RS36555	alpha/beta hydrolase	QEN71_36545	paras_008225	protein-codi	NZ_CP1252	chromosom	1584865	1585842	+	978	0	21	1805	21	1805	
QEN71_RS36560	MFS transporter	QEN71_36550	paras_008226	protein-codi	NZ_CP1252	chromosom	1586028	1587200	+	1173	0	19	2744	15	2203	
QEN71_RS36565	hypothetical protein	QEN71_36555	paras_008227	protein-codi	NZ_CP1252	chromosom	1587246	1587827	+	550	0	12	508	12	508	
QEN71_RS36570	hypothetical protein	QEN71_36560	paras_008228	protein-codi	NZ_CP1252	chromosom	1587796	1587975	-	148	190.0	1	2	0	0	
QEN71_RS36575	hypothetical protein	QEN71_36565	paras_008229	protein-codi	NZ_CP1252	chromosom	1588511	1588900	-	390	0	16	1274	14	989	
QEN71_RS36580	LacI family DNA-binding transcriptional regulator	QEN71_36570	paras_008230	protein-codi	NZ_CP1252	chromosom	1588968	1589972	-	1005	0	10	805	5	349	
QEN71_RS36585	sn-glycerol-3-phosphate ABC transporter ATP-bindi	QEN71_36575	paras_008231	protein-codi	NZ_CP1252	chromosom	1590202	1591353	+	1152	0	13	2166	11	2148	
QEN71_RS36590	extracellular solute-binding protein	QEN71_36580	paras_008232	protein-codi	NZ_CP1252	chromosom	1591496	1592779	+	1284	107.0	23	2519	20	2406	
QEN71_RS36595	sugar ABC transporter permease	QEN71_36585	paras_008233	protein-codi	NZ_CP1252	chromosom	1592896	1593798	+	903	0	21	1511	20	1471	
QEN71_RS36600	carbohydrate ABC transporter permease	QEN71_36590	paras_008234	protein-codi	NZ_CP1252	chromosom	1593828	1594679	+	852	0	12	867	8	642	
QEN71_RS36605	beta-galactosidase	QEN71_36595	paras_008235	protein-codi	NZ_CP1252	chromosom	1594724	1596697	+	1970	0	11	716	10	694	
QEN71_RS36610	beta-galactosidase	QEN71_36600	paras_008236	protein-codi	NZ_CP1252	chromosom	1596694	1599117	+	2420	0	36	3426	27	2594	
QEN71_RS36615	MFS transporter	QEN71_36605	paras_008237	protein-codi	NZ_CP1252	chromosom	1599195	1600565	-	1371	0	28	2857	24	2630	
QEN71_RS36620	LysR family transcriptional regulator	QEN71_36610	paras_008238	protein-codi	NZ_CP1252	chromosom	1600716	1601672	-	957	0	7	677	6	598	
QEN71_RS36625	hypothetical protein	QEN71_36615	paras_008239	protein-codi	NZ_CP1252	chromosom	1601899	1602168	-	270	0	4	143	1	43	
QEN71_RS36630	LLM class flavin-dependent oxidoreductase	QEN71_36620	paras_008240	protein-codi	NZ_CP1252	chromosom	1602511	1603851	+	1337	0	26	3203	25	3113	
QEN71_RS36635	LysR substrate-binding domain-containing protein	QEN71_36625	paras_008241	protein-codi	NZ_CP1252	chromosom	1603848	1604696	-	845	0	6	1266	6	1266	
QEN71_RS36640	haloacid dehalogenase type II	QEN71_36630	paras_008242	protein-codi	NZ_CP1252	chromosom	1604813	1605517	+	705	0	10	882	8	655	
QEN71_RS36645	aldolase	QEN71_36635	paras_008243	protein-codi	NZ_CP1252	chromosom	1605537	1606322	+	786	0	20	3203	16	2744	
QEN71_RS36650	YbaK/EbsC family protein	QEN71_36640	paras_008244	protein-codi	NZ_CP1252	chromosom	1606337	1606822	-	486	0	18	3580	14	1618	
QEN71_RS36655	cysteine synthase A	QEN71_36645	paras_008245	protein-codi	NZ_CP1252	chromosom	1606934	1607908	-	975	0	36	7575	30	7173	
QEN71_RS36660	DeoR/GlpR family DNA-binding transcription regula	QEN71_36650	paras_008246	protein-codi	NZ_CP1252	chromosom	1608050	1608820	-	771	0	8	961	7	793	
QEN71_RS36665	4-hydroxythreonine-4-phosphate dehydrogenase P	QEN71_36655	paras_008247	protein-codi	NZ_CP1252	chromosom	1608885	1609883	-	999	0	10	145	8	103	
QEN71_RS36670	four-carbon acid sugar kinase family protein	QEN71_36660	paras_008248	protein-codi	NZ_CP1252	chromosom	1609922	1611181	-	1256	0	12	2036	10	1645	
QEN71_RS36675	2-keto-3-deoxygluconate permease	QEN71_36665	paras_008249	protein-codi	NZ_CP1252	chromosom	1611178	1612176	-	995	0	24	5781	23	5758	
QEN71_RS36680	aromatic alcohol reductase	QEN71_36670	paras_008250	protein-codi	NZ_CP1252	chromosom	1612418	1613362	-	945	0	9	740	9	740	
QEN71_RS36685	LysR family transcriptional regulator	QEN71_36675	paras_008251	protein-codi	NZ_CP1252	chromosom	1613624	1614514	+	891	0	16	3188	16	3188	
QEN71_RS36690	alpha/beta hydrolase	QEN71_36680	paras_008252	protein-codi	NZ_CP1252	chromosom	1614579	1615358	-	780	0	9	3510	9	3510	
QEN71_RS36695	HD domain-containing phosphohydrolase	QEN71_36685	paras_008253	protein-codi	NZ_CP1252	chromosom	1615515	1616987	+	1473	0	15	3627	15	3627	
QEN71_RS36700	DUF2322 family protein	QEN71_36690	paras_008254	protein-codi	NZ_CP1252	chromosom	1616993	1617325	-	333	0	8	932	5	176	
QEN71_RS36705	RNA polymerase sigma factor	QEN71_36695	paras_008255	protein-codi	NZ_CP1252	chromosom	1617606	1618154	+	545	0	7	785	6	783	
QEN71_RS36710	anti-sigma factor	QEN71_36700	paras_008256	protein-codi	NZ_CP1252	chromosom	1618151	1619083	+	929	0	11	3085	8	2152	
QEN71_RS36715	anti-sigma factor	QEN71_36705	paras_008257	protein-codi	NZ_CP1252	chromosom	1619255	1620175	+	921	0	19	1783	12	444	
QEN71_RS36720	catalase family peroxidase	QEN71_36710	paras_008258	protein-codi	NZ_CP1252	chromosom	1620218	1621270	+	1053	0	20	3547	11	2491	
QEN71_RS36725	response regulator transcription factor	QEN71_36715	paras_008259	protein-codi	NZ_CP1252	chromosom	1621586	1622305	+	720	0	13	2121	6	928	
QEN71_RS36730	phage tail tip lysozyme	QEN71_36720	paras_008260	protein-codi	NZ_CP1252	chromosom	1622544	1623467	-	924	0	10	1094	9	1089	
QEN71_RS36735	hypothetical protein	QEN71_36725	paras_008261	protein-codi	NZ_CP1252	chromosom	1623710	1624009	+	296	0	2	34	2	34	
QEN71_RS36740	lytic transglycosylase domain-containing protein	QEN71_36730	paras_008262	protein-codi	NZ_CP1252	chromosom	1624006	1624503	+	494	0	5	159	5	159	
QEN71_RS36745	hypothetical protein	QEN71_36735	paras_008263	protein-codi	NZ_CP1252	chromosom	1624592	1625299	-	708	0	8	1235	3	471	
QEN71_RS36750	HrpB1 family type III secretion system apparatus pr	QEN71_36740	paras_008264	protein-codi	NZ_CP1252	chromosom	1625606	1626064	+	459	0	3	395	3	395	
QEN71_RS36755	DUF3455 domain-containing protein	QEN71_36745	paras_008265	protein-codi	NZ_CP1252	chromosom	1626070	1626696	+	627	0	19	2411	19	2411	
QEN71_RS36760	NAD(P)/FAD-dependent oxidoreductase	QEN71_36750	paras_008266	protein-codi	NZ_CP1252	chromosom	1626787	1628034	+	1248	0	13	834	10	518	
QEN71_RS36765	type III secretion system outer membrane ring subu	QEN71_36755	paras_008267	protein-codi	NZ_CP1252	chromosom	1628047	1629855	-	1809	0	17	895	13	673	
QEN71_RS36770	helix-turn-helix transcriptional regulator	QEN71_36760	paras_008268	protein-codi	NZ_CP1252	chromosom	1629908	1631365	-	1458	0	17	1743	14	1163	
QEN71_RS36775	type III secretion system export apparatus subunit S	QEN71_36765	paras_008269	protein-codi	NZ_CP1252	chromosom	1631620	1632480	-	857	0	5	278	4	276	
QEN71_RS36780	type III secretion protein	QEN71_36770	paras_008270	protein-codi	NZ_CP1252	chromosom	1632477	1632974	-	490	0	6	168	6	168	
QEN71_RS36785	type III secretion system ATPase SctN	QEN71_36775	paras_008271	protein-codi	NZ_CP1252	chromosom	1632971	1634359	-	1381	0	9	382	6	101	
QEN71_RS36790	type III secretion system stator protein SctL	QEN71_36780	paras_008272	protein-codi	NZ_CP1252	chromosom	1634356	1635111	-	736	0	4	596	2	193	
QEN71_RS36795	type III secretion protein HrpB4	QEN71_36785	paras_008273	protein-codi	NZ_CP1252	chromosom	1635096	1635818	-	703	0	4	214	2	54	TRUE
QEN71_RS36800	type III secretion inner membrane ring lipoprotein S	QEN71_36790	paras_008274	protein-codi	NZ_CP1252	chromosom	1635815	1636573	-	755	0	11	1188	9	973	

QEN71_RS36805	type III secretion protein	QEN71_36795	paras_008275	protein-codi	NZ_CP1252	chromosom	1636615	1637007	-	393	0	2	429	0	0	
QEN71_RS36810	HrpB1 family type III secretion system apparatus pr	QEN71_36800	paras_008276	protein-codi	NZ_CP1252	chromosom	1637023	1637616	-	594	0	6	876	4	507	
QEN71_RS36815	type III secretion system export apparatus subunit S	QEN71_36805	paras_008277	protein-codi	NZ_CP1252	chromosom	1637862	1638950	+	1089	0	5	85	3	51	TRUE
QEN71_RS36820	type III secretion system export apparatus subunit S	QEN71_36810	paras_008278	protein-codi	NZ_CP1252	chromosom	1638983	1641070	+	2088	0	22	1695	13	1129	
QEN71_RS36825	type III secretion system protein SctP	QEN71_36815	paras_008279	protein-codi	NZ_CP1252	chromosom	1641079	1641642	+	564	0	2	177	1	72	TRUE
QEN71_RS36830	type III secretion system cytoplasmic ring protein Sc	QEN71_36820	paras_008280	protein-codi	NZ_CP1252	chromosom	1641676	1642884	+	1186	0	10	1034	5	344	
QEN71_RS36835	type III secretion system export apparatus subunit S	QEN71_36825	paras_008281	protein-codi	NZ_CP1252	chromosom	1642862	1643518	+	634	0	5	288	5	288	
QEN71_RS36840	type III secretion system export apparatus subunit S	QEN71_36830	paras_008282	protein-codi	NZ_CP1252	chromosom	1643548	1643811	+	260	0	3	163	1	97	
QEN71_RS36845	hypothetical protein	QEN71_36835	paras_008283	protein-codi	NZ_CP1252	chromosom	1643808	1644743	+	932	0	3	11	2	5	TRUE
QEN71_RS36850	type III secretion protein	QEN71_36840	paras_008284	protein-codi	NZ_CP1252	chromosom	1644850	1646028	+	1179	0	8	295	6	268	
QEN71_RS36855	type III secretion protein	QEN71_36845	paras_008285	protein-codi	NZ_CP1252	chromosom	1646031	1646273	+	243	0	4	513	2	343	
QEN71_RS36860	hypothetical protein	QEN71_36850	paras_008286	protein-codi	NZ_CP1252	chromosom	1646356	1646577	+	222	0	1	88	0	0	
QEN71_RS36865	CesT family type III secretion system chaperone	QEN71_36855	paras_008287	protein-codi	NZ_CP1252	chromosom	1646663	1647118	+	456	0	3	229	2	186	
QEN71_RS36870	MEKHLA domain-containing protein	QEN71_36860	paras_008288	protein-codi	NZ_CP1252	chromosom	1647178	1647636	+	459	0	4	247	4	247	
QEN71_RS36875	nitrite/sulfite reductase	QEN71_36865	paras_008289	protein-codi	NZ_CP1252	chromosom	1647862	1649652	+	1787	0	31	1704	23	941	
QEN71_RS36880	DUF934 domain-containing protein	QEN71_36870	paras_008290	protein-codi	NZ_CP1252	chromosom	1649649	1650050	+	398	0	8	554	2	121	
QEN71_RS36885	cytochrome o ubiquinol oxidase subunit IV	QEN71_36875	paras_008291	protein-codi	NZ_CP1252	chromosom	1650153	1650536	-	380	0	2	87	2	87	
QEN71_RS36890	cytochrome o ubiquinol oxidase subunit III	QEN71_36880	paras_008292	protein-codi	NZ_CP1252	chromosom	1650533	1651147	-	611	0	8	770	6	673	
QEN71_RS36895	cytochrome o ubiquinol oxidase subunit I	QEN71_36885	paras_008293	protein-codi	NZ_CP1252	chromosom	1651150	1653150	-	2001	0	40	4885	30	3948	
QEN71_RS36900	ubiquinol oxidase subunit II	QEN71_36890	paras_008294	protein-codi	NZ_CP1252	chromosom	1653156	1654088	-	933	0	27	3811	24	3001	
QEN71_RS36905	PLP-dependent aminotransferase family protein	QEN71_36895	paras_008295	protein-codi	NZ_CP1252	chromosom	1654325	1655734	+	1410	0	13	1674	13	1674	
QEN71_RS36910	glucose 1-dehydrogenase	QEN71_36900	paras_008296	protein-codi	NZ_CP1252	chromosom	1656018	1656764	-	747	0	7	241	7	241	
QEN71_RS36915	CBS domain-containing protein	QEN71_36905	paras_008297	protein-codi	NZ_CP1252	chromosom	1657046	1657741	-	696	0	5	758	1	10	
QEN71_RS36920	universal stress protein	QEN71_36910	paras_008298	protein-codi	NZ_CP1252	chromosom	1657900	1658856	+	957	0	8	1017	3	313	
QEN71_RS36925	nitroreductase family protein	pseudo;QEN71_36915		pseudogene	NZ_CP1252	chromosom	1659001	1659698	+	698	0	14	3461	12	3138	
QEN71_RS36930	universal stress protein	QEN71_36920	paras_008300	protein-codi	NZ_CP1252	chromosom	1659732	1660196	-	465	0	9	862	7	657	
QEN71_RS36935	flavodoxin	QEN71_36925	paras_008301	protein-codi	NZ_CP1252	chromosom	1660266	1660793	-	528	0	5	427	1	91	
QEN71_RS36940	zinc-dependent alcohol dehydrogenase family prote	QEN71_36930	paras_008302	protein-codi	NZ_CP1252	chromosom	1660880	1661920	-	1041	0	10	673	7	288	
QEN71_RS36945	universal stress protein	QEN71_36935	paras_008303	protein-codi	NZ_CP1252	chromosom	1662153	1662992	+	840	0	10	1585	8	1284	
QEN71_RS36950	universal stress protein	QEN71_36940	paras_008304	protein-codi	NZ_CP1252	chromosom	1663014	1663853	+	840	0	12	1968	10	1417	
QEN71_RS36955	PAS domain-containing sensor histidine kinase	partial;pseudo;QEN71_36945		protein-codi	NZ_CP1252	chromosom	1663903	1665273	+	1367	0	11	1913	9	1691	
QEN71_RS36960	response regulator transcription factor	QEN71_36950	paras_008306	protein-codi	NZ_CP1252	chromosom	1665270	1665917	+	644	0	10	1693	9	1465	
QEN71_RS36965	response regulator transcription factor	QEN71_36955	paras_008307	protein-codi	NZ_CP1252	chromosom	1666016	1666420	+	405	0	2	587	0	0	
QEN71_RS36970	fumarate/nitrate reduction transcriptional regulator F	QEN71_36960	paras_008308	protein-codi	NZ_CP1252	chromosom	1666438	1667241	+	804	0	12	3016	12	3016	
QEN71_RS36975	nitroreductase family protein	QEN71_36965	paras_008309	protein-codi	NZ_CP1252	chromosom	1667281	1668201	+	921	0	16	2293	15	2072	
QEN71_RS36980	DUF3564 domain-containing protein	QEN71_36970	paras_008310	protein-codi	NZ_CP1252	chromosom	1668369	1668749	+	381	0	6	1299	4	1065	
QEN71_RS36985	hypothetical protein	QEN71_36975	paras_008311	protein-codi	NZ_CP1252	chromosom	1668793	1669110	-	318	0	4	523	2	106	
QEN71_RS36990	PAS domain-containing sensor histidine kinase	QEN71_36980	paras_008312	protein-codi	NZ_CP1252	chromosom	1669344	1670444	+	1101	0	17	3737	7	885	
QEN71_RS36995	sulfate permease	QEN71_36985	paras_008313	protein-codi	NZ_CP1252	chromosom	1670467	1672230	-	1764	0	21	2788	15	2020	
QEN71_RS37000	YeeE/YedE thiosulfate transporter family protein	QEN71_36990	paras_008314	protein-codi	NZ_CP1252	chromosom	1672280	1672711	-	432	0	3	765	1	180	
QEN71_RS37005	YeeE/YedE family protein	QEN71_36995	paras_008315	protein-codi	NZ_CP1252	chromosom	1672713	1673156	-	444	0	7	817	3	51	
QEN71_RS37010	AMP-binding protein	QEN71_37000	paras_008316	protein-codi	NZ_CP1252	chromosom	1673247	1674890	-	1644	0	28	7764	22	6616	
QEN71_RS37015	AraC family transcriptional regulator	QEN71_37005	paras_008317	protein-codi	NZ_CP1252	chromosom	1675199	1676308	+	1110	0	20	5515	19	5341	
QEN71_RS37020	histone deacetylase family protein	QEN71_37010	paras_008318	protein-codi	NZ_CP1252	chromosom	1676476	1677411	+	936	0	19	2172	12	1656	
QEN71_RS37025	2-oxoglutarate dehydrogenase	QEN71_37015	paras_008319	protein-codi	NZ_CP1252	chromosom	1677483	1677878	-	396	0	3	224	0	0	
QEN71_RS37030	peptidase S10	QEN71_37020	paras_008320	protein-codi	NZ_CP1252	chromosom	1677955	1679769	-	1815	0	38	4609	34	4131	
QEN71_RS37035	ankyrin repeat domain-containing protein	QEN71_37025	paras_008321	protein-codi	NZ_CP1252	chromosom	1680264	1680932	-	669	0	7	337	5	189	
QEN71_RS37040	c-type cytochrome	QEN71_37030	paras_008322	protein-codi	NZ_CP1252	chromosom	1680948	1681487	-	540	0	15	1341	12	1095	
QEN71_RS37045	PQQ-binding-like beta-propeller repeat protein	QEN71_37035	paras_008323	protein-codi	NZ_CP1252	chromosom	1681580	1683313	-	1734	0	36	5586	26	4388	
QEN71_RS37050	GntR family transcriptional regulator	QEN71_37040	paras_008324	protein-codi	NZ_CP1252	chromosom	1683715	1684404	-	690	0	8	674	8	674	
QEN71_RS37055	hypothetical protein	QEN71_37045	paras_008325	protein-codi	NZ_CP1252	chromosom	1684525	1684719	-	187	0	4	261	4	261	
QEN71_RS37060	carboxymuconolactone decarboxylase family protei	QEN71_37050	paras_008326	protein-codi	NZ_CP1252	chromosom	1684712	1685497	-	778	0	15	2736	15	2736	
QEN71_RS37065	cupin domain-containing protein	QEN71_37055	paras_008327	protein-codi	NZ_CP1252	chromosom	1685520	1685915	-	392	0	6	514	5	467	
QEN71_RS37070	NAD(P)-dependent oxidoreductase	QEN71_37060	paras_008328	protein-codi	NZ_CP1252	chromosom	1685912	1686832	-	917	0	7	976	4	737	
QEN71_RS37075	MFS transporter	QEN71_37065	paras_008329	protein-codi	NZ_CP1252	chromosom	1686876	1688225	-	1350	0	27	1930	18	1347	
QEN71_RS37080	carboxymuconolactone decarboxylase family protei	QEN71_37070	paras_008330	protein-codi	NZ_CP1252	chromosom	1688301	1688696	-	396	0	4	143	2	132	
QEN71_RS37085	aldehyde dehydrogenase family protein	QEN71_37075	paras_008331	protein-codi	NZ_CP1252	chromosom	1688802	1689986	-	1185	0	18	1481	14	794	
QEN71_RS37090	NAD(P)-dependent oxidoreductase	QEN71_37080	paras_008332	protein-codi	NZ_CP1252	chromosom	1690009	1690896	-	888	0	8	696	6	689	
QEN71_RS37095	NIPSNAP family protein	QEN71_37085	paras_008333	protein-codi	NZ_CP1252	chromosom	1690916	1691242	-	327	0	6	523	4	273	
QEN71_RS37100	NAD(P)-dependent oxidoreductase	QEN71_37090	paras_008334	protein-codi	NZ_CP1252	chromosom	1691273	1692244	-	972	0	3	322	1	305	
QEN71_RS37105	MmgE/PrpD family protein	QEN71_37095	paras_008335	protein-codi	NZ_CP1252	chromosom	1692273	1693658	-	1375	0	19	2594	13	1982	
QEN71_RS37110	SDR family oxidoreductase	QEN71_37100	paras_008336	protein-codi	NZ_CP1252	chromosom	1693648	1694433	-	775	0	14	1363	13	1316	

QEN71_RS37115	N-acyl homoserine lactonase family protein	QEN71_37105	paras_008337	protein-codi	NZ_CP1252	chromosom	1694445	1695245	-	801	0	19	2013	13	1117	
QEN71_RS37120	MFS transporter	QEN71_37110	paras_008338	protein-codi	NZ_CP1252	chromosom	1695312	1696727	-	1416	0	22	3125	17	2567	
QEN71_RS37125	porin	QEN71_37115	paras_008339	protein-codi	NZ_CP1252	chromosom	1697232	1698395	+	1164	0	39	6318	32	5213	
QEN71_RS37130	HAD family phosphatase	QEN71_37120	paras_008340	protein-codi	NZ_CP1252	chromosom	1698505	1699188	+	684	0	11	2151	9	1575	
QEN71_RS37135	HDOD domain-containing protein	QEN71_37125	paras_008341	protein-codi	NZ_CP1252	chromosom	1699231	1700697	-	1467	0	24	7448	20	6963	
QEN71_RS37140	DUF3331 domain-containing protein	QEN71_37130	paras_008342	protein-codi	NZ_CP1252	chromosom	1700882	1701277	-	396	0	5	1622	3	1370	
QEN71_RS37145	TetR family transcriptional regulator	QEN71_37135	paras_008343	protein-codi	NZ_CP1252	chromosom	1701410	1702069	+	660	0	3	230	2	199	
QEN71_RS37150	hypothetical protein	QEN71_37140	paras_008344	protein-codi	NZ_CP1252	chromosom	1702085	1702354	-	270	0	9	1274	9	1274	
QEN71_RS37155	LysR substrate-binding domain-containing protein	QEN71_37145	paras_008345	protein-codi	NZ_CP1252	chromosom	1702605	1703015	-	407	0	1	229	1	229	
QEN71_RS37160	M14 family metallopeptidase	QEN71_37150	paras_008346	protein-codi	NZ_CP1252	chromosom	1703012	1704385	-	1370	0	42	5547	34	4527	
QEN71_RS37165	hypothetical protein	QEN71_37155	paras_008347	protein-codi	NZ_CP1252	chromosom	1704697	1704966	+	270	0	4	434	4	434	
QEN71_RS37170	S8 family serine peptidase	QEN71_37160	paras_008348	protein-codi	NZ_CP1252	chromosom	1705088	1707322	-	2235	0	34	3177	28	2418	
QEN71_RS37175	phosphatase PAP2 family protein	QEN71_37165	paras_008349	protein-codi	NZ_CP1252	chromosom	1707364	1708653	-	1290	0	21	2085	18	1907	
QEN71_RS37180	tetratricopeptide repeat protein	QEN71_37170	paras_008350	protein-codi	NZ_CP1252	chromosom	1708701	1710422	-	1722	0	21	2023	14	1523	
QEN71_RS37185	hypothetical protein	QEN71_37175	paras_008351	protein-codi	NZ_CP1252	chromosom	1710593	1710784	+	192	0	5	371	5	371	
QEN71_RS37190	LuxR C-terminal-related transcriptional regulator	QEN71_37180	paras_008352	protein-codi	NZ_CP1252	chromosom	1710819	1711886	-	1068	0	23	4032	14	2476	
QEN71_RS37195	chromate efflux transporter	QEN71_37185	paras_008353	protein-codi	NZ_CP1252	chromosom	1712044	1713282	-	1239	0	9	2059	5	1078	
QEN71_RS37200	IS4 family transposase	QEN71_37190	paras_008354	protein-codi	NZ_CP1252	chromosom	1713469	1714815	+	1347	2667.0	37	24813	28	21236	
QEN71_RS37205	trypsin-like peptidase domain-containing protein	QEN71_37195	paras_008355	protein-codi	NZ_CP1252	chromosom	1714854	1716425	-	1572	0	8	1199	5	563	
QEN71_RS37210	hypothetical protein	QEN71_37200	paras_008356	protein-codi	NZ_CP1252	chromosom	1716952	1717260	+	309	0	2	179	2	179	
QEN71_RS37215	zinc-dependent alcohol dehydrogenase family prote	QEN71_37205	paras_008357	protein-codi	NZ_CP1252	chromosom	1717297	1718283	+	987	0	21	2456	19	2127	
QEN71_RS37220	glutaminase	QEN71_37210	paras_008358	protein-codi	NZ_CP1252	chromosom	1718291	1719205	-	915	0	12	1167	9	921	
QEN71_RS37225	APC family permease	QEN71_37215	paras_008359	protein-codi	NZ_CP1252	chromosom	1719368	1720999	-	1632	194.0	51	7586	42	5983	
QEN71_RS37230	VIT1/CCC1 transporter family protein	QEN71_37220	paras_008360	protein-codi	NZ_CP1252	chromosom	1721377	1722057	-	681	0	7	1418	7	1418	
QEN71_RS37235	acyltransferase	QEN71_37225	paras_008361	protein-codi	NZ_CP1252	chromosom	1722182	1723372	-	1191	0	29	3719	26	3501	
QEN71_RS37240	AAA family ATPase	QEN71_37230	paras_008362	protein-codi	NZ_CP1252	chromosom	1723494	1725407	-	1914	0	34	4233	22	2290	
QEN71_RS37245	DUF1328 domain-containing protein	QEN71_37235	paras_008363	protein-codi	NZ_CP1252	chromosom	1725885	1726046	-	162	0	10	1153	6	486	
QEN71_RS37250	type II 3-dehydroquinate dehydratase	QEN71_37240	paras_008364	protein-codi	NZ_CP1252	chromosom	1726188	1726625	-	438	0	14	3806	12	3192	
QEN71_RS37255	Gfo/Idh/MocA family oxidoreductase	QEN71_37245	paras_008365	protein-codi	NZ_CP1252	chromosom	1726649	1727710	-	1062	0	18	5063	16	5056	
QEN71_RS37260	LysR family transcriptional regulator	QEN71_37250	paras_008366	protein-codi	NZ_CP1252	chromosom	1727798	1728754	+	957	0	15	1740	9	1239	
QEN71_RS37265	LysR family transcriptional regulator	QEN71_37255	paras_008367	protein-codi	NZ_CP1252	chromosom	1728768	1729694	-	927	0	21	2859	19	2687	
QEN71_RS37270	DsbA family oxidoreductase	QEN71_37260	paras_008368	protein-codi	NZ_CP1252	chromosom	1729816	1730457	+	642	0	13	1798	9	1130	
QEN71_RS37275	MFS transporter	QEN71_37265	paras_008369	protein-codi	NZ_CP1252	chromosom	1730528	1731742	+	1215	0	24	3211	18	2952	
QEN71_RS37280	2-keto-3-deoxygluconate transporter	QEN71_37270	paras_008370	protein-codi	NZ_CP1252	chromosom	1731826	1732851	-	1026	0	15	1258	12	1124	
QEN71_RS37285	5-dehydro-4-deoxy-D-glucuronate isomerase	QEN71_37275	paras_008371	protein-codi	NZ_CP1252	chromosom	1733132	1733968	+	837	0	25	6281	21	5634	
QEN71_RS37290	2-dehydro-3-deoxy-D-gluconate 5-dehydrogenase K	QEN71_37280	paras_008372	protein-codi	NZ_CP1252	chromosom	1733994	1734755	+	762	0	11	1093	6	778	
QEN71_RS37295	DNA-binding transcriptional regulator KdgR	QEN71_37285	paras_008373	protein-codi	NZ_CP1252	chromosom	1734842	1735726	+	885	0	20	3020	17	2961	
QEN71_RS37300	hypothetical protein	QEN71_37290	paras_008374	protein-codi	NZ_CP1252	chromosom	1736001	1736795	+	795	0	30	3405	23	2465	
QEN71_RS37305	Arm DNA-binding domain-containing protein	QEN71_37295	paras_008375	protein-codi	NZ_CP1252	chromosom	1737448	1738014	+	567	0	8	1244	7	1082	
QEN71_RS37310	S49 family peptidase	QEN71_37300	paras_008376	protein-codi	NZ_CP1252	chromosom	1738031	1738975	+	945	0	8	449	7	400	
QEN71_RS37315	hypothetical protein	QEN71_37305	paras_008377	protein-codi	NZ_CP1252	chromosom	1739281	1739814	+	534	0	7	341	7	341	
QEN71_RS37320	hypothetical protein	QEN71_37310	paras_008378	protein-codi	NZ_CP1252	chromosom	1739878	1740426	+	549	0	17	3007	17	3007	
QEN71_RS37325	hypothetical protein	QEN71_37315		protein-codi	NZ_CP1252	chromosom	1740472	1740597	-	126	0	0	0	0	0	
QEN71_RS37330	hypothetical protein	QEN71_37320	paras_008379	protein-codi	NZ_CP1252	chromosom	1740818	1741162	-	345	0	16	1176	11	701	
QEN71_RS37335	type VI secretion system baseplate subunit TssF	QEN71_37325	paras_008380	protein-codi	NZ_CP1252	chromosom	1742912	1744798	+	1887	0	27	2308	19	1806	
QEN71_RS37340	type VI secretion system Vgr family protein	QEN71_37330	paras_008381	protein-codi	NZ_CP1252	chromosom	1744848	1747394	+	2547	0	31	1990	26	1689	
QEN71_RS37345	DUF4123 domain-containing protein	QEN71_37335	paras_008382	protein-codi	NZ_CP1252	chromosom	1747397	1748290	+	890	0	18	1535	13	1301	
QEN71_RS37350	hypothetical protein	QEN71_37340	paras_008383	protein-codi	NZ_CP1252	chromosom	1748287	1748769	+	468	0	6	523	3	293	
QEN71_RS37355	DUF2235 domain-containing protein	QEN71_37345	paras_008384	protein-codi	NZ_CP1252	chromosom	1748759	1750714	+	1945	0	46	4228	38	3610	
QEN71_RS37360	PAAR domain-containing protein	QEN71_37350	paras_008385	protein-codi	NZ_CP1252	chromosom	1750826	1751083	+	258	0	7	279	6	266	
QEN71_RS37365	hypothetical protein	QEN71_37355	paras_008386	protein-codi	NZ_CP1252	chromosom	1751286	1752194	+	909	0	15	1650	14	1633	
QEN71_RS37370	alginate export family protein	QEN71_37360	paras_008387	protein-codi	NZ_CP1252	chromosom	1752780	1754159	-	1380	0	34	2699	30	2639	
QEN71_RS37375	glyoxalase	QEN71_37365	paras_008388	protein-codi	NZ_CP1252	chromosom	1754249	1755184	-	936	0	23	3061	17	2618	
QEN71_RS37380	YoaK family protein	QEN71_37370	paras_008389	protein-codi	NZ_CP1252	chromosom	1755249	1755992	-	725	0	5	49	5	49	
QEN71_RS37385	DUF1427 family protein	QEN71_37375	paras_008390	protein-codi	NZ_CP1252	chromosom	1755974	1756213	-	221	0	6	375	5	337	
QEN71_RS37390	DoxX family protein	QEN71_37380	paras_008391	protein-codi	NZ_CP1252	chromosom	1756268	1756690	-	423	0	4	147	1	7	TRUE
QEN71_RS37395	amidohydrolase	QEN71_37385	paras_008392	protein-codi	NZ_CP1252	chromosom	1756705	1758585	-	1881	0	15	1244	13	1131	
QEN71_RS37400	hydrolase	QEN71_37390	paras_008393	protein-codi	NZ_CP1252	chromosom	1758708	1759343	-	636	0	7	594	6	582	
QEN71_RS37405	winged helix-turn-helix domain-containing protein	QEN71_37395	paras_008394	protein-codi	NZ_CP1252	chromosom	1759574	1762342	-	2769	0	39	5282	31	3489	
QEN71_RS37410	alpha/beta hydrolase	QEN71_37400	paras_008395	protein-codi	NZ_CP1252	chromosom	1762503	1763495	-	993	0	19	2913	14	2066	
QEN71_RS37415	winged helix-turn-helix domain-containing protein	QEN71_37405	paras_008396	protein-codi	NZ_CP1252	chromosom	1763751	1766672	-	2922	0	28	3408	24	2847	
QEN71_RS37420	AraC family transcriptional regulator	QEN71_37410	paras_008397	protein-codi	NZ_CP1252	chromosom	1767151	1768065	+	915	0	18	1308	16	1264	

QEN71_RS37425	HlyD family secretion protein	QEN71_37415	paras_008398	protein-codi	NZ_CP1252	chromosom	1768075	1769040	-		966	0	24	2659	16	1517	
QEN71_RS37430	DUF1656 domain-containing protein	QEN71_37420	paras_008399	protein-codi	NZ_CP1252	chromosom	1769096	1769296	-		184	0	5	252	5	252	
QEN71_RS37435	efflux transporter outer membrane subunit	QEN71_37425	paras_008400	protein-codi	NZ_CP1252	chromosom	1769280	1770776	-		1476	0	19	2796	17	2527	
QEN71_RS37440	FUSC family protein	QEN71_37430	paras_008401	protein-codi	NZ_CP1252	chromosom	1770773	1772935	-		2159	0	28	2861	22	2203	
QEN71_RS37445	LysR family transcriptional regulator	QEN71_37435	paras_008402	protein-codi	NZ_CP1252	chromosom	1773220	1774140	+		921	0	10	1463	9	1444	
QEN71_RS37450	winged helix-turn-helix domain-containing protein	QEN71_37440	paras_008403	protein-codi	NZ_CP1252	chromosom	1774158	1777088	-		2931	0	27	3487	24	3285	
QEN71_RS37455	MBL fold metallo-hydrolase	QEN71_37445	paras_008404	protein-codi	NZ_CP1252	chromosom	1777339	1778328	-		990	0	14	1030	9	887	
QEN71_RS37460	FAD-dependent oxidoreductase	QEN71_37450	paras_008405	protein-codi	NZ_CP1252	chromosom	1778400	1780130	-		1731	0	25	2849	17	2316	
QEN71_RS37465	HD domain-containing protein	QEN71_37455	paras_008406	protein-codi	NZ_CP1252	chromosom	1780173	1780814	-		642	0	9	427	8	418	
QEN71_RS37470	FAD binding domain-containing protein	QEN71_37460	paras_008407	protein-codi	NZ_CP1252	chromosom	1781286	1782491	+		1206	0	16	898	14	752	
QEN71_RS37475	c-type cytochrome	QEN71_37465	paras_008408	protein-codi	NZ_CP1252	chromosom	1782559	1783848	+		1286	0	22	2544	16	2290	
QEN71_RS37480	(2Fe-2S)-binding protein	QEN71_37470	paras_008409	protein-codi	NZ_CP1252	chromosom	1783845	1784333	+		481	0	4	138	4	138	
QEN71_RS37485	molybdopterin-dependent oxidoreductase	QEN71_37475	paras_008410	protein-codi	NZ_CP1252	chromosom	1784330	1786600	+		2267	0	17	2176	16	1771	
QEN71_RS37490	LysR family transcriptional regulator	QEN71_37480	paras_008411	protein-codi	NZ_CP1252	chromosom	1786815	1787777	+		959	0	10	277	7	196	
QEN71_RS37495	hypothetical protein	QEN71_37485	paras_008412	protein-codi	NZ_CP1252	chromosom	1787774	1788136	+		359	0	5	226	3	68	
QEN71_RS37500	YihY/virulence factor BrkB family protein	QEN71_37490	paras_008413	protein-codi	NZ_CP1252	chromosom	1788155	1789018	-		864	0	9	555	7	376	
QEN71_RS37505	hypothetical protein	QEN71_37495	paras_008414	protein-codi	NZ_CP1252	chromosom	1789151	1789321	-		171	0	0	0	0	0	
QEN71_RS37510	FdhF/YdeP family oxidoreductase	QEN71_37500	paras_008415	protein-codi	NZ_CP1252	chromosom	1789488	1791845	+		2350	0	24	1930	15	834	
QEN71_RS37515	cytochrome ubiquinol oxidase subunit I	QEN71_37505	paras_008416	protein-codi	NZ_CP1252	chromosom	1791838	1793241	+		1396	0	16	1255	14	875	
QEN71_RS37520	cytochrome d ubiquinol oxidase subunit II	QEN71_37510	paras_008417	protein-codi	NZ_CP1252	chromosom	1793272	1794261	+		990	0	29	2168	11	738	
QEN71_RS37525	alpha/beta hydrolase	QEN71_37515	paras_008418	protein-codi	NZ_CP1252	chromosom	1794346	1795218	-		873	0	8	1144	8	1144	
QEN71_RS37530	LysR family transcriptional regulator	QEN71_37520	paras_008419	protein-codi	NZ_CP1252	chromosom	1795366	1796343	-		978	0	19	1096	17	1070	
QEN71_RS37535	DUF3331 domain-containing protein	QEN71_37525	paras_008420	protein-codi	NZ_CP1252	chromosom	1796490	1796882	-		393	0	2	102	2	102	
QEN71_RS37540	AraC family transcriptional regulator	QEN71_37530	paras_008421	protein-codi	NZ_CP1252	chromosom	1796950	1797909	-		960	0	4	742	3	643	
QEN71_RS37545	response regulator	QEN71_37535	paras_008422	protein-codi	NZ_CP1252	chromosom	1798261	1798935	-		661	0	6	1476	4	1019	
QEN71_RS37550	PAS domain S-box protein	QEN71_37540	paras_008423	protein-codi	NZ_CP1252	chromosom	1798922	1801138	-		2203	0	29	3925	24	3569	
QEN71_RS37555	response regulator	QEN71_37545	paras_008424	protein-codi	NZ_CP1252	chromosom	1801374	1801757	-		384	0	8	861	6	661	
QEN71_RS37560	H-NS family nucleoid-associated regulatory protein			protein-codi	NZ_CP1252	chromosom	1801803	1802360	+		558	0	25	8192	18	5913	
QEN71_RS37565	PilZ domain-containing protein	QEN71_37555	paras_008426	protein-codi	NZ_CP1252	chromosom	1802548	1803474	-		927	0	10	1326	8	881	
QEN71_RS37570	VOC family protein	QEN71_37560	paras_008427	protein-codi	NZ_CP1252	chromosom	1803746	1804171	+		426	0	5	778	4	674	
QEN71_RS37575	LysR family transcriptional regulator	QEN71_37565	paras_008428	protein-codi	NZ_CP1252	chromosom	1804322	1805269	+		948	0	16	2513	12	1481	
QEN71_RS37580	LysR family transcriptional regulator	QEN71_37570	paras_008429	protein-codi	NZ_CP1252	chromosom	1805278	1806165	-		810	0	8	1209	6	1002	
QEN71_RS37585	hypothetical protein	QEN71_37575	paras_008430	protein-codi	NZ_CP1252	chromosom	1806088	1806336	+		167	0	2	192	2	192	
QEN71_RS37590	DUF4148 domain-containing protein	QEN71_37580	paras_008431	protein-codi	NZ_CP1252	chromosom	1806333	1806644	-		308	0	3	195	2	186	
QEN71_RS37595	hypothetical protein	QEN71_37585		protein-codi	NZ_CP1252	chromosom	1806848	1806973	-		126	0	4	61	2	7	
QEN71_RS37600	thioredoxin family protein	QEN71_37590	paras_008432	protein-codi	NZ_CP1252	chromosom	1807156	1807761	+		606	0	15	1254	15	1254	
QEN71_RS37605	D-cysteine desulfhydrase family protein	QEN71_37595	paras_008433	protein-codi	NZ_CP1252	chromosom	1807803	1808816	-		1014	0	20	1993	17	1818	
QEN71_RS37610	DUF2282 domain-containing protein	QEN71_37600	paras_008434	protein-codi	NZ_CP1252	chromosom	1809111	1809389	+		278	0	2	186	1	118	
QEN71_RS37615	DoxX family protein	QEN71_37605	paras_008435	protein-codi	NZ_CP1252	chromosom	1809389	1809886	+		497	0	3	211	3	211	
QEN71_RS37620	alpha/beta hydrolase	QEN71_37610	paras_008436	protein-codi	NZ_CP1252	chromosom	1809962	1810726	+		765	0	13	952	12	701	
QEN71_RS37625	hypothetical protein	QEN71_37615	paras_008437	protein-codi	NZ_CP1252	chromosom	1810783	1811052	+		270	0	3	150	1	11	
QEN71_RS37630	DUF692 domain-containing protein	QEN71_37620	paras_008438	protein-codi	NZ_CP1252	chromosom	1811127	1812005	+		875	0	10	774	8	610	
QEN71_RS37635	DNA-binding domain-containing protein	QEN71_37625	paras_008439	protein-codi	NZ_CP1252	chromosom	1812002	1812754	+		706	0	7	1559	6	1083	
QEN71_RS37640	tautomerase family protein			protein-codi	NZ_CP1252	chromosom	1812712	1812861	+		107	0	1	5	1	5	
QEN71_RS37645	organic hydroperoxide resistance protein	QEN71_37635	paras_008440	protein-codi	NZ_CP1252	chromosom	1812970	1813395	+		426	0	0	0	0	0	TRUE
QEN71_RS37650	hypothetical protein	QEN71_37640	paras_008441	protein-codi	NZ_CP1252	chromosom	1813471	1813668	-		198	0	3	166	1	2	
QEN71_RS37655	CoA-acylating methylmalonate-semialdehyde dehyd	QEN71_37645	paras_008442	protein-codi	NZ_CP1252	chromosom	1813904	1815415	-		1512	0	19	2187	14	1535	
QEN71_RS37660	LysR family transcriptional regulator	QEN71_37650	paras_008443	protein-codi	NZ_CP1252	chromosom	1815523	1816464	+		942	0	11	1318	7	943	
QEN71_RS37665	hypothetical protein	QEN71_37655	paras_008444	protein-codi	NZ_CP1252	chromosom	1816536	1816808	-		269	0	11	1734	9	1660	
QEN71_RS37670	hypothetical protein	QEN71_37660	paras_008445	protein-codi	NZ_CP1252	chromosom	1816805	1817602	-		794	0	23	2390	19	1948	
QEN71_RS37675	helix-turn-helix transcriptional regulator	QEN71_37665	paras_008446	protein-codi	NZ_CP1252	chromosom	1817760	1818599	-		840	0	11	1144	5	472	
QEN71_RS37680	SDR family oxidoreductase	QEN71_37670	paras_008447	protein-codi	NZ_CP1252	chromosom	1818699	1819589	+		891	0	9	1473	6	791	
QEN71_RS37685	methyl-accepting chemotaxis protein	QEN71_37675	paras_008448	protein-codi	NZ_CP1252	chromosom	1819636	1821537	-		1902	0	28	7628	24	6778	
QEN71_RS37690	PAS domain S-box protein	QEN71_37680	paras_008449	protein-codi	NZ_CP1252	chromosom	1821955	1824492	+		2538	0	31	5995	28	5596	
QEN71_RS37695	hypothetical protein	QEN71_37685	paras_008450	protein-codi	NZ_CP1252	chromosom	1824595	1824843	+		249	0	7	2363	5	1988	
QEN71_RS37700	MFS transporter	QEN71_37690	paras_008451	protein-codi	NZ_CP1252	chromosom	1825243	1826532	+		1290	0	23	4052	14	2215	
QEN71_RS37705	LacI family DNA-binding transcriptional regulator	QEN71_37695	paras_008452	protein-codi	NZ_CP1252	chromosom	1826600	1827643	-		1044	0	18	3260	18	3260	
QEN71_RS37710	NAD(P)-dependent oxidoreductase	QEN71_37700	paras_008453	protein-codi	NZ_CP1252	chromosom	1827748	1828581	+		834	0	19	4139	17	3950	
QEN71_RS37715	hypothetical protein	QEN71_37705	paras_008454	protein-codi	NZ_CP1252	chromosom	1828780	1829085	+		306	0	12	1772	10	1046	
QEN71_RS37720	hypothetical protein	QEN71_37710	paras_008455	protein-codi	NZ_CP1252	chromosom	1829392	1830336	+		945	0	20	6191	15	3693	
QEN71_RS37725	oxidoreductase	QEN71_37715	paras_008456	protein-codi	NZ_CP1252	chromosom	1830431	1831288	-		858	0	10	1466	10	1466	
QEN71_RS37730	LysR family transcriptional regulator	QEN71_37720	paras_008457	protein-codi	NZ_CP1252	chromosom	1831406	1832335	+		930	0	16	2580	11	1849	

QEN71_RS37735	M1 family metallopeptidase	QEN71_37725	paras_008458	protein-codi	NZ_CP1252	chromosom	1832414	1834585	-	2172	0	49	8445	47	8240	
QEN71_RS37740	EAL domain-containing protein	QEN71_37730	paras_008459	protein-codi	NZ_CP1252	chromosom	1835347	1836885	+	1539	0	37	6304	33	6075	
QEN71_RS37745	DUF1348 family protein	QEN71_37735	paras_008460	protein-codi	NZ_CP1252	chromosom	1836916	1837398	-	483	0	5	467	5	467	
QEN71_RS37750	cyanase	QEN71_37740	paras_008461	protein-codi	NZ_CP1252	chromosom	1837469	1837939	-	471	0	5	743	3	310	
QEN71_RS37755	carbonic anhydrase	QEN71_37745	paras_008462	protein-codi	NZ_CP1252	chromosom	1837988	1838629	-	642	0	9	1354	6	707	
QEN71_RS37760	transcriptional regulator CynR	QEN71_37750	paras_008463	protein-codi	NZ_CP1252	chromosom	1838761	1839642	+	882	0	19	1623	16	1236	
QEN71_RS37765	sigma-54 dependent transcriptional regulator	QEN71_37755	paras_008464	protein-codi	NZ_CP1252	chromosom	1839644	1841014	-	1360	0	18	2225	11	1242	
QEN71_RS37770	ATP-binding protein	QEN71_37760	paras_008465	protein-codi	NZ_CP1252	chromosom	1841004	1842887	-	1873	0	21	2590	12	1787	
QEN71_RS37775	2-hydroxycarboxylate transporter family protein	QEN71_37765	paras_008466	protein-codi	NZ_CP1252	chromosom	1842961	1844349	-	1389	0	33	7947	31	7722	
QEN71_RS37780	hypothetical protein	QEN71_37770	paras_008467	protein-codi	NZ_CP1252	chromosom	1844600	1845037	+	438	0	13	1999	10	1561	
QEN71_RS37785	organic hydroperoxide resistance protein	QEN71_37775	paras_008468	protein-codi	NZ_CP1252	chromosom	1845109	1845525	-	417	0	4	431	2	63	
QEN71_RS37790	hypothetical protein	QEN71_37780	paras_008469	protein-codi	NZ_CP1252	chromosom	1845552	1845821	-	270	0	4	453	2	348	
QEN71_RS37795	MarR family transcriptional regulator	QEN71_37785	paras_008470	protein-codi	NZ_CP1252	chromosom	1846081	1846542	-	462	0	6	855	4	34	
QEN71_RS37800	sulfite reductase subunit alpha	QEN71_37790	paras_008471	protein-codi	NZ_CP1252	chromosom	1846670	1850833	-	4164	0	51	4618	35	3024	
QEN71_RS37805	nitrite reductase small subunit NirD	QEN71_37795	paras_008472	protein-codi	NZ_CP1252	chromosom	1850866	1851213	-	348	0	6	336	5	330	
QEN71_RS37810	nitrite reductase large subunit NirB	QEN71_37800	paras_008473	protein-codi	NZ_CP1252	chromosom	1851244	1853808	-	2565	0	27	2740	23	2251	
QEN71_RS37815	MFS transporter	QEN71_37805	paras_008474	protein-codi	NZ_CP1252	chromosom	1853843	1855162	-	1320	0	15	1093	14	1090	
QEN71_RS37820	DUF6402 family protein	QEN71_37810	paras_008475	protein-codi	NZ_CP1252	chromosom	1855920	1857080	-	1161	0	35	1994	27	1591	
QEN71_RS37825	LysR substrate-binding domain-containing protein	QEN71_37815	paras_008476	protein-codi	NZ_CP1252	chromosom	1857249	1858169	-	921	0	11	450	9	288	
QEN71_RS37830	tartrate dehydrogenase	QEN71_37820	paras_008477	protein-codi	NZ_CP1252	chromosom	1858281	1859363	+	1083	0	11	476	5	268	
QEN71_RS37835	MFS transporter	QEN71_37825	paras_008478	protein-codi	NZ_CP1252	chromosom	1859475	1860785	+	1311	0	11	821	7	193	
QEN71_RS37840	CsbD family protein	QEN71_37830	paras_008479	protein-codi	NZ_CP1252	chromosom	1860834	1861013	-	180	0	0	0	0	0	
QEN71_RS37845	hypothetical protein	QEN71_37835	paras_008480	protein-codi	NZ_CP1252	chromosom	1861083	1861499	-	417	0	6	881	5	877	
QEN71_RS37850	GNAT family N-acetyltransferase	QEN71_37840	paras_008481	protein-codi	NZ_CP1252	chromosom	1861844	1862707	+	864	0	18	1131	14	852	
QEN71_RS37855	3-hydroxybutyryl-CoA dehydrogenase	QEN71_37845	paras_008482	protein-codi	NZ_CP1252	chromosom	1862723	1863589	-	867	0	6	502	3	71	
QEN71_RS37860	hypothetical protein	QEN71_37850	paras_008483	protein-codi	NZ_CP1252	chromosom	1863777	1864208	+	432	0	4	1186	4	1186	
QEN71_RS37865	carbonic anhydrase	QEN71_37855	paras_008484	protein-codi	NZ_CP1252	chromosom	1864347	1864889	-	543	0	8	871	4	360	
QEN71_RS37870	nitrilase-related carbon-nitrogen hydrolase	QEN71_37860	paras_008485	protein-codi	NZ_CP1252	chromosom	1864993	1865976	-	984	0	20	2135	17	1977	
QEN71_RS37875	GlxA family transcriptional regulator	QEN71_37865	paras_008486	protein-codi	NZ_CP1252	chromosom	1866172	1867128	+	957	0	15	1210	8	572	
QEN71_RS37880	ABC transporter ATP-binding protein	QEN71_37870	paras_008487	protein-codi	NZ_CP1252	chromosom	1867139	1868887	-	1745	0	24	3029	22	2831	
QEN71_RS37885	ABC transporter permease	QEN71_37875	paras_008488	protein-codi	NZ_CP1252	chromosom	1868884	1869741	-	850	0	13	1078	10	693	
QEN71_RS37890	ABC transporter permease	QEN71_37880	paras_008489	protein-codi	NZ_CP1252	chromosom	1869738	1870667	-	926	0	7	703	5	382	
QEN71_RS37895	ABC transporter substrate-binding protein	QEN71_37885	paras_008490	protein-codi	NZ_CP1252	chromosom	1870669	1872375	-	1707	0	29	2912	23	2343	
QEN71_RS37900	BON domain-containing protein	QEN71_37890	paras_008491	protein-codi	NZ_CP1252	chromosom	1872649	1872996	+	348	0	2	47	2	47	
QEN71_RS37905	LysR family transcriptional regulator	QEN71_37895	paras_008492	protein-codi	NZ_CP1252	chromosom	1873017	1873919	-	903	0	11	770	8	500	
QEN71_RS37910	peroxiredoxin-like family protein	QEN71_37900	paras_008493	protein-codi	NZ_CP1252	chromosom	1874025	1874684	+	660	0	7	541	6	536	
QEN71_RS37915	gamma-glutamylcyclotransferase	QEN71_37905	paras_008494	protein-codi	NZ_CP1252	chromosom	1874842	1875513	+	672	0	4	259	0	0	TRUE
QEN71_RS37920	LysR family transcriptional regulator	QEN71_37910	paras_008495	protein-codi	NZ_CP1252	chromosom	1875539	1876459	+	921	0	4	127	4	127	
QEN71_RS37925	efflux RND transporter periplasmic adaptor subunit	QEN71_37915	paras_008496	protein-codi	NZ_CP1252	chromosom	1876656	1877831	+	1176	0	14	923	14	923	
QEN71_RS37930	multidrug efflux RND transporter permease subunit	QEN71_37920	paras_008497	protein-codi	NZ_CP1252	chromosom	1877850	1881086	+	3237	0	32	2372	26	1449	
QEN71_RS37935	efflux transporter outer membrane subunit	QEN71_37925	paras_008498	protein-codi	NZ_CP1252	chromosom	1881097	1882602	+	1506	0	11	1615	5	359	
QEN71_RS37940	LysR family transcriptional regulator	QEN71_37930	paras_008499	protein-codi	NZ_CP1252	chromosom	1882603	1883514	-	912	0	11	1020	9	964	
QEN71_RS37945	glutathione binding-like protein	QEN71_37935	paras_008500	protein-codi	NZ_CP1252	chromosom	1883850	1884488	+	639	0	12	1117	9	995	
QEN71_RS37950	transglutaminase family protein	QEN71_37940	paras_008501	protein-codi	NZ_CP1252	chromosom	1884545	1885552	+	1004	0	16	467	13	409	
QEN71_RS37955	hypothetical protein	QEN71_37945	paras_008502	protein-codi	NZ_CP1252	chromosom	1885549	1887921	-	2369	0	31	1950	23	1375	
QEN71_RS37960	alpha/beta hydrolase	QEN71_37950	paras_008503	protein-codi	NZ_CP1252	chromosom	1888342	1889160	-	819	0	11	750	6	323	
QEN71_RS37965	LysR substrate-binding domain-containing protein	QEN71_37955	paras_008504	protein-codi	NZ_CP1252	chromosom	1889509	1890426	+	918	0	24	1168	19	1094	
QEN71_RS37970	mechanosensitive ion channel	QEN71_37960	paras_008506	protein-codi	NZ_CP1252	chromosom	1891640	1892851	+	1212	0	33	3974	28	3629	
QEN71_RS37975	LysR family transcriptional regulator	QEN71_37965	paras_008507	protein-codi	NZ_CP1252	chromosom	1892870	1893766	-	897	0	14	1084	10	637	
QEN71_RS37980	NADH:flavin oxidoreductase	QEN71_37970	paras_008508	protein-codi	NZ_CP1252	chromosom	1893872	1894987	+	1116	0	28	4344	18	3659	
QEN71_RS37985	AraC family transcriptional regulator	QEN71_37975	paras_008509	protein-codi	NZ_CP1252	chromosom	1895048	1896031	-	984	0	10	909	9	892	
QEN71_RS37990	carboxymuconolactone decarboxylase family protei	QEN71_37980	paras_008510	protein-codi	NZ_CP1252	chromosom	1896143	1896682	+	540	0	8	603	5	118	
QEN71_RS37995	FAD-dependent oxidoreductase	QEN71_37985	paras_008511	protein-codi	NZ_CP1252	chromosom	1896751	1898172	+	1422	0	18	1981	13	1391	
QEN71_RS38000	hypothetical protein	QEN71_37990	paras_008512	protein-codi	NZ_CP1252	chromosom	1898399	1898674	+	276	0	13	935	11	829	
QEN71_RS38005	DUF2846 domain-containing protein	QEN71_37995	paras_008513	protein-codi	NZ_CP1252	chromosom	1899675	1900130	-	456	0	18	2191	15	2044	
QEN71_RS38010	transcriptional regulator GlcC	QEN71_38000	paras_008514	protein-codi	NZ_CP1252	chromosom	1900287	1901066	-	780	0	13	2159	13	2159	
QEN71_RS38015	glycolate oxidase subunit GlcD	QEN71_38005	paras_008515	protein-codi	NZ_CP1252	chromosom	1901289	1902788	+	1499	0	14	1023	9	550	
QEN71_RS38020	glycolate oxidase subunit GlcE	QEN71_38010	paras_008516	protein-codi	NZ_CP1252	chromosom	1902788	1903855	+	1067	0	6	533	5	528	
QEN71_RS38025	glycolate oxidase subunit GlcF	QEN71_38015	paras_008517	protein-codi	NZ_CP1252	chromosom	1903869	1905137	+	1269	0	9	924	8	798	
QEN71_RS38030	MFS transporter	QEN71_38020	paras_008518	protein-codi	NZ_CP1252	chromosom	1905167	1906522	-	1356	0	26	2835	18	1840	
QEN71_RS38035	MmgE/PrpD family protein	QEN71_38025	paras_008519	protein-codi	NZ_CP1252	chromosom	1906583	1908001	-	1419	0	16	1799	13	1407	
QEN71_RS38040	LysR family transcriptional regulator	QEN71_38030	paras_008520	protein-codi	NZ_CP1252	chromosom	1908169	1909095	+	927	0	5	777	5	777	

QEN71_RS38045	type III secretion system cytoplasmic ring protein Sc	QEN71_38035	paras_008521	protein-codi	NZ_CP1252	chromosome	1909341	1910507	+	1159	0	14	2896	14	2896	
QEN71_RS38050	type III secretion system export apparatus subunit S	QEN71_38040	paras_008522	protein-codi	NZ_CP1252	chromosome	1910500	1911168	+	657	0	10	1190	9	1188	
QEN71_RS38055	lytic transglycosylase domain-containing protein	QEN71_38045	paras_008523	protein-codi	NZ_CP1252	chromosome	1911165	1912010	+	842	0	11	2068	10	2051	
QEN71_RS38060	LWXIA domain-containing protein	QEN71_38050	paras_008524	protein-codi	NZ_CP1252	chromosome	1912192	1924416	+	12225	0	198	35171	159	26798	
QEN71_RS38065	EscU/YscU/HrcU family type III secretion system ex	QEN71_38055	paras_008525	protein-codi	NZ_CP1252	chromosome	1924417	1925448	-	1032	0	13	619	12	582	
QEN71_RS38070	type III secretion system export apparatus subunit S	QEN71_38060	paras_008526	protein-codi	NZ_CP1252	chromosome	1925462	1926265	-	797	0	5	663	2	56	
QEN71_RS38075	hypothetical protein	partial;pseudo;QEN71_38065		protein-codi	NZ_CP1252	chromosome	1926259	1926852	-	579	0	1	9	1	9	TRUE
QEN71_RS38080	type III secretion system ATPase SctN	QEN71_38070	paras_008528	protein-codi	NZ_CP1252	chromosome	1926845	1928215	-	1359	0	12	2895	10	2781	
QEN71_RS38085	type III secretion system stator protein SctL	QEN71_38075	paras_008529	protein-codi	NZ_CP1252	chromosome	1928212	1928847	-	613	0	8	547	8	547	
QEN71_RS38090	SctK family type III secretion system sorting platforn	QEN71_38080	paras_008530	protein-codi	NZ_CP1252	chromosome	1928829	1929536	-	689	0	13	2300	11	1980	
QEN71_RS38095	type III secretion inner membrane ring lipoprotein S	QEN71_38085	paras_008531	protein-codi	NZ_CP1252	chromosome	1929567	1930454	-	888	0	21	3818	19	3278	
QEN71_RS38100	hypothetical protein	QEN71_38090	paras_008532	protein-codi	NZ_CP1252	chromosome	1930458	1930973	-	516	0	3	503	3	503	
QEN71_RS38105	hypothetical protein	QEN71_38095	paras_008533	protein-codi	NZ_CP1252	chromosome	1931113	1931325	-	213	0	4	482	3	290	
QEN71_RS38110	type III secretion system inner membrane ring subu	QEN71_38100	paras_008534	protein-codi	NZ_CP1252	chromosome	1931505	1932851	-	1327	0	23	6518	18	5173	
QEN71_RS38115	type III secretion system outer membrane ring subu	QEN71_38105	paras_008535	protein-codi	NZ_CP1252	chromosome	1932832	1934919	-	2067	0	41	8872	38	8808	
QEN71_RS38120	type III secretion system export apparatus subunit S	QEN71_38110	paras_008536	protein-codi	NZ_CP1252	chromosome	1934919	1935197	-	274	0	0	0	0	0	
QEN71_RS38125	type III secretion system export apparatus subunit S	QEN71_38115	paras_008537	protein-codi	NZ_CP1252	chromosome	1935194	1937389	-	2192	0	18	5035	13	4450	
QEN71_RS38130	autoinducer binding domain-containing protein	QEN71_38120	paras_008538	protein-codi	NZ_CP1252	chromosome	1937703	1938551	-	849	0	28	7404	25	6721	
QEN71_RS38135	autoinducer binding domain-containing protein	QEN71_38125	paras_008539	protein-codi	NZ_CP1252	chromosome	1938888	1939736	+	849	0	15	3200	11	2552	
QEN71_RS38140	ATP-dependent Clp protease proteolytic subunit	QEN71_38130	paras_008540	protein-codi	NZ_CP1252	chromosome	1939968	1940774	+	807	0	14	2428	11	1195	
QEN71_RS38145	glycogen/starch/alpha-glucan phosphorylase	QEN71_38135	paras_008541	protein-codi	NZ_CP1252	chromosome	1940818	1943280	-	2463	0	55	5058	43	3960	
QEN71_RS38150	hypothetical protein	QEN71_38140	paras_008542	protein-codi	NZ_CP1252	chromosome	1943493	1943684	-	192	0	2	99	2	99	
QEN71_RS38155	YaiI/YqxD family protein	QEN71_38145	paras_008543	protein-codi	NZ_CP1252	chromosome	1944193	1944651	-	459	0	13	1002	12	998	
QEN71_RS38160	Rieske 2Fe-2S domain-containing protein	QEN71_38150	paras_008544	protein-codi	NZ_CP1252	chromosome	1944728	1945063	-	332	0	5	421	5	421	
QEN71_RS38165	YciI family protein	QEN71_38155	paras_008545	protein-codi	NZ_CP1252	chromosome	1945060	1945371	-	304	0	5	1131	4	1109	
QEN71_RS38170	intradiol ring-cleavage dioxygenase	QEN71_38160	paras_008546	protein-codi	NZ_CP1252	chromosome	1945368	1946249	-	878	0	14	971	10	903	
QEN71_RS38175	maleylacetate reductase	QEN71_38165	paras_008547	protein-codi	NZ_CP1252	chromosome	1946279	1947346	-	1068	0	19	1857	16	1530	
QEN71_RS38180	aldehyde dehydrogenase family protein	QEN71_38170	paras_008548	protein-codi	NZ_CP1252	chromosome	1947361	1948827	-	1467	0	15	1593	12	1380	
QEN71_RS38185	hydroxyquinol 1,2-dioxygenase	QEN71_38175	paras_008549	protein-codi	NZ_CP1252	chromosome	1948863	1949882	-	1020	0	9	1008	9	1008	
QEN71_RS38190	hydroxyquinol 1,2-dioxygenase	QEN71_38180	paras_008550	protein-codi	NZ_CP1252	chromosome	1949915	1950418	-	504	0	6	673	4	665	
QEN71_RS38195	LysR family transcriptional regulator	QEN71_38185	paras_008551	protein-codi	NZ_CP1252	chromosome	1950525	1951454	-	930	0	7	453	7	453	
QEN71_RS38200	MFS transporter	QEN71_38190	paras_008552	protein-codi	NZ_CP1252	chromosome	1951541	1952821	-	1281	0	12	1085	10	1061	
QEN71_RS38205	hypothetical protein	QEN71_38195	paras_008553	protein-codi	NZ_CP1252	chromosome	1953009	1954781	-	1773	0	26	2228	14	1363	
QEN71_RS38210	flagellar hook protein FlgE	QEN71_38200	paras_008554	protein-codi	NZ_CP1252	chromosome	1954947	1956185	-	1239	0	23	2045	16	1108	
QEN71_RS38215	hypothetical protein	QEN71_38205	paras_008555	protein-codi	NZ_CP1252	chromosome	1956249	1956503	-	255	0	2	68	2	68	
QEN71_RS38220	CPBP family intramembrane metalloprotease	QEN71_38210	paras_008556	protein-codi	NZ_CP1252	chromosome	1957205	1958062	+	858	0	12	1739	10	1283	
QEN71_RS38225	hypothetical protein	QEN71_38215	paras_008557	protein-codi	NZ_CP1252	chromosome	1958234	1959898	+	1665	0	19	1385	16	785	
QEN71_RS38230	chemotaxis protein	QEN71_38220	paras_008558	protein-codi	NZ_CP1252	chromosome	1960090	1961040	+	951	0	9	1352	6	628	
QEN71_RS38235	alkaline phosphatase family protein	QEN71_38225	paras_008559	protein-codi	NZ_CP1252	chromosome	1961323	1962978	+	1656	0	39	3058	36	2371	
QEN71_RS38240	phosphatase PAP2 family protein	QEN71_38230	paras_008560	protein-codi	NZ_CP1252	chromosome	1963213	1963905	+	693	0	37	3673	28	2757	
QEN71_RS38245	2-oxoglutarate dehydrogenase E1 component	QEN71_38235	paras_008561	protein-codi	NZ_CP1252	chromosome	1963958	1966783	-	2826	0	48	5113	34	4011	
QEN71_RS38250	helix-turn-helix transcriptional regulator	QEN71_38240	paras_008562	protein-codi	NZ_CP1252	chromosome	1967127	1967447	-	321	0	3	112	3	112	
QEN71_RS38255	biosynthetic-type acetolactate synthase large subur	QEN71_38245	paras_008563	protein-codi	NZ_CP1252	chromosome	1967464	1969329	-	1866	0	23	1437	15	1180	
QEN71_RS38260	GlxA family transcriptional regulator	QEN71_38250	paras_008564	protein-codi	NZ_CP1252	chromosome	1969631	1970629	+	999	0	9	907	8	805	
QEN71_RS38265	G/U mismatch-specific DNA glycosylase	QEN71_38255	paras_008565	protein-codi	NZ_CP1252	chromosome	1970644	1971198	+	555	0	13	621	9	419	
QEN71_RS38270	LysR family transcriptional regulator	QEN71_38260	paras_008566	protein-codi	NZ_CP1252	chromosome	1971203	1972141	-	939	0	17	1208	14	1063	
QEN71_RS38275	antibiotic biosynthesis monooxygenase	QEN71_38265	paras_008567	protein-codi	NZ_CP1252	chromosome	1972479	1973153	+	675	0	5	264	2	130	
QEN71_RS38280	DUF2964 family protein	QEN71_38270	paras_008568	protein-codi	NZ_CP1252	chromosome	1973233	1973424	+	192	0	1	15	1	15	
QEN71_RS38285	copper chaperone	QEN71_38275	paras_008569	protein-codi	NZ_CP1252	chromosome	1973466	1973666	-	201	0	6	430	5	346	
QEN71_RS38290	MFS transporter	QEN71_38280	paras_008570	protein-codi	NZ_CP1252	chromosome	1974177	1975778	+	1602	0	17	755	13	645	
QEN71_RS38295	HlyD family secretion protein	QEN71_38285	paras_008571	protein-codi	NZ_CP1252	chromosome	1975791	1976867	+	1073	0	10	1082	8	930	
QEN71_RS38300	efflux transporter outer membrane subunit	QEN71_38290	paras_008572	protein-codi	NZ_CP1252	chromosome	1976864	1978405	+	1538	0	15	608	8	404	
QEN71_RS38305	hypothetical protein	QEN71_38295	paras_008573	protein-codi	NZ_CP1252	chromosome	1978487	1978747	+	261	0	7	351	4	247	
QEN71_RS38310	cystathionine gamma-synthase family protein	QEN71_38300	paras_008574	protein-codi	NZ_CP1252	chromosome	1978887	1980170	+	1284	0	19	1580	13	1187	
QEN71_RS38315	TonB-dependent receptor	QEN71_38305	paras_008575	protein-codi	NZ_CP1252	chromosome	1980204	1982300	-	2097	0	67	8222	56	7154	
QEN71_RS38320	sigma 54-interacting transcriptional regulator	QEN71_38310	paras_008576	protein-codi	NZ_CP1252	chromosome	1982682	1985000	+	2308	0	23	3259	19	3080	
QEN71_RS38325	DUF917 family protein	QEN71_38315	paras_008577	protein-codi	NZ_CP1252	chromosome	1984990	1986207	+	1203	0	26	5748	20	4259	
QEN71_RS38330	aspartate/glutamate racemase family protein	QEN71_38320	paras_008578	protein-codi	NZ_CP1252	chromosome	1986204	1986929	+	722	0	16	4163	10	2654	
QEN71_RS38335	hypothetical protein	QEN71_38325		protein-codi	NZ_CP1252	chromosome	1987224	1987352	-	129	0	6	2256	6	2256	
QEN71_RS38340	AraC family transcriptional regulator	QEN71_38330	paras_008579	protein-codi	NZ_CP1252	chromosome	1987622	1988053	+	432	0	16	5203	12	3482	
QEN71_RS38345	CBS domain-containing protein	QEN71_38335	paras_008580	protein-codi	NZ_CP1252	chromosome	1988072	1988527	-	456	0	6	831	1	39	
QEN71_RS38350	thioesterase family protein	QEN71_38340	paras_008581	protein-codi	NZ_CP1252	chromosome	1988732	1989229	+	494	0	18	5857	13	3617	

QEN71_RS38355	sensor histidine kinase	QEN71_38345	paras_008582	protein-codi	NZ_CP1252	chromosom	1989226	1990683	-	1454	0	36	9986	29	7016	
QEN71_RS38360	transcriptional regulator NanR	QEN71_38350	paras_008583	protein-codi	NZ_CP1252	chromosom	1991032	1991736	-	705	0	15	5583	8	3583	
QEN71_RS38365	type II toxin-antitoxin system HipA family toxin	QEN71_38355	paras_008584	protein-codi	NZ_CP1252	chromosom	1991839	1993188	-	1350	0	34	9237	23	7002	
QEN71_RS38370	helix-turn-helix domain-containing protein	QEN71_38360	paras_008585	protein-codi	NZ_CP1252	chromosom	1993191	1993454	-	264	0	5	1063	5	1063	
QEN71_RS38375	four-carbon acid sugar kinase family protein	QEN71_38365	paras_008586	protein-codi	NZ_CP1252	chromosom	1993558	1994940	-	1383	0	13	2157	11	2145	
QEN71_RS38380	ribulose-bisphosphate carboxylase large subunit fa	QEN71_38370	paras_008587	protein-codi	NZ_CP1252	chromosom	1994948	1996192	-	1245	0	20	3586	15	2706	
QEN71_RS38385	phosphogluconate dehydrogenase C-terminal doma	QEN71_38375	paras_008588	protein-codi	NZ_CP1252	chromosom	1996236	1997066	-	831	0	10	1564	8	1518	
QEN71_RS38390	MFS transporter	QEN71_38380	paras_008589	protein-codi	NZ_CP1252	chromosom	1997118	1998497	-	1380	0	19	1727	17	1698	
QEN71_RS38395	hypothetical protein	QEN71_38385	paras_008590	protein-codi	NZ_CP1252	chromosom	1998628	2000577	-	1942	0	19	2329	16	2253	
QEN71_RS38400	Gfo/Idh/MocA family oxidoreductase	QEN71_38390	paras_008591	protein-codi	NZ_CP1252	chromosom	2000570	2001640	-	1063	0	18	2154	13	1334	
QEN71_RS38405	ABC transporter permease	QEN71_38395	paras_008592	protein-codi	NZ_CP1252	chromosom	2001644	2002711	-	1068	0	13	1783	13	1783	
QEN71_RS38410	sugar ABC transporter ATP-binding protein	QEN71_38400	paras_008593	protein-codi	NZ_CP1252	chromosom	2002758	2004314	-	1553	0	16	1150	15	1014	
QEN71_RS38415	DUF2291 domain-containing protein	QEN71_38405	paras_008594	protein-codi	NZ_CP1252	chromosom	2004311	2004958	-	644	0	13	1745	10	1430	
QEN71_RS38420	D-ribose ABC transporter substrate-binding protein	QEN71_38410	paras_008595	protein-codi	NZ_CP1252	chromosom	2005074	2006012	-	939	0	11	2249	11	2249	
QEN71_RS38425	ketopantoate reductase family protein	QEN71_38415	paras_008596	protein-codi	NZ_CP1252	chromosom	2006199	2007134	-	936	0	15	2051	11	1683	
QEN71_RS38430	hypothetical protein	QEN71_38420	paras_008597	protein-codi	NZ_CP1252	chromosom	2007523	2009241	+	1719	0	15	1746	13	1483	
QEN71_RS38435	hypothetical protein	QEN71_38425	paras_008598	protein-codi	NZ_CP1252	chromosom	2009388	2011664	-	2277	0	67	9709	44	5952	
QEN71_RS38440	multidrug effflux MFS transporter	QEN71_38430	paras_008599	protein-codi	NZ_CP1252	chromosom	2012725	2013939	+	1215	0	15	2296	12	1560	
QEN71_RS38445	hypothetical protein	QEN71_38435	paras_008600	protein-codi	NZ_CP1252	chromosom	2013955	2014230	-	276	0	10	903	7	620	
QEN71_RS38450	hypothetical protein	QEN71_38440	paras_008601	protein-codi	NZ_CP1252	chromosom	2014257	2015144	-	888	0	8	325	8	325	
QEN71_RS38455	VOC family protein	QEN71_38445	paras_008602	protein-codi	NZ_CP1252	chromosom	2015449	2015973	-	525	0	12	1156	6	821	
QEN71_RS38460	SDR family oxidoreductase	QEN71_38450	paras_008603	protein-codi	NZ_CP1252	chromosom	2016213	2017004	-	792	0	11	846	8	685	
QEN71_RS38465	MFS transporter	QEN71_38455	paras_008604	protein-codi	NZ_CP1252	chromosom	2017036	2018370	-	1335	0	17	1109	16	843	
QEN71_RS38470	3-keto-5-aminohexanoate cleavage protein	QEN71_38460	paras_008605	protein-codi	NZ_CP1252	chromosom	2018407	2019345	-	939	0	16	1413	10	788	
QEN71_RS38475	AraC family transcriptional regulator	QEN71_38465	paras_008606	protein-codi	NZ_CP1252	chromosom	2019552	2020577	+	1026	0	24	2899	19	2102	
QEN71_RS38480	Na/Pi cotransporter family protein	QEN71_38470	paras_008607	protein-codi	NZ_CP1252	chromosom	2020703	2022397	+	1695	0	15	1761	13	1730	
QEN71_RS38485	ureidoglycolate lyase	QEN71_38475	paras_008608	protein-codi	NZ_CP1252	chromosom	2022484	2023134	+	651	0	9	1253	8	1241	
QEN71_RS38490	GFA family protein	QEN71_38480	paras_008609	protein-codi	NZ_CP1252	chromosom	2023181	2023588	-	408	0	5	836	4	510	
QEN71_RS38495	NAD-dependent succinate-semialdehyde dehydrog	QEN71_38485	paras_008610	protein-codi	NZ_CP1252	chromosom	2023618	2024988	-	1371	0	18	1723	14	1059	
QEN71_RS38500	hypothetical protein	QEN71_38490	paras_008611	protein-codi	NZ_CP1252	chromosom	2025040	2025279	-	236	0	1	76	0	0	
QEN71_RS38505	FAD-binding protein	QEN71_38495	paras_008612	protein-codi	NZ_CP1252	chromosom	2025276	2026220	-	941	111.0	6	258	4	250	
QEN71_RS38510	electron transfer flavoprotein subunit beta/FixA fami	QEN71_38500	paras_008613	protein-codi	NZ_CP1252	chromosom	2026234	2026983	-	746	101.0	5	150	2	34	
QEN71_RS38515	enoyl-CoA hydratase/isomerase family protein	QEN71_38505	paras_008614	protein-codi	NZ_CP1252	chromosom	2026980	2027756	-	769	0	3	130	2	62	TRUE
QEN71_RS38520	hotdog domain-containing protein	QEN71_38510	paras_008615	protein-codi	NZ_CP1252	chromosom	2027753	2028235	-	475	0	6	491	4	320	
QEN71_RS38525	citryl-CoA lyase	QEN71_38515	paras_008616	protein-codi	NZ_CP1252	chromosom	2028232	2029026	-	791	0	11	738	9	535	
QEN71_RS38530	AMP-binding protein	QEN71_38520	paras_008617	protein-codi	NZ_CP1252	chromosom	2029042	2030574	-	1522	0	16	807	12	628	
QEN71_RS38535	acyl-CoA dehydrogenase	QEN71_38525	paras_008618	protein-codi	NZ_CP1252	chromosom	2030564	2031700	-	1126	0	16	1337	16	1337	
QEN71_RS38540	flavin reductase family protein	QEN71_38530	paras_008619	protein-codi	NZ_CP1252	chromosom	2031867	2032517	-	651	0	7	235	5	226	
QEN71_RS38545	MFS transporter	QEN71_38535	paras_008620	protein-codi	NZ_CP1252	chromosom	2032560	2033867	-	1308	0	13	736	12	713	
QEN71_RS38550	RidA family protein	QEN71_38540	paras_008621	protein-codi	NZ_CP1252	chromosom	2033984	2034409	-	426	0	7	676	4	382	
QEN71_RS38555	substrate-binding domain-containing protein	QEN71_38545	paras_008622	protein-codi	NZ_CP1252	chromosom	2034801	2035877	+	1077	0	18	1387	18	1387	
QEN71_RS38560	glucose 1-dehydrogenase	QEN71_38550	paras_008623	protein-codi	NZ_CP1252	chromosom	2035898	2036638	-	741	0	14	1346	12	1314	
QEN71_RS38565	LysR family transcriptional regulator	QEN71_38555	paras_008624	protein-codi	NZ_CP1252	chromosom	2036711	2037634	+	924	0	17	1559	9	611	
QEN71_RS38570	TetR/AcrR family transcriptional regulator	QEN71_38560	paras_008625	protein-codi	NZ_CP1252	chromosom	2037715	2038278	+	564	0	9	386	7	289	
QEN71_RS38575	organic hydroperoxide resistance protein	QEN71_38565	paras_008626	protein-codi	NZ_CP1252	chromosom	2038461	2038886	+	426	0	0	0	0	0	TRUE
QEN71_RS38580	alpha/beta hydrolase	QEN71_38570	paras_008627	protein-codi	NZ_CP1252	chromosom	2038936	2039964	+	1029	0	14	1520	13	1268	
QEN71_RS38585	haloacid dehalogenase type II	QEN71_38575	paras_008628	protein-codi	NZ_CP1252	chromosom	2040019	2040741	-	723	0	9	611	6	547	
QEN71_RS38590	L-fuconate dehydratase	QEN71_38580	paras_008629	protein-codi	NZ_CP1252	chromosom	2040769	2042067	-	1299	0	26	3673	17	2492	
QEN71_RS38595	GntR family transcriptional regulator	QEN71_38585	paras_008630	protein-codi	NZ_CP1252	chromosom	2042241	2043002	+	762	0	16	3267	8	2288	
QEN71_RS38600	sugar ABC transporter ATP-binding protein	QEN71_38590	paras_008631	protein-codi	NZ_CP1252	chromosom	2043064	2044575	+	1512	0	13	1658	12	1592	
QEN71_RS38605	ABC transporter permease	QEN71_38595	paras_008632	protein-codi	NZ_CP1252	chromosom	2044595	2045620	+	1026	0	18	1971	17	1944	
QEN71_RS38610	ABC transporter substrate-binding protein	QEN71_38600	paras_008633	protein-codi	NZ_CP1252	chromosom	2045718	2046659	+	942	0	18	1342	6	747	
QEN71_RS38615	helix-turn-helix domain-containing protein	QEN71_38605	paras_008634	protein-codi	NZ_CP1252	chromosom	2046725	2047690	-	966	0	13	1104	11	1043	
QEN71_RS38620	hypothetical protein	QEN71_38610	paras_008635	protein-codi	NZ_CP1252	chromosom	2047801	2048094	+	294	0	7	1231	6	1118	
QEN71_RS38625	GNAT family N-acetyltransferase	QEN71_38615	paras_008636	protein-codi	NZ_CP1252	chromosom	2048293	2048931	+	639	0	8	989	7	607	
QEN71_RS38630	nucleoside deaminase	QEN71_38620	paras_008637	protein-codi	NZ_CP1252	chromosom	2048969	2049436	-	468	0	13	3128	9	2940	
QEN71_RS38635	methyl-accepting chemotaxis protein	QEN71_38625	paras_008638	protein-codi	NZ_CP1252	chromosom	2049510	2051213	-	1704	0	22	2871	17	2541	
QEN71_RS38640	GFA family protein	QEN71_38630	paras_008639	protein-codi	NZ_CP1252	chromosom	2051361	2051708	+	348	0	14	3493	11	2917	
QEN71_RS38645	MerR family transcriptional regulator	QEN71_38635	paras_008640	protein-codi	NZ_CP1252	chromosom	2051718	2052737	-	1020	0	26	3650	17	2074	
QEN71_RS38650	epoxide hydrolase	QEN71_38640	paras_008641	protein-codi	NZ_CP1252	chromosom	2052857	2054182	-	1326	0	22	1927	18	1183	
QEN71_RS38655	methyl-accepting chemotaxis protein	QEN71_38645	paras_008642	protein-codi	NZ_CP1252	chromosom	2054816	2056375	-	1560	0	14	3135	10	2114	
QEN71_RS38660	succinate dehydrogenase assembly factor 2	QEN71_38650	paras_008643	protein-codi	NZ_CP1252	chromosom	2056647	2056919	-	273	523.0	6	1077	3	226	

QEN71_RS38665	succinate dehydrogenase iron-sulfur subunit	QEN71_38655	paras_008644	protein-codi	NZ_CP1252	chromosom	2057006	2057710	-	705	688.0	13	1802	7	892	
QEN71_RS38670	succinate dehydrogenase flavoprotein subunit	QEN71_38660	paras_008645	protein-codi	NZ_CP1252	chromosom	2057739	2059514	-	1776	3173.0	24	2833	17	1874	
QEN71_RS38675	succinate dehydrogenase, hydrophobic membrane	QEN71_38665	paras_008646	protein-codi	NZ_CP1252	chromosom	2059519	2059887	-	369	731.0	14	2108	9	1821	
QEN71_RS38680	succinate dehydrogenase, cytochrome b556 subuni	QEN71_38670	paras_008647	protein-codi	NZ_CP1252	chromosom	2059891	2060307	-	417	697.0	8	1287	7	1117	
QEN71_RS38685	LysR family transcriptional regulator	QEN71_38675	paras_008648	protein-codi	NZ_CP1252	chromosom	2060507	2061403	+	897	0	7	256	5	251	
QEN71_RS38690	LysE family translocator	QEN71_38680	paras_008649	protein-codi	NZ_CP1252	chromosom	2061432	2062070	-	639	0	15	2185	15	2185	
QEN71_RS38695	LysR substrate-binding domain-containing protein	QEN71_38685	paras_008650	protein-codi	NZ_CP1252	chromosom	2062187	2063038	+	852	0	13	562	13	562	
QEN71_RS38700	NnrU family protein	QEN71_38690	paras_008651	protein-codi	NZ_CP1252	chromosom	2063049	2063627	-	579	0	9	402	7	272	
QEN71_RS38705	DUF1007 family protein	QEN71_38695	paras_008652	protein-codi	NZ_CP1252	chromosom	2063846	2064448	+	596	0	21	3351	19	3071	
QEN71_RS38710	high frequency lysogenization protein HflD	QEN71_38700	paras_008653	protein-codi	NZ_CP1252	chromosom	2064442	2065548	+	1100	0	17	1948	16	1771	
QEN71_RS38715	GGDEF domain-containing protein	QEN71_38705	paras_008654	protein-codi	NZ_CP1252	chromosom	2065568	2066293	-	726	0	7	422	5	244	
QEN71_RS38720	DUF1987 domain-containing protein	QEN71_38710	paras_008655	protein-codi	NZ_CP1252	chromosom	2066298	2066690	-	393	0	5	174	4	119	
QEN71_RS38725	SiaB family protein kinase	QEN71_38715	paras_008656	protein-codi	NZ_CP1252	chromosom	2066709	2067272	-	564	0	6	212	5	63	
QEN71_RS38730	SpoIIE family protein phosphatase	QEN71_38720	paras_008657	protein-codi	NZ_CP1252	chromosom	2067278	2068399	-	1122	0	12	1174	10	943	
QEN71_RS38735	Gfo/Idh/MocA family oxidoreductase	QEN71_38725	paras_008658	protein-codi	NZ_CP1252	chromosom	2068941	2070053	-	1113	0	8	298	6	162	
QEN71_RS38740	porin	QEN71_38730	paras_008659	protein-codi	NZ_CP1252	chromosom	2070114	2071226	-	1113	0	29	1918	27	1720	
QEN71_RS38745	SDR family oxidoreductase	QEN71_38735	paras_008660	protein-codi	NZ_CP1252	chromosom	2071299	2072108	-	806	0	2	140	1	130	
QEN71_RS38750	aldose 1-epimerase	QEN71_38740	paras_008661	protein-codi	NZ_CP1252	chromosom	2072105	2072983	-	875	0	1	4	1	4	TRUE
QEN71_RS38755	aldolase/citrate lyase family protein	QEN71_38745	paras_008662	protein-codi	NZ_CP1252	chromosom	2072986	2073819	-	834	0	6	360	5	358	
QEN71_RS38760	L-arabinonate dehydratase	QEN71_38750	paras_008663	protein-codi	NZ_CP1252	chromosom	2073837	2075579	-	1743	0	16	2823	10	2245	
QEN71_RS38765	sulfite exporter TauE/SafE family protein	QEN71_38755	paras_008664	protein-codi	NZ_CP1252	chromosom	2075585	2076406	-	818	0	5	311	4	302	
QEN71_RS38770	alpha-glucosidase	QEN71_38760	paras_008665	protein-codi	NZ_CP1252	chromosom	2076403	2078433	-	2027	0	30	4433	24	3194	
QEN71_RS38775	solute:sodium symporter family transporter	QEN71_38765	paras_008666	protein-codi	NZ_CP1252	chromosom	2078488	2080236	-	1749	0	33	3956	27	3175	
QEN71_RS38780	NAD-dependent succinate-semialdehyde dehydroge	QEN71_38770	paras_008667	protein-codi	NZ_CP1252	chromosom	2080374	2081840	-	1467	0	20	3406	13	2036	
QEN71_RS38785	SMP-30/gluconolactonase/LRE family protein	QEN71_38775	paras_008668	protein-codi	NZ_CP1252	chromosom	2081864	2082766	-	903	0	23	3233	21	2822	
QEN71_RS38790	LacI family DNA-binding transcriptional regulator	QEN71_38780	paras_008669	protein-codi	NZ_CP1252	chromosom	2082945	2084012	+	1055	0	10	1086	8	691	
QEN71_RS38795	hypothetical protein	QEN71_38785	paras_008670	protein-codi	NZ_CP1252	chromosom	2084000	2084245	-	233	0	5	771	1	193	
QEN71_RS38800	membrane-bound PQQ-dependent dehydrogenase,	QEN71_38790	paras_008671	protein-codi	NZ_CP1252	chromosom	2084695	2087115	+	2421	0	38	5054	23	2962	
QEN71_RS38805	porin	QEN71_38795	paras_008672	protein-codi	NZ_CP1252	chromosom	2087213	2088232	+	1016	0	22	1528	19	1319	
QEN71_RS38810	type II 3-dehydroquinate dehydratase	QEN71_38800	paras_008673	protein-codi	NZ_CP1252	chromosom	2088229	2088675	-	443	0	5	607	3	293	
QEN71_RS38815	MFS transporter	QEN71_38805	paras_008674	protein-codi	NZ_CP1252	chromosom	2088692	2090059	-	1368	0	23	2044	20	1708	
QEN71_RS38820	IclR family transcriptional regulator	QEN71_38810	paras_008675	protein-codi	NZ_CP1252	chromosom	2090270	2091052	+	783	0	9	1134	9	1134	
QEN71_RS38825	aldo/keto reductase	QEN71_38815	paras_008676	protein-codi	NZ_CP1252	chromosom	2091076	2091906	-	831	0	11	830	10	822	
QEN71_RS38830	amidohydrolase family protein	QEN71_38820	paras_008677	protein-codi	NZ_CP1252	chromosom	2091941	2092774	-	834	0	20	2610	18	2373	
QEN71_RS38835	LysR substrate-binding domain-containing protein	QEN71_38825	paras_008678	protein-codi	NZ_CP1252	chromosom	2092893	2093810	+	914	0	11	1761	8	1224	
QEN71_RS38840	SDR family oxidoreductase	QEN71_38830	paras_008679	protein-codi	NZ_CP1252	chromosom	2093807	2094550	-	740	0	5	463	4	430	
QEN71_RS38845	carboxymuconolactone decarboxylase family protei	QEN71_38835	paras_008680	protein-codi	NZ_CP1252	chromosom	2094909	2095367	-	459	0	6	215	6	215	
QEN71_RS38850	NAD(P)H-dependent oxidoreductase	QEN71_38840	paras_008681	protein-codi	NZ_CP1252	chromosom	2095392	2096048	-	653	0	7	438	6	346	
QEN71_RS38855	GNAT family N-acetyltransferase	QEN71_38845	paras_008682	protein-codi	NZ_CP1252	chromosom	2096045	2096482	-	434	0	6	945	6	945	
QEN71_RS38860	PLP-dependent aminotransferase family protein	QEN71_38850	paras_008683	protein-codi	NZ_CP1252	chromosom	2096573	2098027	+	1455	0	18	1428	15	1091	
QEN71_RS38865	Ohr family peroxiredoxin	QEN71_38855	paras_008684	protein-codi	NZ_CP1252	chromosom	2098292	2098807	+	516	0	5	527	2	104	
QEN71_RS38870	DUF3331 domain-containing protein	QEN71_38860	paras_008685	protein-codi	NZ_CP1252	chromosom	2098839	2099249	+	411	0	4	36	3	32	
QEN71_RS38875	alpha/beta hydrolase	QEN71_38865	paras_008686	protein-codi	NZ_CP1252	chromosom	2099266	2100252	+	987	0	13	641	9	471	
QEN71_RS38880	winged helix-turn-helix domain-containing protein	QEN71_38870	paras_008687	protein-codi	NZ_CP1252	chromosom	2100329	2103076	-	2748	0	38	2745	35	2512	
QEN71_RS38885	nuclear transport factor 2 family protein	QEN71_38875	paras_008688	protein-codi	NZ_CP1252	chromosom	2103218	2103592	-	375	0	1	4	0	0	TRUE
QEN71_RS38890	LysR family transcriptional regulator	QEN71_38880	paras_008689	protein-codi	NZ_CP1252	chromosom	2103819	2104835	+	968	0	10	862	6	239	
QEN71_RS38895	TetR/AcrR family transcriptional regulator	QEN71_38885	paras_008690	protein-codi	NZ_CP1252	chromosom	2104787	2105443	-	608	0	5	178	5	178	
QEN71_RS38900	PLP-dependent aminotransferase family protein	QEN71_38890	paras_008691	protein-codi	NZ_CP1252	chromosom	2105791	2107254	+	1464	0	12	1123	11	1102	
QEN71_RS38905	sigma factor-like helix-turn-helix DNA-binding protei	QEN71_38895		pseudogene	NZ_CP1252	chromosom	2107256	2107507	+	252	0	2	139	2	139	
QEN71_RS38910	SDR family oxidoreductase	QEN71_38900	paras_008693	protein-codi	NZ_CP1252	chromosom	2107589	2108332	-	744	0	10	803	5	240	
QEN71_RS38915	LysR family transcriptional regulator	QEN71_38905	paras_008694	protein-codi	NZ_CP1252	chromosom	2108537	2109421	-	885	0	10	854	7	809	
QEN71_RS38920	glucose 1-dehydrogenase	QEN71_38910	paras_008695	protein-codi	NZ_CP1252	chromosom	2109532	2110281	+	750	0	9	573	6	411	
QEN71_RS38925	YbhB/YbcL family Raf kinase inhibitor-like protein	QEN71_38915	paras_008696	protein-codi	NZ_CP1252	chromosom	2110304	2110876	-	573	0	3	503	2	376	
QEN71_RS38930	FAD-dependent oxidoreductase	QEN71_38920	paras_008697	protein-codi	NZ_CP1252	chromosom	2110980	2112185	-	1206	0	7	464	6	457	
QEN71_RS38935	LysR family transcriptional regulator	QEN71_38925	paras_008698	protein-codi	NZ_CP1252	chromosom	2112439	2113341	-	903	0	7	271	2	22	TRUE
QEN71_RS38940	MFS transporter	QEN71_38930	paras_008699	protein-codi	NZ_CP1252	chromosom	2113455	2114660	+	1206	0	16	1208	14	1052	
QEN71_RS38945	2,5-didehydrogluconate reductase DkgB	QEN71_38935	paras_008700	protein-codi	NZ_CP1252	chromosom	2114699	2115505	+	807	0	6	767	5	758	
QEN71_RS38950	alkene reductase	QEN71_38940	paras_008701	protein-codi	NZ_CP1252	chromosom	2115547	2116665	+	1119	0	21	3688	20	3545	
QEN71_RS38955	aldo/keto reductase	QEN71_38945	paras_008702	protein-codi	NZ_CP1252	chromosom	2116678	2117676	+	999	0	25	5523	23	5093	
QEN71_RS38960	DUF4148 domain-containing protein	QEN71_38950	paras_008703	protein-codi	NZ_CP1252	chromosom	2117695	2118369	-	675	0	6	664	4	596	
QEN71_RS38965	glycine betaine/L-proline transporter ProP	QEN71_38955	paras_008704	protein-codi	NZ_CP1252	chromosom	2118777	2120243	+	1467	423.0	39	6362	31	5422	
QEN71_RS38970	PLP-dependent aminotransferase family protein	QEN71_38960	paras_008705	protein-codi	NZ_CP1252	chromosom	2120252	2121586	-	1335	0	21	1448	10	522	

QEN71_RS38975	DJ-1/PfpI family protein	QEN71_38965	paras_008706	protein-codi	NZ_CP1252	chromosom	2121686	2122303	+		618	0	12	1140	8	1061	
QEN71_RS38980	hypothetical protein	QEN71_38970		protein-codi	NZ_CP1252	chromosom	2122408	2122533	-		126	0	1	139	1	139	
QEN71_RS38985	methyl-accepting chemotaxis protein	QEN71_38975	paras_008707	protein-codi	NZ_CP1252	chromosom	2122535	2124427	+		1893	0	28	2274	25	1966	
QEN71_RS38990	glutathione S-transferase	QEN71_38980	paras_008708	protein-codi	NZ_CP1252	chromosom	2124574	2125185	+		612	0	18	2128	13	1020	
QEN71_RS38995	pyridoxamine 5'-phosphate oxidase family protein	QEN71_38985	paras_008709	protein-codi	NZ_CP1252	chromosom	2125256	2127328	+		2073	0	30	6866	26	5629	
QEN71_RS39000	LysR family transcriptional regulator	QEN71_38990	paras_008710	protein-codi	NZ_CP1252	chromosom	2127397	2128302	+		906	0	10	1956	8	1751	
QEN71_RS39005	ATP-binding protein	QEN71_38995	paras_008711	protein-codi	NZ_CP1252	chromosom	2128309	2130081	-		1773	0	22	2216	18	1961	
QEN71_RS39010	MipA/OmpV family protein	QEN71_39000	paras_008712	protein-codi	NZ_CP1252	chromosom	2130258	2131004	-		747	0	25	4188	19	3262	
QEN71_RS39015	alpha/beta fold hydrolase	QEN71_39005	paras_008713	protein-codi	NZ_CP1252	chromosom	2131100	2131990	-		891	0	22	3205	21	3073	
QEN71_RS39020	DUF4118 domain-containing protein	QEN71_39010	paras_008714	protein-codi	NZ_CP1252	chromosom	2131996	2132841	-		846	0	16	3393	14	3242	
QEN71_RS39025	glucose/quinate/shikimate family membrane-bound	QEN71_39015	paras_008715	protein-codi	NZ_CP1252	chromosom	2133190	2135616	+		2427	0	69	14174	42	7412	
QEN71_RS39030	citrate synthase family protein	QEN71_39020	paras_008716	protein-codi	NZ_CP1252	chromosom	2135635	2136768	-		1134	0	23	2847	18	1247	
QEN71_RS39035	MFS transporter	QEN71_39025	paras_008717	protein-codi	NZ_CP1252	chromosom	2136941	2138296	+		1356	0	32	3894	20	2098	
QEN71_RS39040	2-hydroxyacid dehydrogenase	QEN71_39030	paras_008718	protein-codi	NZ_CP1252	chromosom	2138327	2139319	+		993	0	11	1949	10	1798	
QEN71_RS39045	DUF2934 domain-containing protein	QEN71_39035	paras_008719	protein-codi	NZ_CP1252	chromosom	2139401	2139673	+		273	0	5	211	5	211	
QEN71_RS39050	tRNA-Leu	QEN71_39040		tRNA	NZ_CP1252	chromosom	2139787	2139871	+		85	0	2	394	2	394	
QEN71_RS39055	benzoate 1,2-dioxygenase electron transfer compor	QEN71_39045	paras_008721	protein-codi	NZ_CP1252	chromosom	2140381	2143173	+		2793	0	54	3850	44	3198	
QEN71_RS39060	helix-turn-helix domain-containing protein	QEN71_39050	paras_008722	protein-codi	NZ_CP1252	chromosom	2143198	2144133	-		936	0	4	172	4	172	
QEN71_RS39065	ATP-binding protein	QEN71_39055	paras_008723	protein-codi	NZ_CP1252	chromosom	2144304	2146370	-		2067	0	15	1399	10	1156	
QEN71_RS39070	YbdK family carboxylate-amine ligase	QEN71_39060	paras_008724	protein-codi	NZ_CP1252	chromosom	2146621	2147748	-		1128	0	37	4088	27	2609	
QEN71_RS39075	FAD-dependent monooxygenase	QEN71_39065	paras_008725	protein-codi	NZ_CP1252	chromosom	2148106	2149668	+		1563	0	22	3554	17	3249	
QEN71_RS39080	TMEM175 family protein	QEN71_39070	paras_008726	protein-codi	NZ_CP1252	chromosom	2149791	2150450	-		660	0	19	2099	19	2099	
QEN71_RS39085	LysR family transcriptional regulator	QEN71_39075	paras_008727	protein-codi	NZ_CP1252	chromosom	2151183	2152145	-		963	0	14	963	12	826	
QEN71_RS39090	SDR family oxidoreductase	QEN71_39080	paras_008728	protein-codi	NZ_CP1252	chromosom	2152296	2153006	+		711	0	12	673	11	648	
QEN71_RS39095	aldolase	QEN71_39085	paras_008729	protein-codi	NZ_CP1252	chromosom	2153124	2153879	-		756	0	11	873	7	672	
QEN71_RS39100	MFS transporter	QEN71_39090	paras_008730	protein-codi	NZ_CP1252	chromosom	2154114	2155427	+		1314	0	30	2244	20	1470	
QEN71_RS39105	LysR family transcriptional regulator	QEN71_39095	paras_008731	protein-codi	NZ_CP1252	chromosom	2155460	2156347	-		888	0	10	1077	10	1077	
QEN71_RS39110	DeoR/GlpR family DNA-binding transcription regula	QEN71_39100	paras_008732	protein-codi	NZ_CP1252	chromosom	2156496	2157278	+		783	0	8	441	5	72	
QEN71_RS39115	MFS transporter	QEN71_39105	paras_008733	protein-codi	NZ_CP1252	chromosom	2157307	2158443	+		1137	0	9	619	8	617	
QEN71_RS39120	hypothetical protein	QEN71_39110	paras_008734	protein-codi	NZ_CP1252	chromosom	2158727	2159011	+		285	0	9	2021	5	942	
QEN71_RS39125	hypothetical protein	QEN71_39115	paras_008735	protein-codi	NZ_CP1252	chromosom	2159563	2159784	+		185	0	9	3876	9	3876	
QEN71_RS39130	hypothetical protein	QEN71_39120	paras_008736	protein-codi	NZ_CP1252	chromosom	2159748	2160116	+		332	0	15	2974	13	2859	
QEN71_RS39135	30S ribosomal protein S21	QEN71_39125	paras_008737	protein-codi	NZ_CP1252	chromosom	2160228	2160440	-		213	0	13	3284	6	1487	
QEN71_RS39140	hypothetical protein	QEN71_39130	paras_008738	protein-codi	NZ_CP1252	chromosom	2160873	2161097	-		225	0	11	1640	11	1640	
QEN71_RS39145	cold-shock protein	QEN71_39135	paras_008739	protein-codi	NZ_CP1252	chromosom	2161376	2161579	-		204	0	5	371	0	0	
QEN71_RS39150	YncE family protein	QEN71_39140	paras_008740	protein-codi	NZ_CP1252	chromosom	2161876	2162919	-		1044	0	22	3940	19	3420	
QEN71_RS39155	endo alpha-1,4 polygalactosaminidase	QEN71_39145	paras_008741	protein-codi	NZ_CP1252	chromosom	2163214	2164056	+		843	0	10	966	7	707	
QEN71_RS39160	PLP-dependent aminotransferase family protein	QEN71_39150	paras_008742	protein-codi	NZ_CP1252	chromosom	2164100	2165509	-		1410	0	22	1484	20	1455	
QEN71_RS39165	NAD(P)H-dependent oxidoreductase	QEN71_39155	paras_008743	protein-codi	NZ_CP1252	chromosom	2165688	2166338	+		651	0	5	537	4	381	
QEN71_RS39170	DUF3331 domain-containing protein	QEN71_39160	paras_008744	protein-codi	NZ_CP1252	chromosom	2166357	2166641	+		285	0	3	225	2	199	
QEN71_RS39175	SDR family oxidoreductase	QEN71_39165	paras_008745	protein-codi	NZ_CP1252	chromosom	2166655	2167365	+		711	0	13	803	12	783	
QEN71_RS39180	AraC family transcriptional regulator	QEN71_39170	paras_008746	protein-codi	NZ_CP1252	chromosom	2167431	2168387	+		957	0	10	958	7	665	
QEN71_RS39185	nitroreductase family protein	QEN71_39175	paras_008747	protein-codi	NZ_CP1252	chromosom	2168574	2169209	+		636	0	16	1105	14	1068	
QEN71_RS39190	peroxidase-related enzyme	QEN71_39180	paras_008748	protein-codi	NZ_CP1252	chromosom	2169340	2169885	+		546	0	5	635	5	635	
QEN71_RS39195	glutathione S-transferase N-terminal domain-contai	QEN71_39185	paras_008749	protein-codi	NZ_CP1252	chromosom	2170009	2170704	+		696	0	15	1974	14	1960	
QEN71_RS39200	cupin domain-containing protein	QEN71_39190	paras_008750	protein-codi	NZ_CP1252	chromosom	2170766	2171185	+		420	0	11	1087	6	775	
QEN71_RS39205	DUF3788 domain-containing protein	QEN71_39195	paras_008751	protein-codi	NZ_CP1252	chromosom	2171225	2171680	+		456	0	14	1496	14	1496	
QEN71_RS39210	hypothetical protein	QEN71_39200	paras_008752	protein-codi	NZ_CP1252	chromosom	2171868	2172515	-		648	124.0	8	224	6	36	
QEN71_RS39215	hypothetical protein	QEN71_39205	paras_008753	protein-codi	NZ_CP1252	chromosom	2172528	2174906	-		2379	0	75	19484	58	15544	
QEN71_RS39220	type VI secretion system Vgr family protein	partial;pseudo;QEN71_39210		protein-codi	NZ_CP1252	chromosom	2174946	2177474	-		2529	0	116	30150	79	22026	
QEN71_RS39225	IclR family transcriptional regulator C-terminal doma	QEN71_39215	paras_008755	protein-codi	NZ_CP1252	chromosom	2177669	2178511	-		843	0	44	9454	36	8731	
QEN71_RS39230	AMP-binding protein	QEN71_39220	paras_008756	protein-codi	NZ_CP1252	chromosom	2178579	2180120	-		1542	0	32	5724	26	4880	
QEN71_RS39235	ABC transporter permease subunit	QEN71_39225	paras_008757	protein-codi	NZ_CP1252	chromosom	2180148	2181929	-		1782	0	26	2041	23	1664	
QEN71_RS39240	ABC transporter ATP-binding protein	QEN71_39230	paras_008758	protein-codi	NZ_CP1252	chromosom	2181939	2183000	-		1062	0	11	1113	8	1001	
QEN71_RS39245	ABC transporter substrate-binding protein	QEN71_39235	paras_008759	protein-codi	NZ_CP1252	chromosom	2183011	2184072	-		1062	0	17	2584	16	2558	
QEN71_RS39250	enoyl-CoA hydratase/isomerase family protein	QEN71_39240	paras_008760	protein-codi	NZ_CP1252	chromosom	2184127	2184888	-		762	0	6	184	4	124	
QEN71_RS39255	ATP-grasp fold amidoligase family protein	QEN71_39245	paras_008761	protein-codi	NZ_CP1252	chromosom	2185085	2185996	-		912	0	30	3956	26	3592	
QEN71_RS39260	NmrA/HSCARG family protein	QEN71_39250	paras_008762	protein-codi	NZ_CP1252	chromosom	2186262	2187131	-		870	0	17	1407	13	1119	
QEN71_RS39265	LysR family transcriptional regulator	QEN71_39255	paras_008763	protein-codi	NZ_CP1252	chromosom	2187265	2188137	+		873	0	6	324	4	88	
QEN71_RS39270	aminotransferase class V-fold PLP-dependent enzy	QEN71_39260	paras_008764	protein-codi	NZ_CP1252	chromosom	2188188	2189315	-		1128	0	24	3032	15	1605	
QEN71_RS39275	cyclic nucleotide-binding domain-containing thiored	QEN71_39265	paras_008765	protein-codi	NZ_CP1252	chromosom	2189442	2191094	-		1653	0	24	2395	18	1829	
QEN71_RS39280	hypothetical protein	QEN71_39270	paras_008766	protein-codi	NZ_CP1252	chromosom	2191498	2191725	+		228	0	7	402	4	162	

QEN71_RS39285	hypothetical protein	QEN71_39275	paras_008767	protein-codi	NZ_CP1252	chromosom	2191908	2192120	-	213	0	7	999	5	450	
QEN71_RS39290	ABC transporter substrate-binding protein	QEN71_39280	paras_008768	protein-codi	NZ_CP1252	chromosom	2192382	2193116	+	735	0	18	2255	12	1783	
QEN71_RS39295	mandelate racemase/muconate lactonizing enzyme	QEN71_39285	paras_008769	protein-codi	NZ_CP1252	chromosom	2193151	2194350	-	1200	0	23	2105	17	1885	
QEN71_RS39300	CoA ester lyase	QEN71_39290	paras_008770	protein-codi	NZ_CP1252	chromosom	2194423	2195259	-	837	0	1	161	0	0	TRUE
QEN71_RS39305	CaiB/BaiF CoA-transferase family protein	QEN71_39295	paras_008771	protein-codi	NZ_CP1252	chromosom	2195272	2196468	-	1197	0	12	1376	12	1376	
QEN71_RS39310	MaoC family dehydratase N-terminal domain-contai	QEN71_39300	paras_008772	protein-codi	NZ_CP1252	chromosom	2196514	2197368	-	855	0	5	222	4	220	
QEN71_RS39315	acyl-CoA dehydrogenase family protein	QEN71_39305	paras_008773	protein-codi	NZ_CP1252	chromosom	2197384	2198556	-	1173	0	19	1926	7	909	
QEN71_RS39320	LysR substrate-binding domain-containing protein	QEN71_39310	paras_008774	protein-codi	NZ_CP1252	chromosom	2198668	2199597	+	930	0	12	1504	9	1007	
QEN71_RS39325	hypothetical protein	QEN71_39315	paras_008775	protein-codi	NZ_CP1252	chromosom	2199867	2200226	+	360	0	6	449	4	443	
QEN71_RS39330	flagellar hook protein FlgE	QEN71_39320	paras_008776	protein-codi	NZ_CP1252	chromosom	2200283	2201518	+	1236	0	20	1753	13	961	
QEN71_RS39335	hypothetical protein	QEN71_39325	paras_008777	protein-codi	NZ_CP1252	chromosom	2201535	2202161	+	627	0	9	488	3	104	
QEN71_RS39340	LysR family transcriptional regulator	QEN71_39330	paras_008778	protein-codi	NZ_CP1252	chromosom	2202182	2203219	-	1038	0	9	709	7	690	
QEN71_RS39345	response regulator	QEN71_39335	paras_008779	protein-codi	NZ_CP1252	chromosom	2203641	2204015	+	375	0	4	394	2	277	
QEN71_RS39350	response regulator transcription factor	QEN71_39340	paras_008780	protein-codi	NZ_CP1252	chromosom	2204058	2204714	-	657	0	6	289	3	45	
QEN71_RS39355	SDR family oxidoreductase	QEN71_39345	paras_008781	protein-codi	NZ_CP1252	chromosom	2204972	2205727	-	756	0	0	0	0	0	TRUE
QEN71_RS39360	lecithin retinol acyltransferase family protein	QEN71_39350	paras_008782	protein-codi	NZ_CP1252	chromosom	2206005	2206535	+	531	0	2	127	1	11	TRUE
QEN71_RS39365	DHA2 family efflux MFS transporter permease subu	QEN71_39355	paras_008783	protein-codi	NZ_CP1252	chromosom	2206597	2208153	+	1557	0	17	1403	17	1403	
QEN71_RS39370	efflux transporter outer membrane subunit	QEN71_39360	paras_008784	protein-codi	NZ_CP1252	chromosom	2208199	2209689	+	1491	0	22	2363	17	2010	
QEN71_RS39375	HlyD family efflux transporter periplasmic adaptor su	QEN71_39365	paras_008785	protein-codi	NZ_CP1252	chromosom	2209733	2211037	+	1305	0	22	2585	22	2585	
QEN71_RS39380	ATP-binding sensor histidine kinase	QEN71_39370	paras_008786	protein-codi	NZ_CP1252	chromosom	2211234	2216741	+	5508	0	91	10164	76	8258	
QEN71_RS39385	GntR family transcriptional regulator	QEN71_39375	paras_008787	protein-codi	NZ_CP1252	chromosom	2217174	2217908	-	735	0	20	1985	18	1752	
QEN71_RS39390	MmgE/PrpD family protein	QEN71_39380	paras_008788	protein-codi	NZ_CP1252	chromosom	2218217	2219707	+	1491	0	27	2298	26	2229	
QEN71_RS39395	LysR family transcriptional regulator	QEN71_39385	paras_008789	protein-codi	NZ_CP1252	chromosom	2219801	2220715	+	915	0	9	1666	7	1524	
QEN71_RS39400	MFS transporter	QEN71_39390	paras_008790	protein-codi	NZ_CP1252	chromosom	2220728	2222113	-	1386	0	20	1270	15	774	
QEN71_RS39405	GMC family oxidoreductase N-terminal domain-cont	QEN71_39395	paras_008791	protein-codi	NZ_CP1252	chromosom	2222213	2223943	-	1731	0	12	640	9	398	
QEN71_RS39410	enoyl-CoA hydratase-related protein	QEN71_39400	paras_008792	protein-codi	NZ_CP1252	chromosom	2223988	2224794	-	807	0	7	769	5	380	
QEN71_RS39415	AMP-binding protein	QEN71_39405	paras_008793	protein-codi	NZ_CP1252	chromosom	2224800	2226389	-	1582	0	23	1762	16	1178	
QEN71_RS39420	(2Fe-2S)-binding protein	QEN71_39410	paras_008794	protein-codi	NZ_CP1252	chromosom	2226382	2226918	-	525	0	7	568	5	387	
QEN71_RS39425	FAD binding domain-containing protein	QEN71_39415	paras_008795	protein-codi	NZ_CP1252	chromosom	2226915	2227736	-	814	0	3	60	0	0	TRUE
QEN71_RS39430	molybdopterin-dependent oxidoreductase	QEN71_39420	paras_008796	protein-codi	NZ_CP1252	chromosom	2227733	2230804	-	3064	0	34	2955	30	2731	
QEN71_RS39435	UbiX family flavin prenyltransferase	QEN71_39425	paras_008797	protein-codi	NZ_CP1252	chromosom	2230801	2231433	-	621	0	1	3	1	3	TRUE
QEN71_RS39440	UbiD family decarboxylase	QEN71_39430	paras_008798	protein-codi	NZ_CP1252	chromosom	2231426	2232985	-	1552	0	10	676	10	676	
QEN71_RS39445	LysR family transcriptional regulator	QEN71_39435	paras_008799	protein-codi	NZ_CP1252	chromosom	2233282	2234223	+	942	0	9	648	7	301	
QEN71_RS39450	alpha/beta hydrolase	QEN71_39440	paras_008800	protein-codi	NZ_CP1252	chromosom	2234371	2235366	+	996	0	19	1393	17	1233	
QEN71_RS39455	glutathione S-transferase N-terminal domain-contai	QEN71_39445	paras_008801	protein-codi	NZ_CP1252	chromosom	2235421	2236098	+	678	0	19	1626	14	1177	
QEN71_RS39460	DMT family transporter	QEN71_39450	paras_008802	protein-codi	NZ_CP1252	chromosom	2236300	2237178	+	879	0	12	825	9	611	
QEN71_RS39465	glycosyltransferase	QEN71_39455	paras_008803	protein-codi	NZ_CP1252	chromosom	2237189	2238421	-	1233	0	20	3035	18	2507	
QEN71_RS39470	LysR family transcriptional regulator	QEN71_39460	paras_008804	protein-codi	NZ_CP1252	chromosom	2238483	2239400	-	918	0	4	807	4	807	
QEN71_RS39475	MFS transporter	QEN71_39465	paras_008805	protein-codi	NZ_CP1252	chromosom	2239495	2240763	+	1269	0	10	2061	10	2061	
QEN71_RS39480	PAS domain-containing protein	QEN71_39470	paras_008806	protein-codi	NZ_CP1252	chromosom	2241059	2243725	+	2667	0	41	7554	26	4335	
QEN71_RS39485	gas vesicle protein GvpD	QEN71_39475	paras_008807	protein-codi	NZ_CP1252	chromosom	2243778	2245292	+	1515	0	33	4867	20	3022	
QEN71_RS39490	ATP-binding protein	QEN71_39480	paras_008808	protein-codi	NZ_CP1252	chromosom	2245414	2246910	+	1497	0	16	1476	14	1382	
QEN71_RS39495	helix-turn-helix domain-containing protein	QEN71_39485	paras_008809	protein-codi	NZ_CP1252	chromosom	2247145	2248107	+	963	0	23	3828	15	2657	
QEN71_RS39500	hypothetical protein	QEN71_39490	paras_008810	protein-codi	NZ_CP1252	chromosom	2248177	2249265	+	1089	0	27	2960	20	2412	
QEN71_RS39505	ABC transporter ATP-binding protein	QEN71_39495	paras_008811	protein-codi	NZ_CP1252	chromosom	2249306	2251132	-	1827	0	19	1407	11	801	
QEN71_RS39510	hypothetical protein	QEN71_39500	paras_008812	protein-codi	NZ_CP1252	chromosom	2251258	2252532	-	1275	0	42	5799	30	4143	
QEN71_RS39515	alpha/beta fold hydrolase	QEN71_39505	paras_008813	protein-codi	NZ_CP1252	chromosom	2253338	2254690	+	1353	0	17	792	12	529	
QEN71_RS39520	amidohydrolase family protein	QEN71_39510	paras_008814	protein-codi	NZ_CP1252	chromosom	2254776	2255909	-	1130	0	22	2768	16	2089	
QEN71_RS39525	MFS transporter	QEN71_39515	paras_008815	protein-codi	NZ_CP1252	chromosom	2255906	2257213	-	1304	0	23	2147	15	1610	
QEN71_RS39530	CaiB/BaiF CoA-transferase family protein	QEN71_39520	paras_008816	protein-codi	NZ_CP1252	chromosom	2257297	2258565	-	1265	0	23	4615	20	4287	
QEN71_RS39535	hydroxymethylglutaryl-CoA lyase	QEN71_39525	paras_008817	protein-codi	NZ_CP1252	chromosom	2258562	2259542	-	977	0	8	617	5	558	
QEN71_RS39540	IclR family transcriptional regulator	QEN71_39530	paras_008818	protein-codi	NZ_CP1252	chromosom	2259750	2260535	+	786	0	9	871	7	803	
QEN71_RS39545	hypothetical protein	QEN71_39535	paras_008819	protein-codi	NZ_CP1252	chromosom	2260678	2260971	-	294	0	15	1381	13	1138	
QEN71_RS39550	AraC family transcriptional regulator	QEN71_39540	paras_008820	protein-codi	NZ_CP1252	chromosom	2261125	2261958	-	830	0	11	747	9	597	
QEN71_RS39555	NAD(P)H-dependent oxidoreductase	QEN71_39545	paras_008821	protein-codi	NZ_CP1252	chromosom	2261955	2262677	-	711	0	15	1108	13	910	
QEN71_RS39560	enoyl-CoA hydratase-related protein	QEN71_39550	paras_008822	protein-codi	NZ_CP1252	chromosom	2262670	2263404	-	723	0	5	690	5	690	
QEN71_RS39565	DUF2000 domain-containing protein	QEN71_39555	paras_008823	protein-codi	NZ_CP1252	chromosom	2263401	2263937	-	533	0	12	612	6	128	
QEN71_RS39570	catalase family peroxidase	QEN71_39560	paras_008824	protein-codi	NZ_CP1252	chromosom	2264028	2265041	+	1014	0	20	2679	17	2392	
QEN71_RS39575	FAD-dependent oxidoreductase	QEN71_39565	paras_008825	protein-codi	NZ_CP1252	chromosom	2265109	2266635	-	1527	0	27	3800	25	3568	
QEN71_RS39580	argininosuccinate lyase	QEN71_39570	paras_008826	protein-codi	NZ_CP1252	chromosom	2266862	2268361	+	1500	0	31	4511	27	4147	
QEN71_RS39585	gamma-glutamylcyclotransferase family protein	QEN71_39575	paras_008827	protein-codi	NZ_CP1252	chromosom	2268376	2269191	-	816	0	17	2921	14	2279	
QEN71_RS39590	hypothetical protein	QEN71_39580	paras_008828	protein-codi	NZ_CP1252	chromosom	2269315	2271552	-	2238	0	58	6172	50	5312	

QEN71_RS39595	DMT family transporter	QEN71_39585	paras_008829	protein-codi	NZ_CP1252	chromosome	2271738	2272646	-		909	0	30	3521	27	3079
QEN71_RS39600	aromatic ring-hydroxylating dioxygenase subunit alp	QEN71_39590	paras_008830	protein-codi	NZ_CP1252	chromosome	2272783	2274039	-		1257	0	29	2965	17	2456
QEN71_RS39605	LysR substrate-binding domain-containing protein	QEN71_39595	paras_008831	protein-codi	NZ_CP1252	chromosome	2274206	2275123	-		918	0	14	887	10	702
QEN71_RS39610	glycine betaine ABC transporter substrate-binding p	QEN71_39600	paras_008832	protein-codi	NZ_CP1252	chromosome	2275232	2276236	-		1005	0	19	1010	17	699
QEN71_RS39615	glycine betaine/L-proline ABC transporter ATP-bind	QEN71_39605	paras_008833	protein-codi	NZ_CP1252	chromosome	2276443	2277570	-		1120	0	13	1236	10	891
QEN71_RS39620	proline/glycine betaine ABC transporter permease	QEN71_39610	paras_008834	protein-codi	NZ_CP1252	chromosome	2277563	2278450	-		880	0	8	394	4	216
QEN71_RS39625	FAD-binding oxidoreductase	QEN71_39615	paras_008835	protein-codi	NZ_CP1252	chromosome	2278510	2279781	-		1272	0	14	594	13	573
QEN71_RS39630	dihydrodipicolinate synthase family protein	QEN71_39620	paras_008836	protein-codi	NZ_CP1252	chromosome	2279854	2280747	-		894	0	9	653	8	650
QEN71_RS39635	aldehyde dehydrogenase	QEN71_39625	paras_008837	protein-codi	NZ_CP1252	chromosome	2280752	2282251	-		1500	0	9	600	7	264
QEN71_RS39640	GntR family transcriptional regulator	QEN71_39630	paras_008838	protein-codi	NZ_CP1252	chromosome	2282314	2283039	-		726	0	16	2590	12	1893
QEN71_RS39645	hybrid-cluster NAD(P)-dependent oxidoreductase	QEN71_39635	paras_008839	protein-codi	NZ_CP1252	chromosome	2283354	2284484	+		1131	0	16	1048	15	1043
QEN71_RS39650	Xaa-Pro peptidase family protein	QEN71_39640	paras_008840	protein-codi	NZ_CP1252	chromosome	2284552	2285781	+		1230	0	20	1472	16	1214
QEN71_RS39655	dimethylsulfoniopropionate lyase	QEN71_39645	paras_008841	protein-codi	NZ_CP1252	chromosome	2285840	2286430	+		591	0	12	1125	12	1125
QEN71_RS39660	porin	QEN71_39650	paras_008842	protein-codi	NZ_CP1252	chromosome	2286583	2287773	+		1191	0	21	1773	18	1374
QEN71_RS39665	lactonase family protein	QEN71_39655	paras_008843	protein-codi	NZ_CP1252	chromosome	2287796	2288950	-		1155	0	11	1076	9	1054
QEN71_RS39670	helix-turn-helix domain-containing protein	QEN71_39660	paras_008844	protein-codi	NZ_CP1252	chromosome	2289073	2289990	-		918	0	18	1791	10	1109
QEN71_RS39675	porin	QEN71_39665	paras_008845	protein-codi	NZ_CP1252	chromosome	2290251	2291276	-		1026	0	21	2436	11	935
QEN71_RS39680	hypothetical protein	QEN71_39670	paras_008846	protein-codi	NZ_CP1252	chromosome	2291642	2292619	+		978	0	24	2841	17	1780
QEN71_RS39685	methyl-accepting chemotaxis protein	QEN71_39675	paras_008847	protein-codi	NZ_CP1252	chromosome	2292909	2294444	+		1536	0	14	1607	13	1572
QEN71_RS39690	hypothetical protein	QEN71_39680	paras_008848	protein-codi	NZ_CP1252	chromosome	2294529	2296376	-		1848	0	47	5669	33	3585
QEN71_RS39695	phosphate ABC transporter substrate-binding protei	QEN71_39685	paras_008849	protein-codi	NZ_CP1252	chromosome	2296487	2297383	-		897	135.0	10	1056	7	881
QEN71_RS39700	aldolase/citrate lyase family protein	QEN71_39690	paras_008850	protein-codi	NZ_CP1252	chromosome	2297387	2298244	-		858	0	23	2246	19	1833
QEN71_RS39705	protocatechuate 3,4-dioxygenase	QEN71_39695	paras_008851	protein-codi	NZ_CP1252	chromosome	2298285	2299271	-		987	0	26	2480	19	1706
QEN71_RS39710	MFS transporter	QEN71_39700	paras_008852	protein-codi	NZ_CP1252	chromosome	2299351	2300637	-		1287	0	17	1208	15	1057
QEN71_RS39715	ABC transporter substrate-binding protein	QEN71_39705	paras_008853	protein-codi	NZ_CP1252	chromosome	2300772	2301758	-		987	0	14	2748	12	2572
QEN71_RS39720	porin	QEN71_39710	paras_008854	protein-codi	NZ_CP1252	chromosome	2301918	2302943	-		1026	0	33	3583	28	3198
QEN71_RS39725	glutathione S-transferase	QEN71_39715	paras_008855	protein-codi	NZ_CP1252	chromosome	2303399	2304022	-		624	0	11	1326	11	1326
QEN71_RS39730	LysR family transcriptional regulator	QEN71_39720	paras_008856	protein-codi	NZ_CP1252	chromosome	2304171	2305064	+		894	0	3	56	2	31	TRUE
QEN71_RS39735	aconitase family protein	QEN71_39725	paras_008857	protein-codi	NZ_CP1252	chromosome	2305159	2307117	+		1959	0	36	4267	27	3038
QEN71_RS39740	MFS transporter	QEN71_39730	paras_008858	protein-codi	NZ_CP1252	chromosome	2307185	2308627	+		1443	0	31	5018	29	4888
QEN71_RS39745	LysR family transcriptional regulator	QEN71_39735	paras_008859	protein-codi	NZ_CP1252	chromosome	2308672	2309577	-		906	0	20	3308	17	2545
QEN71_RS39755	hydrogenase expression/formation protein HypE	pseudo;QEN71_39745		pseudogene	NZ_CP1252	megaplasmi	51	1045	+		995	0	21	2372	17	2241
QEN71_RS39760	aminocyclopropane-1-carboxylate deaminase/D-cys	partial;pseudo;QEN71_39750		pseudogene	NZ_CP1252	megaplasmi	1149	1409	-		261	0	4	376	4	376
QEN71_RS39765	porin	QEN71_39755	paras_000003	protein-codi	NZ_CP1252	megaplasmi	2080	3249	+		1170	0	38	2204	33	1954
QEN71_RS39770	diaminopropionate ammonia-lyase	partial;pseudo;QEN71_39760		pseudogene	NZ_CP1252	megaplasmi	3427	3687	-		259	0	6	326	5	324
QEN71_RS39775	alcohol dehydrogenase catalytic domain-containing	partial;pseudo;QEN71_39765		pseudogene	NZ_CP1252	megaplasmi	3686	3961	-		274	0	3	353	3	353
QEN71_RS39780	MFS transporter			pseudogene	NZ_CP1252	megaplasmi	4120	4525	-		406	0	11	1016	6	404
QEN71_RS39785	hypothetical protein			protein-codi	NZ_CP1252	megaplasmi	4650	4907	-		258	0	6	562	4	242
QEN71_RS39790	hypothetical protein			protein-codi	NZ_CP1252	megaplasmi	4915	5700	-		786	0	22	3513	14	2509
QEN71_RS39795	iron-sulfur cluster assembly accessory protein	QEN71_39785	paras_000008	protein-codi	NZ_CP1252	megaplasmi	6318	6695	+		378	0	20	4586	15	3615
QEN71_RS39800	IS5 family transposase	QEN71_39790	paras_000009	pseudogene	NZ_CP1252	megaplasmi	6870	7681	-		812	463.0	7	336	7	336
QEN71_RS39805	transposase			protein-codi	NZ_CP1252	megaplasmi	8646	9095	+		353	0	18	2189	17	2150
QEN71_RS39810	transposase			protein-codi	NZ_CP1252	megaplasmi	8999	9868	+		773	0	26	3583	21	3242
QEN71_RS39815	IS5/IS1182 family transposase	partial;pseudo;QEN71_39805		pseudogene	NZ_CP1252	megaplasmi	9881	10042	-		162	0	7	648	3	103
QEN71_RS39820	nitrogenase iron protein	QEN71_39810	paras_000012	protein-codi	NZ_CP1252	megaplasmi	10564	11445	+		882	1360.0	8	781	7	778
QEN71_RS39825	nitrogenase molybdenum-iron protein alpha chain	partial;pseudo;QEN71_39815		pseudogene	NZ_CP1252	megaplasmi	11517	11690	+		174	328.0	0	0	0	0
QEN71_RS39830	EamA family transporter	QEN71_39820	paras_000014	protein-codi	NZ_CP1252	megaplasmi	12162	13070	+		909	0	46	10424	41	8960
QEN71_RS39835	IS5/IS1182 family transposase	partial;pseudo;QEN71_39825		pseudogene	NZ_CP1252	megaplasmi	13690	13870	+		181	0	4	551	4	551
QEN71_RS39840	IS66 family insertion sequence element accessory p	QEN71_39830	paras_000016	protein-codi	NZ_CP1252	megaplasmi	13872	14060	+		189	355.0	3	37	2	35
QEN71_RS39845	IS66 family transposase	partial;pseudo;QEN71_39835		pseudogene	NZ_CP1252	megaplasmi	14124	15446	+		1323	2140.0	9	291	6	35
QEN71_RS39850	site-specific integrase	QEN71_39840	paras_000018	protein-codi	NZ_CP1252	megaplasmi	15649	16527	+		879	1739.0	9	370	6	35
QEN71_RS39855	IS91 family transposase	pseudo;QEN71_39845		pseudogene	NZ_CP1252	megaplasmi	16536	17741	+		1206	2314.0	11	1622	9	890
QEN71_RS39860	site-specific integrase	QEN71_39850	paras_000020	protein-codi	NZ_CP1252	megaplasmi	18228	19460	+		1229	0	42	3619	31	2993
QEN71_RS39865	tyrosine-type recombinase/integrase	QEN71_39855	paras_000021	protein-codi	NZ_CP1252	megaplasmi	19457	20404	+		940	0	24	3101	16	1767
QEN71_RS39870	tyrosine-type recombinase/integrase	QEN71_39860	paras_000022	protein-codi	NZ_CP1252	megaplasmi	20401	21396	+		992	0	14	2279	10	1993
QEN71_RS39875	transposase domain-containing protein	partial;pseudo;QEN71_39865		pseudogene	NZ_CP1252	megaplasmi	21485	21733	+		245	331.0	3	246	1	2
QEN71_RS39880	winged helix-turn-helix transcriptional regulator	QEN71_39870	paras_000024	protein-codi	NZ_CP1252	megaplasmi	21730	21996	-		263	0	5	813	4	683
QEN71_RS39885	IS110 family transposase	pseudo;QEN71_39875		pseudogene	NZ_CP1252	megaplasmi	22242	23380	-		1139	1887.0	14	2280	7	988
QEN71_RS39890	transposase	partial;pseudo;QEN71_39880		pseudogene	NZ_CP1252	megaplasmi	23539	23754	+		216	0	5	737	5	737
QEN71_RS39895	transposase DNA-binding-containing protein			pseudogene	NZ_CP1252	megaplasmi	23815	24657	-		843	1626.0	2	4	2	4
QEN71_RS39900	transposase	QEN71_39890	paras_000028	protein-codi	NZ_CP1252	megaplasmi	24722	25231	+		506	108.0	19	2489	18	2410
QEN71_RS39905	IS66 family insertion sequence element accessory p	QEN71_39895	paras_000029	protein-codi	NZ_CP1252	megaplasmi	25228	25572	+		341	0	2	313	1	6

QEN71_RS39910	IS66 family transposase	pseudo;QEN71_39900		pseudogene	NZ_CP1252	megaplasmi	25603	27153	+	1551	0	46	4898	41	4405	
QEN71_RS39915	IS21-like element helper ATPase IstB	QEN71_39905	paras_000031	protein-codi	NZ_CP1252	megaplasmi	27403	28191	-	789	1561.0	9	302	8	225	
QEN71_RS39920	IS21 family transposase	QEN71_39910	paras_000032	protein-codi	NZ_CP1252	megaplasmi	28198	29721	-	1524	3027.0	16	307	14	298	
QEN71_RS39925	integrase	partial;pseudo;QEN71_39915		pseudogene	NZ_CP1252	megaplasmi	29985	30155	+	171	0	3	248	3	248	
QEN71_RS39930	amino acid adenylation domain-containing protein	QEN71_39920	paras_000033	protein-codi	NZ_CP1252	megaplasmi	30272	44374	-	14103	0	429	128645	334	115987	
QEN71_RS39935	IS5 family transposase	pseudo;QEN71_39925		pseudogene	NZ_CP1252	megaplasmi	44857	45938	-	1082	518.0	23	1603	19	1134	
QEN71_RS39940	helix-turn-helix domain-containing protein	partial;pseudo;QEN71_39930		pseudogene	NZ_CP1252	megaplasmi	46031	46661	+	631	0	20	3021	17	2667	
QEN71_RS39945	IS5/IS1182 family transposase	partial;pseudo;QEN71_39935		pseudogene	NZ_CP1252	megaplasmi	46682	46855	-	174	131.0	4	449	2	122	
QEN71_RS39950	ectoine synthase	QEN71_39940	paras_000036	protein-codi	NZ_CP1252	megaplasmi	47352	47738	-	387	0	17	1311	14	867	
QEN71_RS39955	transposase domain-containing protein	partial;pseudo;QEN71_39945		pseudogene	NZ_CP1252	megaplasmi	48330	48530	-	201	398.0	1	2	1	2	
QEN71_RS39960	transposase domain-containing protein	partial;pseudo;QEN71_39950		pseudogene	NZ_CP1252	megaplasmi	48576	48767	+	192	281.0	4	266	4	266	
QEN71_RS39965	hypothetical protein	QEN71_39955	paras_000039	protein-codi	NZ_CP1252	megaplasmi	48972	49220	+	249	427.0	3	895	3	895	
QEN71_RS39970	transposase	partial;pseudo;QEN71_39960		pseudogene	NZ_CP1252	megaplasmi	49385	49494	-	110	0	0	0	0	0	
QEN71_RS39975	IS3 family transposase	pseudo;QEN71_39965		pseudogene	NZ_CP1252	megaplasmi	49824	50754	+	931	191.0	22	1623	15	842	
QEN71_RS39980	ATP-binding cassette domain-containing protein	QEN71_39970	paras_000042	protein-codi	NZ_CP1252	megaplasmi	50890	52029	-	1116	0	35	3664	24	2628	
QEN71_RS39985	metalloprotease TldD	partial;pseudo;QEN71_39975		pseudogene	NZ_CP1252	megaplasmi	52006	52312	-	283	0	6	1482	5	1461	
QEN71_RS39990	hypothetical protein	QEN71_39980	paras_000044	protein-codi	NZ_CP1252	megaplasmi	52873	53430	-	554	0	10	1036	8	951	
QEN71_RS39995	hypothetical protein	QEN71_39985	paras_000045	protein-codi	NZ_CP1252	megaplasmi	53427	53702	-	272	0	4	412	1	46	
QEN71_RS40000	ATP-binding protein	partial;pseudo;QEN71_39990		pseudogene	NZ_CP1252	megaplasmi	54421	54894	-	474	319.0	7	462	6	405	
QEN71_RS40005	hypothetical protein	QEN71_39995	paras_000047	protein-codi	NZ_CP1252	megaplasmi	55189	55356	+	148	282.0	0	0	0	0	
QEN71_RS40010	recombinase family protein	QEN71_40000	paras_000048	pseudogene	NZ_CP1252	megaplasmi	55337	55510	+	154	289.0	5	531	5	531	
QEN71_RS40015	IS66 family transposase	QEN71_40005	paras_000049	protein-codi	NZ_CP1252	megaplasmi	55552	57117	-	1566	2075.0	29	5074	26	5053	
QEN71_RS40020	IS66 family insertion sequence element accessory p	QEN71_40010	paras_000050	protein-codi	NZ_CP1252	megaplasmi	57181	57516	-	332	659.0	7	1196	7	1196	
QEN71_RS40025	transposase	QEN71_40015	paras_000051	protein-codi	NZ_CP1252	megaplasmi	57513	57905	-	389	255.0	9	1081	8	1070	
QEN71_RS40030	IS5 family transposase	pseudo;QEN71_40020		pseudogene	NZ_CP1252	megaplasmi	58454	59395	-	942	0	17	913	14	871	
QEN71_RS40035	toluene monooxygenase	QEN71_40025	paras_000053	protein-codi	NZ_CP1252	megaplasmi	60262	61764	+	1503	0	35	3292	26	2378	
QEN71_RS40040	toluene-4-monooxygenase system B family protein	QEN71_40030	paras_000054	protein-codi	NZ_CP1252	megaplasmi	61789	62049	+	261	0	2	263	2	263	
QEN71_RS40045	Rieske 2Fe-2S domain-containing protein	QEN71_40035	paras_000055	protein-codi	NZ_CP1252	megaplasmi	62057	62383	+	327	0	4	263	4	263	
QEN71_RS40050	MmoB/DmpM family protein	QEN71_40040	paras_000056	protein-codi	NZ_CP1252	megaplasmi	62403	62717	+	315	0	11	2237	6	775	
QEN71_RS40055	aromatic/alkene monooxygenase hydroxylase subu	QEN71_40045	paras_000057	protein-codi	NZ_CP1252	megaplasmi	62730	63716	+	987	0	28	4943	18	2738	
QEN71_RS40060	shikimate kinase	QEN71_40050	paras_000058	protein-codi	NZ_CP1252	megaplasmi	65044	65619	+	576	0	15	2938	10	2334	
QEN71_RS40065	transposase	pseudo;QEN71_40055		pseudogene	NZ_CP1252	megaplasmi	66534	67610	-	1077	0	37	11329	23	5878	
QEN71_RS40070	IS110 family transposase	QEN71_40060	paras_000060	protein-codi	NZ_CP1252	megaplasmi	68294	69313	+	1020	1173.0	10	3132	8	3058	
QEN71_RS40075	IS110 family transposase	QEN71_40065	paras_000061	protein-codi	NZ_CP1252	megaplasmi	69816	71147	-	1332	2624.0	29	285	22	226	
QEN71_RS40080	IS21 family transposase	QEN71_40070	paras_000062	protein-codi	NZ_CP1252	megaplasmi	71406	72857	+	1448	0	42	5927	39	5687	
QEN71_RS40085	ATP-binding protein	partial;pseudo;QEN71_40075		pseudogene	NZ_CP1252	megaplasmi	72854	73194	+	337	0	2	309	2	309	
QEN71_RS40090	recombinase family protein	QEN71_40080	paras_000064	protein-codi	NZ_CP1252	megaplasmi	73226	75283	-	2038	1220.0	18	921	16	916	
QEN71_RS40095	hypothetical protein	QEN71_40085	paras_000065	protein-codi	NZ_CP1252	megaplasmi	75264	75431	-	148	292.0	0	0	0	0	
QEN71_RS40100	ATP-binding protein	partial;pseudo;QEN71_40090		pseudogene	NZ_CP1252	megaplasmi	75747	75917	+	171	0	0	0	0	0	
QEN71_RS40105	tyrosine-type recombinase/integrase	QEN71_40095	paras_000067	protein-codi	NZ_CP1252	megaplasmi	76003	76614	-	590	909.0	7	859	7	859	
QEN71_RS40110	hypothetical protein	QEN71_40100		protein-codi	NZ_CP1252	megaplasmi	76593	76889	+	249	395.0	2	206	2	206	
QEN71_RS40115	hypothetical protein			protein-codi	NZ_CP1252	megaplasmi	76864	76947	-	58	114.0	2	14	2	14	
QEN71_RS40120	tyrosine-type recombinase/integrase	QEN71_40110	paras_000068	protein-codi	NZ_CP1252	megaplasmi	77025	78002	-	978	1059.0	12	975	11	972	
QEN71_RS40125	tyrosine-type recombinase/integrase	QEN71_40115	paras_000069	protein-codi	NZ_CP1252	megaplasmi	78134	79222	-	1089	1290.0	27	1353	17	828	
QEN71_RS40130	integrase	partial;pseudo;QEN71_40120		pseudogene	NZ_CP1252	megaplasmi	79728	79835	-	108	212.0	0	0	0	0	
QEN71_RS40135	ATP-binding protein	partial;pseudo;QEN71_40125		pseudogene	NZ_CP1252	megaplasmi	80030	80341	+	312	0	7	430	3	79	
QEN71_RS40140	IS110 family transposase	QEN71_40130	paras_000071	protein-codi	NZ_CP1252	megaplasmi	80652	81671	-	1020	1173.0	18	865	13	752	
QEN71_RS40145	HTH-type transcriptional regulator ArgP	QEN71_40135	paras_000072	protein-codi	NZ_CP1252	megaplasmi	82752	83675	-	923	0	10	857	6	383	
QEN71_RS40150	hypothetical protein			protein-codi	NZ_CP1252	megaplasmi	83675	83992	-	317	0	12	725	6	615	
QEN71_RS40155	hypothetical protein	QEN71_40145	paras_000073	protein-codi	NZ_CP1252	megaplasmi	84465	85160	-	696	0	10	762	8	712	
QEN71_RS40160	MmgE/PrpD family protein	QEN71_40150	paras_000074	protein-codi	NZ_CP1252	megaplasmi	85356	86723	-	1368	0	35	2115	29	1707	
QEN71_RS40165	hypothetical protein	QEN71_40155	paras_000075	protein-codi	NZ_CP1252	megaplasmi	86786	87511	-	726	0	9	716	7	641	
QEN71_RS40170	ectoine/hydroxyectoine ABC transporter substrate-b	QEN71_40160	paras_000076	protein-codi	NZ_CP1252	megaplasmi	87540	88412	-	873	0	18	683	13	371	
QEN71_RS40175	PLP-dependent aminotransferase family protein	QEN71_40165	paras_000077	protein-codi	NZ_CP1252	megaplasmi	88917	90137	-	1221	0	30	1544	25	1493	
QEN71_RS40180	aldehyde dehydrogenase family protein	QEN71_40170	paras_000078	protein-codi	NZ_CP1252	megaplasmi	90154	91653	-	1500	0	28	2117	22	1649	
QEN71_RS40185	FAD-binding and (Fe-S)-binding domain-containing	QEN71_40175	paras_000079	protein-codi	NZ_CP1252	megaplasmi	91845	94826	-	2982	0	56	8041	47	6496	
QEN71_RS40190	DUF1338 domain-containing protein	QEN71_40180	paras_000080	protein-codi	NZ_CP1252	megaplasmi	94828	95856	-	1029	0	15	2517	12	1374	
QEN71_RS40195	LysR substrate-binding domain-containing protein	QEN71_40185	paras_000081	protein-codi	NZ_CP1252	megaplasmi	96013	96924	+	912	0	28	3053	24	2684	
QEN71_RS40200	FAD-binding oxidoreductase	QEN71_40190	paras_000082	protein-codi	NZ_CP1252	megaplasmi	97529	98791	-	1263	0	35	3052	28	2451	
QEN71_RS40205	FAD-binding oxidoreductase	QEN71_40195	paras_000083	protein-codi	NZ_CP1252	megaplasmi	99118	100467	-	1350	0	37	2829	26	2079	
QEN71_RS40210	dihydrolipoyl dehydrogenase	QEN71_40200	paras_000084	protein-codi	NZ_CP1252	megaplasmi	100498	102267	-	1770	113.0	19	1555	15	1440	
QEN71_RS40215	dihydrolipoyllysine-residue acetyltransferase	QEN71_40205	paras_000085	protein-codi	NZ_CP1252	megaplasmi	102311	103612	-	1302	0	21	2105	12	1349	

QEN71_RS40220	biosynthetic-type acetolactate synthase large subur	QEN71_40210	paras_000086	protein-codi	NZ_CP1252	megaplasmi	103738	105513	-		1776	0	40	3675	33	3326	
QEN71_RS40225	alpha-ketoglutarate dehydrogenase	QEN71_40215	paras_000087	protein-codi	NZ_CP1252	megaplasmi	105540	108254	-		2715	0	44	3369	36	2730	
QEN71_RS40230	amino acid ABC transporter ATP-binding protein	QEN71_40220	paras_000088	protein-codi	NZ_CP1252	megaplasmi	108664	109443	-		780	1536.0	4	14	4	14	
QEN71_RS40235	ectoine/hydroxyectoine ABC transporter permease s	QEN71_40225	paras_000089	protein-codi	NZ_CP1252	megaplasmi	109472	110131	-		649	1280.0	4	20	3	16	
QEN71_RS40240	amino acid ABC transporter permease	QEN71_40230	paras_000090	protein-codi	NZ_CP1252	megaplasmi	110121	110780	-		649	1279.0	9	65	9	65	
QEN71_RS40245	pyridoxal-phosphate dependent enzyme	QEN71_40235	paras_000091	protein-codi	NZ_CP1252	megaplasmi	111450	112628	-		1175	2329.0	16	85	10	49	
QEN71_RS40250	succinylglutamate desuccinylase/aspartoacylase fa	QEN71_40240	paras_000092	protein-codi	NZ_CP1252	megaplasmi	112625	113641	-		1013	2004.0	11	105	7	24	
QEN71_RS40255	ectoine/hydroxyectoine ABC transporter substrate-b	QEN71_40245	paras_000093	protein-codi	NZ_CP1252	megaplasmi	113733	114575	-		843	1549.0	34	4671	25	4160	
QEN71_RS40260	porin	QEN71_40250	paras_000094	protein-codi	NZ_CP1252	megaplasmi	115015	116166	-		1152	0	56	5330	51	5063	
QEN71_RS40265	amino acid ABC transporter ATP-binding protein	QEN71_40255	paras_000095	protein-codi	NZ_CP1252	megaplasmi	116722	117447	-		726	0	11	991	8	760	
QEN71_RS40270	amino acid ABC transporter permease	QEN71_40260	paras_000096	protein-codi	NZ_CP1252	megaplasmi	117487	118407	-		921	0	25	2554	22	2476	
QEN71_RS40275	transporter substrate-binding domain-containing pro	QEN71_40265	paras_000098	protein-codi	NZ_CP1252	megaplasmi	118979	119782	-		804	0	28	2588	23	1995	
QEN71_RS40280	amino acid ABC transporter ATP-binding protein	QEN71_40270	paras_000099	protein-codi	NZ_CP1252	megaplasmi	120430	121209	-		780	1536.0	15	1088	12	753	
QEN71_RS40285	ectoine/hydroxyectoine ABC transporter permease s	QEN71_40275	paras_000100	protein-codi	NZ_CP1252	megaplasmi	121238	121897	-		649	1280.0	22	2347	16	1627	
QEN71_RS40290	amino acid ABC transporter permease	QEN71_40280	paras_000101	protein-codi	NZ_CP1252	megaplasmi	121887	122546	-		649	1279.0	29	3747	25	3335	
QEN71_RS40295	pyridoxal-phosphate dependent enzyme	QEN71_40285	paras_000102	protein-codi	NZ_CP1252	megaplasmi	123216	124394	-		1175	2329.0	41	8029	27	5065	
QEN71_RS40300	succinylglutamate desuccinylase/aspartoacylase fa	QEN71_40290	paras_000103	protein-codi	NZ_CP1252	megaplasmi	124391	125407	-		1013	2004.0	29	4735	20	2013	
QEN71_RS40305	M24 family metallopeptidase	QEN71_40295	paras_000104	protein-codi	NZ_CP1252	megaplasmi	125428	126624	-		1197	130.0	36	2830	32	2254	
QEN71_RS40310	hypothetical protein	QEN71_40300	paras_000106	protein-codi	NZ_CP1252	megaplasmi	126956	127258	+		303	0	4	547	4	547	
QEN71_RS40315	hypothetical protein	QEN71_40305	paras_000107	protein-codi	NZ_CP1252	megaplasmi	127290	127574	-		285	0	7	676	6	673	
QEN71_RS40320	GntR family transcriptional regulator	QEN71_40310	paras_000108	protein-codi	NZ_CP1252	megaplasmi	127760	128521	-		762	0	50	8910	48	7872	
QEN71_RS40325	ectoine/hydroxyectoine ABC transporter substrate-b	QEN71_40315	paras_000109	protein-codi	NZ_CP1252	megaplasmi	129203	130045	+		843	1549.0	27	4273	21	3061	
QEN71_RS40330	LysR family transcriptional regulator	partial;pseudo;QEN71_40320		pseudogene	NZ_CP1252	megaplasmi	130363	130853	-		491	0	6	885	4	374	
QEN71_RS40335	IS5 family transposase	pseudo;QEN71_40325		pseudogene	NZ_CP1252	megaplasmi	131026	131836	-		811	0	20	2384	16	2214	
QEN71_RS40340	saccharopine dehydrogenase	QEN71_40330	paras_000112	protein-codi	NZ_CP1252	megaplasmi	132441	133526	+		1075	0	19	1525	17	1367	
QEN71_RS40345	ATP-grasp domain-containing protein	QEN71_40335	paras_000113	protein-codi	NZ_CP1252	megaplasmi	133516	134772	+		1246	0	42	2222	37	2033	
QEN71_RS40350	MFS transporter	QEN71_40340	paras_000114	protein-codi	NZ_CP1252	megaplasmi	135272	136999	+		1728	0	62	7805	57	7387	
QEN71_RS40355	IS3 family transposase	QEN71_40345	paras_000115	protein-codi	NZ_CP1252	megaplasmi	137176	137481	-		272	0	9	358	8	331	
QEN71_RS40360	DUF5372 family protein	QEN71_40350	paras_000116	protein-codi	NZ_CP1252	megaplasmi	137448	137684	+		203	0	0	0	0	0	
QEN71_RS40365	helix-turn-helix domain-containing protein	QEN71_40355	paras_000117	protein-codi	NZ_CP1252	megaplasmi	137896	138357	+		462	0	4	1080	4	1080	
QEN71_RS40370	recombinase family protein			protein-codi	NZ_CP1252	megaplasmi	138659	139162	+		504	0	15	2200	13	2147	
QEN71_RS40375	zinc ribbon domain-containing protein			protein-codi	NZ_CP1252	megaplasmi	139340	139921	+		582	0	17	1971	11	1473	
QEN71_RS40380	recombinase family protein			protein-codi	NZ_CP1252	megaplasmi	140062	140589	+		528	0	19	3932	16	2806	
QEN71_RS40385	IS3 family transposase	partial;pseudo;QEN71_40375		pseudogene	NZ_CP1252	megaplasmi	140604	140977	-		363	0	11	1855	9	1772	
QEN71_RS40390	IS21 family transposase	partial;pseudo;QEN71_40380		pseudogene	NZ_CP1252	megaplasmi	140967	141649	+		672	368.0	20	2239	18	2006	
QEN71_RS40395	recombinase family protein	pseudo;QEN71_40385		pseudogene	NZ_CP1252	megaplasmi	141656	142626	-		960	0	36	4719	26	3051	
QEN71_RS40400	hypothetical protein	QEN71_40390	paras_000123	protein-codi	NZ_CP1252	megaplasmi	142616	142804	-		178	0	0	0	0	0	
QEN71_RS40405	helix-turn-helix domain-containing protein	QEN71_40395	paras_000124	protein-codi	NZ_CP1252	megaplasmi	142914	143168	-		226	0	6	371	6	371	
QEN71_RS40410	hypothetical protein	QEN71_40400		protein-codi	NZ_CP1252	megaplasmi	143140	143355	-		187	0	0	0	0	0	
QEN71_RS40415	DUF5372 family protein	QEN71_40405	paras_000125	protein-codi	NZ_CP1252	megaplasmi	143531	143800	-		270	0	8	1115	5	767	
QEN71_RS40420	IS21 family transposase	partial;pseudo;QEN71_40410		pseudogene	NZ_CP1252	megaplasmi	143851	144147	+		286	119.0	8	486	6	251	
QEN71_RS40425	ATP-binding protein	partial;pseudo;QEN71_40415		pseudogene	NZ_CP1252	megaplasmi	144137	144346	+		199	162.0	3	221	3	221	
QEN71_RS40430	helix-turn-helix domain-containing protein	QEN71_40420	paras_000127	protein-codi	NZ_CP1252	megaplasmi	144384	144827	-		444	877.0	12	1505	10	1437	
QEN71_RS40435	recombinase family protein	QEN71_40425		protein-codi	NZ_CP1252	megaplasmi	145032	146441	-		1390	2733.0	48	7533	38	6240	
QEN71_RS40440	hypothetical protein	QEN71_40430	paras_000128	protein-codi	NZ_CP1252	megaplasmi	146422	146589	-		148	292.0	2	17	2	17	
QEN71_RS40445	ATP-binding protein	QEN71_40435	paras_000129	pseudogene	NZ_CP1252	megaplasmi	146896	147359	+		464	142.0	4	829	4	829	
QEN71_RS40450	IS110 family transposase	partial;pseudo;QEN71_40440		pseudogene	NZ_CP1252	megaplasmi	147445	148208	+		764	0	17	2152	15	1898	
QEN71_RS40455	GHMP kinase	QEN71_40445	paras_000131	protein-codi	NZ_CP1252	megaplasmi	148804	149739	+		936	0	32	3328	22	2030	
QEN71_RS40460	pyridoxal-phosphate dependent enzyme	QEN71_40450	paras_000132	protein-codi	NZ_CP1252	megaplasmi	150077	151702	+		1626	0	44	2898	36	2449	
QEN71_RS40465	GntR family transcriptional regulator	QEN71_40455	paras_000133	protein-codi	NZ_CP1252	megaplasmi	152442	153200	+		759	0	27	1937	23	1677	
QEN71_RS40470	hypothetical protein	partial;pseudo;QEN71_40460		pseudogene	NZ_CP1252	megaplasmi	153286	153480	+		195	0	1	25	1	25	
QEN71_RS40475	IS256 family transposase			pseudogene	NZ_CP1252	megaplasmi	153766	154108	-		343	0	4	250	4	250	
QEN71_RS40480	IS110 family transposase	partial;pseudo;QEN71_40470		pseudogene	NZ_CP1252	megaplasmi	154149	154408	+		260	0	3	320	3	320	
QEN71_RS40485	transposase	partial;pseudo;QEN71_40475		pseudogene	NZ_CP1252	megaplasmi	154566	155103	-		538	0	8	1151	4	315	
QEN71_RS40490	IS110 family transposase	QEN71_40480	paras_000138	protein-codi	NZ_CP1252	megaplasmi	155258	156289	+		1032	0	23	1077	17	923	
QEN71_RS40495	IS110 family transposase	QEN71_40485	paras_000139	protein-codi	NZ_CP1252	megaplasmi	156544	157557	+		1014	1997.0	9	47	7	33	
QEN71_RS40500	IS5/IS1182 family transposase	partial;pseudo;QEN71_40490		pseudogene	NZ_CP1252	megaplasmi	157889	158089	-		201	0	4	252	2	93	
QEN71_RS40505	IS110 family transposase	partial;pseudo;QEN71_40495		pseudogene	NZ_CP1252	megaplasmi	158160	158909	+		750	0	18	1247	14	870	
QEN71_RS40510	IS110 family transposase	QEN71_40500	paras_000142	protein-codi	NZ_CP1252	megaplasmi	159148	160161	+		1014	1567.0	8	707	5	262	
QEN71_RS40515	IS1182 family transposase	QEN71_40505	paras_000143	protein-codi	NZ_CP1252	megaplasmi	160566	161978	+		1413	0	34	2909	25	2435	
QEN71_RS40520	IS5 family transposase	pseudo;QEN71_40510		pseudogene	NZ_CP1252	megaplasmi	162138	163411	+		1274	0	25	3180	20	2898	
QEN71_RS40525	IS630 family transposase	QEN71_40515	paras_000145	protein-codi	NZ_CP1252	megaplasmi	163479	164513	+		1035	2041.0	8	105	8	105	

QEN71_RS40530	IS5 family transposase	pseudo;QEN71_40520		pseudogene	NZ_CP1252	megaplasmi	165232	166041	+		810	463.0	7	1490	7	1490
QEN71_RS40535	transposase	partial;pseudo;QEN71_40525		pseudogene	NZ_CP1252	megaplasmi	166865	167651	-		787	0	29	4117	20	2929
QEN71_RS40540	transposase	QEN71_40530	paras_000148	protein-codi	NZ_CP1252	megaplasmi	167683	168036	+		272	171.0	2	233	2	233
QEN71_RS40545	IS66 family transposase	QEN71_40535	paras_000149	protein-codi	NZ_CP1252	megaplasmi	167955	169541	-		1505	2980.0	13	195	10	171
QEN71_RS40550	IS66 family insertion sequence element accessory p	QEN71_40540	paras_000150	protein-codi	NZ_CP1252	megaplasmi	169572	169916	-		341	674.0	3	11	1	3
QEN71_RS40555	transposase	QEN71_40545	paras_000151	protein-codi	NZ_CP1252	megaplasmi	169913	170395	-		479	944.0	11	162	8	144
QEN71_RS40560	NAD(P)/FAD-dependent oxidoreductase	QEN71_40550	paras_000152	protein-codi	NZ_CP1252	megaplasmi	171013	172701	-		1689	0	46	3147	33	1639
QEN71_RS40565	IS5 family transposase	partial;pseudo;QEN71_40555		pseudogene	NZ_CP1252	megaplasmi	173040	173701	-		662	0	11	1057	9	687
QEN71_RS40570	IS6 family transposase	partial;pseudo;QEN71_40560		pseudogene	NZ_CP1252	megaplasmi	174122	174220	-		99	0	0	0	0	0
QEN71_RS40575	cobalamin-dependent protein	QEN71_40565	paras_000154	protein-codi	NZ_CP1252	megaplasmi	174531	176120	+		1589	0	104	21421	74	15310
QEN71_RS40580	LysE family translocator	QEN71_40570	paras_000155	protein-codi	NZ_CP1252	megaplasmi	176120	176731	+		611	0	29	5385	18	3813
QEN71_RS40585	phage integrase family protein	partial;pseudo;QEN71_40575		pseudogene	NZ_CP1252	megaplasmi	176884	177874	+		957	0	18	8018	12	6048
QEN71_RS40590	transposase DNA-binding-containing protein	QEN71_40580	paras_000157	protein-codi	NZ_CP1252	megaplasmi	177841	178155	-		281	0	4	393	4	393
QEN71_RS40595	IS110 family transposase	partial;pseudo;QEN71_40585		pseudogene	NZ_CP1252	megaplasmi	178481	179526	-		1046	259.0	40	7524	27	3951
QEN71_RS40600	IS110 family transposase	partial;pseudo;QEN71_40590		pseudogene	NZ_CP1252	megaplasmi	179660	180570	-		911	1796.0	54	18825	40	14966
QEN71_RS40605	IS66 family insertion sequence element accessory p	QEN71_40595	paras_000160	protein-codi	NZ_CP1252	megaplasmi	181139	181474	+		336	667.0	2	69	2	69
QEN71_RS40610	IS66 family transposase	partial;pseudo;QEN71_40600		pseudogene	NZ_CP1252	megaplasmi	181538	182707	+		1170	965.0	14	1330	12	1046
QEN71_RS40615	ISNCY family transposase	partial;pseudo;QEN71_40605		pseudogene	NZ_CP1252	megaplasmi	182824	183837	+		1014	0	39	8578	36	8209
QEN71_RS40620	hypothetical protein	QEN71_40610	paras_000163	protein-codi	NZ_CP1252	megaplasmi	184308	184685	-		378	0	15	1642	13	1382
QEN71_RS40625	hypothetical protein	QEN71_40615	paras_000164	protein-codi	NZ_CP1252	megaplasmi	187229	187399	+		171	0	9	2623	4	305
QEN71_RS40630	hypothetical protein	QEN71_40620	paras_000165	protein-codi	NZ_CP1252	megaplasmi	187401	187859	+		459	0	21	3057	17	2756
QEN71_RS40635	IS21 family transposase	QEN71_40625	paras_000166	protein-codi	NZ_CP1252	megaplasmi	188407	189900	+		1490	2950.0	10	87	9	40
QEN71_RS40640	IS21-like element helper ATPase IstB	QEN71_40630	paras_000167	protein-codi	NZ_CP1252	megaplasmi	189897	190718	+		818	1618.0	1	2	0	0
QEN71_RS40645	hypothetical protein	QEN71_40635	paras_000168	protein-codi	NZ_CP1252	megaplasmi	190951	191163	-		213	0	5	1355	4	1319
QEN71_RS40650	hypothetical protein	QEN71_40640	paras_000169	protein-codi	NZ_CP1252	megaplasmi	191194	191475	+		282	0	10	3689	10	3689
QEN71_RS40655	transposase	QEN71_40645	paras_000170	protein-codi	NZ_CP1252	megaplasmi	191503	191664	+		162	0	8	1543	5	1379
QEN71_RS40660	hypothetical protein	QEN71_40650	paras_000171	protein-codi	NZ_CP1252	megaplasmi	192262	192480	-		219	0	9	1248	6	1020
QEN71_RS40665	hypothetical protein	QEN71_40655		protein-codi	NZ_CP1252	megaplasmi	192962	193507	+		546	0	23	7577	18	6186
QEN71_RS40670	helix-turn-helix domain-containing protein	QEN71_40660	paras_000172	protein-codi	NZ_CP1252	megaplasmi	193612	193935	+		324	0	10	3071	7	1202
QEN71_RS40675	helix-turn-helix domain-containing protein	QEN71_40665	paras_000173	protein-codi	NZ_CP1252	megaplasmi	194690	195817	+		1128	0	55	16722	43	14601
QEN71_RS40680	glutamine--fructose-6-phosphate transaminase (isor	QEN71_40670	paras_000174	protein-codi	NZ_CP1252	megaplasmi	195866	197683	+		1818	0	94	22845	80	20897
QEN71_RS40685	transposase	partial;pseudo;QEN71_40675		pseudogene	NZ_CP1252	megaplasmi	198048	198548	+		501	0	23	8046	17	7129
QEN71_RS40690	IS630 family transposase	QEN71_40680	paras_000176	protein-codi	NZ_CP1252	megaplasmi	198616	199650	+		1035	2041.0	28	11141	26	10464
QEN71_RS40695	transposase	partial;pseudo;QEN71_40685		pseudogene	NZ_CP1252	megaplasmi	199700	200110	+		411	0	22	5380	22	5380
QEN71_RS40700	hypothetical protein			protein-codi	NZ_CP1252	megaplasmi	200309	200500	-		192	0	7	1023	5	721
QEN71_RS40705	WecB/TagA/CpsF family glycosyltransferase	QEN71_40695	paras_000179	protein-codi	NZ_CP1252	megaplasmi	200748	201521	-		770	0	21	2314	21	2314
QEN71_RS40710	hypothetical protein	QEN71_40700	paras_000180	protein-codi	NZ_CP1252	megaplasmi	201518	202759	-		1234	0	52	4571	50	4470
QEN71_RS40715	heparin lyase I family protein	QEN71_40705	paras_000181	protein-codi	NZ_CP1252	megaplasmi	202756	203595	-		832	0	35	3967	23	2289
QEN71_RS40720	glycosyltransferase	QEN71_40710	paras_000182	protein-codi	NZ_CP1252	megaplasmi	203592	204584	-		985	0	36	3189	27	2573
QEN71_RS40725	hypothetical protein	QEN71_40715	paras_000183	protein-codi	NZ_CP1252	megaplasmi	204581	205297	-		713	0	16	1993	10	927
QEN71_RS40730	lipopolysaccharide biosynthesis protein	QEN71_40720	paras_000184	protein-codi	NZ_CP1252	megaplasmi	205792	207117	-		1326	0	47	4150	35	3331
QEN71_RS40735	UDP-N-acetyl-D-mannosamine dehydrogenase	QEN71_40725	paras_000185	protein-codi	NZ_CP1252	megaplasmi	207164	208411	-		1248	0	29	2883	20	1905
QEN71_RS40740	UDP-N-acetylglucosamine 2-epimerase (non-hydrol	QEN71_40730	paras_000186	protein-codi	NZ_CP1252	megaplasmi	208442	209602	-		1160	0	37	2927	28	2096
QEN71_RS40745	polysaccharide biosynthesis tyrosine autokinase	QEN71_40735	paras_000187	protein-codi	NZ_CP1252	megaplasmi	209602	211857	-		2255	0	62	5226	50	4285
QEN71_RS40750	low molecular weight phosphotyrosine protein phos	QEN71_40740	paras_000188	protein-codi	NZ_CP1252	megaplasmi	211916	212368	-		449	0	8	801	5	399
QEN71_RS40755	polysaccharide biosynthesis/export family protein	QEN71_40745	paras_000189	protein-codi	NZ_CP1252	megaplasmi	212365	213399	-		1031	0	30	2530	18	2225
QEN71_RS40760	undecaprenyl-phosphate glucose phosphotransfera	QEN71_40750	paras_000190	protein-codi	NZ_CP1252	megaplasmi	213564	214943	-		1380	0	27	1658	22	1402
QEN71_RS40765	helix-turn-helix domain-containing protein	QEN71_40755	paras_000191	protein-codi	NZ_CP1252	megaplasmi	215072	216034	-		963	0	9	273	8	252
QEN71_RS40770	dTDP-4-dehydrorhamnose 3,5-epimerase	QEN71_40760	paras_000192	protein-codi	NZ_CP1252	megaplasmi	216540	217121	+		582	0	15	917	9	619
QEN71_RS40775	UTP--glucose-1-phosphate uridylyltransferase GalU	QEN71_40765	paras_000193	protein-codi	NZ_CP1252	megaplasmi	217449	218345	+		897	0	23	1794	18	1247
QEN71_RS40780	transposase	partial;pseudo;QEN71_40770		pseudogene	NZ_CP1252	megaplasmi	218455	218916	-		462	100.0	8	640	7	638
QEN71_RS40785	IS110 family transposase	QEN71_40775	paras_000195	protein-codi	NZ_CP1252	megaplasmi	219047	220063	+		1017	0	25	1901	18	1701
QEN71_RS40790	IS110 family transposase	QEN71_40780	paras_000196	protein-codi	NZ_CP1252	megaplasmi	220322	221335	+		1014	1567.0	34	3174	25	2268
QEN71_RS40795	IS110 family transposase	QEN71_40785	paras_000197	protein-codi	NZ_CP1252	megaplasmi	221712	222728	+		1017	0	26	1926	20	1656
QEN71_RS40800	transposase	partial;pseudo;QEN71_40790		pseudogene	NZ_CP1252	megaplasmi	223066	223547	-		482	514.0	10	1070	9	1042
QEN71_RS40805	nucleotidyl transferase AbiEii/AbiGii toxin family pro	QEN71_40795	paras_000199	protein-codi	NZ_CP1252	megaplasmi	224393	225355	-		959	0	37	5709	31	5244
QEN71_RS40810	type IV toxin-antitoxin system AbiEi family antitoxin	QEN71_40800	paras_000200	protein-codi	NZ_CP1252	megaplasmi	225352	226089	-		734	0	0	0	0	0	TRUE
QEN71_RS40815	hypothetical protein	QEN71_40805	paras_000201	protein-codi	NZ_CP1252	megaplasmi	226292	226435	+		144	0	0	0	0	0
QEN71_RS40820	hypothetical protein	QEN71_40810	paras_000202	protein-codi	NZ_CP1252	megaplasmi	227420	227650	+		231	0	8	2658	3	329
QEN71_RS40825	hypothetical protein	QEN71_40815	paras_000203	protein-codi	NZ_CP1252	megaplasmi	228303	229142	+		836	0	46	9417	35	6354
QEN71_RS40830	hypothetical protein	QEN71_40820	paras_000204	protein-codi	NZ_CP1252	megaplasmi	229139	229789	-		647	0	21	2339	16	1636
QEN71_RS40835	IS110 family transposase	QEN71_40825	paras_000205	protein-codi	NZ_CP1252	megaplasmi	229953	230972	+		1020	1414.0	22	4683	18	4655

QEN71_RS40840	IS21 family transposase	QEN71_40830	paras_000206	protein-codi	NZ_CP1252	megaplasmi	231395	232888	+	1490	0	46	7432	36	5296	
QEN71_RS40845	IS21-like element helper ATPase IstB	QEN71_40835	paras_000207	protein-codi	NZ_CP1252	megaplasmi	232885	233718	+	830	0	21	2184	16	1737	
QEN71_RS40850	tetratricopeptide repeat protein	QEN71_40840	paras_000208	protein-codi	NZ_CP1252	megaplasmi	234491	236440	-	1950	0	55	9494	50	8191	
QEN71_RS40855	hypothetical protein	QEN71_40845	paras_000209	protein-codi	NZ_CP1252	megaplasmi	236822	237385	+	564	0	16	1407	14	1253	
QEN71_RS40860	histidine kinase dimerization/phospho-acceptor dom	QEN71_40850	paras_000210	protein-codi	NZ_CP1252	megaplasmi	237386	237733	+	302	0	3	106	3	106	
QEN71_RS40865	ATP-binding protein	QEN71_40855	paras_000211	protein-codi	NZ_CP1252	megaplasmi	237688	238653	+	920	0	18	1768	16	1652	
QEN71_RS40870	TrbM/KikA/MpfK family conjugal transfer protein	QEN71_40860	paras_000212	protein-codi	NZ_CP1252	megaplasmi	238839	239165	-	327	0	3	202	2	178	
QEN71_RS40875	hypothetical protein	QEN71_40865	paras_000213	protein-codi	NZ_CP1252	megaplasmi	239176	239625	-	449	0	5	319	3	104	
QEN71_RS40880	StbB family protein	QEN71_40870	paras_000214	protein-codi	NZ_CP1252	megaplasmi	239625	240335	-	710	0	13	1254	10	1060	
QEN71_RS40885	hypothetical protein	QEN71_40875	paras_000215	protein-codi	NZ_CP1252	megaplasmi	240404	242194	-	1787	0	28	2702	24	2566	
QEN71_RS40890	type IV secretory system conjugative DNA transfer f	QEN71_40880	paras_000216	protein-codi	NZ_CP1252	megaplasmi	242191	243936	-	1742	0	52	7193	39	4888	
QEN71_RS40895	hypothetical protein	QEN71_40885	paras_000217	protein-codi	NZ_CP1252	megaplasmi	243989	244342	-	354	0	6	331	4	322	
QEN71_RS40900	DUF3717 domain-containing protein	QEN71_40890	paras_000218	protein-codi	NZ_CP1252	megaplasmi	244351	244587	-	237	0	0	0	0	0	
QEN71_RS40905	hypothetical protein	QEN71_40895	paras_000219	protein-codi	NZ_CP1252	megaplasmi	244590	244847	-	254	0	4	299	2	10	
QEN71_RS40910	P-type DNA transfer ATPase VirB11	QEN71_40900	paras_000220	protein-codi	NZ_CP1252	megaplasmi	244844	245881	-	1034	0	8	368	6	319	
QEN71_RS40915	hypothetical protein	QEN71_40905	paras_000221	protein-codi	NZ_CP1252	megaplasmi	245941	246579	-	639	0	18	2901	17	2898	
QEN71_RS40920	TrbI/VirB10 family protein	QEN71_40910	paras_000222	protein-codi	NZ_CP1252	megaplasmi	246599	247954	-	1356	0	25	3540	18	2733	
QEN71_RS40925	TrbG/VirB9 family P-type conjugative transfer protei	QEN71_40915	paras_000223	protein-codi	NZ_CP1252	megaplasmi	247972	248853	-	882	0	22	2944	16	2752	
QEN71_RS40930	type IV secretion system protein	QEN71_40920	paras_000224	protein-codi	NZ_CP1252	megaplasmi	248883	249728	-	846	0	22	2563	17	1883	
QEN71_RS40935	type IV secretion system protein	QEN71_40925	paras_000225	protein-codi	NZ_CP1252	megaplasmi	250040	250954	-	915	0	36	5428	28	3823	
QEN71_RS40940	hypothetical protein	QEN71_40930	paras_000226	protein-codi	NZ_CP1252	megaplasmi	250981	251226	-	235	0	4	253	4	253	
QEN71_RS40945	type IV secretion system protein	QEN71_40935	paras_000227	protein-codi	NZ_CP1252	megaplasmi	251216	251893	-	667	0	20	4339	20	4339	
QEN71_RS40950	transporter	QEN71_40940	paras_000228	protein-codi	NZ_CP1252	megaplasmi	251905	254355	-	2451	0	60	11732	46	9088	
QEN71_RS40955	VirB3 family type IV secretion system protein	QEN71_40945	paras_000229	protein-codi	NZ_CP1252	megaplasmi	254478	254915	-	438	0	6	2567	4	2409	
QEN71_RS40960	hypothetical protein	QEN71_40950	paras_000230	protein-codi	NZ_CP1252	megaplasmi	254925	255275	-	351	0	17	2723	11	1798	
QEN71_RS40965	lytic transglycosylase domain-containing protein	QEN71_40955	paras_000231	protein-codi	NZ_CP1252	megaplasmi	255321	255983	-	663	0	21	6475	21	6475	
QEN71_RS40970	hypothetical protein	QEN71_40960	paras_000232	protein-codi	NZ_CP1252	megaplasmi	256114	256428	-	315	0	11	1925	9	1752	
QEN71_RS40975	5-oxoprolinase subunit PxpA	QEN71_40965	paras_000233	protein-codi	NZ_CP1252	megaplasmi	256527	257315	-	766	0	33	6397	31	6329	
QEN71_RS40980	hypothetical protein	QEN71_40970	paras_000234	protein-codi	NZ_CP1252	megaplasmi	257293	257550	-	231	0	12	2371	12	2371	
QEN71_RS40985	TRAP transporter substrate-binding protein DctP	QEN71_40975	paras_000235	protein-codi	NZ_CP1252	megaplasmi	257547	258503	-	953	0	27	6233	20	5546	
QEN71_RS40990	autoinducer binding domain-containing protein	QEN71_40980	paras_000236	protein-codi	NZ_CP1252	megaplasmi	258749	259792	-	1040	0	39	6734	30	5974	
QEN71_RS40995	EAL domain-containing protein	QEN71_40985	paras_000237	protein-codi	NZ_CP1252	megaplasmi	259789	262053	-	2261	0	71	12825	56	9188	
QEN71_RS41000	ParB/RepB/Spo0J family partition protein	QEN71_40990	paras_000238	protein-codi	NZ_CP1252	megaplasmi	264956	265933	-	974	0	1	4	1	4	TRUE
QEN71_RS41005	ParA family protein	QEN71_40995	paras_000239	protein-codi	NZ_CP1252	megaplasmi	265930	267141	-	1208	0	4	524	1	6	TRUE
QEN71_RS41010	replication initiation protein	QEN71_41000	paras_000240	protein-codi	NZ_CP1252	megaplasmi	267797	269203	+	1407	0	1	11	0	0	TRUE
QEN71_RS41015	transposase	QEN71_41005	paras_000241	protein-codi	NZ_CP1252	megaplasmi	269384	269776	+	389	255.0	8	2406	8	2406	
QEN71_RS41020	IS66 family insertion sequence element accessory p	QEN71_41010	paras_000242	protein-codi	NZ_CP1252	megaplasmi	269773	270108	+	332	659.0	9	2619	8	2600	
QEN71_RS41025	IS66 family transposase	QEN71_41015	paras_000243	protein-codi	NZ_CP1252	megaplasmi	270172	271737	+	1566	1516.0	32	8243	30	7350	
QEN71_RS41030	XRE family transcriptional regulator	QEN71_41020	paras_000244	protein-codi	NZ_CP1252	megaplasmi	272085	272408	-	324	0	18	5966	9	2171	
QEN71_RS41035	hypothetical protein	QEN71_41025	paras_000246	protein-codi	NZ_CP1252	megaplasmi	273265	273573	-	309	0	17	4608	11	2586	
QEN71_RS41040	DUF932 domain-containing protein	QEN71_41030	paras_000247	protein-codi	NZ_CP1252	megaplasmi	273628	274455	-	828	0	35	13307	28	11780	
QEN71_RS41045	antirestriction protein	QEN71_41035	paras_000248	protein-codi	NZ_CP1252	megaplasmi	274508	274921	-	414	0	20	7499	17	7156	
QEN71_RS41050	ParB/RepB/Spo0J family partition protein	QEN71_41040	paras_000249	protein-codi	NZ_CP1252	megaplasmi	275225	277216	+	1992	0	78	26589	64	19469	
QEN71_RS41055	hypothetical protein	QEN71_41045	paras_000250	protein-codi	NZ_CP1252	megaplasmi	277539	278063	-	525	0	8	2478	6	2294	
QEN71_RS41060	hypothetical protein	QEN71_41050	paras_000251	protein-codi	NZ_CP1252	megaplasmi	278451	278660	+	210	0	11	7930	11	7930	
QEN71_RS41065	plasmid mobilization relaxosome protein MobC	QEN71_41055	paras_000252	protein-codi	NZ_CP1252	megaplasmi	278663	279028	+	366	0	12	5801	11	5758	
QEN71_RS41070	hypothetical protein	QEN71_41060		protein-codi	NZ_CP1252	megaplasmi	279062	279223	+	162	0	6	2332	4	1299	
QEN71_RS41075	relaxase/mobilization nuclease domain-containing p	QEN71_41065	paras_000253	protein-codi	NZ_CP1252	megaplasmi	279227	282319	+	3089	0	101	38661	74	30457	
QEN71_RS41080	PH domain-containing protein	QEN71_41070	paras_000254	protein-codi	NZ_CP1252	megaplasmi	282316	282879	+	560	0	31	8463	24	6785	
QEN71_RS41085	hypothetical protein	QEN71_41075	paras_000255	protein-codi	NZ_CP1252	megaplasmi	283253	283600	-	348	0	11	3544	10	3098	
QEN71_RS41090	hypothetical protein	QEN71_41080	paras_000256	protein-codi	NZ_CP1252	megaplasmi	283892	284305	+	414	0	8	1118	4	626	
QEN71_RS41095	H-NS family nucleoid-associated regulatory protein	QEN71_41085	paras_000257	protein-codi	NZ_CP1252	megaplasmi	284365	284700	-	336	0	14	2931	13	2269	
QEN71_RS41100	IS5 family transposase	QEN71_41090	paras_000258	pseudogene	NZ_CP1252	megaplasmi	285210	286162	-	953	0	22	3781	18	3087	
QEN71_RS41105	hypothetical protein	QEN71_41095	paras_000259	protein-codi	NZ_CP1252	megaplasmi	286873	287178	+	306	0	8	511	6	467	
QEN71_RS41110	DUF3320 domain-containing protein	QEN71_41100	paras_000260	protein-codi	NZ_CP1252	megaplasmi	287276	291985	+	4710	0	99	16262	79	13437	
QEN71_RS41115	DUF2130 domain-containing protein	QEN71_41105	paras_000261	protein-codi	NZ_CP1252	megaplasmi	292973	294382	+	1410	0	17	1101	14	997	
QEN71_RS41120	hypothetical protein	QEN71_41110	paras_000262	protein-codi	NZ_CP1252	megaplasmi	294418	295644	+	1227	0	42	3329	36	2836	
QEN71_RS41125	ATP-binding protein	QEN71_41115	paras_000263	protein-codi	NZ_CP1252	megaplasmi	295655	297346	+	1692	0	46	3959	33	2983	
QEN71_RS41130	HU family DNA-binding protein	QEN71_41120	paras_000264	protein-codi	NZ_CP1252	megaplasmi	297675	297953	+	279	0	7	840	4	331	
QEN71_RS41135	DUF1488 family protein	QEN71_41125	paras_000265	protein-codi	NZ_CP1252	megaplasmi	298073	298342	-	270	0	7	1978	6	1919	
QEN71_RS41140	type II toxin-antitoxin system RelE/ParE family toxin	QEN71_41130	paras_000266	protein-codi	NZ_CP1252	megaplasmi	298446	298742	-	293	0	20	4542	20	4542	
QEN71_RS41145	hypothetical protein	QEN71_41135	paras_000267	protein-codi	NZ_CP1252	megaplasmi	298739	299197	-	455	0	4	481	2	389	

QEN71_RS41150	hypothetical protein	QEN71_41140	paras_000268	protein-codi	NZ_CP1252	megaplasmi	299226	299504	-	279	0	9	550	7	478	
QEN71_RS41155	site-specific integrase	QEN71_41145	paras_000269	protein-codi	NZ_CP1252	megaplasmi	299629	301311	-	1683	0	27	2427	15	1408	
QEN71_RS41160	LysR substrate-binding domain-containing protein	QEN71_41150	paras_000270	protein-codi	NZ_CP1252	megaplasmi	301813	302853	-	1041	0	30	2945	27	2806	
QEN71_RS41165	aminotransferase class V-fold PLP-dependent enzy	QEN71_41155	paras_000271	protein-codi	NZ_CP1252	megaplasmi	303279	304481	-	1203	0	38	5213	29	4242	
QEN71_RS41170	MFS transporter	QEN71_41160	paras_000272	protein-codi	NZ_CP1252	megaplasmi	304830	306131	+	1302	0	47	7069	37	5358	
QEN71_RS41175	aspartate/glutamate racemase family protein	QEN71_41165	paras_000273	protein-codi	NZ_CP1252	megaplasmi	306310	307002	+	693	0	16	1337	12	939	
QEN71_RS41180	hydantoinase/oxoprolinase family protein	QEN71_41170	paras_000274	protein-codi	NZ_CP1252	megaplasmi	307184	309283	+	2100	0	49	3831	38	3328	
QEN71_RS41185	hydantoinase B/oxoprolinase family protein	QEN71_41175	paras_000275	protein-codi	NZ_CP1252	megaplasmi	309286	310899	+	1614	0	51	4477	39	2789	
QEN71_RS41190	RidA family protein	QEN71_41180	paras_000276	protein-codi	NZ_CP1252	megaplasmi	310965	311351	+	387	0	5	59	3	27	
QEN71_RS41195	porin	QEN71_41185	paras_000277	protein-codi	NZ_CP1252	megaplasmi	311605	312741	+	1137	0	37	1857	27	1400	
QEN71_RS41200	5-oxoprolinase subunit PxpB	QEN71_41190	paras_000278	protein-codi	NZ_CP1252	megaplasmi	312909	313637	+	725	0	7	599	5	342	
QEN71_RS41205	biotin-dependent carboxyltransferase family protein	QEN71_41195	paras_000279	protein-codi	NZ_CP1252	megaplasmi	313634	314626	+	989	0	27	2588	21	2220	
QEN71_RS41210	acetyl-CoA carboxylase biotin carboxyl carrier prote	QEN71_41200	paras_000280	protein-codi	NZ_CP1252	megaplasmi	314717	315199	+	475	0	14	550	11	395	
QEN71_RS41215	acetyl-CoA carboxylase biotin carboxylase subunit	QEN71_41205	paras_000281	protein-codi	NZ_CP1252	megaplasmi	315192	316556	+	1353	0	32	3615	26	2951	
QEN71_RS41220	hypothetical protein	QEN71_41210	paras_000282	protein-codi	NZ_CP1252	megaplasmi	316553	316978	+	422	0	16	1205	16	1205	
QEN71_RS41225	5-oxoprolinase subunit PxpA	pseudo;QEN71_41215		pseudogene	NZ_CP1252	megaplasmi	317007	317755	+	749	0	18	2087	17	2069	
QEN71_RS41230	LysR substrate-binding domain-containing protein	QEN71_41220	paras_000284	protein-codi	NZ_CP1252	megaplasmi	317900	318823	-	924	0	27	5022	17	3116	
QEN71_RS41235	PLP-dependent lyase/thiolase	QEN71_41225	paras_000285	protein-codi	NZ_CP1252	megaplasmi	319135	320133	+	995	0	24	1952	17	1135	
QEN71_RS41240	ABC transporter ATP-binding protein	QEN71_41230	paras_000286	protein-codi	NZ_CP1252	megaplasmi	320130	321332	+	1199	0	34	1740	33	1731	
QEN71_RS41245	ABC transporter permease	QEN71_41235	paras_000287	protein-codi	NZ_CP1252	megaplasmi	321334	322200	+	867	0	39	2395	33	1800	
QEN71_RS41250	ABC transporter permease	QEN71_41240	paras_000288	protein-codi	NZ_CP1252	megaplasmi	322203	323054	+	852	0	18	1237	12	880	
QEN71_RS41255	extracellular solute-binding protein	QEN71_41245	paras_000289	protein-codi	NZ_CP1252	megaplasmi	323073	324122	+	1050	0	24	1682	19	1540	
QEN71_RS41260	putative hydro-lyase	QEN71_41250	paras_000290	protein-codi	NZ_CP1252	megaplasmi	324152	324949	+	798	0	11	699	7	430	
QEN71_RS41265	Xaa-Pro peptidase family protein	QEN71_41255	paras_000291	protein-codi	NZ_CP1252	megaplasmi	325026	326222	+	1197	0	26	894	18	389	
QEN71_RS41270	tyramine oxidase subunit B	QEN71_41260	paras_000292	protein-codi	NZ_CP1252	megaplasmi	326436	327557	+	1122	0	25	1227	25	1227	
QEN71_RS41275	D-serine ammonia-lyase	QEN71_41265	paras_000293	protein-codi	NZ_CP1252	megaplasmi	328863	330218	+	1356	0	38	2706	30	2386	
QEN71_RS41280	DNA-binding transcriptional regulator DsdC	QEN71_41270	paras_000294	protein-codi	NZ_CP1252	megaplasmi	330294	331235	+	942	0	28	2587	20	1728	
QEN71_RS41285	aldehyde dehydrogenase	QEN71_41275	paras_000295	protein-codi	NZ_CP1252	megaplasmi	331382	332881	-	1500	0	26	2405	20	1966	
QEN71_RS41290	dihydrodipicolinate synthase family protein	QEN71_41280	paras_000296	protein-codi	NZ_CP1252	megaplasmi	332909	333820	-	912	0	17	1776	12	1088	
QEN71_RS41295	LysR family transcriptional regulator	QEN71_41285	paras_000297	protein-codi	NZ_CP1252	megaplasmi	334218	335123	-	906	0	26	3757	17	2495	
QEN71_RS41300	3-hydroxyacyl-CoA dehydrogenase	QEN71_41290	paras_000298	protein-codi	NZ_CP1252	megaplasmi	335325	336278	+	954	0	16	2651	16	2651	
QEN71_RS41305	FAD-dependent oxidoreductase	QEN71_41295	paras_000299	protein-codi	NZ_CP1252	megaplasmi	336302	337591	+	1290	0	18	1215	14	979	
QEN71_RS41310	proline racemase family protein	QEN71_41300	paras_000300	protein-codi	NZ_CP1252	megaplasmi	337622	338653	+	1032	0	22	1999	20	1703	
QEN71_RS41315	MFS transporter	QEN71_41305	paras_000301	protein-codi	NZ_CP1252	megaplasmi	338708	340003	+	1296	0	25	1424	17	1086	
QEN71_RS41320	amidohydrolase family protein	QEN71_41310	paras_000302	protein-codi	NZ_CP1252	megaplasmi	340031	341275	+	1245	0	28	1382	18	933	
QEN71_RS41325	cupin domain-containing protein	QEN71_41315	paras_000303	protein-codi	NZ_CP1252	megaplasmi	341353	341880	+	524	0	11	791	11	791	
QEN71_RS41330	alpha/beta hydrolase	QEN71_41320	paras_000304	protein-codi	NZ_CP1252	megaplasmi	341877	342656	+	772	0	12	692	9	642	
QEN71_RS41335	aspartate dehydrogenase	QEN71_41325	paras_000305	protein-codi	NZ_CP1252	megaplasmi	342653	343459	+	803	0	13	863	12	781	
QEN71_RS41340	porin	QEN71_41330	paras_000306	protein-codi	NZ_CP1252	megaplasmi	343714	344886	+	1173	0	39	3282	32	2671	
QEN71_RS41345	branched-chain amino acid ABC transporter substra	QEN71_41335	paras_000307	protein-codi	NZ_CP1252	megaplasmi	345058	346209	+	1152	0	40	3493	35	2836	
QEN71_RS41350	SDR family NAD(P)-dependent oxidoreductase	QEN71_41340	paras_000308	protein-codi	NZ_CP1252	megaplasmi	346729	346953	+	225	0	4	238	4	238	
QEN71_RS41355	branched-chain amino acid ABC transporter substra	QEN71_41345	paras_000309	protein-codi	NZ_CP1252	megaplasmi	347537	348673	+	1137	0	29	3584	23	3093	
QEN71_RS41360	Lrp/AsnC family transcriptional regulator	QEN71_41350	paras_000310	protein-codi	NZ_CP1252	megaplasmi	348678	349154	-	477	0	14	2903	14	2903	
QEN71_RS41365	M20 aminoacylase family protein	QEN71_41355	paras_000311	protein-codi	NZ_CP1252	megaplasmi	349276	350439	+	1164	0	19	1387	9	516	
QEN71_RS41370	diaminopropionate ammonia-lyase	QEN71_41360	paras_000312	protein-codi	NZ_CP1252	megaplasmi	350587	351816	+	1230	0	35	6183	24	4564	
QEN71_RS41375	APC family permease	QEN71_41365	paras_000313	protein-codi	NZ_CP1252	megaplasmi	351937	353571	+	1635	0	80	13127	69	12395	
QEN71_RS41380	GntR family transcriptional regulator	QEN71_41370	paras_000314	protein-codi	NZ_CP1252	megaplasmi	353690	354595	-	906	0	23	3266	19	2699	
QEN71_RS41385	aconitate hydratase AcnA	QEN71_41375	paras_000315	protein-codi	NZ_CP1252	megaplasmi	354649	357273	-	2625	0	59	12735	45	9155	
QEN71_RS41390	lactonase family protein	QEN71_41380	paras_000316	protein-codi	NZ_CP1252	megaplasmi	357533	358594	+	1062	0	19	3889	14	2967	
QEN71_RS41395	MFS transporter	QEN71_41385	paras_000317	protein-codi	NZ_CP1252	megaplasmi	358650	359984	+	1335	0	37	6400	32	5572	
QEN71_RS41400	porin	partial;pseudo;QEN71_41390		pseudogene	NZ_CP1252	megaplasmi	360116	361290	+	1175	0	45	4870	36	3876	
QEN71_RS41405	transporter substrate-binding domain-containing pro	QEN71_41395	paras_000319	protein-codi	NZ_CP1252	megaplasmi	361876	362610	+	735	0	23	3794	21	3730	
QEN71_RS41410	antibiotic biosynthesis monooxygenase family prote	QEN71_41400	paras_000320	protein-codi	NZ_CP1252	megaplasmi	362642	362935	+	294	0	9	1447	7	1343	
QEN71_RS41415	hypothetical protein	QEN71_41405		protein-codi	NZ_CP1252	megaplasmi	363059	363253	-	195	0	8	316	3	93	
QEN71_RS41420	C4-dicarboxylate transporter DctA	QEN71_41410	paras_000321	protein-codi	NZ_CP1252	megaplasmi	363317	364654	-	1338	0	44	7708	37	6660	
QEN71_RS41425	aspartate/glutamate racemase family protein	QEN71_41415	paras_000322	protein-codi	NZ_CP1252	megaplasmi	364749	365411	-	663	0	17	3672	13	3291	
QEN71_RS41430	GntR family transcriptional regulator	QEN71_41420	paras_000323	protein-codi	NZ_CP1252	megaplasmi	365605	366546	+	942	0	32	8516	28	7743	
QEN71_RS41435	porin	QEN71_41425	paras_000324	protein-codi	NZ_CP1252	megaplasmi	366862	367962	+	1101	0	44	7385	32	5690	
QEN71_RS41440	pyruvate kinase	QEN71_41430	paras_000325	protein-codi	NZ_CP1252	megaplasmi	367993	369468	+	1472	0	26	3698	22	3510	
QEN71_RS41445	NADP-dependent glyceraldehyde-3-phosphate deh	QEN71_41435	paras_000326	protein-codi	NZ_CP1252	megaplasmi	369465	371090	+	1622	0	30	3999	27	3221	
QEN71_RS41450	MFS transporter	QEN71_41440	paras_000327	protein-codi	NZ_CP1252	megaplasmi	371152	372486	+	1335	0	31	4447	28	4252	
QEN71_RS41455	isocitrate/isopropylmalate dehydrogenase family pro	QEN71_41445	paras_000328	protein-codi	NZ_CP1252	megaplasmi	372757	373827	+	1071	0	21	1395	17	776	

QEN71_RS41460	amidohydrolase family protein	QEN71_41450	paras_000329	protein-codi	NZ_CP1252	megaplasmi	373867	374895	+	1029	0	18	1558	14	1232	
QEN71_RS41465	four-carbon acid sugar kinase family protein	QEN71_41455	paras_000330	protein-codi	NZ_CP1252	megaplasmi	374915	376189	+	1275	0	20	2546	15	1944	
QEN71_RS41470	transposase	QEN71_41460	paras_000331	protein-codi	NZ_CP1252	megaplasmi	376439	377779	-	1341	0	26	1691	22	1509	
QEN71_RS41475	IS110 family transposase	partial;pseudo;QEN71_41465		protein-codi	NZ_CP1252	megaplasmi	377958	378812	+	824	0	22	1323	18	1029	
QEN71_RS41480	IS4 family transposase	QEN71_41470	paras_000333	protein-codi	NZ_CP1252	megaplasmi	378782	380215	-	1403	2772.0	19	244	16	227	
QEN71_RS41485	IS110 family transposase	QEN71_41475	paras_000334	protein-codi	NZ_CP1252	megaplasmi	380763	381776	+	1014	1993.0	10	84	8	24	
QEN71_RS41490	DMT family transporter	QEN71_41480	paras_000335	protein-codi	NZ_CP1252	megaplasmi	382187	383086	+	900	0	23	1983	16	1377	
QEN71_RS41495	cobalamin biosynthesis protein CobT	QEN71_41485	paras_000336	protein-codi	NZ_CP1252	megaplasmi	383258	385000	-	1743	0	30	3021	21	2399	
QEN71_RS41500	AAA family ATPase	QEN71_41490	paras_000337	protein-codi	NZ_CP1252	megaplasmi	385003	386208	-	1206	0	29	4318	21	2522	
QEN71_RS41505	aldolase	QEN71_41495	paras_000338	protein-codi	NZ_CP1252	megaplasmi	386250	386888	-	639	0	30	4212	25	4073	
QEN71_RS41510	phosphopyruvate hydratase	QEN71_41500	paras_000339	protein-codi	NZ_CP1252	megaplasmi	387032	388318	+	1287	329.0	18	2450	8	959	
QEN71_RS41515	triose-phosphate isomerase	QEN71_41505	paras_000340	protein-codi	NZ_CP1252	megaplasmi	388398	389171	+	774	0	20	1247	14	820	
QEN71_RS41520	hypothetical protein	QEN71_41510	paras_000341	protein-codi	NZ_CP1252	megaplasmi	389320	389715	+	396	0	11	1046	10	1039	
QEN71_RS41525	transposase	partial;pseudo;QEN71_41515		pseudogene	NZ_CP1252	megaplasmi	390581	391012	-	432	0	8	1032	8	1032	
QEN71_RS41530	porin	QEN71_41520	paras_000344	protein-codi	NZ_CP1252	megaplasmi	391313	392488	-	1176	0	43	4851	41	4727	
QEN71_RS41535	cupin domain-containing protein	QEN71_41525	paras_000345	protein-codi	NZ_CP1252	megaplasmi	392752	393876	-	1125	0	11	1351	8	1046	
QEN71_RS41540	aminotransferase class V-fold PLP-dependent enzy	QEN71_41530	paras_000346	protein-codi	NZ_CP1252	megaplasmi	394000	395133	-	1134	0	10	950	6	209	
QEN71_RS41545	MFS transporter	QEN71_41535	paras_000347	protein-codi	NZ_CP1252	megaplasmi	395137	396429	-	1293	0	30	3311	23	2556	
QEN71_RS41550	M20 family metallopeptidase	QEN71_41540	paras_000348	protein-codi	NZ_CP1252	megaplasmi	396463	397851	-	1389	0	19	2137	16	1952	
QEN71_RS41555	LysR family transcriptional regulator	QEN71_41545	paras_000349	protein-codi	NZ_CP1252	megaplasmi	397974	398897	+	924	0	19	2101	13	1475	
QEN71_RS41560	hypothetical protein	QEN71_41550	paras_000350	protein-codi	NZ_CP1252	megaplasmi	399275	399583	-	308	0	8	502	7	340	
QEN71_RS41565	hypothetical protein			pseudogene	NZ_CP1252	megaplasmi	399583	399945	-	361	0	3	320	3	320	
QEN71_RS41570	divalent metal cation transporter	pseudo;QEN71_41560		pseudogene	NZ_CP1252	megaplasmi	399945	401158	-	1213	0	32	4923	28	4378	
QEN71_RS41575	alpha/beta fold hydrolase	QEN71_41565	paras_000353	protein-codi	NZ_CP1252	megaplasmi	401819	402739	+	917	0	22	5536	15	3185	
QEN71_RS41580	hypothetical protein	QEN71_41570	paras_000354	protein-codi	NZ_CP1252	megaplasmi	402736	404151	+	1408	0	35	6253	33	6000	
QEN71_RS41585	alpha/beta hydrolase	QEN71_41575	paras_000355	protein-codi	NZ_CP1252	megaplasmi	404148	405050	+	899	0	29	7149	24	6317	
QEN71_RS41590	response regulator receiver protein	QEN71_41580	paras_000356	protein-codi	NZ_CP1252	megaplasmi	405140	405799	-	660	0	13	2442	11	1804	
QEN71_RS41595	hypothetical protein	QEN71_41585	paras_000357	protein-codi	NZ_CP1252	megaplasmi	406233	406541	+	309	0	5	1673	4	1656	
QEN71_RS41600	hypothetical protein	QEN71_41590	paras_000358	protein-codi	NZ_CP1252	megaplasmi	406568	406870	+	303	0	19	4080	18	4041	
QEN71_RS41605	hypothetical protein	QEN71_41595	paras_000359	protein-codi	NZ_CP1252	megaplasmi	407154	407306	+	153	0	15	3271	11	1655	
QEN71_RS41610	DUF4304 domain-containing protein	QEN71_41600	paras_000360	protein-codi	NZ_CP1252	megaplasmi	407479	407889	+	411	0	21	4957	18	4761	
QEN71_RS41615	hypothetical protein	QEN71_41605	paras_000361	protein-codi	NZ_CP1252	megaplasmi	408011	408292	+	282	0	8	1299	4	852	
QEN71_RS41620	hypothetical protein	QEN71_41610	paras_000362	protein-codi	NZ_CP1252	megaplasmi	408451	408807	+	357	441.0	19	9009	13	6333	
QEN71_RS41625	hypothetical protein	QEN71_41615	paras_000363	protein-codi	NZ_CP1252	megaplasmi	409048	409338	+	291	573.0	20	12699	17	11009	
QEN71_RS41630	type VI secretion system Vgr family protein	QEN71_41620	paras_000364	protein-codi	NZ_CP1252	megaplasmi	409791	412343	+	2553	5040.0	90	34363	69	27001	
QEN71_RS41635	DUF4123 domain-containing protein	QEN71_41625	paras_000365	protein-codi	NZ_CP1252	megaplasmi	412352	413155	+	804	1536.0	27	11924	26	11917	
QEN71_RS41640	DUF3304 domain-containing protein	QEN71_41630	paras_000366	protein-codi	NZ_CP1252	megaplasmi	413203	413649	+	447	877.0	24	6385	21	5613	
QEN71_RS41645	DUF2235 domain-containing protein	QEN71_41635	paras_000367	protein-codi	NZ_CP1252	megaplasmi	413807	415252	+	1446	2757.0	47	20233	38	14124	
QEN71_RS41650	hypothetical protein	QEN71_41640	paras_000368	protein-codi	NZ_CP1252	megaplasmi	415452	415877	+	426	835.0	11	2410	11	2410	
QEN71_RS41655	hypothetical protein	QEN71_41645	paras_000369	protein-codi	NZ_CP1252	megaplasmi	416309	416611	+	303	501.0	12	2619	7	1634	
QEN71_RS41660	hypothetical protein	QEN71_41650	paras_000370	protein-codi	NZ_CP1252	megaplasmi	416789	417733	+	945	1866.0	19	7107	16	6657	
QEN71_RS41665	hypothetical protein	QEN71_41655	paras_000371	protein-codi	NZ_CP1252	megaplasmi	418033	418389	-	357	441.0	16	3381	12	2661	
QEN71_RS41670	IS1182 family transposase	QEN71_41660	paras_000372	protein-codi	NZ_CP1252	megaplasmi	418645	420090	-	1446	1288.0	44	6383	30	4162	
QEN71_RS41675	hypothetical protein	QEN71_41665	paras_000373	protein-codi	NZ_CP1252	megaplasmi	420349	420669	-	321	0	5	200	3	64	
QEN71_RS41680	hypothetical protein	QEN71_41670	paras_000374	protein-codi	NZ_CP1252	megaplasmi	420964	421305	-	342	0	3	81	3	81	
QEN71_RS41685	hypothetical protein	QEN71_41675	paras_000375	protein-codi	NZ_CP1252	megaplasmi	421426	421719	-	294	0	9	447	9	447	
QEN71_RS41690	hypothetical protein	QEN71_41680		protein-codi	NZ_CP1252	megaplasmi	421781	422176	-	396	0	16	2171	9	688	
QEN71_RS41695	GNAT family N-acetyltransferase	QEN71_41685	paras_000376	protein-codi	NZ_CP1252	megaplasmi	422500	422934	+	435	0	22	3858	18	3413	
QEN71_RS41700	LysE family transporter	QEN71_41690	paras_000377	protein-codi	NZ_CP1252	megaplasmi	423031	423648	+	618	0	24	3349	19	2579	
QEN71_RS41705	VOC family protein	QEN71_41695	paras_000378	protein-codi	NZ_CP1252	megaplasmi	423837	424220	-	384	0	14	1604	13	1587	
QEN71_RS41710	lipocalin-like domain-containing protein	QEN71_41700	paras_000379	protein-codi	NZ_CP1252	megaplasmi	424370	424795	-	426	0	30	6340	27	5648	
QEN71_RS41715	hypothetical protein	QEN71_41705	paras_000380	protein-codi	NZ_CP1252	megaplasmi	425170	425817	-	648	124.0	26	2951	22	2633	
QEN71_RS41720	hypothetical protein	QEN71_41710	paras_000381	protein-codi	NZ_CP1252	megaplasmi	425830	426141	-	312	0	15	2370	9	1159	
QEN71_RS41725	SET domain-containing protein-lysine N-methyltrans	QEN71_41715	paras_000382	protein-codi	NZ_CP1252	megaplasmi	426283	426750	-	468	0	11	2576	8	1575	
QEN71_RS41730	HU family DNA-binding protein	partial;pseudo;QEN71_41720		protein-codi	NZ_CP1252	megaplasmi	427051	427329	+	279	0	2	441	0	0	
QEN71_RS41735	DUF1488 family protein	QEN71_41725	paras_000384	protein-codi	NZ_CP1252	megaplasmi	427446	427715	-	270	0	4	689	2	197	
QEN71_RS41740	tyrosine-type recombinase/integrase	QEN71_41730	paras_000385	protein-codi	NZ_CP1252	megaplasmi	427843	429111	-	1269	0	37	17194	26	7794	
QEN71_RS41745	DNA-binding protein	QEN71_41735	paras_000386	protein-codi	NZ_CP1252	megaplasmi	429288	430376	+	1089	0	6	939	2	733	
QEN71_RS41750	hypothetical protein	QEN71_41740	paras_000387	protein-codi	NZ_CP1252	megaplasmi	430432	430578	+	147	0	3	565	3	565	
QEN71_RS41755	hypothetical protein	QEN71_41745		protein-codi	NZ_CP1252	megaplasmi	430602	431087	+	486	0	9	1594	5	772	
QEN71_RS41760	hypothetical protein	QEN71_41750	paras_000388	protein-codi	NZ_CP1252	megaplasmi	431181	431510	+	330	0	10	7388	7	3538	
QEN71_RS41765	hypothetical protein	QEN71_41755	paras_000389	protein-codi	NZ_CP1252	megaplasmi	431588	431977	+	390	0	7	2111	4	1912	

QEN71_RS41770	AAA family ATPase	QEN71_41760	paras_000390	protein-codi	NZ_CP1252	megaplasmi	432537	433967	+	1431	0	100	10598	76	8808	
QEN71_RS41775	DUF4435 domain-containing protein	QEN71_41765	paras_000391	protein-codi	NZ_CP1252	megaplasmi	433981	434955	+	975	0	43	2782	35	2150	
QEN71_RS41780	AAA family ATPase	QEN71_41770	paras_000392	protein-codi	NZ_CP1252	megaplasmi	435091	436812	+	1722	0	69	8684	51	5942	
QEN71_RS41785	HU family DNA-binding protein	QEN71_41775	paras_000393	protein-codi	NZ_CP1252	megaplasmi	437435	437713	+	275	0	0	0	0	0	TRUE
QEN71_RS41790	PDDEXK nuclease domain-containing protein	QEN71_41780	paras_000394	protein-codi	NZ_CP1252	megaplasmi	437710	438894	+	1181	0	46	9839	39	8508	
QEN71_RS41795	effector-associated domain EAD1-containing protein	QEN71_41785	paras_000395	protein-codi	NZ_CP1252	megaplasmi	439370	440695	+	1326	0	56	10812	37	6437	
QEN71_RS41800	GTPase-associated system all-helical protein GASH	QEN71_41790	paras_000396	protein-codi	NZ_CP1252	megaplasmi	440717	441970	+	1254	0	56	7524	50	7156	
QEN71_RS41805	hypothetical protein	QEN71_41795	paras_000397	protein-codi	NZ_CP1252	megaplasmi	442024	442917	+	886	0	11	1962	11	1962	
QEN71_RS41810	hypothetical protein	QEN71_41800	paras_000398	protein-codi	NZ_CP1252	megaplasmi	442910	443758	+	837	0	31	6967	24	4892	
QEN71_RS41815	hypothetical protein	QEN71_41805	paras_000399	protein-codi	NZ_CP1252	megaplasmi	443755	446133	+	2375	0	77	10103	58	7180	
QEN71_RS41820	hypothetical protein	QEN71_41810	paras_000400	protein-codi	NZ_CP1252	megaplasmi	446357	447148	+	792	0	19	1608	14	976	
QEN71_RS41825	IS66 family transposase	QEN71_41815	paras_000401	protein-codi	NZ_CP1252	megaplasmi	447339	448925	-	1587	3142.0	48	17900	37	14305	
QEN71_RS41830	IS66 family insertion sequence element accessory p	QEN71_41820	paras_000402	protein-codi	NZ_CP1252	megaplasmi	448956	449300	-	341	674.0	14	5055	10	4402	
QEN71_RS41835	transposase	QEN71_41825	paras_000403	protein-codi	NZ_CP1252	megaplasmi	449297	449779	-	479	944.0	28	9964	18	7938	
QEN71_RS41840	RES domain-containing protein	partial;pseudo;QEN71_41830		pseudogene	NZ_CP1252	megaplasmi	449950	450238	+	289	0	6	1593	2	617	
QEN71_RS41845	hypothetical protein	QEN71_41835	paras_000405	protein-codi	NZ_CP1252	megaplasmi	450693	452087	-	1395	147.0	29	5568	15	1640	
QEN71_RS41850	H-NS histone family protein	QEN71_41840	paras_000406	protein-codi	NZ_CP1252	megaplasmi	452446	452772	-	327	0	10	1561	8	1418	
QEN71_RS41855	H-NS family nucleoid-associated regulatory protein	QEN71_41845	paras_000407	protein-codi	NZ_CP1252	megaplasmi	453172	453933	+	762	0	17	2381	12	1790	
QEN71_RS41860	cold-shock protein	QEN71_41850	paras_000408	protein-codi	NZ_CP1252	megaplasmi	454109	454312	-	204	115.0	9	632	5	119	
QEN71_RS41865	DUF3800 domain-containing protein	QEN71_41855	paras_000409	protein-codi	NZ_CP1252	megaplasmi	454786	455433	-	648	0	39	14005	22	5927	
QEN71_RS41870	hypothetical protein	QEN71_41860	paras_000410	protein-codi	NZ_CP1252	megaplasmi	455879	456223	-	345	0	20	6710	20	6710	
QEN71_RS41875	transposase	QEN71_41865		protein-codi	NZ_CP1252	megaplasmi	456449	456841	-	393	0	21	10832	15	6965	
QEN71_RS41880	DUF4158 domain-containing protein	QEN71_41870		protein-codi	NZ_CP1252	megaplasmi	457066	457725	-	660	153.0	23	7495	19	7161	
QEN71_RS41885	hypothetical protein	QEN71_41875	paras_000413	protein-codi	NZ_CP1252	megaplasmi	457899	458597	+	699	0	25	16373	21	15396	
QEN71_RS41890	hypothetical protein	QEN71_41880	paras_000414	protein-codi	NZ_CP1252	megaplasmi	458717	459493	+	777	0	28	11088	25	9959	
QEN71_RS41895	hypothetical protein	QEN71_41885	paras_000415	protein-codi	NZ_CP1252	megaplasmi	459494	460345	+	852	0	43	16689	30	11050	
QEN71_RS41900	response regulator transcription factor	QEN71_41890	paras_000416	protein-codi	NZ_CP1252	megaplasmi	461285	461992	+	708	0	27	9285	16	5693	
QEN71_RS41905	Tn3 family transposase	pseudo;QEN71_41895		pseudogene	NZ_CP1252	megaplasmi	462183	465133	+	2951	0	98	27707	77	19921	
QEN71_RS41910	transposase	partial;pseudo;QEN71_41900		pseudogene	NZ_CP1252	megaplasmi	465496	466042	-	547	0	26	6081	20	4599	
QEN71_RS41915	IS66 family transposase	partial;pseudo;QEN71_41905		pseudogene	NZ_CP1252	megaplasmi	466065	466619	+	555	0	23	3328	21	3247	
QEN71_RS41920	hypothetical protein	QEN71_41910	paras_000420	protein-codi	NZ_CP1252	megaplasmi	467041	467274	-	234	0	7	1419	6	1369	
QEN71_RS41925	7-carboxy-7-deazaguanine synthase QueE	QEN71_41915	paras_000421	protein-codi	NZ_CP1252	megaplasmi	469461	470168	+	708	0	24	4228	15	3548	
QEN71_RS41930	cytolethal distending toxin subunit B family protein	QEN71_41920	paras_000422	protein-codi	NZ_CP1252	megaplasmi	470530	471405	+	876	0	50	4264	37	3134	
QEN71_RS41935	IS5/IS1182 family transposase			pseudogene	NZ_CP1252	megaplasmi	471443	471532	-	90	0	0	0	0	0	
QEN71_RS41940	hypothetical protein	QEN71_41930	paras_000425	protein-codi	NZ_CP1252	megaplasmi	472499	472708	-	210	0	8	2175	8	2175	
QEN71_RS41945	hypothetical protein	pseudo;QEN71_41935		pseudogene	NZ_CP1252	megaplasmi	472735	472979	-	245	0	2	205	0	0	
QEN71_RS41950	hypothetical protein	QEN71_41940	paras_000426	protein-codi	NZ_CP1252	megaplasmi	473205	473498	+	294	0	10	2020	3	788	
QEN71_RS41955	hypothetical protein	QEN71_41945	paras_000427	protein-codi	NZ_CP1252	megaplasmi	473651	474127	+	422	111.0	13	3342	9	2193	
QEN71_RS41960	hypothetical protein	QEN71_41950		protein-codi	NZ_CP1252	megaplasmi	474073	474591	+	460	0	17	2923	15	2913	
QEN71_RS41965	response regulator			pseudogene	NZ_CP1252	megaplasmi	474588	476114	+	1523	0	20	4457	15	2648	
QEN71_RS41970	response regulator transcription factor	partial;pseudo;QEN71_41960		pseudogene	NZ_CP1252	megaplasmi	476137	476693	+	557	147.0	11	1992	4	68	
QEN71_RS41975	IS5 family transposase	pseudo;QEN71_41965		pseudogene	NZ_CP1252	megaplasmi	476736	477547	+	812	1329.0	17	3050	13	1514	
QEN71_RS41980	MFS transporter	partial;pseudo;QEN71_41970		pseudogene	NZ_CP1252	megaplasmi	477823	478341	-	519	0	13	1317	10	1076	
QEN71_RS41985	IS5/IS1182 family transposase	partial;pseudo;QEN71_41975		pseudogene	NZ_CP1252	megaplasmi	478494	478616	+	121	0	5	809	2	513	
QEN71_RS41990	IS6 family transposase	partial;pseudo;QEN71_41980		pseudogene	NZ_CP1252	megaplasmi	478615	478716	+	100	0	3	242	3	242	
QEN71_RS41995	hemerythrin domain-containing protein	QEN71_41985	paras_000433	protein-codi	NZ_CP1252	megaplasmi	479227	479787	-	561	0	17	1220	11	843	
QEN71_RS42000	AAA family ATPase	partial;pseudo;QEN71_41990		pseudogene	NZ_CP1252	megaplasmi	480218	480383	-	166	0	0	0	0	0	
QEN71_RS42005	recombinase family protein	partial;pseudo;QEN71_41995		pseudogene	NZ_CP1252	megaplasmi	480594	481438	-	845	0	20	2857	14	2278	
QEN71_RS42010	transposase domain-containing protein	partial;pseudo;QEN71_42000		pseudogene	NZ_CP1252	megaplasmi	481938	482175	+	192	0	9	849	7	785	
QEN71_RS42015	hypothetical protein			protein-codi	NZ_CP1252	megaplasmi	482130	482342	-	167	0	7	479	4	216	
QEN71_RS42020	ATP-binding cassette domain-containing protein	partial;pseudo;QEN71_42010		pseudogene	NZ_CP1252	megaplasmi	482412	482571	-	160	0	0	0	0	0	
QEN71_RS42025	maltose alpha-D-glucosyltransferase	QEN71_42015	paras_000437	protein-codi	NZ_CP1252	megaplasmi	482631	484694	-	2064	0	65	7277	49	4892	
QEN71_RS42030	aldehyde dehydrogenase family protein	QEN71_42020	paras_000438	protein-codi	NZ_CP1252	megaplasmi	484784	486262	-	1479	0	37	3379	31	3022	
QEN71_RS42035	IS110 family transposase	QEN71_42025	paras_000439	protein-codi	NZ_CP1252	megaplasmi	486995	488020	-	1026	2022.0	1	2	0	0	
QEN71_RS42040	IS21 family transposase	partial;pseudo;QEN71_42030		pseudogene	NZ_CP1252	megaplasmi	488435	488728	-	294	0	8	963	5	384	
QEN71_RS42045	IS5 family transposase	partial;pseudo;QEN71_42035		pseudogene	NZ_CP1252	megaplasmi	488802	489683	-	882	0	31	4305	23	3521	
QEN71_RS42050	radical SAM protein	QEN71_42040	paras_000442	protein-codi	NZ_CP1252	megaplasmi	489826	491265	-	1439	0	95	11013	68	7229	
QEN71_RS42055	glycosyltransferase	QEN71_42045	paras_000443	protein-codi	NZ_CP1252	megaplasmi	491265	493934	-	2669	0	156	15662	127	13344	
QEN71_RS42060	substrate-binding domain-containing protein	QEN71_42050	paras_000444	protein-codi	NZ_CP1252	megaplasmi	494516	494884	-	369	0	11	735	6	407	
QEN71_RS42065	helix-turn-helix domain-containing protein	partial;pseudo;QEN71_42055		pseudogene	NZ_CP1252	megaplasmi	495135	495464	-	256	0	4	855	2	503	
QEN71_RS42070	hypothetical protein	QEN71_42060		protein-codi	NZ_CP1252	megaplasmi	495391	495597	-	133	0	0	0	0	0	
QEN71_RS42075	efflux RND transporter permease subunit	QEN71_42065	paras_000447	protein-codi	NZ_CP1252	megaplasmi	495952	499005	-	3054	287.0	52	4839	37	2396	

QEN71_RS42080	efflux RND transporter periplasmic adaptor subunit	QEN71_42070	paras_000448	protein-codi	NZ_CP1252	megaplasmi	499016	500167	-	1152	192.0	22	1484	18	1307	
QEN71_RS42085	efflux transporter outer membrane subunit	QEN71_42075	paras_000449	protein-codi	NZ_CP1252	megaplasmi	500277	501875	-	1599	0	25	2788	22	2557	
QEN71_RS42090	adenylyl-sulfate kinase	QEN71_42080	paras_000450	protein-codi	NZ_CP1252	megaplasmi	502079	502678	-	600	0	12	1479	8	1261	
QEN71_RS42095	nodulation protein NodU	QEN71_42085	paras_000451	protein-codi	NZ_CP1252	megaplasmi	502880	504598	-	1719	0	52	2750	42	2159	
QEN71_RS42100	nodulation methyltransferase NodS	QEN71_42090	paras_000452	protein-codi	NZ_CP1252	megaplasmi	504611	505240	-	630	0	9	222	7	178	
QEN71_RS42105	NodA family N-acyltransferase	QEN71_42095	paras_000453	protein-codi	NZ_CP1252	megaplasmi	505330	505920	-	591	0	11	754	10	709	
QEN71_RS42110	sulfotransferase	QEN71_42100	paras_000454	protein-codi	NZ_CP1252	megaplasmi	506135	506890	-	756	0	13	325	12	322	
QEN71_RS42115	ABC transporter permease	QEN71_42105	paras_000455	protein-codi	NZ_CP1252	megaplasmi	506988	507779	-	792	0	16	653	10	587	
QEN71_RS42120	nodulation factor ABC transporter ATP-binding prot	QEN71_42110	paras_000456	protein-codi	NZ_CP1252	megaplasmi	507780	508712	-	933	0	18	1217	14	1041	
QEN71_RS42125	chitooligosaccharide synthase NodC	QEN71_42115	paras_000457	protein-codi	NZ_CP1252	megaplasmi	508760	510103	-	1344	0	28	1400	25	1324	
QEN71_RS42130	chitooligosaccharide deacetylase NodB	QEN71_42120	paras_000458	protein-codi	NZ_CP1252	megaplasmi	510506	511186	-	681	0	10	438	10	438	
QEN71_RS42135	LysR family transcriptional regulator	QEN71_42125	paras_000459	protein-codi	NZ_CP1252	megaplasmi	511419	512333	+	882	0	16	583	16	583	
QEN71_RS42140	ATP-grasp domain-containing protein	QEN71_42130	paras_000460	protein-codi	NZ_CP1252	megaplasmi	512301	513632	-	1299	0	32	2344	23	1618	
QEN71_RS42145	hypothetical protein	QEN71_42135	paras_000461	protein-codi	NZ_CP1252	megaplasmi	513645	513827	-	175	0	3	367	1	82	
QEN71_RS42150	hypothetical protein	QEN71_42140		protein-codi	NZ_CP1252	megaplasmi	513820	514074	-	247	0	8	1115	6	1004	
QEN71_RS42155	hypothetical protein	QEN71_42145		protein-codi	NZ_CP1252	megaplasmi	514127	514372	-	246	0	11	1500	11	1500	
QEN71_RS42160	hypothetical protein	QEN71_42150		protein-codi	NZ_CP1252	megaplasmi	514380	514688	-	309	0	6	581	5	468	
QEN71_RS42165	hypothetical protein	QEN71_42155	paras_000462	protein-codi	NZ_CP1252	megaplasmi	514700	515398	-	699	0	23	1117	20	977	
QEN71_RS42170	LysR family transcriptional regulator	QEN71_42160	paras_000463	protein-codi	NZ_CP1252	megaplasmi	515949	516845	+	897	0	14	1272	9	945	
QEN71_RS42175	nif-specific transcriptional activator NifA	QEN71_42165	paras_000464	protein-codi	NZ_CP1252	megaplasmi	517576	519219	+	1644	0	45	6077	31	4595	
QEN71_RS42180	nitrogenase iron-molybdenum cofactor biosynthesis	QEN71_42170	paras_000465	protein-codi	NZ_CP1252	megaplasmi	519401	520933	+	1523	0	49	5724	47	5497	
QEN71_RS42185	nitrogenase iron-molybdenum cofactor biosynthesis	QEN71_42175	paras_000466	protein-codi	NZ_CP1252	megaplasmi	520924	522273	+	1340	0	38	3183	32	2540	
QEN71_RS42190	nitrogen fixation protein NifX	QEN71_42180	paras_000467	protein-codi	NZ_CP1252	megaplasmi	522330	522746	+	417	0	17	2204	9	1228	
QEN71_RS42195	CCE_0567 family metalloprotein	QEN71_42185	paras_000468	protein-codi	NZ_CP1252	megaplasmi	522818	523039	+	218	0	1	33	1	33	
QEN71_RS42200	ferredoxin III, nif-specific	QEN71_42190	paras_000469	protein-codi	NZ_CP1252	megaplasmi	523036	523338	+	299	0	7	264	3	145	
QEN71_RS42205	nitrogen fixation protein NifQ	QEN71_42195	paras_000470	protein-codi	NZ_CP1252	megaplasmi	523355	523927	+	573	0	14	982	12	835	
QEN71_RS42210	hypothetical protein	QEN71_42200	paras_000471	protein-codi	NZ_CP1252	megaplasmi	524072	524557	-	486	0	15	1523	13	1432	
QEN71_RS42215	4Fe-4S dicluster domain-containing protein	QEN71_42205	paras_000472	protein-codi	NZ_CP1252	megaplasmi	524631	524930	-	296	0	8	953	7	744	
QEN71_RS42220	FAD-dependent monooxygenase	QEN71_42210	paras_000473	protein-codi	NZ_CP1252	megaplasmi	524927	526237	-	1303	0	35	5221	26	3511	
QEN71_RS42225	electron transfer flavoprotein subunit alpha/FixB fan	QEN71_42215	paras_000474	protein-codi	NZ_CP1252	megaplasmi	526234	527361	-	1120	0	29	4365	21	2766	
QEN71_RS42230	electron transfer flavoprotein subunit beta/FixA fami	QEN71_42220	paras_000475	protein-codi	NZ_CP1252	megaplasmi	527358	528206	-	845	0	21	2980	11	1424	
QEN71_RS42235	nitrogenase stabilizing/protective protein NifW	QEN71_42225	paras_000476	protein-codi	NZ_CP1252	megaplasmi	528221	528559	-	339	0	6	397	4	133	
QEN71_RS42240	homocitrate synthase	QEN71_42230	paras_000477	protein-codi	NZ_CP1252	megaplasmi	528602	529738	-	1137	0	20	1637	20	1637	
QEN71_RS42245	nitrogenase cofactor biosynthesis protein NifB	QEN71_42235	paras_000478	protein-codi	NZ_CP1252	megaplasmi	530170	531774	+	1605	0	38	3355	31	2393	
QEN71_RS42250	ferredoxin	QEN71_42240	paras_000479	protein-codi	NZ_CP1252	megaplasmi	531841	532035	+	195	385.0	1	6	1	6	
QEN71_RS42255	iron-sulfur cluster assembly accessory protein	QEN71_42245	paras_000480	protein-codi	NZ_CP1252	megaplasmi	532100	532498	+	399	748.0	3	93	2	82	
QEN71_RS42260	nitrogen fixation protein NifZ	QEN71_42250		protein-codi	NZ_CP1252	megaplasmi	532529	532972	+	444	462.0	14	1971	11	1898	
QEN71_RS42265	putative nitrogen fixation protein NifT	QEN71_42255	paras_000482	protein-codi	NZ_CP1252	megaplasmi	533320	533538	+	219	0	7	502	5	448	
QEN71_RS42270	hypothetical protein	QEN71_42260	paras_000483	protein-codi	NZ_CP1252	megaplasmi	533540	533821	+	282	0	20	3909	10	2474	
QEN71_RS42275	IS630 family transposase			pseudogene	NZ_CP1252	megaplasmi	533958	534056	-	99	0	3	388	3	388	
QEN71_RS42280	transposase	QEN71_42270	paras_000484	pseudogene	NZ_CP1252	megaplasmi	534304	534873	-	570	0	24	4454	19	3435	
QEN71_RS42285	IS21 family transposase	QEN71_42275	paras_000485	protein-codi	NZ_CP1252	megaplasmi	535463	536956	+	1490	2950.0	36	6698	28	3945	
QEN71_RS42290	IS21-like element helper ATPase IstB	QEN71_42280	paras_000486	protein-codi	NZ_CP1252	megaplasmi	536953	537774	+	818	1618.0	9	1669	7	1580	
QEN71_RS42295	IS91 family transposase	pseudo;QEN71_42285		pseudogene	NZ_CP1252	megaplasmi	537845	539011	-	1167	2314.0	15	476	13	140	
QEN71_RS42300	site-specific integrase	QEN71_42290	paras_000488	protein-codi	NZ_CP1252	megaplasmi	539020	539898	-	879	1739.0	6	70	4	51	
QEN71_RS42305	IS110 family transposase	partial;pseudo;QEN71_42295		pseudogene	NZ_CP1252	megaplasmi	540332	540645	-	314	0	5	159	3	111	
QEN71_RS42310	aminotransferase class V-fold PLP-dependent enzy	QEN71_42300	paras_000489	protein-codi	NZ_CP1252	megaplasmi	541570	542682	+	1113	0	35	2233	26	1651	
QEN71_RS42315	EAL domain-containing protein	QEN71_42305	paras_000490	protein-codi	NZ_CP1252	megaplasmi	542727	543632	+	906	0	32	1879	26	1103	
QEN71_RS42320	IS4 family transposase	QEN71_42310	paras_000491	protein-codi	NZ_CP1252	megaplasmi	544146	545492	-	1347	0	23	2210	19	1952	
QEN71_RS42325	transposase	partial;pseudo;QEN71_42315		pseudogene	NZ_CP1252	megaplasmi	545529	546125	-	597	0	14	509	9	362	
QEN71_RS42330	ATP-binding protein	partial;pseudo;QEN71_42320		pseudogene	NZ_CP1252	megaplasmi	546201	546733	-	533	0	10	842	7	536	
QEN71_RS42335	IS5 family transposase	pseudo;QEN71_42325		pseudogene	NZ_CP1252	megaplasmi	547010	548463	+	1454	0	15	1049	14	991	
QEN71_RS42340	tyrosine-type recombinase/integrase	QEN71_42330		protein-codi	NZ_CP1252	megaplasmi	548669	549010	-	342	256.0	12	690	10	529	
QEN71_RS42345	hypothetical protein			pseudogene	NZ_CP1252	megaplasmi	549418	549617	-	200	100.0	2	132	2	132	
QEN71_RS42350	site-specific integrase	QEN71_42340	paras_000496	protein-codi	NZ_CP1252	megaplasmi	550333	551199	+	863	0	16	1248	10	856	
QEN71_RS42355	tyrosine-type recombinase/integrase	QEN71_42345	paras_000497	protein-codi	NZ_CP1252	megaplasmi	551196	552188	+	985	0	19	1318	13	1137	
QEN71_RS42360	tyrosine-type recombinase/integrase	QEN71_42350	paras_000498	protein-codi	NZ_CP1252	megaplasmi	552185	553189	+	1001	0	18	1062	14	869	
QEN71_RS42365	IS21 family transposase	partial;pseudo;QEN71_42355		pseudogene	NZ_CP1252	megaplasmi	553194	553722	-	529	374.0	13	2955	9	1906	
QEN71_RS42370	sigma factor-like helix-turn-helix DNA-binding protei	QEN71_42360	paras_000500	protein-codi	NZ_CP1252	megaplasmi	553787	554290	-	504	0	11	1404	9	1364	
QEN71_RS42375	zinc ribbon domain-containing protein	QEN71_42365	paras_000501	protein-codi	NZ_CP1252	megaplasmi	554636	554878	-	243	0	14	1402	7	582	
QEN71_RS42380	ankyrin repeat domain-containing protein	QEN71_42370	paras_000502	protein-codi	NZ_CP1252	megaplasmi	554887	555198	-	312	0	4	684	4	684	
QEN71_RS42385	hypothetical protein	QEN71_42375	paras_000503	protein-codi	NZ_CP1252	megaplasmi	555369	555977	-	609	114.0	12	809	12	809	

QEN71_RS42390	nitrogenase iron protein	QEN71_42380	paras_000504	protein-codi	NZ_CP1252	megaplasmi	556416	557297	+	882	1360.0	23	2552	21	2241	
QEN71_RS42395	nitrogenase molybdenum-iron protein alpha chain	QEN71_42385	paras_000505	protein-codi	NZ_CP1252	megaplasmi	557369	558832	+	1464	328.0	37	2634	36	2562	
QEN71_RS42400	nitrogenase molybdenum-iron protein subunit beta	QEN71_42390	paras_000506	protein-codi	NZ_CP1252	megaplasmi	558904	560463	+	1560	0	31	2232	22	1879	
QEN71_RS42405	AAA family ATPase	QEN71_42395	paras_000507	protein-codi	NZ_CP1252	megaplasmi	560895	566324	-	5430	0	148	14407	119	10840	
QEN71_RS42410	hypothetical protein	QEN71_42400	paras_000508	protein-codi	NZ_CP1252	megaplasmi	566642	566953	-	312	0	0	0	0	0	TRUE
QEN71_RS42415	cache domain-containing protein	pseudo;QEN71_42405		pseudogene	NZ_CP1252	megaplasmi	567157	568515	+	1355	0	25	2489	16	1417	
QEN71_RS42420	response regulator transcription factor	QEN71_42410	paras_000510	protein-codi	NZ_CP1252	megaplasmi	568512	569084	+	569	0	14	1755	9	1173	
QEN71_RS42425	response regulator transcription factor	QEN71_42415	paras_000511	protein-codi	NZ_CP1252	megaplasmi	570482	571114	+	633	147.0	20	4044	11	2898	
QEN71_RS42430	IS110 family transposase	QEN71_42420	paras_000512	protein-codi	NZ_CP1252	megaplasmi	571724	572929	-	1206	195.0	23	4913	13	1316	
QEN71_RS42435	cytochrome P450	QEN71_42425	paras_000513	protein-codi	NZ_CP1252	megaplasmi	574192	575484	+	1293	0	46	7898	34	4268	
QEN71_RS42440	carboxyl transferase domain-containing protein	QEN71_42430	paras_000514	protein-codi	NZ_CP1252	megaplasmi	575721	577079	-	1359	0	67	7444	54	6194	
QEN71_RS42445	amidase family protein	QEN71_42435	paras_000515	protein-codi	NZ_CP1252	megaplasmi	577162	578502	-	1341	0	53	5752	41	4790	
QEN71_RS42450	FAD-dependent oxidoreductase	QEN71_42440	paras_000516	protein-codi	NZ_CP1252	megaplasmi	578533	578943	-	411	0	15	2369	12	1197	
QEN71_RS42455	IS110 family transposase	partial;pseudo;QEN71_42445		pseudogene	NZ_CP1252	megaplasmi	579621	580471	+	847	0	10	1400	5	437	
QEN71_RS42460	transposase	partial;pseudo;QEN71_42450		pseudogene	NZ_CP1252	megaplasmi	580468	580700	+	229	0	10	1152	7	639	
QEN71_RS42465	sterol desaturase family protein	QEN71_42455	paras_000519	protein-codi	NZ_CP1252	megaplasmi	581137	581991	+	855	0	34	2743	25	2133	
QEN71_RS42470	glycosyl hydrolase	QEN71_42460	paras_000520	protein-codi	NZ_CP1252	megaplasmi	582669	583445	+	777	0	39	4177	36	3297	
QEN71_RS42475	ferredoxin	QEN71_42465	paras_000521	protein-codi	NZ_CP1252	megaplasmi	583909	584103	+	195	385.0	3	375	3	375	
QEN71_RS42480	iron-sulfur cluster assembly accessory protein	pseudo;QEN71_42470		pseudogene	NZ_CP1252	megaplasmi	584168	584564	+	397	748.0	7	433	3	134	
QEN71_RS42485	nitrogen fixation protein NifZ	QEN71_42475	paras_000523	protein-codi	NZ_CP1252	megaplasmi	584598	585011	+	414	462.0	16	3810	16	3810	
QEN71_RS42490	putative nitrogen fixation protein NifT	QEN71_42480	paras_000524	protein-codi	NZ_CP1252	megaplasmi	585266	585484	+	219	0	9	1317	9	1317	
QEN71_RS42495	IS110 family transposase	partial;pseudo;QEN71_42485		pseudogene	NZ_CP1252	megaplasmi	585593	586212	+	620	0	16	752	13	584	
QEN71_RS42500	IS110 family transposase	QEN71_42490	paras_000526	protein-codi	NZ_CP1252	megaplasmi	586483	587496	+	1014	1997.0	40	8573	28	7031	
QEN71_RS42505	2-aminoethylphosphonate--pyruvate transaminase	QEN71_42495	paras_000527	protein-codi	NZ_CP1252	megaplasmi	588528	589679	+	1152	0	20	714	18	703	
QEN71_RS42510	PLP-dependent transferase	QEN71_42500	paras_000528	protein-codi	NZ_CP1252	megaplasmi	589681	591003	+	1323	0	25	1401	20	1070	
QEN71_RS42515	fatty acid desaturase family protein	QEN71_42505	paras_000529	protein-codi	NZ_CP1252	megaplasmi	591034	592095	+	1062	0	35	1903	32	1562	
QEN71_RS42520	gamma-glutamyl-gamma-aminobutyrate hydrolase f	QEN71_42510	paras_000530	protein-codi	NZ_CP1252	megaplasmi	592098	592919	+	822	0	26	1598	21	1226	
QEN71_RS42525	LysE family translocator	QEN71_42515	paras_000531	protein-codi	NZ_CP1252	megaplasmi	592990	593586	+	597	0	23	2128	21	2028	
QEN71_RS42530	homoserine O-succinyltransferase	QEN71_42520	paras_000532	protein-codi	NZ_CP1252	megaplasmi	593672	594577	+	906	0	38	2380	30	1558	
QEN71_RS42535	glutamine synthetase family protein	QEN71_42525	paras_000533	protein-codi	NZ_CP1252	megaplasmi	594649	596007	+	1359	0	66	8576	56	6902	
QEN71_RS42540	RES family NAD+ phosphorylase	QEN71_42530	paras_000534	protein-codi	NZ_CP1252	megaplasmi	596309	596830	-	518	0	26	8480	19	5479	
QEN71_RS42545	DUF2384 domain-containing protein	QEN71_42535	paras_000535	protein-codi	NZ_CP1252	megaplasmi	596827	597234	-	404	0	0	0	0	0	TRUE
QEN71_RS42550	IS21 family transposase	QEN71_42540	paras_000536	protein-codi	NZ_CP1252	megaplasmi	597712	599235	+	1524	3027.0	13	187	12	179	
QEN71_RS42555	IS21-like element helper ATPase IstB	QEN71_42545	paras_000537	protein-codi	NZ_CP1252	megaplasmi	599242	600030	+	789	1561.0	9	237	9	237	
QEN71_RS42560	hypothetical protein	QEN71_42550	paras_000538	protein-codi	NZ_CP1252	megaplasmi	600098	600238	-	141	0	1	83	0	0	
QEN71_RS42565	IS66 family insertion sequence element accessory p	QEN71_42555	paras_000539	protein-codi	NZ_CP1252	megaplasmi	600268	600615	-	344	0	10	1916	7	1288	
QEN71_RS42570	transposase	QEN71_42560	paras_000540	protein-codi	NZ_CP1252	megaplasmi	600612	600962	-	347	0	10	1235	6	1106	
QEN71_RS42575	transposase	partial;pseudo;QEN71_42565		pseudogene	NZ_CP1252	megaplasmi	601904	602352	+	449	884.0	28	13704	24	11548	
QEN71_RS42580	carbamoyltransferase HypF	QEN71_42570	paras_000542	protein-codi	NZ_CP1252	megaplasmi	603681	606056	-	2369	0	69	14900	51	10844	
QEN71_RS42585	hydrogenase expression/formation protein HypE	QEN71_42575	paras_000543	protein-codi	NZ_CP1252	megaplasmi	606050	607075	-	1019	0	30	3900	21	2690	
QEN71_RS42590	isochorismatase family cysteine hydrolase	QEN71_42580	paras_000544	protein-codi	NZ_CP1252	megaplasmi	607158	607745	-	588	0	14	2035	12	1909	
QEN71_RS42595	GNAT family N-acetyltransferase	QEN71_42585	paras_000545	protein-codi	NZ_CP1252	megaplasmi	608210	608791	-	582	0	13	1823	11	1687	
QEN71_RS42600	hypothetical protein	QEN71_42590	paras_000546	protein-codi	NZ_CP1252	megaplasmi	609008	609169	+	162	0	3	244	1	5	
QEN71_RS42605	IS630 family transposase	partial;pseudo;QEN71_42595		pseudogene	NZ_CP1252	megaplasmi	609776	610026	-	251	0	10	1094	7	822	
QEN71_RS42610	diaminobutyrate--2-oxoglutarate transaminase	QEN71_42600	paras_000548	protein-codi	NZ_CP1252	megaplasmi	610326	611594	-	1269	0	29	1188	22	874	
QEN71_RS42615	twin-arginine translocation signal domain-containing	partial;pseudo;QEN71_42605		pseudogene	NZ_CP1252	megaplasmi	612356	612789	+	415	0	7	591	7	591	
QEN71_RS42620	nickel-dependent hydrogenase large subunit	partial;pseudo;QEN71_42610		pseudogene	NZ_CP1252	megaplasmi	612771	613771	+	982	0	24	1518	23	1464	
QEN71_RS42625	HypC/HybG/HupF family hydrogenase formation ch	QEN71_42615	paras_000552	protein-codi	NZ_CP1252	megaplasmi	614354	614518	+	165	0	4	76	3	71	
QEN71_RS42630	hypothetical protein	QEN71_42620		protein-codi	NZ_CP1252	megaplasmi	614618	614761	+	144	0	6	648	5	546	
QEN71_RS00005	chromosomal replication initiator protein DnaA	QEN71_00005	paras_000554	protein-codi	NZ_CP1252	chromosom	1	1557	+	1557	0	1	18	1	18	TRUE
QEN71_RS00010	DNA polymerase III subunit beta	QEN71_00010	paras_000555	protein-codi	NZ_CP1252	chromosom	1814	2917	+	1104	0	0	0	0	0	TRUE
QEN71_RS00015	DNA topoisomerase (ATP-hydrolyzing) subunit B	QEN71_00015	paras_000556	protein-codi	NZ_CP1252	chromosom	3031	5502	+	2472	0	6	669	1	3	TRUE
QEN71_RS00020	hypothetical protein	QEN71_00020	paras_000557	protein-codi	NZ_CP1252	chromosom	5571	5804	-	234	0	20	9755	20	9755	
QEN71_RS00025	AraC family transcriptional regulator	QEN71_00025	paras_000558	protein-codi	NZ_CP1252	chromosom	5830	6696	-	867	0	40	13354	27	9245	
QEN71_RS00030	ethanolamine permease	QEN71_00030	paras_000559	protein-codi	NZ_CP1252	chromosom	6947	8350	+	1404	0	50	17633	40	15822	
QEN71_RS00035	ethanolamine ammonia-lyase subunit EutB	QEN71_00035	paras_000560	protein-codi	NZ_CP1252	chromosom	8411	9805	+	1391	0	37	12661	34	12307	
QEN71_RS00040	ethanolamine ammonia-lyase subunit EutC	QEN71_00040	paras_000561	protein-codi	NZ_CP1252	chromosom	9802	10596	+	791	0	25	4205	18	2883	
QEN71_RS00045	DUF779 domain-containing protein	QEN71_00045	paras_000562	protein-codi	NZ_CP1252	chromosom	10650	11042	-	393	0	6	4470	4	1554	
QEN71_RS00050	aldehyde dehydrogenase family protein	QEN71_00050	paras_000563	protein-codi	NZ_CP1252	chromosom	11122	12642	-	1521	0	41	7067	25	4983	
QEN71_RS00055	helix-turn-helix domain-containing protein	QEN71_00055	paras_000564	protein-codi	NZ_CP1252	chromosom	13003	14010	+	1008	0	16	4255	13	3870	
QEN71_RS00060	hypothetical protein	QEN71_00060	paras_000565	protein-codi	NZ_CP1252	chromosom	14444	14629	+	186	0	14	5156	6	1883	
QEN71_RS00065	ATP-dependent RNA helicase DbpA	QEN71_00065	paras_000566	protein-codi	NZ_CP1252	chromosom	14644	16035	+	1392	0	16	3348	9	1445	

QEN71_RS00070	hypothetical protein	QEN71_00070	paras_000567	protein-codi	NZ_CP1252	chromosom	16039	16299	+	261	0	9	2012	4	578	
QEN71_RS00075	pyridoxal phosphate-dependent aminotransferase	QEN71_00075	paras_000568	protein-codi	NZ_CP1252	chromosom	16356	17510	+	1155	0	28	7008	23	5375	
QEN71_RS00080	carbon-nitrogen hydrolase family protein	QEN71_00080	paras_000569	protein-codi	NZ_CP1252	chromosom	17525	18367	+	843	0	17	4130	15	4010	
QEN71_RS00085	ABC transporter substrate-binding protein	QEN71_00085	paras_000570	protein-codi	NZ_CP1252	chromosom	18400	19191	+	792	0	30	5647	21	3872	
QEN71_RS00090	anti-sigma factor	QEN71_00090	paras_000571	protein-codi	NZ_CP1252	chromosom	19366	20103	+	738	0	15	2659	13	2361	
QEN71_RS00095	RNA polymerase sigma factor	QEN71_00095	paras_000572	protein-codi	NZ_CP1252	chromosom	20251	20769	+	515	0	4	206	4	206	
QEN71_RS00100	anti-sigma factor	QEN71_00100	paras_000573	protein-codi	NZ_CP1252	chromosom	20766	21599	+	830	0	7	388	4	147	
QEN71_RS00105	DUF4148 domain-containing protein	QEN71_00105	paras_000574	protein-codi	NZ_CP1252	chromosom	21776	22045	+	270	0	11	2857	11	2857	
QEN71_RS00110	tRNA-Lys	QEN71_00110		tRNA	NZ_CP1252	chromosom	22112	22187	-	76	0	3	10	3	10	
QEN71_RS00115	PaaI family thioesterase	QEN71_00115	paras_000576	protein-codi	NZ_CP1252	chromosom	22401	22805	+	405	0	5	1376	2	251	
QEN71_RS00120	patatin-like phospholipase family protein	QEN71_00120	paras_000577	protein-codi	NZ_CP1252	chromosom	22861	23727	+	867	0	35	9344	28	6505	
QEN71_RS00125	D-2-hydroxyacid dehydrogenase family protein	QEN71_00125	paras_000578	protein-codi	NZ_CP1252	chromosom	23807	24820	-	1014	0	33	12056	26	10207	
QEN71_RS00130	phosphoenolpyruvate carboxykinase (GTP)	QEN71_00130	paras_000579	protein-codi	NZ_CP1252	chromosom	25172	27028	+	1857	0	45	19890	41	17926	
QEN71_RS00135	LysR family transcriptional regulator	QEN71_00135	paras_000580	protein-codi	NZ_CP1252	chromosom	27363	28451	+	1089	0	39	11191	29	8530	
QEN71_RS00140	DNA topoisomerase III	QEN71_00140	paras_000581	protein-codi	NZ_CP1252	chromosom	28551	31211	-	2661	0	9	1843	3	198	
QEN71_RS00145	thioredoxin family protein	QEN71_00145	paras_000582	protein-codi	NZ_CP1252	chromosom	31506	31883	-	378	0	1	2	1	2	TRUE
QEN71_RS00150	DNA-processing protein DprA	QEN71_00150	paras_000583	protein-codi	NZ_CP1252	chromosom	31938	33113	-	1176	0	25	11798	23	11746	
QEN71_RS00155	peptide deformylase	QEN71_00155	paras_000584	protein-codi	NZ_CP1252	chromosom	33369	33872	+	504	0	8	142	7	136	
QEN71_RS00160	methionyl-tRNA formyltransferase	QEN71_00160	paras_000585	protein-codi	NZ_CP1252	chromosom	33896	34879	+	984	0	5	1364	1	9	
QEN71_RS00165	LysE family translocator	QEN71_00165	paras_000586	protein-codi	NZ_CP1252	chromosom	34980	35621	+	642	0	11	4685	10	4682	
QEN71_RS00170	zinc metalloprotease HtpX	QEN71_00170	paras_000587	protein-codi	NZ_CP1252	chromosom	35725	36582	+	858	0	14	3333	10	2463	
QEN71_RS00175	16S rRNA (cytosine(967)-C(5))-methyltransferase R	QEN71_00175	paras_000588	protein-codi	NZ_CP1252	chromosom	36802	38226	+	1421	0	25	6252	17	4078	
QEN71_RS00180	DUF4390 domain-containing protein	QEN71_00180	paras_000589	protein-codi	NZ_CP1252	chromosom	38223	38813	+	576	0	9	2025	7	1797	
QEN71_RS00185	PAS domain-containing sensor histidine kinase	QEN71_00185	paras_000590	protein-codi	NZ_CP1252	chromosom	38803	41223	+	2410	0	42	6052	27	4392	
QEN71_RS00190	response regulator transcription factor EsaR	QEN71_00190	paras_000591	protein-codi	NZ_CP1252	chromosom	41224	41913	+	690	0	8	321	5	192	
QEN71_RS00195	tRNA-Phe	QEN71_00195		tRNA	NZ_CP1252	chromosom	42000	42075	+	76	0	0	0	0	0	
QEN71_RS00200	7-cyano-7-deazaguanine synthase QueC	QEN71_00200	paras_000593	protein-codi	NZ_CP1252	chromosom	42445	43176	+	732	0	13	1780	9	1106	
QEN71_RS00205	7-carboxy-7-deazaguanine synthase	QEN71_00205	paras_000594	protein-codi	NZ_CP1252	chromosom	43248	43880	+	633	0	15	3490	6	714	
QEN71_RS00210	6-carboxytetrahydropterin synthase QueD	QEN71_00210	paras_000595	protein-codi	NZ_CP1252	chromosom	43890	44333	+	444	0	16	1977	10	1292	
QEN71_RS00215	aldolase/citrate lyase family protein	QEN71_00215	paras_000596	protein-codi	NZ_CP1252	chromosom	44518	45300	+	779	0	17	2308	16	2291	
QEN71_RS00220	tetratricopeptide repeat protein	QEN71_00220	paras_000597	protein-codi	NZ_CP1252	chromosom	45297	46007	+	707	0	26	8522	21	6420	
QEN71_RS00225	rod shape-determining protein RodA	QEN71_00225	paras_000598	protein-codi	NZ_CP1252	chromosom	46059	47207	-	1149	0	2	31	2	31	TRUE
QEN71_RS00230	penicillin-binding protein 2	QEN71_00230	paras_000599	protein-codi	NZ_CP1252	chromosom	47218	49611	-	2394	0	3	267	2	35	TRUE
QEN71_RS00235	rod shape-determining protein MreD	QEN71_00235	paras_000600	protein-codi	NZ_CP1252	chromosom	49740	50252	-	509	0	4	208	2	152	
QEN71_RS00240	rod shape-determining protein MreC	QEN71_00240	paras_000601	protein-codi	NZ_CP1252	chromosom	50249	51349	-	1097	0	4	836	3	806	
QEN71_RS00245	rod shape-determining protein	QEN71_00245	paras_000602	protein-codi	NZ_CP1252	chromosom	51477	52520	-	1044	0	3	894	0	0	
QEN71_RS00250	Asp-tRNA(Asn)/Glu-tRNA(Gln) amidotransferase su	QEN71_00250	paras_000603	protein-codi	NZ_CP1252	chromosom	52901	53200	+	300	0	1	175	0	0	TRUE
QEN71_RS00255	Asp-tRNA(Asn)/Glu-tRNA(Gln) amidotransferase su	QEN71_00255	paras_000604	protein-codi	NZ_CP1252	chromosom	53366	54859	+	1494	0	0	0	0	0	TRUE
QEN71_RS00260	Asp-tRNA(Asn)/Glu-tRNA(Gln) amidotransferase su	QEN71_00260	paras_000605	protein-codi	NZ_CP1252	chromosom	54862	56337	+	1476	0	0	0	0	0	TRUE
QEN71_RS00265	polyphosphate kinase 2 family protein	QEN71_00265	paras_000606	protein-codi	NZ_CP1252	chromosom	56444	57280	+	837	0	23	5524	18	4344	
QEN71_RS00270	exodeoxyribonuclease III	QEN71_00270	paras_000607	protein-codi	NZ_CP1252	chromosom	57324	58103	+	780	0	30	6996	24	5737	
QEN71_RS00275	M48 family metallopeptidase	QEN71_00275	paras_000608	protein-codi	NZ_CP1252	chromosom	58119	59327	-	1208	0	36	6681	29	5075	
QEN71_RS00280	AmpG family muropeptide MFS transporter	QEN71_00280	paras_000609	protein-codi	NZ_CP1252	chromosom	59327	60721	-	1394	0	40	9817	27	7044	
QEN71_RS00285	methionine biosynthesis protein MetW	QEN71_00285	paras_000610	protein-codi	NZ_CP1252	chromosom	60735	61343	-	605	0	16	1974	9	839	
QEN71_RS00290	homoserine O-acetyltransferase	QEN71_00290	paras_000611	protein-codi	NZ_CP1252	chromosom	61340	62485	-	1142	0	43	11710	31	9133	
QEN71_RS00295	nucleoid occlusion factor SlmA	QEN71_00295	paras_000612	protein-codi	NZ_CP1252	chromosom	62723	63406	-	684	0	11	3120	8	2629	
QEN71_RS00300	pyrimidine 5'-nucleotidase	QEN71_00300	paras_000613	protein-codi	NZ_CP1252	chromosom	63446	64201	-	756	0	20	6140	15	4311	
QEN71_RS00305	acetylglutamate kinase	QEN71_00305	paras_000614	protein-codi	NZ_CP1252	chromosom	64389	65288	-	900	0	7	169	5	114	
QEN71_RS00310	cysteine-rich CWC family protein	QEN71_00310	paras_000615	protein-codi	NZ_CP1252	chromosom	65630	65869	+	240	0	0	0	0	0	
QEN71_RS00315	hypothetical protein	QEN71_00315	paras_000616	protein-codi	NZ_CP1252	chromosom	65904	67541	-	1638	0	36	9471	27	7770	
QEN71_RS00320	ATP-binding protein	QEN71_00320	paras_000617	protein-codi	NZ_CP1252	chromosom	67644	68948	+	1301	0	13	414	10	209	
QEN71_RS00325	response regulator transcription factor	QEN71_00325	paras_000618	protein-codi	NZ_CP1252	chromosom	68945	69487	+	539	0	1	10	1	10	TRUE
QEN71_RS00330	ATP-dependent protease ATPase subunit HslU	QEN71_00330	paras_000619	protein-codi	NZ_CP1252	chromosom	69569	70915	-	1347	0	25	6836	18	4703	
QEN71_RS00335	ATP-dependent protease subunit HslV	QEN71_00335	paras_000620	protein-codi	NZ_CP1252	chromosom	70925	71461	-	537	0	17	6686	7	1498	
QEN71_RS00340	RNA polymerase-binding protein DksA	QEN71_00340	paras_000621	protein-codi	NZ_CP1252	chromosom	71854	72273	-	420	0	0	0	0	0	TRUE
QEN71_RS00345	GTP-binding protein	QEN71_00345	paras_000622	protein-codi	NZ_CP1252	chromosom	72891	73976	-	1086	0	16	3651	12	3315	
QEN71_RS00350	class I SAM-dependent rRNA methyltransferase	QEN71_00350	paras_000623	protein-codi	NZ_CP1252	chromosom	74153	75355	-	1203	0	35	12578	27	9806	
QEN71_RS00355	tyrosine recombinase XerC	QEN71_00355	paras_000624	protein-codi	NZ_CP1252	chromosom	75441	76364	-	924	0	0	0	0	0	TRUE
QEN71_RS00360	DUF484 family protein	QEN71_00360	paras_000625	protein-codi	NZ_CP1252	chromosom	76375	77100	-	726	0	6	1403	4	1169	
QEN71_RS00365	diaminopimelate epimerase	QEN71_00365	paras_000626	protein-codi	NZ_CP1252	chromosom	77187	78047	-	861	0	0	0	0	0	TRUE
QEN71_RS00370	lipid A biosynthesis lauroyl acyltransferase	QEN71_00370	paras_000627	protein-codi	NZ_CP1252	chromosom	78102	78986	-	885	0	1	77	0	0	TRUE
QEN71_RS00375	methionine adenosyltransferase	QEN71_00375	paras_000628	protein-codi	NZ_CP1252	chromosom	79259	80446	+	1188	0	1	10	1	10	TRUE

QEN71_RS00380	hypothetical protein	QEN71_00380	paras_000629	protein-codi	NZ_CP1252	chromosom	80626	80988	-	363	0	4	1154	2	464	
QEN71_RS00385	phytanoyl-CoA dioxygenase family protein	QEN71_00385	paras_000630	protein-codi	NZ_CP1252	chromosom	81413	82174	+	762	0	13	3845	8	2015	
QEN71_RS00390	DUF3185 family protein	QEN71_00390	paras_000631	protein-codi	NZ_CP1252	chromosom	82217	82417	-	201	0	2	743	2	743	
QEN71_RS00395	L-glyceraldehyde 3-phosphate reductase	QEN71_00395	paras_000632	protein-codi	NZ_CP1252	chromosom	82755	83798	+	1044	0	33	11422	27	10317	
QEN71_RS00400	hypothetical protein	QEN71_00400	paras_000633	protein-codi	NZ_CP1252	chromosom	83915	84097	-	183	0	6	1956	2	363	
QEN71_RS00405	MFS transporter	QEN71_00405	paras_000634	protein-codi	NZ_CP1252	chromosom	84371	85642	-	1272	0	33	12320	24	9347	
QEN71_RS00410	efflux transporter outer membrane subunit	QEN71_00410	paras_000635	protein-codi	NZ_CP1252	chromosom	85785	87275	-	1491	0	24	5141	17	2471	
QEN71_RS00415	efflux RND transporter periplasmic adaptor subunit	QEN71_00415	paras_000636	protein-codi	NZ_CP1252	chromosom	87287	88555	-	1269	0	23	3536	22	3534	
QEN71_RS00420	efflux RND transporter permease subunit	QEN71_00420	paras_000637	protein-codi	NZ_CP1252	chromosom	88622	91804	-	3183	0	67	12250	53	8819	
QEN71_RS00425	hypothetical protein	QEN71_00425	paras_000638	protein-codi	NZ_CP1252	chromosom	92240	92452	+	213	0	12	1880	9	1095	
QEN71_RS00430	serine/threonine protein kinase	QEN71_00430	paras_000639	protein-codi	NZ_CP1252	chromosom	92579	93601	+	1023	0	23	3908	22	3906	
QEN71_RS00435	DUF3857 and transglutaminase domain-containing	QEN71_00435	paras_000640	protein-codi	NZ_CP1252	chromosom	93614	95626	-	2013	0	56	8332	53	7869	
QEN71_RS00440	PepSY-associated TM helix domain-containing prot	QEN71_00440	paras_000641	protein-codi	NZ_CP1252	chromosom	96006	96728	+	723	0	13	921	10	756	
QEN71_RS00445	XRE family transcriptional regulator	QEN71_00445	paras_000642	protein-codi	NZ_CP1252	chromosom	96755	97096	-	325	0	7	3580	7	3580	
QEN71_RS00450	type II toxin-antitoxin system RelE/ParE family toxin	QEN71_00450	paras_000643	protein-codi	NZ_CP1252	chromosom	97080	97454	-	358	0	18	1680	17	1637	
QEN71_RS00455	XdhC family protein	QEN71_00455	paras_000644	protein-codi	NZ_CP1252	chromosom	97669	98697	-	1001	0	21	2461	20	2446	
QEN71_RS00460	carbon monoxide dehydrogenase subunit G	QEN71_00460	paras_000645	protein-codi	NZ_CP1252	chromosom	98670	99251	-	544	0	8	2084	6	1418	
QEN71_RS00465	VWA domain-containing protein	QEN71_00465	paras_000646	protein-codi	NZ_CP1252	chromosom	99242	100483	-	1232	0	19	3927	15	2840	
QEN71_RS00470	MoxR family ATPase	QEN71_00470	paras_000647	protein-codi	NZ_CP1252	chromosom	100489	101376	-	888	0	20	2223	14	1612	
QEN71_RS00475	xanthine dehydrogenase family protein subunit M	QEN71_00475	paras_000648	protein-codi	NZ_CP1252	chromosom	101473	102270	-	798	0	14	3771	5	961	
QEN71_RS00480	xanthine dehydrogenase family protein molybdopter	QEN71_00480	paras_000649	protein-codi	NZ_CP1252	chromosom	102283	104670	-	2388	0	65	16488	59	15091	
QEN71_RS00485	(2Fe-2S)-binding protein	QEN71_00485	paras_000650	protein-codi	NZ_CP1252	chromosom	104681	105181	-	501	0	4	949	4	949	
QEN71_RS00490	hypothetical protein	QEN71_00490	paras_000651	protein-codi	NZ_CP1252	chromosom	105469	105735	-	267	171.0	12	1624	11	1456	
QEN71_RS00495	IS481 family transposase	QEN71_00495	paras_000652	protein-codi	NZ_CP1252	chromosom	106022	107161	-	1140	2266.0	30	27483	25	23194	
QEN71_RS00500	amino acid permease	QEN71_00500	paras_000653	protein-codi	NZ_CP1252	chromosom	107336	108733	-	1398	0	32	10941	22	8916	
QEN71_RS00505	response regulator transcription factor RqpR	QEN71_00505	paras_000654	protein-codi	NZ_CP1252	chromosom	108876	109532	-	657	0	27	10678	22	6886	
QEN71_RS00510	histidine kinase	QEN71_00510	paras_000655	protein-codi	NZ_CP1252	chromosom	109585	110439	-	855	0	0	0	0	0	TRUE
QEN71_RS00515	ferredoxin--NADP reductase	QEN71_00515	paras_000656	protein-codi	NZ_CP1252	chromosom	111006	111776	-	771	0	2	128	0	0	TRUE
QEN71_RS00520	LysE family transporter	QEN71_00520	paras_000657	protein-codi	NZ_CP1252	chromosom	112280	112921	-	642	0	22	6242	15	4315	
QEN71_RS00525	ABC transporter permease	QEN71_00525	paras_000658	protein-codi	NZ_CP1252	chromosom	113016	113996	-	981	0	12	2145	9	1479	
QEN71_RS00530	ABC transporter permease	QEN71_00530	paras_000659	protein-codi	NZ_CP1252	chromosom	114019	115020	-	991	0	23	4844	16	3262	
QEN71_RS00535	sugar ABC transporter ATP-binding protein	QEN71_00535	paras_000660	protein-codi	NZ_CP1252	chromosom	115010	116587	-	1567	0	15	5423	13	5274	
QEN71_RS00540	ABC transporter substrate-binding protein	QEN71_00540	paras_000661	protein-codi	NZ_CP1252	chromosom	116887	117891	-	1005	0	10	3121	4	1813	
QEN71_RS00545	LacI family DNA-binding transcriptional regulator	QEN71_00545	paras_000662	protein-codi	NZ_CP1252	chromosom	118005	119057	-	1053	0	25	6103	22	6052	
QEN71_RS00550	aldose 1-epimerase	QEN71_00550	paras_000663	protein-codi	NZ_CP1252	chromosom	119378	120361	+	984	0	24	6291	23	5958	
QEN71_RS00555	endonuclease/exonuclease/phosphatase family pro	QEN71_00555	paras_000664	protein-codi	NZ_CP1252	chromosom	120375	121172	-	798	0	10	5082	3	101	
QEN71_RS00560	hypothetical protein	QEN71_00560	paras_000665	protein-codi	NZ_CP1252	chromosom	121201	121707	-	507	0	4	269	0	0	TRUE
QEN71_RS00565	AAA family ATPase	QEN71_00565	paras_000666	protein-codi	NZ_CP1252	chromosom	122036	123013	+	978	0	23	8058	19	6808	
QEN71_RS00570	DUF4148 domain-containing protein	QEN71_00570	paras_000667	protein-codi	NZ_CP1252	chromosom	123240	123527	+	288	0	6	2075	6	2075	
QEN71_RS00575	NAD(P)/FAD-dependent oxidoreductase	QEN71_00575	paras_000668	protein-codi	NZ_CP1252	chromosom	123820	124926	+	1107	0	26	10622	26	10622	
QEN71_RS00580	high-potential iron-sulfur protein	QEN71_00580	paras_000669	protein-codi	NZ_CP1252	chromosom	125098	125412	+	315	0	19	5586	10	2452	
QEN71_RS00585	MFS transporter	QEN71_00585	paras_000670	protein-codi	NZ_CP1252	chromosom	125671	126981	+	1311	0	37	8200	27	5110	
QEN71_RS00590	amidase	QEN71_00590	paras_000671	protein-codi	NZ_CP1252	chromosom	126985	128481	-	1497	0	23	5608	21	4889	
QEN71_RS00595	dienelactone hydrolase family protein	QEN71_00595	paras_000672	protein-codi	NZ_CP1252	chromosom	128588	129286	-	699	0	35	14864	26	11383	
QEN71_RS00600	branched-chain amino acid ABC transporter substra	QEN71_00600	paras_000673	protein-codi	NZ_CP1252	chromosom	129973	131118	+	1146	0	34	16274	28	10290	
QEN71_RS00605	cupin-like domain-containing protein	QEN71_00605	paras_000674	protein-codi	NZ_CP1252	chromosom	131281	132279	+	999	0	49	21848	39	16587	
QEN71_RS00610	MFS transporter	QEN71_00610	paras_000675	protein-codi	NZ_CP1252	chromosom	132284	133354	-	1071	0	21	8146	21	8146	
QEN71_RS00615	hypothetical protein	QEN71_00615		protein-codi	NZ_CP1252	chromosom	133364	133495	-	132	0	6	7398	0	0	
QEN71_RS00620	pyridoxamine 5'-phosphate oxidase family protein	QEN71_00620	paras_000676	protein-codi	NZ_CP1252	chromosom	133728	134393	+	666	0	23	5733	21	5508	
QEN71_RS00625	H-NS histone family protein	QEN71_00625	paras_000677	protein-codi	NZ_CP1252	chromosom	134752	135054	+	303	0	13	2418	8	1215	
QEN71_RS00630	cation diffusion facilitator family transporter	QEN71_00630	paras_000678	protein-codi	NZ_CP1252	chromosom	135303	136505	+	1203	0	27	16655	22	13180	
QEN71_RS00635	Lrp/AsnC family transcriptional regulator	QEN71_00635	paras_000679	protein-codi	NZ_CP1252	chromosom	136515	136973	-	459	0	18	7487	13	6592	
QEN71_RS00640	exonuclease	QEN71_00640	paras_000680	protein-codi	NZ_CP1252	chromosom	137077	137691	-	611	0	17	9622	13	6657	
QEN71_RS00645	MBL fold metallo-hydrolase	QEN71_00645	paras_000681	protein-codi	NZ_CP1252	chromosom	137688	138332	-	641	0	11	2841	8	1655	
QEN71_RS00650	septal ring lytic transglycosylase RlpA family protein	QEN71_00650	paras_000682	protein-codi	NZ_CP1252	chromosom	138985	139584	+	600	0	25	7191	20	4827	
QEN71_RS00655	16S rRNA (cytidine(1402)-2'-O)-methyltransferase	QEN71_00655	paras_000683	protein-codi	NZ_CP1252	chromosom	139660	140535	-	874	0	21	8799	14	7090	
QEN71_RS00660	YraN family protein	QEN71_00660	paras_000684	protein-codi	NZ_CP1252	chromosom	140534	141004	+	469	0	5	742	5	742	
QEN71_RS00665	phosphoheptose isomerase	QEN71_00665	paras_000685	protein-codi	NZ_CP1252	chromosom	141169	141756	+	588	0	12	4022	9	3006	
QEN71_RS00670	BON domain-containing protein	QEN71_00670	paras_000686	protein-codi	NZ_CP1252	chromosom	141777	142580	+	800	0	29	10262	21	7484	
QEN71_RS00675	c-type cytochrome	QEN71_00675	paras_000687	protein-codi	NZ_CP1252	chromosom	142577	142921	+	341	0	15	5345	8	2450	
QEN71_RS00680	tRNA-Ala	QEN71_00680		tRNA	NZ_CP1252	chromosom	142977	143052	+	76	129.0	0	0	0	0	
QEN71_RS00685	tyrosine-type recombinase/integrase	QEN71_00685	paras_000689	protein-codi	NZ_CP1252	chromosom	143150	144724	+	1575	0	55	20621	32	14691	

QEN71_RS00690	DEAD/DEAH box helicase	QEN71_00690	paras_000690	protein-codi	NZ_CP1252	chromosom	146102	151267	+	5166	0	219	62999	171	46328	
QEN71_RS00695	DEAD/DEAH box helicase	QEN71_00695	paras_000691	protein-codi	NZ_CP1252	chromosom	151271	154036	+	2766	0	47	9882	30	6224	
QEN71_RS00700	N-6 DNA methylase	QEN71_00700	paras_000692	protein-codi	NZ_CP1252	chromosom	154040	157948	+	3905	0	161	32316	123	24225	
QEN71_RS00705	HNH endonuclease	QEN71_00705	paras_000693	protein-codi	NZ_CP1252	chromosom	157945	158703	+	755	0	27	7124	23	6124	
QEN71_RS00710	DUF4365 domain-containing protein	QEN71_00710	paras_000694	protein-codi	NZ_CP1252	chromosom	158963	159463	+	497	0	25	5399	19	3739	
QEN71_RS00715	hypothetical protein	QEN71_00715	paras_000695	protein-codi	NZ_CP1252	chromosom	159460	160620	+	1157	0	58	11320	54	10076	
QEN71_RS00720	AAA family ATPase	QEN71_00720	paras_000696	protein-codi	NZ_CP1252	chromosom	160786	162957	+	2172	0	97	18883	84	17049	
QEN71_RS00725	hypothetical protein	QEN71_00725	paras_000697	protein-codi	NZ_CP1252	chromosom	163150	164439	+	1290	0	51	11062	41	8639	
QEN71_RS00730	hypothetical protein	QEN71_00730	paras_000698	protein-codi	NZ_CP1252	chromosom	164926	165165	-	240	0	11	3508	6	860	
QEN71_RS00735	IS21-like element helper ATPase IstB	QEN71_00735	paras_000699	protein-codi	NZ_CP1252	chromosom	166799	167587	-	789	1561.0	9	69	8	67	
QEN71_RS00740	IS21 family transposase	QEN71_00740	paras_000700	protein-codi	NZ_CP1252	chromosom	167594	169117	-	1524	3027.0	10	41	9	38	
QEN71_RS00745	hypothetical protein	QEN71_00745	paras_000701	protein-codi	NZ_CP1252	chromosom	169754	169999	+	242	0	8	1294	5	766	
QEN71_RS00750	hypothetical protein	QEN71_00750	paras_000702	protein-codi	NZ_CP1252	chromosom	169996	170217	+	218	0	6	1361	4	648	
QEN71_RS00755	hypothetical protein	QEN71_00755	paras_000703	protein-codi	NZ_CP1252	chromosom	170218	170481	-	264	0	16	2648	13	2297	
QEN71_RS00760	hypothetical protein	QEN71_00760	paras_000704	protein-codi	NZ_CP1252	chromosom	170595	171218	-	624	0	26	4732	23	4397	
QEN71_RS00765	hypothetical protein	QEN71_00765	paras_000705	protein-codi	NZ_CP1252	chromosom	171394	171582	+	189	0	7	1275	6	751	
QEN71_RS00770	ribonuclease H-like domain-containing protein	QEN71_00770	paras_000706	protein-codi	NZ_CP1252	chromosom	171638	171907	+	270	0	10	1576	6	674	
QEN71_RS00775	hypothetical protein	QEN71_00775	paras_000707	protein-codi	NZ_CP1252	chromosom	172045	172836	-	792	0	40	9373	26	5037	
QEN71_RS00780	hypothetical protein	QEN71_00780	paras_000708	protein-codi	NZ_CP1252	chromosom	173571	173939	+	369	0	19	7288	17	6914	
QEN71_RS00785	hypothetical protein	QEN71_00785	paras_000709	protein-codi	NZ_CP1252	chromosom	174625	175017	+	393	0	23	2547	16	1828	
QEN71_RS00790	hypothetical protein	QEN71_00790	paras_000710	protein-codi	NZ_CP1252	chromosom	175027	175794	+	768	0	13	2613	11	2412	
QEN71_RS00795	metallophosphoesterase	QEN71_00795	paras_000711	protein-codi	NZ_CP1252	chromosom	176610	177419	+	810	0	40	10305	38	9874	
QEN71_RS00800	dTMP kinase	QEN71_00800	paras_000712	protein-codi	NZ_CP1252	chromosom	177421	178041	+	617	0	18	2236	15	2125	
QEN71_RS00805	hypothetical protein	QEN71_00805	paras_000713	protein-codi	NZ_CP1252	chromosom	178038	178937	+	886	0	51	9836	43	7736	
QEN71_RS00810	MazG nucleotide pyrophosphohydrolase domain-co	QEN71_00810	paras_000714	protein-codi	NZ_CP1252	chromosom	178928	179272	+	331	0	2	6	1	3	
QEN71_RS00815	thymidylate synthase	QEN71_00815	paras_000715	protein-codi	NZ_CP1252	chromosom	179269	180198	+	926	0	44	3834	37	3036	
QEN71_RS00820	hypothetical protein	QEN71_00820	paras_000716	protein-codi	NZ_CP1252	chromosom	182860	183273	+	414	0	33	9574	29	9256	
QEN71_RS00825	hypothetical protein	QEN71_00825	paras_000717	protein-codi	NZ_CP1252	chromosom	183281	183421	-	141	0	2	901	2	901	
QEN71_RS00830	P27 family phage terminase small subunit			pseudogene	NZ_CP1252	chromosom	183941	184216	+	276	0	5	1495	4	1488	
QEN71_RS00835	AlpA family phage regulatory protein			protein-codi	NZ_CP1252	chromosom	184334	184525	+	192	0	5	2198	5	2198	
QEN71_RS00840	tRNA-Ala	QEN71_00840		tRNA	NZ_CP1252	chromosom	184669	184740	+	72	129.0	2	259	0	0	
QEN71_RS00845	OpgC domain-containing protein	QEN71_00845	paras_000721	protein-codi	NZ_CP1252	chromosom	184982	186088	+	1103	0	43	11208	35	8971	
QEN71_RS00850	tannase/feruloyl esterase family alpha/beta hydrolas	QEN71_00850	paras_000722	protein-codi	NZ_CP1252	chromosom	186085	187794	+	1706	0	61	17798	49	13421	
QEN71_RS00855	STAS domain-containing protein	QEN71_00855	paras_000723	protein-codi	NZ_CP1252	chromosom	188044	188907	+	864	0	9	2819	5	1775	
QEN71_RS00860	STAS domain-containing protein	QEN71_00860	paras_000724	protein-codi	NZ_CP1252	chromosom	188917	189273	+	357	0	1	3	1	3	TRUE
QEN71_RS00865	anti-sigma regulatory factor	QEN71_00865	paras_000725	protein-codi	NZ_CP1252	chromosom	189293	189718	+	410	0	3	226	1	128	TRUE
QEN71_RS00870	ATP-binding protein	QEN71_00870	paras_000726	protein-codi	NZ_CP1252	chromosom	189703	190881	+	1163	0	8	559	4	263	
QEN71_RS00875	ATP-binding protein	QEN71_00875	paras_000727	protein-codi	NZ_CP1252	chromosom	190939	192408	+	1466	0	25	5679	23	5604	
QEN71_RS00880	response regulator	QEN71_00880	paras_000728	protein-codi	NZ_CP1252	chromosom	192405	194075	+	1667	0	20	3889	20	3889	
QEN71_RS00885	response regulator	QEN71_00885	paras_000729	protein-codi	NZ_CP1252	chromosom	194217	199118	+	4902	0	80	19138	60	13520	
QEN71_RS00890	spore coat protein U domain-containing protein	QEN71_00890	paras_000730	protein-codi	NZ_CP1252	chromosom	199134	200102	-	969	0	36	9173	27	6287	
QEN71_RS00895	fimbria/pilus outer membrane usher protein	QEN71_00895	paras_000731	protein-codi	NZ_CP1252	chromosom	200105	202546	-	2442	0	59	9633	51	6940	
QEN71_RS00900	molecular chaperone	QEN71_00900	paras_000732	protein-codi	NZ_CP1252	chromosom	202671	203498	-	828	0	4	606	4	606	
QEN71_RS00905	spore coat U domain-containing protein	QEN71_00905	paras_000733	protein-codi	NZ_CP1252	chromosom	203596	204105	-	510	0	16	6473	13	4840	
QEN71_RS00910	hypothetical protein	QEN71_00910	paras_000734	protein-codi	NZ_CP1252	chromosom	204281	204604	-	324	0	5	3578	5	3578	
QEN71_RS00915	DUF2239 family protein	QEN71_00915	paras_000735	protein-codi	NZ_CP1252	chromosom	204803	205417	+	615	0	7	1616	7	1616	
QEN71_RS00920	methyl-accepting chemotaxis protein	QEN71_00920	paras_000736	protein-codi	NZ_CP1252	chromosom	205759	207309	+	1551	0	25	4017	23	3773	
QEN71_RS00925	solute carrier family 23 protein	QEN71_00925	paras_000737	protein-codi	NZ_CP1252	chromosom	207483	208790	+	1308	0	25	5047	21	4483	
QEN71_RS00930	Na+/H+ antiporter	QEN71_00930	paras_000738	protein-codi	NZ_CP1252	chromosom	208851	210515	-	1665	0	20	4716	15	3544	
QEN71_RS00935	EAL domain-containing protein	QEN71_00935	paras_000739	protein-codi	NZ_CP1252	chromosom	210919	212193	+	1275	0	23	3062	17	1744	
QEN71_RS00940	copper homeostasis protein CutC	QEN71_00940	paras_000740	protein-codi	NZ_CP1252	chromosom	212227	212916	+	690	0	22	3728	18	3539	
QEN71_RS00945	indolepyruvate ferredoxin oxidoreductase family pro	QEN71_00945	paras_000741	protein-codi	NZ_CP1252	chromosom	213517	217110	-	3594	0	71	14970	54	12868	
QEN71_RS00950	IS481 family transposase	QEN71_00950	paras_000742	protein-codi	NZ_CP1252	chromosom	217852	218991	+	1140	2261.0	6	3236	5	3019	
QEN71_RS00955	glutathione S-transferase	QEN71_00955	paras_000743	protein-codi	NZ_CP1252	chromosom	219201	219818	+	618	0	14	4318	6	2008	
QEN71_RS00960	ion channel	QEN71_00960	paras_000744	protein-codi	NZ_CP1252	chromosom	219840	220781	-	942	0	22	6727	18	6275	
QEN71_RS00965	hypothetical protein	QEN71_00965	paras_000745	protein-codi	NZ_CP1252	chromosom	220937	221182	-	246	0	9	2013	8	1931	
QEN71_RS00970	hypothetical protein	QEN71_00970	paras_000746	protein-codi	NZ_CP1252	chromosom	221272	221493	-	222	0	18	6883	11	3895	
QEN71_RS00975	carbohydrate kinase	QEN71_00975	paras_000747	protein-codi	NZ_CP1252	chromosom	221648	222562	-	915	0	10	1830	8	1610	
QEN71_RS00980	AGE family epimerase/isomerase	QEN71_00980	paras_000748	protein-codi	NZ_CP1252	chromosom	222581	223843	-	1259	0	47	14841	38	12124	
QEN71_RS00985	LacI family DNA-binding transcriptional regulator	QEN71_00985	paras_000749	protein-codi	NZ_CP1252	chromosom	223840	224826	-	983	0	11	3709	6	1764	
QEN71_RS00990	phosphodiesterase	QEN71_00990	paras_000750	protein-codi	NZ_CP1252	chromosom	225163	227001	-	1839	0	30	12095	21	5000	
QEN71_RS00995	bile acid:sodium symporter family protein	QEN71_00995	paras_000751	protein-codi	NZ_CP1252	chromosom	227281	228303	-	1023	0	16	4198	14	3759	

QEN71_RS01000	hypothetical protein	QEN71_01000	paras_000752	protein-codi	NZ_CP1252	chromosom	228859	229410	+	552	0	14	3009	12	2093	
QEN71_RS01005	porin	QEN71_01005	paras_000753	protein-codi	NZ_CP1252	chromosom	229960	231075	+	1116	0	65	21241	48	16792	
QEN71_RS01010	LysR family transcriptional regulator	QEN71_01010	paras_000754	protein-codi	NZ_CP1252	chromosom	231247	232185	+	939	0	17	3630	15	3471	
QEN71_RS01015	methylmalonyl-CoA mutase family protein	QEN71_01015	paras_000755	protein-codi	NZ_CP1252	chromosom	232310	235696	+	3387	0	72	17592	59	14757	
QEN71_RS01020	nitronate monooxygenase family protein	QEN71_01020	paras_000756	protein-codi	NZ_CP1252	chromosom	235774	236733	-	960	0	15	6372	13	6253	
QEN71_RS01025	alpha/beta hydrolase	QEN71_01025	paras_000757	protein-codi	NZ_CP1252	chromosom	237007	237897	+	891	0	17	5051	13	3028	
QEN71_RS01030	Lrp/AsnC family transcriptional regulator	QEN71_01030	paras_000758	protein-codi	NZ_CP1252	chromosom	237925	238410	-	486	0	5	480	4	471	
QEN71_RS01035	DMT family transporter	QEN71_01035	paras_000759	protein-codi	NZ_CP1252	chromosom	238524	239432	+	909	0	26	7664	21	6641	
QEN71_RS01040	MBL fold metallo-hydrolase	QEN71_01040	paras_000760	protein-codi	NZ_CP1252	chromosom	239673	240593	-	917	0	11	708	8	523	
QEN71_RS01045	MaoC family dehydratase	QEN71_01045	paras_000761	protein-codi	NZ_CP1252	chromosom	240590	241045	-	448	0	13	1781	9	1074	
QEN71_RS01050	DUF1289 domain-containing protein	QEN71_01050	paras_000762	protein-codi	NZ_CP1252	chromosom	241042	241290	-	241	0	0	0	0	0	
QEN71_RS01055	YbaK/EbsC family protein	QEN71_01055	paras_000763	protein-codi	NZ_CP1252	chromosom	241287	241820	-	530	0	8	1791	4	595	
QEN71_RS01060	hydroxymethylglutaryl-CoA lyase	QEN71_01060	paras_000764	protein-codi	NZ_CP1252	chromosom	241842	242768	-	927	0	15	2525	15	2525	
QEN71_RS01065	glyoxylate/hydroxypyruvate reductase A	QEN71_01065	paras_000765	protein-codi	NZ_CP1252	chromosom	242841	243782	-	942	0	26	4914	20	3835	
QEN71_RS01070	alpha/beta hydrolase	QEN71_01070	paras_000766	protein-codi	NZ_CP1252	chromosom	244027	244629	+	603	0	11	2375	11	2375	
QEN71_RS01075	hypothetical protein	QEN71_01075	paras_000767	protein-codi	NZ_CP1252	chromosom	244656	244886	-	231	0	1	103	1	103	
QEN71_RS01080	sulfate ABC transporter substrate-binding protein	QEN71_01080	paras_000768	protein-codi	NZ_CP1252	chromosom	245251	246276	+	1026	0	35	6153	29	6023	
QEN71_RS01085	biotin synthase BioB	QEN71_01085	paras_000769	protein-codi	NZ_CP1252	chromosom	246842	247909	-	1068	0	15	815	13	80	
QEN71_RS01090	dethiobiotin synthase	QEN71_01090	paras_000770	protein-codi	NZ_CP1252	chromosom	247946	248665	-	716	0	5	116	4	95	
QEN71_RS01095	8-amino-7-oxononanoate synthase	QEN71_01095	paras_000771	protein-codi	NZ_CP1252	chromosom	248662	249846	-	1180	0	8	209	6	165	
QEN71_RS01100	adenosylmethionine--8-amino-7-oxononanoate tran	QEN71_01100	paras_000772	protein-codi	NZ_CP1252	chromosom	249846	251207	-	1361	0	17	326	14	269	
QEN71_RS01105	prolyl oligopeptidase family serine peptidase	QEN71_01105	paras_000773	protein-codi	NZ_CP1252	chromosom	251890	253179	-	1290	0	35	10528	33	10160	
QEN71_RS01110	YchJ family protein	QEN71_01110	paras_000774	protein-codi	NZ_CP1252	chromosom	253740	254186	-	447	0	21	4017	19	3749	
QEN71_RS01115	acyl-CoA dehydrogenase family protein	QEN71_01115	paras_000775	protein-codi	NZ_CP1252	chromosom	254205	255335	-	1131	0	28	6677	17	3311	
QEN71_RS01120	SDR family oxidoreductase	QEN71_01120	paras_000776	protein-codi	NZ_CP1252	chromosom	255385	256062	-	678	0	12	1537	8	984	
QEN71_RS01125	acetyl-CoA C-acetyltransferase	QEN71_01125	paras_000777	protein-codi	NZ_CP1252	chromosom	256127	257317	-	1183	0	9	1578	7	995	
QEN71_RS01130	carbonate dehydratase	QEN71_01130	paras_000778	protein-codi	NZ_CP1252	chromosom	257310	258080	-	763	0	12	274	8	97	
QEN71_RS01135	bifunctional isocitrate dehydrogenase kinase/phosp	QEN71_01135	paras_000779	protein-codi	NZ_CP1252	chromosom	258139	259947	-	1809	0	64	9666	57	8825	
QEN71_RS01140	MerR family DNA-binding transcriptional regulator	QEN71_01140	paras_000780	protein-codi	NZ_CP1252	chromosom	260146	260595	-	450	0	12	2082	10	1959	
QEN71_RS01145	MBL fold metallo-hydrolase	QEN71_01145	paras_000781	protein-codi	NZ_CP1252	chromosom	260773	261843	+	1071	0	18	4656	14	4407	
QEN71_RS01150	ABC transporter substrate-binding protein	QEN71_01150	paras_000782	protein-codi	NZ_CP1252	chromosom	261933	263528	-	1596	0	40	10660	34	8889	
QEN71_RS01155	helical backbone metal receptor	QEN71_01155	paras_000783	protein-codi	NZ_CP1252	chromosom	263741	264562	-	822	0	21	9506	15	8095	
QEN71_RS01160	SDR family oxidoreductase	QEN71_01160	paras_000784	protein-codi	NZ_CP1252	chromosom	264566	265336	-	771	0	22	4574	15	3639	
QEN71_RS01165	thiol:disulfide interchange protein DsbA/DsbL	QEN71_01165	paras_000785	protein-codi	NZ_CP1252	chromosom	265354	265992	-	639	0	20	4233	15	2665	
QEN71_RS01170	SPOR domain-containing protein	QEN71_01170	paras_000786	protein-codi	NZ_CP1252	chromosom	266153	266947	-	795	0	8	246	7	231	
QEN71_RS01175	arginine--tRNA ligase	QEN71_01175	paras_000787	protein-codi	NZ_CP1252	chromosom	267048	268832	-	1785	0	3	119	1	4	TRUE
QEN71_RS01180	DUF1840 domain-containing protein	QEN71_01180	paras_000788	protein-codi	NZ_CP1252	chromosom	269219	269545	+	327	0	12	3630	6	2062	
QEN71_RS01185	acid-shock protein	QEN71_01185	paras_000789	protein-codi	NZ_CP1252	chromosom	269625	269831	-	207	0	5	581	2	499	
QEN71_RS01190	methionine synthase	QEN71_01190	paras_000790	protein-codi	NZ_CP1252	chromosom	270008	272725	-	2718	0	60	13253	49	9039	
QEN71_RS01195	homocysteine S-methyltransferase family protein	QEN71_01195	paras_000791	protein-codi	NZ_CP1252	chromosom	272819	273883	-	1065	0	23	9892	22	9883	
QEN71_RS01200	DUF3567 domain-containing protein	QEN71_01200	paras_000792	protein-codi	NZ_CP1252	chromosom	274198	274455	-	258	0	11	4983	9	4280	
QEN71_RS01205	3-oxoadipate enol-lactonase	QEN71_01205	paras_000793	protein-codi	NZ_CP1252	chromosom	274573	275361	-	789	0	22	6580	17	4812	
QEN71_RS01210	DUF3108 domain-containing protein	QEN71_01210	paras_000794	protein-codi	NZ_CP1252	chromosom	275597	276808	-	1212	0	28	10590	24	9324	
QEN71_RS01215	IclR family transcriptional regulator	QEN71_01215	paras_000795	protein-codi	NZ_CP1252	chromosom	276887	277777	-	891	0	17	3581	9	1765	
QEN71_RS01220	fumarylacetoacetate hydrolase family protein	QEN71_01220	paras_000796	protein-codi	NZ_CP1252	chromosom	277905	278897	+	993	0	16	3765	14	3654	
QEN71_RS01225	ABC transporter substrate-binding protein	QEN71_01225	paras_000797	protein-codi	NZ_CP1252	chromosom	279096	280289	+	1194	0	30	13281	27	12986	
QEN71_RS01230	enoyl-CoA hydratase/isomerase family protein	QEN71_01230	paras_000798	protein-codi	NZ_CP1252	chromosom	280381	281232	+	852	0	21	7985	16	5433	
QEN71_RS01235	transcriptional regulator	QEN71_01235	paras_000799	protein-codi	NZ_CP1252	chromosom	281413	282159	+	747	0	0	0	0	0	TRUE
QEN71_RS01240	acyl-ACP desaturase	QEN71_01240	paras_000800	protein-codi	NZ_CP1252	chromosom	282681	283553	+	873	0	13	809	10	680	
QEN71_RS01245	D-glycero-beta-D-manno-heptose 1-phosphate ade	QEN71_01245	paras_000801	protein-codi	NZ_CP1252	chromosom	283689	284171	+	483	0	2	14	1	3	TRUE
QEN71_RS01250	hypothetical protein	QEN71_01250	paras_000802	protein-codi	NZ_CP1252	chromosom	284214	284450	-	237	0	2	109	0	0	
QEN71_RS01255	type III pantothenate kinase	QEN71_01255	paras_000803	protein-codi	NZ_CP1252	chromosom	284491	285345	-	851	0	0	0	0	0	TRUE
QEN71_RS01260	biotin--[acetyl-CoA-carboxylase] ligase	QEN71_01260	paras_000804	protein-codi	NZ_CP1252	chromosom	285342	286247	-	902	0	1	12	0	0	TRUE
QEN71_RS01265	ABC transporter permease	QEN71_01265	paras_000805	protein-codi	NZ_CP1252	chromosom	286434	287573	+	1136	0	29	12177	26	10362	
QEN71_RS01270	ATP-binding cassette domain-containing protein	QEN71_01270	paras_000806	protein-codi	NZ_CP1252	chromosom	287570	288460	+	887	0	6	1033	5	1022	
QEN71_RS01275	MlaD family protein	QEN71_01275	paras_000807	protein-codi	NZ_CP1252	chromosom	288479	289441	+	963	0	13	2489	11	2090	
QEN71_RS01280	ABC-type transport auxiliary lipoprotein family prote	QEN71_01280	paras_000808	protein-codi	NZ_CP1252	chromosom	289460	290086	+	627	0	5	344	5	344	
QEN71_RS01285	VanZ family protein	QEN71_01285	paras_000809	protein-codi	NZ_CP1252	chromosom	290117	291277	+	1161	0	26	7567	16	4958	
QEN71_RS01290	ferredoxin	QEN71_01290	paras_000810	protein-codi	NZ_CP1252	chromosom	291408	291731	+	324	0	15	5678	15	5678	
QEN71_RS01295	alpha/beta hydrolase	QEN71_01295	paras_000811	protein-codi	NZ_CP1252	chromosom	291765	292409	+	645	0	17	8200	10	4107	
QEN71_RS01300	D-alanyl-D-alanine carboxypeptidase family protein	QEN71_01300	paras_000812	protein-codi	NZ_CP1252	chromosom	292807	294036	+	1230	0	34	18634	31	16932	
QEN71_RS01305	DUF493 family protein	QEN71_01305	paras_000813	protein-codi	NZ_CP1252	chromosom	294133	294402	+	270	0	9	2022	5	1015	

QEN71_RS01310	transcriptional regulator GcvA	QEN71_01310	paras_000814	protein-codi	NZ_CP1252	chromosom	294427	295431	-	1005	0	14	8436	10	5405	
QEN71_RS01315	DUF2917 domain-containing protein	QEN71_01315	paras_000815	protein-codi	NZ_CP1252	chromosom	295530	295856	+	327	0	5	538	3	247	
QEN71_RS01320	lipoyl(octanoyl) transferase LipB	QEN71_01320	paras_000816	protein-codi	NZ_CP1252	chromosom	295930	296697	+	768	0	0	0	0	0	TRUE
QEN71_RS01325	lipoyl synthase	QEN71_01325	paras_000817	protein-codi	NZ_CP1252	chromosom	296711	297712	+	1002	0	2	11	2	11	TRUE
QEN71_RS01330	Na/Pi cotransporter family protein	QEN71_01330	paras_000818	protein-codi	NZ_CP1252	chromosom	297952	299634	+	1683	0	27	5230	21	3667	
QEN71_RS01335	DeoR/GlpR family DNA-binding transcription regula	QEN71_01335	paras_000819	protein-codi	NZ_CP1252	chromosom	299657	300430	-	774	0	18	2421	11	1691	
QEN71_RS01340	glycerophosphodiester phosphodiesterase	QEN71_01340	paras_000820	protein-codi	NZ_CP1252	chromosom	300869	301633	+	765	0	11	1975	6	997	
QEN71_RS01345	ABC transporter substrate-binding protein	QEN71_01345	paras_000821	protein-codi	NZ_CP1252	chromosom	301720	302817	+	1098	0	39	6223	33	5257	
QEN71_RS01350	ABC transporter permease subunit	QEN71_01350	paras_000822	protein-codi	NZ_CP1252	chromosom	302928	303869	+	938	0	31	6802	23	5623	
QEN71_RS01355	ABC transporter permease	QEN71_01355	paras_000823	protein-codi	NZ_CP1252	chromosom	303866	304657	+	788	0	23	4138	21	4111	
QEN71_RS01360	ABC transporter ATP-binding protein	QEN71_01360	paras_000824	protein-codi	NZ_CP1252	chromosom	304679	305752	+	1074	0	13	5142	9	3511	
QEN71_RS01365	dicarboxylate/amino acid:cation symporter	QEN71_01365	paras_000825	protein-codi	NZ_CP1252	chromosom	306116	307396	+	1281	0	32	8450	18	5152	
QEN71_RS01370	ATP-binding protein	QEN71_01370	paras_000826	protein-codi	NZ_CP1252	chromosom	307400	309424	+	2025	0	46	11582	39	10504	
QEN71_RS01375	sigma-54 dependent transcriptional regulator	QEN71_01375	paras_000827	protein-codi	NZ_CP1252	chromosom	309454	310812	+	1359	0	24	4369	16	1915	
QEN71_RS01380	TlpA disulfide reductase family protein	QEN71_01380	paras_000828	protein-codi	NZ_CP1252	chromosom	310891	311415	-	525	0	21	4544	17	3752	
QEN71_RS01385	hypothetical protein	QEN71_01385	paras_000829	protein-codi	NZ_CP1252	chromosom	311646	312242	+	597	0	20	6180	14	5596	
QEN71_RS01390	YifB family Mg chelatase-like AAA ATPase	QEN71_01390	paras_000830	protein-codi	NZ_CP1252	chromosom	312576	314138	-	1563	0	35	14350	22	10503	
QEN71_RS01395	accessory factor UbiK family protein	QEN71_01395	paras_000831	protein-codi	NZ_CP1252	chromosom	314239	314493	-	255	0	0	0	0	0	
QEN71_RS01400	P-II family nitrogen regulator	QEN71_01400	paras_000832	protein-codi	NZ_CP1252	chromosom	314907	315245	+	339	0	14	3383	10	3023	
QEN71_RS01405	ammonium transporter	QEN71_01405	paras_000833	protein-codi	NZ_CP1252	chromosom	315272	316777	+	1506	0	31	9610	21	6561	
QEN71_RS01410	glutamate--cysteine ligase	QEN71_01410	paras_000834	protein-codi	NZ_CP1252	chromosom	317033	318322	+	1290	0	49	29240	33	17133	
QEN71_RS01415	glutathione synthase	QEN71_01415	paras_000835	protein-codi	NZ_CP1252	chromosom	318492	319448	+	957	0	22	8463	14	5971	
QEN71_RS01420	PTS sugar transporter subunit IIB	QEN71_01420	paras_000836	protein-codi	NZ_CP1252	chromosom	319729	320241	+	513	0	9	1117	9	1117	
QEN71_RS01425	HPr family phosphocarrier protein	QEN71_01425	paras_000837	protein-codi	NZ_CP1252	chromosom	320301	320570	+	270	0	6	1114	2	148	
QEN71_RS01430	phosphoenolpyruvate--protein phosphotransferase	QEN71_01430	paras_000838	protein-codi	NZ_CP1252	chromosom	320790	322532	+	1743	0	37	17730	32	14777	
QEN71_RS01435	molybdopterin-synthase adenylyltransferase MoeB	QEN71_01435	paras_000839	protein-codi	NZ_CP1252	chromosom	322536	323294	-	759	0	3	424	0	0	TRUE
QEN71_RS01440	S41 family peptidase	QEN71_01440	paras_000840	protein-codi	NZ_CP1252	chromosom	323439	325031	-	1593	0	33	4098	26	1822	
QEN71_RS01445	2,3-diphosphoglycerate-dependent phosphoglycera	QEN71_01445	paras_000841	protein-codi	NZ_CP1252	chromosom	325426	326172	-	747	0	19	1288	13	514	
QEN71_RS01450	rhodanese-like domain-containing protein	QEN71_01450	paras_000842	protein-codi	NZ_CP1252	chromosom	326325	326750	+	422	0	15	7099	11	4400	
QEN71_RS01455	glutaredoxin 3	QEN71_01455	paras_000843	protein-codi	NZ_CP1252	chromosom	326747	327010	+	260	0	17	9222	12	5039	
QEN71_RS01460	protein-export chaperone SecB	QEN71_01460	paras_000844	protein-codi	NZ_CP1252	chromosom	327205	327675	+	471	0	9	1901	7	1302	
QEN71_RS01465	NAD(P)H-dependent glycerol-3-phosphate dehydro	QEN71_01465	paras_000845	protein-codi	NZ_CP1252	chromosom	327697	328695	+	999	0	20	5669	17	4995	
QEN71_RS01470	O-acetyl-ADP-ribose deacetylase	QEN71_01470	paras_000846	protein-codi	NZ_CP1252	chromosom	328851	329402	+	524	0	9	1734	5	231	
QEN71_RS01475	tRNA (uridine(34)/cytosine(34)/5- carboxymethylam	QEN71_01475	paras_000847	protein-codi	NZ_CP1252	chromosom	329375	329845	-	443	0	8	3012	6	1545	
QEN71_RS01480	ComF family protein	QEN71_01480	paras_000848	protein-codi	NZ_CP1252	chromosom	329863	330606	-	744	0	8	407	6	320	
QEN71_RS01485	methyltransferase domain-containing protein	QEN71_01485	paras_000849	protein-codi	NZ_CP1252	chromosom	330762	331727	+	966	0	14	1921	10	89	
QEN71_RS01490	DUF2244 domain-containing protein	QEN71_01490	paras_000850	protein-codi	NZ_CP1252	chromosom	331995	332489	+	495	0	13	7156	11	7027	
QEN71_RS01495	cytochrome c oxidase subunit II	QEN71_01495	paras_000851	protein-codi	NZ_CP1252	chromosom	332546	334255	+	1710	0	50	17646	38	13632	
QEN71_RS01500	cytochrome c oxidase subunit I	QEN71_01500	paras_000852	protein-codi	NZ_CP1252	chromosom	334300	335913	+	1614	0	52	22260	36	16427	
QEN71_RS01505	cytochrome oxidase small assembly protein	QEN71_01505	paras_000853	protein-codi	NZ_CP1252	chromosom	336005	336142	+	138	0	2	1132	2	1132	
QEN71_RS01510	cytochrome c oxidase assembly protein	QEN71_01510	paras_000854	protein-codi	NZ_CP1252	chromosom	336195	336809	+	615	0	15	3900	14	3891	
QEN71_RS01515	DUF2970 domain-containing protein	QEN71_01515	paras_000855	protein-codi	NZ_CP1252	chromosom	336848	337057	+	210	0	2	282	0	0	
QEN71_RS01520	cytochrome c oxidase subunit 3	QEN71_01520	paras_000856	protein-codi	NZ_CP1252	chromosom	337203	338060	+	858	0	33	10303	18	6726	
QEN71_RS01525	twin transmembrane helix small protein	QEN71_01525	paras_000857	protein-codi	NZ_CP1252	chromosom	338312	338521	-	210	0	6	4559	6	4559	
QEN71_RS01530	SURF1 family protein	QEN71_01530	paras_000858	protein-codi	NZ_CP1252	chromosom	338621	339385	+	765	0	41	13535	36	13047	
QEN71_RS01535	cytochrome C oxidase subunit I	QEN71_01535	paras_000859	protein-codi	NZ_CP1252	chromosom	339456	340145	+	690	0	23	13671	22	13644	
QEN71_RS01540	COX15/CtaA family protein	QEN71_01540	paras_000860	protein-codi	NZ_CP1252	chromosom	340197	341306	+	1110	0	28	8365	15	4177	
QEN71_RS01545	heme o synthase	QEN71_01545	paras_000861	protein-codi	NZ_CP1252	chromosom	341313	342218	+	906	0	40	3835	28	2503	
QEN71_RS01550	SCO family protein	QEN71_01550	paras_000862	protein-codi	NZ_CP1252	chromosom	342223	342840	+	618	0	24	9280	24	9280	
QEN71_RS01555	YciI family protein	QEN71_01555	paras_000863	protein-codi	NZ_CP1252	chromosom	342939	343229	-	291	0	9	7944	2	154	
QEN71_RS01560	methyl-accepting chemotaxis protein	QEN71_01560	paras_000864	protein-codi	NZ_CP1252	chromosom	343458	345236	+	1779	0	33	14723	27	13314	
QEN71_RS01565	FkbM family methyltransferase	QEN71_01565	paras_000865	protein-codi	NZ_CP1252	chromosom	345244	346062	-	819	0	29	15069	22	12082	
QEN71_RS01570	MetQ/NlpA family lipoprotein	QEN71_01570	paras_000866	protein-codi	NZ_CP1252	chromosom	346396	347208	+	813	0	20	7873	19	7646	
QEN71_RS01575	bifunctional helix-turn-helix transcriptional regulator	QEN71_01575	paras_000867	protein-codi	NZ_CP1252	chromosom	347294	348217	-	924	0	18	8709	12	6817	
QEN71_RS01580	GntR family transcriptional regulator	QEN71_01580	paras_000868	protein-codi	NZ_CP1252	chromosom	348594	349328	+	735	0	18	5949	8	2689	
QEN71_RS01585	N-acetylglucosamine-6-phosphate deacetylase	QEN71_01585	paras_000869	protein-codi	NZ_CP1252	chromosom	349409	350512	+	1096	0	28	8238	22	5579	
QEN71_RS01590	SIS domain-containing protein	QEN71_01590	paras_000870	protein-codi	NZ_CP1252	chromosom	350505	351512	+	1000	0	16	6249	12	4240	
QEN71_RS01595	phosphoenolpyruvate--protein phosphotransferase	QEN71_01595	paras_000871	protein-codi	NZ_CP1252	chromosom	351526	354114	+	2589	0	27	5646	17	4295	
QEN71_RS01600	N-acetylglucosamine-specific PTS transporter subu	QEN71_01600	paras_000872	protein-codi	NZ_CP1252	chromosom	354223	355980	+	1758	0	41	12216	36	11180	
QEN71_RS01605	cytochrome bd-I oxidase subunit CydX	QEN71_01605	paras_000873	protein-codi	NZ_CP1252	chromosom	356049	356168	-	120	0	2	695	0	0	
QEN71_RS01610	cytochrome d ubiquinol oxidase subunit II	QEN71_01610	paras_000874	protein-codi	NZ_CP1252	chromosom	356195	357331	-	1137	0	23	4619	15	2985	
QEN71_RS01615	cytochrome ubiquinol oxidase subunit I	QEN71_01615	paras_000875	protein-codi	NZ_CP1252	chromosom	357357	358946	-	1579	0	42	8496	26	4757	

QEN71_RS01620	hypothetical protein	QEN71_01620	paras_000876	protein-codi	NZ_CP1252	chromosom	358936	359208	-	262	0	0	0	0	0	
QEN71_RS01625	RNA polymerase sigma factor RpoH	QEN71_01625	paras_000877	protein-codi	NZ_CP1252	chromosom	359524	360459	-	936	0	6	894	1	6	
QEN71_RS01630	acyloxyacyl hydrolase	QEN71_01630	paras_000878	protein-codi	NZ_CP1252	chromosom	360824	361387	+	564	0	15	8279	8	4405	
QEN71_RS01635	nuclear transport factor 2 family protein	QEN71_01635	paras_000879	protein-codi	NZ_CP1252	chromosom	361433	361816	+	384	0	4	2426	4	2426	
QEN71_RS01640	2-isopropylmalate synthase	QEN71_01640	paras_000880	protein-codi	NZ_CP1252	chromosom	361932	363590	-	1659	0	35	13509	30	11659	
QEN71_RS01645	DNA-binding protein YbiB	QEN71_01645	paras_000881	protein-codi	NZ_CP1252	chromosom	364110	365078	-	969	0	14	7456	10	6920	
QEN71_RS01650	fumarylacetoacetate hydrolase family protein	QEN71_01650	paras_000882	protein-codi	NZ_CP1252	chromosom	365333	366031	+	699	0	21	11164	17	8199	
QEN71_RS01655	maleylacetoacetate isomerase	QEN71_01655	paras_000883	protein-codi	NZ_CP1252	chromosom	366066	366710	+	645	0	32	18063	22	11977	
QEN71_RS01660	signal recognition particle-docking protein FtsY	QEN71_01660	paras_000884	protein-codi	NZ_CP1252	chromosom	366820	367989	-	1170	0	0	0	0	0	TRUE
QEN71_RS01665	16S rRNA (guanine(966)-N(2))-methyltransferase R	QEN71_01665	paras_000885	protein-codi	NZ_CP1252	chromosom	368296	368910	+	615	0	10	2692	4	1255	
QEN71_RS01670	pantetheine-phosphate adenylyltransferase	QEN71_01670	paras_000886	protein-codi	NZ_CP1252	chromosom	369028	369534	+	507	0	1	86	0	0	TRUE
QEN71_RS01675	YfhL family 4Fe-4S dicluster ferredoxin	QEN71_01675	paras_000887	protein-codi	NZ_CP1252	chromosom	369683	369940	+	258	0	8	2792	8	2792	
QEN71_RS01680	histidinol-phosphate transaminase	QEN71_01680	paras_000888	protein-codi	NZ_CP1252	chromosom	370012	371091	-	1080	0	47	19381	37	15976	
QEN71_RS01685	aminoacyl-tRNA hydrolase	QEN71_01685	paras_000889	protein-codi	NZ_CP1252	chromosom	371125	371727	-	603	0	1	16	1	16	TRUE
QEN71_RS01690	50S ribosomal protein L25/general stress protein Ct	QEN71_01690	paras_000890	protein-codi	NZ_CP1252	chromosom	371848	372459	-	612	0	5	817	2	92	
QEN71_RS01695	ribose-phosphate pyrophosphokinase	QEN71_01695	paras_000891	protein-codi	NZ_CP1252	chromosom	372615	373571	-	957	0	1	342	0	0	TRUE
QEN71_RS01700	tRNA-Gln	QEN71_01700		tRNA	NZ_CP1252	chromosom	373619	373695	-	77	0	0	0	0	0	
QEN71_RS01705	4-(cytidine 5'-diphospho)-2-C-methyl-D-erythritol kir	QEN71_01705	paras_000893	protein-codi	NZ_CP1252	chromosom	373747	374628	-	882	0	1	102	0	0	TRUE
QEN71_RS01710	lipoprotein insertase outer membrane protein LolB	QEN71_01710	paras_000894	protein-codi	NZ_CP1252	chromosom	374654	375286	-	632	0	1	48	0	0	TRUE
QEN71_RS01715	tetratricopeptide repeat protein	QEN71_01715	paras_000895	protein-codi	NZ_CP1252	chromosom	375286	377127	-	1841	0	33	6696	26	5655	
QEN71_RS01720	bifunctional DNA-formamidopyrimidine glycosylase/	QEN71_01720	paras_000896	protein-codi	NZ_CP1252	chromosom	377215	378045	+	831	0	38	18521	27	7831	
QEN71_RS01725	A/G-specific adenine glycosylase	QEN71_01725	paras_000897	protein-codi	NZ_CP1252	chromosom	378053	379150	+	1098	0	23	8925	22	8742	
QEN71_RS01730	LON peptidase substrate-binding domain-containing	QEN71_01730	paras_000898	protein-codi	NZ_CP1252	chromosom	379160	379795	-	636	0	14	7854	6	3170	
QEN71_RS01735	RNase adapter RapZ	QEN71_01735	paras_000899	protein-codi	NZ_CP1252	chromosom	379854	380747	-	894	0	40	13512	26	9226	
QEN71_RS01740	HPr(Ser) kinase/phosphatase	QEN71_01740	paras_000900	protein-codi	NZ_CP1252	chromosom	380825	381793	-	969	0	6	35	5	32	
QEN71_RS01745	PTS sugar transporter subunit IIA	QEN71_01745	paras_000901	protein-codi	NZ_CP1252	chromosom	381942	382457	-	516	0	9	836	9	836	
QEN71_RS01750	ribosome-associated translation inhibitor RaiA	QEN71_01750	paras_000902	protein-codi	NZ_CP1252	chromosom	382751	383110	-	360	0	19	10980	14	8940	
QEN71_RS01755	RNA polymerase factor sigma-54	QEN71_01755	paras_000903	protein-codi	NZ_CP1252	chromosom	383285	384802	-	1518	0	44	15235	35	12906	
QEN71_RS01760	LPS export ABC transporter ATP-binding protein	QEN71_01760	paras_000904	protein-codi	NZ_CP1252	chromosom	385032	385814	-	779	0	5	2374	2	4	
QEN71_RS01765	lipopolysaccharide transport periplasmic protein Lpt	QEN71_01765	paras_000905	protein-codi	NZ_CP1252	chromosom	385811	386488	-	674	0	0	0	0	0	TRUE
QEN71_RS01770	LPS export ABC transporter periplasmic protein Lpt	QEN71_01770	paras_000906	protein-codi	NZ_CP1252	chromosom	386521	387114	-	590	0	1	139	0	0	TRUE
QEN71_RS01775	HAD family hydrolase	QEN71_01775	paras_000907	protein-codi	NZ_CP1252	chromosom	387111	387674	-	559	0	3	39	3	39	
QEN71_RS01780	KpsF/GutQ family sugar-phosphate isomerase	QEN71_01780	paras_000908	protein-codi	NZ_CP1252	chromosom	387674	388657	-	983	0	4	2013	3	2008	
QEN71_RS01785	cation:proton antiporter	QEN71_01785	paras_000909	protein-codi	NZ_CP1252	chromosom	388806	390809	+	2004	0	34	15406	30	14435	
QEN71_RS01790	adenine phosphoribosyltransferase	QEN71_01790	paras_000910	protein-codi	NZ_CP1252	chromosom	390934	391500	+	567	0	17	7197	14	5865	
QEN71_RS01795	LysE family translocator	QEN71_01795	paras_000911	protein-codi	NZ_CP1252	chromosom	391526	392140	+	615	0	3	1989	3	1989	
QEN71_RS01800	DUF4743 domain-containing protein	QEN71_01800	paras_000912	protein-codi	NZ_CP1252	chromosom	392182	393030	+	849	0	16	5508	16	5508	
QEN71_RS01805	formyltetrahydrofolate deformylase	QEN71_01805	paras_000913	protein-codi	NZ_CP1252	chromosom	393101	393964	+	864	0	23	8236	18	7300	
QEN71_RS01810	PepSY-associated TM helix domain-containing prot	QEN71_01810	paras_000914	protein-codi	NZ_CP1252	chromosom	394035	395273	-	1239	0	20	5691	19	5685	
QEN71_RS01815	TonB-dependent receptor	QEN71_01815	paras_000915	protein-codi	NZ_CP1252	chromosom	395628	397784	+	2157	0	95	30856	77	25056	
QEN71_RS01820	AI-2E family transporter	QEN71_01820	paras_000916	protein-codi	NZ_CP1252	chromosom	397873	398958	-	1086	0	16	6007	7	2861	
QEN71_RS01825	excinuclease ABC subunit UvrA	QEN71_01825	paras_000917	protein-codi	NZ_CP1252	chromosom	398980	402078	-	3099	0	119	46200	104	43566	
QEN71_RS01830	MFS transporter	QEN71_01830	paras_000918	protein-codi	NZ_CP1252	chromosom	402362	403549	+	1188	0	29	8547	24	6903	
QEN71_RS01835	single-stranded DNA-binding protein	QEN71_01835	paras_000919	protein-codi	NZ_CP1252	chromosom	403677	404210	+	534	0	3	953	1	5	
QEN71_RS01840	SDR family oxidoreductase	QEN71_01840	paras_000920	protein-codi	NZ_CP1252	chromosom	404347	405090	-	744	0	7	1562	7	1562	
QEN71_RS01845	LysR family transcriptional regulator	QEN71_01845	paras_000921	protein-codi	NZ_CP1252	chromosom	405227	406132	+	906	0	29	17304	29	17304	
QEN71_RS01850	hypothetical protein	QEN71_01850	paras_000922	protein-codi	NZ_CP1252	chromosom	406149	406952	-	804	0	29	12394	22	10089	
QEN71_RS01855	methyl-accepting chemotaxis protein	QEN71_01855	paras_000923	protein-codi	NZ_CP1252	chromosom	407296	408945	+	1650	0	25	10458	17	5818	
QEN71_RS01860	c-type cytochrome	QEN71_01860	paras_000924	protein-codi	NZ_CP1252	chromosom	409033	409842	-	809	0	19	5478	15	4641	
QEN71_RS01865	b(o/a)3-type cytochrome-c oxidase subunit 1	QEN71_01865	paras_000925	protein-codi	NZ_CP1252	chromosom	409842	411464	-	1608	0	49	12821	44	10682	
QEN71_RS01870	cytochrome c oxidase subunit II	QEN71_01870	paras_000926	protein-codi	NZ_CP1252	chromosom	411451	412023	-	559	0	8	1346	8	1346	
QEN71_RS01875	hypothetical protein	QEN71_01875	paras_000927	protein-codi	NZ_CP1252	chromosom	412026	412208	-	183	0	5	763	3	473	
QEN71_RS01880	cytochrome c	QEN71_01880	paras_000928	protein-codi	NZ_CP1252	chromosom	412249	413505	-	1257	0	39	12903	31	9642	
QEN71_RS01885	c-type cytochrome	QEN71_01885	paras_000929	protein-codi	NZ_CP1252	chromosom	413610	414332	-	723	0	19	4504	17	3949	
QEN71_RS01890	LysR family transcriptional regulator	QEN71_01890	paras_000930	protein-codi	NZ_CP1252	chromosom	414728	415621	-	894	0	8	1391	8	1391	
QEN71_RS01895	MFS transporter	QEN71_01895	paras_000931	protein-codi	NZ_CP1252	chromosom	415720	417012	+	1293	0	13	3273	13	3273	
QEN71_RS01900	response regulator transcription factor	QEN71_01900	paras_000932	protein-codi	NZ_CP1252	chromosom	417666	418319	+	654	0	9	1359	7	1308	
QEN71_RS01905	response regulator transcription factor	QEN71_01905	paras_000933	protein-codi	NZ_CP1252	chromosom	418434	419138	+	705	0	19	2938	18	2886	
QEN71_RS01910	response regulator transcription factor	QEN71_01910	paras_000934	protein-codi	NZ_CP1252	chromosom	419224	419865	+	642	0	17	5531	15	5229	
QEN71_RS01915	aquaporin	QEN71_01915	paras_000935	protein-codi	NZ_CP1252	chromosom	420050	420793	+	744	0	17	3218	16	3011	
QEN71_RS01920	transporter substrate-binding domain-containing pro	QEN71_01920	paras_000936	protein-codi	NZ_CP1252	chromosom	420802	425259	+	4458	0	75	13005	56	8488	
QEN71_RS01925	CHRD domain-containing protein	QEN71_01925	paras_000937	protein-codi	NZ_CP1252	chromosom	425435	425875	+	441	0	13	3406	11	3131	

QEN71_RS01930	alpha/beta hydrolase	QEN71_01930	paras_000938	protein-codi	NZ_CP1252	chromosom	426045	426995	+	920	0	18	2824	18	2824	
QEN71_RS01935	EcsC family protein	QEN71_01935	paras_000939	protein-codi	NZ_CP1252	chromosom	426965	427807	-	812	0	9	1252	6	950	
QEN71_RS01940	MFS transporter	QEN71_01940	paras_000940	protein-codi	NZ_CP1252	chromosom	427946	429541	+	1596	0	42	14839	31	12650	
QEN71_RS01945	MarR family transcriptional regulator	QEN71_01945	paras_000941	protein-codi	NZ_CP1252	chromosom	429572	430042	+	471	0	3	92	3	92	
QEN71_RS01950	amino acid deaminase	QEN71_01950	paras_000942	protein-codi	NZ_CP1252	chromosom	430112	431389	-	1278	0	31	10975	25	9996	
QEN71_RS01955	MurR/RpiR family transcriptional regulator	QEN71_01955	paras_000943	protein-codi	NZ_CP1252	chromosom	431567	432460	+	894	0	18	5908	16	5557	
QEN71_RS01960	D-aminoacylase	QEN71_01960	paras_000944	protein-codi	NZ_CP1252	chromosom	432464	433951	+	1488	0	28	6806	23	6369	
QEN71_RS01965	RidA family protein	QEN71_01965	paras_000945	protein-codi	NZ_CP1252	chromosom	434007	434393	+	387	0	19	4704	14	3439	
QEN71_RS01970	IS481 family transposase	QEN71_01970	paras_000946	protein-codi	NZ_CP1252	chromosom	434918	436057	-	1140	1793.0	2	264	2	264	
QEN71_RS01975	NADPH-dependent 7-cyano-7-deazaguanine reduct	QEN71_01975	paras_000947	protein-codi	NZ_CP1252	chromosom	436260	437084	-	821	0	38	6118	26	4891	
QEN71_RS01980	5'-nucleotidase	QEN71_01980	paras_000948	protein-codi	NZ_CP1252	chromosom	437081	437983	-	899	0	17	1894	14	1276	
QEN71_RS01985	threonine ammonia-lyase, biosynthetic	QEN71_01985	paras_000949	protein-codi	NZ_CP1252	chromosom	437993	439516	-	1524	0	50	12991	32	10596	
QEN71_RS01990	FAD/FMN-binding oxidoreductase	QEN71_01990	paras_000950	protein-codi	NZ_CP1252	chromosom	440602	444735	+	4134	0	81	32808	59	20537	
QEN71_RS01995	HIT family protein	QEN71_01995	paras_000951	protein-codi	NZ_CP1252	chromosom	444768	445196	+	429	0	7	3808	4	910	
QEN71_RS02000	DUF971 domain-containing protein	QEN71_02000	paras_000952	protein-codi	NZ_CP1252	chromosom	445204	445617	+	414	0	14	7442	14	7442	
QEN71_RS02005	bifunctional demethylmenaquinone methyltransferas	QEN71_02005	paras_000953	protein-codi	NZ_CP1252	chromosom	445688	446419	+	732	0	6	967	1	3	
QEN71_RS02010	TIM44-like domain-containing protein	QEN71_02010	paras_000954	protein-codi	NZ_CP1252	chromosom	446457	447452	+	996	0	25	2591	20	2194	
QEN71_RS02015	SCP2 sterol-binding domain-containing protein	QEN71_02015	paras_000955	protein-codi	NZ_CP1252	chromosom	447561	448205	+	645	0	2	164	1	10	TRUE
QEN71_RS02020	ubiquinone biosynthesis regulatory protein kinase U	QEN71_02020	paras_000956	protein-codi	NZ_CP1252	chromosom	448221	449804	+	1584	0	6	78	2	25	TRUE
QEN71_RS02025	thiopurine S-methyltransferase	QEN71_02025	paras_000957	protein-codi	NZ_CP1252	chromosom	449827	450462	+	636	0	8	4774	7	3951	
QEN71_RS02030	zinc ribbon domain-containing protein	QEN71_02030	paras_000958	protein-codi	NZ_CP1252	chromosom	450601	450954	+	354	0	6	3230	1	746	
QEN71_RS02035	DUF502 domain-containing protein	QEN71_02035	paras_000959	protein-codi	NZ_CP1252	chromosom	451022	451678	+	657	0	9	2951	7	2406	
QEN71_RS02040	aspartate--tRNA ligase	QEN71_02040	paras_000960	protein-codi	NZ_CP1252	chromosom	451751	453550	+	1800	0	4	889	0	0	TRUE
QEN71_RS02045	dihydroneopterin triphosphate diphosphatase	QEN71_02045	paras_000961	protein-codi	NZ_CP1252	chromosom	453722	454198	+	473	0	9	2892	9	2892	
QEN71_RS02050	cardiolipin synthase ClsB	QEN71_02050	paras_000962	protein-codi	NZ_CP1252	chromosom	454195	455472	+	1274	0	42	13659	26	9238	
QEN71_RS02055	TetR/AcrR family transcriptional regulator	QEN71_02055	paras_000963	protein-codi	NZ_CP1252	chromosom	455712	456317	+	606	0	23	10492	14	7822	
QEN71_RS02060	acyl-CoA dehydrogenase C-terminal domain-contai	QEN71_02060	paras_000964	protein-codi	NZ_CP1252	chromosom	456412	458199	+	1788	0	52	15930	40	12638	
QEN71_RS02065	3-hydroxyacyl-CoA dehydrogenase/enoyl-CoA hydr	QEN71_02065	paras_000965	protein-codi	NZ_CP1252	chromosom	458325	460760	+	2436	0	46	20219	36	15929	
QEN71_RS02070	acetyl-CoA C-acyltransferase	QEN71_02070	paras_000966	protein-codi	NZ_CP1252	chromosom	460772	461971	+	1200	0	26	8482	17	3104	
QEN71_RS02075	enoyl-CoA hydratase	QEN71_02075	paras_000967	protein-codi	NZ_CP1252	chromosom	462604	463368	+	765	0	16	2907	9	1928	
QEN71_RS02080	formate dehydrogenase accessory sulfurtransferase	QEN71_02080	paras_000968	protein-codi	NZ_CP1252	chromosom	463399	464220	-	822	0	8	1352	5	957	
QEN71_RS02085	nitrate reductase associated protein	QEN71_02085	paras_000969	protein-codi	NZ_CP1252	chromosom	464341	464817	+	477	0	6	1622	4	1417	
QEN71_RS02090	EAL domain-containing protein	QEN71_02090	paras_000970	protein-codi	NZ_CP1252	chromosom	465002	468124	+	3123	0	74	27491	57	20348	
QEN71_RS02095	acyl-CoA thioesterase	QEN71_02095	paras_000971	protein-codi	NZ_CP1252	chromosom	468167	468562	-	396	0	9	1087	7	759	
QEN71_RS02100	ABC transporter ATP-binding protein/permease	QEN71_02100	paras_000972	protein-codi	NZ_CP1252	chromosom	468804	470672	+	1869	0	68	19160	56	16056	
QEN71_RS02105	nucleotidyltransferase family protein	QEN71_02105	paras_000973	protein-codi	NZ_CP1252	chromosom	471262	471951	-	690	0	29	4904	24	3616	
QEN71_RS02110	phosphotransferase	QEN71_02110	paras_000974	protein-codi	NZ_CP1252	chromosom	471975	473051	-	1077	0	24	11933	19	10095	
QEN71_RS02115	LPS-assembly protein LptD	QEN71_02115	paras_000975	protein-codi	NZ_CP1252	chromosom	473278	475644	+	2367	0	5	17	3	11	TRUE
QEN71_RS02120	peptidylprolyl isomerase	QEN71_02120	paras_000976	protein-codi	NZ_CP1252	chromosom	475785	477188	+	1404	0	17	2145	7	200	
QEN71_RS02125	4-hydroxythreonine-4-phosphate dehydrogenase Pd	QEN71_02125	paras_000977	protein-codi	NZ_CP1252	chromosom	477203	478204	+	1002	0	11	3021	10	2987	
QEN71_RS02130	16S rRNA (adenine(1518)-N(6)/adenine(1519)-N(6)	QEN71_02130	paras_000978	protein-codi	NZ_CP1252	chromosom	478225	479055	+	831	0	11	2299	9	1570	
QEN71_RS02135	porin	QEN71_02135	paras_000979	protein-codi	NZ_CP1252	chromosom	479274	480401	+	1128	0	60	14501	53	12167	
QEN71_RS02140	lactoylglutathione lyase	QEN71_02140	paras_000980	protein-codi	NZ_CP1252	chromosom	480479	480865	+	387	0	21	5467	15	3709	
QEN71_RS02145	SprT family zinc-dependent metalloprotease	QEN71_02145	paras_000981	protein-codi	NZ_CP1252	chromosom	481474	482331	-	858	0	25	12068	21	6936	
QEN71_RS02150	lysophospholipid acyltransferase family protein	QEN71_02150	paras_000982	protein-codi	NZ_CP1252	chromosom	482392	483150	-	759	0	16	2510	13	2217	
QEN71_RS02155	D-glycero-beta-D-manno-heptose 1,7-bisphosphate	QEN71_02155	paras_000983	protein-codi	NZ_CP1252	chromosom	483188	483745	-	558	0	1	2	1	2	TRUE
QEN71_RS02160	glycine--tRNA ligase subunit beta	QEN71_02160	paras_000984	protein-codi	NZ_CP1252	chromosom	483756	485855	-	2100	0	0	0	0	0	TRUE
QEN71_RS02165	glycine--tRNA ligase subunit alpha	QEN71_02165	paras_000985	protein-codi	NZ_CP1252	chromosom	485871	486878	-	1008	0	0	0	0	0	TRUE
QEN71_RS02170	apolipoprotein N-acyltransferase	QEN71_02170	paras_000986	protein-codi	NZ_CP1252	chromosom	487106	488782	-	1677	0	2	53	0	0	TRUE
QEN71_RS02175	HlyC/CorC family transporter	QEN71_02175	paras_000987	protein-codi	NZ_CP1252	chromosom	488819	489715	-	897	0	16	2797	15	2786	
QEN71_RS02180	gamma-glutamylcyclotransferase	QEN71_02180	paras_000988	protein-codi	NZ_CP1252	chromosom	490507	491160	-	654	0	18	5620	16	5368	
QEN71_RS02185	rRNA maturation RNase YbeY	QEN71_02185	paras_000989	protein-codi	NZ_CP1252	chromosom	491243	491704	-	462	0	2	146	0	0	TRUE
QEN71_RS02190	PhoH family protein	QEN71_02190	paras_000990	protein-codi	NZ_CP1252	chromosom	491735	492829	-	1095	0	17	3191	15	2990	
QEN71_RS02195	tRNA (N6-isopentenyl adenosine(37)-C2)-methylthi	QEN71_02195	paras_000991	protein-codi	NZ_CP1252	chromosom	492848	494233	-	1386	0	26	6956	19	4439	
QEN71_RS02200	LysR family transcriptional regulator	QEN71_02200	paras_000992	protein-codi	NZ_CP1252	chromosom	494459	495484	-	1026	0	15	4018	13	3787	
QEN71_RS02205	extradiol dioxygenase	QEN71_02205	paras_000993	protein-codi	NZ_CP1252	chromosom	495598	495993	+	396	0	6	1517	6	1517	
QEN71_RS02210	MFS transporter	QEN71_02210	paras_000994	protein-codi	NZ_CP1252	chromosom	496026	497204	+	1179	0	8	2774	6	1530	
QEN71_RS02215	XRE family transcriptional regulator	QEN71_02215	paras_000995	protein-codi	NZ_CP1252	chromosom	497437	498165	+	729	0	6	868	6	868	
QEN71_RS02220	3,4-dihydroxy-2-butanone-4-phosphate synthase	QEN71_02220	paras_000996	protein-codi	NZ_CP1252	chromosom	498629	499363	+	735	0	28	8378	23	7578	
QEN71_RS02225	HAD family hydrolase	QEN71_02225	paras_000997	protein-codi	NZ_CP1252	chromosom	500030	500716	-	687	0	23	5160	20	4004	
QEN71_RS02230	glycerol kinase GlpK	QEN71_02230	paras_000998	protein-codi	NZ_CP1252	chromosom	500819	502318	-	1500	0	26	8918	18	5318	
QEN71_RS02235	glycerol-3-phosphate dehydrogenase	QEN71_02235	paras_000999	protein-codi	NZ_CP1252	chromosom	502456	503991	-	1536	0	15	1103	11	381	

QEN71_RS02240	hypothetical protein	QEN71_02240	paras_001000	protein-codi	NZ_CP1252	chromosom	504206	504595	+	390	0	11	3700	11	3700	
QEN71_RS02245	hypothetical protein	QEN71_02245	paras_001001	protein-codi	NZ_CP1252	chromosom	504781	504978	-	198	0	4	640	3	482	
QEN71_RS02250	DeoR/GlpR family DNA-binding transcription regula	QEN71_02250	paras_001002	protein-codi	NZ_CP1252	chromosom	505360	506139	-	780	0	6	2047	3	557	
QEN71_RS02255	ferritin-like domain-containing protein	QEN71_02255	paras_001003	protein-codi	NZ_CP1252	chromosom	506553	507380	+	828	0	26	6609	23	6193	
QEN71_RS02260	hypothetical protein	QEN71_02260	paras_001004	protein-codi	NZ_CP1252	chromosom	507459	507980	+	522	0	24	8030	20	5861	
QEN71_RS02265	DEAD/DEAH box helicase	QEN71_02265	paras_001005	protein-codi	NZ_CP1252	chromosom	508033	509586	-	1554	0	19	6693	15	5919	
QEN71_RS02270	hemolysin III family protein	QEN71_02270	paras_001006	protein-codi	NZ_CP1252	chromosom	509786	510406	-	621	0	37	19193	32	16881	
QEN71_RS02275	cytochrome c	QEN71_02275	paras_001007	protein-codi	NZ_CP1252	chromosom	510579	511877	-	1299	0	52	21585	42	17755	
QEN71_RS02280	c-type cytochrome	QEN71_02280	paras_001008	protein-codi	NZ_CP1252	chromosom	511898	512629	-	732	0	34	17138	29	15539	
QEN71_RS02285	copper homeostasis periplasmic binding protein Co	QEN71_02285	paras_001009	protein-codi	NZ_CP1252	chromosom	513080	513466	+	383	0	21	10114	10	4429	
QEN71_RS02290	CopD family protein	QEN71_02290	paras_001010	protein-codi	NZ_CP1252	chromosom	513463	514392	+	926	0	6	1580	2	354	
QEN71_RS02295	galactonate dehydratase	QEN71_02295	paras_001011	protein-codi	NZ_CP1252	chromosom	514652	515800	-	1149	0	29	12220	27	10537	
QEN71_RS02300	FadR/GntR family transcriptional regulator	QEN71_02300	paras_001012	protein-codi	NZ_CP1252	chromosom	516073	516750	+	678	0	9	2861	7	2415	
QEN71_RS02305	DHA2 family efflux MFS transporter permease subu	QEN71_02305	paras_001013	protein-codi	NZ_CP1252	chromosom	516774	518312	-	1539	0	28	13182	24	11956	
QEN71_RS02310	MarR family transcriptional regulator	QEN71_02310	paras_001014	protein-codi	NZ_CP1252	chromosom	518459	518902	+	444	0	4	2038	4	2038	
QEN71_RS02315	tRNA-Met	QEN71_02315		tRNA	NZ_CP1252	chromosom	519019	519095	-	77	0	0	0	0	0	
QEN71_RS02320	MFS transporter	QEN71_02320	paras_001016	protein-codi	NZ_CP1252	chromosom	519182	520840	-	1659	0	46	17917	29	10252	
QEN71_RS02325	sensor histidine kinase	QEN71_02325	paras_001017	protein-codi	NZ_CP1252	chromosom	521069	522616	-	1548	0	38	14218	28	11638	
QEN71_RS02330	response regulator transcription factor	QEN71_02330	paras_001018	protein-codi	NZ_CP1252	chromosom	522625	523374	-	750	0	25	9390	16	5721	
QEN71_RS02335	recombinase RecA	QEN71_02335	paras_001019	protein-codi	NZ_CP1252	chromosom	523611	524687	+	1077	0	20	1706	16	997	
QEN71_RS02340	recombination regulator RecX	QEN71_02340	paras_001020	protein-codi	NZ_CP1252	chromosom	524695	525519	+	825	0	23	5736	18	3930	
QEN71_RS02345	DUF2889 domain-containing protein	QEN71_02345	paras_001021	protein-codi	NZ_CP1252	chromosom	525692	526423	+	732	0	7	1368	4	179	
QEN71_RS02350	ADP-forming succinate--CoA ligase subunit beta	QEN71_02350	paras_001022	protein-codi	NZ_CP1252	chromosom	526479	527648	+	1170	101.0	15	4479	12	4302	
QEN71_RS02355	succinate--CoA ligase subunit alpha	QEN71_02355	paras_001023	protein-codi	NZ_CP1252	chromosom	527765	528646	+	882	0	17	5282	15	4658	
QEN71_RS02360	TerC family protein	QEN71_02360	paras_001024	protein-codi	NZ_CP1252	chromosom	528807	529532	+	726	0	9	967	4	226	
QEN71_RS02365	pilin	QEN71_02365	paras_001025	protein-codi	NZ_CP1252	chromosom	529683	530300	+	618	0	19	4556	15	3355	
QEN71_RS02370	Wzy polymerase domain-containing protein	QEN71_02370	paras_001026	protein-codi	NZ_CP1252	chromosom	530471	532261	+	1791	0	53	14763	37	10652	
QEN71_RS02375	cyclic pyranopterin monophosphate synthase MoaC	QEN71_02375	paras_001027	protein-codi	NZ_CP1252	chromosom	532355	532831	-	477	0	7	2790	4	1148	
QEN71_RS02380	M48 family metalloprotease	QEN71_02380	paras_001028	protein-codi	NZ_CP1252	chromosom	533050	534738	+	1685	0	43	12998	31	8779	
QEN71_RS02385	DUF2946 family protein	QEN71_02385	paras_001029	protein-codi	NZ_CP1252	chromosom	534735	535337	-	591	0	9	2870	9	2870	
QEN71_RS02390	hydrolase	QEN71_02390	paras_001030	protein-codi	NZ_CP1252	chromosom	535330	536418	-	1081	0	22	6811	21	6629	
QEN71_RS02395	nuclear transport factor 2 family protein	QEN71_02395	paras_001031	protein-codi	NZ_CP1252	chromosom	536496	536942	-	447	0	11	5308	11	5308	
QEN71_RS02400	lipopolysaccharide heptosyltransferase II	QEN71_02400	paras_001032	protein-codi	NZ_CP1252	chromosom	537118	538143	-	1026	0	14	589	12	234	
QEN71_RS02405	zinc-finger domain-containing protein	QEN71_02405	paras_001033	protein-codi	NZ_CP1252	chromosom	538355	538549	-	195	0	8	1566	7	1505	
QEN71_RS02410	branched-chain amino acid transaminase	QEN71_02410	paras_001034	protein-codi	NZ_CP1252	chromosom	538621	539544	-	924	0	30	11950	25	10111	
QEN71_RS02415	AzlC family ABC transporter permease	QEN71_02415	paras_001035	protein-codi	NZ_CP1252	chromosom	539842	540597	+	752	0	18	6934	16	6717	
QEN71_RS02420	AzlD domain-containing protein	QEN71_02420	paras_001036	protein-codi	NZ_CP1252	chromosom	540594	540917	+	320	0	5	980	5	980	
QEN71_RS02425	phosphoglycerate kinase	QEN71_02425	paras_001037	protein-codi	NZ_CP1252	chromosom	541133	542326	+	1194	0	13	855	4	116	
QEN71_RS02430	pyruvate kinase	QEN71_02430	paras_001038	protein-codi	NZ_CP1252	chromosom	542815	544251	+	1437	0	24	7049	22	7024	
QEN71_RS02435	fructose-bisphosphate aldolase class II	QEN71_02435	paras_001039	protein-codi	NZ_CP1252	chromosom	544493	545557	+	1065	0	10	152	5	38	
QEN71_RS02440	phosphoribosylaminoimidazolesuccinocarboxamide	QEN71_02440	paras_001040	protein-codi	NZ_CP1252	chromosom	545735	546625	+	891	0	14	1336	9	921	
QEN71_RS02445	5-(carboxyamino)imidazole ribonucleotide mutase	QEN71_02445	paras_001041	protein-codi	NZ_CP1252	chromosom	546650	547159	+	510	0	4	365	3	140	
QEN71_RS02450	5-(carboxyamino)imidazole ribonucleotide synthase	QEN71_02450	paras_001042	protein-codi	NZ_CP1252	chromosom	547233	548441	+	1209	0	11	726	8	504	
QEN71_RS02455	L-threonylcarbamoyladenylate synthase	QEN71_02455	paras_001043	protein-codi	NZ_CP1252	chromosom	548454	549497	+	1044	0	0	0	0	0	TRUE
QEN71_RS02460	SGNH/GDSL hydrolase family protein	QEN71_02460	paras_001044	protein-codi	NZ_CP1252	chromosom	549588	550787	-	1200	0	46	15835	35	9319	
QEN71_RS02465	sterol desaturase family protein	QEN71_02465	paras_001045	protein-codi	NZ_CP1252	chromosom	550942	551862	-	921	0	47	29024	43	27707	
QEN71_RS02470	D-alanyl-D-alanine carboxypeptidase/D-alanyl-D-ala	QEN71_02470	paras_001046	protein-codi	NZ_CP1252	chromosom	552061	553740	-	1680	0	35	17063	30	15594	
QEN71_RS02475	response regulator	QEN71_02475	paras_001047	protein-codi	NZ_CP1252	chromosom	554067	554729	+	663	0	23	10244	16	6886	
QEN71_RS02480	ATP-binding protein	QEN71_02480	paras_001048	protein-codi	NZ_CP1252	chromosom	554734	556035	+	1302	0	21	6978	15	5024	
QEN71_RS02485	DegQ family serine endoprotease	QEN71_02485	paras_001049	protein-codi	NZ_CP1252	chromosom	556149	557657	+	1509	0	22	9004	18	7927	
QEN71_RS02490	carboxypeptidase regulatory-like domain-containing	QEN71_02490	paras_001050	protein-codi	NZ_CP1252	chromosom	557902	558399	+	498	0	16	5793	12	4695	
QEN71_RS02495	DUF427 domain-containing protein	QEN71_02495	paras_001051	protein-codi	NZ_CP1252	chromosom	558797	559252	+	456	0	25	11709	25	11709	
QEN71_RS02500	SRPBCC domain-containing protein	QEN71_02500	paras_001052	protein-codi	NZ_CP1252	chromosom	559286	560032	+	747	0	15	4617	10	2510	
QEN71_RS02505	helix-turn-helix domain-containing protein	QEN71_02505	paras_001053	protein-codi	NZ_CP1252	chromosom	560062	561117	-	1056	0	18	11370	16	11114	
QEN71_RS02510	cysteine hydrolase family protein	QEN71_02510	paras_001054	protein-codi	NZ_CP1252	chromosom	561266	561895	+	630	0	22	9032	15	6154	
QEN71_RS02515	TetR family transcriptional regulator	QEN71_02515	paras_001055	protein-codi	NZ_CP1252	chromosom	561951	562586	-	636	0	8	1843	8	1843	
QEN71_RS02520	efflux RND transporter periplasmic adaptor subunit	QEN71_02520	paras_001056	protein-codi	NZ_CP1252	chromosom	563013	564263	+	1251	0	23	7553	18	7045	
QEN71_RS02525	efflux RND transporter permease subunit	QEN71_02525	paras_001057	protein-codi	NZ_CP1252	chromosom	564282	567476	+	3195	0	66	17638	55	15447	
QEN71_RS02530	efflux transporter outer membrane subunit	QEN71_02530	paras_001058	protein-codi	NZ_CP1252	chromosom	567487	569004	+	1518	0	38	8772	27	6036	
QEN71_RS02535	hypothetical protein	partial;pseudo;QEN71_02535		pseudogene	NZ_CP1252	chromosom	569219	569464	+	246	0	11	3411	11	3411	
QEN71_RS02540	DNA-binding protein	QEN71_02540	paras_001060	protein-codi	NZ_CP1252	chromosom	569566	570090	+	525	0	13	1070	7	562	
QEN71_RS02545	nucleobase:cation symporter-2 family protein	QEN71_02545	paras_001061	protein-codi	NZ_CP1252	chromosom	570164	571579	-	1416	0	18	9246	9	1695	

QEN71_RS02550	FGGY family carbohydrate kinase	QEN71_02550	paras_001062	protein-codi	NZ_CP1252	chromosom	571999	573423	+	1425	0	22	5430	14	3693	
QEN71_RS02555	S-(hydroxymethyl)glutathione dehydrogenase/class	QEN71_02555	paras_001063	protein-codi	NZ_CP1252	chromosom	573620	574726	+	1107	0	32	9594	23	6701	
QEN71_RS02560	S-formylglutathione hydrolase	QEN71_02560	paras_001064	protein-codi	NZ_CP1252	chromosom	574739	575584	+	846	0	22	7121	14	5251	
QEN71_RS02565	metal ABC transporter permease	QEN71_02565	paras_001065	protein-codi	NZ_CP1252	chromosom	575659	576450	-	784	0	15	2178	11	1418	
QEN71_RS02570	ABC transporter ATP-binding protein	QEN71_02570	paras_001066	protein-codi	NZ_CP1252	chromosom	576443	577384	-	930	0	10	4767	10	4767	
QEN71_RS02575	metal ABC transporter solute-binding protein	QEN71_02575	paras_001067	protein-codi	NZ_CP1252	chromosom	577381	578319	-	935	0	23	5093	23	5093	
QEN71_RS02580	Fur family transcriptional regulator	QEN71_02580	paras_001068	protein-codi	NZ_CP1252	chromosom	578366	578857	-	492	0	7	2046	7	2046	
QEN71_RS02585	L-iditol 2-dehydrogenase	QEN71_02585	paras_001069	protein-codi	NZ_CP1252	chromosom	579157	579939	+	783	0	15	4233	12	3857	
QEN71_RS02590	sugar ABC transporter substrate-binding protein	QEN71_02590	paras_001070	protein-codi	NZ_CP1252	chromosom	580028	581350	+	1323	0	45	16357	37	11587	
QEN71_RS02595	sugar ABC transporter permease	QEN71_02595	paras_001071	protein-codi	NZ_CP1252	chromosom	581616	582527	+	908	0	26	4622	24	4489	
QEN71_RS02600	carbohydrate ABC transporter permease	QEN71_02600	paras_001072	protein-codi	NZ_CP1252	chromosom	582524	583390	+	859	0	27	5289	20	4687	
QEN71_RS02605	HAD family phosphatase	QEN71_02605	paras_001073	protein-codi	NZ_CP1252	chromosom	583387	584079	+	689	0	14	3714	10	2029	
QEN71_RS02610	sn-glycerol-3-phosphate ABC transporter ATP-bindi	QEN71_02610	paras_001074	protein-codi	NZ_CP1252	chromosom	584143	585252	+	1110	0	16	3258	14	3176	
QEN71_RS02615	tetratricopeptide repeat protein	QEN71_02615	paras_001075	protein-codi	NZ_CP1252	chromosom	585505	587286	+	1776	0	53	13268	41	10886	
QEN71_RS02620	sugar-binding transcriptional regulator	QEN71_02620	paras_001076	protein-codi	NZ_CP1252	chromosom	587281	588252	-	966	0	11	2066	7	537	
QEN71_RS02625	xylulokinase	QEN71_02625	paras_001077	protein-codi	NZ_CP1252	chromosom	588375	589859	-	1485	0	23	4992	14	1777	
QEN71_RS02630	mannitol dehydrogenase family protein	QEN71_02630	paras_001078	protein-codi	NZ_CP1252	chromosom	590031	591437	-	1407	0	44	11222	37	10340	
QEN71_RS02635	AraC family transcriptional regulator	QEN71_02635	paras_001079	protein-codi	NZ_CP1252	chromosom	592031	593053	+	1023	0	18	4188	12	3604	
QEN71_RS02640	substrate-binding domain-containing protein	QEN71_02640	paras_001080	protein-codi	NZ_CP1252	chromosom	593143	594096	+	954	0	8	573	6	562	
QEN71_RS02645	sugar ABC transporter ATP-binding protein	QEN71_02645	paras_001081	protein-codi	NZ_CP1252	chromosom	594166	595713	+	1534	0	20	4346	17	3858	
QEN71_RS02650	ABC transporter permease	QEN71_02650	paras_001082	protein-codi	NZ_CP1252	chromosom	595700	596701	+	988	0	21	2410	15	1726	
QEN71_RS02655	alcohol dehydrogenase catalytic domain-containing	QEN71_02655	paras_001083	protein-codi	NZ_CP1252	chromosom	596746	597825	+	1080	0	21	3344	19	3025	
QEN71_RS02660	FGGY-family carbohydrate kinase	QEN71_02660	paras_001084	protein-codi	NZ_CP1252	chromosom	597894	599438	+	1541	0	51	9587	41	7381	
QEN71_RS02665	SDR family oxidoreductase	QEN71_02665	paras_001085	protein-codi	NZ_CP1252	chromosom	599435	600175	+	737	0	39	4221	30	3120	
QEN71_RS02670	tryptophan 2,3-dioxygenase	QEN71_02670	paras_001086	protein-codi	NZ_CP1252	chromosom	600791	601726	-	936	0	51	8537	36	4728	
QEN71_RS02675	kynureninase	QEN71_02675	paras_001087	protein-codi	NZ_CP1252	chromosom	601737	602987	-	1251	0	30	10301	22	8195	
QEN71_RS02680	arylformamidase	QEN71_02680	paras_001088	protein-codi	NZ_CP1252	chromosom	603014	603661	-	648	0	18	4917	18	4917	
QEN71_RS02685	Lrp/AsnC family transcriptional regulator	QEN71_02685	paras_001089	protein-codi	NZ_CP1252	chromosom	603794	604300	+	507	0	12	2927	10	2842	
QEN71_RS02690	flavin reductase family protein	QEN71_02690	paras_001090	protein-codi	NZ_CP1252	chromosom	604358	604870	-	513	0	10	3733	10	3733	
QEN71_RS02695	peptide-methionine (S)-S-oxide reductase MsrA	QEN71_02695	paras_001091	protein-codi	NZ_CP1252	chromosom	605177	605722	+	546	0	26	6836	26	6836	
QEN71_RS02700	DUF72 domain-containing protein	QEN71_02700	paras_001092	protein-codi	NZ_CP1252	chromosom	605786	606868	-	1083	0	22	4296	20	4220	
QEN71_RS02705	cyclopropane-fatty-acyl-phospholipid synthase fami	QEN71_02705	paras_001093	protein-codi	NZ_CP1252	chromosom	606990	608204	-	1215	0	47	10642	30	7174	
QEN71_RS02710	pyridoxamine 5'-phosphate oxidase	QEN71_02710	paras_001094	protein-codi	NZ_CP1252	chromosom	608381	609019	-	639	0	0	0	0	0	TRUE
QEN71_RS02715	tRNA cyclic N6-threonylcarbamoyladenosine(37) sy	QEN71_02715	paras_001095	protein-codi	NZ_CP1252	chromosom	609189	610049	+	861	0	22	6327	16	3990	
QEN71_RS02720	thioredoxin	QEN71_02720	paras_001096	protein-codi	NZ_CP1252	chromosom	610125	610973	-	849	0	17	3589	11	2105	
QEN71_RS02725	pirin family protein	QEN71_02725	paras_001097	protein-codi	NZ_CP1252	chromosom	611084	611965	-	882	0	9	2947	9	2947	
QEN71_RS02730	EamA family transporter	QEN71_02730	paras_001098	protein-codi	NZ_CP1252	chromosom	612134	613015	+	882	0	18	7686	16	7177	
QEN71_RS02735	N-acetylmuramoyl-L-alanine amidase	QEN71_02735	paras_001099	protein-codi	NZ_CP1252	chromosom	613662	615197	-	1517	0	8	633	4	22	TRUE
QEN71_RS02740	tRNA (adenosine(37)-N6)-threonylcarbamoyltransfe	QEN71_02740	paras_001100	protein-codi	NZ_CP1252	chromosom	615179	615733	-	536	0	2	7	2	7	
QEN71_RS02745	tRNA epoxyqueuosine(34) reductase QueG	QEN71_02745	paras_001101	protein-codi	NZ_CP1252	chromosom	615751	617043	+	1293	0	26	5597	24	5568	
QEN71_RS02750	methylated-DNA--[protein]-cysteine S-methyltransfe	QEN71_02750	paras_001102	protein-codi	NZ_CP1252	chromosom	617075	617545	+	467	0	15	2225	11	1947	
QEN71_RS02755	site-specific tyrosine recombinase XerD	QEN71_02755	paras_001103	protein-codi	NZ_CP1252	chromosom	617542	618477	+	932	0	0	0	0	0	TRUE
QEN71_RS02760	AMP-binding protein	QEN71_02760	paras_001104	protein-codi	NZ_CP1252	chromosom	618514	620097	-	1584	0	42	6176	38	5320	
QEN71_RS02765	Cys-tRNA(Pro) deacylase	QEN71_02765	paras_001105	protein-codi	NZ_CP1252	chromosom	620240	620731	+	492	0	19	5541	17	5053	
QEN71_RS02770	glycerol-3-phosphate 1-O-acyltransferase PlsY	QEN71_02770	paras_001106	protein-codi	NZ_CP1252	chromosom	620890	621519	+	630	0	1	6	1	6	TRUE
QEN71_RS02775	YajQ family cyclic di-GMP-binding protein	QEN71_02775	paras_001107	protein-codi	NZ_CP1252	chromosom	621582	622067	-	486	0	1	8	1	8	TRUE
QEN71_RS02780	UDP-N-acetylmuramate dehydrogenase	QEN71_02780	paras_001108	protein-codi	NZ_CP1252	chromosom	622240	623280	+	1041	0	2	11	1	5	TRUE
QEN71_RS02785	ornithine carbamoyltransferase	QEN71_02785	paras_001109	protein-codi	NZ_CP1252	chromosom	623379	624308	-	930	0	28	11168	20	8587	
QEN71_RS02790	DUF3579 domain-containing protein	QEN71_02790	paras_001110	protein-codi	NZ_CP1252	chromosom	624771	625106	+	336	0	0	0	0	0	TRUE
QEN71_RS02795	30S ribosomal protein S20	QEN71_02795	paras_001111	protein-codi	NZ_CP1252	chromosom	625308	625586	-	279	0	1	64	0	0	TRUE
QEN71_RS02800	murein biosynthesis integral membrane protein Mur	QEN71_02800	paras_001112	protein-codi	NZ_CP1252	chromosom	626073	627623	+	1551	0	7	1287	2	5	
QEN71_RS02805	SirB1 family protein	QEN71_02805	paras_001113	protein-codi	NZ_CP1252	chromosom	627629	628471	+	843	0	27	7594	25	7376	
QEN71_RS02810	3-hydroxyacyl-CoA dehydrogenase	QEN71_02810	paras_001114	protein-codi	NZ_CP1252	chromosom	628554	629312	-	759	0	6	1265	4	754	
QEN71_RS02815	adenylate kinase	QEN71_02815	paras_001115	protein-codi	NZ_CP1252	chromosom	629548	630213	-	666	0	4	277	1	3	TRUE
QEN71_RS02820	3-deoxy-manno-octulosonate cytidylyltransferase	QEN71_02820	paras_001116	protein-codi	NZ_CP1252	chromosom	630459	631277	-	819	0	5	714	1	2	
QEN71_RS02825	Trm112 family protein	QEN71_02825	paras_001117	protein-codi	NZ_CP1252	chromosom	631406	631609	-	184	0	1	11	1	11	
QEN71_RS02830	tetraacyldisaccharide 4'-kinase	QEN71_02830	paras_001118	protein-codi	NZ_CP1252	chromosom	631590	632606	-	997	0	1	11	1	11	TRUE
QEN71_RS02835	exodeoxyribonuclease VII large subunit	QEN71_02835	paras_001119	protein-codi	NZ_CP1252	chromosom	633199	634578	+	1380	0	19	4542	15	3655	
QEN71_RS02840	Fe-Mn family superoxide dismutase	QEN71_02840	paras_001120	protein-codi	NZ_CP1252	chromosom	634786	635364	+	579	0	1	6	1	6	TRUE
QEN71_RS02845	NYN domain-containing protein	QEN71_02845	paras_001121	protein-codi	NZ_CP1252	chromosom	635549	636856	-	1308	0	25	5968	21	5746	
QEN71_RS02850	isochorismatase family protein	QEN71_02850	paras_001122	protein-codi	NZ_CP1252	chromosom	636954	637553	-	600	0	4	488	4	488	
QEN71_RS02855	TetR/AcrR family transcriptional regulator	QEN71_02855	paras_001123	protein-codi	NZ_CP1252	chromosom	637602	638213	+	612	0	21	4604	13	2433	

QEN71_RS02860	ATP-dependent Clp endopeptidase proteolytic subu	QEN71_02860	paras_001124	protein-codi	NZ_CP1252	chromosom	638223	638876	-	654	0	27	7746	20	7141	
QEN71_RS02865	sigma-70 family RNA polymerase sigma factor	QEN71_02865	paras_001125	protein-codi	NZ_CP1252	chromosom	639037	639996	+	915	0	9	891	9	891	
QEN71_RS02870	EAL domain-containing protein	QEN71_02870	paras_001126	protein-codi	NZ_CP1252	chromosom	639952	641226	-	1230	0	10	1317	6	636	
QEN71_RS02875	multidrug transporter subunit MdtD	QEN71_02875	paras_001127	protein-codi	NZ_CP1252	chromosom	641509	642894	-	1386	0	26	9808	21	8952	
QEN71_RS02880	type II toxin-antitoxin system ParD family antitoxin	QEN71_02880	paras_001128	protein-codi	NZ_CP1252	chromosom	643120	643398	+	275	0	4	616	4	616	
QEN71_RS02885	type II toxin-antitoxin system RelE/ParE family toxin	QEN71_02885	paras_001129	protein-codi	NZ_CP1252	chromosom	643395	643712	+	314	0	17	3379	10	1366	
QEN71_RS02890	PLP-dependent aminotransferase family protein	QEN71_02890	paras_001130	protein-codi	NZ_CP1252	chromosom	643726	645138	-	1413	0	36	11521	29	9165	
QEN71_RS02895	DUF2917 domain-containing protein	QEN71_02895	paras_001131	protein-codi	NZ_CP1252	chromosom	645323	645634	+	312	0	5	274	5	274	
QEN71_RS02900	citrate synthase family protein	QEN71_02900	paras_001132	protein-codi	NZ_CP1252	chromosom	645668	646900	-	1233	0	26	5016	10	1243	
QEN71_RS02905	CoA transferase	QEN71_02905	paras_001133	protein-codi	NZ_CP1252	chromosom	647017	648411	+	1395	0	26	2850	19	2026	
QEN71_RS02910	GntR family transcriptional regulator	QEN71_02910	paras_001134	protein-codi	NZ_CP1252	chromosom	648422	649168	-	747	0	23	4374	11	1957	
QEN71_RS02915	aldo/keto reductase	QEN71_02915	paras_001135	protein-codi	NZ_CP1252	chromosom	649347	650192	+	846	0	10	1770	10	1770	
QEN71_RS02920	hypothetical protein	QEN71_02920	paras_001136	protein-codi	NZ_CP1252	chromosom	650226	650681	-	456	0	8	1707	5	327	
QEN71_RS02925	hypothetical protein	QEN71_02925	paras_001137	protein-codi	NZ_CP1252	chromosom	650945	651913	-	969	0	19	2943	11	1039	
QEN71_RS02930	ABC transporter ATP-binding protein	QEN71_02930	paras_001138	protein-codi	NZ_CP1252	chromosom	652412	654517	-	2102	0	54	9191	40	7229	
QEN71_RS02935	ABC transporter permease	QEN71_02935	paras_001139	protein-codi	NZ_CP1252	chromosom	654514	655449	-	928	0	27	4586	11	2670	
QEN71_RS02940	ABC transporter permease	QEN71_02940	paras_001140	protein-codi	NZ_CP1252	chromosom	655446	656456	-	1007	0	28	6101	21	4162	
QEN71_RS02945	ABC transporter substrate-binding protein	QEN71_02945	paras_001141	protein-codi	NZ_CP1252	chromosom	656488	658173	-	1686	0	34	6796	30	5719	
QEN71_RS02950	HoxN/HupN/NixA family nickel/cobalt transporter	QEN71_02950	paras_001142	protein-codi	NZ_CP1252	chromosom	658397	659413	-	1017	0	16	3835	14	3035	
QEN71_RS02955	cupin domain-containing protein	QEN71_02955	paras_001143	protein-codi	NZ_CP1252	chromosom	659425	659916	-	492	0	12	2244	8	1975	
QEN71_RS02960	elongation factor G	QEN71_02960	paras_001144	protein-codi	NZ_CP1252	chromosom	660610	662715	-	2106	0	50	9133	29	6392	
QEN71_RS02965	DUF192 domain-containing protein	QEN71_02965	paras_001145	protein-codi	NZ_CP1252	chromosom	662958	663473	-	516	0	7	1306	5	991	
QEN71_RS02970	hypothetical protein	QEN71_02970	paras_001146	protein-codi	NZ_CP1252	chromosom	663618	663812	-	195	0	3	321	0	0	
QEN71_RS02975	pseudouridine synthase	QEN71_02975	paras_001147	protein-codi	NZ_CP1252	chromosom	664241	664810	-	570	0	12	2371	8	2092	
QEN71_RS02980	NADP-dependent isocitrate dehydrogenase	QEN71_02980	paras_001148	protein-codi	NZ_CP1252	chromosom	665134	666390	+	1257	0	52	14859	41	12079	
QEN71_RS02985	hypothetical protein	QEN71_02985	paras_001149	protein-codi	NZ_CP1252	chromosom	666532	666873	+	342	0	2	75	2	75	
QEN71_RS02990	multicopper oxidase family protein	QEN71_02990	paras_001150	protein-codi	NZ_CP1252	chromosom	667003	668631	+	1629	0	23	7250	16	5140	
QEN71_RS02995	cold shock domain-containing protein CspD	QEN71_02995	paras_001151	protein-codi	NZ_CP1252	chromosom	668725	668931	-	207	0	5	1196	3	629	
QEN71_RS03000	ATP-dependent Clp protease adapter ClpS	QEN71_03000	paras_001152	protein-codi	NZ_CP1252	chromosom	669471	669785	+	311	0	11	3173	9	2900	
QEN71_RS03005	ATP-dependent Clp protease ATP-binding subunit (	QEN71_03005	paras_001153	protein-codi	NZ_CP1252	chromosom	669782	672082	+	2297	0	34	7872	29	6607	
QEN71_RS03010	dUTP diphosphatase	QEN71_03010	paras_001154	protein-codi	NZ_CP1252	chromosom	672191	672637	-	447	0	0	0	0	0	TRUE
QEN71_RS03015	LLM class flavin-dependent oxidoreductase	QEN71_03015	paras_001155	protein-codi	NZ_CP1252	chromosom	672702	673706	-	1005	0	25	5821	19	4170	
QEN71_RS03020	bifunctional phosphopantothenoylcysteine decarbox	QEN71_03020	paras_001156	protein-codi	NZ_CP1252	chromosom	673780	675003	-	1224	0	0	0	0	0	TRUE
QEN71_RS03025	signal peptidase II	QEN71_03025	paras_001157	protein-codi	NZ_CP1252	chromosom	675153	675653	-	500	0	1	5	1	5	TRUE
QEN71_RS03030	isoleucine--tRNA ligase	QEN71_03030	paras_001158	protein-codi	NZ_CP1252	chromosom	675653	678490	-	2837	0	2	116	1	19	TRUE
QEN71_RS03035	bifunctional riboflavin kinase/FAD synthetase	QEN71_03035	paras_001159	protein-codi	NZ_CP1252	chromosom	678680	679672	-	993	0	4	1019	0	0	
QEN71_RS03040	phosphoribosylglycinamide formyltransferase	QEN71_03040	paras_001160	protein-codi	NZ_CP1252	chromosom	679792	680457	+	662	0	10	5892	7	5261	
QEN71_RS03045	RsmB/NOP family class I SAM-dependent RNA met	QEN71_03045	paras_001161	protein-codi	NZ_CP1252	chromosom	680454	681713	+	1256	0	19	5312	16	4915	
QEN71_RS03050	mechanosensitive ion channel	QEN71_03050	paras_001162	protein-codi	NZ_CP1252	chromosom	681800	683155	+	1356	0	32	16627	28	15995	
QEN71_RS03055	acyl-CoA desaturase	QEN71_03055	paras_001163	protein-codi	NZ_CP1252	chromosom	683361	684557	+	1197	0	17	195	16	144	
QEN71_RS03060	quinolinate synthase NadA	QEN71_03060	paras_001164	protein-codi	NZ_CP1252	chromosom	684690	685820	+	1130	0	27	11815	25	11720	
QEN71_RS03065	carboxylating nicotinate-nucleotide diphosphorylase	QEN71_03065	paras_001165	protein-codi	NZ_CP1252	chromosom	685820	686722	+	902	0	15	2298	11	1974	
QEN71_RS03070	L-aspartate oxidase	QEN71_03070	paras_001166	protein-codi	NZ_CP1252	chromosom	686802	688400	-	1599	0	45	12783	39	11751	
QEN71_RS03075	50S ribosomal protein L33	QEN71_03075	paras_001167	protein-codi	NZ_CP1252	chromosom	688634	688801	-	168	0	5	907	4	609	
QEN71_RS03080	50S ribosomal protein L28	QEN71_03080	paras_001168	protein-codi	NZ_CP1252	chromosom	688812	689045	-	234	0	1	172	0	0	
QEN71_RS03085	DNA repair protein RadC	QEN71_03085	paras_001169	protein-codi	NZ_CP1252	chromosom	689303	690031	-	729	0	20	6185	16	5337	
QEN71_RS03090	peptidylprolyl isomerase	QEN71_03090	paras_001170	protein-codi	NZ_CP1252	chromosom	690221	690676	+	456	0	7	1054	5	595	
QEN71_RS03095	4-hydroxy-3-methylbut-2-enyl diphosphate reductas	QEN71_03095	paras_001171	protein-codi	NZ_CP1252	chromosom	690679	691638	+	960	0	7	52	6	48	
QEN71_RS03100	branched-chain amino acid ABC transporter substra	QEN71_03100	paras_001172	protein-codi	NZ_CP1252	chromosom	692177	693376	+	1200	0	46	21981	36	17806	
QEN71_RS03105	branched-chain amino acid ABC transporter permea	QEN71_03105	paras_001173	protein-codi	NZ_CP1252	chromosom	693605	694555	+	951	0	33	10226	18	6669	
QEN71_RS03110	ABC transporter ATP-binding protein	QEN71_03110	paras_001174	protein-codi	NZ_CP1252	chromosom	694574	695743	+	1170	0	30	10989	28	10707	
QEN71_RS03115	ABC transporter ATP-binding protein	QEN71_03115	paras_001175	protein-codi	NZ_CP1252	chromosom	695762	696538	+	776	0	26	7105	24	5968	
QEN71_RS03120	ABC transporter ATP-binding protein	QEN71_03120	paras_001176	protein-codi	NZ_CP1252	chromosom	696538	697254	+	716	0	31	9172	22	6097	
QEN71_RS03125	GNAT family acetyltransferase	QEN71_03125	paras_001177	protein-codi	NZ_CP1252	chromosom	697913	698371	-	455	0	24	4067	18	3692	
QEN71_RS03130	acetylornithine transaminase	QEN71_03130	paras_001178	protein-codi	NZ_CP1252	chromosom	698368	699552	-	1181	0	31	8446	23	6482	
QEN71_RS03135	CDP-6-deoxy-delta-3,4-glucoseen reductase	QEN71_03135	paras_001179	protein-codi	NZ_CP1252	chromosom	699890	700921	-	1032	0	23	6811	14	5051	
QEN71_RS03140	SDR family NAD(P)-dependent oxidoreductase	QEN71_03140	paras_001180	protein-codi	NZ_CP1252	chromosom	700995	702059	+	1065	0	19	7478	16	6613	
QEN71_RS03145	hypothetical protein	QEN71_03145	paras_001181	protein-codi	NZ_CP1252	chromosom	702083	702271	-	189	0	10	1231	6	707	
QEN71_RS03150	16S rRNA pseudouridine(516) synthase	QEN71_03150	paras_001182	protein-codi	NZ_CP1252	chromosom	702437	703141	-	705	0	17	4934	14	4603	
QEN71_RS03155	glycine betaine/L-proline ABC transporter ATP-bind	QEN71_03155	paras_001183	protein-codi	NZ_CP1252	chromosom	703303	704124	-	822	0	12	808	8	496	
QEN71_RS03160	proline/glycine betaine ABC transporter permease	QEN71_03160	paras_001184	protein-codi	NZ_CP1252	chromosom	704139	705128	-	990	0	14	4479	13	4408	
QEN71_RS03165	ABC transporter substrate-binding protein	QEN71_03165	paras_001185	protein-codi	NZ_CP1252	chromosom	705168	706205	-	1038	0	25	6891	24	6872	

QEN71_RS03170	mandelate racemase/muconate lactonizing enzyme	QEN71_03170	paras_001186	protein-codi	NZ_CP1252	chromosom	706268	707452	-	1185	0	22	4571	14	2762	
QEN71_RS03175	PLP-dependent aminotransferase family protein	QEN71_03175	paras_001187	protein-codi	NZ_CP1252	chromosom	707721	709151	+	1431	0	24	5523	19	4444	
QEN71_RS03180	MFS transporter	QEN71_03180	paras_001188	protein-codi	NZ_CP1252	chromosom	709266	710519	+	1254	0	22	5043	22	5043	
QEN71_RS03185	alpha/beta hydrolase	QEN71_03185	paras_001189	protein-codi	NZ_CP1252	chromosom	710558	711493	-	936	0	17	7348	13	5401	
QEN71_RS03190	amino acid permease	QEN71_03190	paras_001190	protein-codi	NZ_CP1252	chromosom	711764	713164	+	1401	0	46	17311	40	15980	
QEN71_RS03195	tRNA-Leu	QEN71_03195		tRNA	NZ_CP1252	chromosom	713331	713415	-	85	0	0	0	0	0	
QEN71_RS03200	oxygen-independent coproporphyrinogen III oxidase	QEN71_03200	paras_001192	protein-codi	NZ_CP1252	chromosom	713509	714912	-	1404	0	40	9318	31	8014	
QEN71_RS03205	YkgJ family cysteine cluster protein	QEN71_03205	paras_001193	protein-codi	NZ_CP1252	chromosom	715134	715430	+	297	0	6	2044	6	2044	
QEN71_RS03210	DUF1439 domain-containing protein	QEN71_03210	paras_001194	protein-codi	NZ_CP1252	chromosom	715517	716110	+	594	0	23	4300	19	3663	
QEN71_RS03215	undecaprenyl-diphosphate phosphatase	QEN71_03215	paras_001195	protein-codi	NZ_CP1252	chromosom	716190	717026	+	837	0	7	256	3	53	
QEN71_RS03220	tRNA (guanosine(46)-N7)-methyltransferase TrmB	QEN71_03220	paras_001196	protein-codi	NZ_CP1252	chromosom	717110	717889	-	780	0	20	5455	17	4815	
QEN71_RS03225	tRNA-Gly	QEN71_03225		tRNA	NZ_CP1252	chromosom	718080	718153	-	74	0	13	5757	13	5757	
QEN71_RS03230	YggT family protein	QEN71_03230	paras_001198	protein-codi	NZ_CP1252	chromosom	718252	718815	-	564	0	10	2804	8	2346	
QEN71_RS03235	EthD family reductase	QEN71_03235	paras_001199	protein-codi	NZ_CP1252	chromosom	718950	719270	-	321	0	13	3241	10	3231	
QEN71_RS03240	DUF2520 domain-containing protein	QEN71_03240	paras_001200	protein-codi	NZ_CP1252	chromosom	719442	720380	-	939	0	21	7231	18	6703	
QEN71_RS03245	hypothetical protein	QEN71_03245	paras_001201	protein-codi	NZ_CP1252	chromosom	720648	720809	+	162	0	9	4942	7	3176	
QEN71_RS03250	hypothetical protein	QEN71_03250	paras_001202	protein-codi	NZ_CP1252	chromosom	721226	721516	+	291	0	37	10044	28	8793	
QEN71_RS03255	LysE family transporter	QEN71_03255	paras_001203	protein-codi	NZ_CP1252	chromosom	721637	722314	-	678	0	10	3047	5	1129	
QEN71_RS03260	UbiD family decarboxylase	QEN71_03260	paras_001204	protein-codi	NZ_CP1252	chromosom	722372	723949	-	1578	0	39	1872	30	1576	
QEN71_RS03265	transglycosylase SLT domain-containing protein	QEN71_03265	paras_001205	protein-codi	NZ_CP1252	chromosom	724543	725742	+	1200	0	17	4929	16	4903	
QEN71_RS03270	IS4 family transposase	QEN71_03270	paras_001206	protein-codi	NZ_CP1252	chromosom	725807	727153	-	1347	2661.0	12	1147	9	517	
QEN71_RS03275	pyridoxal phosphate-dependent aminotransferase	QEN71_03275	paras_001207	protein-codi	NZ_CP1252	chromosom	727267	728460	-	1194	0	48	15229	35	11761	
QEN71_RS03280	transcription antitermination factor NusB	QEN71_03280	paras_001208	protein-codi	NZ_CP1252	chromosom	728581	729018	-	434	0	3	389	1	16	
QEN71_RS03285	6,7-dimethyl-8-ribityllumazine synthase	QEN71_03285	paras_001209	protein-codi	NZ_CP1252	chromosom	729015	729533	-	515	0	0	0	0	0	TRUE
QEN71_RS03290	bifunctional 3,4-dihydroxy-2-butanone-4-phosphate	QEN71_03290	paras_001210	protein-codi	NZ_CP1252	chromosom	729612	730748	-	1137	0	10	4208	10	4208	
QEN71_RS03295	riboflavin synthase	QEN71_03295	paras_001211	protein-codi	NZ_CP1252	chromosom	730863	731477	-	615	0	3	698	1	4	
QEN71_RS03300	bifunctional diaminohydroxyphosphoribosylaminopy	QEN71_03300	paras_001212	protein-codi	NZ_CP1252	chromosom	731502	732623	-	1122	0	1	2	1	2	TRUE
QEN71_RS03305	glutamate-1-semialdehyde 2,1-aminomutase	QEN71_03305	paras_001213	protein-codi	NZ_CP1252	chromosom	732663	733943	-	1281	0	0	0	0	0	TRUE
QEN71_RS03310	hypothetical protein	QEN71_03310	paras_001214	protein-codi	NZ_CP1252	chromosom	734148	734792	-	645	0	11	4457	11	4457	
QEN71_RS03315	Bcr/CflA family multidrug efflux MFS transporter	QEN71_03315	paras_001215	protein-codi	NZ_CP1252	chromosom	734976	736187	+	1212	0	41	12405	33	7273	
QEN71_RS03320	bifunctional transcriptional regulator/glucokinase	QEN71_03320	paras_001216	protein-codi	NZ_CP1252	chromosom	736401	738317	-	1894	0	50	12487	29	8450	
QEN71_RS03325	6-phosphogluconolactonase	QEN71_03325	paras_001217	protein-codi	NZ_CP1252	chromosom	738295	739002	-	685	0	8	1512	8	1512	
QEN71_RS03330	glucose-6-phosphate dehydrogenase	QEN71_03330	paras_001218	protein-codi	NZ_CP1252	chromosom	739150	740607	-	1458	0	42	10666	30	7173	
QEN71_RS03335	ABC transporter substrate-binding protein	QEN71_03335	paras_001219	protein-codi	NZ_CP1252	chromosom	741137	742384	+	1248	0	26	8867	24	7567	
QEN71_RS03340	sugar ABC transporter permease	QEN71_03340	paras_001220	protein-codi	NZ_CP1252	chromosom	742613	743491	+	868	0	13	3132	10	2272	
QEN71_RS03345	carbohydrate ABC transporter permease	QEN71_03345	paras_001221	protein-codi	NZ_CP1252	chromosom	743481	744338	+	847	0	25	4488	14	3053	
QEN71_RS03350	sn-glycerol-3-phosphate ABC transporter ATP-bindi	QEN71_03350	paras_001222	protein-codi	NZ_CP1252	chromosom	744450	745565	+	1116	0	38	5400	25	3320	
QEN71_RS03355	hypothetical protein	QEN71_03355	paras_001223	protein-codi	NZ_CP1252	chromosom	746139	747503	+	1365	0	87	16336	68	12311	
QEN71_RS03360	IS256 family transposase	pseudo;QEN71_03360		pseudogene	NZ_CP1252	chromosom	748032	749302	-	1271	0	32	5135	28	4232	
QEN71_RS03365	trimeric intracellular cation channel family protein	QEN71_03365	paras_001226	protein-codi	NZ_CP1252	chromosom	749363	749983	-	621	0	37	6913	28	5446	
QEN71_RS03370	Smr/MutS family protein	QEN71_03370	paras_001227	protein-codi	NZ_CP1252	chromosom	749999	750817	-	817	0	17	3554	12	2007	
QEN71_RS03375	hypothetical protein	QEN71_03375		protein-codi	NZ_CP1252	chromosom	750816	750950	+	133	0	8	1180	6	677	
QEN71_RS03380	thioredoxin-disulfide reductase	QEN71_03380	paras_001228	protein-codi	NZ_CP1252	chromosom	750980	751987	-	1008	0	1	5	1	5	TRUE
QEN71_RS03385	DNA translocase FtsK	QEN71_03385	paras_001229	protein-codi	NZ_CP1252	chromosom	752473	754788	+	2316	0	47	9337	36	7234	
QEN71_RS03390	outer membrane lipoprotein chaperone LolA	QEN71_03390	paras_001230	protein-codi	NZ_CP1252	chromosom	754825	755520	+	696	0	2	139	1	11	TRUE
QEN71_RS03395	LysR family transcriptional regulator	QEN71_03395	paras_001231	protein-codi	NZ_CP1252	chromosom	755530	756444	-	915	0	27	6265	20	3412	
QEN71_RS03400	agmatinase	QEN71_03400	paras_001232	protein-codi	NZ_CP1252	chromosom	756607	757599	+	993	0	23	4952	17	4144	
QEN71_RS03405	tetratricopeptide repeat-containing glycosyltransfera	QEN71_03405	paras_001233	protein-codi	NZ_CP1252	chromosom	757847	759478	+	1632	0	56	9552	47	8521	
QEN71_RS03410	putative hydroxymethylpyrimidine transporter CytX	QEN71_03410	paras_001234	protein-codi	NZ_CP1252	chromosom	759492	760808	-	1317	0	27	5457	17	2686	
QEN71_RS03415	replication-associated recombination protein A	QEN71_03415	paras_001235	protein-codi	NZ_CP1252	chromosom	761118	762428	+	1311	0	33	8262	30	8178	
QEN71_RS03420	serine--tRNA ligase	QEN71_03420	paras_001236	protein-codi	NZ_CP1252	chromosom	762520	763818	+	1299	0	2	11	2	11	TRUE
QEN71_RS03425	tRNA-Ser	QEN71_03425		tRNA	NZ_CP1252	chromosom	763968	764058	+	91	0	0	0	0	0	
QEN71_RS03430	hypothetical protein	QEN71_03430	paras_001238	protein-codi	NZ_CP1252	chromosom	764371	764601	+	231	0	0	0	0	0	
QEN71_RS03435	PAS domain-containing sensor histidine kinase	QEN71_03435	paras_001239	protein-codi	NZ_CP1252	chromosom	765121	766461	+	1337	0	14	3107	7	822	
QEN71_RS03440	response regulator	QEN71_03440	paras_001240	protein-codi	NZ_CP1252	chromosom	766458	767129	+	668	0	11	1820	1	198	
QEN71_RS03445	response regulator	QEN71_03445	paras_001241	protein-codi	NZ_CP1252	chromosom	767192	767578	+	387	0	12	1387	8	1200	
QEN71_RS03450	efflux RND transporter periplasmic adaptor subunit	QEN71_03450	paras_001242	protein-codi	NZ_CP1252	chromosom	767818	769071	+	1254	1501.0	11	2207	11	2207	
QEN71_RS03455	multidrug efflux RND transporter permease subunit	QEN71_03455	paras_001243	protein-codi	NZ_CP1252	chromosom	769135	772395	+	3261	3829.0	34	7532	27	5168	
QEN71_RS03460	efflux transporter outer membrane subunit	QEN71_03460	paras_001244	protein-codi	NZ_CP1252	chromosom	772397	773869	+	1469	1167.0	36	7121	21	4789	
QEN71_RS03465	paraquat-inducible protein A	QEN71_03465	paras_001245	protein-codi	NZ_CP1252	chromosom	773866	775215	+	1342	0	23	3579	21	3461	
QEN71_RS03470	MlaD family protein	QEN71_03470	paras_001246	protein-codi	NZ_CP1252	chromosom	775212	776807	+	1588	0	23	3775	21	3687	
QEN71_RS03475	PqiC family protein	QEN71_03475	paras_001247	protein-codi	NZ_CP1252	chromosom	776804	777394	+	587	0	13	2528	8	1671	

QEN71_RS03480	fimbrial protein	QEN71_03480	paras_001248	protein-codi	NZ_CP1252	chromosom	777501	778025	-	525	0	12	1851	10	1313	
QEN71_RS03485	fimbrial protein	QEN71_03485	paras_001249	protein-codi	NZ_CP1252	chromosom	778155	779240	-	1086	0	57	7712	44	5693	
QEN71_RS03490	fimbria/pilus outer membrane usher protein	QEN71_03490	paras_001250	protein-codi	NZ_CP1252	chromosom	779359	782115	-	2757	0	74	9171	61	7012	
QEN71_RS03495	fimbria/pilus periplasmic chaperone	QEN71_03495	paras_001251	protein-codi	NZ_CP1252	chromosom	782314	783057	-	744	0	13	1407	10	1042	
QEN71_RS03500	fimbrial protein	QEN71_03500	paras_001253	protein-codi	NZ_CP1252	chromosom	783284	783832	-	549	0	17	3449	10	1269	
QEN71_RS03505	type I methionyl aminopeptidase	QEN71_03505	paras_001254	protein-codi	NZ_CP1252	chromosom	784338	785123	+	786	0	23	4743	14	2838	
QEN71_RS03510	GNAT family N-acetyltransferase	QEN71_03510	paras_001255	protein-codi	NZ_CP1252	chromosom	785365	785907	+	543	0	11	1281	10	1278	
QEN71_RS03515	septum site-determining protein MinC	QEN71_03515	paras_001256	protein-codi	NZ_CP1252	chromosom	786129	786965	+	837	0	8	1187	7	1046	
QEN71_RS03520	septum site-determining protein MinD	QEN71_03520	paras_001257	protein-codi	NZ_CP1252	chromosom	787055	787870	+	816	0	14	2592	11	2385	
QEN71_RS03525	cell division topological specificity factor MinE	QEN71_03525	paras_001258	protein-codi	NZ_CP1252	chromosom	787886	788140	+	255	0	0	0	0	0	
QEN71_RS03530	YXWGXW repeat-containing protein	QEN71_03530	paras_001259	protein-codi	NZ_CP1252	chromosom	788399	788710	+	312	0	5	1101	5	1101	
QEN71_RS03535	chloride channel protein	QEN71_03535	paras_001260	protein-codi	NZ_CP1252	chromosom	788994	790391	-	1398	0	37	11076	19	6727	
QEN71_RS03540	lipopolysaccharide heptosyltransferase I	QEN71_03540	paras_001261	protein-codi	NZ_CP1252	chromosom	790664	791671	+	1008	0	26	7755	17	4571	
QEN71_RS03545	hypothetical protein	QEN71_03545	paras_001262	protein-codi	NZ_CP1252	chromosom	792444	792623	-	180	0	0	0	0	0	
QEN71_RS03550	MFS transporter	QEN71_03550	paras_001263	protein-codi	NZ_CP1252	chromosom	792832	794139	-	1308	0	32	12297	18	6231	
QEN71_RS03555	TonB family protein	QEN71_03555	paras_001264	protein-codi	NZ_CP1252	chromosom	794559	795065	-	507	0	8	1860	8	1860	
QEN71_RS03560	hypothetical protein	QEN71_03560	paras_001265	protein-codi	NZ_CP1252	chromosom	795244	795393	+	150	0	5	1126	5	1126	
QEN71_RS03565	site-specific integrase	QEN71_03565	paras_001266	protein-codi	NZ_CP1252	chromosom	795527	796723	+	1197	0	38	13610	31	13148	
QEN71_RS03570	hypothetical protein	QEN71_03570	paras_001267	protein-codi	NZ_CP1252	chromosom	797334	798059	-	726	0	51	12481	45	11196	
QEN71_RS03575	hypothetical protein	QEN71_03575	paras_001268	protein-codi	NZ_CP1252	chromosom	798149	798445	-	297	0	20	2400	18	2272	
QEN71_RS03580	RHS repeat-associated core domain-containing pro	QEN71_03580	paras_001269	protein-codi	NZ_CP1252	chromosom	798469	802719	-	4251	0	183	55462	138	46255	
QEN71_RS03585	tetratricopeptide repeat protein	QEN71_03585	paras_001270	protein-codi	NZ_CP1252	chromosom	802725	804089	-	1365	0	43	8872	31	6292	
QEN71_RS03590	hypothetical protein	QEN71_03590	paras_001271	protein-codi	NZ_CP1252	chromosom	804406	804831	-	426	0	9	2165	9	2165	
QEN71_RS03595	hypothetical protein	QEN71_03595	paras_001272	protein-codi	NZ_CP1252	chromosom	805111	805653	-	543	0	15	5740	13	5317	
QEN71_RS03600	hypothetical protein	QEN71_03600	paras_001273	protein-codi	NZ_CP1252	chromosom	805973	806653	-	681	0	15	5665	15	5665	
QEN71_RS03605	hypothetical protein	QEN71_03605	paras_001274	protein-codi	NZ_CP1252	chromosom	806688	807302	-	611	0	7	1930	7	1930	
QEN71_RS03610	AAA family ATPase	QEN71_03610	paras_001275	protein-codi	NZ_CP1252	chromosom	807299	808546	-	1244	0	22	3642	19	3331	
QEN71_RS03615	hypothetical protein	QEN71_03615	paras_001276	protein-codi	NZ_CP1252	chromosom	808723	809115	-	392	0	16	3984	11	2844	
QEN71_RS03620	AlpA family phage regulatory protein	QEN71_03620	paras_001277	protein-codi	NZ_CP1252	chromosom	809115	809330	-	215	0	12	2305	10	1377	
QEN71_RS03625	hypothetical protein	QEN71_03625	paras_001278	protein-codi	NZ_CP1252	chromosom	809452	810252	-	801	0	42	11457	37	10641	
QEN71_RS03630	tRNA-Ser	QEN71_03630		tRNA	NZ_CP1252	chromosom	810543	810632	-	90	0	0	0	0	0	
QEN71_RS03635	radical SAM family heme chaperone HemW	QEN71_03635	paras_001280	protein-codi	NZ_CP1252	chromosom	810690	811904	-	1211	0	28	4955	26	4311	
QEN71_RS03640	RdgB/HAM1 family non-canonical purine NTP pyrop	QEN71_03640	paras_001281	protein-codi	NZ_CP1252	chromosom	811901	812536	-	624	0	8	2780	8	2780	
QEN71_RS03645	ribonuclease PH	QEN71_03645	paras_001282	protein-codi	NZ_CP1252	chromosom	812529	813269	-	733	0	13	570	11	478	
QEN71_RS03650	YicC/YloC family endoribonuclease	QEN71_03650	paras_001283	protein-codi	NZ_CP1252	chromosom	813567	814490	+	924	0	12	3765	2	531	
QEN71_RS03655	guanylate kinase	QEN71_03655	paras_001284	protein-codi	NZ_CP1252	chromosom	814509	815183	+	675	0	4	91	1	16	TRUE
QEN71_RS03660	DNA-directed RNA polymerase subunit omega	QEN71_03660	paras_001285	protein-codi	NZ_CP1252	chromosom	815246	815449	+	204	0	3	265	1	70	
QEN71_RS03665	bifunctional (p)ppGpp synthetase/guanosine-3',5'-bi	QEN71_03665	paras_001286	protein-codi	NZ_CP1252	chromosom	815529	817913	+	2385	0	25	1608	18	1369	
QEN71_RS03670	DUF2591 family protein	QEN71_03670	paras_001287	protein-codi	NZ_CP1252	chromosom	817981	818469	+	489	0	14	4403	6	1144	
QEN71_RS03675	tRNA-Arg	QEN71_03675		tRNA	NZ_CP1252	chromosom	818556	818632	-	77	152.0	6	1389	5	1316	
QEN71_RS03680	tRNA-Arg	QEN71_03680		tRNA	NZ_CP1252	chromosom	818738	818814	-	77	152.0	0	0	0	0	
QEN71_RS03685	transcription elongation factor GreB	QEN71_03685	paras_001290	protein-codi	NZ_CP1252	chromosom	818935	819507	+	573	0	22	10286	18	9156	
QEN71_RS03690	porin	QEN71_03690	paras_001291	protein-codi	NZ_CP1252	chromosom	819626	820789	-	1164	0	38	6296	30	4508	
QEN71_RS03695	hypothetical protein	QEN71_03695	paras_001292	protein-codi	NZ_CP1252	chromosom	821323	821565	-	243	0	4	2246	2	1614	
QEN71_RS03700	cold-shock protein	QEN71_03700	paras_001293	protein-codi	NZ_CP1252	chromosom	821974	822177	+	204	0	4	624	2	280	
QEN71_RS03705	exonuclease domain-containing protein	QEN71_03705	paras_001294	protein-codi	NZ_CP1252	chromosom	822502	823680	+	1175	0	25	7924	25	7924	
QEN71_RS03710	chorismate mutase	QEN71_03710	paras_001295	protein-codi	NZ_CP1252	chromosom	823677	824273	-	593	0	7	2139	7	2139	
QEN71_RS03715	hypothetical protein	QEN71_03715	paras_001296	protein-codi	NZ_CP1252	chromosom	824460	824624	-	149	0	3	466	3	466	
QEN71_RS03720	hypothetical protein	QEN71_03720	paras_001297	protein-codi	NZ_CP1252	chromosom	824609	824758	-	134	0	0	0	0	0	
QEN71_RS03725	DUF2946 domain-containing protein	QEN71_03725	paras_001298	protein-codi	NZ_CP1252	chromosom	824985	825365	+	381	0	16	2670	14	2432	
QEN71_RS03730	hypothetical protein	QEN71_03730	paras_001299	protein-codi	NZ_CP1252	chromosom	825373	825531	-	157	0	3	582	3	582	
QEN71_RS03735	TonB-dependent receptor	QEN71_03735	paras_001300	protein-codi	NZ_CP1252	chromosom	825530	827911	+	2367	0	87	12306	66	9947	
QEN71_RS03740	sialidase family protein	QEN71_03740	paras_001301	protein-codi	NZ_CP1252	chromosom	827899	829152	+	1241	0	28	5136	20	2508	
QEN71_RS03745	TlpA family protein disulfide reductase	QEN71_03745	paras_001302	protein-codi	NZ_CP1252	chromosom	829156	829623	+	468	0	6	1057	6	1057	
QEN71_RS03750	LysR family transcriptional regulator	QEN71_03750	paras_001303	protein-codi	NZ_CP1252	chromosom	829630	830565	-	936	0	12	2280	8	1618	
QEN71_RS03755	CoA transferase	QEN71_03755	paras_001304	protein-codi	NZ_CP1252	chromosom	830659	831909	+	1243	0	17	3462	12	2394	
QEN71_RS03760	acyl-CoA dehydrogenase family protein	QEN71_03760	paras_001305	protein-codi	NZ_CP1252	chromosom	831902	833041	+	1132	0	23	4391	17	2690	
QEN71_RS03765	tRNA-Thr	QEN71_03765		tRNA	NZ_CP1252	chromosom	833097	833172	-	76	0	2	7	1	3	
QEN71_RS03770	class 1 fructose-bisphosphatase	QEN71_03770	paras_001307	protein-codi	NZ_CP1252	chromosom	833254	834270	-	1017	0	0	0	0	0	TRUE
QEN71_RS03775	HigA family addiction module antitoxin	QEN71_03775	paras_001308	protein-codi	NZ_CP1252	chromosom	834422	834862	-	441	0	2	498	2	498	
QEN71_RS03780	type II toxin-antitoxin system RelE/ParE family toxin	QEN71_03780	paras_001309	protein-codi	NZ_CP1252	chromosom	834879	835160	-	282	0	6	2152	4	1709	
QEN71_RS03785	aminopeptidase N	QEN71_03785	paras_001310	protein-codi	NZ_CP1252	chromosom	835319	838021	-	2703	0	56	4467	50	4241	

QEN71_RS03790	DUF4136 domain-containing protein	QEN71_03790	paras_001311	protein-codi	NZ_CP1252	chromosom	838229	838930	+	702	0	37	14417	35	13241	
QEN71_RS03795	TMEM165/GDT1 family protein	QEN71_03795	paras_001312	protein-codi	NZ_CP1252	chromosom	839040	839612	-	573	0	0	0	0	0	TRUE
QEN71_RS03800	beta-ketoacyl synthase chain length factor	QEN71_03800	paras_001313	protein-codi	NZ_CP1252	chromosom	840366	841106	+	724	0	16	3495	11	1648	
QEN71_RS03805	lysophospholipid acyltransferase family protein	QEN71_03805	paras_001314	protein-codi	NZ_CP1252	chromosom	841090	841884	+	758	0	14	5956	9	2549	
QEN71_RS03810	phosphopantetheine-binding protein	QEN71_03810	paras_001315	protein-codi	NZ_CP1252	chromosom	841865	842164	+	280	0	6	1398	5	1226	
QEN71_RS03815	acyl carrier protein	QEN71_03815	paras_001316	protein-codi	NZ_CP1252	chromosom	842167	842418	+	251	0	0	0	0	0	
QEN71_RS03820	hypothetical protein	QEN71_03820	paras_001317	protein-codi	NZ_CP1252	chromosom	842418	843116	+	694	0	23	6135	19	4846	
QEN71_RS03825	AMP-binding protein	QEN71_03825	paras_001318	protein-codi	NZ_CP1252	chromosom	843113	844801	+	1681	0	29	5639	24	5149	
QEN71_RS03830	glycosyltransferase family 2 protein	QEN71_03830	paras_001319	protein-codi	NZ_CP1252	chromosom	844798	846573	+	1758	0	49	12236	38	11006	
QEN71_RS03835	aromatic amino acid ammonia-lyase	QEN71_03835	paras_001320	protein-codi	NZ_CP1252	chromosom	846560	848158	+	1577	0	20	2325	16	2071	
QEN71_RS03840	thioesterase family protein	QEN71_03840	paras_001321	protein-codi	NZ_CP1252	chromosom	848151	848588	+	417	0	17	2791	17	2791	
QEN71_RS03845	outer membrane lipoprotein carrier protein LolA	QEN71_03845	paras_001322	protein-codi	NZ_CP1252	chromosom	848576	849244	+	655	0	13	2927	8	1495	
QEN71_RS03850	MMPL family transporter	QEN71_03850	paras_001323	protein-codi	NZ_CP1252	chromosom	849244	851661	+	2413	0	41	6719	37	6123	
QEN71_RS03855	beta-ketoacyl-[acyl-carrier-protein] synthase family	QEN71_03855	paras_001324	protein-codi	NZ_CP1252	chromosom	851658	852839	+	1178	0	17	2060	11	1473	
QEN71_RS03860	hotdog family protein	QEN71_03860	paras_001325	protein-codi	NZ_CP1252	chromosom	852871	853365	+	491	0	9	1428	7	1089	
QEN71_RS03865	3-ketoacyl-ACP reductase FabG2	QEN71_03865	paras_001326	protein-codi	NZ_CP1252	chromosom	853362	854087	+	721	0	15	3196	10	2236	
QEN71_RS03870	beta-ketoacyl-ACP synthase	QEN71_03870	paras_001327	protein-codi	NZ_CP1252	chromosom	854087	855313	+	1222	0	34	7556	26	6510	
QEN71_RS03875	beta-ketoacyl synthase N-terminal-like domain-cont	QEN71_03875	paras_001328	protein-codi	NZ_CP1252	chromosom	855310	856536	+	1223	0	24	4077	22	3747	
QEN71_RS03880	hypothetical protein	QEN71_03880	paras_001329	protein-codi	NZ_CP1252	chromosom	856610	857044	+	435	0	10	1224	6	856	
QEN71_RS03885	excinuclease ABC subunit A	QEN71_03885	paras_001330	protein-codi	NZ_CP1252	chromosom	857071	857502	+	432	0	11	2119	9	1677	
QEN71_RS03890	class I SAM-dependent methyltransferase	QEN71_03890	paras_001331	protein-codi	NZ_CP1252	chromosom	857548	858312	-	765	0	23	5352	21	5090	
QEN71_RS03895	NAD(P)/FAD-dependent oxidoreductase	QEN71_03895	paras_001332	protein-codi	NZ_CP1252	chromosom	858435	859676	+	1242	0	38	4961	27	2875	
QEN71_RS03900	guanine deaminase	QEN71_03900	paras_001333	protein-codi	NZ_CP1252	chromosom	860296	861633	-	1338	0	30	6201	25	4996	
QEN71_RS03905	adenosine deaminase	QEN71_03905	paras_001334	protein-codi	NZ_CP1252	chromosom	861662	862702	-	1041	0	34	7886	26	5972	
QEN71_RS03910	xanthine dehydrogenase accessory protein XdhC	QEN71_03910	paras_001335	protein-codi	NZ_CP1252	chromosom	862781	863791	-	1011	0	6	1223	6	1223	
QEN71_RS03915	xanthine dehydrogenase molybdopterin binding sub	QEN71_03915	paras_001336	protein-codi	NZ_CP1252	chromosom	863892	866318	-	2423	0	56	12532	49	10493	
QEN71_RS03920	xanthine dehydrogenase small subunit	QEN71_03920	paras_001337	protein-codi	NZ_CP1252	chromosom	866315	867856	-	1538	0	29	9776	24	7172	
QEN71_RS03925	hypothetical protein	QEN71_03925	paras_001338	protein-codi	NZ_CP1252	chromosom	868013	868243	+	231	170.0	6	895	4	648	
QEN71_RS03930	disulfide bond formation protein B	QEN71_03930	paras_001339	protein-codi	NZ_CP1252	chromosom	868638	869144	-	507	0	20	8066	13	7030	
QEN71_RS03935	amidase	QEN71_03935	paras_001340	protein-codi	NZ_CP1252	chromosom	869241	870629	-	1389	0	24	5685	14	3972	
QEN71_RS03940	cation:proton antiporter	QEN71_03940	paras_001341	protein-codi	NZ_CP1252	chromosom	870793	872562	+	1770	0	34	9848	25	7708	
QEN71_RS03945	type 1 glutamine amidotransferase	QEN71_03945	paras_001342	protein-codi	NZ_CP1252	chromosom	872721	874091	+	1371	0	23	4020	20	3718	
QEN71_RS03950	DUF2968 domain-containing protein	QEN71_03950	paras_001343	protein-codi	NZ_CP1252	chromosom	874347	875090	+	744	0	19	4433	17	4355	
QEN71_RS03955	hypothetical protein	QEN71_03955	paras_001344	protein-codi	NZ_CP1252	chromosom	875179	876171	-	993	0	19	2649	12	1496	
QEN71_RS03960	GNAT family N-acetyltransferase	QEN71_03960	paras_001345	protein-codi	NZ_CP1252	chromosom	876328	876894	-	567	0	14	2484	12	2388	
QEN71_RS03965	MFS transporter	QEN71_03965	paras_001346	protein-codi	NZ_CP1252	chromosom	877309	878610	+	1302	0	44	11240	29	8302	
QEN71_RS03970	tetratricopeptide repeat protein	QEN71_03970	paras_001347	protein-codi	NZ_CP1252	chromosom	878694	880541	-	1848	0	18	1876	14	1730	
QEN71_RS03975	metalloregulator ArsR/SmtB family transcription fact	QEN71_03975	paras_001348	protein-codi	NZ_CP1252	chromosom	880688	881032	+	341	0	2	581	2	581	
QEN71_RS03980	cation diffusion facilitator family transporter	QEN71_03980	paras_001349	protein-codi	NZ_CP1252	chromosom	881029	882126	+	1094	0	16	4119	10	1545	
QEN71_RS03985	FAD-dependent monooxygenase	QEN71_03985	paras_001350	protein-codi	NZ_CP1252	chromosom	882236	883972	+	1737	0	28	10183	20	8125	
QEN71_RS03990	formate dehydrogenase subunit delta	QEN71_03990	paras_001351	protein-codi	NZ_CP1252	chromosom	884060	884314	-	255	0	9	2647	3	230	
QEN71_RS03995	formate dehydrogenase subunit alpha	QEN71_03995	paras_001352	protein-codi	NZ_CP1252	chromosom	884324	887263	-	2940	0	76	21375	61	17852	
QEN71_RS04000	formate dehydrogenase beta subunit	QEN71_04000	paras_001353	protein-codi	NZ_CP1252	chromosom	887279	888835	-	1553	0	39	21044	34	16216	
QEN71_RS04005	NAD(P)H-dependent oxidoreductase subunit E	QEN71_04005	paras_001354	protein-codi	NZ_CP1252	chromosom	888832	889350	-	515	0	15	7391	14	7241	
QEN71_RS04010	substrate-binding domain-containing protein	QEN71_04010	paras_001355	protein-codi	NZ_CP1252	chromosom	889485	890567	+	1083	0	26	6399	23	5766	
QEN71_RS04015	hypothetical protein	QEN71_04015	paras_001356	protein-codi	NZ_CP1252	chromosom	890711	890977	+	267	0	9	2384	4	382	
QEN71_RS04020	hypothetical protein	QEN71_04020	paras_001357	protein-codi	NZ_CP1252	chromosom	891058	891417	-	360	0	8	2149	6	2112	
QEN71_RS04025	glycosyltransferase family 4 protein	QEN71_04025	paras_001358	protein-codi	NZ_CP1252	chromosom	891755	892882	+	1128	0	32	12796	20	5932	
QEN71_RS04030	PAAR domain-containing protein	QEN71_04030	paras_001359	protein-codi	NZ_CP1252	chromosom	892922	893206	-	285	0	11	2185	7	1673	
QEN71_RS04040	HAD-IA family hydrolase	QEN71_04040	paras_001361	protein-codi	NZ_CP1252	chromosom	893877	894590	-	714	0	14	2838	14	2838	
QEN71_RS04045	bifunctional 2-polyprenyl-6-hydroxyphenol methylas	QEN71_04045	paras_001362	protein-codi	NZ_CP1252	chromosom	894627	895325	-	699	0	0	0	0	0	TRUE
QEN71_RS04050	OmpA family protein	QEN71_04050	paras_001363	protein-codi	NZ_CP1252	chromosom	895542	896189	-	648	0	15	1969	9	1082	
QEN71_RS04055	DNA gyrase subunit A	QEN71_04055	paras_001364	protein-codi	NZ_CP1252	chromosom	896705	899332	+	2628	0	3	83	2	15	TRUE
QEN71_RS04060	DUF2059 domain-containing protein	QEN71_04060	paras_001365	protein-codi	NZ_CP1252	chromosom	899450	900058	+	609	0	2	2121	0	0	
QEN71_RS04065	3-phosphoserine/phosphohydroxythreonine transam	QEN71_04065	paras_001366	protein-codi	NZ_CP1252	chromosom	900272	901354	+	1083	0	15	4830	12	4479	
QEN71_RS04070	prephenate dehydratase	QEN71_04070	paras_001367	protein-codi	NZ_CP1252	chromosom	901424	902506	+	1083	0	12	4584	10	4060	
QEN71_RS04075	histidinol-phosphate transaminase	QEN71_04075	paras_001368	protein-codi	NZ_CP1252	chromosom	902580	903695	+	1116	0	15	6673	9	2218	
QEN71_RS04080	prephenate dehydrogenase/arogenate dehydrogena	QEN71_04080	paras_001369	protein-codi	NZ_CP1252	chromosom	903747	904709	+	963	0	7	2455	6	2144	
QEN71_RS04085	3-phosphoshikimate 1-carboxyvinyltransferase	QEN71_04085	paras_001370	protein-codi	NZ_CP1252	chromosom	904727	906031	+	1305	0	12	3457	9	3167	
QEN71_RS04090	(d)CMP kinase	QEN71_04090	paras_001371	protein-codi	NZ_CP1252	chromosom	906080	906766	+	687	0	0	0	0	0	TRUE
QEN71_RS04095	30S ribosomal protein S1	QEN71_04095	paras_001372	protein-codi	NZ_CP1252	chromosom	906918	908648	+	1731	0	4	275	3	13	TRUE
QEN71_RS04100	integration host factor subunit beta	QEN71_04100	paras_001373	protein-codi	NZ_CP1252	chromosom	908671	908994	+	324	0	0	0	0	0	TRUE

QEN71_RS04105	LapA family protein	QEN71_04105	paras_001374	protein-codi	NZ_CP1252	chromosom	909326	909619	+	294	0	5	626	3	181	
QEN71_RS04110	lipopolysaccharide assembly protein LapB	QEN71_04110	paras_001375	protein-codi	NZ_CP1252	chromosom	909676	910851	+	1176	0	3	277	1	7	TRUE
QEN71_RS04115	UDP-glucose/GDP-mannose dehydrogenase family	QEN71_04115	paras_001376	protein-codi	NZ_CP1252	chromosom	910942	912354	+	1369	324.0	3	561	2	4	
QEN71_RS04120	D-glycero-beta-D-manno-heptose-7-phosphate kina	QEN71_04120	paras_001377	protein-codi	NZ_CP1252	chromosom	912311	913309	+	955	0	2	19	1	5	TRUE
QEN71_RS04125	ADP-glyceromanno-heptose 6-epimerase	QEN71_04125	paras_001378	protein-codi	NZ_CP1252	chromosom	913391	914383	+	993	0	11	572	6	40	
QEN71_RS04130	helix-hairpin-helix domain-containing protein	QEN71_04130	paras_001379	protein-codi	NZ_CP1252	chromosom	914462	914830	+	369	0	8	2583	4	682	
QEN71_RS04135	cysteine synthase CysM	QEN71_04135	paras_001380	protein-codi	NZ_CP1252	chromosom	915148	916050	+	903	0	20	1895	11	1174	
QEN71_RS04140	lytic murein transglycosylase B	QEN71_04140	paras_001381	protein-codi	NZ_CP1252	chromosom	916128	917336	-	1209	0	49	15753	38	13339	
QEN71_RS04145	histone deacetylase family protein	QEN71_04145	paras_001382	protein-codi	NZ_CP1252	chromosom	917543	918466	+	924	0	22	5900	18	4807	
QEN71_RS04150	enoyl-CoA hydratase	QEN71_04150	paras_001383	protein-codi	NZ_CP1252	chromosom	918498	919307	+	810	0	15	5784	11	2689	
QEN71_RS04155	alpha/beta hydrolase	QEN71_04155	paras_001384	protein-codi	NZ_CP1252	chromosom	919314	920192	-	879	0	14	3260	8	2422	
QEN71_RS04160	methionine ABC transporter ATP-binding protein	QEN71_04160	paras_001385	protein-codi	NZ_CP1252	chromosom	920432	921466	+	1024	0	5	2560	2	151	
QEN71_RS04165	methionine ABC transporter permease	QEN71_04165	paras_001386	protein-codi	NZ_CP1252	chromosom	921456	922109	+	643	0	14	3177	7	1368	
QEN71_RS04170	MetQ/NlpA family ABC transporter substrate-binding	QEN71_04170	paras_001387	protein-codi	NZ_CP1252	chromosom	922135	922935	+	801	0	22	7767	18	5344	
QEN71_RS04175	electron transfer flavoprotein subunit beta/FixA fami	QEN71_04175	paras_001388	protein-codi	NZ_CP1252	chromosom	923185	923934	+	750	362.0	0	0	0	0	
QEN71_RS04180	electron transfer flavoprotein subunit alpha/FixB fan	QEN71_04180	paras_001389	protein-codi	NZ_CP1252	chromosom	923963	924898	+	936	541.0	2	17	1	7	
QEN71_RS04185	acyl-CoA dehydrogenase	QEN71_04185	paras_001390	protein-codi	NZ_CP1252	chromosom	925001	926791	+	1791	386.0	52	21420	42	18640	
QEN71_RS04190	D-amino acid dehydrogenase	QEN71_04190	paras_001391	protein-codi	NZ_CP1252	chromosom	926867	928153	-	1287	0	34	9062	20	4571	
QEN71_RS04195	Lrp/AsnC ligand binding domain-containing protein	QEN71_04195	paras_001392	protein-codi	NZ_CP1252	chromosom	928320	928808	+	489	0	11	6233	9	6126	
QEN71_RS04200	PA0069 family radical SAM protein	QEN71_04200	paras_001393	protein-codi	NZ_CP1252	chromosom	928920	930059	+	1140	0	46	28326	39	27340	
QEN71_RS04205	TM2 domain-containing protein	QEN71_04205	paras_001394	protein-codi	NZ_CP1252	chromosom	930074	930541	-	468	0	19	7985	12	3805	
QEN71_RS04210	30S ribosomal protein S16	QEN71_04210	paras_001395	protein-codi	NZ_CP1252	chromosom	930955	931209	+	255	0	2	864	0	0	
QEN71_RS04215	ribosome maturation factor RimM	QEN71_04215	paras_001396	protein-codi	NZ_CP1252	chromosom	931305	932000	+	696	0	3	449	1	2	
QEN71_RS04220	tRNA (guanosine(37)-N1)-methyltransferase TrmD	QEN71_04220	paras_001397	protein-codi	NZ_CP1252	chromosom	932025	932792	+	768	0	0	0	0	0	TRUE
QEN71_RS04225	50S ribosomal protein L19	QEN71_04225	paras_001398	protein-codi	NZ_CP1252	chromosom	932938	933321	+	384	0	4	416	3	177	
QEN71_RS04230	CoA pyrophosphatase	QEN71_04230	paras_001399	protein-codi	NZ_CP1252	chromosom	933415	934122	+	708	0	10	2063	6	1253	
QEN71_RS04235	CobD/CbiB family protein	QEN71_04235	paras_001400	protein-codi	NZ_CP1252	chromosom	934286	935224	+	939	0	24	6548	20	4814	
QEN71_RS04240	DUF2007 domain-containing protein	QEN71_04240	paras_001401	protein-codi	NZ_CP1252	chromosom	935249	935569	-	321	0	1	163	1	163	
QEN71_RS04245	ribosome small subunit-dependent GTPase A	QEN71_04245	paras_001402	protein-codi	NZ_CP1252	chromosom	935581	936534	-	950	0	5	30	4	28	
QEN71_RS04250	M48 family metallopeptidase	QEN71_04250	paras_001403	protein-codi	NZ_CP1252	chromosom	936531	937790	-	1256	0	24	2410	14	1258	
QEN71_RS04255	oligoribonuclease	QEN71_04255	paras_001404	protein-codi	NZ_CP1252	chromosom	937882	938505	+	624	0	2	1153	0	0	
QEN71_RS04260	molybdopterin adenylyltransferase	QEN71_04260	paras_001405	protein-codi	NZ_CP1252	chromosom	938510	939136	-	607	0	10	3416	10	3416	
QEN71_RS04265	ribosome biogenesis factor YjgA	QEN71_04265	paras_001406	protein-codi	NZ_CP1252	chromosom	939117	939728	-	592	0	13	5900	11	5536	
QEN71_RS04270	metalloprotease PmbA	QEN71_04270	paras_001407	protein-codi	NZ_CP1252	chromosom	939949	941319	+	1371	0	43	9296	32	5550	
QEN71_RS04275	dihydrofolate reductase	QEN71_04275	paras_001408	protein-codi	NZ_CP1252	chromosom	941940	942440	-	501	0	0	0	0	0	TRUE
QEN71_RS04280	sigma-54 dependent transcriptional regulator	QEN71_04280	paras_001409	protein-codi	NZ_CP1252	chromosom	942553	944082	-	1530	0	22	8233	17	7716	
QEN71_RS04285	hypothetical protein	QEN71_04285	paras_001410	protein-codi	NZ_CP1252	chromosom	944107	945573	-	1467	0	49	19480	36	15000	
QEN71_RS04290	peptidase C39	QEN71_04290	paras_001411	protein-codi	NZ_CP1252	chromosom	945685	946491	-	807	0	19	4385	11	1859	
QEN71_RS04295	C39 family peptidase	QEN71_04295	paras_001412	protein-codi	NZ_CP1252	chromosom	946498	947217	-	720	0	12	2129	11	2052	
QEN71_RS04300	hypothetical protein	QEN71_04300	paras_001413	protein-codi	NZ_CP1252	chromosom	947314	948711	-	1398	0	35	7648	32	6924	
QEN71_RS04305	hypothetical protein	QEN71_04305	paras_001414	protein-codi	NZ_CP1252	chromosom	948741	949352	-	608	0	9	909	4	698	
QEN71_RS04310	hypothetical protein	QEN71_04310	paras_001415	protein-codi	NZ_CP1252	chromosom	949349	949861	-	509	0	4	76	4	76	
QEN71_RS04315	hypothetical protein	QEN71_04315	paras_001416	protein-codi	NZ_CP1252	chromosom	949945	950427	-	483	0	11	1690	7	1185	
QEN71_RS04320	sigma-54 dependent transcriptional regulator	QEN71_04320	paras_001417	protein-codi	NZ_CP1252	chromosom	950975	952369	+	1395	0	35	12293	22	6939	
QEN71_RS04325	thymidylate synthase	QEN71_04325	paras_001418	protein-codi	NZ_CP1252	chromosom	952574	953545	+	972	0	0	0	0	0	TRUE
QEN71_RS04330	FecR domain-containing protein	QEN71_04330	paras_001419	protein-codi	NZ_CP1252	chromosom	953637	956297	-	2661	0	69	25408	58	21782	
QEN71_RS04335	helix-turn-helix transcriptional regulator	QEN71_04335	paras_001420	protein-codi	NZ_CP1252	chromosom	956450	957163	-	714	0	8	3092	7	3074	
QEN71_RS04340	hypothetical protein	QEN71_04340	paras_001421	protein-codi	NZ_CP1252	chromosom	957359	957499	+	141	0	2	972	2	972	
QEN71_RS04345	MFS transporter	QEN71_04345	paras_001422	protein-codi	NZ_CP1252	chromosom	957625	958869	+	1245	0	17	5187	15	4609	
QEN71_RS04350	helix-turn-helix domain-containing protein	QEN71_04350	paras_001423	protein-codi	NZ_CP1252	chromosom	958904	959953	+	1050	0	12	2879	11	2876	
QEN71_RS04355	acyl-CoA thioesterase	QEN71_04355	paras_001424	protein-codi	NZ_CP1252	chromosom	960088	960594	+	507	0	11	2160	11	2160	
QEN71_RS04360	class II fumarate hydratase	QEN71_04360	paras_001425	protein-codi	NZ_CP1252	chromosom	960671	962071	-	1401	0	19	5377	13	1681	
QEN71_RS04365	phospholipase D family protein	QEN71_04365	paras_001426	protein-codi	NZ_CP1252	chromosom	962155	963750	-	1596	0	45	15961	37	13427	
QEN71_RS04370	bile acid:sodium symporter family protein	QEN71_04370	paras_001427	protein-codi	NZ_CP1252	chromosom	964095	965054	-	960	0	13	6670	12	6668	
QEN71_RS04375	IS481 family transposase	QEN71_04375	paras_001428	protein-codi	NZ_CP1252	chromosom	965712	966851	-	1140	2266.0	3	22	3	22	
QEN71_RS04380	MATE family efflux transporter	QEN71_04380	paras_001429	protein-codi	NZ_CP1252	chromosom	967023	968414	-	1392	0	37	11572	32	10410	
QEN71_RS04385	RNA-binding S4 domain-containing protein	QEN71_04385	paras_001430	protein-codi	NZ_CP1252	chromosom	968634	968852	-	219	0	7	2193	5	890	
QEN71_RS04390	hypothetical protein	QEN71_04390	paras_001431	protein-codi	NZ_CP1252	chromosom	969175	969642	+	468	0	8	4304	4	2947	
QEN71_RS04395	hypothetical protein	QEN71_04395	paras_001432	protein-codi	NZ_CP1252	chromosom	969690	969953	+	264	0	2	701	0	0	
QEN71_RS04400	ATP-dependent helicase	QEN71_04400	paras_001433	protein-codi	NZ_CP1252	chromosom	970132	972336	+	2205	0	45	16618	39	14771	
QEN71_RS04405	DUF4088 family protein	QEN71_04405	paras_001434	protein-codi	NZ_CP1252	chromosom	972404	973177	-	774	0	17	4883	13	4531	
QEN71_RS04410	AzlD domain-containing protein	QEN71_04410	paras_001435	protein-codi	NZ_CP1252	chromosom	973408	973731	-	320	0	3	3897	0	0	

QEN71_RS04415	AzlC family ABC transporter permease	QEN71_04415	paras_001436	protein-codi	NZ_CP1252	chromosom	973728	974399	-		668	0	20	5998	18	4859	
QEN71_RS04420	AraC family transcriptional regulator	QEN71_04420	paras_001437	protein-codi	NZ_CP1252	chromosom	974582	975409	-		828	0	22	6846	18	5532	
QEN71_RS04425	CreA family protein	QEN71_04425	paras_001438	protein-codi	NZ_CP1252	chromosom	975448	975939	-		492	0	21	10047	19	9765	
QEN71_RS04430	tRNA-Asn	QEN71_04430		tRNA	NZ_CP1252	chromosom	976114	976189	-		76	151.0	4	206	4	206	
QEN71_RS04435	tRNA-Asn	QEN71_04435		tRNA	NZ_CP1252	chromosom	976253	976328	-		76	151.0	0	0	0	0	
QEN71_RS04440	ferredoxin family protein	QEN71_04440	paras_001441	protein-codi	NZ_CP1252	chromosom	976441	976764	-		324	0	1	8	1	8	
QEN71_RS04445	nicotinate phosphoribosyltransferase	QEN71_04445	paras_001442	protein-codi	NZ_CP1252	chromosom	977086	978285	+		1200	0	49	12365	34	8332	
QEN71_RS04450	lactate utilization protein C	QEN71_04450	paras_001443	protein-codi	NZ_CP1252	chromosom	978479	979141	+		663	0	8	282	4	191	
QEN71_RS04455	sodium:proton antiporter	QEN71_04455	paras_001444	protein-codi	NZ_CP1252	chromosom	979164	980570	+		1407	0	9	1024	4	13	
QEN71_RS04460	D-glycerate dehydrogenase	QEN71_04460	paras_001445	protein-codi	NZ_CP1252	chromosom	980625	981614	+		986	0	20	4216	20	4216	
QEN71_RS04465	DNA recombination protein RmuC	QEN71_04465	paras_001446	protein-codi	NZ_CP1252	chromosom	981611	983095	+		1481	0	25	9072	21	7822	
QEN71_RS04470	GNAT family N-acetyltransferase	QEN71_04470	paras_001447	protein-codi	NZ_CP1252	chromosom	983167	983655	-		488	0	22	7179	22	7179	
QEN71_RS04475	hypothetical protein	QEN71_04475	paras_001448	protein-codi	NZ_CP1252	chromosom	983655	983930	-		275	0	6	3413	4	1916	
QEN71_RS04480	molybdopterin-binding protein	QEN71_04480	paras_001449	protein-codi	NZ_CP1252	chromosom	984003	985298	-		1296	0	22	5307	16	3935	
QEN71_RS04485	molybdenum cofactor guanylyltransferase MobA	QEN71_04485	paras_001450	protein-codi	NZ_CP1252	chromosom	985390	986004	-		615	0	10	3564	5	1154	
QEN71_RS04490	GTP 3',8-cyclase MoaA	QEN71_04490	paras_001451	protein-codi	NZ_CP1252	chromosom	986020	987132	-		1113	0	28	12601	23	10921	
QEN71_RS04495	Rne/Rng family ribonuclease	QEN71_04495	paras_001452	protein-codi	NZ_CP1252	chromosom	987305	990598	-		3294	0	9	1246	5	1089	
QEN71_RS04500	RluA family pseudouridine synthase	QEN71_04500	paras_001453	protein-codi	NZ_CP1252	chromosom	991361	992422	+		1042	0	37	10120	29	6720	
QEN71_RS04505	HAD-IA family hydrolase	QEN71_04505	paras_001454	protein-codi	NZ_CP1252	chromosom	992403	993062	+		636	0	12	4037	11	3869	
QEN71_RS04510	Rieske 2Fe-2S domain-containing protein	QEN71_04510	paras_001455	protein-codi	NZ_CP1252	chromosom	993059	993451	+		389	0	26	9194	26	9194	
QEN71_RS04515	S49 family peptidase	QEN71_04515	paras_001456	protein-codi	NZ_CP1252	chromosom	993498	994508	+		1011	0	31	8125	28	7825	
QEN71_RS04520	SAM-dependent methyltransferase	QEN71_04520	paras_001457	protein-codi	NZ_CP1252	chromosom	994565	995284	-		716	0	21	12010	17	10786	
QEN71_RS04525	Maf-like protein	QEN71_04525	paras_001458	protein-codi	NZ_CP1252	chromosom	995281	995898	-		614	0	12	3981	12	3981	
QEN71_RS04530	DUF177 domain-containing protein	QEN71_04530	paras_001459	protein-codi	NZ_CP1252	chromosom	996125	996763	+		639	0	5	1943	5	1943	
QEN71_RS04535	50S ribosomal protein L32	QEN71_04535	paras_001460	protein-codi	NZ_CP1252	chromosom	996877	997056	+		180	0	1	104	0	0	
QEN71_RS04540	phosphate acyltransferase PlsX	QEN71_04540	paras_001461	protein-codi	NZ_CP1252	chromosom	997192	998322	+		1130	0	3	262	0	0	TRUE
QEN71_RS04545	beta-ketoacyl-ACP synthase III	QEN71_04545	paras_001462	protein-codi	NZ_CP1252	chromosom	998322	999311	+		989	0	1	2	0	0	TRUE
QEN71_RS04550	ACP S-malonyltransferase	QEN71_04550	paras_001463	protein-codi	NZ_CP1252	chromosom	999407	1000339	+		933	0	2	11	2	11	TRUE
QEN71_RS04555	3-oxoacyl-ACP reductase FabG	QEN71_04555	paras_001464	protein-codi	NZ_CP1252	chromosom	1000407	1001156	+		750	0	1	5	0	0	TRUE
QEN71_RS04560	acyl carrier protein	QEN71_04560	paras_001465	protein-codi	NZ_CP1252	chromosom	1001305	1001544	+		240	0	1	52	0	0	
QEN71_RS04565	beta-ketoacyl-ACP synthase II	QEN71_04565	paras_001466	protein-codi	NZ_CP1252	chromosom	1001897	1003135	+		1239	0	0	0	0	0	TRUE
QEN71_RS04570	hypothetical protein	QEN71_04570	paras_001467	protein-codi	NZ_CP1252	chromosom	1003140	1003625	+		486	0	6	403	6	403	
QEN71_RS04575	RNA polymerase sigma factor RpoE	QEN71_04575	paras_001468	protein-codi	NZ_CP1252	chromosom	1003758	1004357	+		600	0	24	4368	22	4028	
QEN71_RS04580	sigma-E factor negative regulatory protein	QEN71_04580	paras_001469	protein-codi	NZ_CP1252	chromosom	1004453	1005094	+		642	0	10	4820	8	4034	
QEN71_RS04585	MucB/RseB C-terminal domain-containing protein	QEN71_04585	paras_001470	protein-codi	NZ_CP1252	chromosom	1005099	1006139	+		1041	0	47	11312	31	6354	
QEN71_RS04590	DegQ family serine endoprotease	QEN71_04590	paras_001471	protein-codi	NZ_CP1252	chromosom	1006159	1007667	+		1509	0	32	9251	28	8868	
QEN71_RS04595	glutaredoxin family protein	QEN71_04595	paras_001472	protein-codi	NZ_CP1252	chromosom	1007736	1007942	+		207	0	8	2798	8	2798	
QEN71_RS04600	translation elongation factor 4	QEN71_04600	paras_001473	protein-codi	NZ_CP1252	chromosom	1008109	1009902	+		1794	0	55	6477	44	4398	
QEN71_RS04605	signal peptidase I	QEN71_04605	paras_001474	protein-codi	NZ_CP1252	chromosom	1009922	1010815	+		894	0	7	214	3	16	
QEN71_RS04610	ribonuclease III	QEN71_04610	paras_001475	protein-codi	NZ_CP1252	chromosom	1010990	1012243	+		1254	0	9	1004	5	761	
QEN71_RS04615	GTPase Era	QEN71_04615	paras_001476	protein-codi	NZ_CP1252	chromosom	1012308	1013207	+		886	0	1	72	0	0	TRUE
QEN71_RS04620	DNA repair protein RecO	QEN71_04620	paras_001477	protein-codi	NZ_CP1252	chromosom	1013194	1014216	+		1005	0	25	8887	20	7280	
QEN71_RS04625	pyridoxine 5'-phosphate synthase	QEN71_04625	paras_001478	protein-codi	NZ_CP1252	chromosom	1014213	1014977	+		761	0	2	77	1	16	TRUE
QEN71_RS04630	holo-ACP synthase	QEN71_04630	paras_001479	protein-codi	NZ_CP1252	chromosom	1015000	1015407	+		408	0	1	41	0	0	TRUE
QEN71_RS04635	beta-N-acetylhexosaminidase	QEN71_04635	paras_001480	protein-codi	NZ_CP1252	chromosom	1015474	1016505	+		1032	0	21	6052	16	4840	
QEN71_RS04640	sigma-54 dependent transcriptional regulator	QEN71_04640	paras_001481	protein-codi	NZ_CP1252	chromosom	1016593	1017975	-		1383	0	23	7218	17	5240	
QEN71_RS04645	CsbD family protein	QEN71_04645	paras_001482	protein-codi	NZ_CP1252	chromosom	1018100	1018306	-		207	0	4	652	2	395	
QEN71_RS04650	hypothetical protein	QEN71_04650	paras_001483	protein-codi	NZ_CP1252	chromosom	1018376	1018549	+		174	0	8	1259	5	663	
QEN71_RS04655	elongation factor P	QEN71_04655	paras_001484	protein-codi	NZ_CP1252	chromosom	1018595	1019152	-		558	0	5	103	4	15	
QEN71_RS04660	elongation factor P maturation arginine rhamnosyltr	QEN71_04660	paras_001485	protein-codi	NZ_CP1252	chromosom	1019504	1020685	-		1182	0	11	249	8	134	
QEN71_RS04665	excinuclease ABC subunit UvrC	QEN71_04665	paras_001486	protein-codi	NZ_CP1252	chromosom	1020795	1023005	+		2211	0	51	14382	38	11300	
QEN71_RS04670	CDP-diacylglycerol--glycerol-3-phosphate 3-phosph	QEN71_04670	paras_001487	protein-codi	NZ_CP1252	chromosom	1023114	1023704	+		591	0	3	694	0	0	
QEN71_RS04675	tRNA-Gly	QEN71_04675		tRNA	NZ_CP1252	chromosom	1023853	1023928	+		76	150.0	2	219	1	15	
QEN71_RS04680	tRNA-Gly	QEN71_04680		tRNA	NZ_CP1252	chromosom	1023995	1024070	+		76	150.0	0	0	0	0	
QEN71_RS04685	tRNA-Cys	QEN71_04685		tRNA	NZ_CP1252	chromosom	1024220	1024293	+		74	0	0	0	0	0	
QEN71_RS04690	GNAT family N-acetyltransferase	pseudo;QEN71_04690		pseudogene	NZ_CP1252	chromosom	1024482	1024965	+		484	0	24	9292	17	8252	
QEN71_RS04695	cytochrome c oxidase assembly protein	QEN71_04695	paras_001492	protein-codi	NZ_CP1252	chromosom	1024979	1025869	-		891	0	35	8764	31	8571	
QEN71_RS04700	copper chaperone PCu(A)C	QEN71_04700	paras_001493	protein-codi	NZ_CP1252	chromosom	1025905	1026384	-		480	0	15	4608	12	2677	
QEN71_RS04705	SCO family protein	QEN71_04705	paras_001494	protein-codi	NZ_CP1252	chromosom	1026402	1026980	-		579	0	15	3400	13	3319	
QEN71_RS04710	trehalose-phosphatase	QEN71_04710	paras_001495	protein-codi	NZ_CP1252	chromosom	1027407	1028156	+		750	0	12	3258	11	3216	
QEN71_RS04715	alpha,alpha-trehalose-phosphate synthase (UDP-fo	QEN71_04715	paras_001496	protein-codi	NZ_CP1252	chromosom	1028209	1029639	+		1431	0	46	17093	33	14406	
QEN71_RS04720	ABC transporter ATP-binding protein	QEN71_04720	paras_001497	protein-codi	NZ_CP1252	chromosom	1029729	1031564	-		1836	0	40	11990	28	8919	

QEN71_RS04725	glycosyltransferase family 4 protein	QEN71_04725	paras_001498	protein-codi	NZ_CP1252	chromosom	1032300	1033364	+	1065	0	31	13077	22	10303	
QEN71_RS04730	hypothetical protein	QEN71_04730	paras_001499	protein-codi	NZ_CP1252	chromosom	1033599	1034447	+	849	0	17	4834	17	4834	
QEN71_RS04735	DUF2214 family protein	QEN71_04735	paras_001500	protein-codi	NZ_CP1252	chromosom	1034527	1034985	+	459	0	7	2709	7	2709	
QEN71_RS04740	tRNA-Val	QEN71_04740		tRNA	NZ_CP1252	chromosom	1035146	1035220	-	75	0	0	0	0	0	
QEN71_RS04745	DNA polymerase III subunit epsilon	QEN71_04745	paras_001502	protein-codi	NZ_CP1252	chromosom	1035269	1036018	-	750	0	2	254	1	9	TRUE
QEN71_RS04750	ribonuclease HI	QEN71_04750	paras_001503	protein-codi	NZ_CP1252	chromosom	1036092	1036538	-	443	0	3	995	0	0	
QEN71_RS04755	class I SAM-dependent methyltransferase	QEN71_04755	paras_001504	protein-codi	NZ_CP1252	chromosom	1036535	1037350	-	812	0	7	1017	2	16	
QEN71_RS04760	hydroxyacylglutathione hydrolase	QEN71_04760	paras_001505	protein-codi	NZ_CP1252	chromosom	1037370	1038173	+	804	0	26	11519	19	7064	
QEN71_RS04765	transglycosylase SLT domain-containing protein	QEN71_04765	paras_001506	protein-codi	NZ_CP1252	chromosom	1038329	1040011	+	1683	0	62	26013	46	20771	
QEN71_RS04770	MFS transporter	QEN71_04770	paras_001507	protein-codi	NZ_CP1252	chromosom	1040249	1041493	+	1245	0	35	8749	26	5980	
QEN71_RS04775	glutamine-hydrolyzing carbamoyl-phosphate syntha	QEN71_04775	paras_001508	protein-codi	NZ_CP1252	chromosom	1041946	1043100	+	1155	0	25	2716	16	1497	
QEN71_RS04780	carbamoyl-phosphate synthase large subunit	QEN71_04780	paras_001509	protein-codi	NZ_CP1252	chromosom	1043234	1046488	+	3255	0	79	10540	56	7036	
QEN71_RS04785	transcription elongation factor GreA	QEN71_04785	paras_001510	protein-codi	NZ_CP1252	chromosom	1046640	1047116	+	477	0	23	4701	11	2084	
QEN71_RS04790	DUF4149 domain-containing protein	QEN71_04790	paras_001511	protein-codi	NZ_CP1252	chromosom	1047136	1047642	+	507	0	16	3304	15	3220	
QEN71_RS04795	YhbY family RNA-binding protein	QEN71_04795	paras_001512	protein-codi	NZ_CP1252	chromosom	1047779	1048333	-	555	0	9	1612	7	1055	
QEN71_RS04800	RlmE family RNA methyltransferase	QEN71_04800	paras_001513	protein-codi	NZ_CP1252	chromosom	1048585	1049247	+	663	0	0	0	0	0	TRUE
QEN71_RS04805	ATP-dependent zinc metalloprotease FtsH	QEN71_04805	paras_001514	protein-codi	NZ_CP1252	chromosom	1049434	1051323	+	1890	0	1	5	1	5	TRUE
QEN71_RS04810	dihydropteroate synthase	QEN71_04810	paras_001515	protein-codi	NZ_CP1252	chromosom	1051459	1052343	+	885	0	11	1342	6	23	
QEN71_RS04815	phosphoglucosamine mutase	QEN71_04815	paras_001516	protein-codi	NZ_CP1252	chromosom	1052372	1053730	+	1359	0	1	5	0	0	TRUE
QEN71_RS04820	phosphate ABC transporter substrate-binding protei	QEN71_04820	paras_001517	protein-codi	NZ_CP1252	chromosom	1054050	1055081	+	1032	0	22	1619	18	1586	
QEN71_RS04825	phosphate ABC transporter permease PstC	QEN71_04825	paras_001518	protein-codi	NZ_CP1252	chromosom	1055238	1056236	+	995	0	12	932	11	890	
QEN71_RS04830	phosphate ABC transporter permease PstA	QEN71_04830	paras_001519	protein-codi	NZ_CP1252	chromosom	1056233	1057129	+	893	0	8	107	5	52	
QEN71_RS04835	phosphate ABC transporter ATP-binding protein Ps	QEN71_04835	paras_001520	protein-codi	NZ_CP1252	chromosom	1057145	1057993	+	849	0	24	1493	22	1346	
QEN71_RS04840	phosphate signaling complex protein PhoU	QEN71_04840	paras_001521	protein-codi	NZ_CP1252	chromosom	1058029	1058733	+	705	0	3	380	1	54	
QEN71_RS04845	phosphate regulon transcriptional regulator PhoB	QEN71_04845	paras_001522	protein-codi	NZ_CP1252	chromosom	1058798	1059499	+	702	0	13	2194	9	1549	
QEN71_RS04850	phosphate regulon sensor histidine kinase PhoR	QEN71_04850	paras_001523	protein-codi	NZ_CP1252	chromosom	1059602	1060915	+	1314	0	24	1942	17	1311	
QEN71_RS04855	exopolyphosphatase	QEN71_04855	paras_001524	protein-codi	NZ_CP1252	chromosom	1061586	1063133	-	1548	0	46	6732	28	4000	
QEN71_RS04860	polyphosphate kinase 1	QEN71_04860	paras_001525	protein-codi	NZ_CP1252	chromosom	1063372	1065435	+	2064	0	58	13164	48	11986	
QEN71_RS04865	GNAT family N-acyltransferase	QEN71_04865	paras_001526	protein-codi	NZ_CP1252	chromosom	1066081	1066896	-	816	0	29	6394	28	6360	
QEN71_RS04870	histidine phosphatase family protein	QEN71_04870	paras_001527	protein-codi	NZ_CP1252	chromosom	1067062	1067520	-	459	0	0	0	0	0	TRUE
QEN71_RS04875	tRNA-Pro	QEN71_04875		tRNA	NZ_CP1252	chromosom	1067769	1067845	-	77	0	0	0	0	0	
QEN71_RS04880	DUF3309 family protein	QEN71_04880	paras_001529	protein-codi	NZ_CP1252	chromosom	1067929	1068087	-	159	0	6	1355	4	126	
QEN71_RS04885	hypothetical protein	QEN71_04885	paras_001530	protein-codi	NZ_CP1252	chromosom	1068394	1068567	-	174	0	4	1304	4	1304	
QEN71_RS04890	hypothetical protein	QEN71_04890	paras_001531	protein-codi	NZ_CP1252	chromosom	1068734	1068907	+	174	0	6	757	2	295	
QEN71_RS04895	MATE family efflux transporter	QEN71_04895	paras_001532	protein-codi	NZ_CP1252	chromosom	1068932	1070320	-	1385	0	21	4397	20	4180	
QEN71_RS04900	DUF2288 domain-containing protein	QEN71_04900	paras_001533	protein-codi	NZ_CP1252	chromosom	1070317	1070625	-	305	0	2	18	2	18	
QEN71_RS04905	hypothetical protein	QEN71_04905	paras_001534	protein-codi	NZ_CP1252	chromosom	1071302	1071631	-	330	0	11	2107	5	987	
QEN71_RS04910	peptidoglycan DD-metalloendopeptidase family prot	QEN71_04910	paras_001535	protein-codi	NZ_CP1252	chromosom	1071839	1072552	-	714	0	19	2342	17	2160	
QEN71_RS04915	aldose epimerase	QEN71_04915	paras_001536	protein-codi	NZ_CP1252	chromosom	1072705	1073622	-	916	0	15	4176	12	2804	
QEN71_RS04920	hypothetical protein	QEN71_04920	paras_001537	protein-codi	NZ_CP1252	chromosom	1073621	1073917	+	295	0	13	2066	9	1375	
QEN71_RS04925	undecaprenyl-diphosphate phosphatase	QEN71_04925	paras_001538	protein-codi	NZ_CP1252	chromosom	1074190	1075020	+	831	0	16	3175	14	2805	
QEN71_RS04930	tRNA-Arg	QEN71_04930		tRNA	NZ_CP1252	chromosom	1075157	1075231	+	75	0	0	0	0	0	
QEN71_RS04935	acid phosphatase	QEN71_04935	paras_001540	protein-codi	NZ_CP1252	chromosom	1075434	1077011	-	1578	0	41	6696	26	4172	
QEN71_RS04940	cytochrome c peroxidase	QEN71_04940	paras_001541	protein-codi	NZ_CP1252	chromosom	1077262	1078635	+	1352	0	39	7314	36	6502	
QEN71_RS04945	hypothetical protein	QEN71_04945	paras_001542	protein-codi	NZ_CP1252	chromosom	1078614	1078901	-	266	0	10	2300	5	679	
QEN71_RS04950	RT0821/Lpp0805 family surface protein	QEN71_04950	paras_001543	protein-codi	NZ_CP1252	chromosom	1079149	1079538	+	386	0	2	664	2	664	
QEN71_RS04955	NUDIX hydrolase	QEN71_04955	paras_001544	protein-codi	NZ_CP1252	chromosom	1079535	1079993	+	455	0	2	101	2	101	
QEN71_RS04960	transporter substrate-binding domain-containing pro	QEN71_04960	paras_001545	protein-codi	NZ_CP1252	chromosom	1080008	1080922	-	915	0	18	3010	14	2509	
QEN71_RS04965	glutamine--tRNA ligase/YqeY domain fusion protein	QEN71_04965	paras_001546	protein-codi	NZ_CP1252	chromosom	1081064	1082785	-	1722	0	3	223	1	5	TRUE
QEN71_RS04970	gamma-glutamyltransferase	QEN71_04970	paras_001547	protein-codi	NZ_CP1252	chromosom	1083283	1085088	+	1806	0	55	20607	41	17563	
QEN71_RS04975	alpha/beta hydrolase	QEN71_04975	paras_001548	protein-codi	NZ_CP1252	chromosom	1085141	1085692	+	552	0	8	1646	4	1247	
QEN71_RS04980	CaiB/BaiF CoA-transferase family protein	QEN71_04980	paras_001549	protein-codi	NZ_CP1252	chromosom	1085769	1086989	-	1221	0	37	7774	31	7011	
QEN71_RS04985	alanine--tRNA ligase	QEN71_04985	paras_001550	protein-codi	NZ_CP1252	chromosom	1087300	1089924	+	2625	0	1	2	0	0	TRUE
QEN71_RS04990	LysR family transcriptional regulator	QEN71_04990	paras_001551	protein-codi	NZ_CP1252	chromosom	1090045	1090950	-	906	0	23	9622	23	9622	
QEN71_RS04995	YbfB/YjiJ family MFS transporter	QEN71_04995	paras_001552	protein-codi	NZ_CP1252	chromosom	1091058	1092293	+	1236	0	21	6133	17	5660	
QEN71_RS05000	low specificity L-threonine aldolase	QEN71_05000	paras_001553	protein-codi	NZ_CP1252	chromosom	1092490	1093521	+	1032	0	13	2049	8	752	
QEN71_RS05005	NUDIX domain-containing protein	QEN71_05005	paras_001554	protein-codi	NZ_CP1252	chromosom	1093564	1094094	+	531	0	10	1071	7	825	
QEN71_RS05010	iron-containing alcohol dehydrogenase	QEN71_05010	paras_001555	protein-codi	NZ_CP1252	chromosom	1094162	1095310	+	1149	0	15	2603	6	540	
QEN71_RS05015	thioesterase family protein	QEN71_05015	paras_001556	protein-codi	NZ_CP1252	chromosom	1095345	1095803	+	459	0	21	5947	21	5947	
QEN71_RS05020	branched-chain amino acid ABC transporter permea	QEN71_05020	paras_001557	protein-codi	NZ_CP1252	chromosom	1095807	1096745	+	931	0	15	3786	10	2413	
QEN71_RS05025	branched-chain amino acid ABC transporter permea	QEN71_05025	paras_001558	protein-codi	NZ_CP1252	chromosom	1096738	1097997	+	1248	0	20	2088	18	1845	
QEN71_RS05030	ABC transporter ATP-binding protein	QEN71_05030	paras_001559	protein-codi	NZ_CP1252	chromosom	1097994	1098764	+	763	0	11	599	10	565	

QEN71_RS05035	ABC transporter ATP-binding protein	QEN71_05035	paras_001560	protein-codi	NZ_CP1252	chromosome	1098761	1099474	+	710	0	6	197	4	146	
QEN71_RS05040	hypothetical protein	QEN71_05040	paras_001561	protein-codi	NZ_CP1252	chromosome	1099694	1101850	+	2157	0	36	5270	27	3771	
QEN71_RS05045	5-deoxy-glucuronate isomerase	QEN71_05045	paras_001562	protein-codi	NZ_CP1252	chromosome	1102526	1103332	-	803	0	27	2112	23	1883	
QEN71_RS05050	myo-inosose-2 dehydratase	QEN71_05050	paras_001563	protein-codi	NZ_CP1252	chromosome	1103329	1104249	-	917	0	19	2467	14	1937	
QEN71_RS05055	3D-(3,5/4)-trihydroxycyclohexane-1,2-dione acylhyd	QEN71_05055	paras_001564	protein-codi	NZ_CP1252	chromosome	1104260	1106197	-	1934	0	33	3260	25	2699	
QEN71_RS05060	5-dehydro-2-deoxygluconokinase	QEN71_05060	paras_001565	protein-codi	NZ_CP1252	chromosome	1106194	1108227	-	2030	0	35	3617	26	2826	
QEN71_RS05065	ATP-binding cassette domain-containing protein	QEN71_05065	paras_001566	protein-codi	NZ_CP1252	chromosome	1108274	1109077	-	804	0	10	811	9	749	
QEN71_RS05070	ABC transporter permease	QEN71_05070	paras_001567	protein-codi	NZ_CP1252	chromosome	1109096	1110274	-	1179	0	15	1913	6	347	
QEN71_RS05075	sugar ABC transporter substrate-binding protein	QEN71_05075	paras_001568	protein-codi	NZ_CP1252	chromosome	1110482	1111537	-	1056	0	23	2697	18	2358	
QEN71_RS05080	MurR/RpiR family transcriptional regulator	QEN71_05080	paras_001569	protein-codi	NZ_CP1252	chromosome	1111820	1112701	+	874	0	33	5342	24	3886	
QEN71_RS05085	inositol 2-dehydrogenase	QEN71_05085	paras_001570	protein-codi	NZ_CP1252	chromosome	1112694	1113713	+	1011	0	13	2183	13	2183	
QEN71_RS05090	Gfo/Idh/MocA family oxidoreductase	QEN71_05090	paras_001571	protein-codi	NZ_CP1252	chromosome	1113713	1114735	+	1022	0	15	2439	14	2425	
QEN71_RS05095	sulfurtransferase TusA family protein	QEN71_05095	paras_001572	protein-codi	NZ_CP1252	chromosome	1114853	1115083	+	231	0	0	0	0	0	
QEN71_RS05100	UTP--glucose-1-phosphate uridylyltransferase GalU	QEN71_05100	paras_001573	protein-codi	NZ_CP1252	chromosome	1115791	1116672	-	882	0	3	18	2	6	TRUE
QEN71_RS05105	valine--tRNA ligase	QEN71_05105	paras_001574	protein-codi	NZ_CP1252	chromosome	1116745	1119618	-	2874	0	0	0	0	0	TRUE
QEN71_RS05110	3'-5' exonuclease	QEN71_05110	paras_001575	protein-codi	NZ_CP1252	chromosome	1119812	1122202	+	2387	0	2	34	0	0	TRUE
QEN71_RS05115	5'-methylthioadenosine/adenosylhomocysteine nucl	QEN71_05115	paras_001576	protein-codi	NZ_CP1252	chromosome	1122199	1122987	+	785	0	15	2131	8	1547	
QEN71_RS05120	propionate--CoA ligase	QEN71_05120	paras_001577	protein-codi	NZ_CP1252	chromosome	1122992	1124899	-	1908	0	51	18366	50	18362	
QEN71_RS05125	MFS transporter	QEN71_05125	paras_001578	protein-codi	NZ_CP1252	chromosome	1125685	1126938	-	1254	0	40	9210	31	6128	
QEN71_RS05130	methyl-accepting chemotaxis protein	QEN71_05130	paras_001579	protein-codi	NZ_CP1252	chromosome	1127392	1129053	+	1662	0	22	5883	15	4591	
QEN71_RS05135	HlyD family secretion protein	QEN71_05135	paras_001580	protein-codi	NZ_CP1252	chromosome	1129701	1130561	-	861	0	44	6067	37	5203	
QEN71_RS05140	DUF1656 domain-containing protein	QEN71_05140	paras_001581	protein-codi	NZ_CP1252	chromosome	1130575	1130775	-	190	0	6	557	4	509	
QEN71_RS05145	FUSC family protein	QEN71_05145	paras_001582	protein-codi	NZ_CP1252	chromosome	1130765	1132993	-	2218	0	32	5673	28	4149	
QEN71_RS05150	efflux transporter outer membrane subunit	QEN71_05150	paras_001583	protein-codi	NZ_CP1252	chromosome	1133008	1134564	-	1557	0	36	8991	32	8475	
QEN71_RS05155	LysR family transcriptional regulator	QEN71_05155	paras_001584	protein-codi	NZ_CP1252	chromosome	1134727	1135665	+	939	0	38	10337	30	8766	
QEN71_RS05160	hypothetical protein	QEN71_05160	paras_001585	protein-codi	NZ_CP1252	chromosome	1135836	1135997	+	162	0	2	330	0	0	
QEN71_RS05165	metallophosphoesterase	QEN71_05165	paras_001586	protein-codi	NZ_CP1252	chromosome	1136015	1136845	-	831	0	25	5898	20	4561	
QEN71_RS05170	FAD-binding oxidoreductase	QEN71_05170	paras_001587	protein-codi	NZ_CP1252	chromosome	1136884	1138311	-	1428	0	32	5343	18	2254	
QEN71_RS05175	DUF2069 domain-containing protein	QEN71_05175	paras_001588	protein-codi	NZ_CP1252	chromosome	1138423	1138854	-	432	0	11	3495	11	3495	
QEN71_RS05180	NAD(P)H:quinone oxidoreductase	QEN71_05180	paras_001589	protein-codi	NZ_CP1252	chromosome	1138857	1139459	-	603	0	13	2645	8	861	
QEN71_RS05185	YihY family inner membrane protein	QEN71_05185	paras_001590	protein-codi	NZ_CP1252	chromosome	1139656	1140972	+	1317	0	37	11101	32	9618	
QEN71_RS05190	DUF962 domain-containing protein	QEN71_05190	paras_001591	protein-codi	NZ_CP1252	chromosome	1140994	1141317	-	324	0	14	4479	13	4450	
QEN71_RS05195	alpha/beta hydrolase	QEN71_05195	paras_001592	protein-codi	NZ_CP1252	chromosome	1141459	1142304	-	842	0	16	2765	6	325	
QEN71_RS05200	O-acetylhomoserine aminocarboxypropyltransferase	QEN71_05200	paras_001593	protein-codi	NZ_CP1252	chromosome	1142301	1143638	-	1334	0	20	5421	18	4752	
QEN71_RS05205	CBS domain-containing protein	QEN71_05205	paras_001594	protein-codi	NZ_CP1252	chromosome	1144051	1144518	+	468	0	10	2347	10	2347	
QEN71_RS05210	MFS transporter	QEN71_05210	paras_001595	protein-codi	NZ_CP1252	chromosome	1144663	1146600	+	1938	0	50	15130	48	14230	
QEN71_RS05215	chorismate synthase	QEN71_05215	paras_001596	protein-codi	NZ_CP1252	chromosome	1146743	1147843	+	1101	0	28	4449	22	3960	
QEN71_RS05220	tetratricopeptide repeat protein	QEN71_05220	paras_001597	protein-codi	NZ_CP1252	chromosome	1147890	1149353	+	1464	0	20	4677	14	2826	
QEN71_RS05225	electron transfer flavoprotein-ubiquinone oxidoredu	QEN71_05225	paras_001598	protein-codi	NZ_CP1252	chromosome	1149435	1151108	-	1674	1680.0	4	25	4	25	
QEN71_RS05230	SDR family oxidoreductase	QEN71_05230	paras_001599	protein-codi	NZ_CP1252	chromosome	1151294	1152070	+	777	0	18	4536	16	4385	
QEN71_RS05235	thioesterase family protein	QEN71_05235	paras_001600	protein-codi	NZ_CP1252	chromosome	1152270	1152698	+	429	0	16	4511	13	3121	
QEN71_RS05240	PAS and helix-turn-helix domain-containing protein	QEN71_05240	paras_001601	protein-codi	NZ_CP1252	chromosome	1152772	1153317	-	546	0	11	2640	7	1982	
QEN71_RS05245	CoA transferase subunit A	QEN71_05245	paras_001602	protein-codi	NZ_CP1252	chromosome	1153595	1154299	+	705	0	14	3943	6	2341	
QEN71_RS05250	CoA transferase subunit B	QEN71_05250	paras_001603	protein-codi	NZ_CP1252	chromosome	1154302	1154943	+	642	0	7	2865	2	177	
QEN71_RS05255	alpha/beta hydrolase	QEN71_05255	paras_001604	protein-codi	NZ_CP1252	chromosome	1155131	1156063	+	933	0	25	9418	18	5414	
QEN71_RS05260	RidA family protein	QEN71_05260	paras_001605	protein-codi	NZ_CP1252	chromosome	1156168	1156521	+	354	0	10	5673	8	4320	
QEN71_RS05265	bifunctional (p)ppGpp synthetase/guanosine-3',5'-bi	QEN71_05265	paras_001606	protein-codi	NZ_CP1252	chromosome	1156589	1158829	+	2241	0	57	19597	53	18311	
QEN71_RS05270	tRNA-Val	QEN71_05270		tRNA	NZ_CP1252	chromosome	1158917	1158993	+	77	152.0	1	129	0	0	
QEN71_RS05275	threonine--tRNA ligase	QEN71_05275	paras_001608	protein-codi	NZ_CP1252	chromosome	1159276	1161183	+	1908	0	1	312	0	0	TRUE
QEN71_RS05280	translation initiation factor IF-3	QEN71_05280	paras_001609	protein-codi	NZ_CP1252	chromosome	1161234	1161758	+	525	0	1	1367	0	0	
QEN71_RS05285	50S ribosomal protein L35	QEN71_05285	paras_001610	protein-codi	NZ_CP1252	chromosome	1162016	1162213	+	198	0	0	0	0	0	
QEN71_RS05290	50S ribosomal protein L20	QEN71_05290	paras_001611	protein-codi	NZ_CP1252	chromosome	1162243	1162602	+	360	0	0	0	0	0	TRUE
QEN71_RS05295	phenylalanine--tRNA ligase subunit alpha	QEN71_05295	paras_001612	protein-codi	NZ_CP1252	chromosome	1162761	1163774	+	1014	0	1	12	1	12	TRUE
QEN71_RS05300	phenylalanine--tRNA ligase subunit beta	QEN71_05300	paras_001613	protein-codi	NZ_CP1252	chromosome	1163934	1166366	+	2433	0	0	0	0	0	TRUE
QEN71_RS05305	integration host factor subunit alpha	QEN71_05305	paras_001614	protein-codi	NZ_CP1252	chromosome	1166421	1166831	+	411	0	1	187	0	0	TRUE
QEN71_RS05310	MerR family transcriptional regulator	QEN71_05310	paras_001615	protein-codi	NZ_CP1252	chromosome	1166886	1167284	+	399	0	16	3041	16	3041	
QEN71_RS05315	tRNA-Pro	QEN71_05315		tRNA	NZ_CP1252	chromosome	1167356	1167432	+	77	0	3	195	2	158	
QEN71_RS05320	DUF3303 domain-containing protein	QEN71_05320	paras_001617	protein-codi	NZ_CP1252	chromosome	1167583	1167867	-	285	0	4	2266	2	613	
QEN71_RS05325	excinuclease ABC subunit UvrA	QEN71_05325	paras_001618	protein-codi	NZ_CP1252	chromosome	1168653	1174562	+	5910	0	151	30692	118	23495	
QEN71_RS05330	DMT family transporter	QEN71_05330	paras_001619	protein-codi	NZ_CP1252	chromosome	1174589	1175653	+	1065	0	11	2764	10	2394	
QEN71_RS05335	glutathione binding-like protein	QEN71_05335	paras_001620	protein-codi	NZ_CP1252	chromosome	1175759	1176463	+	705	0	19	3452	17	3393	
QEN71_RS05340	phospholipase C, phosphocholine-specific	QEN71_05340	paras_001621	protein-codi	NZ_CP1252	chromosome	1176555	1178675	-	2121	0	64	12076	51	9813	

QEN71_RS05345	alpha/beta hydrolase	QEN71_05345	paras_001622	protein-codi	NZ_CP1252	chromosom	1179343	1180197	-	855	0	12	2070	10	1766	
QEN71_RS05350	benzoate-CoA ligase family protein	QEN71_05350	paras_001623	protein-codi	NZ_CP1252	chromosom	1180206	1181804	-	1599	0	48	11160	39	7629	
QEN71_RS05355	DUF4863 family protein	QEN71_05355	paras_001624	protein-codi	NZ_CP1252	chromosom	1181891	1182370	-	480	0	7	1186	7	1186	
QEN71_RS05360	helix-turn-helix transcriptional regulator	QEN71_05360	paras_001625	protein-codi	NZ_CP1252	chromosom	1182614	1183567	+	954	0	12	1111	7	690	
QEN71_RS05365	2,3-epoxybenzoyl-CoA dihydrolase	QEN71_05365	paras_001626	protein-codi	NZ_CP1252	chromosom	1183713	1185383	+	1671	0	31	5853	21	4520	
QEN71_RS05370	benzoyl-CoA 2,3-epoxidase subunit BoxB	QEN71_05370	paras_001627	protein-codi	NZ_CP1252	chromosom	1185468	1186895	+	1428	0	32	3237	25	2837	
QEN71_RS05375	benzoyl-CoA 2,3-epoxidase subunit BoxA	QEN71_05375	paras_001628	protein-codi	NZ_CP1252	chromosom	1186930	1188180	+	1251	0	25	3678	22	3353	
QEN71_RS05380	TetR/AcrR family transcriptional regulator	QEN71_05380	paras_001629	protein-codi	NZ_CP1252	chromosom	1188206	1188910	-	705	0	12	1009	11	1005	
QEN71_RS05385	Zn-dependent hydrolase	QEN71_05385	paras_001630	protein-codi	NZ_CP1252	chromosom	1189307	1190584	+	1278	0	17	1444	13	1174	
QEN71_RS05390	NAD(P)-dependent oxidoreductase	QEN71_05390	paras_001631	protein-codi	NZ_CP1252	chromosom	1190700	1192049	+	1350	0	20	3193	16	2925	
QEN71_RS05395	NAD-dependent dihydropyrimidine dehydrogenase	QEN71_05395	paras_001632	protein-codi	NZ_CP1252	chromosom	1192113	1193423	+	1311	0	17	1553	13	879	
QEN71_RS05400	NCS1 family nucleobase:cation symporter-1	QEN71_05400	paras_001633	protein-codi	NZ_CP1252	chromosom	1193469	1194974	+	1506	0	36	4069	25	2479	
QEN71_RS05405	dihydropyrimidinase	QEN71_05405	paras_001634	protein-codi	NZ_CP1252	chromosom	1195009	1196460	+	1452	0	38	3494	24	1685	
QEN71_RS05410	amino acid ABC transporter substrate-binding prote	QEN71_05410	paras_001635	protein-codi	NZ_CP1252	chromosom	1197239	1198021	+	783	0	20	2352	11	1234	
QEN71_RS05415	amino acid ABC transporter permease	QEN71_05415	paras_001636	protein-codi	NZ_CP1252	chromosom	1198044	1198721	+	678	0	23	4594	21	4196	
QEN71_RS05420	amino acid ABC transporter ATP-binding protein	QEN71_05420	paras_001637	protein-codi	NZ_CP1252	chromosom	1198725	1199492	+	768	0	4	392	3	120	
QEN71_RS05425	hypothetical protein	QEN71_05425	paras_001638	protein-codi	NZ_CP1252	chromosom	1199624	1200070	+	447	0	5	892	4	888	
QEN71_RS05430	hypothetical protein	QEN71_05430	paras_001639	protein-codi	NZ_CP1252	chromosom	1200200	1200430	+	231	0	6	684	4	526	
QEN71_RS05435	YadA-like family protein	QEN71_05435	paras_001640	protein-codi	NZ_CP1252	chromosom	1200761	1202758	+	1998	0	40	4138	25	2853	
QEN71_RS05440	DUF3443 domain-containing protein	QEN71_05440	paras_001641	protein-codi	NZ_CP1252	chromosom	1202853	1204175	-	1323	0	35	6866	25	5742	
QEN71_RS05445	DUF2844 domain-containing protein	QEN71_05445	paras_001642	protein-codi	NZ_CP1252	chromosom	1204192	1204671	-	480	0	11	3707	11	3707	
QEN71_RS05450	outer membrane protein assembly factor BamC	QEN71_05450	paras_001643	protein-codi	NZ_CP1252	chromosom	1205170	1206399	+	1230	0	12	676	12	676	
QEN71_RS05455	hypothetical protein	QEN71_05455	paras_001644	protein-codi	NZ_CP1252	chromosom	1206579	1206728	+	150	0	4	766	1	210	
QEN71_RS05460	DMT family transporter	QEN71_05460	paras_001645	protein-codi	NZ_CP1252	chromosom	1206835	1207767	-	933	0	13	2573	12	2559	
QEN71_RS05465	L-lactate permease	QEN71_05465	paras_001646	protein-codi	NZ_CP1252	chromosom	1207968	1209566	-	1599	0	39	9423	33	8445	
QEN71_RS05470	hypothetical protein	QEN71_05470	paras_001647	protein-codi	NZ_CP1252	chromosom	1209862	1210449	-	588	0	52	8341	52	8341	
QEN71_RS05475	glycine zipper 2TM domain-containing protein	QEN71_05475	paras_001648	protein-codi	NZ_CP1252	chromosom	1210805	1211017	-	213	0	2	1178	0	0	
QEN71_RS05480	phosphomethylpyrimidine synthase ThiC	QEN71_05480	paras_001649	protein-codi	NZ_CP1252	chromosom	1211303	1213234	-	1932	0	42	9489	31	5029	
QEN71_RS05485	EamA family transporter	QEN71_05485	paras_001651	protein-codi	NZ_CP1252	chromosom	1214081	1215001	+	921	0	19	6110	13	4940	
QEN71_RS05490	NADPH-dependent 2,4-dienoyl-CoA reductase	QEN71_05490	paras_001652	protein-codi	NZ_CP1252	chromosom	1215030	1217078	-	2049	0	39	6623	24	4977	
QEN71_RS05495	DUF4397 domain-containing protein	QEN71_05495	paras_001653	protein-codi	NZ_CP1252	chromosom	1217343	1218131	+	789	0	17	7678	15	6555	
QEN71_RS05500	entericidin A/B family lipoprotein	QEN71_05500	paras_001654	protein-codi	NZ_CP1252	chromosom	1218338	1218475	-	138	0	0	0	0	0	
QEN71_RS05505	sensor histidine kinase	QEN71_05505	paras_001655	protein-codi	NZ_CP1252	chromosom	1218724	1220229	-	1506	0	13	3818	11	3573	
QEN71_RS05510	DUF1328 family protein	QEN71_05510	paras_001656	protein-codi	NZ_CP1252	chromosom	1220467	1220643	+	177	0	5	610	2	285	
QEN71_RS05515	DUF1328 family protein	QEN71_05515	paras_001657	protein-codi	NZ_CP1252	chromosom	1220741	1220902	+	162	0	8	2211	4	1407	
QEN71_RS05520	ferritin-like domain-containing protein	QEN71_05520	paras_001658	protein-codi	NZ_CP1252	chromosom	1221002	1221547	+	546	0	18	3644	16	3513	
QEN71_RS05525	response regulator transcription factor	QEN71_05525	paras_001659	protein-codi	NZ_CP1252	chromosom	1221653	1222297	+	645	0	16	2340	10	1273	
QEN71_RS05530	response regulator	QEN71_05530	paras_001660	protein-codi	NZ_CP1252	chromosom	1222329	1222757	+	429	0	12	4010	8	2513	
QEN71_RS05535	MFS transporter	QEN71_05535	paras_001661	protein-codi	NZ_CP1252	chromosom	1222773	1223981	-	1209	0	15	3912	10	1735	
QEN71_RS05540	phosphodiesterase	QEN71_05540	paras_001662	protein-codi	NZ_CP1252	chromosom	1224103	1224930	-	828	0	28	4757	20	3086	
QEN71_RS05545	ABC transporter ATP-binding protein	QEN71_05545	paras_001663	protein-codi	NZ_CP1252	chromosom	1225591	1226643	-	1049	0	35	3726	32	3491	
QEN71_RS05550	ABC transporter permease	QEN71_05550	paras_001664	protein-codi	NZ_CP1252	chromosom	1226640	1227557	-	914	0	26	3911	26	3911	
QEN71_RS05555	ABC transporter permease	QEN71_05555	paras_001665	protein-codi	NZ_CP1252	chromosom	1227583	1228407	-	821	0	19	3283	12	1349	
QEN71_RS05560	ABC transporter substrate-binding protein	QEN71_05560	paras_001666	protein-codi	NZ_CP1252	chromosom	1228404	1229450	-	1043	0	24	3861	21	3161	
QEN71_RS05565	LacI family DNA-binding transcriptional regulator	QEN71_05565	paras_001667	protein-codi	NZ_CP1252	chromosom	1229510	1230583	-	1074	0	12	1983	12	1983	
QEN71_RS05570	glutathione S-transferase	QEN71_05570	paras_001668	protein-codi	NZ_CP1252	chromosom	1231689	1232309	+	621	0	17	4846	15	4015	
QEN71_RS05575	hypothetical protein	QEN71_05575	paras_001669	protein-codi	NZ_CP1252	chromosom	1232573	1232764	+	192	0	4	812	2	581	
QEN71_RS05580	FAD-containing oxidoreductase	QEN71_05580	paras_001670	protein-codi	NZ_CP1252	chromosom	1232892	1234295	+	1404	0	31	7025	25	6713	
QEN71_RS05585	cupin domain-containing protein	QEN71_05585	paras_001671	protein-codi	NZ_CP1252	chromosom	1234305	1234646	+	342	0	8	1621	6	1360	
QEN71_RS05590	TMEM175 family protein	QEN71_05590	paras_001672	protein-codi	NZ_CP1252	chromosom	1234777	1235352	+	576	0	23	6641	21	6453	
QEN71_RS05595	hypothetical protein	QEN71_05595	paras_001673	protein-codi	NZ_CP1252	chromosom	1235356	1236144	+	789	0	3	276	3	276	
QEN71_RS05600	metallophosphoesterase family protein	QEN71_05600	paras_001674	protein-codi	NZ_CP1252	chromosom	1236164	1236640	+	477	0	7	1207	6	1140	
QEN71_RS05605	glycine betaine/L-proline transporter ProP	QEN71_05605	paras_001675	protein-codi	NZ_CP1252	chromosom	1236695	1238164	-	1470	423.0	49	26836	38	20762	
QEN71_RS05610	hypothetical protein	QEN71_05610	paras_001676	protein-codi	NZ_CP1252	chromosom	1238437	1238673	+	237	0	5	309	5	309	
QEN71_RS05615	oxidative damage protection protein	QEN71_05615	paras_001677	protein-codi	NZ_CP1252	chromosom	1238911	1239186	-	276	0	6	485	4	306	
QEN71_RS05620	amino-acid N-acetyltransferase	QEN71_05620	paras_001678	protein-codi	NZ_CP1252	chromosom	1239242	1240621	-	1380	0	14	138	8	56	
QEN71_RS05625	ATP-dependent RNA helicase HrpA	QEN71_05625	paras_001679	protein-codi	NZ_CP1252	chromosom	1240645	1244952	+	4308	0	77	17117	57	13444	
QEN71_RS05630	YadA-like family protein	QEN71_05630	paras_001680	protein-codi	NZ_CP1252	chromosom	1245032	1246336	-	1305	0	28	7812	19	6088	
QEN71_RS05635	beta-propeller fold lactonase family protein	QEN71_05635	paras_001681	protein-codi	NZ_CP1252	chromosom	1246892	1247905	+	1014	0	23	9172	19	8134	
QEN71_RS05640	sterol desaturase family protein	QEN71_05640	paras_001682	protein-codi	NZ_CP1252	chromosom	1247959	1248954	+	996	0	36	8694	27	6822	
QEN71_RS05645	EI24 domain-containing protein	QEN71_05645	paras_001683	protein-codi	NZ_CP1252	chromosom	1249108	1249986	+	879	0	22	8138	19	7722	
QEN71_RS05650	molybdopterin-binding protein	QEN71_05650	paras_001684	protein-codi	NZ_CP1252	chromosom	1249999	1250835	+	837	0	21	9463	17	6436	

QEN71_RS05655	rhodanese-like domain-containing protein	QEN71_05655	paras_001685	protein-codi	NZ_CP1252	chromosom	1250992	1251456	-	465	0	7	2046	2	113	
QEN71_RS05660	hypothetical protein	QEN71_05660	paras_001686	protein-codi	NZ_CP1252	chromosom	1251573	1251920	+	340	0	8	1070	6	903	
QEN71_RS05665	type I glutamate--ammonia ligase	QEN71_05665	paras_001687	protein-codi	NZ_CP1252	chromosom	1251913	1253328	+	1408	0	2	315	0	0	TRUE
QEN71_RS05670	nitrogen regulation protein NR(II)	QEN71_05670	paras_001688	protein-codi	NZ_CP1252	chromosom	1253494	1254636	+	1143	0	18	3938	16	3596	
QEN71_RS05675	nitrogen regulation protein NR(I)	QEN71_05675	paras_001689	protein-codi	NZ_CP1252	chromosom	1254708	1256222	+	1515	0	32	6617	20	4328	
QEN71_RS05680	exodeoxyribonuclease III	QEN71_05680	paras_001690	protein-codi	NZ_CP1252	chromosom	1256307	1257083	-	777	0	21	3346	15	2332	
QEN71_RS05685	tRNA-Met	QEN71_05685		tRNA	NZ_CP1252	chromosom	1257254	1257330	-	77	0	1	77	1	77	
QEN71_RS05690	amidohydrolase family protein	QEN71_05690	paras_001692	protein-codi	NZ_CP1252	chromosom	1257506	1258795	+	1290	0	43	14127	39	13807	
QEN71_RS05695	M3 family metallopeptidase	QEN71_05695	paras_001693	protein-codi	NZ_CP1252	chromosom	1258920	1261034	-	2115	0	43	10679	42	10655	
QEN71_RS05700	bifunctional methylenetetrahydrofolate dehydrogena	QEN71_05700	paras_001694	protein-codi	NZ_CP1252	chromosom	1261439	1262296	-	858	0	0	0	0	0	TRUE
QEN71_RS05705	DUF2950 domain-containing protein	QEN71_05705	paras_001695	protein-codi	NZ_CP1252	chromosom	1262603	1263517	-	915	0	33	11022	31	10874	
QEN71_RS05710	DUF3300 domain-containing protein	QEN71_05710	paras_001696	protein-codi	NZ_CP1252	chromosom	1263598	1264926	-	1329	0	28	6872	27	6799	
QEN71_RS05715	oxygen response regulator transcription factor FixJ	QEN71_05715	paras_001697	protein-codi	NZ_CP1252	chromosom	1265316	1265960	-	641	0	6	817	0	0	
QEN71_RS05720	oxygen sensor histidine kinase FixL	pseudo;QEN71_05720		pseudogene	NZ_CP1252	chromosom	1265957	1268474	-	2514	0	59	13582	49	11435	
QEN71_RS05725	pyruvate dehydrogenase (acetyl-transferring), homo	QEN71_05725	paras_001699	protein-codi	NZ_CP1252	chromosom	1268752	1271451	+	2700	0	30	298	23	256	
QEN71_RS05730	dihydrolipoyllysine-residue acetyltransferase	QEN71_05730	paras_001700	protein-codi	NZ_CP1252	chromosom	1271535	1273184	+	1650	0	12	78	7	41	
QEN71_RS05735	dihydrolipoyl dehydrogenase	QEN71_05735	paras_001701	protein-codi	NZ_CP1252	chromosom	1273450	1275507	+	2058	113.0	32	4350	23	2871	
QEN71_RS05740	phasin family protein	QEN71_05740	paras_001702	protein-codi	NZ_CP1252	chromosom	1275871	1276455	-	585	0	11	1813	7	1707	
QEN71_RS05745	D-alanyl-D-alanine endopeptidase	QEN71_05745	paras_001703	protein-codi	NZ_CP1252	chromosom	1277228	1278391	+	1164	0	36	12374	28	9889	
QEN71_RS05750	IclR family transcriptional regulator	QEN71_05750	paras_001704	protein-codi	NZ_CP1252	chromosom	1278488	1279294	-	807	0	24	7868	19	6743	
QEN71_RS05755	(Fe-S)-binding protein	QEN71_05755	paras_001705	protein-codi	NZ_CP1252	chromosom	1279484	1280206	+	723	0	24	11630	24	11630	
QEN71_RS05760	lactate utilization protein B	QEN71_05760	paras_001706	protein-codi	NZ_CP1252	chromosom	1280264	1281676	+	1413	0	46	28920	38	27540	
QEN71_RS05765	hypothetical protein	QEN71_05765	paras_001707	protein-codi	NZ_CP1252	chromosom	1281791	1282306	-	516	0	30	7734	26	5852	
QEN71_RS05770	hypothetical protein	QEN71_05770	paras_001708	protein-codi	NZ_CP1252	chromosom	1282577	1282813	+	237	0	13	5099	6	2521	
QEN71_RS05775	low molecular weight protein-tyrosine-phosphatase	QEN71_05775	paras_001709	protein-codi	NZ_CP1252	chromosom	1282917	1283444	+	528	0	8	2752	6	1794	
QEN71_RS05780	Fe-S cluster assembly transcriptional regulator IscR	QEN71_05780	paras_001710	protein-codi	NZ_CP1252	chromosom	1283555	1284079	+	525	0	4	705	3	697	
QEN71_RS05785	IscS subfamily cysteine desulfurase	QEN71_05785	paras_001711	protein-codi	NZ_CP1252	chromosom	1284149	1285372	+	1224	0	2	4	2	4	TRUE
QEN71_RS05790	Fe-S cluster assembly scaffold IscU	QEN71_05790	paras_001712	protein-codi	NZ_CP1252	chromosom	1285430	1285834	+	405	0	0	0	0	0	TRUE
QEN71_RS05795	iron-sulfur cluster assembly protein IscA	QEN71_05795	paras_001713	protein-codi	NZ_CP1252	chromosom	1285984	1286307	+	324	0	1	278	0	0	
QEN71_RS05800	Fe-S protein assembly co-chaperone HscB	QEN71_05800	paras_001714	protein-codi	NZ_CP1252	chromosom	1286441	1286968	+	528	0	1	2	1	2	TRUE
QEN71_RS05805	Fe-S protein assembly chaperone HscA	QEN71_05805	paras_001715	protein-codi	NZ_CP1252	chromosom	1287052	1288923	+	1872	0	2	301	1	27	TRUE
QEN71_RS05810	ISC system 2Fe-2S type ferredoxin	QEN71_05810	paras_001716	protein-codi	NZ_CP1252	chromosom	1289021	1289362	+	342	0	4	316	0	0	
QEN71_RS05815	Fe-S cluster assembly protein IscX	QEN71_05815	paras_001717	protein-codi	NZ_CP1252	chromosom	1289378	1289575	+	198	0	2	293	0	0	
QEN71_RS05820	glycine zipper 2TM domain-containing protein	QEN71_05820	paras_001718	protein-codi	NZ_CP1252	chromosom	1289657	1290412	-	756	0	22	4939	18	4480	
QEN71_RS05825	lysine--tRNA ligase	QEN71_05825	paras_001719	protein-codi	NZ_CP1252	chromosom	1290771	1292306	-	1536	0	0	0	0	0	TRUE
QEN71_RS05830	peptide chain release factor 2	QEN71_05830	paras_001720	pseudogene	NZ_CP1252	chromosom	1292409	1293513	-	1105	0	3	870	1	11	
QEN71_RS05835	single-stranded-DNA-specific exonuclease RecJ	QEN71_05835	paras_001721	protein-codi	NZ_CP1252	chromosom	1294219	1295925	-	1707	0	22	6690	14	5107	
QEN71_RS05840	regulator	QEN71_05840	paras_001722	protein-codi	NZ_CP1252	chromosom	1295954	1297048	-	1095	0	12	1190	6	521	
QEN71_RS05845	lipoprotein-releasing ABC transporter permease sub	QEN71_05845	paras_001723	protein-codi	NZ_CP1252	chromosom	1297355	1298608	+	1246	0	1	69	0	0	TRUE
QEN71_RS05850	lipoprotein-releasing ABC transporter ATP-binding	QEN71_05850	paras_001724	protein-codi	NZ_CP1252	chromosom	1298601	1299344	+	736	0	2	203	0	0	TRUE
QEN71_RS05855	TatD family hydrolase	QEN71_05855	paras_001725	protein-codi	NZ_CP1252	chromosom	1299381	1300169	+	789	0	23	4261	14	3251	
QEN71_RS05860	DNA internalization-related competence protein Co	QEN71_05860	paras_001726	protein-codi	NZ_CP1252	chromosom	1300270	1302780	+	2511	0	48	10009	29	6947	
QEN71_RS05865	alpha/beta hydrolase	QEN71_05865	paras_001727	protein-codi	NZ_CP1252	chromosom	1302832	1303626	+	795	0	19	3749	16	3208	
QEN71_RS05870	CTP synthase	QEN71_05870	paras_001728	protein-codi	NZ_CP1252	chromosom	1303774	1305435	+	1658	0	2	4	1	2	TRUE
QEN71_RS05875	3-deoxy-8-phosphooctulonate synthase	QEN71_05875	paras_001729	protein-codi	NZ_CP1252	chromosom	1305432	1306286	+	851	0	0	0	0	0	TRUE
QEN71_RS05880	phosphopyruvate hydratase	QEN71_05880	paras_001730	protein-codi	NZ_CP1252	chromosom	1306426	1307709	+	1284	329.0	24	1255	11	163	
QEN71_RS05885	cell division protein FtsB	QEN71_05885	paras_001731	protein-codi	NZ_CP1252	chromosom	1307874	1308302	+	429	0	2	1022	2	1022	
QEN71_RS05890	hypothetical protein	QEN71_05890	paras_001732	protein-codi	NZ_CP1252	chromosom	1308438	1308821	-	384	0	10	1017	8	943	
QEN71_RS05895	Hsp33 family molecular chaperone HslO	QEN71_05895	paras_001733	protein-codi	NZ_CP1252	chromosom	1308945	1309895	-	951	0	21	9393	17	6738	
QEN71_RS05900	gamma carbonic anhydrase family protein	QEN71_05900	paras_001734	protein-codi	NZ_CP1252	chromosom	1309959	1310483	-	525	0	23	10617	14	6027	
QEN71_RS05905	ferritin-like domain-containing protein	QEN71_05905	paras_001735	protein-codi	NZ_CP1252	chromosom	1310689	1311543	+	855	0	21	6802	19	6183	
QEN71_RS05910	alpha/beta hydrolase	QEN71_05910	paras_001736	protein-codi	NZ_CP1252	chromosom	1311625	1312521	+	897	0	16	2944	12	1568	
QEN71_RS05915	3',5'-nucleoside bisphosphate phosphatase	QEN71_05915	paras_001737	protein-codi	NZ_CP1252	chromosom	1312610	1313440	+	831	0	18	2462	15	1548	
QEN71_RS05920	L-threonylcarbamoyladenylate synthase	QEN71_05920	paras_001738	protein-codi	NZ_CP1252	chromosom	1313516	1314151	+	636	0	10	4008	5	1901	
QEN71_RS05925	site-2 protease family protein	QEN71_05925	paras_001739	protein-codi	NZ_CP1252	chromosom	1314210	1314872	+	663	0	9	2918	3	1944	
QEN71_RS05930	tryptophan--tRNA ligase	QEN71_05930	paras_001740	protein-codi	NZ_CP1252	chromosom	1314882	1316084	+	1202	0	4	1267	1	6	
QEN71_RS05935	class I SAM-dependent methyltransferase	QEN71_05935	paras_001741	protein-codi	NZ_CP1252	chromosom	1316084	1316701	+	617	0	15	6717	9	6163	
QEN71_RS05940	4-hydroxy-tetrahydrodipicolinate synthase	QEN71_05940	paras_001742	protein-codi	NZ_CP1252	chromosom	1316744	1317652	+	909	0	5	48	5	48	
QEN71_RS05945	outer membrane protein assembly factor BamC	QEN71_05945	paras_001743	protein-codi	NZ_CP1252	chromosom	1317759	1318907	+	1149	0	21	8406	15	5323	
QEN71_RS05950	MBL fold metallo-hydrolase	QEN71_05950	paras_001744	protein-codi	NZ_CP1252	chromosom	1318916	1319689	+	774	0	9	2955	9	2955	
QEN71_RS05955	Bax inhibitor-1/YccA family protein	QEN71_05955	paras_001745	protein-codi	NZ_CP1252	chromosom	1319792	1320490	-	699	0	21	6274	11	3417	
QEN71_RS05960	nucleoside-diphosphate kinase	QEN71_05960	paras_001746	protein-codi	NZ_CP1252	chromosom	1320816	1321241	+	426	0	1	252	0	0	

QEN71_RS05965	23S rRNA (adenine(2503)-C(2))-methyltransferase	QEN71_05965	paras_001747	protein-codi	NZ_CP1252	chromosom	1321350	1322498	+	1149	0	29	5013	22	4104	
QEN71_RS05970	helix-turn-helix domain-containing protein	QEN71_05970	paras_001748	protein-codi	NZ_CP1252	chromosom	1322709	1323812	+	1104	0	6	2409	2	373	
QEN71_RS05975	flavodoxin-dependent (E)-4-hydroxy-3-methylbut-2-	QEN71_05975	paras_001749	protein-codi	NZ_CP1252	chromosom	1323936	1325249	+	1314	0	3	464	2	5	TRUE
QEN71_RS05980	histidine--tRNA ligase	QEN71_05980	paras_001750	protein-codi	NZ_CP1252	chromosom	1325283	1326623	+	1341	0	3	313	1	5	TRUE
QEN71_RS05985	tetratricopeptide repeat protein	QEN71_05985	paras_001751	protein-codi	NZ_CP1252	chromosom	1326710	1327336	+	627	0	8	1075	7	994	
QEN71_RS05990	outer membrane protein assembly factor BamB	QEN71_05990	paras_001752	protein-codi	NZ_CP1252	chromosom	1327405	1328550	+	1146	0	0	0	0	0	TRUE
QEN71_RS05995	ribosome biogenesis GTPase Der	QEN71_05995	paras_001753	protein-codi	NZ_CP1252	chromosom	1328947	1330287	+	1341	0	0	0	0	0	TRUE
QEN71_RS06000	RNA chaperone Hfq	QEN71_06000	paras_001754	protein-codi	NZ_CP1252	chromosom	1330477	1330713	+	237	0	2	25	0	0	
QEN71_RS06005	GTPase HflX	QEN71_06005	paras_001755	protein-codi	NZ_CP1252	chromosom	1330772	1332016	+	1245	0	36	5913	30	5444	
QEN71_RS06010	FtsH protease activity modulator HflK	QEN71_06010	paras_001756	protein-codi	NZ_CP1252	chromosom	1332047	1333453	+	1407	0	36	5107	30	4331	
QEN71_RS06015	protease modulator HflC	QEN71_06015	paras_001757	protein-codi	NZ_CP1252	chromosom	1333465	1334385	+	921	0	24	4694	18	2873	
QEN71_RS06020	DUF2065 domain-containing protein	QEN71_06020	paras_001758	protein-codi	NZ_CP1252	chromosom	1334429	1334620	+	192	0	11	1610	7	742	
QEN71_RS06025	ATP phosphoribosyltransferase regulatory subunit	QEN71_06025	paras_001759	protein-codi	NZ_CP1252	chromosom	1334883	1336034	+	1152	0	35	6241	27	4797	
QEN71_RS06030	adenylosuccinate synthase	QEN71_06030	paras_001760	protein-codi	NZ_CP1252	chromosom	1336256	1337602	+	1347	0	11	675	8	73	
QEN71_RS06035	phosphoribosyltransferase	QEN71_06035	paras_001761	protein-codi	NZ_CP1252	chromosom	1337609	1338193	+	585	0	21	8004	16	5734	
QEN71_RS06040	potassium transporter Kup	QEN71_06040	paras_001762	protein-codi	NZ_CP1252	chromosom	1338513	1340399	+	1887	0	56	8420	40	6466	
QEN71_RS06045	Tex family protein	QEN71_06045	paras_001763	protein-codi	NZ_CP1252	chromosom	1341027	1343363	-	2337	0	47	5711	25	3221	
QEN71_RS06050	colicin transporter	QEN71_06050	paras_001764	protein-codi	NZ_CP1252	chromosom	1343719	1344534	+	816	0	13	2553	11	1867	
QEN71_RS06055	DUF465 domain-containing protein	QEN71_06055	paras_001765	protein-codi	NZ_CP1252	chromosom	1344653	1344871	+	219	0	2	1147	2	1147	
QEN71_RS06060	ATP-dependent DNA helicase	QEN71_06060	paras_001766	protein-codi	NZ_CP1252	chromosom	1344941	1347205	+	2265	0	46	16541	34	11884	
QEN71_RS06065	hypothetical protein			protein-codi	NZ_CP1252	chromosom	1347461	1347658	+	198	0	5	936	0	0	
QEN71_RS06070	cupin domain-containing protein	QEN71_06070	paras_001768	protein-codi	NZ_CP1252	chromosom	1347978	1349225	-	1248	0	54	17877	35	13792	
QEN71_RS06075	peptidylprolyl isomerase	QEN71_06075	paras_001769	protein-codi	NZ_CP1252	chromosom	1349258	1349809	+	552	0	10	3263	8	2453	
QEN71_RS06080	hypothetical protein	QEN71_06080	paras_001770	protein-codi	NZ_CP1252	chromosom	1349903	1351045	-	1143	0	24	2903	18	2131	
QEN71_RS06085	DNA mismatch repair protein MutS	QEN71_06085	paras_001771	protein-codi	NZ_CP1252	chromosom	1351101	1353779	-	2679	0	60	16740	48	13845	
QEN71_RS06090	lysophospholipid transporter LplT	QEN71_06090	paras_001772	protein-codi	NZ_CP1252	chromosom	1353901	1355199	-	1299	0	29	9149	26	7993	
QEN71_RS06095	inositol monophosphatase family protein	QEN71_06095	paras_001773	protein-codi	NZ_CP1252	chromosom	1355304	1356107	-	804	0	13	1387	7	58	
QEN71_RS06100	RNA methyltransferase	QEN71_06100	paras_001774	protein-codi	NZ_CP1252	chromosom	1356467	1357297	+	831	0	27	14281	21	13312	
QEN71_RS06105	serine O-acetyltransferase	QEN71_06105	paras_001775	protein-codi	NZ_CP1252	chromosom	1357566	1358381	+	816	0	11	2785	11	2785	
QEN71_RS06110	UDP-2,3-diacylglucosamine diphosphatase	QEN71_06110	paras_001776	protein-codi	NZ_CP1252	chromosom	1358449	1359249	-	801	0	6	3309	0	0	
QEN71_RS06115	peptidylprolyl isomerase	QEN71_06115	paras_001777	protein-codi	NZ_CP1252	chromosom	1359316	1359810	-	495	0	7	1249	6	675	
QEN71_RS06120	peptidylprolyl isomerase	QEN71_06120	paras_001778	protein-codi	NZ_CP1252	chromosom	1359975	1360556	-	582	0	20	4104	16	3582	
QEN71_RS06125	tetratricopeptide repeat protein	QEN71_06125	paras_001779	protein-codi	NZ_CP1252	chromosom	1360639	1361439	-	801	0	6	1618	4	969	
QEN71_RS06130	cysteine--tRNA ligase	QEN71_06130	paras_001780	protein-codi	NZ_CP1252	chromosom	1361686	1363083	+	1398	0	1	3	0	0	TRUE
QEN71_RS06135	DNA-3-methyladenine glycosylase	QEN71_06135	paras_001781	protein-codi	NZ_CP1252	chromosom	1363131	1364192	+	1062	0	16	517	8	122	
QEN71_RS06140	acetyl-CoA carboxylase carboxyltransferase subuni	QEN71_06140	paras_001782	protein-codi	NZ_CP1252	chromosom	1364363	1365334	+	972	0	2	191	0	0	TRUE
QEN71_RS06145	tRNA lysidine(34) synthetase TilS	QEN71_06145	paras_001783	protein-codi	NZ_CP1252	chromosom	1365381	1366799	+	1419	0	3	23	3	23	TRUE
QEN71_RS06150	aspartate kinase	QEN71_06150	paras_001784	protein-codi	NZ_CP1252	chromosom	1367261	1368511	+	1251	0	4	589	1	170	
QEN71_RS06155	tRNA-Ser	QEN71_06155		tRNA	NZ_CP1252	chromosom	1368717	1368810	+	94	0	0	0	0	0	
QEN71_RS06160	hypothetical protein	QEN71_06160	paras_001786	protein-codi	NZ_CP1252	chromosom	1369256	1369618	+	363	0	16	3454	11	2750	
QEN71_RS06165	enoyl-ACP reductase FabI	QEN71_06165	paras_001787	protein-codi	NZ_CP1252	chromosom	1369718	1370509	-	792	0	2	322	0	0	TRUE
QEN71_RS06170	extracellular solute-binding protein	QEN71_06170	paras_001788	protein-codi	NZ_CP1252	chromosom	1370650	1372548	+	1899	0	73	18255	51	10479	
QEN71_RS06175	ABC transporter permease subunit	QEN71_06175	paras_001789	protein-codi	NZ_CP1252	chromosom	1372606	1373646	+	1041	0	28	7736	21	4893	
QEN71_RS06180	ABC transporter permease	QEN71_06180	paras_001790	protein-codi	NZ_CP1252	chromosom	1373648	1374751	+	1104	0	49	10397	43	9590	
QEN71_RS06185	dipeptide ABC transporter ATP-binding protein	QEN71_06185	paras_001791	protein-codi	NZ_CP1252	chromosom	1374808	1376367	+	1560	0	26	6296	20	4737	
QEN71_RS06190	C40 family peptidase	QEN71_06190	paras_001792	protein-codi	NZ_CP1252	chromosom	1376528	1377199	+	672	0	24	4803	12	2683	
QEN71_RS06195	patatin-like phospholipase family protein	QEN71_06195	paras_001793	protein-codi	NZ_CP1252	chromosom	1377285	1378238	-	954	0	15	5610	15	5610	
QEN71_RS06200	glutamate--tRNA ligase	QEN71_06200	paras_001794	protein-codi	NZ_CP1252	chromosom	1378640	1380049	+	1410	0	1	9	1	9	TRUE
QEN71_RS06205	tRNA-Ala	QEN71_06205		tRNA	NZ_CP1252	chromosom	1380188	1380263	+	76	0	3	302	1	58	
QEN71_RS06210	tRNA-Glu	QEN71_06210		tRNA	NZ_CP1252	chromosom	1380407	1380482	+	76	150.0	1	11	1	11	
QEN71_RS06215	tRNA-Asp	QEN71_06215		tRNA	NZ_CP1252	chromosom	1380559	1380635	+	77	153.0	3	34	2	32	
QEN71_RS06220	tRNA-Glu	QEN71_06220		tRNA	NZ_CP1252	chromosom	1380702	1380777	+	76	150.0	4	332	4	332	
QEN71_RS06225	tRNA-Asp	QEN71_06225		tRNA	NZ_CP1252	chromosom	1380854	1380930	+	77	125.0	3	877	2	377	
QEN71_RS06230	GNAT family N-acetyltransferase	QEN71_06230	paras_001800	protein-codi	NZ_CP1252	chromosom	1381093	1381557	+	465	0	17	4944	13	4409	
QEN71_RS06235	helix-turn-helix transcriptional regulator	QEN71_06235	paras_001801	protein-codi	NZ_CP1252	chromosom	1381607	1382491	-	885	0	15	5273	14	5240	
QEN71_RS06240	ribonuclease E activity regulator RraA	QEN71_06240	paras_001802	protein-codi	NZ_CP1252	chromosom	1382647	1383141	+	495	0	4	1741	3	1296	
QEN71_RS06245	gamma-glutamylcyclotransferase family protein	QEN71_06245	paras_001803	protein-codi	NZ_CP1252	chromosom	1383191	1383598	+	408	0	9	800	7	616	
QEN71_RS06250	malate synthase A	QEN71_06250	paras_001804	protein-codi	NZ_CP1252	chromosom	1383739	1385331	-	1593	0	33	11484	26	9986	
QEN71_RS06255	haloacid dehalogenase type II	QEN71_06255	paras_001805	protein-codi	NZ_CP1252	chromosom	1385409	1386185	-	777	0	15	3652	13	2951	
QEN71_RS06260	LysR family transcriptional regulator	QEN71_06260	paras_001806	protein-codi	NZ_CP1252	chromosom	1386340	1387242	+	903	0	16	2971	11	2039	
QEN71_RS06265	universal stress protein	QEN71_06265	paras_001807	protein-codi	NZ_CP1252	chromosom	1387501	1387935	+	435	0	12	1109	8	611	
QEN71_RS06270	isocitrate lyase	QEN71_06270	paras_001808	protein-codi	NZ_CP1252	chromosom	1388053	1389357	-	1305	0	27	9070	24	8700	

QEN71_RS06275	DEAD/DEAH box helicase	QEN71_06275	paras_001809	protein-codi	NZ_CP1252	chromosom	1389719	1391416	-	1698	0	25	1991	12	665	
QEN71_RS06280	acyl-CoA-binding protein	QEN71_06280	paras_001810	protein-codi	NZ_CP1252	chromosom	1391850	1392119	-	270	0	14	7838	13	7739	
QEN71_RS06285	tRNA (adenosine(37)-N6)-threonylcarbamoyltransfe	QEN71_06285	paras_001811	protein-codi	NZ_CP1252	chromosom	1392288	1393109	+	818	0	0	0	0	0	TRUE
QEN71_RS06290	ribosomal protein S18-alanine N-acetyltransferase	QEN71_06290	paras_001812	protein-codi	NZ_CP1252	chromosom	1393106	1393600	+	477	0	20	3764	17	2772	
QEN71_RS06295	uracil-DNA glycosylase	QEN71_06295	paras_001813	protein-codi	NZ_CP1252	chromosom	1393587	1394588	+	984	0	2	17	2	17	TRUE
QEN71_RS06300	DUF1853 family protein	QEN71_06300	paras_001814	protein-codi	NZ_CP1252	chromosom	1394585	1395580	+	992	0	14	3403	12	2683	
QEN71_RS06305	bifunctional hydroxymethylpyrimidine kinase/phosph	QEN71_06305	paras_001815	protein-codi	NZ_CP1252	chromosom	1395614	1396420	-	807	0	0	0	0	0	TRUE
QEN71_RS06310	lysophospholipid transporter LplT	QEN71_06310	paras_001816	protein-codi	NZ_CP1252	chromosom	1396482	1397789	-	1308	0	27	2052	23	1682	
QEN71_RS06315	alanine racemase	QEN71_06315	paras_001817	protein-codi	NZ_CP1252	chromosom	1398009	1399079	+	1071	0	2	239	0	0	TRUE
QEN71_RS06320	DNA repair protein RadA	QEN71_06320	paras_001818	protein-codi	NZ_CP1252	chromosom	1399141	1400517	+	1377	0	21	6454	11	4147	
QEN71_RS06325	DUF2866 domain-containing protein	QEN71_06325	paras_001819	protein-codi	NZ_CP1252	chromosom	1400658	1400921	+	264	0	0	0	0	0	
QEN71_RS06330	CaiB/BaiF CoA-transferase family protein	QEN71_06330	paras_001820	protein-codi	NZ_CP1252	chromosom	1400944	1402026	-	1083	0	28	6342	22	5295	
QEN71_RS06335	ATP-binding cassette domain-containing protein	QEN71_06335	paras_001821	protein-codi	NZ_CP1252	chromosom	1402084	1404024	-	1941	0	32	5888	28	5379	
QEN71_RS06340	glutathione peroxidase	QEN71_06340	paras_001822	protein-codi	NZ_CP1252	chromosom	1404151	1404639	+	489	0	14	2257	10	1544	
QEN71_RS06345	cardiolipin synthase	QEN71_06345	paras_001823	protein-codi	NZ_CP1252	chromosom	1404646	1406082	-	1437	0	41	12268	32	8936	
QEN71_RS06350	50S ribosomal protein L3 N(5)-glutamine methyltran	QEN71_06350	paras_001824	protein-codi	NZ_CP1252	chromosom	1406288	1407175	-	888	0	21	4271	21	4271	
QEN71_RS06355	succinyl-diaminopimelate desuccinylase	QEN71_06355	paras_001825	protein-codi	NZ_CP1252	chromosom	1407191	1408330	-	1140	0	17	3159	9	1845	
QEN71_RS06360	ArsC family reductase	QEN71_06360	paras_001826	protein-codi	NZ_CP1252	chromosom	1408430	1408795	-	365	0	5	1575	1	197	
QEN71_RS06365	2,3,4,5-tetrahydropyridine-2,6-dicarboxylate N-succ	QEN71_06365	paras_001827	protein-codi	NZ_CP1252	chromosom	1408795	1409622	-	827	0	0	0	0	0	TRUE
QEN71_RS06370	succinyldiaminopimelate transaminase	QEN71_06370	paras_001828	protein-codi	NZ_CP1252	chromosom	1409720	1410937	-	1218	0	52	20165	46	18820	
QEN71_RS06375	DMT family transporter	QEN71_06375	paras_001829	protein-codi	NZ_CP1252	chromosom	1411312	1412280	+	969	0	16	6474	7	2718	
QEN71_RS06380	chromosome segregation protein SMC	QEN71_06380	paras_001830	protein-codi	NZ_CP1252	chromosom	1412447	1415965	+	3519	0	7	367	2	4	TRUE
QEN71_RS06385	cell division protein ZipA C-terminal FtsZ-binding do	QEN71_06385	paras_001831	protein-codi	NZ_CP1252	chromosom	1416147	1417385	+	1239	0	20	5273	13	3435	
QEN71_RS06390	hypothetical protein	QEN71_06390	paras_001832	protein-codi	NZ_CP1252	chromosom	1417522	1418421	-	900	0	27	9503	23	8349	
QEN71_RS06395	NAD-dependent DNA ligase LigA	QEN71_06395	paras_001833	protein-codi	NZ_CP1252	chromosom	1418605	1420656	+	2048	0	3	1294	1	12	
QEN71_RS06400	peptide deformylase	QEN71_06400	paras_001834	protein-codi	NZ_CP1252	chromosom	1420653	1421186	+	530	0	8	803	6	609	
QEN71_RS06405	pseudouridine synthase	QEN71_06405	paras_001835	protein-codi	NZ_CP1252	chromosom	1421251	1423449	-	2199	0	37	14264	28	13160	
QEN71_RS06410	[protein-PII] uridylyltransferase	QEN71_06410	paras_001836	protein-codi	NZ_CP1252	chromosom	1423456	1426035	-	2580	0	77	16115	57	8776	
QEN71_RS06415	type I methionyl aminopeptidase	QEN71_06415	paras_001837	protein-codi	NZ_CP1252	chromosom	1426061	1426873	-	813	0	42	18744	31	14080	
QEN71_RS06420	30S ribosomal protein S2	QEN71_06420	paras_001838	protein-codi	NZ_CP1252	chromosom	1427272	1428024	+	753	0	3	619	2	6	
QEN71_RS06425	translation elongation factor Ts	QEN71_06425	paras_001839	protein-codi	NZ_CP1252	chromosom	1428214	1429095	+	882	0	5	941	1	5	
QEN71_RS06430	UMP kinase	QEN71_06430	paras_001840	protein-codi	NZ_CP1252	chromosom	1429341	1430054	+	714	0	1	170	0	0	TRUE
QEN71_RS06435	ribosome recycling factor	QEN71_06435	paras_001841	protein-codi	NZ_CP1252	chromosom	1430148	1430708	+	561	0	0	0	0	0	TRUE
QEN71_RS06440	polyprenyl diphosphate synthase	QEN71_06440	paras_001842	protein-codi	NZ_CP1252	chromosom	1430780	1431562	+	776	0	1	56	0	0	TRUE
QEN71_RS06445	CDP-archaeol synthase	QEN71_06445	paras_001843	protein-codi	NZ_CP1252	chromosom	1431556	1432377	+	815	0	3	350	0	0	TRUE
QEN71_RS06450	1-deoxy-D-xylulose-5-phosphate reductoisomerase	QEN71_06450	paras_001844	protein-codi	NZ_CP1252	chromosom	1432399	1433604	+	1206	0	3	125	0	0	TRUE
QEN71_RS06455	RIP metalloprotease RseP	QEN71_06455	paras_001845	protein-codi	NZ_CP1252	chromosom	1433612	1435000	+	1389	0	34	5344	22	3368	
QEN71_RS06460	outer membrane protein assembly factor BamA	QEN71_06460	paras_001846	protein-codi	NZ_CP1252	chromosom	1435066	1437378	+	2313	371.0	0	0	0	0	
QEN71_RS06465	OmpH family outer membrane protein	QEN71_06465	paras_001847	protein-codi	NZ_CP1252	chromosom	1437465	1437956	+	492	0	2	351	2	351	
QEN71_RS06470	UDP-3-O-(3-hydroxymyristoyl)glucosamine N-acyltr	QEN71_06470	paras_001848	protein-codi	NZ_CP1252	chromosom	1438049	1439125	+	1077	0	1	491	0	0	TRUE
QEN71_RS06475	3-hydroxyacyl-ACP dehydratase FabZ	QEN71_06475	paras_001849	protein-codi	NZ_CP1252	chromosom	1439295	1439756	+	462	0	1	432	0	0	
QEN71_RS06480	acyl-ACP--UDP-N-acetylglucosamine O-acyltransfe	QEN71_06480	paras_001850	protein-codi	NZ_CP1252	chromosom	1439945	1440733	+	789	0	0	0	0	0	TRUE
QEN71_RS06485	lipid-A-disaccharide synthase	QEN71_06485	paras_001851	protein-codi	NZ_CP1252	chromosom	1440737	1441906	+	1166	0	0	0	0	0	TRUE
QEN71_RS06490	ribonuclease HII	QEN71_06490	paras_001852	protein-codi	NZ_CP1252	chromosom	1441903	1442640	+	734	0	11	3601	9	1845	
QEN71_RS06495	RNA methyltransferase	QEN71_06495	paras_001853	protein-codi	NZ_CP1252	chromosom	1442710	1443492	+	783	0	11	2521	9	2157	
QEN71_RS06500	pyruvate, water dikinase regulatory protein	QEN71_06500	paras_001854	protein-codi	NZ_CP1252	chromosom	1443552	1444367	-	816	0	16	1453	9	365	
QEN71_RS06505	phosphoenolpyruvate synthase	QEN71_06505	paras_001855	protein-codi	NZ_CP1252	chromosom	1444761	1447166	+	2406	0	50	3711	29	1406	
QEN71_RS06510	NfeD family protein	QEN71_06510	paras_001856	protein-codi	NZ_CP1252	chromosom	1447298	1447750	+	453	0	10	2942	10	2942	
QEN71_RS06515	stomatin-like protein	QEN71_06515	paras_001857	protein-codi	NZ_CP1252	chromosom	1447841	1448770	+	930	0	29	5321	19	2916	
QEN71_RS06520	SsrA-binding protein SmpB	QEN71_06520	paras_001858	protein-codi	NZ_CP1252	chromosom	1449405	1449851	-	447	0	2	375	0	0	
QEN71_RS06525	type II toxin-antitoxin system RatA family toxin	QEN71_06525	paras_001859	protein-codi	NZ_CP1252	chromosom	1449983	1450420	+	434	0	2	13	1	4	TRUE
QEN71_RS06530	RnfH family protein	QEN71_06530	paras_001860	protein-codi	NZ_CP1252	chromosom	1450417	1450749	+	329	0	2	51	0	0	TRUE
QEN71_RS06535	DMT family transporter	QEN71_06535	paras_001861	protein-codi	NZ_CP1252	chromosom	1450844	1451818	-	975	0	24	9133	20	8367	
QEN71_RS06540	IMP dehydrogenase	QEN71_06540	paras_001862	protein-codi	NZ_CP1252	chromosom	1452115	1453575	+	1461	0	2	464	0	0	TRUE
QEN71_RS06545	hypothetical protein	QEN71_06545	paras_001863	protein-codi	NZ_CP1252	chromosom	1453582	1454409	+	828	0	6	1649	4	1185	
QEN71_RS06550	glutamine-hydrolyzing GMP synthase	QEN71_06550	paras_001864	protein-codi	NZ_CP1252	chromosom	1454622	1456205	+	1584	0	3	297	1	6	TRUE
QEN71_RS06555	site-specific integrase	QEN71_06555	paras_001865	protein-codi	NZ_CP1252	chromosom	1456428	1457744	+	1317	0	66	14388	39	7266	
QEN71_RS06560	PLP-dependent aminotransferase family protein	QEN71_06560	paras_001866	protein-codi	NZ_CP1252	chromosom	1457914	1459383	-	1470	0	59	10649	47	9300	
QEN71_RS06565	four-carbon acid sugar kinase family protein	QEN71_06565	paras_001867	protein-codi	NZ_CP1252	chromosom	1459471	1460562	+	1088	0	28	5040	16	3924	
QEN71_RS06570	4-hydroxythreonine-4-phosphate dehydrogenase Po	QEN71_06570	paras_001868	protein-codi	NZ_CP1252	chromosom	1460559	1461560	+	998	0	18	3165	15	2888	
QEN71_RS06575	4-hydroxythreonine-4-phosphate dehydrogenase	QEN71_06575	paras_001869	protein-codi	NZ_CP1252	chromosom	1461588	1462268	+	677	0	19	3035	16	2801	
QEN71_RS06580	cupin domain-containing protein	QEN71_06580	paras_001870	protein-codi	NZ_CP1252	chromosom	1462265	1462642	+	374	0	11	2025	8	1805	

QEN71_RS06585	cyclase family protein	QEN71_06585	paras_001871	protein-codi	NZ_CP1252	chromosom	1462699	1463403	+	705	0	17	3678	15	3446	
QEN71_RS06590	MerR family transcriptional regulator	QEN71_06590	paras_001872	protein-codi	NZ_CP1252	chromosom	1463435	1463860	-	426	0	19	3948	18	3916	
QEN71_RS06595	oxidoreductase	QEN71_06595	paras_001873	protein-codi	NZ_CP1252	chromosom	1463964	1464785	+	822	0	41	6715	34	6016	
QEN71_RS06600	APC family permease	QEN71_06600	paras_001874	protein-codi	NZ_CP1252	chromosom	1464981	1466321	+	1341	0	79	14024	59	10757	
QEN71_RS06605	LysR family transcriptional regulator	QEN71_06605	paras_001875	protein-codi	NZ_CP1252	chromosom	1466428	1467396	-	969	0	54	4762	43	4348	
QEN71_RS06610	MFS transporter	QEN71_06610	paras_001876	protein-codi	NZ_CP1252	chromosom	1468607	1469989	-	1383	0	56	10136	47	9139	
QEN71_RS06615	GntR family transcriptional regulator	QEN71_06615	paras_001877	protein-codi	NZ_CP1252	chromosom	1470227	1470904	+	674	0	28	4523	22	3625	
QEN71_RS06620	FAD-dependent oxidoreductase	QEN71_06620	paras_001878	protein-codi	NZ_CP1252	chromosom	1470901	1472271	-	1367	0	44	6602	32	4138	
QEN71_RS06625	enoyl-CoA hydratase/isomerase family protein	QEN71_06625	paras_001879	protein-codi	NZ_CP1252	chromosom	1472456	1473229	+	766	0	10	714	10	714	
QEN71_RS06630	CoA-transferase	QEN71_06630	paras_001880	protein-codi	NZ_CP1252	chromosom	1473222	1474787	+	1558	0	25	3611	23	3420	
QEN71_RS06635	acyl-CoA dehydrogenase family protein	QEN71_06635	paras_001881	protein-codi	NZ_CP1252	chromosom	1474809	1475954	+	1146	0	20	2078	16	1680	
QEN71_RS06640	electron transfer flavoprotein subunit beta/FixA fami	QEN71_06640	paras_001882	protein-codi	NZ_CP1252	chromosom	1475968	1476717	+	750	0	15	745	6	372	
QEN71_RS06645	electron transfer flavoprotein subunit alpha/FixB fan	QEN71_06645	paras_001883	protein-codi	NZ_CP1252	chromosom	1476732	1477682	+	951	111.0	18	1527	14	1292	
QEN71_RS06650	HTH-type transcriptional regulator ArgP	QEN71_06650	paras_001884	protein-codi	NZ_CP1252	chromosom	1478569	1479543	+	975	0	28	2061	26	2045	
QEN71_RS06655	IS3 family transposase	QEN71_06655	paras_001885	pseudogene	NZ_CP1252	chromosom	1479669	1480909	-	1241	2458.0	21	3031	15	2535	
QEN71_RS06660	hypothetical protein	QEN71_06660	paras_001886	protein-codi	NZ_CP1252	chromosom	1481152	1482420	-	1261	0	37	3302	32	2986	
QEN71_RS06665	fatty acid desaturase	QEN71_06665	paras_001887	protein-codi	NZ_CP1252	chromosom	1482413	1483357	-	937	0	17	1200	14	1148	
QEN71_RS06670	ACT domain-containing protein	QEN71_06670	paras_001888	protein-codi	NZ_CP1252	chromosom	1483565	1483963	+	399	0	10	1450	10	1450	
QEN71_RS06675	hypothetical protein	QEN71_06675	paras_001889	protein-codi	NZ_CP1252	chromosom	1484205	1484741	+	537	0	13	843	12	804	
QEN71_RS06680	AlpA family phage regulatory protein	QEN71_06680	paras_001890	protein-codi	NZ_CP1252	chromosom	1484858	1485091	+	214	0	5	219	4	202	
QEN71_RS06685	hypothetical protein	QEN71_06685	paras_001891	protein-codi	NZ_CP1252	chromosom	1485072	1485368	+	277	0	8	904	8	904	
QEN71_RS06690	hypothetical protein	QEN71_06690	paras_001892	protein-codi	NZ_CP1252	chromosom	1485713	1486312	+	599	0	19	2787	17	1979	
QEN71_RS06695	lytic transglycosylase domain-containing protein	QEN71_06695	paras_001893	protein-codi	NZ_CP1252	chromosom	1486312	1486989	+	677	0	10	464	7	435	
QEN71_RS06700	TrbC/VirB2 family protein	QEN71_06700	paras_001894	protein-codi	NZ_CP1252	chromosom	1487039	1487377	+	339	0	3	113	2	111	
QEN71_RS06705	VirB3 family type IV secretion system protein	QEN71_06705	paras_001895	protein-codi	NZ_CP1252	chromosom	1487393	1487749	+	346	0	2	19	2	19	
QEN71_RS06710	VirB4 family type IV secretion/conjugal transfer ATP	QEN71_06710	paras_001896	protein-codi	NZ_CP1252	chromosom	1487739	1490225	+	2472	0	51	5556	46	4661	
QEN71_RS06715	type IV secretion system protein	QEN71_06715	paras_001897	protein-codi	NZ_CP1252	chromosom	1490222	1491382	+	1157	0	18	2363	15	1856	
QEN71_RS06720	P-type DNA transfer protein VirB5	QEN71_06720	paras_001898	protein-codi	NZ_CP1252	chromosom	1491397	1492092	+	696	0	18	1329	13	1088	
QEN71_RS06725	type IV secretion system protein	QEN71_06725	paras_001899	protein-codi	NZ_CP1252	chromosom	1492276	1492980	+	701	0	20	2114	15	1767	
QEN71_RS06730	TrbG/VirB9 family P-type conjugative transfer protei	QEN71_06730	paras_001900	protein-codi	NZ_CP1252	chromosom	1492977	1493807	+	819	0	16	1597	15	1582	
QEN71_RS06735	type IV secretion system protein VirB10	QEN71_06735	paras_001901	protein-codi	NZ_CP1252	chromosom	1493800	1495044	+	1233	0	19	2712	15	2186	
QEN71_RS06740	P-type DNA transfer ATPase VirB11	QEN71_06740	paras_001902	protein-codi	NZ_CP1252	chromosom	1495041	1496159	+	1115	0	14	1288	13	1235	
QEN71_RS06745	hypothetical protein	QEN71_06745	paras_001903	protein-codi	NZ_CP1252	chromosom	1496167	1496568	+	398	0	12	1904	10	1677	
QEN71_RS06750	type IV secretory system conjugative DNA transfer f	QEN71_06750	paras_001904	protein-codi	NZ_CP1252	chromosom	1496565	1498301	+	1729	0	39	4842	35	4610	
QEN71_RS06755	PriCT-2 domain-containing protein	QEN71_06755	paras_001905	protein-codi	NZ_CP1252	chromosom	1498298	1500163	+	1862	0	31	3315	18	1078	
QEN71_RS06760	DNA primase	QEN71_06760	paras_001906	protein-codi	NZ_CP1252	chromosom	1500214	1501737	+	1524	0	39	4413	35	4048	
QEN71_RS06765	plasmid stabilization protein	QEN71_06765	paras_001907	protein-codi	NZ_CP1252	chromosom	1501746	1502282	+	537	0	9	1713	3	775	
QEN71_RS06770	hypothetical protein	QEN71_06770	paras_001908	protein-codi	NZ_CP1252	chromosom	1502284	1503297	+	1014	0	20	3426	17	2922	
QEN71_RS06775	hypothetical protein	QEN71_06775	paras_001909	protein-codi	NZ_CP1252	chromosom	1503513	1503941	-	429	0	14	2047	9	1214	
QEN71_RS06780	hypothetical protein	QEN71_06780	paras_001910	protein-codi	NZ_CP1252	chromosom	1504087	1504578	-	492	0	10	1774	7	1459	
QEN71_RS06785	helix-turn-helix transcriptional regulator	QEN71_06785	paras_001911	protein-codi	NZ_CP1252	chromosom	1504789	1505112	+	324	0	9	1011	8	923	
QEN71_RS06790	LysR family transcriptional regulator	QEN71_06790	paras_001912	protein-codi	NZ_CP1252	chromosom	1505442	1506401	+	956	0	14	1220	10	1002	
QEN71_RS06795	cupin domain-containing protein	QEN71_06795	paras_001913	protein-codi	NZ_CP1252	chromosom	1506398	1507342	-	941	0	34	5514	22	3759	
QEN71_RS06800	transposase			pseudogene	NZ_CP1252	chromosom	1507840	1507980	-	141	0	4	287	3	152	
QEN71_RS06805	hypothetical protein	QEN71_06805	paras_001915	protein-codi	NZ_CP1252	chromosom	1508407	1508790	-	384	0	7	386	4	348	
QEN71_RS06810	hypothetical protein	QEN71_06810	paras_001916	protein-codi	NZ_CP1252	chromosom	1508802	1509263	+	462	0	7	719	5	600	
QEN71_RS06815	protein mobD	QEN71_06815	paras_001917	protein-codi	NZ_CP1252	chromosom	1509273	1509965	+	685	0	19	1627	16	1146	
QEN71_RS06820	hypothetical protein	QEN71_06820	paras_001918	protein-codi	NZ_CP1252	chromosom	1509958	1510731	+	666	0	16	2086	16	2086	
QEN71_RS06825	hypothetical protein	QEN71_06825	paras_001919	protein-codi	NZ_CP1252	chromosom	1510632	1510877	-	146	0	4	1032	4	1032	
QEN71_RS06830	ParB/RepB/Spo0J family partition protein	QEN71_06830	paras_001920	protein-codi	NZ_CP1252	chromosom	1510963	1513209	-	2247	0	26	2343	23	1931	
QEN71_RS06835	DUF932 domain-containing protein	QEN71_06835	paras_001921	protein-codi	NZ_CP1252	chromosom	1513341	1514174	-	834	0	18	6137	16	5911	
QEN71_RS06840	hypothetical protein	QEN71_06840	paras_001922	protein-codi	NZ_CP1252	chromosom	1515320	1516297	+	978	0	23	6139	17	3496	
QEN71_RS06845	hypothetical protein	QEN71_06845	paras_001923	protein-codi	NZ_CP1252	chromosom	1516576	1517481	+	906	0	39	15169	25	7967	
QEN71_RS06850	hypothetical protein	QEN71_06850	paras_001924	protein-codi	NZ_CP1252	chromosom	1517485	1518117	+	633	0	13	3733	11	2865	
QEN71_RS06855	large ATP-binding protein	QEN71_06855	paras_001925	protein-codi	NZ_CP1252	chromosom	1518129	1520084	+	1956	0	59	12961	32	8289	
QEN71_RS06860	hypothetical protein	QEN71_06860	paras_001926	protein-codi	NZ_CP1252	chromosom	1520126	1522129	+	2004	0	81	22020	62	17242	
QEN71_RS06865	IS21-like element helper ATPase IstB	QEN71_06865	paras_001927	protein-codi	NZ_CP1252	chromosom	1522566	1523354	-	789	1561.0	1	7	1	7	
QEN71_RS06870	IS21 family transposase	QEN71_06870	paras_001928	protein-codi	NZ_CP1252	chromosom	1523361	1524884	-	1524	3027.0	2	4	1	2	
QEN71_RS06875	AAA family ATPase	QEN71_06875	paras_001929	protein-codi	NZ_CP1252	chromosom	1525172	1526674	+	1499	0	47	6050	38	5229	
QEN71_RS06880	HNH endonuclease	QEN71_06880	paras_001930	protein-codi	NZ_CP1252	chromosom	1526671	1527510	+	836	0	31	4397	26	3376	
QEN71_RS06885	DUF4238 domain-containing protein	QEN71_06885	paras_001931	protein-codi	NZ_CP1252	chromosom	1527819	1528733	+	915	0	42	8248	28	5138	
QEN71_RS06890	AAA family ATPase	QEN71_06890	paras_001932	protein-codi	NZ_CP1252	chromosom	1528936	1531593	+	2658	0	30	7123	24	6221	

QEN71_RS06895	hypothetical protein	QEN71_06895	paras_001933	protein-codi	NZ_CP1252	chromosom	1531887	1532666	+	780	0	24	5717	22	5572	
QEN71_RS06900	hypothetical protein	QEN71_06900	paras_001934	protein-codi	NZ_CP1252	chromosom	1532722	1533270	+	549	0	39	9843	34	8876	
QEN71_RS06905	hypothetical protein	QEN71_06905	paras_001935	protein-codi	NZ_CP1252	chromosom	1533320	1534063	-	744	0	52	9392	50	9285	
QEN71_RS06910	hypothetical protein	QEN71_06910	paras_001936	protein-codi	NZ_CP1252	chromosom	1534069	1534518	-	450	0	26	6269	21	4505	
QEN71_RS06915	hypothetical protein	QEN71_06915	paras_001937	protein-codi	NZ_CP1252	chromosom	1534691	1536031	-	1341	0	59	15837	47	13385	
QEN71_RS06920	LA2681 family HEPN domain-containing protein	QEN71_06920	paras_001938	protein-codi	NZ_CP1252	chromosom	1536139	1536723	-	581	0	15	1781	8	410	
QEN71_RS06925	DEAD/DEAH box helicase	QEN71_06925	paras_001939	protein-codi	NZ_CP1252	chromosom	1536720	1539740	-	3017	0	79	13824	59	9246	
QEN71_RS06930	antitoxin	QEN71_06930	paras_001940	protein-codi	NZ_CP1252	chromosom	1539751	1540647	-	896	0	20	3133	16	2715	
QEN71_RS06935	hypothetical protein	QEN71_06935	paras_001941	protein-codi	NZ_CP1252	chromosom	1540647	1541741	-	1094	0	8	267	7	176	
QEN71_RS06940	helix-turn-helix transcriptional regulator	QEN71_06940	paras_001942	protein-codi	NZ_CP1252	chromosom	1541852	1542547	-	696	0	19	5656	15	4689	
QEN71_RS06945	GNAT family N-acetyltransferase	QEN71_06945	paras_001943	protein-codi	NZ_CP1252	chromosom	1543192	1543701	-	510	0	4	1237	0	0	
QEN71_RS06950	UdgX family uracil-DNA binding protein	QEN71_06950	paras_001944	protein-codi	NZ_CP1252	chromosom	1543753	1545243	-	1491	0	28	3739	20	2061	
QEN71_RS06955	putative DNA modification/repair radical SAM protei	QEN71_06955	paras_001945	protein-codi	NZ_CP1252	chromosom	1545260	1546471	-	1212	0	28	2581	25	2125	
QEN71_RS06960	tetratricopeptide repeat protein	QEN71_06960	paras_001946	protein-codi	NZ_CP1252	chromosom	1546735	1548279	+	1545	0	24	3756	20	3284	
QEN71_RS06965	APC family permease	QEN71_06965	paras_001947	protein-codi	NZ_CP1252	chromosom	1548334	1549674	-	1341	0	24	4012	19	3078	
QEN71_RS06970	carbon-nitrogen hydrolase family protein	QEN71_06970	paras_001948	protein-codi	NZ_CP1252	chromosom	1549833	1550678	-	846	0	13	1962	11	1416	
QEN71_RS06975	helix-turn-helix transcriptional regulator	QEN71_06975	paras_001949	protein-codi	NZ_CP1252	chromosom	1550887	1551705	+	819	0	14	1169	12	854	
QEN71_RS06980	glycine zipper 2TM domain-containing protein	QEN71_06980	paras_001950	protein-codi	NZ_CP1252	chromosom	1551776	1552468	-	693	0	25	3228	22	3005	
QEN71_RS06985	hypothetical protein	QEN71_06985		protein-codi	NZ_CP1252	chromosom	1552627	1552752	-	126	0	1	4	1	4	
QEN71_RS06990	DUF1992 domain-containing protein	QEN71_06990	paras_001951	protein-codi	NZ_CP1252	chromosom	1553022	1553423	+	398	0	3	153	3	153	
QEN71_RS06995	tRNA adenosine(34) deaminase TadA	QEN71_06995	paras_001952	protein-codi	NZ_CP1252	chromosom	1553420	1554079	+	656	0	1	36	1	36	TRUE
QEN71_RS07000	muramoyltetrapeptide carboxypeptidase	QEN71_07000	paras_001953	protein-codi	NZ_CP1252	chromosom	1554093	1555019	+	927	0	11	2874	11	2874	
QEN71_RS07005	GntR family transcriptional regulator	QEN71_07005	paras_001954	protein-codi	NZ_CP1252	chromosom	1555347	1556063	+	717	0	5	752	3	439	
QEN71_RS07010	NCS1 family nucleobase:cation symporter-1	QEN71_07010	paras_001955	protein-codi	NZ_CP1252	chromosom	1556456	1557952	+	1497	0	39	5199	28	3714	
QEN71_RS07015	aspartate/glutamate racemase family protein	QEN71_07015	paras_001956	protein-codi	NZ_CP1252	chromosom	1557995	1558849	+	855	0	13	1793	8	801	
QEN71_RS07020	allantoinase PuuE	QEN71_07020	paras_001957	protein-codi	NZ_CP1252	chromosom	1559005	1559958	+	950	0	29	2790	26	2530	
QEN71_RS07025	2-oxo-4-hydroxy-4-carboxy-5-ureidoimidazoline dec	QEN71_07025	paras_001958	protein-codi	NZ_CP1252	chromosom	1559955	1560476	+	518	0	4	501	2	13	
QEN71_RS07030	allantoicase	QEN71_07030	paras_001959	protein-codi	NZ_CP1252	chromosom	1560554	1561567	+	1010	0	23	3351	22	3315	
QEN71_RS07035	ureidoglycolate lyase	QEN71_07035	paras_001960	protein-codi	NZ_CP1252	chromosom	1561564	1562076	+	509	0	5	758	5	758	
QEN71_RS07040	urate hydroxylase PuuD	QEN71_07040	paras_001961	protein-codi	NZ_CP1252	chromosom	1563187	1564377	-	1191	0	36	7745	30	6775	
QEN71_RS07045	hydroxyisourate hydrolase	QEN71_07045	paras_001962	protein-codi	NZ_CP1252	chromosom	1564614	1564967	-	354	0	15	2586	10	2063	
QEN71_RS07050	8-oxoguanine deaminase	QEN71_07050	paras_001963	protein-codi	NZ_CP1252	chromosom	1565599	1567008	+	1410	0	31	8193	23	6290	
QEN71_RS07055	LysR substrate-binding domain-containing protein	QEN71_07055	paras_001964	protein-codi	NZ_CP1252	chromosom	1567071	1568000	-	930	0	35	6628	26	3967	
QEN71_RS07060	ABC transporter ATP-binding protein	QEN71_07060	paras_001965	protein-codi	NZ_CP1252	chromosom	1568302	1569915	+	1614	0	25	4938	21	4473	
QEN71_RS07065	ABC transporter permease	QEN71_07065	paras_001966	protein-codi	NZ_CP1252	chromosom	1569992	1571092	+	1101	0	23	3466	17	2917	
QEN71_RS07070	ABC transporter permease	QEN71_07070	paras_001967	protein-codi	NZ_CP1252	chromosom	1571125	1572054	+	930	0	24	4423	22	3999	
QEN71_RS07075	BMP family ABC transporter substrate-binding prote	QEN71_07075	paras_001968	protein-codi	NZ_CP1252	chromosom	1572135	1573229	+	1095	0	41	8524	35	7211	
QEN71_RS07080	dodecin family protein	QEN71_07080	paras_001969	protein-codi	NZ_CP1252	chromosom	1573436	1573645	-	210	0	4	1498	2	766	
QEN71_RS07085	NAD(P)-dependent oxidoreductase	QEN71_07085	paras_001970	protein-codi	NZ_CP1252	chromosom	1573774	1574721	+	948	0	12	2026	10	1438	
QEN71_RS07090	ABC-F family ATPase	QEN71_07090	paras_001971	protein-codi	NZ_CP1252	chromosom	1574843	1576411	+	1569	0	40	5121	33	4480	
QEN71_RS07095	aldehyde dehydrogenase family protein	QEN71_07095	paras_001972	protein-codi	NZ_CP1252	chromosom	1576580	1578016	+	1437	0	41	7820	25	4666	
QEN71_RS07100	nitronate monooxygenase family protein	QEN71_07100	paras_001973	protein-codi	NZ_CP1252	chromosom	1578201	1579391	+	1191	0	8	1239	8	1239	
QEN71_RS07105	OmpW family outer membrane protein	QEN71_07105	paras_001974	protein-codi	NZ_CP1252	chromosom	1579486	1580130	+	645	0	23	5536	17	4773	
QEN71_RS07110	DUF1289 domain-containing protein	QEN71_07110	paras_001975	protein-codi	NZ_CP1252	chromosom	1580239	1580442	-	204	0	1	24	0	0	
QEN71_RS07115	Nramp family divalent metal transporter	QEN71_07115	paras_001976	protein-codi	NZ_CP1252	chromosom	1580668	1581969	-	1302	0	12	2448	9	1701	
QEN71_RS07120	polyamine ABC transporter substrate-binding protei	QEN71_07120	paras_001977	protein-codi	NZ_CP1252	chromosom	1582480	1583580	+	1101	0	46	13880	37	11332	
QEN71_RS07125	polyamine ABC transporter ATP-binding protein	QEN71_07125	paras_001978	protein-codi	NZ_CP1252	chromosom	1583681	1584844	+	1160	0	28	7690	24	6849	
QEN71_RS07130	ABC transporter permease subunit	QEN71_07130	paras_001979	protein-codi	NZ_CP1252	chromosom	1584841	1585770	+	925	0	30	7144	29	7096	
QEN71_RS07135	ABC transporter permease subunit	QEN71_07135	paras_001980	protein-codi	NZ_CP1252	chromosom	1585770	1586588	+	818	0	18	2455	14	2129	
QEN71_RS07140	methyl-accepting chemotaxis protein	QEN71_07140	paras_001981	protein-codi	NZ_CP1252	chromosom	1586677	1588089	-	1413	0	20	3859	14	3312	
QEN71_RS07145	class I SAM-dependent methyltransferase	QEN71_07145	paras_001982	protein-codi	NZ_CP1252	chromosom	1588570	1589343	+	770	0	25	3599	17	2131	
QEN71_RS07150	alpha/beta hydrolase	QEN71_07150	paras_001983	protein-codi	NZ_CP1252	chromosom	1589340	1590311	-	968	0	34	8298	29	7365	
QEN71_RS07155	NAD(P)/FAD-dependent oxidoreductase	QEN71_07155	paras_001984	protein-codi	NZ_CP1252	chromosom	1590367	1591848	-	1482	0	52	12915	37	8360	
QEN71_RS07160	TetR family transcriptional regulator	QEN71_07160	paras_001985	protein-codi	NZ_CP1252	chromosom	1592047	1592676	+	630	0	6	335	6	335	
QEN71_RS07165	tetratricopeptide repeat protein	QEN71_07165	paras_001986	protein-codi	NZ_CP1252	chromosom	1592783	1594012	-	1230	0	19	2402	18	2302	
QEN71_RS07170	PspA/IM30 family protein	QEN71_07170	paras_001987	protein-codi	NZ_CP1252	chromosom	1594118	1594798	-	681	0	5	1929	4	1882	
QEN71_RS07175	hypothetical protein	QEN71_07175	paras_001988	protein-codi	NZ_CP1252	chromosom	1595310	1595966	-	657	0	6	1721	5	721	
QEN71_RS07180	penicillin-binding protein 1A	QEN71_07180	paras_001989	protein-codi	NZ_CP1252	chromosom	1596590	1599121	+	2532	0	89	19894	79	19164	
QEN71_RS07185	efflux transporter outer membrane subunit	QEN71_07185	paras_001990	protein-codi	NZ_CP1252	chromosom	1599445	1600914	-	1470	0	34	5858	23	4505	
QEN71_RS07190	efflux RND transporter permease subunit	QEN71_07190	paras_001991	protein-codi	NZ_CP1252	chromosom	1600917	1604039	-	3119	0	60	18563	48	14577	
QEN71_RS07195	efflux RND transporter periplasmic adaptor subunit	QEN71_07195	paras_001992	protein-codi	NZ_CP1252	chromosom	1604036	1605163	-	1124	0	16	5173	13	4509	
QEN71_RS07200	TetR/AcrR family transcriptional regulator	QEN71_07200	paras_001993	protein-codi	NZ_CP1252	chromosom	1605438	1606058	+	621	0	19	4299	16	3825	

QEN71_RS07205	cupin domain-containing protein	QEN71_07205	paras_001994	protein-codi	NZ_CP1252	chromosom	1606083	1606457	-	375	0	10	1919	8	1440	
QEN71_RS07210	AraC family transcriptional regulator	QEN71_07210	paras_001995	protein-codi	NZ_CP1252	chromosom	1606548	1607444	-	897	0	22	7758	17	6165	
QEN71_RS07215	DUF2795 domain-containing protein	QEN71_07215	paras_001996	protein-codi	NZ_CP1252	chromosom	1607467	1607697	-	231	0	6	1405	6	1405	
QEN71_RS07220	hypothetical protein	QEN71_07220	paras_001997	protein-codi	NZ_CP1252	chromosom	1607786	1608037	-	252	0	3	362	3	362	
QEN71_RS07225	C45 family peptidase	QEN71_07225	paras_001998	protein-codi	NZ_CP1252	chromosom	1608376	1609713	-	1338	0	41	9293	35	7126	
QEN71_RS07230	ClcB-like voltage-gated chloride channel protein	QEN71_07230	paras_001999	protein-codi	NZ_CP1252	chromosom	1609875	1611608	-	1734	0	48	11993	41	10932	
QEN71_RS07235	glutathione S-transferase	QEN71_07235	paras_002000	protein-codi	NZ_CP1252	chromosom	1611733	1612470	-	738	0	37	9342	27	7282	
QEN71_RS07240	YeiH family protein	QEN71_07240	paras_002001	protein-codi	NZ_CP1252	chromosom	1612639	1613724	-	1086	0	18	3522	15	2293	
QEN71_RS07245	LysR family transcriptional regulator	QEN71_07245	paras_002002	protein-codi	NZ_CP1252	chromosom	1613844	1614758	+	915	0	25	6368	16	4005	
QEN71_RS07250	TIM barrel protein	QEN71_07250	paras_002003	protein-codi	NZ_CP1252	chromosom	1614889	1615653	+	765	0	10	4374	9	4361	
QEN71_RS07255	sugar kinase	QEN71_07255	paras_002004	protein-codi	NZ_CP1252	chromosom	1615694	1616656	+	963	0	23	4393	21	4104	
QEN71_RS07260	MFS transporter	QEN71_07260	paras_002005	protein-codi	NZ_CP1252	chromosom	1616789	1618063	+	1275	0	30	7345	21	4352	
QEN71_RS07265	D-glycerate dehydrogenase	QEN71_07265	paras_002006	protein-codi	NZ_CP1252	chromosom	1618108	1619076	+	965	0	16	1049	12	843	
QEN71_RS07270	LacI family DNA-binding transcriptional regulator	QEN71_07270	paras_002007	protein-codi	NZ_CP1252	chromosom	1619073	1620134	+	1058	0	16	2845	14	2537	
QEN71_RS07275	YhfC family intramembrane metalloprotease	QEN71_07275	paras_002008	protein-codi	NZ_CP1252	chromosom	1620201	1620995	+	795	0	18	5408	13	3117	
QEN71_RS07280	DUF3022 domain-containing protein	QEN71_07280	paras_002009	protein-codi	NZ_CP1252	chromosom	1621050	1621415	+	366	0	10	1603	6	715	
QEN71_RS07285	PGDYG domain-containing protein	QEN71_07285	paras_002010	protein-codi	NZ_CP1252	chromosom	1621440	1621877	-	438	0	12	2309	9	2069	
QEN71_RS07290	Spy/CpxP family protein refolding chaperone	QEN71_07290	paras_002011	protein-codi	NZ_CP1252	chromosom	1622020	1622541	-	522	0	9	2551	9	2551	
QEN71_RS07295	LysR substrate-binding domain-containing protein	QEN71_07295	paras_002012	protein-codi	NZ_CP1252	chromosom	1622695	1623579	+	885	0	27	8022	19	5866	
QEN71_RS07300	Lrp/AsnC family transcriptional regulator	QEN71_07300	paras_002013	protein-codi	NZ_CP1252	chromosom	1623707	1624165	+	459	0	8	2467	8	2467	
QEN71_RS07305	saccharopine dehydrogenase NADP-binding domai	QEN71_07305	paras_002014	protein-codi	NZ_CP1252	chromosom	1624359	1625456	+	1098	0	26	6333	20	4830	
QEN71_RS07310	ATP-binding protein	QEN71_07310	paras_002015	protein-codi	NZ_CP1252	chromosom	1625599	1626219	-	621	0	16	5079	12	4544	
QEN71_RS07315	DNA-binding protein	QEN71_07315	paras_002016	protein-codi	NZ_CP1252	chromosom	1626368	1626613	+	246	0	6	1843	2	1254	
QEN71_RS07320	type II toxin-antitoxin system VapC family toxin	QEN71_07320	paras_002017	protein-codi	NZ_CP1252	chromosom	1626633	1627028	+	396	0	7	3239	7	3239	
QEN71_RS07325	hypothetical protein	QEN71_07325	paras_002018	protein-codi	NZ_CP1252	chromosom	1627118	1627573	+	456	0	18	4772	9	3210	
QEN71_RS07330	magnesium/cobalt transporter CorA	QEN71_07330	paras_002019	protein-codi	NZ_CP1252	chromosom	1627757	1628734	+	978	0	29	7628	19	4905	
QEN71_RS07335	sugar ABC transporter substrate-binding protein	QEN71_07335	paras_002020	protein-codi	NZ_CP1252	chromosom	1629304	1630260	+	957	0	16	3449	14	3381	
QEN71_RS07340	hypothetical protein	QEN71_07340	paras_002021	protein-codi	NZ_CP1252	chromosom	1630343	1630540	-	198	0	6	1681	6	1681	
QEN71_RS07345	sugar ABC transporter ATP-binding protein	QEN71_07345	paras_002022	protein-codi	NZ_CP1252	chromosom	1630591	1632201	+	1607	0	22	8591	16	6523	
QEN71_RS07350	ABC transporter permease	QEN71_07350	paras_002023	protein-codi	NZ_CP1252	chromosom	1632198	1633244	+	1043	0	19	4894	17	4456	
QEN71_RS07355	LacI family DNA-binding transcriptional regulator	QEN71_07355	paras_002024	protein-codi	NZ_CP1252	chromosom	1633379	1634410	+	1032	0	21	5170	15	3832	
QEN71_RS07360	ribokinase	QEN71_07360	paras_002025	protein-codi	NZ_CP1252	chromosom	1634529	1635458	+	930	0	3	540	3	540	
QEN71_RS07365	methyl-accepting chemotaxis protein	QEN71_07365	paras_002026	protein-codi	NZ_CP1252	chromosom	1635703	1637439	+	1737	0	24	6580	24	6580	
QEN71_RS07370	hydratase	QEN71_07370	paras_002027	protein-codi	NZ_CP1252	chromosom	1637817	1638578	+	762	0	17	8381	9	3867	
QEN71_RS07375	PrkA family serine protein kinase	QEN71_07375	paras_002028	protein-codi	NZ_CP1252	chromosom	1639454	1641376	+	1923	0	75	21463	58	16439	
QEN71_RS07380	YeaH/YhbH family protein	QEN71_07380	paras_002029	protein-codi	NZ_CP1252	chromosom	1641518	1642789	+	1268	0	39	6817	27	4708	
QEN71_RS07385	SpoVR family protein	QEN71_07385	paras_002030	protein-codi	NZ_CP1252	chromosom	1642786	1644522	+	1733	0	70	15454	64	14522	
QEN71_RS07390	MFS family transporter	QEN71_07390	paras_002031	protein-codi	NZ_CP1252	chromosom	1644824	1646119	+	1296	0	47	10435	32	7886	
QEN71_RS07395	glycosyltransferase family 2 protein	QEN71_07395	paras_002032	protein-codi	NZ_CP1252	chromosom	1646215	1647192	-	978	0	30	7474	24	4256	
QEN71_RS07400	hypothetical protein	QEN71_07400	paras_002033	protein-codi	NZ_CP1252	chromosom	1647435	1647623	-	189	0	0	0	0	0	
QEN71_RS07405	hypothetical protein	QEN71_07405	paras_002034	protein-codi	NZ_CP1252	chromosom	1647740	1647970	+	231	0	8	1190	7	1081	
QEN71_RS07410	hypothetical protein	QEN71_07410	paras_002035	protein-codi	NZ_CP1252	chromosom	1647985	1648461	-	477	0	13	4098	11	3546	
QEN71_RS07415	MFS transporter	QEN71_07415	paras_002036	protein-codi	NZ_CP1252	chromosom	1648552	1649871	-	1320	0	23	7423	19	6622	
QEN71_RS07420	mechanosensitive ion channel family protein	QEN71_07420	paras_002037	protein-codi	NZ_CP1252	chromosom	1650129	1650986	+	858	0	21	6550	19	6213	
QEN71_RS07425	hypothetical protein	QEN71_07425	paras_002038	protein-codi	NZ_CP1252	chromosom	1651224	1651436	+	213	0	4	1170	4	1170	
QEN71_RS07430	hypothetical protein	QEN71_07430	paras_002039	protein-codi	NZ_CP1252	chromosom	1651747	1652010	+	264	0	5	1325	3	1228	
QEN71_RS07435	cardiolipin synthase	QEN71_07435	paras_002040	protein-codi	NZ_CP1252	chromosom	1652103	1653365	+	1263	0	40	10261	37	9824	
QEN71_RS07440	hypothetical protein	QEN71_07440	paras_002041	protein-codi	NZ_CP1252	chromosom	1653404	1653646	-	243	0	0	0	0	0	
QEN71_RS07445	porin	QEN71_07445	paras_002042	protein-codi	NZ_CP1252	chromosom	1653761	1654837	-	1077	0	36	9889	33	9523	
QEN71_RS07450	high-potential iron-sulfur protein	QEN71_07450	paras_002043	protein-codi	NZ_CP1252	chromosom	1654970	1655284	-	295	0	9	2092	5	636	
QEN71_RS07455	Pr6Pr family membrane protein	QEN71_07455	paras_002044	protein-codi	NZ_CP1252	chromosom	1655265	1656068	-	784	0	23	3957	21	3805	
QEN71_RS07460	esterase-like activity of phytase family protein	QEN71_07460	paras_002045	protein-codi	NZ_CP1252	chromosom	1656210	1657688	-	1479	0	39	5837	37	5540	
QEN71_RS07465	formate/nitrite transporter family protein	QEN71_07465	paras_002046	protein-codi	NZ_CP1252	chromosom	1658000	1658908	-	909	0	11	928	11	928	
QEN71_RS07470	DnaJ domain-containing protein	QEN71_07470	paras_002047	protein-codi	NZ_CP1252	chromosom	1659102	1659743	+	642	0	7	681	6	675	
QEN71_RS07475	hypothetical protein	QEN71_07475	paras_002048	protein-codi	NZ_CP1252	chromosom	1659835	1660374	+	540	0	5	232	5	232	
QEN71_RS07480	hypothetical protein	QEN71_07480	paras_002049	protein-codi	NZ_CP1252	chromosom	1660469	1660756	-	288	0	7	1899	6	1897	
QEN71_RS07485	4-hydroxyphenylacetate 3-monooxygenase, reducta	QEN71_07485	paras_002050	protein-codi	NZ_CP1252	chromosom	1660915	1661454	-	540	0	6	591	2	100	
QEN71_RS07490	response regulator	QEN71_07490	paras_002051	protein-codi	NZ_CP1252	chromosom	1661811	1662470	+	660	0	5	572	1	13	
QEN71_RS07495	SDR family NAD(P)-dependent oxidoreductase	QEN71_07495	paras_002052	protein-codi	NZ_CP1252	chromosom	1662509	1663252	-	744	0	15	950	9	465	
QEN71_RS07500	amidase	QEN71_07500	paras_002053	protein-codi	NZ_CP1252	chromosom	1663304	1664443	-	1136	0	7	944	4	591	
QEN71_RS07505	nuclear transport factor 2 family protein	QEN71_07505	paras_002054	protein-codi	NZ_CP1252	chromosom	1664440	1664967	-	524	0	7	1046	6	1028	
QEN71_RS07510	hypothetical protein	QEN71_07510	paras_002055	protein-codi	NZ_CP1252	chromosom	1664971	1665363	-	393	0	6	204	6	204	

QEN71_RS07515	acyl-CoA dehydrogenase family protein	QEN71_07515	paras_002056	protein-codi	NZ_CP1252	chromosom	1665412	1666608	-	1197	0	14	1111	13	1056	
QEN71_RS07520	ATP-binding protein	QEN71_07520	paras_002057	protein-codi	NZ_CP1252	chromosom	1666816	1668300	+	1485	0	22	2597	16	1510	
QEN71_RS07525	tautomerase family protein	QEN71_07525	paras_002058	protein-codi	NZ_CP1252	chromosom	1668308	1668694	-	387	0	4	820	1	154	
QEN71_RS07530	aromatic-ring-hydroxylating dioxygenase subunit be	QEN71_07530	paras_002059	protein-codi	NZ_CP1252	chromosom	1668765	1669265	-	497	0	11	1969	10	1917	
QEN71_RS07535	aromatic ring-hydroxylating dioxygenase subunit al	QEN71_07535	paras_002060	protein-codi	NZ_CP1252	chromosom	1669262	1670545	-	1280	0	20	1897	14	991	
QEN71_RS07540	LysR family transcriptional regulator	QEN71_07540	paras_002061	protein-codi	NZ_CP1252	chromosom	1670711	1671631	+	921	0	17	3134	15	3057	
QEN71_RS07545	PDR/VanB family oxidoreductase	QEN71_07545	paras_002062	protein-codi	NZ_CP1252	chromosom	1671634	1672602	-	969	0	14	2755	12	2557	
QEN71_RS07550	MDR family oxidoreductase	QEN71_07550	paras_002063	protein-codi	NZ_CP1252	chromosom	1672749	1673729	-	981	0	10	1068	9	813	
QEN71_RS07555	hypothetical protein	QEN71_07555	paras_002064	protein-codi	NZ_CP1252	chromosom	1673949	1674542	-	594	0	14	1975	6	544	
QEN71_RS07560	pyrrolo-quinoline quinone	QEN71_07560	paras_002065	protein-codi	NZ_CP1252	chromosom	1674793	1676538	-	1746	0	53	7585	45	6405	
QEN71_RS07565	hypothetical protein	QEN71_07565	paras_002066	protein-codi	NZ_CP1252	chromosom	1676728	1677774	-	1047	0	24	3790	18	3073	
QEN71_RS07570	hypothetical protein	QEN71_07570	paras_002067	protein-codi	NZ_CP1252	chromosom	1678693	1680615	+	1923	0	80	12291	69	11119	
QEN71_RS07575	16S ribosomal RNA	QEN71_07575		rRNA	NZ_CP1252	chromosom	1681180	1682710	+	1531	3015.0	27	3774	24	3272	
QEN71_RS07580	tRNA-Ile	QEN71_07580		tRNA	NZ_CP1252	chromosom	1682775	1682851	+	77	152.0	1	7	0	0	
QEN71_RS07585	tRNA-Ala	QEN71_07585		tRNA	NZ_CP1252	chromosom	1682911	1682986	+	76	150.0	0	0	0	0	
QEN71_RS07590	23S ribosomal RNA	QEN71_07590		rRNA	NZ_CP1252	chromosom	1683292	1686172	+	2881	5661.0	31	754	25	729	
QEN71_RS07595	5S ribosomal RNA	QEN71_07595		rRNA	NZ_CP1252	chromosom	1686379	1686492	+	114	225.0	2	79	2	79	
QEN71_RS07600	LysR family transcriptional regulator	QEN71_07600	paras_002073	protein-codi	NZ_CP1252	chromosom	1686738	1687736	-	999	0	43	7323	31	5648	
QEN71_RS07605	FUSC family protein	QEN71_07605	paras_002074	protein-codi	NZ_CP1252	chromosom	1687935	1690112	+	2178	0	39	5693	30	4746	
QEN71_RS07610	hypothetical protein	QEN71_07610	paras_002075	protein-codi	NZ_CP1252	chromosom	1690227	1690448	+	222	0	4	648	2	139	
QEN71_RS07615	acetyl-CoA hydrolase/transferase family protein	QEN71_07615	paras_002076	protein-codi	NZ_CP1252	chromosom	1690585	1692099	+	1515	0	17	3104	8	1214	
QEN71_RS07620	metallophosphoesterase	QEN71_07620	paras_002077	protein-codi	NZ_CP1252	chromosom	1692127	1693287	-	1161	0	23	3795	13	1539	
QEN71_RS07625	hypothetical protein	QEN71_07625	paras_002078	protein-codi	NZ_CP1252	chromosom	1693655	1693969	+	315	0	7	950	6	853	
QEN71_RS07630	alpha-ketoglutarate-dependent dioxygenase AlkB	QEN71_07630	paras_002079	protein-codi	NZ_CP1252	chromosom	1694128	1694718	+	591	0	6	2651	6	2651	
QEN71_RS07635	malonate decarboxylase subunit epsilon	QEN71_07635	paras_002080	protein-codi	NZ_CP1252	chromosom	1694794	1695729	-	935	0	19	1681	18	1601	
QEN71_RS07640	malonate decarboxylase holo-ACP synthase	QEN71_07640	paras_002081	protein-codi	NZ_CP1252	chromosom	1695729	1696433	-	703	0	7	538	2	8	
QEN71_RS07645	biotin-independent malonate decarboxylase subunit	QEN71_07645	paras_002082	protein-codi	NZ_CP1252	chromosom	1696433	1697251	-	814	0	6	851	6	851	
QEN71_RS07650	biotin-independent malonate decarboxylase subunit	QEN71_07650	paras_002083	protein-codi	NZ_CP1252	chromosom	1697248	1698111	-	856	0	5	399	4	393	
QEN71_RS07655	malonate decarboxylase subunit delta	QEN71_07655	paras_002084	protein-codi	NZ_CP1252	chromosom	1698108	1698413	-	302	0	4	1556	3	1492	
QEN71_RS07660	triphosphoribosyl-dephospho-CoA synthase	QEN71_07660	paras_002085	protein-codi	NZ_CP1252	chromosom	1698419	1699348	-	930	0	1	18	0	0	TRUE
QEN71_RS07665	malonate decarboxylase subunit alpha	QEN71_07665	paras_002086	protein-codi	NZ_CP1252	chromosom	1699353	1701011	-	1659	0	32	5506	29	4809	
QEN71_RS07670	GntR family transcriptional regulator	QEN71_07670	paras_002087	protein-codi	NZ_CP1252	chromosom	1701064	1701852	-	789	0	12	1365	12	1365	
QEN71_RS07675	MFS transporter	QEN71_07675	paras_002088	protein-codi	NZ_CP1252	chromosom	1702031	1703335	+	1305	0	40	6179	30	5232	
QEN71_RS07680	DMT family transporter	QEN71_07680	paras_002089	protein-codi	NZ_CP1252	chromosom	1703956	1704864	-	909	0	29	4194	22	2684	
QEN71_RS07685	LysR family transcriptional regulator	QEN71_07685	paras_002090	protein-codi	NZ_CP1252	chromosom	1704985	1705881	+	897	0	12	1849	8	1175	
QEN71_RS07690	hypothetical protein	QEN71_07690	paras_002091	protein-codi	NZ_CP1252	chromosom	1706081	1706752	+	672	0	9	751	7	724	
QEN71_RS07695	arylamine N-acetyltransferase	QEN71_07695	paras_002092	protein-codi	NZ_CP1252	chromosom	1706973	1707803	+	831	0	11	1221	4	526	
QEN71_RS07700	isoprenylcysteine carboxylmethyltransferase family	QEN71_07700	paras_002093	protein-codi	NZ_CP1252	chromosom	1707811	1708488	+	678	0	22	4223	19	3949	
QEN71_RS07705	ubiquinone-dependent pyruvate dehydrogenase	QEN71_07705	paras_002094	protein-codi	NZ_CP1252	chromosom	1708854	1710575	+	1722	0	25	3962	20	2739	
QEN71_RS07710	hypothetical protein	QEN71_07710	paras_002095	protein-codi	NZ_CP1252	chromosom	1710938	1711195	+	258	0	6	1175	4	505	
QEN71_RS07715	alpha/beta hydrolase	QEN71_07715	paras_002096	protein-codi	NZ_CP1252	chromosom	1711269	1712066	-	798	0	29	5485	22	4368	
QEN71_RS07720	DUF4148 domain-containing protein	QEN71_07720	paras_002097	protein-codi	NZ_CP1252	chromosom	1712164	1712514	-	351	0	7	744	4	208	
QEN71_RS07725	transporter substrate-binding domain-containing pro	QEN71_07725	paras_002098	protein-codi	NZ_CP1252	chromosom	1713371	1714213	+	843	0	12	1174	7	808	
QEN71_RS07730	ABC transporter permease subunit	QEN71_07730	paras_002099	protein-codi	NZ_CP1252	chromosom	1714298	1715020	+	723	0	14	1692	14	1692	
QEN71_RS07735	ABC transporter permease subunit	QEN71_07735	paras_002100	protein-codi	NZ_CP1252	chromosom	1715036	1715788	+	753	0	12	1317	12	1317	
QEN71_RS07740	ATP-binding cassette domain-containing protein	QEN71_07740	paras_002101	protein-codi	NZ_CP1252	chromosom	1715810	1716601	+	792	0	2	147	1	3	TRUE
QEN71_RS07745	histidine ammonia-lyase	QEN71_07745	paras_002102	protein-codi	NZ_CP1252	chromosom	1716663	1718270	+	1608	0	19	2149	17	2029	
QEN71_RS07750	histidine utilization repressor	QEN71_07750	paras_002103	protein-codi	NZ_CP1252	chromosom	1718304	1719059	+	756	0	15	1728	14	1683	
QEN71_RS07755	histidine utilization repressor	QEN71_07755	paras_002104	protein-codi	NZ_CP1252	chromosom	1719066	1719815	-	750	0	16	1818	9	1344	
QEN71_RS07760	amino acid ABC transporter ATP-binding protein	QEN71_07760	paras_002105	protein-codi	NZ_CP1252	chromosom	1719938	1720804	-	867	0	11	356	8	277	
QEN71_RS07765	amino acid ABC transporter permease	QEN71_07765	paras_002106	protein-codi	NZ_CP1252	chromosom	1720843	1721505	-	663	0	5	960	4	771	
QEN71_RS07770	amino acid ABC transporter permease	QEN71_07770	paras_002107	protein-codi	NZ_CP1252	chromosom	1721516	1722166	-	651	0	16	1175	16	1175	
QEN71_RS07775	transporter substrate-binding domain-containing pro	QEN71_07775	paras_002108	protein-codi	NZ_CP1252	chromosom	1722247	1723086	-	840	0	17	1455	13	1086	
QEN71_RS07780	2-oxoglutarate and iron-dependent oxygenase doma	QEN71_07780	paras_002109	protein-codi	NZ_CP1252	chromosom	1723375	1724382	+	1008	0	29	2005	17	1141	
QEN71_RS07785	FAD-binding oxidoreductase	QEN71_07785	paras_002110	protein-codi	NZ_CP1252	chromosom	1724391	1725791	+	1401	0	10	993	9	956	
QEN71_RS07790	GMC family oxidoreductase N-terminal domain-cont	QEN71_07790	paras_002111	protein-codi	NZ_CP1252	chromosom	1725846	1729244	-	3399	0	49	4641	36	3439	
QEN71_RS07795	hypothetical protein	QEN71_07795	paras_002112	protein-codi	NZ_CP1252	chromosom	1729493	1730305	-	813	0	21	5772	21	5772	
QEN71_RS07800	hybrid sensor histidine kinase/response regulator	QEN71_07800	paras_002113	protein-codi	NZ_CP1252	chromosom	1730635	1732452	+	1818	0	28	5785	17	3533	
QEN71_RS07805	hypothetical protein	QEN71_07805	paras_002114	protein-codi	NZ_CP1252	chromosom	1732714	1733031	+	318	0	3	490	2	381	
QEN71_RS07810	response regulator transcription factor	QEN71_07810	paras_002115	protein-codi	NZ_CP1252	chromosom	1733107	1733862	-	756	0	13	3265	6	2244	
QEN71_RS07815	hypothetical protein	QEN71_07815	paras_002116	protein-codi	NZ_CP1252	chromosom	1734474	1734641	-	145	0	5	1244	3	525	
QEN71_RS07820	cytochrome d ubiquinol oxidase subunit II	QEN71_07820	paras_002117	protein-codi	NZ_CP1252	chromosom	1734619	1735620	-	979	0	21	3553	10	927	

QEN71_RS07825	cytochrome ubiquinol oxidase subunit I	QEN71_07825	paras_002118	protein-codi	NZ_CP1252	chromosom	1735657	1737051	-	1395	0	35	7637	28	5680	
QEN71_RS07830	hypothetical protein	QEN71_07830	paras_002119	protein-codi	NZ_CP1252	chromosom	1737175	1737351	-	177	0	3	339	1	63	
QEN71_RS07835	4-oxalocrotonate tautomerase	QEN71_07835	paras_002120	protein-codi	NZ_CP1252	chromosom	1737933	1738121	-	189	0	12	1635	10	1429	
QEN71_RS07840	class II aldolase/adducin family protein	QEN71_07840	paras_002121	protein-codi	NZ_CP1252	chromosom	1738153	1738932	-	780	0	16	4959	16	4959	
QEN71_RS07845	urea transporter	QEN71_07845	paras_002122	protein-codi	NZ_CP1252	chromosom	1738955	1739902	-	948	0	24	4409	23	4154	
QEN71_RS07850	putative glycolipid-binding domain-containing protei	QEN71_07850	paras_002123	protein-codi	NZ_CP1252	chromosom	1740096	1740650	+	515	0	9	1584	9	1584	
QEN71_RS07855	alpha/beta fold hydrolase	QEN71_07855	paras_002124	protein-codi	NZ_CP1252	chromosom	1740611	1741531	-	877	0	13	2488	10	1750	
QEN71_RS07860	metal-dependent hydrolase	QEN71_07860	paras_002125	protein-codi	NZ_CP1252	chromosom	1741528	1742409	-	878	0	13	1337	13	1337	
QEN71_RS07865	SDR family NAD(P)-dependent oxidoreductase	QEN71_07865	paras_002126	protein-codi	NZ_CP1252	chromosom	1742432	1743340	-	909	0	11	1872	8	1668	
QEN71_RS07870	NAD(P)/FAD-dependent oxidoreductase	QEN71_07870	paras_002127	protein-codi	NZ_CP1252	chromosom	1743371	1744933	-	1563	0	29	3513	22	2822	
QEN71_RS07875	MerR family transcriptional regulator	QEN71_07875	paras_002128	protein-codi	NZ_CP1252	chromosom	1745188	1746048	-	861	0	17	1737	14	1504	
QEN71_RS07880	GNAT family N-acetyltransferase	QEN71_07880	paras_002129	protein-codi	NZ_CP1252	chromosom	1746096	1746644	-	549	0	13	3194	11	3089	
QEN71_RS07885	aromatic acid exporter family protein	QEN71_07885	paras_002130	protein-codi	NZ_CP1252	chromosom	1746924	1748036	+	1113	0	15	2495	11	1612	
QEN71_RS07890	thioesterase family protein	QEN71_07890	paras_002131	protein-codi	NZ_CP1252	chromosom	1748156	1748710	+	555	0	6	473	6	473	
QEN71_RS07895	hypothetical protein	QEN71_07895	paras_002132	protein-codi	NZ_CP1252	chromosom	1748761	1748973	-	213	0	0	0	0	0	
QEN71_RS07900	CerR family C-terminal domain-containing protein	QEN71_07900	paras_002133	protein-codi	NZ_CP1252	chromosom	1749235	1749969	-	735	0	15	2821	13	2617	
QEN71_RS07905	HlyD family secretion protein	QEN71_07905	paras_002134	protein-codi	NZ_CP1252	chromosom	1750104	1751264	+	1153	0	20	2967	19	2850	
QEN71_RS07910	MDR family MFS transporter	QEN71_07910	paras_002135	protein-codi	NZ_CP1252	chromosom	1751257	1752861	+	1597	0	23	4404	19	3590	
QEN71_RS07915	TolC family protein	QEN71_07915	paras_002136	protein-codi	NZ_CP1252	chromosom	1752870	1754384	+	1515	0	12	1868	7	1386	
QEN71_RS07920	TAXI family TRAP transporter solute-binding subuni	QEN71_07920	paras_002137	protein-codi	NZ_CP1252	chromosom	1754468	1755583	-	1116	0	26	3883	20	3248	
QEN71_RS07925	zinc ribbon domain-containing protein	QEN71_07925	paras_002138	protein-codi	NZ_CP1252	chromosom	1755702	1756838	-	1137	0	24	2656	18	1940	
QEN71_RS07930	MDR family MFS transporter	QEN71_07930	paras_002139	protein-codi	NZ_CP1252	chromosom	1757274	1758782	-	1509	0	39	7119	29	5962	
QEN71_RS07935	TetR/AcrR family transcriptional regulator	QEN71_07935	paras_002140	protein-codi	NZ_CP1252	chromosom	1758872	1759600	+	729	0	9	2190	9	2190	
QEN71_RS07940	response regulator	QEN71_07940	paras_002141	protein-codi	NZ_CP1252	chromosom	1759995	1760459	+	465	0	10	2377	4	1428	
QEN71_RS07945	Fur family transcriptional regulator	QEN71_07945	paras_002142	protein-codi	NZ_CP1252	chromosom	1760501	1761082	-	582	0	8	1179	8	1179	
QEN71_RS07950	glutathione binding-like protein	QEN71_07950	paras_002143	protein-codi	NZ_CP1252	chromosom	1761137	1761763	-	627	0	16	5150	11	4231	
QEN71_RS07955	glutathione S-transferase	QEN71_07955	paras_002144	protein-codi	NZ_CP1252	chromosom	1761768	1762394	-	627	0	27	6564	18	3007	
QEN71_RS07960	ATP-binding domain-containing protein	QEN71_07960	paras_002145	protein-codi	NZ_CP1252	chromosom	1762553	1764226	+	1674	0	42	18636	38	17846	
QEN71_RS07965	phosphoribosyltransferase	QEN71_07965	paras_002146	protein-codi	NZ_CP1252	chromosom	1764265	1765212	-	948	0	28	7913	18	3582	
QEN71_RS07970	molybdopterin-dependent oxidoreductase	QEN71_07970	paras_002147	protein-codi	NZ_CP1252	chromosom	1765419	1765943	+	494	0	18	4911	16	4551	
QEN71_RS07975	ATP-binding protein	QEN71_07975	paras_002148	protein-codi	NZ_CP1252	chromosom	1765913	1767766	+	1823	0	36	9997	21	6021	
QEN71_RS07980	helix-turn-helix domain-containing protein	QEN71_07980	paras_002149	protein-codi	NZ_CP1252	chromosom	1767798	1768652	-	855	0	16	2430	9	1518	
QEN71_RS07985	FAD-binding oxidoreductase	QEN71_07985	paras_002150	protein-codi	NZ_CP1252	chromosom	1769111	1770451	+	1341	0	35	9144	17	3922	
QEN71_RS07990	GntR family transcriptional regulator	QEN71_07990	paras_002151	protein-codi	NZ_CP1252	chromosom	1771173	1771913	-	741	0	25	5027	16	3628	
QEN71_RS07995	proline racemase family protein	QEN71_07995	paras_002152	protein-codi	NZ_CP1252	chromosom	1772107	1773114	+	1008	0	28	5578	19	4322	
QEN71_RS08000	delta(1)-pyrroline-2-carboxylate reductase family pr	QEN71_08000	paras_002153	protein-codi	NZ_CP1252	chromosom	1773138	1774055	+	918	0	13	1842	13	1842	
QEN71_RS08005	glucose-6-phosphate dehydrogenase	QEN71_08005	paras_002154	protein-codi	NZ_CP1252	chromosom	1774269	1775753	+	1485	0	43	5810	32	4298	
QEN71_RS08010	type VI secretion system tube protein Hcp	QEN71_08010	paras_002155	protein-codi	NZ_CP1252	chromosom	1775894	1776361	-	457	0	22	2270	19	2208	
QEN71_RS08015	hypothetical protein	QEN71_08015	paras_002156	protein-codi	NZ_CP1252	chromosom	1776351	1776623	-	258	0	8	553	7	547	
QEN71_RS08020	DUF2778 domain-containing protein	QEN71_08020	paras_002157	protein-codi	NZ_CP1252	chromosom	1776620	1777066	-	443	0	15	1224	13	1142	
QEN71_RS08025	hypothetical protein	QEN71_08025	paras_002158	protein-codi	NZ_CP1252	chromosom	1777334	1777588	-	255	0	12	1912	6	791	
QEN71_RS08030	GlxA family transcriptional regulator	QEN71_08030	paras_002159	protein-codi	NZ_CP1252	chromosom	1777718	1778803	-	1086	0	14	1282	11	1060	
QEN71_RS08035	L-serine ammonia-lyase	QEN71_08035	paras_002160	protein-codi	NZ_CP1252	chromosom	1779241	1780629	+	1389	0	23	3123	16	2362	
QEN71_RS08040	sarcosine oxidase subunit beta family protein	QEN71_08040	paras_002161	protein-codi	NZ_CP1252	chromosom	1780667	1781911	+	1245	0	17	1182	12	884	
QEN71_RS08045	sarcosine oxidase subunit delta	QEN71_08045	paras_002162	protein-codi	NZ_CP1252	chromosom	1781991	1782290	+	296	0	6	602	6	602	
QEN71_RS08050	sarcosine oxidase subunit alpha family protein	QEN71_08050	paras_002163	protein-codi	NZ_CP1252	chromosom	1782287	1785286	+	2985	0	44	5861	32	4074	
QEN71_RS08055	sarcosine oxidase subunit gamma	QEN71_08055	paras_002164	protein-codi	NZ_CP1252	chromosom	1785276	1785926	+	636	0	9	1199	7	798	
QEN71_RS08060	dihydroneopterin aldolase	QEN71_08060	paras_002165	protein-codi	NZ_CP1252	chromosom	1785923	1786369	+	443	0	10	1324	10	1324	
QEN71_RS08065	hypothetical protein	QEN71_08065		protein-codi	NZ_CP1252	chromosom	1786384	1786650	+	267	0	8	1399	6	1161	
QEN71_RS08070	LysR family transcriptional regulator	QEN71_08070	paras_002167	protein-codi	NZ_CP1252	chromosom	1787327	1788289	-	963	0	15	1136	15	1136	
QEN71_RS08075	NAD(P)-dependent alcohol dehydrogenase	QEN71_08075	paras_002168	protein-codi	NZ_CP1252	chromosom	1788476	1789528	-	1053	0	18	2158	9	945	
QEN71_RS08080	AraC family transcriptional regulator	QEN71_08080	paras_002169	protein-codi	NZ_CP1252	chromosom	1789919	1790902	+	984	0	17	2380	15	2219	
QEN71_RS08085	VOC family protein	QEN71_08085	paras_002170	protein-codi	NZ_CP1252	chromosom	1790925	1791317	-	393	0	7	710	7	710	
QEN71_RS08090	hypothetical protein	QEN71_08090	paras_002171	protein-codi	NZ_CP1252	chromosom	1791457	1791771	+	315	0	8	1599	4	570	
QEN71_RS08095	GGDEF domain-containing protein	QEN71_08095	paras_002172	protein-codi	NZ_CP1252	chromosom	1791802	1793262	-	1461	0	31	3931	23	2608	
QEN71_RS08100	polysaccharide pyruvyl transferase family protein	QEN71_08100	paras_002173	protein-codi	NZ_CP1252	chromosom	1793943	1795145	-	1203	0	14	1088	13	1079	
QEN71_RS08105	hypothetical protein	QEN71_08105	paras_002174	protein-codi	NZ_CP1252	chromosom	1795536	1795748	+	213	0	8	1076	3	551	
QEN71_RS08110	alpha/beta hydrolase	QEN71_08110	paras_002175	protein-codi	NZ_CP1252	chromosom	1795992	1796813	+	822	0	14	916	8	603	
QEN71_RS08115	DUF2164 domain-containing protein	QEN71_08115	paras_002176	protein-codi	NZ_CP1252	chromosom	1796921	1797184	+	264	0	3	150	3	150	
QEN71_RS08120	LysR family transcriptional regulator	QEN71_08120	paras_002177	protein-codi	NZ_CP1252	chromosom	1797201	1798112	-	912	0	9	446	6	406	
QEN71_RS08125	aldo/keto reductase	QEN71_08125	paras_002178	protein-codi	NZ_CP1252	chromosom	1798241	1799254	+	1014	0	21	5062	13	1842	
QEN71_RS08130	IS481 family transposase	QEN71_08130	paras_002179	protein-codi	NZ_CP1252	chromosom	1799850	1800989	+	1140	2266.0	2	16	2	16	

QEN71_RS08135	hypothetical protein	QEN71_08135	paras_002180	protein-codi	NZ_CP1252	chromosom	1801116	1801268	+	153	190.0	3	106	0	0	
QEN71_RS08140	2-hydroxyacid dehydrogenase	QEN71_08140	paras_002181	protein-codi	NZ_CP1252	chromosom	1801288	1802220	+	933	0	9	356	7	335	
QEN71_RS08145	OsmC family protein	QEN71_08145	paras_002182	protein-codi	NZ_CP1252	chromosom	1802248	1802727	+	480	0	7	644	7	644	
QEN71_RS08150	sigma 54-interacting transcriptional regulator	QEN71_08150	paras_002183	protein-codi	NZ_CP1252	chromosom	1803484	1804827	+	1344	0	25	2567	19	2288	
QEN71_RS08155	metallophosphoesterase	QEN71_08155	paras_002184	protein-codi	NZ_CP1252	chromosom	1805080	1806279	+	1196	0	17	2425	11	1213	
QEN71_RS08160	hypothetical protein	QEN71_08160	paras_002185	protein-codi	NZ_CP1252	chromosom	1806276	1806488	-	209	0	3	936	3	936	
QEN71_RS08165	MFS transporter	QEN71_08165	paras_002186	protein-codi	NZ_CP1252	chromosom	1806741	1808165	-	1425	0	18	2109	18	2109	
QEN71_RS08170	choline dehydrogenase	QEN71_08170	paras_002187	protein-codi	NZ_CP1252	chromosom	1808342	1810060	-	1719	0	39	3374	31	2625	
QEN71_RS08175	betaine-aldehyde dehydrogenase	QEN71_08175	paras_002188	protein-codi	NZ_CP1252	chromosom	1810072	1811541	-	1470	0	18	1820	13	1473	
QEN71_RS08180	transcriptional regulator BetI	QEN71_08180	paras_002189	protein-codi	NZ_CP1252	chromosom	1811585	1812175	-	591	0	7	629	3	202	
QEN71_RS08185	formaldehyde dehydrogenase, glutathione-indepen	QEN71_08185	paras_002190	protein-codi	NZ_CP1252	chromosom	1812372	1813571	+	1200	0	24	3414	21	3371	
QEN71_RS08190	hypothetical protein	QEN71_08190	paras_002191	protein-codi	NZ_CP1252	chromosom	1813717	1814253	-	537	0	16	3538	14	2943	
QEN71_RS08195	AMP-binding protein	QEN71_08195	paras_002192	protein-codi	NZ_CP1252	chromosom	1814500	1816188	+	1689	0	48	7036	35	4935	
QEN71_RS08200	formylglycine-generating enzyme family protein	QEN71_08200	paras_002193	protein-codi	NZ_CP1252	chromosom	1816250	1817371	-	1122	0	24	1979	22	1670	
QEN71_RS08205	sulfatase-like hydrolase/transferase	QEN71_08205	paras_002194	protein-codi	NZ_CP1252	chromosom	1817429	1819087	-	1659	0	31	3929	26	3457	
QEN71_RS08210	SDR family oxidoreductase	QEN71_08210	paras_002195	protein-codi	NZ_CP1252	chromosom	1819331	1820116	+	786	0	9	1872	5	1044	
QEN71_RS08215	DUF1254 domain-containing protein	QEN71_08215	paras_002196	protein-codi	NZ_CP1252	chromosom	1820157	1821626	+	1470	0	36	3939	34	3804	
QEN71_RS08220	LuxR C-terminal-related transcriptional regulator	QEN71_08220	paras_002197	protein-codi	NZ_CP1252	chromosom	1821646	1824348	-	2703	0	30	1428	22	1135	
QEN71_RS08225	enoyl-CoA hydratase/isomerase family protein	QEN71_08225	paras_002198	protein-codi	NZ_CP1252	chromosom	1824529	1825299	+	771	0	3	397	1	248	TRUE
QEN71_RS08230	thiolase family protein	QEN71_08230	paras_002199	protein-codi	NZ_CP1252	chromosom	1825340	1826581	+	1242	0	14	1271	11	1070	
QEN71_RS08235	acyl-CoA dehydrogenase family protein	QEN71_08235	paras_002200	protein-codi	NZ_CP1252	chromosom	1826610	1827752	+	1143	0	8	431	7	429	
QEN71_RS08240	lipid-transfer protein	QEN71_08240	paras_002201	protein-codi	NZ_CP1252	chromosom	1827974	1829155	+	1182	0	23	2997	19	2156	
QEN71_RS08245	acyl-CoA dehydrogenase family protein	QEN71_08245	paras_002202	protein-codi	NZ_CP1252	chromosom	1829166	1830365	+	1200	0	9	1234	6	1011	
QEN71_RS08250	acyl-CoA dehydrogenase family protein	QEN71_08250	paras_002203	protein-codi	NZ_CP1252	chromosom	1830381	1831535	+	1151	0	16	1028	9	568	
QEN71_RS08255	MaoC family dehydratase	QEN71_08255	paras_002204	protein-codi	NZ_CP1252	chromosom	1831532	1831987	+	452	0	1	54	1	54	TRUE
QEN71_RS08260	SDR family NAD(P)-dependent oxidoreductase	QEN71_08260	paras_002205	protein-codi	NZ_CP1252	chromosom	1832024	1832923	+	900	0	12	1115	8	745	
QEN71_RS08265	acyl-CoA dehydrogenase family protein	QEN71_08265	paras_002206	protein-codi	NZ_CP1252	chromosom	1832996	1834141	-	1146	0	13	435	10	243	
QEN71_RS08270	CaiB/BaiF CoA-transferase family protein	QEN71_08270	paras_002207	protein-codi	NZ_CP1252	chromosom	1834202	1835314	-	1113	0	11	612	11	612	
QEN71_RS08275	porin	QEN71_08275	paras_002208	protein-codi	NZ_CP1252	chromosom	1835554	1836669	+	1116	0	26	3293	22	2780	
QEN71_RS08280	SDR family NAD(P)-dependent oxidoreductase	QEN71_08280	paras_002209	protein-codi	NZ_CP1252	chromosom	1836689	1837435	+	747	0	4	262	2	212	
QEN71_RS08285	toll/interleukin-1 receptor domain-containing protein	QEN71_08285	paras_002210	protein-codi	NZ_CP1252	chromosom	1837488	1838699	-	1212	0	23	1819	22	1811	
QEN71_RS08290	toll/interleukin-1 receptor domain-containing protein	QEN71_08290	paras_002211	protein-codi	NZ_CP1252	chromosom	1838794	1840239	+	1446	0	22	1845	18	1696	
QEN71_RS08295	hypothetical protein	QEN71_08295	paras_002212	protein-codi	NZ_CP1252	chromosom	1840244	1844290	+	4047	0	57	6633	44	5007	
QEN71_RS08300	adenosylcobalamin-dependent ribonucleoside-diph	QEN71_08300	paras_002213	protein-codi	NZ_CP1252	chromosom	1844355	1846898	-	2544	0	37	4864	31	4004	
QEN71_RS08305	GlxA family transcriptional regulator	QEN71_08305	paras_002214	protein-codi	NZ_CP1252	chromosom	1847808	1848824	-	1017	0	24	4869	24	4869	
QEN71_RS08310	serine hydroxymethyltransferase	QEN71_08310	paras_002215	protein-codi	NZ_CP1252	chromosom	1849341	1850615	+	1275	0	7	496	7	496	
QEN71_RS08315	dipeptidase	QEN71_08315	paras_002216	protein-codi	NZ_CP1252	chromosom	1850660	1851631	+	972	0	12	531	11	372	
QEN71_RS08320	DUF5943 domain-containing protein	QEN71_08320	paras_002217	protein-codi	NZ_CP1252	chromosom	1851665	1852198	+	534	0	10	469	8	416	
QEN71_RS08325	NADH:flavin oxidoreductase	QEN71_08325	paras_002218	protein-codi	NZ_CP1252	chromosom	1852254	1854317	+	2064	0	14	595	10	406	
QEN71_RS08330	(Fe-S)-binding protein	QEN71_08330	paras_002219	protein-codi	NZ_CP1252	chromosom	1854320	1856239	+	1920	0	20	2066	18	2038	
QEN71_RS08335	electron transfer flavoprotein subunit alpha/FixB fan	QEN71_08335	paras_002220	protein-codi	NZ_CP1252	chromosom	1856243	1857421	+	1175	0	14	1044	8	429	
QEN71_RS08340	electron transfer flavoprotein subunit beta/FixA fami	QEN71_08340	paras_002221	protein-codi	NZ_CP1252	chromosom	1857418	1858197	+	776	0	15	530	5	261	
QEN71_RS08345	aromatic ring-hydroxylating dioxygenase subunit alp	QEN71_08345	paras_002222	protein-codi	NZ_CP1252	chromosom	1858239	1859519	+	1281	0	20	1811	13	1106	
QEN71_RS08350	hybrid-cluster NAD(P)-dependent oxidoreductase	QEN71_08350	paras_002223	protein-codi	NZ_CP1252	chromosom	1859572	1860723	+	1152	0	25	2388	19	2104	
QEN71_RS08355	glycine betaine ABC transporter substrate-binding p	QEN71_08355	paras_002224	protein-codi	NZ_CP1252	chromosom	1861487	1862350	+	864	0	11	1079	9	1061	
QEN71_RS08360	APC family permease	QEN71_08360	paras_002225	protein-codi	NZ_CP1252	chromosom	1862487	1864154	+	1668	0	26	3781	21	2715	
QEN71_RS08365	formyltetrahydrofolate deformylase	QEN71_08365	paras_002226	protein-codi	NZ_CP1252	chromosom	1864230	1865108	-	879	0	11	1364	9	1080	
QEN71_RS08370	LysR family transcriptional regulator	QEN71_08370	paras_002227	protein-codi	NZ_CP1252	chromosom	1865257	1866183	-	927	0	13	1127	12	1101	
QEN71_RS08375	choline-sulfatase	QEN71_08375	paras_002228	protein-codi	NZ_CP1252	chromosom	1866327	1867868	+	1542	0	42	5578	31	3871	
QEN71_RS08380	choline ABC transporter substrate-binding protein	QEN71_08380	paras_002229	protein-codi	NZ_CP1252	chromosom	1867951	1868874	+	924	0	24	3861	18	3575	
QEN71_RS08385	porin	QEN71_08385	paras_002230	protein-codi	NZ_CP1252	chromosom	1869115	1870257	+	1143	0	49	8660	43	7227	
QEN71_RS08390	GlxA family transcriptional regulator	QEN71_08390	paras_002231	protein-codi	NZ_CP1252	chromosom	1870326	1871318	-	993	0	17	2388	17	2388	
QEN71_RS08395	choline ABC transporter substrate-binding protein	QEN71_08395	paras_002232	protein-codi	NZ_CP1252	chromosom	1871615	1872565	+	951	0	20	2958	16	2117	
QEN71_RS08400	3-keto-5-aminohexanoate cleavage protein	QEN71_08400	paras_002233	protein-codi	NZ_CP1252	chromosom	1872651	1873580	+	930	0	8	2982	7	2804	
QEN71_RS08405	L-carnitine dehydrogenase	QEN71_08405	paras_002234	protein-codi	NZ_CP1252	chromosom	1873632	1874597	+	966	0	13	3539	9	2973	
QEN71_RS08410	thioesterase family protein	QEN71_08410	paras_002235	protein-codi	NZ_CP1252	chromosom	1874685	1875176	+	492	0	20	4414	15	3700	
QEN71_RS08415	alpha/beta hydrolase	QEN71_08415	paras_002236	protein-codi	NZ_CP1252	chromosom	1875200	1876135	+	936	0	17	2459	17	2459	
QEN71_RS08420	DUF3331 domain-containing protein	QEN71_08420	paras_002237	protein-codi	NZ_CP1252	chromosom	1876183	1876470	-	288	0	9	1579	3	837	
QEN71_RS08425	AI-2E family transporter	QEN71_08425	paras_002238	protein-codi	NZ_CP1252	chromosom	1876848	1877933	+	1086	0	10	4140	6	1597	
QEN71_RS08430	OpgC domain-containing protein	QEN71_08430	paras_002239	protein-codi	NZ_CP1252	chromosom	1877967	1879079	-	1113	0	29	5016	21	3462	
QEN71_RS08435	UDP-glucose/GDP-mannose dehydrogenase family	QEN71_08435	paras_002240	protein-codi	NZ_CP1252	chromosom	1879503	1880930	+	1428	324.0	17	3514	13	2188	
QEN71_RS08440	low molecular weight protein-tyrosine-phosphatase	QEN71_08440	paras_002241	protein-codi	NZ_CP1252	chromosom	1881001	1881444	+	437	0	10	705	3	36	

QEN71_RS08445	polysaccharide biosynthesis/export family protein	QEN71_08445	paras_002242	protein-codi	NZ_CP1252	chromosom	1881438	1882613	+	1169	0	24	1263	19	858	
QEN71_RS08450	polysaccharide biosynthesis tyrosine autokinase	QEN71_08450	paras_002243	protein-codi	NZ_CP1252	chromosom	1882727	1884940	+	2214	0	29	1060	21	760	
QEN71_RS08455	glycosyltransferase	QEN71_08455	paras_002244	protein-codi	NZ_CP1252	chromosom	1884964	1885926	+	963	0	24	893	22	855	
QEN71_RS08460	glucose-6-phosphate isomerase	QEN71_08460	paras_002245	protein-codi	NZ_CP1252	chromosom	1886055	1887479	+	1425	0	50	3610	40	3286	
QEN71_RS08465	glycosyltransferase family 4 protein	QEN71_08465	paras_002246	protein-codi	NZ_CP1252	chromosom	1887640	1888812	+	1173	204.0	10	717	7	575	
QEN71_RS08470	flippase	QEN71_08470	paras_002247	protein-codi	NZ_CP1252	chromosom	1889182	1890660	+	1475	575.0	28	1793	22	1577	
QEN71_RS08475	glycosyltransferase family 4 protein	QEN71_08475	paras_002248	protein-codi	NZ_CP1252	chromosom	1890657	1893158	+	2498	2213.0	26	1466	21	1197	
QEN71_RS08480	mannose-1-phosphate guanylyltransferase/mannos	QEN71_08480	paras_002249	protein-codi	NZ_CP1252	chromosom	1893203	1894735	+	1533	0	25	1932	16	1168	
QEN71_RS08485	glycosyltransferase family 4 protein	QEN71_08485	paras_002250	protein-codi	NZ_CP1252	chromosom	1894830	1895918	+	1089	0	31	1549	25	1316	
QEN71_RS08490	IS30 family transposase	partial;pseudo;QEN71_08490		pseudogene	NZ_CP1252	chromosom	1895966	1896650	-	685	0	15	1276	15	1276	
QEN71_RS08495	acyltransferase family protein	QEN71_08495	paras_002252	protein-codi	NZ_CP1252	chromosom	1896758	1897738	-	950	0	28	1847	20	1432	
QEN71_RS08500	IS5 family transposase	QEN71_08500	paras_002253	pseudogene	NZ_CP1252	chromosom	1897708	1898519	-	781	0	7	598	7	598	
QEN71_RS08505	undecaprenyl-phosphate glucose phosphotransfera	QEN71_08505	paras_002254	protein-codi	NZ_CP1252	chromosom	1899389	1900759	-	1371	0	20	282	15	221	
QEN71_RS08510	helix-turn-helix domain-containing protein	QEN71_08510	paras_002255	protein-codi	NZ_CP1252	chromosom	1900763	1901764	-	1002	0	7	300	5	234	
QEN71_RS08515	porin	QEN71_08515	paras_002256	protein-codi	NZ_CP1252	chromosom	1902245	1903396	+	1152	0	39	3577	39	3577	
QEN71_RS08520	NUDIX domain-containing protein	QEN71_08520	paras_002257	protein-codi	NZ_CP1252	chromosom	1903505	1904092	-	588	0	17	1995	13	1456	
QEN71_RS08525	DeoR/GlpR family DNA-binding transcription regula	QEN71_08525	paras_002258	protein-codi	NZ_CP1252	chromosom	1904253	1905008	+	756	0	10	3071	10	3071	
QEN71_RS08530	SRPBCC family protein	QEN71_08530	paras_002259	protein-codi	NZ_CP1252	chromosom	1905026	1905433	-	408	0	3	704	2	647	
QEN71_RS08535	peptidoglycan DD-metalloendopeptidase family prot	QEN71_08535	paras_002260	protein-codi	NZ_CP1252	chromosom	1905841	1906827	+	987	0	18	5579	14	2643	
QEN71_RS08540	alpha/beta hydrolase	QEN71_08540	paras_002261	protein-codi	NZ_CP1252	chromosom	1906901	1907731	-	831	0	24	6580	22	6004	
QEN71_RS08545	hypothetical protein	QEN71_08545	paras_002262	protein-codi	NZ_CP1252	chromosom	1907799	1908206	-	408	0	14	2031	14	2031	
QEN71_RS08550	cellulose biosynthesis protein BcsD	QEN71_08550	paras_002263	protein-codi	NZ_CP1252	chromosom	1908547	1909011	+	465	0	17	4303	11	3016	
QEN71_RS08555	cellulose biosynthesis protein BcsP	QEN71_08555	paras_002264	protein-codi	NZ_CP1252	chromosom	1909128	1909940	+	809	0	12	1797	10	1752	
QEN71_RS08560	cellulose biosynthesis protein BcsQ	QEN71_08560	paras_002265	protein-codi	NZ_CP1252	chromosom	1909937	1910725	+	781	0	16	3080	16	3080	
QEN71_RS08565	UDP-forming cellulose synthase catalytic subunit	QEN71_08565	paras_002266	protein-codi	NZ_CP1252	chromosom	1910722	1912953	+	2228	0	49	8423	45	7886	
QEN71_RS08570	cellulose biosynthesis cyclic di-GMP-binding regula	QEN71_08570	paras_002267	protein-codi	NZ_CP1252	chromosom	1912957	1915521	+	2565	0	40	6407	30	4313	
QEN71_RS08575	cellulose synthase complex periplasmic endoglucan	QEN71_08575	paras_002268	protein-codi	NZ_CP1252	chromosom	1915610	1916740	+	1103	0	25	5007	23	4931	
QEN71_RS08580	cellulose synthase subunit BcsC-related outer mem	QEN71_08580	paras_002269	protein-codi	NZ_CP1252	chromosom	1916713	1921407	+	4667	0	128	19331	93	14624	
QEN71_RS08585	(2Fe-2S)-binding protein	QEN71_08585	paras_002270	protein-codi	NZ_CP1252	chromosom	1921686	1922141	+	456	0	6	626	6	626	
QEN71_RS08590	xanthine dehydrogenase family protein molybdopter	QEN71_08590	paras_002271	protein-codi	NZ_CP1252	chromosom	1922143	1924380	+	2238	0	26	3235	17	1802	
QEN71_RS08595	cytochrome c	QEN71_08595	paras_002272	protein-codi	NZ_CP1252	chromosom	1924392	1925654	+	1263	0	44	3105	40	3032	
QEN71_RS08600	amino acid ABC transporter permease	QEN71_08600	paras_002273	protein-codi	NZ_CP1252	chromosom	1926292	1926948	-	653	0	18	929	15	843	
QEN71_RS08605	amino acid ABC transporter permease	QEN71_08605	paras_002274	protein-codi	NZ_CP1252	chromosom	1926945	1927694	-	746	0	21	2870	16	1056	
QEN71_RS08610	transporter substrate-binding domain-containing pro	QEN71_08610	paras_002275	protein-codi	NZ_CP1252	chromosom	1927737	1928546	-	810	0	14	1906	14	1906	
QEN71_RS08615	LysR substrate-binding domain-containing protein	QEN71_08615	paras_002276	protein-codi	NZ_CP1252	chromosom	1928816	1929691	+	876	0	16	2188	13	1466	
QEN71_RS08620	ionic transporter y4hA	QEN71_08620	paras_002277	protein-codi	NZ_CP1252	chromosom	1929711	1930799	-	1089	0	17	3397	12	2646	
QEN71_RS08625	BON domain-containing protein	QEN71_08625	paras_002278	protein-codi	NZ_CP1252	chromosom	1930975	1931331	-	357	0	8	4504	3	1235	
QEN71_RS08630	DNA-3-methyladenine glycosylase	QEN71_08630	paras_002279	protein-codi	NZ_CP1252	chromosom	1931698	1932330	+	633	0	34	15495	27	13434	
QEN71_RS08635	DUF4148 domain-containing protein	QEN71_08635	paras_002280	protein-codi	NZ_CP1252	chromosom	1932541	1932867	+	327	0	7	1546	4	837	
QEN71_RS08640	CBS domain-containing protein	QEN71_08640	paras_002281	protein-codi	NZ_CP1252	chromosom	1933131	1933565	-	435	0	17	5039	10	3146	
QEN71_RS08645	GTP cyclohydrolase II	QEN71_08645	paras_002282	protein-codi	NZ_CP1252	chromosom	1933912	1934553	+	642	0	21	1635	13	730	
QEN71_RS08650	cysteine hydrolase family protein	QEN71_08650	paras_002283	protein-codi	NZ_CP1252	chromosom	1934654	1935199	-	546	0	15	2178	12	1998	
QEN71_RS08655	hypothetical protein	QEN71_08655	paras_002284	protein-codi	NZ_CP1252	chromosom	1935351	1935638	-	288	0	23	5619	20	4801	
QEN71_RS08660	NmrA family NAD(P)-binding protein	QEN71_08660	paras_002285	protein-codi	NZ_CP1252	chromosom	1935896	1936777	-	882	0	18	3843	13	2573	
QEN71_RS08665	AraC family transcriptional regulator	QEN71_08665	paras_002286	protein-codi	NZ_CP1252	chromosom	1936970	1937860	+	891	0	10	1590	8	1279	
QEN71_RS08670	FAD-dependent oxidoreductase	QEN71_08670	paras_002287	protein-codi	NZ_CP1252	chromosom	1937869	1940352	-	2484	0	56	13374	41	10972	
QEN71_RS08675	DeoR/GlpR family DNA-binding transcription regula	QEN71_08675	paras_002288	protein-codi	NZ_CP1252	chromosom	1940575	1941369	+	795	0	6	565	3	95	
QEN71_RS08680	response regulator transcription factor	QEN71_08680	paras_002290	protein-codi	NZ_CP1252	chromosom	1942225	1942911	+	687	0	15	3136	11	2483	
QEN71_RS08685	5,6-dimethylbenzimidazole synthase	QEN71_08685	paras_002291	protein-codi	NZ_CP1252	chromosom	1942932	1943636	-	688	0	20	3070	13	2118	
QEN71_RS08690	cobyrinate a,c-diamide synthase	QEN71_08690	paras_002292	protein-codi	NZ_CP1252	chromosom	1943620	1944924	-	1288	0	20	6676	17	4440	
QEN71_RS08695	cob(I)yrinic acid a,c-diamide adenosyltransferase	QEN71_08695	paras_002293	protein-codi	NZ_CP1252	chromosom	1944928	1945530	-	599	0	11	1430	11	1430	
QEN71_RS08700	cobalamin biosynthesis protein	QEN71_08700	paras_002294	protein-codi	NZ_CP1252	chromosom	1945527	1945946	-	412	0	2	672	2	672	
QEN71_RS08705	uroporphyrinogen-III C-methyltransferase	QEN71_08705	paras_002295	protein-codi	NZ_CP1252	chromosom	1945943	1946659	-	713	0	11	3238	9	2640	
QEN71_RS08710	cobalamin biosynthesis protein CobW	QEN71_08710	paras_002296	protein-codi	NZ_CP1252	chromosom	1947576	1948673	+	1098	0	8	1197	7	1092	
QEN71_RS08715	cobaltochelatase subunit CobN	QEN71_08715	paras_002297	protein-codi	NZ_CP1252	chromosom	1948683	1952465	+	3779	0	81	10806	67	9422	
QEN71_RS08720	ATP-binding protein	QEN71_08720	paras_002298	protein-codi	NZ_CP1252	chromosom	1952462	1953493	+	1028	0	12	1615	9	1428	
QEN71_RS08725	VWA domain-containing protein	QEN71_08725	paras_002299	protein-codi	NZ_CP1252	chromosom	1953523	1954167	+	645	0	2	56	2	56	
QEN71_RS08730	YadA-like family protein	QEN71_08730	paras_002300	protein-codi	NZ_CP1252	chromosom	1955067	1962530	+	7464	0	178	31714	145	27548	
QEN71_RS08735	OmpA family protein	QEN71_08735	paras_002301	protein-codi	NZ_CP1252	chromosom	1962582	1963721	+	1140	0	13	1978	9	1700	
QEN71_RS08740	flagellar brake protein	QEN71_08740	paras_002302	protein-codi	NZ_CP1252	chromosom	1963834	1964784	-	951	0	9	1472	5	531	
QEN71_RS08745	precorrin-3B C(17)-methyltransferase	QEN71_08745	paras_002303	protein-codi	NZ_CP1252	chromosom	1964950	1966677	-	1715	0	36	8775	21	6869	
QEN71_RS08750	precorrin-2 C(20)-methyltransferase	QEN71_08750	paras_002304	protein-codi	NZ_CP1252	chromosom	1966665	1967420	-	743	0	20	9120	17	8581	

QEN71_RS08755	precorrin-8X methylmutase	QEN71_08755	paras_002305	protein-codi	NZ_CP1252	chromosom	1967421	1968047	-	619	0	7	1362	2	99	
QEN71_RS08760	precorrin-3B synthase	QEN71_08760	paras_002306	protein-codi	NZ_CP1252	chromosom	1968040	1969401	-	1354	0	25	5072	23	4339	
QEN71_RS08765	precorrin-6y C5,15-methyltransferase (decarboxylat	QEN71_08765	paras_002307	protein-codi	NZ_CP1252	chromosom	1969799	1971004	+	1205	0	10	2927	3	1427	
QEN71_RS08770	cobalt-precorrin-5B (C(1))-methyltransferase	QEN71_08770	paras_002308	protein-codi	NZ_CP1252	chromosom	1971004	1972104	+	1096	0	19	3558	13	1963	
QEN71_RS08775	cobalt-precorrin-6A reductase	QEN71_08775	paras_002309	protein-codi	NZ_CP1252	chromosom	1972101	1972832	+	728	0	12	3855	12	3855	
QEN71_RS08780	precorrin-4 C(11)-methyltransferase	QEN71_08780	paras_002310	protein-codi	NZ_CP1252	chromosom	1972834	1973565	+	732	0	24	3562	19	3011	
QEN71_RS08785	sodium:solute symporter	QEN71_08785	paras_002311	protein-codi	NZ_CP1252	chromosom	1973806	1975284	-	1475	0	50	7951	39	7224	
QEN71_RS08790	DUF3311 domain-containing protein	QEN71_08790	paras_002312	protein-codi	NZ_CP1252	chromosom	1975281	1975493	-	209	0	2	413	2	413	
QEN71_RS08795	MarC family protein	QEN71_08795	paras_002313	protein-codi	NZ_CP1252	chromosom	1975809	1976480	+	672	0	8	3534	8	3534	
QEN71_RS08800	class II glutamine amidotransferase	QEN71_08800	paras_002314	protein-codi	NZ_CP1252	chromosom	1976553	1977386	+	834	0	17	4038	11	2861	
QEN71_RS08805	EAL domain-containing protein	QEN71_08805	paras_002315	protein-codi	NZ_CP1252	chromosom	1977478	1979793	+	2316	0	56	14418	52	13611	
QEN71_RS08810	endo alpha-1,4 polygalactosaminidase	QEN71_08810	paras_002316	protein-codi	NZ_CP1252	chromosom	1979897	1980835	+	939	0	36	6369	28	5080	
QEN71_RS08815	LysE family translocator	QEN71_08815	paras_002317	protein-codi	NZ_CP1252	chromosom	1980858	1981496	-	639	0	26	6222	20	5619	
QEN71_RS08820	glutathione S-transferase family protein	QEN71_08820	paras_002318	protein-codi	NZ_CP1252	chromosom	1981624	1982382	-	759	0	20	4914	16	4416	
QEN71_RS08825	hypothetical protein	QEN71_08825	paras_002319	protein-codi	NZ_CP1252	chromosom	1982638	1982808	+	171	0	7	499	3	280	
QEN71_RS08830	MFS transporter	QEN71_08830	paras_002320	protein-codi	NZ_CP1252	chromosom	1983603	1984898	+	1296	0	25	4464	20	2230	
QEN71_RS08835	glycoside hydrolase family 31 protein	QEN71_08835	paras_002321	protein-codi	NZ_CP1252	chromosom	1984922	1987363	+	2442	0	65	11448	61	10653	
QEN71_RS08840	porin	QEN71_08840	paras_002322	protein-codi	NZ_CP1252	chromosom	1987431	1988618	+	1188	0	50	8448	48	8376	
QEN71_RS08845	VOC family protein	QEN71_08845	paras_002323	protein-codi	NZ_CP1252	chromosom	1988717	1989109	-	393	0	17	4274	11	1950	
QEN71_RS08850	DMT family transporter	QEN71_08850	paras_002324	protein-codi	NZ_CP1252	chromosom	1989191	1990123	-	933	0	13	2591	9	1468	
QEN71_RS08855	LysR family transcriptional regulator	QEN71_08855	paras_002325	protein-codi	NZ_CP1252	chromosom	1990235	1991161	+	927	0	30	9160	25	8329	
QEN71_RS08860	hypothetical protein	partial;pseudo;QEN71_08860		protein-codi	NZ_CP1252	chromosom	1991280	1991498	+	215	0	8	5665	6	5222	
QEN71_RS08865	hypothetical protein	QEN71_08865	paras_002327	protein-codi	NZ_CP1252	chromosom	1991495	1991800	+	302	0	7	2357	7	2357	
QEN71_RS08870	DNA polymerase IV	QEN71_08870	paras_002328	protein-codi	NZ_CP1252	chromosom	1991941	1993095	+	1155	0	22	6440	20	4668	
QEN71_RS08875	hypothetical protein	QEN71_08875	paras_002329	protein-codi	NZ_CP1252	chromosom	1993124	1993465	-	342	0	11	1905	9	1838	
QEN71_RS08880	PLP-dependent aminotransferase family protein	QEN71_08880	paras_002330	protein-codi	NZ_CP1252	chromosom	1993674	1995170	+	1497	0	28	5275	17	2369	
QEN71_RS08885	phosphate ABC transporter substrate-binding protei	QEN71_08885	paras_002331	protein-codi	NZ_CP1252	chromosom	1995347	1996378	+	1032	0	20	7048	18	6583	
QEN71_RS08890	hypothetical protein	QEN71_08890	paras_002332	protein-codi	NZ_CP1252	chromosom	1996602	1996802	+	201	0	4	915	4	915	
QEN71_RS08895	methyl-accepting chemotaxis protein	QEN71_08895	paras_002333	protein-codi	NZ_CP1252	chromosom	1997031	1998581	+	1551	0	9	1913	2	170	
QEN71_RS08900	hypothetical protein	QEN71_08900	paras_002334	protein-codi	NZ_CP1252	chromosom	1998891	1999889	+	999	0	18	3460	18	3460	
QEN71_RS08905	J domain-containing protein	QEN71_08905	paras_002335	protein-codi	NZ_CP1252	chromosom	2000080	2001228	+	1149	0	26	4384	15	3005	
QEN71_RS08910	TauD/TfdA family dioxygenase	QEN71_08910	paras_002336	protein-codi	NZ_CP1252	chromosom	2001268	2002230	-	963	0	7	295	6	292	
QEN71_RS08915	TerB family tellurite resistance protein	QEN71_08915	paras_002337	protein-codi	NZ_CP1252	chromosom	2002450	2002905	-	456	0	10	941	9	885	
QEN71_RS08920	DUF3563 family protein	QEN71_08920	paras_002338	protein-codi	NZ_CP1252	chromosom	2002960	2003337	-	378	0	3	148	2	124	
QEN71_RS08925	hypothetical protein	QEN71_08925	paras_002339	protein-codi	NZ_CP1252	chromosom	2003433	2003708	+	276	0	10	914	7	826	
QEN71_RS08930	hypothetical protein	QEN71_08930	paras_002340	protein-codi	NZ_CP1252	chromosom	2003760	2004029	+	270	0	4	200	4	200	
QEN71_RS08935	DUF4148 domain-containing protein	QEN71_08935	paras_002341	protein-codi	NZ_CP1252	chromosom	2004137	2004445	+	309	0	8	958	6	874	
QEN71_RS08940	ATP-binding protein	QEN71_08940	paras_002342	protein-codi	NZ_CP1252	chromosom	2004655	2005983	+	1329	0	12	1581	10	1449	
QEN71_RS08945	response regulator transcription factor	QEN71_08945	paras_002343	protein-codi	NZ_CP1252	chromosom	2006207	2006881	+	675	0	14	1098	11	922	
QEN71_RS08950	alpha/beta hydrolase	QEN71_08950	paras_002344	protein-codi	NZ_CP1252	chromosom	2007239	2008231	+	993	0	17	3358	14	3175	
QEN71_RS08955	MFS transporter	QEN71_08955	paras_002345	protein-codi	NZ_CP1252	chromosom	2008264	2009508	-	1245	0	12	1444	8	786	
QEN71_RS08960	LysR substrate-binding domain-containing protein	QEN71_08960	paras_002346	protein-codi	NZ_CP1252	chromosom	2009591	2010529	-	939	0	11	1269	11	1269	
QEN71_RS08965	zinc ribbon domain-containing protein	QEN71_08965	paras_002347	protein-codi	NZ_CP1252	chromosom	2010576	2010812	-	237	0	7	1366	2	272	
QEN71_RS08970	HNH endonuclease	QEN71_08970	paras_002348	protein-codi	NZ_CP1252	chromosom	2010850	2011170	-	321	0	2	1248	2	1248	
QEN71_RS08975	SLATT domain-containing protein	QEN71_08975	paras_002349	protein-codi	NZ_CP1252	chromosom	2011368	2011937	+	570	0	20	4206	20	4206	
QEN71_RS08980	PTS sugar transporter subunit IIA	QEN71_08980	paras_002350	protein-codi	NZ_CP1252	chromosom	2011972	2012922	-	951	0	13	2375	11	2051	
QEN71_RS08985	glutathione S-transferase C-terminal domain-contai	QEN71_08985	paras_002351	protein-codi	NZ_CP1252	chromosom	2013132	2013824	+	693	0	34	5647	31	4706	
QEN71_RS08990	YSC84-related protein	QEN71_08990	paras_002352	protein-codi	NZ_CP1252	chromosom	2013989	2014579	+	591	0	7	1621	5	1260	
QEN71_RS08995	universal stress protein	QEN71_08995	paras_002353	protein-codi	NZ_CP1252	chromosom	2014670	2015197	+	528	0	8	1069	5	987	
QEN71_RS09000	Hsp70 family protein	QEN71_09000	paras_002354	protein-codi	NZ_CP1252	chromosom	2015212	2018037	-	2826	0	26	6110	18	4281	
QEN71_RS09005	Hsp70 family protein	QEN71_09005	paras_002355	protein-codi	NZ_CP1252	chromosom	2018052	2019908	-	1853	0	40	10920	32	9203	
QEN71_RS09010	DUF2760 domain-containing protein	QEN71_09010	paras_002356	protein-codi	NZ_CP1252	chromosom	2019905	2020507	-	599	0	10	4484	8	2797	
QEN71_RS09015	DUF3597 domain-containing protein	QEN71_09015	paras_002357	protein-codi	NZ_CP1252	chromosom	2020863	2021270	+	408	0	2	666	2	666	
QEN71_RS09020	exodeoxyribonuclease III	QEN71_09020	paras_002358	protein-codi	NZ_CP1252	chromosom	2021341	2022240	+	900	0	30	7952	27	7508	
QEN71_RS09025	LLM class oxidoreductase	QEN71_09025	paras_002359	protein-codi	NZ_CP1252	chromosom	2022272	2023213	-	942	0	13	3053	13	3053	
QEN71_RS09030	hypothetical protein	QEN71_09030	paras_002360	protein-codi	NZ_CP1252	chromosom	2023510	2023791	+	282	0	6	506	4	436	
QEN71_RS09035	superinfection immunity protein	QEN71_09035	paras_002361	protein-codi	NZ_CP1252	chromosom	2023852	2024160	+	309	0	4	606	3	571	
QEN71_RS09040	NUDIX domain-containing protein	QEN71_09040	paras_002362	protein-codi	NZ_CP1252	chromosom	2024285	2024758	-	474	0	6	622	6	622	
QEN71_RS09045	S1/P1 Nuclease	QEN71_09045	paras_002363	protein-codi	NZ_CP1252	chromosom	2025066	2027372	+	2307	0	51	6445	44	5663	
QEN71_RS09050	type II toxin-antitoxin system HipA family toxin	QEN71_09050	paras_002364	protein-codi	NZ_CP1252	chromosom	2027402	2028733	-	1332	0	15	977	13	620	
QEN71_RS09055	helix-turn-helix transcriptional regulator	QEN71_09055	paras_002365	protein-codi	NZ_CP1252	chromosom	2028736	2029170	-	435	0	3	35	2	30	
QEN71_RS09060	alkaline phosphatase family protein	QEN71_09060	paras_002366	protein-codi	NZ_CP1252	chromosom	2029524	2030981	+	1458	0	32	2565	28	2380	

QEN71_RS09065	patatin-like phospholipase family protein	QEN71_09065	paras_002367	protein-codi	NZ_CP1252	chromosom	2031087	2032340	-	1254	0	21	2159	17	1927	
QEN71_RS09070	3-hydroxybutyrate dehydrogenase	QEN71_09070	paras_002368	protein-codi	NZ_CP1252	chromosom	2032351	2033133	-	783	0	10	1032	8	870	
QEN71_RS09075	acetoacetate decarboxylase	QEN71_09075	paras_002369	protein-codi	NZ_CP1252	chromosom	2033174	2033983	-	810	0	18	1948	14	1604	
QEN71_RS09080	hypothetical protein	QEN71_09080		protein-codi	NZ_CP1252	chromosom	2034443	2034571	+	129	0	3	1126	0	0	
QEN71_RS09085	LysE family transporter	QEN71_09085	paras_002370	protein-codi	NZ_CP1252	chromosom	2034652	2035290	-	639	0	32	7662	28	7230	
QEN71_RS09090	hypothetical protein	QEN71_09090	paras_002371	protein-codi	NZ_CP1252	chromosom	2035409	2036188	-	780	0	16	3387	16	3387	
QEN71_RS09095	Sir2 family NAD-dependent protein deacetylase	QEN71_09095	paras_002372	protein-codi	NZ_CP1252	chromosom	2036344	2037165	+	822	0	7	2093	7	2093	
QEN71_RS09100	HPF/RaiA family ribosome-associated protein	QEN71_09100	paras_002373	protein-codi	NZ_CP1252	chromosom	2037211	2037567	-	357	0	6	757	4	633	
QEN71_RS09105	LysR substrate-binding domain-containing protein	QEN71_09105	paras_002374	protein-codi	NZ_CP1252	chromosom	2037682	2038566	-	885	0	11	889	7	377	
QEN71_RS09110	TerC family protein	QEN71_09110	paras_002375	protein-codi	NZ_CP1252	chromosom	2038709	2039509	+	801	0	11	2315	8	1603	
QEN71_RS09115	zf-TFIIB domain-containing protein	QEN71_09115	paras_002376	protein-codi	NZ_CP1252	chromosom	2039576	2039884	+	309	0	5	1208	5	1208	
QEN71_RS09120	ABC transporter substrate-binding protein	QEN71_09120	paras_002377	protein-codi	NZ_CP1252	chromosom	2040414	2041352	+	939	0	21	2047	14	1286	
QEN71_RS09125	sugar ABC transporter ATP-binding protein	QEN71_09125	paras_002378	protein-codi	NZ_CP1252	chromosom	2041446	2042960	+	1515	0	22	2247	13	1661	
QEN71_RS09130	ABC transporter permease	QEN71_09130	paras_002379	protein-codi	NZ_CP1252	chromosom	2042969	2044006	+	1034	0	6	272	5	265	
QEN71_RS09135	galactofuranose ABC transporter, permease protein	QEN71_09135	paras_002380	protein-codi	NZ_CP1252	chromosom	2044003	2045100	+	1094	0	18	2158	12	1566	
QEN71_RS09140	AraC family transcriptional regulator	QEN71_09140	paras_002381	protein-codi	NZ_CP1252	chromosom	2045106	2046086	-	981	0	14	1527	6	756	
QEN71_RS09145	NAD(P)H-binding protein	QEN71_09145	paras_002382	protein-codi	NZ_CP1252	chromosom	2046252	2047103	+	782	0	7	246	6	219	
QEN71_RS09150	hypothetical protein	QEN71_09150	paras_002383	protein-codi	NZ_CP1252	chromosom	2047034	2047585	-	482	0	4	373	1	12	
QEN71_RS09155	poly(3-hydroxybutyrate) depolymerase	QEN71_09155	paras_002384	protein-codi	NZ_CP1252	chromosom	2047612	2048667	-	1056	0	34	2661	26	2249	
QEN71_RS09160	phosphate ABC transporter substrate-binding protei	QEN71_09160	paras_002385	protein-codi	NZ_CP1252	chromosom	2048951	2049973	-	1023	0	21	1986	16	1577	
QEN71_RS09165	hypothetical protein	QEN71_09165	paras_002386	protein-codi	NZ_CP1252	chromosom	2050280	2051293	+	1014	0	14	2091	8	1003	
QEN71_RS09170	hypothetical protein	QEN71_09170	paras_002387	protein-codi	NZ_CP1252	chromosom	2051338	2051565	+	228	0	1	9	1	9	
QEN71_RS09175	hypothetical protein	QEN71_09175	paras_002388	protein-codi	NZ_CP1252	chromosom	2051741	2051953	+	213	0	3	326	2	147	
QEN71_RS09180	RNA-binding protein	QEN71_09180	paras_002389	protein-codi	NZ_CP1252	chromosom	2052069	2052326	+	258	0	4	686	3	679	
QEN71_RS09185	polyphosphate kinase 2	QEN71_09185	paras_002390	protein-codi	NZ_CP1252	chromosom	2052449	2053246	+	798	0	18	2113	11	1536	
QEN71_RS09190	PqiC family protein	QEN71_09190	paras_002391	protein-codi	NZ_CP1252	chromosom	2053288	2053893	-	602	0	5	130	4	126	
QEN71_RS09195	MlaD family protein	QEN71_09195	paras_002392	protein-codi	NZ_CP1252	chromosom	2053890	2055491	-	1598	0	17	718	15	672	
QEN71_RS09200	efflux transporter outer membrane subunit	QEN71_09200	paras_002393	protein-codi	NZ_CP1252	chromosom	2055565	2057037	-	1473	1167.0	26	3160	15	2306	
QEN71_RS09205	multidrug efflux RND transporter permease subunit	QEN71_09205	paras_002394	protein-codi	NZ_CP1252	chromosom	2057039	2060299	-	3261	3829.0	35	6073	30	5202	
QEN71_RS09210	efflux RND transporter periplasmic adaptor subunit	QEN71_09210	paras_002395	protein-codi	NZ_CP1252	chromosom	2060363	2061643	-	1281	1501.0	9	1289	8	1256	
QEN71_RS09215	response regulator	QEN71_09215	paras_002396	protein-codi	NZ_CP1252	chromosom	2061778	2062164	-	387	0	9	379	6	159	
QEN71_RS09220	TAXI family TRAP transporter solute-binding subuni	QEN71_09220	paras_002397	protein-codi	NZ_CP1252	chromosom	2062349	2063689	+	1341	0	27	2067	20	1547	
QEN71_RS09225	SulP family inorganic anion transporter	QEN71_09225	paras_002398	protein-codi	NZ_CP1252	chromosom	2063713	2065437	-	1725	0	21	3293	16	2155	
QEN71_RS09230	ATPase	QEN71_09230	paras_002399	protein-codi	NZ_CP1252	chromosom	2065668	2066123	+	456	0	6	867	4	257	
QEN71_RS09235	argininosuccinate synthase	QEN71_09235	paras_002400	protein-codi	NZ_CP1252	chromosom	2066234	2067568	+	1335	0	41	6765	31	5819	
QEN71_RS09240	DUF421 domain-containing protein	QEN71_09240	paras_002401	protein-codi	NZ_CP1252	chromosom	2067680	2068231	-	552	0	11	1489	10	1402	
QEN71_RS09245	phosphoketolase family protein	QEN71_09245	paras_002402	protein-codi	NZ_CP1252	chromosom	2068437	2070821	+	2385	0	51	6236	46	5661	
QEN71_RS09250	hypothetical protein	QEN71_09250		protein-codi	NZ_CP1252	chromosom	2070845	2070967	-	123	0	0	0	0	0	
QEN71_RS09255	hypothetical protein	QEN71_09255	paras_002403	protein-codi	NZ_CP1252	chromosom	2070971	2071156	-	186	0	0	0	0	0	
QEN71_RS09260	hemerythrin domain-containing protein	QEN71_09260	paras_002404	protein-codi	NZ_CP1252	chromosom	2071294	2071848	+	555	0	11	1391	8	1036	
QEN71_RS09265	AAA family ATPase	QEN71_09265	paras_002405	protein-codi	NZ_CP1252	chromosom	2071960	2074671	+	2712	0	43	5407	32	4687	
QEN71_RS09270	hypothetical protein	QEN71_09270	paras_002406	protein-codi	NZ_CP1252	chromosom	2074696	2074989	-	294	0	3	355	1	13	
QEN71_RS09275	hypothetical protein	QEN71_09275	paras_002407	protein-codi	NZ_CP1252	chromosom	2075146	2075316	+	171	0	2	62	1	47	
QEN71_RS09280	efflux transporter outer membrane subunit	QEN71_09280	paras_002408	protein-codi	NZ_CP1252	chromosom	2075332	2076915	-	1584	0	15	1289	13	1236	
QEN71_RS09285	DHA2 family efflux MFS transporter permease subu	QEN71_09285	paras_002409	protein-codi	NZ_CP1252	chromosom	2076917	2078512	-	1592	0	42	4776	39	4604	
QEN71_RS09290	HlyD family secretion protein	QEN71_09290	paras_002410	protein-codi	NZ_CP1252	chromosom	2078509	2079681	-	1169	0	22	5212	20	4911	
QEN71_RS09295	protein-L-isoaspartate(D-aspartate) O-methyltransfe	QEN71_09295	paras_002411	protein-codi	NZ_CP1252	chromosom	2079952	2081970	+	2019	0	40	6083	26	3577	
QEN71_RS09300	AI-2E family transporter	QEN71_09300	paras_002412	protein-codi	NZ_CP1252	chromosom	2081979	2083031	-	1053	0	17	3182	16	3057	
QEN71_RS09305	hypothetical protein			pseudogene	NZ_CP1252	chromosom	2083359	2083571	+	213	0	11	1421	9	702	
QEN71_RS09310	sensor histidine kinase	QEN71_09310	paras_002414	protein-codi	NZ_CP1252	chromosom	2083752	2084507	+	756	0	25	3908	19	3045	
QEN71_RS09315	TIGR01841 family phasin	QEN71_09315	paras_002415	protein-codi	NZ_CP1252	chromosom	2084529	2085050	+	522	0	6	820	5	794	
QEN71_RS09320	MFS transporter	QEN71_09320	paras_002416	protein-codi	NZ_CP1252	chromosom	2085131	2086342	+	1212	0	17	3416	13	3184	
QEN71_RS09325	thiamine pyrophosphate-requiring protein	QEN71_09325	paras_002417	protein-codi	NZ_CP1252	chromosom	2086482	2088275	+	1794	0	19	4239	14	2300	
QEN71_RS09330	cytochrome c oxidase subunit II	QEN71_09330	paras_002418	protein-codi	NZ_CP1252	chromosom	2088550	2089524	+	975	0	12	1168	11	1165	
QEN71_RS09335	cytochrome c oxidase subunit I	QEN71_09335	paras_002419	protein-codi	NZ_CP1252	chromosom	2089592	2091538	+	1947	0	45	7887	43	7842	
QEN71_RS09340	cytochrome c oxidase subunit 3	QEN71_09340	paras_002420	protein-codi	NZ_CP1252	chromosom	2091540	2092166	+	623	0	18	2124	8	923	
QEN71_RS09345	hypothetical protein	QEN71_09345	paras_002421	protein-codi	NZ_CP1252	chromosom	2092163	2092597	+	431	0	0	0	0	0	TRUE
QEN71_RS09350	cytochrome c	QEN71_09350	paras_002422	protein-codi	NZ_CP1252	chromosom	2092603	2093958	+	1356	0	26	3042	23	2887	
QEN71_RS09355	acyltransferase	QEN71_09355	paras_002423	protein-codi	NZ_CP1252	chromosom	2094148	2094705	+	533	0	15	1859	13	1774	
QEN71_RS09360	cytochrome b	QEN71_09360	paras_002424	protein-codi	NZ_CP1252	chromosom	2094681	2095214	-	505	0	13	1385	12	1352	
QEN71_RS09365	catalase family peroxidase	QEN71_09365	paras_002425	protein-codi	NZ_CP1252	chromosom	2095211	2096272	-	1058	0	15	1693	10	1126	
QEN71_RS09370	CAP domain-containing protein	QEN71_09370	paras_002426	protein-codi	NZ_CP1252	chromosom	2096362	2097207	-	846	0	16	2631	14	2593	

QEN71_RS09375	hypothetical protein	QEN71_09375	paras_002427	protein-codi	NZ_CP1252	chromosom	2097533	2098231	+	699	0	13	2256	10	1484	
QEN71_RS09380	extracellular solute-binding protein	QEN71_09380	paras_002428	protein-codi	NZ_CP1252	chromosom	2098436	2099533	+	1094	0	14	1506	12	1307	
QEN71_RS09385	ABC transporter permease	QEN71_09385	paras_002429	protein-codi	NZ_CP1252	chromosom	2099530	2100462	+	925	0	9	310	9	310	
QEN71_RS09390	ABC transporter permease	QEN71_09390	paras_002430	protein-codi	NZ_CP1252	chromosom	2100459	2101328	+	866	0	13	1206	11	830	
QEN71_RS09395	ABC transporter ATP-binding protein	QEN71_09395	paras_002431	protein-codi	NZ_CP1252	chromosom	2101351	2102439	+	1089	0	6	390	5	386	
QEN71_RS09400	LacI family DNA-binding transcriptional regulator	QEN71_09400	paras_002432	protein-codi	NZ_CP1252	chromosom	2102686	2103705	+	1020	0	6	752	5	480	
QEN71_RS09405	extracellular solute-binding protein	QEN71_09405	paras_002433	protein-codi	NZ_CP1252	chromosom	2103819	2105150	+	1332	0	26	2504	20	1748	
QEN71_RS09410	sugar ABC transporter permease	QEN71_09410	paras_002434	protein-codi	NZ_CP1252	chromosom	2105225	2106181	+	953	0	16	1315	13	815	
QEN71_RS09415	carbohydrate ABC transporter permease	QEN71_09415	paras_002435	protein-codi	NZ_CP1252	chromosom	2106178	2107131	+	946	0	20	2097	19	2035	
QEN71_RS09420	hypothetical protein	QEN71_09420	paras_002436	protein-codi	NZ_CP1252	chromosom	2107128	2107322	+	191	0	3	232	3	232	
QEN71_RS09425	ABC transporter ATP-binding protein	QEN71_09425	paras_002437	protein-codi	NZ_CP1252	chromosom	2107326	2108441	+	1108	0	18	3223	11	2108	
QEN71_RS09430	ABC transporter ATP-binding protein	QEN71_09430	paras_002438	protein-codi	NZ_CP1252	chromosom	2108434	2109453	+	1008	0	18	1895	16	1685	
QEN71_RS09435	dihydroxyacetone kinase subunit DhaL	QEN71_09435	paras_002439	protein-codi	NZ_CP1252	chromosom	2109450	2110085	+	632	0	6	1193	4	898	
QEN71_RS09440	dihydroxyacetone kinase subunit DhaK	QEN71_09440	paras_002440	protein-codi	NZ_CP1252	chromosom	2110100	2111089	+	990	0	18	4928	13	3909	
QEN71_RS09445	ribonuclease	QEN71_09445	paras_002441	protein-codi	NZ_CP1252	chromosom	2111151	2111633	-	483	0	21	8825	9	2672	
QEN71_RS09450	type VI secretion system Vgr family protein	QEN71_09450	paras_002442	protein-codi	NZ_CP1252	chromosom	2112085	2115219	+	3135	0	102	26088	77	17083	
QEN71_RS09455	hypothetical protein	QEN71_09455	paras_002443	protein-codi	NZ_CP1252	chromosom	2115263	2115637	+	375	0	5	1067	5	1067	
QEN71_RS09460	hypothetical protein	QEN71_09460	paras_002444	protein-codi	NZ_CP1252	chromosom	2115642	2116196	+	555	0	4	783	1	48	
QEN71_RS09465	RHS repeat-associated core domain-containing pro	QEN71_09465	paras_002445	protein-codi	NZ_CP1252	chromosom	2116228	2120940	+	4713	0	175	37827	145	31032	
QEN71_RS09470	hypothetical protein	QEN71_09470	paras_002446	protein-codi	NZ_CP1252	chromosom	2120980	2121264	+	285	0	5	270	5	270	
QEN71_RS09475	hypothetical protein	QEN71_09475	paras_002447	protein-codi	NZ_CP1252	chromosom	2121406	2121726	+	321	0	5	1374	5	1374	
QEN71_RS09480	nitroreductase	QEN71_09480	paras_002448	protein-codi	NZ_CP1252	chromosom	2121853	2122536	-	684	0	19	3859	15	1929	
QEN71_RS09485	GntR family transcriptional regulator	QEN71_09485	paras_002449	protein-codi	NZ_CP1252	chromosom	2122664	2123395	-	732	0	18	2408	10	1248	
QEN71_RS09490	aromatic ring-hydroxylating dioxygenase subunit alp	QEN71_09490	paras_002450	protein-codi	NZ_CP1252	chromosom	2123783	2125099	+	1317	0	21	2264	17	2181	
QEN71_RS09495	glutamine synthetase family protein	QEN71_09495	paras_002451	protein-codi	NZ_CP1252	chromosom	2125158	2126594	+	1437	0	20	1084	15	838	
QEN71_RS09500	2Fe-2S iron-sulfur cluster-binding protein	QEN71_09500	paras_002452	protein-codi	NZ_CP1252	chromosom	2126620	2126943	+	324	0	7	1390	3	900	
QEN71_RS09505	FAD-dependent oxidoreductase	QEN71_09505	paras_002453	protein-codi	NZ_CP1252	chromosom	2126957	2128213	+	1253	0	16	1086	14	1019	
QEN71_RS09510	gamma-glutamyl-gamma-aminobutyrate hydrolase f	QEN71_09510	paras_002454	protein-codi	NZ_CP1252	chromosom	2128210	2128977	+	764	0	9	850	6	794	
QEN71_RS09515	LysR substrate-binding domain-containing protein	QEN71_09515	paras_002455	protein-codi	NZ_CP1252	chromosom	2128982	2129956	+	975	0	4	744	4	744	
QEN71_RS09520	MFS transporter	QEN71_09520	paras_002456	protein-codi	NZ_CP1252	chromosom	2130245	2131606	+	1362	0	17	1791	15	1659	
QEN71_RS09525	DUF4863 family protein	QEN71_09525	paras_002457	protein-codi	NZ_CP1252	chromosom	2131672	2132223	-	552	0	9	436	8	417	
QEN71_RS09530	isoprenylcysteine carboxylmethyltransferase family	QEN71_09530	paras_002458	protein-codi	NZ_CP1252	chromosom	2132471	2133079	+	609	0	10	1127	9	1110	
QEN71_RS09535	porin	QEN71_09535	paras_002459	protein-codi	NZ_CP1252	chromosom	2133405	2134535	+	1131	0	33	2710	29	2600	
QEN71_RS09540	formaldehyde-activating enzyme	QEN71_09540	paras_002460	protein-codi	NZ_CP1252	chromosom	2134806	2135339	+	534	0	11	1793	4	1134	
QEN71_RS09545	Dyp-type peroxidase	QEN71_09545	paras_002461	protein-codi	NZ_CP1252	chromosom	2135622	2136689	+	1060	0	18	4780	14	3271	
QEN71_RS09550	family 1 encapsulin nanocompartment shell protein	QEN71_09550	paras_002462	protein-codi	NZ_CP1252	chromosom	2136682	2137485	+	796	0	9	946	6	506	
QEN71_RS09555	TetR/AcrR family transcriptional regulator	QEN71_09555	paras_002463	protein-codi	NZ_CP1252	chromosom	2137512	2138093	-	582	0	1	47	1	47	
QEN71_RS09560	antibiotic biosynthesis monooxygenase	QEN71_09560	paras_002464	protein-codi	NZ_CP1252	chromosom	2138346	2138696	+	351	0	6	399	5	216	
QEN71_RS09565	alpha/beta hydrolase	QEN71_09565	paras_002465	protein-codi	NZ_CP1252	chromosom	2138988	2139851	+	860	0	16	915	11	741	
QEN71_RS09570	VOC family protein	QEN71_09570	paras_002466	protein-codi	NZ_CP1252	chromosom	2139848	2140345	+	494	0	16	2349	16	2349	
QEN71_RS09575	LysR family transcriptional regulator	QEN71_09575	paras_002467	protein-codi	NZ_CP1252	chromosom	2140435	2141349	-	915	0	17	1525	15	1413	
QEN71_RS09580	M24 family metallopeptidase	QEN71_09580	paras_002468	protein-codi	NZ_CP1252	chromosom	2141356	2142015	-	660	0	12	938	9	685	
QEN71_RS09585	ester cyclase	QEN71_09585	paras_002469	protein-codi	NZ_CP1252	chromosom	2142116	2142511	-	396	0	12	628	6	182	
QEN71_RS09590	XRE family transcriptional regulator	QEN71_09590	paras_002470	protein-codi	NZ_CP1252	chromosom	2142572	2143216	-	645	0	16	3598	15	3556	
QEN71_RS09595	MFS transporter	QEN71_09595	paras_002471	protein-codi	NZ_CP1252	chromosom	2143339	2144625	+	1287	0	21	5922	14	4097	
QEN71_RS09600	amidase family protein	QEN71_09600	paras_002472	protein-codi	NZ_CP1252	chromosom	2144654	2146180	+	1527	0	30	8509	28	7844	
QEN71_RS09605	RidA family protein	QEN71_09605	paras_002473	protein-codi	NZ_CP1252	chromosom	2146199	2146552	+	354	0	4	683	4	683	
QEN71_RS09610	FAD-dependent oxidoreductase	QEN71_09610	paras_002474	protein-codi	NZ_CP1252	chromosom	2146578	2147810	+	1233	0	29	3491	28	3398	
QEN71_RS09615	YdeI/OmpD-associated family protein	QEN71_09615	paras_002475	protein-codi	NZ_CP1252	chromosom	2147874	2148455	+	582	0	15	5732	11	4341	
QEN71_RS09620	winged helix DNA-binding protein	QEN71_09620	paras_002476	protein-codi	NZ_CP1252	chromosom	2148579	2149100	+	522	0	11	2080	11	2080	
QEN71_RS09625	FUSC family protein	QEN71_09625	paras_002477	protein-codi	NZ_CP1252	chromosom	2149106	2151250	+	2145	0	38	8267	25	5589	
QEN71_RS09630	DUF1656 domain-containing protein	QEN71_09630	paras_002478	protein-codi	NZ_CP1252	chromosom	2151285	2151494	+	206	0	10	1961	8	1717	
QEN71_RS09635	efflux RND transporter periplasmic adaptor subunit	QEN71_09635	paras_002479	protein-codi	NZ_CP1252	chromosom	2151491	2152498	+	1000	0	11	2153	6	112	
QEN71_RS09640	efflux transporter outer membrane subunit	QEN71_09640	paras_002480	protein-codi	NZ_CP1252	chromosom	2152495	2153955	+	1457	0	27	4219	22	3904	
QEN71_RS09645	alpha/beta hydrolase	QEN71_09645	paras_002481	protein-codi	NZ_CP1252	chromosom	2153962	2154774	-	813	0	17	3843	15	3779	
QEN71_RS09650	MFS transporter	QEN71_09650	paras_002482	protein-codi	NZ_CP1252	chromosom	2154835	2156076	-	1220	0	19	4353	19	4353	
QEN71_RS09655	hypothetical protein	QEN71_09655	paras_002483	protein-codi	NZ_CP1252	chromosom	2156055	2156294	+	218	193.0	5	812	4	659	
QEN71_RS09660	NADP-dependent isocitrate dehydrogenase	QEN71_09660	paras_002484	protein-codi	NZ_CP1252	chromosom	2156757	2159000	-	2244	0	6	327	3	19	TRUE
QEN71_RS09665	MBL fold metallo-hydrolase	QEN71_09665	paras_002485	protein-codi	NZ_CP1252	chromosom	2159401	2160537	+	1137	0	20	4870	16	3658	
QEN71_RS09670	hypothetical protein	QEN71_09670	paras_002486	protein-codi	NZ_CP1252	chromosom	2160759	2161124	+	366	0	8	4149	5	1768	
QEN71_RS09675	DUF899 domain-containing protein	QEN71_09675	paras_002487	protein-codi	NZ_CP1252	chromosom	2161157	2161867	-	711	0	17	3214	16	3070	
QEN71_RS09680	glutathione S-transferase family protein	QEN71_09680	paras_002488	protein-codi	NZ_CP1252	chromosom	2161899	2162552	-	654	0	15	3774	9	2981	

QEN71_RS09685	FprA family A-type flavoprotein	QEN71_09685	paras_002489	protein-codi	NZ_CP1252	chromosom	2162677	2163408	-	732	0	12	3406	8	2722	
QEN71_RS09690	DOPA 4,5-dioxygenase family protein	QEN71_09690	paras_002490	protein-codi	NZ_CP1252	chromosom	2163463	2163801	-	339	0	9	3055	6	2209	
QEN71_RS09695	phytoene/squalene synthase family protein	QEN71_09695	paras_002491	protein-codi	NZ_CP1252	chromosom	2163892	2164947	-	1056	0	14	272	14	272	
QEN71_RS09700	ABC transporter ATP-binding protein	QEN71_09700	paras_002492	protein-codi	NZ_CP1252	chromosom	2165452	2167320	+	1869	0	47	13382	34	11634	
QEN71_RS09705	serine/threonine dehydratase family protein	QEN71_09705	paras_002493	protein-codi	NZ_CP1252	chromosom	2167460	2168380	+	921	0	6	537	4	290	
QEN71_RS09710	cytochrome P450	QEN71_09710	paras_002494	protein-codi	NZ_CP1252	chromosom	2168419	2169618	-	1200	0	25	8906	15	6100	
QEN71_RS09715	2-dehydropantoate 2-reductase	QEN71_09715	paras_002495	protein-codi	NZ_CP1252	chromosom	2169660	2170643	-	980	0	13	2899	5	660	
QEN71_RS09720	class II aldolase/adducin family protein	QEN71_09720	paras_002496	protein-codi	NZ_CP1252	chromosom	2170640	2171443	-	800	0	27	6348	24	5224	
QEN71_RS09725	amino acid aminotransferase	QEN71_09725	paras_002497	protein-codi	NZ_CP1252	chromosom	2171466	2172674	-	1209	0	29	6392	21	3702	
QEN71_RS09730	LysR substrate-binding domain-containing protein	QEN71_09730	paras_002498	protein-codi	NZ_CP1252	chromosom	2172829	2173764	+	936	0	9	707	7	572	
QEN71_RS09735	dicarboxylate transporter/tellurite-resistance protein	QEN71_09735	paras_002499	protein-codi	NZ_CP1252	chromosom	2173817	2174899	-	1083	0	20	4402	18	4220	
QEN71_RS09740	hypothetical protein	QEN71_09740	paras_002500	protein-codi	NZ_CP1252	chromosom	2174977	2175162	-	184	0	1	21	1	21	
QEN71_RS09745	TlpA disulfide reductase family protein	QEN71_09745	paras_002501	protein-codi	NZ_CP1252	chromosom	2175161	2175694	+	532	0	11	1460	9	1416	
QEN71_RS09750	glycerophosphodiester phosphodiesterase family pr	QEN71_09750	paras_002502	protein-codi	NZ_CP1252	chromosom	2175705	2176661	-	957	0	11	870	11	870	
QEN71_RS09755	DUF302 domain-containing protein	QEN71_09755	paras_002503	protein-codi	NZ_CP1252	chromosom	2176750	2177166	-	417	0	0	0	0	0	TRUE
QEN71_RS09760	hypothetical protein	QEN71_09760	paras_002504	protein-codi	NZ_CP1252	chromosom	2177202	2177693	-	492	0	7	890	7	890	
QEN71_RS09765	NAD-dependent succinate-semialdehyde dehydrog	QEN71_09765	paras_002505	protein-codi	NZ_CP1252	chromosom	2178072	2179520	-	1449	0	11	1414	6	407	
QEN71_RS09770	PaaI family thioesterase	QEN71_09770	paras_002506	protein-codi	NZ_CP1252	chromosom	2179766	2180146	-	381	0	23	2657	17	2360	
QEN71_RS09775	substrate-binding domain-containing protein	QEN71_09775	paras_002507	protein-codi	NZ_CP1252	chromosom	2180159	2181172	-	1014	0	20	1134	16	897	
QEN71_RS09780	ABC transporter ATP-binding protein	QEN71_09780	paras_002508	protein-codi	NZ_CP1252	chromosom	2181209	2182300	-	1092	0	15	1988	12	1229	
QEN71_RS09785	carbohydrate ABC transporter permease	QEN71_09785	paras_002509	protein-codi	NZ_CP1252	chromosom	2182310	2183224	-	914	0	21	2018	21	2018	
QEN71_RS09790	sugar ABC transporter permease	QEN71_09790	paras_002510	protein-codi	NZ_CP1252	chromosom	2183224	2184147	-	923	0	23	2353	19	2192	
QEN71_RS09795	ABC transporter substrate-binding protein	QEN71_09795	paras_002511	protein-codi	NZ_CP1252	chromosom	2184187	2185431	-	1245	0	18	1958	16	1845	
QEN71_RS09800	GH1 family beta-glucosidase	QEN71_09800	paras_002512	protein-codi	NZ_CP1252	chromosom	2185500	2186891	-	1392	0	30	5199	22	2965	
QEN71_RS09805	acyltransferase	QEN71_09805	paras_002513	protein-codi	NZ_CP1252	chromosom	2187277	2188464	+	1188	0	22	4939	20	4819	
QEN71_RS09810	hypothetical protein	QEN71_09810	paras_002514	protein-codi	NZ_CP1252	chromosom	2188537	2189601	-	1065	0	25	6803	21	4606	
QEN71_RS09815	hypothetical protein	QEN71_09815	paras_002515	protein-codi	NZ_CP1252	chromosom	2189764	2189937	-	174	0	7	1737	3	1002	
QEN71_RS09820	diguanylate cyclase	QEN71_09820	paras_002516	protein-codi	NZ_CP1252	chromosom	2190030	2191859	-	1829	0	22	3250	17	2357	
QEN71_RS09825	extracellular solute-binding protein	QEN71_09825	paras_002517	protein-codi	NZ_CP1252	chromosom	2191859	2192971	-	1112	0	29	5405	20	2973	
QEN71_RS09830	hypothetical protein	QEN71_09830	paras_002518	protein-codi	NZ_CP1252	chromosom	2193078	2193311	+	234	0	16	1199	13	971	
QEN71_RS09835	HAMP domain-containing sensor histidine kinase	QEN71_09835	paras_002519	protein-codi	NZ_CP1252	chromosom	2193374	2194885	-	1508	0	20	2869	17	2778	
QEN71_RS09840	response regulator transcription factor	QEN71_09840	paras_002520	protein-codi	NZ_CP1252	chromosom	2194882	2195613	-	728	0	16	2339	12	2000	
QEN71_RS09845	peptide-methionine (R)-S-oxide reductase MsrB	QEN71_09845	paras_002521	protein-codi	NZ_CP1252	chromosom	2195805	2196347	+	543	0	5	484	3	120	
QEN71_RS09850	cytochrome c biogenesis protein DipZ	QEN71_09850	paras_002522	protein-codi	NZ_CP1252	chromosom	2196384	2198201	+	1818	0	35	5800	25	3222	
QEN71_RS09855	peptide-methionine (S)-S-oxide reductase MsrA	QEN71_09855	paras_002523	protein-codi	NZ_CP1252	chromosom	2198234	2198971	+	738	0	26	5474	22	4678	
QEN71_RS09860	aldo/keto reductase	QEN71_09860	paras_002524	protein-codi	NZ_CP1252	chromosom	2198981	2199862	-	882	0	19	4161	16	3284	
QEN71_RS09865	RidA family protein	QEN71_09865	paras_002525	protein-codi	NZ_CP1252	chromosom	2199934	2200335	-	402	0	3	583	3	583	
QEN71_RS09870	MBL fold metallo-hydrolase	QEN71_09870	paras_002526	protein-codi	NZ_CP1252	chromosom	2200568	2201695	-	1128	0	13	1176	13	1176	
QEN71_RS09875	TetR/AcrR family transcriptional regulator	QEN71_09875	paras_002527	protein-codi	NZ_CP1252	chromosom	2201791	2202426	+	636	0	9	2116	7	1974	
QEN71_RS09880	glycosyltransferase	QEN71_09880	paras_002528	protein-codi	NZ_CP1252	chromosom	2202442	2203104	-	659	0	15	2843	12	2528	
QEN71_RS09885	class I SAM-dependent methyltransferase	QEN71_09885	paras_002529	protein-codi	NZ_CP1252	chromosom	2203101	2203709	-	605	0	16	4400	13	4296	
QEN71_RS09890	acyl-CoA dehydrogenase	QEN71_09890	paras_002530	protein-codi	NZ_CP1252	chromosom	2203722	2204870	-	1149	0	18	2167	12	1313	
QEN71_RS09895	FAD-dependent oxidoreductase	QEN71_09895	paras_002531	protein-codi	NZ_CP1252	chromosom	2205102	2206685	+	1584	0	19	2363	11	1275	
QEN71_RS09900	hypothetical protein	QEN71_09900	paras_002532	protein-codi	NZ_CP1252	chromosom	2206750	2206956	-	207	0	5	265	4	257	
QEN71_RS09905	hypothetical protein	QEN71_09905	paras_002533	protein-codi	NZ_CP1252	chromosom	2206993	2207304	-	312	0	6	1984	6	1984	
QEN71_RS09910	hypothetical protein	QEN71_09910	paras_002534	protein-codi	NZ_CP1252	chromosom	2207450	2207596	-	147	0	0	0	0	0	
QEN71_RS09915	VTT domain-containing protein	QEN71_09915	paras_002535	protein-codi	NZ_CP1252	chromosom	2207799	2210006	+	2204	0	37	5276	33	4944	
QEN71_RS09920	endonuclease/exonuclease/phosphatase family pro	QEN71_09920	paras_002536	protein-codi	NZ_CP1252	chromosom	2210003	2210755	+	749	0	11	1655	4	539	
QEN71_RS09925	hypothetical protein	QEN71_09925	paras_002537	protein-codi	NZ_CP1252	chromosom	2210830	2210991	+	162	0	3	144	0	0	
QEN71_RS09930	hypothetical protein	QEN71_09930	paras_002538	protein-codi	NZ_CP1252	chromosom	2211095	2211697	-	603	0	9	1011	9	1011	
QEN71_RS09935	chemotaxis protein	QEN71_09935	paras_002539	protein-codi	NZ_CP1252	chromosom	2211827	2212237	-	411	0	2	451	0	0	
QEN71_RS09940	UdgX family uracil-DNA binding protein	QEN71_09940	paras_002540	protein-codi	NZ_CP1252	chromosom	2212394	2213083	+	690	0	6	1146	6	1146	
QEN71_RS09945	PAS domain S-box protein	QEN71_09945	paras_002541	protein-codi	NZ_CP1252	chromosom	2213102	2215546	-	2445	0	38	6681	29	5861	
QEN71_RS09950	Lrp/AsnC family transcriptional regulator	QEN71_09950	paras_002542	protein-codi	NZ_CP1252	chromosom	2215642	2216151	-	510	0	5	160	3	129	
QEN71_RS09955	1-aminocyclopropane-1-carboxylate deaminase	QEN71_09955	paras_002543	protein-codi	NZ_CP1252	chromosom	2216346	2217362	+	1017	0	17	2537	13	1349	
QEN71_RS09960	DUF4410 domain-containing protein	QEN71_09960	paras_002544	protein-codi	NZ_CP1252	chromosom	2217472	2218239	-	768	0	10	1684	7	1344	
QEN71_RS09965	hypothetical protein	QEN71_09965	paras_002545	protein-codi	NZ_CP1252	chromosom	2218380	2218709	+	330	0	7	1293	5	1239	
QEN71_RS09970	branched-chain amino acid ABC transporter substra	QEN71_09970	paras_002546	protein-codi	NZ_CP1252	chromosom	2218852	2220000	+	1149	0	18	1424	13	1056	
QEN71_RS09975	ATP-binding sensor histidine kinase	QEN71_09975	paras_002547	protein-codi	NZ_CP1252	chromosom	2220023	2225611	-	5589	0	99	9319	87	7617	
QEN71_RS09980	NAD(P)-dependent oxidoreductase	QEN71_09980	paras_002548	protein-codi	NZ_CP1252	chromosom	2226006	2226821	+	816	0	27	5752	24	5165	
QEN71_RS09985	type II toxin-antitoxin system YhaV family toxin	QEN71_09985	paras_002549	protein-codi	NZ_CP1252	chromosom	2226891	2227397	-	503	0	19	3071	14	2277	
QEN71_RS09990	type II toxin-antitoxin system PrlF family antitoxin	QEN71_09990	paras_002550	protein-codi	NZ_CP1252	chromosom	2227394	2227762	-	365	0	1	3	1	3	TRUE

QEN71_RS09995	porin	QEN71_09995	paras_002551	protein-codi	NZ_CP1252	chromosom	2227916	2229151	-	1236	874.0	38	7934	32	6894	
QEN71_RS10000	dihydrodipicolinate synthase family protein	QEN71_10000	paras_002552	protein-codi	NZ_CP1252	chromosom	2229285	2230220	-	936	0	27	5523	20	4333	
QEN71_RS10005	antibiotic biosynthesis monooxygenase	QEN71_10005	paras_002553	protein-codi	NZ_CP1252	chromosom	2230426	2230731	+	306	0	4	1900	2	947	
QEN71_RS10010	DUF488 family protein	QEN71_10010	paras_002554	protein-codi	NZ_CP1252	chromosom	2230775	2231161	+	387	0	9	3181	7	2727	
QEN71_RS10015	SDR family oxidoreductase	QEN71_10015	paras_002555	protein-codi	NZ_CP1252	chromosom	2231180	2231893	-	714	0	3	385	1	15	
QEN71_RS10020	ester cyclase	QEN71_10020	paras_002556	protein-codi	NZ_CP1252	chromosom	2231930	2232505	-	576	0	8	2841	5	2342	
QEN71_RS10025	LysR family transcriptional regulator	QEN71_10025	paras_002557	protein-codi	NZ_CP1252	chromosom	2232615	2233529	+	915	0	8	1202	4	289	
QEN71_RS10030	hypothetical protein	QEN71_10030	paras_002558	protein-codi	NZ_CP1252	chromosom	2233708	2233974	+	267	0	5	846	2	334	
QEN71_RS10035	winged helix-turn-helix domain-containing protein	QEN71_10035	paras_002559	protein-codi	NZ_CP1252	chromosom	2234010	2235770	-	1761	0	43	7366	37	6651	
QEN71_RS10040	MFS transporter	QEN71_10040	paras_002560	protein-codi	NZ_CP1252	chromosom	2235825	2237123	-	1299	0	25	7482	18	6315	
QEN71_RS10045	methyl-accepting chemotaxis protein	QEN71_10045	paras_002561	protein-codi	NZ_CP1252	chromosom	2237489	2239165	+	1677	0	21	4112	19	4087	
QEN71_RS10050	chemotaxis protein CheW	QEN71_10050	paras_002562	protein-codi	NZ_CP1252	chromosom	2239214	2239693	+	476	0	3	209	1	97	TRUE
QEN71_RS10055	CheR family methyltransferase	QEN71_10055	paras_002563	protein-codi	NZ_CP1252	chromosom	2239690	2241114	+	1401	0	31	5498	31	5498	
QEN71_RS10060	chemotaxis protein CheW	QEN71_10060	paras_002564	protein-codi	NZ_CP1252	chromosom	2241095	2241784	+	666	0	7	1005	5	814	
QEN71_RS10065	hybrid sensor histidine kinase/response regulator	QEN71_10065	paras_002565	protein-codi	NZ_CP1252	chromosom	2241781	2244051	+	2263	0	32	4866	25	2760	
QEN71_RS10070	chemotaxis response regulator protein-glutamate m	QEN71_10070	paras_002566	protein-codi	NZ_CP1252	chromosom	2244048	2245052	+	1001	0	12	2431	11	2407	
QEN71_RS10075	diguanylate cyclase	QEN71_10075	paras_002567	protein-codi	NZ_CP1252	chromosom	2245169	2246287	+	1119	0	24	6457	22	6234	
QEN71_RS10080	NnrS family protein	QEN71_10080	paras_002568	protein-codi	NZ_CP1252	chromosom	2246331	2247548	-	1218	0	25	6700	14	3634	
QEN71_RS10085	hemerythrin domain-containing protein	QEN71_10085	paras_002569	protein-codi	NZ_CP1252	chromosom	2247612	2247998	-	387	0	6	2755	6	2755	
QEN71_RS10090	LacI family DNA-binding transcriptional regulator	QEN71_10090	paras_002570	protein-codi	NZ_CP1252	chromosom	2248193	2249260	-	1068	0	17	2176	16	1879	
QEN71_RS10095	MFS transporter	QEN71_10095	paras_002571	protein-codi	NZ_CP1252	chromosom	2249330	2250655	-	1326	0	37	6810	33	6085	
QEN71_RS10100	ribonuclease activity regulator RraA	QEN71_10100	paras_002572	protein-codi	NZ_CP1252	chromosom	2250740	2251486	-	747	0	8	1010	2	144	
QEN71_RS10105	L-arabinonate dehydratase	QEN71_10105	paras_002573	protein-codi	NZ_CP1252	chromosom	2251514	2253262	-	1749	0	32	7247	21	3734	
QEN71_RS10110	EthD family reductase	QEN71_10110	paras_002574	protein-codi	NZ_CP1252	chromosom	2253472	2253780	-	309	0	9	2758	7	2051	
QEN71_RS10115	transporter	QEN71_10115	paras_002575	protein-codi	NZ_CP1252	chromosom	2253947	2254990	-	1044	0	15	3844	11	2953	
QEN71_RS10120	SDR family oxidoreductase	QEN71_10120	paras_002576	protein-codi	NZ_CP1252	chromosom	2255196	2255945	-	750	0	11	1714	7	775	
QEN71_RS10125	LysR family transcriptional regulator	QEN71_10125	paras_002577	protein-codi	NZ_CP1252	chromosom	2256070	2256993	+	924	0	22	7321	19	6741	
QEN71_RS10130	methyl-accepting chemotaxis protein	QEN71_10130	paras_002578	protein-codi	NZ_CP1252	chromosom	2257163	2258737	+	1575	0	27	6072	23	5820	
QEN71_RS10135	FadR/GntR family transcriptional regulator	QEN71_10135	paras_002579	protein-codi	NZ_CP1252	chromosom	2258774	2259478	-	705	0	13	2203	10	2054	
QEN71_RS10140	(Fe-S)-binding protein	QEN71_10140	paras_002580	protein-codi	NZ_CP1252	chromosom	2259613	2260407	+	791	0	13	1168	6	541	
QEN71_RS10145	lactate utilization protein C	QEN71_10145	paras_002581	protein-codi	NZ_CP1252	chromosom	2260404	2261120	+	709	0	15	1942	15	1942	
QEN71_RS10150	LutB/LldF family L-lactate oxidation iron-sulfur prote	QEN71_10150	paras_002582	protein-codi	NZ_CP1252	chromosom	2261117	2262562	+	1442	0	21	1888	18	1866	
QEN71_RS10155	lactate permease LctP family transporter	QEN71_10155	paras_002583	protein-codi	NZ_CP1252	chromosom	2262678	2264405	+	1728	0	18	1511	12	884	
QEN71_RS10160	LysR substrate-binding domain-containing protein	QEN71_10160	paras_002584	protein-codi	NZ_CP1252	chromosom	2264456	2265334	-	879	0	18	807	10	606	
QEN71_RS10165	DMT family transporter	QEN71_10165	paras_002585	protein-codi	NZ_CP1252	chromosom	2265436	2266356	+	921	0	16	1253	15	1249	
QEN71_RS10170	Fe2+-dependent dioxygenase	QEN71_10170	paras_002586	protein-codi	NZ_CP1252	chromosom	2266361	2267044	-	684	0	12	1735	7	507	
QEN71_RS10175	TonB-dependent siderophore receptor	QEN71_10175	paras_002587	protein-codi	NZ_CP1252	chromosom	2267145	2269382	-	2238	0	64	6044	53	5329	
QEN71_RS10180	ABC transporter substrate-binding protein	QEN71_10180	paras_002588	protein-codi	NZ_CP1252	chromosom	2269722	2271464	-	1743	0	37	4994	35	4689	
QEN71_RS10185	DUF2160 domain-containing protein	QEN71_10185	paras_002589	protein-codi	NZ_CP1252	chromosom	2271550	2271831	-	281	0	4	193	1	10	
QEN71_RS10190	carbohydrate ABC transporter permease	QEN71_10190	paras_002590	protein-codi	NZ_CP1252	chromosom	2271831	2272643	-	804	0	16	736	11	484	
QEN71_RS10195	sugar ABC transporter permease	QEN71_10195	paras_002591	protein-codi	NZ_CP1252	chromosom	2272636	2273529	-	885	0	15	1221	13	1011	
QEN71_RS10200	ABC transporter ATP-binding protein	QEN71_10200	paras_002592	protein-codi	NZ_CP1252	chromosom	2273529	2274638	-	1095	0	11	1331	9	1021	
QEN71_RS10205	ABC transporter ATP-binding protein	QEN71_10205	paras_002593	protein-codi	NZ_CP1252	chromosom	2274625	2275737	-	1099	0	18	2704	12	1395	
QEN71_RS10210	FAD-binding oxidoreductase	QEN71_10210	paras_002594	protein-codi	NZ_CP1252	chromosom	2276614	2278008	+	1395	0	18	2229	14	1301	
QEN71_RS10215	DMT family transporter	QEN71_10215	paras_002595	protein-codi	NZ_CP1252	chromosom	2278118	2278951	+	834	0	12	1707	12	1707	
QEN71_RS10220	hypothetical protein	QEN71_10220	paras_002596	protein-codi	NZ_CP1252	chromosom	2279042	2279566	+	525	0	2	12	2	12	
QEN71_RS10225	DUF2325 domain-containing protein	QEN71_10225	paras_002597	protein-codi	NZ_CP1252	chromosom	2279663	2281003	+	1341	0	22	2355	16	2252	
QEN71_RS10230	winged helix-turn-helix domain-containing protein	QEN71_10230	paras_002598	protein-codi	NZ_CP1252	chromosom	2281034	2281399	-	366	0	6	441	4	407	
QEN71_RS10235	molybdopterin-dependent oxidoreductase	QEN71_10235	paras_002599	protein-codi	NZ_CP1252	chromosom	2281525	2282016	-	492	0	5	1114	5	1114	
QEN71_RS10240	tyrosine-type recombinase/integrase	QEN71_10240	paras_002600	protein-codi	NZ_CP1252	chromosom	2282419	2283660	+	1242	0	58	7082	45	3939	
QEN71_RS10245	hypothetical protein	QEN71_10245	paras_002601	protein-codi	NZ_CP1252	chromosom	2283878	2284369	+	492	0	17	902	14	698	
QEN71_RS10250	hypothetical protein	QEN71_10250	paras_002602	protein-codi	NZ_CP1252	chromosom	2284381	2284575	+	195	0	4	268	4	268	
QEN71_RS10255	hypothetical protein	QEN71_10255	paras_002603	protein-codi	NZ_CP1252	chromosom	2284654	2284917	+	264	0	3	20	2	7	
QEN71_RS10260	hypothetical protein	QEN71_10260	paras_002604	protein-codi	NZ_CP1252	chromosom	2285026	2285394	+	369	0	2	65	2	65	
QEN71_RS10265	hypothetical protein	QEN71_10265	paras_002605	protein-codi	NZ_CP1252	chromosom	2285655	2286011	+	357	0	21	2405	16	1766	
QEN71_RS10270	hypothetical protein	QEN71_10270	paras_002606	protein-codi	NZ_CP1252	chromosom	2286510	2286746	-	237	0	9	1107	8	1092	
QEN71_RS10275	hypothetical protein	QEN71_10275	paras_002607	protein-codi	NZ_CP1252	chromosom	2286781	2286978	-	198	0	6	481	5	202	
QEN71_RS10280	hypothetical protein	QEN71_10280	paras_002608	protein-codi	NZ_CP1252	chromosom	2287956	2288504	-	549	0	7	377	7	377	
QEN71_RS10285	hypothetical protein	QEN71_10285	paras_002609	protein-codi	NZ_CP1252	chromosom	2288636	2288815	-	180	0	1	36	1	36	
QEN71_RS10290	hypothetical protein	QEN71_10290	paras_002610	protein-codi	NZ_CP1252	chromosom	2289047	2289196	-	150	0	1	23	1	23	
QEN71_RS10295	hypothetical protein	QEN71_10295	paras_002611	protein-codi	NZ_CP1252	chromosom	2289236	2290516	-	1273	0	15	769	10	604	
QEN71_RS10300	hypothetical protein	QEN71_10300	paras_002612	protein-codi	NZ_CP1252	chromosom	2290509	2291015	-	499	0	12	814	8	751	

QEN71_RS10305	hypothetical protein	QEN71_10305	paras_002613	protein-codi	NZ_CP1252	chromosom	2291096	2291383	-	288	0	3	132	3	132	
QEN71_RS10310	hypothetical protein	QEN71_10310	paras_002614	protein-codi	NZ_CP1252	chromosom	2291423	2292649	-	1227	0	18	818	15	661	
QEN71_RS10315	hypothetical protein	QEN71_10315	paras_002615	protein-codi	NZ_CP1252	chromosom	2292654	2294666	-	2013	0	19	852	15	610	
QEN71_RS10320	hypothetical protein	QEN71_10320	paras_002616	protein-codi	NZ_CP1252	chromosom	2294728	2295024	-	297	0	5	223	2	124	
QEN71_RS10325	hypothetical protein	QEN71_10325	paras_002617	protein-codi	NZ_CP1252	chromosom	2295028	2295555	-	524	0	4	395	4	395	
QEN71_RS10330	hypothetical protein	QEN71_10330	paras_002618	protein-codi	NZ_CP1252	chromosom	2295552	2295722	-	167	0	3	129	3	129	
QEN71_RS10335	hypothetical protein	QEN71_10335	paras_002619	protein-codi	NZ_CP1252	chromosom	2295733	2296311	-	575	0	12	372	9	311	
QEN71_RS10340	hypothetical protein	QEN71_10340	paras_002620	protein-codi	NZ_CP1252	chromosom	2296308	2296679	-	364	0	9	627	7	616	
QEN71_RS10345	hypothetical protein	QEN71_10345	paras_002621	protein-codi	NZ_CP1252	chromosom	2296676	2296954	-	275	0	2	275	1	219	
QEN71_RS10350	hypothetical protein	QEN71_10350	paras_002622	protein-codi	NZ_CP1252	chromosom	2297194	2297427	-	234	0	2	51	2	51	
QEN71_RS10355	hypothetical protein	QEN71_10355	paras_002623	protein-codi	NZ_CP1252	chromosom	2297695	2298810	-	1116	0	42	2322	38	2144	
QEN71_RS10360	hypothetical protein	QEN71_10360	paras_002624	protein-codi	NZ_CP1252	chromosom	2298957	2299133	-	177	0	8	473	7	471	
QEN71_RS10365	hypothetical protein	QEN71_10365	paras_002625	protein-codi	NZ_CP1252	chromosom	2299435	2299614	+	180	0	9	932	5	552	
QEN71_RS10370	hypothetical protein	QEN71_10370	paras_002626	protein-codi	NZ_CP1252	chromosom	2299668	2300243	-	576	0	26	2970	14	1964	
QEN71_RS10375	TOBE domain-containing protein	QEN71_10375	paras_002627	protein-codi	NZ_CP1252	chromosom	2300520	2300945	-	426	0	3	776	0	0	
QEN71_RS10380	DUF3022 domain-containing protein	QEN71_10380	paras_002628	protein-codi	NZ_CP1252	chromosom	2301162	2301551	+	390	0	11	2559	6	2294	
QEN71_RS10385	YceH family protein	QEN71_10385	paras_002629	protein-codi	NZ_CP1252	chromosom	2301682	2302386	+	705	0	12	1865	7	718	
QEN71_RS10390	acid phosphatase	QEN71_10390	paras_002630	protein-codi	NZ_CP1252	chromosom	2302537	2304234	+	1698	0	38	4805	34	4334	
QEN71_RS10395	hypothetical protein	QEN71_10395	paras_002631	protein-codi	NZ_CP1252	chromosom	2304267	2304506	+	240	0	2	221	0	0	
QEN71_RS10400	M23 family metallopeptidase	QEN71_10400	paras_002632	protein-codi	NZ_CP1252	chromosom	2304693	2305520	+	828	0	19	1508	15	1281	
QEN71_RS10405	glutaredoxin 3	QEN71_10405	paras_002633	protein-codi	NZ_CP1252	chromosom	2305594	2305866	-	273	0	7	1199	2	72	
QEN71_RS10410	DUF4148 domain-containing protein	QEN71_10410	paras_002634	protein-codi	NZ_CP1252	chromosom	2305899	2306237	-	339	0	14	1485	8	1082	
QEN71_RS10415	AraC family transcriptional regulator	QEN71_10415	paras_002635	protein-codi	NZ_CP1252	chromosom	2306491	2307540	-	1050	0	5	806	1	25	
QEN71_RS10420	dihydrolipoyl dehydrogenase	QEN71_10420	paras_002636	protein-codi	NZ_CP1252	chromosom	2307706	2309112	+	1407	0	22	3205	17	2094	
QEN71_RS10425	nitrate reductase subunit alpha	QEN71_10425	paras_002637	protein-codi	NZ_CP1252	chromosom	2309518	2313315	+	3794	0	65	6211	48	3982	
QEN71_RS10430	nitrate reductase subunit beta	QEN71_10430	paras_002638	protein-codi	NZ_CP1252	chromosom	2313312	2314838	+	1523	0	26	3846	22	3589	
QEN71_RS10435	nitrate reductase molybdenum cofactor assembly ch	QEN71_10435	paras_002639	protein-codi	NZ_CP1252	chromosom	2314840	2315568	+	725	0	10	1283	9	1227	
QEN71_RS10440	respiratory nitrate reductase subunit gamma	QEN71_10440	paras_002640	protein-codi	NZ_CP1252	chromosom	2315565	2316248	+	680	0	14	1584	6	546	
QEN71_RS10445	peptidylprolyl isomerase	QEN71_10445	paras_002641	protein-codi	NZ_CP1252	chromosom	2316253	2317032	+	780	0	7	479	5	449	
QEN71_RS10450	nitrate/nitrite transporter	QEN71_10450	paras_002642	protein-codi	NZ_CP1252	chromosom	2317047	2318309	+	1263	0	23	2104	18	1132	
QEN71_RS10455	LysR family transcriptional regulator	QEN71_10455	paras_002643	protein-codi	NZ_CP1252	chromosom	2318340	2319263	-	924	0	13	1053	10	923	
QEN71_RS10460	benzoate 1,2-dioxygenase large subunit	QEN71_10460	paras_002644	protein-codi	NZ_CP1252	chromosom	2319422	2320789	+	1364	0	15	1424	10	634	
QEN71_RS10465	benzoate 1,2-dioxygenase small subunit	QEN71_10465	paras_002645	protein-codi	NZ_CP1252	chromosom	2320786	2321289	+	500	0	18	1824	12	1169	
QEN71_RS10470	benzoate 1,2-dioxygenase electron transfer compor	QEN71_10470	paras_002646	protein-codi	NZ_CP1252	chromosom	2321318	2322340	+	1023	0	23	2122	22	2110	
QEN71_RS10475	1,6-dihydroxycyclohexa-2,4-diene-1-carboxylate de	QEN71_10475	paras_002647	protein-codi	NZ_CP1252	chromosom	2322346	2323125	+	780	0	9	659	5	222	
QEN71_RS10480	hypothetical protein	QEN71_10480	paras_002648	protein-codi	NZ_CP1252	chromosom	2323150	2323539	-	390	0	12	753	8	605	
QEN71_RS10485	porin	QEN71_10485	paras_002649	protein-codi	NZ_CP1252	chromosom	2323572	2324738	+	1167	0	51	5270	44	4496	
QEN71_RS10490	NAD(P)-dependent oxidoreductase	QEN71_10490	paras_002650	protein-codi	NZ_CP1252	chromosom	2324960	2325712	+	749	0	14	2106	13	1922	
QEN71_RS10495	cupin domain-containing protein	QEN71_10495	paras_002651	protein-codi	NZ_CP1252	chromosom	2325709	2326227	+	515	0	5	1043	5	1043	
QEN71_RS10500	PLP-dependent aminotransferase family protein	QEN71_10500	paras_002652	protein-codi	NZ_CP1252	chromosom	2326258	2327796	-	1539	0	23	4676	18	4116	
QEN71_RS10505	AI-2E family transporter	QEN71_10505	paras_002653	protein-codi	NZ_CP1252	chromosom	2328026	2329927	+	1902	0	35	7080	24	5941	
QEN71_RS10510	alpha/beta fold hydrolase	QEN71_10510	paras_002654	protein-codi	NZ_CP1252	chromosom	2330126	2331211	+	1086	0	12	2040	7	1243	
QEN71_RS10515	hypothetical protein	QEN71_10515	paras_002655	protein-codi	NZ_CP1252	chromosom	2331517	2332485	+	969	0	19	1860	14	1498	
QEN71_RS10520	VC0807 family protein	QEN71_10520	paras_002656	protein-codi	NZ_CP1252	chromosom	2332632	2333267	+	632	0	14	1192	9	844	
QEN71_RS10525	amidohydrolase family protein	QEN71_10525	paras_002657	protein-codi	NZ_CP1252	chromosom	2333264	2334460	+	1193	0	9	655	1	92	
QEN71_RS10530	phospholipase C, phosphocholine-specific	QEN71_10530	paras_002658	protein-codi	NZ_CP1252	chromosom	2334530	2336686	-	2157	0	47	4743	41	4029	
QEN71_RS10535	hypothetical protein	QEN71_10535	paras_002659	protein-codi	NZ_CP1252	chromosom	2336789	2337166	-	378	0	1	99	1	99	
QEN71_RS10540	amino acid racemase	QEN71_10540	paras_002660	protein-codi	NZ_CP1252	chromosom	2337360	2338865	-	1506	0	38	8142	29	5139	
QEN71_RS10545	response regulator	QEN71_10545	paras_002661	protein-codi	NZ_CP1252	chromosom	2339239	2339682	+	444	0	6	1181	5	1146	
QEN71_RS10550	YSC84-related protein	QEN71_10550	paras_002662	protein-codi	NZ_CP1252	chromosom	2339930	2340517	+	588	0	19	7159	13	4513	
QEN71_RS10555	glycogen synthase GlgA	QEN71_10555	paras_002663	protein-codi	NZ_CP1252	chromosom	2340954	2342576	+	1623	0	29	5439	23	4597	
QEN71_RS10560	glycogen debranching protein GlgX	QEN71_10560	paras_002664	protein-codi	NZ_CP1252	chromosom	2342698	2344869	-	2172	0	39	5876	35	5353	
QEN71_RS10565	ATP-binding protein	QEN71_10565	paras_002665	protein-codi	NZ_CP1252	chromosom	2345075	2347522	+	2448	0	35	6691	31	6314	
QEN71_RS10570	response regulator	QEN71_10570	paras_002666	protein-codi	NZ_CP1252	chromosom	2347550	2347984	+	435	0	31	6081	25	4774	
QEN71_RS10575	ATP-binding protein	QEN71_10575	paras_002667	protein-codi	NZ_CP1252	chromosom	2348582	2350216	-	1635	0	42	7097	33	5767	
QEN71_RS10580	hypothetical protein	QEN71_10580	paras_002668	protein-codi	NZ_CP1252	chromosom	2350318	2350491	-	174	0	2	256	0	0	
QEN71_RS10585	response regulator	QEN71_10585	paras_002669	protein-codi	NZ_CP1252	chromosom	2350625	2351056	+	432	0	2	271	0	0	
QEN71_RS10590	biliverdin-producing heme oxygenase	QEN71_10590		protein-codi	NZ_CP1252	chromosom	2351072	2351731	-	660	0	11	895	8	863	
QEN71_RS10595	CsbD family protein	QEN71_10595	paras_002671	protein-codi	NZ_CP1252	chromosom	2351937	2352137	-	201	0	2	102	2	102	
QEN71_RS10600	hypothetical protein	QEN71_10600	paras_002672	protein-codi	NZ_CP1252	chromosom	2352255	2352464	-	210	0	3	2073	1	60	
QEN71_RS10605	2OG-Fe(II) oxygenase	QEN71_10605	paras_002673	protein-codi	NZ_CP1252	chromosom	2352625	2353509	-	885	0	22	6028	17	4677	
QEN71_RS10610	nuclear transport factor 2 family protein	QEN71_10610	paras_002674	protein-codi	NZ_CP1252	chromosom	2353590	2354063	-	474	0	5	614	3	347	

QEN71_RS10615	patatin-like phospholipase family protein	QEN71_10615	paras_002675	protein-codi	NZ_CP1252	chromosom	2354233	2355516	-	1284	0	15	1785	14	1752	
QEN71_RS10620	VWA domain-containing protein	QEN71_10620	paras_002676	protein-codi	NZ_CP1252	chromosom	2355681	2357114	-	1434	0	27	4567	17	3453	
QEN71_RS10625	TadE/TadG family type IV pilus assembly protein	QEN71_10625	paras_002677	protein-codi	NZ_CP1252	chromosom	2357166	2357810	-	645	0	12	1391	9	1207	
QEN71_RS10630	TadE/TadG family type IV pilus assembly protein	QEN71_10630	paras_002678	protein-codi	NZ_CP1252	chromosom	2357812	2358351	-	540	0	11	1336	11	1336	
QEN71_RS10635	type II secretion system F family protein	QEN71_10635	paras_002679	protein-codi	NZ_CP1252	chromosom	2358385	2359377	-	993	0	5	821	5	821	
QEN71_RS10640	type II secretion system F family protein	QEN71_10640	paras_002680	protein-codi	NZ_CP1252	chromosom	2359426	2360406	-	981	0	12	918	8	431	
QEN71_RS10645	CpaF family protein	QEN71_10645	paras_002681	protein-codi	NZ_CP1252	chromosom	2360435	2361826	-	1392	0	2	1065	2	1065	
QEN71_RS10650	AAA family ATPase	QEN71_10650	paras_002682	protein-codi	NZ_CP1252	chromosom	2361878	2363080	-	1203	0	7	613	7	613	
QEN71_RS10655	hypothetical protein	QEN71_10655	paras_002683	protein-codi	NZ_CP1252	chromosom	2363135	2363422	-	288	0	2	68	2	68	
QEN71_RS10660	type II and III secretion system protein family protei	QEN71_10660	paras_002684	protein-codi	NZ_CP1252	chromosom	2363461	2365569	-	2109	0	15	2211	8	1427	
QEN71_RS10665	Flp pilus assembly protein CpaB	QEN71_10665	paras_002685	protein-codi	NZ_CP1252	chromosom	2365688	2366527	-	840	0	3	323	3	323	
QEN71_RS10670	hypothetical protein	QEN71_10670	paras_002686	protein-codi	NZ_CP1252	chromosom	2366593	2367123	-	531	0	10	1828	6	1153	
QEN71_RS10675	prepilin peptidase	QEN71_10675	paras_002687	protein-codi	NZ_CP1252	chromosom	2367277	2367816	-	540	0	9	976	8	803	
QEN71_RS10680	Flp family type IVb pilin	QEN71_10680	paras_002688	protein-codi	NZ_CP1252	chromosom	2367926	2368108	-	183	0	5	327	5	327	
QEN71_RS10685	Flp family type IVb pilin	QEN71_10685	paras_002689	protein-codi	NZ_CP1252	chromosom	2368158	2368340	-	183	0	2	182	2	182	
QEN71_RS10690	sigma-54 dependent transcriptional regulator	QEN71_10690	paras_002690	protein-codi	NZ_CP1252	chromosom	2369359	2370864	+	1506	0	26	8573	21	7057	
QEN71_RS10695	DHA2 family efflux MFS transporter permease subu	QEN71_10695	paras_002691	protein-codi	NZ_CP1252	chromosom	2370996	2372606	+	1611	0	29	10073	24	8887	
QEN71_RS10700	NIPSNAP family protein	QEN71_10700	paras_002692	protein-codi	NZ_CP1252	chromosom	2372716	2373057	+	342	0	10	2882	9	2786	
QEN71_RS10705	mechanosensitive ion channel	QEN71_10705	paras_002693	protein-codi	NZ_CP1252	chromosom	2373273	2375888	+	2616	0	33	5187	27	3648	
QEN71_RS10710	SRPBCC family protein	QEN71_10710	paras_002694	protein-codi	NZ_CP1252	chromosom	2375907	2376395	-	489	0	8	2806	7	2661	
QEN71_RS10715	ATP-dependent DNA helicase	QEN71_10715	paras_002695	protein-codi	NZ_CP1252	chromosom	2376412	2378685	-	2270	0	49	9831	37	6660	
QEN71_RS10720	VRR-NUC domain-containing protein	QEN71_10720	paras_002696	protein-codi	NZ_CP1252	chromosom	2378682	2380388	-	1703	0	29	6099	19	2375	
QEN71_RS10725	MFS transporter	QEN71_10725	paras_002697	protein-codi	NZ_CP1252	chromosom	2380460	2382052	-	1593	0	22	4344	18	3210	
QEN71_RS10730	DUF3034 family protein	QEN71_10730	paras_002698	protein-codi	NZ_CP1252	chromosom	2382515	2383414	+	899	0	19	3618	16	2852	
QEN71_RS10735	group 1 truncated hemoglobin	QEN71_10735	paras_002699	protein-codi	NZ_CP1252	chromosom	2383414	2383866	+	448	0	9	1071	7	968	
QEN71_RS10740	methylamine utilization protein	QEN71_10740	paras_002700	protein-codi	NZ_CP1252	chromosom	2383863	2384492	+	626	0	13	939	9	662	
QEN71_RS10745	EAL domain-containing protein	QEN71_10745	paras_002701	protein-codi	NZ_CP1252	chromosom	2384500	2386830	+	2331	0	26	3235	21	2580	
QEN71_RS10750	FUSC family protein	QEN71_10750	paras_002702	protein-codi	NZ_CP1252	chromosom	2386938	2388062	+	1125	0	10	1928	9	1832	
QEN71_RS10755	TIGR03118 family protein	QEN71_10755	paras_002703	protein-codi	NZ_CP1252	chromosom	2388253	2389422	+	1170	0	17	1280	12	790	
QEN71_RS10760	RNA polymerase factor sigma-54	QEN71_10760	paras_002704	protein-codi	NZ_CP1252	chromosom	2389497	2390969	-	1473	0	33	7808	21	5420	
QEN71_RS10765	BON domain-containing protein	QEN71_10765	paras_002705	protein-codi	NZ_CP1252	chromosom	2391419	2391757	+	339	0	1	5	0	0	TRUE
QEN71_RS10770	PA2169 family four-helix-bundle protein	QEN71_10770	paras_002706	protein-codi	NZ_CP1252	chromosom	2391859	2392311	-	453	0	5	927	3	591	
QEN71_RS10775	hypothetical protein	QEN71_10775	paras_002707	protein-codi	NZ_CP1252	chromosom	2392631	2392915	+	285	0	3	193	3	193	
QEN71_RS10780	DUF1488 domain-containing protein	QEN71_10780	paras_002708	protein-codi	NZ_CP1252	chromosom	2392976	2393320	-	345	0	5	465	1	5	
QEN71_RS10785	DUF4142 domain-containing protein	QEN71_10785	paras_002709	protein-codi	NZ_CP1252	chromosom	2393329	2393874	-	546	0	7	1483	7	1483	
QEN71_RS10790	type 1 glutamine amidotransferase domain-containi	QEN71_10790	paras_002710	protein-codi	NZ_CP1252	chromosom	2393945	2394496	-	552	0	8	1013	6	912	
QEN71_RS10795	DNA topoisomerase IB	QEN71_10795	paras_002711	protein-codi	NZ_CP1252	chromosom	2394645	2395850	+	1206	0	31	5442	29	5167	
QEN71_RS10800	hypothetical protein	QEN71_10800	paras_002712	protein-codi	NZ_CP1252	chromosom	2395887	2396180	-	294	0	8	2256	8	2256	
QEN71_RS10805	hypothetical protein	QEN71_10805	paras_002713	protein-codi	NZ_CP1252	chromosom	2396429	2396614	-	186	0	0	0	0	0	
QEN71_RS10810	hypothetical protein	QEN71_10810	paras_002714	protein-codi	NZ_CP1252	chromosom	2396712	2396987	-	276	0	4	519	3	446	
QEN71_RS10815	HAD family hydrolase	QEN71_10815	paras_002715	protein-codi	NZ_CP1252	chromosom	2397345	2398019	+	675	0	8	1283	3	556	
QEN71_RS10820	hypothetical protein	QEN71_10820	paras_002716	protein-codi	NZ_CP1252	chromosom	2398135	2398275	+	141	0	1	49	0	0	
QEN71_RS10825	PRC-barrel domain-containing protein	QEN71_10825	paras_002717	protein-codi	NZ_CP1252	chromosom	2398376	2398882	+	507	0	4	592	2	374	
QEN71_RS10830	DUF3175 domain-containing protein	QEN71_10830	paras_002718	protein-codi	NZ_CP1252	chromosom	2398993	2399427	+	435	0	6	1576	4	926	
QEN71_RS10835	cation:proton antiporter	QEN71_10835	paras_002719	protein-codi	NZ_CP1252	chromosom	2399450	2400796	+	1347	0	27	6400	18	4520	
QEN71_RS10840	bifunctional glycoside hydrolase 114/ polysaccharid	QEN71_10840	paras_002720	protein-codi	NZ_CP1252	chromosom	2401139	2403922	+	2758	0	87	20580	70	14127	
QEN71_RS10845	tetratricopeptide repeat protein	QEN71_10845	paras_002721	protein-codi	NZ_CP1252	chromosom	2403897	2407787	+	3865	0	86	15086	65	12599	
QEN71_RS10850	penicillin-binding protein activator LpoB	QEN71_10850	paras_002722	protein-codi	NZ_CP1252	chromosom	2407813	2408364	+	552	0	10	1152	10	1152	
QEN71_RS10855	PelD GGDEF domain-containing protein	QEN71_10855	paras_002723	protein-codi	NZ_CP1252	chromosom	2408426	2409904	+	1465	0	16	1062	14	995	
QEN71_RS10860	sugar ABC transporter permease	QEN71_10860	paras_002724	protein-codi	NZ_CP1252	chromosom	2409891	2411060	+	1156	0	13	1721	11	1636	
QEN71_RS10865	GT4 family glycosyltransferase PelF	QEN71_10865	paras_002725	protein-codi	NZ_CP1252	chromosom	2411071	2412654	+	1584	0	26	3148	16	1693	
QEN71_RS10870	exopolysaccharide Pel transporter PelG	QEN71_10870	paras_002726	protein-codi	NZ_CP1252	chromosom	2412671	2414041	+	1371	0	45	4458	35	3541	
QEN71_RS10875	IS481 family transposase	QEN71_10875	paras_002727	protein-codi	NZ_CP1252	chromosom	2415454	2416593	+	1140	1112.0	18	18786	16	18208	
QEN71_RS10880	aldo/keto reductase	QEN71_10880	paras_002728	protein-codi	NZ_CP1252	chromosom	2416758	2417801	-	1044	0	24	4028	19	3269	
QEN71_RS10885	DEAD/DEAH box helicase	QEN71_10885	paras_002729	protein-codi	NZ_CP1252	chromosom	2418131	2422624	+	4494	0	65	13092	52	10685	
QEN71_RS10890	alpha,alpha-trehalase TreA	QEN71_10890	paras_002730	protein-codi	NZ_CP1252	chromosom	2422698	2424257	-	1560	0	60	11701	51	9784	
QEN71_RS10895	cyclic di-GMP phosphodiesterase	QEN71_10895	paras_002731	protein-codi	NZ_CP1252	chromosom	2424716	2426719	+	2004	0	34	8275	29	7021	
QEN71_RS10900	crotonase/enoyl-CoA hydratase family protein	QEN71_10900	paras_002732	protein-codi	NZ_CP1252	chromosom	2426959	2427837	-	879	0	13	2412	8	1321	
QEN71_RS10905	malate/lactate/ureidoglycolate dehydrogenase	QEN71_10905	paras_002733	protein-codi	NZ_CP1252	chromosom	2428300	2429406	-	1107	0	15	4604	11	2500	
QEN71_RS10910	M20 family metallopeptidase	QEN71_10910	paras_002734	protein-codi	NZ_CP1252	chromosom	2429507	2430967	-	1461	0	29	10127	25	9370	
QEN71_RS10915	superinfection immunity protein	QEN71_10915	paras_002735	protein-codi	NZ_CP1252	chromosom	2431138	2431446	+	309	0	6	2082	4	1531	
QEN71_RS10920	epoxide hydrolase	QEN71_10920	paras_002736	protein-codi	NZ_CP1252	chromosom	2431483	2432637	+	1155	0	26	5973	24	5725	

QEN71_RS10925	helix-turn-helix domain-containing protein	QEN71_10925	paras_002737	protein-codi	NZ_CP1252	chromosom	2432654	2433073	-	420	0	6	499	2	93	
QEN71_RS10930	multidrug efflux SMR transporter	QEN71_10930	paras_002738	protein-codi	NZ_CP1252	chromosom	2433150	2433467	+	318	0	10	2658	6	2010	
QEN71_RS10935	LysE family translocator	QEN71_10935	paras_002739	protein-codi	NZ_CP1252	chromosom	2433663	2434295	+	633	0	31	16863	30	16766	
QEN71_RS10940	YodC family protein	QEN71_10940	paras_002740	protein-codi	NZ_CP1252	chromosom	2434390	2434671	-	282	0	9	6136	7	5999	
QEN71_RS10945	MFS transporter	QEN71_10945	paras_002741	protein-codi	NZ_CP1252	chromosom	2435206	2436684	-	1479	0	36	14912	25	12506	
QEN71_RS10950	TetR/AcrR family transcriptional regulator	QEN71_10950	paras_002742	protein-codi	NZ_CP1252	chromosom	2436742	2437374	-	633	0	5	759	3	470	
QEN71_RS10955	isocitrate lyase/phosphoenolpyruvate mutase family	QEN71_10955	paras_002743	protein-codi	NZ_CP1252	chromosom	2437461	2438297	-	837	0	21	4695	14	2329	
QEN71_RS10960	OsmC family protein	QEN71_10960	paras_002744	protein-codi	NZ_CP1252	chromosom	2438397	2438822	-	426	0	3	504	3	504	
QEN71_RS10965	DUF4148 domain-containing protein	QEN71_10965	paras_002745	protein-codi	NZ_CP1252	chromosom	2439148	2439441	+	294	0	3	1134	1	5	
QEN71_RS10970	carboxymuconolactone decarboxylase family protei	QEN71_10970	paras_002746	protein-codi	NZ_CP1252	chromosom	2439562	2440116	-	555	0	15	3213	13	3128	
QEN71_RS10975	aspartate aminotransferase family protein	QEN71_10975	paras_002747	protein-codi	NZ_CP1252	chromosom	2440147	2441484	-	1338	0	32	7293	29	6773	
QEN71_RS10980	C45 family peptidase	QEN71_10980	paras_002748	protein-codi	NZ_CP1252	chromosom	2441519	2442559	-	1041	0	9	4619	6	1519	
QEN71_RS10985	MurR/RpiR family transcriptional regulator	QEN71_10985	paras_002749	protein-codi	NZ_CP1252	chromosom	2442680	2443540	-	861	0	11	1645	7	894	
QEN71_RS10990	AAA family ATPase	QEN71_10990	paras_002750	protein-codi	NZ_CP1252	chromosom	2443640	2446276	-	2633	0	31	6257	22	3563	
QEN71_RS10995	DNA repair exonuclease	QEN71_10995	paras_002751	protein-codi	NZ_CP1252	chromosom	2446273	2447385	-	1109	0	24	6042	20	5236	
QEN71_RS11000	HD domain-containing protein	QEN71_11000	paras_002752	protein-codi	NZ_CP1252	chromosom	2447480	2448007	-	528	0	6	2306	4	1957	
QEN71_RS11005	hypothetical protein	QEN71_11005	paras_002753	protein-codi	NZ_CP1252	chromosom	2448384	2448620	+	237	0	15	7499	10	2415	
QEN71_RS11010	hypothetical protein	QEN71_11010	paras_002754	protein-codi	NZ_CP1252	chromosom	2448727	2449098	-	372	0	15	6636	7	2735	
QEN71_RS11015	phytanoyl-CoA dioxygenase family protein	QEN71_11015	paras_002755	protein-codi	NZ_CP1252	chromosom	2449221	2450012	+	792	0	32	8871	24	6329	
QEN71_RS11020	aromatic ring-hydroxylating dioxygenase subunit alp	QEN71_11020	paras_002756	protein-codi	NZ_CP1252	chromosom	2450022	2451278	-	1257	0	30	8694	24	6787	
QEN71_RS11025	LysR substrate-binding domain-containing protein	QEN71_11025	paras_002757	protein-codi	NZ_CP1252	chromosom	2451422	2452351	-	930	0	14	3037	9	1887	
QEN71_RS11030	GlxA family transcriptional regulator	QEN71_11030	paras_002758	protein-codi	NZ_CP1252	chromosom	2452364	2453455	-	1092	0	7	1475	7	1475	
QEN71_RS11035	LysR substrate-binding domain-containing protein	QEN71_11035	paras_002759	protein-codi	NZ_CP1252	chromosom	2453484	2454485	-	1002	0	17	3199	14	2893	
QEN71_RS11040	aldehyde dehydrogenase	QEN71_11040	paras_002760	protein-codi	NZ_CP1252	chromosom	2454724	2456217	-	1494	0	20	1044	13	783	
QEN71_RS11045	FAD-binding oxidoreductase	QEN71_11045	paras_002761	protein-codi	NZ_CP1252	chromosom	2456235	2457566	-	1332	0	19	2140	15	1440	
QEN71_RS11050	dihydrodipicolinate synthase family protein	QEN71_11050	paras_002762	protein-codi	NZ_CP1252	chromosom	2457574	2458461	-	888	0	16	1963	12	1594	
QEN71_RS11055	serine hydroxymethyltransferase	QEN71_11055	paras_002763	protein-codi	NZ_CP1252	chromosom	2458537	2459832	-	1296	0	12	1844	11	1817	
QEN71_RS11060	glycine betaine ABC transporter substrate-binding p	QEN71_11060	paras_002764	protein-codi	NZ_CP1252	chromosom	2459902	2460783	-	882	0	18	1644	15	1563	
QEN71_RS11065	proline/glycine betaine ABC transporter permease	QEN71_11065	paras_002765	protein-codi	NZ_CP1252	chromosom	2460868	2461752	-	881	0	5	308	5	308	
QEN71_RS11070	glycine betaine/L-proline ABC transporter ATP-bind	QEN71_11070	paras_002766	protein-codi	NZ_CP1252	chromosom	2461749	2463038	-	1286	0	11	923	7	585	
QEN71_RS11075	hypothetical protein	QEN71_11075	paras_002767	protein-codi	NZ_CP1252	chromosom	2463554	2463922	-	369	0	2	127	1	111	
QEN71_RS11080	hypothetical protein	QEN71_11080	paras_002768	protein-codi	NZ_CP1252	chromosom	2464194	2464550	-	357	0	2	433	2	433	
QEN71_RS11085	hypothetical protein	QEN71_11085	paras_002769	protein-codi	NZ_CP1252	chromosom	2464714	2465058	-	345	0	7	1522	7	1522	
QEN71_RS11090	tetratricopeptide repeat protein	QEN71_11090	paras_002770	protein-codi	NZ_CP1252	chromosom	2465261	2466067	-	807	0	9	2175	7	1875	
QEN71_RS11095	Ku protein	QEN71_11095	paras_002771	protein-codi	NZ_CP1252	chromosom	2466097	2467029	-	933	0	29	8269	27	8104	
QEN71_RS11100	YukJ family protein	QEN71_11100	paras_002772	protein-codi	NZ_CP1252	chromosom	2467222	2467965	-	744	0	31	8489	25	6494	
QEN71_RS11105	amylo-alpha-1,6-glucosidase	QEN71_11105	paras_002773	protein-codi	NZ_CP1252	chromosom	2468003	2470282	-	2280	0	30	8295	29	8293	
QEN71_RS11110	citrate synthase	QEN71_11110	paras_002774	protein-codi	NZ_CP1252	chromosom	2470621	2471922	-	1302	0	44	4076	32	3181	
QEN71_RS11115	hypothetical protein	QEN71_11115	paras_002775	protein-codi	NZ_CP1252	chromosom	2472347	2472751	+	405	0	7	434	7	434	
QEN71_RS11120	alpha/beta hydrolase	QEN71_11120	paras_002776	protein-codi	NZ_CP1252	chromosom	2472767	2473549	-	783	0	15	2596	9	1344	
QEN71_RS11125	rod shape-determining protein	QEN71_11125	paras_002777	protein-codi	NZ_CP1252	chromosom	2473724	2474779	-	1056	0	13	2093	10	1675	
QEN71_RS11130	dienelactone hydrolase family protein	QEN71_11130	paras_002778	protein-codi	NZ_CP1252	chromosom	2475007	2475666	-	660	0	11	1447	6	1027	
QEN71_RS11135	DHA2 family efflux MFS transporter permease subu	QEN71_11135	paras_002779	protein-codi	NZ_CP1252	chromosom	2475996	2477597	+	1602	0	23	5110	19	4585	
QEN71_RS11140	CoA-acylating methylmalonate-semialdehyde dehyd	QEN71_11140	paras_002780	protein-codi	NZ_CP1252	chromosom	2477846	2479342	+	1497	0	43	10186	28	6524	
QEN71_RS11145	Uma2 family endonuclease	QEN71_11145	paras_002781	protein-codi	NZ_CP1252	chromosom	2479458	2479829	-	372	0	18	4798	10	2272	
QEN71_RS11150	hypothetical protein	QEN71_11150	paras_002782	protein-codi	NZ_CP1252	chromosom	2480356	2480565	+	210	0	10	2128	8	1735	
QEN71_RS11155	NUDIX hydrolase	QEN71_11155	paras_002783	protein-codi	NZ_CP1252	chromosom	2480620	2481258	+	610	0	22	3637	18	2924	
QEN71_RS11160	RDD family protein	QEN71_11160	paras_002784	protein-codi	NZ_CP1252	chromosom	2481230	2481718	+	460	0	11	1007	9	920	
QEN71_RS11165	hypothetical protein	QEN71_11165	paras_002785	protein-codi	NZ_CP1252	chromosom	2482290	2483402	+	1113	0	26	2325	19	2101	
QEN71_RS11170	class I SAM-dependent methyltransferase	QEN71_11170	paras_002786	protein-codi	NZ_CP1252	chromosom	2483681	2484514	+	834	0	10	753	10	753	
QEN71_RS11175	alpha/beta hydrolase	QEN71_11175	paras_002787	protein-codi	NZ_CP1252	chromosom	2484719	2485543	+	825	0	14	1797	12	1522	
QEN71_RS11180	bacterioferritin	QEN71_11180	paras_002788	protein-codi	NZ_CP1252	chromosom	2485999	2486475	+	477	0	14	780	12	660	
QEN71_RS11185	inositol monophosphatase family protein	QEN71_11185	paras_002789	protein-codi	NZ_CP1252	chromosom	2486584	2487366	-	783	0	17	2003	16	1989	
QEN71_RS11190	cache domain-containing protein	QEN71_11190	paras_002790	protein-codi	NZ_CP1252	chromosom	2487476	2487964	-	489	0	12	1752	10	1702	
QEN71_RS11195	adenylate/guanylate cyclase domain-containing pro	QEN71_11195	paras_002791	protein-codi	NZ_CP1252	chromosom	2488132	2491416	+	3285	0	34	4211	26	3168	
QEN71_RS11200	cytosine permease	QEN71_11200	paras_002792	protein-codi	NZ_CP1252	chromosom	2491442	2492857	-	1416	0	26	1972	16	1322	
QEN71_RS11205	acetylornithine deacetylase	QEN71_11205	paras_002793	protein-codi	NZ_CP1252	chromosom	2492936	2494105	-	1162	0	9	516	7	321	
QEN71_RS11210	DUF1028 domain-containing protein	QEN71_11210	paras_002794	protein-codi	NZ_CP1252	chromosom	2494098	2494775	-	670	0	6	957	4	771	
QEN71_RS11215	RidA family protein	QEN71_11215	paras_002795	protein-codi	NZ_CP1252	chromosom	2494786	2495220	-	435	0	10	1184	7	1000	
QEN71_RS11220	NAD(P)/FAD-dependent oxidoreductase	QEN71_11220	paras_002796	protein-codi	NZ_CP1252	chromosom	2495286	2496617	-	1332	0	15	750	12	710	
QEN71_RS11225	LysR family transcriptional regulator	QEN71_11225	paras_002797	protein-codi	NZ_CP1252	chromosom	2496917	2497843	+	927	0	15	786	12	592	
QEN71_RS11230	Rieske 2Fe-2S domain-containing protein	QEN71_11230	paras_002798	protein-codi	NZ_CP1252	chromosom	2498047	2499195	+	1149	0	22	1590	19	1414	

QEN71_RS11235	ABC transporter ATP-binding protein	QEN71_11235	paras_002799	protein-codi	NZ_CP1252	chromosom	2499228	2500415	+	1188	0	15	953	7	617	
QEN71_RS11240	ABC transporter substrate-binding protein	QEN71_11240	paras_002800	protein-codi	NZ_CP1252	chromosom	2500417	2501472	+	1056	0	15	916	14	905	
QEN71_RS11245	ABC transporter permease	QEN71_11245	paras_002801	protein-codi	NZ_CP1252	chromosom	2501521	2502774	+	1254	0	17	1675	14	1522	
QEN71_RS11250	ABC transporter permease	QEN71_11250	paras_002802	protein-codi	NZ_CP1252	chromosom	2502805	2503632	+	828	0	9	569	7	492	
QEN71_RS11255	VOC family protein	QEN71_11255	paras_002803	protein-codi	NZ_CP1252	chromosom	2503636	2504025	-	390	0	10	771	8	558	
QEN71_RS11260	SDR family oxidoreductase	QEN71_11260	paras_002804	protein-codi	NZ_CP1252	chromosom	2504141	2504941	+	791	0	13	1085	7	940	
QEN71_RS11265	short-chain dehydrogenase	partial;pseudo;QEN71_11265		pseudogene	NZ_CP1252	chromosom	2504932	2505057	+	116	0	0	0	0	0	
QEN71_RS11270	DUF3331 domain-containing protein	QEN71_11270	paras_002805	protein-codi	NZ_CP1252	chromosom	2505214	2505726	-	513	0	12	1304	8	972	
QEN71_RS11275	hypothetical protein	QEN71_11275	paras_002806	protein-codi	NZ_CP1252	chromosom	2505856	2506380	-	525	0	18	1282	12	822	
QEN71_RS11280	GlxA family transcriptional regulator	QEN71_11280	paras_002807	protein-codi	NZ_CP1252	chromosom	2506448	2507419	+	972	0	18	1204	14	1008	
QEN71_RS11285	sensor histidine kinase	QEN71_11285	paras_002808	protein-codi	NZ_CP1252	chromosom	2507511	2508635	-	1125	0	24	2517	10	568	
QEN71_RS11290	hypothetical protein	QEN71_11290	paras_002809	protein-codi	NZ_CP1252	chromosom	2508849	2509040	+	192	0	6	493	6	493	
QEN71_RS11295	DUF1223 domain-containing protein	QEN71_11295	paras_002810	protein-codi	NZ_CP1252	chromosom	2509130	2509849	+	720	0	16	1886	10	1572	
QEN71_RS11300	MFS transporter	QEN71_11300	paras_002811	protein-codi	NZ_CP1252	chromosom	2510069	2511364	+	1296	0	27	2348	22	1797	
QEN71_RS11305	substrate-binding domain-containing protein	QEN71_11305	paras_002812	protein-codi	NZ_CP1252	chromosom	2511409	2512191	+	783	0	7	506	6	456	
QEN71_RS11310	C4-dicarboxylate transporter DctA	QEN71_11310	paras_002813	protein-codi	NZ_CP1252	chromosom	2512231	2513538	-	1304	0	19	2101	16	1795	
QEN71_RS11315	LysR family transcriptional regulator	QEN71_11315	paras_002814	protein-codi	NZ_CP1252	chromosom	2513535	2514503	-	965	0	16	1707	13	1394	
QEN71_RS11320	agmatinase	QEN71_11320	paras_002815	protein-codi	NZ_CP1252	chromosom	2514649	2515638	-	990	0	21	2145	18	1984	
QEN71_RS11325	gamma-glutamyltransferase	QEN71_11325	paras_002816	protein-codi	NZ_CP1252	chromosom	2515671	2517263	-	1593	0	23	1530	22	1165	
QEN71_RS11330	aspartate aminotransferase family protein	QEN71_11330	paras_002817	protein-codi	NZ_CP1252	chromosom	2517281	2518651	-	1371	0	15	1184	9	977	
QEN71_RS11335	ATP-binding cassette domain-containing protein	QEN71_11335	paras_002818	protein-codi	NZ_CP1252	chromosom	2518707	2519471	-	765	0	6	532	4	318	
QEN71_RS11340	ABC transporter permease	QEN71_11340	paras_002819	protein-codi	NZ_CP1252	chromosom	2519482	2520207	-	722	0	12	1008	9	783	
QEN71_RS11345	ABC transporter permease subunit	QEN71_11345	paras_002820	protein-codi	NZ_CP1252	chromosom	2520204	2520902	-	695	0	12	527	11	517	
QEN71_RS11350	ABC transporter substrate-binding protein	QEN71_11350	paras_002821	protein-codi	NZ_CP1252	chromosom	2520971	2521765	-	795	0	13	925	7	380	
QEN71_RS11355	GntR family transcriptional regulator	QEN71_11355	paras_002822	protein-codi	NZ_CP1252	chromosom	2521883	2522554	-	672	0	11	2184	7	1400	
QEN71_RS11360	DUF1223 domain-containing protein	QEN71_11360	paras_002823	protein-codi	NZ_CP1252	chromosom	2522864	2523277	+	414	0	7	608	4	142	
QEN71_RS11365	hypothetical protein	QEN71_11365	paras_002824	protein-codi	NZ_CP1252	chromosom	2523399	2523638	-	240	0	3	271	1	146	
QEN71_RS11370	hypothetical protein	QEN71_11370		protein-codi	NZ_CP1252	chromosom	2523974	2524255	-	282	0	5	535	5	535	
QEN71_RS11375	DUF308 domain-containing protein	QEN71_11375	paras_002825	protein-codi	NZ_CP1252	chromosom	2524271	2525512	+	1242	0	35	4811	33	4530	
QEN71_RS11380	DUF3047 domain-containing protein	QEN71_11380	paras_002826	protein-codi	NZ_CP1252	chromosom	2526244	2527017	-	774	0	26	4358	16	2982	
QEN71_RS11385	YciI family protein	QEN71_11385	paras_002827	protein-codi	NZ_CP1252	chromosom	2527402	2527773	+	372	0	6	987	2	672	
QEN71_RS11390	ShlB/FhaC/HecB family hemolysin secretion/activati	QEN71_11390	paras_002828	protein-codi	NZ_CP1252	chromosom	2528007	2529761	-	1755	0	58	10691	43	7669	
QEN71_RS11395	hypothetical protein	QEN71_11395	paras_002829	protein-codi	NZ_CP1252	chromosom	2530638	2530910	-	273	0	15	2117	8	1020	
QEN71_RS11400	hemagglutinin repeat-containing protein	QEN71_11400	paras_002830	protein-codi	NZ_CP1252	chromosom	2530954	2540877	-	9924	0	266	43425	181	25536	
QEN71_RS11405	FadR/GntR family transcriptional regulator	QEN71_11405	paras_002831	protein-codi	NZ_CP1252	chromosom	2541562	2542263	-	702	0	16	3618	16	3618	
QEN71_RS11410	sugar ABC transporter ATP-binding protein	QEN71_11410	paras_002832	protein-codi	NZ_CP1252	chromosom	2542537	2544069	+	1525	0	27	3771	20	2758	
QEN71_RS11415	ABC transporter permease	QEN71_11415	paras_002833	protein-codi	NZ_CP1252	chromosom	2544062	2545093	+	1024	0	11	815	9	681	
QEN71_RS11420	ABC transporter substrate-binding protein	QEN71_11420	paras_002834	protein-codi	NZ_CP1252	chromosom	2545134	2546081	+	948	0	24	2728	18	2232	
QEN71_RS11425	aldo/keto reductase	QEN71_11425	paras_002835	protein-codi	NZ_CP1252	chromosom	2546151	2547167	+	1017	0	25	2966	24	2708	
QEN71_RS11430	zinc-binding alcohol dehydrogenase family protein	QEN71_11430	paras_002836	protein-codi	NZ_CP1252	chromosom	2547315	2548322	+	1008	0	16	1291	14	1265	
QEN71_RS11435	mannitol dehydrogenase family protein	QEN71_11435	paras_002837	protein-codi	NZ_CP1252	chromosom	2548347	2549507	+	1161	0	23	4425	23	4425	
QEN71_RS11440	L-rhamnose mutarotase	QEN71_11440	paras_002838	protein-codi	NZ_CP1252	chromosom	2549559	2549900	+	342	0	8	1177	6	1049	
QEN71_RS11445	alpha-hydroxy-acid oxidizing protein	partial;pseudo;QEN71_11445		pseudogene	NZ_CP1252	chromosom	2549917	2550108	-	192	0	3	54	2	51	
QEN71_RS11450	altronate dehydratase family protein	QEN71_11450	paras_002840	protein-codi	NZ_CP1252	chromosom	2550153	2551700	-	1548	0	19	2028	15	1489	
QEN71_RS11455	MFS transporter	QEN71_11455	paras_002841	protein-codi	NZ_CP1252	chromosom	2551747	2553030	-	1284	0	17	2791	13	1688	
QEN71_RS11460	hypothetical protein	QEN71_11460	paras_002842	protein-codi	NZ_CP1252	chromosom	2553093	2553239	-	147	0	2	587	0	0	
QEN71_RS11465	FadR/GntR family transcriptional regulator	QEN71_11465	paras_002843	protein-codi	NZ_CP1252	chromosom	2553434	2554153	+	720	0	8	1531	6	1457	
QEN71_RS11470	LysR family transcriptional regulator	QEN71_11470	paras_002844	protein-codi	NZ_CP1252	chromosom	2554173	2555105	-	933	0	18	4682	11	3045	
QEN71_RS11475	putative hydro-lyase	QEN71_11475	paras_002845	protein-codi	NZ_CP1252	chromosom	2555332	2556105	+	774	0	8	1015	7	935	
QEN71_RS11480	MFS transporter	QEN71_11480	paras_002846	protein-codi	NZ_CP1252	chromosom	2556326	2557588	+	1263	0	25	3538	21	3141	
QEN71_RS11485	hydantoinase B/oxoprolinase family protein	QEN71_11485	paras_002847	protein-codi	NZ_CP1252	chromosom	2557636	2561271	+	3636	0	59	13170	56	12975	
QEN71_RS11490	ATP-binding cassette domain-containing protein	QEN71_11490	paras_002848	protein-codi	NZ_CP1252	chromosom	2561443	2563104	+	1662	0	24	3565	17	2683	
QEN71_RS11495	helicase	QEN71_11495	paras_002849	protein-codi	NZ_CP1252	chromosom	2563158	2563472	-	315	0	13	3656	7	1696	
QEN71_RS11500	hypothetical protein	pseudo;QEN71_11500		pseudogene	NZ_CP1252	chromosom	2563714	2564358	+	645	0	17	2886	17	2886	
QEN71_RS11505	LysE family translocator	partial;pseudo;QEN71_11505		pseudogene	NZ_CP1252	chromosom	2564530	2564706	-	177	0	1	94	1	94	
QEN71_RS11510	SGNH/GDSL hydrolase family protein	QEN71_11510	paras_002851	protein-codi	NZ_CP1252	chromosom	2564744	2565337	-	594	0	36	6257	31	5420	
QEN71_RS11515	GntR family transcriptional regulator	QEN71_11515	paras_002852	protein-codi	NZ_CP1252	chromosom	2565695	2566417	+	723	0	17	1398	12	670	
QEN71_RS11520	MFS transporter	QEN71_11520	paras_002853	protein-codi	NZ_CP1252	chromosom	2566481	2567776	+	1296	0	33	7604	25	5786	
QEN71_RS11525	amidohydrolase family protein	QEN71_11525	paras_002854	protein-codi	NZ_CP1252	chromosom	2567829	2568713	+	885	0	32	8435	23	3444	
QEN71_RS11530	gluconate:H+ symporter	QEN71_11530	paras_002855	protein-codi	NZ_CP1252	chromosom	2568738	2570123	+	1386	0	40	13721	26	11686	
QEN71_RS11535	decarboxylating 6-phosphogluconate dehydrogenas	QEN71_11535	paras_002856	protein-codi	NZ_CP1252	chromosom	2570230	2571210	-	981	0	27	6974	21	5241	
QEN71_RS11540	transketolase	QEN71_11540	paras_002857	protein-codi	NZ_CP1252	chromosom	2571217	2573313	-	2097	0	41	9001	31	5572	

QEN71_RS11545	transglutaminase domain-containing protein	QEN71_11545	paras_002858	protein-codi	NZ_CP1252	chromosom	2573898	2575025	+	1128	0	32	8643	22	6621	
QEN71_RS11550	HPP family protein	QEN71_11550	paras_002859	protein-codi	NZ_CP1252	chromosom	2575133	2575645	-	513	0	11	2140	4	1258	
QEN71_RS11555	NAD-dependent formate dehydrogenase	QEN71_11555	paras_002860	protein-codi	NZ_CP1252	chromosom	2575651	2576853	-	1203	0	51	12316	37	10055	
QEN71_RS11560	hypothetical protein	QEN71_11560	paras_002861	protein-codi	NZ_CP1252	chromosom	2577054	2578118	-	1065	0	25	3798	17	2686	
QEN71_RS11565	dicarboxylate/amino acid:cation symporter	QEN71_11565	paras_002862	protein-codi	NZ_CP1252	chromosom	2578615	2579922	+	1308	0	25	6136	16	3658	
QEN71_RS11570	amino acid ABC transporter substrate-binding prote	QEN71_11570	paras_002863	protein-codi	NZ_CP1252	chromosom	2579936	2580883	+	948	0	21	4323	21	4323	
QEN71_RS11575	TIM44-like domain-containing protein	QEN71_11575	paras_002864	protein-codi	NZ_CP1252	chromosom	2581361	2582326	-	966	0	9	791	7	654	
QEN71_RS11580	CBS domain-containing protein	QEN71_11580	paras_002865	protein-codi	NZ_CP1252	chromosom	2582418	2582900	-	483	0	21	6218	17	4959	
QEN71_RS11585	transcriptional activator NhaR	QEN71_11585	paras_002866	protein-codi	NZ_CP1252	chromosom	2583048	2583944	+	897	0	19	2694	13	972	
QEN71_RS11590	transketolase family protein	QEN71_11590	paras_002867	protein-codi	NZ_CP1252	chromosom	2584131	2585132	-	998	0	27	3520	23	3211	
QEN71_RS11595	transketolase	QEN71_11595	paras_002868	protein-codi	NZ_CP1252	chromosom	2585129	2585980	-	848	0	15	1436	12	1252	
QEN71_RS11600	MFS transporter	QEN71_11600	paras_002869	protein-codi	NZ_CP1252	chromosom	2586009	2587337	-	1329	0	36	4632	27	4284	
QEN71_RS11605	SDR family NAD(P)-dependent oxidoreductase	QEN71_11605	paras_002870	protein-codi	NZ_CP1252	chromosom	2587481	2588230	-	750	0	7	1476	5	1177	
QEN71_RS11610	LysR substrate-binding domain-containing protein	QEN71_11610	paras_002871	protein-codi	NZ_CP1252	chromosom	2588327	2589241	-	915	0	20	3676	15	2875	
QEN71_RS11615	H-NS family nucleoid-associated regulatory protein	QEN71_11615	paras_002872	protein-codi	NZ_CP1252	chromosom	2589644	2590405	-	762	332.0	12	564	12	564	
QEN71_RS11620	H-NS family nucleoid-associated regulatory protein	QEN71_11620	paras_002873	protein-codi	NZ_CP1252	chromosom	2590583	2590921	+	339	147.0	8	2251	4	333	
QEN71_RS11625	hypothetical protein	QEN71_11625	paras_002874	protein-codi	NZ_CP1252	chromosom	2591105	2591416	+	312	192.0	9	1791	9	1791	
QEN71_RS11630	hypothetical protein	QEN71_11630	paras_002875	protein-codi	NZ_CP1252	chromosom	2591455	2591601	-	147	107.0	6	1088	6	1088	
QEN71_RS11635	DUF3422 domain-containing protein	QEN71_11635	paras_002876	protein-codi	NZ_CP1252	chromosom	2592138	2593472	+	1335	0	43	6065	28	2637	
QEN71_RS11640	aldehyde dehydrogenase (NADP(+))	partial;pseudo;QEN71_11640		pseudogene	NZ_CP1252	chromosom	2593859	2594119	-	261	0	2	226	2	226	
QEN71_RS11645	DUF1330 domain-containing protein	QEN71_11645	paras_002878	protein-codi	NZ_CP1252	chromosom	2594379	2594669	-	291	0	11	3032	8	2875	
QEN71_RS11650	amino acid synthesis family protein	QEN71_11650	paras_002879	protein-codi	NZ_CP1252	chromosom	2594877	2595479	+	571	0	13	3611	9	2064	
QEN71_RS11655	alpha/beta hydrolase	QEN71_11655	paras_002880	protein-codi	NZ_CP1252	chromosom	2595448	2596266	+	787	0	12	2221	9	1745	
QEN71_RS11660	flavin reductase	QEN71_11660	paras_002881	protein-codi	NZ_CP1252	chromosom	2596268	2597191	+	924	0	23	2161	18	1572	
QEN71_RS11665	LLM class flavin-dependent oxidoreductase	QEN71_11665	paras_002882	protein-codi	NZ_CP1252	chromosom	2597202	2598239	+	1038	0	21	3624	15	3131	
QEN71_RS11670	aldehyde dehydrogenase	QEN71_11670	paras_002883	protein-codi	NZ_CP1252	chromosom	2598252	2599721	+	1470	0	29	3140	23	1905	
QEN71_RS11675	cytosine permease	QEN71_11675	paras_002884	protein-codi	NZ_CP1252	chromosom	2599836	2601251	+	1416	0	30	2670	24	1603	
QEN71_RS11680	methyl-accepting chemotaxis protein	QEN71_11680	paras_002885	protein-codi	NZ_CP1252	chromosom	2601324	2602898	-	1575	0	19	1379	14	981	
QEN71_RS11685	aromatic acid/H+ symport family MFS transporter	QEN71_11685	paras_002886	protein-codi	NZ_CP1252	chromosom	2602941	2604305	-	1365	0	32	2702	26	2090	
QEN71_RS11690	NADP-dependent succinate-semialdehyde dehydro	QEN71_11690	paras_002887	protein-codi	NZ_CP1252	chromosom	2604342	2605802	-	1461	0	21	1370	13	709	
QEN71_RS11695	GntR family transcriptional regulator	QEN71_11695	paras_002888	protein-codi	NZ_CP1252	chromosom	2605855	2606562	-	708	0	5	373	5	373	
QEN71_RS11700	ABC transporter ATP-binding protein	QEN71_11700	paras_002889	protein-codi	NZ_CP1252	chromosom	2606575	2607660	-	1086	0	22	2943	20	2923	
QEN71_RS11705	ABC transporter permease	QEN71_11705	paras_002890	protein-codi	NZ_CP1252	chromosom	2607687	2608481	-	795	117.0	3	146	3	146	
QEN71_RS11710	ABC transporter permease	QEN71_11710	paras_002891	protein-codi	NZ_CP1252	chromosom	2608483	2609364	-	882	0	15	4621	10	3702	
QEN71_RS11715	ABC transporter substrate-binding protein	QEN71_11715	paras_002892	protein-codi	NZ_CP1252	chromosom	2609428	2610459	-	1032	152.0	20	3780	15	3122	
QEN71_RS11720	flavin reductase	partial;pseudo;QEN71_11720		pseudogene	NZ_CP1252	chromosom	2610530	2610961	-	432	0	6	2714	6	2714	
QEN71_RS11725	GntR family transcriptional regulator	QEN71_11725	paras_002894	protein-codi	NZ_CP1252	chromosom	2611296	2611982	+	687	0	16	3828	10	2805	
QEN71_RS11730	IS110 family transposase	QEN71_11730	paras_002895	protein-codi	NZ_CP1252	chromosom	2612138	2613151	+	1014	2002.0	22	6407	18	4761	
QEN71_RS11735	prolyl-tRNA synthetase associated domain-containi	QEN71_11735	paras_002896	protein-codi	NZ_CP1252	chromosom	2613694	2614194	-	501	0	10	1035	6	565	
QEN71_RS11740	aspartate ammonia-lyase	QEN71_11740	paras_002897	protein-codi	NZ_CP1252	chromosom	2614269	2615681	-	1405	0	14	2026	10	1726	
QEN71_RS11745	LysR family transcriptional regulator	QEN71_11745	paras_002898	protein-codi	NZ_CP1252	chromosom	2615674	2616657	-	976	0	21	2467	9	1602	
QEN71_RS11750	MFS transporter	QEN71_11750	paras_002899	protein-codi	NZ_CP1252	chromosom	2616684	2618075	-	1392	0	38	5706	34	5392	
QEN71_RS11755	type II asparaginase	QEN71_11755	paras_002900	protein-codi	NZ_CP1252	chromosom	2618250	2619350	-	1101	0	30	4530	21	2959	
QEN71_RS11760	tautomerase family protein	QEN71_11760	paras_002901	protein-codi	NZ_CP1252	chromosom	2619918	2620310	-	393	0	6	524	5	418	
QEN71_RS11765	hexameric tyrosine-coordinated heme protein	QEN71_11765	paras_002902	protein-codi	NZ_CP1252	chromosom	2620399	2620635	-	237	0	5	873	5	873	
QEN71_RS11770	catalase family peroxidase	QEN71_11770	paras_002903	protein-codi	NZ_CP1252	chromosom	2620704	2621705	-	1002	0	22	2326	18	1789	
QEN71_RS11775	MarR family transcriptional regulator	QEN71_11775	paras_002904	protein-codi	NZ_CP1252	chromosom	2621854	2622459	+	606	0	12	1586	9	1360	
QEN71_RS11780	ABC transporter substrate-binding protein	QEN71_11780	paras_002905	protein-codi	NZ_CP1252	chromosom	2622675	2624294	-	1620	0	37	3010	27	2365	
QEN71_RS11785	ABC transporter permease subunit	QEN71_11785	paras_002906	protein-codi	NZ_CP1252	chromosom	2624707	2625546	+	836	0	14	1123	12	1039	
QEN71_RS11790	ABC transporter ATP-binding protein	QEN71_11790	paras_002907	protein-codi	NZ_CP1252	chromosom	2625543	2626286	+	740	0	2	48	2	48	TRUE
QEN71_RS11795	NrtA/SsuA/CpmA family ABC transporter substrate-	QEN71_11795	paras_002908	protein-codi	NZ_CP1252	chromosom	2626362	2627366	+	1005	0	14	1565	10	1027	
QEN71_RS11800	acyl-CoA dehydrogenase family protein	QEN71_11800	paras_002909	protein-codi	NZ_CP1252	chromosom	2627379	2628617	+	1239	0	22	1659	17	1467	
QEN71_RS11805	LLM class flavin-dependent oxidoreductase	QEN71_11805	paras_002910	protein-codi	NZ_CP1252	chromosom	2628679	2629740	-	1062	0	17	800	14	745	
QEN71_RS11810	LLM class flavin-dependent oxidoreductase	QEN71_11810	paras_002911	protein-codi	NZ_CP1252	chromosom	2630031	2631107	+	1077	0	7	206	5	158	
QEN71_RS11815	class II aldolase/adducin family protein	QEN71_11815	paras_002912	protein-codi	NZ_CP1252	chromosom	2631119	2631934	+	815	0	12	724	8	591	
QEN71_RS11820	cysteine dioxygenase	QEN71_11820	paras_002913	protein-codi	NZ_CP1252	chromosom	2631934	2632530	+	592	0	9	532	9	532	
QEN71_RS11825	rhodanese-related sulfurtransferase	QEN71_11825	paras_002914	protein-codi	NZ_CP1252	chromosom	2632527	2634134	+	1604	0	32	3388	23	2322	
QEN71_RS11830	LysR family transcriptional regulator	QEN71_11830	paras_002915	protein-codi	NZ_CP1252	chromosom	2634141	2635052	-	912	0	12	644	12	644	
QEN71_RS11835	ABC transporter substrate-binding protein	QEN71_11835	paras_002916	protein-codi	NZ_CP1252	chromosom	2635243	2636199	+	957	0	10	895	8	718	
QEN71_RS11840	acyl-CoA dehydrogenase family protein	QEN71_11840	paras_002917	protein-codi	NZ_CP1252	chromosom	2636207	2637433	+	1223	0	10	723	6	505	
QEN71_RS11845	LLM class flavin-dependent oxidoreductase	QEN71_11845	paras_002918	protein-codi	NZ_CP1252	chromosom	2637430	2638533	+	1100	0	13	452	9	427	
QEN71_RS11850	ABC transporter substrate-binding protein	QEN71_11850	paras_002919	protein-codi	NZ_CP1252	chromosom	2638586	2639620	-	1035	0	8	651	6	568	

QEN71_RS11855	ABC transporter ATP-binding protein	QEN71_11855	paras_002920	protein-codi	NZ_CP1252	chromosom	2639665	2640453	-	782	0	10	493	8	481	
QEN71_RS11860	ABC transporter permease subunit	QEN71_11860	paras_002921	protein-codi	NZ_CP1252	chromosom	2640447	2641472	-	1019	0	17	1304	17	1304	
QEN71_RS11865	ABC transporter substrate-binding protein	QEN71_11865	paras_002922	protein-codi	NZ_CP1252	chromosom	2641502	2642503	-	1002	0	12	1109	9	578	
QEN71_RS11870	alkanesulfonate monooxygenase	partial;pseudo;QEN71_11870		pseudogene	NZ_CP1252	chromosom	2642752	2642937	-	186	0	6	445	4	362	
QEN71_RS11875	Na/Pi symporter	QEN71_11875	paras_002924	protein-codi	NZ_CP1252	chromosom	2643498	2645402	+	1905	0	46	5848	33	4047	
QEN71_RS11880	cation transporter	partial;pseudo;QEN71_11880		pseudogene	NZ_CP1252	chromosom	2645437	2645793	-	314	0	4	241	3	239	
QEN71_RS11885	metal-sensing transcriptional repressor	partial;pseudo;QEN71_11885		pseudogene	NZ_CP1252	chromosom	2645751	2645957	-	164	0	5	2004	5	2004	
QEN71_RS11890	TnsD family Tn7-like transposition protein	QEN71_11890	paras_002927	protein-codi	NZ_CP1252	chromosom	2646406	2648247	-	1842	0	70	7516	60	5830	
QEN71_RS11895	ATP-binding protein	QEN71_11895	paras_002929	protein-codi	NZ_CP1252	chromosom	2650303	2651847	-	1545	0	33	2270	28	2024	
QEN71_RS11900	DDE-type integrase/transposase/recombinase	QEN71_11900	paras_002930	protein-codi	NZ_CP1252	chromosom	2651857	2654292	-	2436	0	46	2285	38	1716	
QEN71_RS11905	TnsA endonuclease C-terminal domain-containing p	QEN71_11905	paras_002931	protein-codi	NZ_CP1252	chromosom	2654334	2655149	-	816	0	11	850	10	834	
QEN71_RS11910	hypothetical protein	QEN71_11910	paras_002932	protein-codi	NZ_CP1252	chromosom	2655393	2656181	-	789	0	14	1316	11	1113	
QEN71_RS11915	hypothetical protein	QEN71_11915	paras_002933	protein-codi	NZ_CP1252	chromosom	2656906	2658063	+	1158	0	48	7731	43	7071	
QEN71_RS11920	DoxX family protein	QEN71_11920	paras_002934	protein-codi	NZ_CP1252	chromosom	2658411	2658821	-	411	0	11	640	8	434	
QEN71_RS11925	LysE family translocator	QEN71_11925	paras_002935	protein-codi	NZ_CP1252	chromosom	2659408	2660034	+	627	0	5	205	3	149	
QEN71_RS11930	arginase family protein	QEN71_11930	paras_002936	protein-codi	NZ_CP1252	chromosom	2660219	2661091	+	873	0	26	2154	16	1096	
QEN71_RS11935	alkene reductase	QEN71_11935	paras_002937	protein-codi	NZ_CP1252	chromosom	2661134	2662225	-	1092	0	30	2192	21	1806	
QEN71_RS11940	MFS transporter	QEN71_11940	paras_002938	protein-codi	NZ_CP1252	chromosom	2662293	2663648	-	1356	0	27	1218	24	1183	
QEN71_RS11945	GMC family oxidoreductase N-terminal domain-cont	QEN71_11945	paras_002939	protein-codi	NZ_CP1252	chromosom	2663765	2665489	-	1725	0	36	2759	28	2028	
QEN71_RS11950	NADH:flavin oxidoreductase/NADH oxidase	QEN71_11950	paras_002940	protein-codi	NZ_CP1252	chromosom	2665724	2666770	-	1047	0	15	1101	7	710	
QEN71_RS11955	helix-turn-helix domain-containing protein	QEN71_11955	paras_002941	protein-codi	NZ_CP1252	chromosom	2666994	2667929	+	936	0	14	734	11	547	
QEN71_RS11960	arginase family protein	QEN71_11960	paras_002942	protein-codi	NZ_CP1252	chromosom	2667991	2668881	-	891	0	10	444	7	265	
QEN71_RS11965	hypothetical protein			protein-codi	NZ_CP1252	chromosom	2669002	2669244	-	243	0	4	96	3	62	
QEN71_RS11970	EthD domain-containing protein	QEN71_11970	paras_002943	protein-codi	NZ_CP1252	chromosom	2669595	2670005	+	411	0	13	1393	12	1334	
QEN71_RS11975	helix-turn-helix domain-containing protein	QEN71_11975	paras_002944	protein-codi	NZ_CP1252	chromosom	2670053	2670991	-	939	0	26	3684	20	2379	
QEN71_RS11980	hypothetical protein	QEN71_11980	paras_002945	protein-codi	NZ_CP1252	chromosom	2671138	2671515	-	378	0	7	367	7	367	
QEN71_RS11985	porin	QEN71_11985	paras_002946	protein-codi	NZ_CP1252	chromosom	2671809	2672894	+	1086	0	20	1416	19	1339	
QEN71_RS11990	sugar ABC transporter substrate-binding protein	QEN71_11990	paras_002947	protein-codi	NZ_CP1252	chromosom	2672907	2673905	+	999	0	13	876	11	709	
QEN71_RS11995	sugar ABC transporter ATP-binding protein	QEN71_11995	paras_002948	protein-codi	NZ_CP1252	chromosom	2673988	2675496	+	1505	0	32	3099	28	2802	
QEN71_RS12000	ABC transporter permease	QEN71_12000	paras_002949	protein-codi	NZ_CP1252	chromosom	2675493	2676500	+	1004	0	11	1189	10	1017	
QEN71_RS12005	ABC transporter permease	QEN71_12005	paras_002950	protein-codi	NZ_CP1252	chromosom	2676524	2677465	+	942	0	21	1865	12	798	
QEN71_RS12010	GMC family oxidoreductase N-terminal domain-cont	QEN71_12010	paras_002951	protein-codi	NZ_CP1252	chromosom	2677819	2679477	-	1659	0	56	5218	37	3433	
QEN71_RS12015	nuclear transport factor 2 family protein	QEN71_12015	paras_002952	protein-codi	NZ_CP1252	chromosom	2679507	2679938	-	432	0	8	856	3	104	
QEN71_RS12020	homoprotocatechuate degradation operon regulator	QEN71_12020	paras_002953	protein-codi	NZ_CP1252	chromosom	2680451	2680897	-	447	0	14	1675	8	635	
QEN71_RS12025	fumarylacetoacetate hydrolase family protein	QEN71_12025	paras_002954	protein-codi	NZ_CP1252	chromosom	2681014	2681775	+	758	0	14	1088	13	1084	
QEN71_RS12030	fumarylacetoacetate hydrolase family protein	QEN71_12030	paras_002955	protein-codi	NZ_CP1252	chromosom	2681772	2682530	+	755	0	18	1888	14	1713	
QEN71_RS12035	5-carboxymethyl-2-hydroxymuconate semialdehyde	QEN71_12035	paras_002956	protein-codi	NZ_CP1252	chromosom	2682548	2684008	+	1461	0	21	1517	16	1139	
QEN71_RS12040	3,4-dihydroxyphenylacetate 2,3-dioxygenase	QEN71_12040	paras_002957	protein-codi	NZ_CP1252	chromosom	2684034	2684897	+	864	0	18	2158	12	1797	
QEN71_RS12045	5-carboxymethyl-2-hydroxymuconate Delta-isomera	QEN71_12045	paras_002958	protein-codi	NZ_CP1252	chromosom	2684913	2685311	+	399	0	5	169	3	93	
QEN71_RS12050	2-oxo-hepta-3-ene-1,7-dioic acid hydratase	QEN71_12050	paras_002959	protein-codi	NZ_CP1252	chromosom	2685334	2686137	+	804	0	10	992	5	147	
QEN71_RS12055	4-hydroxy-2-oxoheptanedioate aldolase	QEN71_12055	paras_002960	protein-codi	NZ_CP1252	chromosom	2686146	2686961	+	816	0	17	1240	13	906	
QEN71_RS12060	NAD-dependent succinate-semialdehyde dehydroge	QEN71_12060	paras_002961	protein-codi	NZ_CP1252	chromosom	2686983	2688431	+	1449	0	18	1712	9	572	
QEN71_RS12065	helix-turn-helix domain-containing protein	QEN71_12065	paras_002962	protein-codi	NZ_CP1252	chromosom	2688639	2689577	-	939	0	22	3082	15	1560	
QEN71_RS12070	sugar ABC transporter substrate-binding protein	QEN71_12070	paras_002963	protein-codi	NZ_CP1252	chromosom	2690024	2691022	+	999	0	10	659	9	408	
QEN71_RS12075	cytochrome P450	QEN71_12075	paras_002964	protein-codi	NZ_CP1252	chromosom	2692019	2693248	-	1230	0	17	952	14	764	
QEN71_RS12080	PDR/VanB family oxidoreductase	QEN71_12080	paras_002965	protein-codi	NZ_CP1252	chromosom	2693275	2694222	-	948	0	15	1857	14	1843	
QEN71_RS12085	EthD family reductase	QEN71_12085	paras_002966	protein-codi	NZ_CP1252	chromosom	2694491	2694808	-	318	0	2	197	2	197	
QEN71_RS12090	cytosine permease	QEN71_12090	paras_002967	protein-codi	NZ_CP1252	chromosom	2695606	2697123	+	1518	0	25	1394	21	1167	
QEN71_RS12095	D-2-hydroxyacid dehydrogenase family protein	QEN71_12095	paras_002968	protein-codi	NZ_CP1252	chromosom	2697699	2698682	+	984	0	35	2189	25	1613	
QEN71_RS12100	aconitase family protein	QEN71_12100	paras_002969	protein-codi	NZ_CP1252	chromosom	2699063	2700994	-	1932	0	37	2654	23	1448	
QEN71_RS12105	MFS transporter	QEN71_12105	paras_002970	protein-codi	NZ_CP1252	chromosom	2701443	2702882	-	1440	0	22	2343	20	2336	
QEN71_RS12110	4-hydroxyphenylpyruvate dioxygenase	QEN71_12110	paras_002971	protein-codi	NZ_CP1252	chromosom	2702977	2704056	-	1080	0	28	2384	25	2028	
QEN71_RS12115	gamma carbonic anhydrase family protein	QEN71_12115	paras_002972	protein-codi	NZ_CP1252	chromosom	2704084	2704608	-	521	0	12	1064	6	538	
QEN71_RS12120	CaiB/BaiF CoA-transferase family protein	QEN71_12120	paras_002973	protein-codi	NZ_CP1252	chromosom	2704605	2705756	-	1148	0	21	2092	15	1757	
QEN71_RS12125	hydroxymethylglutaryl-CoA lyase	QEN71_12125	paras_002974	protein-codi	NZ_CP1252	chromosom	2705768	2706718	-	951	0	11	1651	7	1553	
QEN71_RS12130	LysR family transcriptional regulator	QEN71_12130	paras_002975	protein-codi	NZ_CP1252	chromosom	2706849	2707745	+	897	0	21	1940	18	1536	
QEN71_RS12135	LysR substrate-binding domain-containing protein	QEN71_12135	paras_002976	protein-codi	NZ_CP1252	chromosom	2707975	2708889	-	915	0	18	1646	14	1067	
QEN71_RS12140	CoA ester lyase	QEN71_12140	paras_002977	protein-codi	NZ_CP1252	chromosom	2708951	2709901	-	947	0	12	1438	7	998	
QEN71_RS12145	CaiB/BaiF CoA-transferase family protein	QEN71_12145	paras_002978	protein-codi	NZ_CP1252	chromosom	2709898	2711076	-	1175	0	26	2870	22	2775	
QEN71_RS12150	L-lactate permease	QEN71_12150	paras_002979	protein-codi	NZ_CP1252	chromosom	2711113	2712888	-	1776	0	24	1708	14	833	
QEN71_RS12155	D-2-hydroxyacid dehydrogenase	QEN71_12155	paras_002980	protein-codi	NZ_CP1252	chromosom	2713290	2714234	+	945	0	22	1566	15	828	
QEN71_RS12160	LysR substrate-binding domain-containing protein	QEN71_12160	paras_002981	protein-codi	NZ_CP1252	chromosom	2714586	2715491	+	906	0	27	1430	22	1151	

QEN71_RS12165	CoA transferase	QEN71_12165	paras_002982	protein-codi	NZ_CP1252	chromosom	2715648	2716802	+	1155	0	30	2010	26	1839	
QEN71_RS12170	hydroxymethylglutaryl-CoA lyase	QEN71_12170	paras_002983	protein-codi	NZ_CP1252	chromosom	2716820	2717785	+	966	0	15	1317	13	1305	
QEN71_RS12175	VOC family protein	QEN71_12175	paras_002984	protein-codi	NZ_CP1252	chromosom	2717787	2718170	+	384	0	5	286	2	41	
QEN71_RS12180	dicarboxylate/amino acid:cation symporter	QEN71_12180	paras_002985	protein-codi	NZ_CP1252	chromosom	2718298	2719611	+	1314	0	32	1369	24	962	
QEN71_RS12185	D-2-hydroxyacid dehydrogenase	QEN71_12185	paras_002986	protein-codi	NZ_CP1252	chromosom	2719715	2720698	+	984	0	16	996	11	795	
QEN71_RS12190	glycerate kinase	QEN71_12190	paras_002987	protein-codi	NZ_CP1252	chromosom	2720970	2722112	+	1143	0	24	2402	19	2146	
QEN71_RS12195	pyruvate kinase	QEN71_12195	paras_002988	protein-codi	NZ_CP1252	chromosom	2722157	2723605	+	1449	0	22	2437	20	2137	
QEN71_RS12200	sugar diacid recognition domain-containing protein	QEN71_12200	paras_002989	protein-codi	NZ_CP1252	chromosom	2723638	2724816	+	1179	0	36	3581	27	2736	
QEN71_RS12205	PLP-dependent aminotransferase family protein	QEN71_12205	paras_002990	protein-codi	NZ_CP1252	chromosom	2725863	2727314	-	1452	0	41	6244	31	3627	
QEN71_RS12210	EAL domain-containing protein	QEN71_12210	paras_002991	protein-codi	NZ_CP1252	chromosom	2728241	2729116	+	876	0	17	1308	15	1137	
QEN71_RS12215	PLP-dependent aminotransferase family protein	QEN71_12215	paras_002992	protein-codi	NZ_CP1252	chromosom	2729182	2730633	+	1452	0	29	2471	22	1919	
QEN71_RS12220	GGDEF and EAL domain-containing protein	QEN71_12220	paras_002993	protein-codi	NZ_CP1252	chromosom	2730890	2732575	+	1686	0	51	8912	42	7750	
QEN71_RS12225	hypothetical protein	QEN71_12225	paras_002994	protein-codi	NZ_CP1252	chromosom	2733032	2733457	-	426	835.0	12	2222	12	2222	
QEN71_RS12230	DUF2235 domain-containing protein	QEN71_12230	paras_002995	protein-codi	NZ_CP1252	chromosom	2733657	2735102	-	1446	2757.0	49	15311	39	11737	
QEN71_RS12235	DUF3304 domain-containing protein	QEN71_12235	paras_002996	protein-codi	NZ_CP1252	chromosom	2735260	2735706	-	447	877.0	19	3387	15	2364	
QEN71_RS12240	DUF4123 domain-containing protein	QEN71_12240	paras_002997	protein-codi	NZ_CP1252	chromosom	2735754	2736557	-	804	1536.0	34	10436	31	9799	
QEN71_RS12245	type VI secretion system Vgr family protein	QEN71_12245	paras_002998	protein-codi	NZ_CP1252	chromosom	2736566	2739118	-	2553	5040.0	89	33117	69	25659	
QEN71_RS12250	hypothetical protein	QEN71_12250	paras_002999	protein-codi	NZ_CP1252	chromosom	2739571	2739861	-	291	573.0	24	13785	20	12372	
QEN71_RS12255	hypothetical protein	QEN71_12255	paras_003000	protein-codi	NZ_CP1252	chromosom	2740139	2740534	-	396	0	19	4013	9	2285	
QEN71_RS12260	hypothetical protein	QEN71_12260	paras_003001	protein-codi	NZ_CP1252	chromosom	2740860	2741435	+	576	0	34	6499	26	4904	
QEN71_RS12265	hypothetical protein	QEN71_12265		protein-codi	NZ_CP1252	chromosom	2741524	2741826	-	303	501.0	18	2931	9	1541	
QEN71_RS12270	hypothetical protein	QEN71_12270	paras_003002	protein-codi	NZ_CP1252	chromosom	2742130	2743074	-	945	1866.0	24	4817	21	4236	
QEN71_RS12275	HAD domain-containing protein	QEN71_12275	paras_003003	protein-codi	NZ_CP1252	chromosom	2743920	2744480	-	561	0	16	2141	11	924	
QEN71_RS12280	HAD domain-containing protein	QEN71_12280	paras_003004	protein-codi	NZ_CP1252	chromosom	2744676	2745236	-	561	0	15	1637	9	1015	
QEN71_RS12285	hypothetical protein	QEN71_12285	paras_003005	protein-codi	NZ_CP1252	chromosom	2745356	2745847	-	492	0	11	1311	11	1311	
QEN71_RS12290	hypothetical protein	QEN71_12290	paras_003006	protein-codi	NZ_CP1252	chromosom	2746596	2747093	-	498	0	11	2349	10	1689	
QEN71_RS12295	helix-turn-helix transcriptional regulator	QEN71_12295	paras_003007	protein-codi	NZ_CP1252	chromosom	2747369	2747752	+	384	0	6	1203	4	1096	
QEN71_RS12300	DNA-binding protein	QEN71_12300	paras_003008	protein-codi	NZ_CP1252	chromosom	2748030	2749064	+	1031	0	15	1306	9	513	
QEN71_RS12305	tyrosine-type recombinase/integrase	QEN71_12305	paras_003009	protein-codi	NZ_CP1252	chromosom	2749061	2750716	+	1652	0	29	3101	20	2312	
QEN71_RS12310	AAA domain-containing protein	QEN71_12310	paras_003010	protein-codi	NZ_CP1252	chromosom	2752149	2756813	+	4661	0	170	24307	133	17977	
QEN71_RS12315	hypothetical protein	QEN71_12315	paras_003011	protein-codi	NZ_CP1252	chromosom	2756810	2758183	+	1370	0	35	3968	30	3498	
QEN71_RS12320	hypothetical protein	QEN71_12320	paras_003012	protein-codi	NZ_CP1252	chromosom	2758736	2760544	-	1762	0	58	4220	46	3556	
QEN71_RS12325	hypothetical protein	QEN71_12325	paras_003013	protein-codi	NZ_CP1252	chromosom	2760498	2761964	-	1420	0	43	4833	37	3949	
QEN71_RS12330	hypothetical protein	QEN71_12330	paras_003014	protein-codi	NZ_CP1252	chromosom	2763389	2764510	+	1122	0	38	8502	31	6110	
QEN71_RS12335	DUF2623 family protein	QEN71_12335	paras_003015	protein-codi	NZ_CP1252	chromosom	2764513	2764845	+	333	0	15	4816	13	4538	
QEN71_RS12340	hypothetical protein	QEN71_12340	paras_003016	protein-codi	NZ_CP1252	chromosom	2764958	2765377	+	420	0	12	2909	5	553	
QEN71_RS12345	PadR family transcriptional regulator	QEN71_12345	paras_003017	protein-codi	NZ_CP1252	chromosom	2766265	2767074	+	810	0	18	2990	15	2131	
QEN71_RS12350	siderophore-interacting protein	QEN71_12350	paras_003018	protein-codi	NZ_CP1252	chromosom	2767097	2767915	+	819	0	14	4185	11	3076	
QEN71_RS12355	MFS transporter	QEN71_12355	paras_003019	protein-codi	NZ_CP1252	chromosom	2767941	2769209	+	1269	0	22	5611	19	4946	
QEN71_RS12360	xanthine dehydrogenase family protein molybdopter	QEN71_12360	paras_003020	protein-codi	NZ_CP1252	chromosom	2769227	2771455	-	2229	0	50	7992	43	6126	
QEN71_RS12365	xanthine dehydrogenase family protein subunit M	QEN71_12365	paras_003021	protein-codi	NZ_CP1252	chromosom	2771479	2772480	-	1002	0	13	2486	13	2486	
QEN71_RS12370	(2Fe-2S)-binding protein	QEN71_12370	paras_003022	protein-codi	NZ_CP1252	chromosom	2772491	2773036	-	546	0	5	472	4	457	
QEN71_RS12375	hypothetical protein	QEN71_12375	paras_003023	protein-codi	NZ_CP1252	chromosom	2773462	2774436	+	971	0	25	4571	17	2754	
QEN71_RS12380	enoyl-CoA hydratase/isomerase family protein	QEN71_12380	paras_003024	protein-codi	NZ_CP1252	chromosom	2774433	2775578	-	1138	0	18	3541	18	3541	
QEN71_RS12385	enoyl-CoA hydratase	QEN71_12385	paras_003025	protein-codi	NZ_CP1252	chromosom	2775575	2776372	-	793	0	6	238	5	228	
QEN71_RS12390	3-hydroxyisobutyrate dehydrogenase	QEN71_12390	paras_003026	protein-codi	NZ_CP1252	chromosom	2776372	2777265	-	893	0	34	5334	27	4733	
QEN71_RS12395	CoA-acylating methylmalonate-semialdehyde dehyd	QEN71_12395	paras_003027	protein-codi	NZ_CP1252	chromosom	2777876	2779405	-	1530	0	35	5706	28	4982	
QEN71_RS12400	AMP-binding protein	QEN71_12400	paras_003028	protein-codi	NZ_CP1252	chromosom	2779453	2781162	-	1710	0	37	6960	28	5285	
QEN71_RS12405	acyl-CoA dehydrogenase	QEN71_12405	paras_003029	protein-codi	NZ_CP1252	chromosom	2781219	2782352	-	1134	0	25	3953	16	2273	
QEN71_RS12410	AraC family transcriptional regulator	QEN71_12410	paras_003030	protein-codi	NZ_CP1252	chromosom	2782597	2783661	+	1065	0	26	9589	20	8808	
QEN71_RS12415	nodulation protein NfeD	QEN71_12415	paras_003031	protein-codi	NZ_CP1252	chromosom	2783740	2785371	+	1628	0	30	12067	27	11584	
QEN71_RS12420	slipin family protein	QEN71_12420	paras_003032	protein-codi	NZ_CP1252	chromosom	2785368	2786147	+	776	0	15	2570	10	1204	
QEN71_RS12425	DUF4148 domain-containing protein	QEN71_12425	paras_003033	protein-codi	NZ_CP1252	chromosom	2786291	2786542	+	252	0	10	1414	6	500	
QEN71_RS12430	NCS2 family permease	QEN71_12430	paras_003034	protein-codi	NZ_CP1252	chromosom	2786941	2788242	+	1302	0	38	5951	23	4013	
QEN71_RS12435	DUF1488 domain-containing protein	QEN71_12435	paras_003035	protein-codi	NZ_CP1252	chromosom	2788888	2789178	-	291	0	16	3697	12	2508	
QEN71_RS12440	DUF2964 domain-containing protein	QEN71_12440	paras_003036	protein-codi	NZ_CP1252	chromosom	2789433	2789699	-	267	0	9	3389	7	2930	
QEN71_RS12445	sigma-54-dependent Fis family transcriptional regul	QEN71_12445	paras_003037	protein-codi	NZ_CP1252	chromosom	2789986	2791923	+	1938	103.0	26	4987	15	3202	
QEN71_RS12450	2Fe-2S iron-sulfur cluster-binding protein	QEN71_12450	paras_003038	protein-codi	NZ_CP1252	chromosom	2792000	2792386	+	387	0	13	5533	11	4413	
QEN71_RS12455	DUF1653 domain-containing protein	QEN71_12455	paras_003039	protein-codi	NZ_CP1252	chromosom	2792411	2792608	-	198	0	10	4459	5	2925	
QEN71_RS12460	PAS domain-containing protein	QEN71_12460	paras_003040	protein-codi	NZ_CP1252	chromosom	2792681	2793325	-	645	0	18	9952	13	7956	
QEN71_RS12465	ornithine cyclodeaminase family protein	QEN71_12465	paras_003041	protein-codi	NZ_CP1252	chromosom	2793418	2794383	-	966	0	24	5659	19	4109	
QEN71_RS12470	ABC transporter substrate-binding protein	QEN71_12470	paras_003042	protein-codi	NZ_CP1252	chromosom	2794712	2795491	+	780	0	13	2971	11	2392	

QEN71_RS12475	FAD-binding oxidoreductase	QEN71_12475	paras_003043	protein-codi	NZ_CP1252	chromosom	2795613	2796857	+	1245	0	26	5112	17	3845	
QEN71_RS12480	type II toxin-antitoxin system RelE/ParE family toxin	QEN71_12480	paras_003044	protein-codi	NZ_CP1252	chromosom	2796970	2797323	+	354	0	9	964	7	650	
QEN71_RS12485	XRE family transcriptional regulator	QEN71_12485	paras_003045	protein-codi	NZ_CP1252	chromosom	2797342	2797626	+	285	0	4	354	2	117	
QEN71_RS12490	methyl-accepting chemotaxis protein	QEN71_12490	paras_003046	protein-codi	NZ_CP1252	chromosom	2797679	2799235	-	1557	0	17	3579	10	1559	
QEN71_RS12495	AEC family transporter	QEN71_12495	paras_003047	protein-codi	NZ_CP1252	chromosom	2799392	2800342	-	951	0	15	9291	12	6247	
QEN71_RS12500	LysR family transcriptional regulator	QEN71_12500	paras_003048	protein-codi	NZ_CP1252	chromosom	2800450	2801319	+	870	0	13	3087	10	2189	
QEN71_RS12505	CbtA family protein	QEN71_12505	paras_003049	protein-codi	NZ_CP1252	chromosom	2801386	2802153	-	768	0	16	4829	10	2626	
QEN71_RS12510	CbtB domain-containing protein	QEN71_12510	paras_003050	protein-codi	NZ_CP1252	chromosom	2802175	2802399	-	225	0	5	7645	5	7645	
QEN71_RS12515	histidine phosphatase family protein	QEN71_12515	paras_003051	protein-codi	NZ_CP1252	chromosom	2802634	2803248	+	577	0	14	10730	11	9989	
QEN71_RS12520	hypothetical protein	QEN71_12520	paras_003052	protein-codi	NZ_CP1252	chromosom	2803211	2803645	-	397	0	6	1727	6	1727	
QEN71_RS12525	hypothetical protein	QEN71_12525	paras_003053	protein-codi	NZ_CP1252	chromosom	2803825	2804043	+	219	0	6	1090	2	323	
QEN71_RS12530	hypothetical protein	QEN71_12530	paras_003054	protein-codi	NZ_CP1252	chromosom	2804558	2804842	+	285	0	4	1921	1	439	
QEN71_RS12535	translation initiation factor IF-1	QEN71_12535	paras_003055	protein-codi	NZ_CP1252	chromosom	2804968	2805231	+	264	0	12	3301	12	3301	
QEN71_RS12540	GntR family transcriptional regulator	QEN71_12540	paras_003056	protein-codi	NZ_CP1252	chromosom	2805397	2806104	-	708	0	13	5492	11	5238	
QEN71_RS12545	cyanate transporter	QEN71_12545	paras_003057	protein-codi	NZ_CP1252	chromosom	2806288	2807550	+	1263	0	19	4662	14	3468	
QEN71_RS12550	ANTAR domain-containing protein	QEN71_12550	paras_003058	protein-codi	NZ_CP1252	chromosom	2807820	2808395	+	576	0	8	812	6	626	
QEN71_RS12555	CmpA/NrtA family ABC transporter substrate-bindin	QEN71_12555	paras_003059	protein-codi	NZ_CP1252	chromosom	2808406	2809539	+	1134	0	26	2032	24	1986	
QEN71_RS12560	choline ABC transporter substrate-binding protein	QEN71_12560	paras_003060	protein-codi	NZ_CP1252	chromosom	2810179	2811120	-	942	0	20	1845	14	1313	
QEN71_RS12565	GlxA family transcriptional regulator	QEN71_12565	paras_003061	protein-codi	NZ_CP1252	chromosom	2811178	2812155	-	978	0	11	653	11	653	
QEN71_RS12570	choline ABC transporter permease subunit	QEN71_12570	paras_003062	protein-codi	NZ_CP1252	chromosom	2812289	2813194	-	898	0	5	300	3	195	
QEN71_RS12575	glycine betaine/L-proline ABC transporter ATP-bind	partial;pseudo;QEN71_12575		pseudogene	NZ_CP1252	chromosom	2813187	2814491	-	1297	0	21	2980	13	1368	
QEN71_RS12580	IlvD/Edd family dehydratase	QEN71_12580	paras_003064	protein-codi	NZ_CP1252	chromosom	2814833	2816545	+	1713	0	16	1851	11	1214	
QEN71_RS12585	dihydrodipicolinate synthase family protein	QEN71_12585	paras_003065	protein-codi	NZ_CP1252	chromosom	2816552	2817484	+	933	0	10	548	9	542	
QEN71_RS12590	hypothetical protein	QEN71_12590	paras_003066	protein-codi	NZ_CP1252	chromosom	2817555	2817776	-	222	0	1	34	0	0	
QEN71_RS12595	response regulator transcription factor	QEN71_12595	paras_003067	protein-codi	NZ_CP1252	chromosom	2818223	2818876	+	654	0	4	73	3	44	
QEN71_RS12600	rhamnose ABC transporter substrate-binding protei	QEN71_12600	paras_003068	protein-codi	NZ_CP1252	chromosom	2818950	2819957	-	1008	0	4	309	4	309	
QEN71_RS12605	ABC transporter permease	QEN71_12605	paras_003069	protein-codi	NZ_CP1252	chromosom	2820034	2821062	-	1021	0	8	990	8	990	
QEN71_RS12610	ABC transporter permease	QEN71_12610	paras_003070	protein-codi	NZ_CP1252	chromosom	2821055	2822116	-	1050	0	7	420	7	420	
QEN71_RS12615	sugar ABC transporter ATP-binding protein	QEN71_12615	paras_003071	protein-codi	NZ_CP1252	chromosom	2822113	2823621	-	1505	0	9	594	7	525	
QEN71_RS12620	amidohydrolase family protein	QEN71_12620	paras_003072	protein-codi	NZ_CP1252	chromosom	2823684	2824577	-	894	0	18	2808	12	1965	
QEN71_RS12625	L-rhamnose mutarotase	QEN71_12625	paras_003073	protein-codi	NZ_CP1252	chromosom	2824624	2824950	-	327	0	8	1434	8	1434	
QEN71_RS12630	LysR family transcriptional regulator	QEN71_12630	paras_003074	protein-codi	NZ_CP1252	chromosom	2825109	2826068	+	960	0	17	1701	15	1341	
QEN71_RS12635	SDR family NAD(P)-dependent oxidoreductase	QEN71_12635	paras_003075	protein-codi	NZ_CP1252	chromosom	2826704	2827486	-	783	0	28	4421	25	3246	
QEN71_RS12640	L-rhamnonate dehydratase	QEN71_12640	paras_003076	protein-codi	NZ_CP1252	chromosom	2827504	2828682	-	1179	0	24	5100	22	5003	
QEN71_RS12645	MFS transporter	QEN71_12645	paras_003077	protein-codi	NZ_CP1252	chromosom	2828723	2830054	-	1332	0	25	4714	23	4131	
QEN71_RS12650	SDR family NAD(P)-dependent oxidoreductase	QEN71_12650	paras_003078	protein-codi	NZ_CP1252	chromosom	2830150	2830902	-	753	0	16	3050	11	1645	
QEN71_RS12655	GDSL-type esterase/lipase family protein	QEN71_12655	paras_003079	protein-codi	NZ_CP1252	chromosom	2831066	2831944	-	879	0	19	3986	14	3070	
QEN71_RS12660	hypothetical protein	QEN71_12660	paras_003080	protein-codi	NZ_CP1252	chromosom	2832202	2832990	+	789	0	10	1611	7	1075	
QEN71_RS12665	CHAT domain-containing protein	QEN71_12665	paras_003081	protein-codi	NZ_CP1252	chromosom	2833036	2835906	-	2871	0	48	11468	44	10532	
QEN71_RS12670	hypothetical protein	QEN71_12670	paras_003082	protein-codi	NZ_CP1252	chromosom	2835989	2836741	-	753	0	14	2230	13	2219	
QEN71_RS12675	toll/interleukin-1 receptor domain-containing protein	QEN71_12675	paras_003083	protein-codi	NZ_CP1252	chromosom	2837092	2838177	+	1086	0	21	4254	12	2919	
QEN71_RS12680	NAD(P)H-dependent oxidoreductase	QEN71_12680	paras_003084	protein-codi	NZ_CP1252	chromosom	2838514	2839185	+	672	0	16	2645	16	2645	
QEN71_RS12685	transcriptional regulator	QEN71_12685	paras_003085	protein-codi	NZ_CP1252	chromosom	2839569	2839793	+	225	0	12	1146	8	1049	
QEN71_RS12690	ferritin-like domain-containing protein	QEN71_12690	paras_003086	protein-codi	NZ_CP1252	chromosom	2839933	2840841	+	909	0	16	2854	11	1488	
QEN71_RS12695	indoleacetamide hydrolase	QEN71_12695	paras_003087	protein-codi	NZ_CP1252	chromosom	2840928	2842382	+	1455	0	28	4191	25	3209	
QEN71_RS12700	CDP-diacylglycerol diphosphatase	QEN71_12700	paras_003088	protein-codi	NZ_CP1252	chromosom	2842389	2843138	+	750	0	11	2356	11	2356	
QEN71_RS12705	fatty acid desaturase	QEN71_12705	paras_003089	protein-codi	NZ_CP1252	chromosom	2843278	2844258	+	981	0	31	8685	26	7499	
QEN71_RS12710	M20 family metallopeptidase	QEN71_12710	paras_003090	protein-codi	NZ_CP1252	chromosom	2844279	2845547	-	1269	0	23	2701	17	1767	
QEN71_RS12715	DUF305 domain-containing protein	QEN71_12715	paras_003091	protein-codi	NZ_CP1252	chromosom	2845785	2846252	+	464	0	6	593	4	336	
QEN71_RS12720	YncE family protein	QEN71_12720	paras_003092	protein-codi	NZ_CP1252	chromosom	2846249	2847736	+	1484	0	31	4541	29	4256	
QEN71_RS12725	hypothetical protein	QEN71_12725	paras_003093	protein-codi	NZ_CP1252	chromosom	2847832	2848095	+	264	0	11	1713	10	1555	
QEN71_RS12730	MFS transporter	QEN71_12730	paras_003094	protein-codi	NZ_CP1252	chromosom	2848130	2849485	-	1356	0	26	3608	20	2790	
QEN71_RS12735	ectoine utilization protein EutA	QEN71_12735	paras_003095	protein-codi	NZ_CP1252	chromosom	2849580	2850359	-	780	0	15	1162	15	1162	
QEN71_RS12740	ectoine/hydroxyectoine ABC transporter permease s	QEN71_12740	paras_003096	protein-codi	NZ_CP1252	chromosom	2850363	2851022	-	660	0	13	956	12	901	
QEN71_RS12745	ectoine/hydroxyectoine ABC transporter permease s	QEN71_12745	paras_003097	protein-codi	NZ_CP1252	chromosom	2851027	2851686	-	660	0	13	1428	10	1286	
QEN71_RS12750	ectoine/hydroxyectoine ABC transporter substrate-b	QEN71_12750	paras_003098	protein-codi	NZ_CP1252	chromosom	2851761	2852600	-	840	0	5	277	5	277	
QEN71_RS12755	ectoine/hydroxyectoine ABC transporter ATP-bindin	QEN71_12755	paras_003099	protein-codi	NZ_CP1252	chromosom	2852665	2853567	-	903	0	4	139	1	6	TRUE
QEN71_RS12760	ectoine hydrolase DoeA	QEN71_12760	paras_003100	protein-codi	NZ_CP1252	chromosom	2853990	2855210	+	1221	0	24	2398	21	1992	
QEN71_RS12765	alcohol dehydrogenase family protein	QEN71_12765	paras_003101	protein-codi	NZ_CP1252	chromosom	2855284	2856378	+	1095	0	16	1284	14	1109	
QEN71_RS12770	porin	QEN71_12770	paras_003102	protein-codi	NZ_CP1252	chromosom	2856542	2857687	+	1146	0	31	2243	22	1804	
QEN71_RS12775	MOSC and FAD-binding oxidoreductase domain-co	QEN71_12775	paras_003103	protein-codi	NZ_CP1252	chromosom	2857694	2859460	-	1767	0	28	3203	23	2686	
QEN71_RS12780	hypothetical protein	QEN71_12780	paras_003104	protein-codi	NZ_CP1252	chromosom	2859464	2859751	-	288	0	4	264	1	46	

QEN71_RS12785	acetamidase/formamidase family protein	QEN71_12785	paras_003105	protein-codi	NZ_CP1252	chromosom	2859931	2861097	-	1167	0	20	1558	15	1338	
QEN71_RS12790	SDR family oxidoreductase	QEN71_12790	paras_003106	protein-codi	NZ_CP1252	chromosom	2861354	2862076	-	723	0	11	459	7	376	
QEN71_RS12795	hypothetical protein	QEN71_12795	paras_003107	protein-codi	NZ_CP1252	chromosom	2862111	2862467	-	357	0	5	582	2	181	
QEN71_RS12800	acyltransferase	QEN71_12800	paras_003108	protein-codi	NZ_CP1252	chromosom	2862655	2863884	-	1230	0	20	1369	17	1294	
QEN71_RS12805	DUF308 domain-containing protein	QEN71_12805	paras_003109	protein-codi	NZ_CP1252	chromosom	2864270	2864848	+	579	0	5	195	5	195	
QEN71_RS12810	hypothetical protein	QEN71_12810	paras_003110	protein-codi	NZ_CP1252	chromosom	2864861	2865250	-	390	0	7	429	5	371	
QEN71_RS12815	hypothetical protein	QEN71_12815	paras_003111	protein-codi	NZ_CP1252	chromosom	2865510	2866646	-	1137	0	27	1804	23	1435	
QEN71_RS12820	filamentous hemagglutinin N-terminal domain-conta	QEN71_12820	paras_003112	protein-codi	NZ_CP1252	chromosom	2867379	2869805	-	2427	0	41	7030	33	4972	
QEN71_RS12825	hypothetical protein	QEN71_12825	paras_003113	protein-codi	NZ_CP1252	chromosom	2869925	2870215	-	289	0	7	2152	6	2150	
QEN71_RS12830	filamentous hemagglutinin N-terminal domain-conta	QEN71_12830	paras_003114	protein-codi	NZ_CP1252	chromosom	2870214	2872493	+	2278	0	32	7714	28	7055	
QEN71_RS12835	ShlB/FhaC/HecB family hemolysin secretion/activat	QEN71_12835	paras_003115	protein-codi	NZ_CP1252	chromosom	2872582	2874312	+	1731	0	39	6721	30	5960	
QEN71_RS12840	glycosyltransferase family 9 protein	QEN71_12840	paras_003116	protein-codi	NZ_CP1252	chromosom	2874440	2875630	+	1191	0	15	2055	13	2005	
QEN71_RS12845	GGDEF domain-containing protein	QEN71_12845	paras_003117	protein-codi	NZ_CP1252	chromosom	2875868	2877019	+	1152	0	11	954	10	782	
QEN71_RS12850	PhnD/SsuA/transferrin family substrate-binding prot	QEN71_12850	paras_003118	protein-codi	NZ_CP1252	chromosom	2877073	2877861	+	789	0	18	3610	13	2205	
QEN71_RS12855	acetyl/propionyl/methylcrotonyl-CoA carboxylase su	QEN71_12855	paras_003119	protein-codi	NZ_CP1252	chromosom	2877884	2879896	-	2013	0	27	3198	18	2510	
QEN71_RS12860	enoyl-CoA hydratase/isomerase family protein	QEN71_12860	paras_003120	protein-codi	NZ_CP1252	chromosom	2879940	2880725	-	786	0	12	1096	9	877	
QEN71_RS12865	carboxyl transferase domain-containing protein	QEN71_12865	paras_003121	protein-codi	NZ_CP1252	chromosom	2880751	2882358	-	1608	0	28	5241	22	3833	
QEN71_RS12870	isovaleryl-CoA dehydrogenase	QEN71_12870	paras_003122	protein-codi	NZ_CP1252	chromosom	2882378	2883559	-	1182	0	28	5867	23	4011	
QEN71_RS12875	TetR/AcrR family transcriptional regulator	QEN71_12875	paras_003123	protein-codi	NZ_CP1252	chromosom	2883884	2884648	+	765	0	13	1912	7	1360	
QEN71_RS12880	hypothetical protein	QEN71_12880	paras_003124	protein-codi	NZ_CP1252	chromosom	2884736	2884891	+	156	0	1	10	0	0	
QEN71_RS12885	PRC-barrel domain-containing protein	QEN71_12885	paras_003125	protein-codi	NZ_CP1252	chromosom	2884948	2885361	+	414	0	10	2815	5	1519	
QEN71_RS12890	LysR family transcriptional regulator	QEN71_12890	paras_003126	protein-codi	NZ_CP1252	chromosom	2885416	2886324	-	909	0	16	3821	11	2429	
QEN71_RS12895	MFS transporter	QEN71_12895	paras_003127	protein-codi	NZ_CP1252	chromosom	2886447	2887982	+	1536	0	23	4020	21	3730	
QEN71_RS12900	glycine zipper family protein	QEN71_12900	paras_003128	protein-codi	NZ_CP1252	chromosom	2888044	2888625	-	582	0	28	3724	16	1847	
QEN71_RS12905	MarR family winged helix-turn-helix transcriptional r	QEN71_12905	paras_003129	protein-codi	NZ_CP1252	chromosom	2888833	2889366	+	530	0	2	86	2	86	
QEN71_RS12910	MFS transporter	QEN71_12910	paras_003130	protein-codi	NZ_CP1252	chromosom	2889363	2890565	+	1199	0	20	4158	17	3503	
QEN71_RS12915	aldehyde dehydrogenase	QEN71_12915	paras_003131	protein-codi	NZ_CP1252	chromosom	2890605	2892098	-	1494	0	27	3116	22	2379	
QEN71_RS12920	cupin domain-containing protein	QEN71_12920	paras_003132	protein-codi	NZ_CP1252	chromosom	2892199	2892750	-	552	0	14	1424	12	1115	
QEN71_RS12925	hypothetical protein	QEN71_12925		protein-codi	NZ_CP1252	chromosom	2893188	2893394	+	207	0	7	1086	4	526	
QEN71_RS12930	gamma-glutamyl-gamma-aminobutyrate hydrolase f	QEN71_12930	paras_003133	protein-codi	NZ_CP1252	chromosom	2893699	2894487	+	789	0	6	745	6	745	
QEN71_RS12935	glutamine synthetase family protein	QEN71_12935	paras_003134	protein-codi	NZ_CP1252	chromosom	2894521	2895855	+	1335	0	20	1303	19	1151	
QEN71_RS12940	aspartate aminotransferase family protein	QEN71_12940	paras_003135	protein-codi	NZ_CP1252	chromosom	2895917	2897362	+	1446	0	27	3665	23	2742	
QEN71_RS12945	polyamine ABC transporter substrate-binding protei	QEN71_12945	paras_003136	protein-codi	NZ_CP1252	chromosom	2897450	2898571	+	1122	0	21	1444	20	1442	
QEN71_RS12950	polyamine ABC transporter ATP-binding protein	partial;pseudo;QEN71_12950		protein-codi	NZ_CP1252	chromosom	2898784	2899947	+	1160	0	15	1120	12	788	
QEN71_RS12955	ABC transporter permease subunit	QEN71_12955	paras_003138	protein-codi	NZ_CP1252	chromosom	2899944	2900921	+	970	0	19	928	16	824	
QEN71_RS12960	ABC transporter permease subunit	QEN71_12960	paras_003139	protein-codi	NZ_CP1252	chromosom	2900918	2901835	+	914	0	3	293	1	195	TRUE
QEN71_RS12965	DUF3138 family protein	QEN71_12965	paras_003140	protein-codi	NZ_CP1252	chromosom	2901874	2903448	+	1575	0	36	2454	32	2178	
QEN71_RS12970	FAD-binding oxidoreductase	QEN71_12970	paras_003141	protein-codi	NZ_CP1252	chromosom	2903498	2904790	+	1289	0	32	3425	31	3319	
QEN71_RS12975	FAD-dependent oxidoreductase	pseudo;QEN71_12975		pseudogene	NZ_CP1252	chromosom	2904787	2906084	+	1294	0	21	2586	15	1968	
QEN71_RS12980	DUF2891 domain-containing protein	QEN71_12980	paras_003143	protein-codi	NZ_CP1252	chromosom	2906226	2907236	+	1011	0	15	2099	10	1640	
QEN71_RS12985	MFS transporter	QEN71_12985	paras_003144	protein-codi	NZ_CP1252	chromosom	2907329	2908777	-	1449	0	33	3572	28	3215	
QEN71_RS12990	SulP family inorganic anion transporter	QEN71_12990	paras_003145	protein-codi	NZ_CP1252	chromosom	2908861	2910354	-	1494	0	9	855	5	223	
QEN71_RS12995	DUF1488 family protein	QEN71_12995	paras_003146	protein-codi	NZ_CP1252	chromosom	2910703	2910972	-	270	0	3	75	1	41	
QEN71_RS13000	DMT family transporter	QEN71_13000	paras_003147	protein-codi	NZ_CP1252	chromosom	2911448	2912335	+	888	0	13	2848	11	2469	
QEN71_RS13005	SRPBCC family protein	QEN71_13005	paras_003148	protein-codi	NZ_CP1252	chromosom	2912358	2912894	-	537	0	11	1181	8	1122	
QEN71_RS13010	DNA-binding protein	QEN71_13010	paras_003149	protein-codi	NZ_CP1252	chromosom	2913057	2913464	+	408	0	8	1396	3	238	
QEN71_RS13015	fructose bisphosphate aldolase	QEN71_13015	paras_003150	protein-codi	NZ_CP1252	chromosom	2913612	2914508	+	897	0	15	1622	11	1493	
QEN71_RS13020	PRC-barrel domain-containing protein	QEN71_13020	paras_003151	protein-codi	NZ_CP1252	chromosom	2914580	2915047	-	468	0	19	5689	15	4503	
QEN71_RS13025	DUF2970 domain-containing protein	QEN71_13025	paras_003152	protein-codi	NZ_CP1252	chromosom	2915323	2915511	+	189	0	2	14	2	14	
QEN71_RS13030	PLP-dependent aminotransferase family protein	QEN71_13030	paras_003153	protein-codi	NZ_CP1252	chromosom	2915522	2916943	-	1422	0	36	4503	24	1996	
QEN71_RS13035	MFS transporter	QEN71_13035	paras_003154	protein-codi	NZ_CP1252	chromosom	2917022	2918554	-	1533	0	14	2047	10	1638	
QEN71_RS13040	LysR substrate-binding domain-containing protein	QEN71_13040	paras_003155	protein-codi	NZ_CP1252	chromosom	2918685	2919563	+	879	0	11	1132	8	1010	
QEN71_RS13045	DUF1488 domain-containing protein	QEN71_13045	paras_003156	protein-codi	NZ_CP1252	chromosom	2919783	2920043	+	261	0	4	736	4	736	
QEN71_RS13050	H-NS family nucleoid-associated regulatory protein	QEN71_13050	paras_003157	protein-codi	NZ_CP1252	chromosom	2920273	2920848	-	576	0	15	1504	10	909	
QEN71_RS13055	hypothetical protein	QEN71_13055	paras_003158	protein-codi	NZ_CP1252	chromosom	2920944	2921231	+	288	0	18	2433	18	2433	
QEN71_RS13060	DUF4148 domain-containing protein	QEN71_13060	paras_003159	protein-codi	NZ_CP1252	chromosom	2921349	2921657	-	309	0	16	2080	16	2080	
QEN71_RS13065	response regulator	QEN71_13065	paras_003160	protein-codi	NZ_CP1252	chromosom	2921860	2922225	-	366	0	2	313	0	0	
QEN71_RS13070	hypothetical protein	QEN71_13070	paras_003161	protein-codi	NZ_CP1252	chromosom	2922307	2922846	-	536	0	11	1151	11	1151	
QEN71_RS13075	methyl-accepting chemotaxis protein	QEN71_13075	paras_003162	protein-codi	NZ_CP1252	chromosom	2922843	2923583	-	737	0	8	750	6	723	
QEN71_RS13080	MCP four helix bundle domain-containing protein	QEN71_13080	paras_003163	protein-codi	NZ_CP1252	chromosom	2923600	2925990	-	2391	0	28	3126	20	2434	
QEN71_RS13085	YihY/virulence factor BrkB family protein	QEN71_13085	paras_003164	protein-codi	NZ_CP1252	chromosom	2926235	2927137	-	903	0	22	2189	19	1983	
QEN71_RS13090	sugar ABC transporter substrate-binding protein	QEN71_13090	paras_003165	protein-codi	NZ_CP1252	chromosom	2927624	2928652	+	1029	0	10	932	8	469	

QEN71_RS13095	ABC transporter permease	QEN71_13095	paras_003166	protein-codi	NZ_CP1252	chromosom	2928774	2929763	+	990	0	21	2752	17	1828	
QEN71_RS13100	ATP-binding cassette domain-containing protein	QEN71_13100	paras_003167	protein-codi	NZ_CP1252	chromosom	2929787	2930596	+	810	0	1	27	1	27	TRUE
QEN71_RS13105	ROK family transcriptional regulator	QEN71_13105	paras_003168	protein-codi	NZ_CP1252	chromosom	2930627	2931844	+	1218	0	25	2080	17	1171	
QEN71_RS13110	transglycosylase domain-containing protein	QEN71_13110	paras_003169	protein-codi	NZ_CP1252	chromosom	2932470	2935526	-	3057	0	74	8730	49	5255	
QEN71_RS13115	FAD-binding oxidoreductase	QEN71_13115	paras_003170	protein-codi	NZ_CP1252	chromosom	2935878	2937155	+	1278	0	37	7239	26	4165	
QEN71_RS13120	TetR family transcriptional regulator	QEN71_13120	paras_003171	protein-codi	NZ_CP1252	chromosom	2937273	2937956	+	684	0	26	6107	19	5329	
QEN71_RS13125	EAL domain-containing protein	QEN71_13125	paras_003172	protein-codi	NZ_CP1252	chromosom	2938596	2941430	-	2835	0	79	12526	65	11090	
QEN71_RS13130	cupin domain-containing protein	QEN71_13130	paras_003173	protein-codi	NZ_CP1252	chromosom	2941715	2942257	+	543	0	7	1545	2	71	
QEN71_RS13135	ABC transporter permease	QEN71_13135	paras_003174	protein-codi	NZ_CP1252	chromosom	2942294	2943172	-	875	0	17	2816	16	2592	
QEN71_RS13140	ABC transporter ATP-binding protein	QEN71_13140	paras_003175	protein-codi	NZ_CP1252	chromosom	2943169	2943996	-	824	0	7	590	7	590	
QEN71_RS13145	ABC transporter substrate-binding protein	QEN71_13145	paras_003176	protein-codi	NZ_CP1252	chromosom	2944265	2945317	-	1053	0	21	2569	19	2549	
QEN71_RS13150	TauD/TfdA family dioxygenase	QEN71_13150	paras_003177	protein-codi	NZ_CP1252	chromosom	2945363	2946241	-	879	0	23	8325	22	8290	
QEN71_RS13155	hypothetical protein	QEN71_13155	paras_003178	protein-codi	NZ_CP1252	chromosom	2946543	2946989	+	447	0	14	2692	9	2184	
QEN71_RS13160	porin	QEN71_13160	paras_003179	protein-codi	NZ_CP1252	chromosom	2947293	2948444	+	1152	0	48	12877	36	8452	
QEN71_RS13165	DUF4148 domain-containing protein	QEN71_13165	paras_003180	protein-codi	NZ_CP1252	chromosom	2948650	2948952	+	303	0	10	2219	9	2168	
QEN71_RS13170	hypothetical protein	QEN71_13170	paras_003181	protein-codi	NZ_CP1252	chromosom	2949336	2949674	+	339	0	7	1651	5	1306	
QEN71_RS13175	DUF72 domain-containing protein	QEN71_13175	paras_003182	protein-codi	NZ_CP1252	chromosom	2949698	2950516	-	819	0	16	3333	11	1640	
QEN71_RS13180	SDR family oxidoreductase	QEN71_13180	paras_003183	protein-codi	NZ_CP1252	chromosom	2950627	2951415	-	789	0	19	4243	18	4238	
QEN71_RS13185	porin	QEN71_13185	paras_003184	protein-codi	NZ_CP1252	chromosom	2951726	2952850	+	1125	0	40	12702	33	9698	
QEN71_RS13190	hypothetical protein	QEN71_13190	paras_003185	protein-codi	NZ_CP1252	chromosom	2953209	2953394	+	186	0	5	714	1	70	
QEN71_RS13195	2-dehydropantoate 2-reductase	QEN71_13195	paras_003186	protein-codi	NZ_CP1252	chromosom	2953456	2954484	-	1029	0	21	5833	15	4539	
QEN71_RS13200	fumarylacetoacetate hydrolase family protein	QEN71_13200	paras_003187	protein-codi	NZ_CP1252	chromosom	2954658	2955455	-	798	0	17	3572	16	3494	
QEN71_RS13205	formyl-CoA transferase	QEN71_13205	paras_003188	protein-codi	NZ_CP1252	chromosom	2955525	2956772	-	1248	329.0	28	4876	28	4876	
QEN71_RS13210	hypothetical protein	QEN71_13210	paras_003189	protein-codi	NZ_CP1252	chromosom	2957667	2957894	-	228	0	9	1169	7	944	
QEN71_RS13215	PAS domain-containing protein	QEN71_13215	paras_003190	protein-codi	NZ_CP1252	chromosom	2957990	2958421	-	432	0	14	1986	11	1191	
QEN71_RS13220	formyl-CoA transferase	QEN71_13220	paras_003191	protein-codi	NZ_CP1252	chromosom	2958543	2959793	-	1251	0	23	4673	20	4051	
QEN71_RS13225	oxalyl-CoA decarboxylase	QEN71_13225	paras_003192	protein-codi	NZ_CP1252	chromosom	2959820	2961559	-	1740	0	37	8357	28	5211	
QEN71_RS13230	GntR family transcriptional regulator	QEN71_13230	paras_003193	protein-codi	NZ_CP1252	chromosom	2961775	2962494	-	720	0	15	8258	13	7397	
QEN71_RS13235	hypothetical protein	QEN71_13235	paras_003194	protein-codi	NZ_CP1252	chromosom	2962775	2963155	+	381	0	18	2885	17	2860	
QEN71_RS13240	oxalate/formate MFS antiporter	QEN71_13240	paras_003195	protein-codi	NZ_CP1252	chromosom	2963752	2965077	-	1326	0	51	16931	42	14901	
QEN71_RS13245	GntR family transcriptional regulator	QEN71_13245	paras_003196	protein-codi	NZ_CP1252	chromosom	2965512	2966231	-	720	0	12	3801	12	3801	
QEN71_RS13250	hypothetical protein	QEN71_13250	paras_003197	protein-codi	NZ_CP1252	chromosom	2966443	2966742	-	300	0	0	0	0	0	TRUE
QEN71_RS13255	hypothetical protein	QEN71_13255	paras_003198	protein-codi	NZ_CP1252	chromosom	2966813	2967535	-	723	0	40	9443	31	6997	
QEN71_RS13260	metallophosphoesterase	QEN71_13260	paras_003199	protein-codi	NZ_CP1252	chromosom	2967703	2969055	+	1353	0	37	12030	26	9198	
QEN71_RS13265	DUF1428 domain-containing protein	QEN71_13265	paras_003200	protein-codi	NZ_CP1252	chromosom	2969072	2969425	-	354	0	8	1822	3	493	
QEN71_RS13270	DUF2946 domain-containing protein	QEN71_13270	paras_003201	protein-codi	NZ_CP1252	chromosom	2969636	2970022	+	387	0	6	513	6	513	
QEN71_RS13275	hypothetical protein	QEN71_13275	paras_003202	protein-codi	NZ_CP1252	chromosom	2970175	2971020	+	846	0	19	3354	19	3354	
QEN71_RS13280	copper resistance protein CopC	QEN71_13280	paras_003203	protein-codi	NZ_CP1252	chromosom	2971034	2971396	+	363	0	9	2230	4	1298	
QEN71_RS13285	mechanosensitive ion channel family protein	QEN71_13285	paras_003204	protein-codi	NZ_CP1252	chromosom	2971599	2973062	+	1464	0	27	1864	17	1042	
QEN71_RS13290	ABC transporter ATP-binding protein	QEN71_13290	paras_003205	protein-codi	NZ_CP1252	chromosom	2973758	2974471	+	706	0	10	1044	5	334	
QEN71_RS13295	FtsX-like permease family protein	QEN71_13295	paras_003206	protein-codi	NZ_CP1252	chromosom	2974464	2977049	+	2577	0	27	5613	22	5255	
QEN71_RS13300	carotenoid 1,2-hydratase	QEN71_13300	paras_003207	protein-codi	NZ_CP1252	chromosom	2977049	2978158	+	1109	0	18	5678	16	5197	
QEN71_RS13305	DUF4148 domain-containing protein	QEN71_13305	paras_003208	protein-codi	NZ_CP1252	chromosom	2978313	2978624	+	312	0	7	1490	4	619	
QEN71_RS13310	sorbosone dehydrogenase family protein	QEN71_13310	paras_003209	protein-codi	NZ_CP1252	chromosom	2978654	2979982	-	1329	0	30	5564	27	5505	
QEN71_RS13315	TAXI family TRAP transporter solute-binding subuni	QEN71_13315	paras_003210	protein-codi	NZ_CP1252	chromosom	2980668	2982023	-	1356	0	37	9928	29	8173	
QEN71_RS13320	tRNA-Val	QEN71_13320		tRNA	NZ_CP1252	chromosom	2982202	2982278	+	77	152.0	3	392	1	48	
QEN71_RS13325	cytochrome o ubiquinol oxidase subunit IV	QEN71_13325	paras_003212	protein-codi	NZ_CP1252	chromosom	2982451	2982792	-	341	0	5	984	5	984	
QEN71_RS13330	cytochrome o ubiquinol oxidase subunit III	QEN71_13330	paras_003213	protein-codi	NZ_CP1252	chromosom	2982792	2983406	-	613	0	9	2298	5	1332	
QEN71_RS13335	cytochrome o ubiquinol oxidase subunit I	QEN71_13335	paras_003214	protein-codi	NZ_CP1252	chromosom	2983406	2985418	-	2012	98.0	54	11581	41	7393	
QEN71_RS13340	ubiquinol oxidase subunit II	QEN71_13340	paras_003215	protein-codi	NZ_CP1252	chromosom	2985422	2986321	-	900	0	18	2117	9	1693	
QEN71_RS13345	DUF3597 domain-containing protein	QEN71_13345	paras_003216	protein-codi	NZ_CP1252	chromosom	2986648	2987046	-	399	0	3	84	3	84	
QEN71_RS13350	OmpA family protein	QEN71_13350	paras_003217	protein-codi	NZ_CP1252	chromosom	2987344	2987844	+	501	0	9	1295	4	792	
QEN71_RS13355	septal ring lytic transglycosylase RlpA family protein	QEN71_13355	paras_003218	protein-codi	NZ_CP1252	chromosom	2988020	2988547	+	524	0	13	874	11	761	
QEN71_RS13360	hypothetical protein	QEN71_13360	paras_003219	protein-codi	NZ_CP1252	chromosom	2988544	2988891	+	344	0	6	853	2	638	
QEN71_RS13365	branched-chain amino acid ABC transporter substra	QEN71_13365	paras_003220	protein-codi	NZ_CP1252	chromosom	2988925	2990067	-	1143	0	27	2947	20	2291	
QEN71_RS13370	hypothetical protein	QEN71_13370	paras_003221	protein-codi	NZ_CP1252	chromosom	2990389	2990664	+	276	0	2	399	0	0	
QEN71_RS13375	alkyl hydroperoxide reductase subunit F	QEN71_13375	paras_003222	protein-codi	NZ_CP1252	chromosom	2990748	2992340	-	1593	0	18	2054	12	1722	
QEN71_RS13380	alkyl hydroperoxide reductase subunit C	QEN71_13380	paras_003223	protein-codi	NZ_CP1252	chromosom	2992524	2993087	-	564	0	15	2955	11	2148	
QEN71_RS13385	DNA polymerase II	QEN71_13385	paras_003224	protein-codi	NZ_CP1252	chromosom	2993334	2995712	+	2379	0	80	17215	61	14657	
QEN71_RS13390	trypsin-like peptidase domain-containing protein	QEN71_13390	paras_003225	protein-codi	NZ_CP1252	chromosom	2996380	2997405	-	1026	0	21	5590	17	5109	
QEN71_RS13395	hypothetical protein	QEN71_13395	paras_003226	protein-codi	NZ_CP1252	chromosom	2997505	2997696	-	192	0	9	2063	2	719	
QEN71_RS13400	hypothetical protein	QEN71_13400	paras_003227	protein-codi	NZ_CP1252	chromosom	2997855	2998046	-	192	0	14	2407	13	1956	

QEN71_RS13405	hypothetical protein	QEN71_13405	paras_003228	protein-codi	NZ_CP1252	chromosom	2998390	2998674	+	285	0	1	6	1	6	
QEN71_RS13410	hypothetical protein	QEN71_13410		protein-codi	NZ_CP1252	chromosom	2998747	2998890	+	144	0	5	1715	3	1398	
QEN71_RS13415	SulP family inorganic anion transporter	QEN71_13415	paras_003229	protein-codi	NZ_CP1252	chromosom	2999038	3000600	-	1563	226.0	16	3435	10	2505	
QEN71_RS13420	carbonic anhydrase	QEN71_13420	paras_003230	protein-codi	NZ_CP1252	chromosom	3000719	3001375	-	657	141.0	10	1524	5	1023	
QEN71_RS13425	DUF427 domain-containing protein	QEN71_13425	paras_003231	protein-codi	NZ_CP1252	chromosom	3001700	3002077	-	378	0	18	5748	16	5572	
QEN71_RS13430	DUF3331 domain-containing protein	QEN71_13430	paras_003232	protein-codi	NZ_CP1252	chromosom	3002605	3003180	+	576	0	9	2184	9	2184	
QEN71_RS13435	LysR family transcriptional regulator	QEN71_13435	paras_003233	protein-codi	NZ_CP1252	chromosom	3003284	3004306	+	1023	0	17	4350	14	4288	
QEN71_RS13440	YbaK/EbsC family protein	QEN71_13440	paras_003234	protein-codi	NZ_CP1252	chromosom	3004362	3004916	+	555	0	1	16	1	16	TRUE
QEN71_RS13445	NAD(P)H:quinone oxidoreductase	QEN71_13445	paras_003235	protein-codi	NZ_CP1252	chromosom	3004937	3005536	-	600	0	17	3907	12	2446	
QEN71_RS13450	DUF4142 domain-containing protein	QEN71_13450	paras_003236	protein-codi	NZ_CP1252	chromosom	3005760	3006335	+	576	0	10	3458	6	2556	
QEN71_RS13455	MgtC/SapB family protein	QEN71_13455	paras_003237	protein-codi	NZ_CP1252	chromosom	3006388	3007095	-	708	0	9	3848	7	3314	
QEN71_RS13460	hypothetical protein	QEN71_13460	paras_003238	protein-codi	NZ_CP1252	chromosom	3007473	3007829	+	357	0	13	6217	7	1821	
QEN71_RS13465	hypothetical protein	QEN71_13465	paras_003239	protein-codi	NZ_CP1252	chromosom	3007844	3008212	+	369	0	9	3889	7	2846	
QEN71_RS13470	lytic transglycosylase domain-containing protein	QEN71_13470	paras_003240	protein-codi	NZ_CP1252	chromosom	3008318	3008740	+	423	0	12	3597	10	3194	
QEN71_RS13475	helix-turn-helix domain-containing protein	QEN71_13475	paras_003241	protein-codi	NZ_CP1252	chromosom	3008753	3009157	-	405	0	20	9077	18	8403	
QEN71_RS13480	hypothetical protein	QEN71_13480	paras_003242	protein-codi	NZ_CP1252	chromosom	3009799	3010191	+	393	0	17	3717	15	3371	
QEN71_RS13485	hypothetical protein	QEN71_13485	paras_003243	protein-codi	NZ_CP1252	chromosom	3010237	3010839	+	603	0	11	4101	6	3160	
QEN71_RS13490	DUF2964 family protein	QEN71_13490	paras_003244	protein-codi	NZ_CP1252	chromosom	3010923	3011150	+	228	0	7	1959	5	1884	
QEN71_RS13495	DUF484 family protein	QEN71_13495	paras_003245	protein-codi	NZ_CP1252	chromosom	3011157	3012251	-	1095	0	13	3133	10	1573	
QEN71_RS13500	YqaE/Pmp3 family membrane protein	QEN71_13500	paras_003246	protein-codi	NZ_CP1252	chromosom	3012399	3012593	-	195	0	6	1355	4	613	
QEN71_RS13505	YbjQ family protein	QEN71_13505	paras_003247	protein-codi	NZ_CP1252	chromosom	3012624	3012959	-	336	0	13	2955	8	1103	
QEN71_RS13510	sulfite exporter TauE/SafE family protein	QEN71_13510	paras_003248	protein-codi	NZ_CP1252	chromosom	3012997	3013719	-	723	0	12	3328	10	2822	
QEN71_RS13515	DUF4148 domain-containing protein	QEN71_13515	paras_003249	protein-codi	NZ_CP1252	chromosom	3013934	3014761	+	828	0	10	1379	10	1379	
QEN71_RS13520	acyl-CoA dehydrogenase family protein	QEN71_13520	paras_003250	protein-codi	NZ_CP1252	chromosom	3014867	3016021	+	1155	0	34	8249	27	6807	
QEN71_RS13525	DUF3141 domain-containing protein	QEN71_13525	paras_003251	protein-codi	NZ_CP1252	chromosom	3016430	3018730	+	2301	0	52	10368	42	8430	
QEN71_RS13530	phosphate acetyltransferase	QEN71_13530	paras_003252	protein-codi	NZ_CP1252	chromosom	3018788	3019753	+	966	0	8	1521	5	428	
QEN71_RS13535	acetate/propionate family kinase	QEN71_13535	paras_003253	protein-codi	NZ_CP1252	chromosom	3019774	3020955	+	1182	0	17	3118	11	2328	
QEN71_RS13540	enoyl-ACP reductase FabI	QEN71_13540	paras_003254	protein-codi	NZ_CP1252	chromosom	3020958	3021737	+	780	0	24	5611	20	4542	
QEN71_RS13545	RNA-binding protein	QEN71_13545	paras_003255	protein-codi	NZ_CP1252	chromosom	3021752	3022057	+	306	0	16	5223	12	3373	
QEN71_RS13550	hypothetical protein	QEN71_13550	paras_003256	protein-codi	NZ_CP1252	chromosom	3022286	3022540	+	255	0	2	341	0	0	
QEN71_RS13555	RNA polymerase sigma factor RpoD	QEN71_13555	paras_003257	protein-codi	NZ_CP1252	chromosom	3022784	3024781	+	1998	0	6	122	6	122	
QEN71_RS13560	PRC-barrel domain-containing protein	QEN71_13560	paras_003258	protein-codi	NZ_CP1252	chromosom	3024914	3025354	-	441	0	7	1649	4	563	
QEN71_RS13565	hypothetical protein	QEN71_13565	paras_003259	protein-codi	NZ_CP1252	chromosom	3025503	3026327	-	825	0	18	2810	10	1282	
QEN71_RS13570	spherulation-specific family 4 protein	QEN71_13570	paras_003260	protein-codi	NZ_CP1252	chromosom	3026539	3027273	-	735	0	33	4058	30	3669	
QEN71_RS13575	fumarylacetoacetate hydrolase family protein	QEN71_13575	paras_003261	protein-codi	NZ_CP1252	chromosom	3027677	3028858	-	1182	0	10	2577	5	1551	
QEN71_RS13580	IlvD/Edd family dehydratase	QEN71_13580	paras_003262	protein-codi	NZ_CP1252	chromosom	3028897	3030690	-	1794	0	33	4592	24	2474	
QEN71_RS13585	LacI family DNA-binding transcriptional regulator	QEN71_13585	paras_003263	protein-codi	NZ_CP1252	chromosom	3030847	3031857	-	1011	0	19	2184	19	2184	
QEN71_RS13590	SMP-30/gluconolactonase/LRE family protein	QEN71_13590	paras_003264	protein-codi	NZ_CP1252	chromosom	3032002	3032892	-	891	0	25	4739	23	4623	
QEN71_RS13595	methyltransferase regulatory domain-containing pro	QEN71_13595	paras_003265	protein-codi	NZ_CP1252	chromosom	3033022	3034605	-	1584	0	47	12418	39	11048	
QEN71_RS13600	uroporphyrinogen-III C-methyltransferase	QEN71_13600	paras_003266	protein-codi	NZ_CP1252	chromosom	3034958	3035722	+	765	0	9	2465	9	2465	
QEN71_RS13605	CBS domain-containing protein	QEN71_13605	paras_003267	protein-codi	NZ_CP1252	chromosom	3035728	3036339	-	612	0	21	5305	15	4716	
QEN71_RS13610	tRNA 2-selenouridine(34) synthase MnmH	QEN71_13610	paras_003268	protein-codi	NZ_CP1252	chromosom	3036553	3037599	+	1047	0	25	7046	22	6462	
QEN71_RS13615	bifunctional diguanylate cyclase/phosphodiesterase	QEN71_13615	paras_003269	protein-codi	NZ_CP1252	chromosom	3037630	3039972	-	2343	0	36	5758	24	4573	
QEN71_RS13620	glutathione transferase	QEN71_13620	paras_003270	protein-codi	NZ_CP1252	chromosom	3040211	3040846	-	636	0	12	1580	7	1035	
QEN71_RS13625	GNAT family N-acetyltransferase	QEN71_13625	paras_003271	protein-codi	NZ_CP1252	chromosom	3040886	3041398	-	513	0	12	1534	9	1358	
QEN71_RS13630	hypothetical protein	QEN71_13630	paras_003272	protein-codi	NZ_CP1252	chromosom	3041628	3041774	+	147	0	2	24	0	0	
QEN71_RS13635	hypothetical protein	QEN71_13635	paras_003273	protein-codi	NZ_CP1252	chromosom	3041788	3042405	+	618	0	3	383	1	34	TRUE
QEN71_RS13640	TonB-dependent siderophore receptor	QEN71_13640	paras_003274	protein-codi	NZ_CP1252	chromosom	3042482	3044722	-	2241	0	82	9354	68	7761	
QEN71_RS13645	gamma-glutamylcyclotransferase family protein	QEN71_13645	paras_003275	protein-codi	NZ_CP1252	chromosom	3044974	3045336	+	363	0	10	2102	7	655	
QEN71_RS13650	hypothetical protein	QEN71_13650	paras_003276	protein-codi	NZ_CP1252	chromosom	3045466	3046005	+	540	0	12	1069	10	933	
QEN71_RS13655	hypothetical protein	QEN71_13655	paras_003277	protein-codi	NZ_CP1252	chromosom	3046034	3046435	-	402	0	10	1688	9	1635	
QEN71_RS13660	aldehyde dehydrogenase family protein	QEN71_13660	paras_003278	protein-codi	NZ_CP1252	chromosom	3046722	3048143	-	1422	0	26	4963	16	3327	
QEN71_RS13665	LysR family transcriptional regulator	QEN71_13665	paras_003279	protein-codi	NZ_CP1252	chromosom	3048246	3049205	+	960	0	15	2709	10	2306	
QEN71_RS13670	YciI family protein	QEN71_13670	paras_003280	protein-codi	NZ_CP1252	chromosom	3049302	3049739	+	438	0	4	476	2	215	
QEN71_RS13675	ABC transporter permease	QEN71_13675	paras_003281	protein-codi	NZ_CP1252	chromosom	3049767	3050933	-	1167	0	10	3170	7	2791	
QEN71_RS13680	ABC transporter permease	QEN71_13680	paras_003282	protein-codi	NZ_CP1252	chromosom	3050946	3052100	-	1155	0	24	3692	23	3687	
QEN71_RS13685	ABC transporter ATP-binding protein	QEN71_13685	paras_003283	protein-codi	NZ_CP1252	chromosom	3052122	3052850	-	725	0	9	617	7	137	
QEN71_RS13690	efflux RND transporter periplasmic adaptor subunit	QEN71_13690	paras_003284	protein-codi	NZ_CP1252	chromosom	3052847	3054055	-	1205	0	15	3291	15	3291	
QEN71_RS13695	class I SAM-dependent methyltransferase	QEN71_13695	paras_003285	protein-codi	NZ_CP1252	chromosom	3054260	3055117	+	858	0	20	3842	15	3223	
QEN71_RS13700	tannase/feruloyl esterase family alpha/beta hydrolas	QEN71_13700	paras_003286	protein-codi	NZ_CP1252	chromosom	3055130	3056938	-	1809	0	32	5510	24	3956	
QEN71_RS13705	MFS transporter	QEN71_13705	paras_003287	protein-codi	NZ_CP1252	chromosom	3057382	3058638	+	1257	0	13	3162	9	2355	
QEN71_RS13710	helix-turn-helix domain-containing protein	QEN71_13710	paras_003288	protein-codi	NZ_CP1252	chromosom	3058654	3059112	-	459	0	10	2300	8	2161	

QEN71_RS13715	isochorismatase family cysteine hydrolase	QEN71_13715	paras_003289	protein-codi	NZ_CP1252	chromosom	3059433	3060083	+	647	0	13	2210	8	1677	
QEN71_RS13720	aldehyde dehydrogenase (NADP(+))	QEN71_13720	paras_003290	protein-codi	NZ_CP1252	chromosom	3060080	3061657	+	1574	0	14	1481	12	1141	
QEN71_RS13725	hypothetical protein	QEN71_13725	paras_003291	protein-codi	NZ_CP1252	chromosom	3061844	3062023	+	180	0	5	540	4	479	
QEN71_RS13730	alpha/beta hydrolase	QEN71_13730	paras_003292	protein-codi	NZ_CP1252	chromosom	3062064	3063050	+	987	0	11	2749	7	1185	
QEN71_RS13735	hypothetical protein	QEN71_13735	paras_003293	protein-codi	NZ_CP1252	chromosom	3063134	3063415	+	282	0	5	1332	4	1325	
QEN71_RS13740	S8 family serine peptidase	QEN71_13740	paras_003294	protein-codi	NZ_CP1252	chromosom	3063496	3065064	+	1569	0	32	10730	30	10538	
QEN71_RS13745	NAD(+) diphosphatase	QEN71_13745	paras_003295	protein-codi	NZ_CP1252	chromosom	3065140	3066099	+	960	0	11	2305	7	1671	
QEN71_RS13750	MFS transporter	QEN71_13750	paras_003296	protein-codi	NZ_CP1252	chromosom	3066295	3067824	+	1530	0	39	5960	29	4518	
QEN71_RS13755	LysR family transcriptional regulator	QEN71_13755	paras_003297	protein-codi	NZ_CP1252	chromosom	3067910	3068857	+	948	0	30	3719	24	2644	
QEN71_RS13760	FecR domain-containing protein	QEN71_13760	paras_003298	protein-codi	NZ_CP1252	chromosom	3069071	3069535	+	461	0	4	346	3	263	
QEN71_RS13765	OmpA family protein	QEN71_13765	paras_003299	protein-codi	NZ_CP1252	chromosom	3069532	3070158	+	623	0	7	552	4	233	
QEN71_RS13770	CHASE2 domain-containing protein	QEN71_13770	paras_003300	protein-codi	NZ_CP1252	chromosom	3070212	3071777	+	1550	0	25	3351	22	3064	
QEN71_RS13775	hypothetical protein	QEN71_13775	paras_003301	protein-codi	NZ_CP1252	chromosom	3071762	3072043	-	266	0	3	407	3	407	
QEN71_RS13780	hypothetical protein	QEN71_13780	paras_003302	protein-codi	NZ_CP1252	chromosom	3072516	3073679	-	1164	0	19	1436	13	828	
QEN71_RS13785	hypothetical protein	QEN71_13785	paras_003303	protein-codi	NZ_CP1252	chromosom	3073790	3074128	-	339	0	6	565	4	516	
QEN71_RS13790	undecaprenyl-phosphate glucose phosphotransfera	QEN71_13790	paras_003304	protein-codi	NZ_CP1252	chromosom	3074272	3075648	-	1377	0	18	2165	12	1608	
QEN71_RS13795	mannose-1-phosphate guanylyltransferase/mannos	QEN71_13795	paras_003305	protein-codi	NZ_CP1252	chromosom	3075690	3077162	-	1473	0	24	2110	17	1401	
QEN71_RS13800	O-antigen ligase family protein	QEN71_13800	paras_003306	protein-codi	NZ_CP1252	chromosom	3077935	3079245	+	1311	0	37	3537	28	2586	
QEN71_RS13805	phosphatase PAP2 family protein	QEN71_13805	paras_003307	protein-codi	NZ_CP1252	chromosom	3079271	3079900	+	630	0	9	1007	7	661	
QEN71_RS13810	glycosyltransferase family 4 protein	QEN71_13810	paras_003308	protein-codi	NZ_CP1252	chromosom	3079910	3081112	-	1203	0	14	1955	14	1955	
QEN71_RS13815	oligosaccharide flippase family protein	QEN71_13815	paras_003309	protein-codi	NZ_CP1252	chromosom	3081182	3082492	-	1311	0	27	1608	19	1194	
QEN71_RS13820	glycosyltransferase family A protein	QEN71_13820	paras_003310	protein-codi	NZ_CP1252	chromosom	3082526	3083536	-	1011	0	25	1126	16	568	
QEN71_RS13825	hypothetical protein	QEN71_13825	paras_003311	protein-codi	NZ_CP1252	chromosom	3083586	3085034	-	1449	0	27	3091	22	2384	
QEN71_RS13830	glycosyltransferase family 1 protein	QEN71_13830	paras_003312	protein-codi	NZ_CP1252	chromosom	3085054	3086169	-	1116	0	18	1054	13	760	
QEN71_RS13835	polysaccharide biosynthesis tyrosine autokinase	QEN71_13835	paras_003313	protein-codi	NZ_CP1252	chromosom	3086228	3088570	-	2343	0	17	1166	10	471	
QEN71_RS13840	helix-turn-helix domain-containing protein	QEN71_13840	paras_003314	protein-codi	NZ_CP1252	chromosom	3089615	3090550	+	936	0	15	962	10	713	
QEN71_RS13845	ATP-grasp fold amidoligase family protein	QEN71_13845	paras_003315	protein-codi	NZ_CP1252	chromosom	3090572	3091582	+	1011	0	23	3654	20	3583	
QEN71_RS13850	hypothetical protein	QEN71_13850	paras_003316	protein-codi	NZ_CP1252	chromosom	3091658	3093211	-	1554	0	61	10896	49	9239	
QEN71_RS13855	helix-turn-helix domain-containing protein	QEN71_13855	paras_003317	protein-codi	NZ_CP1252	chromosom	3094052	3095107	-	1056	0	20	2882	13	1590	
QEN71_RS13860	response regulator receiver protein	QEN71_13860	paras_003318	protein-codi	NZ_CP1252	chromosom	3095238	3096632	-	1395	0	23	8216	20	7200	
QEN71_RS13865	hypothetical protein	QEN71_13865	paras_003319	protein-codi	NZ_CP1252	chromosom	3096916	3098622	-	1707	0	67	16434	49	11118	
QEN71_RS13870	hypothetical protein	QEN71_13870		protein-codi	NZ_CP1252	chromosom	3099031	3099219	-	189	0	9	2597	7	2564	
QEN71_RS13875	NAD(P)/FAD-dependent oxidoreductase	QEN71_13875	paras_003320	protein-codi	NZ_CP1252	chromosom	3099256	3100311	+	1056	0	22	5168	10	2599	
QEN71_RS13880	leucine-rich repeat domain-containing protein	QEN71_13880	paras_003321	protein-codi	NZ_CP1252	chromosom	3100377	3101642	+	1266	0	17	1104	15	1058	
QEN71_RS13885	hypothetical protein	QEN71_13885	paras_003322	protein-codi	NZ_CP1252	chromosom	3101781	3102059	+	279	0	9	3576	4	1314	
QEN71_RS13890	cupin domain-containing protein	QEN71_13890	paras_003323	protein-codi	NZ_CP1252	chromosom	3102113	3102574	-	462	0	7	1638	5	1380	
QEN71_RS13895	DHA2 family efflux MFS transporter permease subu	QEN71_13895	paras_003324	protein-codi	NZ_CP1252	chromosom	3102723	3104159	+	1437	0	32	8765	23	5479	
QEN71_RS13900	hypothetical protein	QEN71_13900	paras_003325	protein-codi	NZ_CP1252	chromosom	3104279	3104545	+	267	0	5	271	5	271	
QEN71_RS13905	hypothetical protein	QEN71_13905	paras_003326	protein-codi	NZ_CP1252	chromosom	3104718	3104858	+	141	0	5	1485	2	384	
QEN71_RS13910	type II secretion system protein	QEN71_13910	paras_003327	protein-codi	NZ_CP1252	chromosom	3104877	3105389	-	499	0	20	4717	17	4249	
QEN71_RS13915	type II secretion system protein	QEN71_13915	paras_003328	protein-codi	NZ_CP1252	chromosom	3105376	3105774	-	378	0	10	3849	9	3661	
QEN71_RS13920	type II secretion system protein	QEN71_13920	paras_003329	protein-codi	NZ_CP1252	chromosom	3105768	3106271	-	493	0	11	5669	11	5669	
QEN71_RS13925	secretin N-terminal domain-containing protein	QEN71_13925	paras_003330	protein-codi	NZ_CP1252	chromosom	3106268	3108784	-	2513	0	38	6047	34	6020	
QEN71_RS13930	hypothetical protein	QEN71_13930	paras_003331	protein-codi	NZ_CP1252	chromosom	3108811	3109329	-	515	0	7	652	5	622	
QEN71_RS13935	hypothetical protein	QEN71_13935	paras_003332	protein-codi	NZ_CP1252	chromosom	3109326	3109913	-	580	0	14	1569	12	1115	
QEN71_RS13940	PilN domain-containing protein	QEN71_13940	paras_003333	protein-codi	NZ_CP1252	chromosom	3109910	3110452	-	535	0	3	615	3	615	
QEN71_RS13945	hypothetical protein	QEN71_13945	paras_003334	protein-codi	NZ_CP1252	chromosom	3110449	3111258	-	798	0	6	581	6	581	
QEN71_RS13950	GspE/PulE family protein	QEN71_13950	paras_003335	protein-codi	NZ_CP1252	chromosom	3111251	3112939	-	1677	0	15	2112	15	2112	
QEN71_RS13955	type II secretion system F family protein	QEN71_13955	paras_003336	protein-codi	NZ_CP1252	chromosom	3112936	3114123	-	1184	0	18	1676	15	1274	
QEN71_RS13960	type II secretion system major pseudopilin GspG	QEN71_13960	paras_003337	protein-codi	NZ_CP1252	chromosom	3114163	3114618	-	456	0	6	898	6	898	
QEN71_RS13965	discoidin domain-containing protein	QEN71_13965	paras_003338	protein-codi	NZ_CP1252	chromosom	3114702	3120293	-	5592	0	108	11387	83	9271	
QEN71_RS13970	VOC family protein	QEN71_13970	paras_003339	protein-codi	NZ_CP1252	chromosom	3121120	3121752	-	633	0	8	497	4	329	
QEN71_RS13975	hypothetical protein	QEN71_13975	paras_003340	protein-codi	NZ_CP1252	chromosom	3121898	3122083	-	184	0	0	0	0	0	
QEN71_RS13980	hypothetical protein	QEN71_13980	paras_003341	protein-codi	NZ_CP1252	chromosom	3122082	3122288	+	201	0	2	59	2	59	
QEN71_RS13985	MFS transporter	QEN71_13985	paras_003342	protein-codi	NZ_CP1252	chromosom	3122285	3123565	-	1277	0	22	1287	20	1234	
QEN71_RS13990	fumarylacetoacetate hydrolase family protein	QEN71_13990	paras_003343	protein-codi	NZ_CP1252	chromosom	3123681	3124541	-	857	0	15	1128	12	795	
QEN71_RS13995	hypothetical protein	QEN71_13995	paras_003344	protein-codi	NZ_CP1252	chromosom	3124538	3125221	-	676	0	7	630	6	235	
QEN71_RS14000	shikimate dehydrogenase	QEN71_14000	paras_003345	protein-codi	NZ_CP1252	chromosom	3125218	3126012	-	787	0	6	91	4	75	
QEN71_RS14005	CoA-transferase	QEN71_14005	paras_003346	protein-codi	NZ_CP1252	chromosom	3126009	3127631	-	1619	0	16	1723	16	1723	
QEN71_RS14010	MaoC/PaaZ C-terminal domain-containing protein	QEN71_14010	paras_003347	protein-codi	NZ_CP1252	chromosom	3127836	3128288	+	453	0	8	1406	7	1394	
QEN71_RS14015	substrate-binding domain-containing protein	QEN71_14015	paras_003348	protein-codi	NZ_CP1252	chromosom	3128476	3129450	+	975	0	19	1712	17	1589	
QEN71_RS14020	DUF2817 domain-containing protein	QEN71_14020	paras_003349	protein-codi	NZ_CP1252	chromosom	3129463	3130584	-	1122	0	26	2547	19	2081	

QEN71_RS14025	FAD-binding oxidoreductase	QEN71_14025	paras_003350	protein-codi	NZ_CP1252	chromosome	3130597	3131730	-	1134	0	20	2687	16	2182	
QEN71_RS14030	aromatic ring-hydroxylating dioxygenase subunit alp	QEN71_14030	paras_003351	protein-codi	NZ_CP1252	chromosome	3131732	3132805	-	1074	0	19	2445	14	1457	
QEN71_RS14035	LysR substrate-binding domain-containing protein	QEN71_14035	paras_003352	protein-codi	NZ_CP1252	chromosome	3132968	3133912	+	945	0	12	741	12	741	
QEN71_RS14040	phytanoyl-CoA dioxygenase family protein	QEN71_14040	paras_003353	protein-codi	NZ_CP1252	chromosome	3133914	3134750	-	837	0	12	1542	10	1295	
QEN71_RS14045	ABC transporter substrate-binding protein	QEN71_14045	paras_003354	protein-codi	NZ_CP1252	chromosome	3134801	3135574	-	774	0	16	1940	12	1765	
QEN71_RS14050	AMP-binding protein	QEN71_14050	paras_003355	protein-codi	NZ_CP1252	chromosome	3135717	3137342	-	1626	0	40	5585	31	4918	
QEN71_RS14055	SDR family NAD(P)-dependent oxidoreductase	QEN71_14055	paras_003356	protein-codi	NZ_CP1252	chromosome	3137436	3138146	-	711	0	10	585	6	378	
QEN71_RS14060	sigma-70 family RNA polymerase sigma factor	QEN71_14060	paras_003357	protein-codi	NZ_CP1252	chromosome	3138311	3138895	+	585	0	9	843	6	610	
QEN71_RS14065	hypothetical protein	QEN71_14065	paras_003358	protein-codi	NZ_CP1252	chromosome	3138908	3139216	+	309	0	2	119	2	119	
QEN71_RS14070	class I SAM-dependent methyltransferase	QEN71_14070	paras_003359	protein-codi	NZ_CP1252	chromosome	3139264	3139980	+	717	0	30	5653	28	5013	
QEN71_RS14075	5S ribosomal RNA	QEN71_14075		rRNA	NZ_CP1252	chromosome	3140075	3140188	-	114	225.0	1	11	0	0	
QEN71_RS14080	23S ribosomal RNA	QEN71_14080		rRNA	NZ_CP1252	chromosome	3140395	3143275	-	2881	5661.0	39	197	32	149	
QEN71_RS14085	tRNA-Ala	QEN71_14085		tRNA	NZ_CP1252	chromosome	3143581	3143656	-	76	150.0	1	2	0	0	
QEN71_RS14090	tRNA-Ile	QEN71_14090		tRNA	NZ_CP1252	chromosome	3143716	3143792	-	77	152.0	1	2	1	2	
QEN71_RS14095	16S ribosomal RNA	QEN71_14095		rRNA	NZ_CP1252	chromosome	3143857	3145387	-	1531	3015.0	11	137	9	131	
QEN71_RS14100	alpha/beta hydrolase	QEN71_14100	paras_003365	protein-codi	NZ_CP1252	chromosome	3145745	3146701	-	957	0	17	2488	15	2309	
QEN71_RS14105	hypothetical protein	QEN71_14105	paras_003366	protein-codi	NZ_CP1252	chromosome	3146931	3147314	-	384	0	2	324	2	324	
QEN71_RS14110	alkaline phosphatase family protein	QEN71_14110	paras_003367	protein-codi	NZ_CP1252	chromosome	3147716	3149551	+	1836	0	14	1151	13	1148	
QEN71_RS14115	cytochrome c peroxidase	QEN71_14115	paras_003368	protein-codi	NZ_CP1252	chromosome	3149690	3151126	+	1437	0	25	1975	19	1445	
QEN71_RS14120	lipoyl synthase	QEN71_14120	paras_003369	protein-codi	NZ_CP1252	chromosome	3151199	3152185	-	987	0	10	846	6	551	
QEN71_RS14125	acetoin dehydrogenase dihydrolipoyllysine-residue	QEN71_14125	paras_003370	protein-codi	NZ_CP1252	chromosome	3152188	3153300	-	1113	0	12	890	9	617	
QEN71_RS14130	alpha-ketoacid dehydrogenase subunit beta	QEN71_14130	paras_003371	protein-codi	NZ_CP1252	chromosome	3153338	3154342	-	1005	0	18	2566	14	1650	
QEN71_RS14135	thiamine pyrophosphate-dependent dehydrogenase	QEN71_14135	paras_003372	protein-codi	NZ_CP1252	chromosome	3154381	3155364	-	984	0	10	1343	8	1104	
QEN71_RS14140	NAD(+)/NADH kinase	QEN71_14140	paras_003373	protein-codi	NZ_CP1252	chromosome	3155441	3156544	-	1104	0	4	933	0	0	
QEN71_RS14145	sigma-54-dependent Fis family transcriptional regul	QEN71_14145	paras_003374	protein-codi	NZ_CP1252	chromosome	3156980	3158923	+	1944	0	37	5875	27	5051	
QEN71_RS14150	YafY family protein	QEN71_14150	paras_003375	protein-codi	NZ_CP1252	chromosome	3158931	3159902	-	972	0	17	4458	16	4295	
QEN71_RS14155	antibiotic biosynthesis monooxygenase	QEN71_14155	paras_003376	protein-codi	NZ_CP1252	chromosome	3159994	3160350	+	353	0	7	2941	1	109	
QEN71_RS14160	amino acid permease	QEN71_14160	paras_003377	protein-codi	NZ_CP1252	chromosome	3160347	3161636	+	1286	0	33	14713	25	11401	
QEN71_RS14165	DUF2917 domain-containing protein	QEN71_14165	paras_003378	protein-codi	NZ_CP1252	chromosome	3161889	3162278	+	390	0	6	244	5	207	
QEN71_RS14170	3-oxoadipyl-CoA thiolase	QEN71_14170	paras_003379	protein-codi	NZ_CP1252	chromosome	3162400	3163602	-	1203	0	15	3809	10	2485	
QEN71_RS14175	IclR family transcriptional regulator	QEN71_14175	paras_003380	protein-codi	NZ_CP1252	chromosome	3163980	3164804	+	825	0	12	1897	9	1189	
QEN71_RS14180	M20 aminoacylase family protein	QEN71_14180	paras_003381	protein-codi	NZ_CP1252	chromosome	3164811	3166001	-	1191	0	13	3570	10	3040	
QEN71_RS14185	phosphonate metabolism protein/1,5-bisphosphokin	QEN71_14185	paras_003382	protein-codi	NZ_CP1252	chromosome	3166102	3166707	-	602	0	23	5268	16	2623	
QEN71_RS14190	DUF1045 domain-containing protein	QEN71_14190	paras_003383	protein-codi	NZ_CP1252	chromosome	3166704	3167447	-	736	0	9	2788	4	1875	
QEN71_RS14195	phosphonate metabolism transcriptional regulator P	QEN71_14195	paras_003384	protein-codi	NZ_CP1252	chromosome	3167444	3168202	-	755	0	17	2851	12	1861	
QEN71_RS14200	phosphonate C-P lyase system protein PhnG	QEN71_14200	paras_003385	protein-codi	NZ_CP1252	chromosome	3168402	3168905	+	503	0	1	3	1	3	TRUE
QEN71_RS14205	phosphonate C-P lyase system protein PhnH	QEN71_14205	paras_003386	protein-codi	NZ_CP1252	chromosome	3168905	3169561	+	655	0	10	1035	5	342	
QEN71_RS14210	carbon-phosphorus lyase complex subunit PhnI	QEN71_14210	paras_003387	protein-codi	NZ_CP1252	chromosome	3169561	3170676	+	1111	0	14	1863	10	1235	
QEN71_RS14215	alpha-D-ribose 1-methylphosphonate 5-phosphate C	QEN71_14215	paras_003388	protein-codi	NZ_CP1252	chromosome	3170673	3171581	+	901	0	12	1090	7	469	
QEN71_RS14220	phosphonate C-P lyase system protein PhnK	QEN71_14220	paras_003389	protein-codi	NZ_CP1252	chromosome	3171578	3172348	+	767	0	8	757	3	161	
QEN71_RS14225	phosphonate C-P lyase system protein PhnL	QEN71_14225	paras_003390	protein-codi	NZ_CP1252	chromosome	3172362	3173117	+	756	0	13	2079	8	1602	
QEN71_RS14230	alpha-D-ribose 1-methylphosphonate 5-triphosphate	QEN71_14230	paras_003391	protein-codi	NZ_CP1252	chromosome	3173155	3174288	+	1134	0	11	1197	8	729	
QEN71_RS14235	ABC transporter substrate-binding protein	QEN71_14235	paras_003392	protein-codi	NZ_CP1252	chromosome	3174638	3175420	+	783	0	18	3310	10	1365	
QEN71_RS14240	succinylglutamate desuccinylase/aspartoacylase fa	QEN71_14240	paras_003393	protein-codi	NZ_CP1252	chromosome	3176241	3177356	+	1116	0	22	4864	13	2451	
QEN71_RS14245	porin	QEN71_14245	paras_003394	protein-codi	NZ_CP1252	chromosome	3177687	3178859	+	1173	0	44	7943	33	6188	
QEN71_RS14250	type II toxin-antitoxin system HicB family antitoxin	QEN71_14250	paras_003395	protein-codi	NZ_CP1252	chromosome	3178984	3179307	-	324	0	11	1160	7	648	
QEN71_RS14255	hypothetical protein	QEN71_14255	paras_003396	protein-codi	NZ_CP1252	chromosome	3179594	3179821	-	228	0	7	1143	7	1143	
QEN71_RS14260	hypothetical protein	QEN71_14260	paras_003397	protein-codi	NZ_CP1252	chromosome	3180109	3180360	+	252	0	3	1369	2	1053	
QEN71_RS14265	threo-3-hydroxy-L-aspartate ammonia-lyase	QEN71_14265	paras_003398	protein-codi	NZ_CP1252	chromosome	3180368	3181339	-	972	0	16	3442	14	3096	
QEN71_RS14270	LysR family transcriptional regulator	QEN71_14270	paras_003399	protein-codi	NZ_CP1252	chromosome	3181470	3182393	-	924	0	6	748	6	748	
QEN71_RS14275	DSD1 family PLP-dependent enzyme	QEN71_14275	paras_003400	protein-codi	NZ_CP1252	chromosome	3182499	3183629	+	1131	0	21	3217	15	2689	
QEN71_RS14280	helix-turn-helix transcriptional regulator	QEN71_14280	paras_003401	protein-codi	NZ_CP1252	chromosome	3183646	3184287	-	642	0	9	1309	7	1096	
QEN71_RS14285	carbonic anhydrase family protein	QEN71_14285	paras_003402	protein-codi	NZ_CP1252	chromosome	3184461	3185207	+	747	0	20	4804	10	1405	
QEN71_RS14290	DUF6232 family protein	QEN71_14290	paras_003403	protein-codi	NZ_CP1252	chromosome	3185304	3185693	+	390	0	0	0	0	0	TRUE
QEN71_RS14295	hypothetical protein	QEN71_14295	paras_003404	protein-codi	NZ_CP1252	chromosome	3185828	3186166	+	339	0	5	1120	3	856	
QEN71_RS14300	hypothetical protein	QEN71_14300	paras_003405	protein-codi	NZ_CP1252	chromosome	3186305	3187372	+	1068	0	14	1508	12	1376	
QEN71_RS14305	glycogen/starch/alpha-glucan phosphorylase	QEN71_14305	paras_003406	protein-codi	NZ_CP1252	chromosome	3187462	3189915	-	2454	0	56	14022	36	6979	
QEN71_RS14310	hypothetical protein	QEN71_14310	paras_003407	protein-codi	NZ_CP1252	chromosome	3190316	3190543	+	228	0	4	1548	2	56	
QEN71_RS14315	MFS transporter	QEN71_14315	paras_003408	protein-codi	NZ_CP1252	chromosome	3190844	3192220	+	1377	0	20	6733	18	6521	
QEN71_RS14320	low-specificity L-threonine aldolase	QEN71_14320	paras_003409	protein-codi	NZ_CP1252	chromosome	3192260	3193264	-	1005	0	22	4887	16	3749	
QEN71_RS14325	EAL domain-containing protein	QEN71_14325	paras_003410	protein-codi	NZ_CP1252	chromosome	3193402	3196149	-	2744	0	60	11505	48	9447	
QEN71_RS14330	transporter substrate-binding domain-containing pro	QEN71_14330	paras_003411	protein-codi	NZ_CP1252	chromosome	3196146	3196931	-	782	0	16	2063	13	1920	

QEN71_RS14335	type II CAAX endopeptidase family protein	QEN71_14335	paras_003412	protein-codi	NZ_CP1252	chromosom	3197175	3198992	-	1818	0	22	2334	18	1709	
QEN71_RS14340	MFS transporter	QEN71_14340	paras_003413	protein-codi	NZ_CP1252	chromosom	3199278	3200636	-	1359	0	14	2470	14	2470	
QEN71_RS14345	hypothetical protein	QEN71_14345	paras_003414	protein-codi	NZ_CP1252	chromosom	3200892	3201086	-	195	0	1	32	1	32	
QEN71_RS14350	oxalate/formate MFS antiporter	QEN71_14350	paras_003415	protein-codi	NZ_CP1252	chromosom	3201513	3202853	+	1341	0	27	5623	19	5085	
QEN71_RS14355	MoxR family ATPase	QEN71_14355	paras_003416	protein-codi	NZ_CP1252	chromosom	3203179	3204198	+	1016	0	23	4358	19	3841	
QEN71_RS14360	MxaS protein	QEN71_14360	paras_003417	protein-codi	NZ_CP1252	chromosom	3204195	3205070	+	868	0	5	151	5	151	
QEN71_RS14365	hypothetical protein	QEN71_14365	paras_003418	protein-codi	NZ_CP1252	chromosom	3205067	3205942	+	868	0	6	330	3	184	
QEN71_RS14370	vWA domain-containing protein	QEN71_14370	paras_003419	protein-codi	NZ_CP1252	chromosom	3205939	3206937	+	991	0	13	669	10	622	
QEN71_RS14375	MxaK protein	QEN71_14375	paras_003420	protein-codi	NZ_CP1252	chromosom	3206934	3207494	+	553	0	15	1091	15	1091	
QEN71_RS14380	vWA domain-containing protein	QEN71_14380	paras_003421	protein-codi	NZ_CP1252	chromosom	3207491	3208453	+	959	0	16	2303	11	1172	
QEN71_RS14385	hypothetical protein	QEN71_14385	paras_003422	protein-codi	NZ_CP1252	chromosom	3208543	3208866	+	324	0	4	339	1	119	
QEN71_RS14390	tetratricopeptide repeat protein	QEN71_14390	paras_003423	protein-codi	NZ_CP1252	chromosom	3208992	3210710	+	1719	0	32	6801	30	6712	
QEN71_RS14395	amine dehydrogenase large subunit	QEN71_14395	paras_003424	protein-codi	NZ_CP1252	chromosom	3210930	3212087	+	1158	0	32	6311	26	5348	
QEN71_RS14400	MauE/DoxX family redox-associated membrane pro	QEN71_14400	paras_003425	protein-codi	NZ_CP1252	chromosom	3212095	3212661	+	563	0	3	290	2	279	
QEN71_RS14405	methylamine dehydrogenase accessory protein Mau	QEN71_14405	paras_003426	protein-codi	NZ_CP1252	chromosom	3212658	3213284	+	623	0	6	267	6	267	
QEN71_RS14410	methylamine dehydrogenase light chain	QEN71_14410	paras_003427	protein-codi	NZ_CP1252	chromosom	3213325	3213870	+	542	0	10	1627	8	1207	
QEN71_RS14415	cytochrome c	QEN71_14415	paras_003428	protein-codi	NZ_CP1252	chromosom	3213867	3214364	+	490	0	8	1540	7	1376	
QEN71_RS14420	cytochrome c	QEN71_14420	paras_003429	protein-codi	NZ_CP1252	chromosom	3214361	3214924	+	560	0	6	734	6	734	
QEN71_RS14425	aldehyde dehydrogenase family protein	QEN71_14425	paras_003430	protein-codi	NZ_CP1252	chromosom	3214948	3216453	+	1506	0	19	1739	15	1507	
QEN71_RS14430	porin	QEN71_14430	paras_003431	protein-codi	NZ_CP1252	chromosom	3216552	3217781	+	1230	0	42	3235	38	2944	
QEN71_RS14435	hypothetical protein	QEN71_14435	paras_003432	protein-codi	NZ_CP1252	chromosom	3217857	3218159	-	303	0	8	1021	5	632	
QEN71_RS14440	EAL domain-containing protein	QEN71_14440	paras_003433	protein-codi	NZ_CP1252	chromosom	3218233	3218424	-	192	0	3	408	3	408	
QEN71_RS14445	2-aminoethylphosphonate aminotransferase	QEN71_14445	paras_003434	protein-codi	NZ_CP1252	chromosom	3219314	3220381	-	1067	0	28	3662	21	3089	
QEN71_RS14450	phosphonopyruvate decarboxylase	QEN71_14450	paras_003435	protein-codi	NZ_CP1252	chromosom	3220381	3221565	-	1180	0	24	5526	19	5081	
QEN71_RS14455	phosphoenolpyruvate mutase	QEN71_14455	paras_003436	protein-codi	NZ_CP1252	chromosom	3221562	3223271	-	1706	0	19	4527	17	3701	
QEN71_RS14460	methyl-accepting chemotaxis protein	QEN71_14460	paras_003437	protein-codi	NZ_CP1252	chromosom	3224223	3225764	+	1542	0	20	3898	20	3898	
QEN71_RS14465	penicillin-binding protein 2	QEN71_14465	paras_003438	protein-codi	NZ_CP1252	chromosom	3226033	3227781	+	1749	0	23	1642	18	1302	
QEN71_RS14470	tetratricopeptide repeat protein	QEN71_14470	paras_003439	protein-codi	NZ_CP1252	chromosom	3227806	3229419	-	1614	0	25	3375	15	2725	
QEN71_RS14475	hemagglutinin	QEN71_14475	paras_003440	protein-codi	NZ_CP1252	chromosom	3229757	3231541	+	1785	0	58	8663	50	7513	
QEN71_RS14480	hypothetical protein	QEN71_14480	paras_003441	protein-codi	NZ_CP1252	chromosom	3231595	3231924	+	330	0	1	212	1	212	
QEN71_RS14485	DUF2866 domain-containing protein	QEN71_14485	paras_003442	protein-codi	NZ_CP1252	chromosom	3232252	3232503	+	252	0	3	202	2	198	
QEN71_RS14490	hypothetical protein	QEN71_14490	paras_003443	protein-codi	NZ_CP1252	chromosom	3232576	3232818	-	241	0	1	8	1	8	
QEN71_RS14495	hypothetical protein	QEN71_14495	paras_003444	protein-codi	NZ_CP1252	chromosom	3232817	3233002	+	170	177.0	1	17	1	17	
QEN71_RS14500	lecithin retinol acyltransferase family protein	QEN71_14500	paras_003445	protein-codi	NZ_CP1252	chromosom	3232989	3233519	-	517	0	10	1407	10	1407	
QEN71_RS14505	hypothetical protein	QEN71_14505	paras_003446	protein-codi	NZ_CP1252	chromosom	3233970	3234254	+	285	0	15	2316	10	1291	
QEN71_RS14510	protein phosphatase 2C domain-containing protein	QEN71_14510	paras_003447	protein-codi	NZ_CP1252	chromosom	3234260	3235030	+	771	0	10	518	9	456	
QEN71_RS14515	sigma-54 dependent transcriptional regulator	QEN71_14515	paras_003448	protein-codi	NZ_CP1252	chromosom	3235037	3236395	-	1355	0	14	1287	10	1131	
QEN71_RS14520	peptidase domain-containing ABC transporter	QEN71_14520	paras_003449	protein-codi	NZ_CP1252	chromosom	3236392	3239448	-	3053	0	37	1956	27	1256	
QEN71_RS14525	peptidylprolyl isomerase	QEN71_14525	paras_003450	protein-codi	NZ_CP1252	chromosom	3239483	3240235	-	753	0	6	637	4	228	
QEN71_RS14530	HlyD family efflux transporter periplasmic adaptor su	QEN71_14530	paras_003451	protein-codi	NZ_CP1252	chromosom	3240334	3241707	-	1351	0	20	1699	19	1696	
QEN71_RS14535	FHA domain-containing protein	QEN71_14535	paras_003452	protein-codi	NZ_CP1252	chromosom	3241685	3244213	-	2506	0	22	2033	18	1634	
QEN71_RS14540	efflux transporter outer membrane subunit	QEN71_14540	paras_003453	protein-codi	NZ_CP1252	chromosom	3244420	3245817	-	1398	0	18	1012	13	942	
QEN71_RS14545	hypothetical protein	QEN71_14545	paras_003454	protein-codi	NZ_CP1252	chromosom	3246185	3246454	-	270	0	6	217	4	170	
QEN71_RS14550	DUF4399 domain-containing protein	QEN71_14550	paras_003455	protein-codi	NZ_CP1252	chromosom	3246785	3247213	-	429	0	6	570	6	570	
QEN71_RS14555	DUF4399 domain-containing protein	QEN71_14555	paras_003456	protein-codi	NZ_CP1252	chromosom	3247276	3247701	-	426	0	6	316	5	298	
QEN71_RS14560	SUMF1/EgtB/PvdO family nonheme iron enzyme	QEN71_14560	paras_003457	protein-codi	NZ_CP1252	chromosom	3247965	3249878	+	1914	0	36	2863	26	2384	
QEN71_RS14565	serine/threonine protein kinase	QEN71_14565	paras_003458	protein-codi	NZ_CP1252	chromosom	3249910	3252027	-	2118	0	40	3748	35	3375	
QEN71_RS14570	hypothetical protein	QEN71_14570	paras_003459	protein-codi	NZ_CP1252	chromosom	3252424	3252693	+	270	0	2	336	1	301	
QEN71_RS14575	hypothetical protein	QEN71_14575	paras_003460	protein-codi	NZ_CP1252	chromosom	3252785	3253069	+	285	0	2	239	1	52	
QEN71_RS14580	hypothetical protein	QEN71_14580	paras_003461	protein-codi	NZ_CP1252	chromosom	3253247	3253471	+	225	0	3	212	1	8	
QEN71_RS14585	hypothetical protein	QEN71_14585	paras_003462	protein-codi	NZ_CP1252	chromosom	3253823	3254020	+	198	0	4	325	4	325	
QEN71_RS14590	hypothetical protein	QEN71_14590	paras_003463	protein-codi	NZ_CP1252	chromosom	3254189	3254980	+	792	0	10	300	8	190	
QEN71_RS14595	hypothetical protein	QEN71_14595	paras_003464	protein-codi	NZ_CP1252	chromosom	3254991	3255455	+	465	0	15	2019	10	1310	
QEN71_RS14600	YigZ family protein	QEN71_14600	paras_003465	protein-codi	NZ_CP1252	chromosom	3255716	3256309	+	594	0	7	491	6	438	
QEN71_RS14605	GMC family oxidoreductase N-terminal domain-cont	QEN71_14605	paras_003466	protein-codi	NZ_CP1252	chromosom	3256392	3258059	-	1668	0	44	3340	35	2431	
QEN71_RS14610	mandelate racemase/muconate lactonizing enzyme	QEN71_14610	paras_003467	protein-codi	NZ_CP1252	chromosom	3258115	3259332	-	1218	0	33	2189	22	1332	
QEN71_RS14615	MFS transporter	QEN71_14615	paras_003468	protein-codi	NZ_CP1252	chromosom	3259333	3260652	-	1320	0	32	4152	16	2295	
QEN71_RS14620	aldehyde dehydrogenase	QEN71_14620	paras_003469	protein-codi	NZ_CP1252	chromosom	3260803	3262227	-	1425	0	35	5614	21	3188	
QEN71_RS14625	hypothetical protein	QEN71_14625	paras_003470	protein-codi	NZ_CP1252	chromosom	3262368	3262604	+	237	0	5	889	3	834	
QEN71_RS14630	H-NS histone family protein	QEN71_14630	paras_003471	protein-codi	NZ_CP1252	chromosom	3262610	3263326	-	717	0	14	1554	9	642	
QEN71_RS14635	bifunctional DedA family/phosphatase PAP2 family	QEN71_14635	paras_003472	protein-codi	NZ_CP1252	chromosom	3263679	3265694	+	2016	0	39	7055	32	6178	
QEN71_RS14640	VTT domain-containing protein	QEN71_14640	paras_003473	protein-codi	NZ_CP1252	chromosom	3265805	3266848	+	1044	0	23	3065	18	2459	

QEN71_RS14645	aspartyl/asparaginyl beta-hydroxylase domain-conta	partial;pseudo;QEN71_14645		pseudogene	NZ_CP1252	chromosom	3267022	3267462	+	441	0	28	5284	23	4900	
QEN71_RS14650	diguanylate cyclase	QEN71_14650	paras_003475	protein-codi	NZ_CP1252	chromosom	3267499	3268578	-	1080	0	8	326	5	101	
QEN71_RS14655	LysR substrate-binding domain-containing protein	QEN71_14655	paras_003476	protein-codi	NZ_CP1252	chromosom	3268920	3269861	-	942	0	14	1540	14	1540	
QEN71_RS14660	inorganic phosphate transporter	QEN71_14660	paras_003477	protein-codi	NZ_CP1252	chromosom	3270105	3271694	+	1590	0	35	5815	21	3839	
QEN71_RS14665	polyphosphate kinase 2	QEN71_14665	paras_003478	protein-codi	NZ_CP1252	chromosom	3271814	3272743	-	930	0	19	2357	15	1918	
QEN71_RS14670	VOC family protein	QEN71_14670	paras_003479	protein-codi	NZ_CP1252	chromosom	3272872	3273258	-	387	0	8	810	8	810	
QEN71_RS14675	gamma-glutamyltransferase	QEN71_14675	paras_003480	protein-codi	NZ_CP1252	chromosom	3273376	3275106	-	1731	0	51	6316	40	5014	
QEN71_RS14680	aspartate aminotransferase family protein	QEN71_14680	paras_003481	protein-codi	NZ_CP1252	chromosom	3275258	3276655	-	1398	0	22	4420	22	4420	
QEN71_RS14685	NAD-dependent succinate-semialdehyde dehydroge	QEN71_14685	paras_003482	protein-codi	NZ_CP1252	chromosom	3276692	3278197	-	1506	0	17	2376	9	1291	
QEN71_RS14690	Lrp/AsnC family transcriptional regulator	QEN71_14690	paras_003483	protein-codi	NZ_CP1252	chromosom	3278912	3279382	-	471	0	13	528	13	528	
QEN71_RS14695	N(2)-acetyl-L-2,4-diaminobutanoate deacetylase Do	QEN71_14695	paras_003484	protein-codi	NZ_CP1252	chromosom	3279386	3280426	-	1041	0	21	2685	18	2357	
QEN71_RS14700	ectoine hydrolase DoeA	QEN71_14700	paras_003485	protein-codi	NZ_CP1252	chromosom	3280431	3281639	-	1209	0	26	2099	22	1779	
QEN71_RS14705	cyclodeaminase	QEN71_14705	paras_003486	protein-codi	NZ_CP1252	chromosom	3281649	3282650	-	998	0	7	357	6	342	
QEN71_RS14710	hydroxyectoine utilization dehydratase EutB	QEN71_14710	paras_003487	protein-codi	NZ_CP1252	chromosom	3282647	3283639	-	989	0	13	1607	10	1353	
QEN71_RS14715	PLP-dependent aminotransferase family protein	QEN71_14715	paras_003488	protein-codi	NZ_CP1252	chromosom	3283795	3285216	-	1422	0	29	4519	23	3895	
QEN71_RS14720	ectoine/hydroxyectoine ABC transporter substrate-b	QEN71_14720	paras_003489	protein-codi	NZ_CP1252	chromosom	3285430	3286284	+	855	0	14	1755	14	1755	
QEN71_RS14725	ectoine/hydroxyectoine ABC transporter permease s	QEN71_14725	paras_003490	protein-codi	NZ_CP1252	chromosom	3286481	3287152	+	668	0	8	932	7	596	
QEN71_RS14730	ectoine/hydroxyectoine ABC transporter permease s	QEN71_14730	paras_003491	protein-codi	NZ_CP1252	chromosom	3287149	3287808	+	656	0	18	3613	17	3509	
QEN71_RS14735	ectoine/hydroxyectoine ABC transporter ATP-bindin	QEN71_14735	paras_003492	protein-codi	NZ_CP1252	chromosom	3287868	3288692	+	825	0	8	1119	7	1058	
QEN71_RS14740	MFS transporter	QEN71_14740	paras_003493	protein-codi	NZ_CP1252	chromosom	3288737	3290140	-	1404	0	21	3542	14	2669	
QEN71_RS14745	phosphocholine cytidylyltransferase family protein	QEN71_14745	paras_003494	protein-codi	NZ_CP1252	chromosom	3290741	3291505	-	765	0	10	2346	9	2323	
QEN71_RS14750	flippase-like domain-containing protein	QEN71_14750	paras_003495	protein-codi	NZ_CP1252	chromosom	3291527	3292531	-	1001	0	13	5163	8	2915	
QEN71_RS14755	2OG-Fe(II) oxygenase	QEN71_14755	paras_003496	protein-codi	NZ_CP1252	chromosom	3292528	3293397	-	862	0	35	12977	33	12188	
QEN71_RS14760	CDP-alcohol phosphatidyltransferase family protein	QEN71_14760	paras_003497	protein-codi	NZ_CP1252	chromosom	3293394	3294083	-	686	0	10	7308	10	7308	
QEN71_RS14765	hypothetical protein	QEN71_14765	paras_003498	protein-codi	NZ_CP1252	chromosom	3294344	3294781	+	438	0	11	4284	7	1228	
QEN71_RS14770	HIT family protein	QEN71_14770	paras_003499	protein-codi	NZ_CP1252	chromosom	3295243	3295671	+	429	0	8	3238	6	2716	
QEN71_RS14775	hypothetical protein	QEN71_14775	paras_003500	protein-codi	NZ_CP1252	chromosom	3295696	3296199	-	504	0	6	1583	6	1583	
QEN71_RS14780	porin	QEN71_14780	paras_003501	protein-codi	NZ_CP1252	chromosom	3296889	3298043	+	1155	0	37	4478	31	4210	
QEN71_RS14785	hypothetical protein	QEN71_14785	paras_003502	protein-codi	NZ_CP1252	chromosom	3298318	3298737	+	420	0	16	2089	9	828	
QEN71_RS14790	sugar ABC transporter ATPase	QEN71_14790	paras_003503	protein-codi	NZ_CP1252	chromosom	3298955	3299293	+	339	0	4	1482	4	1482	
QEN71_RS14795	metal-dependent hydrolase	QEN71_14795	paras_003504	protein-codi	NZ_CP1252	chromosom	3299424	3299933	+	510	0	6	2291	2	1614	
QEN71_RS14800	amino acid carrier protein	QEN71_14800	paras_003505	protein-codi	NZ_CP1252	chromosom	3300097	3301686	+	1590	0	23	3448	21	3336	
QEN71_RS14805	FAD-binding oxidoreductase	QEN71_14805	paras_003506	protein-codi	NZ_CP1252	chromosom	3301697	3302869	-	1173	0	24	4409	19	3989	
QEN71_RS14810	aldehyde dehydrogenase family protein	QEN71_14810	paras_003507	protein-codi	NZ_CP1252	chromosom	3302998	3304497	-	1500	0	28	6219	22	5092	
QEN71_RS14815	XRE family transcriptional regulator	QEN71_14815	paras_003508	protein-codi	NZ_CP1252	chromosom	3304821	3305450	+	630	0	19	3705	12	1192	
QEN71_RS14820	VOC family protein	QEN71_14820	paras_003509	protein-codi	NZ_CP1252	chromosom	3305469	3306596	-	1128	0	21	5672	20	5522	
QEN71_RS14825	pca operon transcription factor PcaQ	QEN71_14825	paras_003510	protein-codi	NZ_CP1252	chromosom	3306727	3307791	-	1065	0	13	2493	9	1110	
QEN71_RS14830	protocatechuate 3,4-dioxygenase subunit beta	QEN71_14830	paras_003511	protein-codi	NZ_CP1252	chromosom	3307900	3308604	+	705	0	18	5058	12	3999	
QEN71_RS14835	protocatechuate 3,4-dioxygenase subunit alpha	QEN71_14835	paras_003512	protein-codi	NZ_CP1252	chromosom	3308607	3309197	+	591	0	16	2485	8	1734	
QEN71_RS14840	class II aldolase/adducin family protein	QEN71_14840	paras_003513	protein-codi	NZ_CP1252	chromosom	3309390	3310160	-	771	0	22	7020	16	5784	
QEN71_RS14845	H-NS family nucleoid-associated regulatory protein	QEN71_14845	paras_003514	protein-codi	NZ_CP1252	chromosom	3310260	3310514	-	255	0	6	561	5	546	
QEN71_RS14850	acyl-CoA synthetase	QEN71_14850	paras_003515	protein-codi	NZ_CP1252	chromosom	3310852	3312504	-	1653	0	66	21308	50	16593	
QEN71_RS14855	hypothetical protein	QEN71_14855	paras_003516	protein-codi	NZ_CP1252	chromosom	3312767	3313075	-	309	0	12	6272	7	387	
QEN71_RS14860	DUF72 domain-containing protein	QEN71_14860	paras_003517	protein-codi	NZ_CP1252	chromosom	3313447	3314343	+	897	0	25	4023	19	3272	
QEN71_RS14865	hypothetical protein	QEN71_14865	paras_003518	protein-codi	NZ_CP1252	chromosom	3314494	3315957	+	1464	0	39	5077	26	3362	
QEN71_RS14870	hypothetical protein	QEN71_14870	paras_003519	protein-codi	NZ_CP1252	chromosom	3315987	3316796	+	810	0	20	1987	19	1930	
QEN71_RS14875	transglycosylase domain-containing protein	QEN71_14875	paras_003520	protein-codi	NZ_CP1252	chromosom	3316805	3319198	-	2394	0	51	6628	46	5901	
QEN71_RS14880	sodium:solute symporter	QEN71_14880	paras_003521	protein-codi	NZ_CP1252	chromosom	3319433	3320908	-	1472	0	48	9136	43	8665	
QEN71_RS14885	DUF3311 domain-containing protein	QEN71_14885	paras_003522	protein-codi	NZ_CP1252	chromosom	3320905	3321102	-	194	0	13	1934	9	1707	
QEN71_RS14890	LysR family transcriptional regulator	QEN71_14890	paras_003523	protein-codi	NZ_CP1252	chromosom	3321275	3322165	-	891	0	19	3545	14	2942	
QEN71_RS14895	pirin family protein	QEN71_14895	paras_003524	protein-codi	NZ_CP1252	chromosom	3322324	3323049	+	726	0	13	2887	11	2552	
QEN71_RS14900	DoxX family protein	QEN71_14900	paras_003525	protein-codi	NZ_CP1252	chromosom	3323108	3323518	+	411	0	17	3137	11	2580	
QEN71_RS14905	porin	QEN71_14905	paras_003526	protein-codi	NZ_CP1252	chromosom	3323761	3324897	+	1137	0	46	11215	38	8609	
QEN71_RS14910	hypothetical protein	QEN71_14910		protein-codi	NZ_CP1252	chromosom	3325050	3325184	+	135	0	3	362	2	341	
QEN71_RS14915	fatty acid desaturase	QEN71_14915	paras_003527	protein-codi	NZ_CP1252	chromosom	3325356	3326390	+	1035	0	21	5936	17	5215	
QEN71_RS14920	non-heme iron oxygenase ferredoxin subunit	QEN71_14920	paras_003528	protein-codi	NZ_CP1252	chromosom	3326422	3326769	+	344	0	8	3575	8	3575	
QEN71_RS14925	FAD-dependent oxidoreductase	QEN71_14925	paras_003529	protein-codi	NZ_CP1252	chromosom	3326766	3328013	+	1240	0	16	2599	15	2300	
QEN71_RS14930	sugar phosphate isomerase/epimerase	QEN71_14930	paras_003530	protein-codi	NZ_CP1252	chromosom	3328010	3328876	+	852	0	15	2469	11	1619	
QEN71_RS14935	LacI family DNA-binding transcriptional regulator	QEN71_14935	paras_003531	protein-codi	NZ_CP1252	chromosom	3328866	3329903	-	1027	0	16	2635	13	2500	
QEN71_RS14940	SDR family oxidoreductase	QEN71_14940	paras_003532	protein-codi	NZ_CP1252	chromosom	3330014	3330841	+	828	0	14	3180	12	2624	
QEN71_RS14945	LysR family transcriptional regulator	QEN71_14945	paras_003533	protein-codi	NZ_CP1252	chromosom	3330889	3331803	-	915	0	19	3107	17	2947	
QEN71_RS14950	hypothetical protein	QEN71_14950	paras_003534	protein-codi	NZ_CP1252	chromosom	3332560	3332769	+	210	0	5	608	4	516	

QEN71_RS14955	phasin family protein	QEN71_14955	paras_003535	protein-codi	NZ_CP1252	chromosom	3332912	3333430	+	519	0	11	2022	8	1919	
QEN71_RS14960	hypothetical protein	QEN71_14960	paras_003536	protein-codi	NZ_CP1252	chromosom	3333550	3333744	-	195	0	0	0	0	0	
QEN71_RS14965	hypothetical protein	QEN71_14965	paras_003537	protein-codi	NZ_CP1252	chromosom	3333923	3334990	+	1068	0	33	7569	27	7084	
QEN71_RS14970	DUF4148 domain-containing protein	QEN71_14970	paras_003538	protein-codi	NZ_CP1252	chromosom	3335075	3335413	-	339	0	12	2604	8	1293	
QEN71_RS14975	TetR/AcrR family transcriptional regulator	QEN71_14975	paras_003539	protein-codi	NZ_CP1252	chromosom	3335507	3336190	-	684	0	17	2674	12	1650	
QEN71_RS14980	glutathione binding-like protein	QEN71_14980	paras_003540	protein-codi	NZ_CP1252	chromosom	3336262	3336885	+	624	0	17	2144	14	1613	
QEN71_RS14985	VOC family protein	QEN71_14985	paras_003541	protein-codi	NZ_CP1252	chromosom	3336907	3337257	+	351	0	5	810	5	810	
QEN71_RS14990	acetoacetate decarboxylase	QEN71_14990	paras_003542	protein-codi	NZ_CP1252	chromosom	3337357	3338112	+	756	0	22	3331	19	3146	
QEN71_RS14995	2-dehydropantoate 2-reductase	QEN71_14995	paras_003543	protein-codi	NZ_CP1252	chromosom	3338170	3339057	-	888	0	15	1477	7	702	
QEN71_RS15000	alpha/beta hydrolase	QEN71_15000	paras_003544	protein-codi	NZ_CP1252	chromosom	3339096	3339992	-	897	0	20	2452	17	2277	
QEN71_RS15005	MFS transporter	QEN71_15005	paras_003545	protein-codi	NZ_CP1252	chromosom	3340289	3341626	+	1338	0	32	3377	29	3313	
QEN71_RS15010	glycosyltransferase family 4 protein	QEN71_15010	paras_003546	protein-codi	NZ_CP1252	chromosom	3341634	3342704	-	1071	0	19	1362	13	938	
QEN71_RS15015	glycosyltransferase family 9 protein	QEN71_15015	paras_003547	protein-codi	NZ_CP1252	chromosom	3342779	3343696	-	918	0	7	459	4	282	
QEN71_RS15020	glycosyltransferase family 9 protein	QEN71_15020	paras_003548	protein-codi	NZ_CP1252	chromosom	3343698	3344600	-	903	0	6	139	4	119	
QEN71_RS15025	hypothetical protein	QEN71_15025	paras_003549	protein-codi	NZ_CP1252	chromosom	3344778	3345629	-	852	0	10	827	7	580	
QEN71_RS15030	LPS-assembly protein LptD	QEN71_15030	paras_003550	protein-codi	NZ_CP1252	chromosom	3345752	3348007	-	2256	0	42	4399	35	3637	
QEN71_RS15035	hypothetical protein	QEN71_15035	paras_003551	protein-codi	NZ_CP1252	chromosom	3348079	3348387	-	301	0	6	481	6	481	
QEN71_RS15040	hypothetical protein	QEN71_15040	paras_003552	protein-codi	NZ_CP1252	chromosom	3348380	3348559	-	172	0	2	88	0	0	
QEN71_RS15045	outer membrane protein assembly factor BamA	QEN71_15045	paras_003553	protein-codi	NZ_CP1252	chromosom	3348869	3351178	+	2310	0	48	5506	38	3707	
QEN71_RS15050	response regulator transcription factor	QEN71_15050	paras_003554	protein-codi	NZ_CP1252	chromosom	3351254	3352078	-	825	0	17	2499	16	2479	
QEN71_RS15055	ATP-binding protein	QEN71_15055	paras_003555	protein-codi	NZ_CP1252	chromosom	3352362	3353906	+	1537	0	23	2887	15	2342	
QEN71_RS15060	response regulator	QEN71_15060	paras_003556	protein-codi	NZ_CP1252	chromosom	3353899	3354663	+	757	0	13	1906	11	1844	
QEN71_RS15065	H-NS family nucleoid-associated regulatory protein	QEN71_15065	paras_003557	protein-codi	NZ_CP1252	chromosom	3354680	3354937	-	258	0	6	856	3	425	
QEN71_RS15070	hypothetical protein	QEN71_15070	paras_003558	protein-codi	NZ_CP1252	chromosom	3355007	3355237	-	231	0	1	634	0	0	
QEN71_RS15075	hypothetical protein	QEN71_15075	paras_003559	protein-codi	NZ_CP1252	chromosom	3355455	3356276	-	822	0	11	1815	9	1515	
QEN71_RS15080	helix-turn-helix transcriptional regulator	QEN71_15080	paras_003560	protein-codi	NZ_CP1252	chromosom	3356377	3356718	+	342	0	10	1563	9	1515	
QEN71_RS15085	acyl-CoA dehydrogenase	QEN71_15085	paras_003561	protein-codi	NZ_CP1252	chromosom	3357262	3359052	-	1791	386.0	38	6460	35	5765	
QEN71_RS15090	AraC family transcriptional regulator	QEN71_15090	paras_003562	protein-codi	NZ_CP1252	chromosom	3359233	3360246	-	1014	0	16	2981	12	2565	
QEN71_RS15095	GntR family transcriptional regulator	QEN71_15095	paras_003563	protein-codi	NZ_CP1252	chromosom	3360274	3360999	-	726	0	11	2088	7	1752	
QEN71_RS15100	cation:dicarboxylase symporter family transporter	QEN71_15100	paras_003564	protein-codi	NZ_CP1252	chromosom	3361232	3362575	+	1344	0	22	2984	18	2433	
QEN71_RS15105	mandelate racemase/muconate lactonizing enzyme	QEN71_15105	paras_003565	protein-codi	NZ_CP1252	chromosom	3362601	3363767	+	1167	0	32	3693	24	2717	
QEN71_RS15110	3-carboxy-cis,cis-muconate cycloisomerase	QEN71_15110	paras_003566	protein-codi	NZ_CP1252	chromosom	3363820	3365190	+	1371	0	27	4161	18	3244	
QEN71_RS15115	alkaline phosphatase family protein	QEN71_15115	paras_003567	protein-codi	NZ_CP1252	chromosom	3365464	3367146	+	1683	0	58	8500	51	7459	
QEN71_RS15120	sugar transporter	QEN71_15120	paras_003568	protein-codi	NZ_CP1252	chromosom	3367375	3368556	+	1182	0	35	8490	28	7281	
QEN71_RS15125	carboxymuconolactone decarboxylase family protei	QEN71_15125	paras_003569	protein-codi	NZ_CP1252	chromosom	3368636	3368941	-	306	0	7	651	6	623	
QEN71_RS15130	AraC family transcriptional regulator	QEN71_15130	paras_003570	protein-codi	NZ_CP1252	chromosom	3369081	3370019	+	939	0	15	2615	11	2360	
QEN71_RS15135	sigma-70 family RNA polymerase sigma factor	QEN71_15135	paras_003571	protein-codi	NZ_CP1252	chromosom	3370299	3370811	+	513	0	8	2769	4	387	
QEN71_RS15140	FecR domain-containing protein	QEN71_15140	paras_003572	protein-codi	NZ_CP1252	chromosom	3370816	3371802	+	987	0	16	1639	8	1071	
QEN71_RS15145	TonB-dependent receptor	QEN71_15145	paras_003573	protein-codi	NZ_CP1252	chromosom	3371915	3374353	+	2439	0	73	7334	62	6865	
QEN71_RS15150	hypothetical protein	QEN71_15150	paras_003574	protein-codi	NZ_CP1252	chromosom	3374425	3375075	+	651	0	12	872	10	684	
QEN71_RS15155	DUF4148 domain-containing protein	QEN71_15155	paras_003575	protein-codi	NZ_CP1252	chromosom	3375128	3375424	-	297	0	2	29	1	26	
QEN71_RS15160	hypothetical protein	QEN71_15160	paras_003576	protein-codi	NZ_CP1252	chromosom	3375835	3376605	+	771	0	17	1724	16	1599	
QEN71_RS15165	hypothetical protein	QEN71_15165	paras_003577	protein-codi	NZ_CP1252	chromosom	3376651	3377082	+	432	0	1	15	0	0	TRUE
QEN71_RS15170	response regulator	QEN71_15170	paras_003578	protein-codi	NZ_CP1252	chromosom	3377242	3377904	+	659	0	16	2473	15	2464	
QEN71_RS15175	ATP-binding protein	QEN71_15175	paras_003579	protein-codi	NZ_CP1252	chromosom	3377901	3379241	+	1337	0	40	5527	30	4831	
QEN71_RS15180	tRNA-Ile	QEN71_15180		tRNA	NZ_CP1252	chromosom	3379300	3379387	-	88	0	0	0	0	0	
QEN71_RS15185	RNA polymerase sigma factor RpoD	QEN71_15185	paras_003581	protein-codi	NZ_CP1252	chromosom	3379580	3381625	-	2046	0	39	19339	36	18308	
QEN71_RS15190	hypothetical protein	QEN71_15190		protein-codi	NZ_CP1252	chromosom	3381687	3382031	+	345	0	13	2305	8	1549	
QEN71_RS15195	DNA primase	QEN71_15195	paras_003582	protein-codi	NZ_CP1252	chromosom	3382063	3383943	-	1881	0	1	8	1	8	TRUE
QEN71_RS15200	GatB/YqeY domain-containing protein	QEN71_15200	paras_003583	protein-codi	NZ_CP1252	chromosom	3384047	3384493	-	447	0	5	1666	4	1490	
QEN71_RS15205	30S ribosomal protein S21	QEN71_15205	paras_003584	protein-codi	NZ_CP1252	chromosom	3384731	3384943	-	213	0	4	445	1	161	
QEN71_RS15210	NAD(P)/FAD-dependent oxidoreductase	QEN71_15210	paras_003585	protein-codi	NZ_CP1252	chromosom	3385053	3386270	-	1218	0	22	6416	17	5112	
QEN71_RS15215	tRNA (adenosine(37)-N6)-threonylcarbamoyltransfe	QEN71_15215	paras_003586	protein-codi	NZ_CP1252	chromosom	3386385	3387410	+	1026	0	0	0	0	0	TRUE
QEN71_RS15220	hypothetical protein	QEN71_15220	paras_003587	protein-codi	NZ_CP1252	chromosom	3387727	3387885	+	159	0	6	1795	3	1075	
QEN71_RS15225	GTP cyclohydrolase FolE2	QEN71_15225	paras_003588	protein-codi	NZ_CP1252	chromosom	3388091	3388897	-	807	0	23	2585	13	1830	
QEN71_RS15230	1-deoxy-D-xylulose-5-phosphate synthase	QEN71_15230	paras_003589	protein-codi	NZ_CP1252	chromosom	3389047	3390945	-	1899	0	4	194	2	11	TRUE
QEN71_RS15235	polyprenyl synthetase family protein	QEN71_15235	paras_003590	protein-codi	NZ_CP1252	chromosom	3391039	3391932	-	890	0	7	249	6	167	
QEN71_RS15240	exodeoxyribonuclease VII small subunit	QEN71_15240	paras_003591	protein-codi	NZ_CP1252	chromosom	3391929	3392246	-	314	0	5	2816	4	1730	
QEN71_RS15245	aromatic ring-hydroxylating dioxygenase subunit alp	QEN71_15245	paras_003592	protein-codi	NZ_CP1252	chromosom	3392533	3393639	+	1107	0	48	17120	42	16296	
QEN71_RS15250	IS4 family transposase	pseudo;QEN71_15250		pseudogene	NZ_CP1252	chromosom	3393696	3394965	-	1270	1320.0	9	536	7	104	
QEN71_RS15255	sulfurtransferase	QEN71_15255	paras_003594	protein-codi	NZ_CP1252	chromosom	3395133	3396005	+	873	0	21	7099	14	5060	
QEN71_RS15260	amino acid dehydrogenase	QEN71_15260	paras_003595	protein-codi	NZ_CP1252	chromosom	3396085	3397158	-	1074	0	30	7018	30	7018	

QEN71_RS15265	Lrp/AsnC family transcriptional regulator	QEN71_15265	paras_003596	protein-codi	NZ_CP1252	chromosom	3397418	3397876	+	459	0	10	1603	10	1603	
QEN71_RS15270	EAL domain-containing protein	QEN71_15270	paras_003597	protein-codi	NZ_CP1252	chromosom	3397883	3399820	-	1938	0	32	6638	24	4396	
QEN71_RS15275	TrkA C-terminal domain-containing protein	QEN71_15275	paras_003598	protein-codi	NZ_CP1252	chromosom	3400089	3401681	-	1593	0	20	3514	16	3016	
QEN71_RS15280	sensor histidine kinase	QEN71_15280	paras_003599	protein-codi	NZ_CP1252	chromosom	3401840	3403240	-	1397	0	12	1029	10	967	
QEN71_RS15285	response regulator	QEN71_15285	paras_003600	protein-codi	NZ_CP1252	chromosom	3403237	3403914	-	674	0	4	739	4	739	
QEN71_RS15290	ABC transporter substrate-binding protein	QEN71_15290	paras_003601	protein-codi	NZ_CP1252	chromosom	3404123	3405139	+	1017	0	25	3008	18	2570	
QEN71_RS15295	ABC transporter ATP-binding protein	QEN71_15295	paras_003602	protein-codi	NZ_CP1252	chromosom	3405191	3405988	+	798	0	8	743	3	322	
QEN71_RS15300	ABC transporter permease	QEN71_15300	paras_003603	protein-codi	NZ_CP1252	chromosom	3406015	3406878	+	864	0	11	1123	4	156	
QEN71_RS15305	heavy metal response regulator transcription factor	QEN71_15305	paras_003604	protein-codi	NZ_CP1252	chromosom	3407044	3407721	+	677	0	14	1196	9	836	
QEN71_RS15310	heavy metal sensor histidine kinase	pseudo;QEN71_15310		pseudogene	NZ_CP1252	chromosom	3407721	3409172	+	1451	0	19	1629	16	1561	
QEN71_RS15315	DUF4148 domain-containing protein	QEN71_15315	paras_003606	protein-codi	NZ_CP1252	chromosom	3409193	3409420	-	228	0	4	97	2	73	
QEN71_RS15320	ATP-binding protein	QEN71_15320	paras_003607	protein-codi	NZ_CP1252	chromosom	3409692	3411026	-	1331	0	20	3680	14	2705	
QEN71_RS15325	response regulator	QEN71_15325	paras_003608	protein-codi	NZ_CP1252	chromosom	3411023	3411685	-	659	0	15	3336	12	2686	
QEN71_RS15330	transcriptional repressor	QEN71_15330	paras_003609	protein-codi	NZ_CP1252	chromosom	3411770	3412222	-	453	0	17	3906	13	3534	
QEN71_RS15335	DUF1810 domain-containing protein	QEN71_15335	paras_003610	protein-codi	NZ_CP1252	chromosom	3412352	3412771	-	420	0	15	2149	15	2149	
QEN71_RS15340	ubiquinol oxidase subunit II	QEN71_15340	paras_003611	protein-codi	NZ_CP1252	chromosom	3413048	3413950	+	903	0	33	6911	16	2021	
QEN71_RS15345	cytochrome o ubiquinol oxidase subunit I	QEN71_15345	paras_003612	protein-codi	NZ_CP1252	chromosom	3413953	3415959	+	2006	98.0	56	10210	38	6513	
QEN71_RS15350	cytochrome o ubiquinol oxidase subunit III	QEN71_15350	paras_003613	protein-codi	NZ_CP1252	chromosom	3415959	3416567	+	608	0	13	2680	5	1403	
QEN71_RS15355	cytochrome o ubiquinol oxidase subunit IV	QEN71_15355	paras_003614	protein-codi	NZ_CP1252	chromosom	3416568	3416912	+	345	0	8	1316	6	658	
QEN71_RS15360	hypothetical protein	QEN71_15360	paras_003615	protein-codi	NZ_CP1252	chromosom	3416989	3417237	-	249	0	2	312	2	312	
QEN71_RS15365	NADH-quinone oxidoreductase subunit M	QEN71_15365	paras_003616	protein-codi	NZ_CP1252	chromosom	3417640	3419175	+	1536	0	27	7751	25	7741	
QEN71_RS15370	histone deacetylase family protein	QEN71_15370	paras_003617	protein-codi	NZ_CP1252	chromosom	3419266	3420294	-	1029	0	24	6150	22	5355	
QEN71_RS15375	Zn-dependent hydrolase	QEN71_15375	paras_003618	protein-codi	NZ_CP1252	chromosom	3420312	3421568	-	1257	0	15	1824	12	1523	
QEN71_RS15380	MFS transporter	QEN71_15380	paras_003619	protein-codi	NZ_CP1252	chromosom	3421602	3422909	-	1308	0	30	7655	15	3444	
QEN71_RS15385	LysR family transcriptional regulator	QEN71_15385	paras_003620	protein-codi	NZ_CP1252	chromosom	3423112	3424068	+	957	0	16	3031	12	2268	
QEN71_RS15390	putative urea ABC transporter substrate-binding pro	QEN71_15390	paras_003621	protein-codi	NZ_CP1252	chromosom	3424426	3425493	+	1068	0	18	2039	15	1352	
QEN71_RS15395	ABC transporter permease subunit	QEN71_15395	paras_003622	protein-codi	NZ_CP1252	chromosom	3425524	3426339	+	812	0	15	1748	12	1311	
QEN71_RS15400	ABC transporter ATP-binding protein	QEN71_15400	paras_003623	protein-codi	NZ_CP1252	chromosom	3426336	3427109	+	770	0	4	171	4	171	
QEN71_RS15405	urea carboxylase-associated family protein	QEN71_15405	paras_003624	protein-codi	NZ_CP1252	chromosom	3427147	3427854	+	708	0	13	768	10	496	
QEN71_RS15410	urea carboxylase-associated family protein	QEN71_15410	paras_003625	protein-codi	NZ_CP1252	chromosom	3427869	3428513	+	645	0	17	1469	15	1226	
QEN71_RS15415	urea carboxylase	QEN71_15415	paras_003626	protein-codi	NZ_CP1252	chromosom	3428711	3432322	+	3612	0	62	5369	52	4787	
QEN71_RS15420	allophanate hydrolase	QEN71_15420	paras_003627	protein-codi	NZ_CP1252	chromosom	3432360	3434189	+	1830	0	31	4063	28	3616	
QEN71_RS15425	hypothetical protein	QEN71_15425	paras_003628	protein-codi	NZ_CP1252	chromosom	3434200	3434604	-	405	0	13	831	8	320	
QEN71_RS15430	glycine zipper 2TM domain-containing protein	QEN71_15430	paras_003629	protein-codi	NZ_CP1252	chromosom	3434643	3435119	-	477	0	2	33	1	11	TRUE
QEN71_RS15435	RidA family protein	QEN71_15435	paras_003630	protein-codi	NZ_CP1252	chromosom	3435320	3435712	+	393	0	7	761	7	761	
QEN71_RS15440	antibiotic biosynthesis monooxygenase	QEN71_15440	paras_003631	protein-codi	NZ_CP1252	chromosom	3435737	3436411	+	675	0	11	1944	5	1270	
QEN71_RS15445	oxidoreductase	QEN71_15445	paras_003632	protein-codi	NZ_CP1252	chromosom	3436416	3437279	-	864	0	8	207	7	121	
QEN71_RS15450	cupin	QEN71_15450	paras_003633	protein-codi	NZ_CP1252	chromosom	3437404	3437670	-	267	0	7	1154	4	771	
QEN71_RS15455	secondary thiamine-phosphate synthase enzyme Yj	QEN71_15455	paras_003634	protein-codi	NZ_CP1252	chromosom	3437787	3438206	+	420	0	11	1346	9	1059	
QEN71_RS15460	hypothetical protein	QEN71_15460	paras_003635	protein-codi	NZ_CP1252	chromosom	3438242	3438574	+	333	0	7	1213	5	1050	
QEN71_RS15465	DUF3562 domain-containing protein	QEN71_15465	paras_003636	protein-codi	NZ_CP1252	chromosom	3438860	3439057	+	198	0	10	1365	7	1058	
QEN71_RS15470	CHAD domain-containing protein	QEN71_15470	paras_003637	protein-codi	NZ_CP1252	chromosom	3439137	3439970	-	834	0	23	2551	18	2115	
QEN71_RS15475	protein tyrosine phosphatase	QEN71_15475	paras_003638	protein-codi	NZ_CP1252	chromosom	3440086	3440604	-	519	0	9	546	6	345	
QEN71_RS15480	FKBP-type peptidyl-prolyl cis-trans isomerase	QEN71_15480	paras_003639	protein-codi	NZ_CP1252	chromosom	3440874	3441278	-	405	0	6	855	3	351	
QEN71_RS15485	carboxymuconolactone decarboxylase family protei	QEN71_15485	paras_003640	protein-codi	NZ_CP1252	chromosom	3441489	3442022	-	534	0	8	1409	8	1409	
QEN71_RS15490	hypothetical protein	QEN71_15490	paras_003641	protein-codi	NZ_CP1252	chromosom	3442258	3443157	+	900	0	51	7690	36	5751	
QEN71_RS15495	hypothetical protein	QEN71_15495	paras_003642	protein-codi	NZ_CP1252	chromosom	3443195	3443449	-	255	0	6	2239	6	2239	
QEN71_RS15500	methyl-accepting chemotaxis protein	QEN71_15500	paras_003643	protein-codi	NZ_CP1252	chromosom	3443792	3445300	-	1509	0	17	2760	10	1251	
QEN71_RS15505	hypothetical protein	QEN71_15505	paras_003644	protein-codi	NZ_CP1252	chromosom	3445659	3445877	-	219	0	6	1772	3	1476	
QEN71_RS15510	response regulator	QEN71_15510	paras_003645	protein-codi	NZ_CP1252	chromosom	3446313	3446786	+	474	0	12	2595	10	2432	
QEN71_RS15515	phospholipase D family protein	QEN71_15515	paras_003646	protein-codi	NZ_CP1252	chromosom	3446873	3448438	+	1566	0	31	9119	25	6951	
QEN71_RS15520	lytic transglycosylase domain-containing protein	QEN71_15520	paras_003647	protein-codi	NZ_CP1252	chromosom	3448585	3449160	+	576	0	16	1676	13	1193	
QEN71_RS15525	LysR family transcriptional regulator	QEN71_15525	paras_003648	protein-codi	NZ_CP1252	chromosom	3449163	3450056	-	894	0	7	2448	5	2012	
QEN71_RS15530	sulfite exporter TauE/SafE family protein	QEN71_15530	paras_003649	protein-codi	NZ_CP1252	chromosom	3450132	3450890	+	747	0	14	1959	7	531	
QEN71_RS15535	hypothetical protein	QEN71_15535	paras_003650	protein-codi	NZ_CP1252	chromosom	3450879	3451298	-	408	0	10	1231	8	1064	
QEN71_RS15540	hypothetical protein	QEN71_15540	paras_003651	protein-codi	NZ_CP1252	chromosom	3451347	3451976	-	630	0	7	1616	7	1616	
QEN71_RS15545	nitroreductase	QEN71_15545	paras_003652	protein-codi	NZ_CP1252	chromosom	3452153	3452824	+	672	0	10	1472	7	981	
QEN71_RS15550	linear amide C-N hydrolase	QEN71_15550	paras_003653	protein-codi	NZ_CP1252	chromosom	3452832	3453923	-	1092	0	31	6355	25	4877	
QEN71_RS15555	GAF domain-containing protein	QEN71_15555	paras_003654	protein-codi	NZ_CP1252	chromosom	3454081	3454569	+	489	0	5	356	3	178	
QEN71_RS15560	catalase	QEN71_15560	paras_003655	protein-codi	NZ_CP1252	chromosom	3454658	3456121	-	1464	0	27	3761	21	2762	
QEN71_RS15565	MurR/RpiR family transcriptional regulator	QEN71_15565	paras_003656	protein-codi	NZ_CP1252	chromosom	3456556	3457434	+	879	0	25	4668	20	2407	
QEN71_RS15570	D-alanyl-D-alanine dipeptidase	QEN71_15570	paras_003657	protein-codi	NZ_CP1252	chromosom	3457440	3458006	+	567	0	18	1597	14	1012	

QEN71_RS15575	ABC transporter substrate-binding protein	QEN71_15575	paras_003658	protein-codi	NZ_CP1252	chromosom	3458051	3459658	+	1608	0	37	4595	31	3621	
QEN71_RS15580	ABC transporter permease	QEN71_15580	paras_003659	protein-codi	NZ_CP1252	chromosom	3459671	3460741	+	1071	0	16	1172	13	1119	
QEN71_RS15585	ABC transporter permease	QEN71_15585	paras_003660	protein-codi	NZ_CP1252	chromosom	3460800	3461714	+	915	0	7	400	7	400	
QEN71_RS15590	ABC transporter ATP-binding protein	QEN71_15590	paras_003661	protein-codi	NZ_CP1252	chromosom	3461716	3462597	+	878	0	13	715	13	715	
QEN71_RS15595	ABC transporter ATP-binding protein	QEN71_15595	paras_003662	protein-codi	NZ_CP1252	chromosom	3462594	3463361	+	764	0	5	240	5	240	
QEN71_RS15600	aromatic acid/H+ symport family MFS transporter	QEN71_15600	paras_003663	protein-codi	NZ_CP1252	chromosom	3463381	3464745	-	1365	0	10	665	7	516	
QEN71_RS15605	NIPSNAP family protein	QEN71_15605	paras_003664	protein-codi	NZ_CP1252	chromosom	3464819	3465157	-	339	0	8	575	8	575	
QEN71_RS15610	FAD-dependent oxidoreductase	QEN71_15610	paras_003665	protein-codi	NZ_CP1252	chromosom	3465169	3466938	-	1770	0	27	2208	22	1346	
QEN71_RS15615	SDR family oxidoreductase	QEN71_15615	paras_003666	protein-codi	NZ_CP1252	chromosom	3466966	3467796	-	831	0	9	1194	8	914	
QEN71_RS15620	helix-turn-helix domain-containing protein	QEN71_15620	paras_003667	protein-codi	NZ_CP1252	chromosom	3467942	3468835	+	894	0	15	806	13	720	
QEN71_RS15625	4-hydroxybenzoate 3-monooxygenase	QEN71_15625	paras_003668	protein-codi	NZ_CP1252	chromosom	3468862	3470034	-	1173	0	20	3053	16	2322	
QEN71_RS15630	BON domain-containing protein	QEN71_15630	paras_003669	protein-codi	NZ_CP1252	chromosom	3470255	3470611	+	353	0	13	2009	10	1716	
QEN71_RS15635	XdhC family protein	QEN71_15635	paras_003670	protein-codi	NZ_CP1252	chromosom	3470608	3471672	-	1061	0	21	2719	21	2719	
QEN71_RS15640	cytochrome c	QEN71_15640	paras_003671	protein-codi	NZ_CP1252	chromosom	3471705	3472952	-	1244	0	28	3040	22	2605	
QEN71_RS15645	(2Fe-2S)-binding protein	QEN71_15645	paras_003672	protein-codi	NZ_CP1252	chromosom	3472949	3473545	-	593	0	9	727	6	524	
QEN71_RS15650	molybdopterin-dependent oxidoreductase	QEN71_15650	paras_003673	protein-codi	NZ_CP1252	chromosom	3473584	3476418	-	2835	0	46	4544	34	3364	
QEN71_RS15655	(2Fe-2S)-binding protein	QEN71_15655	paras_003674	protein-codi	NZ_CP1252	chromosom	3476686	3477147	-	449	0	15	1251	10	602	
QEN71_RS15660	molybdopterin-dependent oxidoreductase	QEN71_15660	paras_003675	protein-codi	NZ_CP1252	chromosom	3477135	3479453	-	2306	0	30	4132	20	2958	
QEN71_RS15665	virulence factor family protein	QEN71_15665	paras_003676	protein-codi	NZ_CP1252	chromosom	3480048	3481325	-	1274	0	17	2493	13	1630	
QEN71_RS15670	bifunctional lysylphosphatidylglycerol flippase/synth	QEN71_15670	paras_003677	protein-codi	NZ_CP1252	chromosom	3481322	3483916	-	2591	0	46	7959	31	4357	
QEN71_RS15675	DHA2 family efflux MFS transporter permease subu	QEN71_15675	paras_003678	protein-codi	NZ_CP1252	chromosom	3484491	3486050	-	1560	0	18	3345	14	2901	
QEN71_RS15680	HlyD family efflux transporter periplasmic adaptor su	QEN71_15680	paras_003679	protein-codi	NZ_CP1252	chromosom	3486126	3487388	-	1263	0	28	4982	26	4613	
QEN71_RS15685	efflux transporter outer membrane subunit	QEN71_15685	paras_003680	protein-codi	NZ_CP1252	chromosom	3487423	3488931	-	1509	0	30	3154	22	2444	
QEN71_RS15690	MarR family transcriptional regulator	QEN71_15690	paras_003681	protein-codi	NZ_CP1252	chromosom	3488969	3489406	-	438	0	2	135	0	0	TRUE
QEN71_RS15695	MarR family transcriptional regulator	QEN71_15695	paras_003682	protein-codi	NZ_CP1252	chromosom	3489560	3489997	-	438	0	2	240	0	0	TRUE
QEN71_RS15700	nitric oxide reductase transcriptional regulator NorR	QEN71_15700	paras_003683	protein-codi	NZ_CP1252	chromosom	3490086	3491684	-	1599	0	20	3435	17	2926	
QEN71_RS15705	NO-inducible flavohemoprotein	QEN71_15705	paras_003684	protein-codi	NZ_CP1252	chromosom	3491817	3492998	+	1182	0	24	6483	20	5851	
QEN71_RS15710	muconolactone Delta-isomerase	QEN71_15710	paras_003685	protein-codi	NZ_CP1252	chromosom	3493138	3493428	-	291	0	12	1103	9	831	
QEN71_RS15715	catechol 1,2-dioxygenase	QEN71_15715	paras_003686	protein-codi	NZ_CP1252	chromosom	3493505	3494407	-	903	0	14	2018	14	2018	
QEN71_RS15720	muconate/chloromuconate family cycloisomerase	QEN71_15720	paras_003687	protein-codi	NZ_CP1252	chromosom	3494488	3495645	-	1158	0	17	2291	8	1223	
QEN71_RS15725	LysR family transcriptional regulator	QEN71_15725	paras_003688	protein-codi	NZ_CP1252	chromosom	3495736	3496629	+	871	0	13	1386	12	1377	
QEN71_RS15730	hypothetical protein	QEN71_15730	paras_003689	protein-codi	NZ_CP1252	chromosom	3496607	3497059	-	430	0	0	0	0	0	TRUE
QEN71_RS15735	YbfB/YjiJ family MFS transporter	QEN71_15735	paras_003690	protein-codi	NZ_CP1252	chromosom	3497176	3498414	-	1239	0	14	877	10	649	
QEN71_RS15740	cupin domain-containing protein	QEN71_15740	paras_003691	protein-codi	NZ_CP1252	chromosom	3498730	3499161	+	432	0	8	1283	3	875	
QEN71_RS15745	DUF3331 domain-containing protein	QEN71_15745	paras_003692	protein-codi	NZ_CP1252	chromosom	3499611	3499934	+	324	0	7	595	7	595	
QEN71_RS15750	NAD(P)H-dependent oxidoreductase	QEN71_15750	paras_003693	protein-codi	NZ_CP1252	chromosom	3500443	3501114	+	672	0	13	1426	11	1369	
QEN71_RS15755	Rrf2 family transcriptional regulator	QEN71_15755	paras_003694	protein-codi	NZ_CP1252	chromosom	3501213	3501764	-	552	0	6	147	5	82	
QEN71_RS15760	LysR family transcriptional regulator	QEN71_15760	paras_003695	protein-codi	NZ_CP1252	chromosom	3501862	3502788	-	927	0	19	2316	18	2143	
QEN71_RS15765	NCS1 family nucleobase:cation symporter-1	QEN71_15765	paras_003696	protein-codi	NZ_CP1252	chromosom	3503006	3504475	-	1470	0	38	5185	35	4942	
QEN71_RS15770	Lrp/AsnC family transcriptional regulator	QEN71_15770	paras_003697	protein-codi	NZ_CP1252	chromosom	3504962	3505435	+	474	0	12	962	12	962	
QEN71_RS15775	GNAT family N-acetyltransferase	QEN71_15775	paras_003698	protein-codi	NZ_CP1252	chromosom	3505460	3506410	-	951	0	17	2293	11	1449	
QEN71_RS15780	TonB-dependent siderophore receptor	QEN71_15780	paras_003699	protein-codi	NZ_CP1252	chromosom	3506461	3508683	-	2223	0	50	4895	38	4092	
QEN71_RS15785	lysine N(6)-hydroxylase/L-ornithine N(5)-oxygenase	QEN71_15785	paras_003700	protein-codi	NZ_CP1252	chromosom	3508733	3510133	-	1401	0	13	652	12	650	
QEN71_RS15790	GNAT family N-acetyltransferase	QEN71_15790	paras_003701	protein-codi	NZ_CP1252	chromosom	3510198	3511223	-	1015	0	8	696	7	692	
QEN71_RS15795	non-ribosomal peptide synthetase	QEN71_15795	paras_003702	protein-codi	NZ_CP1252	chromosom	3511213	3516195	-	4972	0	44	4073	38	3550	
QEN71_RS15800	non-ribosomal peptide synthetase	QEN71_15800	paras_003703	protein-codi	NZ_CP1252	chromosom	3516207	3525809	-	9603	0	92	10450	70	8632	
QEN71_RS15805	cyclic peptide export ABC transporter	QEN71_15805	paras_003704	protein-codi	NZ_CP1252	chromosom	3526074	3527867	+	1794	0	16	1008	15	961	
QEN71_RS15810	iron-siderophore ABC transporter substrate-binding	QEN71_15810	paras_003705	protein-codi	NZ_CP1252	chromosom	3527955	3528956	-	998	0	8	484	7	383	
QEN71_RS15815	siderophore-iron reductase FhuF	QEN71_15815	paras_003706	protein-codi	NZ_CP1252	chromosom	3528953	3529753	-	797	0	13	1560	9	876	
QEN71_RS15820	Fe(3+)-hydroxamate ABC transporter permease Fhu	QEN71_15820	paras_003707	protein-codi	NZ_CP1252	chromosom	3529756	3531870	-	2111	0	25	2901	21	2031	
QEN71_RS15825	ABC transporter ATP-binding protein	QEN71_15825	paras_003708	protein-codi	NZ_CP1252	chromosom	3531867	3532712	-	842	0	12	1637	12	1637	
QEN71_RS15830	TauD/TfdA family dioxygenase	QEN71_15830	paras_003709	protein-codi	NZ_CP1252	chromosom	3532715	3533740	-	1026	0	25	5993	20	5070	
QEN71_RS15835	MbtH family NRPS accessory protein	QEN71_15835	paras_003710	protein-codi	NZ_CP1252	chromosom	3533770	3534003	-	234	0	8	1116	6	1105	
QEN71_RS15840	RNA polymerase factor sigma-70	QEN71_15840	paras_003711	protein-codi	NZ_CP1252	chromosom	3534081	3534704	-	624	0	7	1643	5	1541	
QEN71_RS15845	4'-phosphopantetheinyl transferase superfamily pro	QEN71_15845	paras_003712	protein-codi	NZ_CP1252	chromosom	3534928	3535623	-	696	0	16	4124	12	3463	
QEN71_RS15850	MFS transporter	QEN71_15850	paras_003713	protein-codi	NZ_CP1252	chromosom	3535689	3537020	-	1332	0	38	5141	29	2977	
QEN71_RS15855	DUF2783 domain-containing protein	QEN71_15855	paras_003714	protein-codi	NZ_CP1252	chromosom	3537133	3537375	-	243	0	6	602	6	602	
QEN71_RS15860	FAD-dependent oxidoreductase	QEN71_15860	paras_003715	protein-codi	NZ_CP1252	chromosom	3537415	3539127	-	1713	0	29	4617	20	3315	
QEN71_RS15865	MBL fold metallo-hydrolase	QEN71_15865	paras_003716	protein-codi	NZ_CP1252	chromosom	3539170	3540129	-	960	0	30	4056	27	3525	
QEN71_RS15870	IclR family transcriptional regulator	QEN71_15870	paras_003717	protein-codi	NZ_CP1252	chromosom	3540367	3541209	+	843	0	5	1028	2	674	
QEN71_RS15875	IclR family transcriptional regulator	QEN71_15875	paras_003718	protein-codi	NZ_CP1252	chromosom	3541212	3542012	+	801	0	29	3694	26	3581	
QEN71_RS15880	MurR/RpiR family transcriptional regulator	QEN71_15880	paras_003719	protein-codi	NZ_CP1252	chromosom	3542649	3543515	-	867	0	31	4189	28	3773	

QEN71_RS15885	sn-glycerol-3-phosphate ABC transporter ATP-bindi	QEN71_15885	paras_003720	protein-codi	NZ_CP1252	chromosom	3543559	3544632	-	1074	0	18	3067	12	2073	
QEN71_RS15890	SDR family oxidoreductase	QEN71_15890	paras_003721	protein-codi	NZ_CP1252	chromosom	3544682	3545455	-	774	0	12	1381	10	1031	
QEN71_RS15895	sugar ABC transporter substrate-binding protein	QEN71_15895	paras_003722	protein-codi	NZ_CP1252	chromosom	3545722	3546954	+	1233	0	30	4557	25	4138	
QEN71_RS15900	sugar ABC transporter permease	QEN71_15900	paras_003723	protein-codi	NZ_CP1252	chromosom	3547080	3548009	+	926	0	22	3869	18	3240	
QEN71_RS15905	carbohydrate ABC transporter permease	QEN71_15905	paras_003724	protein-codi	NZ_CP1252	chromosom	3548006	3548851	+	842	0	15	3869	12	2603	
QEN71_RS15910	carboxymuconolactone decarboxylase family protei	QEN71_15910	paras_003725	protein-codi	NZ_CP1252	chromosom	3548990	3549427	+	438	0	8	1380	4	711	
QEN71_RS15915	hypothetical protein	QEN71_15915	paras_003726	protein-codi	NZ_CP1252	chromosom	3549509	3549784	-	276	0	4	1580	2	504	
QEN71_RS15920	dienelactone hydrolase family protein	QEN71_15920	paras_003727	protein-codi	NZ_CP1252	chromosom	3549933	3550643	+	711	0	25	5095	22	4217	
QEN71_RS15925	YoaK family protein	QEN71_15925	paras_003728	protein-codi	NZ_CP1252	chromosom	3550697	3551455	-	759	0	25	5375	19	4702	
QEN71_RS15930	hypothetical protein	QEN71_15930	paras_003729	protein-codi	NZ_CP1252	chromosom	3551649	3552083	-	435	0	10	1620	5	691	
QEN71_RS15935	class I SAM-dependent methyltransferase	QEN71_15935	paras_003730	protein-codi	NZ_CP1252	chromosom	3552275	3553090	-	816	0	13	2033	8	1377	
QEN71_RS15940	metalloregulator ArsR/SmtB family transcription fact	QEN71_15940	paras_003731	protein-codi	NZ_CP1252	chromosom	3553184	3553552	+	340	0	3	456	0	0	
QEN71_RS15945	SRPBCC family protein	QEN71_15945	paras_003732	protein-codi	NZ_CP1252	chromosom	3553524	3554021	+	469	0	7	1625	5	1034	
QEN71_RS15950	ATP-binding protein	QEN71_15950	paras_003733	protein-codi	NZ_CP1252	chromosom	3554057	3555388	-	1332	0	16	1930	8	1233	
QEN71_RS15955	CoA transferase	QEN71_15955	paras_003734	protein-codi	NZ_CP1252	chromosom	3555493	3557463	-	1971	0	29	5061	27	4995	
QEN71_RS15960	LysR substrate-binding domain-containing protein	QEN71_15960	paras_003735	protein-codi	NZ_CP1252	chromosom	3557630	3558529	+	900	0	7	539	5	342	
QEN71_RS15965	enoyl-CoA hydratase-related protein	QEN71_15965	paras_003736	protein-codi	NZ_CP1252	chromosom	3558557	3559360	-	804	0	12	1358	8	1187	
QEN71_RS15970	SDR family NAD(P)-dependent oxidoreductase	QEN71_15970	paras_003737	protein-codi	NZ_CP1252	chromosom	3559371	3560138	-	768	0	5	618	2	50	
QEN71_RS15975	MFS transporter	QEN71_15975	paras_003738	protein-codi	NZ_CP1252	chromosom	3560430	3561734	+	1305	0	15	1949	7	598	
QEN71_RS15980	thiolase family protein	QEN71_15980	paras_003739	protein-codi	NZ_CP1252	chromosom	3561797	3562969	+	1173	0	11	969	8	703	
QEN71_RS15985	fatty acid desaturase	QEN71_15985	paras_003740	protein-codi	NZ_CP1252	chromosom	3563021	3564022	-	1002	0	24	2964	24	2964	
QEN71_RS15990	sugar ABC transporter substrate-binding protein	QEN71_15990	paras_003741	protein-codi	NZ_CP1252	chromosom	3564054	3565097	-	1044	0	17	1463	13	1194	
QEN71_RS15995	LacI family DNA-binding transcriptional regulator	QEN71_15995	paras_003742	protein-codi	NZ_CP1252	chromosom	3565248	3566279	+	1032	0	18	1396	11	990	
QEN71_RS16000	LysR substrate-binding domain-containing protein	QEN71_16000	paras_003743	protein-codi	NZ_CP1252	chromosom	3566287	3567162	-	876	0	6	292	4	274	
QEN71_RS16005	VOC family protein	QEN71_16005	paras_003744	protein-codi	NZ_CP1252	chromosom	3567266	3567691	+	426	0	6	1196	6	1196	
QEN71_RS16010	LacI family DNA-binding transcriptional regulator	QEN71_16010	paras_003745	protein-codi	NZ_CP1252	chromosom	3567743	3568798	-	1056	0	17	3332	15	3003	
QEN71_RS16015	TRAP transporter substrate-binding protein	QEN71_16015	paras_003746	protein-codi	NZ_CP1252	chromosom	3569059	3570081	+	1023	0	18	2497	11	2186	
QEN71_RS16020	TRAP transporter large permease subunit	QEN71_16020	paras_003747	protein-codi	NZ_CP1252	chromosom	3570126	3572024	+	1899	0	19	1819	12	1149	
QEN71_RS16025	VOC family protein	QEN71_16025	paras_003748	protein-codi	NZ_CP1252	chromosom	3572094	3572633	+	536	0	10	785	9	754	
QEN71_RS16030	ribulose-bisphosphate carboxylase large subunit fa	QEN71_16030	paras_003749	protein-codi	NZ_CP1252	chromosom	3572630	3573907	+	1270	0	12	1776	10	1595	
QEN71_RS16035	four-carbon acid sugar kinase family protein	QEN71_16035	paras_003750	protein-codi	NZ_CP1252	chromosom	3573904	3575223	+	1316	0	21	2011	16	1661	
QEN71_RS16040	DNA-binding protein	QEN71_16040	paras_003751	protein-codi	NZ_CP1252	chromosom	3575239	3578688	-	3450	0	29	6307	20	4611	
QEN71_RS16045	bifunctional salicylyl-CoA 5-hydroxylase/oxidoreduc	QEN71_16045	paras_003752	protein-codi	NZ_CP1252	chromosom	3578922	3581294	+	2372	0	45	7455	34	6034	
QEN71_RS16050	SDR family NAD(P)-dependent oxidoreductase	QEN71_16050	paras_003753	protein-codi	NZ_CP1252	chromosom	3581294	3582073	+	775	0	11	1419	11	1419	
QEN71_RS16055	MarR family transcriptional regulator	QEN71_16055	paras_003754	protein-codi	NZ_CP1252	chromosom	3582070	3582627	+	554	0	3	125	2	105	
QEN71_RS16060	enoyl-CoA hydratase family protein	QEN71_16060	paras_003755	protein-codi	NZ_CP1252	chromosom	3582658	3583509	+	852	0	14	2071	10	1228	
QEN71_RS16065	acyl-CoA dehydrogenase family protein	QEN71_16065	paras_003756	protein-codi	NZ_CP1252	chromosom	3583512	3584699	+	1184	0	22	3633	15	2007	
QEN71_RS16070	thioesterase family protein	QEN71_16070	paras_003757	protein-codi	NZ_CP1252	chromosom	3584696	3585139	+	436	0	7	298	7	298	
QEN71_RS16075	RidA family protein	QEN71_16075	paras_003758	protein-codi	NZ_CP1252	chromosom	3585136	3585534	+	395	0	7	1012	6	465	
QEN71_RS16080	AMP-binding protein	QEN71_16080	paras_003759	protein-codi	NZ_CP1252	chromosom	3585599	3587239	+	1641	0	33	2672	21	1753	
QEN71_RS16085	dienelactone hydrolase family protein	QEN71_16085	paras_003760	protein-codi	NZ_CP1252	chromosom	3587315	3588538	+	1224	0	51	12853	48	12176	
QEN71_RS16090	glutathione S-transferase	QEN71_16090	paras_003761	protein-codi	NZ_CP1252	chromosom	3588660	3589331	+	672	0	18	3276	15	2898	
QEN71_RS16095	DHA2 family efflux MFS transporter permease subu	QEN71_16095	paras_003762	protein-codi	NZ_CP1252	chromosom	3589462	3590943	+	1478	0	13	3394	9	2390	
QEN71_RS16100	nuclear transport factor 2 family protein	QEN71_16100	paras_003763	protein-codi	NZ_CP1252	chromosom	3590940	3591302	+	359	0	7	1625	7	1625	
QEN71_RS16105	AraC family transcriptional regulator	QEN71_16105	paras_003764	protein-codi	NZ_CP1252	chromosom	3591378	3592223	+	846	0	21	4895	15	4071	
QEN71_RS16110	flavin reductase family protein	QEN71_16110	paras_003765	protein-codi	NZ_CP1252	chromosom	3592230	3592814	-	585	0	10	3114	10	3114	
QEN71_RS16115	hypothetical protein	QEN71_16115	paras_003766	protein-codi	NZ_CP1252	chromosom	3592978	3593304	-	327	0	6	918	4	830	
QEN71_RS16120	DUF2252 domain-containing protein	QEN71_16120	paras_003767	protein-codi	NZ_CP1252	chromosom	3593554	3594768	-	1215	0	36	14380	25	10315	
QEN71_RS16125	GNAT family protein	QEN71_16125	paras_003768	protein-codi	NZ_CP1252	chromosom	3594994	3595713	+	720	0	26	8631	17	5909	
QEN71_RS16130	type II toxin-antitoxin system PemK/MazF family tox	QEN71_16130	paras_003769	protein-codi	NZ_CP1252	chromosom	3595729	3596073	-	345	0	0	0	0	0	TRUE
QEN71_RS16135	AbrB/MazE/SpoVT family DNA-binding domain-cont	QEN71_16135	paras_003770	protein-codi	NZ_CP1252	chromosom	3596082	3596387	-	306	0	8	1506	3	196	
QEN71_RS16140	GNAT family N-acetyltransferase	QEN71_16140	paras_003771	protein-codi	NZ_CP1252	chromosom	3596612	3597166	-	555	0	19	8549	16	6765	
QEN71_RS16145	VOC family protein	QEN71_16145	paras_003772	protein-codi	NZ_CP1252	chromosom	3597248	3597724	-	477	0	9	2892	7	1855	
QEN71_RS16150	VOC family protein	QEN71_16150	paras_003773	protein-codi	NZ_CP1252	chromosom	3597801	3598286	-	486	0	12	9168	8	1570	
QEN71_RS16155	YXWGXW repeat-containing protein	QEN71_16155	paras_003774	protein-codi	NZ_CP1252	chromosom	3598435	3598653	-	219	0	5	2562	5	2562	
QEN71_RS16160	DUF1501 domain-containing protein	QEN71_16160	paras_003775	protein-codi	NZ_CP1252	chromosom	3598729	3599901	-	1173	0	19	8376	17	7963	
QEN71_RS16165	DUF1800 domain-containing protein	QEN71_16165	paras_003776	protein-codi	NZ_CP1252	chromosom	3599904	3601415	-	1512	0	20	4670	19	4597	
QEN71_RS16170	periplasmic heavy metal sensor	QEN71_16170	paras_003777	protein-codi	NZ_CP1252	chromosom	3601532	3602065	-	530	0	6	545	6	545	
QEN71_RS16175	hypothetical protein	QEN71_16175	paras_003778	protein-codi	NZ_CP1252	chromosom	3602062	3602517	-	448	0	3	121	1	22	TRUE
QEN71_RS16180	RNA polymerase sigma factor	QEN71_16180	paras_003779	protein-codi	NZ_CP1252	chromosom	3602514	3603080	-	563	0	9	2565	9	2565	
QEN71_RS16185	lipoprotein	QEN71_16185		protein-codi	NZ_CP1252	chromosom	3603248	3603352	-	105	0	10	3398	6	2265	
QEN71_RS16190	YXWGXW repeat-containing protein	QEN71_16190	paras_003780	protein-codi	NZ_CP1252	chromosom	3603372	3603674	-	303	0	7	3819	4	587	

QEN71_RS16195	low affinity iron permease family protein	QEN71_16195	paras_003781	protein-codi	NZ_CP1252	chromosom	3603842	3604306	+	465	0	9	2508	9	2508	
QEN71_RS16200	helix-turn-helix transcriptional regulator	QEN71_16200	paras_003782	protein-codi	NZ_CP1252	chromosom	3604310	3605113	+	804	0	14	2559	11	2373	
QEN71_RS16205	DUF1330 domain-containing protein	QEN71_16205	paras_003783	protein-codi	NZ_CP1252	chromosom	3605230	3605523	+	294	0	11	1827	6	1099	
QEN71_RS16210	DUF429 domain-containing protein	QEN71_16210	paras_003784	protein-codi	NZ_CP1252	chromosom	3605582	3606304	-	723	0	7	949	4	708	
QEN71_RS16215	NADP-dependent oxidoreductase	QEN71_16215	paras_003785	protein-codi	NZ_CP1252	chromosom	3606411	3607367	+	957	0	17	3942	15	3494	
QEN71_RS16220	DUF1427 family protein	QEN71_16220	paras_003786	protein-codi	NZ_CP1252	chromosom	3607397	3607573	+	177	0	10	1999	2	409	
QEN71_RS16225	RNA polymerase sigma factor	QEN71_16225	paras_003787	protein-codi	NZ_CP1252	chromosom	3607591	3608883	-	1293	0	27	5760	25	5633	
QEN71_RS16230	VOC family protein	QEN71_16230	paras_003788	protein-codi	NZ_CP1252	chromosom	3608891	3609310	-	420	0	9	2838	6	2077	
QEN71_RS16235	YciI family protein	QEN71_16235	paras_003789	protein-codi	NZ_CP1252	chromosom	3609362	3609775	-	414	0	12	2126	10	2020	
QEN71_RS16240	YciI family protein	QEN71_16240	paras_003790	protein-codi	NZ_CP1252	chromosom	3609895	3610251	-	357	0	13	3050	8	1159	
QEN71_RS16245	glycine zipper 2TM domain-containing protein	QEN71_16245	paras_003791	protein-codi	NZ_CP1252	chromosom	3610481	3610666	+	186	0	8	1263	4	649	
QEN71_RS16250	porin	QEN71_16250	paras_003792	protein-codi	NZ_CP1252	chromosom	3611309	3612436	+	1128	0	40	8625	35	7261	
QEN71_RS16255	SRPBCC family protein	QEN71_16255	paras_003793	protein-codi	NZ_CP1252	chromosom	3612581	3613045	-	465	0	4	315	4	315	
QEN71_RS16260	aromatic ring-hydroxylating dioxygenase subunit alp	QEN71_16260	paras_003794	protein-codi	NZ_CP1252	chromosom	3613223	3614347	-	1125	0	28	6617	20	3745	
QEN71_RS16265	glycosyltransferase	QEN71_16265	paras_003795	protein-codi	NZ_CP1252	chromosom	3614642	3616183	-	1541	0	25	8571	23	8252	
QEN71_RS16270	cytochrome b	QEN71_16270	paras_003796	protein-codi	NZ_CP1252	chromosom	3616183	3616803	-	620	0	14	5898	14	5898	
QEN71_RS16275	LysR family transcriptional regulator	QEN71_16275	paras_003797	protein-codi	NZ_CP1252	chromosom	3617021	3617935	-	915	0	3	1788	3	1788	
QEN71_RS16280	glutathione S-transferase family protein	QEN71_16280	paras_003798	protein-codi	NZ_CP1252	chromosom	3618090	3619070	+	981	0	40	13879	32	11078	
QEN71_RS16285	hypothetical protein	QEN71_16285	paras_003799	protein-codi	NZ_CP1252	chromosom	3619144	3619434	+	291	0	8	2356	6	1911	
QEN71_RS16290	thioredoxin family protein	QEN71_16290	paras_003800	protein-codi	NZ_CP1252	chromosom	3619444	3619767	-	324	0	7	1872	0	0	
QEN71_RS16295	3-deoxy-7-phosphoheptulonate synthase	QEN71_16295	paras_003801	protein-codi	NZ_CP1252	chromosom	3619854	3620978	-	1125	0	20	5750	15	4575	
QEN71_RS16300	alpha/beta fold hydrolase	QEN71_16300	paras_003802	protein-codi	NZ_CP1252	chromosom	3621064	3622146	-	1083	0	28	5296	22	4708	
QEN71_RS16305	SRPBCC family protein	QEN71_16305	paras_003803	protein-codi	NZ_CP1252	chromosom	3622220	3622756	-	537	0	11	1543	7	1081	
QEN71_RS16310	PLP-dependent aminotransferase family protein	QEN71_16310	paras_003804	protein-codi	NZ_CP1252	chromosom	3622807	3624267	-	1461	0	33	7511	27	5402	
QEN71_RS16315	GNAT family N-acetyltransferase	QEN71_16315	paras_003805	protein-codi	NZ_CP1252	chromosom	3624370	3624834	+	461	0	16	3275	13	3161	
QEN71_RS16320	DUF3501 family protein	QEN71_16320	paras_003806	protein-codi	NZ_CP1252	chromosom	3624831	3625412	-	578	0	8	1294	7	1230	
QEN71_RS16325	heterodisulfide reductase-related iron-sulfur binding	QEN71_16325	paras_003807	protein-codi	NZ_CP1252	chromosom	3625438	3626775	-	1338	0	34	9502	23	6620	
QEN71_RS16330	rubrerythrin family protein	QEN71_16330	paras_003808	protein-codi	NZ_CP1252	chromosom	3626982	3627404	-	423	0	18	4093	14	3161	
QEN71_RS16335	DUF3564 domain-containing protein	QEN71_16335	paras_003809	protein-codi	NZ_CP1252	chromosom	3627641	3628000	+	360	0	14	3708	12	3427	
QEN71_RS16340	DNA/RNA non-specific endonuclease	QEN71_16340	paras_003810	protein-codi	NZ_CP1252	chromosom	3628247	3629005	+	751	0	19	6176	15	5483	
QEN71_RS16345	hypothetical protein	QEN71_16345	paras_003811	protein-codi	NZ_CP1252	chromosom	3628998	3629255	+	246	0	2	589	0	0	
QEN71_RS16350	Crp/Fnr family transcriptional regulator	QEN71_16350	paras_003812	protein-codi	NZ_CP1252	chromosom	3629252	3629968	-	713	0	10	3184	7	2980	
QEN71_RS16355	DUF962 domain-containing protein	QEN71_16355	paras_003813	protein-codi	NZ_CP1252	chromosom	3630060	3630605	+	546	0	11	4053	7	3002	
QEN71_RS16360	EamA family transporter RarD	QEN71_16360	paras_003814	protein-codi	NZ_CP1252	chromosom	3630608	3631498	-	891	0	33	10595	25	5693	
QEN71_RS16365	L-histidine N(alpha)-methyltransferase	QEN71_16365	paras_003815	protein-codi	NZ_CP1252	chromosom	3631947	3632927	+	981	0	26	7762	23	6686	
QEN71_RS16370	acylphosphatase	QEN71_16370	paras_003816	protein-codi	NZ_CP1252	chromosom	3632987	3633283	-	297	0	13	4453	8	3977	
QEN71_RS16375	NAD-dependent epimerase/dehydratase family prot	QEN71_16375	paras_003817	protein-codi	NZ_CP1252	chromosom	3633461	3634471	+	1011	0	22	7103	16	6148	
QEN71_RS16380	hypothetical protein	QEN71_16380	paras_003818	protein-codi	NZ_CP1252	chromosom	3634590	3635120	+	531	0	10	5601	7	1775	
QEN71_RS16385	lytic transglycosylase domain-containing protein	QEN71_16385	paras_003819	protein-codi	NZ_CP1252	chromosom	3635152	3635823	-	668	0	21	4776	19	4699	
QEN71_RS16390	glycosyltransferase	QEN71_16390	paras_003820	protein-codi	NZ_CP1252	chromosom	3635820	3636998	-	1175	0	27	8792	23	7915	
QEN71_RS16395	4-hydroxy-3-methylbut-2-enyl diphosphate reductas	QEN71_16395	paras_003821	protein-codi	NZ_CP1252	chromosom	3637474	3638418	+	945	0	24	6222	19	5192	
QEN71_RS16400	adenosyl-hopene transferase HpnH	QEN71_16400	paras_003822	protein-codi	NZ_CP1252	chromosom	3638430	3639587	+	1158	0	43	11918	29	7870	
QEN71_RS16405	ABC transporter substrate-binding protein	QEN71_16405	paras_003823	protein-codi	NZ_CP1252	chromosom	3639668	3640273	-	606	0	14	2156	9	1238	
QEN71_RS16410	MMPL family transporter	QEN71_16410	paras_003824	protein-codi	NZ_CP1252	chromosom	3640457	3643069	+	2613	0	43	8581	23	5957	
QEN71_RS16415	VacJ family lipoprotein	QEN71_16415	paras_003825	protein-codi	NZ_CP1252	chromosom	3643080	3644084	+	1005	0	31	3562	25	2535	
QEN71_RS16420	hotdog domain-containing protein	QEN71_16420	paras_003826	protein-codi	NZ_CP1252	chromosom	3644172	3644645	-	474	0	8	436	6	400	
QEN71_RS16425	phosphorylase	QEN71_16425	paras_003827	protein-codi	NZ_CP1252	chromosom	3644774	3645487	-	713	0	7	871	6	778	
QEN71_RS16430	squalene--hopene cyclase	QEN71_16430	paras_003828	protein-codi	NZ_CP1252	chromosom	3645487	3647520	-	2029	0	50	9740	38	7771	
QEN71_RS16435	hydroxysqualene dehydroxylase HpnE	QEN71_16435	paras_003829	protein-codi	NZ_CP1252	chromosom	3647517	3648806	-	1285	0	18	9767	13	8254	
QEN71_RS16440	presqualene diphosphate synthase HpnD	QEN71_16440	paras_003830	protein-codi	NZ_CP1252	chromosom	3648806	3649654	-	848	0	17	1528	14	1476	
QEN71_RS16445	nuclear transport factor 2 family protein	QEN71_16445	paras_003831	protein-codi	NZ_CP1252	chromosom	3650202	3650624	+	423	0	6	2412	1	239	
QEN71_RS16450	diguanylate cyclase	QEN71_16450	paras_003832	protein-codi	NZ_CP1252	chromosom	3650681	3651619	-	935	0	26	8030	19	6264	
QEN71_RS16455	chemotaxis protein CheC	QEN71_16455	paras_003833	protein-codi	NZ_CP1252	chromosom	3651616	3652239	-	612	0	12	5200	7	893	
QEN71_RS16460	response regulator	QEN71_16460	paras_003834	protein-codi	NZ_CP1252	chromosom	3652232	3652600	-	361	0	11	6350	7	3381	
QEN71_RS16465	hybrid sensor histidine kinase/response regulator	QEN71_16465	paras_003835	protein-codi	NZ_CP1252	chromosom	3653320	3655602	+	2283	0	24	6355	24	6355	
QEN71_RS16470	hypothetical protein	QEN71_16470	paras_003836	protein-codi	NZ_CP1252	chromosom	3655819	3656877	+	1059	0	31	11126	26	8819	
QEN71_RS16475	NADPH-dependent FMN reductase	QEN71_16475	paras_003837	protein-codi	NZ_CP1252	chromosom	3656936	3657487	-	552	0	16	4437	10	3468	
QEN71_RS16480	hypothetical protein	QEN71_16480	paras_003838	protein-codi	NZ_CP1252	chromosom	3657865	3658071	+	207	0	6	1284	6	1284	
QEN71_RS16485	DUF1272 domain-containing protein	QEN71_16485	paras_003839	protein-codi	NZ_CP1252	chromosom	3658155	3658445	-	291	0	10	2741	10	2741	
QEN71_RS16490	sulfonate ABC transporter substrate-binding protein	QEN71_16490	paras_003840	protein-codi	NZ_CP1252	chromosom	3658679	3659644	+	966	0	23	5331	21	4864	
QEN71_RS16495	arsenate reductase (glutaredoxin)	QEN71_16495	paras_003841	protein-codi	NZ_CP1252	chromosom	3659706	3660077	-	372	0	20	3390	15	2033	
QEN71_RS16500	ParA family partition ATPase	QEN71_16500	paras_003842	protein-codi	NZ_CP1252	chromosom	3660628	3661290	+	663	0	0	0	0	0	TRUE

QEN71_RS16505	ParB/RepB/Spo0J family partition protein	QEN71_16505	paras_003843	protein-codi	NZ_CP1252	chromosom	3661315	3662379	+	1065	0	5	1080	2	27	
QEN71_RS16510	replication initiation protein	QEN71_16510	paras_003844	protein-codi	NZ_CP1252	chromosom	3662423	3663772	-	1350	0	3	29	3	29	TRUE
QEN71_RS16515	DNA-binding protein	QEN71_16515	paras_003845	protein-codi	NZ_CP1252	chromosom	3665691	3666896	+	1206	0	21	1499	16	915	
QEN71_RS16520	tyrosine-type recombinase/integrase	QEN71_16520	paras_003846	protein-codi	NZ_CP1252	chromosom	3666978	3668042	-	1065	0	19	4246	17	3423	
QEN71_RS16525	DUF2471 family protein	QEN71_16525	paras_003847	protein-codi	NZ_CP1252	chromosom	3668142	3668540	-	399	0	11	4288	9	4227	
QEN71_RS16530	XRE family transcriptional regulator	QEN71_16530	paras_003848	protein-codi	NZ_CP1252	chromosom	3668625	3669239	-	615	0	8	770	7	756	
QEN71_RS16535	glycine C-acetyltransferase	QEN71_16535	paras_003849	protein-codi	NZ_CP1252	chromosom	3669384	3670583	+	1200	0	18	4401	15	3931	
QEN71_RS16540	L-threonine 3-dehydrogenase	QEN71_16540	paras_003850	protein-codi	NZ_CP1252	chromosom	3670602	3671630	+	1029	0	16	4466	16	4466	
QEN71_RS16545	YceI family protein	QEN71_16545	paras_003851	protein-codi	NZ_CP1252	chromosom	3671650	3672291	-	642	0	15	1989	13	1945	
QEN71_RS16550	TetR/AcrR family transcriptional regulator	QEN71_16550	paras_003852	protein-codi	NZ_CP1252	chromosom	3672408	3673049	+	642	0	10	1886	8	1621	
QEN71_RS16555	nuclear transport factor 2 family protein	QEN71_16555	paras_003853	protein-codi	NZ_CP1252	chromosom	3673072	3673548	+	477	0	13	1922	11	1755	
QEN71_RS16560	hypothetical protein	QEN71_16560	paras_003854	protein-codi	NZ_CP1252	chromosom	3673760	3674287	+	528	0	17	3645	15	2438	
QEN71_RS16565	LysR family transcriptional regulator	QEN71_16565	paras_003855	protein-codi	NZ_CP1252	chromosom	3674645	3675514	+	870	0	15	2273	13	2084	
QEN71_RS16570	zinc-dependent alcohol dehydrogenase family prote	QEN71_16570	paras_003856	protein-codi	NZ_CP1252	chromosom	3675530	3676516	-	987	0	18	3548	14	2567	
QEN71_RS16575	ATP-binding protein	QEN71_16575	paras_003857	protein-codi	NZ_CP1252	chromosom	3676900	3678678	-	1779	0	19	5434	11	2936	
QEN71_RS16580	GFA family protein	QEN71_16580	paras_003858	protein-codi	NZ_CP1252	chromosom	3678857	3679258	+	402	0	14	1959	8	686	
QEN71_RS16585	DUF6496 domain-containing protein	QEN71_16585	paras_003859	protein-codi	NZ_CP1252	chromosom	3679343	3679495	-	153	0	2	333	0	0	
QEN71_RS16590	hypothetical protein	QEN71_16590	paras_003860	protein-codi	NZ_CP1252	chromosom	3679734	3680048	+	315	0	0	0	0	0	TRUE
QEN71_RS16595	hypothetical protein	QEN71_16595	paras_003861	protein-codi	NZ_CP1252	chromosom	3680161	3680442	+	282	0	14	2929	14	2929	
QEN71_RS16600	SDR family NAD(P)-dependent oxidoreductase	QEN71_16600	paras_003862	protein-codi	NZ_CP1252	chromosom	3680540	3681280	+	741	0	16	3918	14	3448	
QEN71_RS16605	hopanoid C-3 methylase HpnR	QEN71_16605	paras_003863	protein-codi	NZ_CP1252	chromosom	3681303	3682844	-	1542	0	43	8716	34	6592	
QEN71_RS16610	sugar efflux transporter	QEN71_16610	paras_003864	protein-codi	NZ_CP1252	chromosom	3683118	3684419	-	1302	0	21	6350	18	5690	
QEN71_RS16615	hypothetical protein	QEN71_16615	paras_003865	protein-codi	NZ_CP1252	chromosom	3684651	3685181	+	531	0	24	4387	17	2568	
QEN71_RS16620	LD-carboxypeptidase	QEN71_16620	paras_003866	protein-codi	NZ_CP1252	chromosom	3685182	3686054	-	869	0	17	3887	15	3791	
QEN71_RS16625	ankyrin repeat domain-containing protein	QEN71_16625	paras_003867	protein-codi	NZ_CP1252	chromosom	3686051	3686578	-	524	0	2	93	2	93	
QEN71_RS16630	hypothetical protein	QEN71_16630	paras_003868	protein-codi	NZ_CP1252	chromosom	3686797	3687261	-	465	0	13	3443	11	3278	
QEN71_RS16635	GNAT family N-acetyltransferase	QEN71_16635	paras_003869	protein-codi	NZ_CP1252	chromosom	3687366	3687893	-	524	0	12	3065	5	817	
QEN71_RS16640	XRE family transcriptional regulator	QEN71_16640	paras_003870	protein-codi	NZ_CP1252	chromosom	3687890	3688480	-	587	0	14	2946	10	2460	
QEN71_RS16645	penicillin acylase family protein	QEN71_16645	paras_003871	protein-codi	NZ_CP1252	chromosom	3688626	3691118	+	2493	0	49	12994	45	12458	
QEN71_RS16650	nucleotide pyrophosphohydrolase	QEN71_16650	paras_003872	protein-codi	NZ_CP1252	chromosom	3691147	3691527	+	381	0	8	709	5	463	
QEN71_RS16655	YceI family protein	QEN71_16655	paras_003873	protein-codi	NZ_CP1252	chromosom	3691630	3692208	-	579	0	13	2317	7	1791	
QEN71_RS16660	YceI family protein	QEN71_16660	paras_003874	protein-codi	NZ_CP1252	chromosom	3692245	3692808	-	564	0	8	901	8	901	
QEN71_RS16665	cytochrome b	QEN71_16665	paras_003875	protein-codi	NZ_CP1252	chromosom	3692851	3693399	-	549	0	20	4052	15	3223	
QEN71_RS16670	hypothetical protein	QEN71_16670	paras_003876	protein-codi	NZ_CP1252	chromosom	3693765	3694376	-	612	0	8	753	8	753	
QEN71_RS16675	selenium-binding family protein	QEN71_16675	paras_003877	protein-codi	NZ_CP1252	chromosom	3694385	3695779	-	1395	0	34	7886	26	5427	
QEN71_RS16680	helix-turn-helix transcriptional regulator	QEN71_16680	paras_003878	protein-codi	NZ_CP1252	chromosom	3696062	3696880	+	819	0	18	2607	18	2607	
QEN71_RS16685	MFS transporter	QEN71_16685	paras_003879	protein-codi	NZ_CP1252	chromosom	3697037	3698398	+	1362	0	28	6260	22	5612	
QEN71_RS16690	FAD-dependent oxidoreductase	QEN71_16690	paras_003880	protein-codi	NZ_CP1252	chromosom	3698422	3700116	+	1695	0	42	7106	35	6634	
QEN71_RS16695	DUF2783 domain-containing protein	QEN71_16695	paras_003881	protein-codi	NZ_CP1252	chromosom	3700144	3700323	+	180	0	6	568	6	568	
QEN71_RS16700	homoprotocatechuate degradation operon regulator	QEN71_16700	paras_003882	protein-codi	NZ_CP1252	chromosom	3700332	3700805	-	474	0	4	1968	4	1968	
QEN71_RS16705	helix-turn-helix domain-containing protein	QEN71_16705	paras_003883	protein-codi	NZ_CP1252	chromosom	3701098	3702060	+	963	0	15	4944	11	3782	
QEN71_RS16710	HD domain-containing protein	QEN71_16710	paras_003884	protein-codi	NZ_CP1252	chromosom	3702201	3702890	-	690	0	19	4380	17	3784	
QEN71_RS16715	LysR substrate-binding domain-containing protein	QEN71_16715	paras_003885	protein-codi	NZ_CP1252	chromosom	3703090	3704022	-	933	0	19	5107	13	4158	
QEN71_RS16720	transporter substrate-binding domain-containing pro	QEN71_16720	paras_003886	protein-codi	NZ_CP1252	chromosom	3704160	3704933	+	774	0	24	2337	16	875	
QEN71_RS16725	M14 family metallopeptidase	QEN71_16725	paras_003887	protein-codi	NZ_CP1252	chromosom	3704984	3706099	+	1116	0	34	10922	17	5486	
QEN71_RS16730	SDR family oxidoreductase	QEN71_16730	paras_003888	protein-codi	NZ_CP1252	chromosom	3706250	3707068	-	819	0	14	2352	11	1531	
QEN71_RS16735	GlxA family transcriptional regulator	QEN71_16735	paras_003889	protein-codi	NZ_CP1252	chromosom	3707196	3708134	+	939	0	16	3947	14	3615	
QEN71_RS16740	hypothetical protein	QEN71_16740	paras_003890	protein-codi	NZ_CP1252	chromosom	3708386	3709036	+	651	0	21	5467	19	5217	
QEN71_RS16745	YSC84-related protein	QEN71_16745	paras_003891	protein-codi	NZ_CP1252	chromosom	3709108	3709695	-	588	0	16	4549	14	4294	
QEN71_RS16750	flagellar motor protein MotB	QEN71_16750	paras_003893	protein-codi	NZ_CP1252	chromosom	3710030	3711013	-	984	0	20	6698	14	4963	
QEN71_RS16755	flagellar motor stator protein MotA	QEN71_16755	paras_003894	protein-codi	NZ_CP1252	chromosom	3711041	3711910	-	870	0	21	2617	16	2041	
QEN71_RS16760	Fic family protein	QEN71_16760	paras_003895	protein-codi	NZ_CP1252	chromosom	3712380	3713552	+	1173	0	29	7113	20	2870	
QEN71_RS16765	DUF2242 domain-containing protein	QEN71_16765	paras_003896	protein-codi	NZ_CP1252	chromosom	3713590	3714444	-	855	0	19	3116	19	3116	
QEN71_RS16770	hybrid sensor histidine kinase/response regulator	QEN71_16770	paras_003897	protein-codi	NZ_CP1252	chromosom	3714621	3715733	-	1109	0	9	1148	7	970	
QEN71_RS16775	chemotaxis protein CheB	QEN71_16775	paras_003898	protein-codi	NZ_CP1252	chromosom	3715730	3716365	-	632	0	8	1186	6	507	
QEN71_RS16780	CheR family methyltransferase	QEN71_16780	paras_003899	protein-codi	NZ_CP1252	chromosom	3716406	3717254	-	849	0	37	7034	28	5654	
QEN71_RS16785	response regulator	QEN71_16785	paras_003900	protein-codi	NZ_CP1252	chromosom	3717274	3720753	-	3480	0	43	6668	33	4486	
QEN71_RS16790	DUF4148 domain-containing protein	QEN71_16790	paras_003901	protein-codi	NZ_CP1252	chromosom	3721058	3721372	-	315	0	9	1068	6	838	
QEN71_RS16795	asparaginase	QEN71_16795	paras_003902	protein-codi	NZ_CP1252	chromosom	3721577	3722635	+	1055	0	24	3984	19	3444	
QEN71_RS16800	hypothetical protein	QEN71_16800	paras_003903	protein-codi	NZ_CP1252	chromosom	3722632	3722871	-	236	0	8	1668	6	1560	
QEN71_RS16805	FadR/GntR family transcriptional regulator	QEN71_16805	paras_003904	protein-codi	NZ_CP1252	chromosom	3723127	3723855	+	729	0	18	4339	16	4144	
QEN71_RS16810	IlvD/Edd family dehydratase	QEN71_16810	paras_003905	protein-codi	NZ_CP1252	chromosom	3723883	3725625	-	1743	99.0	26	3999	22	2934	

QEN71_RS16815	hypothetical protein	QEN71_16815	paras_003906	protein-codi	NZ_CP1252	chromosom	3725925	3726068	+	144	0	1	46	0	0	
QEN71_RS16820	Gfo/Idh/MocA family oxidoreductase	QEN71_16820	paras_003907	protein-codi	NZ_CP1252	chromosom	3726106	3727035	+	930	0	15	2355	10	1766	
QEN71_RS16825	arabinose ABC transporter substrate-binding protei	QEN71_16825	paras_003908	protein-codi	NZ_CP1252	chromosom	3727126	3728130	+	1005	0	10	715	7	593	
QEN71_RS16830	L-arabinose ABC transporter ATP-binding protein A	QEN71_16830	paras_003909	protein-codi	NZ_CP1252	chromosom	3728144	3729706	+	1563	0	20	2561	20	2561	
QEN71_RS16835	L-arabinose ABC transporter permease AraH	QEN71_16835	paras_003910	protein-codi	NZ_CP1252	chromosom	3729784	3730800	+	1017	0	13	1483	8	558	
QEN71_RS16840	M55 family metallopeptidase	QEN71_16840	paras_003911	protein-codi	NZ_CP1252	chromosom	3730841	3731665	-	821	0	11	1025	6	838	
QEN71_RS16845	P1 family peptidase	QEN71_16845	paras_003912	protein-codi	NZ_CP1252	chromosom	3731662	3732720	-	1051	0	13	1291	10	981	
QEN71_RS16850	glutathione ABC transporter permease GsiD	QEN71_16850	paras_003913	protein-codi	NZ_CP1252	chromosom	3732717	3733622	-	902	0	16	2206	16	2206	
QEN71_RS16855	glutathione ABC transporter permease GsiC	QEN71_16855	paras_003914	protein-codi	NZ_CP1252	chromosom	3733630	3734550	-	921	0	7	812	5	783	
QEN71_RS16860	glutathione ABC transporter substrate-binding prote	QEN71_16860	paras_003915	protein-codi	NZ_CP1252	chromosom	3734658	3736214	-	1557	0	28	2577	24	2196	
QEN71_RS16865	dipeptide ABC transporter ATP-binding protein	QEN71_16865	paras_003916	protein-codi	NZ_CP1252	chromosom	3736325	3738214	-	1890	0	17	1736	13	1612	
QEN71_RS16870	isoaspartyl peptidase/L-asparaginase	QEN71_16870	paras_003917	protein-codi	NZ_CP1252	chromosom	3738250	3739230	-	977	0	16	965	8	544	
QEN71_RS16875	MurR/RpiR family transcriptional regulator	QEN71_16875	paras_003918	protein-codi	NZ_CP1252	chromosom	3739227	3740177	-	947	0	4	136	2	91	
QEN71_RS16880	IclR family transcriptional regulator	QEN71_16880	paras_003919	protein-codi	NZ_CP1252	chromosom	3740404	3741327	+	924	0	18	4447	16	3851	
QEN71_RS16885	porin	QEN71_16885	paras_003920	protein-codi	NZ_CP1252	chromosom	3741401	3742474	-	1060	0	38	4869	32	4553	
QEN71_RS16890	hypothetical protein	QEN71_16890	paras_003921	protein-codi	NZ_CP1252	chromosom	3742461	3742844	+	370	0	9	1317	8	1143	
QEN71_RS16895	non-heme iron oxygenase ferredoxin subunit	QEN71_16895	paras_003922	protein-codi	NZ_CP1252	chromosom	3742919	3743236	+	318	0	8	2729	8	2729	
QEN71_RS16900	hypothetical protein	QEN71_16900	paras_003923	protein-codi	NZ_CP1252	chromosom	3743238	3743549	+	312	0	3	437	2	422	
QEN71_RS16905	aromatic ring-hydroxylating dioxygenase subunit alp	QEN71_16905	paras_003924	protein-codi	NZ_CP1252	chromosom	3743593	3744681	+	1081	0	21	3114	15	2413	
QEN71_RS16910	SDR family oxidoreductase	QEN71_16910	paras_003925	protein-codi	NZ_CP1252	chromosom	3744674	3745432	+	751	0	13	1231	13	1231	
QEN71_RS16915	cupin domain-containing protein	QEN71_16915	paras_003926	protein-codi	NZ_CP1252	chromosom	3745468	3746007	+	540	0	13	1138	9	426	
QEN71_RS16920	alpha/beta fold hydrolase	QEN71_16920	paras_003927	protein-codi	NZ_CP1252	chromosom	3746045	3746935	+	887	0	12	1882	6	1137	
QEN71_RS16925	IclR family transcriptional regulator	QEN71_16925	paras_003928	protein-codi	NZ_CP1252	chromosom	3746932	3747792	+	856	0	8	1185	6	608	
QEN71_RS16930	SDR family oxidoreductase	QEN71_16930	paras_003929	protein-codi	NZ_CP1252	chromosom	3747792	3748598	+	798	0	6	180	3	107	
QEN71_RS16935	thiamine pyrophosphate-binding protein	QEN71_16935	paras_003930	protein-codi	NZ_CP1252	chromosom	3748591	3750249	+	1651	0	17	2438	16	2213	
QEN71_RS16940	aspartate dehydrogenase	QEN71_16940	paras_003931	protein-codi	NZ_CP1252	chromosom	3750278	3751087	+	810	0	6	548	5	521	
QEN71_RS16945	aldehyde dehydrogenase	QEN71_16945	paras_003932	protein-codi	NZ_CP1252	chromosom	3751115	3752611	+	1497	0	24	1911	16	1008	
QEN71_RS16950	VOC family protein	QEN71_16950	paras_003933	protein-codi	NZ_CP1252	chromosom	3752625	3753587	+	959	0	14	1408	10	921	
QEN71_RS16955	FAD-dependent oxidoreductase	QEN71_16955	paras_003934	protein-codi	NZ_CP1252	chromosom	3753584	3754840	+	1253	0	20	1639	15	1449	
QEN71_RS16960	MFS transporter	QEN71_16960	paras_003935	protein-codi	NZ_CP1252	chromosom	3754866	3756269	+	1404	0	31	3828	24	3329	
QEN71_RS16965	SDR family oxidoreductase	QEN71_16965	paras_003936	protein-codi	NZ_CP1252	chromosom	3756537	3757286	-	750	0	19	2511	10	851	
QEN71_RS16970	GlxA family transcriptional regulator	QEN71_16970	paras_003937	protein-codi	NZ_CP1252	chromosom	3757365	3758324	-	960	0	16	1906	14	1853	
QEN71_RS16975	hypothetical protein	QEN71_16975	paras_003938	protein-codi	NZ_CP1252	chromosom	3758374	3758610	+	237	0	8	1668	6	684	
QEN71_RS16980	LysR family transcriptional regulator	QEN71_16980	paras_003939	protein-codi	NZ_CP1252	chromosom	3758675	3759601	+	927	0	11	1588	8	932	
QEN71_RS16985	CaiB/BaiF CoA-transferase family protein	QEN71_16985	paras_003940	protein-codi	NZ_CP1252	chromosom	3759712	3760905	+	1190	0	19	2519	16	1789	
QEN71_RS16990	hydroxymethylglutaryl-CoA lyase	QEN71_16990	paras_003941	protein-codi	NZ_CP1252	chromosom	3760902	3761849	+	944	0	12	2023	9	957	
QEN71_RS16995	MFS transporter	QEN71_16995	paras_003942	protein-codi	NZ_CP1252	chromosom	3761916	3763259	+	1344	0	43	8422	35	7200	
QEN71_RS17000	GntR family transcriptional regulator	QEN71_17000	paras_003943	protein-codi	NZ_CP1252	chromosom	3763327	3764052	-	726	0	12	2488	10	2476	
QEN71_RS17005	aminotransferase class V-fold PLP-dependent enzy	QEN71_17005	paras_003944	protein-codi	NZ_CP1252	chromosom	3764195	3765415	+	1221	0	19	1576	15	1196	
QEN71_RS17010	MFS transporter	QEN71_17010	paras_003945	protein-codi	NZ_CP1252	chromosom	3765666	3766952	+	1287	0	31	3065	22	2316	
QEN71_RS17015	2-keto-4-pentenoate hydratase	QEN71_17015	paras_003946	protein-codi	NZ_CP1252	chromosom	3766976	3767719	+	744	0	9	830	8	791	
QEN71_RS17020	FAD-binding and (Fe-S)-binding domain-containing	QEN71_17020	paras_003947	protein-codi	NZ_CP1252	chromosom	3767757	3770768	+	3012	0	56	5345	39	3517	
QEN71_RS17025	aminotransferase	QEN71_17025	paras_003948	protein-codi	NZ_CP1252	chromosom	3771360	3772484	-	1125	0	43	4499	33	3914	
QEN71_RS17030	AraC family transcriptional regulator	QEN71_17030	paras_003949	protein-codi	NZ_CP1252	chromosom	3772686	3773603	-	918	0	14	3211	13	3167	
QEN71_RS17035	EamA family transporter	QEN71_17035	paras_003950	protein-codi	NZ_CP1252	chromosom	3773774	3774673	+	900	0	21	4779	17	3772	
QEN71_RS17040	hypothetical protein	QEN71_17040	paras_003951	protein-codi	NZ_CP1252	chromosom	3774912	3777098	+	2187	0	90	31121	69	23346	
QEN71_RS17045	hypothetical protein	QEN71_17045	paras_003952	protein-codi	NZ_CP1252	chromosom	3777249	3777506	+	258	0	3	612	3	612	
QEN71_RS17050	LysE family translocator	QEN71_17050	paras_003953	protein-codi	NZ_CP1252	chromosom	3777609	3778208	+	600	0	15	4881	15	4881	
QEN71_RS17055	winged helix-turn-helix domain-containing protein	QEN71_17055	paras_003954	protein-codi	NZ_CP1252	chromosom	3778226	3779440	-	1215	0	33	9834	31	9651	
QEN71_RS17060	hypothetical protein	QEN71_17060	paras_003955	protein-codi	NZ_CP1252	chromosom	3779583	3779867	-	285	0	17	5457	17	5457	
QEN71_RS17065	FAD-dependent oxidoreductase	QEN71_17065	paras_003956	protein-codi	NZ_CP1252	chromosom	3780063	3781766	-	1704	0	35	10987	31	10360	
QEN71_RS17070	DedA family protein/thiosulfate sulfurtransferase Gl	QEN71_17070	paras_003957	protein-codi	NZ_CP1252	chromosom	3781821	3782810	-	990	0	26	12161	20	9299	
QEN71_RS17075	hypothetical protein	QEN71_17075	paras_003959	protein-codi	NZ_CP1252	chromosom	3783108	3783359	-	252	0	9	3085	5	1523	
QEN71_RS17080	methylated-DNA--[protein]-cysteine S-methyltransfe	QEN71_17080	paras_003960	protein-codi	NZ_CP1252	chromosom	3783762	3784283	+	522	0	12	3091	12	3091	
QEN71_RS17085	PAS domain-containing methyl-accepting chemotax	QEN71_17085	paras_003961	protein-codi	NZ_CP1252	chromosom	3784449	3786122	-	1674	0	32	8311	20	6500	
QEN71_RS17090	ATP-binding protein	QEN71_17090	paras_003962	protein-codi	NZ_CP1252	chromosom	3786496	3790026	+	3531	0	65	17295	43	9003	
QEN71_RS17095	porin	QEN71_17095	paras_003963	protein-codi	NZ_CP1252	chromosom	3790324	3791433	+	1110	0	61	23529	55	20455	
QEN71_RS17100	EAL domain-containing protein	QEN71_17100	paras_003964	protein-codi	NZ_CP1252	chromosom	3791459	3793783	-	2325	0	35	8571	29	7488	
QEN71_RS17105	acyl CoA:acetate/3-ketoacid CoA transferase	QEN71_17105	paras_003965	protein-codi	NZ_CP1252	chromosom	3794428	3796449	+	2022	0	33	7761	17	4276	
QEN71_RS17110	hypothetical protein	QEN71_17110	paras_003966	protein-codi	NZ_CP1252	chromosom	3796575	3796829	+	255	0	9	1547	7	1221	
QEN71_RS17115	DUF2442 domain-containing protein	QEN71_17115	paras_003967	protein-codi	NZ_CP1252	chromosom	3796932	3797171	+	240	0	3	39	1	6	
QEN71_RS17120	GntP family permease	QEN71_17120	paras_003968	protein-codi	NZ_CP1252	chromosom	3797569	3798972	-	1404	0	33	7132	25	5981	

QEN71_RS17125	sigma 54-interacting transcriptional regulator	QEN71_17125	paras_003969	protein-codi	NZ_CP1252	chromosom	3799342	3800802	+	1461	0	30	6324	22	5155	
QEN71_RS17130	BON domain-containing protein	QEN71_17130	paras_003970	protein-codi	NZ_CP1252	chromosom	3800884	3801420	+	537	0	15	4314	11	3935	
QEN71_RS17135	hypothetical protein	QEN71_17135	paras_003971	protein-codi	NZ_CP1252	chromosom	3801527	3801805	+	279	0	6	752	2	117	
QEN71_RS17140	hypothetical protein	QEN71_17140	paras_003972	protein-codi	NZ_CP1252	chromosom	3802267	3802470	+	204	0	4	391	2	114	
QEN71_RS17145	serine hydrolase domain-containing protein	QEN71_17145	paras_003973	protein-codi	NZ_CP1252	chromosom	3802512	3803711	+	1200	0	22	3460	15	1853	
QEN71_RS17150	H-NS histone family protein	QEN71_17150	paras_003974	protein-codi	NZ_CP1252	chromosom	3803835	3804125	+	291	0	9	2129	5	394	
QEN71_RS17155	formaldehyde-activating enzyme	QEN71_17155	paras_003975	protein-codi	NZ_CP1252	chromosom	3804694	3805212	+	519	0	9	977	4	581	
QEN71_RS17160	aldo/keto reductase	QEN71_17160	paras_003976	protein-codi	NZ_CP1252	chromosom	3805538	3806581	+	1040	0	27	5278	22	3926	
QEN71_RS17165	NAD-dependent succinate-semialdehyde dehydrog	QEN71_17165	paras_003977	protein-codi	NZ_CP1252	chromosom	3806578	3808017	+	1436	101.0	27	3997	13	1786	
QEN71_RS17170	MFS transporter	QEN71_17170	paras_003978	protein-codi	NZ_CP1252	chromosom	3808112	3809515	+	1404	0	14	2218	14	2218	
QEN71_RS17175	fimbrial protein	QEN71_17175	paras_003979	protein-codi	NZ_CP1252	chromosom	3809947	3810516	+	570	0	11	1281	5	357	
QEN71_RS17180	fimbria/pilus periplasmic chaperone	QEN71_17180	paras_003980	protein-codi	NZ_CP1252	chromosom	3810621	3811361	+	741	0	13	2785	11	2047	
QEN71_RS17185	fimbria/pilus outer membrane usher protein	QEN71_17185	paras_003981	protein-codi	NZ_CP1252	chromosom	3811592	3814180	+	2589	0	57	9175	47	6088	
QEN71_RS17190	fimbrial protein	QEN71_17190	paras_003982	protein-codi	NZ_CP1252	chromosom	3814251	3815258	+	1008	0	46	9703	32	7571	
QEN71_RS17195	fimbrial protein	QEN71_17195	paras_003983	protein-codi	NZ_CP1252	chromosom	3815335	3816351	+	1017	0	32	5722	25	3881	
QEN71_RS17200	peptide chain release factor 3	QEN71_17200	paras_003984	protein-codi	NZ_CP1252	chromosom	3816558	3818159	+	1602	0	36	8848	17	3635	
QEN71_RS17205	LysR family transcriptional regulator	QEN71_17205	paras_003985	protein-codi	NZ_CP1252	chromosom	3818798	3819667	-	870	0	25	3119	21	2322	
QEN71_RS17210	GNAT family N-acetyltransferase	QEN71_17210	paras_003986	protein-codi	NZ_CP1252	chromosom	3820026	3822449	+	2424	0	21	3306	14	2639	
QEN71_RS17215	hypothetical protein	QEN71_17215	paras_003987	protein-codi	NZ_CP1252	chromosom	3822480	3823022	-	543	0	3	729	3	729	
QEN71_RS17220	ureidoglycolate lyase	QEN71_17220	paras_003988	protein-codi	NZ_CP1252	chromosom	3823562	3824410	-	849	0	25	2748	20	1568	
QEN71_RS17225	SDR family oxidoreductase	QEN71_17225	paras_003989	protein-codi	NZ_CP1252	chromosom	3824468	3825211	-	744	0	14	3069	10	2309	
QEN71_RS17230	amidohydrolase family protein	QEN71_17230	paras_003990	protein-codi	NZ_CP1252	chromosom	3825309	3826139	-	831	0	19	2088	9	1133	
QEN71_RS17235	aldo/keto reductase	QEN71_17235	paras_003991	protein-codi	NZ_CP1252	chromosom	3826155	3827195	-	1041	0	22	2318	21	2297	
QEN71_RS17240	UxaA family hydrolase	QEN71_17240	paras_003992	protein-codi	NZ_CP1252	chromosom	3827224	3828531	-	1308	0	28	4327	20	2606	
QEN71_RS17245	UxaA family hydrolase	QEN71_17245	paras_003993	protein-codi	NZ_CP1252	chromosom	3828547	3828855	-	309	0	4	310	4	310	
QEN71_RS17250	ABC transporter permease	QEN71_17250	paras_003994	protein-codi	NZ_CP1252	chromosom	3828857	3829882	-	1015	0	10	1042	7	477	
QEN71_RS17255	sugar ABC transporter ATP-binding protein	QEN71_17255	paras_003995	protein-codi	NZ_CP1252	chromosom	3829872	3831398	-	1516	0	7	940	7	940	
QEN71_RS17260	sugar ABC transporter substrate-binding protein	QEN71_17260	paras_003996	protein-codi	NZ_CP1252	chromosom	3831407	3832456	-	1050	0	31	9208	28	7974	
QEN71_RS17265	IclR family transcriptional regulator	QEN71_17265	paras_003997	protein-codi	NZ_CP1252	chromosom	3832731	3833507	+	777	0	39	14882	29	9481	
QEN71_RS17270	PaaI family thioesterase	QEN71_17270	paras_003998	protein-codi	NZ_CP1252	chromosom	3833680	3834099	-	420	0	34	13082	26	10046	
QEN71_RS17275	methyl-accepting chemotaxis protein	QEN71_17275	paras_003999	protein-codi	NZ_CP1252	chromosom	3834351	3835961	+	1611	0	23	9472	18	8868	
QEN71_RS17280	AGE family epimerase/isomerase	QEN71_17280	paras_004000	protein-codi	NZ_CP1252	chromosom	3836053	3837147	-	1095	0	4	29	4	29	
QEN71_RS17285	MgtC/SapB family protein	QEN71_17285	paras_004001	protein-codi	NZ_CP1252	chromosom	3837280	3837966	+	687	0	9	5476	5	4843	
QEN71_RS17290	hypothetical protein	QEN71_17290	paras_004002	protein-codi	NZ_CP1252	chromosom	3838108	3839217	+	1110	0	11	2222	9	1797	
QEN71_RS17295	hypothetical protein	QEN71_17295	paras_004003	protein-codi	NZ_CP1252	chromosom	3839539	3839697	+	159	0	6	1285	1	168	
QEN71_RS17300	hypothetical protein	QEN71_17300	paras_004004	protein-codi	NZ_CP1252	chromosom	3840145	3840423	+	279	0	8	1256	3	110	
QEN71_RS17305	hypothetical protein	QEN71_17305	paras_004005	protein-codi	NZ_CP1252	chromosom	3840814	3841035	+	222	0	7	2931	6	2892	
QEN71_RS17310	flavin reductase family protein	QEN71_17310	paras_004006	protein-codi	NZ_CP1252	chromosom	3841353	3841967	+	615	0	8	1103	4	561	
QEN71_RS17315	polyhydroxyalkanoate depolymerase	QEN71_17315	paras_004007	protein-codi	NZ_CP1252	chromosom	3841988	3843370	-	1383	0	21	4178	17	4039	
QEN71_RS17320	glutathione S-transferase family protein	QEN71_17320	paras_004008	protein-codi	NZ_CP1252	chromosom	3843594	3844244	+	651	0	24	6068	21	5257	
QEN71_RS17325	methyl-accepting chemotaxis protein	QEN71_17325	paras_004009	protein-codi	NZ_CP1252	chromosom	3844283	3846253	-	1971	0	24	3995	16	3077	
QEN71_RS17330	heavy metal translocating P-type ATPase	QEN71_17330	paras_004010	protein-codi	NZ_CP1252	chromosom	3846385	3848907	-	2523	0	39	6356	28	4307	
QEN71_RS17335	FMN-binding negative transcriptional regulator	QEN71_17335	paras_004011	protein-codi	NZ_CP1252	chromosom	3849034	3849678	-	645	0	16	16413	12	10462	
QEN71_RS17340	DUF1214 domain-containing protein	QEN71_17340	paras_004012	protein-codi	NZ_CP1252	chromosom	3849918	3851330	+	1413	0	29	9974	24	8157	
QEN71_RS17345	DUF748 domain-containing protein	QEN71_17345	paras_004013	protein-codi	NZ_CP1252	chromosom	3851572	3855378	+	3807	0	59	10281	40	7091	
QEN71_RS17350	septal ring lytic transglycosylase RlpA family protein	QEN71_17350	paras_004014	protein-codi	NZ_CP1252	chromosom	3855615	3856214	+	600	0	13	3725	13	3725	
QEN71_RS17355	PLP-dependent aminotransferase family protein	QEN71_17355	paras_004015	protein-codi	NZ_CP1252	chromosom	3856243	3857820	-	1578	0	37	7340	31	6388	
QEN71_RS17360	ABC transporter ATP-binding protein	QEN71_17360	paras_004016	protein-codi	NZ_CP1252	chromosom	3858004	3859095	+	1092	0	20	4522	13	1748	
QEN71_RS17365	ABC transporter substrate-binding protein	QEN71_17365	paras_004017	protein-codi	NZ_CP1252	chromosom	3859154	3860206	+	1053	0	29	4137	27	3765	
QEN71_RS17370	ABC transporter permease	QEN71_17370	paras_004018	protein-codi	NZ_CP1252	chromosom	3860313	3861584	+	1272	0	32	4992	25	4210	
QEN71_RS17375	ABC transporter permease	QEN71_17375	paras_004019	protein-codi	NZ_CP1252	chromosom	3861595	3862434	+	840	0	20	4216	18	3155	
QEN71_RS17380	GNAT family protein	QEN71_17380	paras_004020	protein-codi	NZ_CP1252	chromosom	3862556	3863131	+	576	0	12	2004	10	1770	
QEN71_RS17385	GlsB/YeaQ/YmgE family stress response membrane	QEN71_17385	paras_004021	protein-codi	NZ_CP1252	chromosom	3863197	3863448	+	252	0	3	656	3	656	
QEN71_RS17390	efflux transporter outer membrane subunit	QEN71_17390	paras_004022	protein-codi	NZ_CP1252	chromosom	3863503	3864957	-	1455	0	21	2236	16	1859	
QEN71_RS17395	ABC transporter permease	QEN71_17395	paras_004023	protein-codi	NZ_CP1252	chromosom	3864969	3866105	-	1133	0	24	3461	21	3216	
QEN71_RS17400	ABC transporter ATP-binding protein	QEN71_17400	paras_004024	protein-codi	NZ_CP1252	chromosom	3866102	3867058	-	949	0	20	1882	17	1271	
QEN71_RS17405	HlyD family efflux transporter periplasmic adaptor su	QEN71_17405	paras_004025	protein-codi	NZ_CP1252	chromosom	3867055	3868029	-	967	0	13	1600	13	1600	
QEN71_RS17410	TetR/AcrR family transcriptional regulator	QEN71_17410	paras_004026	protein-codi	NZ_CP1252	chromosom	3868026	3868745	-	716	0	8	502	6	462	
QEN71_RS17415	response regulator transcription factor	QEN71_17415	paras_004027	protein-codi	NZ_CP1252	chromosom	3869091	3869729	+	635	0	15	2098	10	1419	
QEN71_RS17420	Hpt domain-containing protein	QEN71_17420	paras_004028	protein-codi	NZ_CP1252	chromosom	3869726	3870103	+	374	0	5	531	5	531	
QEN71_RS17425	hybrid sensor histidine kinase/response regulator	QEN71_17425	paras_004029	protein-codi	NZ_CP1252	chromosom	3870333	3872345	-	2013	0	16	2162	14	2060	
QEN71_RS17430	response regulator transcription factor	QEN71_17430	paras_004030	protein-codi	NZ_CP1252	chromosom	3872358	3872933	-	576	0	5	135	5	135	

QEN71_RS17435	response regulator transcription factor	QEN71_17435	paras_004031	protein-codi	NZ_CP1252	chromosom	3873176	3873760	+	585	0	4	107	4	107	
QEN71_RS17440	Hpt domain-containing protein	QEN71_17440	paras_004032	protein-codi	NZ_CP1252	chromosom	3873780	3874079	+	300	0	3	53	3	53	
QEN71_RS17445	OmpA family protein	QEN71_17445	paras_004033	protein-codi	NZ_CP1252	chromosom	3874107	3875168	-	1062	0	16	1177	16	1177	
QEN71_RS17450	ESPR-type extended signal peptide-containing prot	QEN71_17450	paras_004034	protein-codi	NZ_CP1252	chromosom	3875234	3884626	-	9393	0	157	20411	113	12578	
QEN71_RS17455	pilus assembly protein	QEN71_17455	paras_004035	protein-codi	NZ_CP1252	chromosom	3885067	3885354	+	288	0	9	1400	6	1072	
QEN71_RS17460	pilus assembly protein TadG-related protein	QEN71_17460	paras_004036	protein-codi	NZ_CP1252	chromosom	3885417	3886910	+	1494	0	34	5463	29	4399	
QEN71_RS17465	hypothetical protein	QEN71_17465	paras_004037	protein-codi	NZ_CP1252	chromosom	3886919	3887719	+	801	0	19	1348	13	902	
QEN71_RS17470	hypothetical protein	QEN71_17470	paras_004038	protein-codi	NZ_CP1252	chromosom	3887752	3888417	+	646	0	4	167	4	167	
QEN71_RS17475	hypothetical protein	QEN71_17475	paras_004039	protein-codi	NZ_CP1252	chromosom	3888398	3888595	+	178	0	5	130	2	36	
QEN71_RS17480	Flp pilus assembly protein CpaB	QEN71_17480	paras_004040	protein-codi	NZ_CP1252	chromosom	3888615	3889601	+	987	0	6	212	3	17	
QEN71_RS17485	pilus assembly protein N-terminal domain-containin	QEN71_17485	paras_004041	protein-codi	NZ_CP1252	chromosom	3889612	3890952	+	1341	0	12	769	8	675	
QEN71_RS17490	ATPase, T2SS/T4P/T4SS family	QEN71_17490	paras_004042	protein-codi	NZ_CP1252	chromosom	3890961	3892655	+	1695	0	16	1239	10	601	
QEN71_RS17495	type II secretion system F family protein	QEN71_17495	paras_004043	protein-codi	NZ_CP1252	chromosom	3892666	3893508	+	843	0	12	817	4	266	
QEN71_RS17500	type II secretion system F family protein	QEN71_17500	paras_004044	protein-codi	NZ_CP1252	chromosom	3893515	3894393	+	875	0	10	331	8	238	
QEN71_RS17505	DUF192 domain-containing protein	QEN71_17505	paras_004045	protein-codi	NZ_CP1252	chromosom	3894390	3894740	+	347	0	0	0	0	0	TRUE
QEN71_RS17510	pilus assembly protein	QEN71_17510	paras_004046	protein-codi	NZ_CP1252	chromosom	3894782	3895492	+	710	0	12	1685	11	1617	
QEN71_RS17515	lytic transglycosylase domain-containing protein	QEN71_17515	paras_004047	protein-codi	NZ_CP1252	chromosom	3895492	3896055	+	563	0	13	1942	10	1222	
QEN71_RS17520	type VI secretion system tube protein Hcp	QEN71_17520	paras_004048	protein-codi	NZ_CP1252	chromosom	3896314	3896790	+	477	0	32	5634	26	4777	
QEN71_RS17525	hypothetical protein	QEN71_17525	paras_004049	protein-codi	NZ_CP1252	chromosom	3896792	3897430	+	635	0	39	6891	30	5625	
QEN71_RS17530	hypothetical protein	QEN71_17530	paras_004050	protein-codi	NZ_CP1252	chromosom	3897427	3898014	+	584	0	21	2175	16	1797	
QEN71_RS17535	aconitate hydratase AcnA	QEN71_17535	paras_004051	protein-codi	NZ_CP1252	chromosom	3898180	3900897	-	2718	0	53	5266	41	4485	
QEN71_RS17540	bifunctional 2-methylcitrate dehydratase/aconitate h	QEN71_17540	paras_004052	protein-codi	NZ_CP1252	chromosom	3900946	3902397	-	1452	0	43	8211	27	5129	
QEN71_RS17545	hypothetical protein	QEN71_17545	paras_004053	protein-codi	NZ_CP1252	chromosom	3902422	3902907	-	486	0	6	808	3	747	
QEN71_RS17550	2-methylcitrate synthase	QEN71_17550	paras_004054	protein-codi	NZ_CP1252	chromosom	3903010	3904185	-	1176	352.0	26	4617	16	3256	
QEN71_RS17555	methylisocitrate lyase	QEN71_17555	paras_004055	protein-codi	NZ_CP1252	chromosom	3904211	3905125	-	911	0	23	7971	15	5858	
QEN71_RS17560	aldolase/citrate lyase family protein	QEN71_17560	paras_004056	protein-codi	NZ_CP1252	chromosom	3905122	3906144	-	1019	0	10	2742	8	2383	
QEN71_RS17565	malate dehydrogenase	QEN71_17565	paras_004057	protein-codi	NZ_CP1252	chromosom	3906365	3907348	-	984	0	11	180	8	75	
QEN71_RS17570	GntR family transcriptional regulator	QEN71_17570	paras_004058	protein-codi	NZ_CP1252	chromosom	3907908	3908708	+	801	0	37	12520	25	6952	
QEN71_RS17575	succinate dehydrogenase, cytochrome b556 subuni	QEN71_17575	paras_004059	protein-codi	NZ_CP1252	chromosom	3908861	3909277	+	417	697.0	11	2985	9	2754	
QEN71_RS17580	succinate dehydrogenase, hydrophobic membrane	QEN71_17580	paras_004060	protein-codi	NZ_CP1252	chromosom	3909281	3909649	+	369	731.0	17	4535	12	3587	
QEN71_RS17585	succinate dehydrogenase flavoprotein subunit	QEN71_17585	paras_004061	protein-codi	NZ_CP1252	chromosom	3909654	3911429	+	1776	3173.0	25	6354	17	5339	
QEN71_RS17590	succinate dehydrogenase iron-sulfur subunit	QEN71_17590	paras_004062	protein-codi	NZ_CP1252	chromosom	3911458	3912162	+	705	688.0	16	2197	8	1242	
QEN71_RS17595	succinate dehydrogenase assembly factor 2	QEN71_17595	paras_004063	protein-codi	NZ_CP1252	chromosom	3912164	3912436	+	273	523.0	5	882	4	349	
QEN71_RS17600	citrate synthase	QEN71_17600	paras_004064	protein-codi	NZ_CP1252	chromosom	3912518	3913819	+	1302	0	42	4607	29	3862	
QEN71_RS17605	3-isopropylmalate dehydratase large subunit	QEN71_17605	paras_004065	protein-codi	NZ_CP1252	chromosom	3914082	3915491	+	1410	0	21	4394	13	3607	
QEN71_RS17610	entericidin A/B family lipoprotein	QEN71_17610	paras_004066	protein-codi	NZ_CP1252	chromosom	3915499	3915651	+	153	0	3	527	0	0	
QEN71_RS17615	3-isopropylmalate dehydratase small subunit	QEN71_17615	paras_004067	protein-codi	NZ_CP1252	chromosom	3915742	3916395	+	654	0	16	2738	10	2026	
QEN71_RS17620	3-isopropylmalate dehydrogenase	QEN71_17620	paras_004068	protein-codi	NZ_CP1252	chromosom	3916444	3917511	+	1068	0	26	7464	21	6423	
QEN71_RS17625	aspartate-semialdehyde dehydrogenase	QEN71_17625	paras_004069	protein-codi	NZ_CP1252	chromosom	3917809	3918930	+	1122	0	6	1137	1	7	
QEN71_RS17630	FimV/HubP family polar landmark protein	QEN71_17630	paras_004070	protein-codi	NZ_CP1252	chromosom	3919074	3921398	+	2324	0	21	5038	11	2991	
QEN71_RS17635	tRNA pseudouridine(38-40) synthase TruA	QEN71_17635	paras_004071	protein-codi	NZ_CP1252	chromosom	3921398	3922201	+	799	0	22	5503	18	5323	
QEN71_RS17640	phosphoribosylanthranilate isomerase	QEN71_17640	paras_004072	protein-codi	NZ_CP1252	chromosom	3922198	3922905	+	704	0	15	4540	15	4540	
QEN71_RS17645	tryptophan synthase subunit beta	QEN71_17645	paras_004073	protein-codi	NZ_CP1252	chromosom	3922966	3924159	+	1194	0	27	5274	19	4836	
QEN71_RS17650	site-specific DNA-methyltransferase	QEN71_17650	paras_004074	protein-codi	NZ_CP1252	chromosom	3924171	3925091	+	921	0	21	2186	21	2186	
QEN71_RS17655	tryptophan synthase subunit alpha	QEN71_17655	paras_004075	protein-codi	NZ_CP1252	chromosom	3925124	3925939	+	816	0	18	6653	16	6460	
QEN71_RS17660	acetyl-CoA carboxylase, carboxyltransferase subun	QEN71_17660	paras_004076	protein-codi	NZ_CP1252	chromosom	3926022	3926894	+	873	0	0	0	0	0	TRUE
QEN71_RS17665	bifunctional tetrahydrofolate synthase/dihydrofolate	QEN71_17665	paras_004077	protein-codi	NZ_CP1252	chromosom	3927018	3928328	+	1311	0	3	614	1	2	TRUE
QEN71_RS17670	SPOR domain-containing protein	QEN71_17670	paras_004078	protein-codi	NZ_CP1252	chromosom	3928356	3929294	+	939	0	10	4120	7	3578	
QEN71_RS17675	CvpA family protein	QEN71_17675	paras_004079	protein-codi	NZ_CP1252	chromosom	3929302	3929796	+	495	0	18	5610	10	2694	
QEN71_RS17680	amidophosphoribosyltransferase	QEN71_17680	paras_004080	protein-codi	NZ_CP1252	chromosom	3929932	3931482	+	1551	0	39	13122	25	6129	
QEN71_RS17685	O-succinylhomoserine sulfhydrylase	QEN71_17685	paras_004081	protein-codi	NZ_CP1252	chromosom	3931611	3932795	+	1185	0	37	12461	25	7879	
QEN71_RS17690	AraC family transcriptional regulator	QEN71_17690	paras_004082	protein-codi	NZ_CP1252	chromosom	3933462	3934229	-	768	0	21	5660	16	1837	
QEN71_RS17695	FKBP-type peptidyl-prolyl cis-trans isomerase	QEN71_17695	paras_004083	protein-codi	NZ_CP1252	chromosom	3934261	3934602	-	342	0	11	2778	7	1353	
QEN71_RS17700	HAMP domain-containing sensor histidine kinase	QEN71_17700	paras_004084	protein-codi	NZ_CP1252	chromosom	3934727	3935527	+	801	0	10	4269	7	3373	
QEN71_RS17705	hemolysin family protein	QEN71_17705	paras_004085	protein-codi	NZ_CP1252	chromosom	3935643	3936986	+	1344	0	25	6076	23	5804	
QEN71_RS17710	malto-oligosyltrehalose synthase	QEN71_17710	paras_004086	protein-codi	NZ_CP1252	chromosom	3937011	3939845	-	2831	0	54	8986	47	8216	
QEN71_RS17715	4-alpha-glucanotransferase	QEN71_17715	paras_004087	protein-codi	NZ_CP1252	chromosom	3939842	3942022	-	2177	0	25	5009	21	4546	
QEN71_RS17720	malto-oligosyltrehalose trehalohydrolase	QEN71_17720	paras_004088	protein-codi	NZ_CP1252	chromosom	3942028	3943935	-	1908	0	58	9794	53	9123	
QEN71_RS17725	glycogen debranching protein GlgX	QEN71_17725	paras_004089	protein-codi	NZ_CP1252	chromosom	3944020	3946233	-	2214	0	50	12194	42	10813	
QEN71_RS17730	1,4-alpha-glucan branching protein GlgB	QEN71_17730	paras_004090	protein-codi	NZ_CP1252	chromosom	3947071	3949296	-	2218	0	57	12784	43	9066	
QEN71_RS17735	maltose alpha-D-glucosyltransferase	QEN71_17735	paras_004091	protein-codi	NZ_CP1252	chromosom	3949289	3952753	-	3453	0	67	14492	49	10594	
QEN71_RS17740	DUF3416 domain-containing protein	QEN71_17740	paras_004092	protein-codi	NZ_CP1252	chromosom	3952750	3956193	-	3440	0	52	12211	42	11253	

QEN71_RS17745	DUF1345 domain-containing protein	QEN71_17745	paras_004093	protein-codi	NZ_CP1252	chromosom	3956756	3957406	-	651	0	24	6054	16	5277	
QEN71_RS17750	sugar dehydrogenase complex small subunit	QEN71_17750	paras_004094	protein-codi	NZ_CP1252	chromosom	3957798	3958373	+	576	0	13	1776	7	990	
QEN71_RS17755	GMC family oxidoreductase	QEN71_17755	paras_004095	protein-codi	NZ_CP1252	chromosom	3958458	3960059	+	1602	0	43	12360	41	12224	
QEN71_RS17760	cytochrome c	QEN71_17760	paras_004096	protein-codi	NZ_CP1252	chromosom	3960062	3961513	+	1452	0	54	15218	47	12429	
QEN71_RS17765	metallophosphoesterase	QEN71_17765	paras_004097	protein-codi	NZ_CP1252	chromosom	3962234	3962971	+	738	0	11	1563	8	1282	
QEN71_RS17770	RES family NAD+ phosphorylase	QEN71_17770	paras_004098	protein-codi	NZ_CP1252	chromosom	3962992	3963693	-	698	0	26	6617	19	4612	
QEN71_RS17775	MbcA/ParS/Xre antitoxin family protein	QEN71_17775	paras_004099	protein-codi	NZ_CP1252	chromosom	3963690	3964127	-	434	0	14	5672	9	4265	
QEN71_RS17780	hypothetical protein	QEN71_17780	paras_004100	protein-codi	NZ_CP1252	chromosom	3964232	3964618	+	387	0	7	1567	3	1050	
QEN71_RS17785	LysR family transcriptional regulator	QEN71_17785	paras_004101	protein-codi	NZ_CP1252	chromosom	3964638	3965489	-	852	0	4	616	4	616	
QEN71_RS17790	tautomerase family protein	QEN71_17790	paras_004102	protein-codi	NZ_CP1252	chromosom	3965588	3965971	+	384	0	10	5601	8	5259	
QEN71_RS17795	TOBE domain-containing protein	QEN71_17795	paras_004103	protein-codi	NZ_CP1252	chromosom	3966057	3966887	+	831	0	7	2227	6	2166	
QEN71_RS17800	ATP-binding cassette domain-containing protein	QEN71_17800	paras_004104	protein-codi	NZ_CP1252	chromosom	3966896	3967597	-	702	0	11	3796	9	1877	
QEN71_RS17805	molybdate ABC transporter permease subunit	QEN71_17805	paras_004105	protein-codi	NZ_CP1252	chromosom	3967599	3968273	-	675	0	10	3003	8	2706	
QEN71_RS17810	molybdate ABC transporter substrate-binding protei	QEN71_17810	paras_004106	protein-codi	NZ_CP1252	chromosom	3968307	3969086	-	780	0	15	3165	11	2442	
QEN71_RS17815	phosphatase PAP2 family protein	QEN71_17815	paras_004107	protein-codi	NZ_CP1252	chromosom	3969201	3969914	-	714	0	13	2595	7	867	
QEN71_RS17820	PHB depolymerase family esterase	QEN71_17820	paras_004108	protein-codi	NZ_CP1252	chromosom	3970197	3971387	+	1191	0	22	10366	21	10275	
QEN71_RS17825	DUF3563 family protein	QEN71_17825	paras_004109	protein-codi	NZ_CP1252	chromosom	3971494	3971670	+	177	0	12	3612	8	1465	
QEN71_RS17830	deoxyribodipyrimidine photo-lyase	QEN71_17830	paras_004110	protein-codi	NZ_CP1252	chromosom	3971713	3973227	-	1515	0	35	7795	27	5934	
QEN71_RS17835	organic hydroperoxide resistance protein	QEN71_17835	paras_004111	protein-codi	NZ_CP1252	chromosom	3973377	3973796	-	420	0	13	3185	3	95	
QEN71_RS17840	MarR family transcriptional regulator	QEN71_17840	paras_004112	protein-codi	NZ_CP1252	chromosom	3973952	3974407	-	456	0	18	4671	15	3993	
QEN71_RS17845	BPSS1780 family membrane protein	QEN71_17845	paras_004113	protein-codi	NZ_CP1252	chromosom	3974575	3975390	-	816	0	22	5306	18	4364	
QEN71_RS17850	homoserine kinase	QEN71_17850	paras_004114	protein-codi	NZ_CP1252	chromosom	3975404	3976375	-	972	0	19	1973	11	628	
QEN71_RS17855	hypothetical protein	QEN71_17855	paras_004115	protein-codi	NZ_CP1252	chromosom	3976519	3976923	-	405	0	14	2438	12	1780	
QEN71_RS17860	AMP nucleosidase	QEN71_17860	paras_004116	protein-codi	NZ_CP1252	chromosom	3977143	3978669	+	1527	0	53	16352	40	12565	
QEN71_RS17865	chromate transporter	QEN71_17865	paras_004117	protein-codi	NZ_CP1252	chromosom	3978685	3979215	-	527	0	6	1895	1	11	
QEN71_RS17870	chromate transporter	QEN71_17870	paras_004118	protein-codi	NZ_CP1252	chromosom	3979212	3979844	-	629	0	5	1230	5	1230	
QEN71_RS17875	transcriptional regulator GcvA	QEN71_17875	paras_004119	protein-codi	NZ_CP1252	chromosom	3979943	3980839	+	897	0	15	2086	11	1534	
QEN71_RS17880	TIGR00730 family Rossman fold protein	QEN71_17880	paras_004120	protein-codi	NZ_CP1252	chromosom	3980856	3981599	-	744	0	17	5417	10	3180	
QEN71_RS17885	DNA polymerase I	QEN71_17885	paras_004121	protein-codi	NZ_CP1252	chromosom	3981601	3984342	+	2742	0	55	5315	42	4173	
QEN71_RS17890	NAD(P)/FAD-dependent oxidoreductase	QEN71_17890	paras_004122	protein-codi	NZ_CP1252	chromosom	3984601	3985947	+	1347	0	27	5614	16	1563	
QEN71_RS17895	dienelactone hydrolase family protein	QEN71_17895	paras_004123	protein-codi	NZ_CP1252	chromosom	3986051	3986923	+	873	0	31	7026	28	6334	
QEN71_RS17900	hypothetical protein	QEN71_17900	paras_004124	protein-codi	NZ_CP1252	chromosom	3986940	3987137	-	198	205.0	5	409	3	228	
QEN71_RS17905	FadR/GntR family transcriptional regulator	QEN71_17905	paras_004125	protein-codi	NZ_CP1252	chromosom	3987664	3988368	+	705	0	18	5338	14	2698	
QEN71_RS17910	NAD(P)-dependent oxidoreductase	QEN71_17910	paras_004126	protein-codi	NZ_CP1252	chromosom	3988418	3989308	+	891	0	10	1870	7	357	
QEN71_RS17915	four-carbon acid sugar kinase family protein	QEN71_17915	paras_004127	protein-codi	NZ_CP1252	chromosom	3989326	3990636	+	1307	0	13	1666	13	1666	
QEN71_RS17920	aldolase	QEN71_17920	paras_004128	protein-codi	NZ_CP1252	chromosom	3990633	3991289	+	653	0	17	1212	14	1132	
QEN71_RS17925	MFS transporter	QEN71_17925	paras_004129	protein-codi	NZ_CP1252	chromosom	3991392	3992729	+	1338	0	26	2924	15	1156	
QEN71_RS17930	hydroxypyruvate isomerase family protein	QEN71_17930	paras_004130	protein-codi	NZ_CP1252	chromosom	3992754	3993542	+	789	0	19	2193	14	1490	
QEN71_RS17935	SDR family oxidoreductase	QEN71_17935	paras_004131	protein-codi	NZ_CP1252	chromosom	3993598	3994587	+	990	0	10	1137	7	867	
QEN71_RS17940	hypothetical protein	QEN71_17940	paras_004132	protein-codi	NZ_CP1252	chromosom	3994682	3994939	+	258	0	10	984	9	978	
QEN71_RS17945	MBL fold metallo-hydrolase	QEN71_17945	paras_004133	protein-codi	NZ_CP1252	chromosom	3994950	3995825	-	876	0	20	2082	14	1764	
QEN71_RS17950	MFS transporter	QEN71_17950	paras_004134	protein-codi	NZ_CP1252	chromosom	3995865	3997226	-	1362	0	36	4414	27	3037	
QEN71_RS17955	succinylglutamate desuccinylase/aspartoacylase fa	QEN71_17955	paras_004135	protein-codi	NZ_CP1252	chromosom	3997265	3998281	-	1017	0	17	2574	17	2574	
QEN71_RS17960	IS4 family transposase	QEN71_17960	paras_004136	protein-codi	NZ_CP1252	chromosom	3998367	3999713	-	1347	2667.0	7	2089	3	653	
QEN71_RS17965	LysR family transcriptional regulator	QEN71_17965	paras_004137	protein-codi	NZ_CP1252	chromosom	3999851	4000726	-	876	0	21	10223	16	7204	
QEN71_RS17970	SUMF1/EgtB/PvdO family nonheme iron enzyme	QEN71_17970	paras_004138	protein-codi	NZ_CP1252	chromosom	4000866	4002158	-	1293	0	38	17260	24	10501	
QEN71_RS17975	helix-turn-helix domain-containing protein	QEN71_17975	paras_004139	protein-codi	NZ_CP1252	chromosom	4002320	4003099	-	780	0	10	3522	10	3522	
QEN71_RS17980	universal stress protein	QEN71_17980	paras_004140	protein-codi	NZ_CP1252	chromosom	4003234	4003710	-	477	0	18	8260	11	3998	
QEN71_RS17985	FadR/GntR family transcriptional regulator	QEN71_17985	paras_004141	protein-codi	NZ_CP1252	chromosom	4004023	4004712	+	690	0	8	2561	5	1631	
QEN71_RS17990	MFS transporter	QEN71_17990	paras_004142	protein-codi	NZ_CP1252	chromosom	4004860	4006368	+	1509	0	30	12738	24	9599	
QEN71_RS17995	acyl-homoserine-lactone synthase	QEN71_17995	paras_004143	protein-codi	NZ_CP1252	chromosom	4006401	4006988	-	588	0	20	14149	16	13268	
QEN71_RS18000	helix-turn-helix transcriptional regulator	QEN71_18000	paras_004144	protein-codi	NZ_CP1252	chromosom	4007132	4007464	+	316	0	6	1608	6	1608	
QEN71_RS18005	LuxR family transcriptional regulator	QEN71_18005	paras_004145	protein-codi	NZ_CP1252	chromosom	4007448	4008152	-	688	0	11	5537	9	4912	
QEN71_RS18010	aldehyde dehydrogenase (NADP(+))	QEN71_18010	paras_004146	protein-codi	NZ_CP1252	chromosom	4009008	4010588	+	1581	0	33	10004	32	9939	
QEN71_RS18015	SMP-30/gluconolactonase/LRE family protein	QEN71_18015	paras_004147	protein-codi	NZ_CP1252	chromosom	4010700	4011650	+	951	0	35	16084	31	15361	
QEN71_RS18020	extracellular solute-binding protein	QEN71_18020	paras_004148	protein-codi	NZ_CP1252	chromosom	4011913	4013091	+	1179	0	38	9624	32	7110	
QEN71_RS18025	ABC transporter ATP-binding protein	QEN71_18025	paras_004149	protein-codi	NZ_CP1252	chromosom	4013244	4014323	+	1076	0	23	5806	19	5474	
QEN71_RS18030	sugar ABC transporter permease	QEN71_18030	paras_004150	protein-codi	NZ_CP1252	chromosom	4014320	4015207	+	883	0	24	4156	20	3501	
QEN71_RS18035	ABC transporter permease	QEN71_18035	paras_004151	protein-codi	NZ_CP1252	chromosom	4015207	4016112	+	905	0	31	8894	28	8747	
QEN71_RS18040	L-arabinonate dehydratase	QEN71_18040	paras_004152	protein-codi	NZ_CP1252	chromosom	4016121	4017881	+	1761	0	28	4095	19	3377	
QEN71_RS18045	short chain dehydrogenase	QEN71_18045	paras_004153	protein-codi	NZ_CP1252	chromosom	4017945	4018550	-	606	0	10	3319	5	2027	
QEN71_RS18050	DUF3005 domain-containing protein	QEN71_18050	paras_004154	protein-codi	NZ_CP1252	chromosom	4018742	4019194	-	453	0	4	1235	2	772	

QEN71_RS18055	hypothetical protein	QEN71_18055	paras_004155	protein-codi	NZ_CP1252	chromosom	4019481	4019714	+	234	0	10	5234	6	4382	
QEN71_RS18060	serine O-acetyltransferase EpsC	QEN71_18060	paras_004156	protein-codi	NZ_CP1252	chromosom	4019874	4020806	+	933	0	20	3122	18	2568	
QEN71_RS18065	AraC family transcriptional regulator	QEN71_18065	paras_004157	protein-codi	NZ_CP1252	chromosom	4020846	4021673	+	828	0	5	779	4	562	
QEN71_RS18070	DMT family transporter	QEN71_18070	paras_004158	protein-codi	NZ_CP1252	chromosom	4021774	4022661	+	888	0	25	2965	17	2211	
QEN71_RS18075	GTP cyclohydrolase I FolE	QEN71_18075	paras_004159	protein-codi	NZ_CP1252	chromosom	4023323	4023952	-	630	0	31	5880	17	4016	
QEN71_RS18080	LysR family transcriptional regulator	QEN71_18080	paras_004160	protein-codi	NZ_CP1252	chromosom	4024058	4024981	-	924	0	10	1630	9	1584	
QEN71_RS18085	4-hydroxybenzoate 3-monooxygenase	QEN71_18085	paras_004161	protein-codi	NZ_CP1252	chromosom	4025134	4026312	+	1179	0	28	4604	20	3348	
QEN71_RS18090	3-oxoacid CoA-transferase subunit A	QEN71_18090	paras_004162	protein-codi	NZ_CP1252	chromosom	4026619	4027320	+	702	0	11	2938	11	2938	
QEN71_RS18095	3-oxoacid CoA-transferase subunit B	QEN71_18095	paras_004163	protein-codi	NZ_CP1252	chromosom	4027332	4027991	+	660	0	14	2444	12	2009	
QEN71_RS18100	hypothetical protein	QEN71_18100	paras_004164	protein-codi	NZ_CP1252	chromosom	4028019	4028201	-	121	0	0	0	0	0	
QEN71_RS18105	3-carboxy-cis,cis-muconate cycloisomerase	QEN71_18105	paras_004165	protein-codi	NZ_CP1252	chromosom	4028140	4029504	+	1303	0	5	760	3	348	
QEN71_RS18110	3-oxoadipate enol-lactonase	QEN71_18110	paras_004166	protein-codi	NZ_CP1252	chromosom	4029526	4030317	+	788	0	20	3002	16	2802	
QEN71_RS18115	4-carboxymuconolactone decarboxylase	QEN71_18115	paras_004167	protein-codi	NZ_CP1252	chromosom	4030314	4030706	+	389	0	19	2473	13	2190	
QEN71_RS18120	IS481 family transposase	QEN71_18120	paras_004168	protein-codi	NZ_CP1252	chromosom	4031336	4032475	-	1140	2266.0	4	1959	2	741	
QEN71_RS18125	ABC transporter permease	QEN71_18125	paras_004169	protein-codi	NZ_CP1252	chromosom	4032787	4034697	+	1907	0	45	8454	35	6294	
QEN71_RS18130	ABC transporter ATP-binding protein	QEN71_18130	paras_004170	protein-codi	NZ_CP1252	chromosom	4034694	4035491	+	790	0	7	1447	5	1288	
QEN71_RS18135	ABC transporter ATP-binding protein	QEN71_18135	paras_004171	protein-codi	NZ_CP1252	chromosom	4035488	4036201	+	710	0	16	2773	6	1720	
QEN71_RS18140	ABC transporter substrate-binding protein	QEN71_18140	paras_004172	protein-codi	NZ_CP1252	chromosom	4036327	4037547	+	1221	0	44	11694	38	9831	
QEN71_RS18145	hypothetical protein	QEN71_18145	paras_004173	protein-codi	NZ_CP1252	chromosom	4037622	4037873	-	252	0	5	921	5	921	
QEN71_RS18150	DUF6496 domain-containing protein	QEN71_18150	paras_004174	protein-codi	NZ_CP1252	chromosom	4038098	4038679	+	582	0	4	1263	0	0	
QEN71_RS18155	aspartate aminotransferase family protein	QEN71_18155	paras_004175	protein-codi	NZ_CP1252	chromosom	4038767	4040095	-	1329	0	27	5617	18	1859	
QEN71_RS18160	PLP-dependent aminotransferase family protein	QEN71_18160	paras_004176	protein-codi	NZ_CP1252	chromosom	4040251	4041660	+	1410	0	31	10014	28	8814	
QEN71_RS18165	hypothetical protein	QEN71_18165	paras_004177	protein-codi	NZ_CP1252	chromosom	4041681	4041872	-	192	0	7	1437	3	880	
QEN71_RS18170	sensor domain-containing diguanylate cyclase	QEN71_18170	paras_004178	protein-codi	NZ_CP1252	chromosom	4041998	4043524	-	1527	0	37	17488	34	16078	
QEN71_RS18175	GGDEF domain-containing protein	QEN71_18175	paras_004179	protein-codi	NZ_CP1252	chromosom	4043743	4044855	-	1113	0	32	11289	18	5518	
QEN71_RS18180	hypothetical protein	QEN71_18180	paras_004180	protein-codi	NZ_CP1252	chromosom	4044865	4045494	-	630	0	14	3123	7	2193	
QEN71_RS18185	LysE family translocator	QEN71_18185	paras_004181	protein-codi	NZ_CP1252	chromosom	4045814	4046434	-	621	0	18	6172	16	5542	
QEN71_RS18190	SulP family inorganic anion transporter	QEN71_18190	paras_004182	protein-codi	NZ_CP1252	chromosom	4046611	4048326	+	1716	0	48	13316	40	12444	
QEN71_RS18195	VOC family protein	QEN71_18195	paras_004183	protein-codi	NZ_CP1252	chromosom	4048572	4048970	+	399	0	13	1784	7	1430	
QEN71_RS18200	tat (twin-arginine translocation) pathway signal sequ	QEN71_18200	paras_004184	protein-codi	NZ_CP1252	chromosom	4049026	4049646	+	621	0	5	1723	5	1723	
QEN71_RS18205	GMC family oxidoreductase	QEN71_18205	paras_004185	protein-codi	NZ_CP1252	chromosom	4049662	4051245	+	1584	0	41	8117	36	7115	
QEN71_RS18210	ribbon-helix-helix domain-containing protein	QEN71_18210	paras_004186	protein-codi	NZ_CP1252	chromosom	4051311	4051652	+	342	0	3	158	1	25	
QEN71_RS18215	sensor domain-containing diguanylate cyclase	QEN71_18215	paras_004187	protein-codi	NZ_CP1252	chromosom	4051679	4052731	-	1053	0	18	1629	16	1622	
QEN71_RS18220	LysR family transcriptional regulator	QEN71_18220	paras_004188	protein-codi	NZ_CP1252	chromosom	4052859	4053767	-	909	0	10	1463	4	443	
QEN71_RS18225	glutathione S-transferase	QEN71_18225	paras_004189	protein-codi	NZ_CP1252	chromosom	4053879	4054520	+	642	0	10	1931	9	1884	
QEN71_RS18230	pyridoxamine 5'-phosphate oxidase family protein	QEN71_18230	paras_004190	protein-codi	NZ_CP1252	chromosom	4054573	4056609	+	2037	0	45	5105	44	5100	
QEN71_RS18235	hypothetical protein	QEN71_18235	paras_004191	protein-codi	NZ_CP1252	chromosom	4056612	4056962	-	351	0	10	1982	8	860	
QEN71_RS18240	RNA polymerase sigma factor	QEN71_18240	paras_004192	protein-codi	NZ_CP1252	chromosom	4056976	4057695	-	720	0	13	1785	11	1527	
QEN71_RS18245	cupredoxin family copper-binding protein	QEN71_18245	paras_004193	protein-codi	NZ_CP1252	chromosom	4057716	4058030	-	315	0	7	1742	5	1341	
QEN71_RS18250	DUF4142 domain-containing protein	QEN71_18250	paras_004194	protein-codi	NZ_CP1252	chromosom	4058057	4058596	-	540	0	5	334	5	334	
QEN71_RS18255	chloride channel protein	QEN71_18255	paras_004195	protein-codi	NZ_CP1252	chromosom	4058710	4060527	-	1818	0	14	1356	13	1155	
QEN71_RS18260	helix-turn-helix domain-containing protein	QEN71_18260	paras_004196	protein-codi	NZ_CP1252	chromosom	4060555	4060968	-	414	0	13	1043	11	967	
QEN71_RS18265	HPP family protein	QEN71_18265	paras_004197	protein-codi	NZ_CP1252	chromosom	4061071	4062240	-	1170	0	12	1730	7	771	
QEN71_RS18270	phasin family protein	QEN71_18270	paras_004198	protein-codi	NZ_CP1252	chromosom	4062666	4063085	+	420	0	6	274	4	239	
QEN71_RS18275	putative zinc-binding metallopeptidase	QEN71_18275	paras_004199	protein-codi	NZ_CP1252	chromosom	4063126	4064196	-	1071	0	31	6399	28	6133	
QEN71_RS18280	transglutaminase family protein	QEN71_18280	paras_004200	protein-codi	NZ_CP1252	chromosom	4064545	4067955	+	3411	0	77	13896	58	11373	
QEN71_RS18285	circularly permuted type 2 ATP-grasp protein	QEN71_18285	paras_004201	protein-codi	NZ_CP1252	chromosom	4067997	4070609	+	2609	0	54	6742	42	5182	
QEN71_RS18290	transglutaminase family protein	QEN71_18290	paras_004202	protein-codi	NZ_CP1252	chromosom	4070606	4071496	+	887	0	20	1977	13	773	
QEN71_RS18295	MarR family transcriptional regulator	QEN71_18295	paras_004203	protein-codi	NZ_CP1252	chromosom	4071571	4071996	+	426	0	5	317	4	309	
QEN71_RS18300	MarR family winged helix-turn-helix transcriptional r	QEN71_18300	paras_004204	protein-codi	NZ_CP1252	chromosom	4072057	4072500	+	444	0	6	316	2	141	
QEN71_RS18305	HlyD family secretion protein	QEN71_18305	paras_004205	protein-codi	NZ_CP1252	chromosom	4072587	4073690	+	1104	0	14	1320	11	832	
QEN71_RS18310	DHA2 family efflux MFS transporter permease subu	QEN71_18310	paras_004206	protein-codi	NZ_CP1252	chromosom	4073712	4075292	+	1581	0	15	1324	14	1265	
QEN71_RS18315	efflux transporter outer membrane subunit	QEN71_18315	paras_004207	protein-codi	NZ_CP1252	chromosom	4075339	4076793	+	1455	0	21	2133	17	2007	
QEN71_RS18320	thiolase family protein	QEN71_18320	paras_004208	protein-codi	NZ_CP1252	chromosom	4076868	4078052	+	1185	0	14	1120	6	411	
QEN71_RS18325	2-dehydropantoate 2-reductase	QEN71_18325	paras_004209	protein-codi	NZ_CP1252	chromosom	4078181	4079119	+	939	0	19	2584	11	2045	
QEN71_RS18330	hypothetical protein	QEN71_18330	paras_004210	protein-codi	NZ_CP1252	chromosom	4079193	4079492	+	300	0	2	301	2	301	
QEN71_RS18335	OprD family outer membrane porin	QEN71_18335	paras_004211	protein-codi	NZ_CP1252	chromosom	4079560	4081068	-	1509	0	57	15780	43	12674	
QEN71_RS18340	ATP-binding cassette domain-containing protein	QEN71_18340	paras_004212	protein-codi	NZ_CP1252	chromosom	4081223	4082224	-	998	0	14	3460	7	1641	
QEN71_RS18345	ABC transporter ATP-binding protein	QEN71_18345	paras_004213	protein-codi	NZ_CP1252	chromosom	4082221	4083210	-	986	0	12	5878	10	4806	
QEN71_RS18350	ABC transporter permease subunit	QEN71_18350	paras_004214	protein-codi	NZ_CP1252	chromosom	4083212	4084102	-	891	0	16	7087	12	5986	
QEN71_RS18355	ABC transporter permease subunit	QEN71_18355	paras_004215	protein-codi	NZ_CP1252	chromosom	4084108	4085046	-	939	0	20	10286	10	4243	
QEN71_RS18360	peptide ABC transporter substrate-binding protein	QEN71_18360	paras_004216	protein-codi	NZ_CP1252	chromosom	4085076	4086692	-	1617	0	70	56419	50	37344	

QEN71_RS18365	cupin domain-containing protein	QEN71_18365	paras_004217	protein-codi	NZ_CP1252	chromosom	4086897	4087466	-	570	0	26	11570	18	9501	
QEN71_RS18370	oligopeptide:H+ symporter	QEN71_18370	paras_004218	protein-codi	NZ_CP1252	chromosom	4088215	4089741	+	1527	0	75	26668	66	24357	
QEN71_RS18375	PLP-dependent aminotransferase family protein	QEN71_18375	paras_004219	protein-codi	NZ_CP1252	chromosom	4090417	4091886	+	1470	0	31	8207	21	6017	
QEN71_RS18380	HU family DNA-binding protein	QEN71_18380	paras_004220	protein-codi	NZ_CP1252	chromosom	4092227	4092682	+	456	0	8	279	2	22	
QEN71_RS18385	circularly permuted type 2 ATP-grasp protein	QEN71_18385	paras_004221	protein-codi	NZ_CP1252	chromosom	4093655	4095064	+	1410	0	49	14888	45	13396	
QEN71_RS18390	alpha-E domain-containing protein	QEN71_18390	paras_004222	protein-codi	NZ_CP1252	chromosom	4095182	4096132	+	951	0	22	4420	18	3330	
QEN71_RS18395	transglutaminase family protein	QEN71_18395	paras_004223	protein-codi	NZ_CP1252	chromosom	4096146	4096952	+	807	0	15	3477	11	2662	
QEN71_RS18400	peptidase	QEN71_18400	paras_004224	protein-codi	NZ_CP1252	chromosom	4097104	4098006	+	903	0	25	5481	18	4396	
QEN71_RS18405	porin	QEN71_18405	paras_004225	protein-codi	NZ_CP1252	chromosom	4098161	4099339	-	1179	0	60	21935	52	18689	
QEN71_RS18410	YihY/virulence factor BrkB family protein	QEN71_18410	paras_004226	protein-codi	NZ_CP1252	chromosom	4099737	4101059	-	1323	0	28	8643	19	4176	
QEN71_RS18415	formate dehydrogenase-N subunit alpha	QEN71_18415	paras_004227	protein-codi	NZ_CP1252	chromosom	4101318	4104389	+	3072	0	80	12905	67	11570	
QEN71_RS18420	formate dehydrogenase subunit beta	QEN71_18420	paras_004228	protein-codi	NZ_CP1252	chromosom	4104400	4105338	+	935	0	15	4467	14	4454	
QEN71_RS18425	formate dehydrogenase subunit gamma	QEN71_18425	paras_004229	protein-codi	NZ_CP1252	chromosom	4105335	4105970	+	632	0	17	3826	15	3723	
QEN71_RS18430	formate dehydrogenase accessory protein FdhE	QEN71_18430	paras_004230	protein-codi	NZ_CP1252	chromosom	4106064	4106996	+	933	0	16	1709	16	1709	
QEN71_RS18435	L-seryl-tRNA(Sec) selenium transferase	QEN71_18435	paras_004231	protein-codi	NZ_CP1252	chromosom	4107012	4108457	+	1442	0	20	2895	15	2376	
QEN71_RS18440	selenocysteine-specific translation elongation factor	QEN71_18440	paras_004232	protein-codi	NZ_CP1252	chromosom	4108454	4110394	+	1937	0	33	4248	23	3171	
QEN71_RS18445	tRNA-Sec	QEN71_18445		tRNA	NZ_CP1252	chromosom	4110479	4110574	+	96	0	3	315	3	315	
QEN71_RS18450	AAA family ATPase	QEN71_18450	paras_004235	protein-codi	NZ_CP1252	chromosom	4112051	4113106	-	1056	0	48	2996	39	2545	
QEN71_RS18455	DDE-type integrase/transposase/recombinase	QEN71_18455	paras_004236	protein-codi	NZ_CP1252	chromosom	4113340	4115931	-	2592	0	100	8650	82	7159	
QEN71_RS18460	hypothetical protein	QEN71_18460	paras_004237	protein-codi	NZ_CP1252	chromosom	4117078	4117773	+	696	0	41	4831	30	3954	
QEN71_RS18465	TniQ family protein	QEN71_18465	paras_004238	protein-codi	NZ_CP1252	chromosom	4117904	4119220	-	1317	0	59	8317	43	6770	
QEN71_RS18470	hypothetical protein	QEN71_18470	paras_004239	protein-codi	NZ_CP1252	chromosom	4119234	4119713	-	480	0	36	5421	30	4612	
QEN71_RS18475	DUF6471 domain-containing protein	QEN71_18475		protein-codi	NZ_CP1252	chromosom	4119999	4120226	+	228	0	18	4929	9	2153	
QEN71_RS18480	BPSL0761 family protein			protein-codi	NZ_CP1252	chromosom	4120521	4120829	+	309	0	10	951	9	949	
QEN71_RS18485	IS21 family transposase	QEN71_18480	paras_004240	protein-codi	NZ_CP1252	chromosom	4121331	4122851	+	1521	3018.0	0	0	0	0	
QEN71_RS18490	IS21-like element helper ATPase IstB	QEN71_18485	paras_004241	protein-codi	NZ_CP1252	chromosom	4122857	4123645	+	789	1559.0	0	0	0	0	
QEN71_RS18495	metallophosphoesterase	QEN71_18490		protein-codi	NZ_CP1252	chromosom	4123742	4124548	+	807	0	23	5288	21	4842	
QEN71_RS18500	hypothetical protein	QEN71_18495	paras_004243	protein-codi	NZ_CP1252	chromosom	4124704	4124979	+	276	0	8	1624	6	1279	
QEN71_RS18505	HAD domain-containing protein	QEN71_18500	paras_004245	protein-codi	NZ_CP1252	chromosom	4125096	4126199	+	1104	0	25	5648	14	4427	
QEN71_RS18510	hypothetical protein	QEN71_18505		protein-codi	NZ_CP1252	chromosom	4126385	4126672	+	271	0	6	1012	6	1012	
QEN71_RS18515	hypothetical protein	QEN71_18510	paras_004246	protein-codi	NZ_CP1252	chromosom	4126656	4127318	+	646	0	13	2118	10	1862	
QEN71_RS18520	hypothetical protein	QEN71_18515	paras_004247	protein-codi	NZ_CP1252	chromosom	4127449	4127715	+	267	0	11	2275	8	1956	
QEN71_RS18525	hypothetical protein	QEN71_18520	paras_004248	protein-codi	NZ_CP1252	chromosom	4128131	4128349	-	218	0	6	601	4	490	
QEN71_RS18530	hypothetical protein	QEN71_18525	paras_004249	protein-codi	NZ_CP1252	chromosom	4128349	4128696	-	343	0	4	1094	4	1094	
QEN71_RS18535	hypothetical protein	QEN71_18530	paras_004250	protein-codi	NZ_CP1252	chromosom	4128693	4129007	-	311	0	2	47	1	17	
QEN71_RS18540	translesion DNA synthesis-associated protein ImuA	QEN71_18535	paras_004251	protein-codi	NZ_CP1252	chromosom	4129073	4129783	+	711	0	13	2353	10	1981	
QEN71_RS18545	SOS response-associated peptidase family protein	QEN71_18540	paras_004252	protein-codi	NZ_CP1252	chromosom	4129829	4130791	+	963	107.0	37	7692	23	4265	
QEN71_RS18550	hypothetical protein	QEN71_18545	paras_004253	protein-codi	NZ_CP1252	chromosom	4130830	4131141	-	312	192.0	7	1496	7	1496	
QEN71_RS18555	H-NS family nucleoid-associated regulatory protein	QEN71_18550	paras_004254	protein-codi	NZ_CP1252	chromosom	4131323	4131661	-	339	147.0	6	870	3	168	
QEN71_RS18560	H-NS family nucleoid-associated regulatory protein	QEN71_18555	paras_004255	protein-codi	NZ_CP1252	chromosom	4131831	4132592	+	762	332.0	16	2647	13	2466	
QEN71_RS18565	PAS domain-containing protein	QEN71_18560	paras_004256	protein-codi	NZ_CP1252	chromosom	4133701	4134330	+	619	0	18	4163	11	2181	
QEN71_RS18570	ornithine cyclodeaminase family protein	QEN71_18565	paras_004257	protein-codi	NZ_CP1252	chromosom	4134320	4135291	+	961	0	13	3214	9	2380	
QEN71_RS18575	threonine synthase	QEN71_18570	paras_004258	protein-codi	NZ_CP1252	chromosom	4135552	4136697	+	1146	0	30	3415	22	2329	
QEN71_RS18580	asparaginase domain-containing protein	QEN71_18575	paras_004259	protein-codi	NZ_CP1252	chromosom	4136710	4137639	+	930	0	27	4560	22	4129	
QEN71_RS18585	branched-chain amino acid ABC transporter substra	QEN71_18580	paras_004260	protein-codi	NZ_CP1252	chromosom	4137979	4139124	-	1146	0	36	7741	24	5472	
QEN71_RS18590	hypothetical protein	QEN71_18585	paras_004261	protein-codi	NZ_CP1252	chromosom	4139559	4140257	-	699	0	44	13258	30	8148	
QEN71_RS18595	hypothetical protein	QEN71_18590	paras_004262	protein-codi	NZ_CP1252	chromosom	4140368	4140649	-	282	0	19	8958	14	5862	
QEN71_RS18600	hypothetical protein	QEN71_18595	paras_004263	protein-codi	NZ_CP1252	chromosom	4141320	4141475	+	156	0	4	1966	0	0	
QEN71_RS18605	transposase	QEN71_18600	paras_004264	protein-codi	NZ_CP1252	chromosom	4141837	4143252	+	1416	0	62	28002	51	24685	
QEN71_RS18610	hypothetical protein	QEN71_18605	paras_004265	protein-codi	NZ_CP1252	chromosom	4143485	4143817	+	333	0	18	6381	8	2852	
QEN71_RS18615	IS110 family transposase	QEN71_18610	paras_004266	protein-codi	NZ_CP1252	chromosom	4144876	4145889	-	1014	2002.0	22	5947	19	4636	
QEN71_RS18620	HAD-IA family hydrolase	QEN71_18615	paras_004267	protein-codi	NZ_CP1252	chromosom	4146044	4146661	+	618	0	32	4869	17	2119	
QEN71_RS18625	hypothetical protein	QEN71_18620	paras_004268	protein-codi	NZ_CP1252	chromosom	4147205	4147864	+	660	0	22	3035	16	2184	
QEN71_RS18630	hypothetical protein	QEN71_18625	paras_004269	protein-codi	NZ_CP1252	chromosom	4147952	4148419	-	468	0	20	3501	14	3012	
QEN71_RS18635	hypothetical protein	QEN71_18630	paras_004270	protein-codi	NZ_CP1252	chromosom	4148435	4148839	-	405	0	5	643	2	177	
QEN71_RS18640	DMT family transporter	QEN71_18635	paras_004271	protein-codi	NZ_CP1252	chromosom	4149349	4150293	-	945	0	24	2809	22	2626	
QEN71_RS18645	transcriptional regulator GcvA	QEN71_18640	paras_004272	protein-codi	NZ_CP1252	chromosom	4150408	4151316	+	909	0	22	1682	19	1653	
QEN71_RS18650	BON domain-containing protein	QEN71_18645	paras_004273	protein-codi	NZ_CP1252	chromosom	4151650	4152033	-	384	0	8	774	4	470	
QEN71_RS18655	porin	QEN71_18650	paras_004274	protein-codi	NZ_CP1252	chromosom	4152063	4153214	-	1152	0	53	4466	45	3968	
QEN71_RS18660	Rieske 2Fe-2S domain-containing protein	QEN71_18655	paras_004275	protein-codi	NZ_CP1252	chromosom	4153407	4153754	-	348	0	5	721	4	558	
QEN71_RS18665	fatty acid--CoA ligase	QEN71_18660	paras_004276	protein-codi	NZ_CP1252	chromosom	4153877	4155520	-	1644	0	50	7037	37	5099	
QEN71_RS18670	TauD/TfdA family dioxygenase	QEN71_18665	paras_004277	protein-codi	NZ_CP1252	chromosom	4155529	4156377	-	849	0	14	1111	5	411	

QEN71_RS18675	quinone oxidoreductase	QEN71_18670	paras_004278	protein-codi	NZ_CP1252	chromosom	4156403	4157383	-	981	0	14	1398	14	1398	
QEN71_RS18680	acetoacetate--CoA ligase	QEN71_18675	paras_004279	protein-codi	NZ_CP1252	chromosom	4157472	4159484	-	2013	0	41	4464	35	4010	
QEN71_RS18685	MFS transporter	QEN71_18680	paras_004280	protein-codi	NZ_CP1252	chromosom	4159537	4160877	-	1341	0	24	1705	18	1357	
QEN71_RS18690	3-keto-5-aminohexanoate cleavage protein	QEN71_18685	paras_004281	protein-codi	NZ_CP1252	chromosom	4161017	4162072	-	1056	0	15	1041	13	1027	
QEN71_RS18695	AraC family transcriptional regulator	QEN71_18690	paras_004282	protein-codi	NZ_CP1252	chromosom	4162276	4163271	+	996	0	25	3577	22	3389	
QEN71_RS18700	electron transfer flavoprotein subunit beta/FixA fami	QEN71_18695	paras_004283	protein-codi	NZ_CP1252	chromosom	4163568	4164317	+	750	362.0	6	482	2	254	
QEN71_RS18705	electron transfer flavoprotein subunit alpha/FixB fan	QEN71_18700	paras_004284	protein-codi	NZ_CP1252	chromosom	4164346	4165281	+	936	541.0	12	786	9	673	
QEN71_RS18710	MaoC family dehydratase	QEN71_18705	paras_004285	protein-codi	NZ_CP1252	chromosom	4165365	4165835	+	471	0	6	212	5	210	
QEN71_RS18715	class I poly(R)-hydroxyalkanoic acid synthase	pseudo;QEN71_18710		pseudogene	NZ_CP1252	chromosom	4165850	4167590	+	1741	0	55	6255	41	4509	
QEN71_RS18720	AraC family transcriptional regulator	QEN71_18715	paras_004287	protein-codi	NZ_CP1252	chromosom	4167783	4168760	+	978	0	21	3026	18	2792	
QEN71_RS18725	electron transfer flavoprotein-ubiquinone oxidoredu	QEN71_18720	paras_004288	protein-codi	NZ_CP1252	chromosom	4169010	4170683	+	1674	0	36	6720	26	5025	
QEN71_RS18730	acetyl-CoA C-acyltransferase family protein	QEN71_18725	paras_004289	protein-codi	NZ_CP1252	chromosom	4170748	4171941	+	1194	0	30	4178	21	2657	
QEN71_RS18735	hypothetical protein	QEN71_18730		protein-codi	NZ_CP1252	chromosom	4172607	4172879	-	273	0	10	1820	4	519	
QEN71_RS18740	hypothetical protein	QEN71_18735	paras_004290	protein-codi	NZ_CP1252	chromosom	4173137	4173334	-	198	0	2	405	1	59	
QEN71_RS18745	hypothetical protein	QEN71_18740	paras_004291	protein-codi	NZ_CP1252	chromosom	4173930	4174331	+	402	0	7	356	5	346	
QEN71_RS18750	PAS domain S-box protein	QEN71_18745	paras_004292	protein-codi	NZ_CP1252	chromosom	4175267	4177654	+	2388	0	54	7883	43	6999	
QEN71_RS18755	GFA family protein	QEN71_18750	paras_004293	protein-codi	NZ_CP1252	chromosom	4178129	4178491	+	363	0	18	2871	15	2350	
QEN71_RS18760	IS21-like element helper ATPase IstB	QEN71_18755	paras_004294	protein-codi	NZ_CP1252	chromosom	4178614	4179402	-	789	1561.0	2	4	1	2	
QEN71_RS18765	IS21 family transposase	QEN71_18760	paras_004295	protein-codi	NZ_CP1252	chromosom	4179409	4180932	-	1524	3027.0	3	16	2	12	
QEN71_RS18770	MFS transporter	QEN71_18765	paras_004296	protein-codi	NZ_CP1252	chromosom	4181302	4182489	-	1188	0	33	5797	29	4337	
QEN71_RS18775	CGNR zinc finger domain-containing protein	QEN71_18770	paras_004297	protein-codi	NZ_CP1252	chromosom	4182562	4183131	+	570	0	13	1050	11	579	
QEN71_RS18780	glycosyltransferase family A protein	QEN71_18775	paras_004298	protein-codi	NZ_CP1252	chromosom	4183469	4184473	-	1005	0	38	3738	30	3295	
QEN71_RS18785	helix-turn-helix domain-containing protein	QEN71_18780	paras_004299	protein-codi	NZ_CP1252	chromosom	4184981	4185895	+	915	0	29	2831	16	1660	
QEN71_RS18790	GGDEF domain-containing phosphodiesterase	QEN71_18785	paras_004300	protein-codi	NZ_CP1252	chromosom	4186849	4187901	-	1053	0	27	5197	24	5146	
QEN71_RS18795	hypothetical protein	QEN71_18790	paras_004301	protein-codi	NZ_CP1252	chromosom	4188027	4188533	-	507	164.0	15	5867	15	5867	
QEN71_RS18800	hypothetical protein	QEN71_18795	paras_004302	protein-codi	NZ_CP1252	chromosom	4188584	4188829	+	246	159.0	3	1546	3	1546	
QEN71_RS18805	phasin family protein	QEN71_18800	paras_004303	protein-codi	NZ_CP1252	chromosom	4190114	4190617	+	504	0	19	3581	13	1281	
QEN71_RS18810	hypothetical protein	QEN71_18805	paras_004304	protein-codi	NZ_CP1252	chromosom	4191461	4191658	+	198	0	2	434	2	434	
QEN71_RS18815	TIGR01841 family phasin	QEN71_18810	paras_004305	protein-codi	NZ_CP1252	chromosom	4191787	4192347	+	482	0	13	2142	10	1516	
QEN71_RS18820	hypothetical protein	QEN71_18815		protein-codi	NZ_CP1252	chromosom	4192269	4192529	-	182	159.0	0	0	0	0	
QEN71_RS18825	hypothetical protein	QEN71_18820	paras_004306	protein-codi	NZ_CP1252	chromosom	4192606	4192812	+	207	184.0	6	453	1	16	
QEN71_RS18830	response regulator receiver protein	QEN71_18825	paras_004307	protein-codi	NZ_CP1252	chromosom	4192834	4193877	-	1044	0	26	2693	20	2184	
QEN71_RS18835	hypothetical protein	QEN71_18830	paras_004308	protein-codi	NZ_CP1252	chromosom	4194100	4194360	-	261	0	1	286	1	286	
QEN71_RS18840	hypothetical protein	QEN71_18835	paras_004310	protein-codi	NZ_CP1252	chromosom	4194845	4195369	-	525	0	11	1504	10	1469	
QEN71_RS18845	hypothetical protein	QEN71_18840	paras_004311	protein-codi	NZ_CP1252	chromosom	4195771	4196001	-	231	0	6	589	5	397	
QEN71_RS18850	hemerythrin domain-containing protein	QEN71_18845	paras_004312	protein-codi	NZ_CP1252	chromosom	4196050	4196622	-	573	0	9	556	4	120	
QEN71_RS18855	FdhF/YdeP family oxidoreductase	QEN71_18850	paras_004313	protein-codi	NZ_CP1252	chromosom	4196733	4199012	-	2280	0	36	3049	24	2463	
QEN71_RS18860	DUF3597 domain-containing protein	QEN71_18855	paras_004314	protein-codi	NZ_CP1252	chromosom	4199237	4199713	+	477	0	5	504	2	204	
QEN71_RS18865	CinA family protein	QEN71_18860	paras_004315	protein-codi	NZ_CP1252	chromosom	4199823	4200326	+	504	0	12	1141	8	871	
QEN71_RS18870	ammonia-dependent NAD(+) synthetase	QEN71_18865	paras_004316	protein-codi	NZ_CP1252	chromosom	4200435	4201277	+	843	0	22	2484	18	2024	
QEN71_RS18875	cation:proton antiporter	QEN71_18870	paras_004317	protein-codi	NZ_CP1252	chromosom	4201503	4202876	-	1374	0	24	1935	20	1601	
QEN71_RS18880	hypothetical protein	QEN71_18875	paras_004318	protein-codi	NZ_CP1252	chromosom	4202916	4203107	-	192	0	4	912	2	360	
QEN71_RS18885	cytochrome c oxidase subunit II	QEN71_18880	paras_004319	protein-codi	NZ_CP1252	chromosom	4203299	4204363	+	1061	0	15	1388	15	1388	
QEN71_RS18890	cytochrome c oxidase subunit I	QEN71_18885	paras_004320	protein-codi	NZ_CP1252	chromosom	4204360	4206984	+	2617	0	59	4001	43	3084	
QEN71_RS18895	hypothetical protein	QEN71_18890	paras_004321	protein-codi	NZ_CP1252	chromosom	4206981	4207331	+	322	0	6	369	6	369	
QEN71_RS18900	cytochrome c oxidase assembly protein	QEN71_18895	paras_004322	protein-codi	NZ_CP1252	chromosom	4207307	4208296	+	961	0	21	2088	17	917	
QEN71_RS18905	cytochrome c	QEN71_18900	paras_004323	protein-codi	NZ_CP1252	chromosom	4208293	4209615	+	1319	0	40	5693	32	3998	
QEN71_RS18910	glycosyltransferase family A protein	QEN71_18905	paras_004324	protein-codi	NZ_CP1252	chromosom	4209724	4210392	-	669	0	17	1509	12	1253	
QEN71_RS18915	dodecin family protein	QEN71_18910	paras_004325	protein-codi	NZ_CP1252	chromosom	4210625	4210846	+	222	0	9	658	7	623	
QEN71_RS18920	hypothetical protein	QEN71_18915	paras_004326	protein-codi	NZ_CP1252	chromosom	4210931	4211890	-	960	0	13	1049	12	1030	
QEN71_RS18925	hypothetical protein	QEN71_18920	paras_004327	protein-codi	NZ_CP1252	chromosom	4212875	4213579	+	705	0	8	513	7	505	
QEN71_RS18930	PAS domain S-box protein	QEN71_18925	paras_004328	protein-codi	NZ_CP1252	chromosom	4213981	4216335	-	2355	0	52	4365	43	3604	
QEN71_RS18935	glucose 1-dehydrogenase	QEN71_18930	paras_004329	protein-codi	NZ_CP1252	chromosom	4216650	4217516	+	867	0	16	1412	11	820	
QEN71_RS18940	SDR family oxidoreductase	QEN71_18935	paras_004330	protein-codi	NZ_CP1252	chromosom	4217566	4218567	+	1002	0	16	632	11	376	
QEN71_RS18945	hypothetical protein	QEN71_18940	paras_004331	protein-codi	NZ_CP1252	chromosom	4218597	4219082	+	486	0	14	1419	12	1250	
QEN71_RS18950	ATP-dependent DNA helicase RecQ	QEN71_18945	paras_004332	protein-codi	NZ_CP1252	chromosom	4219124	4220896	-	1773	0	25	1966	21	1825	
QEN71_RS18955	manganese catalase family protein	QEN71_18950	paras_004333	protein-codi	NZ_CP1252	chromosom	4221005	4221895	-	891	0	18	1626	14	1417	
QEN71_RS18960	L-dopachrome tautomerase-related protein	QEN71_18955	paras_004334	protein-codi	NZ_CP1252	chromosom	4221965	4223080	-	1116	0	21	1833	20	1813	
QEN71_RS18965	SDR family oxidoreductase	QEN71_18960	paras_004335	protein-codi	NZ_CP1252	chromosom	4223126	4224121	-	996	0	25	2234	23	2033	
QEN71_RS18970	hypothetical protein	QEN71_18965	paras_004336	protein-codi	NZ_CP1252	chromosom	4224285	4224539	-	255	0	6	361	4	336	
QEN71_RS18975	zinc-dependent alcohol dehydrogenase	QEN71_18970	paras_004337	protein-codi	NZ_CP1252	chromosom	4224665	4225834	-	1170	0	19	2040	13	1711	
QEN71_RS18980	CsbD family protein	QEN71_18975	paras_004338	protein-codi	NZ_CP1252	chromosom	4225881	4226084	-	204	0	5	645	3	524	

QEN71_RS18985	response regulator	QEN71_18980	paras_004339	protein-codi	NZ_CP1252	chromosom	4226284	4226784	+	501	0	14	1863	12	1732	
QEN71_RS18990	hypothetical protein	QEN71_18985	paras_004340	protein-codi	NZ_CP1252	chromosom	4226834	4227034	-	201	0	4	562	4	562	
QEN71_RS18995	ATPase domain-containing protein	QEN71_18990	paras_004341	protein-codi	NZ_CP1252	chromosom	4227276	4228715	+	1440	0	22	3487	15	2972	
QEN71_RS19000	response regulator	QEN71_18995	paras_004342	protein-codi	NZ_CP1252	chromosom	4228735	4229130	+	396	0	11	1911	6	1736	
QEN71_RS19005	response regulator	QEN71_19000	paras_004343	protein-codi	NZ_CP1252	chromosom	4229220	4229576	+	357	0	14	3770	12	3531	
QEN71_RS19010	ATP-binding protein	QEN71_19005	paras_004344	protein-codi	NZ_CP1252	chromosom	4229747	4231210	+	1464	0	12	1187	7	428	
QEN71_RS19015	hypothetical protein	QEN71_19010	paras_004345	protein-codi	NZ_CP1252	chromosom	4231231	4231440	-	210	105.0	7	471	3	173	
QEN71_RS19020	sigma-54 dependent transcriptional regulator	QEN71_19015	paras_004346	protein-codi	NZ_CP1252	chromosom	4232035	4233441	-	1407	1297.0	20	1440	16	1349	
QEN71_RS19025	alpha-amylase			pseudogene	NZ_CP1252	chromosom	4233597	4233963	+	367	0	12	956	8	539	
QEN71_RS19030	ParB/Srx family N-terminal domain-containing prote	QEN71_19025	paras_004347	protein-codi	NZ_CP1252	chromosom	4234292	4234885	+	594	0	11	2521	10	2519	
QEN71_RS19035	phosphatidylserine decarboxylase family protein	QEN71_19030	paras_004348	protein-codi	NZ_CP1252	chromosom	4235056	4236300	+	1245	0	53	9632	52	9521	
QEN71_RS19040	Gfo/Idh/MocA family oxidoreductase	QEN71_19035	paras_004349	protein-codi	NZ_CP1252	chromosom	4236619	4237776	+	1158	0	45	8985	36	7279	
QEN71_RS19045	GNAT family N-acetyltransferase	QEN71_19040	paras_004350	protein-codi	NZ_CP1252	chromosom	4238377	4238814	-	438	0	20	2594	12	1569	
QEN71_RS19050	pyridoxamine 5'-phosphate oxidase family protein	QEN71_19045	paras_004351	protein-codi	NZ_CP1252	chromosom	4238870	4239457	-	588	0	12	1987	9	1013	
QEN71_RS19055	Na/Pi cotransporter family protein	QEN71_19050	paras_004352	protein-codi	NZ_CP1252	chromosom	4239512	4241233	-	1722	0	45	4710	27	3055	
QEN71_RS19060	NUDIX domain-containing protein	QEN71_19055	paras_004353	protein-codi	NZ_CP1252	chromosom	4241829	4242311	-	483	0	19	3910	11	1676	
QEN71_RS19065	hypothetical protein	QEN71_19060	paras_004354	protein-codi	NZ_CP1252	chromosom	4242641	4242838	+	198	0	2	48	2	48	
QEN71_RS19070	SDR family oxidoreductase	partial;pseudo;QEN71_19065		pseudogene	NZ_CP1252	chromosom	4242893	4243144	+	252	0	13	1370	8	1213	
QEN71_RS19075	hypothetical protein	QEN71_19070	paras_004356	protein-codi	NZ_CP1252	chromosom	4243292	4243888	+	597	0	24	5229	17	4279	
QEN71_RS19080	HU family DNA-binding protein	QEN71_19075	paras_004357	protein-codi	NZ_CP1252	chromosom	4244017	4244295	+	279	0	8	570	7	395	
QEN71_RS19085	alpha/beta hydrolase	QEN71_19080	paras_004358	protein-codi	NZ_CP1252	chromosom	4244371	4245204	-	834	0	21	2586	16	2016	
QEN71_RS19090	IS66 family transposase	QEN71_19085	paras_004359	protein-codi	NZ_CP1252	chromosom	4245887	4247473	-	1587	3142.0	2	5	1	2	
QEN71_RS19095	IS66 family insertion sequence element accessory p	QEN71_19090	paras_004360	protein-codi	NZ_CP1252	chromosom	4247504	4247848	-	341	674.0	0	0	0	0	
QEN71_RS19100	transposase	QEN71_19095	paras_004361	protein-codi	NZ_CP1252	chromosom	4247845	4248327	-	479	944.0	2	105	2	105	
QEN71_RS19105	hypothetical protein	QEN71_19100		protein-codi	NZ_CP1252	chromosom	4248504	4248782	+	279	0	16	1858	6	666	
QEN71_RS19110	cation diffusion facilitator family transporter	QEN71_19105	paras_004362	protein-codi	NZ_CP1252	chromosom	4248873	4249814	-	942	0	20	2970	12	1858	
QEN71_RS19115	NAD(P)H-dependent oxidoreductase	QEN71_19110	paras_004363	protein-codi	NZ_CP1252	chromosom	4250061	4251095	+	1035	559.0	14	2015	12	1914	
QEN71_RS19120	glutathione-independent formaldehyde dehydrogena	QEN71_19115	paras_004364	protein-codi	NZ_CP1252	chromosom	4251214	4252350	+	1137	0	15	2010	12	1800	
QEN71_RS19125	NADP-dependent oxidoreductase	QEN71_19120	paras_004365	protein-codi	NZ_CP1252	chromosom	4252400	4253350	+	935	0	16	1429	11	1246	
QEN71_RS19130	SDR family oxidoreductase	QEN71_19125	paras_004366	protein-codi	NZ_CP1252	chromosom	4253335	4254357	-	1007	0	20	1679	17	1312	
QEN71_RS19135	glycosyltransferase family 4 protein	QEN71_19130	paras_004367	protein-codi	NZ_CP1252	chromosom	4254380	4255336	-	946	0	20	2491	10	1119	
QEN71_RS19140	glycosyltransferase family 9 protein	QEN71_19135	paras_004368	protein-codi	NZ_CP1252	chromosom	4255326	4256396	-	1060	0	11	715	10	687	
QEN71_RS19145	glycosyltransferase	QEN71_19140	paras_004369	protein-codi	NZ_CP1252	chromosom	4256462	4257442	-	981	0	16	1152	12	844	
QEN71_RS19150	carbamoyltransferase C-terminal domain-containing	QEN71_19145	paras_004370	protein-codi	NZ_CP1252	chromosom	4257508	4259274	-	1767	0	33	4239	27	3451	
QEN71_RS19155	glycosyltransferase family 9 protein	QEN71_19150	paras_004371	protein-codi	NZ_CP1252	chromosom	4259280	4260485	-	1202	0	10	1039	8	881	
QEN71_RS19160	HAD family hydrolase	QEN71_19155	paras_004372	protein-codi	NZ_CP1252	chromosom	4260482	4261102	-	617	0	9	891	9	891	
QEN71_RS19165	glycosyltransferase family 1 protein	QEN71_19160	paras_004373	protein-codi	NZ_CP1252	chromosom	4261167	4262459	-	1293	0	19	3848	13	2218	
QEN71_RS19170	ATP-binding cassette domain-containing protein	QEN71_19165	paras_004374	protein-codi	NZ_CP1252	chromosom	4262855	4265551	+	2697	0	31	4418	27	3685	
QEN71_RS19175	glycosyltransferase	QEN71_19170	paras_004375	protein-codi	NZ_CP1252	chromosom	4265557	4266558	-	998	0	13	676	11	437	
QEN71_RS19180	DUF2795 domain-containing protein	QEN71_19175	paras_004376	protein-codi	NZ_CP1252	chromosom	4266555	4266803	-	245	0	9	2458	5	990	
QEN71_RS19185	CheR family methyltransferase	QEN71_19180	paras_004377	protein-codi	NZ_CP1252	chromosom	4267487	4271641	+	4155	0	67	9685	52	7020	
QEN71_RS19190	chemotaxis protein CheB	QEN71_19185	paras_004378	protein-codi	NZ_CP1252	chromosom	4271758	4272756	+	999	0	30	4800	26	3825	
QEN71_RS19195	response regulator receiver protein	QEN71_19190	paras_004379	protein-codi	NZ_CP1252	chromosom	4272842	4273492	-	651	0	17	2142	15	1741	
QEN71_RS19200	hypothetical protein			protein-codi	NZ_CP1252	chromosom	4273858	4274154	+	297	0	4	1776	4	1776	
QEN71_RS19205	hypothetical protein	QEN71_19200	paras_004381	protein-codi	NZ_CP1252	chromosom	4274444	4274665	-	222	0	11	1161	9	787	
QEN71_RS19210	divalent metal cation transporter	pseudo;QEN71_19205		pseudogene	NZ_CP1252	chromosom	4274868	4276160	+	1293	0	49	6776	42	6034	
QEN71_RS19215	hypothetical protein	QEN71_19210	paras_004383	protein-codi	NZ_CP1252	chromosom	4276435	4276641	-	207	0	3	135	3	135	
QEN71_RS19220	permease	pseudo;QEN71_19215		pseudogene	NZ_CP1252	chromosom	4277379	4278481	-	1103	0	26	5919	24	5238	
QEN71_RS19225	ATP-binding protein	QEN71_19220	paras_004385	protein-codi	NZ_CP1252	chromosom	4278922	4281714	+	2793	0	68	10847	59	9909	
QEN71_RS19230	CheR family methyltransferase	QEN71_19225	paras_004386	protein-codi	NZ_CP1252	chromosom	4282113	4285775	-	3663	0	82	20511	60	13124	
QEN71_RS19235	PAS domain-containing sensor histidine kinase	QEN71_19230	paras_004387	protein-codi	NZ_CP1252	chromosom	4286112	4287632	+	1521	0	52	9188	43	7065	
QEN71_RS19240	SET domain-containing protein-lysine N-methyltrans	pseudo;QEN71_19235		pseudogene	NZ_CP1252	chromosom	4288027	4288493	-	467	0	11	2661	7	1705	
QEN71_RS19245	HU family DNA-binding protein	QEN71_19240	paras_004389	protein-codi	NZ_CP1252	chromosom	4288731	4288979	+	249	0	5	1189	1	13	
QEN71_RS19250	DUF1488 family protein	QEN71_19245	paras_004390	protein-codi	NZ_CP1252	chromosom	4289094	4289363	-	270	0	3	252	1	88	
QEN71_RS19255	hypothetical protein	QEN71_19250	paras_004391	protein-codi	NZ_CP1252	chromosom	4290020	4290508	-	489	0	12	1709	10	1489	
QEN71_RS19260	hypothetical protein	QEN71_19255	paras_004392	protein-codi	NZ_CP1252	chromosom	4290514	4290705	-	192	0	0	0	0	0	
QEN71_RS19265	IS30 family transposase	partial;pseudo;QEN71_19260		pseudogene	NZ_CP1252	chromosom	4290795	4291013	+	219	0	11	1315	6	613	
QEN71_RS19270	alpha/beta fold hydrolase	QEN71_19265	paras_004394	protein-codi	NZ_CP1252	chromosom	4291084	4292247	-	1164	0	31	3521	23	2109	
QEN71_RS19275	LysR family transcriptional regulator	QEN71_19270	paras_004395	protein-codi	NZ_CP1252	chromosom	4292358	4293326	+	969	0	16	1868	15	1865	
QEN71_RS19280	Na+/H+ antiporter NhaA	QEN71_19275	paras_004396	protein-codi	NZ_CP1252	chromosom	4293869	4295095	+	1227	0	25	2282	21	1905	
QEN71_RS19285	YXWGXW repeat-containing protein	QEN71_19280	paras_004397	protein-codi	NZ_CP1252	chromosom	4295147	4295485	+	339	0	17	2183	17	2183	
QEN71_RS19290	hypothetical protein	QEN71_19285	paras_004398	protein-codi	NZ_CP1252	chromosom	4295886	4296245	+	360	0	6	839	6	839	

QEN71_RS19295	phospholipase D family protein	QEN71_19290	paras_004399	protein-codi	NZ_CP1252	chromosom	4296411	4297943	+	1533	0	36	3131	25	2145	
QEN71_RS19300	AraC family transcriptional regulator	QEN71_19295	paras_004400	protein-codi	NZ_CP1252	chromosom	4298085	4299116	-	1032	0	30	3390	21	2193	
QEN71_RS19305	carboxymuconolactone decarboxylase family protei	QEN71_19300	paras_004401	protein-codi	NZ_CP1252	chromosom	4299259	4299666	+	408	0	5	1100	5	1100	
QEN71_RS19310	3-keto-5-aminohexanoate cleavage protein	QEN71_19305	paras_004402	protein-codi	NZ_CP1252	chromosom	4299706	4300761	+	1056	0	10	903	8	812	
QEN71_RS19315	MFS transporter	QEN71_19310	paras_004403	protein-codi	NZ_CP1252	chromosom	4300840	4302120	+	1281	0	33	5061	29	4589	
QEN71_RS19320	SDR family NAD(P)-dependent oxidoreductase	QEN71_19315	paras_004404	protein-codi	NZ_CP1252	chromosom	4302123	4302959	+	837	0	21	3347	16	2338	
QEN71_RS19325	DUF1289 domain-containing protein	QEN71_19320	paras_004405	protein-codi	NZ_CP1252	chromosom	4302973	4303209	+	233	0	6	805	5	752	
QEN71_RS19330	3-(methylthio)propionyl-CoA ligase	pseudo;QEN71_19325		pseudogene	NZ_CP1252	chromosom	4303206	4304843	+	1634	0	33	3346	26	1705	
QEN71_RS19335	porin	QEN71_19330	paras_004407	protein-codi	NZ_CP1252	chromosom	4304922	4306067	+	1146	0	34	4321	24	3275	
QEN71_RS19340	BON domain-containing protein	QEN71_19335	paras_004408	protein-codi	NZ_CP1252	chromosom	4306101	4306484	+	384	0	8	483	4	199	
QEN71_RS19345	sigma-54 dependent transcriptional regulator	QEN71_19340	paras_004409	protein-codi	NZ_CP1252	chromosom	4307418	4308809	-	1392	1297.0	22	2272	15	1344	
QEN71_RS19350	ATP-grasp fold amidoligase family protein	QEN71_19345	paras_004410	protein-codi	NZ_CP1252	chromosom	4308939	4309781	-	843	0	17	918	15	876	
QEN71_RS19355	tyrosinase family protein	QEN71_19350	paras_004411	protein-codi	NZ_CP1252	chromosom	4310074	4311411	-	1338	0	27	2203	19	1486	
QEN71_RS19360	type II toxin-antitoxin system HicB family antitoxin	QEN71_19355	paras_004412	protein-codi	NZ_CP1252	chromosom	4312066	4312326	+	261	0	4	525	3	495	
QEN71_RS19365	DUF1328 domain-containing protein	QEN71_19360	paras_004413	protein-codi	NZ_CP1252	chromosom	4312435	4312596	-	162	0	1	4	0	0	
QEN71_RS19370	hypothetical protein	QEN71_19365		protein-codi	NZ_CP1252	chromosom	4312770	4313192	-	423	0	5	105	5	105	
QEN71_RS19375	hypothetical protein	QEN71_19370	paras_004414	protein-codi	NZ_CP1252	chromosom	4313411	4313935	+	525	0	10	654	6	120	
QEN71_RS19380	DMT family transporter	QEN71_19375	paras_004415	protein-codi	NZ_CP1252	chromosom	4314035	4314886	-	852	0	16	1509	12	1262	
QEN71_RS19385	hypothetical protein	QEN71_19380	paras_004416	protein-codi	NZ_CP1252	chromosom	4315049	4315543	-	495	0	6	332	4	316	
QEN71_RS19390	alpha-amylase family protein	QEN71_19385	paras_004417	protein-codi	NZ_CP1252	chromosom	4315746	4317410	-	1665	0	26	2976	19	2291	
QEN71_RS19395	alpha-amylase family glycosyl hydrolase	QEN71_19390	paras_004418	protein-codi	NZ_CP1252	chromosom	4317459	4319033	+	1575	0	46	3734	40	3420	
QEN71_RS19400	NAD(P)H-dependent oxidoreductase	partial;pseudo;QEN71_19395		pseudogene	NZ_CP1252	chromosom	4319332	4319855	+	524	559.0	16	3090	13	2433	
QEN71_RS19405	MarR family transcriptional regulator	QEN71_19400	paras_004420	protein-codi	NZ_CP1252	chromosom	4319915	4320430	-	516	0	7	1205	7	1205	
QEN71_RS19410	feruloyl-CoA synthase	QEN71_19405	paras_004421	protein-codi	NZ_CP1252	chromosom	4320505	4322397	-	1893	0	32	5384	26	3841	
QEN71_RS19415	aldehyde dehydrogenase	QEN71_19410	paras_004422	protein-codi	NZ_CP1252	chromosom	4322523	4323974	-	1452	0	12	861	8	614	
QEN71_RS19420	p-hydroxycinnamoyl CoA hydratase/lyase	QEN71_19415	paras_004423	protein-codi	NZ_CP1252	chromosom	4324036	4324872	-	837	0	17	2061	11	1359	
QEN71_RS19425	3-(3-hydroxy-phenyl)propionate transporter MhpT	QEN71_19420	paras_004424	protein-codi	NZ_CP1252	chromosom	4325305	4326549	+	1245	0	16	1780	13	1358	
QEN71_RS19430	porin	QEN71_19425	paras_004425	protein-codi	NZ_CP1252	chromosom	4326594	4327739	+	1146	0	37	3580	34	3397	
QEN71_RS19435	tannase/feruloyl esterase family alpha/beta hydrolas	QEN71_19430	paras_004426	protein-codi	NZ_CP1252	chromosom	4327750	4329459	+	1710	0	33	3426	27	2272	
QEN71_RS19440	cupin domain-containing protein	QEN71_19435	paras_004427	protein-codi	NZ_CP1252	chromosom	4329667	4330113	+	447	0	16	3043	13	2507	
QEN71_RS19445	LssY C-terminal domain-containing protein	QEN71_19440	paras_004428	protein-codi	NZ_CP1252	chromosom	4330464	4331753	+	1286	0	30	4086	20	2197	
QEN71_RS19450	LssY C-terminal domain-containing protein	QEN71_19445	paras_004429	protein-codi	NZ_CP1252	chromosom	4331750	4332997	+	1244	0	33	5408	29	4917	
QEN71_RS19455	hypothetical protein	QEN71_19450	paras_004430	protein-codi	NZ_CP1252	chromosom	4333065	4334138	+	1074	0	14	2834	12	2361	
QEN71_RS19460	shikimate dehydrogenase	QEN71_19455	paras_004431	protein-codi	NZ_CP1252	chromosom	4335364	4336197	-	834	0	12	1902	9	1503	
QEN71_RS19465	CoA transferase	QEN71_19460	paras_004432	protein-codi	NZ_CP1252	chromosom	4336205	4337416	-	1211	0	16	2234	13	2026	
QEN71_RS19470	MaoC family dehydratase	QEN71_19465	paras_004433	protein-codi	NZ_CP1252	chromosom	4337416	4337874	-	458	0	5	155	4	123	
QEN71_RS19475	VOC family protein	QEN71_19470	paras_004434	protein-codi	NZ_CP1252	chromosom	4337876	4338268	-	389	0	5	708	3	137	
QEN71_RS19480	2-dehydropantoate 2-reductase	QEN71_19475	paras_004435	protein-codi	NZ_CP1252	chromosom	4338265	4339215	-	947	0	10	1452	10	1452	
QEN71_RS19485	LysR family transcriptional regulator	QEN71_19480	paras_004436	protein-codi	NZ_CP1252	chromosom	4339327	4340232	+	906	0	15	1492	6	729	
QEN71_RS19490	tannase/feruloyl esterase family alpha/beta hydrolas	QEN71_19485	paras_004437	protein-codi	NZ_CP1252	chromosom	4340716	4342431	+	1716	0	64	8365	43	5148	
QEN71_RS19495	GntR family transcriptional regulator	QEN71_19490	paras_004438	protein-codi	NZ_CP1252	chromosom	4342547	4343308	-	762	0	14	2525	11	2430	
QEN71_RS19500	enoyl-CoA hydratase/isomerase family protein	QEN71_19495	paras_004439	protein-codi	NZ_CP1252	chromosom	4343365	4344096	+	732	0	11	1369	9	1272	
QEN71_RS19505	MFS transporter	QEN71_19500	paras_004440	protein-codi	NZ_CP1252	chromosom	4344124	4345524	+	1401	0	23	2436	17	1487	
QEN71_RS19510	aldehyde dehydrogenase	QEN71_19505	paras_004441	protein-codi	NZ_CP1252	chromosom	4345543	4347054	+	1512	0	20	1981	14	1612	
QEN71_RS19515	LysR family transcriptional regulator	QEN71_19510	paras_004442	protein-codi	NZ_CP1252	chromosom	4347146	4348078	-	933	0	20	1657	14	1383	
QEN71_RS19520	amidohydrolase family protein	QEN71_19515	paras_004443	protein-codi	NZ_CP1252	chromosom	4348211	4349074	+	864	0	6	421	5	265	
QEN71_RS19525	dicarboxylate/amino acid:cation symporter	QEN71_19520	paras_004444	protein-codi	NZ_CP1252	chromosom	4349261	4350523	+	1263	0	22	1958	20	1703	
QEN71_RS19530	UbiX family flavin prenyltransferase	QEN71_19525	paras_004445	protein-codi	NZ_CP1252	chromosom	4350577	4351179	-	603	0	8	515	5	307	
QEN71_RS19535	UbiD family decarboxylase	QEN71_19530	paras_004446	protein-codi	NZ_CP1252	chromosom	4351592	4353232	+	1641	0	19	1590	15	1244	
QEN71_RS19540	porin	QEN71_19535	paras_004447	protein-codi	NZ_CP1252	chromosom	4353422	4354513	+	1092	0	25	1880	20	1503	
QEN71_RS19545	ABC transporter ATP-binding protein	QEN71_19540	paras_004448	protein-codi	NZ_CP1252	chromosom	4354590	4355423	-	834	0	5	353	5	353	
QEN71_RS19550	ABC transporter substrate-binding protein	QEN71_19545	paras_004449	protein-codi	NZ_CP1252	chromosom	4355482	4356477	-	996	0	18	1365	9	389	
QEN71_RS19555	ABC transporter permease	QEN71_19550	paras_004450	protein-codi	NZ_CP1252	chromosom	4356521	4357312	-	792	0	4	272	3	201	
QEN71_RS19560	alpha/beta hydrolase	QEN71_19555	paras_004451	protein-codi	NZ_CP1252	chromosom	4357764	4358600	+	837	0	13	908	8	726	
QEN71_RS19565	xanthine dehydrogenase family protein subunit M	QEN71_19560	paras_004452	protein-codi	NZ_CP1252	chromosom	4358629	4359525	+	896	0	11	1233	9	1194	
QEN71_RS19570	xanthine dehydrogenase family protein molybdopter	QEN71_19565	paras_004453	protein-codi	NZ_CP1252	chromosom	4359525	4361840	+	2315	0	39	4504	29	3638	
QEN71_RS19575	(2Fe-2S)-binding protein	QEN71_19570	paras_004454	protein-codi	NZ_CP1252	chromosom	4361844	4362341	+	498	0	7	375	7	375	
QEN71_RS19580	MFS transporter	QEN71_19575	paras_004455	protein-codi	NZ_CP1252	chromosom	4362398	4363777	+	1380	0	30	3424	26	2976	
QEN71_RS19585	RidA family protein	QEN71_19580	paras_004456	protein-codi	NZ_CP1252	chromosom	4363780	4364157	+	378	0	7	1090	7	1090	
QEN71_RS19590	XdhC family protein	QEN71_19585	paras_004457	protein-codi	NZ_CP1252	chromosom	4364181	4365212	+	1032	0	11	1979	10	1961	
QEN71_RS19595	class I adenylate-forming enzyme family protein	QEN71_19590	paras_004458	protein-codi	NZ_CP1252	chromosom	4365234	4366865	-	1632	0	20	766	20	766	
QEN71_RS19600	LysR substrate-binding domain-containing protein	QEN71_19595	paras_004459	protein-codi	NZ_CP1252	chromosom	4366870	4367835	-	966	0	22	3459	15	2286	

QEN71_RS19605	PaaI family thioesterase	QEN71_19600	paras_004460	protein-codi	NZ_CP1252	chromosome	4368257	4368694	+	438	0	5	1145	4	1130	
QEN71_RS19610	UbiD family decarboxylase	QEN71_19605	paras_004461	protein-codi	NZ_CP1252	chromosome	4368863	4370320	+	1458	0	23	2204	20	2069	
QEN71_RS19615	carbon monoxide dehydrogenase subunit G	QEN71_19610	paras_004462	protein-codi	NZ_CP1252	chromosome	4370441	4371076	+	636	0	3	249	2	203	
QEN71_RS19620	MFS transporter	QEN71_19615	paras_004463	protein-codi	NZ_CP1252	chromosome	4371104	4372519	+	1416	0	19	1892	17	1880	
QEN71_RS19625	DUF4148 domain-containing protein	QEN71_19620	paras_004464	protein-codi	NZ_CP1252	chromosome	4372719	4373015	+	297	0	3	307	0	0	
QEN71_RS19630	4-carboxy-4-hydroxy-2-oxoadipate aldolase/oxaloa	QEN71_19625	paras_004465	protein-codi	NZ_CP1252	chromosome	4373239	4373922	-	684	402.0	9	1974	3	426	
QEN71_RS19635	amidohydrolase family protein	QEN71_19630	paras_004466	protein-codi	NZ_CP1252	chromosome	4373925	4374950	-	1026	390.0	12	752	9	573	
QEN71_RS19640	porin	QEN71_19635	paras_004467	protein-codi	NZ_CP1252	chromosome	4374999	4376045	-	1047	0	22	985	17	765	
QEN71_RS19645	C4-dicarboxylate transporter DctA	QEN71_19640	paras_004468	protein-codi	NZ_CP1252	chromosome	4376216	4377571	-	1356	0	12	716	12	716	
QEN71_RS19650	hypothetical protein	QEN71_19645	paras_004469	protein-codi	NZ_CP1252	chromosome	4377638	4378552	-	915	0	19	2376	17	2314	
QEN71_RS19655	amidohydrolase family protein	QEN71_19650	paras_004470	protein-codi	NZ_CP1252	chromosome	4378587	4379438	-	852	0	16	1423	13	1395	
QEN71_RS19660	LysR substrate-binding domain-containing protein	QEN71_19655	paras_004471	protein-codi	NZ_CP1252	chromosome	4379578	4380834	+	1257	0	16	1335	11	553	
QEN71_RS19665	LysR family transcriptional regulator	partial;pseudo;QEN71_19660		pseudogene	NZ_CP1252	chromosome	4380924	4381037	-	114	0	1	8	1	8	
QEN71_RS19670	DUF1295 domain-containing protein	QEN71_19665	paras_004473	protein-codi	NZ_CP1252	chromosome	4381207	4381986	+	780	0	12	783	11	714	
QEN71_RS19675	cyclopropane-fatty-acyl-phospholipid synthase fami	QEN71_19670	paras_004474	protein-codi	NZ_CP1252	chromosome	4382018	4383115	+	1098	0	22	1305	17	1001	
QEN71_RS19680	lipocalin family protein	QEN71_19675	paras_004475	protein-codi	NZ_CP1252	chromosome	4383126	4383731	+	606	0	13	1163	12	1140	
QEN71_RS19685	cyclopropane-fatty-acyl-phospholipid synthase fami	QEN71_19680	paras_004476	protein-codi	NZ_CP1252	chromosome	4383764	4384975	-	1212	0	20	1869	17	1587	
QEN71_RS19690	DUF1365 domain-containing protein	QEN71_19685	paras_004477	protein-codi	NZ_CP1252	chromosome	4384978	4385775	-	794	0	13	1282	13	1282	
QEN71_RS19695	FAD-dependent oxidoreductase	QEN71_19690	paras_004478	protein-codi	NZ_CP1252	chromosome	4385772	4387070	-	1295	0	16	1387	13	1043	
QEN71_RS19700	betaine/proline/choline family ABC transporter ATP-	QEN71_19695	paras_004479	protein-codi	NZ_CP1252	chromosome	4387469	4388632	-	1160	0	21	2138	15	1402	
QEN71_RS19705	ABC transporter permease	QEN71_19700	paras_004480	protein-codi	NZ_CP1252	chromosome	4388629	4389282	-	650	0	11	1799	9	1413	
QEN71_RS19710	glycine betaine ABC transporter substrate-binding p	QEN71_19705	paras_004481	protein-codi	NZ_CP1252	chromosome	4389303	4390205	-	899	0	24	3374	23	3359	
QEN71_RS19715	ABC transporter permease	QEN71_19710	paras_004482	protein-codi	NZ_CP1252	chromosome	4390202	4390918	-	713	0	17	2200	16	2121	
QEN71_RS19720	TonB-dependent receptor	QEN71_19715	paras_004483	protein-codi	NZ_CP1252	chromosome	4391391	4393796	+	2406	0	59	5942	49	5214	
QEN71_RS19725	5-methyltetrahydropteroyltriglutamate-- homocystei	QEN71_19720	paras_004484	protein-codi	NZ_CP1252	chromosome	4393861	4396152	-	2292	0	47	5882	36	5076	
QEN71_RS19730	LysR family transcriptional regulator	QEN71_19725	paras_004485	protein-codi	NZ_CP1252	chromosome	4396264	4397172	+	909	0	21	3057	18	2563	
QEN71_RS19735	bifunctional aconitate hydratase 2/2-methylisocitrate	QEN71_19730	paras_004486	protein-codi	NZ_CP1252	chromosome	4397345	4399930	-	2586	0	30	3105	18	1035	
QEN71_RS19740	hypothetical protein	QEN71_19735	paras_004487	protein-codi	NZ_CP1252	chromosome	4400256	4400471	-	216	0	8	686	8	686	
QEN71_RS19745	hypothetical protein	QEN71_19740	paras_004488	protein-codi	NZ_CP1252	chromosome	4400941	4401240	+	300	0	3	409	2	13	
QEN71_RS19750	transposase	QEN71_19745	paras_004489	protein-codi	NZ_CP1252	chromosome	4401294	4402049	-	756	0	28	4357	24	3933	
QEN71_RS19755	AAA family ATPase	QEN71_19750	paras_004490	protein-codi	NZ_CP1252	chromosome	4402419	4402931	+	513	0	9	1235	5	721	
QEN71_RS19760	GFA family protein	QEN71_19755	paras_004491	protein-codi	NZ_CP1252	chromosome	4402954	4403379	-	426	0	5	454	3	429	
QEN71_RS19765	DMT family transporter	QEN71_19760	paras_004492	protein-codi	NZ_CP1252	chromosome	4403465	4404307	-	843	0	21	3963	18	3434	
QEN71_RS19770	transcriptional regulator GcvA	QEN71_19765	paras_004493	protein-codi	NZ_CP1252	chromosome	4404431	4405321	+	891	0	20	5025	17	3544	
QEN71_RS19775	FAD-dependent oxidoreductase	QEN71_19770	paras_004494	protein-codi	NZ_CP1252	chromosome	4405345	4407078	-	1734	0	30	3120	25	3034	
QEN71_RS19780	zinc-binding dehydrogenase	QEN71_19775	paras_004495	protein-codi	NZ_CP1252	chromosome	4407131	4408300	-	1170	0	29	5689	24	5002	
QEN71_RS19785	SGNH/GDSL hydrolase family protein	QEN71_19780	paras_004496	protein-codi	NZ_CP1252	chromosome	4408522	4409544	+	1023	0	26	4272	17	2460	
QEN71_RS19790	DUF4239 domain-containing protein	QEN71_19785	paras_004497	protein-codi	NZ_CP1252	chromosome	4409565	4410344	-	780	0	8	1702	6	873	
QEN71_RS19795	NUDIX hydrolase	QEN71_19790	paras_004498	protein-codi	NZ_CP1252	chromosome	4410483	4410968	-	486	0	6	1403	4	1149	
QEN71_RS19800	oxidoreductase	QEN71_19795	paras_004499	protein-codi	NZ_CP1252	chromosome	4411030	4411866	-	837	0	13	1777	11	1011	
QEN71_RS19805	AraC family transcriptional regulator	QEN71_19800	paras_004500	protein-codi	NZ_CP1252	chromosome	4412022	4412936	+	915	0	20	2659	15	2066	
QEN71_RS19810	2-hydroxycarboxylate transporter family protein	QEN71_19805	paras_004501	protein-codi	NZ_CP1252	chromosome	4412954	4414285	-	1332	0	23	2498	19	2115	
QEN71_RS19815	TIGR00366 family protein	QEN71_19810	paras_004502	protein-codi	NZ_CP1252	chromosome	4414300	4415667	-	1368	0	10	1364	8	1080	
QEN71_RS19820	amidohydrolase family protein	QEN71_19815	paras_004503	protein-codi	NZ_CP1252	chromosome	4415690	4416499	-	810	0	11	1367	7	705	
QEN71_RS19825	CoA transferase	QEN71_19820	paras_004504	protein-codi	NZ_CP1252	chromosome	4416540	4417730	-	1191	0	24	2948	14	2036	
QEN71_RS19830	CoA ester lyase	QEN71_19825	paras_004505	protein-codi	NZ_CP1252	chromosome	4417752	4418645	-	894	0	4	753	3	584	
QEN71_RS19835	LysR family transcriptional regulator	QEN71_19830	paras_004506	protein-codi	NZ_CP1252	chromosome	4418745	4419644	+	900	0	9	999	7	886	
QEN71_RS19840	glyoxalase/bleomycin resistance/dioxygenase famil	QEN71_19835	paras_004507	protein-codi	NZ_CP1252	chromosome	4419712	4420572	+	861	0	22	1852	20	1769	
QEN71_RS19845	hypothetical protein	QEN71_19840	paras_004508	protein-codi	NZ_CP1252	chromosome	4420597	4421163	+	567	0	11	811	5	299	
QEN71_RS19850	hypothetical protein	QEN71_19845	paras_004509	protein-codi	NZ_CP1252	chromosome	4421229	4421498	+	270	0	11	693	10	624	
QEN71_RS19855	sugar-transfer associated ATP-grasp domain-contai	QEN71_19850	paras_004510	protein-codi	NZ_CP1252	chromosome	4421843	4422925	+	1083	0	17	1455	12	951	
QEN71_RS19860	aldo/keto reductase	QEN71_19855	paras_004511	protein-codi	NZ_CP1252	chromosome	4423216	4424151	+	936	0	11	1142	9	1092	
QEN71_RS19865	nuclear transport factor 2 family protein	QEN71_19860	paras_004512	protein-codi	NZ_CP1252	chromosome	4424188	4424589	+	376	0	9	1072	9	1072	
QEN71_RS19870	pirin family protein	QEN71_19865	paras_004513	protein-codi	NZ_CP1252	chromosome	4424564	4425310	-	721	0	19	2495	13	1740	
QEN71_RS19875	SDR family oxidoreductase	QEN71_19870	paras_004514	protein-codi	NZ_CP1252	chromosome	4425340	4426101	-	762	0	11	2067	11	2067	
QEN71_RS19880	LysR family transcriptional regulator	QEN71_19875	paras_004515	protein-codi	NZ_CP1252	chromosome	4426235	4427152	+	918	0	13	1338	12	1152	
QEN71_RS19885	DUF488 domain-containing protein	QEN71_19880	paras_004516	protein-codi	NZ_CP1252	chromosome	4427223	4427756	+	534	0	17	1778	13	1577	
QEN71_RS19890	Zn-dependent hydrolase	QEN71_19885	paras_004517	protein-codi	NZ_CP1252	chromosome	4427805	4429055	+	1251	0	8	1950	6	1034	
QEN71_RS19895	hypothetical protein	QEN71_19890	paras_004518	protein-codi	NZ_CP1252	chromosome	4429398	4429775	+	378	0	7	808	4	631	
QEN71_RS19900	hypothetical protein	QEN71_19895	paras_004519	protein-codi	NZ_CP1252	chromosome	4429991	4430227	+	237	0	4	337	0	0	
QEN71_RS19905	low affinity iron permease family protein	QEN71_19900	paras_004520	protein-codi	NZ_CP1252	chromosome	4430266	4430814	-	549	0	7	436	4	286	
QEN71_RS19910	sensor histidine kinase	QEN71_19905	paras_004521	protein-codi	NZ_CP1252	chromosome	4431122	4432231	+	1110	0	17	1506	12	863	

QEN71_RS19915	DUF2795 domain-containing protein	QEN71_19910	paras_004522	protein-codi	NZ_CP1252	chromosom	4432254	4432475	-		222	0	5	539	2	292	
QEN71_RS19920	CsbD family protein	QEN71_19915	paras_004523	protein-codi	NZ_CP1252	chromosom	4432556	4432822	-		267	0	1	4	0	0	
QEN71_RS19925	phage holin family protein	QEN71_19920	paras_004524	protein-codi	NZ_CP1252	chromosom	4433040	4433414	+		371	0	7	954	6	942	
QEN71_RS19930	hypothetical protein	QEN71_19925	paras_004525	protein-codi	NZ_CP1252	chromosom	4433411	4433725	+		311	0	0	0	0	0	TRUE
QEN71_RS19935	hemerythrin domain-containing protein	QEN71_19930	paras_004526	protein-codi	NZ_CP1252	chromosom	4433773	4434315	+		543	0	6	356	6	356	
QEN71_RS19940	hypothetical protein	QEN71_19935	paras_004527	protein-codi	NZ_CP1252	chromosom	4434388	4434684	+		297	0	5	905	5	905	
QEN71_RS19945	acyl-CoA dehydrogenase family protein	QEN71_19940	paras_004528	protein-codi	NZ_CP1252	chromosom	4434903	4436126	+		1224	0	16	1333	14	1234	
QEN71_RS19950	LLM class flavin-dependent oxidoreductase	QEN71_19945	paras_004529	protein-codi	NZ_CP1252	chromosom	4436189	4437517	+		1329	0	27	4262	19	2681	
QEN71_RS19955	hypothetical protein	QEN71_19950	paras_004530	protein-codi	NZ_CP1252	chromosom	4437553	4437810	-		258	0	2	238	0	0	
QEN71_RS19960	MlaD family protein	QEN71_19955	paras_004531	protein-codi	NZ_CP1252	chromosom	4437904	4439469	-		1566	0	26	2942	20	2216	
QEN71_RS19965	DUF3313 domain-containing protein	QEN71_19960	paras_004532	protein-codi	NZ_CP1252	chromosom	4439599	4440261	-		663	0	16	2321	14	2161	
QEN71_RS19970	TetR/AcrR family transcriptional regulator	QEN71_19965	paras_004533	protein-codi	NZ_CP1252	chromosom	4440534	4441130	+		597	0	5	348	4	317	
QEN71_RS19975	NAD-dependent succinate-semialdehyde dehydrog	QEN71_19970	paras_004534	protein-codi	NZ_CP1252	chromosom	4441218	4442684	-		1467	101.0	32	4635	16	1687	
QEN71_RS19980	4-aminobutyrate--2-oxoglutarate transaminase	QEN71_19975	paras_004535	protein-codi	NZ_CP1252	chromosom	4443357	4444640	-		1284	0	24	4909	18	3968	
QEN71_RS19985	PLP-dependent aminotransferase family protein	QEN71_19980	paras_004536	protein-codi	NZ_CP1252	chromosom	4444761	4446320	+		1560	0	53	12501	46	11906	
QEN71_RS19990	DMT family transporter	QEN71_19985	paras_004537	protein-codi	NZ_CP1252	chromosom	4446337	4447173	-		837	0	30	7703	28	7502	
QEN71_RS19995	hypothetical protein	QEN71_19990	paras_004538	protein-codi	NZ_CP1252	chromosom	4447777	4447947	+		167	135.0	4	794	4	794	
QEN71_RS20000	phosphonate ABC transporter, permease protein Ph	QEN71_19995	paras_004539	protein-codi	NZ_CP1252	chromosom	4447944	4448714	-		767	0	16	1803	10	1164	
QEN71_RS20005	phosphonate ABC transporter substrate-binding pro	QEN71_20000	paras_004540	protein-codi	NZ_CP1252	chromosom	4448783	4449751	-		969	0	13	883	13	883	
QEN71_RS20010	phosphonate ABC transporter ATP-binding protein	QEN71_20005	paras_004541	protein-codi	NZ_CP1252	chromosom	4449806	4450660	-		855	0	5	747	5	747	
QEN71_RS20015	hypothetical protein	QEN71_20010	paras_004542	protein-codi	NZ_CP1252	chromosom	4450859	4451386	-		528	0	8	1043	8	1043	
QEN71_RS20020	M14 family zinc carboxypeptidase	QEN71_20015	paras_004543	protein-codi	NZ_CP1252	chromosom	4451460	4452494	+		1031	0	23	3526	17	3065	
QEN71_RS20025	alpha/beta hydrolase	QEN71_20020	paras_004544	protein-codi	NZ_CP1252	chromosom	4452491	4453327	+		833	0	5	1662	5	1662	
QEN71_RS20030	hypothetical protein	QEN71_20025	paras_004545	protein-codi	NZ_CP1252	chromosom	4453376	4453600	-		225	0	9	614	9	614	
QEN71_RS20035	hypothetical protein	QEN71_20030	paras_004546	protein-codi	NZ_CP1252	chromosom	4454075	4455304	+		1230	0	18	4434	10	3082	
QEN71_RS20040	hypothetical protein	QEN71_20035	paras_004547	protein-codi	NZ_CP1252	chromosom	4455321	4456550	+		1230	0	12	4305	7	2730	
QEN71_RS20045	hypothetical protein	QEN71_20040	paras_004548	protein-codi	NZ_CP1252	chromosom	4456752	4457198	+		447	0	10	1325	7	1063	
QEN71_RS20050	C39 family peptidase	QEN71_20045	paras_004549	protein-codi	NZ_CP1252	chromosom	4457202	4457891	+		690	0	17	3653	13	3005	
QEN71_RS20055	hypothetical protein	QEN71_20050	paras_004550	protein-codi	NZ_CP1252	chromosom	4457900	4459108	+		1209	0	18	2552	13	2192	
QEN71_RS20060	acetate kinase	QEN71_20055	paras_004551	protein-codi	NZ_CP1252	chromosom	4459180	4460604	-		1425	0	36	4824	29	4311	
QEN71_RS20065	NAD-glutamate dehydrogenase	QEN71_20060	paras_004552	protein-codi	NZ_CP1252	chromosom	4461126	4465982	+		4857	0	73	8240	60	6818	
QEN71_RS20070	selenide, water dikinase SelD	QEN71_20065	paras_004553	protein-codi	NZ_CP1252	chromosom	4466119	4467180	+		1062	0	13	1759	12	1660	
QEN71_RS20075	cation diffusion facilitator family transporter	QEN71_20070	paras_004554	protein-codi	NZ_CP1252	chromosom	4467223	4468149	+		927	0	20	3736	18	3608	
QEN71_RS20080	purine nucleoside permease	QEN71_20075	paras_004555	protein-codi	NZ_CP1252	chromosom	4468401	4469468	+		1068	0	27	6031	23	5535	
QEN71_RS20085	DUF4148 domain-containing protein	QEN71_20080	paras_004556	protein-codi	NZ_CP1252	chromosom	4469638	4469946	-		309	0	5	1345	4	1340	
QEN71_RS20090	LysR family transcriptional regulator	QEN71_20085	paras_004557	protein-codi	NZ_CP1252	chromosom	4470138	4471142	-		1005	0	29	5159	23	4515	
QEN71_RS20095	alpha/beta hydrolase	QEN71_20090	paras_004558	protein-codi	NZ_CP1252	chromosom	4471338	4472192	+		855	0	11	686	11	686	
QEN71_RS20100	efflux RND transporter periplasmic adaptor subunit	QEN71_20095	paras_004559	protein-codi	NZ_CP1252	chromosom	4472227	4473516	+		1290	0	17	2150	15	2060	
QEN71_RS20105	efflux RND transporter permease subunit	QEN71_20100	paras_004560	protein-codi	NZ_CP1252	chromosom	4473590	4476775	+		3186	0	59	8797	50	6632	
QEN71_RS20110	efflux transporter outer membrane subunit	QEN71_20105	paras_004561	protein-codi	NZ_CP1252	chromosom	4476790	4478343	+		1554	0	20	2417	18	2165	
QEN71_RS20115	Nramp family divalent metal transporter	QEN71_20110	paras_004562	protein-codi	NZ_CP1252	chromosom	4478569	4479897	+		1329	0	29	5380	22	3066	
QEN71_RS20120	flagellar transcriptional regulator FlhC	QEN71_20115	paras_004563	protein-codi	NZ_CP1252	chromosom	4480000	4480623	-		624	0	17	8028	14	7697	
QEN71_RS20125	flagellar transcriptional regulator FlhD	QEN71_20120	paras_004564	protein-codi	NZ_CP1252	chromosom	4480640	4480960	-		321	0	13	2572	6	1897	
QEN71_RS20130	YeeE/YedE family protein	QEN71_20125	paras_004565	protein-codi	NZ_CP1252	chromosom	4481509	4482723	-		1215	0	36	15137	29	13554	
QEN71_RS20135	2OG-Fe(II) oxygenase	QEN71_20130	paras_004566	protein-codi	NZ_CP1252	chromosom	4482997	4483803	-		807	0	25	14407	20	10284	
QEN71_RS20140	GNAT family N-acetyltransferase	QEN71_20135	paras_004567	protein-codi	NZ_CP1252	chromosom	4483915	4484442	-		528	0	15	6396	15	6396	
QEN71_RS20145	helix-turn-helix transcriptional regulator	QEN71_20140	paras_004568	protein-codi	NZ_CP1252	chromosom	4484451	4485356	-		906	0	5	258	5	258	
QEN71_RS20150	hypothetical protein	QEN71_20145	paras_004569	protein-codi	NZ_CP1252	chromosom	4485460	4485714	+		255	0	8	2916	8	2916	
QEN71_RS20155	nuclear transport factor 2 family protein	QEN71_20150	paras_004570	protein-codi	NZ_CP1252	chromosom	4485912	4486280	+		369	0	9	3452	5	2253	
QEN71_RS20160	hypothetical protein			pseudogene	NZ_CP1252	chromosom	4486319	4486559	+		241	0	6	3190	6	3190	
QEN71_RS20165	hypothetical protein			protein-codi	NZ_CP1252	chromosom	4486649	4486987	+		339	0	2	375	2	375	
QEN71_RS20170	tetratricopeptide repeat-containing glycosyltransfera	QEN71_20165	paras_004573	protein-codi	NZ_CP1252	chromosom	4487110	4488672	+		1563	0	35	7005	29	5698	
QEN71_RS20175	Hsp20/alpha crystallin family protein	QEN71_20170	paras_004574	protein-codi	NZ_CP1252	chromosom	4488744	4489148	-		405	0	2	116	1	93	
QEN71_RS20180	Hsp20/alpha crystallin family protein	QEN71_20175	paras_004575	protein-codi	NZ_CP1252	chromosom	4489164	4489598	-		435	0	15	4916	10	3255	
QEN71_RS20185	DUF72 domain-containing protein	QEN71_20180	paras_004576	protein-codi	NZ_CP1252	chromosom	4489839	4490750	-		912	0	36	9044	30	7683	
QEN71_RS20190	deoxyribose-phosphate aldolase	QEN71_20185	paras_004577	protein-codi	NZ_CP1252	chromosom	4491045	4492055	+		1011	0	24	5728	19	4870	
QEN71_RS20195	aldehyde dehydrogenase family protein	QEN71_20190	paras_004578	protein-codi	NZ_CP1252	chromosom	4492069	4494465	+		2397	0	59	18983	44	16330	
QEN71_RS20200	RbsD/FucU family protein	QEN71_20195	paras_004579	protein-codi	NZ_CP1252	chromosom	4494556	4495026	+		471	0	9	3492	9	3492	
QEN71_RS20205	ribokinase	QEN71_20200	paras_004580	protein-codi	NZ_CP1252	chromosom	4495032	4495991	+		960	0	15	5928	8	2061	
QEN71_RS20210	ABC transporter permease	QEN71_20205	paras_004581	protein-codi	NZ_CP1252	chromosom	4496042	4497061	+		1020	0	24	8250	24	8250	
QEN71_RS20215	autoinducer 2 ABC transporter substrate-binding pr	QEN71_20210	paras_004582	protein-codi	NZ_CP1252	chromosom	4497162	4498166	+		1005	0	17	5077	13	2619	
QEN71_RS20220	sugar ABC transporter ATP-binding protein	QEN71_20215	paras_004583	protein-codi	NZ_CP1252	chromosom	4498245	4499777	+		1533	0	33	9720	24	6014	

QEN71_RS20225	ABC transporter permease	QEN71_20220	paras_004584	protein-codi	NZ_CP1252	chromosom	4499791	4500771	+	981	0	56	15391	43	12409	
QEN71_RS20230	DeoR/GlpR family DNA-binding transcription regula	pseudo;QEN71_20225		pseudogene	NZ_CP1252	chromosom	4501400	4502125	-	726	0	42	12334	34	9877	
QEN71_RS20235	DoxX family protein	QEN71_20230	paras_004586	protein-codi	NZ_CP1252	chromosom	4502348	4502785	+	438	0	11	4018	9	2984	
QEN71_RS20240	hypothetical protein	QEN71_20235	paras_004587	protein-codi	NZ_CP1252	chromosom	4502926	4503198	+	273	0	5	1242	3	828	
QEN71_RS20245	MFS transporter	QEN71_20240	paras_004588	protein-codi	NZ_CP1252	chromosom	4503305	4504801	-	1497	0	23	6961	14	3923	
QEN71_RS20250	MFS transporter	QEN71_20245	paras_004589	protein-codi	NZ_CP1252	chromosom	4504890	4506200	-	1311	0	30	9433	20	7177	
QEN71_RS20255	response regulator transcription factor	QEN71_20250	paras_004590	protein-codi	NZ_CP1252	chromosom	4506465	4507190	+	726	0	19	3664	15	2766	
QEN71_RS20260	dihydrodipicolinate synthase family protein	QEN71_20255	paras_004591	protein-codi	NZ_CP1252	chromosom	4507272	4508177	-	906	0	17	3342	10	2263	
QEN71_RS20265	4-hydroxyproline epimerase	QEN71_20260	paras_004592	protein-codi	NZ_CP1252	chromosom	4508525	4509490	+	966	0	13	3209	10	2518	
QEN71_RS20270	FAD-dependent oxidoreductase	QEN71_20265	paras_004593	protein-codi	NZ_CP1252	chromosom	4509499	4510626	+	1120	0	14	5211	14	5211	
QEN71_RS20275	(2Fe-2S)-binding protein	QEN71_20270	paras_004594	protein-codi	NZ_CP1252	chromosom	4510619	4510891	+	261	0	3	532	3	532	
QEN71_RS20280	FAD-dependent oxidoreductase	QEN71_20275	paras_004595	protein-codi	NZ_CP1252	chromosom	4510888	4512171	+	1280	0	14	2647	14	2647	
QEN71_RS20285	AraC family transcriptional regulator	QEN71_20280	paras_004596	protein-codi	NZ_CP1252	chromosom	4512318	4513103	+	786	0	16	4179	16	4179	
QEN71_RS20290	hypothetical protein	QEN71_20285	paras_004597	protein-codi	NZ_CP1252	chromosom	4513126	4513461	+	336	0	22	11252	15	7325	
QEN71_RS20295	hypothetical protein	QEN71_20290	paras_004598	protein-codi	NZ_CP1252	chromosom	4513474	4513785	-	312	0	7	2489	0	0	
QEN71_RS20300	hypothetical protein	QEN71_20295	paras_004599	protein-codi	NZ_CP1252	chromosom	4513923	4514261	-	339	0	10	3369	5	2188	
QEN71_RS20305	MFS transporter	QEN71_20300	paras_004600	protein-codi	NZ_CP1252	chromosom	4514984	4516399	-	1416	0	40	14724	29	11306	
QEN71_RS20310	SDR family oxidoreductase	QEN71_20305	paras_004601	protein-codi	NZ_CP1252	chromosom	4516507	4517319	-	813	0	23	5801	13	3468	
QEN71_RS20315	DUF190 domain-containing protein	QEN71_20310	paras_004602	protein-codi	NZ_CP1252	chromosom	4517404	4517772	-	369	0	3	141	2	139	
QEN71_RS20320	hypothetical protein	QEN71_20315	paras_004603	protein-codi	NZ_CP1252	chromosom	4517781	4518185	-	405	0	6	1097	6	1097	
QEN71_RS20325	DUF190 domain-containing protein	QEN71_20320	paras_004604	protein-codi	NZ_CP1252	chromosom	4518279	4518605	-	327	0	8	707	5	409	
QEN71_RS20330	class I SAM-dependent methyltransferase	QEN71_20325	paras_004605	protein-codi	NZ_CP1252	chromosom	4518773	4519555	+	783	0	10	2021	1	5	
QEN71_RS20335	TetR/AcrR family transcriptional regulator	QEN71_20330	paras_004606	protein-codi	NZ_CP1252	chromosom	4519564	4520145	-	582	0	6	583	6	583	
QEN71_RS20340	ATP-dependent DNA ligase	QEN71_20335	paras_004607	protein-codi	NZ_CP1252	chromosom	4520220	4521902	-	1679	0	26	3044	16	1984	
QEN71_RS20345	ligase-associated DNA damage response exonucle	QEN71_20340	paras_004608	protein-codi	NZ_CP1252	chromosom	4521899	4522990	-	1088	0	19	2717	16	2004	
QEN71_RS20350	hypothetical protein	QEN71_20345	paras_004609	protein-codi	NZ_CP1252	chromosom	4523176	4523574	-	399	0	8	943	6	429	
QEN71_RS20355	catalase HPII	QEN71_20350	paras_004610	protein-codi	NZ_CP1252	chromosom	4523781	4525883	+	2103	0	28	3327	25	3078	
QEN71_RS20360	DUF3564 family protein	QEN71_20355	paras_004611	protein-codi	NZ_CP1252	chromosom	4525960	4526325	-	366	0	12	2801	5	255	
QEN71_RS20365	ABC transporter ATP-binding protein	QEN71_20360	paras_004612	protein-codi	NZ_CP1252	chromosom	4526687	4527634	+	944	0	21	6063	16	4458	
QEN71_RS20370	ABC transporter permease	QEN71_20365	paras_004613	protein-codi	NZ_CP1252	chromosom	4527631	4528392	+	758	0	27	5041	22	4257	
QEN71_RS20375	sensor histidine kinase	QEN71_20370	paras_004614	protein-codi	NZ_CP1252	chromosom	4528506	4529735	+	1230	0	28	6015	22	4522	
QEN71_RS20380	hypothetical protein	QEN71_20375	paras_004615	protein-codi	NZ_CP1252	chromosom	4529805	4529930	-	126	0	4	1894	3	1890	
QEN71_RS20385	OFA family MFS transporter	QEN71_20380	paras_004616	protein-codi	NZ_CP1252	chromosom	4529953	4531356	-	1404	0	39	11624	32	10600	
QEN71_RS20390	hypothetical protein	QEN71_20385	paras_004617	protein-codi	NZ_CP1252	chromosom	4531958	4532173	+	216	0	4	2589	2	1482	
QEN71_RS20395	GntR family transcriptional regulator	QEN71_20390	paras_004618	protein-codi	NZ_CP1252	chromosom	4532270	4532890	+	621	0	15	6192	12	4886	
QEN71_RS20400	succinate--CoA ligase subunit alpha	QEN71_20395	paras_004619	protein-codi	NZ_CP1252	chromosom	4532967	4533839	-	873	0	17	5804	10	4287	
QEN71_RS20405	ADP-forming succinate--CoA ligase subunit beta	QEN71_20400	paras_004620	protein-codi	NZ_CP1252	chromosom	4533841	4535064	-	1224	101.0	22	8420	16	6490	
QEN71_RS20410	formyl-CoA transferase	QEN71_20405	paras_004621	protein-codi	NZ_CP1252	chromosom	4535371	4536618	+	1248	329.0	24	5006	24	5006	
QEN71_RS20415	NADP transhydrogenase subunit beta	QEN71_20410	paras_004622	protein-codi	NZ_CP1252	chromosom	4536688	4537245	+	558	0	13	2573	9	1162	
QEN71_RS20420	hypothetical protein	QEN71_20415	paras_004623	protein-codi	NZ_CP1252	chromosom	4537345	4537602	-	258	0	4	213	2	179	
QEN71_RS20425	LysR family transcriptional regulator	QEN71_20420	paras_004624	protein-codi	NZ_CP1252	chromosom	4537699	4538643	-	945	0	14	833	14	833	
QEN71_RS20430	oxalate/formate MFS antiporter	QEN71_20425	paras_004625	protein-codi	NZ_CP1252	chromosom	4538992	4540332	+	1341	0	23	3979	15	3616	
QEN71_RS20435	LysR family transcriptional regulator	QEN71_20430	paras_004626	protein-codi	NZ_CP1252	chromosom	4540366	4541388	+	1023	0	14	3858	6	1092	
QEN71_RS20440	aldehyde dehydrogenase family protein	QEN71_20435	paras_004627	protein-codi	NZ_CP1252	chromosom	4541459	4542910	+	1452	0	24	4392	12	2603	
QEN71_RS20445	LysE family translocator	QEN71_20440	paras_004628	protein-codi	NZ_CP1252	chromosom	4542987	4543610	-	624	0	3	342	3	342	
QEN71_RS20450	hypothetical protein	QEN71_20445	paras_004629	protein-codi	NZ_CP1252	chromosom	4543759	4544316	-	558	0	10	636	8	469	
QEN71_RS20455	hypothetical protein	QEN71_20450	paras_004630	protein-codi	NZ_CP1252	chromosom	4544537	4545325	+	789	0	18	2087	15	1539	
QEN71_RS20460	glycosyltransferase family 4 protein	QEN71_20455	paras_004631	protein-codi	NZ_CP1252	chromosom	4545329	4546258	+	930	0	12	1622	6	457	
QEN71_RS20465	M23 family metallopeptidase	QEN71_20460	paras_004632	protein-codi	NZ_CP1252	chromosom	4546505	4547893	+	1389	0	29	6649	29	6649	
QEN71_RS20470	DUF2252 domain-containing protein	QEN71_20465	paras_004633	protein-codi	NZ_CP1252	chromosom	4548166	4549587	+	1422	0	26	6774	20	5840	
QEN71_RS20475	GlsB/YeaQ/YmgE family stress response membrane	QEN71_20470	paras_004634	protein-codi	NZ_CP1252	chromosom	4549682	4549933	+	252	0	9	2784	4	2311	
QEN71_RS20480	polysaccharide deacetylase family protein	QEN71_20475	paras_004635	protein-codi	NZ_CP1252	chromosom	4549968	4550828	-	861	0	2	164	0	0	TRUE
QEN71_RS20485	LysR family transcriptional regulator	QEN71_20480	paras_004636	protein-codi	NZ_CP1252	chromosom	4551017	4551892	+	876	0	0	0	0	0	TRUE
QEN71_RS20490	D-amino acid dehydrogenase	QEN71_20485	paras_004637	protein-codi	NZ_CP1252	chromosom	4552007	4553257	+	1251	0	27	5138	23	4761	
QEN71_RS20495	glutamate/aspartate ABC transporter substrate-bind	QEN71_20490	paras_004638	protein-codi	NZ_CP1252	chromosom	4553297	4554196	+	900	0	13	867	11	772	
QEN71_RS20500	SDR family oxidoreductase	QEN71_20495	paras_004639	protein-codi	NZ_CP1252	chromosom	4554246	4555022	+	777	0	10	1230	8	980	
QEN71_RS20505	oxidoreductase-like domain-containing protein	QEN71_20500	paras_004640	protein-codi	NZ_CP1252	chromosom	4555116	4555361	+	246	0	4	316	4	316	
QEN71_RS20510	NAD-dependent protein deacetylase	QEN71_20505	paras_004641	protein-codi	NZ_CP1252	chromosom	4555396	4556241	+	846	0	4	729	4	729	
QEN71_RS20515	pyridoxamine 5'-phosphate oxidase family protein	QEN71_20510	paras_004642	protein-codi	NZ_CP1252	chromosom	4556283	4556888	+	606	0	14	1849	8	866	
QEN71_RS20520	DUF938 domain-containing protein	QEN71_20515	paras_004643	protein-codi	NZ_CP1252	chromosom	4556894	4557508	+	615	0	9	1425	9	1425	
QEN71_RS20525	NADP-dependent phosphogluconate dehydrogenas	QEN71_20520	paras_004644	protein-codi	NZ_CP1252	chromosom	4557819	4559228	+	1410	0	37	15720	31	14394	
QEN71_RS20530	DUF4148 domain-containing protein	QEN71_20525	paras_004645	protein-codi	NZ_CP1252	chromosom	4559540	4559860	+	321	0	9	1508	6	634	

QEN71_RS20535	DUF2968 domain-containing protein	QEN71_20530	paras_004646	protein-codi	NZ_CP1252	chromosom	4559999	4560754	-	756	0	9	589	8	574	
QEN71_RS20540	hypothetical protein	QEN71_20535	paras_004647	protein-codi	NZ_CP1252	chromosom	4560810	4561130	-	321	0	9	1488	6	917	
QEN71_RS20545	cupin	QEN71_20540	paras_004648	protein-codi	NZ_CP1252	chromosom	4561369	4561680	+	312	0	7	3550	4	2791	
QEN71_RS20550	peptidoglycan DD-metalloendopeptidase family prot	QEN71_20545	paras_004649	protein-codi	NZ_CP1252	chromosom	4561964	4562707	+	728	0	16	4052	12	1593	
QEN71_RS20555	hypothetical protein	QEN71_20550	paras_004650	protein-codi	NZ_CP1252	chromosom	4562692	4562946	+	235	0	1	26	0	0	
QEN71_RS20560	hypothetical protein	QEN71_20555	paras_004651	protein-codi	NZ_CP1252	chromosom	4562943	4563221	+	275	0	7	1837	6	1833	
QEN71_RS20565	divalent metal cation transporter	QEN71_20560	paras_004652	protein-codi	NZ_CP1252	chromosom	4563464	4565107	-	1644	0	34	8422	31	8046	
QEN71_RS20570	hypothetical protein	QEN71_20565	paras_004653	protein-codi	NZ_CP1252	chromosom	4565149	4565313	-	165	0	9	2770	8	2749	
QEN71_RS20575	potassium transporter Kup	QEN71_20570	paras_004654	protein-codi	NZ_CP1252	chromosom	4566145	4568067	+	1923	0	44	13675	36	11553	
QEN71_RS20580	nitrile hydratase subunit alpha	QEN71_20575	paras_004655	protein-codi	NZ_CP1252	chromosom	4568070	4568735	+	662	0	16	5370	16	5370	
QEN71_RS20585	nitrile hydratase subunit beta	QEN71_20580	paras_004656	protein-codi	NZ_CP1252	chromosom	4568732	4569397	+	658	0	13	4983	11	4274	
QEN71_RS20590	nitrile hydratase accessory protein	QEN71_20585	paras_004657	protein-codi	NZ_CP1252	chromosom	4569394	4569771	+	374	0	5	1504	5	1504	
QEN71_RS20595	hypothetical protein	QEN71_20590	paras_004658	protein-codi	NZ_CP1252	chromosom	4569807	4569953	-	147	0	0	0	0	0	
QEN71_RS20600	PIG-L family deacetylase	QEN71_20595	paras_004659	protein-codi	NZ_CP1252	chromosom	4570109	4570825	-	717	0	19	6351	14	5670	
QEN71_RS20605	nucleotidyltransferase family protein	QEN71_20600	paras_004660	protein-codi	NZ_CP1252	chromosom	4571107	4571694	-	588	0	20	7469	14	3902	
QEN71_RS20610	IS481 family transposase	QEN71_20605	paras_004661	protein-codi	NZ_CP1252	chromosom	4572240	4573379	-	1140	2266.0	13	141	10	102	
QEN71_RS20615	xanthine dehydrogenase family protein molybdopter	QEN71_20610	paras_004662	protein-codi	NZ_CP1252	chromosom	4573961	4576174	-	2210	0	63	10445	45	6864	
QEN71_RS20620	xanthine dehydrogenase family protein subunit M	QEN71_20615	paras_004663	protein-codi	NZ_CP1252	chromosom	4576171	4577181	-	1007	0	21	3733	21	3733	
QEN71_RS20625	2Fe-2S iron-sulfur cluster-binding protein	QEN71_20620	paras_004664	protein-codi	NZ_CP1252	chromosom	4577193	4577975	-	783	0	12	2691	8	792	
QEN71_RS20630	aspartate carbamoyltransferase	QEN71_20625	paras_004665	protein-codi	NZ_CP1252	chromosom	4578296	4579588	-	1275	0	26	4552	21	3554	
QEN71_RS20635	hypothetical protein	QEN71_20630	paras_004666	protein-codi	NZ_CP1252	chromosom	4579571	4579843	+	255	0	3	118	3	118	
QEN71_RS20640	4-hydroxy-tetrahydrodipicolinate synthase	QEN71_20635	paras_004667	protein-codi	NZ_CP1252	chromosom	4579971	4580834	+	864	0	15	3143	15	3143	
QEN71_RS20645	M23 family metallopeptidase	QEN71_20640	paras_004668	protein-codi	NZ_CP1252	chromosom	4581070	4582146	+	1069	0	8	605	6	464	
QEN71_RS20650	hypothetical protein	QEN71_20645	paras_004669	protein-codi	NZ_CP1252	chromosom	4582139	4582510	+	364	0	2	312	2	312	
QEN71_RS20655	polymer-forming cytoskeletal protein	QEN71_20650	paras_004670	protein-codi	NZ_CP1252	chromosom	4582548	4582994	+	447	0	10	2857	9	2594	
QEN71_RS20660	EAL domain-containing protein	QEN71_20655	paras_004671	protein-codi	NZ_CP1252	chromosom	4583014	4585083	-	2070	0	31	3971	23	3011	
QEN71_RS20665	rhodanese-like domain-containing protein	QEN71_20660	paras_004672	protein-codi	NZ_CP1252	chromosom	4585285	4585719	-	435	0	12	1519	9	1477	
QEN71_RS20670	transcriptional regulator FtrA	QEN71_20665	paras_004673	protein-codi	NZ_CP1252	chromosom	4585817	4586773	+	957	0	20	3094	17	2915	
QEN71_RS20675	nitrogen fixation protein NifQ	QEN71_20670	paras_004674	protein-codi	NZ_CP1252	chromosom	4586796	4587368	+	573	0	3	650	0	0	
QEN71_RS20680	ABC transporter substrate-binding protein	QEN71_20675	paras_004675	protein-codi	NZ_CP1252	chromosom	4587591	4588658	+	1068	0	26	3456	24	3315	
QEN71_RS20685	recombination-associated protein RdgC	QEN71_20680	paras_004676	protein-codi	NZ_CP1252	chromosom	4588760	4589674	+	915	0	16	1450	11	1129	
QEN71_RS20690	hypothetical protein	QEN71_20685	paras_004677	protein-codi	NZ_CP1252	chromosom	4590258	4590479	-	222	0	16	1746	14	1543	
QEN71_RS20695	glutamate/aspartate ABC transporter substrate-bind	QEN71_20690	paras_004678	protein-codi	NZ_CP1252	chromosom	4590611	4591519	-	909	0	16	1869	10	1202	
QEN71_RS20700	hypothetical protein	QEN71_20695	paras_004679	protein-codi	NZ_CP1252	chromosom	4591784	4592212	+	429	0	11	1402	11	1402	
QEN71_RS20705	LysR family transcriptional regulator	QEN71_20700	paras_004680	protein-codi	NZ_CP1252	chromosom	4592231	4593163	-	933	0	10	1340	6	530	
QEN71_RS20710	NmrA family NAD(P)-binding protein	QEN71_20705	paras_004681	protein-codi	NZ_CP1252	chromosom	4593296	4594159	+	864	0	22	2591	16	1669	
QEN71_RS20715	FdhF/YdeP family oxidoreductase	QEN71_20710	paras_004682	protein-codi	NZ_CP1252	chromosom	4594778	4597099	-	2322	0	61	9527	38	6494	
QEN71_RS20720	PLP-dependent aminotransferase family protein	QEN71_20715	paras_004683	protein-codi	NZ_CP1252	chromosom	4597290	4598717	+	1428	0	40	6838	25	3572	
QEN71_RS20725	ArsR family transcriptional regulator	QEN71_20720	paras_004684	protein-codi	NZ_CP1252	chromosom	4598734	4599042	+	309	0	5	1171	5	1171	
QEN71_RS20730	FUSC family protein	QEN71_20725	paras_004685	protein-codi	NZ_CP1252	chromosom	4599061	4600215	-	1155	0	20	3221	18	2931	
QEN71_RS20735	LysE family translocator	QEN71_20730	paras_004686	protein-codi	NZ_CP1252	chromosom	4600247	4600858	-	612	0	4	907	4	907	
QEN71_RS20740	GNAT family N-acetyltransferase	QEN71_20735	paras_004687	protein-codi	NZ_CP1252	chromosom	4601004	4601534	+	531	0	10	1613	9	1586	
QEN71_RS20745	alkene reductase	QEN71_20740	paras_004688	protein-codi	NZ_CP1252	chromosom	4601633	4602694	-	1062	0	23	4975	17	4298	
QEN71_RS20750	metalloregulator ArsR/SmtB family transcription fact	QEN71_20745	paras_004689	protein-codi	NZ_CP1252	chromosom	4602774	4603103	-	330	0	5	490	3	70	
QEN71_RS20755	MFS transporter	QEN71_20750	paras_004690	protein-codi	NZ_CP1252	chromosom	4603236	4604453	-	1218	0	24	5385	18	4220	
QEN71_RS20760	isochorismatase family cysteine hydrolase	QEN71_20755	paras_004691	protein-codi	NZ_CP1252	chromosom	4604473	4605042	-	570	0	13	1789	11	1721	
QEN71_RS20765	inorganic phosphate transporter	QEN71_20760	paras_004692	protein-codi	NZ_CP1252	chromosom	4605149	4606555	-	1407	0	23	2242	19	1905	
QEN71_RS20770	ABC transporter transmembrane domain-containing	QEN71_20765	paras_004693	protein-codi	NZ_CP1252	chromosom	4606688	4608499	+	1812	0	30	1651	23	1219	
QEN71_RS20775	hypothetical protein	QEN71_20770	paras_004694	protein-codi	NZ_CP1252	chromosom	4608678	4609718	+	983	0	20	3939	20	3939	
QEN71_RS20780	hypothetical protein	QEN71_20775	paras_004695	protein-codi	NZ_CP1252	chromosom	4609661	4610014	+	296	0	5	249	5	249	
QEN71_RS20785	alanine dehydrogenase	QEN71_20780	paras_004696	protein-codi	NZ_CP1252	chromosom	4610055	4611173	-	1119	0	18	3131	15	2165	
QEN71_RS20790	phosphonoacetaldehyde dehydrogenase	QEN71_20785	paras_004697	protein-codi	NZ_CP1252	chromosom	4611240	4612697	-	1458	0	34	4292	18	1930	
QEN71_RS20795	phosphonoacetate hydrolase	QEN71_20790	paras_004698	protein-codi	NZ_CP1252	chromosom	4612711	4613946	-	1236	0	30	4492	22	3385	
QEN71_RS20800	putative 2-aminoethylphosphonate ABC transporter	QEN71_20795	paras_004699	protein-codi	NZ_CP1252	chromosom	4614164	4615219	+	1056	0	18	1953	14	1405	
QEN71_RS20805	putative 2-aminoethylphosphonate ABC transporter	QEN71_20800	paras_004700	protein-codi	NZ_CP1252	chromosom	4615330	4616421	+	1088	0	7	624	4	155	
QEN71_RS20810	putative 2-aminoethylphosphonate ABC transporter	QEN71_20805	paras_004701	protein-codi	NZ_CP1252	chromosom	4616418	4618145	+	1724	0	26	2658	26	2658	
QEN71_RS20815	2-aminoethylphosphonate--pyruvate transaminase	QEN71_20810	paras_004702	protein-codi	NZ_CP1252	chromosom	4618180	4619310	+	1131	0	17	1005	17	1005	
QEN71_RS20820	phosphonate utilization associated transcriptional re	QEN71_20815	paras_004703	protein-codi	NZ_CP1252	chromosom	4619369	4620097	-	729	0	11	1969	10	1960	
QEN71_RS20825	NmrA family NAD(P)-binding protein	QEN71_20820	paras_004704	protein-codi	NZ_CP1252	chromosom	4620238	4621119	-	882	0	20	1383	15	1163	
QEN71_RS20830	TetR family transcriptional regulator	QEN71_20825	paras_004705	protein-codi	NZ_CP1252	chromosom	4621209	4621781	-	573	0	6	1411	4	1145	
QEN71_RS20835	permease	QEN71_20830	paras_004706	protein-codi	NZ_CP1252	chromosom	4621886	4623067	-	1182	0	16	6223	16	6223	
QEN71_RS20840	DUF1109 domain-containing protein	QEN71_20835	paras_004707	protein-codi	NZ_CP1252	chromosom	4623146	4623787	-	638	0	6	408	4	306	

QEN71_RS20845	sigma-70 family RNA polymerase sigma factor	QEN71_20840	paras_004708	protein-codi	NZ_CP1252	chromosom	4623784	4624329	-	542	0	7	869	7	869	
QEN71_RS20850	cytochrome b/b6 domain-containing protein	QEN71_20845	paras_004709	protein-codi	NZ_CP1252	chromosom	4624625	4625263	+	639	0	12	2372	8	1001	
QEN71_RS20855	molybdopterin-dependent oxidoreductase	QEN71_20850	paras_004710	protein-codi	NZ_CP1252	chromosom	4625285	4626061	+	777	0	28	3837	22	2970	
QEN71_RS20860	pentapeptide MXKDX repeat protein	QEN71_20855	paras_004711	protein-codi	NZ_CP1252	chromosom	4626127	4626360	+	234	0	4	410	2	242	
QEN71_RS20865	class I SAM-dependent methyltransferase	QEN71_20860	paras_004712	protein-codi	NZ_CP1252	chromosom	4626416	4627477	-	1062	0	12	1790	10	1302	
QEN71_RS20870	MarR family winged helix-turn-helix transcriptional r	QEN71_20865	paras_004713	protein-codi	NZ_CP1252	chromosom	4627661	4628080	+	420	0	5	75	2	45	
QEN71_RS20875	chloride channel protein	QEN71_20870	paras_004714	protein-codi	NZ_CP1252	chromosom	4628173	4629480	+	1308	0	21	2483	15	1921	
QEN71_RS20880	TetR/AcrR family transcriptional regulator	QEN71_20875	paras_004715	protein-codi	NZ_CP1252	chromosom	4629569	4630159	+	591	0	9	596	8	594	
QEN71_RS20885	cysteine hydrolase	QEN71_20880	paras_004716	protein-codi	NZ_CP1252	chromosom	4630245	4630973	+	729	0	15	2693	13	2538	
QEN71_RS20890	SulP family inorganic anion transporter	QEN71_20885	paras_004717	protein-codi	NZ_CP1252	chromosom	4630989	4633112	-	2124	0	29	4171	25	3774	
QEN71_RS20895	adenylate/guanylate cyclase domain-containing pro	QEN71_20890	paras_004718	protein-codi	NZ_CP1252	chromosom	4633436	4636708	+	3267	0	36	4795	26	3482	
QEN71_RS20900	ParB-like protein	QEN71_20895	paras_004719	protein-codi	NZ_CP1252	chromosom	4636703	4637305	-	597	0	9	1257	9	1257	
QEN71_RS20905	sensor domain-containing diguanylate cyclase	QEN71_20900	paras_004720	protein-codi	NZ_CP1252	chromosom	4637376	4638911	-	1536	0	24	3545	15	1856	
QEN71_RS20910	PqiC family protein	QEN71_20905	paras_004721	protein-codi	NZ_CP1252	chromosom	4639171	4639770	-	596	0	13	2396	10	1425	
QEN71_RS20915	MlaD family protein	QEN71_20910	paras_004722	protein-codi	NZ_CP1252	chromosom	4639767	4641062	-	1288	0	14	1761	12	1624	
QEN71_RS20920	paraquat-inducible protein A	QEN71_20915	paras_004723	protein-codi	NZ_CP1252	chromosom	4641059	4642264	-	1202	0	18	1917	17	1519	
QEN71_RS20925	hypothetical protein	QEN71_20920	paras_004724	protein-codi	NZ_CP1252	chromosom	4642369	4642566	-	198	0	8	2161	1	2	
QEN71_RS20930	hypothetical protein	QEN71_20925	paras_004725	protein-codi	NZ_CP1252	chromosom	4642714	4643187	+	466	0	9	1004	7	969	
QEN71_RS20935	sorbosone dehydrogenase family protein	QEN71_20930	paras_004726	protein-codi	NZ_CP1252	chromosom	4643180	4644499	+	1312	0	32	4965	30	4678	
QEN71_RS20940	GNAT family N-acetyltransferase	QEN71_20935		protein-codi	NZ_CP1252	chromosom	4644611	4645153	+	543	0	8	1516	8	1516	
QEN71_RS20945	hypothetical protein	QEN71_20940		protein-codi	NZ_CP1252	chromosom	4645181	4645321	+	141	0	3	361	3	361	
QEN71_RS20950	sensor histidine kinase	QEN71_20945	paras_004727	protein-codi	NZ_CP1252	chromosom	4645334	4645630	+	297	0	1	25	1	25	
QEN71_RS20955	dihydrofolate reductase family protein	QEN71_20950	paras_004728	protein-codi	NZ_CP1252	chromosom	4645643	4646260	-	618	0	13	1090	12	1061	
QEN71_RS20960	glycogen/starch/alpha-glucan phosphorylase	pseudo;QEN71_20955		pseudogene	NZ_CP1252	chromosom	4646335	4648827	-	2493	0	37	3849	21	1963	
QEN71_RS20965	LysR family transcriptional regulator	QEN71_20960	paras_004730	protein-codi	NZ_CP1252	chromosom	4648923	4649852	-	930	0	8	913	5	647	
QEN71_RS20970	response regulator transcription factor	pseudo;QEN71_20965		pseudogene	NZ_CP1252	chromosom	4650159	4650928	+	770	0	11	795	7	232	
QEN71_RS20975	DUF3828 domain-containing protein	QEN71_20970	paras_004732	protein-codi	NZ_CP1252	chromosom	4650991	4651428	-	438	0	7	180	6	175	
QEN71_RS20980	CHAP domain-containing protein	QEN71_20975	paras_004733	protein-codi	NZ_CP1252	chromosom	4651452	4651823	-	372	0	7	661	2	119	
QEN71_RS20985	energy transducer TonB	QEN71_20980	paras_004734	protein-codi	NZ_CP1252	chromosom	4651998	4652366	+	369	0	11	1035	8	577	
QEN71_RS20990	quinone oxidoreductase	QEN71_20985	paras_004735	protein-codi	NZ_CP1252	chromosom	4652497	4653465	-	969	0	10	585	9	582	
QEN71_RS20995	alpha/beta hydrolase	QEN71_20990	paras_004736	protein-codi	NZ_CP1252	chromosom	4653482	4654225	-	744	0	5	572	5	572	
QEN71_RS21000	DUF4148 domain-containing protein	QEN71_20995	paras_004737	protein-codi	NZ_CP1252	chromosom	4654356	4654685	-	330	0	5	421	3	288	
QEN71_RS21005	cytochrome c biogenesis protein DipZ	QEN71_21000	paras_004738	protein-codi	NZ_CP1252	chromosom	4654994	4656751	-	1758	0	21	2716	17	2206	
QEN71_RS21010	response regulator	QEN71_21005	paras_004739	protein-codi	NZ_CP1252	chromosom	4656937	4657692	-	756	0	5	670	2	360	
QEN71_RS21015	alpha/beta hydrolase	QEN71_21010	paras_004740	protein-codi	NZ_CP1252	chromosom	4657741	4658784	-	1044	102.0	28	2180	22	1710	
QEN71_RS21020	FAD-dependent oxidoreductase	QEN71_21015	paras_004741	protein-codi	NZ_CP1252	chromosom	4659032	4660687	-	1656	0	24	2775	23	2749	
QEN71_RS21025	epoxide hydrolase	QEN71_21020	paras_004742	protein-codi	NZ_CP1252	chromosom	4660993	4662264	+	1272	0	25	2056	18	1675	
QEN71_RS21030	trifunctional serine/threonine-protein kinase/ATP-bi	QEN71_21025	paras_004743	protein-codi	NZ_CP1252	chromosom	4662406	4667502	+	5097	0	81	9911	69	7161	
QEN71_RS21035	response regulator	QEN71_21030	paras_004744	protein-codi	NZ_CP1252	chromosom	4667614	4668276	+	663	0	5	646	3	365	
QEN71_RS21040	response regulator	QEN71_21035	paras_004745	protein-codi	NZ_CP1252	chromosom	4668411	4668794	+	384	0	7	904	4	641	
QEN71_RS21045	BON domain-containing protein	QEN71_21040	paras_004746	protein-codi	NZ_CP1252	chromosom	4669006	4669395	+	390	0	0	0	0	0	TRUE
QEN71_RS21050	DUF3331 domain-containing protein	QEN71_21045	paras_004747	protein-codi	NZ_CP1252	chromosom	4669502	4669951	+	450	0	11	790	7	333	
QEN71_RS21055	amidase family protein	QEN71_21050	paras_004748	protein-codi	NZ_CP1252	chromosom	4669991	4671391	+	1401	0	10	892	8	793	
QEN71_RS21060	universal stress protein	QEN71_21055	paras_004749	protein-codi	NZ_CP1252	chromosom	4671443	4671892	-	450	0	8	1057	3	326	
QEN71_RS21065	sigma factor-like helix-turn-helix DNA-binding protei	QEN71_21060	paras_004750	protein-codi	NZ_CP1252	chromosom	4672028	4672435	-	408	0	4	306	4	306	
QEN71_RS21070	aldehyde dehydrogenase	QEN71_21065	paras_004751	protein-codi	NZ_CP1252	chromosom	4672929	4674398	+	1470	0	16	1711	14	1533	
QEN71_RS21075	AraC family transcriptional regulator	QEN71_21070	paras_004752	protein-codi	NZ_CP1252	chromosom	4674451	4675404	+	954	0	9	447	6	294	
QEN71_RS21080	cupin domain-containing protein	QEN71_21075	paras_004753	protein-codi	NZ_CP1252	chromosom	4675427	4675882	+	456	0	17	2277	6	480	
QEN71_RS21085	alpha/beta hydrolase	QEN71_21080	paras_004754	protein-codi	NZ_CP1252	chromosom	4676117	4677142	+	1026	102.0	22	3808	20	3609	
QEN71_RS21090	cytochrome P460 family protein	QEN71_21085	paras_004755	protein-codi	NZ_CP1252	chromosom	4677165	4677695	+	531	0	14	1447	8	772	
QEN71_RS21095	dihydroxy-acid dehydratase	QEN71_21090	paras_004756	protein-codi	NZ_CP1252	chromosom	4677896	4679755	+	1860	0	29	4407	24	4192	
QEN71_RS21100	TIGR03118 family protein	QEN71_21095	paras_004757	protein-codi	NZ_CP1252	chromosom	4679837	4680934	-	1098	0	11	709	6	471	
QEN71_RS21105	condensation protein	QEN71_21100	paras_004758	protein-codi	NZ_CP1252	chromosom	4681274	4682560	+	1287	0	12	930	5	588	
QEN71_RS21110	hypothetical protein	QEN71_21105	paras_004759	protein-codi	NZ_CP1252	chromosom	4682568	4682978	-	411	0	10	1118	10	1118	
QEN71_RS21115	sulfonate ABC transporter substrate-binding protein	QEN71_21110	paras_004760	protein-codi	NZ_CP1252	chromosom	4683062	4684039	-	978	0	16	2312	16	2312	
QEN71_RS21120	MetQ/NlpA family lipoprotein	QEN71_21115	paras_004761	protein-codi	NZ_CP1252	chromosom	4684051	4684878	-	828	0	18	2700	12	1932	
QEN71_RS21125	PepSY-associated TM helix domain-containing prot	QEN71_21120	paras_004762	protein-codi	NZ_CP1252	chromosom	4685188	4686285	-	1098	0	29	5072	26	4510	
QEN71_RS21130	TonB-dependent siderophore receptor	QEN71_21125	paras_004763	protein-codi	NZ_CP1252	chromosom	4686321	4688468	-	2148	0	66	10687	49	7927	
QEN71_RS21135	hypothetical protein	QEN71_21130	paras_004764	protein-codi	NZ_CP1252	chromosom	4688774	4689025	+	252	0	9	1322	3	638	
QEN71_RS21140	hypothetical protein	QEN71_21135	paras_004765	protein-codi	NZ_CP1252	chromosom	4689041	4689796	-	756	0	20	1859	16	1395	
QEN71_RS21145	HAMP domain-containing sensor histidine kinase	QEN71_21140	paras_004766	protein-codi	NZ_CP1252	chromosom	4689887	4691194	-	1308	0	11	1882	5	458	
QEN71_RS21150	LysR family transcriptional regulator	QEN71_21145	paras_004767	protein-codi	NZ_CP1252	chromosom	4691372	4692280	-	909	0	16	2330	11	2205	

QEN71_RS21155	acyl-CoA dehydrogenase family protein	QEN71_21150	paras_004768	protein-codi	NZ_CP1252	chromosom	4692362	4693612	-	1251	0	7	1268	5	808	
QEN71_RS21160	SDR family oxidoreductase	QEN71_21155	paras_004769	protein-codi	NZ_CP1252	chromosom	4693634	4694398	-	765	0	20	3508	12	2820	
QEN71_RS21165	phosphotransferase	QEN71_21160	paras_004770	protein-codi	NZ_CP1252	chromosom	4694439	4695467	-	1029	0	25	3085	22	2831	
QEN71_RS21170	histidine phosphatase family protein	QEN71_21165	paras_004771	protein-codi	NZ_CP1252	chromosom	4695492	4696166	-	675	0	15	2383	6	1292	
QEN71_RS21175	hypothetical protein	QEN71_21170	paras_004772	protein-codi	NZ_CP1252	chromosom	4696401	4696634	-	234	0	0	0	0	0	
QEN71_RS21180	Lrp/AsnC family transcriptional regulator	QEN71_21175	paras_004773	protein-codi	NZ_CP1252	chromosom	4696733	4697197	-	465	0	15	8183	15	8183	
QEN71_RS21185	LysE family translocator	QEN71_21180	paras_004774	protein-codi	NZ_CP1252	chromosom	4697332	4697961	+	630	0	13	4244	10	1846	
QEN71_RS21190	DUF1289 domain-containing protein	QEN71_21185	paras_004775	protein-codi	NZ_CP1252	chromosom	4697999	4698172	-	174	0	5	1830	3	325	
QEN71_RS21195	YnfA family protein	QEN71_21190	paras_004776	protein-codi	NZ_CP1252	chromosom	4698349	4698669	+	321	0	15	6327	12	4410	
QEN71_RS21200	DUF1993 domain-containing protein	QEN71_21195	paras_004777	protein-codi	NZ_CP1252	chromosom	4698737	4699249	-	513	0	15	3586	11	3161	
QEN71_RS21205	hypothetical protein	QEN71_21200		protein-codi	NZ_CP1252	chromosom	4699379	4699567	-	187	0	4	947	4	947	
QEN71_RS21210	S53 family peptidase	QEN71_21205	paras_004778	protein-codi	NZ_CP1252	chromosom	4699566	4701128	+	1561	0	28	8992	16	4394	
QEN71_RS21215	peptidase S1	QEN71_21210	paras_004779	protein-codi	NZ_CP1252	chromosom	4701250	4702914	+	1665	0	62	18569	53	15502	
QEN71_RS21220	hypothetical protein	QEN71_21215	paras_004780	protein-codi	NZ_CP1252	chromosom	4703149	4703766	+	618	0	9	1293	9	1293	
QEN71_RS21225	hypothetical protein	QEN71_21220	paras_004781	protein-codi	NZ_CP1252	chromosom	4703874	4704218	+	345	0	8	1827	5	1036	
QEN71_RS21230	RNA-binding protein	QEN71_21225	paras_004782	protein-codi	NZ_CP1252	chromosom	4704373	4704648	+	276	0	10	2534	5	1744	
QEN71_RS21235	ammonia-dependent NAD(+) synthetase	QEN71_21230	paras_004783	protein-codi	NZ_CP1252	chromosom	4704676	4705512	-	837	0	15	5004	11	2911	
QEN71_RS21240	L,D-transpeptidase	QEN71_21235	paras_004784	protein-codi	NZ_CP1252	chromosom	4705806	4706876	+	1071	0	25	6741	15	4664	
QEN71_RS21245	DMT family transporter	QEN71_21240	paras_004785	protein-codi	NZ_CP1252	chromosom	4706895	4707776	-	882	0	9	2387	7	1644	
QEN71_RS21250	AarF/UbiB family protein	QEN71_21245	paras_004786	protein-codi	NZ_CP1252	chromosom	4707871	4709427	-	1557	0	21	9101	14	7577	
QEN71_RS21255	DeoR/GlpR family DNA-binding transcription regula	QEN71_21250	paras_004787	protein-codi	NZ_CP1252	chromosom	4709723	4710478	+	756	0	22	6946	11	1751	
QEN71_RS21260	2-aminoethylphosphonate--pyruvate transaminase	QEN71_21255	paras_004788	protein-codi	NZ_CP1252	chromosom	4710494	4711603	-	1110	0	21	7724	21	7724	
QEN71_RS21265	2-aminoethylphosphonate ABC transporter substrat	QEN71_21260	paras_004789	protein-codi	NZ_CP1252	chromosom	4711965	4713062	+	1098	0	33	8428	29	8206	
QEN71_RS21270	2-aminoethylphosphonate ABC transport system AT	QEN71_21265	paras_004790	protein-codi	NZ_CP1252	chromosom	4713184	4714287	+	1081	0	11	3737	11	3737	
QEN71_RS21275	2-aminoethylphosphonate ABC transporter permeas	QEN71_21270	paras_004791	protein-codi	NZ_CP1252	chromosom	4714265	4715188	+	900	0	17	5125	11	3256	
QEN71_RS21280	2-aminoethylphosphonate ABC transport system, m	QEN71_21275	paras_004792	protein-codi	NZ_CP1252	chromosom	4715188	4716048	+	860	0	9	1645	9	1645	
QEN71_RS21285	phosphonate utilization associated transcriptional re	QEN71_21280	paras_004793	protein-codi	NZ_CP1252	chromosom	4716128	4716889	+	762	0	14	6117	12	5152	
QEN71_RS21290	phosphonoacetate hydrolase	QEN71_21285	paras_004794	protein-codi	NZ_CP1252	chromosom	4716933	4718204	+	1268	0	34	10996	24	8322	
QEN71_RS21295	phosphonoacetaldehyde dehydrogenase	QEN71_21290	paras_004795	protein-codi	NZ_CP1252	chromosom	4718201	4719658	+	1454	0	30	6444	26	5914	
QEN71_RS21300	HD domain-containing protein	QEN71_21295	paras_004796	protein-codi	NZ_CP1252	chromosom	4719663	4720223	+	561	0	20	7991	11	2580	
QEN71_RS21305	16S ribosomal RNA	QEN71_21300		rRNA	NZ_CP1252	chromosom	4720712	4722242	+	1531	3015.0	102	81514	83	62581	
QEN71_RS21310	tRNA-Ile	QEN71_21305		tRNA	NZ_CP1252	chromosom	4722307	4722383	+	77	152.0	3	2917	2	1003	
QEN71_RS21315	tRNA-Ala	QEN71_21310		tRNA	NZ_CP1252	chromosom	4722443	4722518	+	76	150.0	1	1224	0	0	
QEN71_RS21320	23S ribosomal RNA	QEN71_21315		rRNA	NZ_CP1252	chromosom	4722824	4725704	+	2881	5661.0	195	152202	155	114980	
QEN71_RS21325	5S ribosomal RNA	QEN71_21320		rRNA	NZ_CP1252	chromosom	4725911	4726024	+	114	225.0	6	4519	4	3504	
QEN71_RS21330	MFS transporter	QEN71_21325	paras_004802	protein-codi	NZ_CP1252	chromosom	4726262	4727581	-	1320	0	58	15095	49	13602	
QEN71_RS21335	xylulokinase	QEN71_21330	paras_004803	protein-codi	NZ_CP1252	chromosom	4727935	4729401	+	1467	0	19	2475	14	2035	
QEN71_RS21340	DUF1349 domain-containing protein	QEN71_21335	paras_004804	protein-codi	NZ_CP1252	chromosom	4729454	4730029	-	576	0	13	1558	10	1258	
QEN71_RS21345	cytochrome b	QEN71_21340	paras_004805	protein-codi	NZ_CP1252	chromosom	4730070	4730609	-	540	0	17	3005	13	2537	
QEN71_RS21350	catalase family peroxidase	QEN71_21345	paras_004806	protein-codi	NZ_CP1252	chromosom	4730622	4731692	-	1071	0	22	2199	17	1639	
QEN71_RS21355	DUF4148 domain-containing protein	QEN71_21350	paras_004807	protein-codi	NZ_CP1252	chromosom	4731912	4732250	+	339	0	6	1088	3	945	
QEN71_RS21360	DUF1330 domain-containing protein	QEN71_21355	paras_004808	protein-codi	NZ_CP1252	chromosom	4732333	4732656	-	324	0	5	771	4	769	
QEN71_RS21365	EAL domain-containing protein	QEN71_21360	paras_004809	protein-codi	NZ_CP1252	chromosom	4733138	4733908	+	771	0	8	444	4	229	
QEN71_RS21370	hypothetical protein	QEN71_21365	paras_004810	protein-codi	NZ_CP1252	chromosom	4733951	4734394	-	444	0	3	198	3	198	
QEN71_RS21375	TldD/PmbA family protein	QEN71_21370	paras_004811	protein-codi	NZ_CP1252	chromosom	4734782	4736218	+	1433	0	25	3068	19	2408	
QEN71_RS21380	metallopeptidase TldD-related protein	QEN71_21375	paras_004812	protein-codi	NZ_CP1252	chromosom	4736215	4737552	+	1334	0	20	1735	19	1683	
QEN71_RS21385	poly-beta-1,6 N-acetyl-D-glucosamine export porin	QEN71_21380	paras_004813	protein-codi	NZ_CP1252	chromosom	4738421	4740466	+	2046	0	36	2787	32	2585	
QEN71_RS21390	poly-beta-1,6-N-acetyl-D-glucosamine N-deacetylas	QEN71_21385	paras_004814	protein-codi	NZ_CP1252	chromosom	4740473	4742515	+	2043	0	26	1987	25	1959	
QEN71_RS21395	poly-beta-1,6-N-acetyl-D-glucosamine synthase	QEN71_21390	paras_004815	protein-codi	NZ_CP1252	chromosom	4742583	4743884	+	1302	0	21	2937	14	1968	
QEN71_RS21400	hypothetical protein	QEN71_21395	paras_004816	protein-codi	NZ_CP1252	chromosom	4743957	4744412	+	456	0	3	240	3	240	
QEN71_RS21405	glutamine--fructose-6-phosphate transaminase (isom	QEN71_21400	paras_004817	protein-codi	NZ_CP1252	chromosom	4744459	4746288	+	1830	0	31	2647	26	2165	
QEN71_RS21410	hypothetical protein	QEN71_21405	paras_004818	protein-codi	NZ_CP1252	chromosom	4746298	4747524	+	1227	0	29	5678	29	5678	
QEN71_RS21415	GFA family protein	QEN71_21410	paras_004819	protein-codi	NZ_CP1252	chromosom	4747679	4748050	+	372	0	10	556	9	552	
QEN71_RS21420	response regulator	QEN71_21415	paras_004820	protein-codi	NZ_CP1252	chromosom	4748086	4748832	+	747	0	13	1312	4	391	
QEN71_RS21425	HAMP domain-containing sensor histidine kinase	QEN71_21420	paras_004821	protein-codi	NZ_CP1252	chromosom	4748874	4750184	+	1300	0	24	2514	24	2514	
QEN71_RS21430	LysR substrate-binding domain-containing protein	QEN71_21425	paras_004822	protein-codi	NZ_CP1252	chromosom	4750174	4751085	-	901	0	9	1481	4	152	
QEN71_RS21435	nitrilase-related carbon-nitrogen hydrolase	QEN71_21430	paras_004823	protein-codi	NZ_CP1252	chromosom	4751184	4752080	+	897	0	8	2044	8	2044	
QEN71_RS21440	SET domain-containing protein-lysine N-methyltrans	QEN71_21435	paras_004824	protein-codi	NZ_CP1252	chromosom	4752192	4752650	+	459	0	18	4698	17	4692	
QEN71_RS21445	hypothetical protein	QEN71_21440	paras_004825	protein-codi	NZ_CP1252	chromosom	4752727	4752993	+	267	0	2	184	2	184	
QEN71_RS21450	hypothetical protein	QEN71_21445	paras_004826	protein-codi	NZ_CP1252	chromosom	4753082	4753288	-	207	0	9	3029	5	2351	
QEN71_RS21455	ATP-binding protein	QEN71_21450	paras_004827	protein-codi	NZ_CP1252	chromosom	4753537	4754973	-	1437	0	11	1796	11	1796	
QEN71_RS21460	response regulator	QEN71_21455	paras_004828	protein-codi	NZ_CP1252	chromosom	4754976	4755635	-	660	0	9	1165	8	992	

QEN71_RS21465	cytochrome b/b6 domain-containing protein	QEN71_21460	paras_004829	protein-codi	NZ_CP1252	chromosom	4755841	4756380	+		540	0	11	3274	7	2014	
QEN71_RS21470	hypothetical protein	QEN71_21465	paras_004830	protein-codi	NZ_CP1252	chromosom	4756582	4756941	+		360	0	8	1222	5	434	
QEN71_RS21475	bacteriohopanetetrol glucosamine biosynthesis glyc	QEN71_21470	paras_004831	protein-codi	NZ_CP1252	chromosom	4757025	4758245	+		1221	0	19	2622	14	1505	
QEN71_RS21480	hopanoid biosynthesis associated radical SAM prot	QEN71_21475	paras_004832	protein-codi	NZ_CP1252	chromosom	4758314	4759738	+		1421	507.0	26	3235	21	2694	
QEN71_RS21485	hopanoid biosynthesis-associated protein HpnK	QEN71_21480	paras_004833	protein-codi	NZ_CP1252	chromosom	4759735	4760595	+		857	0	20	3340	15	1865	
QEN71_RS21490	lysylphosphatidylglycerol synthase domain-containi	QEN71_21485	paras_004834	protein-codi	NZ_CP1252	chromosom	4760610	4761653	+		1044	0	9	1779	9	1779	
QEN71_RS21495	Fur family transcriptional regulator	QEN71_21490	paras_004835	protein-codi	NZ_CP1252	chromosom	4761786	4762253	+		468	0	9	1819	7	820	
QEN71_RS21500	carbonic anhydrase	QEN71_21495	paras_004836	protein-codi	NZ_CP1252	chromosom	4762697	4763401	+		705	0	20	3553	14	1318	
QEN71_RS21505	PLP-dependent aminotransferase family protein	QEN71_21500	paras_004837	protein-codi	NZ_CP1252	chromosom	4763450	4764856	-		1407	0	30	7281	21	5254	
QEN71_RS21510	hypothetical protein	QEN71_21505	paras_004838	protein-codi	NZ_CP1252	chromosom	4765609	4765755	+		147	0	1	14	0	0	
QEN71_RS21515	hypothetical protein	QEN71_21510	paras_004839	protein-codi	NZ_CP1252	chromosom	4765805	4766791	+		983	0	16	1307	11	741	
QEN71_RS21520	iron-containing redox enzyme family protein	QEN71_21515	paras_004840	protein-codi	NZ_CP1252	chromosom	4766788	4767786	+		995	0	20	2271	17	2125	
QEN71_RS21525	hypothetical protein	QEN71_21520	paras_004841	protein-codi	NZ_CP1252	chromosom	4767795	4769300	+		1495	0	32	3045	26	2655	
QEN71_RS21530	aspartate aminotransferase family protein	QEN71_21525	paras_004842	protein-codi	NZ_CP1252	chromosom	4769290	4770549	+		1245	0	7	531	3	163	
QEN71_RS21535	EamA family transporter RarD	QEN71_21530	paras_004843	protein-codi	NZ_CP1252	chromosom	4770546	4771640	+		1091	0	29	9615	21	7013	
QEN71_RS21540	TerB N-terminal domain-containing protein	QEN71_21535	paras_004844	protein-codi	NZ_CP1252	chromosom	4772193	4774484	+		2288	0	41	9722	32	7568	
QEN71_RS21545	ATP-binding protein	QEN71_21540	paras_004845	protein-codi	NZ_CP1252	chromosom	4774481	4775791	+		1278	0	24	5419	24	5419	
QEN71_RS21550	DEAD/DEAH box helicase	QEN71_21545	paras_004846	protein-codi	NZ_CP1252	chromosom	4775763	4777997	+		2206	0	30	9897	28	9849	
QEN71_RS21555	MDR family MFS transporter	QEN71_21550	paras_004847	protein-codi	NZ_CP1252	chromosom	4778170	4779681	+		1512	0	16	5146	13	4477	
QEN71_RS21560	LysR family transcriptional regulator	QEN71_21555	paras_004848	protein-codi	NZ_CP1252	chromosom	4779706	4780599	-		894	0	9	983	6	685	
QEN71_RS21565	class C beta-lactamase	QEN71_21560	paras_004849	pseudogene	NZ_CP1252	chromosom	4780823	4781896	+		1074	0	32	6742	27	6309	
QEN71_RS21570	serine hydrolase	QEN71_21565	paras_004850	protein-codi	NZ_CP1252	chromosom	4781910	4782992	-		1083	0	23	3523	20	2974	
QEN71_RS21575	MFS transporter	QEN71_21570	paras_004851	protein-codi	NZ_CP1252	chromosom	4783034	4784353	-		1320	0	23	4331	21	4081	
QEN71_RS21580	M20 family metallopeptidase	QEN71_21575	paras_004852	protein-codi	NZ_CP1252	chromosom	4784364	4785785	-		1422	0	11	1573	5	881	
QEN71_RS21585	LysR family transcriptional regulator	QEN71_21580	paras_004853	protein-codi	NZ_CP1252	chromosom	4785922	4786827	+		906	0	17	2513	15	1830	
QEN71_RS21590	Fic family protein	QEN71_21585	paras_004854	protein-codi	NZ_CP1252	chromosom	4786922	4787683	+		762	0	18	736	15	617	
QEN71_RS21595	gluconate transporter	partial;pseudo;QEN71_21590		pseudogene	NZ_CP1252	chromosom	4787696	4787809	-		114	0	0	0	0	0	
QEN71_RS21600	porin	QEN71_21595	paras_004856	protein-codi	NZ_CP1252	chromosom	4788224	4789363	+		1140	0	44	4677	35	3520	
QEN71_RS21605	hypothetical protein	QEN71_21600	paras_004857	protein-codi	NZ_CP1252	chromosom	4789612	4790067	+		456	0	15	3708	15	3708	
QEN71_RS21610	ProQ/FinO family protein	QEN71_21605	paras_004858	protein-codi	NZ_CP1252	chromosom	4790219	4790893	-		675	0	3	752	3	752	
QEN71_RS21615	porin	QEN71_21610	paras_004859	protein-codi	NZ_CP1252	chromosom	4791215	4792432	-		1218	874.0	52	10224	47	9870	
QEN71_RS21620	sugar phosphate isomerase/epimerase and 4-hydro	QEN71_21615	paras_004860	protein-codi	NZ_CP1252	chromosom	4792643	4794541	-		1899	0	54	10099	43	9029	
QEN71_RS21625	3-keto-5-aminohexanoate cleavage protein	QEN71_21620	paras_004861	protein-codi	NZ_CP1252	chromosom	4794577	4795422	-		846	0	9	1944	9	1944	
QEN71_RS21630	IclR family transcriptional regulator C-terminal doma	QEN71_21625	paras_004862	protein-codi	NZ_CP1252	chromosom	4795803	4796573	+		771	0	20	4596	14	2295	
QEN71_RS21635	phosphoribosyltransferase	QEN71_21630	paras_004863	protein-codi	NZ_CP1252	chromosom	4796607	4797275	+		669	0	17	3673	15	2911	
QEN71_RS21640	UTP--glucose-1-phosphate uridylyltransferase GalU	QEN71_21635	paras_004864	protein-codi	NZ_CP1252	chromosom	4797352	4798245	-		894	0	18	2913	15	2600	
QEN71_RS21645	hypothetical protein	QEN71_21640	paras_004865	protein-codi	NZ_CP1252	chromosom	4798506	4798649	-		144	0	2	75	1	71	
QEN71_RS21650	acyltransferase	QEN71_21645	paras_004866	protein-codi	NZ_CP1252	chromosom	4798898	4800079	+		1182	0	28	5002	23	4273	
QEN71_RS21655	glycosyltransferase family 4 protein	QEN71_21650	paras_004867	protein-codi	NZ_CP1252	chromosom	4800149	4802644	-		2492	2213.0	28	3104	23	2081	
QEN71_RS21660	oligosaccharide flippase family protein	QEN71_21655	paras_004868	protein-codi	NZ_CP1252	chromosom	4802641	4804119	-		1475	575.0	20	2353	16	2132	
QEN71_RS21665	hypothetical protein	QEN71_21660	paras_004869	protein-codi	NZ_CP1252	chromosom	4804188	4806344	-		2157	0	53	7142	36	5610	
QEN71_RS21670	acyltransferase	QEN71_21665	paras_004870	protein-codi	NZ_CP1252	chromosom	4806903	4808003	+		1097	0	37	4032	37	4032	
QEN71_RS21675	SGNH/GDSL hydrolase family protein	QEN71_21670	paras_004871	protein-codi	NZ_CP1252	chromosom	4808000	4808782	+		775	0	18	2925	18	2925	
QEN71_RS21680	GDP-mannose 4,6-dehydratase	QEN71_21675	paras_004872	protein-codi	NZ_CP1252	chromosom	4808779	4809822	+		1040	0	25	2697	18	1877	
QEN71_RS21685	HipA domain-containing protein	QEN71_21680	paras_004873	protein-codi	NZ_CP1252	chromosom	4809866	4811230	-		1364	0	27	5309	19	4093	
QEN71_RS21690	helix-turn-helix domain-containing protein	QEN71_21685	paras_004874	protein-codi	NZ_CP1252	chromosom	4811230	4811547	-		317	0	1	13	0	0	TRUE
QEN71_RS21695	glycosyltransferase family 4 protein	QEN71_21690	paras_004875	protein-codi	NZ_CP1252	chromosom	4811779	4812939	-		1161	204.0	22	5306	12	1244	
QEN71_RS21700	glycosyltransferase family 4 protein	QEN71_21695	paras_004876	protein-codi	NZ_CP1252	chromosom	4812971	4814206	-		1236	0	21	3982	19	3777	
QEN71_RS21705	glucose-6-phosphate isomerase	QEN71_21700	paras_004877	protein-codi	NZ_CP1252	chromosom	4814207	4815667	-		1461	0	24	3771	24	3771	
QEN71_RS21710	glycosyltransferase	QEN71_21705	paras_004878	protein-codi	NZ_CP1252	chromosom	4815671	4816828	-		1154	0	19	3101	15	2538	
QEN71_RS21715	glycosyltransferase	QEN71_21710	paras_004879	protein-codi	NZ_CP1252	chromosom	4816825	4817775	-		947	0	10	1057	7	769	
QEN71_RS21720	polysaccharide biosynthesis tyrosine autokinase	QEN71_21715	paras_004880	protein-codi	NZ_CP1252	chromosom	4817816	4820008	-		2193	0	34	4114	25	2351	
QEN71_RS21725	polysaccharide biosynthesis/export family protein	QEN71_21720	paras_004881	protein-codi	NZ_CP1252	chromosom	4820197	4821378	-		1175	0	28	4480	19	3165	
QEN71_RS21730	low molecular weight phosphotyrosine protein phos	QEN71_21725	paras_004882	protein-codi	NZ_CP1252	chromosom	4821372	4821848	-		470	0	3	732	2	212	
QEN71_RS21735	UDP-glucose/GDP-mannose dehydrogenase family	QEN71_21730	paras_004883	protein-codi	NZ_CP1252	chromosom	4821886	4823304	-		1419	0	21	2795	11	1460	
QEN71_RS21740	undecaprenyl-phosphate glucose phosphotransfera	QEN71_21735	paras_004884	protein-codi	NZ_CP1252	chromosom	4823384	4824781	-		1398	0	23	4002	17	2837	
QEN71_RS21745	hypothetical protein	QEN71_21740	paras_004885	protein-codi	NZ_CP1252	chromosom	4824985	4825206	-		222	0	6	1631	5	1629	
QEN71_RS21750	mannose-1-phosphate guanylyltransferase/mannos	QEN71_21745	paras_004886	protein-codi	NZ_CP1252	chromosom	4825395	4826942	+		1548	0	23	4859	14	1579	
QEN71_RS21755	transposase			pseudogene	NZ_CP1252	chromosom	4828188	4828406	-		219	0	8	1243	6	522	
QEN71_RS21760	YciI-like protein	QEN71_21755	paras_004888	protein-codi	NZ_CP1252	chromosom	4828957	4829253	-		297	0	10	5970	5	1257	
QEN71_RS21765	PsiF family protein	QEN71_21760	paras_004889	protein-codi	NZ_CP1252	chromosom	4829505	4829801	+		297	0	0	0	0	0	TRUE
QEN71_RS21770	hypothetical protein	QEN71_21765	paras_004890	protein-codi	NZ_CP1252	chromosom	4830095	4830541	+		447	0	10	3583	10	3583	

QEN71_RS21775	YSC84-related protein	QEN71_21770	paras_004891	protein-codi	NZ_CP1252	chromosom	4830941	4831528	+	588	0	16	4351	12	3873	
QEN71_RS21780	glycosyltransferase family 4 protein	QEN71_21775	paras_004892	protein-codi	NZ_CP1252	chromosom	4831761	4832831	-	1071	0	29	7382	19	4630	
QEN71_RS21785	AAA family ATPase	QEN71_21780	paras_004893	protein-codi	NZ_CP1252	chromosom	4833151	4834314	-	1164	0	20	3076	12	2300	
QEN71_RS21790	alpha,alpha-trehalose-phosphate synthase (UDP-fo	QEN71_21785	paras_004894	protein-codi	NZ_CP1252	chromosom	4834355	4835785	-	1431	0	28	5669	27	5421	
QEN71_RS21795	Rap1a/Tai family immunity protein	QEN71_21790	paras_004895	protein-codi	NZ_CP1252	chromosom	4836039	4836395	-	357	0	14	3758	14	3758	
QEN71_RS21800	ankyrin repeat domain-containing protein	QEN71_21795	paras_004896	protein-codi	NZ_CP1252	chromosom	4836514	4837260	-	747	0	10	3888	8	3603	
QEN71_RS21805	TatD family hydrolase	QEN71_21800	paras_004897	protein-codi	NZ_CP1252	chromosom	4837312	4838103	-	792	0	31	8640	29	7913	
QEN71_RS21810	DNA polymerase III subunit delta'	QEN71_21805	paras_004898	protein-codi	NZ_CP1252	chromosom	4838170	4839219	-	1050	0	2	137	0	0	TRUE
QEN71_RS21815	dTMP kinase	QEN71_21810	paras_004899	protein-codi	NZ_CP1252	chromosom	4839313	4839933	-	620	0	10	1469	7	891	
QEN71_RS21820	endolytic transglycosylase MltG	QEN71_21815	paras_004900	protein-codi	NZ_CP1252	chromosom	4839933	4840943	-	1010	0	37	13773	29	12571	
QEN71_RS21825	folate-binding protein YgfZ	QEN71_21820	paras_004901	protein-codi	NZ_CP1252	chromosom	4841021	4842070	+	1050	0	4	315	0	0	TRUE
QEN71_RS21830	NRDE family protein	QEN71_21825	paras_004902	protein-codi	NZ_CP1252	chromosom	4842165	4842989	+	825	0	22	6945	20	6535	
QEN71_RS21835	alpha/beta hydrolase	QEN71_21830	paras_004903	protein-codi	NZ_CP1252	chromosom	4843002	4843961	-	960	0	35	11152	30	10162	
QEN71_RS21840	SDR family oxidoreductase	QEN71_21835	paras_004904	protein-codi	NZ_CP1252	chromosom	4844049	4844918	-	870	0	20	4107	15	3322	
QEN71_RS21845	NADP-dependent oxidoreductase	QEN71_21840	paras_004905	protein-codi	NZ_CP1252	chromosom	4844951	4845949	-	999	0	23	5670	23	5670	
QEN71_RS21850	PaaI family thioesterase	QEN71_21845	paras_004906	protein-codi	NZ_CP1252	chromosom	4846014	4846487	-	474	0	6	3960	5	2683	
QEN71_RS21855	GYD domain-containing protein	QEN71_21850	paras_004907	protein-codi	NZ_CP1252	chromosom	4846963	4847256	+	294	0	9	6008	2	662	
QEN71_RS21860	TRIC cation channel family protein	QEN71_21855	paras_004908	protein-codi	NZ_CP1252	chromosom	4847308	4847931	-	624	0	5	1020	5	1020	
QEN71_RS21865	LEA type 2 family protein	QEN71_21860	paras_004909	protein-codi	NZ_CP1252	chromosom	4848140	4848631	+	492	0	5	834	3	770	
QEN71_RS21870	SH3 domain-containing protein	QEN71_21865	paras_004910	protein-codi	NZ_CP1252	chromosom	4848671	4849501	-	831	0	27	6209	25	5814	
QEN71_RS21875	DUF2945 domain-containing protein	QEN71_21870	paras_004911	protein-codi	NZ_CP1252	chromosom	4849627	4849845	+	219	0	8	1004	6	800	
QEN71_RS21880	acetyl-CoA C-acyltransferase	QEN71_21875	paras_004912	protein-codi	NZ_CP1252	chromosom	4849931	4851109	-	1179	0	9	1135	8	1069	
QEN71_RS21885	bifunctional nicotinamidase/pyrazinamidase	QEN71_21880	paras_004913	protein-codi	NZ_CP1252	chromosom	4851159	4851785	-	627	0	7	1388	5	967	
QEN71_RS21890	3-hydroxyacyl-CoA dehydrogenase NAD-binding do	QEN71_21885	paras_004914	protein-codi	NZ_CP1252	chromosom	4851824	4853908	-	2085	0	47	7314	34	5713	
QEN71_RS21895	3-(methylthio)propionyl-CoA ligase	QEN71_21890	paras_004915	protein-codi	NZ_CP1252	chromosom	4854089	4855786	-	1698	0	38	8897	29	7029	
QEN71_RS21900	YdiU family protein	QEN71_21895	paras_004916	protein-codi	NZ_CP1252	chromosom	4855930	4857486	+	1557	0	29	4988	19	2587	
QEN71_RS21905	septation protein A	QEN71_21900	paras_004917	protein-codi	NZ_CP1252	chromosom	4857741	4858271	+	531	0	5	736	1	7	
QEN71_RS21910	BolA family protein	QEN71_21905	paras_004918	protein-codi	NZ_CP1252	chromosom	4858273	4858587	+	315	0	7	2204	6	2144	
QEN71_RS21915	peptidylprolyl isomerase	QEN71_21910	paras_004919	protein-codi	NZ_CP1252	chromosom	4858598	4859377	+	780	0	18	3226	13	2439	
QEN71_RS21920	phosphoribosylformylglycinamidine synthase	QEN71_21915	paras_004920	protein-codi	NZ_CP1252	chromosom	4860040	4864119	-	4080	0	80	16071	54	11192	
QEN71_RS21925	FAD-dependent oxidoreductase	QEN71_21920	paras_004921	protein-codi	NZ_CP1252	chromosom	4864587	4865888	+	1302	0	20	8145	16	7151	
QEN71_RS21930	NAD(P)H-hydrate dehydratase	QEN71_21925	paras_004922	protein-codi	NZ_CP1252	chromosom	4866000	4867592	+	1593	0	34	14191	30	13724	
QEN71_RS21935	glucose-6-phosphate isomerase	QEN71_21930	paras_004923	protein-codi	NZ_CP1252	chromosom	4867861	4869483	+	1623	0	37	2207	32	1879	
QEN71_RS21940	ABC transporter ATP-binding protein	QEN71_21935	paras_004924	protein-codi	NZ_CP1252	chromosom	4869576	4870262	-	685	0	8	2605	3	887	
QEN71_RS21945	arylesterase	QEN71_21940	paras_004925	protein-codi	NZ_CP1252	chromosom	4870261	4870980	+	718	0	31	9888	24	7079	
QEN71_RS21950	hypothetical protein	QEN71_21945	paras_004926	protein-codi	NZ_CP1252	chromosom	4871123	4871464	+	342	0	11	4614	6	4139	
QEN71_RS21955	SurA N-terminal domain-containing protein	QEN71_21950	paras_004927	protein-codi	NZ_CP1252	chromosom	4871569	4873497	-	1929	0	41	8228	30	5426	
QEN71_RS21960	tRNA-Asp	QEN71_21955		tRNA	NZ_CP1252	chromosom	4873596	4873672	-	77	153.0	2	54	2	54	
QEN71_RS21965	tRNA-Val	QEN71_21960		tRNA	NZ_CP1252	chromosom	4873714	4873789	-	76	0	2	22	1	3	
QEN71_RS21970	HU family DNA-binding protein	QEN71_21965	paras_004930	protein-codi	NZ_CP1252	chromosom	4873858	4874130	-	273	0	7	626	2	89	
QEN71_RS21975	endopeptidase La	QEN71_21970	paras_004931	protein-codi	NZ_CP1252	chromosom	4874493	4876910	-	2418	0	42	3287	33	1621	
QEN71_RS21980	ATP-dependent Clp protease ATP-binding subunit (	QEN71_21975	paras_004932	protein-codi	NZ_CP1252	chromosom	4877095	4878366	-	1272	0	34	8401	19	4960	
QEN71_RS21985	ATP-dependent Clp endopeptidase proteolytic subu	QEN71_21980	paras_004933	protein-codi	NZ_CP1252	chromosom	4878574	4879233	-	660	0	14	493	12	457	
QEN71_RS21990	trigger factor	QEN71_21985	paras_004934	protein-codi	NZ_CP1252	chromosom	4879416	4880762	-	1347	0	23	6445	17	4013	
QEN71_RS21995	glycerate kinase	QEN71_21990	paras_004935	protein-codi	NZ_CP1252	chromosom	4881282	4882427	+	1146	0	9	2358	6	1357	
QEN71_RS22000	MarR family transcriptional regulator	QEN71_21995	paras_004936	protein-codi	NZ_CP1252	chromosom	4882491	4882946	+	456	0	13	3704	9	2281	
QEN71_RS22005	hypothetical protein	QEN71_22000	paras_004937	protein-codi	NZ_CP1252	chromosom	4883375	4883512	+	138	0	2	1107	0	0	
QEN71_RS22010	tRNA-Leu	QEN71_22005		tRNA	NZ_CP1252	chromosom	4883691	4883777	-	87	0	2	13	2	13	
QEN71_RS22015	squalene/phytoene synthase family protein	QEN71_22010	paras_004939	protein-codi	NZ_CP1252	chromosom	4884057	4884890	+	834	0	23	7058	14	4184	
QEN71_RS22020	DUF1501 domain-containing protein	QEN71_22015	paras_004940	protein-codi	NZ_CP1252	chromosom	4884906	4886054	-	1149	0	24	9328	18	7680	
QEN71_RS22025	hypothetical protein	QEN71_22020	paras_004941	protein-codi	NZ_CP1252	chromosom	4886118	4886453	-	336	0	11	6363	8	2134	
QEN71_RS22030	two-component system response regulator OmpR	QEN71_22025	paras_004942	protein-codi	NZ_CP1252	chromosom	4886692	4887417	+	726	0	18	5883	15	4924	
QEN71_RS22035	ATP-binding protein	QEN71_22030	paras_004943	protein-codi	NZ_CP1252	chromosom	4887461	4888810	+	1350	0	34	10021	34	10021	
QEN71_RS22040	peroxiredoxin	QEN71_22035	paras_004944	protein-codi	NZ_CP1252	chromosom	4889064	4889612	+	549	0	15	6441	13	6027	
QEN71_RS22045	carboxymuconolactone decarboxylase family protei	QEN71_22040	paras_004945	protein-codi	NZ_CP1252	chromosom	4889689	4890213	+	525	0	16	5535	10	3478	
QEN71_RS22050	2-C-methyl-D-erythritol 2,4-cyclodiphosphate syntha	QEN71_22045	paras_004946	protein-codi	NZ_CP1252	chromosom	4890296	4890775	-	480	0	0	0	0	0	TRUE
QEN71_RS22055	2-C-methyl-D-erythritol 4-phosphate cytidylyltransfe	QEN71_22050	paras_004947	protein-codi	NZ_CP1252	chromosom	4890838	4891548	-	711	0	1	3	1	3	TRUE
QEN71_RS22060	transcription-repair coupling factor	QEN71_22055	paras_004948	protein-codi	NZ_CP1252	chromosom	4891842	4895336	+	3495	0	72	18055	63	14669	
QEN71_RS22065	M20 family metallopeptidase	QEN71_22060	paras_004949	protein-codi	NZ_CP1252	chromosom	4895341	4896528	+	1188	0	28	5336	26	5082	
QEN71_RS22070	acetylornithine deacetylase	QEN71_22065	paras_004950	protein-codi	NZ_CP1252	chromosom	4896685	4897932	+	1248	0	12	2614	12	2614	
QEN71_RS22075	threonine/serine dehydratase	QEN71_22070	paras_004951	protein-codi	NZ_CP1252	chromosom	4898012	4899034	+	1023	0	25	6307	20	6110	
QEN71_RS22080	large conductance mechanosensitive channel prote	QEN71_22075	paras_004952	protein-codi	NZ_CP1252	chromosom	4899223	4899672	+	450	0	12	4075	9	3432	

QEN71_RS22085	response regulator	QEN71_22080	paras_004953	protein-codi	NZ_CP1252	chromosom	4899781	4901772	-	1992	0	22	9371	18	7565	
QEN71_RS22090	response regulator	QEN71_22085	paras_004954	protein-codi	NZ_CP1252	chromosom	4901859	4902350	-	492	0	19	5604	17	4745	
QEN71_RS22095	ATP-binding protein	QEN71_22090	paras_004955	protein-codi	NZ_CP1252	chromosom	4902520	4904130	-	1611	0	41	21899	34	18098	
QEN71_RS22100	hypothetical protein	QEN71_22095	paras_004956	protein-codi	NZ_CP1252	chromosom	4904170	4904439	+	270	0	12	2969	9	1934	
QEN71_RS22105	hypothetical protein	QEN71_22100	paras_004957	protein-codi	NZ_CP1252	chromosom	4904634	4905128	+	495	0	13	4189	9	2581	
QEN71_RS22110	AsmA family protein	QEN71_22105	paras_004958	protein-codi	NZ_CP1252	chromosom	4905243	4907684	-	2442	0	37	7577	28	6193	
QEN71_RS22115	ATP-binding cassette domain-containing protein	QEN71_22110	paras_004959	protein-codi	NZ_CP1252	chromosom	4907813	4908532	+	716	0	6	2132	6	2132	
QEN71_RS22120	iron export ABC transporter permease subunit FetB	QEN71_22115	paras_004960	protein-codi	NZ_CP1252	chromosom	4908529	4909335	+	803	0	24	5600	16	3722	
QEN71_RS22125	DUF6013 family protein	QEN71_22120	paras_004961	protein-codi	NZ_CP1252	chromosom	4909411	4909971	-	561	0	9	2069	8	1700	
QEN71_RS22130	zinc-binding alcohol dehydrogenase family protein	QEN71_22125	paras_004962	protein-codi	NZ_CP1252	chromosom	4910716	4911732	-	1017	0	19	7837	19	7837	
QEN71_RS22135	LysR family transcriptional regulator	QEN71_22130	paras_004963	protein-codi	NZ_CP1252	chromosom	4911899	4912849	+	951	0	14	2478	11	1966	
QEN71_RS22140	hypothetical protein	QEN71_22135	paras_004964	protein-codi	NZ_CP1252	chromosom	4912857	4913171	-	315	0	2	529	2	529	
QEN71_RS22145	DNA ligase D	QEN71_22140	paras_004965	protein-codi	NZ_CP1252	chromosom	4913245	4916097	-	2853	0	45	11705	36	9996	
QEN71_RS22150	Ku protein	QEN71_22145	paras_004966	protein-codi	NZ_CP1252	chromosom	4916160	4917125	-	966	0	23	3997	17	2320	
QEN71_RS22155	cytochrome P450	QEN71_22150	paras_004967	protein-codi	NZ_CP1252	chromosom	4917318	4918541	-	1224	0	9	1433	5	597	
QEN71_RS22160	phosphoserine phosphatase SerB	QEN71_22155	paras_004968	protein-codi	NZ_CP1252	chromosom	4918743	4919582	+	840	0	14	2511	12	1853	
QEN71_RS22165	cystathionine beta-lyase	QEN71_22160	paras_004969	protein-codi	NZ_CP1252	chromosom	4919672	4920856	-	1185	0	26	5113	23	4728	
QEN71_RS22170	hypothetical protein	QEN71_22165	paras_004970	protein-codi	NZ_CP1252	chromosom	4920907	4921536	-	630	0	11	2210	9	2100	
QEN71_RS22175	acetyl-CoA C-acyltransferase family protein	QEN71_22170	paras_004971	protein-codi	NZ_CP1252	chromosom	4921662	4922846	-	1185	0	21	8457	16	7124	
QEN71_RS22180	sugar kinase	QEN71_22175	paras_004972	protein-codi	NZ_CP1252	chromosom	4922994	4923932	-	939	0	15	3543	14	3310	
QEN71_RS22185	30S ribosomal protein S12 methylthiotransferase Ri	QEN71_22180	paras_004973	protein-codi	NZ_CP1252	chromosom	4923935	4925326	-	1392	0	23	4895	16	2293	
QEN71_RS22190	polyhydroxyalkanoate synthesis repressor PhaR	QEN71_22185	paras_004974	protein-codi	NZ_CP1252	chromosom	4925690	4926268	-	579	0	24	4141	16	3275	
QEN71_RS22195	3-ketoacyl-ACP reductase	QEN71_22190	paras_004975	protein-codi	NZ_CP1252	chromosom	4926417	4927157	-	741	0	15	4929	4	1486	
QEN71_RS22200	acetyl-CoA C-acetyltransferase	QEN71_22195	paras_004976	protein-codi	NZ_CP1252	chromosom	4927329	4928510	-	1182	0	15	2623	9	1661	
QEN71_RS22205	class I poly(R)-hydroxyalkanoic acid synthase	QEN71_22200	paras_004977	protein-codi	NZ_CP1252	chromosom	4928700	4930688	-	1989	0	56	19185	50	18341	
QEN71_RS22210	peptidoglycan editing factor PgeF	QEN71_22205	paras_004978	protein-codi	NZ_CP1252	chromosom	4931124	4931963	-	836	0	16	4885	10	2660	
QEN71_RS22215	RluA family pseudouridine synthase	QEN71_22210	paras_004979	protein-codi	NZ_CP1252	chromosom	4931960	4933282	-	1319	0	21	3191	8	479	
QEN71_RS22220	outer membrane protein assembly factor BamD	QEN71_22215	paras_004980	protein-codi	NZ_CP1252	chromosom	4933322	4934179	+	858	0	3	624	1	10	
QEN71_RS22225	23S rRNA (uracil(1939)-C(5))-methyltransferase Rlr	QEN71_22220	paras_004981	protein-codi	NZ_CP1252	chromosom	4934285	4935589	-	1305	0	26	7673	20	6142	
QEN71_RS22230	endonuclease/exonuclease/phosphatase family pro	QEN71_22225	paras_004982	protein-codi	NZ_CP1252	chromosom	4935793	4936656	+	864	0	16	3059	13	3041	
QEN71_RS22235	3'-5' exonuclease	QEN71_22230	paras_004983	protein-codi	NZ_CP1252	chromosom	4937310	4938086	-	777	0	46	13564	37	10972	
QEN71_RS22240	RNA polymerase sigma factor RpoS	QEN71_22235	paras_004984	protein-codi	NZ_CP1252	chromosom	4938093	4939187	-	1095	0	18	2651	14	2277	
QEN71_RS22245	peptidoglycan DD-metalloendopeptidase family prot	QEN71_22240	paras_004985	protein-codi	NZ_CP1252	chromosom	4939204	4940151	-	948	0	33	1526	23	834	
QEN71_RS22250	protein-L-isoaspartate(D-aspartate) O-methyltransfe	QEN71_22245	paras_004986	protein-codi	NZ_CP1252	chromosom	4940157	4941215	-	1055	0	14	1522	13	1517	
QEN71_RS22255	5'/3'-nucleotidase SurE	QEN71_22250	paras_004987	protein-codi	NZ_CP1252	chromosom	4941212	4941970	-	755	0	22	5483	15	4575	
QEN71_RS22260	NADPH:quinone oxidoreductase family protein	QEN71_22255	paras_004988	protein-codi	NZ_CP1252	chromosom	4942237	4943211	+	975	0	14	4638	12	4605	
QEN71_RS22265	ABC transporter permease	QEN71_22260	paras_004989	protein-codi	NZ_CP1252	chromosom	4943293	4944117	-	825	0	23	7665	21	7453	
QEN71_RS22270	ABC transporter ATP-binding protein	QEN71_22265	paras_004990	protein-codi	NZ_CP1252	chromosom	4944119	4944943	-	825	0	9	2949	4	1354	
QEN71_RS22275	ABC transporter substrate-binding protein	QEN71_22270	paras_004991	protein-codi	NZ_CP1252	chromosom	4945080	4946117	-	1038	0	18	4944	16	4815	
QEN71_RS22280	CaiB/BaiF CoA-transferase family protein	QEN71_22275	paras_004992	protein-codi	NZ_CP1252	chromosom	4946196	4947428	-	1233	0	27	8458	21	6240	
QEN71_RS22285	recombination mediator RecR	QEN71_22280	paras_004993	protein-codi	NZ_CP1252	chromosom	4947483	4948079	-	597	0	11	4919	6	1872	
QEN71_RS22290	YbaB/EbfC family nucleoid-associated protein	QEN71_22285	paras_004994	protein-codi	NZ_CP1252	chromosom	4948212	4948538	-	327	0	2	254	0	0	
QEN71_RS22295	DNA polymerase III subunit gamma/tau	QEN71_22290	paras_004995	protein-codi	NZ_CP1252	chromosom	4948611	4950920	-	2310	0	2	15	0	0	TRUE
QEN71_RS22305	thioredoxin TrxA	QEN71_22300	paras_004997	protein-codi	NZ_CP1252	chromosom	4951643	4951969	+	327	0	1	2	1	2	
QEN71_RS22310	transcription termination factor Rho	QEN71_22305	paras_004998	protein-codi	NZ_CP1252	chromosom	4952258	4953520	+	1263	0	0	0	0	0	TRUE
QEN71_RS22315	MerR family transcriptional regulator	QEN71_22310	paras_004999	protein-codi	NZ_CP1252	chromosom	4953680	4954189	+	510	0	9	2263	9	2263	
QEN71_RS22320	zinc-dependent peptidase	QEN71_22315	paras_005000	protein-codi	NZ_CP1252	chromosom	4954282	4955118	+	837	0	25	8027	16	5425	
QEN71_RS22325	type B 50S ribosomal protein L31	QEN71_22320	paras_005001	protein-codi	NZ_CP1252	chromosom	4955272	4955535	+	264	0	3	1928	0	0	
QEN71_RS22330	glycosyltransferase family 39 protein	QEN71_22325	paras_005002	protein-codi	NZ_CP1252	chromosom	4955811	4957568	+	1758	0	11	1604	9	667	
QEN71_RS22335	MATE family efflux transporter	QEN71_22330	paras_005003	protein-codi	NZ_CP1252	chromosom	4957571	4958950	+	1380	0	55	20363	50	18796	
QEN71_RS22340	carboxymuconolactone decarboxylase family protei	QEN71_22335	paras_005004	protein-codi	NZ_CP1252	chromosom	4959079	4959489	-	407	0	7	2537	6	2530	
QEN71_RS22345	MerR family transcriptional regulator	QEN71_22340	paras_005005	protein-codi	NZ_CP1252	chromosom	4959486	4959902	-	413	0	18	4037	14	3312	
QEN71_RS22350	MG2 domain-containing protein	QEN71_22345	paras_005006	protein-codi	NZ_CP1252	chromosom	4960120	4966134	+	6015	0	98	30547	80	26965	
QEN71_RS22355	penicillin-binding protein 1C	QEN71_22350	paras_005007	protein-codi	NZ_CP1252	chromosom	4966171	4968507	+	2337	0	44	8894	41	8565	
QEN71_RS22360	helix-hairpin-helix domain-containing protein	QEN71_22355	paras_005008	protein-codi	NZ_CP1252	chromosom	4968690	4969247	+	558	0	4	764	2	85	
QEN71_RS22365	YidB family protein	QEN71_22360	paras_005009	protein-codi	NZ_CP1252	chromosom	4969379	4969822	+	444	0	7	1768	5	1321	
QEN71_RS22370	ATP-dependent chaperone ClpB	QEN71_22365	paras_005010	protein-codi	NZ_CP1252	chromosom	4969932	4972529	-	2598	0	40	10729	33	8025	
QEN71_RS22375	Rrf2 family transcriptional regulator	QEN71_22370	paras_005011	protein-codi	NZ_CP1252	chromosom	4972819	4973316	-	498	0	23	9828	13	6095	
QEN71_RS22380	group III truncated hemoglobin	QEN71_22375	paras_005012	protein-codi	NZ_CP1252	chromosom	4973556	4974116	+	561	0	20	5383	15	4809	
QEN71_RS22385	molybdopterin synthase catalytic subunit MoaE	QEN71_22380	paras_005013	protein-codi	NZ_CP1252	chromosom	4974240	4974716	-	477	0	5	670	4	667	
QEN71_RS22390	molybdopterin converting factor subunit 1	QEN71_22385	paras_005014	protein-codi	NZ_CP1252	chromosom	4974738	4974995	-	258	0	3	339	1	108	
QEN71_RS22395	molybdopterin molybdotransferase MoeA	QEN71_22390	paras_005015	protein-codi	NZ_CP1252	chromosom	4975011	4976231	-	1221	0	11	2675	9	2446	

QEN71_RS22400	threonine synthase	QEN71_22395	paras_005016	protein-codi	NZ_CP1252	chromosom	4976929	4978377	-	1449	0	36	3017	25	2065	
QEN71_RS22405	homoserine dehydrogenase	QEN71_22400	paras_005017	protein-codi	NZ_CP1252	chromosom	4978408	4979739	-	1332	0	25	4580	24	4577	
QEN71_RS22410	pyridoxal phosphate-dependent aminotransferase	QEN71_22405	paras_005018	protein-codi	NZ_CP1252	chromosom	4979797	4981044	-	1248	0	38	13982	34	13054	
QEN71_RS22415	Mth938-like domain-containing protein	QEN71_22410	paras_005019	protein-codi	NZ_CP1252	chromosom	4981501	4981875	+	375	0	17	5476	13	3836	
QEN71_RS22420	glycosyltransferase family 39 protein	QEN71_22415	paras_005020	protein-codi	NZ_CP1252	chromosom	4982282	4984087	+	1806	0	2	40	2	40	TRUE
QEN71_RS22425	SMR family transporter	QEN71_22420	paras_005021	protein-codi	NZ_CP1252	chromosom	4984116	4984487	+	372	0	6	1697	6	1697	
QEN71_RS22430	DegT/DnrJ/EryC1/StrS aminotransferase family prot	QEN71_22425	paras_005022	protein-codi	NZ_CP1252	chromosom	4984747	4985898	+	1152	0	2	56	1	2	TRUE
QEN71_RS22435	glycosyltransferase	QEN71_22430	paras_005023	protein-codi	NZ_CP1252	chromosom	4985916	4986965	+	1046	0	1	11	1	11	TRUE
QEN71_RS22440	formyltransferase	QEN71_22435	paras_005024	protein-codi	NZ_CP1252	chromosom	4986962	4987897	+	928	0	0	0	0	0	TRUE
QEN71_RS22445	bifunctional UDP-4-keto-pentose/UDP-xylose synth	QEN71_22440	paras_005025	protein-codi	NZ_CP1252	chromosom	4987894	4988940	+	1043	0	1	250	0	0	TRUE
QEN71_RS22450	polysaccharide deacetylase family protein	QEN71_22445	paras_005026	protein-codi	NZ_CP1252	chromosom	4988953	4989849	+	897	0	3	115	1	2	TRUE
QEN71_RS22455	peroxiredoxin	QEN71_22450	paras_005027	protein-codi	NZ_CP1252	chromosom	4990068	4990529	+	462	0	15	4458	13	3144	
QEN71_RS22460	PhoH family protein	QEN71_22455	paras_005028	protein-codi	NZ_CP1252	chromosom	4990895	4992715	+	1821	0	49	18505	33	11272	
QEN71_RS22465	C40 family peptidase	QEN71_22460	paras_005029	protein-codi	NZ_CP1252	chromosom	4992971	4994038	+	1068	0	32	11121	30	10233	
QEN71_RS22470	SDR family oxidoreductase	QEN71_22465	paras_005030	protein-codi	NZ_CP1252	chromosom	4994171	4994977	+	807	0	19	8010	16	5876	
QEN71_RS22475	inorganic phosphate transporter	QEN71_22470	paras_005031	protein-codi	NZ_CP1252	chromosom	4995011	4996021	-	1010	0	31	12545	24	11365	
QEN71_RS22480	DUF47 family protein	QEN71_22475	paras_005032	protein-codi	NZ_CP1252	chromosom	4996021	4996647	-	626	0	9	2642	6	2379	
QEN71_RS22485	replicative DNA helicase	QEN71_22480	paras_005033	protein-codi	NZ_CP1252	chromosom	4996797	4998185	-	1389	0	4	138	1	2	TRUE
QEN71_RS22490	50S ribosomal protein L9	QEN71_22485	paras_005034	protein-codi	NZ_CP1252	chromosom	4998360	4998812	-	453	0	4	709	2	228	
QEN71_RS22495	30S ribosomal protein S18	QEN71_22490	paras_005035	protein-codi	NZ_CP1252	chromosom	4998840	4999115	-	276	0	2	155	0	0	TRUE
QEN71_RS22500	primosomal replication protein N	QEN71_22495	paras_005036	protein-codi	NZ_CP1252	chromosom	4999118	4999417	-	300	0	1	11	0	0	TRUE
QEN71_RS22505	30S ribosomal protein S6	QEN71_22500	paras_005037	protein-codi	NZ_CP1252	chromosom	4999494	4999868	-	375	0	0	0	0	0	TRUE
QEN71_RS22510	anti-sigma factor	QEN71_22505	paras_005038	protein-codi	NZ_CP1252	chromosom	5000070	5001020	-	940	0	19	1962	16	1873	
QEN71_RS22515	RNA polymerase sigma factor	QEN71_22510	paras_005039	protein-codi	NZ_CP1252	chromosom	5001010	5001849	-	829	0	12	2939	12	2939	
QEN71_RS22520	metallophosphoesterase	QEN71_22515	paras_005040	protein-codi	NZ_CP1252	chromosom	5002006	5002899	+	894	0	21	4518	15	2896	
QEN71_RS22525	cupredoxin domain-containing protein	QEN71_22520	paras_005041	protein-codi	NZ_CP1252	chromosom	5002967	5003290	+	324	0	4	709	3	659	
QEN71_RS22530	LysR family transcriptional regulator	QEN71_22525	paras_005042	protein-codi	NZ_CP1252	chromosom	5003312	5004223	-	912	0	24	4605	13	2174	
QEN71_RS22535	glyoxylate carboligase	QEN71_22530	paras_005043	protein-codi	NZ_CP1252	chromosom	5004411	5006186	+	1776	0	41	7628	37	7465	
QEN71_RS22540	hydroxypyruvate isomerase	QEN71_22535	paras_005044	protein-codi	NZ_CP1252	chromosom	5006312	5007112	+	801	0	13	3111	13	3111	
QEN71_RS22545	2-hydroxy-3-oxopropionate reductase	QEN71_22540	paras_005045	protein-codi	NZ_CP1252	chromosom	5007193	5008101	+	909	0	5	630	3	256	
QEN71_RS22550	IS481 family transposase	QEN71_22545	paras_005046	protein-codi	NZ_CP1252	chromosom	5008518	5009657	+	1140	2266.0	27	16676	20	8775	
QEN71_RS22555	cytochrome c	QEN71_22550	paras_005047	protein-codi	NZ_CP1252	chromosom	5010229	5010534	-	302	0	9	825	6	781	
QEN71_RS22560	PQQ-dependent sugar dehydrogenase	QEN71_22555	paras_005048	protein-codi	NZ_CP1252	chromosom	5010531	5011793	-	1259	0	34	6378	24	4894	
QEN71_RS22565	asparaginase	QEN71_22560	paras_005049	protein-codi	NZ_CP1252	chromosom	5012102	5013130	-	1029	0	27	5704	15	2690	
QEN71_RS22570	CysB family HTH-type transcriptional regulator	QEN71_22565	paras_005050	protein-codi	NZ_CP1252	chromosom	5013209	5014135	-	927	0	18	3537	12	2905	
QEN71_RS22575	sulfate ABC transporter ATP-binding protein	QEN71_22570	paras_005051	protein-codi	NZ_CP1252	chromosom	5014191	5015249	-	1059	0	25	3055	21	2901	
QEN71_RS22580	sulfate ABC transporter permease subunit CysW	QEN71_22575	paras_005052	protein-codi	NZ_CP1252	chromosom	5015265	5016233	-	965	0	17	2594	16	2581	
QEN71_RS22585	sulfate ABC transporter permease subunit CysT	QEN71_22580	paras_005053	protein-codi	NZ_CP1252	chromosom	5016230	5017123	-	890	0	17	2593	9	1302	
QEN71_RS22590	sulfate ABC transporter substrate-binding protein	QEN71_22585	paras_005054	protein-codi	NZ_CP1252	chromosom	5017243	5018286	-	1044	0	28	5615	24	4651	
QEN71_RS22595	transcriptional repressor LexA	QEN71_22590	paras_005055	protein-codi	NZ_CP1252	chromosom	5018652	5019302	+	651	0	15	4137	12	3810	
QEN71_RS22600	hypothetical protein	QEN71_22595	paras_005056	protein-codi	NZ_CP1252	chromosom	5019330	5019674	+	345	0	5	551	3	438	
QEN71_RS22605	DUF2939 domain-containing protein	QEN71_22600	paras_005057	protein-codi	NZ_CP1252	chromosom	5019789	5020451	+	663	0	21	6239	20	6185	
QEN71_RS22610	universal stress protein	QEN71_22605	paras_005058	protein-codi	NZ_CP1252	chromosom	5020482	5020946	-	465	0	4	1157	0	0	
QEN71_RS22615	nodulation factor ABC transporter ATP-binding prot	QEN71_22610	paras_005059	protein-codi	NZ_CP1252	chromosom	5021210	5022124	+	915	0	11	2947	7	1704	
QEN71_RS22620	ABC transporter permease	QEN71_22615	paras_005060	protein-codi	NZ_CP1252	chromosom	5022147	5022974	+	828	0	28	11999	22	8380	
QEN71_RS22625	hypothetical protein	QEN71_22620	paras_005061	protein-codi	NZ_CP1252	chromosom	5023040	5023462	-	423	0	5	2112	5	2112	
QEN71_RS22630	permease	QEN71_22625	paras_005062	protein-codi	NZ_CP1252	chromosom	5023693	5024751	-	1059	0	18	3219	12	1422	
QEN71_RS22635	NADP(H)-dependent aldo-keto reductase	QEN71_22630	paras_005063	protein-codi	NZ_CP1252	chromosom	5025031	5026083	-	1053	0	29	6759	24	5797	
QEN71_RS22640	DUF3820 family protein	QEN71_22635	paras_005064	protein-codi	NZ_CP1252	chromosom	5026302	5026517	+	216	0	8	3099	8	3099	
QEN71_RS22645	tRNA-His	QEN71_22640		tRNA	NZ_CP1252	chromosom	5026621	5026696	-	76	0	0	0	0	0	
QEN71_RS22650	tRNA dihydrouridine(20/20a) synthase DusA	QEN71_22645	paras_005066	protein-codi	NZ_CP1252	chromosom	5026789	5027790	-	1002	0	27	8992	27	8992	
QEN71_RS22655	hypothetical protein	QEN71_22650	paras_005067	protein-codi	NZ_CP1252	chromosom	5028161	5028427	+	267	0	8	2323	5	1590	
QEN71_RS22660	hypothetical protein	QEN71_22655	paras_005068	protein-codi	NZ_CP1252	chromosom	5028808	5029059	-	252	0	4	658	4	658	
QEN71_RS22665	hypothetical protein	QEN71_22660	paras_005069	protein-codi	NZ_CP1252	chromosom	5029131	5029331	-	201	0	4	798	4	798	
QEN71_RS22670	hypothetical protein	QEN71_22665	paras_005070	protein-codi	NZ_CP1252	chromosom	5029536	5029811	+	276	0	4	682	3	640	
QEN71_RS22675	molybdopterin-binding protein	QEN71_22670	paras_005071	protein-codi	NZ_CP1252	chromosom	5029868	5030083	-	216	0	4	1226	4	1226	
QEN71_RS22680	ATP-binding cassette domain-containing protein	QEN71_22675	paras_005072	protein-codi	NZ_CP1252	chromosom	5030139	5031092	-	950	0	10	932	5	563	
QEN71_RS22685	aliphatic sulfonate ABC transporter permease SsuC	QEN71_22680	paras_005073	protein-codi	NZ_CP1252	chromosom	5031089	5031913	-	821	0	20	2437	17	2080	
QEN71_RS22690	FMNH2-dependent alkanesulfonate monooxygenas	QEN71_22685	paras_005074	protein-codi	NZ_CP1252	chromosom	5031971	5033128	-	1158	0	16	2041	8	982	
QEN71_RS22695	hypothetical protein	QEN71_22690	paras_005075	protein-codi	NZ_CP1252	chromosom	5033713	5034132	+	420	0	1	6	0	0	TRUE
QEN71_RS22700	enoyl-CoA hydratase/isomerase family protein	QEN71_22695	paras_005076	protein-codi	NZ_CP1252	chromosom	5034158	5035288	-	1131	0	27	4197	25	3508	
QEN71_RS22705	TetR/AcrR family transcriptional regulator	QEN71_22700	paras_005077	protein-codi	NZ_CP1252	chromosom	5035391	5036116	-	726	0	21	3266	16	2863	

QEN71_RS22710	NAD-dependent epimerase/dehydratase family prot	QEN71_22705	paras_005078	protein-codi	NZ_CP1252	chromosom	5036258	5037238	+	981	0	22	4917	17	4080	
QEN71_RS22715	DUF1488 domain-containing protein	QEN71_22710	paras_005079	protein-codi	NZ_CP1252	chromosom	5037281	5037568	-	288	0	3	505	3	505	
QEN71_RS22720	hypothetical protein	QEN71_22715	paras_005080	protein-codi	NZ_CP1252	chromosom	5037801	5038076	-	276	0	12	4782	11	4713	
QEN71_RS22725	hypothetical protein	QEN71_22720	paras_005081	protein-codi	NZ_CP1252	chromosom	5038232	5038465	+	234	0	1	23	0	0	
QEN71_RS22730	hypothetical protein	QEN71_22725	paras_005082	protein-codi	NZ_CP1252	chromosom	5038580	5038933	+	354	0	7	1684	7	1684	
QEN71_RS22735	multidrug efflux SMR transporter	QEN71_22730	paras_005083	protein-codi	NZ_CP1252	chromosom	5038975	5039313	-	339	0	11	5768	8	2028	
QEN71_RS22740	heme-degrading domain-containing protein	QEN71_22735	paras_005084	protein-codi	NZ_CP1252	chromosom	5039317	5039814	-	498	0	14	3728	9	2261	
QEN71_RS22745	tRNA-Met	QEN71_22740		tRNA	NZ_CP1252	chromosom	5040097	5040173	+	77	0	4	646	4	646	
QEN71_RS22750	NUDIX hydrolase	QEN71_22745	paras_005086	protein-codi	NZ_CP1252	chromosom	5040365	5040910	+	546	0	14	4429	9	2104	
QEN71_RS22755	leucyl/phenylalanyl-tRNA--protein transferase	QEN71_22750	paras_005087	protein-codi	NZ_CP1252	chromosom	5040927	5041664	+	738	0	17	5391	15	5272	
QEN71_RS22760	arginyltransferase	QEN71_22755	paras_005088	protein-codi	NZ_CP1252	chromosom	5041833	5042672	+	840	0	35	12000	30	11247	
QEN71_RS22765	quinone-dependent dihydroorotate dehydrogenase	QEN71_22760	paras_005089	protein-codi	NZ_CP1252	chromosom	5042775	5043809	+	1035	0	14	1652	8	825	
QEN71_RS22770	signal peptidase I	QEN71_22765	paras_005090	protein-codi	NZ_CP1252	chromosom	5043820	5044521	-	702	0	18	2972	10	1396	
QEN71_RS22775	Gfo/Idh/MocA family oxidoreductase	QEN71_22770	paras_005091	protein-codi	NZ_CP1252	chromosom	5044660	5045664	-	1005	0	22	3474	21	3338	
QEN71_RS22780	FadR/GntR family transcriptional regulator	QEN71_22775	paras_005092	protein-codi	NZ_CP1252	chromosom	5045782	5046552	-	771	0	11	1099	7	860	
QEN71_RS22785	hypothetical protein	QEN71_22780		protein-codi	NZ_CP1252	chromosom	5047655	5047912	-	258	0	6	410	6	410	
QEN71_RS22790	Crp/Fnr family transcriptional regulator	QEN71_22785	paras_005093	protein-codi	NZ_CP1252	chromosom	5048056	5048790	+	735	0	7	2018	5	1868	
QEN71_RS22795	4'-phosphopantetheinyl transferase superfamily pro	QEN71_22790	paras_005094	protein-codi	NZ_CP1252	chromosom	5049465	5050175	+	711	0	6	1088	6	1088	
QEN71_RS22800	non-ribosomal peptide synthetase	QEN71_22795	paras_005095	protein-codi	NZ_CP1252	chromosom	5050704	5060624	+	9921	0	138	15034	113	11373	
QEN71_RS22805	condensation domain-containing protein	QEN71_22800	paras_005096	protein-codi	NZ_CP1252	chromosom	5060628	5062955	+	2328	0	38	5430	30	4660	
QEN71_RS22810	SGNH/GDSL hydrolase family protein	QEN71_22805	paras_005097	protein-codi	NZ_CP1252	chromosom	5062978	5064156	+	1179	0	19	2023	17	1811	
QEN71_RS22815	hydrolase	QEN71_22810	paras_005098	protein-codi	NZ_CP1252	chromosom	5064406	5066370	+	1952	0	23	2477	19	1649	
QEN71_RS22820	oligosaccharide flippase family protein	QEN71_22815	paras_005099	protein-codi	NZ_CP1252	chromosom	5066358	5067653	+	1283	0	10	2172	7	1853	
QEN71_RS22825	glycosyltransferase family 25 protein	QEN71_22820	paras_005100	protein-codi	NZ_CP1252	chromosom	5067864	5068649	-	786	0	17	2028	13	1803	
QEN71_RS22830	acyltransferase	QEN71_22825	paras_005101	protein-codi	NZ_CP1252	chromosom	5068918	5070096	+	1179	0	32	2142	24	1479	
QEN71_RS22835	acyltransferase	QEN71_22830	paras_005102	protein-codi	NZ_CP1252	chromosom	5070355	5071443	-	1089	0	26	4194	21	3269	
QEN71_RS22840	acyltransferase	QEN71_22835	paras_005103	protein-codi	NZ_CP1252	chromosom	5071445	5072548	-	1104	0	31	3808	29	3571	
QEN71_RS22845	glycosyltransferase family 1 protein	QEN71_22840	paras_005104	protein-codi	NZ_CP1252	chromosom	5072638	5073744	-	1107	0	24	3686	14	1716	
QEN71_RS22850	glycosyltransferase	QEN71_22845	paras_005105	protein-codi	NZ_CP1252	chromosom	5073796	5074704	-	909	0	11	1346	11	1346	
QEN71_RS22855	hypothetical protein	QEN71_22850	paras_005106	protein-codi	NZ_CP1252	chromosom	5074834	5076252	-	1419	0	34	6414	20	3460	
QEN71_RS22860	glycosyltransferase family 4 protein	QEN71_22855	paras_005107	protein-codi	NZ_CP1252	chromosom	5076311	5077531	-	1208	0	21	3968	18	3856	
QEN71_RS22865	glycosyltransferase family 1 protein	QEN71_22860	paras_005108	protein-codi	NZ_CP1252	chromosom	5077519	5078622	-	1091	0	22	4937	22	4937	
QEN71_RS22870	calcium-binding protein	QEN71_22865	paras_005109	protein-codi	NZ_CP1252	chromosom	5079042	5080412	+	1371	0	36	6611	33	6342	
QEN71_RS22875	cupin-like domain-containing protein	QEN71_22870	paras_005110	protein-codi	NZ_CP1252	chromosom	5080473	5081465	-	993	0	20	2971	12	1498	
QEN71_RS22880	methyltransferase, TIGR04325 family	QEN71_22875	paras_005111	protein-codi	NZ_CP1252	chromosom	5081564	5082415	-	852	0	21	2006	16	1533	
QEN71_RS22885	acyltransferase	QEN71_22880	paras_005112	protein-codi	NZ_CP1252	chromosom	5082747	5083781	+	1035	0	35	6127	27	4528	
QEN71_RS22890	GNVR domain-containing protein	QEN71_22885	paras_005113	protein-codi	NZ_CP1252	chromosom	5084244	5086496	-	2253	0	54	8295	34	5648	
QEN71_RS22895	polysaccharide biosynthesis/export family protein	QEN71_22890	paras_005114	protein-codi	NZ_CP1252	chromosom	5086554	5087642	-	1089	0	25	4036	19	2783	
QEN71_RS22900	sugar transferase	QEN71_22895	paras_005115	protein-codi	NZ_CP1252	chromosom	5088017	5089084	-	1068	0	21	2332	13	1259	
QEN71_RS22905	ribose-5-phosphate isomerase RpiA	QEN71_22900	paras_005116	protein-codi	NZ_CP1252	chromosom	5089697	5090392	-	696	0	1	8	0	0	TRUE
QEN71_RS22910	N-acetylmuramoyl-L-alanine amidase	QEN71_22905	paras_005117	protein-codi	NZ_CP1252	chromosom	5090477	5091343	-	867	0	34	4866	24	3816	
QEN71_RS22915	IS481 family transposase	QEN71_22910	paras_005118	protein-codi	NZ_CP1252	chromosom	5091569	5092708	-	1140	2266.0	0	0	0	0	
QEN71_RS22920	23S rRNA (guanosine(2251)-2'-O)-methyltransferas	QEN71_22915	paras_005119	protein-codi	NZ_CP1252	chromosom	5092909	5093652	-	744	0	24	3132	19	2817	
QEN71_RS22925	ribonuclease R	QEN71_22920	paras_005120	protein-codi	NZ_CP1252	chromosom	5093782	5096238	-	2457	0	56	7593	52	6999	
QEN71_RS22930	tRNA-Leu	QEN71_22925		tRNA	NZ_CP1252	chromosom	5096494	5096580	+	87	152.0	2	161	2	161	
QEN71_RS22935	tRNA-Leu	QEN71_22930		tRNA	NZ_CP1252	chromosom	5096759	5096845	+	87	152.0	4	607	4	607	
QEN71_RS22940	GNAT family N-acetyltransferase	QEN71_22935	paras_005123	protein-codi	NZ_CP1252	chromosom	5096917	5097411	-	495	0	7	1967	5	299	
QEN71_RS22945	MFS transporter	QEN71_22940	paras_005124	protein-codi	NZ_CP1252	chromosom	5097730	5098959	+	1230	0	30	7820	24	7080	
QEN71_RS22950	TetR/AcrR family transcriptional regulator	QEN71_22945	paras_005125	protein-codi	NZ_CP1252	chromosom	5099232	5100086	+	855	0	14	4926	12	4222	
QEN71_RS22955	acyl-CoA dehydrogenase	QEN71_22950	paras_005126	protein-codi	NZ_CP1252	chromosom	5100287	5102785	+	2499	0	49	15668	40	11272	
QEN71_RS22960	3-oxoacyl-ACP reductase	QEN71_22955	paras_005127	protein-codi	NZ_CP1252	chromosom	5102916	5104331	+	1416	0	22	7850	18	7017	
QEN71_RS22965	MaoC/PaaZ C-terminal domain-containing protein	QEN71_22960	paras_005128	protein-codi	NZ_CP1252	chromosom	5104336	5105253	+	918	0	18	3004	12	1416	
QEN71_RS22970	acetyl-CoA C-acetyltransferase	QEN71_22965	paras_005129	protein-codi	NZ_CP1252	chromosom	5105341	5106681	+	1341	0	31	5564	25	4303	
QEN71_RS22975	AMP-binding protein	QEN71_22970	paras_005130	protein-codi	NZ_CP1252	chromosom	5106759	5108549	+	1791	0	39	5200	32	3780	
QEN71_RS22980	DUF1571 domain-containing protein	QEN71_22975	paras_005131	protein-codi	NZ_CP1252	chromosom	5108652	5109542	+	891	0	24	5432	22	5167	
QEN71_RS22985	hypothetical protein	QEN71_22980	paras_005132	protein-codi	NZ_CP1252	chromosom	5109543	5109935	-	393	0	4	356	4	356	
QEN71_RS22990	FAD-binding oxidoreductase	QEN71_22985	paras_005133	protein-codi	NZ_CP1252	chromosom	5109944	5111524	-	1581	0	45	10368	33	5843	
QEN71_RS22995	hypothetical protein	QEN71_22990	paras_005134	protein-codi	NZ_CP1252	chromosom	5111594	5112097	-	504	0	16	6603	13	5346	
QEN71_RS23000	anti-sigma factor	QEN71_22995	paras_005135	protein-codi	NZ_CP1252	chromosom	5112336	5113130	-	795	0	26	3644	23	3253	
QEN71_RS23005	sigma-70 family RNA polymerase sigma factor	QEN71_23000	paras_005136	protein-codi	NZ_CP1252	chromosom	5113162	5113680	-	519	0	19	8261	18	8175	
QEN71_RS23010	hypothetical protein	QEN71_23005	paras_005137	protein-codi	NZ_CP1252	chromosom	5113714	5113863	-	150	0	5	214	4	204	
QEN71_RS23015	RNA chaperone Hfq	QEN71_23010	paras_005138	protein-codi	NZ_CP1252	chromosom	5114367	5114813	+	447	0	18	4928	14	3075	

QEN71_RS23020	hypothetical protein	QEN71_23015	paras_005139	protein-codi	NZ_CP1252	chromosom	5114868	5115104	-	237	0	9	2729	3	92	
QEN71_RS23025	hypothetical protein	QEN71_23020	paras_005140	protein-codi	NZ_CP1252	chromosom	5115375	5115545	+	171	0	0	0	0	0	
QEN71_RS23030	DUF2968 domain-containing protein	QEN71_23025	paras_005141	protein-codi	NZ_CP1252	chromosom	5115717	5116430	-	714	0	22	8231	21	8083	
QEN71_RS23035	DUF2147 domain-containing protein	QEN71_23030	paras_005142	protein-codi	NZ_CP1252	chromosom	5116778	5117263	+	486	0	12	4591	10	4476	
QEN71_RS23040	transposase	QEN71_23035	paras_005143	protein-codi	NZ_CP1252	chromosom	5117393	5117842	-	450	0	16	5152	12	3974	
QEN71_RS23045	cell division protein ZapE	QEN71_23040	paras_005144	protein-codi	NZ_CP1252	chromosom	5118000	5119097	-	1098	0	51	8413	38	5839	
QEN71_RS23050	dihydrolipoyl dehydrogenase	QEN71_23045	paras_005145	protein-codi	NZ_CP1252	chromosom	5119195	5120625	-	1431	0	2	2176	0	0	
QEN71_RS23055	2-oxoglutarate dehydrogenase complex dihydrolipo	QEN71_23050	paras_005146	protein-codi	NZ_CP1252	chromosom	5120764	5122044	-	1281	0	2	318	0	0	TRUE
QEN71_RS23060	2-oxoglutarate dehydrogenase E1 component	QEN71_23055	paras_005147	protein-codi	NZ_CP1252	chromosom	5122187	5125042	-	2856	0	5	180	1	3	TRUE
QEN71_RS23065	translational GTPase TypA	QEN71_23060	paras_005148	protein-codi	NZ_CP1252	chromosom	5125406	5127232	-	1827	0	12	211	8	88	
QEN71_RS23070	MarR family winged helix-turn-helix transcriptional r	QEN71_23065	paras_005149	protein-codi	NZ_CP1252	chromosom	5127547	5128062	+	516	0	7	564	4	133	
QEN71_RS23075	efflux transporter outer membrane subunit	QEN71_23070	paras_005150	protein-codi	NZ_CP1252	chromosom	5128119	5129627	+	1509	0	31	9495	28	8713	
QEN71_RS23080	EmrA/EmrK family multidrug efflux transporter perip	QEN71_23075	paras_005151	protein-codi	NZ_CP1252	chromosom	5129697	5130917	+	1221	0	39	11056	29	8519	
QEN71_RS23085	DHA2 family efflux MFS transporter permease subu	QEN71_23080	paras_005152	protein-codi	NZ_CP1252	chromosom	5130954	5132519	+	1566	0	42	9168	34	6839	
QEN71_RS23090	tRNA pseudouridine(55) synthase TruB	QEN71_23085	paras_005153	protein-codi	NZ_CP1252	chromosom	5133119	5134048	-	930	0	30	9136	19	5225	
QEN71_RS23095	30S ribosome-binding factor RbfA	QEN71_23090	paras_005154	protein-codi	NZ_CP1252	chromosom	5134073	5134438	-	366	0	0	0	0	0	TRUE
QEN71_RS23100	translation initiation factor IF-2	QEN71_23095	paras_005155	protein-codi	NZ_CP1252	chromosom	5134604	5137504	-	2901	0	3	398	1	2	TRUE
QEN71_RS23105	transcription termination factor NusA	QEN71_23100	paras_005156	protein-codi	NZ_CP1252	chromosom	5137601	5139076	-	1472	0	3	689	1	24	TRUE
QEN71_RS23110	ribosome maturation factor RimP	QEN71_23105	paras_005157	protein-codi	NZ_CP1252	chromosom	5139073	5139531	-	455	0	10	3155	9	2808	
QEN71_RS23115	pseudouridine synthase	QEN71_23110	paras_005158	protein-codi	NZ_CP1252	chromosom	5139951	5141780	-	1830	0	3	18	2	14	TRUE
QEN71_RS23120	SMC-Scp complex subunit ScpB	QEN71_23115	paras_005159	protein-codi	NZ_CP1252	chromosom	5141841	5142884	-	1044	0	1	2	1	2	TRUE
QEN71_RS23125	pyridoxal phosphate-dependent aminotransferase	QEN71_23120	paras_005160	protein-codi	NZ_CP1252	chromosom	5143198	5144382	-	1185	0	28	17648	26	17062	
QEN71_RS23130	LysR family transcriptional regulator	QEN71_23125	paras_005161	protein-codi	NZ_CP1252	chromosom	5144535	5145437	-	903	0	4	2133	4	2133	
QEN71_RS23135	transposase	partial;pseudo;QEN71_23130		protein-codi	NZ_CP1252	chromosom	5145855	5146256	+	402	0	5	1085	5	1085	
QEN71_RS23140	hypothetical protein	QEN71_23135	paras_005163	protein-codi	NZ_CP1252	chromosom	5146629	5146898	+	270	0	3	722	3	722	
QEN71_RS23145	hypothetical protein	QEN71_23140	paras_005164	protein-codi	NZ_CP1252	chromosom	5147227	5147541	-	315	0	13	2440	7	1085	
QEN71_RS23150	YdcF family protein	QEN71_23145	paras_005165	protein-codi	NZ_CP1252	chromosom	5147838	5148524	+	687	0	19	5389	19	5389	
QEN71_RS23155	hypothetical protein	QEN71_23150	paras_005166	protein-codi	NZ_CP1252	chromosom	5148866	5149102	+	237	0	5	1271	3	543	
QEN71_RS23160	DNA-binding protein	QEN71_23155	paras_005167	protein-codi	NZ_CP1252	chromosom	5149323	5149580	+	258	0	2	511	2	511	
QEN71_RS23165	putative quinol monooxygenase	QEN71_23160	paras_005168	protein-codi	NZ_CP1252	chromosom	5149669	5149959	-	291	0	5	1093	5	1093	
QEN71_RS23170	EAL domain-containing protein	QEN71_23165	paras_005169	protein-codi	NZ_CP1252	chromosom	5150287	5151561	+	1275	0	24	5448	16	2926	
QEN71_RS23175	alternative ribosome rescue aminoacyl-tRNA hydrol	QEN71_23170	paras_005170	protein-codi	NZ_CP1252	chromosom	5151571	5151978	-	408	0	3	515	2	509	
QEN71_RS23180	VC0807 family protein	QEN71_23175	paras_005171	protein-codi	NZ_CP1252	chromosom	5152106	5152765	+	660	0	10	1361	10	1361	
QEN71_RS23185	exodeoxyribonuclease V subunit alpha	QEN71_23180	paras_005172	protein-codi	NZ_CP1252	chromosom	5152790	5154835	-	2042	0	10	207	6	57	
QEN71_RS23190	exodeoxyribonuclease V subunit beta	QEN71_23185	paras_005173	protein-codi	NZ_CP1252	chromosom	5154832	5158557	-	3718	0	11	314	8	303	
QEN71_RS23195	exodeoxyribonuclease V subunit gamma	QEN71_23190	paras_005174	protein-codi	NZ_CP1252	chromosom	5158554	5161919	-	3362	0	4	64	3	30	TRUE
QEN71_RS23200	FUSC family membrane protein	QEN71_23195	paras_005175	protein-codi	NZ_CP1252	chromosom	5162118	5164544	+	2427	0	56	10418	42	7969	
QEN71_RS23205	hypothetical protein	QEN71_23200	paras_005176	protein-codi	NZ_CP1252	chromosom	5164567	5164773	-	207	0	9	1012	7	765	
QEN71_RS23210	Lrp/AsnC family transcriptional regulator	QEN71_23205	paras_005177	protein-codi	NZ_CP1252	chromosom	5164909	5165391	-	483	0	12	1600	12	1600	
QEN71_RS23215	cysteine dioxygenase family protein	QEN71_23210	paras_005178	protein-codi	NZ_CP1252	chromosom	5165524	5166156	+	633	0	19	7169	12	3821	
QEN71_RS23220	mechanosensitive ion channel family protein	QEN71_23215	paras_005179	protein-codi	NZ_CP1252	chromosom	5166241	5168898	-	2658	0	26	5876	19	3909	
QEN71_RS23225	NAD(P)-dependent oxidoreductase	QEN71_23220	paras_005180	protein-codi	NZ_CP1252	chromosom	5169085	5169732	-	648	0	7	1858	6	1826	
QEN71_RS23230	Rrf2 family transcriptional regulator	QEN71_23225	paras_005181	protein-codi	NZ_CP1252	chromosom	5169840	5170304	-	465	0	8	1168	3	950	
QEN71_RS23235	hypothetical protein	QEN71_23230	paras_005182	protein-codi	NZ_CP1252	chromosom	5170400	5170861	-	462	0	4	1144	2	338	
QEN71_RS23240	DJ-1/PfpI family protein	QEN71_23235	paras_005183	protein-codi	NZ_CP1252	chromosom	5171011	5171592	+	582	0	12	2711	10	2558	
QEN71_RS23245	hypothetical protein	QEN71_23240	paras_005184	protein-codi	NZ_CP1252	chromosom	5171690	5172013	+	324	0	6	427	2	65	
QEN71_RS23250	sensor histidine kinase	QEN71_23245	paras_005185	protein-codi	NZ_CP1252	chromosom	5172036	5173688	-	1653	0	22	4390	15	2990	
QEN71_RS23255	ammonium transporter	QEN71_23250	paras_005186	protein-codi	NZ_CP1252	chromosom	5174114	5175316	+	1203	0	21	4474	18	4155	
QEN71_RS23260	fluoride efflux transporter CrcB	QEN71_23255	paras_005187	protein-codi	NZ_CP1252	chromosom	5175588	5175968	+	381	0	7	4565	4	1491	
QEN71_RS23265	hypothetical protein	QEN71_23260	paras_005188	protein-codi	NZ_CP1252	chromosom	5176205	5176456	+	252	0	11	3862	2	632	
QEN71_RS23270	hypothetical protein	QEN71_23265	paras_005189	protein-codi	NZ_CP1252	chromosom	5176471	5176737	+	267	0	6	804	6	804	
QEN71_RS23275	hypothetical protein			protein-codi	NZ_CP1252	chromosom	5176768	5177148	-	381	0	4	777	2	41	
QEN71_RS23280	CHAD domain-containing protein	QEN71_23275	paras_005191	protein-codi	NZ_CP1252	chromosom	5177484	5178359	-	876	0	9	1503	9	1503	
QEN71_RS23285	hypothetical protein	QEN71_23280	paras_005192	protein-codi	NZ_CP1252	chromosom	5178483	5179346	-	864	0	24	8707	15	3109	
QEN71_RS23290	PRC-barrel domain-containing protein	QEN71_23285	paras_005193	protein-codi	NZ_CP1252	chromosom	5179546	5180010	+	465	0	9	2903	9	2903	
QEN71_RS23295	IclR family transcriptional regulator	QEN71_23290	paras_005194	protein-codi	NZ_CP1252	chromosom	5180665	5181501	+	837	0	13	1772	13	1772	
QEN71_RS23300	MdtA/MuxA family multidrug efflux RND transporter	QEN71_23295	paras_005195	protein-codi	NZ_CP1252	chromosom	5181987	5183411	+	1425	0	22	4051	21	3995	
QEN71_RS23305	MdtB/MuxB family multidrug efflux RND transporter	QEN71_23300	paras_005196	protein-codi	NZ_CP1252	chromosom	5183427	5186576	+	3150	0	64	13646	53	11469	
QEN71_RS23310	efflux RND transporter permease subunit	QEN71_23305	paras_005197	protein-codi	NZ_CP1252	chromosom	5186594	5189899	+	3306	0	73	14864	54	11107	
QEN71_RS23315	hypothetical protein	QEN71_23310	paras_005198	protein-codi	NZ_CP1252	chromosom	5190565	5191029	-	465	0	12	1114	8	405	
QEN71_RS23320	peroxiredoxin	QEN71_23315	paras_005199	protein-codi	NZ_CP1252	chromosom	5191088	5191645	-	558	0	31	6933	25	5338	
QEN71_RS23325	EAL domain-containing protein	QEN71_23320	paras_005200	protein-codi	NZ_CP1252	chromosom	5191780	5193105	-	1326	0	17	2806	14	1761	

QEN71_RS23330	sensor domain-containing phosphodiesterase	QEN71_23325	paras_005201	protein-codi	NZ_CP1252	chromosom	5193451	5195280	+	1830	0	44	14781	44	14781	
QEN71_RS23335	HDOD domain-containing protein	QEN71_23330	paras_005202	protein-codi	NZ_CP1252	chromosom	5195366	5196823	+	1458	0	25	5635	17	4576	
QEN71_RS23340	EAL domain-containing protein	QEN71_23335	paras_005203	protein-codi	NZ_CP1252	chromosom	5196930	5198309	-	1380	0	20	4111	17	3566	
QEN71_RS23345	ABC transporter substrate-binding protein	QEN71_23340	paras_005204	protein-codi	NZ_CP1252	chromosom	5198631	5199428	-	798	0	19	3213	11	2625	
QEN71_RS23350	succinylglutamate desuccinylase	QEN71_23345	paras_005205	protein-codi	NZ_CP1252	chromosom	5200340	5201398	-	1042	0	25	3166	17	2127	
QEN71_RS23355	N-succinylarginine dihydrolase	QEN71_23350	paras_005206	protein-codi	NZ_CP1252	chromosom	5201382	5202722	-	1324	0	10	83	7	55	
QEN71_RS23360	succinylglutamate-semialdehyde dehydrogenase	QEN71_23355	paras_005207	protein-codi	NZ_CP1252	chromosom	5202732	5204195	-	1464	0	7	204	4	91	TRUE
QEN71_RS23365	arginine N-succinyltransferase	QEN71_23360	paras_005208	protein-codi	NZ_CP1252	chromosom	5204204	5205229	-	1022	0	3	164	1	3	TRUE
QEN71_RS23370	arginine/ornithine succinyltransferase subunit alpha	QEN71_23365	paras_005209	protein-codi	NZ_CP1252	chromosom	5205226	5206278	-	1049	0	10	2527	10	2527	
QEN71_RS23375	aspartate aminotransferase family protein	QEN71_23370	paras_005210	protein-codi	NZ_CP1252	chromosom	5206317	5207546	-	1230	0	5	149	4	142	
QEN71_RS23380	GlxA family transcriptional regulator	QEN71_23375	paras_005211	protein-codi	NZ_CP1252	chromosom	5208317	5209339	-	1023	0	14	730	10	335	
QEN71_RS23385	ABC transporter ATP-binding protein	QEN71_23380	paras_005212	protein-codi	NZ_CP1252	chromosom	5209364	5210137	-	774	0	4	265	3	257	
QEN71_RS23390	ABC transporter permease	QEN71_23385	paras_005213	protein-codi	NZ_CP1252	chromosom	5210274	5210984	-	711	0	29	4603	26	4195	
QEN71_RS23395	histidine ABC transporter permease HisQ	QEN71_23390	paras_005214	protein-codi	NZ_CP1252	chromosom	5211033	5211722	-	690	0	18	2572	14	2459	
QEN71_RS23400	class I SAM-dependent methyltransferase	QEN71_23395	paras_005215	protein-codi	NZ_CP1252	chromosom	5212160	5212768	+	609	0	11	5045	7	4596	
QEN71_RS23405	patatin-like phospholipase family protein	QEN71_23400	paras_005216	protein-codi	NZ_CP1252	chromosom	5212789	5213817	-	1029	0	6	602	3	169	
QEN71_RS23410	hypothetical protein	QEN71_23405	paras_005217	protein-codi	NZ_CP1252	chromosom	5213930	5214472	-	543	0	5	2663	3	2030	
QEN71_RS23415	LLM class flavin-dependent oxidoreductase	QEN71_23410	paras_005218	protein-codi	NZ_CP1252	chromosom	5215337	5216329	+	993	0	16	4015	13	3044	
QEN71_RS23420	alpha/beta hydrolase	QEN71_23415	paras_005219	protein-codi	NZ_CP1252	chromosom	5216380	5217465	-	1086	0	34	10843	32	9722	
QEN71_RS23425	glucose-1-phosphate adenylyltransferase	QEN71_23420	paras_005220	protein-codi	NZ_CP1252	chromosom	5217916	5219184	+	1269	0	28	11870	27	11867	
QEN71_RS23430	glycogen synthase GlgA	QEN71_23425	paras_005221	protein-codi	NZ_CP1252	chromosom	5219344	5220804	+	1461	0	35	7150	28	6422	
QEN71_RS23435	pyridoxal kinase PdxY	QEN71_23430	paras_005222	protein-codi	NZ_CP1252	chromosom	5221084	5221947	+	864	0	10	1519	6	1038	
QEN71_RS23440	hypothetical protein	QEN71_23435	paras_005223	protein-codi	NZ_CP1252	chromosom	5222045	5222506	+	462	0	20	2727	20	2727	
QEN71_RS23445	IS481 family transposase	QEN71_23440	paras_005224	protein-codi	NZ_CP1252	chromosom	5222664	5223803	-	1140	2266.0	12	214	9	116	
QEN71_RS23450	DUF3857 domain-containing protein	QEN71_23445	paras_005225	protein-codi	NZ_CP1252	chromosom	5224356	5226257	-	1898	0	41	6325	34	5231	
QEN71_RS23455	AraC family transcriptional regulator	QEN71_23450	paras_005226	protein-codi	NZ_CP1252	chromosom	5226254	5227174	-	917	0	14	3248	14	3248	
QEN71_RS23460	hypothetical protein	QEN71_23455	paras_005227	protein-codi	NZ_CP1252	chromosom	5227283	5227831	+	549	0	11	3635	11	3635	
QEN71_RS23465	DHA2 family efflux MFS transporter permease subu	QEN71_23460	paras_005228	protein-codi	NZ_CP1252	chromosom	5227838	5229229	-	1392	0	18	2729	16	2468	
QEN71_RS23470	aminotransferase class I/II-fold pyridoxal phosphate	QEN71_23465	paras_005229	protein-codi	NZ_CP1252	chromosom	5229350	5230573	-	1224	0	25	6057	23	5366	
QEN71_RS23475	LysR family transcriptional regulator	QEN71_23470	paras_005230	protein-codi	NZ_CP1252	chromosom	5230698	5231552	+	855	0	6	850	4	654	
QEN71_RS23480	hypothetical protein	QEN71_23475	paras_005231	protein-codi	NZ_CP1252	chromosom	5231575	5232237	-	663	0	8	1465	4	1095	
QEN71_RS23485	N-formylglutamate deformylase	QEN71_23480	paras_005232	protein-codi	NZ_CP1252	chromosom	5232571	5233383	-	813	0	28	5582	28	5582	
QEN71_RS23490	formimidoylglutamate deiminase	QEN71_23485	paras_005233	protein-codi	NZ_CP1252	chromosom	5233430	5234821	-	1392	0	46	5266	33	3759	
QEN71_RS23495	imidazolonepropionase	QEN71_23490	paras_005234	protein-codi	NZ_CP1252	chromosom	5234824	5236038	-	1215	0	16	1877	14	1830	
QEN71_RS23500	HutD family protein	QEN71_23495	paras_005235	protein-codi	NZ_CP1252	chromosom	5236069	5236680	-	612	0	2	236	2	236	
QEN71_RS23505	urocanate hydratase	QEN71_23500	paras_005236	protein-codi	NZ_CP1252	chromosom	5236689	5238377	-	1689	0	25	5978	24	5966	
QEN71_RS23510	histidine utilization repressor	QEN71_23505	paras_005237	protein-codi	NZ_CP1252	chromosom	5238439	5239134	-	696	0	8	483	8	483	
QEN71_RS23515	histidine ammonia-lyase	QEN71_23510	paras_005238	protein-codi	NZ_CP1252	chromosom	5239177	5240700	-	1524	0	16	3041	10	2587	
QEN71_RS23520	NAD-dependent epimerase/dehydratase family prot	QEN71_23515	paras_005239	protein-codi	NZ_CP1252	chromosom	5241095	5242000	+	906	0	16	2826	15	2811	
QEN71_RS23525	hypothetical protein	QEN71_23520	paras_005240	protein-codi	NZ_CP1252	chromosom	5242164	5242346	+	183	0	5	285	4	251	
QEN71_RS23530	thioredoxin family protein	QEN71_23525	paras_005241	protein-codi	NZ_CP1252	chromosom	5242389	5243165	-	777	0	22	4397	21	4299	
QEN71_RS23535	SRPBCC domain-containing protein	QEN71_23530	paras_005242	protein-codi	NZ_CP1252	chromosom	5243217	5243645	-	335	0	5	1474	4	950	
QEN71_RS23540	metalloregulator ArsR/SmtB family transcription fact	QEN71_23535	paras_005243	protein-codi	NZ_CP1252	chromosom	5243552	5243974	-	329	0	7	693	6	685	
QEN71_RS23545	NCS2 family permease	QEN71_23540	paras_005244	protein-codi	NZ_CP1252	chromosom	5244076	5245485	-	1410	0	20	3847	16	3226	
QEN71_RS23550	PRC-barrel domain-containing protein	QEN71_23545	paras_005245	protein-codi	NZ_CP1252	chromosom	5246066	5247103	+	1034	0	5	1300	3	750	
QEN71_RS23555	MFS transporter	QEN71_23550	paras_005246	protein-codi	NZ_CP1252	chromosom	5247100	5248371	+	1268	0	29	4881	19	3529	
QEN71_RS23560	IS481 family transposase	QEN71_23555	paras_005247	protein-codi	NZ_CP1252	chromosom	5248552	5249691	-	1140	2266.0	7	51	6	45	
QEN71_RS23565	MFS transporter	QEN71_23560	paras_005248	protein-codi	NZ_CP1252	chromosom	5249902	5251251	-	1350	0	54	8627	45	7018	
QEN71_RS23570	M20 aminoacylase family protein	QEN71_23565	paras_005249	protein-codi	NZ_CP1252	chromosom	5251270	5252442	-	1173	0	12	855	10	819	
QEN71_RS23575	LysR substrate-binding domain-containing protein	QEN71_23570	paras_005250	protein-codi	NZ_CP1252	chromosom	5252581	5253531	+	951	0	7	267	5	226	
QEN71_RS23580	aldehyde dehydrogenase family protein	QEN71_23575	paras_005251	protein-codi	NZ_CP1252	chromosom	5253547	5254974	-	1428	0	26	1655	19	1276	
QEN71_RS23585	cupin domain-containing protein	QEN71_23580	paras_005252	protein-codi	NZ_CP1252	chromosom	5255126	5255497	+	372	0	15	393	6	82	
QEN71_RS23590	FAD-dependent oxidoreductase	QEN71_23585	paras_005253	protein-codi	NZ_CP1252	chromosom	5256156	5257547	-	1392	0	40	2840	31	2544	
QEN71_RS23595	cytosine permease	QEN71_23590	paras_005254	protein-codi	NZ_CP1252	chromosom	5257609	5259198	-	1590	0	28	2790	20	2170	
QEN71_RS23600	LysR family transcriptional regulator	QEN71_23595	paras_005255	protein-codi	NZ_CP1252	chromosom	5259459	5260412	-	954	0	16	1168	14	1103	
QEN71_RS23605	NAD(P)/FAD-dependent oxidoreductase	QEN71_23600	paras_005256	protein-codi	NZ_CP1252	chromosom	5260540	5261892	+	1353	0	18	1235	13	1098	
QEN71_RS23610	LysR family transcriptional regulator ArgP	QEN71_23605	paras_005257	protein-codi	NZ_CP1252	chromosom	5261905	5262798	-	894	0	14	1030	13	1028	
QEN71_RS23615	LysE/ArgO family amino acid transporter	QEN71_23610	paras_005258	protein-codi	NZ_CP1252	chromosom	5262897	5263520	+	624	0	10	1986	10	1986	
QEN71_RS23620	endo alpha-1,4 polygalactosaminidase	QEN71_23615		pseudogene	NZ_CP1252	chromosom	5263611	5264348	-	738	0	19	1831	14	878	
QEN71_RS23625	exopolysaccharide Pel transporter PelG	QEN71_23620	paras_005260	protein-codi	NZ_CP1252	chromosom	5264511	5265884	-	1373	0	56	5697	44	4769	
QEN71_RS23630	GT4 family glycosyltransferase PelF	QEN71_23625	paras_005261	protein-codi	NZ_CP1252	chromosom	5265884	5267500	-	1612	0	40	5101	32	4498	
QEN71_RS23635	hypothetical protein	QEN71_23630	paras_005262	protein-codi	NZ_CP1252	chromosom	5267497	5268546	-	1038	0	18	2755	16	2597	

QEN71_RS23640	PelD GGDEF domain-containing protein	QEN71_23635	paras_005263	protein-codi	NZ_CP1252	chromosom	5268539	5269942	-	1396	0	15	1598	12	887	
QEN71_RS23645	penicillin-binding protein activator LpoB	QEN71_23640	paras_005264	protein-codi	NZ_CP1252	chromosom	5270049	5270600	-	552	0	4	911	4	911	
QEN71_RS23650	tetratricopeptide repeat protein	QEN71_23645	paras_005265	protein-codi	NZ_CP1252	chromosom	5270653	5274621	-	3958	0	96	17770	75	13780	
QEN71_RS23655	sugar ABC transporter	QEN71_23650	paras_005266	protein-codi	NZ_CP1252	chromosom	5274611	5277472	-	2851	0	81	19782	63	15113	
QEN71_RS23660	AraC family transcriptional regulator	QEN71_23655	paras_005267	protein-codi	NZ_CP1252	chromosom	5277976	5278791	+	816	0	23	7774	15	5650	
QEN71_RS23665	LysE family transporter	QEN71_23660	paras_005268	protein-codi	NZ_CP1252	chromosom	5278880	5279530	+	651	0	16	4047	12	3689	
QEN71_RS23670	DUF1328 domain-containing protein	QEN71_23665	paras_005269	protein-codi	NZ_CP1252	chromosom	5279711	5279872	+	162	0	8	2042	6	1615	
QEN71_RS23675	hypothetical protein	QEN71_23670	paras_005270	protein-codi	NZ_CP1252	chromosom	5279970	5280173	-	204	0	8	2840	8	2840	
QEN71_RS23680	TetR/AcrR family transcriptional regulator	QEN71_23675	paras_005271	protein-codi	NZ_CP1252	chromosom	5280368	5281039	-	672	0	15	3725	11	2642	
QEN71_RS23685	LysR substrate-binding domain-containing protein	QEN71_23680	paras_005272	protein-codi	NZ_CP1252	chromosom	5281212	5282111	-	900	0	7	1092	7	1092	
QEN71_RS23690	EamA family transporter	QEN71_23685	paras_005273	protein-codi	NZ_CP1252	chromosom	5282227	5283120	+	894	0	20	7135	20	7135	
QEN71_RS23695	SgcJ/EcaC family oxidoreductase	QEN71_23690	paras_005274	protein-codi	NZ_CP1252	chromosom	5283226	5283627	+	402	0	2	401	0	0	
QEN71_RS23700	alpha-ketoglutarate dehydrogenase	QEN71_23695	paras_005275	protein-codi	NZ_CP1252	chromosom	5283634	5286357	-	2724	0	76	17312	62	12792	
QEN71_RS23705	Lrp/AsnC family transcriptional regulator	QEN71_23700	paras_005276	protein-codi	NZ_CP1252	chromosom	5286516	5287061	+	546	0	11	2408	11	2408	
QEN71_RS23710	acyl-CoA synthetase	QEN71_23705	paras_005277	protein-codi	NZ_CP1252	chromosom	5287063	5288700	-	1638	0	22	6350	11	2962	
QEN71_RS23715	hypothetical protein	QEN71_23710	paras_005278	protein-codi	NZ_CP1252	chromosom	5288926	5289198	+	273	0	14	2698	9	1581	
QEN71_RS23720	DUF2591 domain-containing protein	QEN71_23715	paras_005279	protein-codi	NZ_CP1252	chromosom	5289220	5289597	-	378	0	4	213	0	0	
QEN71_RS23725	SGNH/GDSL hydrolase family protein	QEN71_23720	paras_005280	protein-codi	NZ_CP1252	chromosom	5289623	5290537	+	915	0	31	6356	17	2338	
QEN71_RS23730	PAAR domain-containing protein	QEN71_23725	paras_005281	protein-codi	NZ_CP1252	chromosom	5290564	5290851	-	288	0	3	106	0	0	TRUE
QEN71_RS23735	hypothetical protein	QEN71_23730	paras_005282	protein-codi	NZ_CP1252	chromosom	5291250	5291435	+	186	0	2	150	2	150	
QEN71_RS23740	tRNA-Ser	QEN71_23735		tRNA	NZ_CP1252	chromosom	5291494	5291584	+	91	0	0	0	0	0	
QEN71_RS23745	hypothetical protein	QEN71_23740	paras_005284	protein-codi	NZ_CP1252	chromosom	5291748	5292230	-	475	0	21	4355	16	4072	
QEN71_RS23750	hypothetical protein	QEN71_23745	paras_005285	protein-codi	NZ_CP1252	chromosom	5292223	5292981	-	751	0	17	2805	10	1710	
QEN71_RS23755	sodium:solute symporter family protein	QEN71_23750	paras_005286	protein-codi	NZ_CP1252	chromosom	5293146	5295164	-	2015	0	74	21804	45	9975	
QEN71_RS23760	DUF4212 domain-containing protein	QEN71_23755	paras_005287	protein-codi	NZ_CP1252	chromosom	5295161	5295526	-	362	0	18	5735	18	5735	
QEN71_RS23765	acetate--CoA ligase	QEN71_23760	paras_005288	protein-codi	NZ_CP1252	chromosom	5295674	5297656	-	1983	0	48	15725	44	14470	
QEN71_RS23770	DMT family transporter	QEN71_23765	paras_005289	protein-codi	NZ_CP1252	chromosom	5298022	5298921	+	900	0	15	2213	13	2106	
QEN71_RS23775	TIGR00645 family protein	QEN71_23770	paras_005290	protein-codi	NZ_CP1252	chromosom	5299155	5299757	+	603	0	11	1773	11	1773	
QEN71_RS23780	fumarate hydratase	QEN71_23775	paras_005291	protein-codi	NZ_CP1252	chromosom	5299818	5301341	+	1524	0	31	7379	14	3320	
QEN71_RS23785	bacterioferritin	QEN71_23780	paras_005292	protein-codi	NZ_CP1252	chromosom	5301512	5301991	+	480	0	19	4479	13	3764	
QEN71_RS23790	glutamate racemase	QEN71_23785	paras_005293	protein-codi	NZ_CP1252	chromosom	5302103	5302987	+	885	0	0	0	0	0	TRUE
QEN71_RS23795	(2Fe-2S)-binding protein	QEN71_23790	paras_005294	protein-codi	NZ_CP1252	chromosom	5303217	5303453	+	237	0	2	443	1	11	
QEN71_RS23800	hypothetical protein	QEN71_23795	paras_005295	protein-codi	NZ_CP1252	chromosom	5303489	5303806	-	226	0	5	981	4	778	
QEN71_RS23805	energy transducer TonB	QEN71_23800	paras_005296	protein-codi	NZ_CP1252	chromosom	5303715	5304446	+	640	0	3	19	0	0	TRUE
QEN71_RS23810	MotA/TolQ/ExbB proton channel family protein	QEN71_23805	paras_005297	protein-codi	NZ_CP1252	chromosom	5304484	5305215	+	732	0	4	275	2	10	TRUE
QEN71_RS23815	biopolymer transporter ExbD	QEN71_23810	paras_005298	protein-codi	NZ_CP1252	chromosom	5305232	5305651	+	420	0	0	0	0	0	TRUE
QEN71_RS23820	LysR family transcriptional regulator	QEN71_23815	paras_005299	protein-codi	NZ_CP1252	chromosom	5306034	5307014	-	981	0	22	8238	18	6878	
QEN71_RS23825	pirin family protein	QEN71_23820	paras_005300	protein-codi	NZ_CP1252	chromosom	5307136	5308008	+	873	0	28	8091	22	6340	
QEN71_RS23830	OsmC family protein	QEN71_23825	paras_005301	protein-codi	NZ_CP1252	chromosom	5308149	5308547	+	399	0	4	1975	4	1975	
QEN71_RS23835	SRPBCC family protein	QEN71_23830	paras_005302	protein-codi	NZ_CP1252	chromosom	5308611	5309102	+	492	0	17	4727	11	3169	
QEN71_RS23840	hemin uptake protein HemP	QEN71_23835	paras_005303	protein-codi	NZ_CP1252	chromosom	5309245	5309517	+	273	0	7	3412	5	1502	
QEN71_RS23845	heme-binding protein	QEN71_23840	paras_005304	protein-codi	NZ_CP1252	chromosom	5309794	5310201	-	408	0	9	1478	7	1242	
QEN71_RS23850	4Fe-4S binding protein	QEN71_23845	paras_005305	protein-codi	NZ_CP1252	chromosom	5310239	5311648	-	1406	0	44	5527	36	4896	
QEN71_RS23855	FTR1 family protein	QEN71_23850	paras_005306	protein-codi	NZ_CP1252	chromosom	5311645	5312490	-	842	0	24	2477	16	1941	
QEN71_RS23860	cupredoxin domain-containing protein	QEN71_23855	paras_005307	protein-codi	NZ_CP1252	chromosom	5312554	5312889	-	336	0	7	785	7	785	
QEN71_RS23865	iron transporter	QEN71_23860	paras_005308	protein-codi	NZ_CP1252	chromosom	5312959	5313507	-	549	0	17	1927	13	1758	
QEN71_RS23870	excinuclease ABC subunit UvrB	QEN71_23865	paras_005309	protein-codi	NZ_CP1252	chromosom	5313860	5315953	-	2094	0	71	21951	60	19130	
QEN71_RS23875	amino acid aminotransferase	QEN71_23870	paras_005310	protein-codi	NZ_CP1252	chromosom	5316332	5317531	+	1200	0	32	5961	20	3847	
QEN71_RS23880	patatin-like phospholipase family protein	QEN71_23875	paras_005311	protein-codi	NZ_CP1252	chromosom	5317628	5318881	-	1254	0	37	9189	37	9189	
QEN71_RS23885	3-hydroxybutyrate dehydrogenase	QEN71_23880	paras_005312	protein-codi	NZ_CP1252	chromosom	5318908	5319705	-	798	0	21	8411	19	7291	
QEN71_RS23890	aldo/keto reductase	QEN71_23885	paras_005313	protein-codi	NZ_CP1252	chromosom	5319921	5320892	+	972	0	37	11205	32	9850	
QEN71_RS23895	23S rRNA (adenine(2030)-N(6))-methyltransferase	QEN71_23890	paras_005314	protein-codi	NZ_CP1252	chromosom	5321005	5321850	+	846	0	33	9374	31	9082	
QEN71_RS23900	ABC transporter substrate-binding protein	QEN71_23895	paras_005315	protein-codi	NZ_CP1252	chromosom	5321860	5322894	-	1035	0	29	8908	20	5663	
QEN71_RS23905	hypothetical protein	QEN71_23900	paras_005316	protein-codi	NZ_CP1252	chromosom	5323065	5323415	-	351	0	14	6289	9	5246	
QEN71_RS23910	DUF3563 family protein	QEN71_23905	paras_005317	protein-codi	NZ_CP1252	chromosom	5323616	5323762	-	147	0	8	1374	4	503	
QEN71_RS23915	Cu(I)-responsive transcriptional regulator	QEN71_23910	paras_005318	protein-codi	NZ_CP1252	chromosom	5324024	5324470	-	447	0	8	1737	6	1359	
QEN71_RS23920	heavy-metal-associated domain-containing protein	QEN71_23915	paras_005319	protein-codi	NZ_CP1252	chromosom	5324605	5324814	+	210	0	4	1003	1	7	
QEN71_RS23925	SDR family oxidoreductase	QEN71_23920	paras_005320	protein-codi	NZ_CP1252	chromosom	5324829	5325743	+	915	0	20	4423	17	4181	
QEN71_RS23930	error-prone DNA polymerase	QEN71_23925	paras_005321	protein-codi	NZ_CP1252	chromosom	5325758	5328907	-	3150	0	84	16892	69	14019	
QEN71_RS23935	DNA polymerase Y family protein	QEN71_23930	paras_005322	protein-codi	NZ_CP1252	chromosom	5328914	5330383	-	1399	0	14	4022	8	3282	
QEN71_RS23940	translesion DNA synthesis-associated protein ImuA	QEN71_23935	paras_005323	protein-codi	NZ_CP1252	chromosom	5330313	5331020	-	637	0	10	1309	8	1162	
QEN71_RS23945	5S ribosomal RNA	QEN71_23940		rRNA	NZ_CP1252	chromosom	5331490	5331603	-	114	225.0	2	620	1	413	

QEN71_RS23950	23S ribosomal RNA	QEN71_23945		rRNA	NZ_CP1252	chromosom	5331810	5334690	-	2881	5661.0	10	885	7	813	
QEN71_RS23955	tRNA-Ala	QEN71_23950		tRNA	NZ_CP1252	chromosom	5334996	5335071	-	76	150.0	1	14	1	14	
QEN71_RS23960	tRNA-Ile	QEN71_23955		tRNA	NZ_CP1252	chromosom	5335131	5335207	-	77	152.0	2	22	1	18	
QEN71_RS23965	16S ribosomal RNA	QEN71_23960		rRNA	NZ_CP1252	chromosom	5335272	5336802	-	1531	3015.0	16	337	15	334	
QEN71_RS23970	AI-2E family transporter	QEN71_23965	paras_005329	protein-codi	NZ_CP1252	chromosom	5337263	5338384	-	1122	0	36	24257	34	23451	
QEN71_RS23975	DUF2863 family protein	QEN71_23970	paras_005330	protein-codi	NZ_CP1252	chromosom	5338603	5339817	+	1215	0	39	14549	34	13701	
QEN71_RS23980	response regulator transcription factor	QEN71_23975	paras_005331	protein-codi	NZ_CP1252	chromosom	5340016	5340906	+	891	0	21	11954	15	10179	
QEN71_RS23985	energy-coupling factor ABC transporter permease	QEN71_23980	paras_005332	protein-codi	NZ_CP1252	chromosom	5341000	5341674	+	675	0	24	9518	9	2061	
QEN71_RS23990	hypothetical protein	QEN71_23985	paras_005333	protein-codi	NZ_CP1252	chromosom	5341885	5342526	+	642	0	5	289	4	254	
QEN71_RS23995	M14-type cytosolic carboxypeptidase	QEN71_23990	paras_005334	protein-codi	NZ_CP1252	chromosom	5342668	5343834	-	1167	0	52	14784	42	12813	
QEN71_RS24000	peroxide stress protein YaaA	QEN71_23995	paras_005335	protein-codi	NZ_CP1252	chromosom	5343864	5344646	-	783	0	25	7361	14	2269	
QEN71_RS24005	putative toxin-antitoxin system toxin component, PIN	QEN71_24000	paras_005336	protein-codi	NZ_CP1252	chromosom	5344704	5345222	+	519	0	5	202	5	202	
QEN71_RS24010	pyridoxal phosphate-dependent aminotransferase	QEN71_24005	paras_005337	protein-codi	NZ_CP1252	chromosom	5345297	5346487	+	1191	0	44	13850	40	12850	
QEN71_RS24015	glutathione S-transferase	QEN71_24010	paras_005338	protein-codi	NZ_CP1252	chromosom	5346594	5347241	-	648	0	21	5381	12	1627	
QEN71_RS24020	3-hydroxyacyl-CoA dehydrogenase	QEN71_24015	paras_005339	protein-codi	NZ_CP1252	chromosom	5347368	5348942	+	1574	0	26	6830	15	4759	
QEN71_RS24025	enoyl-CoA hydratase	QEN71_24020	paras_005340	protein-codi	NZ_CP1252	chromosom	5348942	5349730	+	784	0	9	3318	7	2347	
QEN71_RS24030	histidine phosphatase family protein	QEN71_24025	paras_005341	protein-codi	NZ_CP1252	chromosom	5349727	5350449	+	719	0	16	4434	3	304	
QEN71_RS24035	phosphotransferase	QEN71_24030	paras_005342	protein-codi	NZ_CP1252	chromosom	5350482	5351588	+	1107	0	35	10578	32	10013	
QEN71_RS24040	acyl-CoA dehydrogenase family protein	QEN71_24035	paras_005343	protein-codi	NZ_CP1252	chromosom	5351647	5352876	+	1230	0	31	13021	15	4776	
QEN71_RS24045	glutathione binding-like protein	QEN71_24040	paras_005344	protein-codi	NZ_CP1252	chromosom	5353585	5354286	+	702	0	28	6408	22	4527	
QEN71_RS24050	MaoC family dehydratase	QEN71_24045	paras_005345	protein-codi	NZ_CP1252	chromosom	5354311	5354775	-	461	0	9	1478	3	623	
QEN71_RS24055	MaoC family dehydratase	QEN71_24050	paras_005346	protein-codi	NZ_CP1252	chromosom	5354772	5355230	-	455	0	8	4369	0	0	
QEN71_RS24060	acyl-CoA dehydrogenase	QEN71_24055	paras_005347	protein-codi	NZ_CP1252	chromosom	5355263	5356390	-	1128	0	33	7759	28	6970	
QEN71_RS24065	acyl-CoA dehydrogenase family protein	QEN71_24060	paras_005348	protein-codi	NZ_CP1252	chromosom	5356401	5357597	-	1197	0	32	7617	18	5204	
QEN71_RS24070	DUF1178 family protein	QEN71_24065	paras_005349	protein-codi	NZ_CP1252	chromosom	5357653	5358096	-	444	0	8	3499	3	1215	
QEN71_RS24075	NUDIX hydrolase	QEN71_24070	paras_005350	protein-codi	NZ_CP1252	chromosom	5358343	5358933	-	591	0	19	9429	18	9208	
QEN71_RS24080	DUF2818 family protein	QEN71_24075	paras_005351	protein-codi	NZ_CP1252	chromosom	5358975	5359271	-	296	0	7	255	4	173	
QEN71_RS24085	NADH-quinone oxidoreductase subunit NuoN	QEN71_24080	paras_005352	protein-codi	NZ_CP1252	chromosom	5359271	5360749	-	1478	0	12	1671	6	40	
QEN71_RS24090	NADH-quinone oxidoreductase subunit M	QEN71_24085	paras_005353	protein-codi	NZ_CP1252	chromosom	5360775	5362268	-	1494	0	35	3925	27	2891	
QEN71_RS24095	NADH-quinone oxidoreductase subunit L	QEN71_24090	paras_005354	protein-codi	NZ_CP1252	chromosom	5362291	5364360	-	2070	0	8	385	1	3	TRUE
QEN71_RS24100	NADH-quinone oxidoreductase subunit NuoK	QEN71_24095	paras_005355	protein-codi	NZ_CP1252	chromosom	5364379	5364684	-	306	0	2	52	0	0	TRUE
QEN71_RS24105	NADH-quinone oxidoreductase subunit J	QEN71_24100	paras_005356	protein-codi	NZ_CP1252	chromosom	5364703	5365371	-	669	0	2	14	2	14	
QEN71_RS24110	NADH-quinone oxidoreductase subunit NuoI	QEN71_24105	paras_005357	protein-codi	NZ_CP1252	chromosom	5365546	5366034	-	489	0	1	125	0	0	TRUE
QEN71_RS24115	NADH-quinone oxidoreductase subunit NuoH	QEN71_24110	paras_005358	protein-codi	NZ_CP1252	chromosom	5366049	5367113	-	1065	0	7	236	4	17	
QEN71_RS24120	NADH-quinone oxidoreductase subunit NuoG	QEN71_24115	paras_005359	protein-codi	NZ_CP1252	chromosom	5367114	5369447	-	2334	0	2	117	1	9	TRUE
QEN71_RS24125	NADH-quinone oxidoreductase subunit NuoF	QEN71_24120	paras_005360	protein-codi	NZ_CP1252	chromosom	5369543	5370871	-	1325	0	4	43	1	3	TRUE
QEN71_RS24130	NADH-quinone oxidoreductase subunit NuoE	QEN71_24125	paras_005361	protein-codi	NZ_CP1252	chromosom	5370868	5371353	-	482	0	1	5	1	5	TRUE
QEN71_RS24135	NADH-quinone oxidoreductase subunit D	QEN71_24130	paras_005362	protein-codi	NZ_CP1252	chromosom	5371523	5372776	-	1254	0	1	6	1	6	TRUE
QEN71_RS24140	NADH-quinone oxidoreductase subunit C	QEN71_24135	paras_005363	protein-codi	NZ_CP1252	chromosom	5372786	5373388	-	603	0	3	44	1	4	TRUE
QEN71_RS24145	NADH-quinone oxidoreductase subunit B	QEN71_24140	paras_005364	protein-codi	NZ_CP1252	chromosom	5373409	5373888	-	480	0	1	130	0	0	TRUE
QEN71_RS24150	NADH-quinone oxidoreductase subunit A	QEN71_24145	paras_005365	protein-codi	NZ_CP1252	chromosom	5373951	5374310	-	360	0	4	27	3	15	
QEN71_RS24155	tRNA-Leu	QEN71_24150		tRNA	NZ_CP1252	chromosom	5374483	5374567	-	85	0	0	0	0	0	
QEN71_RS24160	preprotein translocase subunit SecG	QEN71_24155	paras_005367	protein-codi	NZ_CP1252	chromosom	5374680	5375072	-	393	0	4	1245	4	1245	
QEN71_RS24165	triose-phosphate isomerase	QEN71_24160	paras_005368	protein-codi	NZ_CP1252	chromosom	5375160	5375939	-	780	0	10	1376	9	1319	
QEN71_RS24170	NAD(P)H-quinone oxidoreductase	QEN71_24165	paras_005369	protein-codi	NZ_CP1252	chromosom	5376094	5377110	-	1017	0	18	6850	15	5462	
QEN71_RS24175	polyribonucleotide nucleotidyltransferase	QEN71_24170	paras_005370	protein-codi	NZ_CP1252	chromosom	5377238	5379376	-	2139	0	5	497	2	306	TRUE
QEN71_RS24180	30S ribosomal protein S15	QEN71_24175	paras_005371	protein-codi	NZ_CP1252	chromosom	5379708	5379977	-	270	0	2	1170	0	0	
QEN71_RS24185	branched-chain amino acid ABC transporter substra	QEN71_24180	paras_005372	protein-codi	NZ_CP1252	chromosom	5380137	5381408	-	1272	0	33	12359	32	12304	
QEN71_RS24190	hypothetical protein	QEN71_24185	paras_005373	protein-codi	NZ_CP1252	chromosom	5381591	5382124	+	534	0	20	7892	20	7892	
QEN71_RS24195	2-isopropylmalate synthase	QEN71_24190	paras_005374	protein-codi	NZ_CP1252	chromosom	5382246	5383796	-	1551	0	25	8553	15	5272	
QEN71_RS24200	IS481 family transposase	QEN71_24195	paras_005375	protein-codi	NZ_CP1252	chromosom	5384237	5385376	-	1140	2266.0	17	555	14	505	
QEN71_RS24205	CDP-diacylglycerol--serine O-phosphatidyltransfera	QEN71_24200	paras_005376	protein-codi	NZ_CP1252	chromosom	5385950	5386822	-	873	0	2	25	1	4	TRUE
QEN71_RS24210	phosphatidylserine decarboxylase	QEN71_24205	paras_005377	protein-codi	NZ_CP1252	chromosom	5386843	5387481	-	639	0	0	0	0	0	TRUE
QEN71_RS24215	ketol-acid reductoisomerase	QEN71_24210	paras_005378	protein-codi	NZ_CP1252	chromosom	5387606	5388622	-	1017	0	31	6319	22	4874	
QEN71_RS24220	acetolactate synthase small subunit	QEN71_24215	paras_005379	protein-codi	NZ_CP1252	chromosom	5388722	5389213	-	492	0	6	740	6	740	
QEN71_RS24225	acetolactate synthase 3 catalytic subunit	QEN71_24220	paras_005380	protein-codi	NZ_CP1252	chromosom	5389325	5391088	-	1764	0	60	24624	39	15196	
QEN71_RS24230	RNA polymerase sigma factor	QEN71_24225	paras_005381	protein-codi	NZ_CP1252	chromosom	5391497	5392060	+	560	0	17	6149	16	5679	
QEN71_RS24235	DUF3619 family protein	QEN71_24230	paras_005382	protein-codi	NZ_CP1252	chromosom	5392057	5392488	+	428	0	7	593	5	327	
QEN71_RS24240	DUF3106 domain-containing protein	QEN71_24235	paras_005383	protein-codi	NZ_CP1252	chromosom	5392506	5393264	+	759	0	11	2151	4	983	
QEN71_RS24245	RDD family protein	QEN71_24240	paras_005384	protein-codi	NZ_CP1252	chromosom	5393349	5393855	+	507	0	20	4032	16	3727	
QEN71_RS24250	ABC transporter permease	QEN71_24245	paras_005385	protein-codi	NZ_CP1252	chromosom	5394487	5395791	-	1305	0	46	10000	31	6490	
QEN71_RS24255	sugar ABC transporter ATP-binding protein	QEN71_24250	paras_005386	protein-codi	NZ_CP1252	chromosom	5395811	5397376	-	1566	0	22	2405	22	2405	

QEN71_RS24260	substrate-binding domain-containing protein	QEN71_24255	paras_005387	protein-codi	NZ_CP1252	chromosom	5397457	5398413	-	957	0	15	1272	13	1144	
QEN71_RS24265	LacI family DNA-binding transcriptional regulator	QEN71_24260	paras_005388	protein-codi	NZ_CP1252	chromosom	5398534	5399580	-	1047	0	14	2746	12	2484	
QEN71_RS24270	UDP-2,3-diacylglucosamine diphosphatase	QEN71_24265	paras_005389	protein-codi	NZ_CP1252	chromosom	5399861	5400802	+	942	0	22	3490	18	2512	
QEN71_RS24275	glycosyltransferase family 1 protein	QEN71_24270	paras_005390	protein-codi	NZ_CP1252	chromosom	5400837	5401868	+	1032	0	20	1988	16	1385	
QEN71_RS24280	diacylglycerol kinase	QEN71_24275	paras_005391	protein-codi	NZ_CP1252	chromosom	5401909	5402619	+	711	0	14	2303	12	2113	
QEN71_RS24285	TetR/AcrR family transcriptional regulator	QEN71_24280	paras_005392	protein-codi	NZ_CP1252	chromosom	5403389	5404105	+	717	0	27	7932	23	6473	
QEN71_RS24290	TIGR00730 family Rossman fold protein	QEN71_24285	paras_005393	protein-codi	NZ_CP1252	chromosom	5404108	5404692	+	585	0	13	1860	6	1223	
QEN71_RS24295	SDR family oxidoreductase	QEN71_24290	paras_005394	protein-codi	NZ_CP1252	chromosom	5404732	5405478	+	747	0	18	5110	14	3986	
QEN71_RS24300	glycosyl transferase	QEN71_24295	paras_005395	protein-codi	NZ_CP1252	chromosom	5405724	5407658	+	1935	0	35	8498	33	8034	
QEN71_RS24305	DUF924 family protein	QEN71_24300	paras_005396	protein-codi	NZ_CP1252	chromosom	5407668	5408303	-	632	0	25	6434	17	4236	
QEN71_RS24310	alanyl-tRNA editing protein	QEN71_24305	paras_005397	protein-codi	NZ_CP1252	chromosom	5408300	5409031	-	728	0	6	713	4	558	
QEN71_RS24315	group II truncated hemoglobin	QEN71_24310	paras_005398	protein-codi	NZ_CP1252	chromosom	5409182	5409592	-	411	0	7	2362	5	2032	
QEN71_RS24320	ABC transporter permease	QEN71_24315	paras_005399	protein-codi	NZ_CP1252	chromosom	5409637	5412126	-	2490	0	40	13503	35	11298	
QEN71_RS24325	DUF4126 domain-containing protein	QEN71_24320	paras_005400	protein-codi	NZ_CP1252	chromosom	5412243	5412854	-	612	0	11	3877	6	947	
QEN71_RS24330	hypothetical protein	QEN71_24325	paras_005401	protein-codi	NZ_CP1252	chromosom	5412885	5413124	-	240	0	11	5484	11	5484	
QEN71_RS24335	quaternary ammonium compound efflux SMR transp	QEN71_24330	paras_005402	protein-codi	NZ_CP1252	chromosom	5413703	5414023	-	321	0	13	1479	8	807	
QEN71_RS24340	two-component system response regulator KdpE	QEN71_24335	paras_005403	protein-codi	NZ_CP1252	chromosom	5414164	5414862	-	695	0	20	4726	7	1634	
QEN71_RS24345	DUF4118 domain-containing protein	QEN71_24340	paras_005404	protein-codi	NZ_CP1252	chromosom	5414859	5417714	-	2852	0	47	8920	32	5292	
QEN71_RS24350	potassium-transporting ATPase subunit KdpC	QEN71_24345	paras_005405	protein-codi	NZ_CP1252	chromosom	5418418	5418999	-	582	0	23	2629	19	2538	
QEN71_RS24355	potassium-transporting ATPase subunit KdpB	QEN71_24350	paras_005406	protein-codi	NZ_CP1252	chromosom	5419041	5421125	-	2085	0	13	2252	8	1375	
QEN71_RS24360	potassium-transporting ATPase subunit KdpA	QEN71_24355	paras_005407	protein-codi	NZ_CP1252	chromosom	5421169	5422974	-	1802	0	33	5977	25	3914	
QEN71_RS24365	K(+)-transporting ATPase subunit F	QEN71_24360	paras_005408	protein-codi	NZ_CP1252	chromosom	5422971	5423063	-	85	0	4	324	4	324	
QEN71_RS24370	potassium ABC transporter ATPase	QEN71_24365		protein-codi	NZ_CP1252	chromosom	5423060	5423173	-	110	0	4	273	4	273	
QEN71_RS24375	quinone oxidoreductase	QEN71_24370	paras_005409	protein-codi	NZ_CP1252	chromosom	5423334	5424308	-	975	0	38	8181	37	8146	
QEN71_RS24380	methylglyoxal synthase	QEN71_24375	paras_005410	protein-codi	NZ_CP1252	chromosom	5424372	5424761	-	390	0	5	605	5	605	
QEN71_RS24385	SDR family oxidoreductase	QEN71_24380	paras_005411	protein-codi	NZ_CP1252	chromosom	5424839	5425618	-	780	0	9	2619	2	32	
QEN71_RS24390	uracil phosphoribosyltransferase	QEN71_24385	paras_005412	protein-codi	NZ_CP1252	chromosom	5425863	5426513	+	651	0	23	6529	15	3004	
QEN71_RS24395	YebC/PmpR family DNA-binding transcriptional regu	QEN71_24390	paras_005413	protein-codi	NZ_CP1252	chromosom	5427493	5428221	+	729	0	17	3090	9	1247	
QEN71_RS24400	phosphoribosylamine--glycine ligase	QEN71_24395	paras_005414	protein-codi	NZ_CP1252	chromosom	5428523	5429800	+	1278	0	20	2426	11	1803	
QEN71_RS24405	oxygen-dependent coproporphyrinogen oxidase	QEN71_24400	paras_005415	protein-codi	NZ_CP1252	chromosom	5430065	5430997	+	933	0	0	0	0	0	TRUE
QEN71_RS24410	nicotinate-nucleotide adenylyltransferase	QEN71_24405	paras_005416	protein-codi	NZ_CP1252	chromosom	5431049	5431768	+	720	0	3	74	1	4	TRUE
QEN71_RS24415	ribosome silencing factor	QEN71_24410	paras_005417	protein-codi	NZ_CP1252	chromosom	5431771	5432229	+	459	0	12	6634	7	3420	
QEN71_RS24420	23S rRNA (pseudouridine(1915)-N(3))-methyltransf	QEN71_24415	paras_005418	protein-codi	NZ_CP1252	chromosom	5432300	5432770	+	471	0	13	2911	6	1034	
QEN71_RS24425	Maf family protein	QEN71_24420	paras_005419	protein-codi	NZ_CP1252	chromosom	5432932	5433558	+	627	0	23	5950	16	3238	
QEN71_RS24430	ribonuclease G	QEN71_24425	paras_005420	protein-codi	NZ_CP1252	chromosom	5433568	5435037	+	1470	0	38	5097	26	3139	
QEN71_RS24435	hypothetical protein	QEN71_24430	paras_005421	protein-codi	NZ_CP1252	chromosom	5435106	5435294	+	189	0	13	2150	10	1274	
QEN71_RS24440	hypothetical protein	QEN71_24435	paras_005422	protein-codi	NZ_CP1252	chromosom	5435481	5435642	+	162	0	2	366	2	366	
QEN71_RS24445	hypothetical protein	QEN71_24440	paras_005423	protein-codi	NZ_CP1252	chromosom	5435816	5436112	+	297	0	11	5212	11	5212	
QEN71_RS24450	O-antigen ligase family protein	QEN71_24445	paras_005424	protein-codi	NZ_CP1252	chromosom	5436140	5437414	-	1275	0	37	8443	33	8292	
QEN71_RS24455	glycosyltransferase family 4 protein	QEN71_24450	paras_005425	protein-codi	NZ_CP1252	chromosom	5437652	5438656	-	1005	0	37	13590	28	11257	
QEN71_RS24460	lipid A export permease/ATP-binding protein MsbA	QEN71_24455	paras_005426	protein-codi	NZ_CP1252	chromosom	5438742	5440535	-	1794	0	0	0	0	0	TRUE
QEN71_RS24465	glycosyltransferase family 2 protein	QEN71_24460	paras_005427	protein-codi	NZ_CP1252	chromosom	5440877	5441644	+	768	0	12	2679	8	1611	
QEN71_RS24470	DNA polymerase III subunit alpha	QEN71_24465	paras_005428	protein-codi	NZ_CP1252	chromosom	5441691	5445251	-	3561	0	8	1034	4	22	TRUE
QEN71_RS24475	sulfurtransferase	QEN71_24470	paras_005429	protein-codi	NZ_CP1252	chromosom	5445535	5446410	+	876	0	21	8017	13	6878	
QEN71_RS24480	tRNA glutamyl-Q(34) synthetase GluQRS	QEN71_24475	paras_005430	protein-codi	NZ_CP1252	chromosom	5446480	5447364	+	885	0	22	8099	14	6288	
QEN71_RS24485	DEAD/DEAH box helicase	QEN71_24480	paras_005431	protein-codi	NZ_CP1252	chromosom	5447424	5448875	-	1452	0	19	3233	10	2217	
QEN71_RS24490	aldehyde dehydrogenase family protein	QEN71_24485	paras_005432	protein-codi	NZ_CP1252	chromosom	5449306	5450742	-	1437	0	30	7314	19	5361	
QEN71_RS24495	acetolactate synthase large subunit	QEN71_24490	paras_005433	protein-codi	NZ_CP1252	chromosom	5450822	5452474	-	1653	0	34	10286	28	9767	
QEN71_RS24500	MliC family protein	QEN71_24495	paras_005434	protein-codi	NZ_CP1252	chromosom	5452690	5453061	-	372	0	21	11408	19	11336	
QEN71_RS24505	hypothetical protein	QEN71_24500	paras_005435	protein-codi	NZ_CP1252	chromosom	5453130	5453333	-	204	0	2	219	0	0	
QEN71_RS24510	formate-dependent phosphoribosylglycinamide form	QEN71_24505	paras_005436	protein-codi	NZ_CP1252	chromosom	5453375	5454589	-	1215	0	18	7732	15	6271	
QEN71_RS24515	META domain-containing protein	QEN71_24510	paras_005437	protein-codi	NZ_CP1252	chromosom	5454860	5455453	+	594	0	11	4320	9	3897	
QEN71_RS24520	DNA translocase FtsK			protein-codi	NZ_CP1252	chromosom	5455569	5459429	+	3861	0	51	8932	41	6627	
QEN71_RS24525	lactonase family protein	QEN71_24520	paras_005439	protein-codi	NZ_CP1252	chromosom	5459545	5460702	-	1158	0	57	27371	43	20788	
QEN71_RS24530	DUF3096 domain-containing protein	QEN71_24525	paras_005440	protein-codi	NZ_CP1252	chromosom	5461125	5461280	+	156	0	6	4905	6	4905	
QEN71_RS24535	hypothetical protein	QEN71_24530	paras_005441	protein-codi	NZ_CP1252	chromosom	5461484	5461729	-	246	0	8	2621	8	2621	
QEN71_RS24540	glycoside hydrolase family 15 protein	partial;pseudo;QEN71_24535		protein-codi	NZ_CP1252	chromosom	5461974	5464097	+	2124	0	40	13401	33	10138	
QEN71_RS24545	polyhydroxyalkanoate depolymerase	QEN71_24540	paras_005443	protein-codi	NZ_CP1252	chromosom	5464319	5465599	+	1281	0	53	17426	41	14654	
QEN71_RS24550	TetR family transcriptional regulator	QEN71_24545	paras_005444	protein-codi	NZ_CP1252	chromosom	5465756	5466388	-	633	0	33	11742	29	11599	
QEN71_RS24555	electron transport complex subunit RsxB	QEN71_24550	paras_005445	protein-codi	NZ_CP1252	chromosom	5466797	5467672	+	876	0	5	1512	4	1456	
QEN71_RS24560	endonuclease III	QEN71_24555	paras_005446	protein-codi	NZ_CP1252	chromosom	5467682	5468326	+	645	0	14	2909	13	2732	
QEN71_RS24565	DUF1841 family protein	QEN71_24560	paras_005447	protein-codi	NZ_CP1252	chromosom	5468453	5468887	+	435	0	6	1700	4	1105	

QEN71_RS24570	cytochrome c	QEN71_24565	paras_005448	protein-codi	NZ_CP1252	chromosom	5468983	5469366	-	384	0	22	10401	17	8134	
QEN71_RS24575	cytochrome c	QEN71_24570	paras_005449	protein-codi	NZ_CP1252	chromosom	5469477	5469857	-	381	0	20	6591	18	5732	
QEN71_RS24580	MoxR family ATPase	QEN71_24575	paras_005450	protein-codi	NZ_CP1252	chromosom	5470231	5471073	+	843	0	19	10782	15	8515	
QEN71_RS24585	VWA domain-containing protein	QEN71_24580	paras_005451	protein-codi	NZ_CP1252	chromosom	5471099	5472274	+	1176	0	46	11422	28	5808	
QEN71_RS24590	benzoate/H(+) symporter BenE family transporter	QEN71_24585	paras_005452	protein-codi	NZ_CP1252	chromosom	5472397	5473608	+	1212	0	18	8299	10	4811	
QEN71_RS24595	transaldolase	QEN71_24590	paras_005453	protein-codi	NZ_CP1252	chromosom	5473859	5474812	+	954	0	26	11802	15	6640	
QEN71_RS24600	VOC family protein	QEN71_24595	paras_005454	protein-codi	NZ_CP1252	chromosom	5475012	5475446	+	435	0	5	1815	2	195	
QEN71_RS24605	FUSC family protein	QEN71_24600	paras_005455	protein-codi	NZ_CP1252	chromosom	5475449	5476627	-	1179	0	28	7965	28	7965	
QEN71_RS24610	sodium:solute symporter	QEN71_24605	paras_005456	protein-codi	NZ_CP1252	chromosom	5476758	5478308	-	1547	0	49	13449	47	13083	
QEN71_RS24615	DUF3311 domain-containing protein	QEN71_24610	paras_005457	protein-codi	NZ_CP1252	chromosom	5478305	5478532	-	224	0	11	1868	11	1868	
QEN71_RS24620	spermidine synthase	QEN71_24615	paras_005458	protein-codi	NZ_CP1252	chromosom	5478867	5479784	-	914	0	11	1206	9	1113	
QEN71_RS24625	DNA-deoxyinosine glycosylase	QEN71_24620	paras_005459	protein-codi	NZ_CP1252	chromosom	5479781	5480254	-	469	0	17	10096	12	3865	
QEN71_RS24630	chorismate lyase	QEN71_24625	paras_005460	protein-codi	NZ_CP1252	chromosom	5480254	5480946	-	692	0	15	3456	13	3374	
QEN71_RS24635	hypothetical protein	QEN71_24630	paras_005461	protein-codi	NZ_CP1252	chromosom	5481127	5481393	+	267	0	5	1092	5	1092	
QEN71_RS24640	molecular chaperone HtpG	QEN71_24635	paras_005462	protein-codi	NZ_CP1252	chromosom	5481485	5483377	-	1893	0	45	12504	39	11656	
QEN71_RS24645	PLP-dependent aminotransferase family protein	QEN71_24640	paras_005463	protein-codi	NZ_CP1252	chromosom	5483513	5484982	-	1470	0	35	8235	29	7276	
QEN71_RS24650	VOC family protein	QEN71_24645	paras_005464	protein-codi	NZ_CP1252	chromosom	5485399	5486079	+	681	0	12	3068	9	2698	
QEN71_RS24655	DMT family transporter	QEN71_24650	paras_005465	protein-codi	NZ_CP1252	chromosom	5486110	5487015	+	906	0	11	2390	8	2085	
QEN71_RS24660	PLP-dependent aminotransferase family protein	QEN71_24655	paras_005466	protein-codi	NZ_CP1252	chromosom	5487080	5488276	+	1197	0	26	5517	26	5517	
QEN71_RS24665	RidA family protein	QEN71_24660	paras_005467	protein-codi	NZ_CP1252	chromosom	5488392	5488850	+	459	0	13	6585	5	1476	
QEN71_RS24670	PhzF family phenazine biosynthesis protein	QEN71_24665	paras_005468	protein-codi	NZ_CP1252	chromosom	5488993	5489883	+	891	0	27	6819	21	5097	
QEN71_RS24675	hypothetical protein	QEN71_24670	paras_005469	protein-codi	NZ_CP1252	chromosom	5489897	5490136	+	240	0	4	1401	4	1401	
QEN71_RS24680	EAL domain-containing protein	QEN71_24675	paras_005470	protein-codi	NZ_CP1252	chromosom	5490390	5492765	+	2376	0	53	12971	43	10456	
QEN71_RS24685	chromate transporter	QEN71_24680	paras_005471	protein-codi	NZ_CP1252	chromosom	5492944	5494161	+	1218	0	35	6947	24	5070	
QEN71_RS24690	CopD family protein	QEN71_24685	paras_005472	protein-codi	NZ_CP1252	chromosom	5494248	5494706	-	459	0	5	1776	4	1529	
QEN71_RS24695	DNA topoisomerase IV subunit A	QEN71_24690	paras_005473	protein-codi	NZ_CP1252	chromosom	5494897	5497218	-	2322	0	1	13	1	13	TRUE
QEN71_RS24700	DNA topoisomerase IV subunit B	QEN71_24695	paras_005474	protein-codi	NZ_CP1252	chromosom	5497277	5499262	-	1986	0	2	20	0	0	TRUE
QEN71_RS24705	nucleotidyltransferase family protein	QEN71_24700	paras_005475	protein-codi	NZ_CP1252	chromosom	5499624	5499914	+	287	0	5	642	5	642	
QEN71_RS24710	DUF86 domain-containing protein	QEN71_24705	paras_005476	protein-codi	NZ_CP1252	chromosom	5499911	5500270	+	356	0	9	4258	7	4126	
QEN71_RS24715	ATP-binding cassette domain-containing protein	QEN71_24710	paras_005477	protein-codi	NZ_CP1252	chromosom	5500292	5502217	-	1926	0	37	10343	29	8013	
QEN71_RS24720	DUF4399 domain-containing protein	QEN71_24715	paras_005478	protein-codi	NZ_CP1252	chromosom	5502424	5502825	-	402	0	2	70	2	70	
QEN71_RS24725	rubredoxin	QEN71_24720	paras_005479	protein-codi	NZ_CP1252	chromosom	5503102	5503284	+	183	0	4	2521	3	1578	
QEN71_RS24730	class I SAM-dependent methyltransferase	QEN71_24725	paras_005480	protein-codi	NZ_CP1252	chromosom	5503397	5504137	-	741	0	12	3680	5	1816	
QEN71_RS24735	tRNA-Arg	QEN71_24730		tRNA	NZ_CP1252	chromosom	5504260	5504334	+	75	0	8	376	8	376	
QEN71_RS24740	hypothetical protein	QEN71_24735	paras_005482	protein-codi	NZ_CP1252	chromosom	5504790	5505062	+	273	0	4	990	2	903	
QEN71_RS24745	hypothetical protein	QEN71_24740	paras_005483	protein-codi	NZ_CP1252	chromosom	5505463	5505786	+	324	0	4	2298	1	206	
QEN71_RS24750	DNA (cytosine-5-)-methyltransferase	QEN71_24745	paras_005484	protein-codi	NZ_CP1252	chromosom	5506090	5507391	+	1301	0	21	8150	21	8150	
QEN71_RS24755	very short patch repair endonuclease	QEN71_24750	paras_005485	protein-codi	NZ_CP1252	chromosom	5507391	5507837	+	446	0	2	262	2	262	
QEN71_RS24760	YbhB/YbcL family Raf kinase inhibitor-like protein	QEN71_24755	paras_005486	protein-codi	NZ_CP1252	chromosom	5508029	5508538	+	510	0	12	4971	6	2774	
QEN71_RS24765	MFS transporter	QEN71_24760	paras_005487	protein-codi	NZ_CP1252	chromosom	5508675	5510105	+	1431	0	17	4179	13	2747	
QEN71_RS24770	hypothetical protein	QEN71_24765	paras_005488	protein-codi	NZ_CP1252	chromosom	5510238	5510825	+	588	0	2	963	2	963	
QEN71_RS24775	alpha,alpha-trehalose-phosphate synthase (UDP-fo	QEN71_24770	paras_005489	protein-codi	NZ_CP1252	chromosom	5510917	5512338	-	1422	0	50	16570	47	15853	
QEN71_RS24780	hypothetical protein	QEN71_24775	paras_005490	protein-codi	NZ_CP1252	chromosom	5512539	5512751	-	213	0	2	885	2	885	
QEN71_RS24785	hypothetical protein	QEN71_24780	paras_005491	protein-codi	NZ_CP1252	chromosom	5512925	5513155	-	231	0	6	2006	6	2006	
QEN71_RS24790	sigma-54 dependent transcriptional regulator	QEN71_24785	paras_005492	protein-codi	NZ_CP1252	chromosom	5513292	5514695	-	1404	776.0	22	13047	13	4828	
QEN71_RS24795	hypothetical protein	QEN71_24790	paras_005493	protein-codi	NZ_CP1252	chromosom	5515002	5515253	-	252	0	5	1173	2	719	
QEN71_RS24800	DUF883 family protein	QEN71_24795	paras_005494	protein-codi	NZ_CP1252	chromosom	5515332	5515649	-	318	0	3	1152	3	1152	
QEN71_RS24805	sigma-54 dependent transcriptional regulator	QEN71_24800	paras_005495	protein-codi	NZ_CP1252	chromosom	5516091	5517146	+	1056	0	22	7369	15	4801	
QEN71_RS24810	hypothetical protein	QEN71_24805	paras_005496	protein-codi	NZ_CP1252	chromosom	5517493	5518080	+	584	0	6	2242	6	2242	
QEN71_RS24815	hypothetical protein	QEN71_24810	paras_005497	protein-codi	NZ_CP1252	chromosom	5518077	5518637	+	557	0	10	2156	8	2084	
QEN71_RS24820	IS4 family transposase	QEN71_24815	paras_005498	protein-codi	NZ_CP1252	chromosom	5518699	5520045	-	1347	2662.0	26	2245	20	1652	
QEN71_RS24825	nitrate/sulfonate/bicarbonate ABC transporter ATP-l	QEN71_24820	paras_005499	protein-codi	NZ_CP1252	chromosom	5520185	5521531	-	1347	0	21	4485	16	3764	
QEN71_RS24830	ABC transporter permease subunit	QEN71_24825	paras_005500	protein-codi	NZ_CP1252	chromosom	5521554	5523308	-	1755	0	38	7627	28	6089	
QEN71_RS24835	sensor domain-containing diguanylate cyclase	QEN71_24830	paras_005501	protein-codi	NZ_CP1252	chromosom	5524041	5525720	+	1680	0	37	14884	24	10360	
QEN71_RS24840	hypothetical protein	QEN71_24835	paras_005502	protein-codi	NZ_CP1252	chromosom	5525870	5526136	+	267	0	13	3952	11	3531	
QEN71_RS24845	ATP-binding protein	QEN71_24840	paras_005503	protein-codi	NZ_CP1252	chromosom	5526243	5527574	-	1315	0	20	5661	17	4732	
QEN71_RS24850	response regulator	QEN71_24845	paras_005504	protein-codi	NZ_CP1252	chromosom	5527558	5528283	-	709	0	11	1615	5	441	
QEN71_RS24855	periplasmic heavy metal sensor	QEN71_24850	paras_005505	protein-codi	NZ_CP1252	chromosom	5528508	5529059	+	552	0	3	638	2	449	
QEN71_RS24860	pirin family protein	QEN71_24855	paras_005506	protein-codi	NZ_CP1252	chromosom	5529143	5529850	-	708	0	14	7708	12	7651	
QEN71_RS24865	ABC transporter substrate-binding protein	QEN71_24860	paras_005507	protein-codi	NZ_CP1252	chromosom	5530303	5531079	+	777	0	19	4592	10	1449	
QEN71_RS24870	histidine ABC transporter permease HisQ	QEN71_24865	paras_005508	protein-codi	NZ_CP1252	chromosom	5531380	5532069	+	686	0	24	5706	15	4609	
QEN71_RS24875	ABC transporter permease	QEN71_24870	paras_005509	protein-codi	NZ_CP1252	chromosom	5532066	5532779	+	710	0	43	11436	32	8795	

QEN71_RS24880	ATP-binding cassette domain-containing protein	QEN71_24875	paras_005510	protein-codi	NZ_CP1252	chromosom	5532810	5533595	+	786	0	7	1054	6	1046	
QEN71_RS24885	porin	QEN71_24880	paras_005511	protein-codi	NZ_CP1252	chromosom	5534176	5535306	+	1131	0	45	8677	29	6153	
QEN71_RS24890	P-II family nitrogen regulator	QEN71_24885	paras_005512	protein-codi	NZ_CP1252	chromosom	5535533	5535871	-	339	0	9	2248	6	2016	
QEN71_RS24895	NAD(+) synthase	QEN71_24890	paras_005513	protein-codi	NZ_CP1252	chromosom	5536003	5538060	-	2058	0	33	585	29	485	
QEN71_RS24900	GNAT family N-acetyltransferase	QEN71_24895	paras_005514	protein-codi	NZ_CP1252	chromosom	5538378	5539550	+	1173	0	31	5718	27	5106	
QEN71_RS24905	GIY-YIG nuclease family protein	QEN71_24900	paras_005515	protein-codi	NZ_CP1252	chromosom	5539569	5539886	+	318	0	15	3427	11	3122	
QEN71_RS24910	H-NS family nucleoid-associated regulatory protein	QEN71_24905	paras_005516	protein-codi	NZ_CP1252	chromosom	5540040	5540375	+	336	0	2	1036	1	576	
QEN71_RS24915	hypothetical protein	QEN71_24910	paras_005517	protein-codi	NZ_CP1252	chromosom	5540391	5541014	+	624	0	18	4103	12	3467	
QEN71_RS24920	inorganic diphosphatase	QEN71_24915	paras_005518	protein-codi	NZ_CP1252	chromosom	5541076	5541603	-	528	0	2	337	0	0	
QEN71_RS24925	aldehyde dehydrogenase family protein	QEN71_24920	paras_005519	protein-codi	NZ_CP1252	chromosom	5541904	5543343	+	1440	0	28	8575	21	5165	
QEN71_RS24930	SDR family oxidoreductase	QEN71_24925	paras_005520	protein-codi	NZ_CP1252	chromosom	5543408	5544166	+	759	0	18	3773	12	2582	
QEN71_RS24935	MFS transporter	QEN71_24930	paras_005521	protein-codi	NZ_CP1252	chromosom	5544398	5545717	+	1320	0	58	17809	34	9496	
QEN71_RS24940	heme biosynthesis protein HemY	QEN71_24935	paras_005522	protein-codi	NZ_CP1252	chromosom	5546341	5547531	-	1191	0	31	12193	21	10083	
QEN71_RS24945	fused uroporphyrinogen-III synthase HemD/membra	QEN71_24940	paras_005523	protein-codi	NZ_CP1252	chromosom	5547534	5549594	-	2060	0	23	3299	18	2526	
QEN71_RS24950	hydroxymethylbilane synthase	QEN71_24945	paras_005524	protein-codi	NZ_CP1252	chromosom	5549594	5550577	-	983	0	0	0	0	0	TRUE
QEN71_RS24955	phosphoenolpyruvate carboxylase	QEN71_24950	paras_005525	protein-codi	NZ_CP1252	chromosom	5550943	5554044	+	3102	0	57	15840	42	12937	
QEN71_RS24960	class I SAM-dependent methyltransferase	QEN71_24955	paras_005526	protein-codi	NZ_CP1252	chromosom	5554677	5555429	-	753	0	29	4896	20	2911	
QEN71_RS24965	helix-turn-helix transcriptional regulator	QEN71_24960	paras_005527	protein-codi	NZ_CP1252	chromosom	5555583	5556395	+	813	0	14	3793	12	3652	
QEN71_RS24970	argininosuccinate lyase	QEN71_24965	paras_005528	protein-codi	NZ_CP1252	chromosom	5556523	5557929	+	1407	0	15	2093	7	1046	
QEN71_RS24975	bestrophin family ion channel	QEN71_24970	paras_005529	protein-codi	NZ_CP1252	chromosom	5558104	5559024	+	921	0	21	4902	17	3740	
QEN71_RS24980	DUF799 domain-containing protein	QEN71_24975	paras_005530	protein-codi	NZ_CP1252	chromosom	5559084	5559758	-	675	0	27	7181	23	6026	
QEN71_RS24985	DUF4810 domain-containing protein	QEN71_24980	paras_005531	protein-codi	NZ_CP1252	chromosom	5559761	5560126	-	366	0	13	3900	9	1815	
QEN71_RS24990	CsgG/HfaB family protein	QEN71_24985	paras_005532	protein-codi	NZ_CP1252	chromosom	5560163	5560840	-	678	0	23	7643	19	6236	
QEN71_RS24995	lysozyme inhibitor LprI family protein	QEN71_24990	paras_005533	protein-codi	NZ_CP1252	chromosom	5561062	5561760	-	699	0	6	1427	6	1427	
QEN71_RS25000	arginine/lysine/ornithine decarboxylase	QEN71_24995	paras_005534	protein-codi	NZ_CP1252	chromosom	5561763	5564045	-	2283	0	0	0	0	0	TRUE
QEN71_RS25005	dCTP deaminase	QEN71_25000	paras_005535	protein-codi	NZ_CP1252	chromosom	5564128	5564697	-	570	0	0	0	0	0	TRUE
QEN71_RS25010	superoxide dismutase family protein	QEN71_25005	paras_005536	protein-codi	NZ_CP1252	chromosom	5565010	5565543	-	534	0	11	4978	7	3647	
QEN71_RS25015	iron-sulfur cluster carrier protein ApbC	QEN71_25010	paras_005537	protein-codi	NZ_CP1252	chromosom	5565732	5566820	-	1089	0	14	1445	9	204	
QEN71_RS25020	OmpA family protein	QEN71_25015	paras_005538	protein-codi	NZ_CP1252	chromosom	5567090	5567740	+	651	0	13	3512	8	1907	
QEN71_RS25025	methionine--tRNA ligase	QEN71_25020	paras_005539	protein-codi	NZ_CP1252	chromosom	5568295	5570475	+	2181	0	9	2203	1	4	
QEN71_RS25030	IS4 family transposase	QEN71_25025	paras_005540	protein-codi	NZ_CP1252	chromosom	5570547	5571893	-	1347	2662.0	50	31454	39	27196	
QEN71_RS25035	hypothetical protein	QEN71_25030	paras_005541	protein-codi	NZ_CP1252	chromosom	5572086	5572247	+	162	0	0	0	0	0	
QEN71_RS25040	adhesin	QEN71_25035	paras_005542	protein-codi	NZ_CP1252	chromosom	5572249	5574162	+	1914	0	44	13728	40	12093	
QEN71_RS25045	translocation/assembly module TamB domain-conta	QEN71_25040	paras_005543	protein-codi	NZ_CP1252	chromosom	5574248	5578552	-	4305	0	49	9012	35	7597	
QEN71_RS25050	autotransporter assembly complex family protein	QEN71_25045	paras_005544	protein-codi	NZ_CP1252	chromosom	5578569	5580416	-	1848	0	63	13141	57	11185	
QEN71_RS25055	DUF3460 family protein	QEN71_25050	paras_005545	protein-codi	NZ_CP1252	chromosom	5580729	5580914	+	186	0	3	749	1	286	
QEN71_RS25060	ScpA family protein	QEN71_25055	paras_005546	protein-codi	NZ_CP1252	chromosom	5580915	5581787	+	873	0	2	538	0	0	
QEN71_RS25065	pantoate--beta-alanine ligase	QEN71_25060	paras_005547	protein-codi	NZ_CP1252	chromosom	5582094	5582927	+	834	0	0	0	0	0	TRUE
QEN71_RS25070	aspartate 1-decarboxylase	QEN71_25065	paras_005548	protein-codi	NZ_CP1252	chromosom	5582946	5583332	+	387	0	7	873	5	587	
QEN71_RS25075	ParA family protein	QEN71_25070	paras_005549	protein-codi	NZ_CP1252	chromosom	5583397	5584101	-	705	0	10	1522	7	1109	
QEN71_RS25080	PaaI family thioesterase	QEN71_25075	paras_005550	protein-codi	NZ_CP1252	chromosom	5584329	5584748	+	420	0	9	1584	5	655	
QEN71_RS25085	tetratricopeptide repeat protein	QEN71_25080	paras_005551	protein-codi	NZ_CP1252	chromosom	5584882	5586552	+	1671	0	28	7764	20	6310	
QEN71_RS25090	DoxX family protein	QEN71_25085	paras_005552	protein-codi	NZ_CP1252	chromosom	5586591	5587025	-	435	0	6	2492	6	2492	
QEN71_RS25095	cobyric acid synthase	QEN71_25090	paras_005553	protein-codi	NZ_CP1252	chromosom	5587148	5588602	-	1455	0	19	5533	18	5530	
QEN71_RS25100	bifunctional adenosylcobinamide kinase/adenosylco	QEN71_25095	paras_005554	protein-codi	NZ_CP1252	chromosom	5588732	5589280	+	545	0	9	2679	9	2679	
QEN71_RS25105	adenosylcobinamide-phosphate synthase CbiB	QEN71_25100	paras_005555	protein-codi	NZ_CP1252	chromosom	5589277	5590218	+	938	0	19	2645	15	2386	
QEN71_RS25110	threonine-phosphate decarboxylase CobD	QEN71_25105	paras_005556	protein-codi	NZ_CP1252	chromosom	5590222	5591223	+	1002	0	17	2234	13	2056	
QEN71_RS25115	cobalamin-binding protein	QEN71_25110	paras_005557	protein-codi	NZ_CP1252	chromosom	5591229	5592140	+	912	0	18	5035	18	5035	
QEN71_RS25120	alpha-ribazole phosphatase	QEN71_25115	paras_005558	protein-codi	NZ_CP1252	chromosom	5592156	5592746	-	575	0	14	2424	12	2307	
QEN71_RS25125	adenosylcobinamide-GDP ribazoletransferase	QEN71_25120	paras_005559	protein-codi	NZ_CP1252	chromosom	5592731	5593480	-	734	0	36	12200	29	10812	
QEN71_RS25130	nicotinate-nucleotide--dimethylbenzimidazole phosp	QEN71_25125	paras_005560	protein-codi	NZ_CP1252	chromosom	5593567	5594619	-	1053	0	16	4352	8	1674	
QEN71_RS25135	ABC transporter ATP-binding protein	QEN71_25130		protein-codi	NZ_CP1252	chromosom	5594656	5595465	-	806	0	9	2928	9	2928	
QEN71_RS25140	iron ABC transporter permease	QEN71_25135	paras_005561	protein-codi	NZ_CP1252	chromosom	5595462	5596511	-	1046	0	4	2384	4	2384	
QEN71_RS25145	TonB-dependent receptor	QEN71_25140	paras_005562	protein-codi	NZ_CP1252	chromosom	5596518	5598473	-	1956	0	64	15510	53	12980	
QEN71_RS25150	ATPase	QEN71_25145	paras_005563	protein-codi	NZ_CP1252	chromosom	5599034	5599396	+	359	0	5	1141	4	940	
QEN71_RS25155	cell division protein ZapA	QEN71_25150	paras_005564	protein-codi	NZ_CP1252	chromosom	5599393	5599707	+	311	0	11	1357	7	941	
QEN71_RS25165	EVE domain-containing protein	QEN71_25160	paras_005566	protein-codi	NZ_CP1252	chromosom	5600047	5600517	+	471	0	11	4768	11	4768	
QEN71_RS25170	SIMPL domain-containing protein	QEN71_25165	paras_005567	protein-codi	NZ_CP1252	chromosom	5600738	5601460	+	723	0	27	8759	24	6896	
QEN71_RS25175	prolipoprotein diacylglyceryl transferase	QEN71_25170	paras_005568	protein-codi	NZ_CP1252	chromosom	5602250	5603155	-	906	0	11	2725	2	8	
QEN71_RS25180	LysR family transcriptional regulator	QEN71_25175	paras_005569	protein-codi	NZ_CP1252	chromosom	5603226	5604179	-	954	0	19	4820	15	4065	
QEN71_RS25185	dihydroxy-acid dehydratase	QEN71_25180	paras_005570	protein-codi	NZ_CP1252	chromosom	5604338	5606011	+	1674	0	38	16216	27	11729	
QEN71_RS25190	c-type cytochrome	QEN71_25185	paras_005571	protein-codi	NZ_CP1252	chromosom	5606171	5606497	+	327	0	9	1804	5	1214	

QEN71_RS25195	DUF2486 family protein	QEN71_25190	paras_005572	protein-codi	NZ_CP1252	chromosom	5606516	5607271	-	756	0	11	3129	9	2418	
QEN71_RS25200	DNA polymerase III subunit chi	QEN71_25195	paras_005573	protein-codi	NZ_CP1252	chromosom	5607365	5607781	-	417	0	13	1881	10	718	
QEN71_RS25205	leucyl aminopeptidase	QEN71_25200	paras_005574	protein-codi	NZ_CP1252	chromosom	5607828	5609339	-	1512	0	22	1684	17	1433	
QEN71_RS25210	LPS export ABC transporter permease LptF	QEN71_25205	paras_005575	protein-codi	NZ_CP1252	chromosom	5609476	5610588	+	1113	0	1	5	0	0	TRUE
QEN71_RS25215	LPS export ABC transporter permease LptG	QEN71_25210	paras_005576	protein-codi	NZ_CP1252	chromosom	5610592	5611740	+	1149	0	2	94	1	15	TRUE
QEN71_RS25220	CbiX/SirB N-terminal domain-containing protein	QEN71_25215	paras_005577	protein-codi	NZ_CP1252	chromosom	5611791	5612168	+	378	0	3	280	2	267	
QEN71_RS25225	class A beta-lactamase	QEN71_25220	paras_005578	protein-codi	NZ_CP1252	chromosom	5612751	5613644	-	894	0	26	6383	21	4488	
QEN71_RS25230	uroporphyrinogen-III C-methyltransferase	QEN71_25225	paras_005579	protein-codi	NZ_CP1252	chromosom	5613771	5614538	-	768	0	16	3713	12	2371	
QEN71_RS25235	sulfate adenylyltransferase subunit 1	QEN71_25230	paras_005580	protein-codi	NZ_CP1252	chromosom	5614567	5615883	-	1317	0	23	4673	18	4012	
QEN71_RS25240	sulfate adenylyltransferase subunit CysD	QEN71_25235	paras_005581	protein-codi	NZ_CP1252	chromosom	5615907	5616869	-	963	0	23	2922	17	2379	
QEN71_RS25245	phosphoadenylyl-sulfate reductase	QEN71_25240	paras_005582	protein-codi	NZ_CP1252	chromosom	5616977	5617717	-	737	0	4	463	4	463	
QEN71_RS25250	DUF934 domain-containing protein	QEN71_25245	paras_005583	protein-codi	NZ_CP1252	chromosom	5617714	5618259	-	542	0	14	1517	11	1413	
QEN71_RS25255	nitrite/sulfite reductase	QEN71_25250	paras_005584	protein-codi	NZ_CP1252	chromosom	5618278	5619957	-	1680	0	47	11844	28	4785	
QEN71_RS25260	CysB family HTH-type transcriptional regulator	QEN71_25255	paras_005585	protein-codi	NZ_CP1252	chromosom	5620196	5621137	+	942	0	29	10290	16	5141	
QEN71_RS25265	ABC transporter substrate-binding protein	QEN71_25260	paras_005586	protein-codi	NZ_CP1252	chromosom	5621214	5622398	+	1185	0	31	8171	23	6700	
QEN71_RS25270	hypothetical protein	QEN71_25265	paras_005587	protein-codi	NZ_CP1252	chromosom	5622439	5623827	-	1389	0	22	3739	17	2898	
QEN71_RS25275	hypothetical protein	QEN71_25270	paras_005588	protein-codi	NZ_CP1252	chromosom	5623977	5624234	+	258	0	6	1440	4	1067	
QEN71_RS25280	Gfo/Idh/MocA family oxidoreductase	QEN71_25275	paras_005589	protein-codi	NZ_CP1252	chromosom	5624250	5625395	-	1146	0	25	2773	22	2698	
QEN71_RS25285	sugar phosphate isomerase/epimerase	QEN71_25280	paras_005590	protein-codi	NZ_CP1252	chromosom	5625427	5626476	-	1050	0	19	1538	19	1538	
QEN71_RS25290	substrate-binding domain-containing protein	QEN71_25285	paras_005591	protein-codi	NZ_CP1252	chromosom	5626560	5627528	-	969	0	27	1982	20	1634	
QEN71_RS25295	ABC transporter permease	QEN71_25290	paras_005592	protein-codi	NZ_CP1252	chromosom	5628134	5629147	-	994	0	31	2947	26	2480	
QEN71_RS25300	sugar ABC transporter ATP-binding protein	QEN71_25295	paras_005593	protein-codi	NZ_CP1252	chromosom	5629128	5630615	-	1468	0	16	3432	12	2537	
QEN71_RS25305	LacI family DNA-binding transcriptional regulator	QEN71_25300	paras_005594	protein-codi	NZ_CP1252	chromosom	5630627	5631613	-	987	0	16	2830	16	2830	
QEN71_RS25310	protein-L-isoaspartate O-methyltransferase	QEN71_25305	paras_005595	protein-codi	NZ_CP1252	chromosom	5631973	5632626	+	654	0	7	424	5	177	
QEN71_RS25315	rhodanese-like domain-containing protein	QEN71_25310	paras_005596	protein-codi	NZ_CP1252	chromosom	5632658	5632981	+	324	0	4	293	2	216	
QEN71_RS25320	hypothetical protein	QEN71_25315	paras_005597	protein-codi	NZ_CP1252	chromosom	5632995	5633156	-	162	0	3	118	2	25	
QEN71_RS25325	DUF2844 domain-containing protein	QEN71_25320	paras_005598	protein-codi	NZ_CP1252	chromosom	5633186	5633641	+	452	0	7	960	7	960	
QEN71_RS25330	DUF3443 domain-containing protein	QEN71_25325	paras_005599	protein-codi	NZ_CP1252	chromosom	5633638	5635062	+	1421	0	50	9237	34	6874	
QEN71_RS25335	urea ABC transporter substrate-binding protein	QEN71_25330	paras_005600	protein-codi	NZ_CP1252	chromosom	5635488	5636789	+	1302	0	23	3137	19	2678	
QEN71_RS25340	urea ABC transporter permease subunit UrtB	QEN71_25335	paras_005601	protein-codi	NZ_CP1252	chromosom	5636881	5638500	+	1620	0	25	4054	23	3779	
QEN71_RS25345	urea ABC transporter permease subunit UrtC	QEN71_25340	paras_005602	protein-codi	NZ_CP1252	chromosom	5638502	5639695	+	1193	0	21	2444	18	2415	
QEN71_RS25350	urea ABC transporter ATP-binding protein UrtD	QEN71_25345	paras_005603	protein-codi	NZ_CP1252	chromosom	5639695	5640549	+	854	0	15	558	13	496	
QEN71_RS25355	urea ABC transporter ATP-binding subunit UrtE	QEN71_25350	paras_005604	protein-codi	NZ_CP1252	chromosom	5641143	5641835	+	693	0	30	2175	23	1955	
QEN71_RS25360	urease accessory protein UreD	QEN71_25355	paras_005605	protein-codi	NZ_CP1252	chromosom	5641871	5642752	+	882	0	9	726	6	436	
QEN71_RS25365	transporter substrate-binding domain-containing pro	QEN71_25360	paras_005606	protein-codi	NZ_CP1252	chromosom	5642961	5643764	+	804	0	9	1397	9	1397	
QEN71_RS25370	urease subunit gamma	QEN71_25365	paras_005607	protein-codi	NZ_CP1252	chromosom	5643888	5644190	+	303	0	5	733	5	733	
QEN71_RS25375	urease subunit beta	QEN71_25370	paras_005608	protein-codi	NZ_CP1252	chromosom	5644201	5644506	+	306	0	7	404	6	401	
QEN71_RS25380	urease subunit alpha	QEN71_25375	paras_005609	protein-codi	NZ_CP1252	chromosom	5644551	5646257	+	1707	0	29	3098	15	1342	
QEN71_RS25385	urease accessory protein UreE	QEN71_25380	paras_005610	protein-codi	NZ_CP1252	chromosom	5646270	5646851	+	568	0	15	2323	13	2032	
QEN71_RS25390	urease accessory UreF family protein	QEN71_25385	paras_005611	protein-codi	NZ_CP1252	chromosom	5646838	5647518	+	667	0	10	1250	4	424	
QEN71_RS25395	urease accessory protein UreG	QEN71_25390	paras_005612	protein-codi	NZ_CP1252	chromosom	5647539	5648180	+	642	0	37	6307	32	5563	
QEN71_RS25400	lipid IV(A) 3-deoxy-D-manno-octulosonic acid transf	QEN71_25395	paras_005613	protein-codi	NZ_CP1252	chromosom	5648788	5650089	-	1302	0	3	410	0	0	TRUE
QEN71_RS25405	Kdo hydroxylase family protein	QEN71_25400	paras_005614	protein-codi	NZ_CP1252	chromosom	5650178	5651062	-	885	0	6	662	5	609	
QEN71_RS25410	lipopolysaccharide heptosyltransferase I	QEN71_25405	paras_005615	protein-codi	NZ_CP1252	chromosom	5651093	5652034	-	942	0	2	10	2	10	TRUE
QEN71_RS25415	phosphomannomutase/phosphoglucomutase	QEN71_25410	paras_005616	protein-codi	NZ_CP1252	chromosom	5652837	5654231	-	1395	0	2	17	0	0	TRUE
QEN71_RS25420	oligosaccharide flippase family protein	QEN71_25415	paras_005617	protein-codi	NZ_CP1252	chromosom	5654479	5655741	+	1259	0	28	4884	21	3951	
QEN71_RS25425	glycosyltransferase	QEN71_25420	paras_005618	protein-codi	NZ_CP1252	chromosom	5655738	5656586	+	845	0	18	2043	13	1475	
QEN71_RS25430	glycosyltransferase family 4 protein	QEN71_25425	paras_005619	protein-codi	NZ_CP1252	chromosom	5656617	5657789	+	1173	0	50	8312	40	6727	
QEN71_RS25435	UDP-glucose 4-epimerase GalE	QEN71_25430	paras_005620	protein-codi	NZ_CP1252	chromosom	5657801	5658823	+	1023	0	37	7223	29	5420	
QEN71_RS25440	hypothetical protein	QEN71_25435	paras_005621	protein-codi	NZ_CP1252	chromosom	5659045	5659488	+	444	0	11	2421	10	2417	
QEN71_RS25445	acyltransferase	QEN71_25440	paras_005622	protein-codi	NZ_CP1252	chromosom	5660026	5661039	+	1014	0	41	5443	27	4315	
QEN71_RS25450	transcriptional regulator	QEN71_25445	paras_005623	protein-codi	NZ_CP1252	chromosom	5661510	5662424	+	915	0	35	4250	30	3429	
QEN71_RS25455	hypothetical protein	partial;pseudo;QEN71_25450		pseudogene	NZ_CP1252	chromosom	5662531	5663487	-	957	0	45	7348	32	4705	
QEN71_RS25460	mannose-1-phosphate guanylyltransferase/mannos	QEN71_25455	paras_005625	protein-codi	NZ_CP1252	chromosom	5663968	5665509	+	1542	0	28	4470	23	3870	
QEN71_RS25465	tetratricopeptide repeat protein	QEN71_25460	paras_005626	protein-codi	NZ_CP1252	chromosom	5666151	5667959	-	1809	0	88	10984	73	8838	
QEN71_RS25470	hypothetical protein	QEN71_25465	paras_005627	protein-codi	NZ_CP1252	chromosom	5668137	5668592	-	456	0	22	3390	19	3045	
QEN71_RS25475	hypothetical protein	QEN71_25470	paras_005628	protein-codi	NZ_CP1252	chromosom	5668847	5669332	-	486	0	17	2531	11	1219	
QEN71_RS25480	hypothetical protein	QEN71_25475	paras_005629	protein-codi	NZ_CP1252	chromosom	5669348	5669608	-	261	0	13	1742	7	652	
QEN71_RS25485	ATP-binding protein	partial;pseudo;QEN71_25480		pseudogene	NZ_CP1252	chromosom	5669641	5669835	+	195	0	1	27	1	27	
QEN71_RS25490	Imm72 family immunity protein	QEN71_25485	paras_005631	protein-codi	NZ_CP1252	chromosom	5670569	5670934	-	366	0	15	2752	12	2400	
QEN71_RS25495	IS21-like element helper ATPase IstB	QEN71_25490	paras_005632	protein-codi	NZ_CP1252	chromosom	5671076	5671864	-	789	1561.0	14	17654	13	14471	
QEN71_RS25500	IS21 family transposase	QEN71_25495	paras_005633	protein-codi	NZ_CP1252	chromosom	5671871	5673394	-	1524	3027.0	30	30918	26	23800	

QEN71_RS25505	Imm71 family immunity protein	QEN71_25500	paras_005634	protein-codi	NZ_CP1252	chromosom	5673651	5674328	-	678	0	32	6205	27	4992	
QEN71_RS25510	DUF3396 domain-containing protein	QEN71_25505	paras_005635	protein-codi	NZ_CP1252	chromosom	5674427	5675761	-	1335	0	61	13115	52	11300	
QEN71_RS25515	VRR-NUC domain-containing protein	QEN71_25510	paras_005636	protein-codi	NZ_CP1252	chromosom	5675813	5677063	-	1251	0	44	9303	35	6671	
QEN71_RS25520	type VI secretion system Vgr family protein	QEN71_25515	paras_005637	protein-codi	NZ_CP1252	chromosom	5677085	5679853	-	2769	0	111	37588	86	32165	
QEN71_RS25525	integrase domain-containing protein	QEN71_25520	paras_005638	protein-codi	NZ_CP1252	chromosom	5681465	5682484	+	1020	0	14	2844	11	2761	
QEN71_RS25530	hypothetical protein	QEN71_25525	paras_005639	protein-codi	NZ_CP1252	chromosom	5682579	5683034	+	456	0	8	788	8	788	
QEN71_RS25535	hypothetical protein	QEN71_25530	paras_005640	protein-codi	NZ_CP1252	chromosom	5683162	5683914	+	753	0	17	1992	8	912	
QEN71_RS25540	hypothetical protein	QEN71_25535	paras_005641	protein-codi	NZ_CP1252	chromosom	5684224	5685066	+	843	0	20	1622	20	1622	
QEN71_RS25545	hypothetical protein	QEN71_25540	paras_005642	protein-codi	NZ_CP1252	chromosom	5685200	5686126	-	927	0	28	3015	26	2911	
QEN71_RS25550	hypothetical protein	QEN71_25545	paras_005643	protein-codi	NZ_CP1252	chromosom	5686643	5686921	-	279	0	5	209	4	189	
QEN71_RS25555	AAA family ATPase	QEN71_25550	paras_005644	protein-codi	NZ_CP1252	chromosom	5687011	5688996	-	1986	0	59	4966	49	4468	
QEN71_RS25560	methyl-accepting chemotaxis protein	QEN71_25555	paras_005645	protein-codi	NZ_CP1252	chromosom	5691065	5692615	-	1551	0	11	1374	8	1312	
QEN71_RS25565	hypothetical protein	QEN71_25560	paras_005646	protein-codi	NZ_CP1252	chromosom	5692754	5693191	-	438	0	2	7	1	5	TRUE
QEN71_RS25570	glycosyltransferase	QEN71_25565	paras_005647	protein-codi	NZ_CP1252	chromosom	5693329	5694429	+	1101	0	39	5157	30	3003	
QEN71_RS25575	glycosyltransferase 87 family protein	QEN71_25570	paras_005648	protein-codi	NZ_CP1252	chromosom	5694454	5696295	-	1842	0	48	9459	27	4290	
QEN71_RS25580	GtrA family protein	QEN71_25575	paras_005649	protein-codi	NZ_CP1252	chromosom	5696304	5696699	-	392	0	13	2302	13	2302	
QEN71_RS25585	glycosyltransferase family 2 protein	QEN71_25580	paras_005650	protein-codi	NZ_CP1252	chromosom	5696696	5697670	-	971	0	47	10633	37	9240	
QEN71_RS25590	nucleoside-diphosphate sugar epimerase/dehydrata	QEN71_25585	paras_005651	protein-codi	NZ_CP1252	chromosom	5697849	5699738	-	1890	0	57	13264	48	11393	
QEN71_RS25595	glycosyltransferase family 4 protein	QEN71_25590	paras_005652	protein-codi	NZ_CP1252	chromosom	5699744	5700772	-	1029	0	21	6895	13	2388	
QEN71_RS25600	SDR family oxidoreductase	QEN71_25595	paras_005653	protein-codi	NZ_CP1252	chromosom	5700780	5701736	-	953	0	24	9651	15	6433	
QEN71_RS25605	glycosyltransferase family 2 protein	QEN71_25600	paras_005654	protein-codi	NZ_CP1252	chromosom	5701733	5702578	-	842	0	47	22490	38	18280	
QEN71_RS25610	mannose-1-phosphate guanylyltransferase/mannos	QEN71_25605	paras_005655	protein-codi	NZ_CP1252	chromosom	5703137	5704570	+	1434	0	41	11096	26	6360	
QEN71_RS25615	GtrA family protein	QEN71_25610	paras_005656	protein-codi	NZ_CP1252	chromosom	5704722	5705180	+	459	0	25	5376	19	3915	
QEN71_RS25620	glycosyltransferase family 2 protein	QEN71_25615	paras_005657	protein-codi	NZ_CP1252	chromosom	5705481	5706632	+	1152	0	33	9564	23	5050	
QEN71_RS25625	glycosyltransferase family 2 protein	QEN71_25620	paras_005658	protein-codi	NZ_CP1252	chromosom	5706676	5708574	+	1895	0	79	26017	54	14020	
QEN71_RS25630	NAD-dependent epimerase/dehydratase family prot	QEN71_25625	paras_005659	protein-codi	NZ_CP1252	chromosom	5708571	5709503	+	929	0	36	6137	30	5444	
QEN71_RS25635	hypothetical protein	QEN71_25630	paras_005660	protein-codi	NZ_CP1252	chromosom	5709747	5712356	+	2610	0	83	22312	61	17805	
QEN71_RS25640	glycosyltransferase family 2 protein	QEN71_25635	paras_005661	protein-codi	NZ_CP1252	chromosom	5712368	5713312	+	945	0	42	11809	33	10382	
QEN71_RS25645	hypothetical protein	QEN71_25640	paras_005662	protein-codi	NZ_CP1252	chromosom	5714488	5715999	-	1512	0	104	19913	88	17528	
QEN71_RS25650	hypothetical protein			protein-codi	NZ_CP1252	chromosom	5716192	5716377	+	186	0	9	1146	6	1032	
QEN71_RS25655	class I SAM-dependent methyltransferase	QEN71_25650	paras_005664	protein-codi	NZ_CP1252	chromosom	5716469	5717143	-	675	0	37	6700	27	3286	
QEN71_RS25660	glycosyltransferase family 2 protein	QEN71_25655	paras_005665	protein-codi	NZ_CP1252	chromosom	5717147	5718097	-	951	0	56	9009	43	6177	
QEN71_RS25665	glycosyltransferase family 39 protein	QEN71_25660	paras_005666	protein-codi	NZ_CP1252	chromosom	5718623	5720380	-	1758	0	85	13342	71	11823	
QEN71_RS25670	sulfotransferase family 2 domain-containing protein	QEN71_25665	paras_005667	protein-codi	NZ_CP1252	chromosom	5720509	5721339	-	831	0	31	2582	21	1669	
QEN71_RS25675	hypothetical protein	QEN71_25670	paras_005668	protein-codi	NZ_CP1252	chromosom	5721722	5722645	+	924	0	51	13367	41	11648	
QEN71_RS25680	phytanoyl-CoA dioxygenase family protein	QEN71_25675	paras_005669	protein-codi	NZ_CP1252	chromosom	5722668	5723501	+	834	0	40	9880	35	9176	
QEN71_RS25685	methyltransferase domain-containing protein	QEN71_25680	paras_005670	protein-codi	NZ_CP1252	chromosom	5723580	5724605	-	1026	0	35	5680	27	4403	
QEN71_RS25690	glycosyltransferase family 4 protein	QEN71_25685	paras_005671	protein-codi	NZ_CP1252	chromosom	5724755	5726140	-	1386	0	68	8032	54	7178	
QEN71_RS25695	glycosyltransferase family 4 protein	QEN71_25690	paras_005672	protein-codi	NZ_CP1252	chromosom	5726192	5727805	-	1613	0	68	8962	62	7869	
QEN71_RS25700	DegT/DnrJ/EryC1/StrS family aminotransferase	QEN71_25695	paras_005673	protein-codi	NZ_CP1252	chromosom	5727805	5728902	-	1097	0	42	7588	24	3856	
QEN71_RS25705	FdtA/QdtA family cupin domain-containing protein	QEN71_25700	paras_005674	protein-codi	NZ_CP1252	chromosom	5728987	5729397	-	397	0	19	2918	13	2359	
QEN71_RS25710	GNAT family N-acetyltransferase	QEN71_25705	paras_005675	protein-codi	NZ_CP1252	chromosom	5729384	5730358	-	961	0	61	10937	43	8330	
QEN71_RS25715	acetyltransferase	QEN71_25710	paras_005676	protein-codi	NZ_CP1252	chromosom	5730361	5731038	-	678	0	26	4826	18	2966	
QEN71_RS25720	SAM-dependent methyltransferase	QEN71_25715	paras_005677	protein-codi	NZ_CP1252	chromosom	5731048	5731797	-	750	0	24	5847	20	5051	
QEN71_RS25725	ABC transporter ATP-binding protein	QEN71_25720	paras_005678	protein-codi	NZ_CP1252	chromosom	5731825	5733180	-	1345	0	42	15915	31	13978	
QEN71_RS25730	ABC transporter permease	QEN71_25725	paras_005679	protein-codi	NZ_CP1252	chromosom	5733170	5734024	-	844	0	23	5745	16	4323	
QEN71_RS25735	glycosyltransferase family 2 protein	QEN71_25730	paras_005680	protein-codi	NZ_CP1252	chromosom	5734183	5735094	-	893	0	24	8057	23	7997	
QEN71_RS25740	dTDP-4-dehydrorhamnose reductase	QEN71_25735	paras_005681	protein-codi	NZ_CP1252	chromosom	5735076	5735969	-	875	0	16	2953	14	353	
QEN71_RS25745	dTDP-4-dehydrorhamnose 3,5-epimerase	QEN71_25740	paras_005682	protein-codi	NZ_CP1252	chromosom	5735977	5736528	-	536	0	5	38	5	38	
QEN71_RS25750	glucose-1-phosphate thymidylyltransferase RfbA	QEN71_25745	paras_005683	protein-codi	NZ_CP1252	chromosom	5736513	5737406	-	878	0	23	376	15	86	
QEN71_RS25755	dTDP-glucose 4,6-dehydratase	QEN71_25750	paras_005684	protein-codi	NZ_CP1252	chromosom	5737417	5738478	-	1062	0	15	603	10	175	
QEN71_RS25760	GDP-mannose 4,6-dehydratase	QEN71_25755	paras_005685	protein-codi	NZ_CP1252	chromosom	5738678	5739580	-	903	0	28	6505	23	3741	
QEN71_RS25765	symmetrical bis(5'-nucleosyl)-tetraphosphatase	QEN71_25760	paras_005686	protein-codi	NZ_CP1252	chromosom	5739935	5740783	+	849	0	5	1995	2	1409	
QEN71_RS25770	lysophospholipid acyltransferase family protein	QEN71_25765	paras_005687	protein-codi	NZ_CP1252	chromosom	5740800	5741630	-	831	0	27	13516	17	11580	
QEN71_RS25775	dihydroorotase	QEN71_25770	paras_005688	protein-codi	NZ_CP1252	chromosom	5741681	5742958	-	1278	0	30	14473	25	13441	
QEN71_RS25780	aspartate carbamoyltransferase catalytic subunit	QEN71_25775	paras_005689	protein-codi	NZ_CP1252	chromosom	5743013	5744053	-	1041	0	23	10672	16	9863	
QEN71_RS25785	bifunctional pyr operon transcriptional regulator/ura	QEN71_25780	paras_005690	protein-codi	NZ_CP1252	chromosom	5744122	5744634	-	499	0	13	5569	12	5548	
QEN71_RS25790	Holliday junction resolvase RuvX	QEN71_25785	paras_005691	protein-codi	NZ_CP1252	chromosom	5744621	5745076	-	438	0	4	1658	2	6	
QEN71_RS25795	YqgE/AlgH family protein	QEN71_25790	paras_005692	protein-codi	NZ_CP1252	chromosom	5745073	5745648	-	572	0	12	4244	10	3888	
QEN71_RS25800	hypothetical protein	QEN71_25795	paras_005693	protein-codi	NZ_CP1252	chromosom	5745918	5747327	-	1410	0	37	16889	33	15816	
QEN71_RS25805	rubredoxin	QEN71_25800	paras_005694	protein-codi	NZ_CP1252	chromosom	5747724	5747894	-	171	0	6	482	2	74	
QEN71_RS25810	hydroxymethylpyrimidine/phosphomethylpyrimidine	QEN71_25805	paras_005695	protein-codi	NZ_CP1252	chromosom	5748158	5749018	+	861	0	22	11231	22	11231	

QEN71_RS25815	adenylyl-sulfate kinase	QEN71_25810	paras_005696	protein-codi	NZ_CP1252	chromosom	5749445	5750047	+	603	0	16	11776	12	9809	
QEN71_RS25820	chaperonin GroEL	QEN71_25815	paras_005697	protein-codi	NZ_CP1252	chromosom	5750185	5751825	-	1641	0	4	849	1	5	TRUE
QEN71_RS25825	co-chaperone GroES	QEN71_25820	paras_005698	protein-codi	NZ_CP1252	chromosom	5751869	5752159	-	291	0	2	831	0	0	
QEN71_RS25830	GAF domain-containing protein	QEN71_25825	paras_005699	protein-codi	NZ_CP1252	chromosom	5752688	5753512	+	825	0	27	4448	18	3426	
QEN71_RS25835	hypothetical protein	QEN71_25830	paras_005700	protein-codi	NZ_CP1252	chromosom	5753684	5753893	+	210	0	4	347	3	253	
QEN71_RS25840	zinc ribbon domain-containing protein	QEN71_25835	paras_005701	protein-codi	NZ_CP1252	chromosom	5753970	5754890	-	921	0	7	419	5	405	
QEN71_RS25845	MFS transporter	QEN71_25840	paras_005702	protein-codi	NZ_CP1252	chromosom	5755349	5756746	-	1398	0	33	6476	32	6469	
QEN71_RS25850	RNA-guided endonuclease TnpB family protein	QEN71_25845	paras_005703	protein-codi	NZ_CP1252	chromosom	5756860	5757993	+	1134	0	37	4367	23	3101	
QEN71_RS25855	methyl-accepting chemotaxis protein	QEN71_25850	paras_005704	protein-codi	NZ_CP1252	chromosom	5758080	5758817	-	738	0	14	1771	7	1298	
QEN71_RS25860	sugar ABC transporter permease	QEN71_25855	paras_005705	protein-codi	NZ_CP1252	chromosom	5759284	5760480	-	1193	0	24	2818	19	2270	
QEN71_RS25865	D-xylose ABC transporter ATP-binding protein	QEN71_25860	paras_005706	protein-codi	NZ_CP1252	chromosom	5760477	5762060	-	1580	0	12	1672	12	1672	
QEN71_RS25870	D-xylose ABC transporter substrate-binding protein	QEN71_25865	paras_005707	protein-codi	NZ_CP1252	chromosom	5762160	5763188	-	1029	0	15	1902	12	1533	
QEN71_RS25875	xylose isomerase	QEN71_25870	paras_005708	protein-codi	NZ_CP1252	chromosom	5763415	5764740	-	1326	0	29	1720	20	1263	
QEN71_RS25880	DNA-binding transcriptional regulator	QEN71_25875	paras_005709	protein-codi	NZ_CP1252	chromosom	5765007	5766230	+	1224	0	37	4767	25	1555	
QEN71_RS25885	aldose epimerase family protein	QEN71_25880	paras_005710	protein-codi	NZ_CP1252	chromosom	5766262	5767353	+	1092	0	24	3376	22	3227	
QEN71_RS25890	PepSY-associated TM helix domain-containing prot	QEN71_25885	paras_005711	protein-codi	NZ_CP1252	chromosom	5767644	5768357	+	714	0	7	3763	7	3763	
QEN71_RS25895	SDR family oxidoreductase	QEN71_25890	paras_005712	protein-codi	NZ_CP1252	chromosom	5768436	5769209	-	774	0	4	286	3	284	
QEN71_RS25900	TetR/AcrR family transcriptional regulator	QEN71_25895	paras_005713	protein-codi	NZ_CP1252	chromosom	5769373	5769957	+	585	0	8	1452	6	1089	
QEN71_RS25905	hypothetical protein	QEN71_25900	paras_005714	protein-codi	NZ_CP1252	chromosom	5770210	5770470	+	261	0	5	915	3	740	
QEN71_RS25910	carbohydrate ABC transporter permease	QEN71_25905	paras_005715	protein-codi	NZ_CP1252	chromosom	5770540	5771391	-	852	0	14	2187	9	1108	
QEN71_RS25915	sugar ABC transporter permease	QEN71_25910	paras_005716	protein-codi	NZ_CP1252	chromosom	5771405	5772358	-	954	0	36	4678	29	3649	
QEN71_RS25920	extracellular solute-binding protein	QEN71_25915	paras_005717	protein-codi	NZ_CP1252	chromosom	5772426	5773691	-	1266	107.0	36	4829	30	4355	
QEN71_RS25925	beta-galactosidase	QEN71_25920	paras_005718	protein-codi	NZ_CP1252	chromosom	5773777	5775759	-	1983	0	47	4070	34	3063	
QEN71_RS25930	sn-glycerol-3-phosphate ABC transporter ATP-bindi	QEN71_25925	paras_005719	protein-codi	NZ_CP1252	chromosom	5775771	5776922	-	1152	0	21	3810	10	2626	
QEN71_RS25935	LacI family DNA-binding transcriptional regulator	QEN71_25930	paras_005720	protein-codi	NZ_CP1252	chromosom	5777149	5778183	+	1035	0	17	1508	15	1318	
QEN71_RS25940	MerR family transcriptional regulator	QEN71_25935	paras_005721	protein-codi	NZ_CP1252	chromosom	5778444	5779427	+	984	0	21	1965	17	1647	
QEN71_RS25945	sensor domain-containing diguanylate cyclase	QEN71_25940	paras_005722	protein-codi	NZ_CP1252	chromosom	5779483	5780442	-	960	0	23	4694	10	2086	
QEN71_RS25950	hybrid sensor histidine kinase/response regulator	QEN71_25945	paras_005723	protein-codi	NZ_CP1252	chromosom	5780608	5782209	-	1602	0	33	5729	24	5141	
QEN71_RS25955	lipocalin family protein	QEN71_25950	paras_005724	protein-codi	NZ_CP1252	chromosom	5782538	5783134	-	597	0	21	4414	18	4211	
QEN71_RS25960	aldo/keto reductase	QEN71_25955	paras_005725	protein-codi	NZ_CP1252	chromosom	5783325	5784320	+	992	0	31	4343	26	3385	
QEN71_RS25965	YbhB/YbcL family Raf kinase inhibitor-like protein	QEN71_25960	paras_005726	protein-codi	NZ_CP1252	chromosom	5784317	5784880	-	560	0	15	1985	11	1579	
QEN71_RS25970	ROK family protein	QEN71_25965	paras_005727	protein-codi	NZ_CP1252	chromosom	5784966	5785775	-	810	0	15	1860	12	1845	
QEN71_RS25975	glucose-6-phosphate dehydrogenase	QEN71_25970	paras_005728	protein-codi	NZ_CP1252	chromosom	5785822	5787411	-	1590	0	34	3321	28	2729	
QEN71_RS25980	glucosidase	QEN71_25975	paras_005729	protein-codi	NZ_CP1252	chromosom	5787515	5790250	-	2736	0	83	15032	70	13760	
QEN71_RS25985	amylo-alpha-1,6-glucosidase	QEN71_25980	paras_005730	protein-codi	NZ_CP1252	chromosom	5790307	5792283	-	1977	0	45	9054	44	8985	
QEN71_RS25990	ATP-binding protein	QEN71_25985	paras_005731	protein-codi	NZ_CP1252	chromosom	5792373	5793770	-	1394	0	29	5756	22	4714	
QEN71_RS25995	response regulator	QEN71_25990	paras_005732	protein-codi	NZ_CP1252	chromosom	5793767	5794480	-	710	0	14	3187	8	1295	
QEN71_RS26000	EF-hand domain-containing protein	QEN71_25995	paras_005733	protein-codi	NZ_CP1252	chromosom	5794554	5794847	-	294	0	8	2197	7	2026	
QEN71_RS26005	hypothetical protein	QEN71_26000	paras_005734	protein-codi	NZ_CP1252	chromosom	5795178	5795534	+	357	0	20	5652	16	3879	
QEN71_RS26010	ferritin-like domain-containing protein	QEN71_26005	paras_005735	protein-codi	NZ_CP1252	chromosom	5795650	5796120	+	471	0	17	2991	14	2670	
QEN71_RS26015	efflux transporter outer membrane subunit	QEN71_26010	paras_005736	protein-codi	NZ_CP1252	chromosom	5796613	5798064	+	1452	0	37	7012	32	6141	
QEN71_RS26020	efflux RND transporter periplasmic adaptor subunit	QEN71_26015	paras_005737	protein-codi	NZ_CP1252	chromosom	5798166	5799317	+	1152	192.0	23	2347	21	1819	
QEN71_RS26025	efflux RND transporter permease subunit	QEN71_26020	paras_005738	protein-codi	NZ_CP1252	chromosom	5799328	5802387	+	3060	287.0	83	10293	62	7285	
QEN71_RS26030	LysE family translocator	QEN71_26025	paras_005739	protein-codi	NZ_CP1252	chromosom	5802985	5803626	+	642	0	14	1838	12	1505	
QEN71_RS26035	EamA family transporter RarD	QEN71_26030	paras_005740	protein-codi	NZ_CP1252	chromosom	5803650	5804525	-	876	0	26	6875	19	5765	
QEN71_RS26040	sulfite exporter TauE/SafE family protein	QEN71_26035	paras_005741	protein-codi	NZ_CP1252	chromosom	5804846	5805634	+	789	0	7	1046	7	1046	
QEN71_RS26045	hypothetical protein	QEN71_26040	paras_005742	protein-codi	NZ_CP1252	chromosom	5805671	5805925	-	255	0	10	2748	4	248	
QEN71_RS26050	D-(-)-3-hydroxybutyrate oligomer hydrolase	QEN71_26045	paras_005743	protein-codi	NZ_CP1252	chromosom	5806153	5808273	-	2121	0	47	13174	34	9381	
QEN71_RS26055	BON domain-containing protein	QEN71_26050	paras_005744	protein-codi	NZ_CP1252	chromosom	5808774	5809130	-	357	0	8	1600	4	1245	
QEN71_RS26060	TAXI family TRAP transporter solute-binding subuni	QEN71_26055	paras_005745	protein-codi	NZ_CP1252	chromosom	5809702	5811231	+	1530	0	38	13049	35	10858	
QEN71_RS26065	2-hydroxychromene-2-carboxylate isomerase	QEN71_26060	paras_005746	protein-codi	NZ_CP1252	chromosom	5811398	5812042	+	645	0	14	2960	9	2118	
QEN71_RS26070	LysR substrate-binding domain-containing protein	QEN71_26065	paras_005747	protein-codi	NZ_CP1252	chromosom	5812270	5813298	+	1029	0	20	2730	16	2093	
QEN71_RS26075	xanthine dehydrogenase accessory protein XdhC	QEN71_26070	paras_005748	protein-codi	NZ_CP1252	chromosom	5813300	5813890	-	591	0	7	1127	7	1127	
QEN71_RS26080	xanthine dehydrogenase molybdopterin binding sub	QEN71_26075	paras_005749	protein-codi	NZ_CP1252	chromosom	5813936	5816287	-	2352	0	51	11239	44	8287	
QEN71_RS26085	xanthine dehydrogenase small subunit	QEN71_26080	paras_005750	protein-codi	NZ_CP1252	chromosom	5816321	5817847	-	1527	0	28	8155	23	5652	
QEN71_RS26090	MFS transporter	QEN71_26085	paras_005751	protein-codi	NZ_CP1252	chromosom	5818247	5819458	+	1212	0	38	10547	28	8014	
QEN71_RS26095	hypothetical protein	QEN71_26090	paras_005752	protein-codi	NZ_CP1252	chromosom	5819461	5819622	-	162	167.0	8	1098	6	468	
QEN71_RS26100	aminotransferase class V-fold PLP-dependent enzy	QEN71_26095	paras_005753	protein-codi	NZ_CP1252	chromosom	5820082	5821308	-	1219	0	30	6346	18	3730	
QEN71_RS26105	hypothetical protein	QEN71_26100	paras_005754	protein-codi	NZ_CP1252	chromosom	5821301	5821465	+	157	0	0	0	0	0	
QEN71_RS26110	bifunctional aspartate transaminase/aspartate 4-de	QEN71_26105	paras_005755	protein-codi	NZ_CP1252	chromosom	5821482	5823128	-	1647	0	47	9005	40	7799	
QEN71_RS26115	aspartate-alanine antiporter	QEN71_26110	paras_005756	protein-codi	NZ_CP1252	chromosom	5823255	5824940	-	1686	0	37	9578	27	7599	
QEN71_RS26120	aspartate-alanine antiporter	QEN71_26115	paras_005757	protein-codi	NZ_CP1252	chromosom	5825092	5826780	-	1689	0	27	5882	17	3648	

QEN71_RS26125	LysR family transcriptional regulator	QEN71_26120	paras_005758	protein-codi	NZ_CP1252	chromosom	5827374	5828363	+	990	0	21	5732	17	4777	
QEN71_RS26130	2-hydroxyacid dehydrogenase	QEN71_26125	paras_005759	protein-codi	NZ_CP1252	chromosom	5828427	5829434	+	1008	0	18	4605	15	4403	
QEN71_RS26135	hypothetical protein	QEN71_26130	paras_005760	protein-codi	NZ_CP1252	chromosom	5829478	5830041	-	564	0	17	3597	13	2879	
QEN71_RS26140	crotonase/enoyl-CoA hydratase family protein	QEN71_26135	paras_005761	protein-codi	NZ_CP1252	chromosom	5830314	5831168	+	855	0	4	136	4	136	
QEN71_RS26145	MFS transporter	QEN71_26140	paras_005762	protein-codi	NZ_CP1252	chromosom	5831373	5832530	+	1158	0	9	2211	9	2211	
QEN71_RS26150	MFS transporter	QEN71_26145	paras_005763	protein-codi	NZ_CP1252	chromosom	5832628	5833785	+	1158	0	15	4721	13	4699	
QEN71_RS26155	fumarylacetoacetase	QEN71_26150	paras_005764	protein-codi	NZ_CP1252	chromosom	5833846	5835156	-	1307	0	21	7087	19	6044	
QEN71_RS26160	homogentisate 1,2-dioxygenase	QEN71_26155	paras_005765	protein-codi	NZ_CP1252	chromosom	5835153	5836475	-	1319	0	35	11408	30	10640	
QEN71_RS26165	MFS transporter	QEN71_26160	paras_005766	protein-codi	NZ_CP1252	chromosom	5836495	5837850	-	1356	0	16	2997	12	2414	
QEN71_RS26170	EAL domain-containing protein	QEN71_26165	paras_005767	protein-codi	NZ_CP1252	chromosom	5838072	5838902	+	831	0	22	6552	18	6029	
QEN71_RS26175	IclR family transcriptional regulator	QEN71_26170	paras_005768	protein-codi	NZ_CP1252	chromosom	5838909	5839694	-	786	0	19	7066	19	7066	
QEN71_RS26180	acyl-CoA dehydrogenase	QEN71_26175	paras_005769	protein-codi	NZ_CP1252	chromosom	5839854	5841044	+	1191	0	20	7481	13	5045	
QEN71_RS26185	peroxiredoxin	QEN71_26180	paras_005770	protein-codi	NZ_CP1252	chromosom	5841135	5841773	-	639	0	23	9841	12	3236	
QEN71_RS26190	DUF883 family protein	QEN71_26185	paras_005771	protein-codi	NZ_CP1252	chromosom	5842168	5842470	+	303	0	6	2106	4	1892	
QEN71_RS26195	phage holin family protein	QEN71_26190	paras_005772	protein-codi	NZ_CP1252	chromosom	5842591	5842974	+	384	0	6	1382	4	1105	
QEN71_RS26200	DUF3318 domain-containing protein	QEN71_26195	paras_005773	protein-codi	NZ_CP1252	chromosom	5843020	5843505	+	486	0	6	2093	4	1737	
QEN71_RS26205	type IV pilin protein	pseudo;QEN71_26200		pseudogene	NZ_CP1252	chromosom	5843533	5843950	-	418	0	22	2706	14	949	
QEN71_RS26210	hypothetical protein	QEN71_26205	paras_005775	protein-codi	NZ_CP1252	chromosom	5843974	5844498	-	521	0	12	4781	12	4781	
QEN71_RS26215	PilW family protein	QEN71_26210	paras_005776	protein-codi	NZ_CP1252	chromosom	5844495	5845253	-	755	0	31	10583	20	5565	
QEN71_RS26220	prepilin-type N-terminal cleavage/methylation doma	QEN71_26215	paras_005777	protein-codi	NZ_CP1252	chromosom	5845331	5845747	-	413	0	11	2127	11	2127	
QEN71_RS26225	GspH/FimT family pseudopilin	QEN71_26220	paras_005778	protein-codi	NZ_CP1252	chromosom	5845744	5846304	-	557	0	11	3384	11	3384	
QEN71_RS26230	transcriptional regulator NrdR	QEN71_26225	paras_005779	protein-codi	NZ_CP1252	chromosom	5846476	5846967	-	492	0	5	750	5	750	
QEN71_RS26235	serine hydroxymethyltransferase	QEN71_26230	paras_005780	protein-codi	NZ_CP1252	chromosom	5846974	5848221	-	1248	141.0	26	2409	22	2112	
QEN71_RS26240	bifunctional NADP-dependent 3-hydroxy acid dehyd	QEN71_26235	paras_005781	protein-codi	NZ_CP1252	chromosom	5848723	5849469	+	747	0	13	4790	9	3089	
QEN71_RS26245	tol-pal system-associated acyl-CoA thioesterase	QEN71_26240	paras_005782	protein-codi	NZ_CP1252	chromosom	5849605	5850084	+	480	0	17	5740	12	2947	
QEN71_RS26250	protein TolQ	QEN71_26245	paras_005783	protein-codi	NZ_CP1252	chromosom	5850253	5850930	+	678	0	11	533	7	278	
QEN71_RS26255	protein TolR	QEN71_26250	paras_005784	protein-codi	NZ_CP1252	chromosom	5850941	5851387	+	443	0	5	158	5	158	
QEN71_RS26260	cell envelope integrity protein TolA	QEN71_26255	paras_005785	protein-codi	NZ_CP1252	chromosom	5851384	5852427	+	1040	0	7	147	7	147	
QEN71_RS26265	Tol-Pal system beta propeller repeat protein TolB	QEN71_26260	paras_005786	protein-codi	NZ_CP1252	chromosom	5852544	5853836	+	1293	0	38	1539	30	623	
QEN71_RS26270	peptidoglycan-associated lipoprotein Pal	QEN71_26265	paras_005787	protein-codi	NZ_CP1252	chromosom	5853856	5854365	+	510	0	15	1627	9	206	
QEN71_RS26275	tol-pal system protein YbgF	QEN71_26270	paras_005788	protein-codi	NZ_CP1252	chromosom	5854381	5855130	+	750	0	21	1853	17	1466	
QEN71_RS26280	tRNA-Lys	QEN71_26275		tRNA	NZ_CP1252	chromosom	5855341	5855416	+	76	0	0	0	0	0	
QEN71_RS26285	hypothetical protein	QEN71_26280		protein-codi	NZ_CP1252	chromosom	5855628	5855843	-	216	0	9	920	8	814	
QEN71_RS26290	glycosyltransferase	QEN71_26285	paras_005790	protein-codi	NZ_CP1252	chromosom	5855930	5857090	+	1161	0	29	6817	22	5384	
QEN71_RS26295	glycosyltransferase	QEN71_26290	paras_005791	protein-codi	NZ_CP1252	chromosom	5857161	5858228	-	1067	0	20	4042	16	3514	
QEN71_RS26300	oligosaccharide flippase family protein	QEN71_26295	paras_005792	protein-codi	NZ_CP1252	chromosom	5858228	5859475	-	1247	0	30	6471	23	5148	
QEN71_RS26305	glycosyltransferase	QEN71_26300	paras_005793	protein-codi	NZ_CP1252	chromosom	5859736	5860995	+	1260	0	36	6413	27	4225	
QEN71_RS26310	acyltransferase	QEN71_26305	paras_005794	protein-codi	NZ_CP1252	chromosom	5861053	5862327	-	1275	0	26	5421	23	4912	
QEN71_RS26315	acyltransferase	QEN71_26310	paras_005795	protein-codi	NZ_CP1252	chromosom	5862414	5863559	-	1146	0	34	5662	30	5457	
QEN71_RS26320	hypothetical protein	QEN71_26315	paras_005796	protein-codi	NZ_CP1252	chromosom	5863992	5865491	+	1500	0	37	8128	27	5097	
QEN71_RS26325	glycosyltransferase	QEN71_26320	paras_005797	protein-codi	NZ_CP1252	chromosom	5865588	5866883	+	1296	0	37	6156	32	5360	
QEN71_RS26330	GMC family oxidoreductase	QEN71_26325	paras_005798	protein-codi	NZ_CP1252	chromosom	5867030	5868769	-	1740	0	38	6187	33	5591	
QEN71_RS26335	DapH/DapD/GlmU-related protein	QEN71_26330	paras_005799	protein-codi	NZ_CP1252	chromosom	5869025	5869735	-	711	0	16	3482	9	2655	
QEN71_RS26340	hypothetical protein	QEN71_26335	paras_005800	protein-codi	NZ_CP1252	chromosom	5869947	5870585	+	639	0	12	1394	5	840	
QEN71_RS26345	glycosyltransferase WbuB	QEN71_26340	paras_005801	protein-codi	NZ_CP1252	chromosom	5870709	5872016	-	1308	0	32	5140	18	2036	
QEN71_RS26350	glycosyltransferase family 4 protein	QEN71_26345	paras_005802	protein-codi	NZ_CP1252	chromosom	5872128	5873270	-	1143	0	28	2634	22	1495	
QEN71_RS26355	GDP-L-fucose synthase	QEN71_26350	paras_005803	protein-codi	NZ_CP1252	chromosom	5873377	5874393	-	1017	0	20	3027	18	2949	
QEN71_RS26360	GDP-mannose 4,6-dehydratase	QEN71_26355	paras_005804	protein-codi	NZ_CP1252	chromosom	5874398	5875525	-	1128	0	33	5102	27	4500	
QEN71_RS26365	polysaccharide biosynthesis tyrosine autokinase	QEN71_26360	paras_005805	protein-codi	NZ_CP1252	chromosom	5875640	5877865	-	2226	0	29	3994	18	3389	
QEN71_RS26370	polysaccharide biosynthesis/export family protein	QEN71_26365	paras_005806	protein-codi	NZ_CP1252	chromosom	5877953	5879092	-	1140	0	34	5056	25	3766	
QEN71_RS26375	undecaprenyl-phosphate glucose phosphotransfera	QEN71_26370	paras_005807	protein-codi	NZ_CP1252	chromosom	5879148	5880533	-	1386	0	44	6828	27	4219	
QEN71_RS26380	mannose-1-phosphate guanylyltransferase/mannos	QEN71_26375	paras_005808	protein-codi	NZ_CP1252	chromosom	5880604	5882121	-	1518	0	29	4048	19	1778	
QEN71_RS26385	helix-turn-helix domain-containing protein	QEN71_26380	paras_005809	protein-codi	NZ_CP1252	chromosom	5882197	5883201	-	1005	0	15	3443	8	3092	
QEN71_RS26390	hypothetical protein	QEN71_26385	paras_005810	protein-codi	NZ_CP1252	chromosom	5884166	5886370	+	2205	0	62	12021	55	11034	
QEN71_RS26395	hypothetical protein	QEN71_26390	paras_005811	protein-codi	NZ_CP1252	chromosom	5886550	5886690	+	141	0	3	160	2	154	
QEN71_RS26400	GDP-mannose mannosyl hydrolase	QEN71_26395	paras_005812	protein-codi	NZ_CP1252	chromosom	5886813	5887286	+	474	0	27	5028	21	4133	
QEN71_RS26405	UTP--glucose-1-phosphate uridylyltransferase GalU	QEN71_26400	paras_005813	protein-codi	NZ_CP1252	chromosom	5887368	5888300	-	933	0	23	2849	19	2621	
QEN71_RS26410	helix-turn-helix domain-containing protein	QEN71_26405	paras_005814	protein-codi	NZ_CP1252	chromosom	5888521	5889555	+	1035	0	11	1503	8	1362	
QEN71_RS26415	MFS transporter	QEN71_26410	paras_005815	protein-codi	NZ_CP1252	chromosom	5889697	5891187	+	1491	0	34	3179	28	2962	
QEN71_RS26420	hypothetical protein	QEN71_26415	paras_005816	protein-codi	NZ_CP1252	chromosom	5891367	5891801	+	435	0	9	830	9	830	
QEN71_RS26425	mechanosensitive ion channel family protein	QEN71_26420	paras_005817	protein-codi	NZ_CP1252	chromosom	5892077	5892889	+	813	0	26	5110	19	4243	
QEN71_RS26430	DedA family protein	QEN71_26425	paras_005818	protein-codi	NZ_CP1252	chromosom	5892954	5893643	-	690	0	6	260	4	221	

QEN71_RS26435	DNA mismatch repair endonuclease MutL	QEN71_26430	paras_005819	protein-codi	NZ_CP1252	chromosom	5893845	5895914	+	2070	0	31	6729	28	6150	
QEN71_RS26440	tRNA (adenosine(37)-N6)-dimethylallyltransferase M	QEN71_26435	paras_005820	protein-codi	NZ_CP1252	chromosom	5895949	5896902	+	954	0	29	1337	21	1151	
QEN71_RS26445	phosphoribosylformylglycinamidine cyclo-ligase	QEN71_26440	paras_005821	protein-codi	NZ_CP1252	chromosom	5897551	5898618	-	1068	0	22	3323	18	2627	
QEN71_RS26450	AI-2E family transporter	QEN71_26445	paras_005822	protein-codi	NZ_CP1252	chromosom	5898716	5899792	+	1077	0	25	7590	17	5737	
QEN71_RS26455	DnaA regulatory inactivator Hda	QEN71_26450	paras_005823	protein-codi	NZ_CP1252	chromosom	5899847	5900620	+	766	0	3	715	0	0	
QEN71_RS26460	HAD family hydrolase	QEN71_26455	paras_005824	protein-codi	NZ_CP1252	chromosom	5900613	5901299	+	675	0	15	2511	15	2511	
QEN71_RS26465	polynucleotide adenylyltransferase PcnB	QEN71_26460	paras_005825	protein-codi	NZ_CP1252	chromosom	5901296	5902891	+	1592	0	12	2012	12	2012	
QEN71_RS26470	2-amino-4-hydroxy-6- hydroxymethyldihydropteridin	QEN71_26465	paras_005826	protein-codi	NZ_CP1252	chromosom	5902940	5903494	+	555	0	2	31	1	10	TRUE
QEN71_RS26475	deoxynucleoside kinase	QEN71_26470	paras_005827	protein-codi	NZ_CP1252	chromosom	5903506	5904186	+	681	0	27	8660	21	6442	
QEN71_RS26480	3-methyl-2-oxobutanoate hydroxymethyltransferase	QEN71_26475	paras_005828	protein-codi	NZ_CP1252	chromosom	5904229	5905044	+	816	0	22	4270	13	1842	
QEN71_RS26485	aminodeoxychorismate synthase component I	QEN71_26480	paras_005829	protein-codi	NZ_CP1252	chromosom	5905381	5907246	-	1866	0	52	13112	43	11111	
QEN71_RS26490	molecular chaperone DnaJ	QEN71_26485	paras_005830	protein-codi	NZ_CP1252	chromosom	5907271	5908404	-	1134	0	10	256	7	49	
QEN71_RS26495	molecular chaperone DnaK	QEN71_26490	paras_005831	protein-codi	NZ_CP1252	chromosom	5908660	5910609	-	1950	0	5	242	2	8	TRUE
QEN71_RS26500	thioredoxin family protein	QEN71_26495	paras_005832	protein-codi	NZ_CP1252	chromosom	5910763	5911230	-	468	0	13	2047	11	1946	
QEN71_RS26505	nucleotide exchange factor GrpE	QEN71_26500	paras_005833	protein-codi	NZ_CP1252	chromosom	5911247	5911861	-	615	0	3	2295	0	0	
QEN71_RS26510	hypothetical protein	QEN71_26505	paras_005834	protein-codi	NZ_CP1252	chromosom	5912208	5912369	+	133	0	8	2016	8	2016	
QEN71_RS26515	RNA-binding S4 domain-containing protein	QEN71_26510	paras_005835	protein-codi	NZ_CP1252	chromosom	5912341	5912748	-	379	0	8	2309	5	1043	
QEN71_RS26520	ferrochelatase	QEN71_26515	paras_005836	protein-codi	NZ_CP1252	chromosom	5912822	5913892	-	1071	0	2	112	1	8	TRUE
QEN71_RS26525	heat-inducible transcriptional repressor HrcA	QEN71_26520	paras_005837	protein-codi	NZ_CP1252	chromosom	5914054	5915073	-	1020	0	38	15394	20	6628	
QEN71_RS26530	NAD kinase	QEN71_26525	paras_005838	protein-codi	NZ_CP1252	chromosom	5915355	5916257	+	903	0	5	126	2	13	TRUE
QEN71_RS26535	DNA repair protein RecN	QEN71_26530	paras_005839	protein-codi	NZ_CP1252	chromosom	5916331	5917998	+	1668	0	21	4310	13	1372	
QEN71_RS26540	bifunctional [glutamate--ammonia ligase]-adenylyl-L	QEN71_26535	paras_005840	protein-codi	NZ_CP1252	chromosom	5918075	5920867	-	2793	0	62	14899	42	12099	
QEN71_RS26545	AsmA-like C-terminal region-containing protein	QEN71_26540	paras_005841	protein-codi	NZ_CP1252	chromosom	5921054	5925286	+	4233	0	66	26385	46	17631	
QEN71_RS26550	carbon-nitrogen hydrolase family protein	QEN71_26545	paras_005842	protein-codi	NZ_CP1252	chromosom	5925403	5926251	+	849	0	27	8653	21	7027	
QEN71_RS26555	metalloprotease TldD	QEN71_26550	paras_005843	protein-codi	NZ_CP1252	chromosom	5926362	5927828	+	1467	0	45	12332	33	10465	
QEN71_RS26560	3-deoxy-7-phosphoheptulonate synthase AroG	QEN71_26555	paras_005844	protein-codi	NZ_CP1252	chromosom	5928225	5929298	+	1074	0	27	7593	19	6682	
QEN71_RS26565	IS481 family transposase	QEN71_26560	paras_005845	protein-codi	NZ_CP1252	chromosom	5929450	5930589	-	1140	2266.0	9	1530	9	1530	
QEN71_RS26570	cob(I)yrinic acid a,c-diamide adenosyltransferase	QEN71_26565	paras_005846	protein-codi	NZ_CP1252	chromosom	5931159	5931725	-	567	0	19	5543	15	4307	
QEN71_RS26575	FAD-linked oxidase C-terminal domain-containing p	QEN71_26570	paras_005847	protein-codi	NZ_CP1252	chromosom	5931929	5933344	+	1416	0	21	3974	17	3474	
QEN71_RS26580	FAD-linked oxidase C-terminal domain-containing p	QEN71_26575	paras_005848	protein-codi	NZ_CP1252	chromosom	5933409	5934914	+	1506	0	33	8757	26	7373	
QEN71_RS26585	glycolate oxidase subunit GlcE	QEN71_26580	paras_005849	protein-codi	NZ_CP1252	chromosom	5935134	5936222	+	1089	0	15	3555	9	1437	
QEN71_RS26590	glycolate oxidase subunit GlcF	QEN71_26585	paras_005850	protein-codi	NZ_CP1252	chromosom	5936232	5937458	+	1227	0	29	7074	23	5331	
QEN71_RS26595	YggS family pyridoxal phosphate-dependent enzym	QEN71_26590	paras_005851	protein-codi	NZ_CP1252	chromosom	5937576	5938274	+	699	0	6	973	4	752	
QEN71_RS26600	pyrroline-5-carboxylate reductase	QEN71_26595	paras_005852	protein-codi	NZ_CP1252	chromosom	5938289	5939104	+	816	0	7	2156	7	2156	
QEN71_RS26605	4-hydroxybenzoate octaprenyltransferase	QEN71_26600	paras_005853	protein-codi	NZ_CP1252	chromosom	5939183	5940046	-	864	0	4	570	1	10	
QEN71_RS26610	Dps family protein	QEN71_26605	paras_005854	protein-codi	NZ_CP1252	chromosom	5940202	5940678	-	477	0	18	5334	11	3200	
QEN71_RS26615	hypothetical protein	QEN71_26610	paras_005855	protein-codi	NZ_CP1252	chromosom	5940851	5941036	-	186	0	4	1385	4	1385	
QEN71_RS26620	catalase	QEN71_26615	paras_005856	protein-codi	NZ_CP1252	chromosom	5941100	5942548	-	1449	0	35	8384	31	7527	
QEN71_RS26625	LysR substrate-binding domain-containing protein	QEN71_26620	paras_005857	protein-codi	NZ_CP1252	chromosom	5942927	5943886	-	960	0	19	6809	16	4614	
QEN71_RS26630	ATP-dependent DNA helicase RecG	QEN71_26625	paras_005858	protein-codi	NZ_CP1252	chromosom	5944011	5946233	-	2223	0	20	808	17	652	
QEN71_RS26635	tRNA preQ1(34) S-adenosylmethionine ribosyltrans	QEN71_26630	paras_005859	protein-codi	NZ_CP1252	chromosom	5946450	5947511	+	1062	0	30	6769	22	5877	
QEN71_RS26640	tRNA guanosine(34) transglycosylase Tgt	QEN71_26635	paras_005860	protein-codi	NZ_CP1252	chromosom	5947595	5948803	+	1209	0	25	3844	19	1867	
QEN71_RS26645	preprotein translocase subunit YajC	QEN71_26640	paras_005861	protein-codi	NZ_CP1252	chromosom	5948996	5949325	+	330	0	3	196	2	42	
QEN71_RS26650	protein translocase subunit SecD	QEN71_26645	paras_005862	protein-codi	NZ_CP1252	chromosom	5949447	5951492	+	2046	0	2	78	0	0	TRUE
QEN71_RS26655	protein translocase subunit SecF	QEN71_26650	paras_005863	protein-codi	NZ_CP1252	chromosom	5951511	5952461	+	951	0	2	6	2	6	TRUE
QEN71_RS26660	MFS transporter	QEN71_26655	paras_005864	protein-codi	NZ_CP1252	chromosom	5952575	5953852	-	1278	0	42	9327	36	7765	
QEN71_RS26665	YceI family protein	QEN71_26660	paras_005865	protein-codi	NZ_CP1252	chromosom	5954120	5954689	-	570	0	18	5817	14	5090	
QEN71_RS26670	YceI family protein	QEN71_26665	paras_005866	protein-codi	NZ_CP1252	chromosom	5954742	5955305	-	564	0	20	4109	12	2141	
QEN71_RS26675	cytochrome b	QEN71_26670	paras_005867	protein-codi	NZ_CP1252	chromosom	5955314	5955886	-	573	0	24	7857	17	5135	
QEN71_RS26680	paraquat-inducible protein A	QEN71_26675	paras_005868	protein-codi	NZ_CP1252	chromosom	5956299	5957045	+	747	0	7	2727	6	2387	
QEN71_RS26685	paraquat-inducible protein A	QEN71_26680	paras_005869	protein-codi	NZ_CP1252	chromosom	5957053	5957724	+	664	0	15	4854	13	3869	
QEN71_RS26690	MlaD family protein	QEN71_26685	paras_005870	protein-codi	NZ_CP1252	chromosom	5957717	5959351	+	1623	0	19	5869	16	5716	
QEN71_RS26695	PqiC family protein	QEN71_26690	paras_005871	protein-codi	NZ_CP1252	chromosom	5959348	5960049	+	698	0	12	3711	8	1717	
QEN71_RS26700	class I SAM-dependent RNA methyltransferase	QEN71_26695	paras_005872	protein-codi	NZ_CP1252	chromosom	5960120	5961403	+	1284	0	19	3265	11	1296	
QEN71_RS26705	VOC family protein	QEN71_26700	paras_005873	protein-codi	NZ_CP1252	chromosom	5961571	5961954	+	384	0	6	511	6	511	
QEN71_RS26710	YafY family protein	QEN71_26705	paras_005874	protein-codi	NZ_CP1252	chromosom	5962038	5962724	+	687	0	21	2141	19	2105	
QEN71_RS26715	IS481 family transposase	QEN71_26710	paras_005875	protein-codi	NZ_CP1252	chromosom	5963264	5964403	-	1140	910.0	14	3037	13	2977	
QEN71_RS26720	NAD(P)(+) transhydrogenase (Re/Si-specific) subur	QEN71_26715	paras_005876	protein-codi	NZ_CP1252	chromosom	5964630	5966081	-	1452	0	51	11764	33	8614	
QEN71_RS26725	NAD(P) transhydrogenase subunit alpha	QEN71_26720	paras_005877	protein-codi	NZ_CP1252	chromosom	5966082	5966405	-	324	0	11	2790	5	619	
QEN71_RS26730	Re/Si-specific NAD(P)(+) transhydrogenase subunit	QEN71_26725	paras_005878	protein-codi	NZ_CP1252	chromosom	5966425	5967567	-	1143	0	43	9278	35	6891	
QEN71_RS26735	NUDIX hydrolase	QEN71_26730	paras_005879	protein-codi	NZ_CP1252	chromosom	5967858	5968355	+	498	0	11	3691	10	3652	
QEN71_RS26740	tRNA 2-thiouridine(34) synthase MnmA	QEN71_26735	paras_005880	protein-codi	NZ_CP1252	chromosom	5968405	5969559	+	1155	0	0	0	0	0	TRUE

QEN71_RS26745	FMN-binding glutamate synthase family protein	QEN71_26740	paras_005881	protein-codi	NZ_CP1252	chromosom	5969764	5971377	+	1614	0	28	4445	17	2794	
QEN71_RS26750	M48 family metallopeptidase	QEN71_26745	paras_005882	protein-codi	NZ_CP1252	chromosom	5971471	5972226	-	756	0	18	3663	14	3048	
QEN71_RS26755	glutathione S-transferase N-terminal domain-contai	QEN71_26750	paras_005883	protein-codi	NZ_CP1252	chromosom	5972423	5973031	-	609	0	24	8029	16	5333	
QEN71_RS26760	aminopeptidase P N-terminal domain-containing pro	QEN71_26755	paras_005884	protein-codi	NZ_CP1252	chromosom	5973362	5974753	+	1392	0	41	14787	35	13066	
QEN71_RS26765	UbiH/UbiF/VisC/COQ6 family ubiquinone biosynthe	QEN71_26760	paras_005885	protein-codi	NZ_CP1252	chromosom	5974777	5975985	+	1209	0	0	0	0	0	TRUE
QEN71_RS26770	tRNA dihydrouridine synthase DusB	QEN71_26765	paras_005886	protein-codi	NZ_CP1252	chromosom	5976191	5977255	+	1061	0	22	4343	18	3398	
QEN71_RS26775	Fis family transcriptional regulator	QEN71_26770	paras_005887	protein-codi	NZ_CP1252	chromosom	5977252	5977485	+	230	0	12	1107	8	956	
QEN71_RS26780	bifunctional phosphoribosylaminoimidazolecarboxar	QEN71_26775	paras_005888	protein-codi	NZ_CP1252	chromosom	5977530	5979095	+	1566	0	28	2304	24	2166	
QEN71_RS26785	crossover junction endodeoxyribonuclease RuvC	QEN71_26780	paras_005889	protein-codi	NZ_CP1252	chromosom	5979221	5979763	+	543	0	5	197	3	31	
QEN71_RS26790	LysE family translocator	QEN71_26785	paras_005890	protein-codi	NZ_CP1252	chromosom	5979776	5980408	-	633	0	22	8114	16	7375	
QEN71_RS26795	Holliday junction branch migration protein RuvA	QEN71_26790	paras_005891	protein-codi	NZ_CP1252	chromosom	5980574	5981155	+	582	0	5	81	5	81	
QEN71_RS26800	Holliday junction branch migration DNA helicase Ru	QEN71_26795	paras_005892	protein-codi	NZ_CP1252	chromosom	5981265	5982329	+	1065	0	7	1258	4	16	
QEN71_RS26805	histidine phosphatase family protein	QEN71_26800	paras_005893	protein-codi	NZ_CP1252	chromosom	5982385	5983056	-	672	0	13	3685	13	3685	
QEN71_RS26810	D-aminoacyl-tRNA deacylase	QEN71_26805	paras_005894	protein-codi	NZ_CP1252	chromosom	5983081	5983539	-	459	0	17	5315	11	2541	
QEN71_RS26815	tyrosine--tRNA ligase	QEN71_26810	paras_005895	protein-codi	NZ_CP1252	chromosom	5983583	5984830	-	1248	0	0	0	0	0	TRUE
QEN71_RS26820	anhydro-N-acetylmuramic acid kinase	QEN71_26815	paras_005896	protein-codi	NZ_CP1252	chromosom	5985098	5986189	+	1092	0	26	21222	22	20510	
QEN71_RS26825	iron-sulfur cluster insertion protein ErpA	QEN71_26820	paras_005897	protein-codi	NZ_CP1252	chromosom	5986284	5986649	-	366	0	5	299	2	19	
QEN71_RS26830	30S ribosomal protein S9	QEN71_26825	paras_005898	protein-codi	NZ_CP1252	chromosom	5986913	5987305	-	393	0	1	13	1	13	TRUE
QEN71_RS26835	50S ribosomal protein L13	QEN71_26830	paras_005899	protein-codi	NZ_CP1252	chromosom	5987317	5987745	-	429	0	0	0	0	0	TRUE
QEN71_RS26840	OsmC family protein	QEN71_26835	paras_005900	protein-codi	NZ_CP1252	chromosom	5988349	5988771	+	423	0	5	2189	5	2189	
QEN71_RS26850	DUF3025 domain-containing protein	partial;pseudo;QEN71_26845		pseudogene	NZ_CP1252	chromosom	5989355	5989975	-	621	0	8	1410	8	1410	
QEN71_RS26855	dihydroorotase	QEN71_26850	paras_005903	protein-codi	NZ_CP1252	chromosom	5990150	5991214	-	1065	0	30	4201	25	4085	
QEN71_RS26860	class II glutamine amidotransferase	QEN71_26855	paras_005904	protein-codi	NZ_CP1252	chromosom	5991431	5992336	+	906	0	22	4530	17	3650	
QEN71_RS26865	hypothetical protein	QEN71_26860	paras_005905	protein-codi	NZ_CP1252	chromosom	5992342	5992791	-	450	0	15	2185	13	1734	
QEN71_RS26870	amino acid ABC transporter ATP-binding protein	QEN71_26865	paras_005906	protein-codi	NZ_CP1252	chromosom	5993046	5993771	-	722	0	8	1209	4	364	
QEN71_RS26875	glutamate/aspartate ABC transporter permease GltI	QEN71_26870	paras_005907	protein-codi	NZ_CP1252	chromosom	5993768	5994445	-	673	0	21	4768	19	4086	
QEN71_RS26880	amino acid ABC transporter permease	QEN71_26875	paras_005908	protein-codi	NZ_CP1252	chromosom	5994445	5995185	-	740	0	29	7685	19	5109	
QEN71_RS26885	glutamate/aspartate ABC transporter substrate-bind	QEN71_26880	paras_005909	protein-codi	NZ_CP1252	chromosom	5995275	5996168	-	894	0	20	3821	12	2181	
QEN71_RS26890	Glu/Leu/Phe/Val dehydrogenase	QEN71_26885	paras_005910	protein-codi	NZ_CP1252	chromosom	5996536	5997828	-	1293	0	18	758	9	299	
QEN71_RS26895	LysR substrate-binding domain-containing protein	QEN71_26890	paras_005911	protein-codi	NZ_CP1252	chromosom	5997992	5998948	-	957	0	24	1142	21	955	
QEN71_RS26900	adenylosuccinate lyase	QEN71_26895	paras_005912	protein-codi	NZ_CP1252	chromosom	5999102	6000490	+	1389	0	3	571	0	0	TRUE
QEN71_RS26905	gluconokinase	QEN71_26900	paras_005913	protein-codi	NZ_CP1252	chromosom	6001122	6001616	-	495	0	13	2444	11	2356	
QEN71_RS26910	GntP family permease	QEN71_26905	paras_005914	protein-codi	NZ_CP1252	chromosom	6001627	6002988	-	1362	0	21	5608	14	3614	
QEN71_RS26915	bifunctional 4-hydroxy-2-oxoglutarate aldolase/2-de	QEN71_26910	paras_005915	protein-codi	NZ_CP1252	chromosom	6003246	6003887	-	642	0	0	0	0	0	TRUE
QEN71_RS26920	phosphogluconate dehydratase	QEN71_26915	paras_005916	protein-codi	NZ_CP1252	chromosom	6003918	6005822	-	1905	0	39	16087	35	14541	
QEN71_RS26925	MurR/RpiR family transcriptional regulator	QEN71_26920	paras_005917	protein-codi	NZ_CP1252	chromosom	6006119	6006958	+	840	0	18	6052	14	3897	
QEN71_RS26930	hypothetical protein	partial;pseudo;QEN71_26925		protein-codi	NZ_CP1252	chromosom	6007187	6007519	-	333	0	11	1817	8	798	
QEN71_RS26935	CopD family protein	QEN71_26930	paras_005919	protein-codi	NZ_CP1252	chromosom	6007758	6008174	-	417	0	0	0	0	0	TRUE
QEN71_RS26940	glutamate-5-semialdehyde dehydrogenase	QEN71_26935	paras_005920	protein-codi	NZ_CP1252	chromosom	6008209	6009480	-	1272	0	30	8301	23	6802	
QEN71_RS26945	DNA polymerase III subunit delta	QEN71_26940	paras_005921	protein-codi	NZ_CP1252	chromosom	6009701	6010825	-	1125	0	2	139	0	0	TRUE
QEN71_RS26950	LPS assembly lipoprotein LptE	QEN71_26945	paras_005922	protein-codi	NZ_CP1252	chromosom	6010877	6011428	-	552	0	12	1145	12	1145	
QEN71_RS26955	leucine--tRNA ligase	QEN71_26950	paras_005923	protein-codi	NZ_CP1252	chromosom	6011471	6014065	-	2595	0	2	9	2	9	TRUE
QEN71_RS26960	biopolymer transporter ExbD	QEN71_26955	paras_005924	protein-codi	NZ_CP1252	chromosom	6014292	6014765	-	474	0	8	609	6	424	
QEN71_RS26965	MotA/TolQ/ExbB proton channel family protein	QEN71_26960	paras_005925	protein-codi	NZ_CP1252	chromosom	6014809	6015546	-	738	0	24	10853	16	7265	
QEN71_RS26970	4-hydroxy-tetrahydrodipicolinate reductase	QEN71_26965	paras_005926	protein-codi	NZ_CP1252	chromosom	6015611	6016408	-	798	0	3	400	1	9	TRUE
QEN71_RS26975	outer membrane protein assembly factor BamE	QEN71_26970	paras_005927	protein-codi	NZ_CP1252	chromosom	6016573	6017454	-	882	0	3	602	1	20	
QEN71_RS26980	ferric iron uptake transcriptional regulator	QEN71_26975	paras_005928	protein-codi	NZ_CP1252	chromosom	6017566	6017994	+	429	0	0	0	0	0	TRUE
QEN71_RS26985	ureidoglycolate lyase	QEN71_26980	paras_005929	protein-codi	NZ_CP1252	chromosom	6018110	6018622	-	513	0	8	1910	8	1910	
QEN71_RS26990	allantoicase	QEN71_26985	paras_005930	protein-codi	NZ_CP1252	chromosom	6018653	6019663	-	1011	0	30	7411	21	6235	
QEN71_RS26995	C4-dicarboxylate transporter DctA	QEN71_26990	paras_005931	protein-codi	NZ_CP1252	chromosom	6019792	6021117	-	1326	0	26	5737	26	5737	
QEN71_RS27000	FadR/GntR family transcriptional regulator	QEN71_26995	paras_005932	protein-codi	NZ_CP1252	chromosom	6021370	6022062	+	693	0	37	13167	31	12136	
QEN71_RS27005	type I glyceraldehyde-3-phosphate dehydrogenase	QEN71_27000	paras_005933	protein-codi	NZ_CP1252	chromosom	6022249	6023259	-	1011	0	15	854	8	322	
QEN71_RS27010	transketolase	QEN71_27005	paras_005934	protein-codi	NZ_CP1252	chromosom	6023365	6025383	-	2019	0	8	334	4	46	TRUE
QEN71_RS27015	glyoxalase/bleomycin resistance/extradiol dioxygen	QEN71_27010	paras_005935	protein-codi	NZ_CP1252	chromosom	6026008	6026421	+	414	0	12	7488	11	7411	
QEN71_RS27020	16S rRNA (uracil(1498)-N(3))-methyltransferase	QEN71_27015	paras_005936	protein-codi	NZ_CP1252	chromosom	6026441	6027181	+	741	0	11	946	7	431	
QEN71_RS27025	barstar family protein	QEN71_27020	paras_005937	protein-codi	NZ_CP1252	chromosom	6027279	6027839	-	561	0	15	5279	11	4869	
QEN71_RS27030	ribonuclease	QEN71_27025	paras_005938	protein-codi	NZ_CP1252	chromosom	6027856	6028254	-	399	0	27	5907	22	5286	
QEN71_RS27035	group II intron reverse transcriptase/maturase	QEN71_27030	paras_005939	protein-codi	NZ_CP1252	chromosom	6028382	6030106	-	1725	0	92	41500	75	34853	
QEN71_RS27040	NADP-dependent malic enzyme	QEN71_27035	paras_005940	protein-codi	NZ_CP1252	chromosom	6031495	6033789	-	2295	0	9	751	4	13	TRUE
QEN71_RS27045	thiamine-phosphate kinase	QEN71_27040	paras_005941	protein-codi	NZ_CP1252	chromosom	6034040	6035035	+	985	0	4	77	3	21	
QEN71_RS27050	phosphatidylglycerophosphatase A	QEN71_27045	paras_005942	protein-codi	NZ_CP1252	chromosom	6035025	6035666	+	631	0	2	10	2	10	
QEN71_RS27055	CinA family protein	QEN71_27050	paras_005943	protein-codi	NZ_CP1252	chromosom	6035700	6036200	+	501	0	6	3570	6	3570	

QEN71_RS27060	cupin domain-containing protein	QEN71_27055	paras_005944	protein-codi	NZ_CP1252	chromosom	6036209	6036721	+	513	0	18	8069	16	7527	
QEN71_RS27065	orotidine-5'-phosphate decarboxylase	QEN71_27060	paras_005945	protein-codi	NZ_CP1252	chromosom	6036752	6037567	-	816	0	24	2944	22	2803	
QEN71_RS27070	aldose 1-epimerase	QEN71_27065	paras_005946	protein-codi	NZ_CP1252	chromosom	6037693	6038751	-	1059	0	22	6995	21	6810	
QEN71_RS27075	SMP-30/gluconolactonase/LRE family protein	QEN71_27070	paras_005947	protein-codi	NZ_CP1252	chromosom	6038960	6039880	-	917	0	32	7522	28	6938	
QEN71_RS27080	SDR family oxidoreductase	QEN71_27075	paras_005948	protein-codi	NZ_CP1252	chromosom	6039877	6040677	-	797	0	11	1962	9	1823	
QEN71_RS27085	L-arabinose ABC transporter permease AraH	QEN71_27080	paras_005949	protein-codi	NZ_CP1252	chromosom	6040718	6041734	-	1017	0	21	5392	17	4177	
QEN71_RS27090	L-arabinose ABC transporter ATP-binding protein A	QEN71_27085	paras_005950	protein-codi	NZ_CP1252	chromosom	6041839	6043353	-	1515	0	28	8938	24	8137	
QEN71_RS27095	arabinose ABC transporter substrate-binding protei	QEN71_27090	paras_005951	protein-codi	NZ_CP1252	chromosom	6043440	6044438	-	999	0	15	3212	13	2951	
QEN71_RS27100	SDR family oxidoreductase	QEN71_27095	paras_005952	protein-codi	NZ_CP1252	chromosom	6044653	6045438	-	782	0	6	881	3	496	
QEN71_RS27105	2-dehydro-3-deoxy-6-phosphogalactonate aldolase	QEN71_27100	paras_005953	protein-codi	NZ_CP1252	chromosom	6045435	6046115	-	677	0	6	897	4	854	
QEN71_RS27110	2-dehydro-3-deoxygalactonokinase	QEN71_27105	paras_005954	protein-codi	NZ_CP1252	chromosom	6046181	6047221	-	1037	0	13	4195	11	4070	
QEN71_RS27115	IclR family transcriptional regulator	QEN71_27110	paras_005955	protein-codi	NZ_CP1252	chromosom	6047218	6048171	-	950	0	14	5014	13	5008	
QEN71_RS27120	outer membrane beta-barrel protein	QEN71_27115	paras_005956	protein-codi	NZ_CP1252	chromosom	6048704	6049219	+	516	0	14	4947	10	4129	
QEN71_RS27125	monofunctional biosynthetic peptidoglycan transgly	QEN71_27120	paras_005957	protein-codi	NZ_CP1252	chromosom	6049302	6050030	-	729	0	27	9111	24	8270	
QEN71_RS27130	shikimate dehydrogenase	QEN71_27125	paras_005958	protein-codi	NZ_CP1252	chromosom	6050089	6050961	-	869	0	17	4823	13	3410	
QEN71_RS27135	RNB domain-containing ribonuclease	QEN71_27130	paras_005959	protein-codi	NZ_CP1252	chromosom	6050958	6053048	-	2087	0	13	89	12	86	
QEN71_RS27140	YqiA/YcfP family alpha/beta fold hydrolase	QEN71_27135	paras_005960	protein-codi	NZ_CP1252	chromosom	6053189	6053791	-	599	0	18	7064	16	6076	
QEN71_RS27145	UDP-N-acetylmuramate:L-alanyl-gamma-D-glutamy	QEN71_27140	paras_005961	protein-codi	NZ_CP1252	chromosom	6053788	6055197	-	1406	0	25	4726	21	3808	
QEN71_RS27150	UDP-N-acetylmuramate--alanine ligase	QEN71_27145	paras_005962	protein-codi	NZ_CP1252	chromosom	6055402	6056055	+	654	0	19	8162	19	8162	
QEN71_RS27155	TlpA disulfide reductase family protein	QEN71_27150	paras_005963	protein-codi	NZ_CP1252	chromosom	6056082	6056630	+	549	0	20	4736	14	2817	
QEN71_RS27160	type II 3-dehydroquinate dehydratase	QEN71_27155	paras_005964	protein-codi	NZ_CP1252	chromosom	6056825	6057289	+	465	0	10	4754	10	4754	
QEN71_RS27165	acetyl-CoA carboxylase biotin carboxyl carrier prote	QEN71_27160	paras_005965	protein-codi	NZ_CP1252	chromosom	6057358	6057825	+	468	0	0	0	0	0	TRUE
QEN71_RS27170	acetyl-CoA carboxylase biotin carboxylase subunit	QEN71_27165	paras_005966	protein-codi	NZ_CP1252	chromosom	6057967	6059334	+	1368	0	2	719	0	0	TRUE
QEN71_RS27175	50S ribosomal protein L11 methyltransferase	QEN71_27170	paras_005967	protein-codi	NZ_CP1252	chromosom	6059338	6060240	+	903	0	19	4976	10	2256	
QEN71_RS27180	DUF3426 domain-containing protein	QEN71_27175	paras_005968	protein-codi	NZ_CP1252	chromosom	6060322	6062085	+	1764	0	27	6981	11	1568	
QEN71_RS27185	thiol peroxidase	QEN71_27180	paras_005969	protein-codi	NZ_CP1252	chromosom	6062179	6062679	+	501	0	6	1857	2	172	
QEN71_RS27190	carbohydrate kinase family protein	QEN71_27185	paras_005970	protein-codi	NZ_CP1252	chromosom	6062790	6063734	+	945	0	24	4139	16	3213	
QEN71_RS27195	glycine zipper 2TM domain-containing protein	QEN71_27190	paras_005971	protein-codi	NZ_CP1252	chromosom	6063830	6064318	+	489	0	8	3164	5	2674	
QEN71_RS27200	histone H1-like DNA-binding protein	QEN71_27195	paras_005972	protein-codi	NZ_CP1252	chromosom	6064554	6065165	-	612	0	4	450	2	225	
QEN71_RS27205	hypothetical protein	QEN71_27200	paras_005973	protein-codi	NZ_CP1252	chromosom	6065196	6065522	-	327	0	19	2602	15	1971	
QEN71_RS27210	ribonucleotide-diphosphate reductase subunit beta	QEN71_27205	paras_005974	protein-codi	NZ_CP1252	chromosom	6065602	6066825	-	1224	0	1	2	0	0	TRUE
QEN71_RS27215	hypothetical protein	QEN71_27210	paras_005975	protein-codi	NZ_CP1252	chromosom	6066888	6067331	-	444	0	6	3308	3	806	
QEN71_RS27220	ribonucleoside-diphosphate reductase subunit alpha	QEN71_27215	paras_005976	protein-codi	NZ_CP1252	chromosom	6067433	6070432	-	3000	0	2	59	1	2	TRUE
QEN71_RS27225	1,6-anhydro-N-acetylmuramyl-L-alanine amidase Ar	QEN71_27220	paras_005977	protein-codi	NZ_CP1252	chromosom	6070924	6071544	-	617	0	20	6123	15	4724	
QEN71_RS27230	PP0621 family protein	QEN71_27225	paras_005978	protein-codi	NZ_CP1252	chromosom	6071541	6071885	-	341	0	2	27	1	2	
QEN71_RS27235	cytochrome c biogenesis protein CcsA	QEN71_27230	paras_005979	protein-codi	NZ_CP1252	chromosom	6071911	6072852	-	942	0	22	10495	10	4568	
QEN71_RS27240	signal recognition particle protein	QEN71_27235	paras_005980	protein-codi	NZ_CP1252	chromosom	6072932	6074299	+	1368	0	2	203	0	0	TRUE
QEN71_RS27245	hypoxanthine-guanine phosphoribosyltransferase	QEN71_27240	paras_005981	protein-codi	NZ_CP1252	chromosom	6074450	6075001	+	552	0	10	3628	7	3242	
QEN71_RS27250	MarC family protein	QEN71_27245	paras_005982	protein-codi	NZ_CP1252	chromosom	6075073	6075678	-	606	0	2	869	0	0	
QEN71_RS27255	proline--tRNA ligase	QEN71_27250	paras_005983	protein-codi	NZ_CP1252	chromosom	6075691	6077427	-	1737	0	0	0	0	0	TRUE
QEN71_RS27260	RNA pyrophosphohydrolase	QEN71_27255	paras_005984	protein-codi	NZ_CP1252	chromosom	6077707	6078399	+	693	0	23	11451	21	10567	
QEN71_RS27265	CNP1-like family protein	QEN71_27260	paras_005985	protein-codi	NZ_CP1252	chromosom	6078475	6079068	+	594	0	37	17632	28	14560	
QEN71_RS27270	glutamate 5-kinase	QEN71_27265	paras_005986	protein-codi	NZ_CP1252	chromosom	6079152	6080270	-	1119	0	22	7455	16	6004	
QEN71_RS27275	GTPase ObgE	QEN71_27270	paras_005987	protein-codi	NZ_CP1252	chromosom	6080347	6081465	-	1119	0	7	1764	4	371	
QEN71_RS27280	50S ribosomal protein L27	QEN71_27275	paras_005988	protein-codi	NZ_CP1252	chromosom	6081699	6081959	-	261	0	0	0	0	0	
QEN71_RS27285	50S ribosomal protein L21	QEN71_27280	paras_005989	protein-codi	NZ_CP1252	chromosom	6081984	6082295	-	312	0	0	0	0	0	TRUE
QEN71_RS27290	polyprenyl synthetase family protein	QEN71_27285	paras_005990	protein-codi	NZ_CP1252	chromosom	6082534	6083526	+	993	0	2	167	0	0	TRUE
QEN71_RS27295	tRNA-Pro	QEN71_27290		tRNA	NZ_CP1252	chromosom	6083575	6083651	+	77	0	4	60	3	41	
QEN71_RS27300	HlyC/CorC family transporter	QEN71_27295	paras_005992	protein-codi	NZ_CP1252	chromosom	6083831	6085135	+	1305	0	25	6060	19	4760	
QEN71_RS27305	ATPase, T2SS/T4P/T4SS family	QEN71_27300	paras_005993	protein-codi	NZ_CP1252	chromosom	6085232	6086485	+	1250	0	14	4962	12	3840	
QEN71_RS27310	type II secretion system F family protein	QEN71_27305	paras_005994	protein-codi	NZ_CP1252	chromosom	6086482	6087702	+	1217	0	39	11654	28	6844	
QEN71_RS27315	A24 family peptidase	QEN71_27310	paras_005995	protein-codi	NZ_CP1252	chromosom	6087733	6088662	+	930	0	20	4103	15	2839	
QEN71_RS27320	dephospho-CoA kinase	QEN71_27315	paras_005996	protein-codi	NZ_CP1252	chromosom	6088666	6089268	+	603	0	2	626	0	0	
QEN71_RS27325	cell division protein ZapD	QEN71_27320	paras_005997	protein-codi	NZ_CP1252	chromosom	6089464	6090219	+	756	0	21	9989	15	8737	
QEN71_RS27330	DNA gyrase inhibitor YacG	QEN71_27325	paras_005998	protein-codi	NZ_CP1252	chromosom	6090279	6090473	+	195	0	6	2068	2	268	
QEN71_RS27335	NUDIX domain-containing protein	QEN71_27330	paras_005999	protein-codi	NZ_CP1252	chromosom	6090502	6090942	-	437	0	11	3273	11	3273	
QEN71_RS27340	ATP-binding protein	QEN71_27335	paras_006000	protein-codi	NZ_CP1252	chromosom	6090939	6091808	-	866	0	20	6739	18	6389	
QEN71_RS27345	bifunctional glutamate N-acetyltransferase/amino-a	QEN71_27340	paras_006001	protein-codi	NZ_CP1252	chromosom	6091848	6093089	-	1242	0	21	1396	16	1138	
QEN71_RS27350	preprotein translocase subunit SecA	QEN71_27345	paras_006002	protein-codi	NZ_CP1252	chromosom	6093239	6096049	-	2811	0	6	952	2	17	TRUE
QEN71_RS27355	DciA family protein	QEN71_27350	paras_006003	protein-codi	NZ_CP1252	chromosom	6096358	6096861	+	504	0	5	2739	1	10	
QEN71_RS27360	UDP-3-O-acyl-N-acetylglucosamine deacetylase	QEN71_27355	paras_006004	protein-codi	NZ_CP1252	chromosom	6096955	6097872	-	918	0	2	2198	0	0	
QEN71_RS27365	peroxiredoxin	QEN71_27360	paras_006005	protein-codi	NZ_CP1252	chromosom	6098144	6098653	-	510	0	10	1835	5	144	

QEN71_RS27370	cell division protein FtsZ	QEN71_27365	paras_006006	protein-codi	NZ_CP1252	chromosom	6098892	6100085	-	1194	0	2	804	0	0	
QEN71_RS27375	cell division protein FtsA	QEN71_27370	paras_006007	protein-codi	NZ_CP1252	chromosom	6100209	6101441	-	1233	0	1	67	0	0	TRUE
QEN71_RS27380	cell division protein FtsQ/DivIB	QEN71_27375	paras_006008	protein-codi	NZ_CP1252	chromosom	6101469	6102221	-	753	0	2	87	1	4	TRUE
QEN71_RS27385	D-alanine--D-alanine ligase	QEN71_27380	paras_006009	protein-codi	NZ_CP1252	chromosom	6102237	6103178	-	938	0	1	17	0	0	TRUE
QEN71_RS27390	UDP-N-acetylmuramate--L-alanine ligase	QEN71_27385	paras_006010	protein-codi	NZ_CP1252	chromosom	6103175	6104569	-	1387	0	1	3	1	3	TRUE
QEN71_RS27395	undecaprenyldiphospho-muramoylpentapeptide bet	QEN71_27390	paras_006011	protein-codi	NZ_CP1252	chromosom	6104566	6105684	-	1111	0	0	0	0	0	TRUE
QEN71_RS27400	putative lipid II flippase FtsW	QEN71_27395	paras_006012	protein-codi	NZ_CP1252	chromosom	6105681	6106952	-	1264	0	1	373	0	0	TRUE
QEN71_RS27405	UDP-N-acetylmuramoyl-L-alanine--D-glutamate liga	QEN71_27400	paras_006013	protein-codi	NZ_CP1252	chromosom	6106949	6108463	-	1511	0	1	347	0	0	TRUE
QEN71_RS27410	phospho-N-acetylmuramoyl-pentapeptide- transfera	QEN71_27405	paras_006014	protein-codi	NZ_CP1252	chromosom	6108511	6109680	-	1170	0	0	0	0	0	TRUE
QEN71_RS27415	UDP-N-acetylmuramoyl-tripeptide--D-alanyl-D- alan	QEN71_27410	paras_006015	protein-codi	NZ_CP1252	chromosom	6109698	6111113	-	1412	0	3	175	1	8	TRUE
QEN71_RS27420	UDP-N-acetylmuramoyl-L-alanyl-D-glutamate--2, 6-	QEN71_27415	paras_006016	protein-codi	NZ_CP1252	chromosom	6111110	6112648	-	1531	0	0	0	0	0	TRUE
QEN71_RS27425	penicillin-binding protein 2	QEN71_27420	paras_006017	protein-codi	NZ_CP1252	chromosom	6112645	6114519	-	1867	0	26	6340	24	5993	
QEN71_RS27430	cell division protein FtsL	QEN71_27425	paras_006018	protein-codi	NZ_CP1252	chromosom	6114516	6114866	-	343	0	0	0	0	0	TRUE
QEN71_RS27435	16S rRNA (cytosine(1402)-N(4))-methyltransferase	QEN71_27430	paras_006019	protein-codi	NZ_CP1252	chromosom	6114863	6115804	-	938	0	4	42	2	12	TRUE
QEN71_RS27440	division/cell wall cluster transcriptional repressor Mr	QEN71_27435	paras_006020	protein-codi	NZ_CP1252	chromosom	6115832	6116260	-	429	0	4	597	4	597	
QEN71_RS27445	2-polyprenyl-3-methyl-6-methoxy-1,4-benzoquinone	QEN71_27440	paras_006021	protein-codi	NZ_CP1252	chromosom	6116663	6117292	-	630	0	2	107	0	0	TRUE
QEN71_RS27450	porin	QEN71_27445	paras_006022	protein-codi	NZ_CP1252	chromosom	6117584	6118744	+	1161	0	68	27252	58	22889	
QEN71_RS27455	long-chain fatty acid--CoA ligase	QEN71_27450	paras_006023	protein-codi	NZ_CP1252	chromosom	6118950	6120623	-	1674	0	47	17123	36	12777	
QEN71_RS27460	molybdopterin oxidoreductase family protein	QEN71_27455	paras_006024	protein-codi	NZ_CP1252	chromosom	6120784	6122862	-	2079	0	55	18211	52	17294	
QEN71_RS27465	M20 aminoacylase family protein	QEN71_27460	paras_006025	protein-codi	NZ_CP1252	chromosom	6123032	6124225	+	1194	0	44	14400	29	10669	
QEN71_RS27470	5S ribosomal RNA	QEN71_27465		rRNA	NZ_CP1252	chromosom	6124502	6124615	-	114	225.0	4	27	2	5	
QEN71_RS27475	23S ribosomal RNA	QEN71_27470		rRNA	NZ_CP1252	chromosom	6124814	6127694	-	2881	5656.0	77	2948	61	2809	
QEN71_RS27480	tRNA-Ala	QEN71_27475		tRNA	NZ_CP1252	chromosom	6128000	6128075	-	76	150.0	1	3	0	0	
QEN71_RS27485	tRNA-Ile	QEN71_27480		tRNA	NZ_CP1252	chromosom	6128136	6128212	-	77	152.0	1	4	1	4	
QEN71_RS27490	16S ribosomal RNA	QEN71_27485		rRNA	NZ_CP1252	chromosom	6128277	6129807	-	1531	3015.0	32	4556	27	4512	
QEN71_RS27495	enoyl-CoA hydratase	QEN71_27490	paras_006031	protein-codi	NZ_CP1252	chromosom	6130266	6131042	-	777	0	34	16527	27	12899	
QEN71_RS27500	phenylacetic acid degradation protein PaaN	QEN71_27495	paras_006032	protein-codi	NZ_CP1252	chromosom	6131179	6132870	+	1692	0	43	19771	33	17661	
QEN71_RS27505	3-oxoadipyl-CoA thiolase	QEN71_27500	paras_006033	protein-codi	NZ_CP1252	chromosom	6133009	6134211	+	1203	0	24	7822	17	5724	
QEN71_RS27510	2-(1,2-epoxy-1,2-dihydrophenyl)acetyl-CoA isomera	QEN71_27505	paras_006034	protein-codi	NZ_CP1252	chromosom	6134254	6135045	+	792	0	10	2092	2	254	
QEN71_RS27515	hydroxyphenylacetyl-CoA thioesterase PaaI	QEN71_27510	paras_006035	protein-codi	NZ_CP1252	chromosom	6135047	6135508	+	462	0	12	8996	12	8996	
QEN71_RS27520	phenylacetate--CoA ligase PaaK	QEN71_27515	paras_006036	protein-codi	NZ_CP1252	chromosom	6135587	6136891	+	1305	0	39	16883	27	15180	
QEN71_RS27525	MltA domain-containing protein	QEN71_27520	paras_006037	protein-codi	NZ_CP1252	chromosom	6136994	6138226	-	1153	0	8	75	6	59	
QEN71_RS27530	hypothetical protein	QEN71_27525	paras_006038	protein-codi	NZ_CP1252	chromosom	6138147	6138320	+	94	0	3	630	0	0	
QEN71_RS27535	Co2+/Mg2+ efflux protein ApaG	QEN71_27530	paras_006039	protein-codi	NZ_CP1252	chromosom	6138345	6138719	-	375	0	21	8422	21	8422	
QEN71_RS27540	ribulose-phosphate 3-epimerase	QEN71_27535	paras_006040	protein-codi	NZ_CP1252	chromosom	6138987	6139670	+	684	0	10	459	8	415	
QEN71_RS27545	phosphoglycolate phosphatase	QEN71_27540	paras_006041	protein-codi	NZ_CP1252	chromosom	6139672	6140400	+	729	0	24	14255	15	6982	
QEN71_RS27550	anthranilate synthase component I	QEN71_27545	paras_006042	protein-codi	NZ_CP1252	chromosom	6140818	6142311	+	1494	0	53	20161	47	17286	
QEN71_RS27555	aminodeoxychorismate/anthranilate synthase comp	QEN71_27550	paras_006043	protein-codi	NZ_CP1252	chromosom	6142324	6142911	+	588	0	18	4029	9	699	
QEN71_RS27560	anthranilate phosphoribosyltransferase	QEN71_27555	paras_006044	protein-codi	NZ_CP1252	chromosom	6142929	6143960	+	1032	0	19	5261	17	4466	
QEN71_RS27565	indole-3-glycerol phosphate synthase TrpC	QEN71_27560	paras_006045	protein-codi	NZ_CP1252	chromosom	6143979	6144764	+	786	0	12	2293	10	1928	
QEN71_RS27570	CYTH domain-containing protein	QEN71_27565	paras_006046	protein-codi	NZ_CP1252	chromosom	6144795	6145430	+	636	0	9	4189	7	4049	
QEN71_RS27575	uracil-DNA glycosylase	QEN71_27570	paras_006047	protein-codi	NZ_CP1252	chromosom	6145521	6146336	+	816	0	3	13	3	13	
QEN71_RS27580	NAD(P)H-dependent oxidoreductase	QEN71_27575	paras_006048	protein-codi	NZ_CP1252	chromosom	6146429	6147025	-	597	0	22	7293	20	7207	
QEN71_RS27585	PDZ domain-containing protein	QEN71_27580	paras_006049	protein-codi	NZ_CP1252	chromosom	6147168	6148958	-	1791	0	60	20804	51	15807	
QEN71_RS27590	DsbC family protein	QEN71_27585	paras_006050	protein-codi	NZ_CP1252	chromosom	6149093	6149818	-	726	0	15	5111	9	3109	
QEN71_RS27595	UbiH/UbiF family hydroxylase	QEN71_27590	paras_006051	protein-codi	NZ_CP1252	chromosom	6149992	6151164	-	1173	0	0	0	0	0	TRUE
QEN71_RS27600	redox-regulated ATPase YchF	QEN71_27595	paras_006052	protein-codi	NZ_CP1252	chromosom	6151576	6152670	+	1095	0	21	4715	13	2759	
QEN71_RS27605	sn-glycerol-3-phosphate ABC transporter substrate-	QEN71_27600	paras_006053	protein-codi	NZ_CP1252	chromosom	6152941	6154266	+	1326	0	55	13608	46	11736	
QEN71_RS27610	sn-glycerol-3-phosphate ABC transporter permease	QEN71_27605	paras_006054	protein-codi	NZ_CP1252	chromosom	6154355	6155239	+	881	0	26	5301	18	3632	
QEN71_RS27615	sn-glycerol-3-phosphate ABC transporter permease	QEN71_27610	paras_006055	protein-codi	NZ_CP1252	chromosom	6155236	6156081	+	842	0	14	3149	6	2490	
QEN71_RS27620	sn-glycerol-3-phosphate import ATP-binding protein	QEN71_27615	paras_006056	protein-codi	NZ_CP1252	chromosom	6156094	6157185	+	1092	0	28	9317	23	8032	
QEN71_RS27625	energy-dependent translational throttle protein EttA	QEN71_27620	paras_006057	protein-codi	NZ_CP1252	chromosom	6157898	6159565	+	1668	0	43	9926	28	5517	
QEN71_RS27630	DUF748 domain-containing protein	QEN71_27625	paras_006058	protein-codi	NZ_CP1252	chromosom	6159777	6160916	+	1140	0	18	5591	13	4402	
QEN71_RS27635	HAD family phosphatase	QEN71_27630	paras_006059	protein-codi	NZ_CP1252	chromosom	6160944	6161567	-	620	0	11	2441	9	1264	
QEN71_RS27640	cysteine hydrolase family protein	QEN71_27635	paras_006060	protein-codi	NZ_CP1252	chromosom	6161564	6162115	-	548	0	13	5260	11	3617	
QEN71_RS27645	aminopeptidase P family protein	QEN71_27640	paras_006061	protein-codi	NZ_CP1252	chromosom	6162231	6164045	-	1815	0	32	9385	20	6350	
QEN71_RS27650	hypothetical protein	QEN71_27645	paras_006062	protein-codi	NZ_CP1252	chromosom	6164065	6164319	-	254	0	3	343	2	328	
QEN71_RS27655	hypothetical protein	QEN71_27650	paras_006063	protein-codi	NZ_CP1252	chromosom	6164319	6164462	-	143	0	13	3257	9	2158	
QEN71_RS27660	response regulator transcription factor	QEN71_27655	paras_006064	protein-codi	NZ_CP1252	chromosom	6164507	6165253	+	747	0	30	4317	18	2680	
QEN71_RS27665	glutamyl-tRNA reductase	QEN71_27660	paras_006065	protein-codi	NZ_CP1252	chromosom	6165309	6166598	+	1290	0	2	18	0	0	TRUE
QEN71_RS27670	peptide chain release factor 1	QEN71_27665	paras_006066	protein-codi	NZ_CP1252	chromosom	6166731	6167813	+	1083	0	0	0	0	0	TRUE
QEN71_RS27675	peptide chain release factor N(5)-glutamine methylt	QEN71_27670	paras_006067	protein-codi	NZ_CP1252	chromosom	6167823	6168671	+	849	0	13	934	9	703	

QEN71_RS27680	Grx4 family monothiol glutaredoxin	QEN71_27675	paras_006068	protein-codi	NZ_CP1252	chromosom	6168765	6169076	+	312	0	5	1233	5	1233	
QEN71_RS27685	UbiX family flavin prenyltransferase	QEN71_27680	paras_006069	protein-codi	NZ_CP1252	chromosom	6169101	6169700	+	600	0	10	1296	6	428	
QEN71_RS27690	class III extradiol ring-cleavage dioxygenase	QEN71_27685	paras_006070	protein-codi	NZ_CP1252	chromosom	6169824	6170609	+	786	0	14	5332	9	1496	
QEN71_RS27695	APC family permease	QEN71_27690	paras_006071	protein-codi	NZ_CP1252	chromosom	6170687	6172291	-	1605	194.0	61	24215	49	20772	
QEN71_RS27700	cold-shock protein	QEN71_27695	paras_006072	protein-codi	NZ_CP1252	chromosom	6172752	6172955	+	204	0	4	332	2	76	
QEN71_RS27705	Hsp70 family protein	QEN71_27700	paras_006073	protein-codi	NZ_CP1252	chromosom	6173050	6174318	-	1269	0	41	17584	31	13462	
QEN71_RS27710	MFS transporter	QEN71_27705	paras_006074	protein-codi	NZ_CP1252	chromosom	6174570	6176030	+	1461	0	37	16126	32	13071	
QEN71_RS27715	nitroreductase family protein	QEN71_27710	paras_006075	protein-codi	NZ_CP1252	chromosom	6176099	6176692	-	594	0	15	3402	14	3394	
QEN71_RS27720	MFS transporter	QEN71_27715	paras_006076	protein-codi	NZ_CP1252	chromosom	6176876	6178159	-	1284	0	28	10477	21	6487	
QEN71_RS27725	TetR/AcrR family transcriptional regulator	QEN71_27720	paras_006077	protein-codi	NZ_CP1252	chromosom	6178327	6178959	+	633	0	20	4336	14	3150	
QEN71_RS27730	DUF1415 domain-containing protein	QEN71_27725	paras_006078	protein-codi	NZ_CP1252	chromosom	6178987	6179556	+	570	0	13	4079	13	4079	
QEN71_RS27735	SAM-dependent methyltransferase	QEN71_27730	paras_006079	protein-codi	NZ_CP1252	chromosom	6179570	6180406	-	837	0	42	14287	29	9130	
QEN71_RS27740	hypothetical protein	QEN71_27735	paras_006080	protein-codi	NZ_CP1252	chromosom	6180799	6181119	+	321	0	5	649	2	406	
QEN71_RS27745	DUF1059 domain-containing protein	QEN71_27740	paras_006081	protein-codi	NZ_CP1252	chromosom	6181216	6181425	-	210	0	4	2534	0	0	
QEN71_RS27750	TIGR03862 family flavoprotein	pseudo;QEN71_27745		pseudogene	NZ_CP1252	chromosom	6181748	6182904	+	1157	0	25	6957	17	3766	
QEN71_RS27755	class I SAM-dependent methyltransferase	QEN71_27750	paras_006083	protein-codi	NZ_CP1252	chromosom	6182921	6184045	-	1125	0	15	3760	13	3682	
QEN71_RS27760	tRNA-Thr	QEN71_27755		tRNA	NZ_CP1252	chromosom	6184234	6184309	-	76	0	0	0	0	0	
QEN71_RS27765	ClpXP protease specificity-enhancing factor	QEN71_27760	paras_006085	protein-codi	NZ_CP1252	chromosom	6184329	6184808	-	480	0	16	4778	13	4110	
QEN71_RS27770	glutathione S-transferase N-terminal domain-contai	QEN71_27765	paras_006086	protein-codi	NZ_CP1252	chromosom	6184884	6185495	-	612	0	0	0	0	0	TRUE
QEN71_RS27775	cytochrome c1	QEN71_27770	paras_006087	protein-codi	NZ_CP1252	chromosom	6185591	6186346	-	756	0	29	4006	26	2247	
QEN71_RS27780	cytochrome bc complex cytochrome b subunit	QEN71_27775	paras_006088	protein-codi	NZ_CP1252	chromosom	6186370	6187752	-	1382	0	44	5162	31	3707	
QEN71_RS27785	ubiquinol-cytochrome c reductase iron-sulfur subun	QEN71_27780	paras_006089	protein-codi	NZ_CP1252	chromosom	6187752	6188369	-	617	0	21	3792	15	3030	
QEN71_RS27790	Nif3-like dinuclear metal center hexameric protein	QEN71_27785	paras_006090	protein-codi	NZ_CP1252	chromosom	6188589	6189335	-	747	0	36	15393	18	8340	
QEN71_RS27795	Do family serine endopeptidase	QEN71_27790	paras_006091	protein-codi	NZ_CP1252	chromosom	6189355	6190563	+	1209	0	16	3347	16	3347	
QEN71_RS27800	twin-arginine translocase subunit TatC	QEN71_27795	paras_006092	protein-codi	NZ_CP1252	chromosom	6190662	6191441	-	780	0	12	125	11	116	
QEN71_RS27805	Sec-independent protein translocase protein TatB	QEN71_27800	paras_006093	protein-codi	NZ_CP1252	chromosom	6191484	6192035	-	552	0	8	220	6	141	
QEN71_RS27810	Sec-independent protein translocase subunit TatA	QEN71_27805	paras_006094	protein-codi	NZ_CP1252	chromosom	6192091	6192327	-	237	0	3	419	1	324	
QEN71_RS27815	histidine triad nucleotide-binding protein	QEN71_27810	paras_006095	protein-codi	NZ_CP1252	chromosom	6192642	6193007	-	366	0	13	3746	13	3746	
QEN71_RS27820	membrane protein	QEN71_27815	paras_006096	protein-codi	NZ_CP1252	chromosom	6193054	6193449	-	396	0	26	10926	24	10565	
QEN71_RS27825	phosphoribosyl-ATP diphosphatase	QEN71_27820	paras_006097	protein-codi	NZ_CP1252	chromosom	6193553	6193927	-	371	0	4	1319	2	901	
QEN71_RS27830	phosphoribosyl-AMP cyclohydrolase	QEN71_27825	paras_006098	protein-codi	NZ_CP1252	chromosom	6193924	6194334	-	407	0	5	3207	2	557	
QEN71_RS27835	imidazole glycerol phosphate synthase subunit HisF	QEN71_27830	paras_006099	protein-codi	NZ_CP1252	chromosom	6194336	6195109	-	774	0	22	6250	14	3495	
QEN71_RS27840	1-(5-phosphoribosyl)-5-[(5- phosphoribosylamino)m	QEN71_27835	paras_006100	protein-codi	NZ_CP1252	chromosom	6195237	6195989	-	753	0	21	4302	15	2559	
QEN71_RS27845	imidazole glycerol phosphate synthase subunit HisH	QEN71_27840	paras_006101	protein-codi	NZ_CP1252	chromosom	6196200	6196841	-	638	0	19	5153	15	5046	
QEN71_RS27850	MarC family protein	QEN71_27845	paras_006102	protein-codi	NZ_CP1252	chromosom	6196838	6197458	-	617	0	27	8756	19	7347	
QEN71_RS27855	imidazoleglycerol-phosphate dehydratase HisB	QEN71_27850	paras_006103	protein-codi	NZ_CP1252	chromosom	6197534	6198121	-	588	0	15	1752	13	1346	
QEN71_RS27860	histidinol-phosphate transaminase	QEN71_27855	paras_006104	protein-codi	NZ_CP1252	chromosom	6198173	6199243	-	1071	0	23	5971	19	5453	
QEN71_RS27865	histidinol dehydrogenase	QEN71_27860	paras_006105	protein-codi	NZ_CP1252	chromosom	6199390	6200712	-	1323	0	30	7345	28	6679	
QEN71_RS27870	ATP phosphoribosyltransferase	QEN71_27865	paras_006106	protein-codi	NZ_CP1252	chromosom	6200765	6201463	-	695	0	13	2848	13	2848	
QEN71_RS27875	UDP-N-acetylglucosamine 1-carboxyvinyltransferas	QEN71_27870	paras_006107	protein-codi	NZ_CP1252	chromosom	6201460	6202722	-	1259	0	2	28	0	0	TRUE
QEN71_RS27880	BolA family protein	QEN71_27875	paras_006108	protein-codi	NZ_CP1252	chromosom	6202978	6203217	-	240	0	3	1482	2	1342	
QEN71_RS27885	ABC transporter permease	QEN71_27880	paras_006109	protein-codi	NZ_CP1252	chromosom	6203228	6203983	-	752	0	15	4621	12	3079	
QEN71_RS27890	ABC transporter ATP-binding protein	QEN71_27885	paras_006110	protein-codi	NZ_CP1252	chromosom	6203980	6204906	-	923	0	6	1626	3	1495	
QEN71_RS27895	STAS domain-containing protein	QEN71_27890	paras_006111	protein-codi	NZ_CP1252	chromosom	6205174	6205479	-	302	0	6	381	4	297	
QEN71_RS27900	ABC transporter substrate-binding protein	QEN71_27895	paras_006112	protein-codi	NZ_CP1252	chromosom	6205476	6206108	-	629	0	26	2996	20	2718	
QEN71_RS27905	VacJ family lipoprotein	QEN71_27900	paras_006113	protein-codi	NZ_CP1252	chromosom	6206247	6207215	-	969	0	43	15200	38	10831	
QEN71_RS27910	outer membrane lipid asymmetry maintenance prote	QEN71_27905	paras_006114	protein-codi	NZ_CP1252	chromosom	6207228	6207782	-	555	0	19	2895	15	2497	
QEN71_RS27915	lipid asymmetry maintenance ABC transporter perm	QEN71_27910	paras_006115	protein-codi	NZ_CP1252	chromosom	6207854	6208621	-	764	0	21	2448	18	1912	
QEN71_RS27920	ABC transporter ATP-binding protein	QEN71_27915	paras_006116	protein-codi	NZ_CP1252	chromosom	6208618	6209439	-	818	0	16	3170	16	3170	
QEN71_RS27925	thiamine phosphate synthase	QEN71_27920	paras_006117	protein-codi	NZ_CP1252	chromosom	6209526	6210641	-	1112	0	6	741	0	0	
QEN71_RS27930	thiazole synthase	QEN71_27925	paras_006118	protein-codi	NZ_CP1252	chromosom	6210638	6211462	-	821	0	0	0	0	0	TRUE
QEN71_RS27935	sulfur carrier protein ThiS	QEN71_27930	paras_006119	protein-codi	NZ_CP1252	chromosom	6211505	6211702	-	198	0	0	0	0	0	
QEN71_RS27940	FAD-dependent oxidoreductase	QEN71_27935	paras_006120	protein-codi	NZ_CP1252	chromosom	6211713	6212861	-	1149	0	11	4448	10	4272	
QEN71_RS27945	ABC transporter ATP-binding protein/permease	QEN71_27940	paras_006121	protein-codi	NZ_CP1252	chromosom	6213509	6215242	+	1734	0	45	9037	38	7505	
QEN71_RS27950	hypothetical protein	QEN71_27945	paras_006122	protein-codi	NZ_CP1252	chromosom	6215501	6216010	+	510	0	4	292	2	280	
QEN71_RS27955	hypothetical protein	QEN71_27950	paras_006123	protein-codi	NZ_CP1252	chromosom	6216098	6216649	+	552	0	33	5713	28	4290	
QEN71_RS27960	response regulator	QEN71_27955	paras_006124	protein-codi	NZ_CP1252	chromosom	6217371	6218117	+	747	0	22	5111	12	2848	
QEN71_RS27965	flagellar basal body L-ring protein FlgH	QEN71_27960	paras_006125	protein-codi	NZ_CP1252	chromosom	6218182	6218868	-	687	0	13	1737	11	1485	
QEN71_RS27970	hypothetical protein	QEN71_27965	paras_006126	protein-codi	NZ_CP1252	chromosom	6218913	6219638	-	726	0	15	4255	11	3373	
QEN71_RS27975	hypothetical protein	QEN71_27970	paras_006127	protein-codi	NZ_CP1252	chromosom	6219669	6219878	-	210	0	2	37	2	37	
QEN71_RS27980	hypothetical protein	QEN71_27975	paras_006128	protein-codi	NZ_CP1252	chromosom	6219895	6220086	-	192	0	0	0	0	0	
QEN71_RS27985	response regulator	QEN71_27980	paras_006129	protein-codi	NZ_CP1252	chromosom	6220490	6221227	+	738	0	13	4886	10	3974	

QEN71_RS27990	ATP-binding protein	QEN71_27985	paras_006130	protein-codi	NZ_CP1252	chromosom	6221244	6222554	+	1311	0	12	2746	7	1763	
QEN71_RS27995	tyrosine-protein phosphatase	QEN71_27990	paras_006131	protein-codi	NZ_CP1252	chromosom	6222734	6223699	-	966	0	18	5475	13	4726	
QEN71_RS28000	RNA 2',3'-cyclic phosphodiesterase	QEN71_27995	paras_006132	protein-codi	NZ_CP1252	chromosom	6223969	6224613	-	645	0	12	2366	8	1927	
QEN71_RS28005	glutamate synthase subunit beta	QEN71_28000	paras_006133	protein-codi	NZ_CP1252	chromosom	6224835	6226301	-	1467	0	21	5172	9	968	
QEN71_RS28010	glutamate synthase-related protein	QEN71_28005	paras_006134	protein-codi	NZ_CP1252	chromosom	6226412	6231115	-	4704	0	103	32757	83	28588	
QEN71_RS28015	transposase	QEN71_28010	paras_006135	protein-codi	NZ_CP1252	chromosom	6231433	6232140	-	708	0	29	10891	25	6594	
QEN71_RS28020	OmpW family outer membrane protein	QEN71_28015	paras_006136	protein-codi	NZ_CP1252	chromosom	6232328	6233050	+	723	0	41	14023	28	8585	
QEN71_RS28025	DUF883 family protein	QEN71_28020	paras_006137	protein-codi	NZ_CP1252	chromosom	6233399	6233782	+	384	0	7	3220	3	895	
QEN71_RS28030	deoxyguanosinetriphosphate triphosphohydrolase	QEN71_28025	paras_006138	protein-codi	NZ_CP1252	chromosom	6233918	6235111	-	1194	0	33	6707	22	3855	
QEN71_RS28035	3-dehydroquinate synthase	QEN71_28030	paras_006139	protein-codi	NZ_CP1252	chromosom	6235190	6236281	-	1092	0	14	4457	10	4242	
QEN71_RS28040	shikimate kinase	QEN71_28035	paras_006140	protein-codi	NZ_CP1252	chromosom	6236289	6236840	-	552	0	15	4761	8	2190	
QEN71_RS28045	type IV pilus secretin PilQ	QEN71_28040	paras_006141	protein-codi	NZ_CP1252	chromosom	6236988	6238553	-	1562	0	19	6529	12	3211	
QEN71_RS28050	type 4a pilus biogenesis protein PilO	QEN71_28045	paras_006142	protein-codi	NZ_CP1252	chromosom	6238550	6239464	-	907	0	10	4588	10	4588	
QEN71_RS28055	fimbrial assembly protein	QEN71_28050	paras_006143	protein-codi	NZ_CP1252	chromosom	6239461	6240102	-	634	0	2	593	0	0	
QEN71_RS28060	pilus assembly protein PilM	QEN71_28055	paras_006144	protein-codi	NZ_CP1252	chromosom	6240099	6241058	-	956	0	18	1983	16	1747	
QEN71_RS28065	penicillin-binding protein 1A	QEN71_28060	paras_006145	protein-codi	NZ_CP1252	chromosom	6241313	6243703	+	2391	0	62	2984	46	1198	
QEN71_RS28070	iron donor protein CyaY	QEN71_28065	paras_006146	protein-codi	NZ_CP1252	chromosom	6243805	6244122	-	318	0	5	1040	3	515	
QEN71_RS28075	lipoprotein	QEN71_28070	paras_006147	protein-codi	NZ_CP1252	chromosom	6244158	6244457	+	300	0	6	1340	3	324	
QEN71_RS28080	diaminopimelate decarboxylase	QEN71_28075	paras_006148	protein-codi	NZ_CP1252	chromosom	6244467	6245729	+	1263	0	41	12492	26	6837	
QEN71_RS28085	protein-methionine-sulfoxide reductase heme-bindir	QEN71_28080	paras_006149	protein-codi	NZ_CP1252	chromosom	6245810	6246511	-	702	0	18	4165	11	2582	
QEN71_RS28090	protein-methionine-sulfoxide reductase catalytic sub	QEN71_28085	paras_006150	protein-codi	NZ_CP1252	chromosom	6246522	6247517	-	996	0	34	13324	26	10421	
QEN71_RS28095	c-type cytochrome biogenesis protein CcsB	QEN71_28090	paras_006151	protein-codi	NZ_CP1252	chromosom	6247692	6248909	-	1218	0	28	1730	22	878	
QEN71_RS28100	cytochrome c biogenesis protein ResB	QEN71_28095	paras_006152	protein-codi	NZ_CP1252	chromosom	6248914	6251139	-	2226	0	37	762	30	566	
QEN71_RS28105	c-type cytochrome	QEN71_28100	paras_006153	protein-codi	NZ_CP1252	chromosom	6251266	6251919	-	654	0	21	3127	15	1685	
QEN71_RS28110	ribosome biogenesis GTP-binding protein YihA/Ysx	QEN71_28105	paras_006154	protein-codi	NZ_CP1252	chromosom	6252199	6252864	+	666	0	3	852	0	0	
QEN71_RS28115	porphobilinogen synthase	QEN71_28110	paras_006155	protein-codi	NZ_CP1252	chromosom	6253112	6254110	+	999	0	0	0	0	0	TRUE
QEN71_RS28120	protein-disulfide reductase DsbD	QEN71_28115	paras_006156	protein-codi	NZ_CP1252	chromosom	6254237	6256090	-	1846	0	31	478	24	406	
QEN71_RS28125	divalent-cation tolerance protein CutA	QEN71_28120	paras_006157	protein-codi	NZ_CP1252	chromosom	6256083	6256409	-	319	0	11	2010	8	949	
QEN71_RS28130	50S ribosomal protein L17	QEN71_28125	paras_006158	protein-codi	NZ_CP1252	chromosom	6256566	6256961	-	396	0	3	321	1	11	
QEN71_RS28135	DNA-directed RNA polymerase subunit alpha	QEN71_28130	paras_006159	protein-codi	NZ_CP1252	chromosom	6257114	6258091	-	978	0	3	929	0	0	
QEN71_RS28140	30S ribosomal protein S4	QEN71_28135	paras_006160	protein-codi	NZ_CP1252	chromosom	6258216	6258839	-	624	0	3	85	1	44	TRUE
QEN71_RS28145	30S ribosomal protein S11	QEN71_28140	paras_006161	protein-codi	NZ_CP1252	chromosom	6258986	6259390	-	405	0	2	305	1	3	
QEN71_RS28150	30S ribosomal protein S13	QEN71_28145	paras_006162	protein-codi	NZ_CP1252	chromosom	6259419	6259784	-	366	0	0	0	0	0	TRUE
QEN71_RS28155	50S ribosomal protein L36	QEN71_28150	paras_006163	protein-codi	NZ_CP1252	chromosom	6259826	6259942	-	117	0	0	0	0	0	
QEN71_RS28160	translation initiation factor IF-1	QEN71_28155	paras_006164	protein-codi	NZ_CP1252	chromosom	6259963	6260181	-	219	0	5	311	4	270	
QEN71_RS28165	preprotein translocase subunit SecY	QEN71_28160	paras_006165	protein-codi	NZ_CP1252	chromosom	6260190	6261536	-	1347	0	2	21	2	21	TRUE
QEN71_RS28170	50S ribosomal protein L15	QEN71_28165	paras_006166	protein-codi	NZ_CP1252	chromosom	6261583	6262017	-	435	0	0	0	0	0	TRUE
QEN71_RS28175	50S ribosomal protein L30	QEN71_28170	paras_006167	protein-codi	NZ_CP1252	chromosom	6262048	6262230	-	183	0	0	0	0	0	
QEN71_RS28180	30S ribosomal protein S5	QEN71_28175	paras_006168	protein-codi	NZ_CP1252	chromosom	6262241	6262759	-	519	0	0	0	0	0	TRUE
QEN71_RS28185	50S ribosomal protein L18	QEN71_28180	paras_006169	protein-codi	NZ_CP1252	chromosom	6262774	6263139	-	366	0	0	0	0	0	TRUE
QEN71_RS28190	50S ribosomal protein L6	QEN71_28185	paras_006170	protein-codi	NZ_CP1252	chromosom	6263152	6263682	-	531	0	0	0	0	0	TRUE
QEN71_RS28195	30S ribosomal protein S8	QEN71_28190	paras_006171	protein-codi	NZ_CP1252	chromosom	6263702	6264097	-	396	0	0	0	0	0	TRUE
QEN71_RS28200	30S ribosomal protein S14	QEN71_28195	paras_006172	protein-codi	NZ_CP1252	chromosom	6264112	6264417	-	306	0	1	192	0	0	
QEN71_RS28205	50S ribosomal protein L5	QEN71_28200	paras_006173	protein-codi	NZ_CP1252	chromosom	6264425	6264964	-	540	0	0	0	0	0	TRUE
QEN71_RS28210	50S ribosomal protein L24	QEN71_28205	paras_006174	protein-codi	NZ_CP1252	chromosom	6264981	6265289	-	309	0	0	0	0	0	TRUE
QEN71_RS28215	50S ribosomal protein L14	QEN71_28210	paras_006175	protein-codi	NZ_CP1252	chromosom	6265299	6265667	-	369	0	1	10	1	10	TRUE
QEN71_RS28220	30S ribosomal protein S17	QEN71_28215	paras_006176	protein-codi	NZ_CP1252	chromosom	6265959	6266231	-	269	0	1	27	0	0	
QEN71_RS28225	50S ribosomal protein L29	QEN71_28220	paras_006177	protein-codi	NZ_CP1252	chromosom	6266228	6266422	-	191	0	1	22	1	22	
QEN71_RS28230	50S ribosomal protein L16	QEN71_28225	paras_006178	protein-codi	NZ_CP1252	chromosom	6266433	6266849	-	417	0	0	0	0	0	TRUE
QEN71_RS28235	30S ribosomal protein S3	QEN71_28230	paras_006179	protein-codi	NZ_CP1252	chromosom	6266852	6267646	-	795	0	1	8	1	8	TRUE
QEN71_RS28240	50S ribosomal protein L22	QEN71_28235	paras_006180	protein-codi	NZ_CP1252	chromosom	6267658	6267987	-	330	0	0	0	0	0	TRUE
QEN71_RS28245	30S ribosomal protein S19	QEN71_28240	paras_006181	protein-codi	NZ_CP1252	chromosom	6268000	6268275	-	276	0	0	0	0	0	TRUE
QEN71_RS28250	50S ribosomal protein L2	QEN71_28245	paras_006182	protein-codi	NZ_CP1252	chromosom	6268286	6269113	-	828	0	2	16	1	2	TRUE
QEN71_RS28255	50S ribosomal protein L23	QEN71_28250	paras_006183	protein-codi	NZ_CP1252	chromosom	6269116	6269430	-	311	0	0	0	0	0	TRUE
QEN71_RS28260	50S ribosomal protein L4	QEN71_28255	paras_006184	protein-codi	NZ_CP1252	chromosom	6269427	6270047	-	616	0	0	0	0	0	TRUE
QEN71_RS28265	50S ribosomal protein L3	QEN71_28260	paras_006185	protein-codi	NZ_CP1252	chromosom	6270047	6270706	-	659	0	1	5	0	0	TRUE
QEN71_RS28270	30S ribosomal protein S10	QEN71_28265	paras_006186	protein-codi	NZ_CP1252	chromosom	6270895	6271206	-	312	0	2	210	0	0	
QEN71_RS28275	elongation factor Tu	QEN71_28270	paras_006187	protein-codi	NZ_CP1252	chromosom	6271334	6272524	-	1191	2317.0	3	119	0	0	
QEN71_RS28280	elongation factor G	QEN71_28275	paras_006188	protein-codi	NZ_CP1252	chromosom	6272573	6274675	-	2103	0	5	773	2	11	TRUE
QEN71_RS28285	30S ribosomal protein S7	QEN71_28280	paras_006189	protein-codi	NZ_CP1252	chromosom	6274813	6275283	-	471	0	1	291	0	0	
QEN71_RS28290	30S ribosomal protein S12	QEN71_28285	paras_006190	protein-codi	NZ_CP1252	chromosom	6275462	6275842	-	381	0	0	0	0	0	TRUE
QEN71_RS28295	DNA helicase RecQ	QEN71_28290	paras_006191	protein-codi	NZ_CP1252	chromosom	6276063	6277910	-	1848	0	49	18950	42	16744	

QEN71_RS28300	DNA-directed RNA polymerase subunit beta'	QEN71_28295	paras_006192	protein-codi	NZ_CP1252	chromosom	6278123	6282364	-	4242	0	8	727	3	34	TRUE
QEN71_RS28305	DNA-directed RNA polymerase subunit beta	QEN71_28300	paras_006193	protein-codi	NZ_CP1252	chromosom	6282386	6286492	-	4107	0	1	6	1	6	TRUE
QEN71_RS28310	50S ribosomal protein L7/L12	QEN71_28305	paras_006194	protein-codi	NZ_CP1252	chromosom	6286862	6287239	-	378	0	1	8	0	0	TRUE
QEN71_RS28315	50S ribosomal protein L10	QEN71_28310	paras_006195	protein-codi	NZ_CP1252	chromosom	6287315	6287812	-	498	0	1	3	0	0	TRUE
QEN71_RS28320	50S ribosomal protein L1	QEN71_28315	paras_006196	protein-codi	NZ_CP1252	chromosom	6288117	6288815	-	699	0	2	108	1	5	TRUE
QEN71_RS28325	50S ribosomal protein L11	QEN71_28320	paras_006197	protein-codi	NZ_CP1252	chromosom	6288816	6289247	-	432	0	1	15	1	15	TRUE
QEN71_RS28330	transcription termination/antitermination protein Nus	QEN71_28325	paras_006198	protein-codi	NZ_CP1252	chromosom	6289398	6289955	-	558	0	1	618	0	0	
QEN71_RS28335	preprotein translocase subunit SecE	QEN71_28330	paras_006199	protein-codi	NZ_CP1252	chromosom	6289957	6290337	-	381	0	0	0	0	0	TRUE
QEN71_RS28340	tRNA-Trp	QEN71_28335		tRNA	NZ_CP1252	chromosom	6290373	6290448	-	76	0	0	0	0	0	
QEN71_RS28345	elongation factor Tu	QEN71_28340	paras_006201	protein-codi	NZ_CP1252	chromosom	6290518	6291708	-	1191	2317.0	21	1870	20	1859	
QEN71_RS28350	tRNA-Thr	QEN71_28345		tRNA	NZ_CP1252	chromosom	6291774	6291848	-	75	0	0	0	0	0	
QEN71_RS28355	tRNA-Gly	QEN71_28350		tRNA	NZ_CP1252	chromosom	6291869	6291942	-	74	0	1	18	1	18	
QEN71_RS28360	tRNA-Tyr	QEN71_28355		tRNA	NZ_CP1252	chromosom	6291988	6292073	-	86	0	0	0	0	0	
QEN71_RS28365	5S ribosomal RNA	QEN71_28360		rRNA	NZ_CP1252	chromosom	6292216	6292329	-	114	225.0	3	235	2	146	
QEN71_RS28370	23S ribosomal RNA	QEN71_28365		rRNA	NZ_CP1252	chromosom	6292528	6295408	-	2881	4155.0	173	21817	140	14313	
QEN71_RS28375	tRNA-Ala	QEN71_28370		tRNA	NZ_CP1252	chromosom	6295714	6295789	-	76	150.0	1	6	1	6	
QEN71_RS28380	tRNA-Ile	QEN71_28375		tRNA	NZ_CP1252	chromosom	6295850	6295926	-	77	152.0	3	858	2	786	
QEN71_RS28385	16S ribosomal RNA	QEN71_28380		rRNA	NZ_CP1252	chromosom	6295991	6297521	-	1531	3015.0	92	8963	75	7696	
QEN71_RS28390	SGNH/GDSL hydrolase family protein	QEN71_28385	paras_006210	protein-codi	NZ_CP1252	chromosom	6297980	6299248	-	1269	0	46	24811	41	24077	
QEN71_RS28395	1,2-phenylacetyl-CoA epoxidase subunit PaaA	QEN71_28390	paras_006211	protein-codi	NZ_CP1252	chromosom	6299419	6300417	+	999	0	22	12222	20	9138	
QEN71_RS28400	1,2-phenylacetyl-CoA epoxidase subunit PaaB	QEN71_28395	paras_006212	protein-codi	NZ_CP1252	chromosom	6300442	6300726	+	285	0	9	6021	6	2089	
QEN71_RS28405	1,2-phenylacetyl-CoA epoxidase subunit PaaC	QEN71_28400	paras_006213	protein-codi	NZ_CP1252	chromosom	6300745	6301548	+	804	0	27	8312	21	7050	
QEN71_RS28410	1,2-phenylacetyl-CoA epoxidase subunit PaaD	QEN71_28405	paras_006214	protein-codi	NZ_CP1252	chromosom	6301577	6302137	+	561	0	9	2295	3	605	
QEN71_RS28415	1,2-phenylacetyl-CoA epoxidase subunit PaaE	QEN71_28410	paras_006215	protein-codi	NZ_CP1252	chromosom	6302139	6303227	+	1089	0	30	7151	21	4730	
QEN71_RS28420	DUF1835 domain-containing protein	QEN71_28415	paras_006216	protein-codi	NZ_CP1252	chromosom	6303427	6304242	+	816	0	8	1656	5	937	
QEN71_RS28425	TetR/AcrR family transcriptional regulator	QEN71_28420	paras_006217	protein-codi	NZ_CP1252	chromosom	6304260	6304889	+	630	0	23	8256	17	5892	
QEN71_RS28430	GNAT family N-acetyltransferase	QEN71_28425	paras_006218	protein-codi	NZ_CP1252	chromosom	6305138	6305803	+	666	0	18	8377	15	7931	
QEN71_RS28435	Lrp/AsnC family transcriptional regulator	QEN71_28430	paras_006219	protein-codi	NZ_CP1252	chromosom	6305887	6306402	-	516	0	12	4682	10	4079	
QEN71_RS28440	4-hydroxyphenylpyruvate dioxygenase	QEN71_28435	paras_006220	protein-codi	NZ_CP1252	chromosom	6306556	6307653	+	1098	0	38	21290	34	19168	
QEN71_RS28445	hypothetical protein	QEN71_28440	paras_006221	protein-codi	NZ_CP1252	chromosom	6307776	6307964	-	189	0	0	0	0	0	
QEN71_RS28450	indolepyruvate ferredoxin oxidoreductase family pro	QEN71_28445	paras_006222	protein-codi	NZ_CP1252	chromosom	6308312	6311929	+	3618	0	86	26009	68	22028	
QEN71_RS28455	NADP-dependent malic enzyme	QEN71_28450	paras_006223	protein-codi	NZ_CP1252	chromosom	6312006	6314291	-	2286	0	48	13211	34	10051	
QEN71_RS28460	MFS transporter	QEN71_28455	paras_006224	protein-codi	NZ_CP1252	chromosom	6314588	6315820	-	1233	0	38	8196	31	6653	
QEN71_RS28465	orotate phosphoribosyltransferase	QEN71_28460	paras_006225	protein-codi	NZ_CP1252	chromosom	6316167	6316853	+	687	0	19	2514	17	2389	
QEN71_RS28470	response regulator	QEN71_28465	paras_006226	protein-codi	NZ_CP1252	chromosom	6316967	6317674	-	708	0	22	2149	20	1962	
QEN71_RS28475	YbhB/YbcL family Raf kinase inhibitor-like protein	QEN71_28470	paras_006227	protein-codi	NZ_CP1252	chromosom	6317940	6318569	+	630	0	17	4845	10	2797	
QEN71_RS28480	flavodoxin family protein	QEN71_28475	paras_006228	protein-codi	NZ_CP1252	chromosom	6318679	6319254	+	576	0	14	4950	8	3416	
QEN71_RS28485	N-acetyl-gamma-glutamyl-phosphate reductase	QEN71_28480	paras_006229	protein-codi	NZ_CP1252	chromosom	6319422	6320369	+	948	0	22	5387	15	3056	
QEN71_RS28490	DUF2957 domain-containing protein	QEN71_28485	paras_006230	protein-codi	NZ_CP1252	chromosom	6320910	6322304	+	1395	0	44	12841	33	8865	
QEN71_RS28495	DUF2957 domain-containing protein	QEN71_28490	paras_006231	protein-codi	NZ_CP1252	chromosom	6322422	6323669	+	1248	0	23	7585	17	5836	
QEN71_RS28500	OmpW family protein	QEN71_28495	paras_006232	protein-codi	NZ_CP1252	chromosom	6323869	6324702	+	834	0	26	10034	18	6777	
QEN71_RS28505	LysE family translocator	QEN71_28500	paras_006233	protein-codi	NZ_CP1252	chromosom	6324940	6325596	-	657	0	16	5569	12	3151	
QEN71_RS28510	NAD(P)-dependent oxidoreductase	QEN71_28505	paras_006234	protein-codi	NZ_CP1252	chromosom	6325745	6326614	-	870	0	9	2955	8	2706	
QEN71_RS28515	Mut7-C RNAse domain-containing protein	QEN71_28510	paras_006235	protein-codi	NZ_CP1252	chromosom	6326716	6327471	+	756	0	24	6373	20	4962	
QEN71_RS28520	hypothetical protein	QEN71_28515	paras_006236	protein-codi	NZ_CP1252	chromosom	6327553	6327840	+	288	0	0	0	0	0	TRUE
QEN71_RS28525	hypothetical protein	QEN71_28520	paras_006237	protein-codi	NZ_CP1252	chromosom	6327971	6328180	-	210	0	4	2202	2	236	
QEN71_RS28530	DMT family transporter	QEN71_28525	paras_006238	protein-codi	NZ_CP1252	chromosom	6328515	6329450	+	936	0	13	6669	13	6669	
QEN71_RS28535	hypothetical protein	QEN71_28530	paras_006239	protein-codi	NZ_CP1252	chromosom	6329490	6329756	-	263	0	9	2732	7	2511	
QEN71_RS28540	hypothetical protein	QEN71_28535	paras_006240	protein-codi	NZ_CP1252	chromosom	6329753	6329950	-	194	0	2	139	0	0	
QEN71_RS28545	YbfB/YjiJ family MFS transporter	QEN71_28540	paras_006241	protein-codi	NZ_CP1252	chromosom	6330216	6331391	-	1176	0	32	9287	21	7826	
QEN71_RS28550	diguanylate cyclase	QEN71_28545	paras_006242	protein-codi	NZ_CP1252	chromosom	6331739	6333349	+	1611	0	33	10663	25	7919	
QEN71_RS28555	GGDEF domain-containing protein	QEN71_28550	paras_006243	protein-codi	NZ_CP1252	chromosom	6333628	6334578	+	951	0	8	1001	1	11	
QEN71_RS28560	LacI family DNA-binding transcriptional regulator	QEN71_28555	paras_006244	protein-codi	NZ_CP1252	chromosom	6334592	6335641	-	1050	0	23	3478	19	3009	
QEN71_RS28565	phytanoyl-CoA dioxygenase family protein	QEN71_28560	paras_006245	protein-codi	NZ_CP1252	chromosom	6335742	6336938	+	1197	0	20	4202	18	3962	
QEN71_RS28570	Gfo/Idh/MocA family oxidoreductase	QEN71_28565	paras_006246	protein-codi	NZ_CP1252	chromosom	6336957	6338111	+	1155	0	29	10473	23	8427	
QEN71_RS28575	hypothetical protein	QEN71_28570	paras_006247	protein-codi	NZ_CP1252	chromosom	6338803	6339069	-	267	0	5	740	1	19	
QEN71_RS28580	methyltransferase domain-containing protein	QEN71_28575	paras_006248	protein-codi	NZ_CP1252	chromosom	6339750	6340562	+	813	0	16	2159	7	1554	
QEN71_RS28585	glutamine--fructose-6-phosphate transaminase (iso	QEN71_28580	paras_006249	protein-codi	NZ_CP1252	chromosom	6340688	6342505	-	1818	0	3	84	1	2	TRUE
QEN71_RS28590	bifunctional UDP-N-acetylglucosamine diphosphory	QEN71_28585	paras_006250	protein-codi	NZ_CP1252	chromosom	6342566	6343927	-	1362	0	0	0	0	0	TRUE
QEN71_RS28595	tRNA 2-thiocytidine(32) synthetase TtcA	QEN71_28590	paras_006251	protein-codi	NZ_CP1252	chromosom	6344059	6345087	-	1029	0	20	8679	19	8646	
QEN71_RS28600	dihydroneopterin aldolase	QEN71_28595	paras_006252	protein-codi	NZ_CP1252	chromosom	6345091	6345483	-	393	0	0	0	0	0	TRUE
QEN71_RS28605	SDR family oxidoreductase	QEN71_28600	paras_006253	protein-codi	NZ_CP1252	chromosom	6345534	6346364	-	831	0	16	4575	13	4224	

QEN71_RS28610	class I SAM-dependent methyltransferase	QEN71_28605	paras_006254	protein-codi	NZ_CP1252	chromosom	6346378	6347571	+	1194	0	13	124	10	55	
QEN71_RS28615	DUF2905 domain-containing protein	QEN71_28610	paras_006255	protein-codi	NZ_CP1252	chromosom	6347587	6347781	+	195	0	4	504	4	504	
QEN71_RS28620	multifunctional CCA addition/repair protein	QEN71_28615	paras_006256	protein-codi	NZ_CP1252	chromosom	6347797	6349056	-	1256	0	9	1946	7	1266	
QEN71_RS28625	glutathione S-transferase family protein	QEN71_28620	paras_006257	protein-codi	NZ_CP1252	chromosom	6349053	6349700	-	644	0	11	2874	7	2498	
QEN71_RS28630	complex I NDUFA9 subunit family protein	QEN71_28625	paras_006258	protein-codi	NZ_CP1252	chromosom	6349734	6350690	-	957	0	25	9376	20	8578	
QEN71_RS28635	lytic transglycosylase domain-containing protein	QEN71_28630	paras_006259	protein-codi	NZ_CP1252	chromosom	6350786	6352759	-	1974	0	65	15662	46	11480	
QEN71_RS28640	5-formyltetrahydrofolate cyclo-ligase	QEN71_28635	paras_006260	protein-codi	NZ_CP1252	chromosom	6352791	6353396	+	606	0	36	7816	29	6085	
QEN71_RS28645	winged helix DNA-binding protein	QEN71_28640	paras_006261	protein-codi	NZ_CP1252	chromosom	6353553	6354065	-	513	0	33	11009	29	9980	
QEN71_RS28650	5-oxoprolinase subunit PxpB	QEN71_28645	paras_006262	protein-codi	NZ_CP1252	chromosom	6354266	6354919	+	650	0	13	4719	13	4719	
QEN71_RS28655	biotin-dependent carboxyltransferase family protein	QEN71_28650	paras_006263	protein-codi	NZ_CP1252	chromosom	6354916	6355968	+	1049	0	20	4616	12	2370	
QEN71_RS28660	5-oxoprolinase subunit PxpA	QEN71_28655	paras_006264	protein-codi	NZ_CP1252	chromosom	6355985	6356737	+	753	0	6	3221	6	3221	
QEN71_RS28665	DUF969 domain-containing protein	QEN71_28660	paras_006265	protein-codi	NZ_CP1252	chromosom	6357003	6357758	+	752	0	6	1553	4	417	
QEN71_RS28670	DUF979 domain-containing protein	QEN71_28665	paras_006266	protein-codi	NZ_CP1252	chromosom	6357755	6358711	+	953	0	15	4627	10	2271	
QEN71_RS28675	tetratricopeptide repeat protein	QEN71_28670	paras_006267	protein-codi	NZ_CP1252	chromosom	6358926	6359528	+	596	0	10	2583	10	2583	
QEN71_RS28680	TraB/GumN family protein	QEN71_28675	paras_006268	protein-codi	NZ_CP1252	chromosom	6359522	6360715	-	1179	0	23	8813	21	8568	
QEN71_RS28685	peptide ABC transporter ATP-binding protein	QEN71_28680	paras_006269	protein-codi	NZ_CP1252	chromosom	6360708	6361733	-	1014	0	26	14270	17	12163	
QEN71_RS28690	ABC transporter ATP-binding protein	QEN71_28685	paras_006270	protein-codi	NZ_CP1252	chromosom	6361730	6362734	-	1001	0	17	6826	17	6826	
QEN71_RS28695	ABC transporter permease subunit	QEN71_28690	paras_006271	protein-codi	NZ_CP1252	chromosom	6362736	6363653	-	918	0	14	6670	11	5595	
QEN71_RS28700	ABC transporter permease subunit	QEN71_28695	paras_006272	protein-codi	NZ_CP1252	chromosom	6363659	6364672	-	1014	0	27	7159	16	3673	
QEN71_RS28705	ABC transporter substrate-binding protein	QEN71_28700	paras_006273	protein-codi	NZ_CP1252	chromosom	6364827	6366458	-	1632	0	67	27957	51	21630	
QEN71_RS28710	ABC transporter substrate-binding protein	QEN71_28705	paras_006274	protein-codi	NZ_CP1252	chromosom	6366644	6368287	-	1644	0	67	21383	61	20001	
QEN71_RS28715	methylenetetrahydrofolate reductase [NAD(P)H]	QEN71_28710	paras_006275	protein-codi	NZ_CP1252	chromosom	6368468	6369298	-	831	0	16	2448	14	2147	
QEN71_RS28720	phage holin family protein	QEN71_28715	paras_006276	protein-codi	NZ_CP1252	chromosom	6369319	6369672	-	354	0	8	4749	6	3749	
QEN71_RS28725	adenosylhomocysteinase	QEN71_28720	paras_006277	protein-codi	NZ_CP1252	chromosom	6369823	6371235	-	1413	0	16	1321	10	83	
QEN71_RS28730	glycosyltransferase family A protein	QEN71_28725	paras_006278	protein-codi	NZ_CP1252	chromosom	6371433	6372248	-	816	0	26	6713	21	3992	
QEN71_RS28735	LrgB family protein	QEN71_28730	paras_006279	protein-codi	NZ_CP1252	chromosom	6372340	6373062	-	723	0	16	6682	14	5670	
QEN71_RS28740	CidA/LrgA family protein	QEN71_28735	paras_006280	protein-codi	NZ_CP1252	chromosom	6373087	6373578	-	492	0	5	1918	3	1257	
QEN71_RS28745	LysR family transcriptional regulator	QEN71_28740	paras_006281	protein-codi	NZ_CP1252	chromosom	6373703	6374662	+	960	0	16	2455	9	1847	
QEN71_RS28750	OmpA family protein	QEN71_28745	paras_006282	protein-codi	NZ_CP1252	chromosom	6374725	6376497	-	1773	0	16	2884	13	2647	
QEN71_RS28755	GMC family oxidoreductase N-terminal domain-cont	QEN71_28750	paras_006283	protein-codi	NZ_CP1252	chromosom	6376619	6378298	-	1680	0	42	15756	32	12966	
QEN71_RS28760	isovaleryl-CoA dehydrogenase	QEN71_28755	paras_006284	protein-codi	NZ_CP1252	chromosom	6378617	6380290	+	1674	0	24	6249	17	4527	
QEN71_RS28765	coniferyl aldehyde dehydrogenase	QEN71_28760	paras_006285	protein-codi	NZ_CP1252	chromosom	6380337	6381755	+	1419	0	28	6703	20	5576	
QEN71_RS28770	EscU/YscU/HrcU family type III secretion system ex	QEN71_28765	paras_006286	protein-codi	NZ_CP1252	chromosom	6381765	6382100	-	332	0	9	1849	7	1550	
QEN71_RS28775	flagellar hook-length control protein FliK	QEN71_28770	paras_006287	protein-codi	NZ_CP1252	chromosom	6382097	6383479	-	1379	0	9	1405	6	1045	
QEN71_RS28780	flagellar protein FliT	QEN71_28775	paras_006288	protein-codi	NZ_CP1252	chromosom	6383548	6383877	-	326	0	6	679	1	8	
QEN71_RS28785	flagellar export chaperone FliS	QEN71_28780	paras_006289	protein-codi	NZ_CP1252	chromosom	6383874	6384308	-	431	0	21	14315	19	13973	
QEN71_RS28790	flagellar hook-basal body complex protein FliE	QEN71_28785	paras_006290	protein-codi	NZ_CP1252	chromosom	6384655	6384990	-	336	0	2	286	0	0	
QEN71_RS28795	flagellar basal-body MS-ring/collar protein FliF	QEN71_28790	paras_006291	protein-codi	NZ_CP1252	chromosom	6385228	6387012	+	1774	0	52	32225	45	31066	
QEN71_RS28800	flagellar motor switch protein FliG	QEN71_28795	paras_006292	protein-codi	NZ_CP1252	chromosom	6387002	6387997	+	971	0	17	5064	10	3252	
QEN71_RS28805	flagellar assembly protein FliH	QEN71_28800	paras_006293	protein-codi	NZ_CP1252	chromosom	6387984	6388664	+	660	0	13	1835	9	1509	
QEN71_RS28810	flagellar protein export ATPase FliI	QEN71_28805	paras_006294	protein-codi	NZ_CP1252	chromosom	6388658	6390301	+	1637	0	26	4396	17	2526	
QEN71_RS28815	flagellar export protein FliJ	QEN71_28810	paras_006295	protein-codi	NZ_CP1252	chromosom	6390316	6390765	+	450	0	12	3092	9	2982	
QEN71_RS28820	flagellar hook-length control protein FliK	QEN71_28815	paras_006296	protein-codi	NZ_CP1252	chromosom	6390830	6392347	+	1518	0	11	1229	6	877	
QEN71_RS28825	flagellar basal body-associated protein FliL	QEN71_28820	paras_006297	protein-codi	NZ_CP1252	chromosom	6393034	6393537	+	504	0	8	3998	8	3998	
QEN71_RS28830	flagellar motor switch protein FliM	QEN71_28825	paras_006298	protein-codi	NZ_CP1252	chromosom	6393603	6394601	+	991	0	21	9549	18	9078	
QEN71_RS28835	flagellar motor switch protein FliN	QEN71_28830	paras_006299	protein-codi	NZ_CP1252	chromosom	6394594	6395058	+	457	0	3	276	1	26	
QEN71_RS28840	flagellar biosynthetic protein FliO	QEN71_28835	paras_006300	protein-codi	NZ_CP1252	chromosom	6395124	6395681	+	558	0	14	5102	10	3607	
QEN71_RS28845	flagellar type III secretion system pore protein FliP	QEN71_28840	paras_006301	protein-codi	NZ_CP1252	chromosom	6395792	6396562	+	771	0	12	4974	11	4626	
QEN71_RS28850	flagellar biosynthesis protein FliQ	QEN71_28845	paras_006302	protein-codi	NZ_CP1252	chromosom	6396590	6396859	+	270	0	7	1885	7	1885	
QEN71_RS28855	flagellar biosynthetic protein FliR	QEN71_28850	paras_006303	protein-codi	NZ_CP1252	chromosom	6396885	6397667	+	783	0	27	6993	22	5763	
QEN71_RS28860	flagellar hook-associated protein FlgL	QEN71_28855	paras_006304	protein-codi	NZ_CP1252	chromosom	6398308	6399528	-	1221	0	40	11298	26	8821	
QEN71_RS28865	flagellar hook-associated protein FlgK	QEN71_28860	paras_006305	protein-codi	NZ_CP1252	chromosom	6399543	6401516	-	1974	0	68	15202	51	12365	
QEN71_RS28870	hypothetical protein	QEN71_28865		protein-codi	NZ_CP1252	chromosom	6401579	6401803	+	225	134.0	8	1974	4	1227	
QEN71_RS28875	flagellar brake protein	QEN71_28870	paras_006306	protein-codi	NZ_CP1252	chromosom	6402290	6403039	-	750	0	29	7271	20	5193	
QEN71_RS28880	flagellar assembly peptidoglycan hydrolase FlgJ	QEN71_28875	paras_006307	protein-codi	NZ_CP1252	chromosom	6403410	6404342	-	933	0	18	1112	16	1095	
QEN71_RS28885	flagellar basal body P-ring protein FlgI	QEN71_28880	paras_006308	protein-codi	NZ_CP1252	chromosom	6404355	6405536	-	1182	0	10	1958	6	789	
QEN71_RS28890	flagellar basal body L-ring protein FlgH	QEN71_28885	paras_006309	protein-codi	NZ_CP1252	chromosom	6405539	6406249	-	711	0	15	2643	9	1222	
QEN71_RS28895	flagellar basal-body rod protein FlgG	QEN71_28890	paras_006310	protein-codi	NZ_CP1252	chromosom	6406285	6407073	-	789	0	15	1495	8	718	
QEN71_RS28900	flagellar basal-body rod protein FlgF	QEN71_28895	paras_006311	protein-codi	NZ_CP1252	chromosom	6407125	6407883	-	759	0	13	2199	6	396	
QEN71_RS28905	flagellar hook protein FlgE	QEN71_28900	paras_006312	protein-codi	NZ_CP1252	chromosom	6407906	6409414	-	1509	0	37	6384	28	4769	
QEN71_RS28910	flagellar hook assembly protein FlgD	QEN71_28905	paras_006313	protein-codi	NZ_CP1252	chromosom	6409448	6410158	-	711	0	12	1693	3	996	
QEN71_RS28915	flagellar basal body rod protein FlgC	QEN71_28910	paras_006314	protein-codi	NZ_CP1252	chromosom	6410170	6410595	-	426	0	9	1578	9	1578	

QEN71_RS28920	flagellar basal body rod protein FlgB	QEN71_28915	paras_006315	protein-codi	NZ_CP1252	chromosom	6410703	6411194	-	492	0	2	307	2	307	
QEN71_RS28925	flagellar basal body P-ring formation chaperone Flg	QEN71_28920	paras_006316	protein-codi	NZ_CP1252	chromosom	6411434	6412738	+	1305	0	13	2285	11	2243	
QEN71_RS28930	flagellar biosynthesis anti-sigma factor FlgM	QEN71_28925	paras_006317	protein-codi	NZ_CP1252	chromosom	6412845	6413183	+	339	0	3	1018	2	966	
QEN71_RS28935	flagellar protein FlgN	QEN71_28930	paras_006318	protein-codi	NZ_CP1252	chromosom	6413281	6413727	+	447	0	11	1423	7	1313	
QEN71_RS28940	RNA polymerase sigma factor FliA	QEN71_28935	paras_006319	protein-codi	NZ_CP1252	chromosom	6414518	6415252	-	735	0	28	2770	19	1318	
QEN71_RS28945	AAA family ATPase	QEN71_28940	paras_006320	protein-codi	NZ_CP1252	chromosom	6415275	6416144	-	862	0	14	2344	9	1308	
QEN71_RS28950	flagellar biosynthesis protein FlhF	QEN71_28945	paras_006321	protein-codi	NZ_CP1252	chromosom	6416137	6418020	-	1876	0	24	7761	16	3552	
QEN71_RS28955	flagellar biosynthesis protein FlhA	QEN71_28950	paras_006322	protein-codi	NZ_CP1252	chromosom	6418352	6420451	-	2096	0	20	3990	12	2400	
QEN71_RS28960	flagellar biosynthesis protein FlhB	QEN71_28955	paras_006323	protein-codi	NZ_CP1252	chromosom	6420448	6421656	-	1205	0	19	5215	14	3776	
QEN71_RS28965	DUF3443 domain-containing protein	QEN71_28960	paras_006324	protein-codi	NZ_CP1252	chromosom	6421857	6423119	-	1263	0	39	14369	30	12122	
QEN71_RS28970	DUF2844 domain-containing protein	QEN71_28965	paras_006325	protein-codi	NZ_CP1252	chromosom	6423136	6423693	-	558	0	22	5911	20	5037	
QEN71_RS28975	protein phosphatase CheZ	QEN71_28970	paras_006326	protein-codi	NZ_CP1252	chromosom	6423981	6424700	-	720	0	8	2876	7	2861	
QEN71_RS28980	chemotaxis response regulator CheY	QEN71_28975	paras_006327	protein-codi	NZ_CP1252	chromosom	6424702	6425097	-	396	0	7	1019	6	905	
QEN71_RS28985	chemotaxis response regulator protein-glutamate m	QEN71_28980	paras_006328	protein-codi	NZ_CP1252	chromosom	6425171	6426271	-	1101	0	26	9487	21	7107	
QEN71_RS28990	chemoreceptor glutamine deamidase CheD	QEN71_28985	paras_006329	protein-codi	NZ_CP1252	chromosom	6426277	6427053	-	773	0	15	2965	8	902	
QEN71_RS28995	CheR family methyltransferase	QEN71_28990	paras_006330	protein-codi	NZ_CP1252	chromosom	6427050	6427991	-	938	0	16	5920	15	5553	
QEN71_RS29000	methyl-accepting chemotaxis protein	QEN71_28995	paras_006331	protein-codi	NZ_CP1252	chromosom	6428240	6430039	-	1800	0	8	1930	6	1678	
QEN71_RS29005	chemotaxis protein CheW	QEN71_29000	paras_006332	protein-codi	NZ_CP1252	chromosom	6430116	6430643	-	528	0	10	2335	8	2064	
QEN71_RS29010	chemotaxis protein CheA	QEN71_29005	paras_006333	protein-codi	NZ_CP1252	chromosom	6430684	6432999	-	2316	0	29	9538	23	7489	
QEN71_RS29015	response regulator	QEN71_29010	paras_006334	protein-codi	NZ_CP1252	chromosom	6433073	6433435	-	363	0	3	101	3	101	
QEN71_RS29020	flagellar motor protein MotB	QEN71_29015	paras_006335	protein-codi	NZ_CP1252	chromosom	6433505	6434617	-	1113	0	13	3969	11	3846	
QEN71_RS29025	flagellar motor stator protein MotA	QEN71_29020	paras_006336	protein-codi	NZ_CP1252	chromosom	6434630	6435490	-	861	0	21	9598	13	7596	
QEN71_RS29030	flagellar transcriptional regulator FlhC	QEN71_29025	paras_006337	protein-codi	NZ_CP1252	chromosom	6435662	6436273	-	612	0	19	9337	19	9337	
QEN71_RS29035	flagellar transcriptional regulator FlhD	QEN71_29030	paras_006338	protein-codi	NZ_CP1252	chromosom	6436403	6436723	-	321	0	11	5131	10	4816	
QEN71_RS29040	glycosyltransferase family 4 protein	QEN71_29035	paras_006339	protein-codi	NZ_CP1252	chromosom	6437399	6438463	+	1065	0	27	9073	15	4857	
QEN71_RS29045	H-NS histone family protein	QEN71_29040	paras_006340	protein-codi	NZ_CP1252	chromosom	6438766	6439068	-	303	0	13	2902	9	1186	
QEN71_RS29050	aquaporin Z	QEN71_29045	paras_006341	protein-codi	NZ_CP1252	chromosom	6439358	6440107	+	750	0	28	8660	17	6331	
QEN71_RS29055	hypothetical protein	QEN71_29050	paras_006342	protein-codi	NZ_CP1252	chromosom	6440477	6440779	+	303	0	16	4852	14	4162	
QEN71_RS29060	Cof-type HAD-IIB family hydrolase	QEN71_29055	paras_006343	protein-codi	NZ_CP1252	chromosom	6440868	6441689	-	822	0	33	15328	27	12601	
QEN71_RS29065	BadF/BadG/BcrA/BcrD ATPase family protein	QEN71_29060	paras_006344	protein-codi	NZ_CP1252	chromosom	6441803	6442687	-	885	0	18	4757	16	4237	
QEN71_RS29070	DNA-3-methyladenine glycosylase I	QEN71_29065	paras_006345	protein-codi	NZ_CP1252	chromosom	6442839	6443438	+	600	0	19	5793	15	4762	
QEN71_RS29075	hypothetical protein	QEN71_29070	paras_006346	protein-codi	NZ_CP1252	chromosom	6443889	6444314	+	426	0	26	12032	23	11821	
QEN71_RS29080	30S ribosomal protein S21	QEN71_29075	paras_006347	protein-codi	NZ_CP1252	chromosom	6444460	6444672	-	213	0	2	491	0	0	
QEN71_RS29085	aldo/keto reductase	QEN71_29080	paras_006348	protein-codi	NZ_CP1252	chromosom	6444972	6445913	-	942	0	27	9517	24	8220	
QEN71_RS29090	flagellin domain-containing protein	QEN71_29085	paras_006349	protein-codi	NZ_CP1252	chromosom	6446158	6446976	+	819	463.0	10	3046	4	1241	
QEN71_RS29095	flagellar filament capping protein FliD	QEN71_29090	paras_006350	protein-codi	NZ_CP1252	chromosom	6447146	6448573	+	1428	0	24	5559	15	3785	
QEN71_RS29100	flagellar protein FliT	QEN71_29095	paras_006351	protein-codi	NZ_CP1252	chromosom	6448599	6448895	+	297	0	3	492	1	44	
QEN71_RS29105	glycosyltransferase family 41 protein	QEN71_29100	paras_006352	protein-codi	NZ_CP1252	chromosom	6449045	6451486	-	2442	0	69	14488	56	11787	
QEN71_RS29110	tetratricopeptide repeat protein	QEN71_29105	paras_006353	protein-codi	NZ_CP1252	chromosom	6451502	6453874	-	2373	0	55	12164	43	8408	
QEN71_RS29115	class I SAM-dependent methyltransferase	QEN71_29110	paras_006354	protein-codi	NZ_CP1252	chromosom	6453894	6455099	-	1202	0	13	1209	11	1032	
QEN71_RS29120	SDR family oxidoreductase	QEN71_29115	paras_006355	protein-codi	NZ_CP1252	chromosom	6455096	6456037	-	934	0	25	3954	22	3826	
QEN71_RS29125	tetratricopeptide repeat protein	QEN71_29120	paras_006356	protein-codi	NZ_CP1252	chromosom	6456034	6457914	-	1873	0	44	12527	41	11271	
QEN71_RS29130	tetratricopeptide repeat protein	QEN71_29125	paras_006357	protein-codi	NZ_CP1252	chromosom	6457911	6460037	-	2123	0	29	7598	27	7015	
QEN71_RS29135	glycosyltransferase	QEN71_29130	paras_006358	protein-codi	NZ_CP1252	chromosom	6460072	6460761	-	690	0	30	6195	18	4023	
QEN71_RS29140	methyltransferase domain-containing protein	QEN71_29135	paras_006359	protein-codi	NZ_CP1252	chromosom	6461207	6462910	+	1704	0	40	8687	29	7168	
QEN71_RS29145	DegT/DnrJ/EryC1/StrS family aminotransferase	QEN71_29140	paras_006360	protein-codi	NZ_CP1252	chromosom	6463278	6464354	+	1077	0	37	8515	30	7212	
QEN71_RS29150	hypothetical protein	QEN71_29145	paras_006361	protein-codi	NZ_CP1252	chromosom	6464417	6465403	+	987	0	27	3699	22	3374	
QEN71_RS29155	WbqC family protein	QEN71_29150	paras_006362	protein-codi	NZ_CP1252	chromosom	6465412	6466131	-	720	0	31	3782	24	3293	
QEN71_RS29160	DegT/DnrJ/EryC1/StrS family aminotransferase	QEN71_29155	paras_006363	protein-codi	NZ_CP1252	chromosom	6466133	6467314	-	1178	0	32	4877	28	4461	
QEN71_RS29165	aminotransferase class I/II-fold pyridoxal phosphate	QEN71_29160	paras_006364	protein-codi	NZ_CP1252	chromosom	6467311	6468507	-	1193	0	30	5573	25	4830	
QEN71_RS29170	class I SAM-dependent methyltransferase	QEN71_29165	paras_006365	protein-codi	NZ_CP1252	chromosom	6468530	6469222	-	693	0	23	4025	11	2456	
QEN71_RS29175	tRNA-Arg	QEN71_29170		tRNA	NZ_CP1252	chromosom	6469605	6469680	-	76	0	0	0	0	0	
QEN71_RS29180	c-type cytochrome	QEN71_29175	paras_006367	protein-codi	NZ_CP1252	chromosom	6470034	6470993	+	960	0	38	14540	30	12098	
QEN71_RS29185	UvrD-helicase domain-containing protein	QEN71_29180	paras_006368	protein-codi	NZ_CP1252	chromosom	6471076	6473163	-	2088	0	54	7766	45	5955	
QEN71_RS29190	oxidoreductase	QEN71_29185	paras_006369	protein-codi	NZ_CP1252	chromosom	6473505	6474554	+	1050	0	30	14786	29	14779	
QEN71_RS29195	hypothetical protein	QEN71_29190	paras_006370	protein-codi	NZ_CP1252	chromosom	6474767	6475126	+	360	0	9	5588	4	1874	
QEN71_RS29200	hypothetical protein	QEN71_29195	paras_006371	protein-codi	NZ_CP1252	chromosom	6475145	6475963	-	819	0	34	19389	16	6717	
QEN71_RS29205	glycine cleavage system aminomethyltransferase G	QEN71_29200	paras_006372	protein-codi	NZ_CP1252	chromosom	6476583	6477701	+	1119	0	27	10264	19	7534	
QEN71_RS29210	glycine cleavage system protein GcvH	QEN71_29205	paras_006373	protein-codi	NZ_CP1252	chromosom	6477816	6478196	+	381	0	11	3532	6	2768	
QEN71_RS29215	aminomethyl-transferring glycine dehydrogenase	QEN71_29210	paras_006374	protein-codi	NZ_CP1252	chromosom	6478261	6481191	+	2931	0	75	24196	62	22940	
QEN71_RS29220	alginate lyase family protein	QEN71_29215	paras_006375	protein-codi	NZ_CP1252	chromosom	6481328	6482464	+	1137	0	33	9761	28	8730	
QEN71_RS29225	L-serine ammonia-lyase	QEN71_29220	paras_006376	protein-codi	NZ_CP1252	chromosom	6482586	6483974	+	1389	0	31	7071	23	5610	

QEN71_RS29230	thiamine pyrophosphate-binding protein	QEN71_29225	paras_006377	protein-codi	NZ_CP1252	chromosom	6484049	6485749	+	1701	0	44	13702	35	11361	
QEN71_RS29235	branched-chain amino acid ABC transporter substra	QEN71_29230	paras_006378	protein-codi	NZ_CP1252	chromosom	6486524	6487672	-	1149	0	41	12595	26	8726	
QEN71_RS29240	trifunctional transcriptional regulator/proline dehydro	QEN71_29235	paras_006379	protein-codi	NZ_CP1252	chromosom	6487761	6491702	-	3942	0	95	30960	80	27560	
QEN71_RS29245	hypothetical protein	QEN71_29240	paras_006380	protein-codi	NZ_CP1252	chromosom	6492010	6492168	+	159	0	2	328	2	328	
QEN71_RS29250	hypothetical protein	QEN71_29245	paras_006381	protein-codi	NZ_CP1252	chromosom	6492223	6492462	-	236	0	0	0	0	0	
QEN71_RS29255	putative sulfate exporter family transporter	QEN71_29250	paras_006382	protein-codi	NZ_CP1252	chromosom	6492459	6493883	-	1421	0	30	9847	17	5655	
QEN71_RS29260	primosomal protein N'	QEN71_29255	paras_006383	protein-codi	NZ_CP1252	chromosom	6494058	6496316	-	2259	0	10	1556	7	410	
QEN71_RS29265	uroporphyrinogen decarboxylase	QEN71_29260	paras_006384	protein-codi	NZ_CP1252	chromosom	6496759	6497862	-	1104	0	2	8	2	8	TRUE
QEN71_RS29270	transporter substrate-binding domain-containing pro	QEN71_29265	paras_006385	protein-codi	NZ_CP1252	chromosom	6498038	6498850	+	813	0	20	6952	14	3833	
QEN71_RS29275	AMP-binding protein	QEN71_29270	paras_006386	protein-codi	NZ_CP1252	chromosom	6499639	6501369	-	1731	0	45	13331	39	9976	
QEN71_RS29280	F0F1 ATP synthase subunit epsilon	QEN71_29275	paras_006387	protein-codi	NZ_CP1252	chromosom	6501569	6501994	-	426	0	2	371	1	22	
QEN71_RS29285	F0F1 ATP synthase subunit beta	QEN71_29280	paras_006388	protein-codi	NZ_CP1252	chromosom	6502072	6503466	-	1395	0	0	0	0	0	TRUE
QEN71_RS29290	F0F1 ATP synthase subunit gamma	QEN71_29285	paras_006389	protein-codi	NZ_CP1252	chromosom	6503558	6504445	-	888	0	2	14	2	14	TRUE
QEN71_RS29295	F0F1 ATP synthase subunit alpha	QEN71_29290	paras_006390	protein-codi	NZ_CP1252	chromosom	6504536	6506077	-	1542	0	2	41	1	29	TRUE
QEN71_RS29300	F0F1 ATP synthase subunit delta	QEN71_29295	paras_006391	protein-codi	NZ_CP1252	chromosom	6506136	6506675	-	540	0	0	0	0	0	TRUE
QEN71_RS29305	F0F1 ATP synthase subunit B	QEN71_29300	paras_006392	protein-codi	NZ_CP1252	chromosom	6506678	6507148	-	471	0	0	0	0	0	TRUE
QEN71_RS29310	F0F1 ATP synthase subunit C	QEN71_29305	paras_006393	protein-codi	NZ_CP1252	chromosom	6507285	6507554	-	270	0	0	0	0	0	
QEN71_RS29315	F0F1 ATP synthase subunit A	QEN71_29310	paras_006394	protein-codi	NZ_CP1252	chromosom	6507642	6508493	-	852	0	0	0	0	0	TRUE
QEN71_RS29320	ATP synthase subunit I	QEN71_29315	paras_006395	protein-codi	NZ_CP1252	chromosom	6508639	6509184	-	546	0	15	2816	12	2304	
QEN71_RS29325	sodium:proton antiporter	QEN71_29320	paras_006396	protein-codi	NZ_CP1252	chromosom	6509467	6510615	-	1149	0	26	5719	16	3433	
QEN71_RS29330	ParB/RepB/Spo0J family partition protein	QEN71_29325	paras_006397	protein-codi	NZ_CP1252	chromosom	6510627	6511517	-	891	0	3	754	0	0	
QEN71_RS29335	ParA family protein	QEN71_29330	paras_006398	protein-codi	NZ_CP1252	chromosom	6511556	6512326	-	771	0	3	376	1	3	TRUE
QEN71_RS29340	16S rRNA (guanine(527)-N(7))-methyltransferase R	QEN71_29335	paras_006399	protein-codi	NZ_CP1252	chromosom	6512375	6513052	-	674	0	10	937	9	780	
QEN71_RS29345	tRNA uridine-5-carboxymethylaminomethyl(34) synt	QEN71_29340	paras_006400	protein-codi	NZ_CP1252	chromosom	6513049	6515007	-	1955	0	16	276	14	245	
QEN71_RS29350	ABC transporter ATP-binding protein	QEN71_29345	paras_006401	protein-codi	NZ_CP1252	chromosom	6515184	6515945	-	761	0	14	5642	9	4773	
QEN71_RS29355	branched-chain amino acid ABC transporter ATP-bi	QEN71_29350	paras_006402	protein-codi	NZ_CP1252	chromosom	6515945	6517729	-	1784	0	67	23423	56	19380	
QEN71_RS29360	branched-chain amino acid ABC transporter permea	QEN71_29355	paras_006403	protein-codi	NZ_CP1252	chromosom	6517734	6518789	-	1056	0	37	14489	26	9534	
QEN71_RS29365	ABC transporter substrate-binding protein	QEN71_29360	paras_006404	protein-codi	NZ_CP1252	chromosom	6519010	6520170	-	1161	0	31	8629	28	8334	
QEN71_RS29370	ABC transporter substrate-binding protein	QEN71_29365	paras_006405	protein-codi	NZ_CP1252	chromosom	6520420	6521586	-	1167	0	29	8094	23	7251	
QEN71_RS29375	branched-chain amino acid ABC transporter permea	QEN71_29370	paras_006406	protein-codi	NZ_CP1252	chromosom	6522078	6523049	-	971	0	25	5167	21	3887	
QEN71_RS29380	branched-chain amino acid ABC transporter permea	QEN71_29375	paras_006407	protein-codi	NZ_CP1252	chromosom	6523049	6523933	-	884	0	20	2593	16	2246	
QEN71_RS29385	ABC transporter substrate-binding protein	QEN71_29380	paras_006408	protein-codi	NZ_CP1252	chromosom	6524072	6525286	-	1215	0	39	9633	32	8132	
QEN71_RS29390	ABC transporter ATP-binding protein	QEN71_29385	paras_006409	protein-codi	NZ_CP1252	chromosom	6525347	6526102	-	752	0	10	1583	7	1362	
QEN71_RS29395	ABC transporter ATP-binding protein	QEN71_29390	paras_006410	protein-codi	NZ_CP1252	chromosom	6526099	6526878	-	776	0	16	2633	6	429	
QEN71_RS29400	choline dehydrogenase	QEN71_29395	paras_006411	protein-codi	NZ_CP1252	chromosom	6527122	6528816	-	1695	0	45	7163	38	6185	
QEN71_RS29405	CoA-acylating methylmalonate-semialdehyde dehyd	QEN71_29400	paras_006412	protein-codi	NZ_CP1252	chromosom	6528859	6530376	-	1518	0	25	4192	14	2223	
QEN71_RS29410	LysR family transcriptional regulator	QEN71_29405	paras_006413	protein-codi	NZ_CP1252	chromosom	6530536	6531435	+	900	0	6	2581	6	2581	
QEN71_RS29415	class IV adenylate cyclase	QEN71_29410	paras_006414	protein-codi	NZ_CP1252	chromosom	6531695	6532225	+	531	0	19	7327	15	5992	
QEN71_RS29420	Lrp/AsnC family transcriptional regulator	QEN71_29415	paras_006415	protein-codi	NZ_CP1252	chromosom	6532245	6532736	-	492	0	4	529	4	529	
QEN71_RS29425	phenylalanine 4-monooxygenase	QEN71_29420	paras_006416	protein-codi	NZ_CP1252	chromosom	6532869	6533759	+	891	0	39	13256	36	10856	
QEN71_RS29430	4a-hydroxytetrahydrobiopterin dehydratase	QEN71_29425	paras_006417	protein-codi	NZ_CP1252	chromosom	6533876	6534172	+	297	0	9	1695	3	244	
QEN71_RS29435	DUF3717 domain-containing protein	QEN71_29430	paras_006418	protein-codi	NZ_CP1252	chromosom	6534268	6534498	-	231	0	16	5877	9	2840	
QEN71_RS29440	response regulator transcription factor	QEN71_29435	paras_006419	protein-codi	NZ_CP1252	chromosom	6534616	6535302	+	687	0	16	7712	13	6340	
QEN71_RS29445	sensor histidine kinase	QEN71_29440	paras_006420	protein-codi	NZ_CP1252	chromosom	6535506	6537005	+	1500	0	28	10347	16	7914	
QEN71_RS29450	SET domain-containing protein-lysine N-methyltrans	QEN71_29445	paras_006421	protein-codi	NZ_CP1252	chromosom	6537056	6537538	-	483	0	16	3456	16	3456	
QEN71_RS29455	NADH:flavin oxidoreductase/NADH oxidase	QEN71_29450	paras_006422	protein-codi	NZ_CP1252	chromosom	6537780	6538892	-	1113	0	29	6798	19	5339	
QEN71_RS29460	MOSC domain-containing protein	QEN71_29455	paras_006423	protein-codi	NZ_CP1252	chromosom	6538987	6539718	-	732	0	20	6068	18	5851	
QEN71_RS29465	glutamine amidotransferase	QEN71_29460	paras_006424	protein-codi	NZ_CP1252	chromosom	6539728	6540441	-	714	0	14	3005	12	2944	
QEN71_RS29470	MarR family winged helix-turn-helix transcriptional r	QEN71_29465	paras_006425	protein-codi	NZ_CP1252	chromosom	6540587	6541231	-	645	133.0	3	22	1	7	
QEN71_RS29475	YbdK family carboxylate-amine ligase	QEN71_29470	paras_006426	protein-codi	NZ_CP1252	chromosom	6541896	6543011	-	1116	0	28	10735	24	8022	
QEN71_RS29480	cation:proton antiporter	QEN71_29475	paras_006427	protein-codi	NZ_CP1252	chromosom	6543141	6544343	-	1203	0	30	10141	27	9921	
QEN71_RS29485	bifunctional tRNA (5-methylaminomethyl-2-thiouridi	QEN71_29480	paras_006428	protein-codi	NZ_CP1252	chromosom	6545237	6547204	-	1966	0	36	18559	27	9905	
QEN71_RS29490	hypothetical protein	QEN71_29485	paras_006429	protein-codi	NZ_CP1252	chromosom	6547203	6547508	+	304	0	12	4216	6	1442	
QEN71_RS29495	HU family DNA-binding protein	QEN71_29490	paras_006430	protein-codi	NZ_CP1252	chromosom	6547673	6547951	+	279	0	5	483	3	241	
QEN71_RS29500	GTP-binding protein	QEN71_29495	paras_006431	protein-codi	NZ_CP1252	chromosom	6548059	6549336	-	1278	0	15	3322	14	3233	
QEN71_RS29505	lytic transglycosylase domain-containing protein	QEN71_29500	paras_006432	protein-codi	NZ_CP1252	chromosom	6549474	6549929	-	456	0	20	7383	18	6881	
QEN71_RS29510	type II secretion system secretin GspD	QEN71_29505	paras_006433	protein-codi	NZ_CP1252	chromosom	6550447	6552825	+	2375	0	48	21705	40	17286	
QEN71_RS29515	type II secretion system ATPase GspE	QEN71_29510	paras_006434	protein-codi	NZ_CP1252	chromosom	6552822	6554393	+	1568	0	22	9324	16	4963	
QEN71_RS29520	type II secretion system inner membrane protein Gs	QEN71_29515	paras_006435	protein-codi	NZ_CP1252	chromosom	6554398	6555615	+	1218	0	23	7929	23	7929	
QEN71_RS29525	type II secretion system protein N	QEN71_29520	paras_006436	protein-codi	NZ_CP1252	chromosom	6555706	6556119	-	414	0	7	3491	5	601	
QEN71_RS29530	type II secretion system protein GspG	QEN71_29525	paras_006437	protein-codi	NZ_CP1252	chromosom	6556289	6556765	+	477	0	14	4131	11	2358	
QEN71_RS29535	GspH/FimT family pseudopilin	QEN71_29530	paras_006438	protein-codi	NZ_CP1252	chromosom	6556824	6557348	+	524	0	14	3930	9	2636	

QEN71_RS29540	type II secretion system minor pseudopilin GspI	QEN71_29535	paras_006439	protein-codi	NZ_CP1252	chromosom	6557348	6557779	+	408	0	2	274	2	274	
QEN71_RS29545	prepilin-type N-terminal cleavage/methylation doma	QEN71_29540	paras_006440	protein-codi	NZ_CP1252	chromosom	6557757	6558461	+	682	0	11	4980	11	4980	
QEN71_RS29550	type II secretion system minor pseudopilin GspK	QEN71_29545	paras_006441	protein-codi	NZ_CP1252	chromosom	6558471	6559574	+	1104	0	29	12638	18	9683	
QEN71_RS29555	type II secretion system protein GspL	QEN71_29550	paras_006442	protein-codi	NZ_CP1252	chromosom	6559603	6560991	+	1385	0	12	2320	11	2285	
QEN71_RS29560	type II secretion system protein M	QEN71_29555	paras_006443	protein-codi	NZ_CP1252	chromosom	6560988	6561491	+	500	0	1	4	1	4	TRUE
QEN71_RS29565	type II secretion system protein N	QEN71_29560	paras_006444	protein-codi	NZ_CP1252	chromosom	6561514	6562293	+	780	0	10	2859	6	1859	
QEN71_RS29570	efflux transporter outer membrane subunit	QEN71_29565	paras_006445	protein-codi	NZ_CP1252	chromosom	6562394	6563995	-	1602	0	29	8466	23	6425	
QEN71_RS29575	hypothetical protein	QEN71_29570	paras_006446	protein-codi	NZ_CP1252	chromosom	6564292	6564657	-	366	0	5	1177	3	708	
QEN71_RS29580	MarR family transcriptional regulator	pseudo;QEN71_29575		pseudogene	NZ_CP1252	chromosom	6564908	6565358	+	451	0	14	4014	8	2274	
QEN71_RS29585	DHA2 family efflux MFS transporter permease subu	pseudo;QEN71_29580		pseudogene	NZ_CP1252	chromosom	6565559	6567121	+	1563	0	36	14767	33	13728	
QEN71_RS29590	OmpA family protein	QEN71_29585	paras_006449	protein-codi	NZ_CP1252	chromosom	6567155	6567880	-	726	0	16	3086	12	2035	
QEN71_RS29595	hypothetical protein	QEN71_29590	paras_006450	protein-codi	NZ_CP1252	chromosom	6567984	6568460	-	477	0	14	3837	8	2658	
QEN71_RS29600	glutamate--cysteine ligase	QEN71_29595	paras_006451	protein-codi	NZ_CP1252	chromosom	6568697	6570310	-	1614	0	51	12135	45	10126	
QEN71_RS29605	DNA-3-methyladenine glycosylase	QEN71_29600	paras_006452	protein-codi	NZ_CP1252	chromosom	6570643	6571566	-	920	0	13	4758	5	1441	
QEN71_RS29610	bifunctional DNA-binding transcriptional regulator/O	QEN71_29605	paras_006453	protein-codi	NZ_CP1252	chromosom	6571563	6572693	-	1127	0	18	4640	13	3501	
QEN71_RS29615	VOC family protein	QEN71_29610	paras_006454	protein-codi	NZ_CP1252	chromosom	6573061	6573474	+	414	0	8	1477	5	303	
QEN71_RS29620	YaeQ family protein	QEN71_29615	paras_006455	protein-codi	NZ_CP1252	chromosom	6573530	6574108	-	579	0	17	6301	12	3892	
QEN71_RS29625	hypothetical protein	QEN71_29620	paras_006456	protein-codi	NZ_CP1252	chromosom	6574629	6574970	-	342	0	14	3597	11	2377	
QEN71_RS29630	LysE family translocator	QEN71_29625	paras_006457	protein-codi	NZ_CP1252	chromosom	6575511	6576137	+	627	0	19	3771	19	3771	
QEN71_RS29635	hypothetical protein	QEN71_29630	paras_006458	protein-codi	NZ_CP1252	chromosom	6576289	6576456	+	168	0	2	1315	0	0	
QEN71_RS29640	hypothetical protein	QEN71_29635		protein-codi	NZ_CP1252	chromosom	6576493	6576624	+	132	0	4	1833	2	235	
QEN71_RS29645	hypothetical protein	QEN71_29640	paras_006459	protein-codi	NZ_CP1252	chromosom	6576885	6577163	+	279	0	8	1895	6	809	
QEN71_RS29650	integrase arm-type DNA-binding domain-containing	QEN71_29645	paras_006460	protein-codi	NZ_CP1252	chromosom	6577400	6578578	-	1179	0	40	12075	29	8888	
QEN71_RS29655	tRNA uridine-5-carboxymethylaminomethyl(34) synt	QEN71_29650	paras_006461	protein-codi	NZ_CP1252	chromosom	6578693	6580093	-	1401	0	19	776	13	506	
QEN71_RS29660	transcriptional regulator	QEN71_29655	paras_006462	protein-codi	NZ_CP1252	chromosom	6580442	6580864	+	423	0	17	2543	13	2268	
QEN71_RS29665	membrane protein insertase YidC	QEN71_29660	paras_006463	protein-codi	NZ_CP1252	chromosom	6580966	6582618	-	1653	0	4	23	1	3	TRUE
QEN71_RS29670	membrane protein insertion efficiency factor YidD	QEN71_29665	paras_006464	protein-codi	NZ_CP1252	chromosom	6582649	6582879	-	231	0	12	4154	10	3315	
QEN71_RS29675	ribonuclease P protein component	QEN71_29670	paras_006465	protein-codi	NZ_CP1252	chromosom	6582953	6583462	-	510	0	2	1128	2	1128	
QEN71_RS29680	50S ribosomal protein L34	QEN71_29675	paras_006466	protein-codi	NZ_CP1252	chromosom	6583488	6583622	-	135	0	0	0	0	0	
QEN71_RS42640	helix-turn-helix domain-containing protein	QEN71_42630	paras_006467	protein-codi	NZ_CP1252	megaplasmi	609	2078	+	1470	0	65	13178	50	10219	
QEN71_RS42645	IS6 family transposase	pseudo;QEN71_42635		pseudogene	NZ_CP1252	megaplasmi	2214	2783	+	570	0	14	4335	12	4258	
QEN71_RS42650	hypothetical protein	QEN71_42640	paras_006469	protein-codi	NZ_CP1252	megaplasmi	3977	4396	+	420	0	3	772	3	772	
QEN71_RS42655	hypothetical protein	QEN71_42645	paras_006470	protein-codi	NZ_CP1252	megaplasmi	4571	4993	+	423	0	15	3683	11	3229	
QEN71_RS42660	hypothetical protein	QEN71_42650	paras_006471	protein-codi	NZ_CP1252	megaplasmi	5299	5595	+	293	0	12	3757	10	2687	
QEN71_RS42665	patatin-like phospholipase family protein	QEN71_42655	paras_006472	protein-codi	NZ_CP1252	megaplasmi	5592	6455	+	860	0	22	3522	16	2855	
QEN71_RS42670	transposase	partial;pseudo;QEN71_42660		pseudogene	NZ_CP1252	megaplasmi	6545	6850	+	306	184.0	8	1310	6	1166	
QEN71_RS42675	IS3 family transposase	QEN71_42665	paras_006474	pseudogene	NZ_CP1252	megaplasmi	6881	8115	-	1235	1422.0	19	492	15	360	
QEN71_RS42680	VOC family protein	QEN71_42670	paras_006475	protein-codi	NZ_CP1252	megaplasmi	8837	9385	+	549	0	30	11627	21	9293	
QEN71_RS42685	amino acid adenylation domain-containing protein	QEN71_42675	paras_006476	protein-codi	NZ_CP1252	megaplasmi	10298	20290	+	9993	0	325	129423	232	105427	
QEN71_RS42690	IS6 family transposase	partial;pseudo;QEN71_42680		pseudogene	NZ_CP1252	megaplasmi	20322	20417	+	96	0	0	0	0	0	
QEN71_RS42695	IS110 family transposase	QEN71_42685	paras_006478	protein-codi	NZ_CP1252	megaplasmi	20718	21743	-	1026	2022.0	18	123	11	67	
QEN71_RS42700	IS21-like element helper ATPase IstB	QEN71_42690	paras_006479	protein-codi	NZ_CP1252	megaplasmi	21891	22679	-	789	1510.0	7	173	5	35	
QEN71_RS42705	IS21 family transposase	partial;pseudo;QEN71_42695		pseudogene	NZ_CP1252	megaplasmi	22865	23725	+	861	368.0	37	7884	31	6981	
QEN71_RS42710	IS21 family transposase	QEN71_42700	paras_006481	protein-codi	NZ_CP1252	megaplasmi	23943	25463	+	1521	3018.0	10	60	7	36	
QEN71_RS42715	IS21-like element helper ATPase IstB	QEN71_42705	paras_006482	protein-codi	NZ_CP1252	megaplasmi	25469	26257	+	789	1559.0	4	24	3	21	
QEN71_RS42720	IS21 family transposase	partial;pseudo;QEN71_42710		pseudogene	NZ_CP1252	megaplasmi	26331	27011	+	681	219.0	23	1473	14	737	
QEN71_RS42725	IS21-like element helper ATPase IstB	pseudo;QEN71_42715		pseudogene	NZ_CP1252	megaplasmi	27024	27790	+	767	0	19	2327	14	1591	
QEN71_RS42730	IS21 family transposase	QEN71_42720	paras_006485	protein-codi	NZ_CP1252	megaplasmi	27918	28964	-	1047	1969.0	10	189	9	184	
QEN71_RS42735	IS6 family transposase	QEN71_42725	paras_006486	protein-codi	NZ_CP1252	megaplasmi	29034	29699	+	666	0	19	2598	14	2057	
QEN71_RS42740	hypothetical protein	QEN71_42730	paras_006487	protein-codi	NZ_CP1252	megaplasmi	29996	30253	+	258	0	4	638	4	638	
QEN71_RS42745	ATP-binding protein	partial;pseudo;QEN71_42735		pseudogene	NZ_CP1252	megaplasmi	30458	30616	-	159	0	4	420	3	412	
QEN71_RS42750	tyrosine-type recombinase/integrase	QEN71_42740	paras_006489	protein-codi	NZ_CP1252	megaplasmi	30669	31307	-	639	0	21	1802	16	1577	
QEN71_RS42755	IS3 family transposase	partial;pseudo;QEN71_42745		pseudogene	NZ_CP1252	megaplasmi	31474	31557	-	84	0	8	711	6	650	
QEN71_RS42760	transposase domain-containing protein	partial;pseudo;QEN71_42750		pseudogene	NZ_CP1252	megaplasmi	31593	31799	-	207	173.0	3	970	1	212	
QEN71_RS42765	IS5 family transposase	QEN71_42755	paras_006492	protein-codi	NZ_CP1252	megaplasmi	31816	32628	-	813	1603.0	2	33	1	5	
QEN71_RS42770	site-specific integrase	QEN71_42760	paras_006493	protein-codi	NZ_CP1252	megaplasmi	32832	33710	+	879	174.0	8	401	8	401	
QEN71_RS42775	transposase zinc-binding domain-containing protein	partial;pseudo;QEN71_42765		pseudogene	NZ_CP1252	megaplasmi	33719	33976	+	258	0	4	304	2	216	
QEN71_RS42780	transposase	QEN71_42770	paras_006495	protein-codi	NZ_CP1252	megaplasmi	34004	34546	+	542	0	8	1207	5	1021	
QEN71_RS42785	IS66 family insertion sequence element accessory p	QEN71_42775	paras_006496	protein-codi	NZ_CP1252	megaplasmi	34546	34899	+	353	0	16	1456	10	650	
QEN71_RS42790	IS66 family transposase	QEN71_42780	paras_006497	protein-codi	NZ_CP1252	megaplasmi	34988	36628	+	1641	0	58	9220	43	7317	
QEN71_RS42795	IS91 family transposase	partial;pseudo;QEN71_42785		pseudogene	NZ_CP1252	megaplasmi	36714	37676	+	963	139.0	26	3459	21	2547	
QEN71_RS42800	hypothetical protein	QEN71_42790	paras_006499	protein-codi	NZ_CP1252	megaplasmi	37958	38113	-	103	0	6	1410	4	996	

QEN71_RS42805	transposase domain-containing protein	partial;pseudo;QEN71_42795		pseudogene	NZ_CP1252	megaplasmi	38061	38261	+	148	293.0	5	3375	3	3142	
QEN71_RS42810	IS6 family transposase	partial;pseudo;QEN71_42800		pseudogene	NZ_CP1252	megaplasmi	38297	38542	-	246	0	11	2413	11	2413	
QEN71_RS42815	ATPase	partial;pseudo;QEN71_42805		pseudogene	NZ_CP1252	megaplasmi	38581	38772	+	192	0	0	0	0	0	
QEN71_RS42820	hypothetical protein	QEN71_42810	paras_006502	protein-codi	NZ_CP1252	megaplasmi	39477	45623	+	6136	0	204	61296	166	49191	
QEN71_RS42825	hypothetical protein	QEN71_42815	paras_006503	protein-codi	NZ_CP1252	megaplasmi	45613	46935	+	1312	0	52	10507	37	8806	
QEN71_RS42830	transposase	QEN71_42820	paras_006504	protein-codi	NZ_CP1252	megaplasmi	47045	47437	+	389	132.0	17	5581	15	5123	
QEN71_RS42835	IS66 family insertion sequence element accessory p	QEN71_42825	paras_006505	protein-codi	NZ_CP1252	megaplasmi	47434	47769	+	332	655.0	25	14004	22	13376	
QEN71_RS42840	hypothetical protein	QEN71_42830	paras_006506	protein-codi	NZ_CP1252	megaplasmi	47812	47952	+	141	111.0	2	12	1	3	
QEN71_RS42845	IS21 family transposase	QEN71_42835	paras_006507	protein-codi	NZ_CP1252	megaplasmi	47994	49010	+	1013	1283.0	23	3935	19	3046	
QEN71_RS42850	IS21-like element helper ATPase IstB	QEN71_42840	paras_006508	protein-codi	NZ_CP1252	megaplasmi	49007	49795	+	785	485.0	17	2364	13	2052	
QEN71_RS42855	IS21 family transposase	QEN71_42845	paras_006509	protein-codi	NZ_CP1252	megaplasmi	50022	51542	+	1521	3018.0	38	10503	32	8788	
QEN71_RS42860	IS21-like element helper ATPase IstB	QEN71_42850	paras_006510	protein-codi	NZ_CP1252	megaplasmi	51548	52336	+	789	1559.0	26	6003	23	5609	
QEN71_RS42865	IS21-like element helper ATPase IstB	QEN71_42855	paras_006511	protein-codi	NZ_CP1252	megaplasmi	52528	53286	-	759	0	14	1932	11	1275	
QEN71_RS42870	IS110 family transposase	QEN71_42860	paras_006512	protein-codi	NZ_CP1252	megaplasmi	53388	54425	+	1038	0	35	3378	28	3029	
QEN71_RS42875	IS21 family transposase	pseudo;QEN71_42865		pseudogene	NZ_CP1252	megaplasmi	54681	56258	-	1578	0	53	6782	40	4268	
QEN71_RS42880	IS66 family transposase	partial;pseudo;QEN71_42870		pseudogene	NZ_CP1252	megaplasmi	56551	57417	+	867	1439.0	33	12675	25	11772	
QEN71_RS42885	tyrosine-type recombinase/integrase	QEN71_42875	paras_006515	protein-codi	NZ_CP1252	megaplasmi	57533	58756	+	1224	0	41	5064	36	4420	
QEN71_RS42890	tyrosine-type recombinase/integrase	QEN71_42880	paras_006516	protein-codi	NZ_CP1252	megaplasmi	58957	59709	+	745	0	26	3628	23	2856	
QEN71_RS42895	tyrosine-type recombinase/integrase	QEN71_42885	paras_006517	protein-codi	NZ_CP1252	megaplasmi	59702	60700	+	991	0	26	2422	20	1979	
QEN71_RS42900	site-specific integrase	QEN71_42890	paras_006518	protein-codi	NZ_CP1252	megaplasmi	60975	61853	+	879	779.0	8	666	7	664	
QEN71_RS42905	IS91 family transposase	QEN71_42895	paras_006519	protein-codi	NZ_CP1252	megaplasmi	61862	63067	+	1206	2398.0	2	22	1	20	
QEN71_RS42910	IS66 family transposase	partial;pseudo;QEN71_42900		pseudogene	NZ_CP1252	megaplasmi	63443	63958	+	516	387.0	22	4756	12	3243	
QEN71_RS42915	IS4 family transposase	QEN71_42905	paras_006521	protein-codi	NZ_CP1252	megaplasmi	63967	65400	-	1434	2832.0	58	20035	50	18624	
QEN71_RS42920	hypothetical protein	QEN71_42910	paras_006522	protein-codi	NZ_CP1252	megaplasmi	65663	66070	-	408	0	26	6102	24	5098	
QEN71_RS42925	hypothetical protein	QEN71_42915	paras_006523	protein-codi	NZ_CP1252	megaplasmi	66786	68186	-	1401	147.0	31	7568	15	3197	
QEN71_RS42930	hypothetical protein	QEN71_42920	paras_006524	protein-codi	NZ_CP1252	megaplasmi	68394	68588	+	195	0	3	670	2	613	
QEN71_RS42935	sel1 repeat family protein	QEN71_42925	paras_006525	protein-codi	NZ_CP1252	megaplasmi	68649	69380	-	732	0	20	5011	15	4907	
QEN71_RS42940	CopG family transcriptional regulator	QEN71_42930	paras_006526	protein-codi	NZ_CP1252	megaplasmi	69435	69860	+	426	0	7	252	7	252	
QEN71_RS42945	PHB depolymerase family esterase	QEN71_42935	paras_006527	protein-codi	NZ_CP1252	megaplasmi	69905	70996	+	1092	0	21	2550	17	1968	
QEN71_RS42950	alkaline phosphatase family protein	QEN71_42940	paras_006528	protein-codi	NZ_CP1252	megaplasmi	71168	72091	-	924	0	20	3886	18	3087	
QEN71_RS42955	hypothetical protein	QEN71_42945	paras_006530	protein-codi	NZ_CP1252	megaplasmi	72637	73293	-	657	0	26	6379	17	5379	
QEN71_RS42960	MarR family winged helix-turn-helix transcriptional r	QEN71_42950	paras_006531	protein-codi	NZ_CP1252	megaplasmi	73880	74524	-	645	133.0	13	2684	7	838	
QEN71_RS42965	hypothetical protein	QEN71_42955	paras_006532	protein-codi	NZ_CP1252	megaplasmi	75518	75910	+	393	0	9	1245	5	1092	
QEN71_RS42970	sterol desaturase family protein			pseudogene	NZ_CP1252	megaplasmi	76392	76547	-	156	0	7	677	5	605	
QEN71_RS42975	MBL fold metallo-hydrolase	QEN71_42965	paras_006533	protein-codi	NZ_CP1252	megaplasmi	76557	77414	-	858	0	29	9600	23	6418	
QEN71_RS42980	hypothetical protein	QEN71_42970		protein-codi	NZ_CP1252	megaplasmi	77653	78417	-	765	386.0	26	9364	20	6587	
QEN71_RS42985	IS21 family transposase	QEN71_42975	paras_006535	protein-codi	NZ_CP1252	megaplasmi	78488	80011	+	1524	3027.0	26	20060	22	14993	
QEN71_RS42990	IS21-like element helper ATPase IstB	QEN71_42980	paras_006536	protein-codi	NZ_CP1252	megaplasmi	80018	80806	+	765	1515.0	13	15618	13	15618	
QEN71_RS42995	serine hydrolase	QEN71_42985	paras_006537	protein-codi	NZ_CP1252	megaplasmi	80783	81262	-	456	174.0	11	1080	10	977	
QEN71_RS43000	H-NS histone family protein	QEN71_42990	paras_006538	protein-codi	NZ_CP1252	megaplasmi	81716	82042	-	327	0	13	2840	9	1450	
QEN71_RS43005	H-NS family nucleoid-associated regulatory protein	QEN71_42995	paras_006539	protein-codi	NZ_CP1252	megaplasmi	82449	83195	+	747	0	22	5152	18	5024	
QEN71_RS43010	cold-shock protein	QEN71_43000	paras_006540	protein-codi	NZ_CP1252	megaplasmi	83347	83550	-	204	115.0	9	3026	1	79	
QEN71_RS43015	hypothetical protein	QEN71_43005		protein-codi	NZ_CP1252	megaplasmi	84226	84609	+	384	0	23	6669	21	6510	
QEN71_RS43020	hypothetical protein	QEN71_43010	paras_006541	protein-codi	NZ_CP1252	megaplasmi	84818	85276	-	459	0	17	5771	15	5685	
QEN71_RS43025	hypothetical protein	QEN71_43015	paras_006542	protein-codi	NZ_CP1252	megaplasmi	85901	86122	+	222	0	17	6826	10	3410	
QEN71_RS43030	hypothetical protein	QEN71_43020	paras_006543	protein-codi	NZ_CP1252	megaplasmi	86349	86597	-	249	427.0	2	646	2	646	
QEN71_RS43035	IS66 family transposase	pseudo;QEN71_43025		pseudogene	NZ_CP1252	megaplasmi	86803	88391	-	1589	281.0	76	29703	60	24175	
QEN71_RS43040	IS66 family insertion sequence element accessory p	QEN71_43030	paras_006545	protein-codi	NZ_CP1252	megaplasmi	88479	88832	-	353	0	9	1620	9	1620	
QEN71_RS43045	transposase	QEN71_43035	paras_006546	protein-codi	NZ_CP1252	megaplasmi	88832	89299	-	467	0	7	2227	7	2227	
QEN71_RS43050	DUF4158 domain-containing protein	QEN71_43040	paras_006547	protein-codi	NZ_CP1252	megaplasmi	89449	89955	-	507	153.0	30	11606	28	11032	
QEN71_RS43055	CBS domain-containing protein	partial;pseudo;QEN71_43045		pseudogene	NZ_CP1252	megaplasmi	90100	90309	-	210	0	0	0	0	0	
QEN71_RS43060	hypothetical protein	QEN71_43050	paras_006549	protein-codi	NZ_CP1252	megaplasmi	90645	90917	-	273	0	12	3809	7	1373	
QEN71_RS43065	SDR family oxidoreductase	QEN71_43055	paras_006550	protein-codi	NZ_CP1252	megaplasmi	91262	92281	+	1020	0	29	5251	18	2952	
QEN71_RS43070	YoaK family protein	QEN71_43060	paras_006551	protein-codi	NZ_CP1252	megaplasmi	92327	92995	+	669	0	21	4406	17	3879	
QEN71_RS43075	glycoside hydrolase family 65 protein	QEN71_43065	paras_006552	protein-codi	NZ_CP1252	megaplasmi	93018	95462	-	2437	0	91	20493	78	17195	
QEN71_RS43080	PfkB family carbohydrate kinase	QEN71_43070	paras_006553	protein-codi	NZ_CP1252	megaplasmi	95455	96411	-	949	0	34	6793	26	5791	
QEN71_RS43085	aldose epimerase family protein	pseudo;QEN71_43075		pseudogene	NZ_CP1252	megaplasmi	96491	97634	-	1144	0	45	11091	45	11091	
QEN71_RS43090	AI-2E family transporter	partial;pseudo;QEN71_43080		pseudogene	NZ_CP1252	megaplasmi	97729	98286	-	558	0	9	2191	6	1288	
QEN71_RS43095	hypothetical protein			pseudogene	NZ_CP1252	megaplasmi	99346	99602	+	257	0	6	1720	4	1014	
QEN71_RS43100	hypothetical protein	QEN71_43090	paras_006556	protein-codi	NZ_CP1252	megaplasmi	99712	99966	+	255	0	18	8523	15	8436	
QEN71_RS43105	membrane-bound PQQ-dependent dehydrogenase,	QEN71_43095	paras_006557	pseudogene	NZ_CP1252	megaplasmi	101414	103857	+	2444	0	97	14746	70	11157	
QEN71_RS43110	hypothetical protein	QEN71_43100	paras_006558	protein-codi	NZ_CP1252	megaplasmi	104663	105859	-	1197	0	47	4044	41	3585	

QEN71_RS43115	cytochrome c3 family protein	QEN71_43105	paras_006559	protein-codi	NZ_CP1252	megaplasmi	106709	107359	+	651	0	16	1808	15	1791	
QEN71_RS43120	4Fe-4S dicluster domain-containing protein	QEN71_43110	paras_006560	protein-codi	NZ_CP1252	megaplasmi	107364	110228	+	2861	0	59	7417	42	5049	
QEN71_RS43125	polysulfide reductase NrfD	QEN71_43115	paras_006561	protein-codi	NZ_CP1252	megaplasmi	110225	111610	+	1378	0	39	3868	34	3231	
QEN71_RS43130	DUF3341 domain-containing protein	QEN71_43120	paras_006562	protein-codi	NZ_CP1252	megaplasmi	111607	112140	+	526	0	12	898	12	898	
QEN71_RS43135	cytochrome c	QEN71_43125	paras_006563	protein-codi	NZ_CP1252	megaplasmi	112137	112763	+	619	0	18	3482	16	3243	
QEN71_RS43140	hypothetical protein	QEN71_43130	paras_006564	protein-codi	NZ_CP1252	megaplasmi	112760	113848	+	1077	0	30	3215	24	2695	
QEN71_RS43145	hypothetical protein	QEN71_43135	paras_006565	protein-codi	NZ_CP1252	megaplasmi	113841	114275	+	427	0	8	701	6	669	
QEN71_RS43150	SCO family protein	QEN71_43140	paras_006566	protein-codi	NZ_CP1252	megaplasmi	114368	115153	+	782	0	20	1741	18	1709	
QEN71_RS43155	cytochrome c oxidase subunit II	QEN71_43145	paras_006567	protein-codi	NZ_CP1252	megaplasmi	115150	116100	+	927	0	26	3256	20	2303	
QEN71_RS43160	cytochrome c oxidase subunit I	QEN71_43150	paras_006568	protein-codi	NZ_CP1252	megaplasmi	116081	117703	+	1599	0	61	9017	52	7655	
QEN71_RS43165	cytochrome c oxidase subunit 3	QEN71_43155	paras_006569	protein-codi	NZ_CP1252	megaplasmi	117700	118341	+	634	0	17	2190	10	1210	
QEN71_RS43170	cytochrome C oxidase subunit IV family protein	QEN71_43160	paras_006570	protein-codi	NZ_CP1252	megaplasmi	118338	118679	+	338	0	11	1279	7	825	
QEN71_RS43175	ATP-binding protein	QEN71_43165	paras_006571	protein-codi	NZ_CP1252	megaplasmi	119108	121147	-	2040	0	34	5653	32	5541	
QEN71_RS43180	helix-turn-helix transcriptional regulator	QEN71_43170	paras_006572	protein-codi	NZ_CP1252	megaplasmi	121157	121435	-	279	0	14	2232	11	1374	
QEN71_RS43185	DUF1643 domain-containing protein	partial;pseudo;QEN71_43175		pseudogene	NZ_CP1252	megaplasmi	122173	122391	-	219	0	10	2425	7	2211	
QEN71_RS43190	hypothetical protein	QEN71_43180	paras_006574	protein-codi	NZ_CP1252	megaplasmi	122430	122672	+	243	0	11	1748	11	1748	
QEN71_RS43195	hypothetical protein	QEN71_43185	paras_006575	protein-codi	NZ_CP1252	megaplasmi	122775	123071	-	297	0	14	3575	12	2928	
QEN71_RS43200	hypothetical protein	QEN71_43190	paras_006576	protein-codi	NZ_CP1252	megaplasmi	123264	123482	+	219	0	9	1691	8	1435	
QEN71_RS43205	hypothetical protein	partial;pseudo;QEN71_43195		protein-codi	NZ_CP1252	megaplasmi	124135	125232	-	1098	123.0	23	2992	15	1018	
QEN71_RS43210	low affinity iron permease family protein	QEN71_43200	paras_006578	protein-codi	NZ_CP1252	megaplasmi	125494	126036	-	543	0	18	3852	14	3009	
QEN71_RS43215	NUDIX domain-containing protein	QEN71_43205	paras_006579	pseudogene	NZ_CP1252	megaplasmi	126147	126506	+	360	0	10	2499	8	2383	
QEN71_RS43220	PAS domain-containing hybrid sensor histidine kina	QEN71_43210	paras_006580	protein-codi	NZ_CP1252	megaplasmi	126626	128623	+	1998	0	46	9670	37	7934	
QEN71_RS43225	response regulator	QEN71_43215	paras_006581	protein-codi	NZ_CP1252	megaplasmi	128829	130127	-	1299	0	41	6989	28	5141	
QEN71_RS43230	hypothetical protein	QEN71_43220		protein-codi	NZ_CP1252	megaplasmi	130128	131006	-	879	0	37	10406	29	9601	
QEN71_RS43235	response regulator	QEN71_43225	paras_006582	protein-codi	NZ_CP1252	megaplasmi	131151	131990	-	840	0	23	8167	20	7658	
QEN71_RS43240	sensor domain-containing diguanylate cyclase	QEN71_43230	paras_006583	protein-codi	NZ_CP1252	megaplasmi	132388	133797	+	1410	0	72	37660	52	31901	
QEN71_RS43245	hypothetical protein	QEN71_43235	paras_006584	protein-codi	NZ_CP1252	megaplasmi	133849	134094	-	246	0	13	3301	11	2781	
QEN71_RS43250	sigma-54 dependent transcriptional regulator	QEN71_43240	paras_006585	protein-codi	NZ_CP1252	megaplasmi	134477	135868	+	1392	776.0	26	10659	18	7763	
QEN71_RS43255	branched-chain amino acid ABC transporter permease			pseudogene	NZ_CP1252	megaplasmi	136232	136324	-	93	0	3	291	3	291	
QEN71_RS43260	response regulator	QEN71_43250	paras_006586	protein-codi	NZ_CP1252	megaplasmi	136632	136979	+	348	0	12	1454	10	1259	
QEN71_RS43265	type II toxin-antitoxin system RelE/ParE family toxin	QEN71_43255	paras_006587	protein-codi	NZ_CP1252	megaplasmi	137328	137666	-	338	0	11	3177	6	2601	
QEN71_RS43270	type II toxin-antitoxin system Phd/YefM family antito	QEN71_43260	paras_006588	protein-codi	NZ_CP1252	megaplasmi	137666	137950	-	284	0	4	173	2	161	
QEN71_RS43275	DUF1819 family protein	QEN71_43265	paras_006589	protein-codi	NZ_CP1252	megaplasmi	138854	139405	+	548	0	8	2194	6	1721	
QEN71_RS43280	DUF1788 domain-containing protein	QEN71_43270	paras_006590	protein-codi	NZ_CP1252	megaplasmi	139402	139980	+	575	0	11	2553	5	278	
QEN71_RS43285	BREX system P-loop protein BrxC	QEN71_43275	paras_006591	protein-codi	NZ_CP1252	megaplasmi	139996	143652	+	3657	0	65	12684	48	9438	
QEN71_RS43290	BREX-1 system adenine-specific DNA-methyltransfe	QEN71_43280	paras_006592	protein-codi	NZ_CP1252	megaplasmi	143678	147226	+	3541	0	94	26896	78	21070	
QEN71_RS43295	BREX-1 system phosphatase PglZ type A	QEN71_43285	paras_006593	protein-codi	NZ_CP1252	megaplasmi	147219	149837	+	2611	0	41	7569	33	6394	
QEN71_RS43300	protease Lon-related BREX system protein BrxL	QEN71_43290	paras_006594	protein-codi	NZ_CP1252	megaplasmi	149846	151915	+	2070	0	65	12361	54	10148	
QEN71_RS43305	hypothetical protein	QEN71_43295	paras_006595	protein-codi	NZ_CP1252	megaplasmi	151937	152473	+	537	0	15	1992	13	1946	
QEN71_RS43310	hypothetical protein	QEN71_43300	paras_006596	protein-codi	NZ_CP1252	megaplasmi	152794	154614	+	1821	0	61	4463	47	3714	
QEN71_RS43315	helix-turn-helix transcriptional regulator	QEN71_43305	paras_006597	protein-codi	NZ_CP1252	megaplasmi	155469	155864	-	396	0	12	1379	12	1379	
QEN71_RS43320	AAA family ATPase	QEN71_43310	paras_006598	protein-codi	NZ_CP1252	megaplasmi	156155	157738	-	1584	0	66	8215	48	5566	
QEN71_RS43325	hypothetical protein	QEN71_43315	paras_006599	protein-codi	NZ_CP1252	megaplasmi	158174	158575	+	402	0	16	2467	12	1751	
QEN71_RS43330	hypothetical protein	QEN71_43320	paras_006600	protein-codi	NZ_CP1252	megaplasmi	158990	159571	+	582	0	23	2932	17	2187	
QEN71_RS43335	hypothetical protein	QEN71_43325	paras_006601	protein-codi	NZ_CP1252	megaplasmi	159711	159932	+	222	0	10	2181	4	399	
QEN71_RS43340	hypothetical protein	QEN71_43330	paras_006602	protein-codi	NZ_CP1252	megaplasmi	160270	160908	+	639	0	24	2490	17	1919	
QEN71_RS43345	hypothetical protein	QEN71_43335	paras_006603	protein-codi	NZ_CP1252	megaplasmi	160978	161955	-	978	0	38	4984	30	4448	
QEN71_RS43350	hypothetical protein	QEN71_43340	paras_006604	protein-codi	NZ_CP1252	megaplasmi	162578	163048	-	471	0	17	5413	14	4686	
QEN71_RS43355	DUF746 domain-containing protein	QEN71_43345	paras_006605	protein-codi	NZ_CP1252	megaplasmi	163112	164557	-	1446	0	23	5019	17	4371	
QEN71_RS43360	AAA family ATPase	QEN71_43350	paras_006606	protein-codi	NZ_CP1252	megaplasmi	164775	166232	+	1458	0	54	19764	43	16402	
QEN71_RS43365	hypothetical protein	QEN71_43355	paras_006607	protein-codi	NZ_CP1252	megaplasmi	166434	166985	+	552	0	39	21191	19	5312	
QEN71_RS43370	helix-turn-helix domain-containing protein	partial;pseudo;QEN71_43360		pseudogene	NZ_CP1252	megaplasmi	167357	167905	+	549	0	8	1202	8	1202	
QEN71_RS43375	HU family DNA-binding protein	QEN71_43365	paras_006609	protein-codi	NZ_CP1252	megaplasmi	168108	168386	-	279	0	1	1234	0	0	
QEN71_RS43380	hypothetical protein	QEN71_43370	paras_006610	protein-codi	NZ_CP1252	megaplasmi	168691	169227	-	537	0	34	11146	31	9834	
QEN71_RS43385	RES family NAD+ phosphorylase	QEN71_43375	paras_006611	protein-codi	NZ_CP1252	megaplasmi	170333	170833	-	497	0	21	7337	18	5782	
QEN71_RS43390	DUF2384 domain-containing protein	QEN71_43380	paras_006612	protein-codi	NZ_CP1252	megaplasmi	170830	171351	-	518	0	17	3879	10	1982	
QEN71_RS43395	hypothetical protein	QEN71_43385	paras_006613	protein-codi	NZ_CP1252	megaplasmi	171494	171898	+	405	0	11	7389	11	7389	
QEN71_RS43400	hypothetical protein	QEN71_43390	paras_006614	protein-codi	NZ_CP1252	megaplasmi	172032	172628	+	597	0	29	9165	22	7884	
QEN71_RS43405	hypothetical protein	QEN71_43395	paras_006615	protein-codi	NZ_CP1252	megaplasmi	172809	173567	-	759	0	35	16255	33	15810	
QEN71_RS43410	IS110 family transposase	QEN71_43400	paras_006616	protein-codi	NZ_CP1252	megaplasmi	173845	174870	-	1026	2022.0	51	15940	37	12347	
QEN71_RS43415	IS21-like element helper ATPase IstB	QEN71_43405	paras_006617	protein-codi	NZ_CP1252	megaplasmi	175018	175857	-	836	1543.0	17	2201	15	1918	
QEN71_RS43420	IS21 family transposase	QEN71_43410	paras_006618	protein-codi	NZ_CP1252	megaplasmi	175854	176900	-	1043	1969.0	41	7550	35	6790	

QEN71_RS43425	hypothetical protein	QEN71_43415	paras_006619	protein-codi	NZ_CP1252	megaplasmi	176966	177199	-	234	0	9	1814	4	555	
QEN71_RS43430	hypothetical protein	QEN71_43420	paras_006620	protein-codi	NZ_CP1252	megaplasmi	177684	178079	-	396	0	14	2811	13	2628	
QEN71_RS43435	hypothetical protein	QEN71_43425	paras_006621	protein-codi	NZ_CP1252	megaplasmi	178241	178606	-	366	0	10	1534	6	741	
QEN71_RS43440	hypothetical protein	QEN71_43430	paras_006622	protein-codi	NZ_CP1252	megaplasmi	178656	178892	-	237	0	9	906	9	906	
QEN71_RS43445	hypothetical protein	QEN71_43435	paras_006623	protein-codi	NZ_CP1252	megaplasmi	178925	179416	-	492	0	16	2619	14	2182	
QEN71_RS43450	hypothetical protein	QEN71_43440	paras_006624	protein-codi	NZ_CP1252	megaplasmi	180054	180239	-	186	0	8	796	6	782	
QEN71_RS43455	hypothetical protein	QEN71_43445	paras_006625	protein-codi	NZ_CP1252	megaplasmi	180506	181264	-	759	0	26	2894	19	2303	
QEN71_RS43460	hypothetical protein	QEN71_43450	paras_006626	protein-codi	NZ_CP1252	megaplasmi	181295	181615	-	321	0	3	501	3	501	
QEN71_RS43465	hypothetical protein	QEN71_43455	paras_006628	protein-codi	NZ_CP1252	megaplasmi	181929	182222	-	294	0	5	747	5	747	
QEN71_RS43470	hypothetical protein	QEN71_43460	paras_006629	protein-codi	NZ_CP1252	megaplasmi	182269	182499	-	231	0	9	1170	8	1009	
QEN71_RS43475	IS5 family transposase	QEN71_43465	paras_006630	protein-codi	NZ_CP1252	megaplasmi	182990	183802	-	813	1603.0	22	2973	15	1959	
QEN71_RS43480	hypothetical protein	QEN71_43470	paras_006631	protein-codi	NZ_CP1252	megaplasmi	183845	184144	-	300	0	11	1167	8	852	
QEN71_RS43485	hypothetical protein	QEN71_43475	paras_006632	protein-codi	NZ_CP1252	megaplasmi	184193	184819	-	627	0	32	4330	25	3749	
QEN71_RS43490	hypothetical protein	QEN71_43480	paras_006633	protein-codi	NZ_CP1252	megaplasmi	184888	185274	-	387	0	18	2326	10	979	
QEN71_RS43495	hypothetical protein	QEN71_43485	paras_006634	protein-codi	NZ_CP1252	megaplasmi	185807	186085	-	279	0	12	2669	11	2661	
QEN71_RS43500	hypothetical protein	QEN71_43490	paras_006635	protein-codi	NZ_CP1252	megaplasmi	186146	186520	-	375	0	16	3123	10	2260	
QEN71_RS43505	hypothetical protein	QEN71_43495	paras_006636	protein-codi	NZ_CP1252	megaplasmi	186537	187037	-	501	0	21	2491	15	1536	
QEN71_RS43510	hypothetical protein	QEN71_43500	paras_006637	protein-codi	NZ_CP1252	megaplasmi	187055	187267	-	213	0	10	1427	9	1420	
QEN71_RS43515	hypothetical protein	QEN71_43505	paras_006638	protein-codi	NZ_CP1252	megaplasmi	187285	187533	-	245	0	5	292	4	285	
QEN71_RS43520	hypothetical protein	QEN71_43510	paras_006639	protein-codi	NZ_CP1252	megaplasmi	187530	187931	-	398	0	4	805	4	805	
QEN71_RS43525	hypothetical protein	QEN71_43515	paras_006640	protein-codi	NZ_CP1252	megaplasmi	187969	188466	-	498	0	15	2068	10	1288	
QEN71_RS43530	hypothetical protein	QEN71_43520	paras_006641	protein-codi	NZ_CP1252	megaplasmi	189050	189277	-	228	0	6	657	3	307	
QEN71_RS43535	hypothetical protein	QEN71_43525	paras_006642	protein-codi	NZ_CP1252	megaplasmi	189295	189516	-	222	0	6	1649	3	1301	
QEN71_RS43540	hypothetical protein	QEN71_43530	paras_006643	protein-codi	NZ_CP1252	megaplasmi	189546	189884	-	339	0	10	2079	7	1883	
QEN71_RS43545	type II toxin-antitoxin system PemK/MazF family tox	QEN71_43535	paras_006644	protein-codi	NZ_CP1252	megaplasmi	190327	190647	-	314	0	3	152	3	152	
QEN71_RS43550	AbrB/MazE/SpoVT family DNA-binding domain-cont	QEN71_43540	paras_006645	protein-codi	NZ_CP1252	megaplasmi	190641	190889	-	242	0	4	72	2	51	
QEN71_RS43555	tyrosine-type recombinase/integrase	QEN71_43545	paras_006646	protein-codi	NZ_CP1252	megaplasmi	191017	191382	-	366	0	14	2756	9	2310	
QEN71_RS43560	site-specific integrase	partial;pseudo;QEN71_43550		pseudogene	NZ_CP1252	megaplasmi	191447	191689	-	243	0	9	482	8	467	
QEN71_RS43565	site-specific integrase	QEN71_43555	paras_006647	protein-codi	NZ_CP1252	megaplasmi	192247	193185	+	939	0	25	2944	18	2118	
QEN71_RS43570	hypothetical protein	QEN71_43560	paras_006648	protein-codi	NZ_CP1252	megaplasmi	193298	193753	+	456	0	22	4996	12	2165	
QEN71_RS43575	hypothetical protein	QEN71_43565	paras_006649	protein-codi	NZ_CP1252	megaplasmi	193816	194091	-	276	0	8	1759	8	1759	
QEN71_RS43580	hypothetical protein	QEN71_43570	paras_006650	protein-codi	NZ_CP1252	megaplasmi	194334	194792	+	459	0	15	3812	5	2501	
QEN71_RS43585	translesion DNA synthesis-associated protein ImuA	QEN71_43575	paras_006651	protein-codi	NZ_CP1252	megaplasmi	194949	195656	+	637	0	14	2755	11	2409	
QEN71_RS43590	DNA polymerase Y family protein	QEN71_43580	paras_006652	protein-codi	NZ_CP1252	megaplasmi	195586	197058	+	1402	0	23	6791	13	5184	
QEN71_RS43595	error-prone DNA polymerase	QEN71_43585	paras_006653	protein-codi	NZ_CP1252	megaplasmi	197062	200181	+	3120	0	86	17191	77	16521	
QEN71_RS43600	DUF2471 family protein	QEN71_43590	paras_006654	protein-codi	NZ_CP1252	megaplasmi	200204	200503	+	300	0	4	730	0	0	
QEN71_RS43605	DNA ligase	QEN71_43595	paras_006655	protein-codi	NZ_CP1252	megaplasmi	200900	201538	-	625	0	7	1779	5	1302	
QEN71_RS43610	hypothetical protein	QEN71_43600	paras_006656	protein-codi	NZ_CP1252	megaplasmi	201525	201797	-	259	0	8	1378	8	1378	
QEN71_RS43615	SOS response-associated peptidase family protein	QEN71_43605	paras_006657	protein-codi	NZ_CP1252	megaplasmi	201813	202616	+	804	0	24	4960	17	3707	
QEN71_RS43620	phosphoadenosine phosphosulfate reductase family	QEN71_43610	paras_006658	protein-codi	NZ_CP1252	megaplasmi	202712	204520	-	1809	0	53	6771	37	4470	
QEN71_RS43625	hypothetical protein	QEN71_43615	paras_006659	protein-codi	NZ_CP1252	megaplasmi	204588	205415	-	828	0	16	2326	13	1944	
QEN71_RS43630	hypothetical protein	QEN71_43620	paras_006660	protein-codi	NZ_CP1252	megaplasmi	205957	206142	-	186	0	6	1388	5	1301	
QEN71_RS43635	SMC family ATPase	QEN71_43625	paras_006661	protein-codi	NZ_CP1252	megaplasmi	206218	208539	-	2309	0	38	4165	30	3286	
QEN71_RS43640	hypothetical protein	QEN71_43630	paras_006662	protein-codi	NZ_CP1252	megaplasmi	208527	209060	-	521	0	5	341	3	52	
QEN71_RS43645	metallophosphatase family protein	QEN71_43635	paras_006663	protein-codi	NZ_CP1252	megaplasmi	209278	210609	-	1332	0	18	1710	14	979	
QEN71_RS43650	NADAR family protein	QEN71_43640	paras_006664	protein-codi	NZ_CP1252	megaplasmi	210689	211171	-	483	0	9	568	6	330	
QEN71_RS43655	hypothetical protein	QEN71_43645	paras_006665	protein-codi	NZ_CP1252	megaplasmi	211184	211393	-	210	0	4	432	0	0	
QEN71_RS43660	hypothetical protein	QEN71_43650	paras_006666	protein-codi	NZ_CP1252	megaplasmi	211562	212872	-	1307	0	31	5262	25	4326	
QEN71_RS43665	hypothetical protein	QEN71_43655	paras_006667	protein-codi	NZ_CP1252	megaplasmi	212869	213135	-	263	0	0	0	0	0	
QEN71_RS43670	DUF1845 domain-containing protein	QEN71_43660	paras_006668	protein-codi	NZ_CP1252	megaplasmi	213792	214394	+	603	0	14	1580	11	1428	
QEN71_RS43675	hypothetical protein	QEN71_43665	paras_006669	protein-codi	NZ_CP1252	megaplasmi	214469	215377	+	909	0	17	3957	15	3700	
QEN71_RS43680	polymer-forming cytoskeletal protein	QEN71_43670	paras_006670	protein-codi	NZ_CP1252	megaplasmi	215391	215867	+	477	0	12	2523	9	2308	
QEN71_RS43685	hypothetical protein	QEN71_43675	paras_006671	protein-codi	NZ_CP1252	megaplasmi	215887	216183	+	293	0	3	319	3	319	
QEN71_RS43690	hypothetical protein	QEN71_43680	paras_006672	protein-codi	NZ_CP1252	megaplasmi	216180	216698	+	515	0	12	2811	9	2736	
QEN71_RS43695	ATP-dependent helicase	QEN71_43685	paras_006673	protein-codi	NZ_CP1252	megaplasmi	216882	218606	-	1725	0	46	11219	43	10635	
QEN71_RS43700	ATPase	QEN71_43690	paras_006674	protein-codi	NZ_CP1252	megaplasmi	218617	219447	-	831	0	11	1093	7	744	
QEN71_RS43705	DUF4400 domain-containing protein	QEN71_43695	paras_006675	protein-codi	NZ_CP1252	megaplasmi	219780	220394	-	607	0	14	2623	13	2489	
QEN71_RS43710	hypothetical protein	QEN71_43700	paras_006676	protein-codi	NZ_CP1252	megaplasmi	220387	220641	-	203	0	6	632	6	632	
QEN71_RS43715	hypothetical protein	QEN71_43705	paras_006677	protein-codi	NZ_CP1252	megaplasmi	220598	221116	-	475	0	9	2050	3	268	
QEN71_RS43720	conjugative transfer system coupling protein TraD	QEN71_43710	paras_006678	protein-codi	NZ_CP1252	megaplasmi	221126	222997	-	1868	0	55	11171	41	6354	
QEN71_RS43725	TraI domain-containing protein	QEN71_43715	paras_006679	protein-codi	NZ_CP1252	megaplasmi	222994	224355	-	1354	0	12	1484	9	1271	
QEN71_RS43730	type IV secretion system protein	QEN71_43720	paras_006680	protein-codi	NZ_CP1252	megaplasmi	224352	225227	-	872	0	21	2551	21	2551	

QEN71_RS43735	type VI secretion protein	QEN71_43725	paras_006681	protein-codi	NZ_CP1252	megaplasmi	225359	226123	-	765	0	13	1772	10	1354	
QEN71_RS43740	type IV secretion system protein VirB4	QEN71_43730	paras_006682	protein-codi	NZ_CP1252	megaplasmi	226164	228626	-	2456	0	46	5354	36	4439	
QEN71_RS43745	VirB3 family type IV secretion system protein	QEN71_43735	paras_006683	protein-codi	NZ_CP1252	megaplasmi	228620	228928	-	302	0	18	2918	16	2554	
QEN71_RS43750	conjugal transfer protein TrbC	QEN71_43740	paras_006684	protein-codi	NZ_CP1252	megaplasmi	228930	229283	-	354	0	6	639	6	639	
QEN71_RS43755	ATPase, T2SS/T4P/T4SS family	QEN71_43745	paras_006685	protein-codi	NZ_CP1252	megaplasmi	229298	230347	-	1050	0	20	2263	11	1066	
QEN71_RS43760	VWA-like domain-containing protein	QEN71_43750	paras_006686	protein-codi	NZ_CP1252	megaplasmi	230688	231977	-	1290	0	34	6305	25	5197	
QEN71_RS43765	hypothetical protein	QEN71_43755	paras_006687	protein-codi	NZ_CP1252	megaplasmi	231988	232899	-	912	0	23	7666	20	7577	
QEN71_RS43770	ATP-binding protein	QEN71_43760	paras_006688	protein-codi	NZ_CP1252	megaplasmi	233019	234044	-	1026	0	21	2552	13	1252	
QEN71_RS43775	hypothetical protein	QEN71_43765	paras_006689	protein-codi	NZ_CP1252	megaplasmi	234311	235642	-	1332	0	21	9688	15	5171	
QEN71_RS43780	hypothetical protein	QEN71_43770	paras_006690	protein-codi	NZ_CP1252	megaplasmi	235740	236198	-	459	0	13	2086	11	1816	
QEN71_RS43785	hypothetical protein	QEN71_43775	paras_006691	protein-codi	NZ_CP1252	megaplasmi	236432	236896	-	461	0	18	5196	9	2711	
QEN71_RS43790	hypothetical protein	QEN71_43780	paras_006692	protein-codi	NZ_CP1252	megaplasmi	236893	237285	-	389	0	14	3166	10	2961	
QEN71_RS43795	hypothetical protein	QEN71_43785	paras_006693	protein-codi	NZ_CP1252	megaplasmi	237368	237829	-	462	0	7	2510	4	1476	
QEN71_RS43800	hypothetical protein	QEN71_43790		protein-codi	NZ_CP1252	megaplasmi	237861	238184	-	324	0	6	3213	6	3213	
QEN71_RS43805	hypothetical protein	QEN71_43795	paras_006694	protein-codi	NZ_CP1252	megaplasmi	238317	238601	-	285	0	8	1944	3	944	
QEN71_RS43810	response regulator	QEN71_43800	paras_006695	protein-codi	NZ_CP1252	megaplasmi	239954	240442	-	489	0	17	5089	14	4647	
QEN71_RS43815	hybrid sensor histidine kinase/response regulator	QEN71_43805	paras_006696	protein-codi	NZ_CP1252	megaplasmi	240522	241556	-	1035	0	30	9351	21	7913	
QEN71_RS43820	PAS domain-containing protein	QEN71_43810		protein-codi	NZ_CP1252	megaplasmi	241782	242102	-	321	0	13	8073	9	3473	
QEN71_RS43825	hypothetical protein	QEN71_43815	paras_006697	protein-codi	NZ_CP1252	megaplasmi	242828	243097	-	270	0	11	1762	11	1762	
QEN71_RS43830	hypothetical protein	QEN71_43820	paras_006698	protein-codi	NZ_CP1252	megaplasmi	243393	243806	-	414	0	23	5078	18	3304	
QEN71_RS43835	phosphoadenosine phosphosulfate reductase famil	QEN71_43825	paras_006699	protein-codi	NZ_CP1252	megaplasmi	244005	245204	+	1200	0	18	3294	16	2882	
QEN71_RS43840	hypothetical protein	QEN71_43830	paras_006700	protein-codi	NZ_CP1252	megaplasmi	245271	245600	+	330	0	10	1956	4	1043	
QEN71_RS43845	site-specific integrase	QEN71_43835	paras_006701	protein-codi	NZ_CP1252	megaplasmi	246217	247368	+	1120	0	43	7775	38	7458	
QEN71_RS43850	tyrosine-type recombinase/integrase	QEN71_43840	paras_006702	protein-codi	NZ_CP1252	megaplasmi	247337	248998	+	1626	0	57	8342	48	7394	
QEN71_RS43855	tyrosine-type recombinase/integrase	QEN71_43845	paras_006703	protein-codi	NZ_CP1252	megaplasmi	248995	250914	+	1912	0	63	9869	55	7897	
QEN71_RS43860	TetR family transcriptional regulator	QEN71_43850	paras_006704	protein-codi	NZ_CP1252	megaplasmi	250911	251411	+	497	0	12	812	11	810	
QEN71_RS43865	DUF5131 family protein	QEN71_43855	paras_006705	protein-codi	NZ_CP1252	megaplasmi	251415	252422	+	1008	0	36	7719	24	4448	
QEN71_RS43870	hypothetical protein	QEN71_43860	paras_006706	protein-codi	NZ_CP1252	megaplasmi	252521	253699	-	1179	0	45	4722	35	3626	
QEN71_RS43875	hypothetical protein	QEN71_43865	paras_006707	protein-codi	NZ_CP1252	megaplasmi	253704	254177	-	474	0	6	609	5	605	
QEN71_RS43880	PRTRC system ThiF family protein	QEN71_43870	paras_006708	protein-codi	NZ_CP1252	megaplasmi	254270	255088	-	815	0	23	2497	21	2276	
QEN71_RS43885	PRTRC system protein A	QEN71_43875	paras_006709	protein-codi	NZ_CP1252	megaplasmi	255085	255720	-	628	0	11	1601	10	1100	
QEN71_RS43890	PRTRC system protein B	QEN71_43880	paras_006710	protein-codi	NZ_CP1252	megaplasmi	255717	256448	-	728	0	21	2896	15	2011	
QEN71_RS43895	PRTRC system protein F	QEN71_43885	paras_006711	protein-codi	NZ_CP1252	megaplasmi	256459	257598	-	1140	0	22	2739	16	1901	
QEN71_RS43900	PRTRC system protein C	QEN71_43890	paras_006712	protein-codi	NZ_CP1252	megaplasmi	257695	257913	-	219	0	12	1940	7	983	
QEN71_RS43905	PRTRC system protein E	QEN71_43895	paras_006713	protein-codi	NZ_CP1252	megaplasmi	257923	258405	-	483	0	7	1120	5	570	
QEN71_RS43910	PRTRC system ParB family protein	QEN71_43900	paras_006714	protein-codi	NZ_CP1252	megaplasmi	258501	260177	-	1677	0	33	3694	25	3367	
QEN71_RS43915	hypothetical protein	QEN71_43905	paras_006715	protein-codi	NZ_CP1252	megaplasmi	260434	260682	-	245	0	1	66	0	0	
QEN71_RS43920	SNF2-related protein	QEN71_43910	paras_006716	protein-codi	NZ_CP1252	megaplasmi	260679	263798	-	3116	0	67	11256	55	10143	
QEN71_RS43925	hypothetical protein	QEN71_43915	paras_006717	protein-codi	NZ_CP1252	megaplasmi	263860	264657	-	798	0	29	9509	18	5570	
QEN71_RS43930	hypothetical protein	QEN71_43920	paras_006718	protein-codi	NZ_CP1252	megaplasmi	265306	265677	+	368	0	6	1251	6	1251	
QEN71_RS43935	hypothetical protein	QEN71_43925	paras_006719	protein-codi	NZ_CP1252	megaplasmi	265674	265952	+	271	0	15	3450	13	2986	
QEN71_RS43940	hypothetical protein	QEN71_43930	paras_006720	protein-codi	NZ_CP1252	megaplasmi	265949	266227	+	275	0	14	3646	8	2722	
QEN71_RS43945	DUF1173 family protein	QEN71_43935	paras_006721	protein-codi	NZ_CP1252	megaplasmi	266254	267465	+	1212	0	28	8236	17	4497	
QEN71_RS43950	hypothetical protein	QEN71_43940	paras_006722	protein-codi	NZ_CP1252	megaplasmi	267524	267997	+	474	0	17	5445	13	4411	
QEN71_RS43955	hypothetical protein	QEN71_43945	paras_006723	protein-codi	NZ_CP1252	megaplasmi	268051	268380	+	330	0	4	601	4	601	
QEN71_RS43960	hypothetical protein	QEN71_43950	paras_006724	protein-codi	NZ_CP1252	megaplasmi	268439	268831	+	393	0	18	6040	13	4160	
QEN71_RS43965	Lar family restriction alleviation protein	QEN71_43955	paras_006725	protein-codi	NZ_CP1252	megaplasmi	268938	269612	+	675	0	12	2763	9	1996	
QEN71_RS43970	hypothetical protein	QEN71_43960	paras_006726	protein-codi	NZ_CP1252	megaplasmi	269688	269828	+	141	0	12	2143	12	2143	
QEN71_RS43975	integrase domain-containing protein	QEN71_43965	paras_006727	protein-codi	NZ_CP1252	megaplasmi	270198	271328	-	1131	0	40	6936	27	5038	
QEN71_RS43980	PRTRC system protein D	QEN71_43970	paras_006728	protein-codi	NZ_CP1252	megaplasmi	272582	273601	+	1016	0	31	3866	28	3747	
QEN71_RS43985	hypothetical protein	QEN71_43975	paras_006729	protein-codi	NZ_CP1252	megaplasmi	273598	273990	+	389	0	7	2639	7	2639	
QEN71_RS43990	hypothetical protein	QEN71_43980	paras_006730	protein-codi	NZ_CP1252	megaplasmi	274090	274242	+	145	0	2	125	2	125	
QEN71_RS43995	hypothetical protein	QEN71_43985	paras_006731	protein-codi	NZ_CP1252	megaplasmi	274235	274465	+	223	0	7	790	1	42	
QEN71_RS44000	hypothetical protein	QEN71_43990	paras_006732	protein-codi	NZ_CP1252	megaplasmi	274537	274758	-	222	0	9	879	7	831	
QEN71_RS44005	primase-helicase zinc-binding domain-containing pr	QEN71_43995	paras_006733	protein-codi	NZ_CP1252	megaplasmi	274840	275958	-	1119	0	26	3960	22	3123	
QEN71_RS44010	hypothetical protein	QEN71_44000	paras_006734	protein-codi	NZ_CP1252	megaplasmi	276217	277044	-	828	0	17	6981	11	4930	
QEN71_RS44015	hypothetical protein	QEN71_44005	paras_006735	protein-codi	NZ_CP1252	megaplasmi	277053	277487	-	435	0	12	2872	9	1409	
QEN71_RS44020	hypothetical protein	QEN71_44010	paras_006736	protein-codi	NZ_CP1252	megaplasmi	277584	277913	-	330	0	14	1742	13	1605	
QEN71_RS44025	nuclease-related domain-containing protein	QEN71_44015	paras_006737	protein-codi	NZ_CP1252	megaplasmi	278290	278967	+	678	0	17	2384	10	1350	
QEN71_RS44030	hypothetical protein	QEN71_44020	paras_006738	protein-codi	NZ_CP1252	megaplasmi	279309	280592	+	1284	0	29	8912	17	6054	
QEN71_RS44035	FlhC family transcriptional regulator	QEN71_44025	paras_006739	protein-codi	NZ_CP1252	megaplasmi	280610	281842	+	1233	0	41	9827	35	8054	
QEN71_RS44040	hypothetical protein	QEN71_44030	paras_006740	protein-codi	NZ_CP1252	megaplasmi	281858	282028	+	171	0	3	365	3	365	

QEN71_RS44045	hypothetical protein	QEN71_44035	paras_006741	protein-codi	NZ_CP1252	megaplasmi	282030	282422	+	393	0	22	3884	21	3572	
QEN71_RS44050	CAP domain-containing protein	QEN71_44040	paras_006742	protein-codi	NZ_CP1252	megaplasmi	282592	283671	+	1080	0	48	13313	32	9553	
QEN71_RS44055	hypothetical protein	QEN71_44045	paras_006743	protein-codi	NZ_CP1252	megaplasmi	284423	285643	-	1221	0	48	9780	35	6970	
QEN71_RS44060	type 4 pilus major pilin	QEN71_44050	paras_006744	protein-codi	NZ_CP1252	megaplasmi	285656	286249	-	594	0	22	2165	18	1864	
QEN71_RS44065	type II secretion system protein	QEN71_44055	paras_006745	protein-codi	NZ_CP1252	megaplasmi	286306	287424	-	1119	0	34	3375	22	1781	
QEN71_RS44070	ATPase, T2SS/T4P/T4SS family	QEN71_44060	paras_006746	protein-codi	NZ_CP1252	megaplasmi	287433	289844	-	2412	0	61	8199	48	6652	
QEN71_RS44075	hypothetical protein	QEN71_44065	paras_006747	protein-codi	NZ_CP1252	megaplasmi	289849	290433	-	571	0	17	2217	14	2050	
QEN71_RS44080	hypothetical protein	QEN71_44070	paras_006748	protein-codi	NZ_CP1252	megaplasmi	290420	291697	-	1264	0	40	8761	31	6150	
QEN71_RS44085	type II secretory pathway protein	QEN71_44075	paras_006749	protein-codi	NZ_CP1252	megaplasmi	291706	293337	-	1632	0	50	8260	41	5842	
QEN71_RS44090	toxin co-regulated pilus biosynthesis Q family protei	QEN71_44080	paras_006750	protein-codi	NZ_CP1252	megaplasmi	293342	293962	-	621	0	17	1710	13	1465	
QEN71_RS44095	lytic transglycosylase domain-containing protein	QEN71_44085	paras_006751	protein-codi	NZ_CP1252	megaplasmi	293965	294393	-	425	0	23	5718	16	4476	
QEN71_RS44100	TrbG/VirB9 family P-type conjugative transfer protei	QEN71_44090	paras_006752	protein-codi	NZ_CP1252	megaplasmi	294390	295667	-	1270	0	36	6688	30	6034	
QEN71_RS44105	transglycosylase SLT domain-containing protein	QEN71_44095	paras_006753	protein-codi	NZ_CP1252	megaplasmi	295664	296470	-	795	0	21	2974	20	2947	
QEN71_RS44110	TrbI/VirB10 family protein	QEN71_44100	paras_006754	protein-codi	NZ_CP1252	megaplasmi	296463	297725	-	1255	0	34	5394	27	4486	
QEN71_RS44115	TrbG/VirB9 family P-type conjugative transfer protei	QEN71_44105	paras_006755	protein-codi	NZ_CP1252	megaplasmi	297728	298603	-	876	0	27	5732	22	4935	
QEN71_RS44120	type IV secretion system protein	QEN71_44110	paras_006756	protein-codi	NZ_CP1252	megaplasmi	298609	299319	-	711	0	22	4254	18	3235	
QEN71_RS44125	replication initiation protein	QEN71_44115	paras_006757	protein-codi	NZ_CP1252	megaplasmi	299942	301336	-	1395	0	3	761	1	6	TRUE
QEN71_RS44130	AAA family ATPase	QEN71_44120	paras_006758	protein-codi	NZ_CP1252	megaplasmi	302395	303600	+	1202	0	3	3538	0	0	
QEN71_RS44135	ParB/RepB/Spo0J family partition protein	QEN71_44125	paras_006759	protein-codi	NZ_CP1252	megaplasmi	303597	304586	+	986	0	2	708	0	0	
QEN71_RS44140	hypothetical protein	QEN71_44130	paras_006760	protein-codi	NZ_CP1252	megaplasmi	305271	306143	+	873	0	43	16680	27	10972	
QEN71_RS44145	hypothetical protein	QEN71_44135	paras_006762	protein-codi	NZ_CP1252	megaplasmi	307951	308181	-	231	0	4	711	2	375	
QEN71_RS44150	Mu transposase C-terminal domain-containing prote	QEN71_44140	paras_006763	protein-codi	NZ_CP1252	megaplasmi	309625	311250	+	1626	0	49	6566	37	5537	
QEN71_RS44155	TniB family NTP-binding protein	QEN71_44145	paras_006764	protein-codi	NZ_CP1252	megaplasmi	311257	312129	+	847	0	19	1705	18	1508	
QEN71_RS44160	TniQ family protein	QEN71_44150	paras_006765	protein-codi	NZ_CP1252	megaplasmi	312104	312634	+	505	0	20	2360	14	1165	
QEN71_RS44165	ATP-binding protein	partial;pseudo;QEN71_44155		pseudogene	NZ_CP1252	megaplasmi	312677	312859	-	183	0	4	771	0	0	
QEN71_RS44170	IS21 family transposase	QEN71_44160		pseudogene	NZ_CP1252	megaplasmi	313084	313443	+	360	105.0	9	872	8	767	
QEN71_RS44175	hypothetical protein	QEN71_44165		protein-codi	NZ_CP1252	megaplasmi	313646	314203	+	485	956.0	2	22	2	22	
QEN71_RS44180	tyrosine-type recombinase/integrase	partial;pseudo;QEN71_44170		pseudogene	NZ_CP1252	megaplasmi	314131	314349	+	142	281.0	1	21	1	21	
QEN71_RS44185	hypothetical protein	QEN71_44175	paras_006769	protein-codi	NZ_CP1252	megaplasmi	314346	315023	+	607	1194.0	11	929	10	927	
QEN71_RS44190	tyrosine-type recombinase/integrase	QEN71_44180	paras_006770	protein-codi	NZ_CP1252	megaplasmi	314957	315712	+	685	1352.0	13	1563	13	1563	
QEN71_RS44195	tyrosine-type recombinase/integrase	QEN71_44185	paras_006771	pseudogene	NZ_CP1252	megaplasmi	315709	316709	+	997	1953.0	22	2405	20	2322	
QEN71_RS44200	IS21 family transposase	partial;pseudo;QEN71_44190		pseudogene	NZ_CP1252	megaplasmi	316809	317920	+	1112	0	18	1921	9	1119	
QEN71_RS44205	IS21-like element helper ATPase IstB	QEN71_44195	paras_006773	protein-codi	NZ_CP1252	megaplasmi	317927	318715	+	789	0	19	2511	19	2511	
QEN71_RS44210	transposase			protein-codi	NZ_CP1252	megaplasmi	318836	318979	-	137	227.0	7	606	6	485	
QEN71_RS44215	IS5/IS1182 family transposase			pseudogene	NZ_CP1252	megaplasmi	318973	319113	-	134	258.0	4	573	4	573	
QEN71_RS44220	RolB family protein	QEN71_44210	paras_006775	protein-codi	NZ_CP1252	megaplasmi	319945	320592	-	648	0	42	5955	34	5078	
QEN71_RS44225	hypothetical protein	QEN71_44215	paras_006776	protein-codi	NZ_CP1252	megaplasmi	321164	321526	+	363	299.0	12	531	12	531	
QEN71_RS44230	IS110 family transposase	QEN71_44220	paras_006777	protein-codi	NZ_CP1252	megaplasmi	321606	322667	+	1062	2107.0	12	92	8	64	
QEN71_RS44235	IS5 family transposase	QEN71_44225	paras_006778	protein-codi	NZ_CP1252	megaplasmi	322975	324090	-	1116	2016.0	14	227	8	143	
QEN71_RS44240	transposase	partial;pseudo;QEN71_44230		pseudogene	NZ_CP1252	megaplasmi	324257	324631	-	375	0	6	831	3	228	
QEN71_RS44245	IS5 family transposase	QEN71_44235	paras_006780	protein-codi	NZ_CP1252	megaplasmi	324780	326102	+	1323	0	22	3815	21	3811	
QEN71_RS44250	transposase	partial;pseudo;QEN71_44240		pseudogene	NZ_CP1252	megaplasmi	326266	326376	-	111	0	1	96	1	96	
QEN71_RS44255	N-acetylmuramidase family protein	QEN71_44245	paras_006782	protein-codi	NZ_CP1252	megaplasmi	326483	327274	-	792	0	31	4306	20	2210	
QEN71_RS44260	IS3 family transposase	QEN71_44250	paras_006783	pseudogene	NZ_CP1252	megaplasmi	327411	328576	-	1166	0	47	6015	37	4684	
QEN71_RS44265	hypothetical protein	QEN71_44255	paras_006784	protein-codi	NZ_CP1252	megaplasmi	328667	329404	-	738	0	20	3360	16	2907	
QEN71_RS44270	caspase family protein	QEN71_44260	paras_006785	protein-codi	NZ_CP1252	megaplasmi	329487	330494	-	1008	0	41	4949	36	4436	
QEN71_RS44275	transposase	partial;pseudo;QEN71_44265		pseudogene	NZ_CP1252	megaplasmi	331591	331893	+	302	184.0	9	2634	7	2001	
QEN71_RS44280	IS110 family transposase	partial;pseudo;QEN71_44270		pseudogene	NZ_CP1252	megaplasmi	331893	332432	-	539	0	11	915	9	844	
QEN71_RS44285	IS110 family transposase	pseudo;QEN71_44275		pseudogene	NZ_CP1252	megaplasmi	332503	333524	+	1022	0	15	1002	14	955	
QEN71_RS44290	IS110 family transposase	partial;pseudo;QEN71_44280		pseudogene	NZ_CP1252	megaplasmi	333783	334046	-	264	0	10	1782	6	1247	
QEN71_RS44295	IS3 family transposase	partial;pseudo;QEN71_44285		pseudogene	NZ_CP1252	megaplasmi	334170	334316	+	147	191.0	6	867	2	120	
QEN71_RS44300	transposase	partial;pseudo;QEN71_44290		pseudogene	NZ_CP1252	megaplasmi	334434	334620	+	187	0	6	1068	2	38	
QEN71_RS44305	IS5 family transposase	pseudo;QEN71_44295		pseudogene	NZ_CP1252	megaplasmi	334759	335520	-	762	0	23	2416	12	873	
QEN71_RS44310	IS5/IS1182 family transposase	partial;pseudo;QEN71_44300		pseudogene	NZ_CP1252	megaplasmi	336395	336520	+	126	0	4	529	2	133	
QEN71_RS44315	IS3 family transposase	partial;pseudo;QEN71_44305		pseudogene	NZ_CP1252	megaplasmi	336557	337078	-	522	1027.0	38	16611	29	14800	
QEN71_RS44320	IS110 family transposase	partial;pseudo;QEN71_44310		pseudogene	NZ_CP1252	megaplasmi	337216	337974	+	759	1414.0	18	8495	11	3052	
QEN71_RS44325	IS3 family transposase	QEN71_44315	paras_006796	pseudogene	NZ_CP1252	megaplasmi	338036	339276	+	1241	2458.0	21	3796	16	2824	
QEN71_RS44330	IS110 family transposase	partial;pseudo;QEN71_44320		pseudogene	NZ_CP1252	megaplasmi	339315	339578	+	264	378.0	15	8086	15	8086	
QEN71_RS44335	IS110 family transposase	QEN71_44325	paras_006798	protein-codi	NZ_CP1252	megaplasmi	340079	341410	-	1332	2624.0	75	22687	53	15987	
QEN71_RS44340	IS21 family transposase	QEN71_44330	paras_006799	protein-codi	NZ_CP1252	megaplasmi	341669	343120	+	1448	367.0	25	3257	21	2639	
QEN71_RS44345	ATP-binding protein	partial;pseudo;QEN71_44335		pseudogene	NZ_CP1252	megaplasmi	343117	343635	+	515	105.0	2	54	0	0	
QEN71_RS44350	tyrosine-type recombinase/integrase	QEN71_44340	paras_006801	protein-codi	NZ_CP1252	megaplasmi	343721	344749	-	1025	909.0	33	3835	24	2498	

QEN71_RS44355	tyrosine-type recombinase/integrase	QEN71_44345	paras_006802	protein-codi	NZ_CP1252	megaplasmi	344746	345723	-	970	1052.0	28	2330	22	1763	
QEN71_RS44360	tyrosine-type recombinase/integrase	QEN71_44350	paras_006803	protein-codi	NZ_CP1252	megaplasmi	345720	346943	-	1220	1290.0	51	8594	43	6659	
QEN71_RS44365	IS91 family transposase	QEN71_44355	paras_006804	protein-codi	NZ_CP1252	megaplasmi	347433	348641	-	1209	0	21	3323	21	3323	
QEN71_RS44370	site-specific integrase	QEN71_44360	paras_006805	protein-codi	NZ_CP1252	megaplasmi	348651	349529	-	879	0	14	714	10	550	
QEN71_RS44375	ATP-binding protein	partial;pseudo;QEN71_44365		pseudogene	NZ_CP1252	megaplasmi	349724	350035	+	312	159.0	8	377	4	190	
QEN71_RS44380	IS3 family transposase	partial;pseudo;QEN71_44370		pseudogene	NZ_CP1252	megaplasmi	350107	350828	-	722	1426.0	17	2413	12	1887	
QEN71_RS44385	integrase			pseudogene	NZ_CP1252	megaplasmi	351082	351165	+	84	0	3	223	1	67	
QEN71_RS44390	site-specific integrase	QEN71_44380	paras_006808	protein-codi	NZ_CP1252	megaplasmi	351305	352315	-	1007	0	31	5026	29	4961	
QEN71_RS44395	tyrosine-type recombinase/integrase	QEN71_44385	paras_006809	protein-codi	NZ_CP1252	megaplasmi	352312	353244	-	929	0	38	3600	24	2273	
QEN71_RS44400	IS110 family transposase	QEN71_44390	paras_006811	protein-codi	NZ_CP1252	megaplasmi	353778	354908	+	1131	259.0	33	5281	21	3547	
QEN71_RS44405	IS5 family transposase	QEN71_44395	paras_006812	protein-codi	NZ_CP1252	megaplasmi	355209	356015	+	807	1170.0	13	756	7	487	
QEN71_RS44410	group II intron maturase-specific domain-containing	QEN71_44400	paras_006813	protein-codi	NZ_CP1252	megaplasmi	356122	356490	+	369	0	16	2176	12	1765	
QEN71_RS44415	transposase	partial;pseudo;QEN71_44405		pseudogene	NZ_CP1252	megaplasmi	356683	357028	-	346	0	9	2415	8	2313	
QEN71_RS44420	hypothetical protein			pseudogene	NZ_CP1252	megaplasmi	357133	357455	+	323	0	5	678	4	525	
QEN71_RS44425	hypothetical protein	QEN71_44415	paras_006815	protein-codi	NZ_CP1252	megaplasmi	358200	358721	+	522	0	42	9764	31	8681	
QEN71_RS44430	transposase	partial;pseudo;QEN71_44420		pseudogene	NZ_CP1252	megaplasmi	359248	359358	+	111	0	8	1601	8	1601	
QEN71_RS44435	tyrosine-type recombinase/integrase	QEN71_44425	paras_006816	protein-codi	NZ_CP1252	megaplasmi	359641	359808	-	168	0	10	1242	8	695	
QEN71_RS44440	ATP-binding protein	partial;pseudo;QEN71_44430		pseudogene	NZ_CP1252	megaplasmi	360268	360741	-	474	493.0	8	1089	6	857	
QEN71_RS44445	hypothetical protein	QEN71_44435	paras_006818	protein-codi	NZ_CP1252	megaplasmi	361036	361203	+	148	292.0	4	1161	4	1161	
QEN71_RS44450	recombinase family protein	QEN71_44440	paras_006819	protein-codi	NZ_CP1252	megaplasmi	361184	363241	+	2038	3497.0	64	12878	44	7522	
QEN71_RS44455	ATP-binding protein	partial;pseudo;QEN71_44445		pseudogene	NZ_CP1252	megaplasmi	363270	363602	-	329	435.0	10	1448	9	1235	
QEN71_RS44460	IS21 family transposase	QEN71_44450	paras_006821	protein-codi	NZ_CP1252	megaplasmi	363599	364615	-	1013	1283.0	19	3071	13	2194	
QEN71_RS44465	IS110 family transposase	QEN71_44455	paras_006822	protein-codi	NZ_CP1252	megaplasmi	365269	366294	-	1026	0	18	1643	16	1304	
QEN71_RS44470	transposase			pseudogene	NZ_CP1252	megaplasmi	366604	367422	-	819	0	22	2750	19	1977	
QEN71_RS44475	tyrosine-type recombinase/integrase	QEN71_44465	paras_006824	pseudogene	NZ_CP1252	megaplasmi	367473	368473	-	997	1953.0	14	2332	12	2314	
QEN71_RS44480	tyrosine-type recombinase/integrase	QEN71_44470	paras_006825	protein-codi	NZ_CP1252	megaplasmi	368470	369225	-	685	1352.0	16	1739	16	1739	
QEN71_RS44485	hypothetical protein	QEN71_44475	paras_006826	protein-codi	NZ_CP1252	megaplasmi	369159	369836	-	607	1194.0	15	2671	14	2593	
QEN71_RS44490	tyrosine-type recombinase/integrase	partial;pseudo;QEN71_44480		pseudogene	NZ_CP1252	megaplasmi	369833	370051	-	142	281.0	2	527	2	527	
QEN71_RS44495	hypothetical protein	QEN71_44485		protein-codi	NZ_CP1252	megaplasmi	369979	370536	-	485	956.0	8	2179	7	1531	
QEN71_RS44500	IS21 family transposase	partial;pseudo;QEN71_44490		pseudogene	NZ_CP1252	megaplasmi	370668	371786	+	1119	567.0	18	6789	15	6589	
QEN71_RS44505	IS21-like element helper ATPase IstB	QEN71_44495	paras_006829	protein-codi	NZ_CP1252	megaplasmi	371793	372581	+	789	248.0	18	6704	17	6701	
QEN71_RS44510	hypothetical protein	QEN71_44500	paras_006830	protein-codi	NZ_CP1252	megaplasmi	373406	373621	-	216	0	5	358	4	235	
QEN71_RS44515	IS110 family transposase	QEN71_44505	paras_006831	protein-codi	NZ_CP1252	megaplasmi	373999	375177	+	1179	195.0	21	4905	10	1937	
QEN71_RS44520	IS21 family transposase	partial;pseudo;QEN71_44510		pseudogene	NZ_CP1252	megaplasmi	375271	375474	+	204	0	3	436	3	436	
QEN71_RS44525	hypothetical protein	QEN71_44515		protein-codi	NZ_CP1252	megaplasmi	375629	376186	+	485	956.0	7	687	7	687	
QEN71_RS44530	tyrosine-type recombinase/integrase	partial;pseudo;QEN71_44520		pseudogene	NZ_CP1252	megaplasmi	376114	376332	+	142	281.0	1	175	1	175	
QEN71_RS44535	hypothetical protein	QEN71_44525	paras_006834	protein-codi	NZ_CP1252	megaplasmi	376329	376520	+	184	241.0	2	276	1	219	
QEN71_RS44540	IS21 family transposase	partial;pseudo;QEN71_44530		pseudogene	NZ_CP1252	megaplasmi	376517	377194	+	674	0	10	636	7	454	
QEN71_RS44545	tyrosine-type recombinase/integrase	pseudo;QEN71_44535		pseudogene	NZ_CP1252	megaplasmi	377717	378561	+	841	0	13	1904	11	1846	
QEN71_RS44550	tyrosine-type recombinase/integrase	QEN71_44540	paras_006837	protein-codi	NZ_CP1252	megaplasmi	378558	379148	+	579	0	13	1088	10	778	
QEN71_RS44555	tyrosine-type recombinase/integrase	QEN71_44545	paras_006838	protein-codi	NZ_CP1252	megaplasmi	379141	380136	+	988	0	20	2839	16	2538	
QEN71_RS44560	site-specific integrase	QEN71_44550	paras_006839	protein-codi	NZ_CP1252	megaplasmi	380403	381281	+	879	1257.0	28	6266	20	5065	
QEN71_RS44565	IS91 family transposase	QEN71_44555	paras_006840	protein-codi	NZ_CP1252	megaplasmi	381290	382495	+	1206	2398.0	31	8058	28	6718	
QEN71_RS44570	hypothetical protein	QEN71_44560		protein-codi	NZ_CP1252	megaplasmi	382805	383287	+	483	0	19	4618	11	1656	
QEN71_RS44575	hypothetical protein	QEN71_44565	paras_006841	protein-codi	NZ_CP1252	megaplasmi	383349	383699	+	334	0	4	582	4	582	
QEN71_RS44580	ATP-binding protein	pseudo;QEN71_44570		pseudogene	NZ_CP1252	megaplasmi	383683	384436	+	733	0	17	1303	14	1193	
QEN71_RS44585	hypothetical protein	QEN71_44575	paras_006843	protein-codi	NZ_CP1252	megaplasmi	384433	384654	+	218	0	2	109	2	109	
QEN71_RS44590	hypothetical protein	QEN71_44580	paras_006844	protein-codi	NZ_CP1252	megaplasmi	384959	385438	-	480	0	15	1506	10	1050	
QEN71_RS44595	IS3 family transposase	partial;pseudo;QEN71_44585		pseudogene	NZ_CP1252	megaplasmi	385735	386482	+	748	0	22	3521	19	3367	
QEN71_RS44600	IS91 family transposase	partial;pseudo;QEN71_44590		pseudogene	NZ_CP1252	megaplasmi	386869	387897	-	1029	106.0	32	4983	27	3527	
QEN71_RS44605	tyrosine-type recombinase/integrase	QEN71_44595	paras_006847	protein-codi	NZ_CP1252	megaplasmi	388058	389305	+	1244	0	41	6890	28	4684	
QEN71_RS44610	hypothetical protein	QEN71_44600	paras_006848	protein-codi	NZ_CP1252	megaplasmi	389302	389475	+	170	0	5	972	4	970	
QEN71_RS44615	tyrosine-type recombinase/integrase	QEN71_44605	paras_006849	protein-codi	NZ_CP1252	megaplasmi	389554	390114	+	561	0	21	3568	13	2638	
QEN71_RS44620	IS110 family transposase	QEN71_44610	paras_006850	protein-codi	NZ_CP1252	megaplasmi	390340	391401	+	1062	2107.0	16	3734	11	2343	
QEN71_RS44625	IS5 family transposase	pseudo;QEN71_44615		pseudogene	NZ_CP1252	megaplasmi	391709	392822	-	1114	2016.0	24	4717	19	3585	
QEN71_RS44630	transposase zinc-binding domain-containing protein	partial;pseudo;QEN71_44620		pseudogene	NZ_CP1252	megaplasmi	393420	393659	-	240	262.0	2	201	2	201	
QEN71_RS44635	integrase			pseudogene	NZ_CP1252	megaplasmi	393804	393923	+	120	0	3	90	1	4	
QEN71_RS44640	transposase	partial;pseudo;QEN71_44630		pseudogene	NZ_CP1252	megaplasmi	394033	394503	+	471	0	8	773	7	729	
QEN71_RS44645	IS5/IS1182 family transposase	partial;pseudo;QEN71_44635		pseudogene	NZ_CP1252	megaplasmi	395226	395362	-	137	0	2	147	2	147	
QEN71_RS44650	IS5 family transposase	QEN71_44640	paras_006855	protein-codi	NZ_CP1252	megaplasmi	395441	396247	+	807	1170.0	27	6836	20	5991	
QEN71_RS44655	transposase	partial;pseudo;QEN71_44645		pseudogene	NZ_CP1252	megaplasmi	396382	397014	+	633	1059.0	15	2086	5	1083	
QEN71_RS44660	IS110 family transposase	QEN71_44650	paras_006857	protein-codi	NZ_CP1252	megaplasmi	397322	398383	-	1062	2107.0	15	4042	11	3001	
