# Supplementary material for: Extensive spontaneous genome reduction in Paraburkholderia sabiae
Source: Sci Rep. 2026 Apr 29;16:19843. doi: 10.1038/s41598-026-49026-9 (PMC13316101; doi:10.1038/s41598-026-49026-9)
Supplement: Supplementary file 2 — Supplementary Material 2 [file 41598_2026_49026_MOESM2_ESM.pdf]

Extensive spontaneous genome reduction in *Paraburkholderia sabiae*

Kim Bolli[1], Colin Waichler[1], Yilei Liu[1], Daphné Golaz[1], Adam M. Deutschbauer[2,3], Leo Eberl[1], Sebastian J. Hug[1], Marta Torres[1]* & Gabriella Pessi[1]*

[1]Department of Plant and Microbial Biology, University of Zurich, CH-8057 Zurich, CH

[2]Environmental Genomics and Systems Biology Division, Lawrence Berkeley National Laboratory, Berkeley, CA 94720, USA

[3]Department of Plant and Microbial Biology, University of California, Berkeley, CA 94720, USA

***Correspondence:**

Gabriella Pessi, gabriella.pessi@botinst.uzh.ch

Marta Torres, marta.torres@botinst.uzh.ch

Supplementary Data

Supplementary Table 1: Strains, plasmids and oligonucleotides used in this study.

Supplementary Table 2: *Psabiae* _ML3 gene information, including scaffold, begin, end, strand, number of insertions, number of reads, duplicates (dupScore), number of TA sites, GC content. See separate excel file.

Supplementary Fig. 1: Competition assay on plate.

Supplementary Fig. 2: A: Control PCRs on *P. sabiae* Δ2 strain.

Supplementary Fig. 3: Abundance of genes from each COG category in *P. sabiae*'s second replicon (2,001 genes total) and whole genome (8,859 genes total).

Supplementary Fig. 4: Heatmap of the Average presence (AP) comparison of *Paraburkholderia* replicons.

Supplementary Fig. 5: Growth: of *P. sabiae* wild type and Δ2 in LBNS and AB with 10 mM potassium succinate at 28°C.

Supplementary Fig. 6: Representative TEM pictures of *P. sabiae* wild-type and Δ2.

Supplementary Fig. 7: Nodule occupancy of *Mimosa caesalpiniifolia* plants inoculated by *P. sabiae* wild type (WT) and Δ2 strain.

Supplementary Table 1: Strains, plasmids and oligonucleotides used in this study.

Strains	Description	Reference
Paraburkholderia sabiae		
LMG24235^T	Wild type, isolated from *Mimosa caesalpiniifolia*	[1]
T6SS-1_IM	*tssC* (QEN71_RS31700) insertion mutant ; Cm^R	[2]
Δ2	Spontaneous loss of the second replicon by LMG24235^T	This study
WT-GFP	Fluorescent deletion mutant generated with pSHAFT2_GFP_Km^R	This study
Δ2-GFP	Spontaneous loss of second replicon by WT-GFP	This study
Paraburkholderia phymatum		
STM815^T	Wild type, isolated from *Mimosa pudica*	[3]
STM815^T -pPROBE	Wild type harboring pPROBE-NT empty vector; Km^R	[4]
Escherichia coli		
c118 λ-pir	*Δ(ara-leu) araD Δlac74 galE galK phoA20 thi1 rpsE rpoB argE(Am)recAI λ pir;* Strep^R	[5]
DH5a	pRK2013, Helper strain, Cm^R	Invitrogen
AMD290	WM3064 (DAP auxotroph) harbouring the pHLL250 mariner transposon vector library	[6]
***Pseudomonas syringae* pv. tomato DC3000 (Pto)**		
DC3000	Wild type	
DC3000 -Tn7	Wild type, Tagged with mini Tn7 (Gm) PA1/04/04-egfp-a (GFP); Gm^R	[7]
**Plasmids**		
pSHAFT2	Broad-host-range suicide plasmid, mobilizable for conjugation; Cm^R	[8]
pSHAFT2_GFP_KmR	pSHAFT2_GFP_KmR_sabiae using Gibson assembly to generate a *P. sabiae* WT-GFP	This study

**Oligonucleotides**	Sequence (5' – 3')	Purpose of primer/primer pair	**Reference**
TssC-1_F_EcoRI	GCGCgaattcAGAAGAGTTCGGCACATTCG	Amplification of T6SS-1 QEN71_RS31700	Hug et al. 2023
TssC-1_R_SalI	GCGCgtcgacGCGTTACACCACAGGTACT		Hug et al. 2023
Paras_006942_F	GCATGGGACACCTCCATTC	Amplification of RepA Chromosome 2 QEN71_RS30105	This study
Paras_006942_R	TCGACCGGCATCAACCTTAT		This study
SctV-IM_A_F_XbaI_2	GCGCtctagaCAATCTTGTCGTCGGCATGG	Amplification of the *sctV* (QEN71_RS36820) of the T3SS-A	This study
SctV-IM_A_R_XhoI_2	GCGCctcgagGAAAGAGCGGCCAAGGAAAG		This study
SctV-IM_B_F_XhoI	GCGCctcgagGACACCAGTAGCGACGGA	Amplification of the sctV (QEN71_RS38125) of the T3SS-B	This study
SctV-IM_B_R_XbaI	GCGCtctagaCGTGGTGTTTCTCGTGATTG		This study
T5bSS-1_F_XhoI	GCGCctcgagGCTGTTGACCTGATTGACGA	Amplification of the *cdiA* (QEN71_RS11400) of the T5bSS-1	This study
T5bSS-1_R_XbaI	GCGCtctagaCAAAAAGAATCAAGGCGAAACG		This study
paras_000364_F_XbaI	GCGCtctagaGCAGCGATGTACTTGTACCG	Amplification of the pSymb VgrG (QEN71_RS41630)	This study
paras_000364_R_XhoI	GCGCctcgagAGACCTTCCGATTCCAGCAAT		This study
sab_up_fwd	GTATCCGGGAGCTCTTAGGCCTTTCTCGCCGCTTCTAAACAGTC	Amplification of upstream homology arm for Gibson assembly Intergenic region between QEN71_RS00040 and QEN71_RS00045	This study
sab_up_rev	GTCAAAGGCCTTTCAATGCATCCGGGCACTTC		This study
GFP_fwd	CCGGATGCATTGAAAGGCCTTTGACATTTGCAG	Amplification of gfp from pSHAFT-GFP for Gibson assembly	This study
GFP_rev	ATTATTCGACGCTCAGTTGTACAGTTCATCCATGC		This study
KanR_fwd	ACTGTACAACTGAGCGTCGAATAATTCCGCTAG	Amplification of kanR from p34E-KanR for Gibson assembly	This study
KanR_rev	CCCGGATGTTCTAGCGTCGAATTAATTCCGCG		This study
sab_down_fwd	TTAATTCGACGCTAGAACATCCGGGCACAC	Amplification of downstream homology arm for Gibson assembly Intergenic region between QEN71_RS00040 and QEN71_RS00045	This study
sab_down_rev	AGATCTAATCTAGAAAGGTACCAACAGAGAGGAAGCGAGATGAG		This study
pSHAFT_F	CTTCAGCTGATGTGTGATAACATACT	Sequencing pSHAFT insert	[9]
pSHAFT_R1	AACGCACTGAGAAGCCCTTA	Sequencing pSHAFT insert	[9]

Supplementary Table 2: *Psabiae* _ML3 gene information, including scaffold, begin, end, strand, number of insertions, number of reads, duplicates (dupScore), number of TA sites, GC content, etc. See separate excel file.

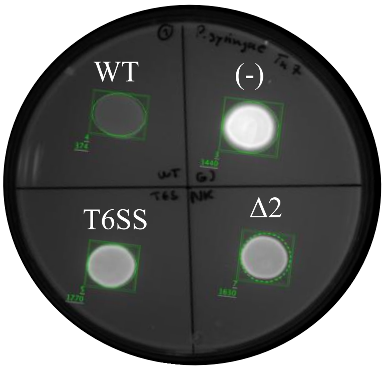

Supplementary Fig. 1: Competition assay on plate. (-): positive control *P. syringae* DC3000 Tn7 (GFP) alone. Wild type (WT): *P. syringae* DC3000 with *P. sabiae* wild-type; T6S: *P. syringae* DC3000 with *P. sabiae* T6SS-1_IM; NK: *P. syringae* DC3000 with non-killing *P. sabiae* strain (Δ2). The numbers indicate the amount of GFP expressed by the target strain *P. syringae* DC3000 as quantified by the LI-COR instrument.

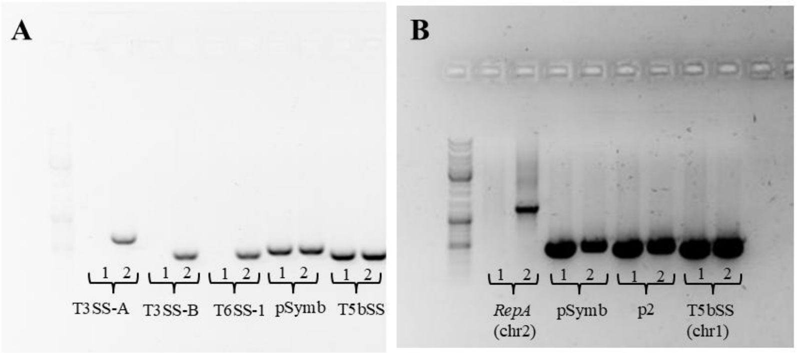

Supplementary Fig. 2: A: Control PCRs on the *P. sabiae* non-killing strain (Δ2)(1) and on the sequenced *P. sabiae* LMG24235^T strain [2] **B:** Control PCR of different genes present on each replicon. See primers in Supplementary Table 1.

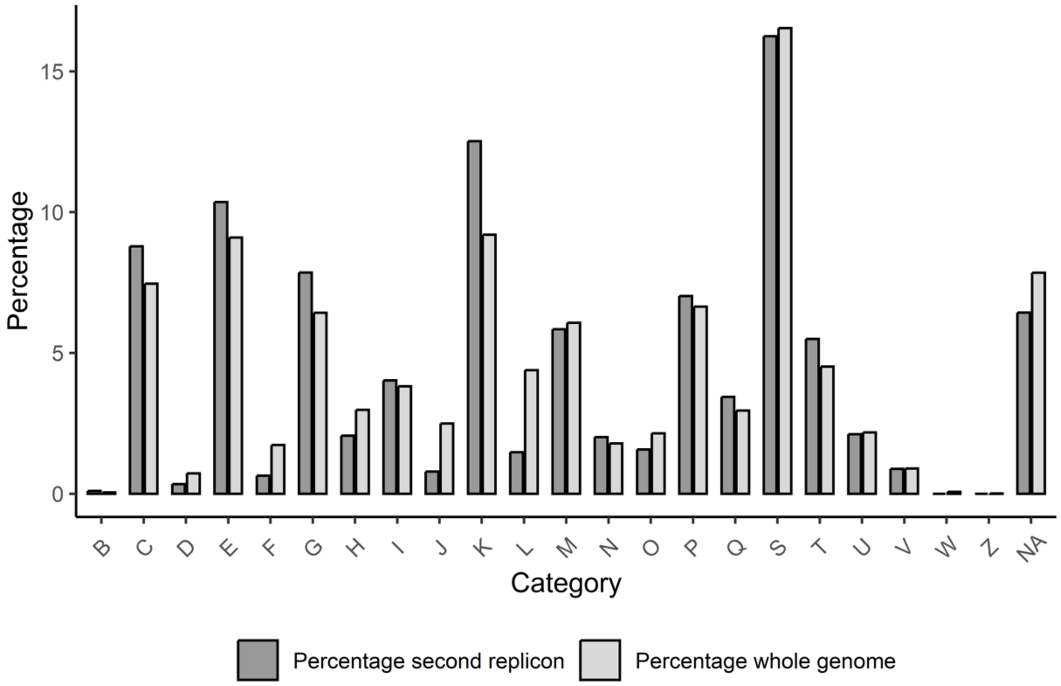

Supplementary Fig. 3: Abundance of genes from each COG category in *P. sabiae*'s second replicon (2,001 genes total) and whole genome (8,859 genes total). The different categories are indicated by the following letters: B, chromatin structure and dynamics; C, energy production and conversion; D, cell cycle control, cell division, chromosome partitioning; E, amino acid transport and metabolism; F, nucleotide transport and metabolism; G, carbohydrate transport and metabolism; H, coenzyme transport

and metabolism; I, lipid transport and metabolism; J, translation, ribosomal structure and biogenesis; K, transcription; L, replication, recombination and repair; M, cell wall/membrane/envelope biogenesis; N, cell motility; O, posttranslational modification, protein turnover, chaperone; P, inorganic ion transport and metabolism; Q, secondary metabolites biosynthesis, transport and catabolism; R, general function prediction only; S, function unknown; T, signal transduction mechanisms; U, intracellular trafficking, secretion, and vesicular transport; V, defence mechanisms; W, extracellular structures ;Z, cytoskeleton; NA, not categorizes.

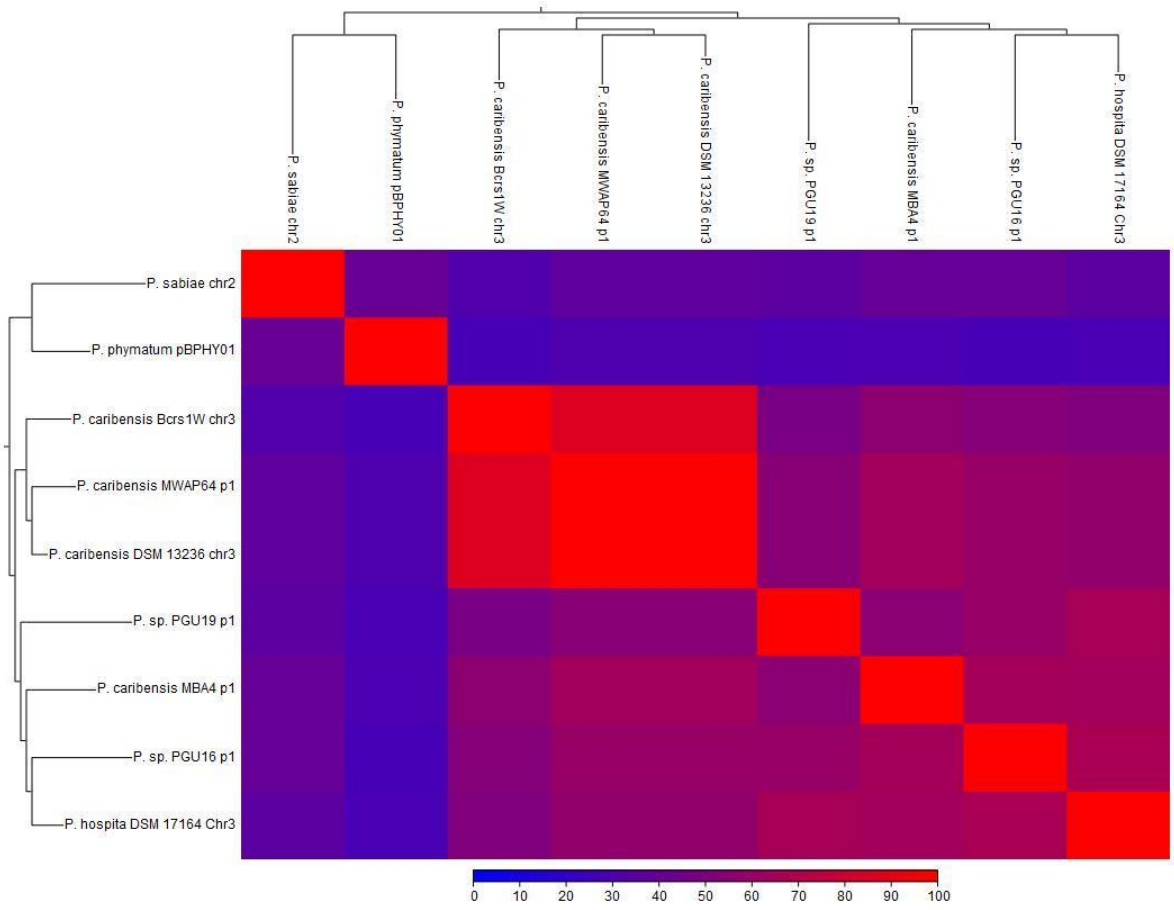

Supplementary Fig. 4: Heatmap of Average Presence (AP) Similarity Among *Paraburkholderia* Replicons. Colors represent pairwise AP similarity values (0–100%), with red indicating high similarity and blue indicating low similarity. The dendrogram shows hierarchical clustering based on shared genomic content. The heatmap was generated using CLC Genomics Workbench v11.0.

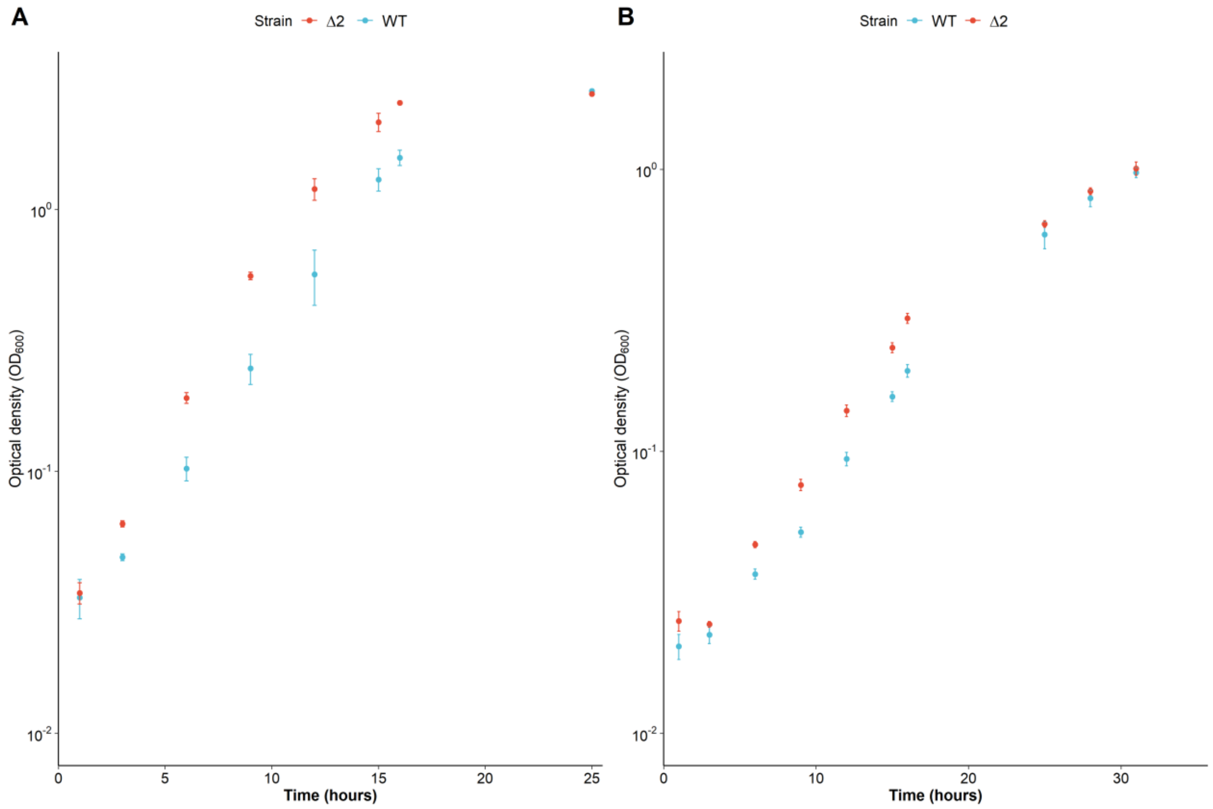

Supplementary Fig. 5: Growth of *P. sabiae* wild type and Δ2 in LBNS (**A**) and (B)AB with 10 mM potassium succinate at 28°C. Time points were taking every 3 h from 3 biological replicates.

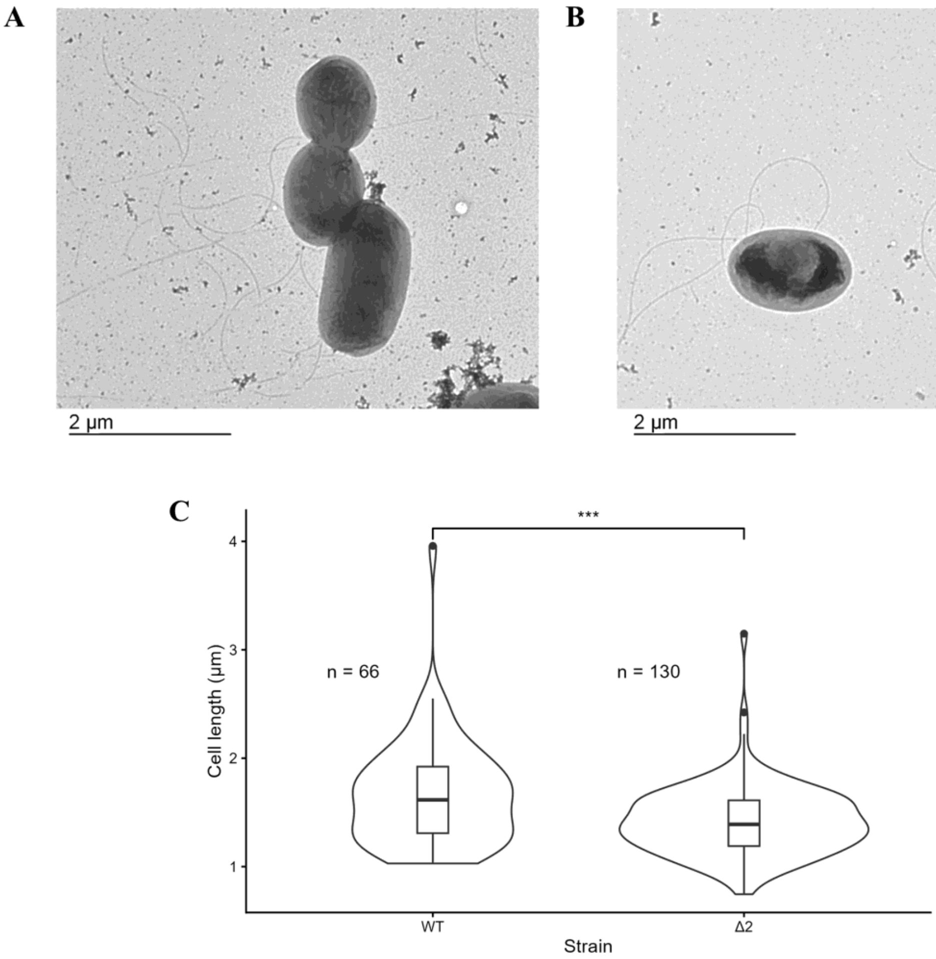

Supplementary Fig. 6: Representative TEM pictures of *P. sabiae* wild-type (**A**) and Δ2 (**B**) cells grown in ABS minimal medium at 28°C and 180rpm for 17 hours. Filaments are flagella. (**C**) Cell length is the total of cells recorded from 3 independent replicates.

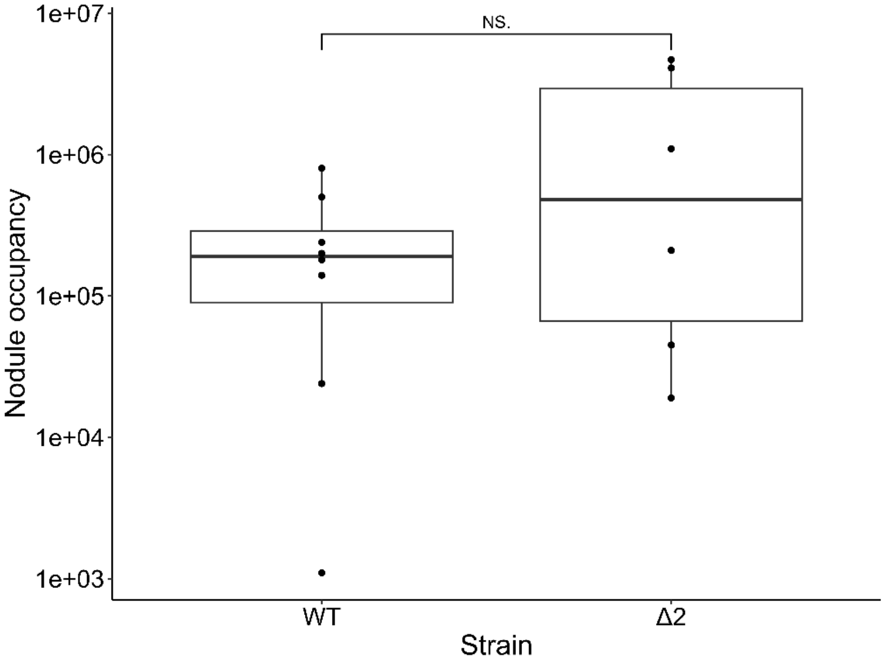

Supplementary Fig. 7: Nodule occupancy of *Mimosa caesalpiniifolia* plants inoculated by *P. sabiae* wild type (WT) and Δ2 strain 28 days after inoculation. One nodule per plant, 2 nodules per biological replicate were used to perform the analysis, *t-test;* ns p-value > 0.05.

1. Chen W-M et al. *Burkholderia sabiae* sp. nov., isolated from root nodules of *Mimosa caesalpiniifolia*. *International Journal of Systematic and Evolutionary Microbiology* 2008;**58**:2174–2179.

2. Hug S et al. *Paraburkholderia sabiae* Uses One Type VI Secretion System (T6SS-1) as a Powerful Weapon against Notorious Plant Pathogens. *Microbiology Spectrum* 2023;**11**:e01622-23. https://doi.org/10.1128/spectrum.01622-23
3. Moulin L et al. Nodulation of legumes by members of the β-subclass of Proteobacteria. *Nature* 2001;**411**:948–950. https://doi.org/10.1038/35082070
4. Liu Y et al. The Exopolysaccharide Cepacian Plays a Role in the Establishment of the *Paraburkholderia phymatum – Phaseolus vulgaris* Symbiosis. *Front Microbiol* 2020;**11**. https://doi.org/10.3389/fmicb.2020.01600
5. Herrero M, de Lorenzo V, Timmis KN. Transposon vectors containing non-antibiotic resistance selection markers for cloning and stable chromosomal insertion of foreign genes in gram-negative bacteria. *Journal of bacteriology* 1990;**172**:6557–6567.
6. Liu H et al. Magic Pools: Parallel Assessment of Transposon Delivery Vectors in Bacteria. *mSystems* 2018. https://doi.org/10.1128/msystems.00143-17
7. Purtschert-Montenegro G et al. *Pseudomonas putida* mediates bacterial killing, biofilm invasion and biocontrol with a type IVB secretion system. *Nat Microbiol* 2022;**7**:1547–1557. https://doi.org/10.1038/s41564-022-01209-6
8. Shastri S et al. An efficient system for the generation of marked genetic mutants in members of the genus *Burkholderia*. *Plasmid* 2017;**89**:49–56.
9. Lardi M et al. Transcriptome Analysis of *Paraburkholderia phymatum* under Nitrogen Starvation and during Symbiosis with *Phaseolus Vulgaris*. *Genes* 2017;**8**:389. https://doi.org/10.3390/genes8120389
